# Utility of the *trnH*–*psbA* Intergenic Spacer Region and Its Combinations as Plant DNA Barcodes: A Meta-Analysis

**DOI:** 10.1371/journal.pone.0048833

**Published:** 2012-11-14

**Authors:** Xiaohui Pang, Chang Liu, Linchun Shi, Rui Liu, Dong Liang, Huan Li, Stacey S. Cherny, Shilin Chen

**Affiliations:** 1 Institute of Medicinal Plant Development, Chinese Academy of Medical Sciences and Peking Union Medical College, Beijing, People's Republic of China; 2 School of Information Management, Central China Normal University, Wuhan, People's Republic of China; 3 School of Computer Science and Engineering, Beihang University, Beijing, People's Republic of China; 4 The State Key Laboratory of Brain and Cognitive Sciences, Department of Psychiatry, The University of Hong Kong, Pokfulam, Hong Kong; University of Gottingen, Germany

## Abstract

**Background:**

The *trnH*–*psbA* intergenic spacer region has been used in many DNA barcoding studies. However, a comprehensive evaluation with rigorous sequence preprocessing and statistical testing on the utility of *trnH*–*psbA* and its combinations as DNA barcodes is lacking.

**Methodology/Principal Findings:**

Sequences were searched from GenBank for a meta-analysis on the usefulness of *trnH*–*psbA* and its combinations as DNA barcodes. After preprocessing, we constructed full and matching data sets that contained 17 983 *trnH*–*psbA* sequences and 2190 sets of *trnH*–*psbA*, *matK*, *rbcL*, and ITS2 sequences from the same sample, repectively. These datasets were used to analyze the ability of *trnH*–*psbA* and its combinations to discriminate species by the BLAST and BLAST+P methods. The Fisher's exact test was used to evaluate the significance of performance differences. For the full data set, the identification success rates of *trnH*–*psbA* exceeded 70% in 18 families and 12 genera, respectively. For the matching data set, the identification rates of *trnH*–*psbA* were significantly higher than those of the other loci in two families and four genera. Similarly, the identification rates of *trnH*–*psbA*+ITS2 were significantly higher than those of *matK*+*rbcL* in 18 families and 21 genera.

**Conclusion/Significane:**

This study provides valuable information on the higher utility of *trnH*–*psbA* and its combinations. We found that *trnH*–*psbA*+ITS2 combination performs better or equally well compared with other combinations in most taxonomic groups investigated. This information will guide the optimal usage of *trnH*–*psbA* and its combinations for species identification.

## Introduction

Accurate species identification is a prerequisite for conducting numerous basic and applied studies on monitoring and conserving natural resources, blocking the traffic of endangered and invasive species, as well as controlling the quality of pharmaceutical and food products. Species identification is primarily based on morphology [1]. The classic method of species determination frequently also warrants expert interpretation. However, most species are studied by only a few specialists, and even specialists frequently encounter specimens that cannot be reliably identified. Thus, a rapid, subjective, and accurate method for species identification is urgently needed.

DNA barcoding is based on sequence diversity within a short and standardized gene region for species discrimination. This method can identify known species and discover novel ones [2], [3]. In animals, the *CO1* gene is widely used as the DNA barcode for efficient and accurate species determination [2], [4]–[9]. In plants, the situation is much more complicated. A variety of DNA regions have been proposed as barcodes for plant identification [10]–[20], but no region has been found to have characteristics for DNA barcoding as favorable as those of *CO1*. The Plant Working Group of the Consortium for the Barcode of Life [21] examined the suitability of seven leading candidate markers and proposed the two-locus combination of *matK*+*rbcL* as the core plant barcode. However, the lack of discriminatory power of this proposed barcode in some plant groups and the absence of primer universality for *matK* render the barcode questionable [22]. Further evaluation of other noncoding markers, particularly *trnH*–*psbA* and the internal transcribed spacers of nuclear ribosomal DNA (nrITS/nrITS2), is necessary before a universal plant barcode can be designated [22]. Recently, the usefulness of the ITS region as a DNA barcode has been further assessed by the China Plant BOL Group, who proposed that ITS should be used as the core plant barcode [23]. Although the *trnH*–*psbA* region has been evaluated in such studies in comparison with other markers alone or in combination, several shortcomings exist. First, most studies focus only on particular groups of plants. Second, the sequences are not rigorously processed. For example, intraspecific inversions and *rps19* insertions, which are both well known to be present in the *trnH*–*psbA* region, are not explicitly examined and processed, which may negatively affect the species identification success rate. Third, the *trnH*–*psbA*+ITS2 combination is not evaluated at the family and genus levels. Most importantly, the statistical tests for the differences in identification success rates among various single markers or combinations are not performed.

In the present study, we performed a meta-analysis based on multiple sources of data available in public databases to evaluate the utility of the *trnH*–*psbA* region and its combinations with other markers as plant DNA barcodes.

## Methods

### Search Strategy, Inclusion and Exclusion Criteria, and Data Extraction

**Figure 1:**
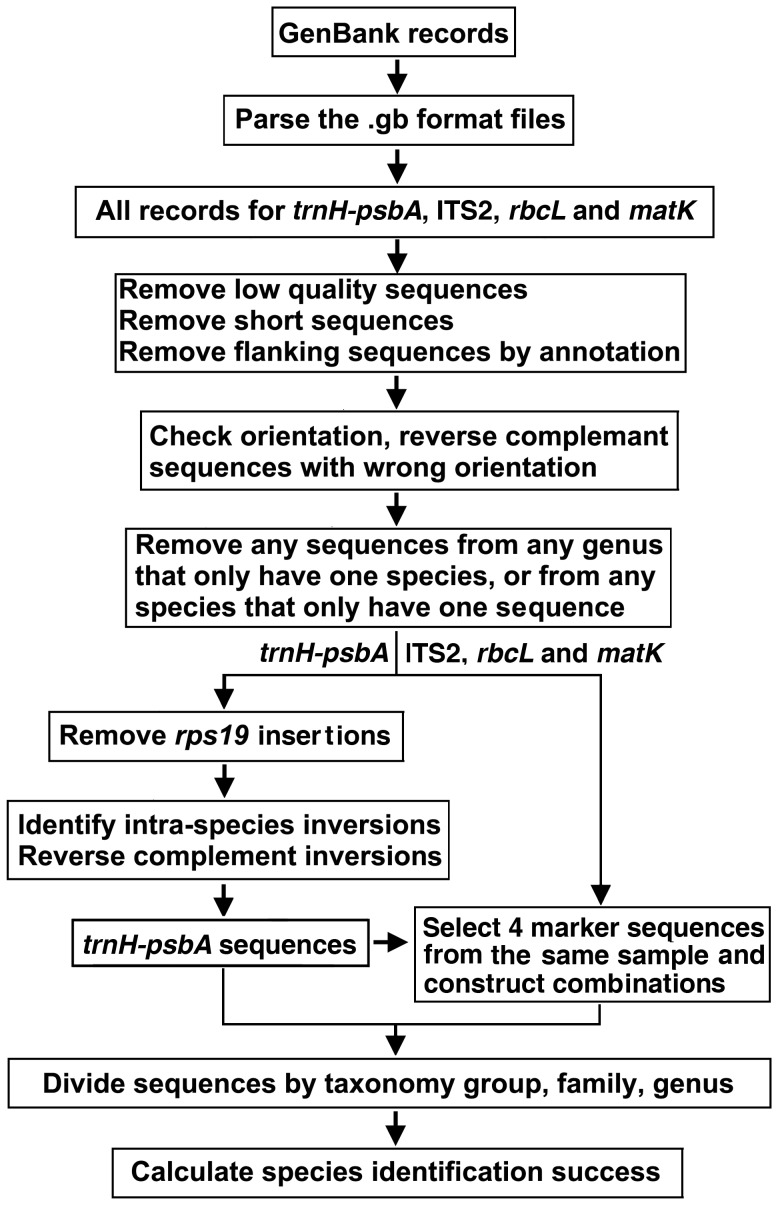
Workflow diagram for data processing and analysis.

The sequences of the *trnH*–*psbA* intergenic region as well as its *psbA* and *trnH* flanking regions from eudicotyledons, monocotyledons, gymnosperms, mosses, and ferns were extracted from GenBank (at end of July 2012) using the keywords “*psbA*” and “*trnH*” in the annotations. The *trnH*–*psbA* intergenic regions were further annotated using either BLAST or a hidden Markov model program to identify and trim the flanking regions. The plant sequences of *matK*, *rbcL*, and ITS2 were extracted from GenBank using the keywords “*matK*”, “*rbcL*”, and “Internal Transcribed Spacer 2”, respectively. Sequences from public databases such as GenBank/EMBL are generally not assessed following the standards of barcoding with respect to access to vouchers and sequence quality, among others. Thus, all sequences were subjected to rigorous processing to reduce dimensions of errors further. Low-quality sequences with more than 1% nucleotides being “Ns” were removed. The longest and shortest 1% sequences were treated as outliers and excluded. The orientation of each sequence was checked, and sequences in the *psbA*–*trnH* orientation were reverse-complemented to the *trnH*–*psbA* orientation. All sequences were searched using BLAST against a local *rps19* reference database that contained all sequences downloaded from GenBank. An *E* value less than 1×10^−5^ was used as a cutoff to determine if a sequence had an *rps19* insertion. The species with putative *rps19* sequences are listed in Table S1. To minimize the inflation of sequence divergence by intraspecific inversion, we developed custom Perl scripts to identify inversions, select a reference orientation, and reverse-complement all inversions that were in opposite orientation to the reference. The species with sequence inversions are shown in Table S2. Sequences belonging to any genus or family that contained only one species and those belonging to species for which only one sequence was available were also removed. The data processing pipeline is shown in Figure 1. The PRISMA checklist and flow diagram are available in the Supporting Information (PRISMA Checklist S1 and PRISMA Flow Diagram S1).

After processing, the remaining sequences were used to construct data sets. The full data set (Table S3) contained a total of 17 983 *trnH*–*psbA* sequences from 3495 species representing 498 genera in 149 families, which were used to analyze the ability of *trnH*–*psbA* to identify species in various taxonomic groups. We also constructed a matching data set that consisted of *trnH*–*psbA*, *matK*, *rbcL*, and ITS2 sequences with the same voucher number (same sample). This data set contained 2190 sets of *trnH*–*psbA*, *matK*, *rbcL*, and ITS2 sequences from 586 species belonging to 71 genera and 47 families (Table S4). The data set was used to assess the relative species identification performance of *trnH*–*psbA* and its combinations compared with other suggested single markers and *matK*+*rbcL*.

### Data Analysis

Sequence lengths were calculated for all *trnH*–*psbA* sequences of eudicotyledons, monocotyledons, gymnosperms, mosses, and ferns. The preprocessed sequences were aligned using ClustalW, followed by manual correction of the alignment, and Kimura's two-parameter distances were calculated using DNADIST from the EMBOSS package (Version 6.2). Three parameters were used to evaluate interspecific differences: (i) average interspecific distance between all species in each genus with more than one species (all interspecific distances), (ii) mean pairwise distance within each genus with at least two species (theta prime), and (iii) smallest interspecific distance within each genus with more than one species (minimum interspecific distance). Another set of three parameters was used to evaluate intraspecific differences: (i) average intraspecific distance between all samples within each species with at least two representatives (all intraspecific differences), (ii) mean pairwise distance within each species with more than one individual (theta), and (iii) maximum intraspecific distance within each species with more than one representative (coalescent depth). The intraspecific differences and interspecific variations of congeneric species of *trnH*–*psbA* in the five major plant taxonomic groups were calculated using a custom Perl script as previously described [17], [24]–[26].

BLAST and BLAST+P distance were performed using custom-written Perl scripts. Reference databases containing all sequences from the matching data set or the full data set were constructed. Using BLAST, we searched the reference database using the query sequence and inferred its identity based on the top hit, as previously described [27]. For the BLAST+P method, we first performed the BLAST search to select the significant (*E* value<1×10^−5^) and top (maximum number, 100) sequences. Multiple sequence alignment, distance calculation, and identity determination were then performed for these sequences. The identity of a query was inferred based on those of its neighbors with the shortest distance. The significant differences between the identification success rates of markers were tested by the Fisher's exact test implemented using JMP software (SAS Corp, NC, USA).

## Results

### Universality of the Intraspecific Inversion and *rps19* Insertions in *trnH*–*psbA*

Two characteristics of *trnH*–*psbA* in some taxa reportedly complicate the use of the region as a DNA barcode. These characteristics are intraspecific inversions and *rps19* insertions, which inflate intraspecific variation. In this study, we calculated the percentages of species with inversions and sequences with *rps19* insertions in different families and genera to evaluate the universality of the two characteristics. Generally, sequences from 57 families and 111 genera contained inversions, accounting for 38.3% and 22.3% of all 149 families and 498 genera, respectively (Tables S5 and S6). On the other hand, sequences from 41 families and 135 genera had *rps19* insertions, comprising 27.5% and 27.1% of the total of 149 families and 498 genera, respectively (Tables S7 and S8). The percentages of species with inversions in 14 of the 57 families exceeded 30% (Table S5), and the percentages of sequences with *rps19* insertions were higher than 50% in 24 of the 41 families containing *rps19* insertions and 100% in 14 (Table S7). Among the 111 genera with inversions, the percentages of species with inversions were more than 30% for 58 genera and 100% for 5 (Table S6). In the 135 genera with *rps19* insertions, the percentages of sequences with *rps19* insertions exceeded 50% in 108 genera and 100% in 97 (Table S8). We included steps to identify the inversions and *rps19* insertions as well as perform the necessary corrections (such as the reorientation of the inversions and removal of the *rps19* insertions) in our data processing pipeline. All data reported hereafter were subjected to these corrections.

### Sequence Length of *trnH*–*psbA*

The sequence length of a marker affects the efficiency of PCR amplification. Consequently, we examined the length distribution of the *trnH*–*psbA* sequences. As shown in Figure S1, the lengths of *trnH*–*psbA* sequences in eudicotyledons, monocotyledons, gymnosperms, ferns, and mosses ranged from 152 bp to 851 bp, from 151 bp to 905 bp, from 283 bp to 1006 bp, from 167 bp to 547 bp, and from 103 bp to 265 bp, respectively. The corresponding average lengths obtained were 357, 357, 573, 429, and 141 bp, respectively.

### Genetic Divergences of *trnH*–*psbA*

For ferns, the maximal intraspecific distance of *trnH*–*psbA* was generally smaller than the corresponding smallest interspecific divergence between congeneric species (Table 1). For the other four groups, the intraspecific variation and interspecific distance did not show clear separation (Table S9). *trnH*–*psbA* demonstrated a clear DNA barcoding gap only in ferns (Figure S2).

**Table 1:** Identification success rates of *trnH*–*psbA* in the five major plant taxonomic groups using BLAST and BLAST+P distance.

Taxa	No. of families	No. of genera	No. of species	No. of samples	BLAST	BLAST+P distance
					Success (%)	Success (%)
Eudicotyledons	92	332	2437	13727	51.1	64.5
Monocotyledons	32	126	782	3054	45.7	54.7
Gymnosperms	7	12	126	633	35.5	37.0
Mosses	9	14	52	277	72.2	78.3
Ferns	9	14	98	292	75.0	75.3

### Ability of *trnH*–*psbA* to Identify Species in Various Taxonomic Groups

The BLAST and BLAST+P distance methods were used to evaluate the performance of *trnH*–*psbA* in species identification. Using BLAST, the *trnH*–*psbA* region correctly identified 51.1%, 45.7%, 35.5%, 72.2%, and 75.0% of 13 727 eudicotyledon, 3054 monocotyledon, 633 gymnosperm, 277 moss, and 292 fern sequences at the species level, respectively (Table 1). The success rates of *trnH*–*psbA* identification in the five groups were higher using the BLAST+P distance method than using BLAST. When the BLAST+P distance method was used, the corresponding species identification rates of *trnH*–*psbA* for these groups were 64.5%, 54.7%, 37.0%, 78.3%, and 75.3%, respectively (Table 1).

**Figure 2:**
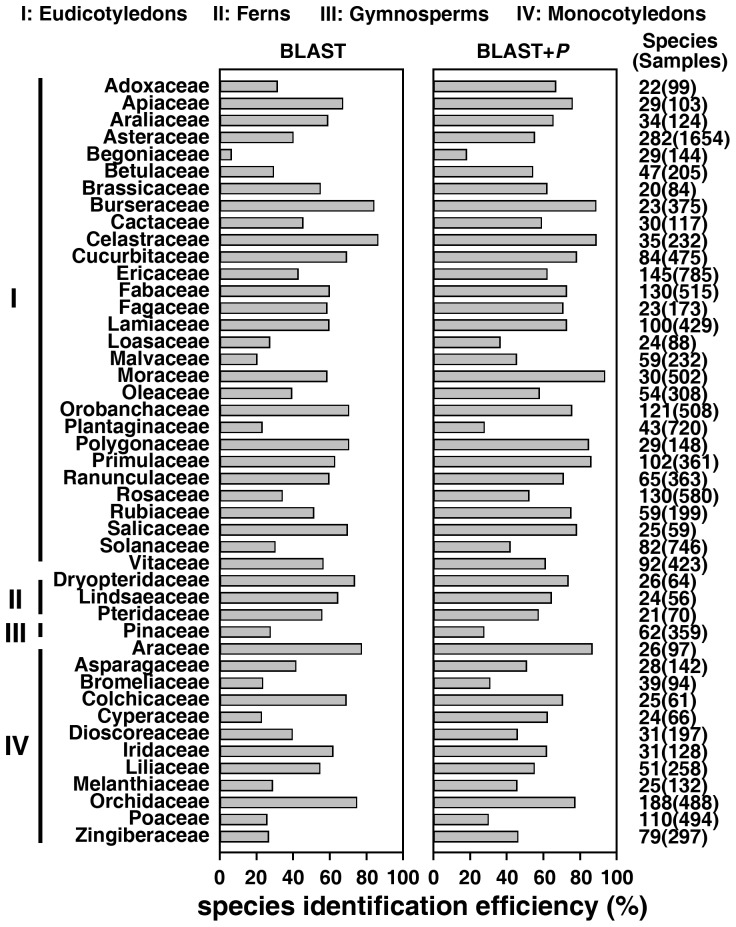
Success rates of *trnH*–*psbA* in discriminating closely related species in families with at least 20 species using BLAST (left) and BLAST+P distance (right).

We also analyzed the species identification ability of *trnH*–*psbA* in different plant families. Among the 45 families with at least 20 species, the rates of successful identification of 7 families were higher than 70% using BLAST (Figure 2). The success rate of using *trnH*–*psbA* in discriminating members of family Begoniaceae was the lowest at 6.3%. On the other hand, the success rate of using *trnH*–*psbA* to differentiate among family Celastraceae was the highest at 86.2% (Figure 2). Using the BLAST+P distance method, identification success rates exceeded 70% in 18 families (Figure 2). The species identification success rate for Begoniaceae was the lowest at 18.1%, whereas the rate for Moraceae was the highest at 93.4% (Figure 2). The success rates of using *trnH*–*psbA* to identify families with fewer than 20 species are listed in Table S10.

**Figure 3:**
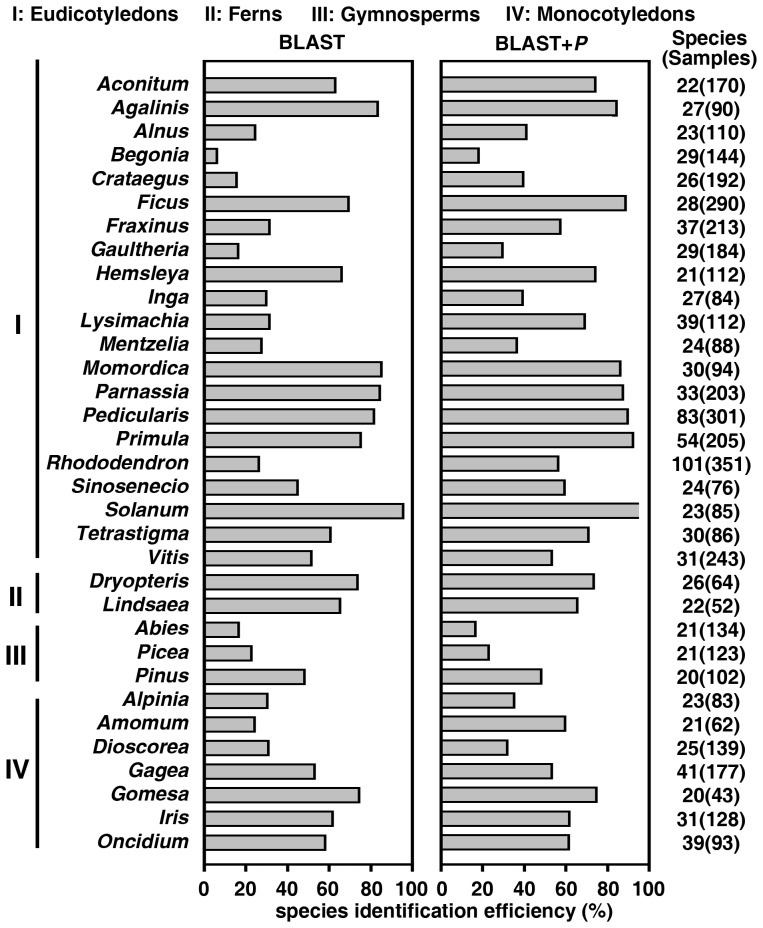
Identification success rates of *trnH*–*psbA* in genera with at least 20 species using BLAST (left) and BLAST+P distance (right).

We also analyzed the species identification ability of *trnH*–*psbA* in different genera. Among the 33 genera with at least 20 species, identification success rates exceeded 70% for 8 genera using BLAST (Figure 3). When the BLAST+P distance method was used, the success rates of *trnH*–*psbA* identification were higher than 70% in 12 genera. The success rates for discriminating among the *Abies*, *Begonia*, and *Picea* genera were the lowest at 16.4%, 18.1%, and 22.8%, respectively (Figure 3). The success rates for the identification of *Ficus*, *Pedicularis*, *Primula*, and *Solanum* species were the highest at 88.6%, 89.7%, 92.2%, and 96.5%, respectively (Figure 3). For 12 species-rich, taxonomically complex genera (*Vitis*, *Gagea*, *Rhododendron*, *Fraxinus*, *Oncidium*, *Iris*, *Lysimachia*, *Tetrastigma*, *Momordica*, *Parnassia*, *Pedicularis*, and *Primula*), with 86–351 samples from 30–101 species in this study, *trnH*–*psbA* exhibited successful identification rates within the range of 53.1%–92.2% (Figure 3). The success rates for the identification of genera with fewer than 20 species are presented in Table S11.

To determine easily the family- or genus-specific discriminatory power of *trnH*–*psbA*, we added a Check Performance module in the *trnH*–*psbA* Web server (http://psba-trnh-plantidit.dnsalias.org). Potential users of DNA barcoding technology can check the discriminatory power of *trnH*–*psbA* in various taxonomic groups and determine whether *trnH*–*psbA* works well for a given group.

### Relative Performance of *trnH*–*psbA* and Its Combinations in Species Identification Compared with Other Suggested Single Markers and the Core Barcode *matK*+*rbcL*

**Figure 4:**
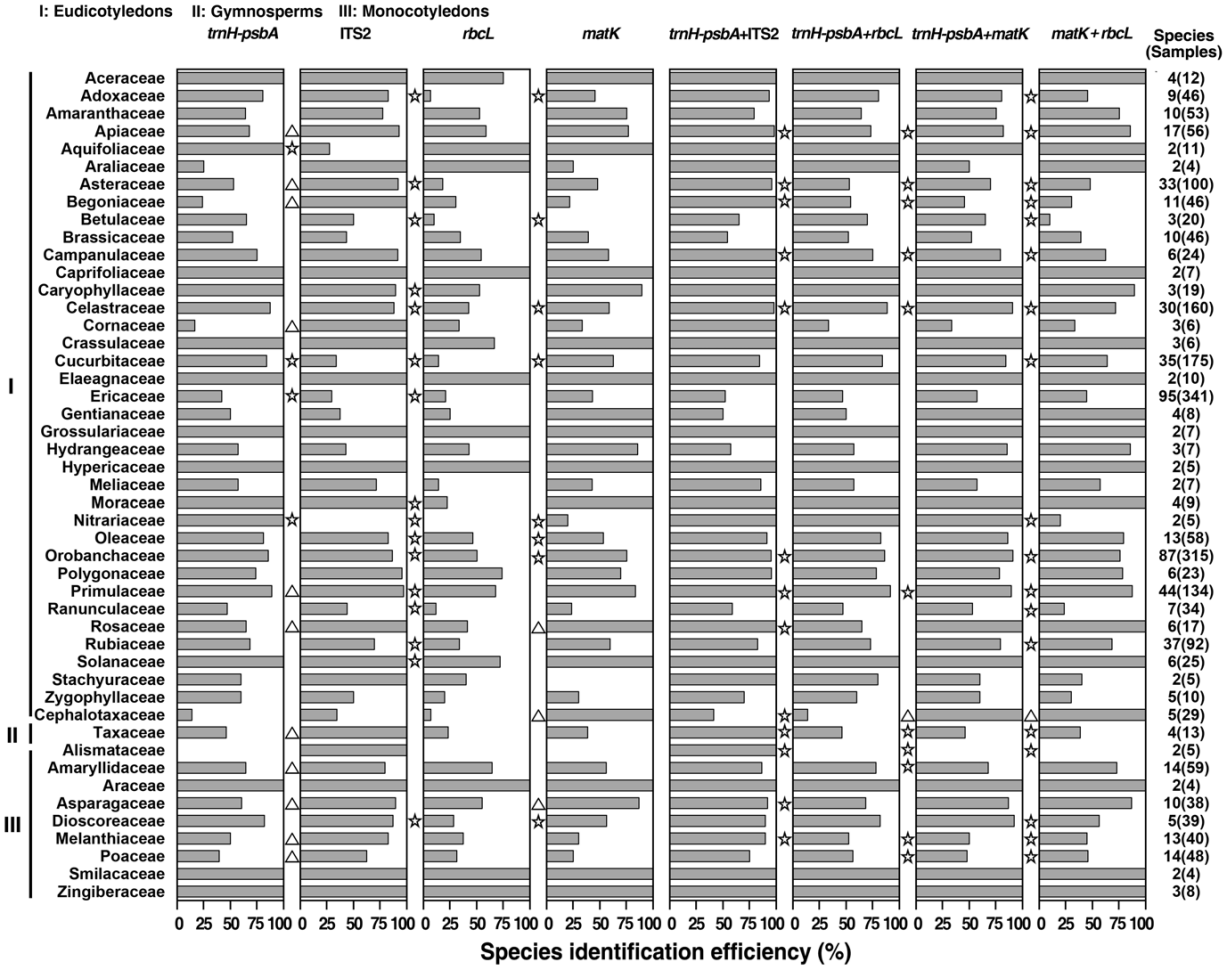
Comparison of identification success rates for markers in different families and the corresponding statistical test results. The statistical tests were carried out between *trnH*–*psbA* and the other three single markers, or *trnH*–*psbA*+ITS2 and the other three marker combinations, respectively. The significant differences are indicated to the left of the column for the corresponding marker or marker combination. “☆” indicates that the identification success rates for *trnH*–*psbA* or *trnH*–*psbA*+ITS2 are significantly higher than those of the corresponding marker or marker combination. “▵” indicates that the identification success rates for *trnH*–*psbA* or *trnH*–*psbA*+ITS2 are significantly lower than those of the corresponding marker or marker combination.

**Figure 5:**
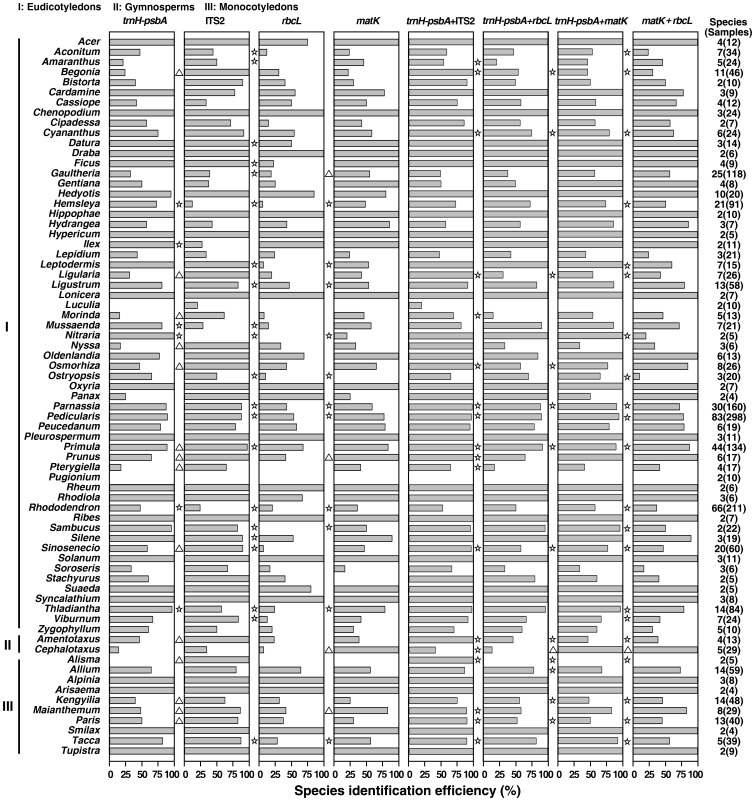
Comparison of the identification success rates for markers in different genera and the corresponding statistical test results. The statistical tests were carried out between *trnH*–*psbA* and the other three single markers, or *trnH*–*psbA*+ITS2 and three other marker combinations, respectively. The significant differences are indicated to the left of the column for the corresponding marker or marker combination. “☆” indicates that the identification success rates for *trnH*–*psbA* or *trnH*–*psbA*+ITS2 are significantly higher than those of the corresponding marker or marker combination. “▵” indicates that the identification success rates for *trnH*–*psbA* or *trnH*–*psbA*+ITS2 are significantly lower than those of the corresponding marker or marker combination.

To further evaluate the discriminatory ability of the *trnH*–*psbA* region, we compared its identification rates with those of the other three most recommended markers (*matK*, *rbcL*, and ITS2) based on the matching data set using the BLAST+P distance method. For the 47 families tested, *trnH*–*psbA* exhibited the highest identification efficiency among the four DNA barcodes in 19 families (Figure 4). Using the Fisher's exact test, we found that *trnH*–*psbA* had a significantly higher discrimination rate than the other three DNA barcodes in Cucurbitaceae and Nitrariaceae (Figure 4). Detailed information on the identification success rate of single markers at the family level can be found in Table S12. Among the 71 genera tested, the discrimination rates of ITS2, *trnH*–*psbA*, *matK*, and *rbcL* were higher than 80% in 45, 34, 30, and 19 genera, respectively (Figure 5). The *trnH*–*psbA* region had the highest identification rate among the four DNA barcodes in 36 genera (Figure 5). Fisher's exact test demonstrated that the identification success rates of *trnH*–*psbA* were significantly higher than those of the other three DNA barcodes in *Nitraria*, *Thladiantha*, *Hemsleya*, and *Rhododendron* (Figure 5). ITS2 and *trnH*–*psbA* showed superior identification ability in *Primula* (97.0% with ITS2, 88.8% with *trnH*–*psbA*), *Pedicularis* (87.9% with ITS2, 89.6% with *trnH*–*psbA*), and *Parnassia* (88.1% with ITS2, 87.5% with *trnH*–*psbA*), which had at least 30 species each. No significant difference was found between the identification rates of the two markers in *Pedicularis* and *Parnassia* (Figure 5). The four DNA barcodes showed low discrimination rates in *Rhododendron* (24.2% with ITS2, 47.4% with *trnH*–*psbA*, 20.4% with *rbcL*, 36.0% with *matK*) (Figure 5). Detailed information on the identification success rate of single markers at the genus level can be found in Table S13.

In addition to comparing the performance of individual markers, we also combined *trnH*–*psbA* with the above-described three loci and compared the various combinations with *matK*+*rbcL*. The *trnH*–*psbA*+ITS2 combination showed the highest discrimination rate among the four two-locus combinations in 41 of the 47 families (Figure 4). Fisher's exact test showed that *trnH*–*psbA*+ITS2 had significantly higher identification efficiency than the other three combinations in nine families (Begoniaceae, Apiaceae, Asteraceae, Celastraceae, Primulaceae, Campanulaceae, Alismataceae, Taxaceae, and Melanthiaceae) (Figure 4). The *trnH*–*psbA*+ITS2 combination had significantly higher identification efficiency than *matK*+*rbcL* in 18 families, whereas *matK*+*rbcL* had significantly higher identification efficiency than *trnH*–*psbA*+ITS2 in one family (Figure 4). Detailed information on the identification success rate of two-locus combinations at the family level can be found in Table S14. Among the 71 genera tested, *trnH*–*psbA*+ITS2 provided the highest discrimination rate among the four combinations for 61 genera (Figure 5). Fisher's exact test showed that the discrimination rates of *trnH*–*psbA*+ITS2 were significantly higher than those of the other three combinations in nine genera (*Begonia*, *Parnassia*, *Ligularia*, *Alisma*, *Cyananthus*, *Amentotaxus*, *Primula*, *Paris*, and *Sinosenecio*) (Figure 5). In particular, *trnH*–*psbA*+ITS2 had significantly higher identification efficiency than *matK*+*rbcL* in 21 genera, whereas the discrimination success rates of *matK*+*rbcL* were significantly higher than those of *trnH*–*psbA*+ITS2 for one genus (Figure 5). *trnH*–*psbA*+ITS2 performed well in discriminating among the species-rich genera *Primula*, *Parnassia*, *Pedicularis*, and *Rhododendron*, for which the discrimination success rates were 100.0%, 98.1%, 97.0%, and 52.6%, respectively. The identification rates of *trnH*–*psbA*+*matK*, *trnH*–*psbA*+*rbcL*, and *matK*+*rbcL* were as follows: 89.6%, 91.8%, and 87.3% for *Primula*; 90.6%, 88.8%, and 71.9% for *Parnassia*; 94.0%, 90.3%, and 77.9% for *Pedicularis*; and 57.3%, 50.7%, and 36.5% for *Rhododendron*, respectively. Detailed information on the identification success rate of two-locus combinations at the genus level can be found in Table S15.

## Discussion

*trnH*–*psbA* is widely accepted as a supplementary DNA barcode. A number of studies have also examined the relative performance of *trnH*–*psbA* in a taxonomic setting [28], [29]. However, these studies have focused on particular taxon settings using different data, different analytical methods, and inconsistent parameters to evaluate the performance of this spacer region. Consequently, the results of studies on different taxa, even on the same taxon, are difficult to compare and generalize. A systematic evaluation is needed to estimate uniformly the efficacy of using *trnH*–*psbA* in species determination across various groups. The present study aimed to perform such comprehensive evaluation in a systematic and standard way.

For the full data set constructed in this study, the *trnH*–*psbA* region demonstrated different discrimination abilities in various plant taxonomic groups. For example, under the BLAST+P distance method, the identification success rates for 13 727 eudicotyledons, 3054 monocotyledons, 633 gymnosperms, 277 mosses, and 292 ferns were 64.5%, 54.7%, 37.0%, 78.3%, and 75.3% at the species level, respectively. Among the 45 families with at least 20 species tested, *trnH*–*psbA* provided a high discrimination rate (>70%) in 18 families, with Moraceae being the highest at 93.4%. Among 33 genera with at least 20 species, the success rates of *trnH*–*psbA* identification were higher than 70% in 12 genera, with *Solanum* being the highest at 96.5%. For the matching data set, *trnH*–*psbA* had a significantly higher identification rate than the other three suggested loci in Cucurbitaceae, Nitrariaceae, *Nitraria*, *Thladiantha*, *Hemsleya*, and *Rhododendron*. To the best of our knowledge, this study is by far the most comprehensive and systematic analysis of *trnH*–*psbA* as a DNA barcode. The results guide the optimal use of *trnH*–*psbA* for species identification.

Three problems that may generate spurious results are inherent in the current study design. First, the sequences from GenBank/EMBL may not be assessed following the standards of barcoding. Our solution was to subject the sequence data to a very rigorous preparation process. We initially downloaded all sequences from GenBank. Some species had only one representative sequence, and these sequences may have contained more errors. Intraspecific variation also cannot be calculated for these species; similarly, some genera have only one species. Thus, congeneric, interspecific distances cannot be calculated for these genera. Consequently, sequences for single-sequence species or single-species genera were filtered, and only those belonging to genera with at least two species and species with at least two sequences were retained.

Second, the *trnH*–*psbA* intergenic spacer is highly problematic [30]–[32] because of very frequent intrapopulation inversions that can lead to an overestimation of species diversity when directly correlating sequence information to taxonomic entities. A fragment of *rps19* was frequently found as an insertion in some groups, which also led to an overestimation of the species diversity. Our study also systematically evaluated the prevalence of intraspecific inversions and *rps19* insertions in *trnH*–*psbA* sequences. Among the total of 149 families and 498 genera tested, 57 families and 111 genera contained species with inversions and 41 families and 135 genera included sequences with *rps19* insertions.

To solve this problem, we developed a pipeline that can perform the following steps: (1) identify and mask out the *rps19* fragment, and (2) identify intraspecific inversions, select a reference orientation, and reverse-complement inversions in the wrong orientations. These steps led to an improved alignment of *trnH*–*psbA* sequences and will be described in detail in a separate paper. We also tested novel methods based on hybrid local alignment, global alignment, and alignment-free methods to compare the query sequence and the database, which significantly improved the discriminatory power of *trnH*–*psbA* (http://psba-trnh-plantidit.dnsalias.org).

Lastly, this study was designed as a meta-analysis. Accordingly, we adopted a heterogeneous sampling strategy instead of other strategies in a particular geographical (all species in an area) or taxon (all species in a genus or family) setting. To ensure the reliability of the results, we carried out a stringent data preprocessing step. Such a systematic evaluation enabled this study to provide information on the prevalence of intraspecific inversions and *rps19* insertions, and offers a species identification performance metric for *trnH*–*psbA* in various taxon settings. Such information cannot be obtained using sequences from particular geographical or taxonomic settings.

The essence of DNA barcoding technology is the use of a universal barcode. However, in reality, “regardless of the region(s) ultimately adopted for plant barcoding, there will always be some species that would be better resolved by some other region” [12]. For example, research has shown that *trnH*–*psbA* exhibits better discrimination ability than *matK*+*rbcL* in angiosperm genera, such as *Ficus* [33] and *Alnus* [34]. The *matK*+*rbcL* barcode is even more problematic for non-angiosperms, particularly ferns, because of the difficulty in generating full-length *matK* sequences [35], [36] or the insufficient discriminatory capacity of *rbcL* [37]–[39]. In the present study, we compared the combinations of *trnH*–*psbA* with three other popular markers (ITS2, *rbcL*, and *matK*) to the core DNA barcode *matK*+*rbcL* using the matching data set. The *trnH*–*psbA*+ITS2 combination performed the best among all four two-locus combinations in the majority of taxa examined at the family or genus level. Among the 47 families and 71 genera tested, *trnH*–*psbA*+ITS2 had significantly higher identification efficiency than *matK*+*rbcL* in 18 families and 21 genera, wherease the discrimination success rates of *matK*+*rbcL* were significantly higher than those of *trnH*–*psbA*+ITS2 only for one family and one genus. These findings indicated the need to describe supplementary barcodes to complete the core barcode *matK*+*rbcL*. This study showed that the *trnH*–*psbA*+ITS2 combination may even be a more effective plant DNA barcode than the core barcode.

## Supporting Information

Table S1 **List of sequences containing** ***rps19*** **.**
(PDF)

Table S2 **List of species with inversions in their sequences.**
(PDF)

Table S3 **List of** ***trnH*** **–** ***psbA*** **samples used in this study.** The names of the corresponding group, family, genus, and species, as well as the GenBank accession number for each sample are shown.
(PDF)

Table S4 **List of** ***trnH*** **–** ***psbA*** **, ITS2,** ***rbcL*** **, and** ***matK*** **samples with the same voucher numbers.** The names of the corresponding group, family, genus, and species, the voucher number, as well as the GenBank accession number for each sample are shown.
(PDF)

Table S5 **Percentages of species with inversions in different families.**
(PDF)

Table S6 **Percentages of species with inversions in different genera.**
(PDF)

Table S7 **Percentages of sequences with** ***rps19*** **insertions in different families.**
(PDF)

Table S8 **Percentages of sequences with** ***rps19*** **insertions in different genera.**
(PDF)

Table S9 **Intra- and interspecific distances of congeneric species in the five major plant taxonomic groups.**
(PDF)

Table S10 **Identification success rates of** ***trnH*** **–** ***psbA*** **using BLAST and BLAST+P distance in selected families with fewer than 20 species.**
(PDF)

Table S11 **Identification success rates of** ***trnH*** **–** ***psbA*** **using BLAST and BLAST+P distance in genera with fewer than 20 species.**
(PDF)

Table S12 **Detailed information on the identification success rate of single markers at the family level and the corresponding statistical test results.**
(PDF)

Table S13 **Detailed information on the identification success rate of single markers at the genus level and the corresponding statistical test results.**
(PDF)

Table S14 **Detailed information on the identification success rate of two-locus combinations at the family level and the corresponding statistical test results.**
(PDF)

Table S15 **Detailed information on the identification success rate of two-locus combinations at the genus level and the corresponding statistical test results.**
(PDF)

Figure S1 **Box plots of the lengths of** ***trnH*** **–** ***psbA*** **sequences in the five major plant taxonomic groups.** In each box plot, the box shows the interquartile range of the data, which is defined as the difference between the 75th and 25th percentiles. The continuous and dotted lines across the box represent the median and average values, respectively.
(TIF)

Figure S2 **Inter- and intraspecific divergences of** ***trnH*** **–** ***psbA*** **sequences in the five major plant taxonomic groups.** Sequence divergence across all species for which sequences of multiple individuals are presented is illustrated. Divergence is shown as a scatter plot between the maximal intraspecific and minimal interspecific distances. A black line is drawn where the two distances are equal.
(TIF)

PRISMA Checklist S1 (DOC)

PRISMA Flow Diagram S1 (DOC)
