# Supplementary material for: Utility of the trnH–psbA Intergenic Spacer Region and Its Combinations as Plant DNA Barcodes: A Meta-Analysis
Source: PLoS One. 2012 Nov 14;7(11):e48833. doi: 10.1371/journal.pone.0048833 (PMC3498263; doi:10.1371/journal.pone.0048833)
Supplement: Table S1 — List of sequences containing rps19 . (PDF) [file pone.0048833.s001.pdf]

Table S1. List of sequences containing *rps19* .

Taxonomy group	Family	Genus	Species	GenBank accesstion no.
Eudicotyledons	Asteraceae	*Achillea*	*Achillea millefolium*	FJ395492
Eudicotyledons	Asteraceae	*Eupatorium*	*Eupatorium perfoliatum*	GU014469
Eudicotyledons	Melastomataceae	*Melastoma*	*Melastoma candidum*	JF708230
Eudicotyledons	Acanthaceae	*Ruellia*	*Ruellia elegans*	GU003917
Eudicotyledons	Lamiaceae	*Glechoma*	*Glechoma hederacea*	FJ395524
Eudicotyledons	Lamiaceae	*Mentha*	*Mentha spicata*	EU585744
Eudicotyledons	Rosaceae	*Potentilla*	*Potentilla reptans*	FJ395525
Eudicotyledons	Asteraceae	*Senecio*	*Senecio vulgaris*	FJ395482
Eudicotyledons	Fagaceae	*Quercus*	*Quercus phillyraeoides*	AB650376
Eudicotyledons	Fagaceae	*Quercus*	*Quercus phillyraeoides*	AB650377
Eudicotyledons	Fagaceae	*Quercus*	*Quercus phillyraeoides*	AB650378
Eudicotyledons	Fagaceae	*Quercus*	*Quercus phillyraeoides*	AB650379
Eudicotyledons	Fagaceae	*Quercus*	*Quercus phillyraeoides*	AB650380
Eudicotyledons	Fagaceae	*Quercus*	*Quercus phillyraeoides*	AB650381
Eudicotyledons	Fagaceae	*Quercus*	*Quercus phillyraeoides*	AB650382
Eudicotyledons	Fagaceae	*Quercus*	*Quercus phillyraeoides*	AB650383
Eudicotyledons	Fagaceae	*Quercus*	*Quercus phillyraeoides*	AB650384
Eudicotyledons	Fagaceae	*Quercus*	*Quercus phillyraeoides*	AB650385
Eudicotyledons	Fagaceae	*Quercus*	*Quercus phillyraeoides*	AB650386
Eudicotyledons	Fagaceae	*Quercus*	*Quercus phillyraeoides*	AB650387
Eudicotyledons	Fagaceae	*Quercus*	*Quercus phillyraeoides*	AB650388
Eudicotyledons	Fagaceae	*Quercus*	*Quercus phillyraeoides*	AB650389
Eudicotyledons	Fagaceae	*Quercus*	*Quercus phillyraeoides*	AB650390
Eudicotyledons	Fagaceae	*Quercus*	*Quercus phillyraeoides*	AB650391
Eudicotyledons	Fagaceae	*Quercus*	*Quercus phillyraeoides*	AB650392
Eudicotyledons	Fagaceae	*Quercus*	*Quercus phillyraeoides*	AB650393
Eudicotyledons	Fagaceae	*Quercus*	*Quercus phillyraeoides*	AB650394
Eudicotyledons	Fagaceae	*Quercus*	*Quercus phillyraeoides*	AB650395
Eudicotyledons	Fagaceae	*Quercus*	*Quercus phillyraeoides*	AB650396
Eudicotyledons	Fagaceae	*Quercus*	*Quercus phillyraeoides*	AB650397
Eudicotyledons	Fagaceae	*Quercus*	*Quercus phillyraeoides*	AB650398
Eudicotyledons	Fagaceae	*Quercus*	*Quercus phillyraeoides*	AB650399
Eudicotyledons	Fagaceae	*Quercus*	*Quercus phillyraeoides*	AB650400
Eudicotyledons	Fagaceae	*Quercus*	*Quercus phillyraeoides*	AB650401
Eudicotyledons	Fagaceae	*Quercus*	*Quercus phillyraeoides*	AB650402
Eudicotyledons	Fagaceae	*Quercus*	*Quercus phillyraeoides*	AB650403
Eudicotyledons	Fagaceae	*Quercus*	*Quercus phillyraeoides*	AB650404
Eudicotyledons	Fagaceae	*Quercus*	*Quercus phillyraeoides*	AB650405
Eudicotyledons	Fagaceae	*Quercus*	*Quercus phillyraeoides*	AB650406
Eudicotyledons	Fagaceae	*Quercus*	*Quercus phillyraeoides*	AB650407
Eudicotyledons	Fagaceae	*Quercus*	*Quercus phillyraeoides*	AB650408
Eudicotyledons	Fagaceae	*Quercus*	*Quercus phillyraeoides*	AB650409
Eudicotyledons	Fagaceae	*Quercus*	*Quercus phillyraeoides*	AB650410
Eudicotyledons	Fagaceae	*Quercus*	*Quercus phillyraeoides*	AB650411
Eudicotyledons	Fagaceae	*Quercus*	*Quercus phillyraeoides*	AB650412
Eudicotyledons	Fagaceae	*Quercus*	*Quercus phillyraeoides*	AB650413
Eudicotyledons	Fagaceae	*Quercus*	*Quercus phillyraeoides*	AB650414
Eudicotyledons	Fagaceae	*Quercus*	*Quercus phillyraeoides*	AB650415
Eudicotyledons	Fagaceae	*Quercus*	*Quercus phillyraeoides*	AB650416
Eudicotyledons	Fagaceae	*Quercus*	*Quercus phillyraeoides*	AB650417
Eudicotyledons	Fagaceae	*Quercus*	*Quercus phillyraeoides*	AB650418

Eudicotyledons	Fagaceae	*Quercus*	*Quercus phillyraeoides*	AB650419
Eudicotyledons	Fagaceae	*Quercus*	*Quercus phillyraeoides*	AB650420
Eudicotyledons	Fagaceae	*Quercus*	*Quercus phillyraeoides*	AB650421
Eudicotyledons	Fagaceae	*Quercus*	*Quercus phillyraeoides*	AB650422
Eudicotyledons	Fagaceae	*Quercus*	*Quercus phillyraeoides*	AB650423
Eudicotyledons	Fagaceae	*Quercus*	*Quercus phillyraeoides*	AB650424
Eudicotyledons	Fagaceae	*Quercus*	*Quercus phillyraeoides*	AB650425
Eudicotyledons	Fagaceae	*Quercus*	*Quercus phillyraeoides*	AB650426
Eudicotyledons	Fagaceae	*Quercus*	*Quercus phillyraeoides*	AB650427
Eudicotyledons	Fagaceae	*Quercus*	*Quercus phillyraeoides*	AB650428
Eudicotyledons	Fagaceae	*Quercus*	*Quercus phillyraeoides*	AB650429
Eudicotyledons	Fagaceae	*Quercus*	*Quercus phillyraeoides*	AB650430
Eudicotyledons	Fagaceae	*Quercus*	*Quercus phillyraeoides*	AB650431
Eudicotyledons	Fagaceae	*Quercus*	*Quercus phillyraeoides*	AB650432
Eudicotyledons	Fagaceae	*Quercus*	*Quercus phillyraeoides*	AB650433
Eudicotyledons	Fagaceae	*Quercus*	*Quercus phillyraeoides*	AB650434
Eudicotyledons	Fagaceae	*Quercus*	*Quercus phillyraeoides*	AB650435
Eudicotyledons	Fagaceae	*Quercus*	*Quercus phillyraeoides*	AB650436
Eudicotyledons	Fagaceae	*Quercus*	*Quercus phillyraeoides*	AB650437
Eudicotyledons	Fagaceae	*Quercus*	*Quercus phillyraeoides*	AB650438
Eudicotyledons	Fagaceae	*Quercus*	*Quercus phillyraeoides*	AB650439
Eudicotyledons	Fagaceae	*Quercus*	*Quercus phillyraeoides*	AB650440
Eudicotyledons	Fagaceae	*Quercus*	*Quercus phillyraeoides*	AB650441
Eudicotyledons	Fagaceae	*Quercus*	*Quercus phillyraeoides*	AB650442
Eudicotyledons	Fagaceae	*Quercus*	*Quercus phillyraeoides*	AB650443
Eudicotyledons	Fagaceae	*Quercus*	*Quercus phillyraeoides*	AB650444
Eudicotyledons	Fagaceae	*Quercus*	*Quercus phillyraeoides*	AB650445
Eudicotyledons	Fagaceae	*Quercus*	*Quercus phillyraeoides*	AB650446
Eudicotyledons	Fagaceae	*Quercus*	*Quercus phillyraeoides*	AB650447
Eudicotyledons	Fagaceae	*Quercus*	*Quercus phillyraeoides*	AB650448
Eudicotyledons	Fagaceae	*Quercus*	*Quercus phillyraeoides*	AB650449
Eudicotyledons	Fagaceae	*Quercus*	*Quercus phillyraeoides*	AB650450
Eudicotyledons	Fagaceae	*Quercus*	*Quercus phillyraeoides*	AB650451
Eudicotyledons	Fagaceae	*Quercus*	*Quercus phillyraeoides*	AB650452
Eudicotyledons	Fagaceae	*Quercus*	*Quercus phillyraeoides*	AB650453
Eudicotyledons	Fagaceae	*Quercus*	*Quercus phillyraeoides*	AB650454
Eudicotyledons	Fagaceae	*Quercus*	*Quercus phillyraeoides*	AB650455
Eudicotyledons	Fagaceae	*Quercus*	*Quercus phillyraeoides*	AB650456
Eudicotyledons	Fagaceae	*Quercus*	*Quercus phillyraeoides*	AB650457
Eudicotyledons	Fagaceae	*Quercus*	*Quercus phillyraeoides*	AB650458
Eudicotyledons	Fagaceae	*Quercus*	*Quercus phillyraeoides*	AB650459
Eudicotyledons	Fagaceae	*Quercus*	*Quercus phillyraeoides*	AB650460
Eudicotyledons	Fagaceae	*Quercus*	*Quercus phillyraeoides*	AB650461
Eudicotyledons	Fagaceae	*Quercus*	*Quercus phillyraeoides*	AB650462
Eudicotyledons	Fagaceae	*Quercus*	*Quercus phillyraeoides*	AB650463
Eudicotyledons	Fagaceae	*Quercus*	*Quercus phillyraeoides*	AB650464
Eudicotyledons	Fagaceae	*Quercus*	*Quercus phillyraeoides*	AB650465
Eudicotyledons	Rosaceae	*Sorbaria*	*Sorbaria sorbifolia*	JN047241
Eudicotyledons	Rubiaceae	*Galium*	*Galium mollugo*	FJ395515
Eudicotyledons	Amaranthaceae	*Chenopodium*	*Chenopodium album*	JN044279
Eudicotyledons	Polygonaceae	*Rumex*	*Rumex acetosa*	FJ395473
Eudicotyledons	Asteraceae	*Cirsium*	*Cirsium arvense*	FJ395528
Eudicotyledons	Solanaceae	*Solanum*	*Solanum dulcamara*	FJ395554

Eudicotyledons	Aquifoliaceae	*Ilex*	*Ilex latifolia*	JN044947
Eudicotyledons	Aquifoliaceae	*Ilex*	*Ilex latifolia*	JN044948
Eudicotyledons	Aquifoliaceae	*Ilex*	*Ilex latifolia*	JN044949
Eudicotyledons	Aquifoliaceae	*Ilex*	*Ilex latifolia*	JN044950
Eudicotyledons	Fabaceae	*Trifolium*	*Trifolium pratense*	FJ395480
Eudicotyledons	Rosaceae	*Rosa*	*Rosa canina*	FJ395548
Eudicotyledons	Asteraceae	*Jacobaea*	*Jacobaea vulgaris*	FJ395449
Eudicotyledons	Rosaceae	*Prunus*	*Prunus spinosa*	FJ395549
Eudicotyledons	Rosaceae	*Prunus*	*Prunus spinosa*	FR865110
Eudicotyledons	Asteraceae	*Arctium*	*Arctium minus*	FJ395501
Eudicotyledons	Polygonaceae	*Rumex*	*Rumex crispus*	FJ395508
Eudicotyledons	Polygonaceae	*Rumex*	*Rumex crispus*	JN047048
Eudicotyledons	Polygonaceae	*Rumex*	*Rumex crispus*	JN047049
Eudicotyledons	Polygonaceae	*Rumex*	*Rumex nepalensis*	JN047051
Eudicotyledons	Polygonaceae	*Rumex*	*Rumex nepalensis*	JN047052
Eudicotyledons	Grossulariaceae	*Ribes*	*Ribes rubrum*	FJ395461
Eudicotyledons	Aquifoliaceae	*Ilex*	*Ilex kaushue*	JN044944
Eudicotyledons	Aquifoliaceae	*Ilex*	*Ilex kaushue*	JN044945
Eudicotyledons	Aquifoliaceae	*Ilex*	*Ilex kaushue*	JN044946
Eudicotyledons	Aquifoliaceae	*Ilex*	*Ilex pentagona*	JN044951
Eudicotyledons	Aquifoliaceae	*Ilex*	*Ilex pentagona*	JN044952
Eudicotyledons	Aquifoliaceae	*Ilex*	*Ilex pentagona*	JN044953
Eudicotyledons	Asteraceae	*Sonchus*	*Sonchus asper*	FJ395510
Eudicotyledons	Polygonaceae	*Polygonum*	*Polygonum aviculare*	FJ395458
Eudicotyledons	Apiaceae	*Pleurospermum*	*Pleurospermum giraldii*	JN046389
Eudicotyledons	Apiaceae	*Pleurospermum*	*Pleurospermum giraldii*	JN046390
Eudicotyledons	Apiaceae	*Pleurospermum*	*Pleurospermum giraldii*	JN046391
Eudicotyledons	Apiaceae	*Pleurospermum*	*Pleurospermum giraldii*	JN046392
Eudicotyledons	Zygophyllaceae	*Larrea*	*Larrea tridentata*	JF266708
Eudicotyledons	Zygophyllaceae	*Larrea*	*Larrea tridentata*	JF266709
Eudicotyledons	Zygophyllaceae	*Larrea*	*Larrea tridentata*	JF266710
Eudicotyledons	Zygophyllaceae	*Larrea*	*Larrea tridentata*	JF266711
Eudicotyledons	Zygophyllaceae	*Larrea*	*Larrea tridentata*	JF266712
Eudicotyledons	Zygophyllaceae	*Larrea*	*Larrea tridentata*	JF266713
Eudicotyledons	Zygophyllaceae	*Larrea*	*Larrea tridentata*	JF266714
Eudicotyledons	Zygophyllaceae	*Larrea*	*Larrea tridentata*	JF266715
Eudicotyledons	Zygophyllaceae	*Larrea*	*Larrea tridentata*	JF266716
Eudicotyledons	Zygophyllaceae	*Larrea*	*Larrea tridentata*	JF266717
Eudicotyledons	Zygophyllaceae	*Larrea*	*Larrea tridentata*	JF266718
Eudicotyledons	Zygophyllaceae	*Larrea*	*Larrea tridentata*	JF266719
Eudicotyledons	Zygophyllaceae	*Larrea*	*Larrea tridentata*	JF266720
Eudicotyledons	Zygophyllaceae	*Larrea*	*Larrea tridentata*	JF266721
Eudicotyledons	Zygophyllaceae	*Larrea*	*Larrea tridentata*	JF266722
Eudicotyledons	Zygophyllaceae	*Larrea*	*Larrea tridentata*	JF266723
Eudicotyledons	Zygophyllaceae	*Larrea*	*Larrea tridentata*	JF266724
Eudicotyledons	Zygophyllaceae	*Larrea*	*Larrea tridentata*	JF266725
Eudicotyledons	Zygophyllaceae	*Larrea*	*Larrea tridentata*	JF266726
Eudicotyledons	Zygophyllaceae	*Larrea*	*Larrea tridentata*	JF266727
Eudicotyledons	Zygophyllaceae	*Larrea*	*Larrea tridentata*	JF266728
Eudicotyledons	Zygophyllaceae	*Larrea*	*Larrea tridentata*	JF266729
Eudicotyledons	Zygophyllaceae	*Larrea*	*Larrea tridentata*	JF266730
Eudicotyledons	Zygophyllaceae	*Larrea*	*Larrea tridentata*	JF266731
Eudicotyledons	Zygophyllaceae	*Larrea*	*Larrea tridentata*	JF266732

Eudicotyledons	Zygophyllaceae	*Larrea*	*Larrea tridentata*	JF266733
Eudicotyledons	Zygophyllaceae	*Larrea*	*Larrea tridentata*	JF266734
Eudicotyledons	Zygophyllaceae	*Larrea*	*Larrea tridentata*	JF266735
Eudicotyledons	Zygophyllaceae	*Larrea*	*Larrea tridentata*	JF266736
Eudicotyledons	Zygophyllaceae	*Larrea*	*Larrea tridentata*	JF266737
Eudicotyledons	Zygophyllaceae	*Larrea*	*Larrea tridentata*	JF266738
Eudicotyledons	Zygophyllaceae	*Larrea*	*Larrea tridentata*	JF266739
Eudicotyledons	Zygophyllaceae	*Larrea*	*Larrea tridentata*	JF266740
Eudicotyledons	Zygophyllaceae	*Larrea*	*Larrea tridentata*	JF266741
Eudicotyledons	Zygophyllaceae	*Larrea*	*Larrea tridentata*	JF266742
Eudicotyledons	Zygophyllaceae	*Larrea*	*Larrea tridentata*	JF266743
Eudicotyledons	Zygophyllaceae	*Larrea*	*Larrea tridentata*	JF266744
Eudicotyledons	Zygophyllaceae	*Larrea*	*Larrea tridentata*	JF266745
Eudicotyledons	Zygophyllaceae	*Larrea*	*Larrea tridentata*	JF266746
Eudicotyledons	Zygophyllaceae	*Larrea*	*Larrea tridentata*	JF266747
Eudicotyledons	Zygophyllaceae	*Larrea*	*Larrea tridentata*	JF266748
Eudicotyledons	Zygophyllaceae	*Larrea*	*Larrea tridentata*	JF266749
Eudicotyledons	Zygophyllaceae	*Larrea*	*Larrea tridentata*	JF266750
Eudicotyledons	Zygophyllaceae	*Larrea*	*Larrea tridentata*	JF266751
Eudicotyledons	Zygophyllaceae	*Larrea*	*Larrea tridentata*	JF266752
Eudicotyledons	Zygophyllaceae	*Larrea*	*Larrea tridentata*	JF266753
Eudicotyledons	Zygophyllaceae	*Larrea*	*Larrea tridentata*	JF266754
Eudicotyledons	Zygophyllaceae	*Larrea*	*Larrea tridentata*	JF266755
Eudicotyledons	Zygophyllaceae	*Larrea*	*Larrea tridentata*	JF266756
Eudicotyledons	Zygophyllaceae	*Larrea*	*Larrea tridentata*	JF266757
Eudicotyledons	Zygophyllaceae	*Larrea*	*Larrea tridentata*	JF266758
Eudicotyledons	Zygophyllaceae	*Larrea*	*Larrea tridentata*	JF266759
Eudicotyledons	Zygophyllaceae	*Larrea*	*Larrea tridentata*	JF266760
Eudicotyledons	Zygophyllaceae	*Larrea*	*Larrea tridentata*	JF266763
Eudicotyledons	Zygophyllaceae	*Larrea*	*Larrea tridentata*	JF266764
Eudicotyledons	Zygophyllaceae	*Larrea*	*Larrea tridentata*	JF266765
Eudicotyledons	Zygophyllaceae	*Larrea*	*Larrea tridentata*	JF266766
Eudicotyledons	Zygophyllaceae	*Larrea*	*Larrea tridentata*	JF266767
Eudicotyledons	Zygophyllaceae	*Larrea*	*Larrea tridentata*	JF266768
Eudicotyledons	Zygophyllaceae	*Larrea*	*Larrea tridentata*	JF266769
Eudicotyledons	Zygophyllaceae	*Larrea*	*Larrea tridentata*	JF266770
Eudicotyledons	Zygophyllaceae	*Larrea*	*Larrea tridentata*	JF266771
Eudicotyledons	Zygophyllaceae	*Larrea*	*Larrea tridentata*	JF266772
Eudicotyledons	Zygophyllaceae	*Larrea*	*Larrea tridentata*	JF266773
Eudicotyledons	Zygophyllaceae	*Larrea*	*Larrea tridentata*	JF266774
Eudicotyledons	Zygophyllaceae	*Larrea*	*Larrea tridentata*	JF266775
Eudicotyledons	Zygophyllaceae	*Larrea*	*Larrea tridentata*	JF266779
Eudicotyledons	Zygophyllaceae	*Larrea*	*Larrea tridentata*	JF266780
Eudicotyledons	Zygophyllaceae	*Larrea*	*Larrea tridentata*	JF266781
Eudicotyledons	Zygophyllaceae	*Larrea*	*Larrea tridentata*	JF266782
Eudicotyledons	Zygophyllaceae	*Larrea*	*Larrea tridentata*	JF266783
Eudicotyledons	Zygophyllaceae	*Larrea*	*Larrea tridentata*	JF266784
Eudicotyledons	Zygophyllaceae	*Larrea*	*Larrea tridentata*	JF266785
Eudicotyledons	Zygophyllaceae	*Larrea*	*Larrea tridentata*	JF266786
Eudicotyledons	Zygophyllaceae	*Larrea*	*Larrea tridentata*	JF266787
Eudicotyledons	Zygophyllaceae	*Larrea*	*Larrea tridentata*	JF266788
Eudicotyledons	Zygophyllaceae	*Larrea*	*Larrea tridentata*	JF266789
Eudicotyledons	Zygophyllaceae	*Larrea*	*Larrea tridentata*	JF266790

Eudicotyledons	Zygophyllaceae	*Larrea*	*Larrea tridentata*	JF266791
Eudicotyledons	Zygophyllaceae	*Larrea*	*Larrea tridentata*	JF266792
Eudicotyledons	Zygophyllaceae	*Larrea*	*Larrea tridentata*	JF266793
Eudicotyledons	Zygophyllaceae	*Larrea*	*Larrea tridentata*	JF266794
Eudicotyledons	Zygophyllaceae	*Larrea*	*Larrea tridentata*	JF266795
Eudicotyledons	Zygophyllaceae	*Larrea*	*Larrea tridentata*	JF266796
Eudicotyledons	Zygophyllaceae	*Larrea*	*Larrea tridentata*	JF266797
Eudicotyledons	Zygophyllaceae	*Larrea*	*Larrea tridentata*	JF266798
Eudicotyledons	Zygophyllaceae	*Larrea*	*Larrea tridentata*	JF266799
Eudicotyledons	Zygophyllaceae	*Larrea*	*Larrea tridentata*	JF266800
Eudicotyledons	Zygophyllaceae	*Larrea*	*Larrea tridentata*	JF266801
Eudicotyledons	Zygophyllaceae	*Larrea*	*Larrea tridentata*	JF266802
Eudicotyledons	Zygophyllaceae	*Larrea*	*Larrea tridentata*	JF266803
Eudicotyledons	Zygophyllaceae	*Larrea*	*Larrea tridentata*	JF266804
Eudicotyledons	Zygophyllaceae	*Larrea*	*Larrea tridentata*	JF266805
Eudicotyledons	Zygophyllaceae	*Larrea*	*Larrea tridentata*	JF266806
Eudicotyledons	Zygophyllaceae	*Larrea*	*Larrea tridentata*	JF266807
Eudicotyledons	Zygophyllaceae	*Larrea*	*Larrea tridentata*	JF266808
Eudicotyledons	Zygophyllaceae	*Larrea*	*Larrea tridentata*	JF266809
Eudicotyledons	Zygophyllaceae	*Larrea*	*Larrea tridentata*	JF266810
Eudicotyledons	Zygophyllaceae	*Larrea*	*Larrea tridentata*	JF266811
Eudicotyledons	Zygophyllaceae	*Larrea*	*Larrea tridentata*	JF266812
Eudicotyledons	Zygophyllaceae	*Larrea*	*Larrea tridentata*	JF266813
Eudicotyledons	Zygophyllaceae	*Larrea*	*Larrea tridentata*	JF266814
Eudicotyledons	Zygophyllaceae	*Larrea*	*Larrea tridentata*	JF266815
Eudicotyledons	Zygophyllaceae	*Larrea*	*Larrea tridentata*	JF266816
Eudicotyledons	Zygophyllaceae	*Larrea*	*Larrea tridentata*	JF266817
Eudicotyledons	Zygophyllaceae	*Larrea*	*Larrea tridentata*	JF266818
Eudicotyledons	Zygophyllaceae	*Larrea*	*Larrea tridentata*	JF266819
Eudicotyledons	Zygophyllaceae	*Larrea*	*Larrea nitida*	JF266822
Eudicotyledons	Zygophyllaceae	*Larrea*	*Larrea nitida*	JF266824
Monocotyledons	Juncaceae	*Juncus*	*Juncus effusus*	HQ596739
Monocotyledons	Juncaceae	*Juncus*	*Juncus effusus*	GQ434955
Monocotyledons	Juncaceae	*Juncus*	*Juncus effusus*	GQ434956
Monocotyledons	Arecaceae	*Sabal*	*Sabal minor*	GQ248388
Monocotyledons	Arecaceae	*Sabal*	*Sabal minor*	EF688519
Monocotyledons	Asparagaceae	*Polygonatum*	*Polygonatum humile*	JN046415
Monocotyledons	Asparagaceae	*Polygonatum*	*Polygonatum humile*	JN046416
Monocotyledons	Asparagaceae	*Polygonatum*	*Polygonatum humile*	JN046417
Monocotyledons	Asparagaceae	*Polygonatum*	*Polygonatum humile*	JN046418
Monocotyledons	Asparagaceae	*Maianthemum*	*Maianthemum racemosum*	HQ596763
Monocotyledons	Asparagaceae	*Maianthemum*	*Maianthemum racemosum*	EU850237
Monocotyledons	Asparagaceae	*Maianthemum*	*Maianthemum racemosum*	EU850238
Monocotyledons	Asparagaceae	*Maianthemum*	*Maianthemum racemosum*	JN045467
Monocotyledons	Asparagaceae	*Maianthemum*	*Maianthemum racemosum*	JN045468
Monocotyledons	Colchicaceae	*Colchicum*	*Colchicum autumnale*	JF934069
Monocotyledons	Bromeliaceae	*Aechmea*	*Aechmea allenii*	EF110654
Monocotyledons	Bromeliaceae	*Aechmea*	*Aechmea allenii*	HQ913722
Monocotyledons	Poaceae	*Elymus*	*Elymus repens*	FJ395519
Monocotyledons	Poaceae	*Elymus*	*Elymus repens*	HQ596680
Monocotyledons	Poaceae	*Elymus*	*Elymus repens*	EU531722
Monocotyledons	Poaceae	*Stipa*	*Stipa parviflora*	EU204777
Monocotyledons	Poaceae	*Stipa*	*Stipa parviflora*	EU204778

Monocotyledons	Poaceae	*Elymus*	*Elymus sibiricus*	HQ221837
Monocotyledons	Poaceae	*Elymus*	*Elymus sibiricus*	HQ221842
Monocotyledons	Poaceae	*Zizania*	*Zizania latifolia*	GU177392
Monocotyledons	Poaceae	*Zizania*	*Zizania latifolia*	GU177393
Monocotyledons	Poaceae	*Agrostis*	*Agrostis stolonifera*	HQ596586
Monocotyledons	Poaceae	*Agrostis*	*Agrostis stolonifera*	HQ596587
Monocotyledons	Cyperaceae	*Carex*	*Carex laxiflora*	HQ596627
Monocotyledons	Cyperaceae	*Carex*	*Carex laxiflora*	HQ596628
Monocotyledons	Poaceae	*Zizania*	*Zizania aquatica*	GU177368
Monocotyledons	Poaceae	*Zizania*	*Zizania aquatica*	GU177376
Monocotyledons	Poaceae	*Zizania*	*Zizania aquatica*	GU177377
Monocotyledons	Poaceae	*Zizania*	*Zizania aquatica*	GU177378
Monocotyledons	Poaceae	*Zizania*	*Zizania aquatica*	GU177379
Monocotyledons	Poaceae	*Zizania*	*Zizania aquatica*	GU177380
Monocotyledons	Poaceae	*Zizania*	*Zizania aquatica*	GU177381
Monocotyledons	Poaceae	*Zizania*	*Zizania aquatica*	GU177382
Monocotyledons	Poaceae	*Zizania*	*Zizania aquatica*	GU177386
Monocotyledons	Poaceae	*Zizania*	*Zizania aquatica*	GU177387
Monocotyledons	Poaceae	*Zizania*	*Zizania aquatica*	GU177388
Monocotyledons	Poaceae	*Zizania*	*Zizania aquatica*	GU177390
Monocotyledons	Orchidaceae	*Cymbidium*	*Cymbidium ensifolium*	FJ527765
Monocotyledons	Orchidaceae	*Cymbidium*	*Cymbidium ensifolium*	FJ527766
Monocotyledons	Asparagaceae	*Polygonatum*	*Polygonatum odoratum*	GQ434924
Monocotyledons	Asparagaceae	*Polygonatum*	*Polygonatum odoratum*	GQ434925
Monocotyledons	Asparagaceae	*Polygonatum*	*Polygonatum cirrhifolium*	EU850212
Monocotyledons	Asparagaceae	*Polygonatum*	*Polygonatum cirrhifolium*	JN046400
Monocotyledons	Asparagaceae	*Polygonatum*	*Polygonatum cirrhifolium*	JN046401
Monocotyledons	Asparagaceae	*Polygonatum*	*Polygonatum cirrhifolium*	JN046402
Monocotyledons	Asparagaceae	*Polygonatum*	*Polygonatum cirrhifolium*	JN046403
Monocotyledons	Asparagaceae	*Polygonatum*	*Polygonatum cirrhifolium*	JN046404
Monocotyledons	Asparagaceae	*Polygonatum*	*Polygonatum cirrhifolium*	JN046405
Monocotyledons	Asparagaceae	*Polygonatum*	*Polygonatum cirrhifolium*	JN046406
Monocotyledons	Asparagaceae	*Polygonatum*	*Polygonatum cirrhifolium*	JN046407
Monocotyledons	Asparagaceae	*Polygonatum*	*Polygonatum cirrhifolium*	JN046408
Monocotyledons	Asparagaceae	*Polygonatum*	*Polygonatum cirrhifolium*	JN046409
Monocotyledons	Asparagaceae	*Polygonatum*	*Polygonatum cirrhifolium*	JN046410
Monocotyledons	Asparagaceae	*Polygonatum*	*Polygonatum cirrhifolium*	JN046411
Monocotyledons	Asparagaceae	*Polygonatum*	*Polygonatum cirrhifolium*	JN046412
Monocotyledons	Asparagaceae	*Maianthemum*	*Maianthemum japonicum*	GQ434900
Monocotyledons	Asparagaceae	*Maianthemum*	*Maianthemum japonicum*	EU850231
Monocotyledons	Asparagaceae	*Maianthemum*	*Maianthemum japonicum*	EU850232
Monocotyledons	Asparagaceae	*Maianthemum*	*Maianthemum japonicum*	EU850240
Monocotyledons	Asparagaceae	*Maianthemum*	*Maianthemum japonicum*	JN045454
Monocotyledons	Asparagaceae	*Maianthemum*	*Maianthemum japonicum*	JN045455
Monocotyledons	Asparagaceae	*Maianthemum*	*Maianthemum japonicum*	JN045456
Monocotyledons	Asparagaceae	*Maianthemum*	*Maianthemum japonicum*	JN045457
Monocotyledons	Asparagaceae	*Maianthemum*	*Maianthemum japonicum*	JN045458
Monocotyledons	Asparagaceae	*Maianthemum*	*Maianthemum japonicum*	JN045459
Monocotyledons	Poaceae	*Zizania*	*Zizania palustris*	GU177369
Monocotyledons	Poaceae	*Zizania*	*Zizania palustris*	GU177370
Monocotyledons	Poaceae	*Zizania*	*Zizania palustris*	GU177371
Monocotyledons	Poaceae	*Zizania*	*Zizania palustris*	GU177372
Monocotyledons	Poaceae	*Zizania*	*Zizania palustris*	GU177373

Monocotyledons	Poaceae	*Zizania*	*Zizania palustris*	GU177374
Monocotyledons	Poaceae	*Zizania*	*Zizania palustris*	GU177375
Monocotyledons	Poaceae	*Zizania*	*Zizania palustris*	GU177383
Monocotyledons	Poaceae	*Zizania*	*Zizania palustris*	GU177384
Monocotyledons	Poaceae	*Zizania*	*Zizania palustris*	GU177385
Monocotyledons	Poaceae	*Zizania*	*Zizania palustris*	GU177389
Monocotyledons	Bromeliaceae	*Aechmea*	*Aechmea distichantha*	JN204613
Monocotyledons	Bromeliaceae	*Aechmea*	*Aechmea distichantha*	JN204614
Monocotyledons	Zingiberaceae	*Gagnepainia*	*Gagnepainia godefroyi*	GQ386049
Monocotyledons	Zingiberaceae	*Gagnepainia*	*Gagnepainia godefroyi*	GQ386050
Monocotyledons	Arecaceae	*Arenga*	*Arenga hookeriana*	JF345017
Monocotyledons	Arecaceae	*Arenga*	*Arenga hookeriana*	JF345025
Monocotyledons	Arecaceae	*Arenga*	*Arenga hookeriana*	JF345026
Monocotyledons	Arecaceae	*Arenga*	*Arenga hookeriana*	JF345027
Monocotyledons	Arecaceae	*Arenga*	*Arenga hookeriana*	JF345028
Monocotyledons	Arecaceae	*Wallichia*	*Wallichia disticha*	JF345073
Monocotyledons	Arecaceae	*Wallichia*	*Wallichia disticha*	JF345074
Monocotyledons	Orchidaceae	*Cischweinfia*	*Cischweinfia dasyandra*	FJ564133
Monocotyledons	Orchidaceae	*Cischweinfia*	*Cischweinfia dasyandra*	FJ564646
Monocotyledons	Orchidaceae	*Cuitlauzina*	*Cuitlauzina pendula*	FJ564076
Monocotyledons	Orchidaceae	*Cuitlauzina*	*Cuitlauzina pendula*	FJ564664
Monocotyledons	Orchidaceae	*Fernandezia*	*Fernandezia ionanthera*	FJ564101
Monocotyledons	Orchidaceae	*Fernandezia*	*Fernandezia ionanthera*	FJ564527
Monocotyledons	Orchidaceae	*Lockhartia*	*Lockhartia oerstedii*	EU213746
Monocotyledons	Orchidaceae	*Lockhartia*	*Lockhartia oerstedii*	FJ563978
Monocotyledons	Arecaceae	*Arenga*	*Arenga caudata*	JF345007
Monocotyledons	Arecaceae	*Arenga*	*Arenga caudata*	JF345008
Monocotyledons	Arecaceae	*Arenga*	*Arenga caudata*	JF345009
Monocotyledons	Arecaceae	*Arenga*	*Arenga caudata*	JF345010
Monocotyledons	Arecaceae	*Arenga*	*Arenga caudata*	JF345011
Monocotyledons	Arecaceae	*Arenga*	*Arenga caudata*	JF345012
Monocotyledons	Arecaceae	*Arenga*	*Arenga caudata*	JF345013
Monocotyledons	Arecaceae	*Arenga*	*Arenga caudata*	JF345014
Monocotyledons	Arecaceae	*Arenga*	*Arenga caudata*	JF345015
Monocotyledons	Arecaceae	*Arenga*	*Arenga caudata*	JF345016
Monocotyledons	Arecaceae	*Arenga*	*Arenga undulatifolia*	JF345039
Monocotyledons	Arecaceae	*Arenga*	*Arenga undulatifolia*	JF345040
Monocotyledons	Orchidaceae	*Prosthechea*	*Prosthechea cochleata*	EU213757
Monocotyledons	Orchidaceae	*Prosthechea*	*Prosthechea cochleata*	EU213758
Monocotyledons	Arecaceae	*Hyphaene*	*Hyphaene coriacea*	EU213775
Monocotyledons	Arecaceae	*Hyphaene*	*Hyphaene coriacea*	EU213776
Monocotyledons	Arecaceae	*Hyphaene*	*Hyphaene coriacea*	EU213777
Monocotyledons	Arecaceae	*Hyphaene*	*Hyphaene coriacea*	EU213778
Monocotyledons	Orchidaceae	*Rhynchostele*	*Rhynchostele beloglossa*	EU213707
Monocotyledons	Orchidaceae	*Rhynchostele*	*Rhynchostele beloglossa*	FJ564093
Monocotyledons	Orchidaceae	*Rhynchostele*	*Rhynchostele beloglossa*	FJ564134
Monocotyledons	Orchidaceae	*Comparettia*	*Comparettia macroplectron*	FJ564052
Monocotyledons	Orchidaceae	*Comparettia*	*Comparettia macroplectron*	FJ564656
Monocotyledons	Orchidaceae	*Cyrtochilum*	*Cyrtochilum cimiciferum*	FJ563997
Monocotyledons	Orchidaceae	*Cyrtochilum*	*Cyrtochilum cimiciferum*	FJ564239
Monocotyledons	Orchidaceae	*Cyrtochilum*	*Cyrtochilum cimiciferum*	FJ564518
Monocotyledons	Orchidaceae	*Cyrtochilum*	*Cyrtochilum edwardii*	FJ564091
Monocotyledons	Orchidaceae	*Cyrtochilum*	*Cyrtochilum edwardii*	FJ564669

Monocotyledons	Orchidaceae	*Erycina*	*Erycina crista-galli*	EU213751
Monocotyledons	Orchidaceae	*Erycina*	*Erycina crista-galli*	FJ564144
Monocotyledons	Orchidaceae	*Erycina*	*Erycina echinata*	FJ564063
Monocotyledons	Orchidaceae	*Erycina*	*Erycina echinata*	FJ564434
Monocotyledons	Orchidaceae	*Erycina*	*Erycina hyalinobulbon*	FJ564026
Monocotyledons	Orchidaceae	*Erycina*	*Erycina hyalinobulbon*	FJ564435
Monocotyledons	Orchidaceae	*Erycina*	*Erycina pumilio*	EU213737
Monocotyledons	Orchidaceae	*Erycina*	*Erycina pumilio*	EU213738
Monocotyledons	Orchidaceae	*Erycina*	*Erycina pumilio*	FJ564149
Monocotyledons	Orchidaceae	*Erycina*	*Erycina pumilio*	FJ564556
Monocotyledons	Orchidaceae	*Erycina*	*Erycina pusilla*	FJ564021
Monocotyledons	Orchidaceae	*Erycina*	*Erycina pusilla*	FJ564542
Monocotyledons	Orchidaceae	*Ionopsis*	*Ionopsis minutiflora*	FJ564136
Monocotyledons	Orchidaceae	*Ionopsis*	*Ionopsis minutiflora*	FJ564462
Monocotyledons	Orchidaceae	*Ionopsis*	*Ionopsis minutiflora*	FJ564565
Monocotyledons	Orchidaceae	*Ionopsis*	*Ionopsis satyrioides*	FJ564135
Monocotyledons	Orchidaceae	*Ionopsis*	*Ionopsis satyrioides*	FJ564266
Monocotyledons	Orchidaceae	*Ionopsis*	*Ionopsis utricularioides*	FJ564022
Monocotyledons	Orchidaceae	*Ionopsis*	*Ionopsis utricularioides*	FJ564559
Monocotyledons	Orchidaceae	*Gomesa*	*Gomesa flexuosa*	FJ564049
Monocotyledons	Orchidaceae	*Gomesa*	*Gomesa flexuosa*	FJ564546
Monocotyledons	Orchidaceae	*Gomesa*	*Gomesa flexuosa*	FJ564662
Monocotyledons	Orchidaceae	*Rhynchostele*	*Rhynchostele bictoniensis*	GQ248385
Monocotyledons	Orchidaceae	*Rhynchostele*	*Rhynchostele bictoniensis*	EU213764
Monocotyledons	Orchidaceae	*Rhynchostele*	*Rhynchostele bictoniensis*	FJ564004
Monocotyledons	Orchidaceae	*Tolumnia*	*Tolumnia calochila*	FJ564087
Monocotyledons	Orchidaceae	*Tolumnia*	*Tolumnia calochila*	FJ564232
Monocotyledons	Orchidaceae	*Trichopilia*	*Trichopilia subulata*	FJ564073
Monocotyledons	Orchidaceae	*Trichopilia*	*Trichopilia subulata*	FJ564550
Monocotyledons	Orchidaceae	*Cyrtochilum*	*Cyrtochilum angustatum*	FJ564068
Monocotyledons	Orchidaceae	*Cyrtochilum*	*Cyrtochilum angustatum*	FJ564260
Monocotyledons	Orchidaceae	*Cyrtochilum*	*Cyrtochilum pardinum*	FJ564092
Monocotyledons	Orchidaceae	*Cyrtochilum*	*Cyrtochilum pardinum*	FJ564170
Monocotyledons	Orchidaceae	*Cyrtochilum*	*Cyrtochilum ramosissimum*	FJ564056
Monocotyledons	Orchidaceae	*Cyrtochilum*	*Cyrtochilum ramosissimum*	FJ564483
Monocotyledons	Orchidaceae	*Cyrtochilum*	*Cyrtochilum tricostatum*	FJ564086
Monocotyledons	Orchidaceae	*Cyrtochilum*	*Cyrtochilum tricostatum*	FJ564102
Monocotyledons	Orchidaceae	*Cyrtochilum*	*Cyrtochilum tricostatum*	FJ564151
Monocotyledons	Orchidaceae	*Cyrtochilum*	*Cyrtochilum tricostatum*	FJ564243
Monocotyledons	Orchidaceae	*Cyrtochilum*	*Cyrtochilum tricostatum*	FJ564247
Monocotyledons	Orchidaceae	*Cyrtochilum*	*Cyrtochilum tricostatum*	FJ564486
Monocotyledons	Orchidaceae	*Miltoniopsis*	*Miltoniopsis vexillaria*	FJ564060
Monocotyledons	Orchidaceae	*Miltoniopsis*	*Miltoniopsis vexillaria*	FJ564341
Monocotyledons	Poaceae	*Paspalum*	*Paspalum quadrifarium*	AY941123
Monocotyledons	Poaceae	*Paspalum*	*Paspalum quadrifarium*	AY941127
Monocotyledons	Orchidaceae	*Caucaea*	*Caucaea cucullata*	FJ564196
Monocotyledons	Orchidaceae	*Caucaea*	*Caucaea cucullata*	FJ564530
Monocotyledons	Orchidaceae	*Caucaea*	*Caucaea phalaenopsis*	FJ564006
Monocotyledons	Orchidaceae	*Caucaea*	*Caucaea phalaenopsis*	FJ564529
Monocotyledons	Orchidaceae	*Caucaea*	*Caucaea radiata*	FJ564281
Monocotyledons	Orchidaceae	*Caucaea*	*Caucaea radiata*	FJ564582
Monocotyledons	Orchidaceae	*Cyrtochilum*	*Cyrtochilum meirax*	FJ564444
Monocotyledons	Orchidaceae	*Cyrtochilum*	*Cyrtochilum meirax*	FJ564608

Monocotyledons	Orchidaceae	*Miltoniopsis*	*Miltoniopsis bismarkii*	FJ564469
Monocotyledons	Orchidaceae	*Miltoniopsis*	*Miltoniopsis bismarkii*	FJ564619
Monocotyledons	Asparagaceae	*Maianthemum*	*Maianthemum bifolium*	EU850234
Monocotyledons	Asparagaceae	*Maianthemum*	*Maianthemum bifolium*	EU850235
Monocotyledons	Asparagaceae	*Maianthemum*	*Maianthemum bifolium*	JN045441
Monocotyledons	Asparagaceae	*Maianthemum*	*Maianthemum bifolium*	JN045442
Monocotyledons	Asparagaceae	*Maianthemum*	*Maianthemum bifolium*	JN045443
Monocotyledons	Zingiberaceae	*Gagnepainia*	*Gagnepainia thoreliana*	GQ386051
Monocotyledons	Zingiberaceae	*Gagnepainia*	*Gagnepainia thoreliana*	GQ386052
Monocotyledons	Zingiberaceae	*Gagnepainia*	*Gagnepainia thoreliana*	GQ386053
Monocotyledons	Zingiberaceae	*Gagnepainia*	*Gagnepainia thoreliana*	GQ386054
Monocotyledons	Poaceae	*Dendrocalamus*	*Dendrocalamus strictus*	GU391004
Monocotyledons	Poaceae	*Dendrocalamus*	*Dendrocalamus strictus*	GU063120
Monocotyledons	Orchidaceae	*Prosthechea*	*Prosthechea fragrans*	EU213759
Monocotyledons	Orchidaceae	*Prosthechea*	*Prosthechea fragrans*	EU213760
Monocotyledons	Orchidaceae	*Cyrtochilum*	*Cyrtochilum serratum*	FJ564014
Monocotyledons	Orchidaceae	*Cyrtochilum*	*Cyrtochilum serratum*	FJ564123
Monocotyledons	Asparagaceae	*Polygonatum*	*Polygonatum acuminatifolium*	JN046398
Monocotyledons	Asparagaceae	*Polygonatum*	*Polygonatum acuminatifolium*	JN046399
Monocotyledons	Asparagaceae	*Polygonatum*	*Polygonatum sibiricum*	GQ434879
Monocotyledons	Asparagaceae	*Polygonatum*	*Polygonatum sibiricum*	GQ434880
Monocotyledons	Asparagaceae	*Polygonatum*	*Polygonatum sibiricum*	EU850210
Monocotyledons	Asparagaceae	*Polygonatum*	*Polygonatum verticillatum*	JN046419
Monocotyledons	Asparagaceae	*Polygonatum*	*Polygonatum verticillatum*	JN046420
Monocotyledons	Asparagaceae	*Polygonatum*	*Polygonatum verticillatum*	JN046421
Monocotyledons	Asparagaceae	*Polygonatum*	*Polygonatum verticillatum*	JN046422
Monocotyledons	Asparagaceae	*Maianthemum*	*Maianthemum atropurpureum*	EU850218
Monocotyledons	Asparagaceae	*Maianthemum*	*Maianthemum atropurpureum*	EU850219
Monocotyledons	Asparagaceae	*Maianthemum*	*Maianthemum atropurpureum*	EU850223
Monocotyledons	Asparagaceae	*Maianthemum*	*Maianthemum atropurpureum*	JN045433
Monocotyledons	Asparagaceae	*Maianthemum*	*Maianthemum atropurpureum*	JN045434
Monocotyledons	Asparagaceae	*Maianthemum*	*Maianthemum atropurpureum*	JN045435
Monocotyledons	Asparagaceae	*Maianthemum*	*Maianthemum atropurpureum*	JN045436
Monocotyledons	Asparagaceae	*Maianthemum*	*Maianthemum atropurpureum*	JN045437
Monocotyledons	Asparagaceae	*Maianthemum*	*Maianthemum atropurpureum*	JN045438
Monocotyledons	Asparagaceae	*Maianthemum*	*Maianthemum atropurpureum*	JN045439
Monocotyledons	Asparagaceae	*Maianthemum*	*Maianthemum atropurpureum*	JN045440
Monocotyledons	Asparagaceae	*Maianthemum*	*Maianthemum paniculatum*	EU850242
Monocotyledons	Asparagaceae	*Maianthemum*	*Maianthemum paniculatum*	EU850243
Monocotyledons	Asparagaceae	*Maianthemum*	*Maianthemum purpureum*	EU850221
Monocotyledons	Asparagaceae	*Maianthemum*	*Maianthemum purpureum*	JN045460
Monocotyledons	Asparagaceae	*Maianthemum*	*Maianthemum purpureum*	JN045461
Monocotyledons	Asparagaceae	*Maianthemum*	*Maianthemum purpureum*	JN045462
Monocotyledons	Asparagaceae	*Maianthemum*	*Maianthemum purpureum*	JN045463
Monocotyledons	Asparagaceae	*Maianthemum*	*Maianthemum purpureum*	JN045464
Monocotyledons	Asparagaceae	*Maianthemum*	*Maianthemum purpureum*	JN045465
Monocotyledons	Asparagaceae	*Maianthemum*	*Maianthemum purpureum*	JN045466
Monocotyledons	Asparagaceae	*Maianthemum*	*Maianthemum tatsienense*	EU850228
Monocotyledons	Asparagaceae	*Maianthemum*	*Maianthemum tatsienense*	JN045469
Monocotyledons	Asparagaceae	*Maianthemum*	*Maianthemum tatsienense*	JN045470
Monocotyledons	Asparagaceae	*Maianthemum*	*Maianthemum tatsienense*	JN045471
Monocotyledons	Asparagaceae	*Maianthemum*	*Maianthemum tatsienense*	JN045472
Monocotyledons	Asparagaceae	*Maianthemum*	*Maianthemum tatsienense*	JN045473

Monocotyledons	Asparagaceae	*Maianthemum*	*Maianthemum tatsienense*	JN045474
Monocotyledons	Asparagaceae	*Maianthemum*	*Maianthemum tubiferum*	EU850225
Monocotyledons	Asparagaceae	*Maianthemum*	*Maianthemum tubiferum*	JN045475
Monocotyledons	Colchicaceae	*Colchicum*	*Colchicum filifolium*	DQ088323
Monocotyledons	Colchicaceae	*Colchicum*	*Colchicum filifolium*	JF934102
Monocotyledons	Bromeliaceae	*Aechmea*	*Aechmea nudicaulis*	JN204618
Monocotyledons	Bromeliaceae	*Aechmea*	*Aechmea nudicaulis*	JN204619
Monocotyledons	Bromeliaceae	*Tillandsia*	*Tillandsia tenuifolia*	JN204667
Monocotyledons	Bromeliaceae	*Tillandsia*	*Tillandsia tenuifolia*	JN204668
Monocotyledons	Juncaceae	*Juncus*	*Juncus dudleyi*	HQ596737
Monocotyledons	Juncaceae	*Juncus*	*Juncus dudleyi*	HQ596738
Monocotyledons	Poaceae	*Elymus*	*Elymus brevipes*	HQ221851
Monocotyledons	Poaceae	*Elymus*	*Elymus brevipes*	HQ652802
Monocotyledons	Cyperaceae	*Carex*	*Carex hitchcockiana*	FJ597291
Monocotyledons	Cyperaceae	*Carex*	*Carex hitchcockiana*	FJ597292
Monocotyledons	Cyperaceae	*Carex*	*Carex hitchcockiana*	FJ597293
Monocotyledons	Cyperaceae	*Carex*	*Carex hitchcockiana*	FJ597294
Monocotyledons	Cyperaceae	*Carex*	*Carex oligocarpa*	FJ597295
Monocotyledons	Cyperaceae	*Carex*	*Carex oligocarpa*	FJ597296
Monocotyledons	Cyperaceae	*Carex*	*Carex oligocarpa*	DQ006177
Monocotyledons	Poaceae	*Paspalum*	*Paspalum dasypleurum*	AY769128
Monocotyledons	Poaceae	*Paspalum*	*Paspalum dasypleurum*	DQ104301
Monocotyledons	Poaceae	*Paspalum*	*Paspalum dilatatum*	AY769130
Monocotyledons	Poaceae	*Paspalum*	*Paspalum dilatatum*	DQ104295
Monocotyledons	Poaceae	*Paspalum*	*Paspalum dilatatum*	DQ104296
Monocotyledons	Poaceae	*Paspalum*	*Paspalum dilatatum*	DQ104297
Monocotyledons	Poaceae	*Paspalum*	*Paspalum dilatatum*	DQ104298
Monocotyledons	Poaceae	*Paspalum*	*Paspalum dilatatum*	DQ104299
Monocotyledons	Poaceae	*Paspalum*	*Paspalum pauciciliatum*	AY769144
Monocotyledons	Poaceae	*Paspalum*	*Paspalum pauciciliatum*	DQ104302
Monocotyledons	Poaceae	*Paspalum*	*Paspalum urvillei*	AY769148
Monocotyledons	Poaceae	*Paspalum*	*Paspalum urvillei*	DQ104300
Monocotyledons	Bromeliaceae	*Aechmea*	*Aechmea racinae*	EF110659
Monocotyledons	Bromeliaceae	*Aechmea*	*Aechmea racinae*	HQ913655
Monocotyledons	Cyperaceae	*Carex*	*Carex amphibola*	FJ597264
Monocotyledons	Cyperaceae	*Carex*	*Carex amphibola*	FJ597265
Monocotyledons	Cyperaceae	*Carex*	*Carex amphibola*	FJ597266
Monocotyledons	Cyperaceae	*Carex*	*Carex amphibola*	DQ006175
Monocotyledons	Poaceae	*Calamagrostis*	*Calamagrostis muiriana*	DQ113917
Monocotyledons	Poaceae	*Calamagrostis*	*Calamagrostis muiriana*	DQ113918
Monocotyledons	Poaceae	*Calamagrostis*	*Calamagrostis muiriana*	DQ113919
Monocotyledons	Poaceae	*Calamagrostis*	*Calamagrostis muiriana*	DQ113920
Monocotyledons	Poaceae	*Calamagrostis*	*Calamagrostis muiriana*	DQ113921
Monocotyledons	Poaceae	*Calamagrostis*	*Calamagrostis breweri*	DQ113922
Monocotyledons	Poaceae	*Calamagrostis*	*Calamagrostis breweri*	DQ113923
Monocotyledons	Poaceae	*Calamagrostis*	*Calamagrostis breweri*	DQ113924
Monocotyledons	Poaceae	*Calamagrostis*	*Calamagrostis breweri*	DQ113925
Monocotyledons	Poaceae	*Agrostis*	*Agrostis gigantea*	FJ395454
Monocotyledons	Poaceae	*Agrostis*	*Agrostis gigantea*	HQ596584
Monocotyledons	Poaceae	*Agrostis*	*Agrostis gigantea*	HQ596585
Monocotyledons	Orchidaceae	*Fernandezia*	*Fernandezia tica*	FJ564340
Monocotyledons	Orchidaceae	*Fernandezia*	*Fernandezia tica*	FJ564442
Monocotyledons	Poaceae	*Stipa*	*Stipa papposa*	EU489274

Monocotyledons	Poaceae	*Stipa*	*Stipa papposa*	EU204706
Monocotyledons	Poaceae	*Stipa*	*Stipa papposa*	EU204707
Monocotyledons	Poaceae	*Stipa*	*Stipa papposa*	EU204708
Monocotyledons	Poaceae	*Stipa*	*Stipa papposa*	EU204709
Monocotyledons	Poaceae	*Stipa*	*Stipa papposa*	EU204710
Monocotyledons	Poaceae	*Elymus*	*Elymus nutans*	HQ221835
Monocotyledons	Poaceae	*Elymus*	*Elymus nutans*	HQ221845
Monocotyledons	Poaceae	*Elymus*	*Elymus nutans*	HQ221847
Monocotyledons	Asparagaceae	*Maianthemum*	*Maianthemum canadense*	HQ596762
Monocotyledons	Asparagaceae	*Maianthemum*	*Maianthemum canadense*	EU850236
Monocotyledons	Orchidaceae	*Cuitlauzina*	*Cuitlauzina candida*	FJ564070
Monocotyledons	Orchidaceae	*Cuitlauzina*	*Cuitlauzina candida*	FJ564670
Monocotyledons	Orchidaceae	*Comparettia*	*Comparettia bennettii*	FJ564115
Monocotyledons	Orchidaceae	*Comparettia*	*Comparettia bennettii*	FJ564365
Monocotyledons	Orchidaceae	*Tsiorchis*	*Tsiorchis kimballiana*	HQ404447
Monocotyledons	Orchidaceae	*Tsiorchis*	*Tsiorchis kimballiana*	HQ404448
Monocotyledons	Orchidaceae	*Tsiorchis*	*Tsiorchis kimballiana*	HQ404449
Monocotyledons	Orchidaceae	*Tsiorchis*	*Tsiorchis kimballiana*	HQ404450
Monocotyledons	Orchidaceae	*Tsiorchis*	*Tsiorchis kimballiana*	HQ404451
Monocotyledons	Orchidaceae	*Tsiorchis*	*Tsiorchis kimballiana*	HQ404452
Monocotyledons	Bromeliaceae	*Aechmea*	*Aechmea lingulata*	EF110667
Monocotyledons	Bromeliaceae	*Aechmea*	*Aechmea lingulata*	JN204616
Monocotyledons	Bromeliaceae	*Aechmea*	*Aechmea lingulata*	JN204617
Monocotyledons	Bromeliaceae	*Aechmea*	*Aechmea lingulata*	HQ913652
Monocotyledons	Poaceae	*Calamagrostis*	*Calamagrostis erectifolia*	GQ248257
Monocotyledons	Poaceae	*Calamagrostis*	*Calamagrostis erectifolia*	EF590676
Monocotyledons	Arecaceae	*Sabal*	*Sabal etonia*	GQ248387
Monocotyledons	Arecaceae	*Sabal*	*Sabal etonia*	EF688515
Monocotyledons	Orchidaceae	*Paraholcoglossum*	*Paraholcoglossum subulifolium*	HQ404477
Monocotyledons	Orchidaceae	*Paraholcoglossum*	*Paraholcoglossum subulifolium*	HQ404478
Monocotyledons	Orchidaceae	*Paraholcoglossum*	*Paraholcoglossum subulifolium*	HQ404479
Monocotyledons	Orchidaceae	*Rhynchostele*	*Rhynchostele cordata*	GQ248386
Monocotyledons	Orchidaceae	*Rhynchostele*	*Rhynchostele cordata*	FJ564158
Monocotyledons	Orchidaceae	*Rhynchostele*	*Rhynchostele cordata*	FJ564666
Monocotyledons	Arecaceae	*Hyphaene*	*Hyphaene petersiana*	EU213779
Monocotyledons	Arecaceae	*Hyphaene*	*Hyphaene petersiana*	EU213780
Monocotyledons	Orchidaceae	*Prosthechea*	*Prosthechea radiata*	EU213761
Monocotyledons	Orchidaceae	*Prosthechea*	*Prosthechea radiata*	EU213762
Monocotyledons	Orchidaceae	*Prosthechea*	*Prosthechea radiata*	EU213763
Monocotyledons	Orchidaceae	*Trichopilia*	*Trichopilia tortilis*	EU213769
Monocotyledons	Orchidaceae	*Trichopilia*	*Trichopilia tortilis*	EU213770
Monocotyledons	Orchidaceae	*Trichopilia*	*Trichopilia tortilis*	EU213771
Monocotyledons	Orchidaceae	*Trichopilia*	*Trichopilia turialbae*	EU213772
Monocotyledons	Orchidaceae	*Trichopilia*	*Trichopilia turialbae*	EU213773
Monocotyledons	Orchidaceae	*Trichopilia*	*Trichopilia turialbae*	EU213774
Monocotyledons	Orchidaceae	*Trichopilia*	*Trichopilia turialbae*	FJ564132
Monocotyledons	Orchidaceae	*Trichopilia*	*Trichopilia turialbae*	FJ564632
Monocotyledons	Poaceae	*Gigantochloa*	*Gigantochloa scortechinii*	GU063129
Monocotyledons	Poaceae	*Gigantochloa*	*Gigantochloa scortechinii*	HQ697908
Monocotyledons	Poaceae	*Gigantochloa*	*Gigantochloa scortechinii*	HQ697909
Monocotyledons	Poaceae	*Gigantochloa*	*Gigantochloa scortechinii*	HQ697910
Monocotyledons	Poaceae	*Gigantochloa*	*Gigantochloa scortechinii*	HQ697911
Monocotyledons	Poaceae	*Gigantochloa*	*Gigantochloa scortechinii*	HQ697912

Monocotyledons	Orchidaceae	*Paraholcoglossun*	*Paraholcoglossum amesianum*	HQ404436
Monocotyledons	Orchidaceae	*Paraholcoglossun*	*Paraholcoglossum amesianum*	HQ404437
Monocotyledons	Orchidaceae	*Paraholcoglossun*	*Paraholcoglossum amesianum*	HQ404438
Monocotyledons	Orchidaceae	*Paraholcoglossun*	*Paraholcoglossum amesianum*	HQ404439
Monocotyledons	Orchidaceae	*Paraholcoglossun*	*Paraholcoglossum amesianum*	HQ404440
Monocotyledons	Orchidaceae	*Paraholcoglossun*	*Paraholcoglossum amesianum*	HQ404441
Monocotyledons	Orchidaceae	*Tsiorchis*	*Tsiorchis wangii*	HQ404480
Monocotyledons	Orchidaceae	*Tsiorchis*	*Tsiorchis wangii*	HQ404481
Monocotyledons	Orchidaceae	*Tsiorchis*	*Tsiorchis wangii*	HQ404482
Monocotyledons	Asparagaceae	*Maianthemum*	*Maianthemum dahuricum*	EU850233
Monocotyledons	Asparagaceae	*Maianthemum*	*Maianthemum dahuricum*	JN045444
Monocotyledons	Asparagaceae	*Maianthemum*	*Maianthemum gigas*	EU850241
Monocotyledons	Asparagaceae	*Maianthemum*	*Maianthemum gigas*	JN045445
Monocotyledons	Asparagaceae	*Maianthemum*	*Maianthemum gongshanense*	EU850214
Monocotyledons	Asparagaceae	*Maianthemum*	*Maianthemum gongshanense*	JN045446
Monocotyledons	Asparagaceae	*Maianthemum*	*Maianthemum henryi*	EU850213
Monocotyledons	Asparagaceae	*Maianthemum*	*Maianthemum henryi*	EU850217
Monocotyledons	Asparagaceae	*Maianthemum*	*Maianthemum henryi*	EU850224
Monocotyledons	Asparagaceae	*Maianthemum*	*Maianthemum henryi*	JN045447
Monocotyledons	Asparagaceae	*Maianthemum*	*Maianthemum henryi*	JN045448
Monocotyledons	Asparagaceae	*Maianthemum*	*Maianthemum henryi*	JN045449
Monocotyledons	Asparagaceae	*Maianthemum*	*Maianthemum henryi*	JN045450
Monocotyledons	Asparagaceae	*Maianthemum*	*Maianthemum henryi*	JN045451
Monocotyledons	Asparagaceae	*Maianthemum*	*Maianthemum henryi*	JN045452
Monocotyledons	Asparagaceae	*Maianthemum*	*Maianthemum henryi*	JN045453
Monocotyledons	Poaceae	*Dendrocalamus*	*Dendrocalamus pendulus*	HQ697902
Monocotyledons	Poaceae	*Dendrocalamus*	*Dendrocalamus pendulus*	HQ697903
Monocotyledons	Poaceae	*Dendrocalamus*	*Dendrocalamus pendulus*	HQ697904
Monocotyledons	Poaceae	*Gigantochloa*	*Gigantochloa balui*	GU391008
Monocotyledons	Poaceae	*Gigantochloa*	*Gigantochloa balui*	GU063125
Monocotyledons	Orchidaceae	*Gomesa*	*Gomesa colorata*	FJ564107
Monocotyledons	Orchidaceae	*Gomesa*	*Gomesa colorata*	EU935667
Monocotyledons	Orchidaceae	*Gomesa*	*Gomesa cornigera*	FJ564387
Monocotyledons	Orchidaceae	*Gomesa*	*Gomesa cornigera*	EU935680
Monocotyledons	Orchidaceae	*Gomesa*	*Gomesa echinata*	FJ564383
Monocotyledons	Orchidaceae	*Gomesa*	*Gomesa echinata*	EU935677
Monocotyledons	Orchidaceae	*Gomesa*	*Gomesa kautskyi*	FJ564400
Monocotyledons	Orchidaceae	*Gomesa*	*Gomesa kautskyi*	EU935686
Monocotyledons	Orchidaceae	*Gomesa*	*Gomesa lietzei*	FJ564638
Monocotyledons	Orchidaceae	*Gomesa*	*Gomesa lietzei*	EU935689
Monocotyledons	Orchidaceae	*Gomesa*	*Gomesa pubes*	FJ563995
Monocotyledons	Orchidaceae	*Gomesa*	*Gomesa pubes*	EU935681
Monocotyledons	Orchidaceae	*Tolumnia*	*Tolumnia pulchella*	FJ564265
Monocotyledons	Orchidaceae	*Tolumnia*	*Tolumnia pulchella*	EU935692
Monocotyledons	Orchidaceae	*Gomesa*	*Gomesa sarcodes*	FJ564399
Monocotyledons	Orchidaceae	*Gomesa*	*Gomesa sarcodes*	EU935682
Monocotyledons	Orchidaceae	*Gomesa*	*Gomesa silvana*	FJ564672
Monocotyledons	Orchidaceae	*Gomesa*	*Gomesa silvana*	EU935676
Monocotyledons	Orchidaceae	*Gomesa*	*Gomesa truncata*	FJ564384
Monocotyledons	Orchidaceae	*Gomesa*	*Gomesa truncata*	EU935691
Monocotyledons	Orchidaceae	*Gomesa*	*Gomesa venusta*	FJ564310
Monocotyledons	Orchidaceae	*Gomesa*	*Gomesa venusta*	EU935668
Monocotyledons	Orchidaceae	*Gomesa*	*Gomesa widgrenii*	FJ564671

Monocotyledons	Orchidaceae	*Gomesa*	*Gomesa widgrenii*	EU935679
Monocotyledons	Orchidaceae	*Gomesa*	*Gomesa barbata*	FJ564410
Monocotyledons	Orchidaceae	*Gomesa*	*Gomesa barbata*	EU935662
Monocotyledons	Orchidaceae	*Gomesa*	*Gomesa concolor*	FJ564261
Monocotyledons	Orchidaceae	*Gomesa*	*Gomesa concolor*	FJ564663
Monocotyledons	Orchidaceae	*Gomesa*	*Gomesa concolor*	EU935664
Monocotyledons	Orchidaceae	*Gomesa*	*Gomesa hydrophila*	FJ564396
Monocotyledons	Orchidaceae	*Gomesa*	*Gomesa hydrophila*	EU935665
Monocotyledons	Orchidaceae	*Erycina*	*Erycina glossomystax*	FJ564592
Monocotyledons	Orchidaceae	*Erycina*	*Erycina glossomystax*	FJ564593
Monocotyledons	Orchidaceae	*Erycina*	*Erycina glossomystax*	FJ564595
Monocotyledons	Orchidaceae	*Lockhartia*	*Lockhartia bennettii*	FJ563975
Monocotyledons	Orchidaceae	*Lockhartia*	*Lockhartia bennettii*	FJ564528
Monocotyledons	Orchidaceae	*Odontoglossum*	*Odontoglossum sanguineum*	FJ564028
Monocotyledons	Orchidaceae	*Odontoglossum*	*Odontoglossum sanguineum*	FJ564501
Monocotyledons	Orchidaceae	*Ornithocephalus*	*Ornithocephalus bicornis*	FJ564567
Monocotyledons	Orchidaceae	*Ornithocephalus*	*Ornithocephalus bicornis*	FJ564647
Monocotyledons	Orchidaceae	*Ornithocephalus*	*Ornithocephalus suarezii*	FJ564563
Monocotyledons	Orchidaceae	*Ornithocephalus*	*Ornithocephalus suarezii*	FJ564566
Monocotyledons	Orchidaceae	*Trichocentrum*	*Trichocentrum lindenii*	FJ564304
Monocotyledons	Orchidaceae	*Trichocentrum*	*Trichocentrum lindenii*	FJ564545
Monocotyledons	Orchidaceae	*Trichocentrum*	*Trichocentrum stipitatum*	FJ564126
Monocotyledons	Orchidaceae	*Trichocentrum*	*Trichocentrum stipitatum*	FJ564659
Monocotyledons	Orchidaceae	*Gomesa*	*Gomesa warmingii*	FJ564089
Monocotyledons	Orchidaceae	*Gomesa*	*Gomesa warmingii*	FJ564391
Monocotyledons	Orchidaceae	*Gomesa*	*Gomesa warmingii*	FJ564409
Monocotyledons	Orchidaceae	*Cischweinfia*	*Cischweinfia popowiana*	FJ564312
Monocotyledons	Orchidaceae	*Cischweinfia*	*Cischweinfia popowiana*	FJ564679
Monocotyledons	Orchidaceae	*Cischweinfia*	*Cischweinfia pusilla*	FJ564122
Monocotyledons	Orchidaceae	*Cischweinfia*	*Cischweinfia pusilla*	FJ564651
Monocotyledons	Orchidaceae	*Cuitlauzina*	*Cuitlauzina pulchella*	FJ564003
Monocotyledons	Orchidaceae	*Cuitlauzina*	*Cuitlauzina pulchella*	FJ564174
Monocotyledons	Orchidaceae	*Cyrtochilum*	*Cyrtochilum hoeijeri*	FJ564313
Monocotyledons	Orchidaceae	*Cyrtochilum*	*Cyrtochilum hoeijeri*	FJ564443
Monocotyledons	Orchidaceae	*Cyrtochilum*	*Cyrtochilum trifurcatum*	FJ564110
Monocotyledons	Orchidaceae	*Cyrtochilum*	*Cyrtochilum trifurcatum*	FJ564504
Monocotyledons	Orchidaceae	*Ornithocephalus*	*Ornithocephalus dalstroemii*	FJ563990
Monocotyledons	Orchidaceae	*Ornithocephalus*	*Ornithocephalus dalstroemii*	FJ564601
Monocotyledons	Orchidaceae	*Ornithocephalus*	*Ornithocephalus dalstroemii*	FJ564613
Monocotyledons	Orchidaceae	*Tolumnia*	*Tolumnia guianensis*	FJ564218
Monocotyledons	Orchidaceae	*Tolumnia*	*Tolumnia guianensis*	FJ564233
Monocotyledons	Orchidaceae	*Tolumnia*	*Tolumnia prionochila*	FJ564219
Monocotyledons	Orchidaceae	*Tolumnia*	*Tolumnia prionochila*	FJ564252
Monocotyledons	Orchidaceae	*Comparettia*	*Comparettia corydaloides*	FJ564280
Monocotyledons	Orchidaceae	*Comparettia*	*Comparettia corydaloides*	FJ564360
Monocotyledons	Orchidaceae	*Comparettia*	*Comparettia tungurahuae*	FJ564523
Monocotyledons	Orchidaceae	*Comparettia*	*Comparettia tungurahuae*	FJ564611
Monocotyledons	Orchidaceae	*Gomesa*	*Gomesa forbesii*	FJ564408
Monocotyledons	Orchidaceae	*Gomesa*	*Gomesa forbesii*	FJ564681
Monocotyledons	Orchidaceae	*Gomesa*	*Gomesa gardneri*	FJ564684
Monocotyledons	Orchidaceae	*Gomesa*	*Gomesa gardneri*	EU935666
Monocotyledons	Orchidaceae	*Gomesa*	*Gomesa longipes*	FJ564358
Monocotyledons	Orchidaceae	*Gomesa*	*Gomesa longipes*	FJ564394

Monocotyledons	Orchidaceae	*Gomesa*	*Gomesa varicosa*	FJ564411
Monocotyledons	Orchidaceae	*Gomesa*	*Gomesa varicosa*	FJ564682
Monocotyledons	Orchidaceae	*Trichocentrum*	*Trichocentrum cosymbephorum*	FJ564301
Monocotyledons	Orchidaceae	*Trichocentrum*	*Trichocentrum cosymbephorum*	FJ564473
Monocotyledons	Bromeliaceae	*Tillandsia*	*Tillandsia bulbosa*	JN204663
Monocotyledons	Bromeliaceae	*Tillandsia*	*Tillandsia bulbosa*	JN204664
Monocotyledons	Orchidaceae	*Cymbidium*	*Cymbidium haematodes*	HM008996
Monocotyledons	Orchidaceae	*Cymbidium*	*Cymbidium haematodes*	JN412741
Monocotyledons	Cyperaceae	*Carex*	*Carex acidicola*	FJ597262
Monocotyledons	Cyperaceae	*Carex*	*Carex acidicola*	FJ597263
Monocotyledons	Cyperaceae	*Carex*	*Carex acidicola*	FJ597311
Monocotyledons	Cyperaceae	*Carex*	*Carex bulbostylis*	FJ597268
Monocotyledons	Cyperaceae	*Carex*	*Carex bulbostylis*	FJ597269
Monocotyledons	Cyperaceae	*Carex*	*Carex calcifugens*	FJ597270
Monocotyledons	Cyperaceae	*Carex*	*Carex calcifugens*	FJ597271
Monocotyledons	Cyperaceae	*Carex*	*Carex conoidea*	FJ597272
Monocotyledons	Cyperaceae	*Carex*	*Carex conoidea*	FJ597273
Monocotyledons	Cyperaceae	*Carex*	*Carex corrugata*	FJ597274
Monocotyledons	Cyperaceae	*Carex*	*Carex corrugata*	FJ597275
Monocotyledons	Cyperaceae	*Carex*	*Carex corrugata*	FJ597276
Monocotyledons	Cyperaceae	*Carex*	*Carex corrugata*	FJ597277
Monocotyledons	Cyperaceae	*Carex*	*Carex flaccosperma*	FJ597278
Monocotyledons	Cyperaceae	*Carex*	*Carex flaccosperma*	FJ597279
Monocotyledons	Cyperaceae	*Carex*	*Carex flaccosperma*	FJ597280
Monocotyledons	Cyperaceae	*Carex*	*Carex glaucodea*	FJ597281
Monocotyledons	Cyperaceae	*Carex*	*Carex glaucodea*	FJ597282
Monocotyledons	Cyperaceae	*Carex*	*Carex glaucodea*	FJ597283
Monocotyledons	Cyperaceae	*Carex*	*Carex glaucodea*	FJ597284
Monocotyledons	Cyperaceae	*Carex*	*Carex glaucodea*	FJ597285
Monocotyledons	Cyperaceae	*Carex*	*Carex godfreyi*	FJ597286
Monocotyledons	Cyperaceae	*Carex*	*Carex godfreyi*	FJ597287
Monocotyledons	Cyperaceae	*Carex*	*Carex grisea*	FJ597288
Monocotyledons	Cyperaceae	*Carex*	*Carex grisea*	FJ597289
Monocotyledons	Cyperaceae	*Carex*	*Carex grisea*	FJ597290
Monocotyledons	Cyperaceae	*Carex*	*Carex ouachitana*	FJ597297
Monocotyledons	Cyperaceae	*Carex*	*Carex ouachitana*	FJ597298
Monocotyledons	Cyperaceae	*Carex*	*Carex ouachitana*	FJ597299
Monocotyledons	Cyperaceae	*Carex*	*Carex paeninsulae*	FJ597300
Monocotyledons	Cyperaceae	*Carex*	*Carex paeninsulae*	FJ597301
Monocotyledons	Cyperaceae	*Carex*	*Carex paeninsulae*	FJ597302
Monocotyledons	Cyperaceae	*Carex*	*Carex pigra*	FJ597303
Monocotyledons	Cyperaceae	*Carex*	*Carex pigra*	FJ597304
Monocotyledons	Cyperaceae	*Carex*	*Carex planispicata*	FJ597305
Monocotyledons	Cyperaceae	*Carex*	*Carex planispicata*	FJ597306
Monocotyledons	Cyperaceae	*Carex*	*Carex planispicata*	FJ597307
Monocotyledons	Cyperaceae	*Carex*	*Carex thornei*	FJ597308
Monocotyledons	Cyperaceae	*Carex*	*Carex thornei*	FJ597309
Monocotyledons	Cyperaceae	*Carex*	*Carex thornei*	FJ597310
Monocotyledons	Bromeliaceae	*Billbergia*	*Billbergia euphemiae*	JN204630
Monocotyledons	Bromeliaceae	*Billbergia*	*Billbergia euphemiae*	JN204631
Monocotyledons	Bromeliaceae	*Billbergia*	*Billbergia euphemiae*	JN204632
Monocotyledons	Bromeliaceae	*Aechmea*	*Aechmea blumenavii*	JN204599
Monocotyledons	Bromeliaceae	*Aechmea*	*Aechmea blumenavii*	JN204600

Monocotyledons	Bromeliaceae	*Aechmea*	*Aechmea bromeliifolia*	JN204601
Monocotyledons	Bromeliaceae	*Aechmea*	*Aechmea bromeliifolia*	JN204602
Monocotyledons	Bromeliaceae	*Aechmea*	*Aechmea bromeliifolia*	JN204603
Monocotyledons	Bromeliaceae	*Aechmea*	*Aechmea bromeliifolia*	HQ913649
Monocotyledons	Bromeliaceae	*Aechmea*	*Aechmea coelestis*	JN204608
Monocotyledons	Bromeliaceae	*Aechmea*	*Aechmea coelestis*	JN204609
Monocotyledons	Bromeliaceae	*Aechmea*	*Aechmea ramosa*	JN204623
Monocotyledons	Bromeliaceae	*Aechmea*	*Aechmea ramosa*	JN204624
Monocotyledons	Bromeliaceae	*Aechmea*	*Aechmea recurvata*	JN204625
Monocotyledons	Bromeliaceae	*Aechmea*	*Aechmea recurvata*	JN204626
Monocotyledons	Bromeliaceae	*Aechmea*	*Aechmea recurvata*	JN204627
Monocotyledons	Arecaceae	*Arenga*	*Arenga engleri*	JF345020
Monocotyledons	Arecaceae	*Arenga*	*Arenga engleri*	JF345021
Monocotyledons	Arecaceae	*Arenga*	*Arenga engleri*	JF345022
Monocotyledons	Arecaceae	*Arenga*	*Arenga engleri*	JF345023
Monocotyledons	Orchidaceae	*Odontoglossum*	*Odontoglossum hybrid cultivar*	GU136252
Monocotyledons	Orchidaceae	*Odontoglossum*	*Odontoglossum hybrid cultivar*	GU136260
Monocotyledons	Arecaceae	*Arenga*	*Arenga westerhoutii*	JF345041
Monocotyledons	Arecaceae	*Arenga*	*Arenga westerhoutii*	JF345042
Monocotyledons	Asparagaceae	*Polygonatum*	*Polygonatum griffithii*	JN046413
Monocotyledons	Asparagaceae	*Polygonatum*	*Polygonatum griffithii*	JN046414
Monocotyledons	Asparagaceae	*Tupistra*	*Tupistra grandistigma*	JN047462
Monocotyledons	Asparagaceae	*Tupistra*	*Tupistra grandistigma*	JN047463
Monocotyledons	Asparagaceae	*Tupistra*	*Tupistra grandistigma*	JN047464
Monocotyledons	Asparagaceae	*Tupistra*	*Tupistra grandistigma*	JN047465
Monocotyledons	Asparagaceae	*Tupistra*	*Tupistra grandistigma*	JN047466
Monocotyledons	Asparagaceae	*Tupistra*	*Tupistra longispica*	JN047467
Monocotyledons	Asparagaceae	*Tupistra*	*Tupistra longispica*	JN047468
Monocotyledons	Asparagaceae	*Tupistra*	*Tupistra longispica*	JN047469
Monocotyledons	Asparagaceae	*Tupistra*	*Tupistra longispica*	JN047470
Monocotyledons	Asparagaceae	*Tupistra*	*Tupistra longispica*	JN047471
Monocotyledons	Asparagaceae	*Tupistra*	*Tupistra pingbianensis*	JN047472
Monocotyledons	Asparagaceae	*Tupistra*	*Tupistra pingbianensis*	JN047473
Monocotyledons	Asparagaceae	*Tupistra*	*Tupistra pingbianensis*	JN047474
Monocotyledons	Asparagaceae	*Tupistra*	*Tupistra pingbianensis*	JN047475
Monocotyledons	Arecaceae	*Arenga*	*Arenga hastata*	JF345005
Monocotyledons	Arecaceae	*Arenga*	*Arenga hastata*	JF345019
Monocotyledons	Arecaceae	*Arenga*	*Arenga hastata*	JF345024
Monocotyledons	Arecaceae	*Arenga*	*Arenga obtusifolia*	JF345032
Monocotyledons	Arecaceae	*Arenga*	*Arenga obtusifolia*	JF345033
Monocotyledons	Arecaceae	*Arenga*	*Arenga ryukyuensis*	JF345036
Monocotyledons	Arecaceae	*Arenga*	*Arenga ryukyuensis*	JF345037
Monocotyledons	Arecaceae	*Wallichia*	*Wallichia gracilis*	JF345072
Monocotyledons	Arecaceae	*Wallichia*	*Wallichia gracilis*	JF345075
Monocotyledons	Arecaceae	*Wallichia*	*Wallichia gracilis*	JF345076
Monocotyledons	Bromeliaceae	*Aechmea*	*Aechmea bicolor*	JN204597
Monocotyledons	Bromeliaceae	*Aechmea*	*Aechmea bicolor*	JN204598
Monocotyledons	Bromeliaceae	*Aechmea*	*Aechmea caudata*	JN204605
Monocotyledons	Bromeliaceae	*Aechmea*	*Aechmea caudata*	JN204606
Monocotyledons	Bromeliaceae	*Aechmea*	*Aechmea caudata*	JN204607
Monocotyledons	Bromeliaceae	*Aechmea*	*Aechmea curranii*	JN204610
Monocotyledons	Bromeliaceae	*Aechmea*	*Aechmea curranii*	JN204611
Monocotyledons	Bromeliaceae	*Aechmea*	*Aechmea curranii*	JN204612

Monocotyledons	Bromeliaceae	*Aechmea*	*Aechmea phanerophlebia*	JN204620
Monocotyledons	Bromeliaceae	*Aechmea*	*Aechmea phanerophlebia*	JN204621
Monocotyledons	Bromeliaceae	*Aechmea*	*Aechmea phanerophlebia*	JN204622
Monocotyledons	Bromeliaceae	*Billbergia*	*Billbergia distachya*	JN204628
Monocotyledons	Bromeliaceae	*Billbergia*	*Billbergia distachya*	JN204629
Monocotyledons	Bromeliaceae	*Billbergia*	*Billbergia chlorostica*	JN204633
Monocotyledons	Bromeliaceae	*Billbergia*	*Billbergia chlorostica*	JN204634
Monocotyledons	Bromeliaceae	*Billbergia*	*Billbergia chlorostica*	JN204635
Monocotyledons	Bromeliaceae	*Tillandsia*	*Tillandsia polystachia*	JN204665
Monocotyledons	Bromeliaceae	*Tillandsia*	*Tillandsia polystachia*	JN204666
Monocotyledons	Colchicaceae	*Colchicum*	*Colchicum bivonae*	JF934074
Monocotyledons	Iridaceae	*Iris*	*Iris germanica*	JN044979
Monocotyledons	Iridaceae	*Iris*	*Iris germanica*	JN044980
Monocotyledons	Iridaceae	*Iris*	*Iris chrysographes*	JN044960
Monocotyledons	Iridaceae	*Iris*	*Iris chrysographes*	JN044961
Monocotyledons	Iridaceae	*Iris*	*Iris chrysographes*	JN044962
Monocotyledons	Iridaceae	*Iris*	*Iris ensata*	JN044975
Monocotyledons	Iridaceae	*Iris*	*Iris ensata*	JN044976
Monocotyledons	Iridaceae	*Iris*	*Iris domestica*	GQ434907
Monocotyledons	Iridaceae	*Iris*	*Iris domestica*	GQ434908
Monocotyledons	Iridaceae	*Iris*	*Iris pseudacorus*	JN045000
Monocotyledons	Iridaceae	*Iris*	*Iris pseudacorus*	JN045001
Monocotyledons	Iridaceae	*Iris*	*Iris pseudacorus*	JN045002
Monocotyledons	Iridaceae	*Iris*	*Iris tectorum*	GQ435429
Monocotyledons	Iridaceae	*Iris*	*Iris tectorum*	GQ435430
Monocotyledons	Iridaceae	*Iris*	*Iris tectorum*	JN045015
Monocotyledons	Iridaceae	*Iris*	*Iris tectorum*	JN045016
Monocotyledons	Iridaceae	*Iris*	*Iris tectorum*	JN045017
Monocotyledons	Iridaceae	*Iris*	*Iris tectorum*	JN045018
Monocotyledons	Iridaceae	*Iris*	*Iris tectorum*	JN045019
Monocotyledons	Iridaceae	*Iris*	*Iris tectorum*	JN045020
Monocotyledons	Iridaceae	*Iris*	*Iris forrestii*	JN044977
Monocotyledons	Iridaceae	*Iris*	*Iris forrestii*	JN044978
Monocotyledons	Iridaceae	*Iris*	*Iris dichotoma*	JN044973
Monocotyledons	Iridaceae	*Iris*	*Iris dichotoma*	JN044974
Monocotyledons	Iridaceae	*Iris*	*Iris humilis*	FM253735
Monocotyledons	Iridaceae	*Iris*	*Iris humilis*	FM253736
Monocotyledons	Iridaceae	*Iris*	*Iris humilis*	FM253737
Monocotyledons	Iridaceae	*Iris*	*Iris humilis*	FM253738
Monocotyledons	Iridaceae	*Iris*	*Iris humilis*	FM253739
Monocotyledons	Iridaceae	*Iris*	*Iris humilis*	FM253740
Monocotyledons	Iridaceae	*Iris*	*Iris humilis*	FM253741
Monocotyledons	Iridaceae	*Iris*	*Iris humilis*	FM253742
Monocotyledons	Iridaceae	*Iris*	*Iris humilis*	FM253743
Monocotyledons	Iridaceae	*Iris*	*Iris humilis*	FM253744
Monocotyledons	Iridaceae	*Iris*	*Iris humilis*	FM253745
Monocotyledons	Iridaceae	*Iris*	*Iris humilis*	FM253746
Monocotyledons	Iridaceae	*Iris*	*Iris humilis*	FM253747
Monocotyledons	Iridaceae	*Iris*	*Iris lactea*	JN044989
Monocotyledons	Iridaceae	*Iris*	*Iris lactea*	JN044990
Monocotyledons	Iridaceae	*Iris*	*Iris lactea*	JN044991
Monocotyledons	Iridaceae	*Iris*	*Iris lactea*	JN044992
Monocotyledons	Iridaceae	*Iris*	*Iris loczyi*	JN044994

Monocotyledons	Iridaceae	*Iris*	*Iris loczyi*	JN044995
Monocotyledons	Iridaceae	*Iris*	*Iris loczyi*	JN044996
Monocotyledons	Iridaceae	*Iris*	*Iris ruthenica*	JN045005
Monocotyledons	Iridaceae	*Iris*	*Iris ruthenica*	JN045006
Monocotyledons	Iridaceae	*Iris*	*Iris ruthenica*	JN045007
Monocotyledons	Iridaceae	*Iris*	*Iris ruthenica*	JN045008
Monocotyledons	Iridaceae	*Iris*	*Iris ruthenica*	JN045009
Monocotyledons	Iridaceae	*Iris*	*Iris sanguinea*	JN045010
Monocotyledons	Iridaceae	*Iris*	*Iris sanguinea*	JN045011
Monocotyledons	Iridaceae	*Iris*	*Iris sanguinea*	JN045012
Monocotyledons	Iridaceae	*Iris*	*Iris tenuifolia*	JN045021
Monocotyledons	Iridaceae	*Iris*	*Iris tenuifolia*	JN045022
Monocotyledons	Iridaceae	*Iris*	*Iris tigridia*	JN045023
Monocotyledons	Iridaceae	*Iris*	*Iris tigridia*	JN045024
Monocotyledons	Iridaceae	*Iris*	*Iris uniflora*	JN045025
Monocotyledons	Iridaceae	*Iris*	*Iris uniflora*	JN045026
Monocotyledons	Iridaceae	*Iris*	*Iris ventricosa*	JN045028
Monocotyledons	Iridaceae	*Iris*	*Iris ventricosa*	JN045029
Monocotyledons	Zingiberaceae	*Amomum*	*Amomum glabrum*	JN043930
Monocotyledons	Zingiberaceae	*Amomum*	*Amomum glabrum*	JN043931
Monocotyledons	Zingiberaceae	*Amomum*	*Amomum longipetiolatum*	JN043938
Monocotyledons	Zingiberaceae	*Amomum*	*Amomum longipetiolatum*	JN043939
Monocotyledons	Zingiberaceae	*Amomum*	*Amomum villosum*	GQ118656
Monocotyledons	Zingiberaceae	*Amomum*	*Amomum villosum*	GU180423
Monocotyledons	Iridaceae	*Iris*	*Iris delavayi*	JN044971
Monocotyledons	Iridaceae	*Iris*	*Iris delavayi*	JN044972
Monocotyledons	Iridaceae	*Iris*	*Iris missouriensis*	EF434718
Monocotyledons	Iridaceae	*Iris*	*Iris missouriensis*	EF434719
Monocotyledons	Iridaceae	*Iris*	*Iris missouriensis*	EF434720
Monocotyledons	Iridaceae	*Iris*	*Iris missouriensis*	EF434721
Monocotyledons	Iridaceae	*Iris*	*Iris missouriensis*	EF434723
Monocotyledons	Iridaceae	*Iris*	*Iris missouriensis*	EF434724
Monocotyledons	Xanthorrhoeaceae	*Haworthia*	*Haworthia aristata*	HQ646850
Monocotyledons	Xanthorrhoeaceae	*Haworthia*	*Haworthia aristata*	HQ646851
Monocotyledons	Xanthorrhoeaceae	*Haworthia*	*Haworthia attenuata*	HQ646880
Monocotyledons	Xanthorrhoeaceae	*Haworthia*	*Haworthia attenuata*	HQ646881
Monocotyledons	Xanthorrhoeaceae	*Haworthia*	*Haworthia cymbiformis*	HQ646865
Monocotyledons	Xanthorrhoeaceae	*Haworthia*	*Haworthia cymbiformis*	HQ646866
Monocotyledons	Xanthorrhoeaceae	*Haworthia*	*Haworthia cooperi*	HQ646854
Monocotyledons	Xanthorrhoeaceae	*Haworthia*	*Haworthia cooperi*	HQ646855
Monocotyledons	Xanthorrhoeaceae	*Haworthia*	*Haworthia cooperi*	HQ646856
Monocotyledons	Xanthorrhoeaceae	*Haworthia*	*Haworthia cooperi*	HQ646857
Monocotyledons	Xanthorrhoeaceae	*Haworthia*	*Haworthia cooperi*	HQ646858
Monocotyledons	Xanthorrhoeaceae	*Haworthia*	*Haworthia cooperi*	HQ646859
Monocotyledons	Xanthorrhoeaceae	*Haworthia*	*Haworthia cooperi*	HQ646860
Monocotyledons	Xanthorrhoeaceae	*Haworthia*	*Haworthia cooperi*	HQ646861
Monocotyledons	Xanthorrhoeaceae	*Haworthia*	*Haworthia cooperi*	HQ646862
Monocotyledons	Xanthorrhoeaceae	*Haworthia*	*Haworthia cooperi*	HQ646863
Monocotyledons	Xanthorrhoeaceae	*Haworthia*	*Haworthia cooperi*	HQ646864
Monocotyledons	Xanthorrhoeaceae	*Haworthia*	*Haworthia glauca*	HQ646884
Monocotyledons	Xanthorrhoeaceae	*Haworthia*	*Haworthia glauca*	HQ646885
Monocotyledons	Xanthorrhoeaceae	*Haworthia*	*Haworthia glauca*	HQ646886
Monocotyledons	Zingiberaceae	*Amomum*	*Amomum compactum*	GQ118660

Monocotyledons	Zingiberaceae	*Amomum*	*Amomum compactum*	JN043924
Monocotyledons	Zingiberaceae	*Amomum*	*Amomum compactum*	JN043925
Monocotyledons	Zingiberaceae	*Amomum*	*Amomum compactum*	JN043926
Monocotyledons	Zingiberaceae	*Amomum*	*Amomum koenigii*	JN043932
Monocotyledons	Zingiberaceae	*Amomum*	*Amomum koenigii*	JN043933
Monocotyledons	Zingiberaceae	*Amomum*	*Amomum koenigii*	JN043934
Monocotyledons	Zingiberaceae	*Amomum*	*Amomum koenigii*	JN043935
Monocotyledons	Zingiberaceae	*Amomum*	*Amomum maximum*	JN043940
Monocotyledons	Zingiberaceae	*Amomum*	*Amomum maximum*	JN043941
Monocotyledons	Zingiberaceae	*Amomum*	*Amomum maximum*	JN043942
Monocotyledons	Zingiberaceae	*Amomum*	*Amomum menglaense*	JN043943
Monocotyledons	Zingiberaceae	*Amomum*	*Amomum menglaense*	JN043944
Monocotyledons	Zingiberaceae	*Amomum*	*Amomum menglaense*	JN043945
Monocotyledons	Zingiberaceae	*Amomum*	*Amomum menglaense*	JN043946
Monocotyledons	Zingiberaceae	*Amomum*	*Amomum purpureorubrum*	JN043959
Monocotyledons	Zingiberaceae	*Amomum*	*Amomum purpureorubrum*	JN043960
Monocotyledons	Zingiberaceae	*Amomum*	*Amomum quadratolaminare*	JN043961
Monocotyledons	Zingiberaceae	*Amomum*	*Amomum quadratolaminare*	JN043962
Monocotyledons	Zingiberaceae	*Amomum*	*Amomum quadratolaminare*	JN043963
Monocotyledons	Zingiberaceae	*Amomum*	*Amomum quadratolaminare*	JN043964
Monocotyledons	Zingiberaceae	*Amomum*	*Amomum quadratolaminare*	JN043965
Monocotyledons	Zingiberaceae	*Amomum*	*Amomum sericeum*	JN043972
Monocotyledons	Zingiberaceae	*Amomum*	*Amomum sericeum*	JN043973
Monocotyledons	Zingiberaceae	*Amomum*	*Amomum subcapitatum*	JN043974
Monocotyledons	Zingiberaceae	*Amomum*	*Amomum subcapitatum*	JN043975
Monocotyledons	Orchidaceae	*Rhynchostylis*	*Rhynchostylis retusa*	GQ251332
Monocotyledons	Orchidaceae	*Rhynchostylis*	*Rhynchostylis retusa*	GQ251333
Monocotyledons	Orchidaceae	*Rhynchostylis*	*Rhynchostylis retusa*	GQ251334
Monocotyledons	Orchidaceae	*Rhynchostylis*	*Rhynchostylis gigantea*	GQ251323
Monocotyledons	Orchidaceae	*Rhynchostylis*	*Rhynchostylis gigantea*	GQ251324
Monocotyledons	Orchidaceae	*Rhynchostylis*	*Rhynchostylis gigantea*	GQ251325
Monocotyledons	Orchidaceae	*Rhynchostylis*	*Rhynchostylis gigantea*	GQ251326
Monocotyledons	Orchidaceae	*Rhynchostylis*	*Rhynchostylis gigantea*	GQ251327
Monocotyledons	Orchidaceae	*Rhynchostylis*	*Rhynchostylis gigantea*	GQ251328
Monocotyledons	Orchidaceae	*Rhynchostylis*	*Rhynchostylis gigantea*	GQ251329
Monocotyledons	Orchidaceae	*Rhynchostylis*	*Rhynchostylis gigantea*	GQ251330
Monocotyledons	Orchidaceae	*Rhynchostylis*	*Rhynchostylis gigantea*	GQ251331
Monocotyledons	Iridaceae	*Iris*	*Iris collettii*	JN044963
Monocotyledons	Iridaceae	*Iris*	*Iris collettii*	JN044964
Monocotyledons	Iridaceae	*Iris*	*Iris collettii*	JN044965
Monocotyledons	Zingiberaceae	*Amomum*	*Amomum petaloideum*	JN043955
Monocotyledons	Zingiberaceae	*Amomum*	*Amomum petaloideum*	JN043956
Monocotyledons	Zingiberaceae	*Amomum*	*Amomum petaloideum*	JN043957
Monocotyledons	Zingiberaceae	*Amomum*	*Amomum petaloideum*	JN043958
Monocotyledons	Iridaceae	*Iris*	*Iris mandshurica*	FM253719
Monocotyledons	Iridaceae	*Iris*	*Iris mandshurica*	FM253720
Monocotyledons	Iridaceae	*Iris*	*Iris mandshurica*	FM253721
Monocotyledons	Iridaceae	*Iris*	*Iris mandshurica*	FM253722
Monocotyledons	Iridaceae	*Iris*	*Iris mandshurica*	FM253723
Monocotyledons	Iridaceae	*Iris*	*Iris mandshurica*	FM253724
Monocotyledons	Iridaceae	*Iris*	*Iris mandshurica*	FM253725
Monocotyledons	Iridaceae	*Iris*	*Iris mandshurica*	FM253726
Monocotyledons	Iridaceae	*Iris*	*Iris mandshurica*	FM253727

Monocotyledons	Iridaceae	*Iris*	*Iris mandshurica*	FM253728
Monocotyledons	Iridaceae	*Iris*	*Iris mandshurica*	FM253729
Monocotyledons	Iridaceae	*Iris*	*Iris mandshurica*	FM253730
Monocotyledons	Iridaceae	*Iris*	*Iris mandshurica*	FM253731
Monocotyledons	Iridaceae	*Iris*	*Iris mandshurica*	FM253732
Monocotyledons	Iridaceae	*Iris*	*Iris mandshurica*	FM253733
Monocotyledons	Iridaceae	*Iris*	*Iris mandshurica*	FM253734
Monocotyledons	Orchidaceae	*Aulosepalum*	*Aulosepalum pyramidale*	AM884884
Monocotyledons	Orchidaceae	*Aulosepalum*	*Aulosepalum pyramidale*	AM884885
Monocotyledons	Orchidaceae	*Aulosepalum*	*Aulosepalum ramentaceum*	AM884882
Monocotyledons	Orchidaceae	*Aulosepalum*	*Aulosepalum ramentaceum*	AM884883
Monocotyledons	Orchidaceae	*Aulosepalum*	*Aulosepalum oestlundii*	AM884886
Monocotyledons	Orchidaceae	*Aulosepalum*	*Aulosepalum oestlundii*	AM884887
Monocotyledons	Orchidaceae	*Aulosepalum*	*Aulosepalum hemichreum*	AM884889
Monocotyledons	Orchidaceae	*Aulosepalum*	*Aulosepalum hemichreum*	AM884890
Monocotyledons	Orchidaceae	*Holcoglossum*	*Holcoglossum flavescens*	HQ404442
Monocotyledons	Orchidaceae	*Holcoglossum*	*Holcoglossum flavescens*	HQ404443
Monocotyledons	Orchidaceae	*Holcoglossum*	*Holcoglossum flavescens*	HQ404444
Monocotyledons	Orchidaceae	*Holcoglossum*	*Holcoglossum flavescens*	HQ404445
Monocotyledons	Orchidaceae	*Holcoglossum*	*Holcoglossum flavescens*	HQ404446
Monocotyledons	Orchidaceae	*Holcoglossum*	*Holcoglossum lingulatum*	HQ404453
Monocotyledons	Orchidaceae	*Holcoglossum*	*Holcoglossum lingulatum*	HQ404454
Monocotyledons	Orchidaceae	*Holcoglossum*	*Holcoglossum lingulatum*	HQ404455
Monocotyledons	Orchidaceae	*Holcoglossum*	*Holcoglossum nujiangense*	HQ404456
Monocotyledons	Orchidaceae	*Holcoglossum*	*Holcoglossum nujiangense*	HQ404457
Monocotyledons	Orchidaceae	*Holcoglossum*	*Holcoglossum nujiangense*	HQ404458
Monocotyledons	Orchidaceae	*Holcoglossum*	*Holcoglossum nujiangense*	HQ404459
Monocotyledons	Orchidaceae	*Holcoglossum*	*Holcoglossum nujiangense*	HQ404460
Monocotyledons	Orchidaceae	*Holcoglossum*	*Holcoglossum rupestre*	HQ404467
Monocotyledons	Orchidaceae	*Holcoglossum*	*Holcoglossum rupestre*	HQ404468
Monocotyledons	Orchidaceae	*Holcoglossum*	*Holcoglossum rupestre*	HQ404469
Monocotyledons	Orchidaceae	*Holcoglossum*	*Holcoglossum rupestre*	HQ404470
Monocotyledons	Orchidaceae	*Holcoglossum*	*Holcoglossum rupestre*	HQ404471
Monocotyledons	Orchidaceae	*Holcoglossum*	*Holcoglossum sinicum*	HQ404472
Monocotyledons	Orchidaceae	*Holcoglossum*	*Holcoglossum sinicum*	HQ404473
Monocotyledons	Orchidaceae	*Holcoglossum*	*Holcoglossum sinicum*	HQ404474
Monocotyledons	Orchidaceae	*Holcoglossum*	*Holcoglossum sinicum*	HQ404475
Monocotyledons	Orchidaceae	*Holcoglossum*	*Holcoglossum sinicum*	HQ404476
Monocotyledons	Orchidaceae	*Holcoglossum*	*Holcoglossum weixiense*	HQ404483
Monocotyledons	Orchidaceae	*Holcoglossum*	*Holcoglossum weixiense*	HQ404484
Monocotyledons	Orchidaceae	*Holcoglossum*	*Holcoglossum weixiense*	HQ404485
Monocotyledons	Orchidaceae	*Holcoglossum*	*Holcoglossum weixiense*	HQ404486
Monocotyledons	Orchidaceae	*Holcoglossum*	*Holcoglossum weixiense*	HQ404487
Monocotyledons	Iridaceae	*Iris*	*Iris vorobievii*	FM253702
Monocotyledons	Iridaceae	*Iris*	*Iris vorobievii*	FM253703
Monocotyledons	Iridaceae	*Iris*	*Iris vorobievii*	FM253704
Monocotyledons	Iridaceae	*Iris*	*Iris vorobievii*	FM253705
Monocotyledons	Iridaceae	*Iris*	*Iris vorobievii*	FM253706
Monocotyledons	Iridaceae	*Iris*	*Iris vorobievii*	FM253707
Monocotyledons	Iridaceae	*Iris*	*Iris vorobievii*	FM253708
Monocotyledons	Iridaceae	*Iris*	*Iris vorobievii*	FM253709
Monocotyledons	Iridaceae	*Iris*	*Iris vorobievii*	FM253710
Monocotyledons	Iridaceae	*Iris*	*Iris vorobievii*	FM253711

Monocotyledons	Iridaceae	*Iris*	*Iris vorobievii*	FM253712
Monocotyledons	Iridaceae	*Iris*	*Iris vorobievii*	FM253713
Monocotyledons	Iridaceae	*Iris*	*Iris vorobievii*	FM253714
Monocotyledons	Iridaceae	*Iris*	*Iris vorobievii*	FM253715
Monocotyledons	Iridaceae	*Iris*	*Iris vorobievii*	FM253716
Monocotyledons	Iridaceae	*Iris*	*Iris vorobievii*	FM253717
Monocotyledons	Iridaceae	*Iris*	*Iris vorobievii*	FM253718
Monocotyledons	Eriocaulaceae	*Paepalanthus*	*Paepalanthus lamarckii*	EU924377
Monocotyledons	Eriocaulaceae	*Paepalanthus*	*Paepalanthus lamarckii*	HQ843070
Monocotyledons	Iridaceae	*Iris*	*Iris bulleyana*	JN044956
Monocotyledons	Iridaceae	*Iris*	*Iris bulleyana*	JN044957
Monocotyledons	Iridaceae	*Iris*	*Iris bulleyana*	JN044958
Monocotyledons	Iridaceae	*Iris*	*Iris decora*	JN044969
Monocotyledons	Iridaceae	*Iris*	*Iris decora*	JN044970
Monocotyledons	Zingiberaceae	*Amomum*	*Amomum longiligulare*	GQ118658
Monocotyledons	Zingiberaceae	*Amomum*	*Amomum longiligulare*	GQ464983
Monocotyledons	Zingiberaceae	*Amomum*	*Amomum longiligulare*	GU180431
Monocotyledons	Zingiberaceae	*Amomum*	*Amomum krervanh*	GQ118657
Monocotyledons	Zingiberaceae	*Amomum*	*Amomum krervanh*	JN043936
Monocotyledons	Zingiberaceae	*Amomum*	*Amomum krervanh*	JN043937
Monocotyledons	Orchidaceae	*Rhynchostylis*	*Rhynchostylis coelestis*	GQ251335
Monocotyledons	Orchidaceae	*Rhynchostylis*	*Rhynchostylis coelestis*	GQ251336
Monocotyledons	Orchidaceae	*Rhynchostylis*	*Rhynchostylis coelestis*	GQ251337
Monocotyledons	Iridaceae	*Iris*	*Iris rossii*	JN045003
Monocotyledons	Iridaceae	*Iris*	*Iris rossii*	JN045004
Monocotyledons	Eriocaulaceae	*Paepalanthus*	*Paepalanthus sp. MJGA-2011*	HQ843079
Monocotyledons	Eriocaulaceae	*Paepalanthus*	*Paepalanthus sp. MJGA-2011*	HQ843080
Monocotyledons	Eriocaulaceae	*Paepalanthus*	*Paepalanthus sp. MJGA-2011*	HQ843081
Monocotyledons	Eriocaulaceae	*Paepalanthus*	*Paepalanthus sp. MJGA-2011*	HQ843082
Monocotyledons	Orchidaceae	*Holcoglossum*	*Holcoglossum quasipinifolium*	HQ404462
Monocotyledons	Orchidaceae	*Holcoglossum*	*Holcoglossum quasipinifolium*	HQ404463
Monocotyledons	Orchidaceae	*Holcoglossum*	*Holcoglossum quasipinifolium*	HQ404464
Monocotyledons	Orchidaceae	*Holcoglossum*	*Holcoglossum quasipinifolium*	HQ404465
Monocotyledons	Orchidaceae	*Holcoglossum*	*Holcoglossum quasipinifolium*	HQ404466
Monocotyledons	Iridaceae	*Iris*	*Iris anguifuga*	JN044954
Monocotyledons	Iridaceae	*Iris*	*Iris anguifuga*	JN044955
Monocotyledons	Iridaceae	*Iris*	*Iris confusa*	JN044966
Monocotyledons	Iridaceae	*Iris*	*Iris confusa*	JN044967
Monocotyledons	Iridaceae	*Iris*	*Iris confusa*	JN044968
Monocotyledons	Iridaceae	*Iris*	*Iris goniocarpa*	JN044981
Monocotyledons	Iridaceae	*Iris*	*Iris goniocarpa*	JN044982
Monocotyledons	Iridaceae	*Iris*	*Iris speculatrix*	JN045013
Monocotyledons	Iridaceae	*Iris*	*Iris speculatrix*	JN045014
Monocotyledons	Zingiberaceae	*Amomum*	*Amomum dealbatum*	JN043927
Monocotyledons	Zingiberaceae	*Amomum*	*Amomum dealbatum*	JN043928
Monocotyledons	Zingiberaceae	*Amomum*	*Amomum dealbatum*	JN043929
Monocotyledons	Zingiberaceae	*Amomum*	*Amomum microcarpum*	JN043947
Monocotyledons	Zingiberaceae	*Amomum*	*Amomum microcarpum*	JN043948
Monocotyledons	Zingiberaceae	*Amomum*	*Amomum muricarpum*	JN043949
Monocotyledons	Zingiberaceae	*Amomum*	*Amomum muricarpum*	JN043950
Monocotyledons	Zingiberaceae	*Amomum*	*Amomum muricarpum*	JN043951
Monocotyledons	Zingiberaceae	*Amomum*	*Amomum neoaurantiacum*	JN043952
Monocotyledons	Zingiberaceae	*Amomum*	*Amomum neoaurantiacum*	JN043953

Monocotyledons	Zingiberaceae	*Amomum*	*Amomum neoaurantiacum*	JN043954
Monocotyledons	Zingiberaceae	*Amomum*	*Amomum repoeense*	JN043966
Monocotyledons	Zingiberaceae	*Amomum*	*Amomum repoeense*	JN043967
Monocotyledons	Zingiberaceae	*Amomum*	*Amomum repoeense*	JN043968
Monocotyledons	Zingiberaceae	*Amomum*	*Amomum repoeense*	JN043969
Monocotyledons	Zingiberaceae	*Amomum*	*Amomum verrucosum*	JN043976
Monocotyledons	Zingiberaceae	*Amomum*	*Amomum verrucosum*	JN043977
Monocotyledons	Zingiberaceae	*Amomum*	*Amomum verrucosum*	JN043978
Monocotyledons	Zingiberaceae	*Amomum*	*Amomum scarlatinum*	JN043970
Monocotyledons	Zingiberaceae	*Amomum*	*Amomum scarlatinum*	JN043971
Monocotyledons	Xanthorrhoeaceae	*Haworthia*	*Haworthia mucronata*	HQ646870
Monocotyledons	Xanthorrhoeaceae	*Haworthia*	*Haworthia mucronata*	HQ646871
Monocotyledons	Xanthorrhoeaceae	*Haworthia*	*Haworthia pulchella*	HQ646874
Monocotyledons	Xanthorrhoeaceae	*Haworthia*	*Haworthia pulchella*	HQ646875
Monocotyledons	Xanthorrhoeaceae	*Haworthia*	*Haworthia rossouwii*	HQ646877
Monocotyledons	Xanthorrhoeaceae	*Haworthia*	*Haworthia rossouwii*	HQ646878
Monocotyledons	Xanthorrhoeaceae	*Haworthia*	*Haworthia venosa*	HQ646890
Monocotyledons	Xanthorrhoeaceae	*Haworthia*	*Haworthia venosa*	HQ646891
Monocotyledons	Xanthorrhoeaceae	*Haworthia*	*Haworthia venosa*	HQ646892
Monocotyledons	Acoraceae	*Acorus*	*Acorus calamus*	DQ008899
Monocotyledons	Acoraceae	*Acorus*	*Acorus calamus*	EU814673
Monocotyledons	Acoraceae	*Acorus*	*Acorus calamus*	EU814674
Monocotyledons	Acoraceae	*Acorus*	*Acorus calamus*	EU814675
Monocotyledons	Acoraceae	*Acorus*	*Acorus calamus*	EU814676
Monocotyledons	Acoraceae	*Acorus*	*Acorus calamus*	JF708224
Monocotyledons	Araceae	*Lemna*	*Lemna minor*	FJ395556
Monocotyledons	Poaceae	*Oryza*	*Oryza rufipogon*	GU575244
Monocotyledons	Poaceae	*Oryza*	*Oryza rufipogon*	GU575248
Monocotyledons	Poaceae	*Oryza*	*Oryza rufipogon*	GU575259
Monocotyledons	Poaceae	*Oryza*	*Oryza rufipogon*	GU575260
Monocotyledons	Poaceae	*Oryza*	*Oryza sativa*	GU575253
Monocotyledons	Poaceae	*Oryza*	*Oryza sativa*	GU575271
Monocotyledons	Poaceae	*Oryza*	*Oryza sativa*	GU575284
Monocotyledons	Poaceae	*Oryza*	*Oryza sativa*	GQ435008
Monocotyledons	Poaceae	*Panicum*	*Panicum miliaceum*	HQ596781
Monocotyledons	Poaceae	*Panicum*	*Panicum miliaceum*	FR667854
Monocotyledons	Poaceae	*Poa*	*Poa pratensis*	FJ395514
Monocotyledons	Poaceae	*Poa*	*Poa pratensis*	HQ596794
Monocotyledons	Poaceae	*Poa*	*Poa pratensis*	FJ766223
Monocotyledons	Poaceae	*Bambusa*	*Bambusa multiplex*	GU390999
Monocotyledons	Poaceae	*Bambusa*	*Bambusa multiplex*	GU063085
Monocotyledons	Poaceae	*Thinopyrum*	*Thinopyrum bessarabicum*	HQ221789
Monocotyledons	Poaceae	*Thinopyrum*	*Thinopyrum bessarabicum*	HQ221813
Monocotyledons	Poaceae	*Thinopyrum*	*Thinopyrum bessarabicum*	HQ652840
Monocotyledons	Poaceae	*Pseudoroegneria*	*Pseudoroegneria spicata*	HQ221770
Monocotyledons	Poaceae	*Pseudoroegneria*	*Pseudoroegneria spicata*	HQ652795
Monocotyledons	Poaceae	*Cenchrus*	*Cenchrus ciliaris*	HQ876941
Monocotyledons	Poaceae	*Cenchrus*	*Cenchrus ciliaris*	HQ876942
Monocotyledons	Poaceae	*Glyceria*	*Glyceria borealis*	DQ665463
Monocotyledons	Poaceae	*Glyceria*	*Glyceria borealis*	DQ665464
Monocotyledons	Poaceae	*Glyceria*	*Glyceria borealis*	DQ665465
Monocotyledons	Poaceae	*Glyceria*	*Glyceria borealis*	DQ665466
Monocotyledons	Poaceae	*Glyceria*	*Glyceria borealis*	DQ665467

Monocotyledons	Poaceae	*Glyceria*	*Glyceria borealis*	DQ665468
Monocotyledons	Poaceae	*Glyceria*	*Glyceria borealis*	DQ665469
Monocotyledons	Poaceae	*Glyceria*	*Glyceria borealis*	DQ665470
Monocotyledons	Poaceae	*Glyceria*	*Glyceria striata*	DQ665535
Monocotyledons	Poaceae	*Glyceria*	*Glyceria striata*	DQ665536
Monocotyledons	Poaceae	*Glyceria*	*Glyceria striata*	DQ665537
Monocotyledons	Poaceae	*Glyceria*	*Glyceria striata*	DQ665538
Monocotyledons	Poaceae	*Glyceria*	*Glyceria striata*	DQ665539
Monocotyledons	Poaceae	*Glyceria*	*Glyceria striata*	DQ665540
Monocotyledons	Poaceae	*Glyceria*	*Glyceria striata*	DQ665541
Monocotyledons	Poaceae	*Glyceria*	*Glyceria striata*	DQ665542
Monocotyledons	Poaceae	*Glyceria*	*Glyceria striata*	DQ665543
Monocotyledons	Poaceae	*Glyceria*	*Glyceria striata*	DQ665544
Monocotyledons	Poaceae	*Glyceria*	*Glyceria striata*	DQ665545
Monocotyledons	Poaceae	*Glyceria*	*Glyceria striata*	DQ665546
Monocotyledons	Poaceae	*Glyceria*	*Glyceria striata*	HQ596721
Monocotyledons	Poaceae	*Glyceria*	*Glyceria striata*	HQ596722
Monocotyledons	Poaceae	*Panicum*	*Panicum virgatum*	JF901575
Monocotyledons	Poaceae	*Panicum*	*Panicum virgatum*	JF901576
Monocotyledons	Dioscoreaceae	*Tacca*	*Tacca plantaginea*	JN047352
Monocotyledons	Dioscoreaceae	*Tacca*	*Tacca plantaginea*	JN047353
Monocotyledons	Dioscoreaceae	*Tacca*	*Tacca plantaginea*	JN047354
Monocotyledons	Dioscoreaceae	*Tacca*	*Tacca plantaginea*	JN047355
Monocotyledons	Dioscoreaceae	*Tacca*	*Tacca plantaginea*	JN047356
Monocotyledons	Dioscoreaceae	*Tacca*	*Tacca plantaginea*	JN047357
Monocotyledons	Dioscoreaceae	*Tacca*	*Tacca plantaginea*	JN047358
Monocotyledons	Poaceae	*Eragrostis*	*Eragrostis cilianensis*	HQ876961
Monocotyledons	Poaceae	*Eragrostis*	*Eragrostis cilianensis*	HQ876962
Monocotyledons	Poaceae	*Eragrostis*	*Eragrostis cilianensis*	HQ876963
Monocotyledons	Poaceae	*Eragrostis*	*Eragrostis cilianensis*	HQ876964
Monocotyledons	Poaceae	*Glyceria*	*Glyceria declinata*	DQ665475
Monocotyledons	Poaceae	*Glyceria*	*Glyceria declinata*	DQ665476
Monocotyledons	Poaceae	*Glyceria*	*Glyceria declinata*	DQ665477
Monocotyledons	Poaceae	*Glyceria*	*Glyceria declinata*	DQ665478
Monocotyledons	Poaceae	*Glyceria*	*Glyceria declinata*	DQ665479
Monocotyledons	Poaceae	*Glyceria*	*Glyceria declinata*	DQ665480
Monocotyledons	Poaceae	*Glyceria*	*Glyceria declinata*	DQ665481
Monocotyledons	Poaceae	*Glyceria*	*Glyceria declinata*	DQ665482
Monocotyledons	Poaceae	*Glyceria*	*Glyceria declinata*	DQ665483
Monocotyledons	Poaceae	*Glyceria*	*Glyceria declinata*	DQ665484
Monocotyledons	Orchidaceae	*Cypripedium*	*Cypripedium calceolus*	GQ248281
Monocotyledons	Orchidaceae	*Cypripedium*	*Cypripedium calceolus*	JF796982
Monocotyledons	Orchidaceae	*Cypripedium*	*Cypripedium flavum*	JQ004986
Monocotyledons	Orchidaceae	*Cypripedium*	*Cypripedium flavum*	JF796948
Monocotyledons	Acoraceae	*Acorus*	*Acorus gramineus*	GQ248236
Monocotyledons	Acoraceae	*Acorus*	*Acorus gramineus*	FJ874978
Monocotyledons	Acoraceae	*Acorus*	*Acorus gramineus*	FJ874979
Monocotyledons	Acoraceae	*Acorus*	*Acorus gramineus*	FJ874980
Monocotyledons	Acoraceae	*Acorus*	*Acorus gramineus*	FJ874981
Monocotyledons	Acoraceae	*Acorus*	*Acorus gramineus*	FJ874982
Monocotyledons	Acoraceae	*Acorus*	*Acorus gramineus*	DQ008892
Monocotyledons	Acoraceae	*Acorus*	*Acorus gramineus*	DQ008895
Monocotyledons	Acoraceae	*Acorus*	*Acorus gramineus*	DQ008898

Monocotyledons	Acoraceae	*Acorus*	*Acorus gramineus*	EU814677
Monocotyledons	Acoraceae	*Acorus*	*Acorus gramineus*	EU814679
Monocotyledons	Poaceae	*Bambusa*	*Bambusa vulgaris*	FJ644251
Monocotyledons	Poaceae	*Bambusa*	*Bambusa vulgaris*	GU063097
Monocotyledons	Poaceae	*Bambusa*	*Bambusa vulgaris*	EF589631
Monocotyledons	Poaceae	*Jarava*	*Jarava ichu*	EU489267
Monocotyledons	Poaceae	*Jarava*	*Jarava ichu*	EU489268
Monocotyledons	Dioscoreaceae	*Tacca*	*Tacca leontopetaloides*	JN047344
Monocotyledons	Dioscoreaceae	*Tacca*	*Tacca leontopetaloides*	JN047345
Monocotyledons	Dioscoreaceae	*Tacca*	*Tacca leontopetaloides*	JN047346
Monocotyledons	Dioscoreaceae	*Tacca*	*Tacca leontopetaloides*	JN047347
Monocotyledons	Dioscoreaceae	*Tacca*	*Tacca leontopetaloides*	JN047348
Monocotyledons	Dioscoreaceae	*Tacca*	*Tacca leontopetaloides*	JN047349
Monocotyledons	Dioscoreaceae	*Tacca*	*Tacca leontopetaloides*	JN047350
Monocotyledons	Dioscoreaceae	*Tacca*	*Tacca leontopetaloides*	JN047351
Monocotyledons	Poaceae	*Glyceria*	*Glyceria grandis*	DQ665507
Monocotyledons	Poaceae	*Glyceria*	*Glyceria grandis*	DQ665508
Monocotyledons	Poaceae	*Glyceria*	*Glyceria grandis*	DQ665509
Monocotyledons	Poaceae	*Glyceria*	*Glyceria grandis*	HQ596720
Monocotyledons	Dioscoreaceae	*Tacca*	*Tacca chantieri*	JN047324
Monocotyledons	Dioscoreaceae	*Tacca*	*Tacca chantieri*	JN047325
Monocotyledons	Dioscoreaceae	*Tacca*	*Tacca chantieri*	JN047326
Monocotyledons	Dioscoreaceae	*Tacca*	*Tacca chantieri*	JN047327
Monocotyledons	Dioscoreaceae	*Tacca*	*Tacca chantieri*	JN047328
Monocotyledons	Poaceae	*Thinopyrum*	*Thinopyrum intermedium*	HQ221771
Monocotyledons	Poaceae	*Thinopyrum*	*Thinopyrum intermedium*	HQ221772
Monocotyledons	Poaceae	*Thinopyrum*	*Thinopyrum intermedium*	HQ221773
Monocotyledons	Poaceae	*Thinopyrum*	*Thinopyrum intermedium*	HQ221774
Monocotyledons	Poaceae	*Thinopyrum*	*Thinopyrum intermedium*	HQ221775
Monocotyledons	Poaceae	*Thinopyrum*	*Thinopyrum intermedium*	HQ221776
Monocotyledons	Poaceae	*Thinopyrum*	*Thinopyrum intermedium*	HQ221777
Monocotyledons	Poaceae	*Thinopyrum*	*Thinopyrum intermedium*	HQ221778
Monocotyledons	Poaceae	*Thinopyrum*	*Thinopyrum intermedium*	HQ221779
Monocotyledons	Poaceae	*Thinopyrum*	*Thinopyrum intermedium*	HQ221780
Monocotyledons	Poaceae	*Thinopyrum*	*Thinopyrum intermedium*	HQ221781
Monocotyledons	Poaceae	*Thinopyrum*	*Thinopyrum intermedium*	HQ221782
Monocotyledons	Poaceae	*Thinopyrum*	*Thinopyrum intermedium*	HQ221783
Monocotyledons	Poaceae	*Thinopyrum*	*Thinopyrum intermedium*	HQ221784
Monocotyledons	Poaceae	*Thinopyrum*	*Thinopyrum intermedium*	HQ221785
Monocotyledons	Poaceae	*Thinopyrum*	*Thinopyrum intermedium*	HQ221786
Monocotyledons	Poaceae	*Thinopyrum*	*Thinopyrum intermedium*	HQ221787
Monocotyledons	Poaceae	*Thinopyrum*	*Thinopyrum intermedium*	HQ221788
Monocotyledons	Poaceae	*Thinopyrum*	*Thinopyrum intermedium*	HQ221790
Monocotyledons	Poaceae	*Thinopyrum*	*Thinopyrum intermedium*	HQ221792
Monocotyledons	Poaceae	*Thinopyrum*	*Thinopyrum intermedium*	HQ221793
Monocotyledons	Poaceae	*Thinopyrum*	*Thinopyrum intermedium*	HQ221794
Monocotyledons	Poaceae	*Thinopyrum*	*Thinopyrum intermedium*	HQ221795
Monocotyledons	Poaceae	*Thinopyrum*	*Thinopyrum intermedium*	HQ221796
Monocotyledons	Poaceae	*Thinopyrum*	*Thinopyrum intermedium*	HQ221797
Monocotyledons	Poaceae	*Thinopyrum*	*Thinopyrum intermedium*	HQ221798
Monocotyledons	Poaceae	*Thinopyrum*	*Thinopyrum intermedium*	HQ221799
Monocotyledons	Poaceae	*Thinopyrum*	*Thinopyrum intermedium*	HQ221800
Monocotyledons	Poaceae	*Thinopyrum*	*Thinopyrum intermedium*	HQ221801

Monocotyledons	Poaceae	*Thinopyrum*	*Thinopyrum intermedium*	HQ221802
Monocotyledons	Poaceae	*Thinopyrum*	*Thinopyrum intermedium*	HQ221803
Monocotyledons	Poaceae	*Thinopyrum*	*Thinopyrum intermedium*	HQ221804
Monocotyledons	Poaceae	*Thinopyrum*	*Thinopyrum intermedium*	HQ221805
Monocotyledons	Poaceae	*Thinopyrum*	*Thinopyrum intermedium*	HQ221806
Monocotyledons	Poaceae	*Poa*	*Poa annua*	HQ596792
Monocotyledons	Poaceae	*Poa*	*Poa annua*	HQ596793
Monocotyledons	Poaceae	*Poa*	*Poa annua*	FJ493300
Monocotyledons	Poaceae	*Poa*	*Poa annua*	EU750479
Monocotyledons	Poaceae	*Poa*	*Poa annua*	EU750480
Monocotyledons	Orchidaceae	*Dendrobium*	*Dendrobium nobile*	FJ216469
Monocotyledons	Orchidaceae	*Dendrobium*	*Dendrobium nobile*	FJ216482
Monocotyledons	Orchidaceae	*Dendrobium*	*Dendrobium nobile*	GQ248287
Monocotyledons	Orchidaceae	*Dendrobium*	*Dendrobium nobile*	EU672797
Monocotyledons	Orchidaceae	*Dendrobium*	*Dendrobium nobile*	EF590687
Monocotyledons	Orchidaceae	*Dendrobium*	*Dendrobium nobile*	EU887941
Monocotyledons	Orchidaceae	*Dendrobium*	*Dendrobium nobile*	EU887942
Monocotyledons	Orchidaceae	*Dendrobium*	*Dendrobium fimbriatum*	FJ216468
Monocotyledons	Orchidaceae	*Dendrobium*	*Dendrobium fimbriatum*	EU672798
Monocotyledons	Orchidaceae	*Dendrobium*	*Dendrobium loddigesii*	EU881986
Monocotyledons	Orchidaceae	*Dendrobium*	*Dendrobium loddigesii*	EU887940
Monocotyledons	Orchidaceae	*Dendrobium*	*Dendrobium thyrsiflorum*	EU672799
Monocotyledons	Orchidaceae	*Dendrobium*	*Dendrobium thyrsiflorum*	EU887929
Monocotyledons	Poaceae	*Nassella*	*Nassella tenuis*	EU204723
Monocotyledons	Poaceae	*Nassella*	*Nassella tenuis*	EU204724
Monocotyledons	Orchidaceae	*Cattleya*	*Cattleya aclandiae*	GQ248259
Monocotyledons	Orchidaceae	*Cattleya*	*Cattleya aclandiae*	EU140023
Monocotyledons	Orchidaceae	*Cattleya*	*Cattleya labiata*	EU140040
Monocotyledons	Orchidaceae	*Cattleya*	*Cattleya labiata*	EU140041
Monocotyledons	Acoraceae	*Acorus*	*Acorus tatarinowii*	GQ435343
Monocotyledons	Acoraceae	*Acorus*	*Acorus tatarinowii*	GQ435344
Monocotyledons	Acoraceae	*Acorus*	*Acorus tatarinowii*	GQ435345
Monocotyledons	Acoraceae	*Acorus*	*Acorus tatarinowii*	FJ874983
Monocotyledons	Acoraceae	*Acorus*	*Acorus tatarinowii*	FJ874984
Monocotyledons	Acoraceae	*Acorus*	*Acorus tatarinowii*	FJ874985
Monocotyledons	Acoraceae	*Acorus*	*Acorus tatarinowii*	DQ008890
Monocotyledons	Acoraceae	*Acorus*	*Acorus tatarinowii*	DQ008891
Monocotyledons	Acoraceae	*Acorus*	*Acorus tatarinowii*	DQ008897
Monocotyledons	Acoraceae	*Acorus*	*Acorus tatarinowii*	EU814678
Monocotyledons	Poaceae	*Pseudoroegneria*	*Pseudoroegneria strigosa*	HQ221768
Monocotyledons	Poaceae	*Pseudoroegneria*	*Pseudoroegneria strigosa*	HQ652797
Monocotyledons	Orchidaceae	*Oncidium*	*Oncidium hybrid cultivar*	GU136268
Monocotyledons	Orchidaceae	*Oncidium*	*Oncidium hybrid cultivar*	GU136276
Monocotyledons	Orchidaceae	*Oncidium*	*Oncidium hybrid cultivar*	GU136284
Monocotyledons	Orchidaceae	*Oncidium*	*Oncidium hybrid cultivar*	GU175343
Monocotyledons	Orchidaceae	*Oncidium*	*Oncidium hybrid cultivar*	GU175351
Monocotyledons	Orchidaceae	*Cattleya*	*Cattleya bicolor*	EU140026
Monocotyledons	Orchidaceae	*Cattleya*	*Cattleya bicolor*	EU140027
Monocotyledons	Orchidaceae	*Cattleya*	*Cattleya nobilior*	GQ248260
Monocotyledons	Orchidaceae	*Cattleya*	*Cattleya nobilior*	EU140047
Monocotyledons	Orchidaceae	*Encyclia*	*Encyclia cordigera*	EU213732
Monocotyledons	Orchidaceae	*Encyclia*	*Encyclia cordigera*	EU213733
Monocotyledons	Orchidaceae	*Encyclia*	*Encyclia cordigera*	EU213734

Monocotyledons	Orchidaceae	*Encyclia*	*Encyclia cordigera*	EU213735
Monocotyledons	Orchidaceae	*Dendrobium*	*Dendrobium devonianum*	FJ216478
Monocotyledons	Orchidaceae	*Dendrobium*	*Dendrobium devonianum*	GQ162802
Monocotyledons	Orchidaceae	*Dendrobium*	*Dendrobium gratiosissimum*	FJ216472
Monocotyledons	Orchidaceae	*Dendrobium*	*Dendrobium gratiosissimum*	GQ153536
Monocotyledons	Orchidaceae	*Dendrobium*	*Dendrobium moniliforme*	GQ162803
Monocotyledons	Orchidaceae	*Dendrobium*	*Dendrobium moniliforme*	EU672796
Monocotyledons	Orchidaceae	*Dendrobium*	*Dendrobium moniliforme*	EU887938
Monocotyledons	Orchidaceae	*Dendrobium*	*Dendrobium officinale*	FJ216479
Monocotyledons	Orchidaceae	*Dendrobium*	*Dendrobium officinale*	GQ153537
Monocotyledons	Orchidaceae	*Phalaenopsis*	*Phalaenopsis amabilis*	FJ460367
Monocotyledons	Orchidaceae	*Phalaenopsis*	*Phalaenopsis amabilis*	FJ460368
Monocotyledons	Orchidaceae	*Phalaenopsis*	*Phalaenopsis amabilis*	FJ460369
Monocotyledons	Orchidaceae	*Phalaenopsis*	*Phalaenopsis amabilis*	FJ460370
Monocotyledons	Orchidaceae	*Phalaenopsis*	*Phalaenopsis amabilis*	FJ460373
Monocotyledons	Orchidaceae	*Phalaenopsis*	*Phalaenopsis amabilis*	FJ460374
Monocotyledons	Orchidaceae	*Phalaenopsis*	*Phalaenopsis amabilis*	FJ460388
Monocotyledons	Orchidaceae	*Phalaenopsis*	*Phalaenopsis amabilis*	FJ460389
Monocotyledons	Orchidaceae	*Phalaenopsis*	*Phalaenopsis amabilis*	FJ460390
Monocotyledons	Orchidaceae	*Phalaenopsis*	*Phalaenopsis amabilis*	FJ460394
Monocotyledons	Orchidaceae	*Phalaenopsis*	*Phalaenopsis amabilis*	FJ460397
Monocotyledons	Orchidaceae	*Phalaenopsis*	*Phalaenopsis amabilis*	FJ460399
Monocotyledons	Orchidaceae	*Phalaenopsis*	*Phalaenopsis amabilis*	FJ460400
Monocotyledons	Orchidaceae	*Phalaenopsis*	*Phalaenopsis amabilis*	FJ460404
Monocotyledons	Poaceae	*Setaria*	*Setaria verticillata*	FJ766230
Monocotyledons	Poaceae	*Setaria*	*Setaria verticillata*	HQ876991
Monocotyledons	Orchidaceae	*Cypripedium*	*Cypripedium parviflorum*	HQ596661
Monocotyledons	Orchidaceae	*Cypripedium*	*Cypripedium parviflorum*	JF796968
Monocotyledons	Orchidaceae	*Dendrobium*	*Dendrobium crepidatum*	FJ216488
Monocotyledons	Orchidaceae	*Dendrobium*	*Dendrobium crepidatum*	GQ162800
Monocotyledons	Orchidaceae	*Dendrobium*	*Dendrobium crepidatum*	EU887935
Monocotyledons	Orchidaceae	*Dendrobium*	*Dendrobium aphyllum*	FJ216486
Monocotyledons	Orchidaceae	*Dendrobium*	*Dendrobium aphyllum*	EU887939
Monocotyledons	Orchidaceae	*Dendrobium*	*Dendrobium chrysanthum*	GQ162805
Monocotyledons	Orchidaceae	*Dendrobium*	*Dendrobium chrysanthum*	EU887934
Monocotyledons	Orchidaceae	*Oncidium*	*Oncidium harryanum*	FJ564027
Monocotyledons	Orchidaceae	*Oncidium*	*Oncidium harryanum*	FJ564165
Monocotyledons	Orchidaceae	*Oncidium*	*Oncidium cheirophorum*	FJ564148
Monocotyledons	Orchidaceae	*Oncidium*	*Oncidium cheirophorum*	FJ564308
Monocotyledons	Orchidaceae	*Oncidium*	*Oncidium ghiesbreghtianum*	FJ564050
Monocotyledons	Orchidaceae	*Oncidium*	*Oncidium ghiesbreghtianum*	FJ564152
Monocotyledons	Orchidaceae	*Oncidium*	*Oncidium ghiesbreghtianum*	FJ564657
Monocotyledons	Orchidaceae	*Oncidium*	*Oncidium reichenheimii*	FJ564013
Monocotyledons	Orchidaceae	*Oncidium*	*Oncidium reichenheimii*	FJ564175
Monocotyledons	Orchidaceae	*Oncidium*	*Oncidium leucochilum*	FJ564012
Monocotyledons	Orchidaceae	*Oncidium*	*Oncidium leucochilum*	FJ564635
Monocotyledons	Orchidaceae	*Oncidium*	*Oncidium poikilostalix*	FJ564029
Monocotyledons	Orchidaceae	*Oncidium*	*Oncidium poikilostalix*	FJ564660
Monocotyledons	Poaceae	*Cenchrus*	*Cenchrus purpureus*	GU135321
Monocotyledons	Poaceae	*Cenchrus*	*Cenchrus purpureus*	GU135404
Monocotyledons	Orchidaceae	*Oncidium*	*Oncidium vulcanicum*	FJ564153
Monocotyledons	Orchidaceae	*Oncidium*	*Oncidium vulcanicum*	FJ564248
Monocotyledons	Orchidaceae	*Oncidium*	*Oncidium aureum*	FJ564096

Monocotyledons	Orchidaceae	*Oncidium*	*Oncidium aureum*	FJ564562
Monocotyledons	Orchidaceae	*Oncidium*	*Oncidium armatum*	FJ564169
Monocotyledons	Orchidaceae	*Oncidium*	*Oncidium armatum*	FJ564193
Monocotyledons	Orchidaceae	*Oncidium*	*Oncidium aspidorhinum*	FJ564203
Monocotyledons	Orchidaceae	*Oncidium*	*Oncidium aspidorhinum*	FJ564256
Monocotyledons	Orchidaceae	*Oncidium*	*Oncidium cirrhosum*	FJ564032
Monocotyledons	Orchidaceae	*Oncidium*	*Oncidium cirrhosum*	FJ564180
Monocotyledons	Orchidaceae	*Oncidium*	*Oncidium cirrhosum*	FJ564569
Monocotyledons	Orchidaceae	*Oncidium*	*Oncidium alexandrae*	FJ564166
Monocotyledons	Orchidaceae	*Oncidium*	*Oncidium alexandrae*	FJ564188
Monocotyledons	Orchidaceae	*Oncidium*	*Oncidium lehmannii*	FJ564484
Monocotyledons	Orchidaceae	*Oncidium*	*Oncidium lehmannii*	FJ564541
Monocotyledons	Orchidaceae	*Oncidium*	*Oncidium epidendroides*	FJ564173
Monocotyledons	Orchidaceae	*Oncidium*	*Oncidium epidendroides*	FJ564201
Monocotyledons	Orchidaceae	*Oncidium*	*Oncidium epidendroides*	FJ564241
Monocotyledons	Orchidaceae	*Oncidium*	*Oncidium epidendroides*	FJ564488
Monocotyledons	Orchidaceae	*Oncidium*	*Oncidium hallii*	FJ563973
Monocotyledons	Orchidaceae	*Oncidium*	*Oncidium hallii*	FJ564159
Monocotyledons	Orchidaceae	*Oncidium*	*Oncidium luteopurpureum*	FJ564071
Monocotyledons	Orchidaceae	*Oncidium*	*Oncidium luteopurpureum*	FJ564540
Monocotyledons	Orchidaceae	*Oncidium*	*Oncidium nevadense*	FJ564090
Monocotyledons	Orchidaceae	*Oncidium*	*Oncidium nevadense*	FJ564150
Monocotyledons	Orchidaceae	*Oncidium*	*Oncidium portmannii*	FJ564164
Monocotyledons	Orchidaceae	*Oncidium*	*Oncidium portmannii*	FJ564172
Monocotyledons	Orchidaceae	*Oncidium*	*Oncidium portmannii*	FJ564490
Monocotyledons	Orchidaceae	*Oncidium*	*Oncidium tenuoides*	FJ564192
Monocotyledons	Orchidaceae	*Oncidium*	*Oncidium tenuoides*	FJ564531
Monocotyledons	Orchidaceae	*Oncidium*	*Oncidium baueri*	FJ564224
Monocotyledons	Orchidaceae	*Oncidium*	*Oncidium baueri*	FJ564534
Monocotyledons	Orchidaceae	*Oncidium*	*Oncidium chrysomorphum*	FJ564189
Monocotyledons	Orchidaceae	*Oncidium*	*Oncidium chrysomorphum*	FJ564513
Monocotyledons	Orchidaceae	*Oncidium*	*Oncidium endocharis*	FJ564088
Monocotyledons	Orchidaceae	*Oncidium*	*Oncidium endocharis*	FJ564369
Monocotyledons	Orchidaceae	*Oncidium*	*Oncidium fuscatum*	FJ564038
Monocotyledons	Orchidaceae	*Oncidium*	*Oncidium fuscatum*	FJ564227
Monocotyledons	Orchidaceae	*Oncidium*	*Oncidium heteranthum*	FJ564537
Monocotyledons	Orchidaceae	*Oncidium*	*Oncidium heteranthum*	FJ564618
Monocotyledons	Orchidaceae	*Oncidium*	*Oncidium incurvum*	FJ564437
Monocotyledons	Orchidaceae	*Oncidium*	*Oncidium incurvum*	FJ564630
Monocotyledons	Orchidaceae	*Oncidium*	*Oncidium isthmi*	FJ564221
Monocotyledons	Orchidaceae	*Oncidium*	*Oncidium isthmi*	FJ564543
Monocotyledons	Orchidaceae	*Oncidium*	*Oncidium retusum*	FJ564005
Monocotyledons	Orchidaceae	*Oncidium*	*Oncidium retusum*	FJ564215
Monocotyledons	Orchidaceae	*Oncidium*	*Oncidium retusum*	FJ564578
Monocotyledons	Orchidaceae	*Oncidium*	*Oncidium retusum*	FJ564580
Monocotyledons	Orchidaceae	*Oncidium*	*Oncidium gramineum*	FJ564238
Monocotyledons	Orchidaceae	*Oncidium*	*Oncidium gramineum*	FJ564500
Monocotyledons	Orchidaceae	*Oncidium*	*Oncidium gramineum*	FJ564617
Monocotyledons	Orchidaceae	*Oncidium*	*Oncidium tigroides*	FJ564548
Monocotyledons	Orchidaceae	*Oncidium*	*Oncidium tigroides*	FJ564570
Monocotyledons	Orchidaceae	*Dendrobium*	*Dendrobium chrysotoxum*	EU672792
Monocotyledons	Orchidaceae	*Dendrobium*	*Dendrobium chrysotoxum*	EU887923
Monocotyledons	Orchidaceae	*Dendrobium*	*Dendrobium chrysotoxum*	EU887924

Monocotyledons	Orchidaceae	*Dendrobium*	*Dendrobium chrysotoxum*	EU887925
Monocotyledons	Orchidaceae	*Dendrobium*	*Dendrobium densiflorum*	FJ216480
Monocotyledons	Orchidaceae	*Dendrobium*	*Dendrobium densiflorum*	FJ216487
Monocotyledons	Orchidaceae	*Dendrobium*	*Dendrobium lindleyi*	GQ248286
Monocotyledons	Orchidaceae	*Dendrobium*	*Dendrobium lindleyi*	EF590688
Monocotyledons	Orchidaceae	*Dendrobium*	*Dendrobium williamsonii*	EU672795
Monocotyledons	Orchidaceae	*Dendrobium*	*Dendrobium williamsonii*	EU887927
Monocotyledons	Poaceae	*Eragrostis*	*Eragrostis tenuifolia*	GQ248300
Monocotyledons	Poaceae	*Eragrostis*	*Eragrostis tenuifolia*	HQ876971
Monocotyledons	Poaceae	*Eragrostis*	*Eragrostis tenuifolia*	HQ876972
Monocotyledons	Dioscoreaceae	*Tacca*	*Tacca integrifolia*	JN047329
Monocotyledons	Dioscoreaceae	*Tacca*	*Tacca integrifolia*	JN047330
Monocotyledons	Dioscoreaceae	*Tacca*	*Tacca integrifolia*	JN047331
Monocotyledons	Dioscoreaceae	*Tacca*	*Tacca integrifolia*	JN047332
Monocotyledons	Dioscoreaceae	*Tacca*	*Tacca integrifolia*	JN047333
Monocotyledons	Dioscoreaceae	*Tacca*	*Tacca integrifolia*	JN047334
Monocotyledons	Dioscoreaceae	*Tacca*	*Tacca integrifolia*	JN047335
Monocotyledons	Dioscoreaceae	*Tacca*	*Tacca integrifolia*	JN047336
Monocotyledons	Dioscoreaceae	*Tacca*	*Tacca integrifolia*	JN047337
Monocotyledons	Dioscoreaceae	*Tacca*	*Tacca integrifolia*	JN047338
Monocotyledons	Dioscoreaceae	*Tacca*	*Tacca integrifolia*	JN047339
Monocotyledons	Dioscoreaceae	*Tacca*	*Tacca integrifolia*	JN047340
Monocotyledons	Dioscoreaceae	*Tacca*	*Tacca integrifolia*	JN047341
Monocotyledons	Dioscoreaceae	*Tacca*	*Tacca integrifolia*	JN047342
Monocotyledons	Dioscoreaceae	*Tacca*	*Tacca integrifolia*	JN047343
Monocotyledons	Orchidaceae	*Dendrobium*	*Dendrobium primulinum*	GQ153535
Monocotyledons	Orchidaceae	*Dendrobium*	*Dendrobium primulinum*	EU887936
Monocotyledons	Orchidaceae	*Dendrobium*	*Dendrobium primulinum*	EU887937
Monocotyledons	Orchidaceae	*Dendrobium*	*Dendrobium wardianum*	GQ162801
Monocotyledons	Orchidaceae	*Dendrobium*	*Dendrobium wardianum*	EU887930
Monocotyledons	Orchidaceae	*Dendrobium*	*Dendrobium wardianum*	EU887931
Monocotyledons	Orchidaceae	*Dendrobium*	*Dendrobium hancockii*	FJ216481
Monocotyledons	Orchidaceae	*Dendrobium*	*Dendrobium hancockii*	EU672800
Monocotyledons	Poaceae	*Nassella*	*Nassella trichotoma*	EU489305
Monocotyledons	Poaceae	*Nassella*	*Nassella trichotoma*	EU204726
Monocotyledons	Poaceae	*Nassella*	*Nassella trichotoma*	EU204727
Monocotyledons	Poaceae	*Bambusa*	*Bambusa bambos*	GU390993
Monocotyledons	Poaceae	*Bambusa*	*Bambusa bambos*	GU063075
Monocotyledons	Poaceae	*Eragrostis*	*Eragrostis heteromera*	HQ876966
Monocotyledons	Poaceae	*Eragrostis*	*Eragrostis heteromera*	HQ876967
Monocotyledons	Orchidaceae	*Phalaenopsis*	*Phalaenopsis aphrodite*	FJ460375
Monocotyledons	Orchidaceae	*Phalaenopsis*	*Phalaenopsis aphrodite*	FJ460376
Monocotyledons	Orchidaceae	*Phalaenopsis*	*Phalaenopsis aphrodite*	FJ460377
Monocotyledons	Orchidaceae	*Phalaenopsis*	*Phalaenopsis aphrodite*	FJ460378
Monocotyledons	Orchidaceae	*Phalaenopsis*	*Phalaenopsis aphrodite*	FJ460379
Monocotyledons	Orchidaceae	*Phalaenopsis*	*Phalaenopsis aphrodite*	FJ460384
Monocotyledons	Orchidaceae	*Phalaenopsis*	*Phalaenopsis aphrodite*	FJ460401
Monocotyledons	Orchidaceae	*Phalaenopsis*	*Phalaenopsis aphrodite*	FJ460402
Monocotyledons	Orchidaceae	*Phalaenopsis*	*Phalaenopsis aphrodite*	FJ460403
Monocotyledons	Orchidaceae	*Phalaenopsis*	*Phalaenopsis sanderiana*	FJ460366
Monocotyledons	Orchidaceae	*Phalaenopsis*	*Phalaenopsis sanderiana*	FJ460380
Monocotyledons	Orchidaceae	*Phalaenopsis*	*Phalaenopsis sanderiana*	FJ460381
Monocotyledons	Orchidaceae	*Bulbophyllum*	*Bulbophyllum nutans*	EF200433

Monocotyledons	Orchidaceae	*Bulbophyllum*	*Bulbophyllum nutans*	EF200434
Monocotyledons	Orchidaceae	*Bulbophyllum*	*Bulbophyllum nutans*	EF200435
Monocotyledons	Orchidaceae	*Bulbophyllum*	*Bulbophyllum nutans*	EF200436
Monocotyledons	Poaceae	*Urochloa*	*Urochloa dictyoneura*	HQ876937
Monocotyledons	Poaceae	*Urochloa*	*Urochloa dictyoneura*	HQ876938
Monocotyledons	Poaceae	*Glyceria*	*Glyceria fluitans*	DQ665497
Monocotyledons	Poaceae	*Glyceria*	*Glyceria fluitans*	DQ665498
Monocotyledons	Poaceae	*Glyceria*	*Glyceria fluitans*	DQ665499
Monocotyledons	Poaceae	*Glyceria*	*Glyceria fluitans*	DQ665500
Monocotyledons	Poaceae	*Glyceria*	*Glyceria fluitans*	DQ665501
Monocotyledons	Poaceae	*Glyceria*	*Glyceria fluitans*	DQ665502
Monocotyledons	Poaceae	*Glyceria*	*Glyceria fluitans*	DQ665503
Monocotyledons	Poaceae	*Glyceria*	*Glyceria fluitans*	DQ665504
Monocotyledons	Poaceae	*Glyceria*	*Glyceria fluitans*	DQ665505
Monocotyledons	Poaceae	*Glyceria*	*Glyceria fluitans*	DQ665506
Monocotyledons	Acoraceae	*Acorus*	*Acorus americanus*	FJ874967
Monocotyledons	Acoraceae	*Acorus*	*Acorus americanus*	FJ874968
Monocotyledons	Acoraceae	*Acorus*	*Acorus americanus*	FJ874969
Monocotyledons	Acoraceae	*Acorus*	*Acorus americanus*	DQ008896
Monocotyledons	Acoraceae	*Acorus*	*Acorus americanus*	EU814671
Monocotyledons	Acoraceae	*Acorus*	*Acorus americanus*	EU814672
Monocotyledons	Poaceae	*Poa*	*Poa compressa*	EU750481
Monocotyledons	Poaceae	*Poa*	*Poa compressa*	EU750482
Monocotyledons	Poaceae	*Poa*	*Poa compressa*	EU750483
Monocotyledons	Poaceae	*Setaria*	*Setaria pumila*	HQ596843
Monocotyledons	Poaceae	*Setaria*	*Setaria pumila*	HQ876989
Monocotyledons	Poaceae	*Bambusa*	*Bambusa tuldoides*	GU391001
Monocotyledons	Poaceae	*Bambusa*	*Bambusa tuldoides*	GU063083
Monocotyledons	Poaceae	*Bambusa*	*Bambusa sinospinosa*	GU391000
Monocotyledons	Poaceae	*Bambusa*	*Bambusa sinospinosa*	GU063077
Monocotyledons	Poaceae	*Bambusa*	*Bambusa blumeana*	GU390994
Monocotyledons	Poaceae	*Bambusa*	*Bambusa blumeana*	GU063076
Monocotyledons	Poaceae	*Glyceria*	*Glyceria canadensis*	DQ665471
Monocotyledons	Poaceae	*Glyceria*	*Glyceria canadensis*	DQ665472
Monocotyledons	Poaceae	*Glyceria*	*Glyceria canadensis*	DQ665473
Monocotyledons	Poaceae	*Glyceria*	*Glyceria canadensis*	DQ665474
Monocotyledons	Poaceae	*Glyceria*	*Glyceria elata*	DQ665485
Monocotyledons	Poaceae	*Glyceria*	*Glyceria elata*	DQ665486
Monocotyledons	Poaceae	*Glyceria*	*Glyceria elata*	DQ665487
Monocotyledons	Poaceae	*Glyceria*	*Glyceria elata*	DQ665488
Monocotyledons	Poaceae	*Glyceria*	*Glyceria elata*	DQ665489
Monocotyledons	Poaceae	*Glyceria*	*Glyceria elata*	DQ665490
Monocotyledons	Poaceae	*Glyceria*	*Glyceria elata*	DQ665491
Monocotyledons	Poaceae	*Glyceria*	*Glyceria elata*	DQ665492
Monocotyledons	Poaceae	*Glyceria*	*Glyceria elata*	DQ665493
Monocotyledons	Poaceae	*Glyceria*	*Glyceria elata*	DQ665494
Monocotyledons	Poaceae	*Glyceria*	*Glyceria elata*	DQ665495
Monocotyledons	Poaceae	*Glyceria*	*Glyceria elata*	DQ665496
Monocotyledons	Poaceae	*Glyceria*	*Glyceria leptostachya*	DQ665510
Monocotyledons	Poaceae	*Glyceria*	*Glyceria leptostachya*	DQ665511
Monocotyledons	Poaceae	*Glyceria*	*Glyceria leptostachya*	DQ665512
Monocotyledons	Poaceae	*Glyceria*	*Glyceria leptostachya*	DQ665513
Monocotyledons	Poaceae	*Glyceria*	*Glyceria leptostachya*	DQ665514

Monocotyledons	Poaceae	*Glyceria*	*Glyceria maxima*	DQ665515
Monocotyledons	Poaceae	*Glyceria*	*Glyceria maxima*	DQ665516
Monocotyledons	Poaceae	*Glyceria*	*Glyceria notata*	DQ665518
Monocotyledons	Poaceae	*Glyceria*	*Glyceria notata*	DQ665519
Monocotyledons	Poaceae	*Glyceria*	*Glyceria occidentalis*	DQ665520
Monocotyledons	Poaceae	*Glyceria*	*Glyceria occidentalis*	DQ665521
Monocotyledons	Poaceae	*Glyceria*	*Glyceria occidentalis*	DQ665522
Monocotyledons	Poaceae	*Glyceria*	*Glyceria occidentalis*	DQ665523
Monocotyledons	Poaceae	*Glyceria*	*Glyceria occidentalis*	DQ665524
Monocotyledons	Poaceae	*Glyceria*	*Glyceria occidentalis*	DQ665525
Monocotyledons	Poaceae	*Glyceria*	*Glyceria occidentalis*	DQ665526
Monocotyledons	Poaceae	*Glyceria*	*Glyceria occidentalis*	DQ665527
Monocotyledons	Poaceae	*Glyceria*	*Glyceria occidentalis*	DQ665528
Monocotyledons	Poaceae	*Glyceria*	*Glyceria occidentalis*	DQ665529
Monocotyledons	Poaceae	*Glyceria*	*Glyceria septentrionalis*	DQ665530
Monocotyledons	Poaceae	*Glyceria*	*Glyceria septentrionalis*	DQ665531
Monocotyledons	Poaceae	*Glyceria*	*Glyceria septentrionalis*	DQ665532
Monocotyledons	Poaceae	*Glyceria*	*Glyceria septentrionalis*	DQ665533
Monocotyledons	Poaceae	*Glyceria*	*Glyceria septentrionalis*	DQ665534
Monocotyledons	Poaceae	*Jarava*	*Jarava leptostachya*	EU489270
Monocotyledons	Poaceae	*Jarava*	*Jarava leptostachya*	EU489271
Monocotyledons	Poaceae	*Nassella*	*Nassella cordobensis*	EU204713
Monocotyledons	Poaceae	*Nassella*	*Nassella cordobensis*	EU204714
Monocotyledons	Poaceae	*Nassella*	*Nassella hyalina*	EU204715
Monocotyledons	Poaceae	*Nassella*	*Nassella hyalina*	EU204716
Monocotyledons	Poaceae	*Nassella*	*Nassella hyalina*	EU204717
Monocotyledons	Poaceae	*Nassella*	*Nassella neesiana*	EU489297
Monocotyledons	Poaceae	*Nassella*	*Nassella neesiana*	EU204719
Monocotyledons	Poaceae	*Nassella*	*Nassella neesiana*	EU204720
Monocotyledons	Orchidaceae	*Bulbophyllum*	*Bulbophyllum longiflorum*	EF200405
Monocotyledons	Orchidaceae	*Bulbophyllum*	*Bulbophyllum longiflorum*	EF200406
Monocotyledons	Poaceae	*Eragrostis*	*Eragrostis pectinacea*	HQ596686
Monocotyledons	Poaceae	*Eragrostis*	*Eragrostis pectinacea*	HQ596687
Monocotyledons	Poaceae	*Eragrostis*	*Eragrostis pectinacea*	GQ248299
Monocotyledons	Poaceae	*Eragrostis*	*Eragrostis pectinacea*	EF590696
Monocotyledons	Orchidaceae	*Bulbophyllum*	*Bulbophyllum analamazoatrae*	EF200408
Monocotyledons	Orchidaceae	*Bulbophyllum*	*Bulbophyllum analamazoatrae*	EF200409
Monocotyledons	Orchidaceae	*Bulbophyllum*	*Bulbophyllum elliottii*	EF200388
Monocotyledons	Orchidaceae	*Bulbophyllum*	*Bulbophyllum elliottii*	EF200389
Monocotyledons	Orchidaceae	*Bulbophyllum*	*Bulbophyllum elliottii*	EF200390
Monocotyledons	Orchidaceae	*Bulbophyllum*	*Bulbophyllum elliottii*	EF200391
Monocotyledons	Orchidaceae	*Bulbophyllum*	*Bulbophyllum francoisii*	EF200411
Monocotyledons	Orchidaceae	*Bulbophyllum*	*Bulbophyllum francoisii*	EF200417
Monocotyledons	Orchidaceae	*Bulbophyllum*	*Bulbophyllum henrici*	EF200480
Monocotyledons	Orchidaceae	*Bulbophyllum*	*Bulbophyllum henrici*	EF200491
Monocotyledons	Orchidaceae	*Bulbophyllum*	*Bulbophyllum longivaginans*	EF200463
Monocotyledons	Orchidaceae	*Bulbophyllum*	*Bulbophyllum longivaginans*	EF200464
Monocotyledons	Orchidaceae	*Bulbophyllum*	*Bulbophyllum matitanense*	EF200393
Monocotyledons	Orchidaceae	*Bulbophyllum*	*Bulbophyllum matitanense*	EF200396
Monocotyledons	Orchidaceae	*Bulbophyllum*	*Bulbophyllum oxycalyx*	EF200412
Monocotyledons	Orchidaceae	*Bulbophyllum*	*Bulbophyllum oxycalyx*	EF200416
Monocotyledons	Orchidaceae	*Bulbophyllum*	*Bulbophyllum pachypus*	EF200466
Monocotyledons	Orchidaceae	*Bulbophyllum*	*Bulbophyllum pachypus*	EF200471

Monocotyledons	Orchidaceae	*Bulbophyllum*	*Bulbophyllum rauhii*	EF200413
Monocotyledons	Orchidaceae	*Bulbophyllum*	*Bulbophyllum rauhii*	EF200414
Monocotyledons	Orchidaceae	*Bulbophyllum*	*Bulbophyllum falcatum*	GQ339616
Monocotyledons	Orchidaceae	*Bulbophyllum*	*Bulbophyllum falcatum*	JF693811
Monocotyledons	Orchidaceae	*Cypripedium*	*Cypripedium debile*	JF796983
Monocotyledons	Orchidaceae	*Cypripedium*	*Cypripedium debile*	JF796986
Monocotyledons	Orchidaceae	*Cypripedium*	*Cypripedium debile*	JF796987
Monocotyledons	Orchidaceae	*Cypripedium*	*Cypripedium henryi*	JQ080173
Monocotyledons	Orchidaceae	*Cypripedium*	*Cypripedium henryi*	JF796956
Monocotyledons	Poaceae	*Jarava*	*Jarava eriostachya*	EU204702
Monocotyledons	Poaceae	*Jarava*	*Jarava eriostachya*	EU204703
Monocotyledons	Poaceae	*Jarava*	*Jarava eriostachya*	EU204704
Monocotyledons	Poaceae	*Jarava*	*Jarava eriostachya*	EU204705
Monocotyledons	Poaceae	*Jarava*	*Jarava humilis*	EU489263
Monocotyledons	Poaceae	*Jarava*	*Jarava humilis*	EU489264
Monocotyledons	Poaceae	*Jarava*	*Jarava humilis*	EU489265
Monocotyledons	Poaceae	*Jarava*	*Jarava humilis*	EU204732
Monocotyledons	Poaceae	*Jarava*	*Jarava humilis*	EU204733
Monocotyledons	Orchidaceae	*Encyclia*	*Encyclia alata*	EU213730
Monocotyledons	Orchidaceae	*Encyclia*	*Encyclia alata*	EU213731
Monocotyledons	Orchidaceae	*Oncidium*	*Oncidium obryzatoides*	EU213752
Monocotyledons	Orchidaceae	*Oncidium*	*Oncidium obryzatoides*	FJ564374
Monocotyledons	Orchidaceae	*Oncidium*	*Oncidium storkii*	EU213753
Monocotyledons	Orchidaceae	*Oncidium*	*Oncidium storkii*	FJ564375
Monocotyledons	Poaceae	*Jarava*	*Jarava frigida*	EU489260
Monocotyledons	Poaceae	*Jarava*	*Jarava frigida*	EU489261
Monocotyledons	Orchidaceae	*Leochilus*	*Leochilus inconspicuus*	FJ564430
Monocotyledons	Orchidaceae	*Leochilus*	*Leochilus inconspicuus*	FJ564440
Monocotyledons	Orchidaceae	*Leochilus*	*Leochilus leiboldi*	FJ564139
Monocotyledons	Orchidaceae	*Leochilus*	*Leochilus leiboldi*	FJ564428
Monocotyledons	Orchidaceae	*Oncidium*	*Oncidium echinops*	FJ564327
Monocotyledons	Orchidaceae	*Oncidium*	*Oncidium echinops*	FJ564505
Monocotyledons	Orchidaceae	*Oncidium*	*Oncidium echinops*	FJ564594
Monocotyledons	Orchidaceae	*Oncidium*	*Oncidium peruvianoides*	FJ564458
Monocotyledons	Orchidaceae	*Oncidium*	*Oncidium peruvianoides*	FJ564512
Monocotyledons	Poaceae	*Tripogon*	*Tripogon wightii*	FJ610317
Monocotyledons	Poaceae	*Tripogon*	*Tripogon wightii*	FJ610318
Monocotyledons	Poaceae	*Tripogon*	*Tripogon wightii*	FJ610319
Monocotyledons	Poaceae	*Tripogon*	*Tripogon wightii*	FJ610320
Monocotyledons	Poaceae	*Tripogon*	*Tripogon wightii*	FJ610321
Monocotyledons	Orchidaceae	*Oncidium*	*Oncidium heterodactylum*	FJ564516
Monocotyledons	Orchidaceae	*Oncidium*	*Oncidium heterodactylum*	FJ564536
Monocotyledons	Orchidaceae	*Oncidium*	*Oncidium heterodactylum*	FJ564538
Monocotyledons	Orchidaceae	*Oncidium*	*Oncidium iricolor*	FJ564371
Monocotyledons	Orchidaceae	*Oncidium*	*Oncidium iricolor*	FJ564628
Monocotyledons	Orchidaceae	*Oncidium*	*Oncidium boothianum*	FJ564535
Monocotyledons	Orchidaceae	*Oncidium*	*Oncidium boothianum*	FJ564581
Monocotyledons	Orchidaceae	*Oncidium*	*Oncidium boothianum*	FJ564588
Monocotyledons	Orchidaceae	*Oncidium*	*Oncidium boothianum*	FJ564589
Monocotyledons	Orchidaceae	*Oncidium*	*Oncidium schmidtianum*	FJ564514
Monocotyledons	Orchidaceae	*Oncidium*	*Oncidium schmidtianum*	FJ564576
Monocotyledons	Orchidaceae	*Oncidium*	*Oncidium schroederianum*	FJ564198
Monocotyledons	Orchidaceae	*Oncidium*	*Oncidium schroederianum*	FJ564429

Monocotyledons	Orchidaceae	*Cypripedium*	*Cypripedium micranthum*	GQ248282
Monocotyledons	Orchidaceae	*Cypripedium*	*Cypripedium micranthum*	JF796964
Monocotyledons	Poaceae	*Tripogon*	*Tripogon copei*	FJ707304
Monocotyledons	Poaceae	*Tripogon*	*Tripogon copei*	FJ707305
Monocotyledons	Poaceae	*Tripogon*	*Tripogon copei*	FJ707306
Monocotyledons	Poaceae	*Tripogon*	*Tripogon copei*	FJ707307
Monocotyledons	Poaceae	*Tripogon*	*Tripogon copei*	FJ707308
Monocotyledons	Poaceae	*Bambusa*	*Bambusa distegia*	GU390995
Monocotyledons	Poaceae	*Bambusa*	*Bambusa distegia*	GU063081
Monocotyledons	Orchidaceae	*Bulbophyllum*	*Bulbophyllum exaltatum*	GQ339640
Monocotyledons	Orchidaceae	*Bulbophyllum*	*Bulbophyllum exaltatum*	GQ339641
Monocotyledons	Poaceae	*Cenchrus*	*Cenchrus stramineus*	HQ876987
Monocotyledons	Poaceae	*Cenchrus*	*Cenchrus stramineus*	HQ876988
Monocotyledons	Poaceae	*Sporobolus*	*Sporobolus ioclados*	HQ876994
Monocotyledons	Poaceae	*Sporobolus*	*Sporobolus ioclados*	HQ876995
Monocotyledons	Poaceae	*Sporobolus*	*Sporobolus rangei*	HQ877000
Monocotyledons	Poaceae	*Sporobolus*	*Sporobolus rangei*	HQ877001
Monocotyledons	Poaceae	*Urochloa*	*Urochloa brachyura*	HQ877006
Monocotyledons	Poaceae	*Urochloa*	*Urochloa brachyura*	HQ877007
Monocotyledons	Poaceae	*Urochloa*	*Urochloa brachyura*	HQ877008
Monocotyledons	Dioscoreaceae	*Tacca*	*Tacca ampliplacenta*	JN047314
Monocotyledons	Dioscoreaceae	*Tacca*	*Tacca ampliplacenta*	JN047315
Monocotyledons	Dioscoreaceae	*Tacca*	*Tacca ampliplacenta*	JN047316
Monocotyledons	Dioscoreaceae	*Tacca*	*Tacca ampliplacenta*	JN047317
Monocotyledons	Dioscoreaceae	*Tacca*	*Tacca ampliplacenta*	JN047318
Monocotyledons	Dioscoreaceae	*Tacca*	*Tacca ampliplacenta*	JN047319
Monocotyledons	Dioscoreaceae	*Tacca*	*Tacca ampliplacenta*	JN047320
Monocotyledons	Dioscoreaceae	*Tacca*	*Tacca ampliplacenta*	JN047321
Monocotyledons	Dioscoreaceae	*Tacca*	*Tacca ampliplacenta*	JN047322
Monocotyledons	Dioscoreaceae	*Tacca*	*Tacca ampliplacenta*	JN047323
Monocotyledons	Dioscoreaceae	*Tacca*	*Tacca subflabellata*	JN047359
Monocotyledons	Dioscoreaceae	*Tacca*	*Tacca subflabellata*	JN047360
Monocotyledons	Dioscoreaceae	*Tacca*	*Tacca subflabellata*	JN047361
Monocotyledons	Dioscoreaceae	*Tacca*	*Tacca subflabellata*	JN047362
Monocotyledons	Poaceae	*Festuca*	*Festuca arundinacea*	HQ596699
Monocotyledons	Poaceae	*Festuca*	*Festuca arundinacea*	HQ596753
Monocotyledons	Musaceae	*Musa*	*Musa acuminata*	FJ871854
Monocotyledons	Musaceae	*Musa*	*Musa acuminata*	FJ871855
Monocotyledons	Musaceae	*Musa*	*Musa acuminata*	FJ871856
Monocotyledons	Musaceae	*Musa*	*Musa acuminata*	FJ871857
Monocotyledons	Musaceae	*Musa*	*Musa acuminata*	FJ871858
Monocotyledons	Musaceae	*Musa*	*Musa acuminata*	FJ871859
Monocotyledons	Musaceae	*Musa*	*Musa acuminata*	FJ871860
Monocotyledons	Musaceae	*Musa*	*Musa acuminata*	FJ871861
Monocotyledons	Musaceae	*Musa*	*Musa acuminata*	FJ871862
Monocotyledons	Musaceae	*Musa*	*Musa acuminata*	FJ871863
Monocotyledons	Musaceae	*Musa*	*Musa acuminata*	FJ871864
Monocotyledons	Musaceae	*Musa*	*Musa acuminata*	FJ871865
Monocotyledons	Musaceae	*Musa*	*Musa acuminata*	FJ871866
Monocotyledons	Musaceae	*Musa*	*Musa acuminata*	FJ871867
Monocotyledons	Musaceae	*Musa*	*Musa acuminata*	FJ871868
Monocotyledons	Musaceae	*Musa*	*Musa acuminata*	FJ871869
Monocotyledons	Musaceae	*Musa*	*Musa acuminata*	FJ871915

Monocotyledons	Musaceae	*Musa*	*Musa acuminata*	FJ871916
Monocotyledons	Amaryllidaceae	*Allium*	*Allium tuberosum*	GQ434884
Monocotyledons	Amaryllidaceae	*Allium*	*Allium tuberosum*	GQ434885
Monocotyledons	Amaryllidaceae	*Allium*	*Allium tuberosum*	GQ434886
Monocotyledons	Amaryllidaceae	*Allium*	*Allium tuberosum*	GQ434887
Monocotyledons	Amaryllidaceae	*Allium*	*Allium tuberosum*	GQ434888
Monocotyledons	Asparagaceae	*Asparagus*	*Asparagus officinalis*	HM990123
Monocotyledons	Asparagaceae	*Asparagus*	*Asparagus officinalis*	HM990126
Monocotyledons	Asparagaceae	*Asparagus*	*Asparagus officinalis*	HM990127
Monocotyledons	Asparagaceae	*Asparagus*	*Asparagus officinalis*	HM990129
Monocotyledons	Asparagaceae	*Asparagus*	*Asparagus officinalis*	HM990131
Monocotyledons	Asparagaceae	*Asparagus*	*Asparagus officinalis*	HM990132
Monocotyledons	Asparagaceae	*Asparagus*	*Asparagus officinalis*	HM990133
Monocotyledons	Asparagaceae	*Asparagus*	*Asparagus officinalis*	HM990134
Monocotyledons	Asparagaceae	*Asparagus*	*Asparagus officinalis*	HM990135
Monocotyledons	Asparagaceae	*Asparagus*	*Asparagus officinalis*	HM990136
Monocotyledons	Asparagaceae	*Asparagus*	*Asparagus officinalis*	HM990137
Monocotyledons	Asparagaceae	*Asparagus*	*Asparagus officinalis*	HM990139
Monocotyledons	Asparagaceae	*Asparagus*	*Asparagus officinalis*	HM990142
Monocotyledons	Asparagaceae	*Asparagus*	*Asparagus officinalis*	HM990143
Monocotyledons	Asparagaceae	*Asparagus*	*Asparagus officinalis*	HM990144
Monocotyledons	Asparagaceae	*Asparagus*	*Asparagus officinalis*	HM990146
Monocotyledons	Asparagaceae	*Asparagus*	*Asparagus officinalis*	HM990147
Monocotyledons	Pandanaceae	*Pandanus*	*Pandanus tectorius*	JN407018
Monocotyledons	Pandanaceae	*Pandanus*	*Pandanus tectorius*	JN407019
Monocotyledons	Pandanaceae	*Pandanus*	*Pandanus tectorius*	JN407020
Monocotyledons	Typhaceae	*Typha*	*Typha latifolia*	EU750605
Monocotyledons	Typhaceae	*Typha*	*Typha latifolia*	EU750606
Monocotyledons	Typhaceae	*Typha*	*Typha latifolia*	HQ913730
Monocotyledons	Commelinaceae	*Commelina*	*Commelina benghalensis*	JN044308
Monocotyledons	Commelinaceae	*Commelina*	*Commelina benghalensis*	JN044309
Monocotyledons	Commelinaceae	*Commelina*	*Commelina benghalensis*	JN044310
Monocotyledons	Bromeliaceae	*Vriesea*	*Vriesea malzinei*	FM958006
Monocotyledons	Bromeliaceae	*Vriesea*	*Vriesea malzinei*	HQ913733
Monocotyledons	Smilacaceae	*Smilax*	*Smilax china*	GU372817
Monocotyledons	Poaceae	*Kengyilia*	*Kengyilia alatavica*	JN045083
Monocotyledons	Poaceae	*Kengyilia*	*Kengyilia alatavica*	JN045084
Monocotyledons	Poaceae	*Kengyilia*	*Kengyilia alatavica*	HQ652842
Monocotyledons	Melanthiaceae	*Trillium*	*Trillium erectum*	DQ404230
Monocotyledons	Melanthiaceae	*Trillium*	*Trillium erectum*	HQ596871
Monocotyledons	Poaceae	*Festuca*	*Festuca rubra*	DQ369760
Monocotyledons	Poaceae	*Festuca*	*Festuca rubra*	DQ369762
Monocotyledons	Poaceae	*Festuca*	*Festuca rubra*	FJ395494
Monocotyledons	Poaceae	*Piptatherum*	*Piptatherum miliaceum*	EU204759
Monocotyledons	Poaceae	*Piptatherum*	*Piptatherum miliaceum*	EU204760
Monocotyledons	Poaceae	*Piptatherum*	*Piptatherum miliaceum*	EU204761
Monocotyledons	Musaceae	*Musa*	*Musa schizocarpa*	FJ871921
Monocotyledons	Musaceae	*Musa*	*Musa schizocarpa*	FJ871922
Monocotyledons	Musaceae	*Musa*	*Musa balbisiana*	FJ871870
Monocotyledons	Musaceae	*Musa*	*Musa balbisiana*	FJ871871
Monocotyledons	Musaceae	*Musa*	*Musa balbisiana*	FJ871872
Monocotyledons	Musaceae	*Musa*	*Musa balbisiana*	FJ871873
Monocotyledons	Musaceae	*Musa*	*Musa balbisiana*	FJ871874

Monocotyledons	Musaceae	*Musa*	*Musa balbisiana*	FJ871876
Monocotyledons	Melanthiaceae	*Trillium*	*Trillium grandiflorum*	DQ404229
Monocotyledons	Melanthiaceae	*Trillium*	*Trillium grandiflorum*	HQ596872
Monocotyledons	Dioscoreaceae	*Dioscorea*	*Dioscorea communis*	FJ395544
Monocotyledons	Typhaceae	*Typha*	*Typha angustifolia*	GQ435010
Monocotyledons	Typhaceae	*Typha*	*Typha angustifolia*	EU750603
Monocotyledons	Typhaceae	*Typha*	*Typha angustifolia*	EU750604
Monocotyledons	Liliaceae	*Lilium*	*Lilium pumilum*	HM053713
Monocotyledons	Liliaceae	*Lilium*	*Lilium pumilum*	HM053714
Monocotyledons	Liliaceae	*Lilium*	*Lilium pumilum*	HM053715
Monocotyledons	Liliaceae	*Lilium*	*Lilium pumilum*	HM053716
Monocotyledons	Liliaceae	*Lilium*	*Lilium pumilum*	HM053717
Monocotyledons	Liliaceae	*Lilium*	*Lilium pumilum*	HM053718
Monocotyledons	Liliaceae	*Lilium*	*Lilium pumilum*	HM053719
Monocotyledons	Liliaceae	*Lilium*	*Lilium pumilum*	HM053720
Monocotyledons	Liliaceae	*Lilium*	*Lilium pumilum*	HM053721
Monocotyledons	Liliaceae	*Lilium*	*Lilium pumilum*	HM053722
Monocotyledons	Liliaceae	*Lilium*	*Lilium pumilum*	GQ434916
Monocotyledons	Melanthiaceae	*Trillium*	*Trillium ovatum*	DQ404228
Monocotyledons	Melanthiaceae	*Trillium*	*Trillium ovatum*	AY727187
Monocotyledons	Melanthiaceae	*Paris*	*Paris fargesii*	DQ404251
Monocotyledons	Melanthiaceae	*Paris*	*Paris fargesii*	JN045717
Monocotyledons	Melanthiaceae	*Paris*	*Paris fargesii*	JN045718
Monocotyledons	Melanthiaceae	*Paris*	*Paris fargesii*	JN045719
Monocotyledons	Melanthiaceae	*Paris*	*Paris fargesii*	JN045720
Monocotyledons	Melanthiaceae	*Paris*	*Paris fargesii*	JN045721
Monocotyledons	Melanthiaceae	*Paris*	*Paris fargesii*	JN045722
Monocotyledons	Melanthiaceae	*Paris*	*Paris thibetica*	DQ404250
Monocotyledons	Melanthiaceae	*Paris*	*Paris thibetica*	JN045763
Monocotyledons	Melanthiaceae	*Paris*	*Paris thibetica*	JN045764
Monocotyledons	Melanthiaceae	*Paris*	*Paris thibetica*	JN045765
Monocotyledons	Melanthiaceae	*Paris*	*Paris thibetica*	JN045766
Monocotyledons	Melanthiaceae	*Paris*	*Paris thibetica*	JN045767
Monocotyledons	Melanthiaceae	*Paris*	*Paris thibetica*	JN045768
Monocotyledons	Melanthiaceae	*Paris*	*Paris thibetica*	JN045769
Monocotyledons	Melanthiaceae	*Paris*	*Paris thibetica*	JN045770
Monocotyledons	Melanthiaceae	*Paris*	*Paris thibetica*	JN045771
Monocotyledons	Melanthiaceae	*Paris*	*Paris incompleta*	DQ404237
Monocotyledons	Melanthiaceae	*Paris*	*Paris incompleta*	JN045723
Monocotyledons	Melanthiaceae	*Paris*	*Paris incompleta*	JN045724
Monocotyledons	Melanthiaceae	*Paris*	*Paris incompleta*	JN045725
Monocotyledons	Liliaceae	*Lilium*	*Lilium philadelphicum*	DQ122704
Monocotyledons	Liliaceae	*Lilium*	*Lilium philadelphicum*	DQ122705
Monocotyledons	Liliaceae	*Lilium*	*Lilium philadelphicum*	DQ122706
Monocotyledons	Liliaceae	*Lilium*	*Lilium philadelphicum*	DQ122707
Monocotyledons	Liliaceae	*Lilium*	*Lilium philadelphicum*	DQ122708
Monocotyledons	Liliaceae	*Lilium*	*Lilium philadelphicum*	DQ122709
Monocotyledons	Liliaceae	*Lilium*	*Lilium philadelphicum*	DQ122710
Monocotyledons	Liliaceae	*Lilium*	*Lilium philadelphicum*	DQ122711
Monocotyledons	Liliaceae	*Lilium*	*Lilium philadelphicum*	DQ122712
Monocotyledons	Liliaceae	*Lilium*	*Lilium philadelphicum*	DQ122713
Monocotyledons	Liliaceae	*Lilium*	*Lilium philadelphicum*	DQ122714
Monocotyledons	Liliaceae	*Lilium*	*Lilium philadelphicum*	DQ122715

Monocotyledons	Liliaceae	*Lilium*	*Lilium philadelphicum*	DQ122716
Monocotyledons	Liliaceae	*Lilium*	*Lilium philadelphicum*	DQ122717
Monocotyledons	Liliaceae	*Lilium*	*Lilium philadelphicum*	DQ122718
Monocotyledons	Liliaceae	*Lilium*	*Lilium philadelphicum*	DQ122719
Monocotyledons	Liliaceae	*Lilium*	*Lilium philadelphicum*	DQ122720
Monocotyledons	Amaryllidaceae	*Allium*	*Allium macrostemon*	GQ434917
Monocotyledons	Amaryllidaceae	*Allium*	*Allium macrostemon*	GQ434918
Monocotyledons	Amaryllidaceae	*Allium*	*Allium macrostemon*	GQ434919
Monocotyledons	Poaceae	*Festuca*	*Festuca idahoensis*	DQ369764
Monocotyledons	Poaceae	*Festuca*	*Festuca idahoensis*	DQ369766
Monocotyledons	Poaceae	*Festuca*	*Festuca idahoensis*	DQ369770
Monocotyledons	Poaceae	*Festuca*	*Festuca idahoensis*	DQ369774
Monocotyledons	Poaceae	*Festuca*	*Festuca idahoensis*	DQ369778
Monocotyledons	Poaceae	*Festuca*	*Festuca valesiaca*	DQ369758
Monocotyledons	Poaceae	*Festuca*	*Festuca valesiaca*	DQ369776
Monocotyledons	Asparagaceae	*Asparagus*	*Asparagus cochinchinensis*	GQ434909
Monocotyledons	Asparagaceae	*Asparagus*	*Asparagus cochinchinensis*	GQ434910
Monocotyledons	Asparagaceae	*Asparagus*	*Asparagus cochinchinensis*	GQ434911
Monocotyledons	Poaceae	*Kengyilia*	*Kengyilia rigidula*	JN045115
Monocotyledons	Poaceae	*Kengyilia*	*Kengyilia rigidula*	JN045116
Monocotyledons	Poaceae	*Kengyilia*	*Kengyilia rigidula*	JN045117
Monocotyledons	Poaceae	*Kengyilia*	*Kengyilia rigidula*	HQ221838
Monocotyledons	Poaceae	*Kengyilia*	*Kengyilia rigidula*	HQ221841
Monocotyledons	Poaceae	*Kengyilia*	*Kengyilia rigidula*	HQ221848
Monocotyledons	Poaceae	*Kengyilia*	*Kengyilia rigidula*	HQ221849
Monocotyledons	Poaceae	*Kengyilia*	*Kengyilia rigidula*	HQ221850
Monocotyledons	Poaceae	*Kengyilia*	*Kengyilia rigidula*	HQ652818
Monocotyledons	Poaceae	*Kengyilia*	*Kengyilia rigidula*	HQ652819
Monocotyledons	Poaceae	*Kengyilia*	*Kengyilia rigidula*	HQ652826
Monocotyledons	Poaceae	*Kengyilia*	*Kengyilia rigidula*	HQ652827
Monocotyledons	Poaceae	*Kengyilia*	*Kengyilia rigidula*	HQ652830
Monocotyledons	Poaceae	*Kengyilia*	*Kengyilia rigidula*	HQ652831
Monocotyledons	Poaceae	*Kengyilia*	*Kengyilia rigidula*	HQ652833
Monocotyledons	Poaceae	*Kengyilia*	*Kengyilia grandiglumis*	JN045092
Monocotyledons	Poaceae	*Kengyilia*	*Kengyilia grandiglumis*	JN045093
Monocotyledons	Poaceae	*Kengyilia*	*Kengyilia grandiglumis*	JN045094
Monocotyledons	Poaceae	*Kengyilia*	*Kengyilia grandiglumis*	HQ652811
Monocotyledons	Poaceae	*Kengyilia*	*Kengyilia grandiglumis*	HQ652829
Monocotyledons	Poaceae	*Kengyilia*	*Kengyilia grandiglumis*	HQ652851
Monocotyledons	Poaceae	*Kengyilia*	*Kengyilia kokonorica*	JN045098
Monocotyledons	Poaceae	*Kengyilia*	*Kengyilia kokonorica*	JN045099
Monocotyledons	Poaceae	*Kengyilia*	*Kengyilia kokonorica*	JN045100
Monocotyledons	Poaceae	*Kengyilia*	*Kengyilia kokonorica*	JN045101
Monocotyledons	Poaceae	*Kengyilia*	*Kengyilia kokonorica*	HQ652832
Monocotyledons	Poaceae	*Kengyilia*	*Kengyilia kokonorica*	HQ652834
Monocotyledons	Poaceae	*Kengyilia*	*Kengyilia kokonorica*	HQ652857
Monocotyledons	Poaceae	*Kengyilia*	*Kengyilia kokonorica*	HQ652858
Monocotyledons	Poaceae	*Kengyilia*	*Kengyilia laxiflora*	JN045102
Monocotyledons	Poaceae	*Kengyilia*	*Kengyilia laxiflora*	JN045103
Monocotyledons	Poaceae	*Kengyilia*	*Kengyilia laxiflora*	JN045104
Monocotyledons	Poaceae	*Kengyilia*	*Kengyilia laxiflora*	HQ652835
Monocotyledons	Poaceae	*Kengyilia*	*Kengyilia laxiflora*	HQ652836
Monocotyledons	Poaceae	*Kengyilia*	*Kengyilia laxiflora*	HQ652859

Monocotyledons	Poaceae	*Kengyilia*	*Kengyilia melanthera*	JN045108
Monocotyledons	Poaceae	*Kengyilia*	*Kengyilia melanthera*	JN045109
Monocotyledons	Poaceae	*Kengyilia*	*Kengyilia melanthera*	JN045110
Monocotyledons	Poaceae	*Kengyilia*	*Kengyilia melanthera*	HQ221839
Monocotyledons	Poaceae	*Kengyilia*	*Kengyilia melanthera*	HQ221840
Monocotyledons	Poaceae	*Kengyilia*	*Kengyilia melanthera*	HQ221843
Monocotyledons	Poaceae	*Kengyilia*	*Kengyilia melanthera*	HQ221844
Monocotyledons	Poaceae	*Kengyilia*	*Kengyilia melanthera*	HQ221846
Monocotyledons	Poaceae	*Kengyilia*	*Kengyilia melanthera*	HQ652801
Monocotyledons	Poaceae	*Kengyilia*	*Kengyilia melanthera*	HQ652810
Monocotyledons	Poaceae	*Kengyilia*	*Kengyilia melanthera*	HQ652864
Monocotyledons	Poaceae	*Kengyilia*	*Kengyilia melanthera*	HQ652865
Monocotyledons	Poaceae	*Kengyilia*	*Kengyilia melanthera*	HQ652866
Monocotyledons	Poaceae	*Kengyilia*	*Kengyilia mutica*	JN045113
Monocotyledons	Poaceae	*Kengyilia*	*Kengyilia mutica*	JN045114
Monocotyledons	Poaceae	*Kengyilia*	*Kengyilia mutica*	HQ652823
Monocotyledons	Poaceae	*Kengyilia*	*Kengyilia mutica*	HQ652825
Monocotyledons	Poaceae	*Kengyilia*	*Kengyilia mutica*	HQ652867
Monocotyledons	Poaceae	*Kengyilia*	*Kengyilia thoroldiana*	JN045121
Monocotyledons	Poaceae	*Kengyilia*	*Kengyilia thoroldiana*	JN045122
Monocotyledons	Poaceae	*Kengyilia*	*Kengyilia thoroldiana*	JN045123
Monocotyledons	Poaceae	*Kengyilia*	*Kengyilia thoroldiana*	HQ652814
Monocotyledons	Poaceae	*Kengyilia*	*Kengyilia thoroldiana*	HQ652815
Monocotyledons	Poaceae	*Kengyilia*	*Kengyilia thoroldiana*	HQ652816
Monocotyledons	Poaceae	*Kengyilia*	*Kengyilia thoroldiana*	HQ652824
Monocotyledons	Poaceae	*Kengyilia*	*Kengyilia batalinii*	JN045085
Monocotyledons	Poaceae	*Kengyilia*	*Kengyilia batalinii*	JN045086
Monocotyledons	Poaceae	*Kengyilia*	*Kengyilia batalinii*	JN045087
Monocotyledons	Poaceae	*Kengyilia*	*Kengyilia batalinii*	HQ652843
Monocotyledons	Poaceae	*Kengyilia*	*Kengyilia batalinii*	HQ652844
Monocotyledons	Poaceae	*Kengyilia*	*Kengyilia batalinii*	HQ652845
Monocotyledons	Poaceae	*Kengyilia*	*Kengyilia batalinii*	HQ652846
Monocotyledons	Musaceae	*Musa*	*Musa basjoo*	FJ871913
Monocotyledons	Musaceae	*Musa*	*Musa basjoo*	FJ871914
Monocotyledons	Musaceae	*Musa*	*Musa basjoo*	JN045497
Monocotyledons	Musaceae	*Musa*	*Musa basjoo*	JN045498
Monocotyledons	Musaceae	*Musa*	*Musa basjoo*	JN045499
Monocotyledons	Amaryllidaceae	*Allium*	*Allium przewalskianum*	GU121988
Monocotyledons	Amaryllidaceae	*Allium*	*Allium przewalskianum*	GU121989
Monocotyledons	Amaryllidaceae	*Allium*	*Allium przewalskianum*	GU121990
Monocotyledons	Amaryllidaceae	*Allium*	*Allium przewalskianum*	GU121991
Monocotyledons	Liliaceae	*Erythronium*	*Erythronium elegans*	EU311877
Monocotyledons	Liliaceae	*Erythronium*	*Erythronium elegans*	EU311878
Monocotyledons	Liliaceae	*Erythronium*	*Erythronium elegans*	EU311879
Monocotyledons	Liliaceae	*Erythronium*	*Erythronium elegans*	EU311880
Monocotyledons	Liliaceae	*Erythronium*	*Erythronium elegans*	EU311881
Monocotyledons	Liliaceae	*Erythronium*	*Erythronium elegans*	EU311882
Monocotyledons	Liliaceae	*Erythronium*	*Erythronium elegans*	EU311883
Monocotyledons	Liliaceae	*Erythronium*	*Erythronium elegans*	EU311884
Monocotyledons	Liliaceae	*Erythronium*	*Erythronium elegans*	EU311885
Monocotyledons	Liliaceae	*Erythronium*	*Erythronium elegans*	EU311886
Monocotyledons	Liliaceae	*Erythronium*	*Erythronium elegans*	EU311887
Monocotyledons	Liliaceae	*Erythronium*	*Erythronium elegans*	EU311888

Monocotyledons	Liliaceae	*Erythronium*	*Erythronium elegans*	EU311889
Monocotyledons	Liliaceae	*Erythronium*	*Erythronium elegans*	EU311890
Monocotyledons	Liliaceae	*Erythronium*	*Erythronium elegans*	EU311891
Monocotyledons	Liliaceae	*Erythronium*	*Erythronium elegans*	EU311892
Monocotyledons	Liliaceae	*Erythronium*	*Erythronium klamathense*	EU311852
Monocotyledons	Liliaceae	*Erythronium*	*Erythronium klamathense*	EU311853
Monocotyledons	Liliaceae	*Erythronium*	*Erythronium montanum*	EU311854
Monocotyledons	Liliaceae	*Erythronium*	*Erythronium montanum*	EU311855
Monocotyledons	Liliaceae	*Erythronium*	*Erythronium montanum*	EU311856
Monocotyledons	Liliaceae	*Erythronium*	*Erythronium montanum*	EU311857
Monocotyledons	Liliaceae	*Erythronium*	*Erythronium montanum*	EU311858
Monocotyledons	Liliaceae	*Erythronium*	*Erythronium montanum*	EU311859
Monocotyledons	Liliaceae	*Erythronium*	*Erythronium montanum*	EU311860
Monocotyledons	Liliaceae	*Erythronium*	*Erythronium montanum*	EU311861
Monocotyledons	Liliaceae	*Erythronium*	*Erythronium oregonum*	EU311862
Monocotyledons	Liliaceae	*Erythronium*	*Erythronium oregonum*	EU311863
Monocotyledons	Liliaceae	*Erythronium*	*Erythronium oregonum*	EU311864
Monocotyledons	Liliaceae	*Erythronium*	*Erythronium oregonum*	EU311865
Monocotyledons	Liliaceae	*Erythronium*	*Erythronium oregonum*	EU311866
Monocotyledons	Liliaceae	*Erythronium*	*Erythronium oregonum*	EU311867
Monocotyledons	Liliaceae	*Erythronium*	*Erythronium quinaultense*	EU311893
Monocotyledons	Liliaceae	*Erythronium*	*Erythronium quinaultense*	EU311894
Monocotyledons	Liliaceae	*Erythronium*	*Erythronium quinaultense*	EU311895
Monocotyledons	Liliaceae	*Erythronium*	*Erythronium quinaultense*	EU311896
Monocotyledons	Liliaceae	*Erythronium*	*Erythronium quinaultense*	EU311897
Monocotyledons	Liliaceae	*Erythronium*	*Erythronium quinaultense*	EU311898
Monocotyledons	Liliaceae	*Erythronium*	*Erythronium quinaultense*	EU311899
Monocotyledons	Liliaceae	*Erythronium*	*Erythronium revolutum*	EU311868
Monocotyledons	Liliaceae	*Erythronium*	*Erythronium revolutum*	EU311869
Monocotyledons	Liliaceae	*Erythronium*	*Erythronium revolutum*	EU311870
Monocotyledons	Liliaceae	*Erythronium*	*Erythronium revolutum*	EU311871
Monocotyledons	Liliaceae	*Erythronium*	*Erythronium revolutum*	EU311872
Monocotyledons	Liliaceae	*Erythronium*	*Erythronium revolutum*	EU311873
Monocotyledons	Liliaceae	*Erythronium*	*Erythronium revolutum*	EU311874
Monocotyledons	Liliaceae	*Erythronium*	*Erythronium revolutum*	EU311875
Monocotyledons	Liliaceae	*Erythronium*	*Erythronium revolutum*	EU311876
Monocotyledons	Colchicaceae	*Androcymbium*	*Androcymbium austrocapense*	DQ088279
Monocotyledons	Colchicaceae	*Androcymbium*	*Androcymbium austrocapense*	DQ088280
Monocotyledons	Melanthiaceae	*Paris*	*Paris cronquistii*	JN045701
Monocotyledons	Melanthiaceae	*Paris*	*Paris cronquistii*	JN045702
Monocotyledons	Melanthiaceae	*Paris*	*Paris cronquistii*	JN045703
Monocotyledons	Melanthiaceae	*Paris*	*Paris daliensis*	DQ404260
Monocotyledons	Melanthiaceae	*Paris*	*Paris daliensis*	JN045704
Monocotyledons	Melanthiaceae	*Paris*	*Paris daliensis*	JN045705
Monocotyledons	Melanthiaceae	*Paris*	*Paris luquanensis*	GU178892
Monocotyledons	Melanthiaceae	*Paris*	*Paris luquanensis*	JN045726
Monocotyledons	Melanthiaceae	*Paris*	*Paris luquanensis*	JN045727
Monocotyledons	Melanthiaceae	*Paris*	*Paris luquanensis*	JN045728
Monocotyledons	Melanthiaceae	*Paris*	*Paris marmorata*	DQ404256
Monocotyledons	Melanthiaceae	*Paris*	*Paris marmorata*	GU178890
Monocotyledons	Melanthiaceae	*Paris*	*Paris marmorata*	JN045735
Monocotyledons	Melanthiaceae	*Paris*	*Paris marmorata*	JN045736
Monocotyledons	Melanthiaceae	*Paris*	*Paris marmorata*	JN045737

Monocotyledons	Melanthiaceae	*Paris*	*Paris marmorata*	JN045738
Monocotyledons	Melanthiaceae	*Paris*	*Paris marmorata*	JN045739
Monocotyledons	Musaceae	*Musa*	*Musa textilis*	FJ871897
Monocotyledons	Melanthiaceae	*Paris*	*Paris quadrifolia*	DQ404238
Monocotyledons	Melanthiaceae	*Paris*	*Paris quadrifolia*	JN045757
Monocotyledons	Melanthiaceae	*Paris*	*Paris quadrifolia*	JN045758
Monocotyledons	Melanthiaceae	*Paris*	*Paris quadrifolia*	JN045759
Monocotyledons	Asparagaceae	*Asparagus*	*Asparagus densiflorus*	HM990125
Monocotyledons	Asparagaceae	*Asparagus*	*Asparagus densiflorus*	HM990128
Monocotyledons	Asparagaceae	*Asparagus*	*Asparagus densiflorus*	HM990130
Monocotyledons	Asparagaceae	*Asparagus*	*Asparagus densiflorus*	HM990141
Monocotyledons	Colchicaceae	*Androcymbium*	*Androcymbium gramineum*	DQ088292
Monocotyledons	Colchicaceae	*Androcymbium*	*Androcymbium gramineum*	DQ088293
Monocotyledons	Colchicaceae	*Androcymbium*	*Androcymbium wyssianum*	DQ088316
Monocotyledons	Colchicaceae	*Androcymbium*	*Androcymbium wyssianum*	DQ088317
Monocotyledons	Colchicaceae	*Androcymbium*	*Androcymbium huntleyi*	DQ088297
Monocotyledons	Colchicaceae	*Androcymbium*	*Androcymbium irroratum*	DQ088299
Monocotyledons	Bromeliaceae	*Vriesea*	*Vriesea carinata*	JN204671
Monocotyledons	Bromeliaceae	*Vriesea*	*Vriesea carinata*	JN204672
Monocotyledons	Dioscoreaceae	*Dioscorea*	*Dioscorea zingiberensis*	JQ260269
Monocotyledons	Dioscoreaceae	*Dioscorea*	*Dioscorea zingiberensis*	JQ260270
Monocotyledons	Dioscoreaceae	*Dioscorea*	*Dioscorea zingiberensis*	JQ260275
Monocotyledons	Dioscoreaceae	*Dioscorea*	*Dioscorea zingiberensis*	DQ131105
Monocotyledons	Dioscoreaceae	*Dioscorea*	*Dioscorea zingiberensis*	DQ191747
Monocotyledons	Dioscoreaceae	*Dioscorea*	*Dioscorea zingiberensis*	DQ191749
Monocotyledons	Bromeliaceae	*Nidularium*	*Nidularium procerum*	JN204655
Monocotyledons	Bromeliaceae	*Nidularium*	*Nidularium procerum*	JN204656
Monocotyledons	Poaceae	*Festuca*	*Festuca roemeri*	DQ369768
Monocotyledons	Poaceae	*Festuca*	*Festuca roemeri*	DQ369772
Monocotyledons	Poaceae	*Festuca*	*Festuca roemeri*	DQ369780
Monocotyledons	Poaceae	*Festuca*	*Festuca roemeri*	DQ369782
Monocotyledons	Poaceae	*Festuca*	*Festuca roemeri*	DQ369784
Monocotyledons	Poaceae	*Festuca*	*Festuca roemeri*	DQ369786
Monocotyledons	Poaceae	*Festuca*	*Festuca roemeri*	DQ369788
Monocotyledons	Poaceae	*Festuca*	*Festuca roemeri*	DQ369790
Monocotyledons	Melanthiaceae	*Paris*	*Paris delavayi*	GU178891
Monocotyledons	Melanthiaceae	*Paris*	*Paris delavayi*	JN045706
Monocotyledons	Melanthiaceae	*Paris*	*Paris delavayi*	JN045707
Monocotyledons	Melanthiaceae	*Paris*	*Paris delavayi*	JN045708
Monocotyledons	Melanthiaceae	*Paris*	*Paris delavayi*	JN045709
Monocotyledons	Melanthiaceae	*Paris*	*Paris delavayi*	JN045710
Monocotyledons	Melanthiaceae	*Paris*	*Paris delavayi*	JN045711
Monocotyledons	Melanthiaceae	*Paris*	*Paris dulongensis*	DQ404241
Monocotyledons	Melanthiaceae	*Paris*	*Paris dulongensis*	JN045712
Monocotyledons	Melanthiaceae	*Paris*	*Paris dulongensis*	JN045713
Monocotyledons	Melanthiaceae	*Paris*	*Paris dunniana*	DQ404259
Monocotyledons	Melanthiaceae	*Paris*	*Paris dunniana*	JN045714
Monocotyledons	Melanthiaceae	*Paris*	*Paris dunniana*	JN045715
Monocotyledons	Melanthiaceae	*Paris*	*Paris dunniana*	JN045716
Monocotyledons	Melanthiaceae	*Paris*	*Paris mairei*	DQ404247
Monocotyledons	Melanthiaceae	*Paris*	*Paris mairei*	JN045729
Monocotyledons	Melanthiaceae	*Paris*	*Paris mairei*	JN045730
Monocotyledons	Melanthiaceae	*Paris*	*Paris mairei*	JN045731

Monocotyledons	Melanthiaceae	*Paris*	*Paris mairei*	JN045732
Monocotyledons	Melanthiaceae	*Paris*	*Paris mairei*	JN045733
Monocotyledons	Melanthiaceae	*Paris*	*Paris mairei*	JN045734
Monocotyledons	Melanthiaceae	*Paris*	*Paris rugosa*	DQ404245
Monocotyledons	Melanthiaceae	*Paris*	*Paris rugosa*	JN045760
Monocotyledons	Melanthiaceae	*Paris*	*Paris rugosa*	JN045761
Monocotyledons	Melanthiaceae	*Paris*	*Paris rugosa*	JN045762
Monocotyledons	Melanthiaceae	*Paris*	*Paris vaniotii*	DQ404243
Monocotyledons	Melanthiaceae	*Paris*	*Paris vaniotii*	JN045772
Monocotyledons	Melanthiaceae	*Paris*	*Paris vaniotii*	JN045773
Monocotyledons	Melanthiaceae	*Paris*	*Paris vaniotii*	JN045774
Monocotyledons	Melanthiaceae	*Paris*	*Paris vietnamensis*	DQ404246
Monocotyledons	Melanthiaceae	*Paris*	*Paris vietnamensis*	GU178893
Monocotyledons	Melanthiaceae	*Paris*	*Paris vietnamensis*	JN045775
Monocotyledons	Melanthiaceae	*Paris*	*Paris vietnamensis*	JN045776
Monocotyledons	Melanthiaceae	*Paris*	*Paris vietnamensis*	JN045777
Monocotyledons	Melanthiaceae	*Paris*	*Paris vietnamensis*	JN045778
Monocotyledons	Melanthiaceae	*Paris*	*Paris vietnamensis*	JN045779
Monocotyledons	Melanthiaceae	*Paris*	*Paris vietnamensis*	JN045780
Monocotyledons	Poaceae	*Kengyilia*	*Kengyilia gobicola*	JN045090
Monocotyledons	Poaceae	*Kengyilia*	*Kengyilia gobicola*	JN045091
Monocotyledons	Poaceae	*Kengyilia*	*Kengyilia gobicola*	HQ652849
Monocotyledons	Poaceae	*Kengyilia*	*Kengyilia gobicola*	HQ652850
Monocotyledons	Poaceae	*Kengyilia*	*Kengyilia hirsuta*	JN045095
Monocotyledons	Poaceae	*Kengyilia*	*Kengyilia hirsuta*	JN045096
Monocotyledons	Poaceae	*Kengyilia*	*Kengyilia hirsuta*	JN045097
Monocotyledons	Poaceae	*Kengyilia*	*Kengyilia hirsuta*	HQ652852
Monocotyledons	Poaceae	*Kengyilia*	*Kengyilia hirsuta*	HQ652853
Monocotyledons	Poaceae	*Kengyilia*	*Kengyilia hirsuta*	HQ652854
Monocotyledons	Poaceae	*Kengyilia*	*Kengyilia hirsuta*	HQ652855
Monocotyledons	Poaceae	*Kengyilia*	*Kengyilia longiglumis*	JN045105
Monocotyledons	Poaceae	*Kengyilia*	*Kengyilia longiglumis*	JN045106
Monocotyledons	Poaceae	*Kengyilia*	*Kengyilia longiglumis*	JN045107
Monocotyledons	Poaceae	*Kengyilia*	*Kengyilia longiglumis*	HQ652791
Monocotyledons	Poaceae	*Kengyilia*	*Kengyilia longiglumis*	HQ652860
Monocotyledons	Poaceae	*Kengyilia*	*Kengyilia longiglumis*	HQ652861
Monocotyledons	Poaceae	*Kengyilia*	*Kengyilia longiglumis*	HQ652862
Monocotyledons	Poaceae	*Kengyilia*	*Kengyilia longiglumis*	HQ652863
Monocotyledons	Poaceae	*Kengyilia*	*Kengyilia stenachyra*	JN045118
Monocotyledons	Poaceae	*Kengyilia*	*Kengyilia stenachyra*	JN045119
Monocotyledons	Poaceae	*Kengyilia*	*Kengyilia stenachyra*	JN045120
Monocotyledons	Poaceae	*Kengyilia*	*Kengyilia stenachyra*	HQ652812
Monocotyledons	Poaceae	*Kengyilia*	*Kengyilia stenachyra*	HQ652820
Monocotyledons	Poaceae	*Kengyilia*	*Kengyilia stenachyra*	HQ652828
Monocotyledons	Poaceae	*Kengyilia*	*Kengyilia tahelacana*	HQ652813
Monocotyledons	Poaceae	*Kengyilia*	*Kengyilia tahelacana*	HQ652822
Monocotyledons	Commelinaceae	*Commelina*	*Commelina imberbis*	GQ248271
Monocotyledons	Commelinaceae	*Commelina*	*Commelina imberbis*	EF590681
Monocotyledons	Poaceae	*Amelichloa*	*Amelichloa brachychaeta*	EU204669
Monocotyledons	Poaceae	*Amelichloa*	*Amelichloa brachychaeta*	EU204670
Monocotyledons	Poaceae	*Amelichloa*	*Amelichloa brachychaeta*	EU204671
Monocotyledons	Poaceae	*Amelichloa*	*Amelichloa brachychaeta*	EU204672
Monocotyledons	Poaceae	*Amelichloa*	*Amelichloa brachychaeta*	EU204673

Monocotyledons	Poaceae	*Amelichloa*	*Amelichloa brachychaeta*	EU204674
Monocotyledons	Poaceae	*Amelichloa*	*Amelichloa caudata*	EU489241
Monocotyledons	Poaceae	*Amelichloa*	*Amelichloa caudata*	EU204675
Monocotyledons	Poaceae	*Amelichloa*	*Amelichloa caudata*	EU204676
Monocotyledons	Poaceae	*Amelichloa*	*Amelichloa caudata*	EU204677
Monocotyledons	Poaceae	*Amelichloa*	*Amelichloa caudata*	EU204678
Monocotyledons	Poaceae	*Amelichloa*	*Amelichloa caudata*	EU204679
Monocotyledons	Poaceae	*Amelichloa*	*Amelichloa clandestina*	EU489242
Monocotyledons	Poaceae	*Amelichloa*	*Amelichloa clandestina*	EU204680
Monocotyledons	Poaceae	*Amelichloa*	*Amelichloa clandestina*	EU204681
Monocotyledons	Poaceae	*Amelichloa*	*Amelichloa clandestina*	EU204682
Monocotyledons	Poaceae	*Amelichloa*	*Amelichloa clandestina*	EU204683
Monocotyledons	Poaceae	*Piptatherum*	*Piptatherum holciforme*	EU204755
Monocotyledons	Poaceae	*Piptatherum*	*Piptatherum holciforme*	EU204756
Monocotyledons	Musaceae	*Musa*	*Musa itinerans*	FJ871877
Monocotyledons	Musaceae	*Musa*	*Musa itinerans*	FJ871878
Monocotyledons	Musaceae	*Musa*	*Musa itinerans*	FJ871879
Monocotyledons	Musaceae	*Musa*	*Musa itinerans*	FJ871880
Monocotyledons	Musaceae	*Musa*	*Musa itinerans*	FJ871881
Monocotyledons	Musaceae	*Musa*	*Musa itinerans*	FJ871882
Monocotyledons	Musaceae	*Musa*	*Musa itinerans*	FJ871884
Monocotyledons	Musaceae	*Musa*	*Musa maclayi*	FJ871901
Monocotyledons	Musaceae	*Musa*	*Musa nagensium*	FJ871919
Monocotyledons	Musaceae	*Musa*	*Musa nagensium*	FJ871920
Monocotyledons	Musaceae	*Musa*	*Musa rubinea*	FJ871889
Monocotyledons	Musaceae	*Musa*	*Musa rubinea*	FJ871890
Monocotyledons	Musaceae	*Musa*	*Musa rubinea*	FJ871891
Monocotyledons	Musaceae	*Musa*	*Musa rubinea*	FJ871892
Monocotyledons	Musaceae	*Musa*	*Musa yunnanensis*	FJ871885
Monocotyledons	Musaceae	*Musa*	*Musa yunnanensis*	FJ871886
Monocotyledons	Musaceae	*Musa*	*Musa yunnanensis*	FJ871887
Monocotyledons	Musaceae	*Musa*	*Musa yunnanensis*	FJ871888
Monocotyledons	Orchidaceae	*Grandiphyllum*	*Grandiphyllum auriculum*	FJ564447
Monocotyledons	Orchidaceae	*Grandiphyllum*	*Grandiphyllum auriculum*	FJ564676
Monocotyledons	Orchidaceae	*Grandiphyllum*	*Grandiphyllum hians*	FJ564025
Monocotyledons	Orchidaceae	*Grandiphyllum*	*Grandiphyllum hians*	FJ564235
Monocotyledons	Musaceae	*Musa*	*Musa paracoccinea*	FJ871904
Monocotyledons	Musaceae	*Musa*	*Musa viridis*	FJ871893
Monocotyledons	Musaceae	*Musa*	*Musa viridis*	FJ871894
Monocotyledons	Musaceae	*Musa*	*Musa viridis*	FJ871895
Monocotyledons	Asparagaceae	*Asparagus*	*Asparagus aethiopicus*	GU135328
Monocotyledons	Asparagaceae	*Asparagus*	*Asparagus aethiopicus*	GU135434
Monocotyledons	Asparagaceae	*Asparagus*	*Asparagus aethiopicus*	HM990140
Monocotyledons	Smilacaceae	*Smilax*	*Smilax glabra*	GU372812
Monocotyledons	Smilacaceae	*Smilax*	*Smilax glabra*	GU372815
Monocotyledons	Smilacaceae	*Smilax*	*Smilax glabra*	JN047204
Monocotyledons	Smilacaceae	*Smilax*	*Smilax glabra*	JN047205
Monocotyledons	Asparagaceae	*Asparagus*	*Asparagus trichophyllus*	GQ434913
Monocotyledons	Asparagaceae	*Asparagus*	*Asparagus trichophyllus*	GQ434914
Monocotyledons	Arecaceae	*Caryota*	*Caryota maxima*	HQ415573
Monocotyledons	Arecaceae	*Caryota*	*Caryota maxima*	JF345044
Monocotyledons	Arecaceae	*Caryota*	*Caryota maxima*	JF345045
Monocotyledons	Arecaceae	*Caryota*	*Caryota maxima*	JF345050

Monocotyledons	Arecaceae	*Caryota*	*Caryota maxima*	JF345051
Monocotyledons	Arecaceae	*Caryota*	*Caryota maxima*	JF345052
Monocotyledons	Arecaceae	*Caryota*	*Caryota maxima*	JF345053
Monocotyledons	Arecaceae	*Caryota*	*Caryota maxima*	JF345065
Monocotyledons	Poaceae	*Kengyilia*	*Kengyilia geminata*	JN045088
Monocotyledons	Poaceae	*Kengyilia*	*Kengyilia geminata*	JN045089
Monocotyledons	Poaceae	*Kengyilia*	*Kengyilia geminata*	HQ652847
Monocotyledons	Poaceae	*Kengyilia*	*Kengyilia geminata*	HQ652848
Monocotyledons	Smilacaceae	*Smilax*	*Smilax lanceifolia*	JN047206
Monocotyledons	Smilacaceae	*Smilax*	*Smilax lanceifolia*	JN047207
Monocotyledons	Commelinaceae	*Commelina*	*Commelina paludosa*	JN044311
Monocotyledons	Commelinaceae	*Commelina*	*Commelina paludosa*	JN044312
Monocotyledons	Melanthiaceae	*Paris*	*Paris caobangensis*	JN045699
Monocotyledons	Melanthiaceae	*Paris*	*Paris caobangensis*	JN045700
Monocotyledons	Arecaceae	*Caryota*	*Caryota obtusa*	JF345046
Monocotyledons	Arecaceae	*Caryota*	*Caryota obtusa*	JF345047
Monocotyledons	Arecaceae	*Caryota*	*Caryota obtusa*	JF345061
Monocotyledons	Arecaceae	*Caryota*	*Caryota obtusa*	JF345062
Monocotyledons	Arecaceae	*Caryota*	*Caryota obtusa*	JF345063
Monocotyledons	Arecaceae	*Caryota*	*Caryota obtusa*	JF345064
Monocotyledons	Arecaceae	*Caryota*	*Caryota kiriwongensis*	JF345048
Monocotyledons	Arecaceae	*Caryota*	*Caryota kiriwongensis*	JF345049
Monocotyledons	Arecaceae	*Caryota*	*Caryota monostachya*	JF345056
Monocotyledons	Arecaceae	*Caryota*	*Caryota monostachya*	JF345057
Monocotyledons	Arecaceae	*Caryota*	*Caryota monostachya*	JF345058
Monocotyledons	Arecaceae	*Caryota*	*Caryota no*	JF345059
Monocotyledons	Arecaceae	*Caryota*	*Caryota no*	JF345060
Monocotyledons	Bromeliaceae	*Hohenbergia*	*Hohenbergia ramageana*	JN204639
Monocotyledons	Bromeliaceae	*Hohenbergia*	*Hohenbergia ramageana*	JN204640
Monocotyledons	Bromeliaceae	*Hohenbergia*	*Hohenbergia ridleyi*	JN204641
Monocotyledons	Bromeliaceae	*Hohenbergia*	*Hohenbergia ridleyi*	JN204642
Monocotyledons	Bromeliaceae	*Nidularium*	*Nidularium altimontanum*	JN204646
Monocotyledons	Bromeliaceae	*Nidularium*	*Nidularium altimontanum*	JN204647
Monocotyledons	Bromeliaceae	*Nidularium*	*Nidularium angustibracteatum*	JN204648
Monocotyledons	Bromeliaceae	*Nidularium*	*Nidularium angustibracteatum*	JN204649
Monocotyledons	Bromeliaceae	*Nidularium*	*Nidularium innocentii*	JN204650
Monocotyledons	Bromeliaceae	*Nidularium*	*Nidularium innocentii*	JN204651
Monocotyledons	Bromeliaceae	*Nidularium*	*Nidularium innocentii*	JN204652
Monocotyledons	Bromeliaceae	*Nidularium*	*Nidularium krisgreeniae*	JN204653
Monocotyledons	Bromeliaceae	*Nidularium*	*Nidularium krisgreeniae*	JN204654
Monocotyledons	Bromeliaceae	*Pitcairnia*	*Pitcairnia encholirioides*	JN204657
Monocotyledons	Bromeliaceae	*Pitcairnia*	*Pitcairnia encholirioides*	JN204658
Monocotyledons	Bromeliaceae	*Pitcairnia*	*Pitcairnia flammea*	JN204659
Monocotyledons	Bromeliaceae	*Pitcairnia*	*Pitcairnia flammea*	JN204660
Monocotyledons	Bromeliaceae	*Vriesea*	*Vriesea cacuminis*	JN204669
Monocotyledons	Bromeliaceae	*Vriesea*	*Vriesea cacuminis*	JN204670
Monocotyledons	Bromeliaceae	*Vriesea*	*Vriesea erythrodactylon*	JN204675
Monocotyledons	Bromeliaceae	*Vriesea*	*Vriesea erythrodactylon*	JN204676
Monocotyledons	Bromeliaceae	*Vriesea*	*Vriesea erythrodactylon*	JN204677
Monocotyledons	Bromeliaceae	*Vriesea*	*Vriesea friburgensis*	JN204678
Monocotyledons	Bromeliaceae	*Vriesea*	*Vriesea friburgensis*	JN204679
Monocotyledons	Bromeliaceae	*Vriesea*	*Vriesea friburgensis*	JN204680
Monocotyledons	Bromeliaceae	*Vriesea*	*Vriesea friburgensis*	JN204681

Monocotyledons	Bromeliaceae	*Vriesea*	*Vriesea heterostachys*	JN204683
Monocotyledons	Bromeliaceae	*Vriesea*	*Vriesea heterostachys*	JN204684
Monocotyledons	Bromeliaceae	*Vriesea*	*Vriesea incurvata*	JN204685
Monocotyledons	Bromeliaceae	*Vriesea*	*Vriesea incurvata*	JN204686
Monocotyledons	Bromeliaceae	*Vriesea*	*Vriesea longicaulis*	JN204687
Monocotyledons	Bromeliaceae	*Vriesea*	*Vriesea longicaulis*	JN204688
Monocotyledons	Bromeliaceae	*Vriesea*	*Vriesea platynema*	JN204673
Monocotyledons	Bromeliaceae	*Vriesea*	*Vriesea platynema*	JN204689
Monocotyledons	Bromeliaceae	*Vriesea*	*Vriesea platynema*	JN204690
Monocotyledons	Bromeliaceae	*Vriesea*	*Vriesea procera*	JN204691
Monocotyledons	Bromeliaceae	*Vriesea*	*Vriesea procera*	JN204692
Monocotyledons	Bromeliaceae	*Vriesea*	*Vriesea procera*	JN204693
Monocotyledons	Hypoxidaceae	*Pauridia*	*Pauridia longituba*	FM206348
Monocotyledons	Hypoxidaceae	*Pauridia*	*Pauridia longituba*	FM206349
Monocotyledons	Hypoxidaceae	*Pauridia*	*Pauridia longituba*	FM206350
Monocotyledons	Hypoxidaceae	*Pauridia*	*Pauridia longituba*	FM206351
Monocotyledons	Hypoxidaceae	*Pauridia*	*Pauridia longituba*	FM206352
Monocotyledons	Hypoxidaceae	*Pauridia*	*Pauridia longituba*	FM206353
Monocotyledons	Hypoxidaceae	*Pauridia*	*Pauridia longituba*	FM206354
Monocotyledons	Hypoxidaceae	*Pauridia*	*Pauridia longituba*	FM206355
Monocotyledons	Hypoxidaceae	*Pauridia*	*Pauridia longituba*	FM206356
Monocotyledons	Hypoxidaceae	*Pauridia*	*Pauridia longituba*	FM206357
Monocotyledons	Hypoxidaceae	*Pauridia*	*Pauridia longituba*	FM206358
Monocotyledons	Hypoxidaceae	*Pauridia*	*Pauridia longituba*	FM206359
Monocotyledons	Hypoxidaceae	*Pauridia*	*Pauridia longituba*	FM206360
Monocotyledons	Hypoxidaceae	*Pauridia*	*Pauridia longituba*	FM206361
Monocotyledons	Hypoxidaceae	*Pauridia*	*Pauridia longituba*	FM206362
Monocotyledons	Hypoxidaceae	*Pauridia*	*Pauridia longituba*	FM206363
Monocotyledons	Hypoxidaceae	*Pauridia*	*Pauridia longituba*	FM206364
Monocotyledons	Hypoxidaceae	*Pauridia*	*Pauridia longituba*	FM206365
Monocotyledons	Hypoxidaceae	*Pauridia*	*Pauridia longituba*	FM206366
Monocotyledons	Hypoxidaceae	*Pauridia*	*Pauridia longituba*	FM206367
Monocotyledons	Hypoxidaceae	*Pauridia*	*Pauridia longituba*	FM206368
Monocotyledons	Hypoxidaceae	*Pauridia*	*Pauridia longituba*	FM206369
Monocotyledons	Hypoxidaceae	*Pauridia*	*Pauridia longituba*	FM206370
Monocotyledons	Hypoxidaceae	*Pauridia*	*Pauridia longituba*	FM206371
Monocotyledons	Hypoxidaceae	*Pauridia*	*Pauridia longituba*	FM206372
Monocotyledons	Hypoxidaceae	*Pauridia*	*Pauridia longituba*	FM206373
Monocotyledons	Hypoxidaceae	*Pauridia*	*Pauridia longituba*	FM206374
Monocotyledons	Hypoxidaceae	*Pauridia*	*Pauridia longituba*	FM206375
Monocotyledons	Hypoxidaceae	*Pauridia*	*Pauridia longituba*	FM206376
Monocotyledons	Hypoxidaceae	*Pauridia*	*Pauridia longituba*	FM206377
Monocotyledons	Hypoxidaceae	*Pauridia*	*Pauridia longituba*	FM206378
Monocotyledons	Hypoxidaceae	*Pauridia*	*Pauridia longituba*	FM206379
Monocotyledons	Hypoxidaceae	*Pauridia*	*Pauridia longituba*	FM206380
Monocotyledons	Hyacinthaceae	*Hyacinthoides*	*Hyacinthoides aristidis*	FJ423302
Monocotyledons	Hyacinthaceae	*Hyacinthoides*	*Hyacinthoides aristidis*	FJ423303
Monocotyledons	Hyacinthaceae	*Hyacinthoides*	*Hyacinthoides hispanica*	FJ423321
Monocotyledons	Hyacinthaceae	*Hyacinthoides*	*Hyacinthoides hispanica*	FJ423322
Monocotyledons	Hyacinthaceae	*Hyacinthoides*	*Hyacinthoides hispanica*	FJ423323
Monocotyledons	Hyacinthaceae	*Hyacinthoides*	*Hyacinthoides hispanica*	FJ423324
Monocotyledons	Hyacinthaceae	*Hyacinthoides*	*Hyacinthoides hispanica*	FJ423325
Monocotyledons	Hyacinthaceae	*Hyacinthoides*	*Hyacinthoides hispanica*	FJ423326

Monocotyledons	Hyacinthaceae	*Hyacinthoides*	*Hyacinthoides hispanica*	FJ423327
Monocotyledons	Hyacinthaceae	*Hyacinthoides*	*Hyacinthoides hispanica*	FJ423328
Monocotyledons	Hyacinthaceae	*Hyacinthoides*	*Hyacinthoides hispanica*	FJ423329
Monocotyledons	Hyacinthaceae	*Hyacinthoides*	*Hyacinthoides hispanica*	FJ423330
Monocotyledons	Hyacinthaceae	*Hyacinthoides*	*Hyacinthoides hispanica*	FJ423331
Monocotyledons	Hyacinthaceae	*Hyacinthoides*	*Hyacinthoides hispanica*	FJ423332
Monocotyledons	Hyacinthaceae	*Hyacinthoides*	*Hyacinthoides hispanica*	FJ423333
Monocotyledons	Hyacinthaceae	*Hyacinthoides*	*Hyacinthoides hispanica*	FJ423334
Monocotyledons	Hyacinthaceae	*Hyacinthoides*	*Hyacinthoides hispanica*	FJ423335
Monocotyledons	Hyacinthaceae	*Hyacinthoides*	*Hyacinthoides hispanica*	FJ423336
Monocotyledons	Hyacinthaceae	*Hyacinthoides*	*Hyacinthoides hispanica*	FJ423337
Monocotyledons	Hyacinthaceae	*Hyacinthoides*	*Hyacinthoides hispanica*	FJ423338
Monocotyledons	Hyacinthaceae	*Hyacinthoides*	*Hyacinthoides hispanica*	FJ423340
Monocotyledons	Hyacinthaceae	*Hyacinthoides*	*Hyacinthoides hispanica*	FJ423341
Monocotyledons	Hyacinthaceae	*Hyacinthoides*	*Hyacinthoides hispanica*	FJ423348
Monocotyledons	Hyacinthaceae	*Hyacinthoides*	*Hyacinthoides hispanica*	FJ423349
Monocotyledons	Hyacinthaceae	*Hyacinthoides*	*Hyacinthoides hispanica*	FJ423350
Monocotyledons	Hyacinthaceae	*Hyacinthoides*	*Hyacinthoides hispanica*	FJ423351
Monocotyledons	Hyacinthaceae	*Hyacinthoides*	*Hyacinthoides hispanica*	FJ423352
Monocotyledons	Hyacinthaceae	*Hyacinthoides*	*Hyacinthoides italica*	FJ423289
Monocotyledons	Hyacinthaceae	*Hyacinthoides*	*Hyacinthoides italica*	FJ423290
Monocotyledons	Hyacinthaceae	*Hyacinthoides*	*Hyacinthoides italica*	FJ423291
Monocotyledons	Hyacinthaceae	*Hyacinthoides*	*Hyacinthoides italica*	FJ423292
Monocotyledons	Hyacinthaceae	*Hyacinthoides*	*Hyacinthoides italica*	FJ423293
Monocotyledons	Hyacinthaceae	*Hyacinthoides*	*Hyacinthoides lingulata*	FJ423294
Monocotyledons	Hyacinthaceae	*Hyacinthoides*	*Hyacinthoides lingulata*	FJ423295
Monocotyledons	Hyacinthaceae	*Hyacinthoides*	*Hyacinthoides lingulata*	FJ423296
Monocotyledons	Hyacinthaceae	*Hyacinthoides*	*Hyacinthoides lingulata*	FJ423297
Monocotyledons	Hyacinthaceae	*Hyacinthoides*	*Hyacinthoides lingulata*	FJ423298
Monocotyledons	Hyacinthaceae	*Hyacinthoides*	*Hyacinthoides non-scripta*	FJ423316
Monocotyledons	Hyacinthaceae	*Hyacinthoides*	*Hyacinthoides non-scripta*	FJ423317
Monocotyledons	Hyacinthaceae	*Hyacinthoides*	*Hyacinthoides non-scripta*	FJ423318
Monocotyledons	Hyacinthaceae	*Hyacinthoides*	*Hyacinthoides non-scripta*	FJ423319
Monocotyledons	Hyacinthaceae	*Hyacinthoides*	*Hyacinthoides non-scripta*	FJ423320
Monocotyledons	Hyacinthaceae	*Hyacinthoides*	*Hyacinthoides reverchonii*	FJ423304
Monocotyledons	Hyacinthaceae	*Hyacinthoides*	*Hyacinthoides reverchonii*	FJ423305
Monocotyledons	Hyacinthaceae	*Hyacinthoides*	*Hyacinthoides reverchonii*	FJ423306
Monocotyledons	Hyacinthaceae	*Hyacinthoides*	*Hyacinthoides reverchonii*	FJ423307
Monocotyledons	Hyacinthaceae	*Hyacinthoides*	*Hyacinthoides reverchonii*	FJ423308
Monocotyledons	Zingiberaceae	*Alpinia*	*Alpinia galanga*	GQ435039
Monocotyledons	Zingiberaceae	*Alpinia*	*Alpinia galanga*	GQ435040
Monocotyledons	Zingiberaceae	*Alpinia*	*Alpinia galanga*	GQ435041
Monocotyledons	Zingiberaceae	*Alpinia*	*Alpinia galanga*	EU552522
Monocotyledons	Zingiberaceae	*Alpinia*	*Alpinia galanga*	EU552528
Monocotyledons	Zingiberaceae	*Alpinia*	*Alpinia galanga*	GU180424
Monocotyledons	Zingiberaceae	*Alpinia*	*Alpinia galanga*	JN043838
Monocotyledons	Zingiberaceae	*Alpinia*	*Alpinia galanga*	JN043839
Monocotyledons	Zingiberaceae	*Alpinia*	*Alpinia galanga*	JN043840
Monocotyledons	Zingiberaceae	*Alpinia*	*Alpinia galanga*	JN043841
Monocotyledons	Zingiberaceae	*Alpinia*	*Alpinia galanga*	JN043842
Monocotyledons	Zingiberaceae	*Alpinia*	*Alpinia galanga*	JN043843
Monocotyledons	Zingiberaceae	*Alpinia*	*Alpinia zerumbet*	GQ435051
Monocotyledons	Zingiberaceae	*Alpinia*	*Alpinia zerumbet*	GU180443

Monocotyledons	Zingiberaceae	*Alpinia*	*Alpinia zerumbet*	GU180444
Monocotyledons	Zingiberaceae	*Alpinia*	*Alpinia zerumbet*	GU180445
Monocotyledons	Zingiberaceae	*Alpinia*	*Alpinia zerumbet*	JN043874
Monocotyledons	Zingiberaceae	*Alpinia*	*Alpinia zerumbet*	JN043875
Monocotyledons	Zingiberaceae	*Alpinia*	*Alpinia zerumbet*	JN043876
Monocotyledons	Zingiberaceae	*Alpinia*	*Alpinia zerumbet*	JN043877
Monocotyledons	Zingiberaceae	*Alpinia*	*Alpinia zerumbet*	JN043878
Monocotyledons	Zingiberaceae	*Boesenbergia*	*Boesenbergia plicata*	DQ408316
Monocotyledons	Zingiberaceae	*Boesenbergia*	*Boesenbergia plicata*	DQ408317
Monocotyledons	Zingiberaceae	*Boesenbergia*	*Boesenbergia rotunda*	DQ408325
Monocotyledons	Zingiberaceae	*Boesenbergia*	*Boesenbergia rotunda*	DQ408326
Monocotyledons	Zingiberaceae	*Boesenbergia*	*Boesenbergia rotunda*	DQ408327
Monocotyledons	Zingiberaceae	*Kaempferia*	*Kaempferia elegans*	GQ386005
Monocotyledons	Zingiberaceae	*Kaempferia*	*Kaempferia elegans*	GQ386006
Monocotyledons	Zingiberaceae	*Kaempferia*	*Kaempferia elegans*	GQ386007
Monocotyledons	Zingiberaceae	*Kaempferia*	*Kaempferia elegans*	GQ386008
Monocotyledons	Zingiberaceae	*Kaempferia*	*Kaempferia elegans*	GQ386009
Monocotyledons	Zingiberaceae	*Kaempferia*	*Kaempferia elegans*	GQ386010
Monocotyledons	Zingiberaceae	*Kaempferia*	*Kaempferia elegans*	GQ386011
Monocotyledons	Zingiberaceae	*Kaempferia*	*Kaempferia elegans*	GU180452
Monocotyledons	Zingiberaceae	*Kaempferia*	*Kaempferia elegans*	GU180453
Monocotyledons	Zingiberaceae	*Kaempferia*	*Kaempferia galanga*	GQ385977
Monocotyledons	Zingiberaceae	*Kaempferia*	*Kaempferia galanga*	GQ385979
Monocotyledons	Zingiberaceae	*Kaempferia*	*Kaempferia galanga*	GQ385980
Monocotyledons	Zingiberaceae	*Kaempferia*	*Kaempferia galanga*	GQ435048
Monocotyledons	Zingiberaceae	*Kaempferia*	*Kaempferia galanga*	GQ435049
Monocotyledons	Zingiberaceae	*Kaempferia*	*Kaempferia galanga*	GU180437
Monocotyledons	Zingiberaceae	*Kaempferia*	*Kaempferia galanga*	GU180438
Monocotyledons	Zingiberaceae	*Kaempferia*	*Kaempferia galanga*	GU180439
Monocotyledons	Zingiberaceae	*Kaempferia*	*Kaempferia parviflora*	DQ408334
Monocotyledons	Zingiberaceae	*Kaempferia*	*Kaempferia parviflora*	GQ386012
Monocotyledons	Zingiberaceae	*Kaempferia*	*Kaempferia parviflora*	GQ386013
Monocotyledons	Zingiberaceae	*Kaempferia*	*Kaempferia parviflora*	GQ386014
Monocotyledons	Zingiberaceae	*Kaempferia*	*Kaempferia parviflora*	GQ386015
Monocotyledons	Zingiberaceae	*Kaempferia*	*Kaempferia roscoeana*	GQ386029
Monocotyledons	Zingiberaceae	*Kaempferia*	*Kaempferia roscoeana*	GQ386030
Monocotyledons	Zingiberaceae	*Kaempferia*	*Kaempferia rotunda*	GQ386031
Monocotyledons	Zingiberaceae	*Kaempferia*	*Kaempferia rotunda*	GQ386032
Monocotyledons	Zingiberaceae	*Kaempferia*	*Kaempferia rotunda*	GQ386033
Monocotyledons	Zingiberaceae	*Kaempferia*	*Kaempferia rotunda*	GQ386034
Monocotyledons	Zingiberaceae	*Kaempferia*	*Kaempferia rotunda*	GQ386035
Monocotyledons	Zingiberaceae	*Kaempferia*	*Kaempferia rotunda*	GQ386036
Monocotyledons	Zingiberaceae	*Kaempferia*	*Kaempferia rotunda*	GQ386037
Monocotyledons	Zingiberaceae	*Kaempferia*	*Kaempferia rotunda*	GU180430
Monocotyledons	Zingiberaceae	*Alpinia*	*Alpinia blepharocalyx*	JN043817
Monocotyledons	Zingiberaceae	*Alpinia*	*Alpinia blepharocalyx*	JN043818
Monocotyledons	Zingiberaceae	*Alpinia*	*Alpinia blepharocalyx*	JN043819
Monocotyledons	Zingiberaceae	*Alpinia*	*Alpinia conchigera*	JN043826
Monocotyledons	Zingiberaceae	*Alpinia*	*Alpinia conchigera*	JN043827
Monocotyledons	Zingiberaceae	*Alpinia*	*Alpinia conchigera*	JN043828
Monocotyledons	Zingiberaceae	*Alpinia*	*Alpinia intermedia*	JN043850
Monocotyledons	Zingiberaceae	*Alpinia*	*Alpinia intermedia*	JN043851
Monocotyledons	Zingiberaceae	*Alpinia*	*Alpinia intermedia*	JN043852

Monocotyledons	Zingiberaceae	*Alpinia*	*Alpinia intermedia*	JN043853
Monocotyledons	Zingiberaceae	*Alpinia*	*Alpinia nigra*	JN043858
Monocotyledons	Zingiberaceae	*Alpinia*	*Alpinia nigra*	JN043859
Monocotyledons	Zingiberaceae	*Alpinia*	*Alpinia polyantha*	GU180425
Monocotyledons	Zingiberaceae	*Alpinia*	*Alpinia polyantha*	JN043871
Monocotyledons	Zingiberaceae	*Alpinia*	*Alpinia polyantha*	JN043872
Monocotyledons	Zingiberaceae	*Alpinia*	*Alpinia polyantha*	JN043873
Monocotyledons	Amaryllidaceae	*Lycoris*	*Lycoris squamigera*	GQ923941
Monocotyledons	Amaryllidaceae	*Lycoris*	*Lycoris squamigera*	HM748829
Monocotyledons	Zingiberaceae	*Alpinia*	*Alpinia aquatica*	JN043815
Monocotyledons	Zingiberaceae	*Alpinia*	*Alpinia aquatica*	JN043816
Monocotyledons	Zingiberaceae	*Alpinia*	*Alpinia formosana*	JN043833
Monocotyledons	Zingiberaceae	*Alpinia*	*Alpinia formosana*	JN043834
Monocotyledons	Zingiberaceae	*Alpinia*	*Alpinia guinanensis*	JN043846
Monocotyledons	Zingiberaceae	*Alpinia*	*Alpinia guinanensis*	JN043847
Monocotyledons	Zingiberaceae	*Alpinia*	*Alpinia hainanensis*	GU180421
Monocotyledons	Zingiberaceae	*Alpinia*	*Alpinia hainanensis*	JN043848
Monocotyledons	Zingiberaceae	*Alpinia*	*Alpinia hainanensis*	JN043849
Monocotyledons	Zingiberaceae	*Alpinia*	*Alpinia oxyphylla*	GU180446
Monocotyledons	Zingiberaceae	*Alpinia*	*Alpinia oxyphylla*	GU180447
Monocotyledons	Zingiberaceae	*Alpinia*	*Alpinia oxyphylla*	JN043863
Monocotyledons	Zingiberaceae	*Alpinia*	*Alpinia oxyphylla*	JN043864
Monocotyledons	Zingiberaceae	*Alpinia*	*Alpinia oxyphylla*	JN043865
Monocotyledons	Zingiberaceae	*Alpinia*	*Alpinia oxyphylla*	JN043866
Monocotyledons	Zingiberaceae	*Curcuma*	*Curcuma aromatica*	GQ435050
Monocotyledons	Zingiberaceae	*Curcuma*	*Curcuma aromatica*	GU180440
Monocotyledons	Zingiberaceae	*Curcuma*	*Curcuma aromatica*	GU180441
Monocotyledons	Zingiberaceae	*Curcuma*	*Curcuma aromatica*	GU180442
Monocotyledons	Zingiberaceae	*Curcuma*	*Curcuma aromatica*	GU180448
Monocotyledons	Zingiberaceae	*Curcuma*	*Curcuma aromatica*	GU180449
Monocotyledons	Zingiberaceae	*Curcuma*	*Curcuma aromatica*	GU180450
Monocotyledons	Zingiberaceae	*Curcuma*	*Curcuma aromatica*	GU180451
Monocotyledons	Zingiberaceae	*Curcuma*	*Curcuma kwangsiensis*	GQ435042
Monocotyledons	Zingiberaceae	*Curcuma*	*Curcuma kwangsiensis*	GQ435043
Monocotyledons	Zingiberaceae	*Curcuma*	*Curcuma kwangsiensis*	EU552520
Monocotyledons	Zingiberaceae	*Curcuma*	*Curcuma kwangsiensis*	GU180429
Monocotyledons	Zingiberaceae	*Curcuma*	*Curcuma kwangsiensis*	JF730251
Monocotyledons	Zingiberaceae	*Curcuma*	*Curcuma longa*	FJ687416
Monocotyledons	Zingiberaceae	*Curcuma*	*Curcuma longa*	GU180434
Monocotyledons	Zingiberaceae	*Curcuma*	*Curcuma longa*	GU180435
Monocotyledons	Zingiberaceae	*Curcuma*	*Curcuma longa*	GU180436
Monocotyledons	Zingiberaceae	*Curcuma*	*Curcuma longa*	JF730221
Monocotyledons	Zingiberaceae	*Curcuma*	*Curcuma longa*	JF730222
Monocotyledons	Zingiberaceae	*Curcuma*	*Curcuma longa*	JF730223
Monocotyledons	Zingiberaceae	*Curcuma*	*Curcuma longa*	JF730224
Monocotyledons	Zingiberaceae	*Curcuma*	*Curcuma longa*	JF730225
Monocotyledons	Zingiberaceae	*Curcuma*	*Curcuma longa*	JF730226
Monocotyledons	Zingiberaceae	*Curcuma*	*Curcuma longa*	JF730227
Monocotyledons	Zingiberaceae	*Curcuma*	*Curcuma longa*	JF730228
Monocotyledons	Zingiberaceae	*Curcuma*	*Curcuma longa*	JF730229
Monocotyledons	Zingiberaceae	*Curcuma*	*Curcuma longa*	JF730230
Monocotyledons	Zingiberaceae	*Curcuma*	*Curcuma longa*	JF730231
Monocotyledons	Zingiberaceae	*Curcuma*	*Curcuma longa*	JF730232

Monocotyledons	Zingiberaceae	*Curcuma*	*Curcuma longa*	JF730233
Monocotyledons	Zingiberaceae	*Curcuma*	*Curcuma longa*	JF730234
Monocotyledons	Zingiberaceae	*Curcuma*	*Curcuma longa*	JF730235
Monocotyledons	Zingiberaceae	*Curcuma*	*Curcuma longa*	JF730236
Monocotyledons	Zingiberaceae	*Curcuma*	*Curcuma longa*	JF730237
Monocotyledons	Zingiberaceae	*Curcuma*	*Curcuma longa*	JF730238
Monocotyledons	Zingiberaceae	*Curcuma*	*Curcuma longa*	JF730239
Monocotyledons	Zingiberaceae	*Curcuma*	*Curcuma longa*	JF730253
Monocotyledons	Zingiberaceae	*Curcuma*	*Curcuma longa*	JF730254
Monocotyledons	Zingiberaceae	*Curcuma*	*Curcuma longa*	JF730255
Monocotyledons	Zingiberaceae	*Curcuma*	*Curcuma phaeocaulis*	EU552524
Monocotyledons	Zingiberaceae	*Curcuma*	*Curcuma phaeocaulis*	JF730247
Monocotyledons	Zingiberaceae	*Curcuma*	*Curcuma phaeocaulis*	JF730248
Monocotyledons	Zingiberaceae	*Curcuma*	*Curcuma phaeocaulis*	JF730249
Monocotyledons	Zingiberaceae	*Curcuma*	*Curcuma phaeocaulis*	JF730256
Monocotyledons	Zingiberaceae	*Curcuma*	*Curcuma phaeocaulis*	JF730257
Monocotyledons	Zingiberaceae	*Curcuma*	*Curcuma sichuanensis*	JF730240
Monocotyledons	Zingiberaceae	*Curcuma*	*Curcuma sichuanensis*	JF730241
Monocotyledons	Zingiberaceae	*Curcuma*	*Curcuma sichuanensis*	JF730242
Monocotyledons	Zingiberaceae	*Curcuma*	*Curcuma sichuanensis*	JF730243
Monocotyledons	Zingiberaceae	*Curcuma*	*Curcuma sichuanensis*	JF730244
Monocotyledons	Zingiberaceae	*Curcuma*	*Curcuma sichuanensis*	JF730245
Monocotyledons	Zingiberaceae	*Curcuma*	*Curcuma sichuanensis*	JF730246
Monocotyledons	Zingiberaceae	*Curcuma*	*Curcuma wenyujin*	EU552525
Monocotyledons	Zingiberaceae	*Curcuma*	*Curcuma wenyujin*	JF730252
Monocotyledons	Zingiberaceae	*Curcuma*	*Curcuma zedoaria*	FJ687417
Monocotyledons	Zingiberaceae	*Curcuma*	*Curcuma zedoaria*	GU180426
Monocotyledons	Amaryllidaceae	*Lycoris*	*Lycoris aurea*	GQ923938
Monocotyledons	Amaryllidaceae	*Lycoris*	*Lycoris aurea*	HM748822
Monocotyledons	Stemonaceae	*Stemona*	*Stemona tuberosa*	GQ434873
Monocotyledons	Stemonaceae	*Stemona*	*Stemona tuberosa*	GQ434874
Monocotyledons	Stemonaceae	*Stemona*	*Stemona tuberosa*	AB373199
Monocotyledons	Zingiberaceae	*Alpinia*	*Alpinia calcarata*	JN043820
Monocotyledons	Zingiberaceae	*Alpinia*	*Alpinia calcarata*	JN043821
Monocotyledons	Zingiberaceae	*Alpinia*	*Alpinia calcarata*	JN043822
Monocotyledons	Zingiberaceae	*Alpinia*	*Alpinia calcarata*	JN043823
Monocotyledons	Zingiberaceae	*Alpinia*	*Alpinia carolinensis*	JN043824
Monocotyledons	Zingiberaceae	*Alpinia*	*Alpinia carolinensis*	JN043825
Monocotyledons	Zingiberaceae	*Alpinia*	*Alpinia elegans*	JN043829
Monocotyledons	Zingiberaceae	*Alpinia*	*Alpinia elegans*	JN043830
Monocotyledons	Zingiberaceae	*Alpinia*	*Alpinia foxworthyi*	JN043835
Monocotyledons	Zingiberaceae	*Alpinia*	*Alpinia foxworthyi*	JN043836
Monocotyledons	Zingiberaceae	*Alpinia*	*Alpinia foxworthyi*	JN043837
Monocotyledons	Zingiberaceae	*Alpinia*	*Alpinia officinarum*	EU552529
Monocotyledons	Zingiberaceae	*Alpinia*	*Alpinia officinarum*	GU180427
Monocotyledons	Zingiberaceae	*Alpinia*	*Alpinia officinarum*	GU180428
Monocotyledons	Zingiberaceae	*Kaempferia*	*Kaempferia pulchra*	GQ386025
Monocotyledons	Zingiberaceae	*Kaempferia*	*Kaempferia pulchra*	GQ386026
Monocotyledons	Zingiberaceae	*Kaempferia*	*Kaempferia pulchra*	GQ386027
Monocotyledons	Zingiberaceae	*Kaempferia*	*Kaempferia pulchra*	GQ386028
Monocotyledons	Amaryllidaceae	*Lycoris*	*Lycoris albiflora*	GQ923943
Monocotyledons	Amaryllidaceae	*Lycoris*	*Lycoris albiflora*	HM748820
Monocotyledons	Zingiberaceae	*Alpinia*	*Alpinia jianganfeng*	JN043854

Monocotyledons	Zingiberaceae	*Alpinia*	*Alpinia jianganfeng*	JN043855
Monocotyledons	Amaryllidaceae	*Lycoris*	*Lycoris radiata*	GQ923942
Monocotyledons	Amaryllidaceae	*Lycoris*	*Lycoris radiata*	HM748828
Monocotyledons	Zingiberaceae	*Kaempferia*	*Kaempferia angustifolia*	GQ386041
Monocotyledons	Zingiberaceae	*Kaempferia*	*Kaempferia angustifolia*	GQ386042
Monocotyledons	Zingiberaceae	*Boesenbergia*	*Boesenbergia longiflora*	DQ408311
Monocotyledons	Zingiberaceae	*Boesenbergia*	*Boesenbergia longiflora*	DQ408312
Monocotyledons	Amaryllidaceae	*Lycoris*	*Lycoris chinensis*	GQ923949
Monocotyledons	Amaryllidaceae	*Lycoris*	*Lycoris chinensis*	HM748827
Monocotyledons	Amaryllidaceae	*Lycoris*	*Lycoris longituba*	GQ923944
Monocotyledons	Amaryllidaceae	*Lycoris*	*Lycoris longituba*	HM748817
Monocotyledons	Zingiberaceae	*Alpinia*	*Alpinia kwangsiensis*	GU180422
Monocotyledons	Zingiberaceae	*Alpinia*	*Alpinia kwangsiensis*	JN043856
Monocotyledons	Zingiberaceae	*Alpinia*	*Alpinia kwangsiensis*	JN043857
Monocotyledons	Amaryllidaceae	*Lycoris*	*Lycoris anhuiensis*	GQ923948
Monocotyledons	Amaryllidaceae	*Lycoris*	*Lycoris anhuiensis*	HM748823
Monocotyledons	Amaryllidaceae	*Lycoris*	*Lycoris sprengeri*	GQ923945
Monocotyledons	Amaryllidaceae	*Lycoris*	*Lycoris sprengeri*	HM748815
Monocotyledons	Amaryllidaceae	*Lycoris*	*Lycoris rosea*	GQ923947
Monocotyledons	Amaryllidaceae	*Lycoris*	*Lycoris rosea*	HM748825
Monocotyledons	Amaryllidaceae	*Lycoris*	*Lycoris haywardii*	GQ923950
Monocotyledons	Amaryllidaceae	*Lycoris*	*Lycoris haywardii*	HM748816
Monocotyledons	Amaryllidaceae	*Lycoris*	*Lycoris incarnata*	GQ923939
Monocotyledons	Amaryllidaceae	*Lycoris*	*Lycoris incarnata*	HM748830
Monocotyledons	Amaryllidaceae	*Lycoris*	*Lycoris straminea*	GQ923946
Monocotyledons	Amaryllidaceae	*Lycoris*	*Lycoris straminea*	HM748826
Monocotyledons	Zingiberaceae	*Boesenbergia*	*Boesenbergia curtisii*	DQ408309
Monocotyledons	Zingiberaceae	*Boesenbergia*	*Boesenbergia curtisii*	DQ408310
Monocotyledons	Zingiberaceae	*Boesenbergia*	*Boesenbergia longipes*	DQ408313
Monocotyledons	Zingiberaceae	*Boesenbergia*	*Boesenbergia longipes*	DQ408314
Monocotyledons	Zingiberaceae	*Kaempferia*	*Kaempferia candida*	GQ386003
Monocotyledons	Zingiberaceae	*Kaempferia*	*Kaempferia candida*	GQ386004
Monocotyledons	Zingiberaceae	*Kaempferia*	*Kaempferia marginata*	GQ385976
Monocotyledons	Zingiberaceae	*Kaempferia*	*Kaempferia marginata*	GQ385981
Monocotyledons	Zingiberaceae	*Kaempferia*	*Kaempferia marginata*	GQ385982
Monocotyledons	Zingiberaceae	*Kaempferia*	*Kaempferia marginata*	GQ385983
Monocotyledons	Zingiberaceae	*Kaempferia*	*Kaempferia marginata*	GQ385984
Monocotyledons	Zingiberaceae	*Alpinia*	*Alpinia oblongifolia*	JN043860
Monocotyledons	Zingiberaceae	*Alpinia*	*Alpinia oblongifolia*	JN043861
Monocotyledons	Zingiberaceae	*Alpinia*	*Alpinia oblongifolia*	JN043862
Monocotyledons	Zingiberaceae	*Curcuma*	*Curcuma cochinchinensis*	GQ248280
Monocotyledons	Zingiberaceae	*Curcuma*	*Curcuma cochinchinensis*	EF590684
Monocotyledons	Poaceae	*Pappostipa*	*Pappostipa vaginata*	EU489283
Monocotyledons	Poaceae	*Pappostipa*	*Pappostipa vaginata*	EU489284
Monocotyledons	Poaceae	*Pappostipa*	*Pappostipa vaginata*	EU204749
Monocotyledons	Poaceae	*Pappostipa*	*Pappostipa vaginata*	EU204750
Monocotyledons	Zingiberaceae	*Curcuma*	*Curcuma attenuata*	GQ248279
Monocotyledons	Zingiberaceae	*Curcuma*	*Curcuma attenuata*	EF590683
Monocotyledons	Poaceae	*Pappostipa*	*Pappostipa chrysophylla*	EU489257
Monocotyledons	Poaceae	*Pappostipa*	*Pappostipa chrysophylla*	EU489258
Monocotyledons	Poaceae	*Pappostipa*	*Pappostipa chrysophylla*	EU489259
Monocotyledons	Poaceae	*Pappostipa*	*Pappostipa chrysophylla*	EU204729
Monocotyledons	Poaceae	*Pappostipa*	*Pappostipa chrysophylla*	EU204730

Monocotyledons	Poaceae	*Pappostipa*	*Pappostipa speciosa*	EU489279
Monocotyledons	Poaceae	*Pappostipa*	*Pappostipa speciosa*	EU489281
Monocotyledons	Poaceae	*Pappostipa*	*Pappostipa speciosa*	EU489282
Monocotyledons	Poaceae	*Pappostipa*	*Pappostipa speciosa*	EU204736
Monocotyledons	Poaceae	*Pappostipa*	*Pappostipa speciosa*	EU204737
Monocotyledons	Poaceae	*Pappostipa*	*Pappostipa speciosa*	EU204738
Monocotyledons	Poaceae	*Pappostipa*	*Pappostipa speciosa*	EU204739
Monocotyledons	Poaceae	*Pappostipa*	*Pappostipa speciosa*	EU204740
Monocotyledons	Poaceae	*Pappostipa*	*Pappostipa speciosa*	EU204741
Monocotyledons	Poaceae	*Pappostipa*	*Pappostipa speciosa*	EU204742
Monocotyledons	Poaceae	*Pappostipa*	*Pappostipa speciosa*	EU204744
Monocotyledons	Poaceae	*Pappostipa*	*Pappostipa speciosa*	EU204745
Monocotyledons	Poaceae	*Pappostipa*	*Pappostipa major*	EU204743
Monocotyledons	Poaceae	*Pappostipa*	*Pappostipa major*	EU204746
Monocotyledons	Stemonaceae	*Stemona*	*Stemona collinsae*	AB373194
Monocotyledons	Stemonaceae	*Stemona*	*Stemona collinsae*	AB373195
Monocotyledons	Hypoxidaceae	*Pauridia*	*Pauridia minuta*	FM201362
Monocotyledons	Hypoxidaceae	*Pauridia*	*Pauridia minuta*	FM201363
Monocotyledons	Hypoxidaceae	*Pauridia*	*Pauridia minuta*	FM201364
Monocotyledons	Hypoxidaceae	*Pauridia*	*Pauridia minuta*	FM201365
Monocotyledons	Hypoxidaceae	*Pauridia*	*Pauridia minuta*	FM201366
Monocotyledons	Hypoxidaceae	*Pauridia*	*Pauridia minuta*	FM201367
Monocotyledons	Hypoxidaceae	*Pauridia*	*Pauridia minuta*	FM201368
Monocotyledons	Hypoxidaceae	*Pauridia*	*Pauridia minuta*	FM201369
Monocotyledons	Hypoxidaceae	*Pauridia*	*Pauridia minuta*	FM201370
Monocotyledons	Hypoxidaceae	*Pauridia*	*Pauridia minuta*	FM201371
Monocotyledons	Hypoxidaceae	*Pauridia*	*Pauridia minuta*	FM201372
Monocotyledons	Hypoxidaceae	*Pauridia*	*Pauridia minuta*	FM201373
Monocotyledons	Hypoxidaceae	*Pauridia*	*Pauridia minuta*	FM201374
Monocotyledons	Hypoxidaceae	*Pauridia*	*Pauridia minuta*	FM201375
Monocotyledons	Hypoxidaceae	*Pauridia*	*Pauridia minuta*	FM201376
Monocotyledons	Hypoxidaceae	*Pauridia*	*Pauridia minuta*	FM201377
Monocotyledons	Hypoxidaceae	*Pauridia*	*Pauridia minuta*	FM201378
Monocotyledons	Hypoxidaceae	*Pauridia*	*Pauridia minuta*	FM201379
Monocotyledons	Hypoxidaceae	*Pauridia*	*Pauridia minuta*	FM201380
Monocotyledons	Hypoxidaceae	*Pauridia*	*Pauridia minuta*	FM201381
Monocotyledons	Hypoxidaceae	*Pauridia*	*Pauridia minuta*	FM201382
Monocotyledons	Hypoxidaceae	*Pauridia*	*Pauridia minuta*	FM201383
Monocotyledons	Hypoxidaceae	*Pauridia*	*Pauridia minuta*	FM201384
Monocotyledons	Hypoxidaceae	*Pauridia*	*Pauridia minuta*	FM201385
Monocotyledons	Hypoxidaceae	*Pauridia*	*Pauridia minuta*	FM201386
Monocotyledons	Hypoxidaceae	*Pauridia*	*Pauridia minuta*	FM201387
Monocotyledons	Hypoxidaceae	*Pauridia*	*Pauridia minuta*	FM201388
Monocotyledons	Hypoxidaceae	*Pauridia*	*Pauridia minuta*	FM201389
Monocotyledons	Hypoxidaceae	*Pauridia*	*Pauridia minuta*	FM201390
Monocotyledons	Hypoxidaceae	*Pauridia*	*Pauridia minuta*	FM201391
Monocotyledons	Hypoxidaceae	*Pauridia*	*Pauridia minuta*	FM201392
Monocotyledons	Hypoxidaceae	*Pauridia*	*Pauridia minuta*	FM201393
Monocotyledons	Hypoxidaceae	*Pauridia*	*Pauridia minuta*	FM201394
Monocotyledons	Hypoxidaceae	*Pauridia*	*Pauridia minuta*	FM201395
Monocotyledons	Hypoxidaceae	*Pauridia*	*Pauridia minuta*	FM201396
Monocotyledons	Hypoxidaceae	*Pauridia*	*Pauridia minuta*	FM201397
Monocotyledons	Hypoxidaceae	*Pauridia*	*Pauridia minuta*	FM201398

Monocotyledons	Hypoxidaceae	*Pauridia*	*Pauridia minuta*	FM201399
Monocotyledons	Hypoxidaceae	*Pauridia*	*Pauridia minuta*	FM201400
Monocotyledons	Hypoxidaceae	*Pauridia*	*Pauridia minuta*	FM201401
Monocotyledons	Hypoxidaceae	*Pauridia*	*Pauridia minuta*	FM201402
Monocotyledons	Hypoxidaceae	*Pauridia*	*Pauridia minuta*	FM201403
Monocotyledons	Hypoxidaceae	*Pauridia*	*Pauridia minuta*	FM201404
Monocotyledons	Hypoxidaceae	*Pauridia*	*Pauridia minuta*	FM201405
Monocotyledons	Hypoxidaceae	*Pauridia*	*Pauridia minuta*	FM201406
Monocotyledons	Hypoxidaceae	*Pauridia*	*Pauridia minuta*	FM201407
Monocotyledons	Hypoxidaceae	*Pauridia*	*Pauridia minuta*	FM201408
Monocotyledons	Hypoxidaceae	*Pauridia*	*Pauridia minuta*	FM201409
Monocotyledons	Hypoxidaceae	*Pauridia*	*Pauridia minuta*	FM201410
Monocotyledons	Hypoxidaceae	*Pauridia*	*Pauridia minuta*	FM201411
Monocotyledons	Hypoxidaceae	*Pauridia*	*Pauridia minuta*	FM201412
Monocotyledons	Hypoxidaceae	*Pauridia*	*Pauridia minuta*	FM201413
Monocotyledons	Hypoxidaceae	*Pauridia*	*Pauridia minuta*	FM201414
Monocotyledons	Hypoxidaceae	*Pauridia*	*Pauridia minuta*	FM201415
Monocotyledons	Hypoxidaceae	*Pauridia*	*Pauridia minuta*	FM201416
Monocotyledons	Hypoxidaceae	*Pauridia*	*Pauridia minuta*	FM201417
Monocotyledons	Hypoxidaceae	*Pauridia*	*Pauridia minuta*	FM201418
Monocotyledons	Hypoxidaceae	*Pauridia*	*Pauridia minuta*	FM201419
Monocotyledons	Hypoxidaceae	*Pauridia*	*Pauridia minuta*	FM201420
Monocotyledons	Hypoxidaceae	*Pauridia*	*Pauridia minuta*	FM201421
Monocotyledons	Hypoxidaceae	*Pauridia*	*Pauridia minuta*	FM201422
Monocotyledons	Hypoxidaceae	*Pauridia*	*Pauridia minuta*	FM201423
Monocotyledons	Hypoxidaceae	*Pauridia*	*Pauridia minuta*	FM201424
Monocotyledons	Hypoxidaceae	*Pauridia*	*Pauridia minuta*	FM201425
Monocotyledons	Hypoxidaceae	*Pauridia*	*Pauridia minuta*	FM201426
Monocotyledons	Hypoxidaceae	*Pauridia*	*Pauridia minuta*	FM201427
Monocotyledons	Hypoxidaceae	*Pauridia*	*Pauridia minuta*	FM201428
Monocotyledons	Hypoxidaceae	*Pauridia*	*Pauridia minuta*	FM201429
Monocotyledons	Hypoxidaceae	*Pauridia*	*Pauridia minuta*	FM201430
Monocotyledons	Hypoxidaceae	*Pauridia*	*Pauridia minuta*	FM201431
Monocotyledons	Hypoxidaceae	*Pauridia*	*Pauridia minuta*	FM201432
Monocotyledons	Hypoxidaceae	*Pauridia*	*Pauridia minuta*	FM201433
Monocotyledons	Hypoxidaceae	*Pauridia*	*Pauridia minuta*	FM201434
Monocotyledons	Hypoxidaceae	*Pauridia*	*Pauridia minuta*	FM201435
Monocotyledons	Hypoxidaceae	*Pauridia*	*Pauridia minuta*	FM201436
Monocotyledons	Hypoxidaceae	*Pauridia*	*Pauridia minuta*	FM201437
Monocotyledons	Hypoxidaceae	*Pauridia*	*Pauridia minuta*	FM201438
Monocotyledons	Hypoxidaceae	*Pauridia*	*Pauridia minuta*	FM201439
Monocotyledons	Hypoxidaceae	*Pauridia*	*Pauridia minuta*	FM201440
Monocotyledons	Hypoxidaceae	*Pauridia*	*Pauridia minuta*	FM201441
Monocotyledons	Hypoxidaceae	*Pauridia*	*Pauridia minuta*	FM201442
Monocotyledons	Hypoxidaceae	*Pauridia*	*Pauridia minuta*	FM201443
Monocotyledons	Hypoxidaceae	*Pauridia*	*Pauridia minuta*	FM201444
Monocotyledons	Hypoxidaceae	*Pauridia*	*Pauridia minuta*	FM201445
Monocotyledons	Hypoxidaceae	*Pauridia*	*Pauridia minuta*	FM201446
Monocotyledons	Hypoxidaceae	*Pauridia*	*Pauridia minuta*	FM201447
Monocotyledons	Hypoxidaceae	*Pauridia*	*Pauridia minuta*	FM201448
Monocotyledons	Hypoxidaceae	*Pauridia*	*Pauridia minuta*	FM201449
Monocotyledons	Hypoxidaceae	*Pauridia*	*Pauridia minuta*	FM201450
Monocotyledons	Hypoxidaceae	*Pauridia*	*Pauridia minuta*	FM201451

Monocotyledons	Hyacinthaceae	*Hyacinthoides*	*Hyacinthoides ciliolata*	FJ423299
Monocotyledons	Hyacinthaceae	*Hyacinthoides*	*Hyacinthoides ciliolata*	FJ423300
Monocotyledons	Hyacinthaceae	*Hyacinthoides*	*Hyacinthoides ciliolata*	FJ423301
Monocotyledons	Hyacinthaceae	*Hyacinthoides*	*Hyacinthoides flahaultiana*	FJ423313
Monocotyledons	Hyacinthaceae	*Hyacinthoides*	*Hyacinthoides flahaultiana*	FJ423314
Monocotyledons	Hyacinthaceae	*Hyacinthoides*	*Hyacinthoides flahaultiana*	FJ423315
Monocotyledons	Hyacinthaceae	*Hyacinthoides*	*Hyacinthoides mauritanica*	FJ423309
Monocotyledons	Hyacinthaceae	*Hyacinthoides*	*Hyacinthoides mauritanica*	FJ423310
Monocotyledons	Hyacinthaceae	*Hyacinthoides*	*Hyacinthoides mauritanica*	FJ423311
Monocotyledons	Hyacinthaceae	*Hyacinthoides*	*Hyacinthoides mauritanica*	FJ423312
Monocotyledons	Hyacinthaceae	*Hyacinthoides*	*Hyacinthoides paivae*	FJ423346
Monocotyledons	Hyacinthaceae	*Hyacinthoides*	*Hyacinthoides paivae*	FJ423347
Monocotyledons	Zingiberaceae	*Kaempferia*	*Kaempferia sp. JT-2010a*	GQ385994
Monocotyledons	Zingiberaceae	*Kaempferia*	*Kaempferia sp. JT-2010a*	GQ385995
Monocotyledons	Zingiberaceae	*Kaempferia*	*Kaempferia sp. JT-2010b*	GQ385998
Monocotyledons	Zingiberaceae	*Kaempferia*	*Kaempferia sp. JT-2010b*	GQ385999
Monocotyledons	Zingiberaceae	*Kaempferia*	*Kaempferia fallax*	GQ386018
Monocotyledons	Zingiberaceae	*Kaempferia*	*Kaempferia fallax*	GQ386019
Monocotyledons	Zingiberaceae	*Kaempferia*	*Kaempferia fallax*	GQ386020
Monocotyledons	Zingiberaceae	*Kaempferia*	*Kaempferia fallax*	GQ386021
Monocotyledons	Zingiberaceae	*Kaempferia*	*Kaempferia filifolia*	GQ386022
Monocotyledons	Zingiberaceae	*Kaempferia*	*Kaempferia filifolia*	GQ386023
Monocotyledons	Zingiberaceae	*Kaempferia*	*Kaempferia filifolia*	GQ386024
Monocotyledons	Zingiberaceae	*Kaempferia*	*Kaempferia laotica*	GQ385985
Monocotyledons	Zingiberaceae	*Kaempferia*	*Kaempferia laotica*	GQ385987
Monocotyledons	Zingiberaceae	*Kaempferia*	*Kaempferia larsenii*	GQ385989
Monocotyledons	Zingiberaceae	*Kaempferia*	*Kaempferia larsenii*	GQ385990
Monocotyledons	Zingiberaceae	*Kaempferia*	*Kaempferia larsenii*	GQ385991
Monocotyledons	Zingiberaceae	*Kaempferia*	*Kaempferia larsenii*	GQ385992
Monocotyledons	Zingiberaceae	*Kaempferia*	*Kaempferia larsenii*	GQ385993
Monocotyledons	Zingiberaceae	*Kaempferia*	*Kaempferia sp. JT-2010c*	GQ386043
Monocotyledons	Zingiberaceae	*Kaempferia*	*Kaempferia sp. JT-2010c*	GQ386044
Monocotyledons	Zingiberaceae	*Kaempferia*	*Kaempferia sp. JT-2010c*	GQ386045
Monocotyledons	Zingiberaceae	*Kaempferia*	*Kaempferia sp. JT-2010c*	GQ386046
Monocotyledons	Zingiberaceae	*Kaempferia*	*Kaempferia sp. JT-2010d*	GQ385996
Monocotyledons	Zingiberaceae	*Kaempferia*	*Kaempferia sp. JT-2010d*	GQ385997
Monocotyledons	Zingiberaceae	*Kaempferia*	*Kaempferia siamensis*	GQ386039
Monocotyledons	Zingiberaceae	*Kaempferia*	*Kaempferia siamensis*	GQ386040
Monocotyledons	Zingiberaceae	*Kaempferia*	*Kaempferia sp. nov. 1 JT-2010*	GQ386001
Monocotyledons	Zingiberaceae	*Kaempferia*	*Kaempferia sp. nov. 1 JT-2010*	GQ386002
Monocotyledons	Poaceae	*Digitaria*	*Digitaria macroblephara*	HQ876950
Monocotyledons	Poaceae	*Digitaria*	*Digitaria macroblephara*	HQ876951
Monocotyledons	Poaceae	*Digitaria*	*Digitaria macroblephara*	HQ876952
Monocotyledons	Poaceae	*Digitaria*	*Digitaria abyssinica*	HQ876953
Monocotyledons	Poaceae	*Digitaria*	*Digitaria abyssinica*	HQ876954
Monocotyledons	Poaceae	*Digitaria*	*Digitaria ternata*	HQ876955
Monocotyledons	Poaceae	*Digitaria*	*Digitaria ternata*	HQ876956
Monocotyledons	Zingiberaceae	*Alpinia*	*Alpinia emaculata*	JN043831
Monocotyledons	Zingiberaceae	*Alpinia*	*Alpinia emaculata*	JN043832
Monocotyledons	Zingiberaceae	*Alpinia*	*Alpinia platychilus*	JN043867
Monocotyledons	Zingiberaceae	*Alpinia*	*Alpinia platychilus*	JN043868
Monocotyledons	Zingiberaceae	*Alpinia*	*Alpinia platychilus*	JN043869
Monocotyledons	Zingiberaceae	*Alpinia*	*Alpinia platychilus*	JN043870

Monocotyledons	Zingiberaceae	*Alpinia*	*Alpinia graminifolia*	JN043844
Monocotyledons	Zingiberaceae	*Alpinia*	*Alpinia graminifolia*	JN043845
