# Supplementary material for: Utility of the trnH–psbA Intergenic Spacer Region and Its Combinations as Plant DNA Barcodes: A Meta-Analysis
Source: PLoS One. 2012 Nov 14;7(11):e48833. doi: 10.1371/journal.pone.0048833 (PMC3498263; doi:10.1371/journal.pone.0048833)
Supplement: Table S2 — List of species with inversions in their sequences. (PDF) [file pone.0048833.s002.pdf]

Table S2. List of species with inversions in their sequences.

Taxonomy group	Family	Genus	Species	GenBank accesstion no.
Eudicotyledons	Paeoniaceae	*Paeonia*	*Paeonia anomala*	DQ313731
Eudicotyledons	Paeoniaceae	*Paeonia*	*Paeonia anomala*	DQ313732
Eudicotyledons	Paeoniaceae	*Paeonia*	*Paeonia anomala*	DQ313733
Eudicotyledons	Paeoniaceae	*Paeonia*	*Paeonia anomala*	DQ313734
Eudicotyledons	Paeoniaceae	*Paeonia*	*Paeonia anomala*	DQ313735
Eudicotyledons	Paeoniaceae	*Paeonia*	*Paeonia anomala*	GQ435203
Eudicotyledons	Paeoniaceae	*Paeonia*	*Paeonia anomala*	GQ435204
Eudicotyledons	Paeoniaceae	*Paeonia*	*Paeonia jishanensis*	GU367369
Eudicotyledons	Paeoniaceae	*Paeonia*	*Paeonia jishanensis*	GU367370
Eudicotyledons	Vitaceae	*Ampelopsis*	*Ampelopsis megalophylla*	JQ182521
Eudicotyledons	Vitaceae	*Ampelopsis*	*Ampelopsis megalophylla*	HQ656484
Eudicotyledons	Melastomataceae	*Melastoma*	*Melastoma dodecandrum*	GQ435424
Eudicotyledons	Melastomataceae	*Melastoma*	*Melastoma dodecandrum*	JN040997
Eudicotyledons	Vitaceae	*Cayratia*	*Cayratia japonica*	JQ182535
Eudicotyledons	Vitaceae	*Cayratia*	*Cayratia japonica*	HM585664
Eudicotyledons	Vitaceae	*Cayratia*	*Cayratia japonica*	JF437078
Eudicotyledons	Vitaceae	*Cayratia*	*Cayratia japonica*	JF437082
Eudicotyledons	Vitaceae	*Cayratia*	*Cayratia japonica*	JF437083
Eudicotyledons	Vitaceae	*Cayratia*	*Cayratia japonica*	JF437084
Eudicotyledons	Fagaceae	*Castanopsis*	*Castanopsis fissa*	HQ415580
Eudicotyledons	Fagaceae	*Castanopsis*	*Castanopsis fissa*	JN044211
Eudicotyledons	Fagaceae	*Castanopsis*	*Castanopsis fissa*	JN044212
Eudicotyledons	Fagaceae	*Castanopsis*	*Castanopsis fissa*	JN044213
Eudicotyledons	Fabaceae	*Inga*	*Inga punctata*	GQ118882
Eudicotyledons	Fabaceae	*Inga*	*Inga punctata*	GQ982259
Eudicotyledons	Fabaceae	*Inga*	*Inga punctata*	AM922040
Eudicotyledons	Fabaceae	*Inga*	*Inga punctata*	AM922041
Eudicotyledons	Fabaceae	*Inga*	*Inga punctata*	AM922042
Eudicotyledons	Fabaceae	*Inga*	*Inga punctata*	AM922043
Eudicotyledons	Fabaceae	*Inga*	*Inga punctata*	AM922054
Eudicotyledons	Plantaginaceae	*Synthyris*	*Synthyris dissecta*	EU232348
Eudicotyledons	Plantaginaceae	*Synthyris*	*Synthyris dissecta*	EU232349
Eudicotyledons	Plantaginaceae	*Synthyris*	*Synthyris dissecta*	EU232350
Eudicotyledons	Plantaginaceae	*Synthyris*	*Synthyris dissecta*	EU232351
Eudicotyledons	Plantaginaceae	*Synthyris*	*Synthyris dissecta*	EU232352
Eudicotyledons	Plantaginaceae	*Synthyris*	*Synthyris dissecta*	EU232353
Eudicotyledons	Plantaginaceae	*Synthyris*	*Synthyris dissecta*	EU232354
Eudicotyledons	Plantaginaceae	*Synthyris*	*Synthyris dissecta*	EU232355
Eudicotyledons	Plantaginaceae	*Synthyris*	*Synthyris dissecta*	EU232356
Eudicotyledons	Plantaginaceae	*Synthyris*	*Synthyris dissecta*	EU232357
Eudicotyledons	Plantaginaceae	*Synthyris*	*Synthyris dissecta*	EU232358
Eudicotyledons	Plantaginaceae	*Synthyris*	*Synthyris dissecta*	EU232359
Eudicotyledons	Plantaginaceae	*Synthyris*	*Synthyris dissecta*	EU232360
Eudicotyledons	Plantaginaceae	*Synthyris*	*Synthyris dissecta*	EU232361
Eudicotyledons	Plantaginaceae	*Synthyris*	*Synthyris dissecta*	EU232362
Eudicotyledons	Plantaginaceae	*Synthyris*	*Synthyris dissecta*	EU232363
Eudicotyledons	Plantaginaceae	*Synthyris*	*Synthyris dissecta*	EU232364
Eudicotyledons	Plantaginaceae	*Synthyris*	*Synthyris dissecta*	EU232365
Eudicotyledons	Plantaginaceae	*Synthyris*	*Synthyris dissecta*	EU232366
Eudicotyledons	Plantaginaceae	*Synthyris*	*Synthyris dissecta*	EU232367
Eudicotyledons	Plantaginaceae	*Synthyris*	*Synthyris dissecta*	EU232368

Eudicotyledons	Plantaginaceae	*Synthyris*	*Synthyris dissecta*	EU232369
Eudicotyledons	Plantaginaceae	*Synthyris*	*Synthyris dissecta*	EU232370
Eudicotyledons	Plantaginaceae	*Synthyris*	*Synthyris dissecta*	EU232371
Eudicotyledons	Plantaginaceae	*Synthyris*	*Synthyris dissecta*	EU232372
Eudicotyledons	Plantaginaceae	*Synthyris*	*Synthyris dissecta*	EU232373
Eudicotyledons	Plantaginaceae	*Synthyris*	*Synthyris dissecta*	EU232374
Eudicotyledons	Plantaginaceae	*Synthyris*	*Synthyris dissecta*	EU232375
Eudicotyledons	Plantaginaceae	*Synthyris*	*Synthyris dissecta*	EU232376
Eudicotyledons	Plantaginaceae	*Synthyris*	*Synthyris dissecta*	EU232377
Eudicotyledons	Plantaginaceae	*Synthyris*	*Synthyris dissecta*	EU232378
Eudicotyledons	Plantaginaceae	*Synthyris*	*Synthyris dissecta*	EU232379
Eudicotyledons	Plantaginaceae	*Synthyris*	*Synthyris dissecta*	EU232380
Eudicotyledons	Plantaginaceae	*Synthyris*	*Synthyris dissecta*	EU232381
Eudicotyledons	Plantaginaceae	*Synthyris*	*Synthyris dissecta*	EU232382
Eudicotyledons	Plantaginaceae	*Synthyris*	*Synthyris dissecta*	EU232383
Eudicotyledons	Plantaginaceae	*Synthyris*	*Synthyris dissecta*	EU232384
Eudicotyledons	Plantaginaceae	*Synthyris*	*Synthyris dissecta*	EU232385
Eudicotyledons	Plantaginaceae	*Synthyris*	*Synthyris dissecta*	EU232386
Eudicotyledons	Plantaginaceae	*Synthyris*	*Synthyris dissecta*	EU232387
Eudicotyledons	Plantaginaceae	*Synthyris*	*Synthyris dissecta*	EU232388
Eudicotyledons	Plantaginaceae	*Synthyris*	*Synthyris dissecta*	EU232389
Eudicotyledons	Plantaginaceae	*Synthyris*	*Synthyris dissecta*	EU232390
Eudicotyledons	Plantaginaceae	*Synthyris*	*Synthyris dissecta*	EU232391
Eudicotyledons	Plantaginaceae	*Synthyris*	*Synthyris dissecta*	EU232392
Eudicotyledons	Plantaginaceae	*Synthyris*	*Synthyris dissecta*	EU232393
Eudicotyledons	Plantaginaceae	*Synthyris*	*Synthyris dissecta*	EU232394
Eudicotyledons	Plantaginaceae	*Synthyris*	*Synthyris dissecta*	EU232395
Eudicotyledons	Plantaginaceae	*Synthyris*	*Synthyris dissecta*	EU232396
Eudicotyledons	Plantaginaceae	*Synthyris*	*Synthyris dissecta*	EU232397
Eudicotyledons	Plantaginaceae	*Synthyris*	*Synthyris dissecta*	EU232398
Eudicotyledons	Plantaginaceae	*Synthyris*	*Synthyris dissecta*	EU232399
Eudicotyledons	Plantaginaceae	*Synthyris*	*Synthyris dissecta*	EU232400
Eudicotyledons	Plantaginaceae	*Synthyris*	*Synthyris dissecta*	EU232401
Eudicotyledons	Plantaginaceae	*Synthyris*	*Synthyris dissecta*	EU232402
Eudicotyledons	Plantaginaceae	*Synthyris*	*Synthyris dissecta*	EU232403
Eudicotyledons	Plantaginaceae	*Synthyris*	*Synthyris dissecta*	EU232404
Eudicotyledons	Plantaginaceae	*Synthyris*	*Synthyris dissecta*	EU232405
Eudicotyledons	Plantaginaceae	*Synthyris*	*Synthyris dissecta*	EU232406
Eudicotyledons	Plantaginaceae	*Synthyris*	*Synthyris dissecta*	EU232422
Eudicotyledons	Plantaginaceae	*Synthyris*	*Synthyris dissecta*	EU232423
Eudicotyledons	Plantaginaceae	*Synthyris*	*Synthyris dissecta*	EU232424
Eudicotyledons	Plantaginaceae	*Synthyris*	*Synthyris dissecta*	EU232425
Eudicotyledons	Plantaginaceae	*Synthyris*	*Synthyris dissecta*	EU232426
Eudicotyledons	Plantaginaceae	*Synthyris*	*Synthyris dissecta*	EU232427
Eudicotyledons	Plantaginaceae	*Synthyris*	*Synthyris dissecta*	EU232428
Eudicotyledons	Plantaginaceae	*Synthyris*	*Synthyris dissecta*	EU232429
Eudicotyledons	Plantaginaceae	*Synthyris*	*Synthyris dissecta*	EU232430
Eudicotyledons	Plantaginaceae	*Synthyris*	*Synthyris dissecta*	EU232431
Eudicotyledons	Plantaginaceae	*Synthyris*	*Synthyris dissecta*	EU232432
Eudicotyledons	Plantaginaceae	*Synthyris*	*Synthyris dissecta*	EU232433
Eudicotyledons	Plantaginaceae	*Synthyris*	*Synthyris dissecta*	EU232434
Eudicotyledons	Plantaginaceae	*Synthyris*	*Synthyris dissecta*	EU232435
Eudicotyledons	Plantaginaceae	*Synthyris*	*Synthyris dissecta*	EU232436

Eudicotyledons	Plantaginaceae	*Synthyris*	*Synthyris dissecta*	EU232438
Eudicotyledons	Plantaginaceae	*Synthyris*	*Synthyris dissecta*	EU232439
Eudicotyledons	Plantaginaceae	*Synthyris*	*Synthyris dissecta*	EU232440
Eudicotyledons	Plantaginaceae	*Synthyris*	*Synthyris dissecta*	EU232441
Eudicotyledons	Plantaginaceae	*Synthyris*	*Synthyris dissecta*	EU232442
Eudicotyledons	Plantaginaceae	*Synthyris*	*Synthyris dissecta*	EU232443
Eudicotyledons	Plantaginaceae	*Synthyris*	*Synthyris dissecta*	EU232444
Eudicotyledons	Plantaginaceae	*Synthyris*	*Synthyris dissecta*	EU232445
Eudicotyledons	Plantaginaceae	*Synthyris*	*Synthyris dissecta*	EU232446
Eudicotyledons	Plantaginaceae	*Synthyris*	*Synthyris dissecta*	EU232447
Eudicotyledons	Plantaginaceae	*Synthyris*	*Synthyris dissecta*	EU232448
Eudicotyledons	Plantaginaceae	*Synthyris*	*Synthyris dissecta*	EU232449
Eudicotyledons	Plantaginaceae	*Synthyris*	*Synthyris dissecta*	EU232450
Eudicotyledons	Plantaginaceae	*Synthyris*	*Synthyris dissecta*	EU232451
Eudicotyledons	Plantaginaceae	*Synthyris*	*Synthyris dissecta*	EU232452
Eudicotyledons	Plantaginaceae	*Synthyris*	*Synthyris dissecta*	EU232453
Eudicotyledons	Plantaginaceae	*Synthyris*	*Synthyris dissecta*	EU232454
Eudicotyledons	Plantaginaceae	*Synthyris*	*Synthyris dissecta*	EU232455
Eudicotyledons	Plantaginaceae	*Synthyris*	*Synthyris dissecta*	EU232456
Eudicotyledons	Plantaginaceae	*Synthyris*	*Synthyris dissecta*	EU232457
Eudicotyledons	Plantaginaceae	*Synthyris*	*Synthyris dissecta*	EU232458
Eudicotyledons	Plantaginaceae	*Synthyris*	*Synthyris dissecta*	EU232459
Eudicotyledons	Plantaginaceae	*Synthyris*	*Synthyris dissecta*	EU232460
Eudicotyledons	Plantaginaceae	*Synthyris*	*Synthyris dissecta*	EU232461
Eudicotyledons	Plantaginaceae	*Synthyris*	*Synthyris dissecta*	EU232462
Eudicotyledons	Plantaginaceae	*Synthyris*	*Synthyris dissecta*	EU232463
Eudicotyledons	Plantaginaceae	*Synthyris*	*Synthyris dissecta*	EU232464
Eudicotyledons	Plantaginaceae	*Synthyris*	*Synthyris dissecta*	EU232465
Eudicotyledons	Plantaginaceae	*Synthyris*	*Synthyris dissecta*	EU232466
Eudicotyledons	Plantaginaceae	*Synthyris*	*Synthyris dissecta*	EF550011
Eudicotyledons	Plantaginaceae	*Synthyris*	*Synthyris dissecta*	EF550012
Eudicotyledons	Plantaginaceae	*Synthyris*	*Synthyris dissecta*	EF550013
Eudicotyledons	Plantaginaceae	*Synthyris*	*Synthyris dissecta*	EF550014
Eudicotyledons	Plantaginaceae	*Synthyris*	*Synthyris dissecta*	EF550015
Eudicotyledons	Plantaginaceae	*Synthyris*	*Synthyris dissecta*	EF550016
Eudicotyledons	Plantaginaceae	*Synthyris*	*Synthyris dissecta*	EF550017
Eudicotyledons	Plantaginaceae	*Synthyris*	*Synthyris dissecta*	EF550018
Eudicotyledons	Plantaginaceae	*Synthyris*	*Synthyris dissecta*	EF550020
Eudicotyledons	Plantaginaceae	*Synthyris*	*Synthyris dissecta*	EF550022
Eudicotyledons	Plantaginaceae	*Synthyris*	*Synthyris dissecta*	EF550023
Eudicotyledons	Plantaginaceae	*Synthyris*	*Synthyris dissecta*	EF550024
Eudicotyledons	Plantaginaceae	*Synthyris*	*Synthyris dissecta*	EF550025
Eudicotyledons	Plantaginaceae	*Synthyris*	*Synthyris dissecta*	EF550026
Eudicotyledons	Plantaginaceae	*Synthyris*	*Synthyris dissecta*	EF550027
Eudicotyledons	Plantaginaceae	*Synthyris*	*Synthyris dissecta*	EF550028
Eudicotyledons	Plantaginaceae	*Synthyris*	*Synthyris dissecta*	EF550029
Eudicotyledons	Plantaginaceae	*Synthyris*	*Synthyris dissecta*	EF550030
Eudicotyledons	Polygonaceae	*Oxyria*	*Oxyria digyna*	JQ080918
Eudicotyledons	Polygonaceae	*Oxyria*	*Oxyria digyna*	JQ080919
Eudicotyledons	Polygonaceae	*Oxyria*	*Oxyria digyna*	JQ080920
Eudicotyledons	Polygonaceae	*Oxyria*	*Oxyria digyna*	JQ080921
Eudicotyledons	Polygonaceae	*Oxyria*	*Oxyria digyna*	JQ080922
Eudicotyledons	Polygonaceae	*Oxyria*	*Oxyria digyna*	JQ080923

Eudicotyledons	Polygonaceae	*Oxyria*	*Oxyria digyna*	JQ080924
Eudicotyledons	Polygonaceae	*Oxyria*	*Oxyria digyna*	JQ080925
Eudicotyledons	Polygonaceae	*Oxyria*	*Oxyria digyna*	JQ080926
Eudicotyledons	Polygonaceae	*Oxyria*	*Oxyria digyna*	JQ080927
Eudicotyledons	Polygonaceae	*Oxyria*	*Oxyria digyna*	JQ080928
Eudicotyledons	Polygonaceae	*Oxyria*	*Oxyria digyna*	JQ080929
Eudicotyledons	Polygonaceae	*Oxyria*	*Oxyria digyna*	JQ080930
Eudicotyledons	Polygonaceae	*Oxyria*	*Oxyria digyna*	JQ080931
Eudicotyledons	Polygonaceae	*Oxyria*	*Oxyria digyna*	JQ080932
Eudicotyledons	Polygonaceae	*Oxyria*	*Oxyria digyna*	JQ080933
Eudicotyledons	Polygonaceae	*Oxyria*	*Oxyria digyna*	JQ080934
Eudicotyledons	Polygonaceae	*Oxyria*	*Oxyria digyna*	JQ080935
Eudicotyledons	Polygonaceae	*Oxyria*	*Oxyria digyna*	JQ080936
Eudicotyledons	Polygonaceae	*Oxyria*	*Oxyria digyna*	JQ080937
Eudicotyledons	Polygonaceae	*Oxyria*	*Oxyria digyna*	JQ080938
Eudicotyledons	Polygonaceae	*Oxyria*	*Oxyria digyna*	JQ080939
Eudicotyledons	Polygonaceae	*Oxyria*	*Oxyria digyna*	JQ080940
Eudicotyledons	Polygonaceae	*Oxyria*	*Oxyria digyna*	JQ080941
Eudicotyledons	Polygonaceae	*Oxyria*	*Oxyria digyna*	JQ080942
Eudicotyledons	Polygonaceae	*Oxyria*	*Oxyria digyna*	JQ080943
Eudicotyledons	Polygonaceae	*Oxyria*	*Oxyria digyna*	JQ080944
Eudicotyledons	Polygonaceae	*Oxyria*	*Oxyria digyna*	JQ080945
Eudicotyledons	Polygonaceae	*Oxyria*	*Oxyria digyna*	JQ080946
Eudicotyledons	Polygonaceae	*Oxyria*	*Oxyria digyna*	JQ080947
Eudicotyledons	Polygonaceae	*Oxyria*	*Oxyria digyna*	JQ080948
Eudicotyledons	Polygonaceae	*Oxyria*	*Oxyria digyna*	JQ080949
Eudicotyledons	Polygonaceae	*Oxyria*	*Oxyria digyna*	JQ080950
Eudicotyledons	Polygonaceae	*Oxyria*	*Oxyria digyna*	JQ080951
Eudicotyledons	Polygonaceae	*Oxyria*	*Oxyria digyna*	JQ080952
Eudicotyledons	Polygonaceae	*Oxyria*	*Oxyria digyna*	JQ080953
Eudicotyledons	Polygonaceae	*Oxyria*	*Oxyria digyna*	JQ080954
Eudicotyledons	Polygonaceae	*Oxyria*	*Oxyria digyna*	JQ080955
Eudicotyledons	Polygonaceae	*Oxyria*	*Oxyria digyna*	JQ080956
Eudicotyledons	Polygonaceae	*Oxyria*	*Oxyria digyna*	JQ080957
Eudicotyledons	Polygonaceae	*Oxyria*	*Oxyria digyna*	JQ080958
Eudicotyledons	Polygonaceae	*Oxyria*	*Oxyria digyna*	JQ080959
Eudicotyledons	Polygonaceae	*Oxyria*	*Oxyria digyna*	JQ080960
Eudicotyledons	Polygonaceae	*Oxyria*	*Oxyria digyna*	JQ080961
Eudicotyledons	Polygonaceae	*Oxyria*	*Oxyria digyna*	JQ080962
Eudicotyledons	Polygonaceae	*Oxyria*	*Oxyria digyna*	GQ435162
Eudicotyledons	Polygonaceae	*Oxyria*	*Oxyria digyna*	EU554051
Eudicotyledons	Polygonaceae	*Oxyria*	*Oxyria digyna*	JN045690
Eudicotyledons	Polygonaceae	*Oxyria*	*Oxyria digyna*	JN045691
Eudicotyledons	Polygonaceae	*Oxyria*	*Oxyria digyna*	JN045692
Eudicotyledons	Polygonaceae	*Oxyria*	*Oxyria digyna*	JN045693
Eudicotyledons	Polygonaceae	*Oxyria*	*Oxyria digyna*	JN045694
Eudicotyledons	Polygonaceae	*Oxyria*	*Oxyria digyna*	JN045695
Eudicotyledons	Vitaceae	*Ampelopsis*	*Ampelopsis cantoniensis*	JQ182498
Eudicotyledons	Vitaceae	*Ampelopsis*	*Ampelopsis cantoniensis*	JN043979
Eudicotyledons	Vitaceae	*Ampelopsis*	*Ampelopsis cantoniensis*	JN043980
Eudicotyledons	Vitaceae	*Ampelopsis*	*Ampelopsis cantoniensis*	JN043981
Eudicotyledons	Vitaceae	*Ampelopsis*	*Ampelopsis cantoniensis*	JN043982
Eudicotyledons	Vitaceae	*Ampelopsis*	*Ampelopsis cantoniensis*	HM585660

Eudicotyledons	Vitaceae	*Ampelopsis*	*Ampelopsis cantoniensis*	JF437062
Eudicotyledons	Malvaceae	*Althaea*	*Althaea hirsuta*	EF419620
Eudicotyledons	Malvaceae	*Althaea*	*Althaea hirsuta*	EF419621
Eudicotyledons	Malvaceae	*Althaea*	*Althaea hirsuta*	EF419622
Eudicotyledons	Malvaceae	*Althaea*	*Althaea hirsuta*	EF419623
Eudicotyledons	Malvaceae	*Althaea*	*Althaea hirsuta*	EF419624
Eudicotyledons	Malvaceae	*Althaea*	*Althaea hirsuta*	EF419625
Eudicotyledons	Polygonaceae	*Bistorta*	*Bistorta macrophylla*	JN046427
Eudicotyledons	Polygonaceae	*Bistorta*	*Bistorta macrophylla*	JN046428
Eudicotyledons	Polygonaceae	*Bistorta*	*Bistorta macrophylla*	JN046429
Eudicotyledons	Polygonaceae	*Bistorta*	*Bistorta macrophylla*	JN046430
Eudicotyledons	Polygonaceae	*Bistorta*	*Bistorta macrophylla*	JN046431
Eudicotyledons	Polygonaceae	*Bistorta*	*Bistorta macrophylla*	JN046432
Eudicotyledons	Polygonaceae	*Bistorta*	*Bistorta macrophylla*	JN046433
Eudicotyledons	Polygonaceae	*Bistorta*	*Bistorta macrophylla*	JN046434
Eudicotyledons	Rosaceae	*Cliffortia*	*Cliffortia linearifolia*	EU937691
Eudicotyledons	Rosaceae	*Cliffortia*	*Cliffortia linearifolia*	EU937692
Eudicotyledons	Rosaceae	*Cliffortia*	*Cliffortia marginata*	EU937668
Eudicotyledons	Rosaceae	*Cliffortia*	*Cliffortia marginata*	EU937669
Eudicotyledons	Oleaceae	*Ligustrum*	*Ligustrum strongylophyllum*	JF830267
Eudicotyledons	Oleaceae	*Ligustrum*	*Ligustrum strongylophyllum*	JF830268
Eudicotyledons	Oleaceae	*Ligustrum*	*Ligustrum strongylophyllum*	JF830269
Eudicotyledons	Oleaceae	*Ligustrum*	*Ligustrum strongylophyllum*	JN045244
Eudicotyledons	Oleaceae	*Ligustrum*	*Ligustrum strongylophyllum*	JN045245
Eudicotyledons	Rosaceae	*Pyracantha*	*Pyracantha crenulata*	JN046706
Eudicotyledons	Rosaceae	*Pyracantha*	*Pyracantha crenulata*	JN046707
Eudicotyledons	Rosaceae	*Pyracantha*	*Pyracantha crenulata*	JN046708
Eudicotyledons	Fagaceae	*Castanopsis*	*Castanopsis lamontii*	JN044214
Eudicotyledons	Fagaceae	*Castanopsis*	*Castanopsis lamontii*	JN044215
Eudicotyledons	Fagaceae	*Castanopsis*	*Castanopsis lamontii*	JN044216
Eudicotyledons	Fagaceae	*Castanopsis*	*Castanopsis lamontii*	JN044217
Eudicotyledons	Fagaceae	*Castanopsis*	*Castanopsis lamontii*	JN044218
Eudicotyledons	Fabaceae	*Schotia*	*Schotia brachypetala*	GQ405092
Eudicotyledons	Fabaceae	*Schotia*	*Schotia brachypetala*	GQ405098
Eudicotyledons	Fabaceae	*Schotia*	*Schotia brachypetala*	GQ405109
Eudicotyledons	Fabaceae	*Schotia*	*Schotia brachypetala*	GQ405121
Eudicotyledons	Fabaceae	*Schotia*	*Schotia brachypetala*	GQ405131
Eudicotyledons	Fabaceae	*Schotia*	*Schotia brachypetala*	GQ405132
Eudicotyledons	Lamiaceae	*Mentha*	*Mentha spicata*	DQ667354
Eudicotyledons	Lamiaceae	*Mentha*	*Mentha spicata*	EU585744
Eudicotyledons	Lamiaceae	*Mentha*	*Mentha spicata*	EU627573
Eudicotyledons	Lamiaceae	*Mentha*	*Mentha spicata*	EU627578
Eudicotyledons	Lamiaceae	*Mentha*	*Mentha spicata*	FR726101
Eudicotyledons	Lamiaceae	*Mentha*	*Mentha spicata*	FR726102
Eudicotyledons	Lamiaceae	*Mentha*	*Mentha spicata*	FJ513095
Eudicotyledons	Lamiaceae	*Mentha*	*Mentha spicata*	AY643666
Eudicotyledons	Lamiaceae	*Mentha*	*Mentha spicata*	AY643667
Eudicotyledons	Lamiaceae	*Mentha*	*Mentha spicata*	AY643668
Eudicotyledons	Lamiaceae	*Mentha*	*Mentha spicata*	AY643669
Eudicotyledons	Lamiaceae	*Mentha*	*Mentha spicata*	AY643670
Eudicotyledons	Lamiaceae	*Mentha*	*Mentha spicata*	AY643671
Eudicotyledons	Lamiaceae	*Mentha*	*Mentha spicata*	AY643672
Eudicotyledons	Lamiaceae	*Mentha*	*Mentha spicata*	AY643673

Eudicotyledons	Lamiaceae	*Mentha*	*Mentha spicata*	AY643674
Eudicotyledons	Lamiaceae	*Mentha*	*Mentha spicata*	AY643675
Eudicotyledons	Lamiaceae	*Mentha*	*Mentha spicata*	AY643676
Eudicotyledons	Lamiaceae	*Mentha*	*Mentha spicata*	AY643677
Eudicotyledons	Lamiaceae	*Mentha*	*Mentha spicata*	AY643678
Eudicotyledons	Lamiaceae	*Mentha*	*Mentha spicata*	HQ902826
Eudicotyledons	Lamiaceae	*Mentha*	*Mentha spicata*	HQ902827
Eudicotyledons	Lamiaceae	*Mentha*	*Mentha spicata*	HQ902854
Eudicotyledons	Lamiaceae	*Mentha*	*Mentha spicata*	HQ902865
Eudicotyledons	Rosaceae	*Rubus*	*Rubus idaeus*	EU750527
Eudicotyledons	Rosaceae	*Rubus*	*Rubus idaeus*	EU750528
Eudicotyledons	Rosaceae	*Rubus*	*Rubus idaeus*	EU750529
Eudicotyledons	Rosaceae	*Rubus*	*Rubus idaeus*	EU750530
Eudicotyledons	Rosaceae	*Rubus*	*Rubus idaeus*	EU750531
Eudicotyledons	Papaveraceae	*Papaver*	*Papaver rhoeas*	GQ248360
Eudicotyledons	Papaveraceae	*Papaver*	*Papaver rhoeas*	JN584665
Eudicotyledons	Papaveraceae	*Papaver*	*Papaver rhoeas*	EF590721
Eudicotyledons	Lamiaceae	*Mentha*	*Mentha x piperita*	FR726096
Eudicotyledons	Lamiaceae	*Mentha*	*Mentha x piperita*	FR726097
Eudicotyledons	Lamiaceae	*Mentha*	*Mentha x piperita*	FR726098
Eudicotyledons	Lamiaceae	*Mentha*	*Mentha x piperita*	AY643698
Eudicotyledons	Lamiaceae	*Mentha*	*Mentha x piperita*	AY643699
Eudicotyledons	Lamiaceae	*Mentha*	*Mentha x piperita*	AY643700
Eudicotyledons	Lamiaceae	*Mentha*	*Mentha x piperita*	AY643701
Eudicotyledons	Lamiaceae	*Mentha*	*Mentha x piperita*	AY643702
Eudicotyledons	Lamiaceae	*Mentha*	*Mentha x piperita*	AY643703
Eudicotyledons	Lamiaceae	*Mentha*	*Mentha x piperita*	AY643704
Eudicotyledons	Lamiaceae	*Mentha*	*Mentha x piperita*	AY643705
Eudicotyledons	Lamiaceae	*Mentha*	*Mentha x piperita*	AY643706
Eudicotyledons	Lamiaceae	*Mentha*	*Mentha x piperita*	AY643707
Eudicotyledons	Rutaceae	*Citrus*	*Citrus x paradisi*	JN315366
Eudicotyledons	Rutaceae	*Citrus*	*Citrus x paradisi*	JN315367
Eudicotyledons	Rutaceae	*Citrus*	*Citrus x paradisi*	HM446909
Eudicotyledons	Lamiaceae	*Mentha*	*Mentha longifolia*	AY643679
Eudicotyledons	Lamiaceae	*Mentha*	*Mentha longifolia*	AY643680
Eudicotyledons	Lamiaceae	*Mentha*	*Mentha longifolia*	AY643681
Eudicotyledons	Lamiaceae	*Mentha*	*Mentha longifolia*	AY643682
Eudicotyledons	Lamiaceae	*Mentha*	*Mentha longifolia*	AY643683
Eudicotyledons	Lamiaceae	*Mentha*	*Mentha longifolia*	AY643684
Eudicotyledons	Lamiaceae	*Mentha*	*Mentha longifolia*	AY643685
Eudicotyledons	Lamiaceae	*Mentha*	*Mentha longifolia*	AY643686
Eudicotyledons	Lamiaceae	*Mentha*	*Mentha longifolia*	HQ902825
Eudicotyledons	Lamiaceae	*Mentha*	*Mentha suaveolens*	AY643687
Eudicotyledons	Lamiaceae	*Mentha*	*Mentha suaveolens*	AY643688
Eudicotyledons	Lamiaceae	*Mentha*	*Mentha suaveolens*	AY643689
Eudicotyledons	Lamiaceae	*Mentha*	*Mentha suaveolens*	AY643690
Eudicotyledons	Lamiaceae	*Mentha*	*Mentha suaveolens*	AY643691
Eudicotyledons	Lamiaceae	*Mentha*	*Mentha suaveolens*	AY643692
Eudicotyledons	Lamiaceae	*Mentha*	*Mentha suaveolens*	AY643693
Eudicotyledons	Lamiaceae	*Mentha*	*Mentha suaveolens*	AY643694
Eudicotyledons	Lamiaceae	*Mentha*	*Mentha suaveolens*	AY643695
Eudicotyledons	Lamiaceae	*Mentha*	*Mentha suaveolens*	AY643696
Eudicotyledons	Lamiaceae	*Mentha*	*Mentha suaveolens*	AY643697

Eudicotyledons	Lamiaceae	*Mentha*	*Mentha suaveolens*	HQ902829
Eudicotyledons	Lamiaceae	*Mentha*	*Mentha suaveolens*	HQ902855
Eudicotyledons	Lamiaceae	*Salvia*	*Salvia officinalis*	JQ339263
Eudicotyledons	Lamiaceae	*Salvia*	*Salvia officinalis*	DQ667342
Eudicotyledons	Lamiaceae	*Salvia*	*Salvia officinalis*	FR726139
Eudicotyledons	Lamiaceae	*Salvia*	*Salvia officinalis*	FR726140
Eudicotyledons	Lamiaceae	*Salvia*	*Salvia officinalis*	FR726141
Eudicotyledons	Lamiaceae	*Salvia*	*Salvia officinalis*	FJ513122
Eudicotyledons	Gentianaceae	*Gentiana*	*Gentiana algida*	HM460868
Eudicotyledons	Gentianaceae	*Gentiana*	*Gentiana algida*	HM460869
Eudicotyledons	Gentianaceae	*Gentiana*	*Gentiana algida*	HM460870
Eudicotyledons	Caprifoliaceae	*Valeriana*	*Valeriana jatamansi*	GU477680
Eudicotyledons	Caprifoliaceae	*Valeriana*	*Valeriana jatamansi*	GU477681
Eudicotyledons	Caprifoliaceae	*Valeriana*	*Valeriana jatamansi*	GU477682
Eudicotyledons	Caprifoliaceae	*Valeriana*	*Valeriana jatamansi*	GU477683
Eudicotyledons	Caprifoliaceae	*Valeriana*	*Valeriana jatamansi*	GU477684
Eudicotyledons	Caprifoliaceae	*Valeriana*	*Valeriana jatamansi*	GU477685
Eudicotyledons	Caprifoliaceae	*Valeriana*	*Valeriana jatamansi*	GU477686
Eudicotyledons	Caprifoliaceae	*Valeriana*	*Valeriana jatamansi*	GU477687
Eudicotyledons	Caprifoliaceae	*Valeriana*	*Valeriana jatamansi*	GQ434936
Eudicotyledons	Rosaceae	*Rubus*	*Rubus occidentalis*	HQ596824
Eudicotyledons	Rosaceae	*Rubus*	*Rubus occidentalis*	EU750532
Eudicotyledons	Rosaceae	*Rubus*	*Rubus occidentalis*	EU750533
Eudicotyledons	Rosaceae	*Rubus*	*Rubus occidentalis*	EU750534
Eudicotyledons	Rosaceae	*Rubus*	*Rubus occidentalis*	EU750535
Eudicotyledons	Moraceae	*Ficus*	*Ficus insipida*	GQ438012
Eudicotyledons	Moraceae	*Ficus*	*Ficus insipida*	GQ438013
Eudicotyledons	Moraceae	*Ficus*	*Ficus insipida*	GQ438014
Eudicotyledons	Moraceae	*Ficus*	*Ficus insipida*	GQ438015
Eudicotyledons	Moraceae	*Ficus*	*Ficus insipida*	GQ438016
Eudicotyledons	Moraceae	*Ficus*	*Ficus insipida*	GQ438017
Eudicotyledons	Moraceae	*Ficus*	*Ficus insipida*	GQ438018
Eudicotyledons	Moraceae	*Ficus*	*Ficus insipida*	GQ438019
Eudicotyledons	Moraceae	*Ficus*	*Ficus insipida*	GQ438020
Eudicotyledons	Moraceae	*Ficus*	*Ficus insipida*	GQ438021
Eudicotyledons	Moraceae	*Ficus*	*Ficus insipida*	GQ438022
Eudicotyledons	Moraceae	*Ficus*	*Ficus insipida*	GQ438023
Eudicotyledons	Moraceae	*Ficus*	*Ficus insipida*	GQ438024
Eudicotyledons	Moraceae	*Ficus*	*Ficus insipida*	GQ438025
Eudicotyledons	Moraceae	*Ficus*	*Ficus insipida*	GQ438026
Eudicotyledons	Moraceae	*Ficus*	*Ficus insipida*	GQ438027
Eudicotyledons	Moraceae	*Ficus*	*Ficus insipida*	GQ438028
Eudicotyledons	Moraceae	*Ficus*	*Ficus insipida*	GQ438029
Eudicotyledons	Moraceae	*Ficus*	*Ficus insipida*	GQ438030
Eudicotyledons	Moraceae	*Ficus*	*Ficus insipida*	GQ438031
Eudicotyledons	Moraceae	*Ficus*	*Ficus insipida*	GQ438032
Eudicotyledons	Moraceae	*Ficus*	*Ficus insipida*	GQ438033
Eudicotyledons	Moraceae	*Ficus*	*Ficus insipida*	GQ438034
Eudicotyledons	Moraceae	*Ficus*	*Ficus insipida*	GQ438035
Eudicotyledons	Moraceae	*Ficus*	*Ficus insipida*	GQ438036
Eudicotyledons	Moraceae	*Ficus*	*Ficus insipida*	GQ438037
Eudicotyledons	Moraceae	*Ficus*	*Ficus insipida*	GQ438038
Eudicotyledons	Moraceae	*Ficus*	*Ficus insipida*	GQ438039

Eudicotyledons	Moraceae	*Ficus*	*Ficus insipida*	GQ438040
Eudicotyledons	Moraceae	*Ficus*	*Ficus insipida*	GQ438041
Eudicotyledons	Moraceae	*Ficus*	*Ficus insipida*	GQ438042
Eudicotyledons	Moraceae	*Ficus*	*Ficus insipida*	GQ438043
Eudicotyledons	Moraceae	*Ficus*	*Ficus insipida*	GQ438044
Eudicotyledons	Moraceae	*Ficus*	*Ficus insipida*	GQ438045
Eudicotyledons	Moraceae	*Ficus*	*Ficus insipida*	GQ438046
Eudicotyledons	Moraceae	*Ficus*	*Ficus insipida*	GQ438047
Eudicotyledons	Moraceae	*Ficus*	*Ficus insipida*	GQ438048
Eudicotyledons	Moraceae	*Ficus*	*Ficus insipida*	GQ438049
Eudicotyledons	Moraceae	*Ficus*	*Ficus insipida*	GQ438050
Eudicotyledons	Moraceae	*Ficus*	*Ficus insipida*	GQ438051
Eudicotyledons	Moraceae	*Ficus*	*Ficus insipida*	GQ438052
Eudicotyledons	Moraceae	*Ficus*	*Ficus insipida*	GQ438053
Eudicotyledons	Moraceae	*Ficus*	*Ficus insipida*	GQ438054
Eudicotyledons	Moraceae	*Ficus*	*Ficus insipida*	GQ438055
Eudicotyledons	Moraceae	*Ficus*	*Ficus insipida*	GQ438056
Eudicotyledons	Moraceae	*Ficus*	*Ficus insipida*	GQ438057
Eudicotyledons	Moraceae	*Ficus*	*Ficus insipida*	GQ438058
Eudicotyledons	Moraceae	*Ficus*	*Ficus insipida*	GQ438059
Eudicotyledons	Moraceae	*Ficus*	*Ficus insipida*	GQ438060
Eudicotyledons	Moraceae	*Ficus*	*Ficus insipida*	GQ438061
Eudicotyledons	Moraceae	*Ficus*	*Ficus insipida*	GQ438062
Eudicotyledons	Moraceae	*Ficus*	*Ficus insipida*	GQ438063
Eudicotyledons	Moraceae	*Ficus*	*Ficus insipida*	GQ438064
Eudicotyledons	Moraceae	*Ficus*	*Ficus insipida*	GQ438065
Eudicotyledons	Moraceae	*Ficus*	*Ficus insipida*	GQ438066
Eudicotyledons	Moraceae	*Ficus*	*Ficus insipida*	GQ438067
Eudicotyledons	Moraceae	*Ficus*	*Ficus insipida*	GQ438068
Eudicotyledons	Moraceae	*Ficus*	*Ficus insipida*	GQ438069
Eudicotyledons	Moraceae	*Ficus*	*Ficus insipida*	GQ438070
Eudicotyledons	Moraceae	*Ficus*	*Ficus insipida*	GQ438071
Eudicotyledons	Moraceae	*Ficus*	*Ficus insipida*	GQ438072
Eudicotyledons	Moraceae	*Ficus*	*Ficus insipida*	GQ438073
Eudicotyledons	Moraceae	*Ficus*	*Ficus insipida*	GQ438074
Eudicotyledons	Moraceae	*Ficus*	*Ficus insipida*	GQ438075
Eudicotyledons	Moraceae	*Ficus*	*Ficus insipida*	GQ438076
Eudicotyledons	Moraceae	*Ficus*	*Ficus insipida*	GQ438077
Eudicotyledons	Moraceae	*Ficus*	*Ficus insipida*	GQ438078
Eudicotyledons	Moraceae	*Ficus*	*Ficus insipida*	GQ438079
Eudicotyledons	Moraceae	*Ficus*	*Ficus insipida*	GQ438081
Eudicotyledons	Moraceae	*Ficus*	*Ficus insipida*	GQ438082
Eudicotyledons	Moraceae	*Ficus*	*Ficus insipida*	GQ438083
Eudicotyledons	Moraceae	*Ficus*	*Ficus insipida*	GQ438084
Eudicotyledons	Moraceae	*Ficus*	*Ficus insipida*	GQ438085
Eudicotyledons	Moraceae	*Ficus*	*Ficus insipida*	GQ438086
Eudicotyledons	Moraceae	*Ficus*	*Ficus insipida*	GQ438087
Eudicotyledons	Moraceae	*Ficus*	*Ficus insipida*	GQ438088
Eudicotyledons	Moraceae	*Ficus*	*Ficus insipida*	GQ438089
Eudicotyledons	Moraceae	*Ficus*	*Ficus insipida*	GQ438090
Eudicotyledons	Moraceae	*Ficus*	*Ficus insipida*	GQ438091
Eudicotyledons	Moraceae	*Ficus*	*Ficus insipida*	GQ438092
Eudicotyledons	Moraceae	*Ficus*	*Ficus insipida*	GQ438093

Eudicotyledons	Moraceae	*Ficus*	*Ficus insipida*	GQ438094
Eudicotyledons	Moraceae	*Ficus*	*Ficus insipida*	GQ438095
Eudicotyledons	Moraceae	*Ficus*	*Ficus insipida*	GQ438096
Eudicotyledons	Moraceae	*Ficus*	*Ficus insipida*	GQ438097
Eudicotyledons	Moraceae	*Ficus*	*Ficus insipida*	GQ438098
Eudicotyledons	Moraceae	*Ficus*	*Ficus insipida*	GQ438099
Eudicotyledons	Moraceae	*Ficus*	*Ficus insipida*	GQ438100
Eudicotyledons	Moraceae	*Ficus*	*Ficus insipida*	GQ438101
Eudicotyledons	Moraceae	*Ficus*	*Ficus insipida*	GQ438102
Eudicotyledons	Moraceae	*Ficus*	*Ficus insipida*	GQ438103
Eudicotyledons	Moraceae	*Ficus*	*Ficus insipida*	GQ438104
Eudicotyledons	Moraceae	*Ficus*	*Ficus insipida*	GQ438105
Eudicotyledons	Moraceae	*Ficus*	*Ficus insipida*	GQ438106
Eudicotyledons	Moraceae	*Ficus*	*Ficus insipida*	GQ438107
Eudicotyledons	Moraceae	*Ficus*	*Ficus insipida*	GQ438108
Eudicotyledons	Moraceae	*Ficus*	*Ficus insipida*	GQ438109
Eudicotyledons	Moraceae	*Ficus*	*Ficus insipida*	GQ438110
Eudicotyledons	Moraceae	*Ficus*	*Ficus insipida*	GQ438111
Eudicotyledons	Moraceae	*Ficus*	*Ficus insipida*	GQ438112
Eudicotyledons	Moraceae	*Ficus*	*Ficus insipida*	GQ438113
Eudicotyledons	Moraceae	*Ficus*	*Ficus insipida*	GQ438114
Eudicotyledons	Moraceae	*Ficus*	*Ficus insipida*	GQ438115
Eudicotyledons	Moraceae	*Ficus*	*Ficus insipida*	GQ438116
Eudicotyledons	Moraceae	*Ficus*	*Ficus insipida*	GQ438117
Eudicotyledons	Moraceae	*Ficus*	*Ficus insipida*	GQ438118
Eudicotyledons	Moraceae	*Ficus*	*Ficus insipida*	GQ438119
Eudicotyledons	Moraceae	*Ficus*	*Ficus insipida*	GQ438120
Eudicotyledons	Moraceae	*Ficus*	*Ficus insipida*	GQ438121
Eudicotyledons	Moraceae	*Ficus*	*Ficus insipida*	GQ438122
Eudicotyledons	Moraceae	*Ficus*	*Ficus insipida*	GQ438123
Eudicotyledons	Moraceae	*Ficus*	*Ficus insipida*	GQ438124
Eudicotyledons	Moraceae	*Ficus*	*Ficus insipida*	GQ438125
Eudicotyledons	Moraceae	*Ficus*	*Ficus insipida*	GQ438126
Eudicotyledons	Moraceae	*Ficus*	*Ficus insipida*	GQ438127
Eudicotyledons	Moraceae	*Ficus*	*Ficus insipida*	GQ438128
Eudicotyledons	Moraceae	*Ficus*	*Ficus insipida*	GQ438129
Eudicotyledons	Moraceae	*Ficus*	*Ficus insipida*	GQ438130
Eudicotyledons	Moraceae	*Ficus*	*Ficus insipida*	GQ438131
Eudicotyledons	Moraceae	*Ficus*	*Ficus insipida*	GQ438132
Eudicotyledons	Moraceae	*Ficus*	*Ficus insipida*	GQ438133
Eudicotyledons	Moraceae	*Ficus*	*Ficus insipida*	GQ438134
Eudicotyledons	Moraceae	*Ficus*	*Ficus insipida*	GQ438135
Eudicotyledons	Moraceae	*Ficus*	*Ficus insipida*	GQ438136
Eudicotyledons	Moraceae	*Ficus*	*Ficus insipida*	GQ438137
Eudicotyledons	Moraceae	*Ficus*	*Ficus insipida*	GQ438138
Eudicotyledons	Moraceae	*Ficus*	*Ficus insipida*	GQ438139
Eudicotyledons	Moraceae	*Ficus*	*Ficus insipida*	GQ438140
Eudicotyledons	Moraceae	*Ficus*	*Ficus insipida*	GQ438141
Eudicotyledons	Moraceae	*Ficus*	*Ficus insipida*	GQ438142
Eudicotyledons	Moraceae	*Ficus*	*Ficus insipida*	GQ438143
Eudicotyledons	Moraceae	*Ficus*	*Ficus insipida*	GQ438144
Eudicotyledons	Moraceae	*Ficus*	*Ficus insipida*	GQ438145
Eudicotyledons	Moraceae	*Ficus*	*Ficus insipida*	GQ438146

Eudicotyledons	Moraceae	*Ficus*	*Ficus insipida*	GQ438147
Eudicotyledons	Moraceae	*Ficus*	*Ficus insipida*	GQ438148
Eudicotyledons	Moraceae	*Ficus*	*Ficus insipida*	GQ438149
Eudicotyledons	Moraceae	*Ficus*	*Ficus insipida*	GQ438150
Eudicotyledons	Moraceae	*Ficus*	*Ficus insipida*	GQ438151
Eudicotyledons	Moraceae	*Ficus*	*Ficus insipida*	GQ438152
Eudicotyledons	Moraceae	*Ficus*	*Ficus insipida*	GQ438153
Eudicotyledons	Moraceae	*Ficus*	*Ficus insipida*	GQ438154
Eudicotyledons	Moraceae	*Ficus*	*Ficus insipida*	GQ438155
Eudicotyledons	Moraceae	*Ficus*	*Ficus insipida*	GQ438156
Eudicotyledons	Moraceae	*Ficus*	*Ficus insipida*	GQ438157
Eudicotyledons	Moraceae	*Ficus*	*Ficus insipida*	GQ438158
Eudicotyledons	Moraceae	*Ficus*	*Ficus insipida*	GQ438159
Eudicotyledons	Moraceae	*Ficus*	*Ficus insipida*	GQ438160
Eudicotyledons	Moraceae	*Ficus*	*Ficus insipida*	GQ438161
Eudicotyledons	Moraceae	*Ficus*	*Ficus insipida*	GQ438162
Eudicotyledons	Moraceae	*Ficus*	*Ficus insipida*	GQ438163
Eudicotyledons	Moraceae	*Ficus*	*Ficus insipida*	GQ438164
Eudicotyledons	Moraceae	*Ficus*	*Ficus insipida*	GQ438165
Eudicotyledons	Moraceae	*Ficus*	*Ficus insipida*	GQ438166
Eudicotyledons	Moraceae	*Ficus*	*Ficus insipida*	GQ438167
Eudicotyledons	Moraceae	*Ficus*	*Ficus insipida*	GQ438168
Eudicotyledons	Moraceae	*Ficus*	*Ficus insipida*	GQ438169
Eudicotyledons	Moraceae	*Ficus*	*Ficus insipida*	GQ438170
Eudicotyledons	Moraceae	*Ficus*	*Ficus insipida*	GQ438171
Eudicotyledons	Moraceae	*Ficus*	*Ficus insipida*	GQ438172
Eudicotyledons	Moraceae	*Ficus*	*Ficus insipida*	GQ438173
Eudicotyledons	Moraceae	*Ficus*	*Ficus insipida*	GQ438174
Eudicotyledons	Moraceae	*Ficus*	*Ficus insipida*	GQ438175
Eudicotyledons	Moraceae	*Ficus*	*Ficus insipida*	GQ438176
Eudicotyledons	Moraceae	*Ficus*	*Ficus insipida*	GQ438177
Eudicotyledons	Moraceae	*Ficus*	*Ficus insipida*	GQ438178
Eudicotyledons	Moraceae	*Ficus*	*Ficus insipida*	GQ438179
Eudicotyledons	Moraceae	*Ficus*	*Ficus insipida*	GQ438180
Eudicotyledons	Moraceae	*Ficus*	*Ficus insipida*	GQ438181
Eudicotyledons	Moraceae	*Ficus*	*Ficus insipida*	GQ438182
Eudicotyledons	Moraceae	*Ficus*	*Ficus insipida*	GQ438183
Eudicotyledons	Moraceae	*Ficus*	*Ficus insipida*	GQ438184
Eudicotyledons	Moraceae	*Ficus*	*Ficus insipida*	GQ438185
Eudicotyledons	Moraceae	*Ficus*	*Ficus insipida*	GQ438186
Eudicotyledons	Moraceae	*Ficus*	*Ficus insipida*	GQ438187
Eudicotyledons	Moraceae	*Ficus*	*Ficus insipida*	GQ438188
Eudicotyledons	Moraceae	*Ficus*	*Ficus insipida*	GQ438189
Eudicotyledons	Moraceae	*Ficus*	*Ficus insipida*	GQ438190
Eudicotyledons	Moraceae	*Ficus*	*Ficus insipida*	GQ438191
Eudicotyledons	Moraceae	*Ficus*	*Ficus insipida*	GQ438193
Eudicotyledons	Moraceae	*Ficus*	*Ficus insipida*	GQ438194
Eudicotyledons	Moraceae	*Ficus*	*Ficus insipida*	GQ438195
Eudicotyledons	Moraceae	*Ficus*	*Ficus insipida*	GQ438196
Eudicotyledons	Moraceae	*Ficus*	*Ficus insipida*	GQ438197
Eudicotyledons	Moraceae	*Ficus*	*Ficus insipida*	GQ438198
Eudicotyledons	Moraceae	*Ficus*	*Ficus insipida*	GQ438199
Eudicotyledons	Moraceae	*Ficus*	*Ficus insipida*	GQ438200

Eudicotyledons	Moraceae	*Ficus*	*Ficus insipida*	GQ438201
Eudicotyledons	Moraceae	*Ficus*	*Ficus insipida*	GQ438202
Eudicotyledons	Moraceae	*Ficus*	*Ficus insipida*	GQ438203
Eudicotyledons	Moraceae	*Ficus*	*Ficus insipida*	GQ438204
Eudicotyledons	Moraceae	*Ficus*	*Ficus insipida*	GQ438205
Eudicotyledons	Moraceae	*Ficus*	*Ficus insipida*	GQ438206
Eudicotyledons	Moraceae	*Ficus*	*Ficus insipida*	GQ438207
Eudicotyledons	Moraceae	*Ficus*	*Ficus insipida*	GQ438208
Eudicotyledons	Moraceae	*Ficus*	*Ficus insipida*	GQ438209
Eudicotyledons	Moraceae	*Ficus*	*Ficus insipida*	GQ438210
Eudicotyledons	Moraceae	*Ficus*	*Ficus insipida*	GQ438211
Eudicotyledons	Moraceae	*Ficus*	*Ficus insipida*	GQ982221
Eudicotyledons	Crassulaceae	*Aeonium*	*Aeonium balsamiferum*	AY082192
Eudicotyledons	Crassulaceae	*Aeonium*	*Aeonium balsamiferum*	AY082202
Eudicotyledons	Solanaceae	*Lycium*	*Lycium ruthenicum*	HM195011
Eudicotyledons	Solanaceae	*Lycium*	*Lycium ruthenicum*	HM195012
Eudicotyledons	Solanaceae	*Lycium*	*Lycium ruthenicum*	JN045288
Eudicotyledons	Solanaceae	*Lycium*	*Lycium ruthenicum*	JN045289
Eudicotyledons	Solanaceae	*Lycium*	*Lycium ruthenicum*	JN045290
Eudicotyledons	Ranunculaceae	*Ranunculus*	*Ranunculus crithmifolius*	FJ744168
Eudicotyledons	Ranunculaceae	*Ranunculus*	*Ranunculus crithmifolius*	FJ744169
Eudicotyledons	Ranunculaceae	*Ranunculus*	*Ranunculus crithmifolius*	FJ744170
Eudicotyledons	Ranunculaceae	*Ranunculus*	*Ranunculus insignis*	FJ744174
Eudicotyledons	Ranunculaceae	*Ranunculus*	*Ranunculus insignis*	FJ744175
Eudicotyledons	Ranunculaceae	*Ranunculus*	*Ranunculus insignis*	FJ744176
Eudicotyledons	Fabaceae	*Schotia*	*Schotia latifolia*	GQ405091
Eudicotyledons	Fabaceae	*Schotia*	*Schotia latifolia*	GQ405093
Eudicotyledons	Fabaceae	*Schotia*	*Schotia latifolia*	GQ405096
Eudicotyledons	Fabaceae	*Schotia*	*Schotia latifolia*	GQ405102
Eudicotyledons	Fabaceae	*Schotia*	*Schotia latifolia*	GQ405111
Eudicotyledons	Fabaceae	*Schotia*	*Schotia latifolia*	GQ405112
Eudicotyledons	Fabaceae	*Schotia*	*Schotia latifolia*	GQ405116
Eudicotyledons	Fabaceae	*Schotia*	*Schotia latifolia*	GQ405118
Eudicotyledons	Fabaceae	*Schotia*	*Schotia latifolia*	GQ405119
Eudicotyledons	Fabaceae	*Schotia*	*Schotia latifolia*	GQ405120
Eudicotyledons	Fabaceae	*Schotia*	*Schotia latifolia*	GQ405126
Eudicotyledons	Moraceae	*Ficus*	*Ficus benjamina*	GU935092
Eudicotyledons	Moraceae	*Ficus*	*Ficus benjamina*	GU935093
Eudicotyledons	Moraceae	*Ficus*	*Ficus benjamina*	FN675798
Eudicotyledons	Moraceae	*Ficus*	*Ficus benjamina*	JN044511
Eudicotyledons	Moraceae	*Ficus*	*Ficus benjamina*	JN044512
Eudicotyledons	Lamiaceae	*Mentha*	*Mentha aquatica*	FR726099
Eudicotyledons	Lamiaceae	*Mentha*	*Mentha aquatica*	FR726100
Eudicotyledons	Lamiaceae	*Mentha*	*Mentha aquatica*	AY643656
Eudicotyledons	Lamiaceae	*Mentha*	*Mentha aquatica*	AY643657
Eudicotyledons	Lamiaceae	*Mentha*	*Mentha aquatica*	AY643658
Eudicotyledons	Lamiaceae	*Mentha*	*Mentha aquatica*	AY643659
Eudicotyledons	Lamiaceae	*Mentha*	*Mentha aquatica*	AY643660
Eudicotyledons	Lamiaceae	*Mentha*	*Mentha aquatica*	AY643661
Eudicotyledons	Lamiaceae	*Mentha*	*Mentha aquatica*	AY643662
Eudicotyledons	Asteraceae	*Encelia*	*Encelia farinosa*	DQ661036
Eudicotyledons	Asteraceae	*Encelia*	*Encelia farinosa*	DQ661037
Eudicotyledons	Asteraceae	*Encelia*	*Encelia farinosa*	DQ661038

Eudicotyledons	Asteraceae	*Encelia*	*Encelia farinosa*	DQ661039
Eudicotyledons	Asteraceae	*Encelia*	*Encelia farinosa*	DQ661040
Eudicotyledons	Asteraceae	*Encelia*	*Encelia farinosa*	DQ661041
Eudicotyledons	Asteraceae	*Encelia*	*Encelia farinosa*	DQ661042
Eudicotyledons	Moraceae	*Brosimum*	*Brosimum alicastrum*	GQ436998
Eudicotyledons	Moraceae	*Brosimum*	*Brosimum alicastrum*	GQ436999
Eudicotyledons	Moraceae	*Brosimum*	*Brosimum alicastrum*	GQ437000
Eudicotyledons	Moraceae	*Brosimum*	*Brosimum alicastrum*	GQ437001
Eudicotyledons	Moraceae	*Brosimum*	*Brosimum alicastrum*	GQ437002
Eudicotyledons	Moraceae	*Brosimum*	*Brosimum alicastrum*	GQ437003
Eudicotyledons	Moraceae	*Brosimum*	*Brosimum alicastrum*	GQ437004
Eudicotyledons	Moraceae	*Brosimum*	*Brosimum alicastrum*	GQ437005
Eudicotyledons	Moraceae	*Brosimum*	*Brosimum alicastrum*	GQ437006
Eudicotyledons	Moraceae	*Brosimum*	*Brosimum alicastrum*	GQ437007
Eudicotyledons	Moraceae	*Brosimum*	*Brosimum alicastrum*	GQ437008
Eudicotyledons	Moraceae	*Brosimum*	*Brosimum alicastrum*	GQ437009
Eudicotyledons	Moraceae	*Brosimum*	*Brosimum alicastrum*	GQ437010
Eudicotyledons	Moraceae	*Brosimum*	*Brosimum alicastrum*	GQ437011
Eudicotyledons	Moraceae	*Brosimum*	*Brosimum alicastrum*	GQ437012
Eudicotyledons	Moraceae	*Brosimum*	*Brosimum alicastrum*	GQ437013
Eudicotyledons	Moraceae	*Brosimum*	*Brosimum alicastrum*	GQ437014
Eudicotyledons	Moraceae	*Brosimum*	*Brosimum alicastrum*	GQ437015
Eudicotyledons	Moraceae	*Brosimum*	*Brosimum alicastrum*	GQ437016
Eudicotyledons	Moraceae	*Brosimum*	*Brosimum alicastrum*	GQ437017
Eudicotyledons	Moraceae	*Brosimum*	*Brosimum alicastrum*	GQ437018
Eudicotyledons	Moraceae	*Brosimum*	*Brosimum alicastrum*	GQ437019
Eudicotyledons	Moraceae	*Brosimum*	*Brosimum alicastrum*	GQ437020
Eudicotyledons	Moraceae	*Brosimum*	*Brosimum alicastrum*	GQ437021
Eudicotyledons	Moraceae	*Brosimum*	*Brosimum alicastrum*	GQ437022
Eudicotyledons	Moraceae	*Brosimum*	*Brosimum alicastrum*	GQ437023
Eudicotyledons	Moraceae	*Brosimum*	*Brosimum alicastrum*	GQ437024
Eudicotyledons	Moraceae	*Brosimum*	*Brosimum alicastrum*	GQ437025
Eudicotyledons	Moraceae	*Brosimum*	*Brosimum alicastrum*	GQ437026
Eudicotyledons	Moraceae	*Brosimum*	*Brosimum alicastrum*	GQ437027
Eudicotyledons	Moraceae	*Brosimum*	*Brosimum alicastrum*	GQ437028
Eudicotyledons	Moraceae	*Brosimum*	*Brosimum alicastrum*	GQ437029
Eudicotyledons	Moraceae	*Brosimum*	*Brosimum alicastrum*	GQ437030
Eudicotyledons	Moraceae	*Brosimum*	*Brosimum alicastrum*	GQ437031
Eudicotyledons	Moraceae	*Brosimum*	*Brosimum alicastrum*	GQ437032
Eudicotyledons	Moraceae	*Brosimum*	*Brosimum alicastrum*	GQ437033
Eudicotyledons	Moraceae	*Brosimum*	*Brosimum alicastrum*	GQ437034
Eudicotyledons	Moraceae	*Brosimum*	*Brosimum alicastrum*	GQ437035
Eudicotyledons	Moraceae	*Brosimum*	*Brosimum alicastrum*	GQ437036
Eudicotyledons	Moraceae	*Brosimum*	*Brosimum alicastrum*	GQ437037
Eudicotyledons	Moraceae	*Brosimum*	*Brosimum alicastrum*	GQ437038
Eudicotyledons	Moraceae	*Brosimum*	*Brosimum alicastrum*	GQ437039
Eudicotyledons	Moraceae	*Brosimum*	*Brosimum alicastrum*	GQ437040
Eudicotyledons	Moraceae	*Brosimum*	*Brosimum alicastrum*	GQ437041
Eudicotyledons	Moraceae	*Brosimum*	*Brosimum alicastrum*	GQ437042
Eudicotyledons	Moraceae	*Brosimum*	*Brosimum alicastrum*	GQ437043
Eudicotyledons	Moraceae	*Brosimum*	*Brosimum alicastrum*	GQ437044
Eudicotyledons	Moraceae	*Brosimum*	*Brosimum alicastrum*	GQ437045
Eudicotyledons	Moraceae	*Brosimum*	*Brosimum alicastrum*	GQ437046

Eudicotyledons	Moraceae	*Brosimum*	*Brosimum alicastrum*	GQ437047
Eudicotyledons	Moraceae	*Brosimum*	*Brosimum alicastrum*	GQ437048
Eudicotyledons	Moraceae	*Brosimum*	*Brosimum alicastrum*	GQ437049
Eudicotyledons	Moraceae	*Brosimum*	*Brosimum alicastrum*	GQ437050
Eudicotyledons	Moraceae	*Brosimum*	*Brosimum alicastrum*	GQ437051
Eudicotyledons	Moraceae	*Brosimum*	*Brosimum alicastrum*	GQ437052
Eudicotyledons	Moraceae	*Brosimum*	*Brosimum alicastrum*	GQ437053
Eudicotyledons	Moraceae	*Brosimum*	*Brosimum alicastrum*	GQ437054
Eudicotyledons	Moraceae	*Brosimum*	*Brosimum alicastrum*	GQ437055
Eudicotyledons	Moraceae	*Brosimum*	*Brosimum alicastrum*	GQ437056
Eudicotyledons	Moraceae	*Brosimum*	*Brosimum alicastrum*	GQ437057
Eudicotyledons	Moraceae	*Brosimum*	*Brosimum alicastrum*	GQ437058
Eudicotyledons	Moraceae	*Brosimum*	*Brosimum alicastrum*	GQ437059
Eudicotyledons	Moraceae	*Brosimum*	*Brosimum alicastrum*	GQ437060
Eudicotyledons	Moraceae	*Brosimum*	*Brosimum alicastrum*	GQ437061
Eudicotyledons	Moraceae	*Brosimum*	*Brosimum alicastrum*	GQ437062
Eudicotyledons	Moraceae	*Brosimum*	*Brosimum alicastrum*	GQ437063
Eudicotyledons	Moraceae	*Brosimum*	*Brosimum alicastrum*	GQ437064
Eudicotyledons	Moraceae	*Brosimum*	*Brosimum alicastrum*	GQ437065
Eudicotyledons	Moraceae	*Brosimum*	*Brosimum alicastrum*	GQ437066
Eudicotyledons	Moraceae	*Brosimum*	*Brosimum alicastrum*	GQ437067
Eudicotyledons	Moraceae	*Brosimum*	*Brosimum alicastrum*	GQ437068
Eudicotyledons	Moraceae	*Brosimum*	*Brosimum alicastrum*	GQ437069
Eudicotyledons	Moraceae	*Brosimum*	*Brosimum alicastrum*	GQ437070
Eudicotyledons	Moraceae	*Brosimum*	*Brosimum alicastrum*	GQ437071
Eudicotyledons	Moraceae	*Brosimum*	*Brosimum alicastrum*	GQ437072
Eudicotyledons	Moraceae	*Brosimum*	*Brosimum alicastrum*	GQ437073
Eudicotyledons	Moraceae	*Brosimum*	*Brosimum alicastrum*	GQ437074
Eudicotyledons	Moraceae	*Brosimum*	*Brosimum alicastrum*	GQ437075
Eudicotyledons	Moraceae	*Brosimum*	*Brosimum alicastrum*	GQ437076
Eudicotyledons	Moraceae	*Brosimum*	*Brosimum alicastrum*	GQ437077
Eudicotyledons	Moraceae	*Brosimum*	*Brosimum alicastrum*	GQ437078
Eudicotyledons	Moraceae	*Brosimum*	*Brosimum alicastrum*	GQ437079
Eudicotyledons	Moraceae	*Brosimum*	*Brosimum alicastrum*	GQ437080
Eudicotyledons	Moraceae	*Brosimum*	*Brosimum alicastrum*	GQ437081
Eudicotyledons	Moraceae	*Brosimum*	*Brosimum alicastrum*	GQ437082
Eudicotyledons	Moraceae	*Brosimum*	*Brosimum alicastrum*	GQ437083
Eudicotyledons	Moraceae	*Brosimum*	*Brosimum alicastrum*	GQ437084
Eudicotyledons	Moraceae	*Brosimum*	*Brosimum alicastrum*	GQ437085
Eudicotyledons	Moraceae	*Brosimum*	*Brosimum alicastrum*	GQ437086
Eudicotyledons	Moraceae	*Brosimum*	*Brosimum alicastrum*	GQ437087
Eudicotyledons	Moraceae	*Brosimum*	*Brosimum alicastrum*	GQ437088
Eudicotyledons	Moraceae	*Brosimum*	*Brosimum alicastrum*	GQ437089
Eudicotyledons	Moraceae	*Brosimum*	*Brosimum alicastrum*	GQ437090
Eudicotyledons	Moraceae	*Brosimum*	*Brosimum alicastrum*	GQ437091
Eudicotyledons	Moraceae	*Brosimum*	*Brosimum alicastrum*	GQ437092
Eudicotyledons	Moraceae	*Brosimum*	*Brosimum alicastrum*	GQ437093
Eudicotyledons	Moraceae	*Brosimum*	*Brosimum alicastrum*	GQ437094
Eudicotyledons	Moraceae	*Brosimum*	*Brosimum alicastrum*	GQ437095
Eudicotyledons	Moraceae	*Brosimum*	*Brosimum alicastrum*	GQ437096
Eudicotyledons	Moraceae	*Brosimum*	*Brosimum alicastrum*	GQ437097
Eudicotyledons	Moraceae	*Brosimum*	*Brosimum alicastrum*	GQ437098
Eudicotyledons	Moraceae	*Brosimum*	*Brosimum alicastrum*	GQ437099

Eudicotyledons	Moraceae	*Brosimum*	*Brosimum alicastrum*	GQ437100
Eudicotyledons	Moraceae	*Brosimum*	*Brosimum alicastrum*	GQ437101
Eudicotyledons	Moraceae	*Brosimum*	*Brosimum alicastrum*	GQ437102
Eudicotyledons	Moraceae	*Brosimum*	*Brosimum alicastrum*	GQ437103
Eudicotyledons	Moraceae	*Brosimum*	*Brosimum alicastrum*	GQ437104
Eudicotyledons	Moraceae	*Brosimum*	*Brosimum alicastrum*	GQ437105
Eudicotyledons	Moraceae	*Brosimum*	*Brosimum alicastrum*	GQ437106
Eudicotyledons	Moraceae	*Brosimum*	*Brosimum alicastrum*	GQ437107
Eudicotyledons	Moraceae	*Brosimum*	*Brosimum alicastrum*	GQ437108
Eudicotyledons	Moraceae	*Brosimum*	*Brosimum alicastrum*	GQ437109
Eudicotyledons	Moraceae	*Brosimum*	*Brosimum alicastrum*	GQ437110
Eudicotyledons	Moraceae	*Brosimum*	*Brosimum alicastrum*	GQ437111
Eudicotyledons	Moraceae	*Brosimum*	*Brosimum alicastrum*	GQ437112
Eudicotyledons	Moraceae	*Brosimum*	*Brosimum alicastrum*	GQ437113
Eudicotyledons	Moraceae	*Brosimum*	*Brosimum alicastrum*	GQ437114
Eudicotyledons	Moraceae	*Brosimum*	*Brosimum alicastrum*	GQ437115
Eudicotyledons	Moraceae	*Brosimum*	*Brosimum alicastrum*	GQ437116
Eudicotyledons	Moraceae	*Brosimum*	*Brosimum alicastrum*	GQ437117
Eudicotyledons	Moraceae	*Brosimum*	*Brosimum alicastrum*	GQ437118
Eudicotyledons	Moraceae	*Brosimum*	*Brosimum alicastrum*	GQ437119
Eudicotyledons	Moraceae	*Brosimum*	*Brosimum alicastrum*	GQ437120
Eudicotyledons	Moraceae	*Brosimum*	*Brosimum alicastrum*	GQ437121
Eudicotyledons	Moraceae	*Brosimum*	*Brosimum alicastrum*	GQ437122
Eudicotyledons	Moraceae	*Brosimum*	*Brosimum alicastrum*	GQ437123
Eudicotyledons	Moraceae	*Brosimum*	*Brosimum alicastrum*	GQ437124
Eudicotyledons	Moraceae	*Brosimum*	*Brosimum alicastrum*	GQ437125
Eudicotyledons	Moraceae	*Brosimum*	*Brosimum alicastrum*	GQ437126
Eudicotyledons	Moraceae	*Brosimum*	*Brosimum alicastrum*	GQ437127
Eudicotyledons	Moraceae	*Brosimum*	*Brosimum alicastrum*	GQ437128
Eudicotyledons	Moraceae	*Brosimum*	*Brosimum alicastrum*	GQ437129
Eudicotyledons	Moraceae	*Brosimum*	*Brosimum alicastrum*	GQ437130
Eudicotyledons	Moraceae	*Brosimum*	*Brosimum alicastrum*	GQ437131
Eudicotyledons	Moraceae	*Brosimum*	*Brosimum alicastrum*	GQ437132
Eudicotyledons	Moraceae	*Brosimum*	*Brosimum alicastrum*	GQ437133
Eudicotyledons	Moraceae	*Brosimum*	*Brosimum alicastrum*	GQ437134
Eudicotyledons	Moraceae	*Brosimum*	*Brosimum alicastrum*	GQ437135
Eudicotyledons	Moraceae	*Brosimum*	*Brosimum alicastrum*	GQ437136
Eudicotyledons	Moraceae	*Brosimum*	*Brosimum alicastrum*	GQ437137
Eudicotyledons	Moraceae	*Brosimum*	*Brosimum alicastrum*	GQ437138
Eudicotyledons	Moraceae	*Brosimum*	*Brosimum alicastrum*	GQ437139
Eudicotyledons	Moraceae	*Brosimum*	*Brosimum alicastrum*	GQ437140
Eudicotyledons	Moraceae	*Brosimum*	*Brosimum alicastrum*	GQ437141
Eudicotyledons	Moraceae	*Brosimum*	*Brosimum alicastrum*	GQ437142
Eudicotyledons	Moraceae	*Brosimum*	*Brosimum alicastrum*	GQ437143
Eudicotyledons	Moraceae	*Brosimum*	*Brosimum alicastrum*	GQ437144
Eudicotyledons	Moraceae	*Brosimum*	*Brosimum alicastrum*	GQ437145
Eudicotyledons	Moraceae	*Brosimum*	*Brosimum alicastrum*	GQ437146
Eudicotyledons	Moraceae	*Brosimum*	*Brosimum alicastrum*	GQ437147
Eudicotyledons	Moraceae	*Brosimum*	*Brosimum alicastrum*	GQ437148
Eudicotyledons	Moraceae	*Brosimum*	*Brosimum alicastrum*	GQ437149
Eudicotyledons	Moraceae	*Brosimum*	*Brosimum alicastrum*	GQ437150
Eudicotyledons	Moraceae	*Brosimum*	*Brosimum alicastrum*	GQ437151
Eudicotyledons	Moraceae	*Brosimum*	*Brosimum alicastrum*	GQ437152

Eudicotyledons	Moraceae	*Brosimum*	*Brosimum alicastrum*	GQ437153
Eudicotyledons	Moraceae	*Brosimum*	*Brosimum alicastrum*	GQ437154
Eudicotyledons	Moraceae	*Brosimum*	*Brosimum alicastrum*	GQ437155
Eudicotyledons	Moraceae	*Brosimum*	*Brosimum alicastrum*	GQ437156
Eudicotyledons	Moraceae	*Brosimum*	*Brosimum alicastrum*	GQ437157
Eudicotyledons	Moraceae	*Brosimum*	*Brosimum alicastrum*	GQ437158
Eudicotyledons	Moraceae	*Brosimum*	*Brosimum alicastrum*	GQ437159
Eudicotyledons	Moraceae	*Brosimum*	*Brosimum alicastrum*	GQ437160
Eudicotyledons	Moraceae	*Brosimum*	*Brosimum alicastrum*	GQ437161
Eudicotyledons	Moraceae	*Brosimum*	*Brosimum alicastrum*	GQ437162
Eudicotyledons	Moraceae	*Brosimum*	*Brosimum alicastrum*	GQ437163
Eudicotyledons	Moraceae	*Brosimum*	*Brosimum alicastrum*	GQ437164
Eudicotyledons	Moraceae	*Brosimum*	*Brosimum alicastrum*	GQ437165
Eudicotyledons	Moraceae	*Brosimum*	*Brosimum alicastrum*	GQ437166
Eudicotyledons	Moraceae	*Brosimum*	*Brosimum alicastrum*	GQ437167
Eudicotyledons	Moraceae	*Brosimum*	*Brosimum alicastrum*	GQ437168
Eudicotyledons	Moraceae	*Brosimum*	*Brosimum alicastrum*	GQ437169
Eudicotyledons	Moraceae	*Brosimum*	*Brosimum alicastrum*	GQ437170
Eudicotyledons	Moraceae	*Brosimum*	*Brosimum alicastrum*	GQ437171
Eudicotyledons	Moraceae	*Brosimum*	*Brosimum alicastrum*	GQ437172
Eudicotyledons	Moraceae	*Brosimum*	*Brosimum alicastrum*	GQ437173
Eudicotyledons	Moraceae	*Brosimum*	*Brosimum alicastrum*	GQ437174
Eudicotyledons	Moraceae	*Brosimum*	*Brosimum alicastrum*	GQ437175
Eudicotyledons	Moraceae	*Brosimum*	*Brosimum alicastrum*	GQ437176
Eudicotyledons	Moraceae	*Brosimum*	*Brosimum alicastrum*	GQ437177
Eudicotyledons	Moraceae	*Brosimum*	*Brosimum alicastrum*	GQ437178
Eudicotyledons	Moraceae	*Brosimum*	*Brosimum alicastrum*	GQ437179
Eudicotyledons	Moraceae	*Brosimum*	*Brosimum alicastrum*	GQ437180
Eudicotyledons	Moraceae	*Brosimum*	*Brosimum alicastrum*	GQ437181
Eudicotyledons	Moraceae	*Brosimum*	*Brosimum alicastrum*	GQ437182
Eudicotyledons	Moraceae	*Brosimum*	*Brosimum alicastrum*	GQ437183
Eudicotyledons	Moraceae	*Brosimum*	*Brosimum alicastrum*	GQ437184
Eudicotyledons	Moraceae	*Brosimum*	*Brosimum alicastrum*	GQ437185
Eudicotyledons	Moraceae	*Brosimum*	*Brosimum alicastrum*	GQ437186
Eudicotyledons	Moraceae	*Brosimum*	*Brosimum alicastrum*	GQ437187
Eudicotyledons	Moraceae	*Brosimum*	*Brosimum alicastrum*	GQ437188
Eudicotyledons	Moraceae	*Brosimum*	*Brosimum alicastrum*	GQ437189
Eudicotyledons	Moraceae	*Brosimum*	*Brosimum alicastrum*	GQ437190
Eudicotyledons	Moraceae	*Brosimum*	*Brosimum alicastrum*	GQ437191
Eudicotyledons	Moraceae	*Brosimum*	*Brosimum alicastrum*	GQ437192
Eudicotyledons	Moraceae	*Brosimum*	*Brosimum alicastrum*	GQ437193
Eudicotyledons	Moraceae	*Brosimum*	*Brosimum alicastrum*	GQ437194
Eudicotyledons	Moraceae	*Brosimum*	*Brosimum alicastrum*	GQ437195
Eudicotyledons	Moraceae	*Brosimum*	*Brosimum alicastrum*	GQ437196
Eudicotyledons	Moraceae	*Brosimum*	*Brosimum alicastrum*	GQ437197
Eudicotyledons	Moraceae	*Brosimum*	*Brosimum alicastrum*	GQ437198
Eudicotyledons	Moraceae	*Brosimum*	*Brosimum alicastrum*	GQ437199
Eudicotyledons	Moraceae	*Brosimum*	*Brosimum alicastrum*	GQ437200
Eudicotyledons	Moraceae	*Brosimum*	*Brosimum alicastrum*	GQ437201
Eudicotyledons	Moraceae	*Brosimum*	*Brosimum alicastrum*	GQ437202
Eudicotyledons	Moraceae	*Brosimum*	*Brosimum alicastrum*	GQ437203
Eudicotyledons	Moraceae	*Brosimum*	*Brosimum alicastrum*	GQ437204
Eudicotyledons	Moraceae	*Brosimum*	*Brosimum alicastrum*	GQ437205

Eudicotyledons	Moraceae	*Brosimum*	*Brosimum alicastrum*	GQ437206
Eudicotyledons	Moraceae	*Brosimum*	*Brosimum alicastrum*	GQ982162
Eudicotyledons	Lamiaceae	*Isodon*	*Isodon lophanthoides*	JN045037
Eudicotyledons	Lamiaceae	*Isodon*	*Isodon lophanthoides*	JN045038
Eudicotyledons	Loranthaceae	*Macrosolen*	*Macrosolen cochinchinensis*	HQ317809
Eudicotyledons	Loranthaceae	*Macrosolen*	*Macrosolen cochinchinensis*	HQ317810
Eudicotyledons	Loranthaceae	*Macrosolen*	*Macrosolen cochinchinensis*	HQ317811
Eudicotyledons	Loranthaceae	*Macrosolen*	*Macrosolen cochinchinensis*	JN687575
Eudicotyledons	Loranthaceae	*Scurrula*	*Scurrula chingii*	HQ317814
Eudicotyledons	Loranthaceae	*Scurrula*	*Scurrula chingii*	HQ317815
Eudicotyledons	Loranthaceae	*Taxillus*	*Taxillus chinensis*	HQ317821
Eudicotyledons	Loranthaceae	*Taxillus*	*Taxillus chinensis*	HQ317822
Eudicotyledons	Loranthaceae	*Taxillus*	*Taxillus chinensis*	HQ317823
Eudicotyledons	Loranthaceae	*Taxillus*	*Taxillus chinensis*	HQ317824
Eudicotyledons	Loranthaceae	*Taxillus*	*Taxillus chinensis*	HQ317825
Eudicotyledons	Loranthaceae	*Taxillus*	*Taxillus chinensis*	HQ317826
Eudicotyledons	Loranthaceae	*Taxillus*	*Taxillus chinensis*	HQ317827
Eudicotyledons	Loranthaceae	*Taxillus*	*Taxillus chinensis*	HQ317828
Eudicotyledons	Loranthaceae	*Taxillus*	*Taxillus chinensis*	HQ317829
Eudicotyledons	Loranthaceae	*Taxillus*	*Taxillus chinensis*	HQ317830
Eudicotyledons	Loranthaceae	*Taxillus*	*Taxillus chinensis*	HQ317831
Eudicotyledons	Loranthaceae	*Taxillus*	*Taxillus chinensis*	GQ435467
Eudicotyledons	Malvaceae	*Cristaria*	*Cristaria aspera*	AY371674
Eudicotyledons	Malvaceae	*Cristaria*	*Cristaria aspera*	AY371685
Eudicotyledons	Malvaceae	*Cristaria*	*Cristaria multifida*	AY371681
Eudicotyledons	Malvaceae	*Cristaria*	*Cristaria multifida*	AY371684
Eudicotyledons	Lamiaceae	*Clinopodium*	*Clinopodium ashei*	DQ667348
Eudicotyledons	Lamiaceae	*Clinopodium*	*Clinopodium ashei*	AY943551
Eudicotyledons	Lamiaceae	*Clinopodium*	*Clinopodium ashei*	AY943552
Eudicotyledons	Lamiaceae	*Clinopodium*	*Clinopodium ashei*	AY943553
Eudicotyledons	Lamiaceae	*Clinopodium*	*Clinopodium ashei*	AY943554
Eudicotyledons	Ranunculaceae	*Coptis*	*Coptis teeta*	JN862875
Eudicotyledons	Ranunculaceae	*Coptis*	*Coptis teeta*	JN862876
Eudicotyledons	Ranunculaceae	*Coptis*	*Coptis teeta*	JN862877
Eudicotyledons	Ranunculaceae	*Coptis*	*Coptis teeta*	AB163743
Eudicotyledons	Ranunculaceae	*Coptis*	*Coptis teeta*	HQ829539
Eudicotyledons	Ranunculaceae	*Coptis*	*Coptis teeta*	HQ829540
Eudicotyledons	Ranunculaceae	*Coptis*	*Coptis teeta*	HQ829541
Eudicotyledons	Ranunculaceae	*Coptis*	*Coptis teeta*	HQ829542
Eudicotyledons	Ranunculaceae	*Coptis*	*Coptis chinensis*	GQ435206
Eudicotyledons	Ranunculaceae	*Coptis*	*Coptis chinensis*	GQ435207
Eudicotyledons	Ranunculaceae	*Coptis*	*Coptis chinensis*	JN862866
Eudicotyledons	Ranunculaceae	*Coptis*	*Coptis chinensis*	JN862867
Eudicotyledons	Ranunculaceae	*Coptis*	*Coptis chinensis*	JN862868
Eudicotyledons	Ranunculaceae	*Coptis*	*Coptis chinensis*	JN862869
Eudicotyledons	Ranunculaceae	*Coptis*	*Coptis chinensis*	JN862870
Eudicotyledons	Ranunculaceae	*Coptis*	*Coptis chinensis*	JN862872
Eudicotyledons	Ranunculaceae	*Coptis*	*Coptis chinensis*	AB163745
Eudicotyledons	Ranunculaceae	*Coptis*	*Coptis chinensis*	HQ829527
Eudicotyledons	Ranunculaceae	*Coptis*	*Coptis chinensis*	HQ829528
Eudicotyledons	Ranunculaceae	*Coptis*	*Coptis chinensis*	HQ829529
Eudicotyledons	Lamiaceae	*Salvia*	*Salvia fruticosa*	EU627579
Eudicotyledons	Lamiaceae	*Salvia*	*Salvia fruticosa*	FJ513109

Eudicotyledons	Lamiaceae	*Salvia*	*Salvia fruticosa*	HQ902840
Eudicotyledons	Lamiaceae	*Salvia*	*Salvia fruticosa*	HQ902859
Eudicotyledons	Lamiaceae	*Salvia*	*Salvia greatae*	DQ667339
Eudicotyledons	Lamiaceae	*Salvia*	*Salvia greatae*	HQ418921
Eudicotyledons	Tamaricaceae	*Myricaria*	*Myricaria germanica*	EU914131
Eudicotyledons	Tamaricaceae	*Myricaria*	*Myricaria germanica*	EU914132
Eudicotyledons	Tamaricaceae	*Myricaria*	*Myricaria germanica*	HQ680684
Eudicotyledons	Tamaricaceae	*Myricaria*	*Myricaria germanica*	HQ680687
Eudicotyledons	Lamiaceae	*Mentha*	*Mentha arvensis*	DQ667410
Eudicotyledons	Lamiaceae	*Mentha*	*Mentha arvensis*	HQ596770
Eudicotyledons	Lamiaceae	*Mentha*	*Mentha arvensis*	AY643665
Eudicotyledons	Lamiaceae	*Mentha*	*Mentha canadensis*	GQ434942
Eudicotyledons	Lamiaceae	*Mentha*	*Mentha canadensis*	JN406990
Eudicotyledons	Lamiaceae	*Mentha*	*Mentha canadensis*	JN406991
Eudicotyledons	Lamiaceae	*Mentha*	*Mentha canadensis*	JN406992
Eudicotyledons	Lamiaceae	*Mentha*	*Mentha canadensis*	JN406993
Eudicotyledons	Lamiaceae	*Mentha*	*Mentha canadensis*	AY643663
Eudicotyledons	Lamiaceae	*Mentha*	*Mentha canadensis*	AY643664
Eudicotyledons	Lamiaceae	*Mentha*	*Mentha canadensis*	HM590119
Eudicotyledons	Lamiaceae	*Phlomis*	*Phlomis lychnitis*	AY792624
Eudicotyledons	Lamiaceae	*Phlomis*	*Phlomis lychnitis*	AY792625
Eudicotyledons	Lamiaceae	*Phlomis*	*Phlomis lychnitis*	AY792626
Eudicotyledons	Lamiaceae	*Phlomis*	*Phlomis lychnitis*	AY792627
Eudicotyledons	Lamiaceae	*Phlomis*	*Phlomis lychnitis*	AY792628
Eudicotyledons	Lamiaceae	*Phlomis*	*Phlomis lychnitis*	AY792629
Eudicotyledons	Lamiaceae	*Phlomis*	*Phlomis lychnitis*	AY792630
Eudicotyledons	Lamiaceae	*Phlomis*	*Phlomis lychnitis*	AY792631
Eudicotyledons	Lamiaceae	*Phlomis*	*Phlomis lychnitis*	AY792632
Eudicotyledons	Lamiaceae	*Phlomis*	*Phlomis lychnitis*	AY792633
Eudicotyledons	Lamiaceae	*Phlomis*	*Phlomis lychnitis*	AY792634
Eudicotyledons	Lamiaceae	*Phlomis*	*Phlomis lychnitis*	AY792635
Eudicotyledons	Lamiaceae	*Phlomis*	*Phlomis lychnitis*	AY792636
Eudicotyledons	Lamiaceae	*Phlomis*	*Phlomis lychnitis*	AY792637
Eudicotyledons	Lamiaceae	*Phlomis*	*Phlomis lychnitis*	AY792638
Eudicotyledons	Lamiaceae	*Phlomis*	*Phlomis lychnitis*	AY792639
Eudicotyledons	Lamiaceae	*Phlomis*	*Phlomis lychnitis*	AY792640
Eudicotyledons	Lamiaceae	*Phlomis*	*Phlomis lychnitis*	AY792641
Eudicotyledons	Lamiaceae	*Phlomis*	*Phlomis lychnitis*	AY792642
Eudicotyledons	Lamiaceae	*Phlomis*	*Phlomis lychnitis*	AY792643
Eudicotyledons	Lamiaceae	*Phlomis*	*Phlomis lychnitis*	AY792644
Eudicotyledons	Lamiaceae	*Phlomis*	*Phlomis lychnitis*	AY792645
Eudicotyledons	Lamiaceae	*Phlomis*	*Phlomis lychnitis*	AY792646
Eudicotyledons	Lamiaceae	*Conradina*	*Conradina brevifolia*	AY943531
Eudicotyledons	Lamiaceae	*Conradina*	*Conradina brevifolia*	AY943532
Eudicotyledons	Lamiaceae	*Conradina*	*Conradina brevifolia*	AY943533
Eudicotyledons	Lamiaceae	*Conradina*	*Conradina brevifolia*	AY943534
Eudicotyledons	Lamiaceae	*Conradina*	*Conradina brevifolia*	AY943535
Eudicotyledons	Lamiaceae	*Conradina*	*Conradina canescens*	DQ667349
Eudicotyledons	Lamiaceae	*Conradina*	*Conradina canescens*	AY943536
Eudicotyledons	Lamiaceae	*Conradina*	*Conradina canescens*	AY943537
Eudicotyledons	Lamiaceae	*Conradina*	*Conradina canescens*	AY943538
Eudicotyledons	Lamiaceae	*Conradina*	*Conradina grandiflora*	AY943544
Eudicotyledons	Lamiaceae	*Conradina*	*Conradina grandiflora*	AY943545

Eudicotyledons	Lamiaceae	*Conradina*	*Conradina grandiflora*	AY943546
Eudicotyledons	Lamiaceae	*Conradina*	*Conradina grandiflora*	AY943547
Eudicotyledons	Lamiaceae	*Conradina*	*Conradina sp. Edwards et al. 133*	AY943548
Eudicotyledons	Lamiaceae	*Conradina*	*Conradina sp. Edwards et al. 133*	AY943549
Eudicotyledons	Lamiaceae	*Salvia*	*Salvia cacaliifolia*	DQ667367
Eudicotyledons	Lamiaceae	*Salvia*	*Salvia cacaliifolia*	HQ418903
Eudicotyledons	Lamiaceae	*Salvia*	*Salvia patens*	DQ667361
Eudicotyledons	Lamiaceae	*Salvia*	*Salvia patens*	HQ418937
Eudicotyledons	Lamiaceae	*Salvia*	*Salvia prunelloides*	DQ667371
Eudicotyledons	Lamiaceae	*Salvia*	*Salvia prunelloides*	HQ418938
Eudicotyledons	Rutaceae	*Citrus*	*Citrus limetta*	JN315368
Eudicotyledons	Rutaceae	*Citrus*	*Citrus limetta*	JN315369
Eudicotyledons	Lamiaceae	*Isodon*	*Isodon japonicus*	AB446238
Eudicotyledons	Lamiaceae	*Isodon*	*Isodon japonicus*	AB446239
Eudicotyledons	Lamiaceae	*Isodon*	*Isodon japonicus*	AB446240
Eudicotyledons	Lamiaceae	*Isodon*	*Isodon japonicus*	AB556804
Eudicotyledons	Lamiaceae	*Isodon*	*Isodon japonicus*	AB556815
Eudicotyledons	Lamiaceae	*Isodon*	*Isodon japonicus*	FJ513115
Eudicotyledons	Lamiaceae	*Isodon*	*Isodon trichocarpus*	AB556805
Eudicotyledons	Lamiaceae	*Isodon*	*Isodon trichocarpus*	AB556810
Eudicotyledons	Lamiaceae	*Clinopodium*	*Clinopodium chinense*	EU590865
Eudicotyledons	Lamiaceae	*Clinopodium*	*Clinopodium chinense*	FJ513089
Eudicotyledons	Lamiaceae	*Isodon*	*Isodon longitubus*	AB446204
Eudicotyledons	Lamiaceae	*Isodon*	*Isodon longitubus*	AB446205
Eudicotyledons	Lamiaceae	*Isodon*	*Isodon longitubus*	AB446206
Eudicotyledons	Lamiaceae	*Isodon*	*Isodon longitubus*	AB446207
Eudicotyledons	Lamiaceae	*Isodon*	*Isodon longitubus*	AB446208
Eudicotyledons	Lamiaceae	*Isodon*	*Isodon longitubus*	AB446209
Eudicotyledons	Lamiaceae	*Isodon*	*Isodon longitubus*	AB446210
Eudicotyledons	Lamiaceae	*Isodon*	*Isodon longitubus*	AB446211
Eudicotyledons	Lamiaceae	*Isodon*	*Isodon longitubus*	AB446212
Eudicotyledons	Lamiaceae	*Isodon*	*Isodon longitubus*	AB446213
Eudicotyledons	Lamiaceae	*Isodon*	*Isodon longitubus*	AB446214
Eudicotyledons	Lamiaceae	*Isodon*	*Isodon longitubus*	AB446215
Eudicotyledons	Lamiaceae	*Isodon*	*Isodon longitubus*	AB446216
Eudicotyledons	Lamiaceae	*Isodon*	*Isodon longitubus*	AB446217
Eudicotyledons	Lamiaceae	*Isodon*	*Isodon longitubus*	AB446218
Eudicotyledons	Lamiaceae	*Isodon*	*Isodon longitubus*	AB446219
Eudicotyledons	Lamiaceae	*Isodon*	*Isodon longitubus*	AB446220
Eudicotyledons	Lamiaceae	*Isodon*	*Isodon longitubus*	AB446221
Eudicotyledons	Lamiaceae	*Isodon*	*Isodon longitubus*	AB446222
Eudicotyledons	Lamiaceae	*Isodon*	*Isodon longitubus*	AB556814
Eudicotyledons	Lamiaceae	*Isodon*	*Isodon longitubus*	AB556817
Eudicotyledons	Meliaceae	*Carapa*	*Carapa procera*	GQ428752
Eudicotyledons	Meliaceae	*Carapa*	*Carapa procera*	FJ039000
Eudicotyledons	Meliaceae	*Carapa*	*Carapa procera*	FJ039001
Eudicotyledons	Meliaceae	*Carapa*	*Carapa procera*	JF288754
Eudicotyledons	Rosaceae	*Potentilla*	*Potentilla argentea*	GQ384965
Eudicotyledons	Rosaceae	*Potentilla*	*Potentilla argentea*	GQ384977
Eudicotyledons	Rosaceae	*Potentilla*	*Potentilla argentea*	GQ384986
Eudicotyledons	Rosaceae	*Potentilla*	*Potentilla argentea*	HM776523
Eudicotyledons	Rosaceae	*Potentilla*	*Potentilla argentea*	HM776524
Eudicotyledons	Rosaceae	*Potentilla*	*Potentilla argentea*	HM776525

Eudicotyledons	Rosaceae	*Potentilla*	*Potentilla argentea*	HM776526
Eudicotyledons	Rosaceae	*Potentilla*	*Potentilla argentea*	HM776527
Eudicotyledons	Rosaceae	*Potentilla*	*Potentilla argentea*	HM776528
Eudicotyledons	Rosaceae	*Potentilla*	*Potentilla argentea*	HM776529
Eudicotyledons	Rosaceae	*Potentilla*	*Potentilla argentea*	HM776530
Eudicotyledons	Rosaceae	*Potentilla*	*Potentilla argentea*	HM776531
Eudicotyledons	Rosaceae	*Potentilla*	*Potentilla argentea*	HM776532
Eudicotyledons	Rosaceae	*Potentilla*	*Potentilla argentea*	HM776533
Eudicotyledons	Rosaceae	*Potentilla*	*Potentilla argentea*	HM776534
Eudicotyledons	Rosaceae	*Potentilla*	*Potentilla argentea*	HM776535
Eudicotyledons	Rosaceae	*Potentilla*	*Potentilla argentea*	HM776536
Eudicotyledons	Rosaceae	*Potentilla*	*Potentilla argentea*	HM776537
Eudicotyledons	Rosaceae	*Potentilla*	*Potentilla argentea*	HM776538
Eudicotyledons	Rosaceae	*Potentilla*	*Potentilla argentea*	HM776539
Eudicotyledons	Rosaceae	*Potentilla*	*Potentilla argentea*	HM776540
Eudicotyledons	Rosaceae	*Potentilla*	*Potentilla argentea*	HM776541
Eudicotyledons	Rosaceae	*Potentilla*	*Potentilla argentea*	HM776542
Eudicotyledons	Rosaceae	*Potentilla*	*Potentilla argentea*	HM776543
Eudicotyledons	Rosaceae	*Potentilla*	*Potentilla argentea*	HM776544
Eudicotyledons	Rosaceae	*Potentilla*	*Potentilla argentea*	HM776545
Eudicotyledons	Rosaceae	*Potentilla*	*Potentilla argentea*	HM776546
Eudicotyledons	Rosaceae	*Potentilla*	*Potentilla argentea*	HM776547
Eudicotyledons	Rosaceae	*Potentilla*	*Potentilla argentea*	HM776548
Eudicotyledons	Rosaceae	*Potentilla*	*Potentilla argentea*	HM776549
Eudicotyledons	Rosaceae	*Potentilla*	*Potentilla argentea*	HM776550
Eudicotyledons	Rosaceae	*Potentilla*	*Potentilla argentea*	HM776551
Eudicotyledons	Rosaceae	*Potentilla*	*Potentilla argentea*	HM776552
Eudicotyledons	Rosaceae	*Potentilla*	*Potentilla argentea*	HM776553
Eudicotyledons	Rosaceae	*Potentilla*	*Potentilla argentea*	HM776554
Eudicotyledons	Rosaceae	*Potentilla*	*Potentilla argentea*	HM776555
Eudicotyledons	Rosaceae	*Potentilla*	*Potentilla argentea*	HM776556
Eudicotyledons	Rosaceae	*Potentilla*	*Potentilla argentea*	HM776557
Eudicotyledons	Rosaceae	*Potentilla*	*Potentilla argentea*	HM776558
Eudicotyledons	Rosaceae	*Potentilla*	*Potentilla argentea*	HM776559
Eudicotyledons	Rosaceae	*Potentilla*	*Potentilla argentea*	HM776560
Eudicotyledons	Rosaceae	*Potentilla*	*Potentilla argentea*	HM776561
Eudicotyledons	Rosaceae	*Potentilla*	*Potentilla argentea*	HM776562
Eudicotyledons	Rosaceae	*Potentilla*	*Potentilla argentea*	HM776563
Eudicotyledons	Rosaceae	*Potentilla*	*Potentilla argentea*	HM776564
Eudicotyledons	Rosaceae	*Potentilla*	*Potentilla argentea*	HM776565
Eudicotyledons	Rosaceae	*Potentilla*	*Potentilla argentea*	HM776566
Eudicotyledons	Rosaceae	*Potentilla*	*Potentilla argentea*	HM776567
Eudicotyledons	Rosaceae	*Potentilla*	*Potentilla argentea*	HM776568
Eudicotyledons	Rosaceae	*Potentilla*	*Potentilla argentea*	HM776573
Eudicotyledons	Rosaceae	*Potentilla*	*Potentilla argentea*	HM776574
Eudicotyledons	Rosaceae	*Potentilla*	*Potentilla argentea*	HM776575
Eudicotyledons	Rosaceae	*Potentilla*	*Potentilla velutina*	GQ384966
Eudicotyledons	Rosaceae	*Potentilla*	*Potentilla velutina*	GQ384969
Eudicotyledons	Onagraceae	*Chamerion*	*Chamerion conspersum*	JN044478
Eudicotyledons	Onagraceae	*Chamerion*	*Chamerion conspersum*	JN129844
Eudicotyledons	Brassicaceae	*Capsella*	*Capsella bursa-pastoris*	FJ395502
Eudicotyledons	Brassicaceae	*Capsella*	*Capsella bursa-pastoris*	HQ596620
Eudicotyledons	Brassicaceae	*Capsella*	*Capsella bursa-pastoris*	FR822344

Eudicotyledons	Brassicaceae	*Capsella*	*Capsella bursa-pastoris*	FR822345
Eudicotyledons	Brassicaceae	*Capsella*	*Capsella bursa-pastoris*	FR822346
Eudicotyledons	Brassicaceae	*Capsella*	*Capsella bursa-pastoris*	FJ493269
Eudicotyledons	Solanaceae	*Solanum*	*Solanum melongena*	HM016405
Eudicotyledons	Solanaceae	*Solanum*	*Solanum melongena*	HM016406
Eudicotyledons	Solanaceae	*Solanum*	*Solanum melongena*	HM016407
Eudicotyledons	Solanaceae	*Solanum*	*Solanum melongena*	HM016408
Eudicotyledons	Solanaceae	*Solanum*	*Solanum melongena*	HM016409
Eudicotyledons	Solanaceae	*Solanum*	*Solanum melongena*	HM016410
Eudicotyledons	Fabaceae	*Canavalia*	*Canavalia cathartica*	GU396816
Eudicotyledons	Fabaceae	*Canavalia*	*Canavalia cathartica*	HQ707430
Eudicotyledons	Fabaceae	*Canavalia*	*Canavalia cathartica*	HQ707432
Eudicotyledons	Rosaceae	*Chaenomeles*	*Chaenomeles cathayensis*	JQ390648
Eudicotyledons	Rosaceae	*Chaenomeles*	*Chaenomeles cathayensis*	JQ390652
Eudicotyledons	Caryophyllaceae	*Silene*	*Silene latifolia*	EF091495
Eudicotyledons	Caryophyllaceae	*Silene*	*Silene latifolia*	EF091496
Eudicotyledons	Caryophyllaceae	*Silene*	*Silene latifolia*	EF091497
Eudicotyledons	Caryophyllaceae	*Silene*	*Silene latifolia*	EF091498
Eudicotyledons	Caryophyllaceae	*Silene*	*Silene latifolia*	EF091499
Eudicotyledons	Caryophyllaceae	*Silene*	*Silene latifolia*	EF091500
Eudicotyledons	Caryophyllaceae	*Silene*	*Silene latifolia*	EF091501
Eudicotyledons	Caryophyllaceae	*Silene*	*Silene latifolia*	EF091502
Eudicotyledons	Caryophyllaceae	*Silene*	*Silene latifolia*	EF091503
Eudicotyledons	Caryophyllaceae	*Silene*	*Silene latifolia*	EF091504
Eudicotyledons	Caryophyllaceae	*Silene*	*Silene latifolia*	EF091505
Eudicotyledons	Caryophyllaceae	*Silene*	*Silene latifolia*	EF091506
Eudicotyledons	Caryophyllaceae	*Silene*	*Silene latifolia*	EF091507
Eudicotyledons	Caryophyllaceae	*Silene*	*Silene latifolia*	EF091508
Eudicotyledons	Caryophyllaceae	*Silene*	*Silene latifolia*	EF091509
Eudicotyledons	Caryophyllaceae	*Silene*	*Silene latifolia*	EF091510
Eudicotyledons	Caryophyllaceae	*Silene*	*Silene latifolia*	EF091511
Eudicotyledons	Caryophyllaceae	*Silene*	*Silene latifolia*	EF091512
Eudicotyledons	Caryophyllaceae	*Silene*	*Silene latifolia*	EF091513
Eudicotyledons	Caryophyllaceae	*Silene*	*Silene latifolia*	EF091514
Eudicotyledons	Caryophyllaceae	*Silene*	*Silene latifolia*	EF091515
Eudicotyledons	Caryophyllaceae	*Silene*	*Silene latifolia*	EF091516
Eudicotyledons	Caryophyllaceae	*Silene*	*Silene latifolia*	EF091517
Eudicotyledons	Caryophyllaceae	*Silene*	*Silene latifolia*	EF091518
Eudicotyledons	Caryophyllaceae	*Silene*	*Silene latifolia*	EF091519
Eudicotyledons	Caryophyllaceae	*Silene*	*Silene latifolia*	EF091520
Eudicotyledons	Caryophyllaceae	*Silene*	*Silene latifolia*	EF091521
Eudicotyledons	Caryophyllaceae	*Silene*	*Silene latifolia*	EF091522
Eudicotyledons	Caryophyllaceae	*Silene*	*Silene latifolia*	EF091524
Eudicotyledons	Caryophyllaceae	*Silene*	*Silene latifolia*	EF091525
Eudicotyledons	Caryophyllaceae	*Silene*	*Silene latifolia*	EF091526
Eudicotyledons	Caryophyllaceae	*Silene*	*Silene latifolia*	EF091528
Eudicotyledons	Caryophyllaceae	*Silene*	*Silene latifolia*	EF091529
Eudicotyledons	Caryophyllaceae	*Silene*	*Silene latifolia*	EF091530
Eudicotyledons	Caryophyllaceae	*Silene*	*Silene latifolia*	EF091531
Eudicotyledons	Caryophyllaceae	*Silene*	*Silene latifolia*	EF091532
Eudicotyledons	Caryophyllaceae	*Silene*	*Silene latifolia*	GU562391
Eudicotyledons	Caryophyllaceae	*Silene*	*Silene latifolia*	AF518904
Eudicotyledons	Caryophyllaceae	*Silene*	*Silene latifolia*	AF518905

Eudicotyledons	Caryophyllaceae	*Silene*	*Silene latifolia*	AF518906
Eudicotyledons	Caryophyllaceae	*Silene*	*Silene latifolia*	AF518907
Eudicotyledons	Caryophyllaceae	*Silene*	*Silene latifolia*	AF518908
Eudicotyledons	Caryophyllaceae	*Silene*	*Silene latifolia*	AF518909
Eudicotyledons	Caryophyllaceae	*Silene*	*Silene latifolia*	AF518910
Eudicotyledons	Caryophyllaceae	*Silene*	*Silene latifolia*	AF518911
Eudicotyledons	Caryophyllaceae	*Silene*	*Silene latifolia*	AF518912
Eudicotyledons	Caryophyllaceae	*Silene*	*Silene latifolia*	AF518913
Eudicotyledons	Caryophyllaceae	*Silene*	*Silene latifolia*	AF518914
Eudicotyledons	Caryophyllaceae	*Silene*	*Silene latifolia*	AF518915
Eudicotyledons	Caryophyllaceae	*Silene*	*Silene latifolia*	AF518916
Eudicotyledons	Caryophyllaceae	*Silene*	*Silene latifolia*	AF518917
Eudicotyledons	Caryophyllaceae	*Silene*	*Silene latifolia*	AF518918
Eudicotyledons	Caryophyllaceae	*Silene*	*Silene latifolia*	AF518919
Eudicotyledons	Caryophyllaceae	*Silene*	*Silene latifolia*	AF518920
Eudicotyledons	Caryophyllaceae	*Silene*	*Silene latifolia*	AF518921
Eudicotyledons	Caryophyllaceae	*Silene*	*Silene latifolia*	AF518922
Eudicotyledons	Caryophyllaceae	*Silene*	*Silene latifolia*	AF518923
Eudicotyledons	Caryophyllaceae	*Silene*	*Silene latifolia*	AF518924
Eudicotyledons	Caryophyllaceae	*Silene*	*Silene latifolia*	AF518925
Eudicotyledons	Caryophyllaceae	*Silene*	*Silene latifolia*	AF518926
Eudicotyledons	Caryophyllaceae	*Silene*	*Silene latifolia*	AF518927
Eudicotyledons	Caryophyllaceae	*Silene*	*Silene latifolia*	AF518928
Eudicotyledons	Caryophyllaceae	*Silene*	*Silene latifolia*	EU750546
Eudicotyledons	Caryophyllaceae	*Silene*	*Silene latifolia*	EU750547
Eudicotyledons	Caryophyllaceae	*Silene*	*Silene latifolia*	FN821213
Eudicotyledons	Caryophyllaceae	*Silene*	*Silene latifolia*	FN821214
Eudicotyledons	Caryophyllaceae	*Silene*	*Silene latifolia*	FN821215
Eudicotyledons	Caryophyllaceae	*Silene*	*Silene latifolia*	FN821216
Eudicotyledons	Caryophyllaceae	*Silene*	*Silene latifolia*	FN821217
Eudicotyledons	Caryophyllaceae	*Silene*	*Silene latifolia*	FN821218
Eudicotyledons	Caryophyllaceae	*Silene*	*Silene latifolia*	FN821219
Eudicotyledons	Caryophyllaceae	*Silene*	*Silene latifolia*	FN821220
Eudicotyledons	Caryophyllaceae	*Silene*	*Silene latifolia*	FN821221
Eudicotyledons	Caryophyllaceae	*Silene*	*Silene latifolia*	FN821222
Eudicotyledons	Caryophyllaceae	*Silene*	*Silene latifolia*	FN821223
Eudicotyledons	Caryophyllaceae	*Silene*	*Silene latifolia*	FN821224
Eudicotyledons	Caryophyllaceae	*Silene*	*Silene latifolia*	FN821225
Eudicotyledons	Oleaceae	*Fraxinus*	*Fraxinus excelsior*	FJ395513
Eudicotyledons	Oleaceae	*Fraxinus*	*Fraxinus excelsior*	HM367417
Eudicotyledons	Oleaceae	*Fraxinus*	*Fraxinus excelsior*	HM367418
Eudicotyledons	Oleaceae	*Fraxinus*	*Fraxinus excelsior*	HM367419
Eudicotyledons	Oleaceae	*Fraxinus*	*Fraxinus excelsior*	HM367420
Eudicotyledons	Oleaceae	*Fraxinus*	*Fraxinus excelsior*	HM367421
Eudicotyledons	Oleaceae	*Fraxinus*	*Fraxinus excelsior*	HM367422
Eudicotyledons	Oleaceae	*Fraxinus*	*Fraxinus excelsior*	HM367423
Eudicotyledons	Lamiaceae	*Origanum*	*Origanum vulgare*	DQ667353
Eudicotyledons	Lamiaceae	*Origanum*	*Origanum vulgare*	HQ596773
Eudicotyledons	Lamiaceae	*Origanum*	*Origanum vulgare*	FR726129
Eudicotyledons	Lamiaceae	*Origanum*	*Origanum vulgare*	FR726130
Eudicotyledons	Lamiaceae	*Origanum*	*Origanum vulgare*	FR726131
Eudicotyledons	Lamiaceae	*Origanum*	*Origanum vulgare*	FR726132
Eudicotyledons	Lamiaceae	*Origanum*	*Origanum vulgare*	FR726133

Eudicotyledons	Lamiaceae	*Origanum*	*Origanum vulgare*	FR865080
Eudicotyledons	Lamiaceae	*Origanum*	*Origanum vulgare*	FJ513100
Eudicotyledons	Caryophyllaceae	*Silene*	*Silene vulgaris*	EF094072
Eudicotyledons	Caryophyllaceae	*Silene*	*Silene vulgaris*	EF094073
Eudicotyledons	Caryophyllaceae	*Silene*	*Silene vulgaris*	EF094074
Eudicotyledons	Caryophyllaceae	*Silene*	*Silene vulgaris*	EF094075
Eudicotyledons	Caryophyllaceae	*Silene*	*Silene vulgaris*	EF094076
Eudicotyledons	Caryophyllaceae	*Silene*	*Silene vulgaris*	EF094077
Eudicotyledons	Caryophyllaceae	*Silene*	*Silene vulgaris*	EF094078
Eudicotyledons	Caryophyllaceae	*Silene*	*Silene vulgaris*	EF094079
Eudicotyledons	Caryophyllaceae	*Silene*	*Silene vulgaris*	EF094080
Eudicotyledons	Caryophyllaceae	*Silene*	*Silene vulgaris*	EF094081
Eudicotyledons	Caryophyllaceae	*Silene*	*Silene vulgaris*	EF094082
Eudicotyledons	Caryophyllaceae	*Silene*	*Silene vulgaris*	EF094083
Eudicotyledons	Caryophyllaceae	*Silene*	*Silene vulgaris*	EF094084
Eudicotyledons	Caryophyllaceae	*Silene*	*Silene vulgaris*	EF094085
Eudicotyledons	Caryophyllaceae	*Silene*	*Silene vulgaris*	EF094086
Eudicotyledons	Caryophyllaceae	*Silene*	*Silene vulgaris*	EF094087
Eudicotyledons	Caryophyllaceae	*Silene*	*Silene vulgaris*	EF094088
Eudicotyledons	Caryophyllaceae	*Silene*	*Silene vulgaris*	EF094089
Eudicotyledons	Caryophyllaceae	*Silene*	*Silene vulgaris*	EF094090
Eudicotyledons	Caryophyllaceae	*Silene*	*Silene vulgaris*	EF094091
Eudicotyledons	Caryophyllaceae	*Silene*	*Silene vulgaris*	EF094092
Eudicotyledons	Caryophyllaceae	*Silene*	*Silene vulgaris*	EF094093
Eudicotyledons	Caryophyllaceae	*Silene*	*Silene vulgaris*	HE602491
Eudicotyledons	Caryophyllaceae	*Silene*	*Silene vulgaris*	AF518957
Eudicotyledons	Caryophyllaceae	*Silene*	*Silene vulgaris*	AF518958
Eudicotyledons	Caryophyllaceae	*Silene*	*Silene vulgaris*	AF518959
Eudicotyledons	Caryophyllaceae	*Silene*	*Silene vulgaris*	AF518960
Eudicotyledons	Caryophyllaceae	*Silene*	*Silene vulgaris*	AF518961
Eudicotyledons	Caryophyllaceae	*Silene*	*Silene vulgaris*	AF518962
Eudicotyledons	Caryophyllaceae	*Silene*	*Silene vulgaris*	AF518963
Eudicotyledons	Caryophyllaceae	*Silene*	*Silene vulgaris*	AF518964
Eudicotyledons	Caryophyllaceae	*Silene*	*Silene vulgaris*	AF518965
Eudicotyledons	Caryophyllaceae	*Silene*	*Silene vulgaris*	AF518966
Eudicotyledons	Caryophyllaceae	*Silene*	*Silene vulgaris*	AF518967
Eudicotyledons	Caryophyllaceae	*Silene*	*Silene vulgaris*	AF518968
Eudicotyledons	Caryophyllaceae	*Silene*	*Silene vulgaris*	AF518969
Eudicotyledons	Caryophyllaceae	*Silene*	*Silene vulgaris*	AF518970
Eudicotyledons	Caryophyllaceae	*Silene*	*Silene vulgaris*	AF518971
Eudicotyledons	Caryophyllaceae	*Silene*	*Silene vulgaris*	AF518972
Eudicotyledons	Caryophyllaceae	*Silene*	*Silene vulgaris*	AF518973
Eudicotyledons	Caryophyllaceae	*Silene*	*Silene vulgaris*	AF518974
Eudicotyledons	Caryophyllaceae	*Silene*	*Silene vulgaris*	AF518975
Eudicotyledons	Caryophyllaceae	*Silene*	*Silene vulgaris*	AF518976
Eudicotyledons	Caryophyllaceae	*Silene*	*Silene vulgaris*	AF518977
Eudicotyledons	Caryophyllaceae	*Silene*	*Silene vulgaris*	AF518978
Eudicotyledons	Caryophyllaceae	*Silene*	*Silene vulgaris*	AF518979
Eudicotyledons	Caryophyllaceae	*Silene*	*Silene vulgaris*	AF518980
Eudicotyledons	Caryophyllaceae	*Silene*	*Silene vulgaris*	AF518981
Eudicotyledons	Caryophyllaceae	*Silene*	*Silene vulgaris*	AF518982
Eudicotyledons	Caryophyllaceae	*Silene*	*Silene vulgaris*	AF518983
Eudicotyledons	Caryophyllaceae	*Silene*	*Silene vulgaris*	AF518984

Eudicotyledons	Caryophyllaceae	*Silene*	*Silene vulgaris*	AY629274
Eudicotyledons	Caryophyllaceae	*Silene*	*Silene vulgaris*	AY629275
Eudicotyledons	Caryophyllaceae	*Silene*	*Silene vulgaris*	AY629276
Eudicotyledons	Caryophyllaceae	*Silene*	*Silene vulgaris*	AY629277
Eudicotyledons	Caryophyllaceae	*Silene*	*Silene vulgaris*	AY629278
Eudicotyledons	Caryophyllaceae	*Silene*	*Silene vulgaris*	AY629279
Eudicotyledons	Caryophyllaceae	*Silene*	*Silene vulgaris*	AY629280
Eudicotyledons	Caryophyllaceae	*Silene*	*Silene vulgaris*	AY629281
Eudicotyledons	Caryophyllaceae	*Silene*	*Silene vulgaris*	AY629282
Eudicotyledons	Caryophyllaceae	*Silene*	*Silene vulgaris*	AY629283
Eudicotyledons	Caryophyllaceae	*Silene*	*Silene vulgaris*	AY629284
Eudicotyledons	Caryophyllaceae	*Silene*	*Silene vulgaris*	AY629285
Eudicotyledons	Caryophyllaceae	*Silene*	*Silene vulgaris*	AY629286
Eudicotyledons	Caryophyllaceae	*Silene*	*Silene vulgaris*	AY629287
Eudicotyledons	Caryophyllaceae	*Silene*	*Silene vulgaris*	AY629288
Eudicotyledons	Caryophyllaceae	*Silene*	*Silene vulgaris*	AY629289
Eudicotyledons	Caryophyllaceae	*Silene*	*Silene vulgaris*	AY629290
Eudicotyledons	Caryophyllaceae	*Silene*	*Silene vulgaris*	AY629291
Eudicotyledons	Caryophyllaceae	*Silene*	*Silene vulgaris*	AY629292
Eudicotyledons	Caryophyllaceae	*Silene*	*Silene vulgaris*	AY629294
Eudicotyledons	Caryophyllaceae	*Silene*	*Silene vulgaris*	AY629295
Eudicotyledons	Caryophyllaceae	*Silene*	*Silene vulgaris*	AY629296
Eudicotyledons	Caryophyllaceae	*Silene*	*Silene vulgaris*	AY629297
Eudicotyledons	Caryophyllaceae	*Silene*	*Silene vulgaris*	EU750548
Eudicotyledons	Caryophyllaceae	*Silene*	*Silene vulgaris*	EU750549
Eudicotyledons	Caryophyllaceae	*Silene*	*Silene vulgaris*	EU750550
Eudicotyledons	Caryophyllaceae	*Silene*	*Silene vulgaris*	EU805570
Eudicotyledons	Caryophyllaceae	*Silene*	*Silene vulgaris*	EU805571
Eudicotyledons	Caryophyllaceae	*Silene*	*Silene vulgaris*	EU805572
Eudicotyledons	Caryophyllaceae	*Silene*	*Silene vulgaris*	EU805573
Eudicotyledons	Caryophyllaceae	*Silene*	*Silene vulgaris*	EU805574
Eudicotyledons	Gentianaceae	*Swertia*	*Swertia tetraptera*	JN047299
Eudicotyledons	Gentianaceae	*Swertia*	*Swertia tetraptera*	JN047300
Eudicotyledons	Apiaceae	*Angelica*	*Angelica decursiva*	GQ435318
Eudicotyledons	Apiaceae	*Angelica*	*Angelica decursiva*	GQ435319
Eudicotyledons	Caryophyllaceae	*Silene*	*Silene aprica*	JN047101
Eudicotyledons	Caryophyllaceae	*Silene*	*Silene aprica*	JN047102
Eudicotyledons	Caryophyllaceae	*Silene*	*Silene aprica*	JN047103
Eudicotyledons	Caryophyllaceae	*Silene*	*Silene aprica*	JN047104
Eudicotyledons	Caryophyllaceae	*Silene*	*Silene aprica*	JN047105
Eudicotyledons	Caryophyllaceae	*Silene*	*Silene aprica*	JN047106
Eudicotyledons	Caryophyllaceae	*Silene*	*Silene aprica*	JN047107
Eudicotyledons	Caryophyllaceae	*Silene*	*Silene aprica*	JN047108
Eudicotyledons	Caryophyllaceae	*Silene*	*Silene aprica*	JN047109
Eudicotyledons	Caryophyllaceae	*Silene*	*Silene aprica*	JN047110
Eudicotyledons	Caryophyllaceae	*Silene*	*Silene aprica*	JN047111
Eudicotyledons	Caryophyllaceae	*Silene*	*Silene aprica*	JN047112
Eudicotyledons	Oleaceae	*Fraxinus*	*Fraxinus mandshurica*	HM367487
Eudicotyledons	Oleaceae	*Fraxinus*	*Fraxinus mandshurica*	HM367488
Eudicotyledons	Oleaceae	*Fraxinus*	*Fraxinus mandshurica*	HM367489
Eudicotyledons	Oleaceae	*Fraxinus*	*Fraxinus mandshurica*	HM367490
Eudicotyledons	Oleaceae	*Fraxinus*	*Fraxinus mandshurica*	HM367491
Eudicotyledons	Oleaceae	*Fraxinus*	*Fraxinus mandshurica*	HM367492

Eudicotyledons	Oleaceae	*Fraxinus*	*Fraxinus mandshurica*	HM367493
Eudicotyledons	Oleaceae	*Fraxinus*	*Fraxinus mandshurica*	HM367494
Eudicotyledons	Oleaceae	*Fraxinus*	*Fraxinus mandshurica*	HM367495
Eudicotyledons	Oleaceae	*Fraxinus*	*Fraxinus platypoda*	HM367513
Eudicotyledons	Oleaceae	*Fraxinus*	*Fraxinus platypoda*	HM367514
Eudicotyledons	Oleaceae	*Fraxinus*	*Fraxinus platypoda*	HM367515
Eudicotyledons	Oleaceae	*Fraxinus*	*Fraxinus platypoda*	HM367516
Eudicotyledons	Oleaceae	*Fraxinus*	*Fraxinus platypoda*	HM367517
Eudicotyledons	Oleaceae	*Fraxinus*	*Fraxinus platypoda*	HM367518
Eudicotyledons	Oleaceae	*Fraxinus*	*Fraxinus platypoda*	HM367519
Eudicotyledons	Oleaceae	*Fraxinus*	*Fraxinus platypoda*	HM367520
Eudicotyledons	Oleaceae	*Fraxinus*	*Fraxinus platypoda*	HM367521
Eudicotyledons	Oleaceae	*Fraxinus*	*Fraxinus platypoda*	HM367522
Eudicotyledons	Oleaceae	*Fraxinus*	*Fraxinus platypoda*	HM367523
Eudicotyledons	Oleaceae	*Fraxinus*	*Fraxinus platypoda*	HM367524
Eudicotyledons	Oleaceae	*Fraxinus*	*Fraxinus chinensis*	GQ435221
Eudicotyledons	Oleaceae	*Fraxinus*	*Fraxinus chinensis*	HM367402
Eudicotyledons	Oleaceae	*Fraxinus*	*Fraxinus chinensis*	HM367403
Eudicotyledons	Oleaceae	*Fraxinus*	*Fraxinus chinensis*	HM367404
Eudicotyledons	Oleaceae	*Fraxinus*	*Fraxinus chinensis*	HM367405
Eudicotyledons	Oleaceae	*Fraxinus*	*Fraxinus chinensis*	HM367406
Eudicotyledons	Oleaceae	*Fraxinus*	*Fraxinus chinensis*	HM367407
Eudicotyledons	Oleaceae	*Fraxinus*	*Fraxinus chinensis*	HM367408
Eudicotyledons	Oleaceae	*Fraxinus*	*Fraxinus chinensis*	HM367409
Eudicotyledons	Oleaceae	*Fraxinus*	*Fraxinus cuspidata*	HM367410
Eudicotyledons	Oleaceae	*Fraxinus*	*Fraxinus cuspidata*	HM367411
Eudicotyledons	Oleaceae	*Fraxinus*	*Fraxinus cuspidata*	HM367412
Eudicotyledons	Oleaceae	*Fraxinus*	*Fraxinus cuspidata*	HM367413
Eudicotyledons	Oleaceae	*Fraxinus*	*Fraxinus cuspidata*	HM367414
Eudicotyledons	Plantaginaceae	*Veronica*	*Veronica densifolia*	FJ848125
Eudicotyledons	Plantaginaceae	*Veronica*	*Veronica densifolia*	FJ848126
Eudicotyledons	Plantaginaceae	*Veronica*	*Veronica salicifolia*	FJ848141
Eudicotyledons	Plantaginaceae	*Veronica*	*Veronica salicifolia*	FJ848142
Eudicotyledons	Ranunculaceae	*Eranthis*	*Eranthis byunsanensis*	JF505810
Eudicotyledons	Ranunculaceae	*Eranthis*	*Eranthis byunsanensis*	JF505811
Eudicotyledons	Ranunculaceae	*Eranthis*	*Eranthis byunsanensis*	JF505812
Eudicotyledons	Ranunculaceae	*Eranthis*	*Eranthis byunsanensis*	JF505813
Eudicotyledons	Ranunculaceae	*Eranthis*	*Eranthis byunsanensis*	JF505814
Eudicotyledons	Ranunculaceae	*Eranthis*	*Eranthis byunsanensis*	JF505815
Eudicotyledons	Ranunculaceae	*Eranthis*	*Eranthis byunsanensis*	JF505816
Eudicotyledons	Ranunculaceae	*Eranthis*	*Eranthis byunsanensis*	JF505817
Eudicotyledons	Ranunculaceae	*Eranthis*	*Eranthis byunsanensis*	JF505818
Eudicotyledons	Ranunculaceae	*Eranthis*	*Eranthis byunsanensis*	JF505819
Eudicotyledons	Ranunculaceae	*Eranthis*	*Eranthis byunsanensis*	JF505820
Eudicotyledons	Ranunculaceae	*Eranthis*	*Eranthis byunsanensis*	JF505821
Eudicotyledons	Ranunculaceae	*Eranthis*	*Eranthis byunsanensis*	JF505822
Eudicotyledons	Ranunculaceae	*Eranthis*	*Eranthis byunsanensis*	JF505823
Eudicotyledons	Ranunculaceae	*Eranthis*	*Eranthis byunsanensis*	JF505824
Eudicotyledons	Ranunculaceae	*Eranthis*	*Eranthis byunsanensis*	JF505825
Eudicotyledons	Ranunculaceae	*Eranthis*	*Eranthis byunsanensis*	JF505826
Eudicotyledons	Ranunculaceae	*Eranthis*	*Eranthis byunsanensis*	JF505827
Eudicotyledons	Ranunculaceae	*Eranthis*	*Eranthis byunsanensis*	JF505828
Eudicotyledons	Ranunculaceae	*Eranthis*	*Eranthis byunsanensis*	JF505829

Eudicotyledons	Ranunculaceae	*Eranthis*	*Eranthis byunsanensis*	JF505830
Eudicotyledons	Ranunculaceae	*Eranthis*	*Eranthis byunsanensis*	JF505831
Eudicotyledons	Ranunculaceae	*Eranthis*	*Eranthis byunsanensis*	JF505832
Eudicotyledons	Ranunculaceae	*Eranthis*	*Eranthis byunsanensis*	JF505833
Eudicotyledons	Ranunculaceae	*Eranthis*	*Eranthis byunsanensis*	JF505834
Eudicotyledons	Saxifragaceae	*Mitella*	*Mitella japonica*	AB492506
Eudicotyledons	Saxifragaceae	*Mitella*	*Mitella japonica*	AB492507
Eudicotyledons	Saxifragaceae	*Mitella*	*Mitella japonica*	AB492508
Eudicotyledons	Saxifragaceae	*Mitella*	*Mitella japonica*	AB492509
Eudicotyledons	Saxifragaceae	*Mitella*	*Mitella japonica*	AB492510
Eudicotyledons	Saxifragaceae	*Mitella*	*Mitella japonica*	AB492511
Eudicotyledons	Saxifragaceae	*Mitella*	*Mitella japonica*	AB492512
Eudicotyledons	Saxifragaceae	*Mitella*	*Mitella japonica*	AB492513
Eudicotyledons	Saxifragaceae	*Mitella*	*Mitella japonica*	AB492514
Eudicotyledons	Saxifragaceae	*Mitella*	*Mitella japonica*	AB492515
Eudicotyledons	Saxifragaceae	*Mitella*	*Mitella japonica*	AB492516
Eudicotyledons	Saxifragaceae	*Mitella*	*Mitella japonica*	AB492517
Eudicotyledons	Saxifragaceae	*Mitella*	*Mitella japonica*	AB492518
Eudicotyledons	Saxifragaceae	*Mitella*	*Mitella japonica*	AB492519
Eudicotyledons	Saxifragaceae	*Mitella*	*Mitella japonica*	AB492520
Eudicotyledons	Saxifragaceae	*Mitella*	*Mitella japonica*	AB492521
Eudicotyledons	Saxifragaceae	*Mitella*	*Mitella japonica*	AB492522
Eudicotyledons	Saxifragaceae	*Mitella*	*Mitella japonica*	AB492523
Eudicotyledons	Saxifragaceae	*Mitella*	*Mitella japonica*	AB492524
Eudicotyledons	Saxifragaceae	*Mitella*	*Mitella japonica*	AB492525
Eudicotyledons	Saxifragaceae	*Mitella*	*Mitella japonica*	AB492526
Eudicotyledons	Saxifragaceae	*Mitella*	*Mitella japonica*	AB492527
Eudicotyledons	Saxifragaceae	*Mitella*	*Mitella japonica*	AB492528
Eudicotyledons	Saxifragaceae	*Mitella*	*Mitella japonica*	AB492529
Eudicotyledons	Saxifragaceae	*Mitella*	*Mitella japonica*	AB492530
Eudicotyledons	Saxifragaceae	*Mitella*	*Mitella japonica*	AB492531
Eudicotyledons	Saxifragaceae	*Mitella*	*Mitella japonica*	AB492532
Eudicotyledons	Saxifragaceae	*Mitella*	*Mitella japonica*	AB492533
Eudicotyledons	Saxifragaceae	*Mitella*	*Mitella japonica*	AB492534
Eudicotyledons	Saxifragaceae	*Mitella*	*Mitella pauciflora*	AB492591
Eudicotyledons	Saxifragaceae	*Mitella*	*Mitella pauciflora*	AB492592
Eudicotyledons	Saxifragaceae	*Mitella*	*Mitella pauciflora*	AB492593
Eudicotyledons	Saxifragaceae	*Mitella*	*Mitella pauciflora*	AB492594
Eudicotyledons	Saxifragaceae	*Mitella*	*Mitella pauciflora*	AB492595
Eudicotyledons	Saxifragaceae	*Mitella*	*Mitella pauciflora*	AB492596
Eudicotyledons	Saxifragaceae	*Mitella*	*Mitella pauciflora*	AB492597
Eudicotyledons	Saxifragaceae	*Mitella*	*Mitella pauciflora*	AB492598
Eudicotyledons	Saxifragaceae	*Mitella*	*Mitella pauciflora*	AB492599
Eudicotyledons	Saxifragaceae	*Mitella*	*Mitella pauciflora*	AB492600
Eudicotyledons	Saxifragaceae	*Mitella*	*Mitella pauciflora*	AB492601
Eudicotyledons	Saxifragaceae	*Mitella*	*Mitella pauciflora*	AB492602
Eudicotyledons	Saxifragaceae	*Mitella*	*Mitella pauciflora*	AB492603
Eudicotyledons	Saxifragaceae	*Mitella*	*Mitella pauciflora*	AB492604
Eudicotyledons	Saxifragaceae	*Mitella*	*Mitella pauciflora*	AB492605
Eudicotyledons	Saxifragaceae	*Mitella*	*Mitella pauciflora*	AB492606
Eudicotyledons	Saxifragaceae	*Mitella*	*Mitella pauciflora*	AB492607
Eudicotyledons	Saxifragaceae	*Mitella*	*Mitella pauciflora*	AB492608
Eudicotyledons	Saxifragaceae	*Mitella*	*Mitella pauciflora*	AB492609

Eudicotyledons	Saxifragaceae	*Mitella*	*Mitella pauciflora*	AB492610
Eudicotyledons	Saxifragaceae	*Mitella*	*Mitella pauciflora*	AB492611
Eudicotyledons	Saxifragaceae	*Mitella*	*Mitella yoshinagae*	AB492535
Eudicotyledons	Saxifragaceae	*Mitella*	*Mitella yoshinagae*	AB492536
Eudicotyledons	Saxifragaceae	*Mitella*	*Mitella yoshinagae*	AB492537
Eudicotyledons	Saxifragaceae	*Mitella*	*Mitella yoshinagae*	AB492538
Eudicotyledons	Saxifragaceae	*Mitella*	*Mitella yoshinagae*	AB492539
Eudicotyledons	Saxifragaceae	*Mitella*	*Mitella yoshinagae*	AB492540
Eudicotyledons	Saxifragaceae	*Mitella*	*Mitella yoshinagae*	AB492541
Eudicotyledons	Saxifragaceae	*Mitella*	*Mitella yoshinagae*	AB492542
Eudicotyledons	Saxifragaceae	*Mitella*	*Mitella yoshinagae*	AB492543
Eudicotyledons	Saxifragaceae	*Mitella*	*Mitella yoshinagae*	AB492544
Eudicotyledons	Saxifragaceae	*Mitella*	*Mitella yoshinagae*	AB492545
Eudicotyledons	Saxifragaceae	*Mitella*	*Mitella yoshinagae*	AB492546
Eudicotyledons	Saxifragaceae	*Mitella*	*Mitella yoshinagae*	AB492547
Eudicotyledons	Saxifragaceae	*Mitella*	*Mitella yoshinagae*	AB492548
Eudicotyledons	Oleaceae	*Fraxinus*	*Fraxinus texensis*	HM367558
Eudicotyledons	Oleaceae	*Fraxinus*	*Fraxinus texensis*	HM367559
Eudicotyledons	Rosaceae	*Chaenomeles*	*Chaenomeles speciosa*	JQ390646
Eudicotyledons	Rosaceae	*Chaenomeles*	*Chaenomeles speciosa*	JQ390649
Eudicotyledons	Rosaceae	*Chaenomeles*	*Chaenomeles speciosa*	JQ390650
Eudicotyledons	Rosaceae	*Chaenomeles*	*Chaenomeles speciosa*	JQ390651
Eudicotyledons	Rosaceae	*Chaenomeles*	*Chaenomeles speciosa*	JQ390653
Eudicotyledons	Oleaceae	*Fraxinus*	*Fraxinus greggii*	HM367428
Eudicotyledons	Oleaceae	*Fraxinus*	*Fraxinus greggii*	HM367429
Eudicotyledons	Oleaceae	*Fraxinus*	*Fraxinus greggii*	HM367430
Eudicotyledons	Oleaceae	*Fraxinus*	*Fraxinus greggii*	HM367431
Eudicotyledons	Oleaceae	*Fraxinus*	*Fraxinus greggii*	HM367432
Eudicotyledons	Oleaceae	*Fraxinus*	*Fraxinus greggii*	HM367433
Eudicotyledons	Oleaceae	*Fraxinus*	*Fraxinus angustifolia*	HE659569
Eudicotyledons	Oleaceae	*Fraxinus*	*Fraxinus angustifolia*	HM367365
Eudicotyledons	Oleaceae	*Fraxinus*	*Fraxinus angustifolia*	HM367366
Eudicotyledons	Oleaceae	*Fraxinus*	*Fraxinus angustifolia*	HM367367
Eudicotyledons	Oleaceae	*Fraxinus*	*Fraxinus angustifolia*	HM367368
Eudicotyledons	Oleaceae	*Fraxinus*	*Fraxinus angustifolia*	HM367369
Eudicotyledons	Oleaceae	*Fraxinus*	*Fraxinus angustifolia*	HM367370
Eudicotyledons	Oleaceae	*Fraxinus*	*Fraxinus angustifolia*	HM367371
Eudicotyledons	Oleaceae	*Fraxinus*	*Fraxinus angustifolia*	HM367372
Eudicotyledons	Oleaceae	*Fraxinus*	*Fraxinus angustifolia*	HM367373
Eudicotyledons	Oleaceae	*Fraxinus*	*Fraxinus angustifolia*	HM367374
Eudicotyledons	Oleaceae	*Fraxinus*	*Fraxinus angustifolia*	HM367375
Eudicotyledons	Oleaceae	*Fraxinus*	*Fraxinus angustifolia*	HM367376
Eudicotyledons	Oleaceae	*Fraxinus*	*Fraxinus angustifolia*	HM367377
Eudicotyledons	Oleaceae	*Fraxinus*	*Fraxinus angustifolia*	HM367378
Eudicotyledons	Ericaceae	*Rhododendron*	*Rhododendron fortunei*	HQ706969
Eudicotyledons	Ericaceae	*Rhododendron*	*Rhododendron fortunei*	HQ706970
Eudicotyledons	Ericaceae	*Rhododendron*	*Rhododendron fortunei*	HQ706971
Eudicotyledons	Ericaceae	*Rhododendron*	*Rhododendron fortunei*	HQ706972
Eudicotyledons	Ericaceae	*Rhododendron*	*Rhododendron latoucheae*	HQ426990
Eudicotyledons	Ericaceae	*Rhododendron*	*Rhododendron latoucheae*	HQ706992
Eudicotyledons	Ranunculaceae	*Eranthis*	*Eranthis pinnatifida*	JF505843
Eudicotyledons	Ranunculaceae	*Eranthis*	*Eranthis pinnatifida*	JF505844
Eudicotyledons	Ranunculaceae	*Eranthis*	*Eranthis pinnatifida*	JF505845

Eudicotyledons	Ranunculaceae	*Eranthis*	*Eranthis pinnatifida*	JF505846
Eudicotyledons	Ranunculaceae	*Eranthis*	*Eranthis pinnatifida*	JF505847
Eudicotyledons	Ranunculaceae	*Eranthis*	*Eranthis pinnatifida*	JF505848
Eudicotyledons	Saxifragaceae	*Mitella*	*Mitella formosana*	AB492549
Eudicotyledons	Saxifragaceae	*Mitella*	*Mitella formosana*	AB492550
Eudicotyledons	Saxifragaceae	*Mitella*	*Mitella formosana*	AB492551
Eudicotyledons	Saxifragaceae	*Mitella*	*Mitella formosana*	AB492552
Eudicotyledons	Saxifragaceae	*Mitella*	*Mitella formosana*	AB492553
Eudicotyledons	Saxifragaceae	*Mitella*	*Mitella formosana*	AB492554
Eudicotyledons	Saxifragaceae	*Mitella*	*Mitella formosana*	AB492555
Eudicotyledons	Saxifragaceae	*Mitella*	*Mitella formosana*	AB492556
Eudicotyledons	Lamiaceae	*Origanum*	*Origanum majorana*	FR726122
Eudicotyledons	Lamiaceae	*Origanum*	*Origanum majorana*	FR726123
Eudicotyledons	Lamiaceae	*Origanum*	*Origanum majorana*	FR726124
Eudicotyledons	Lamiaceae	*Origanum*	*Origanum majorana*	FR726125
Eudicotyledons	Lamiaceae	*Origanum*	*Origanum majorana*	HQ902835
Eudicotyledons	Ericaceae	*Rhododendron*	*Rhododendron delavayi*	JN046793
Eudicotyledons	Ericaceae	*Rhododendron*	*Rhododendron delavayi*	JN046794
Eudicotyledons	Ericaceae	*Rhododendron*	*Rhododendron delavayi*	JN046795
Eudicotyledons	Ericaceae	*Rhododendron*	*Rhododendron delavayi*	JN046796
Eudicotyledons	Ericaceae	*Rhododendron*	*Rhododendron delavayi*	JN046797
Eudicotyledons	Ericaceae	*Rhododendron*	*Rhododendron delavayi*	HM636525
Eudicotyledons	Ericaceae	*Rhododendron*	*Rhododendron delavayi*	HQ706960
Eudicotyledons	Ericaceae	*Rhododendron*	*Rhododendron delavayi*	HQ706961
Eudicotyledons	Ericaceae	*Rhododendron*	*Rhododendron irroratum*	JN046846
Eudicotyledons	Ericaceae	*Rhododendron*	*Rhododendron irroratum*	JN046847
Eudicotyledons	Ericaceae	*Rhododendron*	*Rhododendron irroratum*	JN046848
Eudicotyledons	Ericaceae	*Rhododendron*	*Rhododendron irroratum*	JN046849
Eudicotyledons	Ericaceae	*Rhododendron*	*Rhododendron irroratum*	HQ706986
Eudicotyledons	Ericaceae	*Rhododendron*	*Rhododendron irroratum*	HQ706987
Eudicotyledons	Ericaceae	*Rhododendron*	*Rhododendron irroratum*	HQ706988
Eudicotyledons	Orobanchaceae	*Pedicularis*	*Pedicularis rex*	JN046094
Eudicotyledons	Orobanchaceae	*Pedicularis*	*Pedicularis rex*	JN046095
Eudicotyledons	Orobanchaceae	*Pedicularis*	*Pedicularis rex*	JN046096
Eudicotyledons	Orobanchaceae	*Pedicularis*	*Pedicularis rex*	JN046097
Eudicotyledons	Orobanchaceae	*Pedicularis*	*Pedicularis rex*	JN046098
Eudicotyledons	Solanaceae	*Petunia*	*Petunia altiplana*	DQ791909
Eudicotyledons	Solanaceae	*Petunia*	*Petunia altiplana*	DQ791910
Eudicotyledons	Solanaceae	*Petunia*	*Petunia altiplana*	DQ791911
Eudicotyledons	Solanaceae	*Petunia*	*Petunia altiplana*	DQ791912
Eudicotyledons	Solanaceae	*Petunia*	*Petunia altiplana*	DQ791913
Eudicotyledons	Solanaceae	*Petunia*	*Petunia altiplana*	DQ791914
Eudicotyledons	Solanaceae	*Petunia*	*Petunia altiplana*	DQ791915
Eudicotyledons	Solanaceae	*Petunia*	*Petunia altiplana*	DQ791916
Eudicotyledons	Solanaceae	*Petunia*	*Petunia altiplana*	DQ791917
Eudicotyledons	Solanaceae	*Petunia*	*Petunia altiplana*	DQ791918
Eudicotyledons	Solanaceae	*Petunia*	*Petunia altiplana*	DQ791919
Eudicotyledons	Solanaceae	*Petunia*	*Petunia altiplana*	DQ791920
Eudicotyledons	Solanaceae	*Petunia*	*Petunia altiplana*	DQ791921
Eudicotyledons	Solanaceae	*Petunia*	*Petunia altiplana*	DQ791922
Eudicotyledons	Solanaceae	*Petunia*	*Petunia altiplana*	DQ791923
Eudicotyledons	Solanaceae	*Petunia*	*Petunia altiplana*	DQ791924
Eudicotyledons	Solanaceae	*Petunia*	*Petunia altiplana*	DQ791925

Eudicotyledons	Solanaceae	*Petunia*	*Petunia altiplana*	DQ791926
Eudicotyledons	Solanaceae	*Petunia*	*Petunia altiplana*	DQ791927
Eudicotyledons	Solanaceae	*Petunia*	*Petunia altiplana*	DQ791928
Eudicotyledons	Solanaceae	*Petunia*	*Petunia altiplana*	DQ791929
Eudicotyledons	Solanaceae	*Petunia*	*Petunia altiplana*	DQ791930
Eudicotyledons	Solanaceae	*Petunia*	*Petunia altiplana*	DQ791931
Eudicotyledons	Solanaceae	*Petunia*	*Petunia altiplana*	DQ791932
Eudicotyledons	Solanaceae	*Petunia*	*Petunia altiplana*	DQ791933
Eudicotyledons	Solanaceae	*Petunia*	*Petunia altiplana*	DQ791934
Eudicotyledons	Solanaceae	*Petunia*	*Petunia altiplana*	DQ791935
Eudicotyledons	Solanaceae	*Petunia*	*Petunia altiplana*	DQ791936
Eudicotyledons	Solanaceae	*Petunia*	*Petunia altiplana*	DQ791937
Eudicotyledons	Solanaceae	*Petunia*	*Petunia altiplana*	DQ791938
Eudicotyledons	Solanaceae	*Petunia*	*Petunia altiplana*	DQ791939
Eudicotyledons	Solanaceae	*Petunia*	*Petunia altiplana*	DQ791940
Eudicotyledons	Solanaceae	*Petunia*	*Petunia altiplana*	DQ791941
Eudicotyledons	Solanaceae	*Petunia*	*Petunia altiplana*	DQ791942
Eudicotyledons	Solanaceae	*Petunia*	*Petunia altiplana*	DQ791943
Eudicotyledons	Solanaceae	*Petunia*	*Petunia altiplana*	DQ791944
Eudicotyledons	Solanaceae	*Petunia*	*Petunia altiplana*	DQ791945
Eudicotyledons	Solanaceae	*Petunia*	*Petunia altiplana*	DQ791946
Eudicotyledons	Solanaceae	*Petunia*	*Petunia altiplana*	DQ791947
Eudicotyledons	Solanaceae	*Petunia*	*Petunia altiplana*	DQ791948
Eudicotyledons	Solanaceae	*Petunia*	*Petunia altiplana*	DQ791949
Eudicotyledons	Solanaceae	*Petunia*	*Petunia altiplana*	DQ791950
Eudicotyledons	Solanaceae	*Petunia*	*Petunia altiplana*	DQ791951
Eudicotyledons	Solanaceae	*Petunia*	*Petunia altiplana*	DQ791952
Eudicotyledons	Solanaceae	*Petunia*	*Petunia altiplana*	DQ791953
Eudicotyledons	Solanaceae	*Petunia*	*Petunia altiplana*	DQ791954
Eudicotyledons	Solanaceae	*Petunia*	*Petunia altiplana*	DQ791955
Eudicotyledons	Solanaceae	*Petunia*	*Petunia altiplana*	DQ791956
Eudicotyledons	Solanaceae	*Petunia*	*Petunia altiplana*	DQ791957
Eudicotyledons	Solanaceae	*Petunia*	*Petunia altiplana*	DQ791958
Eudicotyledons	Solanaceae	*Petunia*	*Petunia altiplana*	DQ791959
Eudicotyledons	Solanaceae	*Petunia*	*Petunia altiplana*	DQ791960
Eudicotyledons	Solanaceae	*Petunia*	*Petunia altiplana*	DQ791961
Eudicotyledons	Solanaceae	*Petunia*	*Petunia altiplana*	DQ791962
Eudicotyledons	Solanaceae	*Petunia*	*Petunia altiplana*	DQ791963
Eudicotyledons	Solanaceae	*Petunia*	*Petunia altiplana*	DQ791964
Eudicotyledons	Solanaceae	*Petunia*	*Petunia altiplana*	DQ791965
Eudicotyledons	Solanaceae	*Petunia*	*Petunia altiplana*	DQ791966
Eudicotyledons	Solanaceae	*Petunia*	*Petunia altiplana*	DQ791967
Eudicotyledons	Solanaceae	*Petunia*	*Petunia altiplana*	DQ791968
Eudicotyledons	Solanaceae	*Petunia*	*Petunia altiplana*	DQ791969
Eudicotyledons	Solanaceae	*Petunia*	*Petunia altiplana*	DQ791970
Eudicotyledons	Solanaceae	*Petunia*	*Petunia altiplana*	DQ791971
Eudicotyledons	Solanaceae	*Petunia*	*Petunia altiplana*	DQ791972
Eudicotyledons	Solanaceae	*Petunia*	*Petunia altiplana*	DQ791973
Eudicotyledons	Solanaceae	*Petunia*	*Petunia altiplana*	DQ791974
Eudicotyledons	Solanaceae	*Petunia*	*Petunia altiplana*	DQ791975
Eudicotyledons	Solanaceae	*Petunia*	*Petunia altiplana*	DQ791976
Eudicotyledons	Solanaceae	*Petunia*	*Petunia altiplana*	DQ791977
Eudicotyledons	Solanaceae	*Petunia*	*Petunia altiplana*	DQ791978

Eudicotyledons	Solanaceae	*Petunia*	*Petunia altiplana*	DQ791979
Eudicotyledons	Solanaceae	*Petunia*	*Petunia altiplana*	DQ791980
Eudicotyledons	Solanaceae	*Petunia*	*Petunia altiplana*	DQ791981
Eudicotyledons	Solanaceae	*Petunia*	*Petunia altiplana*	DQ791982
Eudicotyledons	Solanaceae	*Petunia*	*Petunia altiplana*	DQ791983
Eudicotyledons	Solanaceae	*Petunia*	*Petunia altiplana*	DQ791984
Eudicotyledons	Solanaceae	*Petunia*	*Petunia altiplana*	DQ791985
Eudicotyledons	Solanaceae	*Petunia*	*Petunia altiplana*	DQ791986
Eudicotyledons	Solanaceae	*Petunia*	*Petunia altiplana*	DQ791987
Eudicotyledons	Solanaceae	*Petunia*	*Petunia altiplana*	DQ791988
Eudicotyledons	Solanaceae	*Petunia*	*Petunia altiplana*	DQ791989
Eudicotyledons	Solanaceae	*Petunia*	*Petunia altiplana*	DQ791990
Eudicotyledons	Solanaceae	*Petunia*	*Petunia altiplana*	DQ791991
Eudicotyledons	Solanaceae	*Petunia*	*Petunia altiplana*	DQ791992
Eudicotyledons	Solanaceae	*Petunia*	*Petunia altiplana*	DQ791993
Eudicotyledons	Solanaceae	*Petunia*	*Petunia altiplana*	DQ791994
Eudicotyledons	Solanaceae	*Petunia*	*Petunia altiplana*	DQ791995
Eudicotyledons	Solanaceae	*Petunia*	*Petunia altiplana*	DQ791996
Eudicotyledons	Solanaceae	*Petunia*	*Petunia altiplana*	DQ791997
Eudicotyledons	Solanaceae	*Petunia*	*Petunia altiplana*	DQ791998
Eudicotyledons	Solanaceae	*Petunia*	*Petunia altiplana*	AY772887
Eudicotyledons	Solanaceae	*Petunia*	*Petunia altiplana*	AY772888
Eudicotyledons	Solanaceae	*Petunia*	*Petunia altiplana*	AY772889
Eudicotyledons	Solanaceae	*Petunia*	*Petunia altiplana*	AY772890
Eudicotyledons	Solanaceae	*Petunia*	*Petunia altiplana*	AY772891
Eudicotyledons	Solanaceae	*Petunia*	*Petunia bonjardinensis*	DQ791999
Eudicotyledons	Solanaceae	*Petunia*	*Petunia bonjardinensis*	DQ792000
Eudicotyledons	Solanaceae	*Petunia*	*Petunia bonjardinensis*	DQ792001
Eudicotyledons	Solanaceae	*Petunia*	*Petunia bonjardinensis*	DQ792002
Eudicotyledons	Solanaceae	*Petunia*	*Petunia bonjardinensis*	DQ792003
Eudicotyledons	Solanaceae	*Petunia*	*Petunia bonjardinensis*	DQ792004
Eudicotyledons	Solanaceae	*Petunia*	*Petunia bonjardinensis*	DQ792005
Eudicotyledons	Solanaceae	*Petunia*	*Petunia bonjardinensis*	DQ792006
Eudicotyledons	Solanaceae	*Petunia*	*Petunia bonjardinensis*	DQ792007
Eudicotyledons	Solanaceae	*Petunia*	*Petunia bonjardinensis*	DQ792008
Eudicotyledons	Solanaceae	*Petunia*	*Petunia bonjardinensis*	DQ792009
Eudicotyledons	Solanaceae	*Petunia*	*Petunia bonjardinensis*	DQ792010
Eudicotyledons	Solanaceae	*Petunia*	*Petunia bonjardinensis*	DQ792011
Eudicotyledons	Solanaceae	*Petunia*	*Petunia bonjardinensis*	DQ792012
Eudicotyledons	Solanaceae	*Petunia*	*Petunia bonjardinensis*	DQ792013
Eudicotyledons	Solanaceae	*Petunia*	*Petunia bonjardinensis*	DQ792014
Eudicotyledons	Solanaceae	*Petunia*	*Petunia bonjardinensis*	DQ792015
Eudicotyledons	Solanaceae	*Petunia*	*Petunia bonjardinensis*	DQ792016
Eudicotyledons	Solanaceae	*Petunia*	*Petunia bonjardinensis*	DQ792017
Eudicotyledons	Solanaceae	*Petunia*	*Petunia bonjardinensis*	DQ792018
Eudicotyledons	Solanaceae	*Petunia*	*Petunia bonjardinensis*	DQ792019
Eudicotyledons	Solanaceae	*Petunia*	*Petunia bonjardinensis*	DQ792020
Eudicotyledons	Solanaceae	*Petunia*	*Petunia bonjardinensis*	DQ792021
Eudicotyledons	Solanaceae	*Petunia*	*Petunia bonjardinensis*	DQ792022
Eudicotyledons	Solanaceae	*Petunia*	*Petunia bonjardinensis*	DQ792023
Eudicotyledons	Solanaceae	*Petunia*	*Petunia bonjardinensis*	DQ792024
Eudicotyledons	Solanaceae	*Petunia*	*Petunia bonjardinensis*	DQ792025
Eudicotyledons	Solanaceae	*Petunia*	*Petunia bonjardinensis*	DQ792026

Eudicotyledons	Solanaceae	*Petunia*	*Petunia bonjardinensis*	DQ792027
Eudicotyledons	Solanaceae	*Petunia*	*Petunia bonjardinensis*	DQ792028
Eudicotyledons	Solanaceae	*Petunia*	*Petunia bonjardinensis*	DQ792029
Eudicotyledons	Solanaceae	*Petunia*	*Petunia bonjardinensis*	DQ792030
Eudicotyledons	Solanaceae	*Petunia*	*Petunia bonjardinensis*	DQ792031
Eudicotyledons	Solanaceae	*Petunia*	*Petunia bonjardinensis*	DQ792032
Eudicotyledons	Solanaceae	*Petunia*	*Petunia bonjardinensis*	DQ792033
Eudicotyledons	Solanaceae	*Petunia*	*Petunia bonjardinensis*	DQ792034
Eudicotyledons	Solanaceae	*Petunia*	*Petunia bonjardinensis*	DQ792035
Eudicotyledons	Solanaceae	*Petunia*	*Petunia bonjardinensis*	DQ792036
Eudicotyledons	Solanaceae	*Petunia*	*Petunia bonjardinensis*	DQ792037
Eudicotyledons	Solanaceae	*Petunia*	*Petunia bonjardinensis*	DQ792038
Eudicotyledons	Solanaceae	*Petunia*	*Petunia bonjardinensis*	DQ792039
Eudicotyledons	Solanaceae	*Petunia*	*Petunia bonjardinensis*	DQ792040
Eudicotyledons	Solanaceae	*Petunia*	*Petunia bonjardinensis*	DQ792041
Eudicotyledons	Solanaceae	*Petunia*	*Petunia bonjardinensis*	DQ792042
Eudicotyledons	Solanaceae	*Petunia*	*Petunia bonjardinensis*	DQ792043
Eudicotyledons	Solanaceae	*Petunia*	*Petunia bonjardinensis*	DQ792044
Eudicotyledons	Solanaceae	*Petunia*	*Petunia bonjardinensis*	DQ792045
Eudicotyledons	Solanaceae	*Petunia*	*Petunia bonjardinensis*	DQ792046
Eudicotyledons	Solanaceae	*Petunia*	*Petunia bonjardinensis*	DQ792047
Eudicotyledons	Solanaceae	*Petunia*	*Petunia bonjardinensis*	DQ792048
Eudicotyledons	Solanaceae	*Petunia*	*Petunia bonjardinensis*	DQ792049
Eudicotyledons	Solanaceae	*Petunia*	*Petunia bonjardinensis*	DQ792050
Eudicotyledons	Solanaceae	*Petunia*	*Petunia bonjardinensis*	DQ792051
Eudicotyledons	Solanaceae	*Petunia*	*Petunia bonjardinensis*	DQ792052
Eudicotyledons	Solanaceae	*Petunia*	*Petunia bonjardinensis*	DQ792053
Eudicotyledons	Solanaceae	*Petunia*	*Petunia bonjardinensis*	DQ792054
Eudicotyledons	Solanaceae	*Petunia*	*Petunia bonjardinensis*	DQ792055
Eudicotyledons	Solanaceae	*Petunia*	*Petunia bonjardinensis*	DQ792056
Eudicotyledons	Solanaceae	*Petunia*	*Petunia bonjardinensis*	DQ792057
Eudicotyledons	Solanaceae	*Petunia*	*Petunia bonjardinensis*	DQ792058
Eudicotyledons	Solanaceae	*Petunia*	*Petunia bonjardinensis*	AY772911
Eudicotyledons	Solanaceae	*Petunia*	*Petunia bonjardinensis*	AY772912
Eudicotyledons	Solanaceae	*Petunia*	*Petunia bonjardinensis*	AY772913
Eudicotyledons	Solanaceae	*Petunia*	*Petunia bonjardinensis*	AY772914
Eudicotyledons	Solanaceae	*Petunia*	*Petunia bonjardinensis*	AY772915
Eudicotyledons	Celastraceae	*Euonymus*	*Euonymus oxyphyllus*	AB525283
Eudicotyledons	Celastraceae	*Euonymus*	*Euonymus oxyphyllus*	AB525284
Eudicotyledons	Celastraceae	*Euonymus*	*Euonymus oxyphyllus*	AB525285
Eudicotyledons	Celastraceae	*Euonymus*	*Euonymus oxyphyllus*	AB525286
Eudicotyledons	Celastraceae	*Euonymus*	*Euonymus oxyphyllus*	AB525287
Eudicotyledons	Celastraceae	*Euonymus*	*Euonymus oxyphyllus*	AB525288
Eudicotyledons	Celastraceae	*Euonymus*	*Euonymus oxyphyllus*	AB525289
Eudicotyledons	Celastraceae	*Euonymus*	*Euonymus oxyphyllus*	AB525290
Eudicotyledons	Celastraceae	*Euonymus*	*Euonymus oxyphyllus*	AB525291
Eudicotyledons	Celastraceae	*Euonymus*	*Euonymus oxyphyllus*	AB525292
Eudicotyledons	Celastraceae	*Euonymus*	*Euonymus oxyphyllus*	AB525293
Eudicotyledons	Celastraceae	*Euonymus*	*Euonymus oxyphyllus*	AB525294
Eudicotyledons	Celastraceae	*Euonymus*	*Euonymus oxyphyllus*	AB525295
Eudicotyledons	Celastraceae	*Euonymus*	*Euonymus oxyphyllus*	AB525296
Eudicotyledons	Celastraceae	*Euonymus*	*Euonymus oxyphyllus*	AB525297
Eudicotyledons	Celastraceae	*Euonymus*	*Euonymus oxyphyllus*	AB525298

Eudicotyledons	Celastraceae	*Euonymus*	*Euonymus oxyphyllus*	AB525299
Eudicotyledons	Celastraceae	*Euonymus*	*Euonymus oxyphyllus*	AB525300
Eudicotyledons	Celastraceae	*Euonymus*	*Euonymus oxyphyllus*	AB525301
Eudicotyledons	Celastraceae	*Euonymus*	*Euonymus oxyphyllus*	AB525302
Eudicotyledons	Celastraceae	*Euonymus*	*Euonymus oxyphyllus*	AB525303
Eudicotyledons	Celastraceae	*Euonymus*	*Euonymus oxyphyllus*	AB525304
Eudicotyledons	Celastraceae	*Euonymus*	*Euonymus oxyphyllus*	AB525305
Eudicotyledons	Celastraceae	*Euonymus*	*Euonymus oxyphyllus*	AB525306
Eudicotyledons	Celastraceae	*Euonymus*	*Euonymus oxyphyllus*	AB525307
Eudicotyledons	Celastraceae	*Euonymus*	*Euonymus oxyphyllus*	AB525308
Eudicotyledons	Celastraceae	*Euonymus*	*Euonymus oxyphyllus*	AB525309
Eudicotyledons	Apiaceae	*Heracleum*	*Heracleum dissectum*	EF042117
Eudicotyledons	Apiaceae	*Heracleum*	*Heracleum dissectum*	GU967815
Eudicotyledons	Ericaceae	*Rhododendron*	*Rhododendron aganniphum*	JN046733
Eudicotyledons	Ericaceae	*Rhododendron*	*Rhododendron aganniphum*	JN046734
Eudicotyledons	Ericaceae	*Rhododendron*	*Rhododendron aganniphum*	HQ706948
Eudicotyledons	Ericaceae	*Rhododendron*	*Rhododendron selense*	JN046955
Eudicotyledons	Ericaceae	*Rhododendron*	*Rhododendron selense*	JN046956
Eudicotyledons	Ericaceae	*Rhododendron*	*Rhododendron selense*	JN046957
Eudicotyledons	Ericaceae	*Rhododendron*	*Rhododendron selense*	JN046958
Eudicotyledons	Solanaceae	*Solanum*	*Solanum lichtensteinii*	HM016421
Eudicotyledons	Solanaceae	*Solanum*	*Solanum lichtensteinii*	HM016426
Eudicotyledons	Oleaceae	*Fraxinus*	*Fraxinus berlandieriana*	HM367389
Eudicotyledons	Oleaceae	*Fraxinus*	*Fraxinus berlandieriana*	HM367390
Eudicotyledons	Oleaceae	*Fraxinus*	*Fraxinus berlandieriana*	HM367391
Eudicotyledons	Oleaceae	*Fraxinus*	*Fraxinus bungeana*	HM367394
Eudicotyledons	Oleaceae	*Fraxinus*	*Fraxinus bungeana*	HM367395
Eudicotyledons	Oleaceae	*Fraxinus*	*Fraxinus bungeana*	HM367396
Eudicotyledons	Oleaceae	*Fraxinus*	*Fraxinus lanuginosa*	HM367443
Eudicotyledons	Oleaceae	*Fraxinus*	*Fraxinus lanuginosa*	HM367444
Eudicotyledons	Oleaceae	*Fraxinus*	*Fraxinus lanuginosa*	HM367445
Eudicotyledons	Oleaceae	*Fraxinus*	*Fraxinus lanuginosa*	HM367446
Eudicotyledons	Oleaceae	*Fraxinus*	*Fraxinus lanuginosa*	HM367447
Eudicotyledons	Oleaceae	*Fraxinus*	*Fraxinus lanuginosa*	HM367448
Eudicotyledons	Oleaceae	*Fraxinus*	*Fraxinus lanuginosa*	HM367449
Eudicotyledons	Oleaceae	*Fraxinus*	*Fraxinus lanuginosa*	HM367450
Eudicotyledons	Oleaceae	*Fraxinus*	*Fraxinus lanuginosa*	HM367451
Eudicotyledons	Oleaceae	*Fraxinus*	*Fraxinus lanuginosa*	HM367452
Eudicotyledons	Oleaceae	*Fraxinus*	*Fraxinus lanuginosa*	HM367453
Eudicotyledons	Oleaceae	*Fraxinus*	*Fraxinus lanuginosa*	HM367454
Eudicotyledons	Oleaceae	*Fraxinus*	*Fraxinus lanuginosa*	HM367455
Eudicotyledons	Oleaceae	*Fraxinus*	*Fraxinus lanuginosa*	HM367456
Eudicotyledons	Oleaceae	*Fraxinus*	*Fraxinus lanuginosa*	HM367457
Eudicotyledons	Oleaceae	*Fraxinus*	*Fraxinus lanuginosa*	HM367458
Eudicotyledons	Oleaceae	*Fraxinus*	*Fraxinus lanuginosa*	HM367460
Eudicotyledons	Oleaceae	*Fraxinus*	*Fraxinus lanuginosa*	HM367461
Eudicotyledons	Oleaceae	*Fraxinus*	*Fraxinus lanuginosa*	HM367462
Eudicotyledons	Oleaceae	*Fraxinus*	*Fraxinus lanuginosa*	HM367463
Eudicotyledons	Oleaceae	*Fraxinus*	*Fraxinus lanuginosa*	HM367464
Eudicotyledons	Oleaceae	*Fraxinus*	*Fraxinus lanuginosa*	HM367465
Eudicotyledons	Oleaceae	*Fraxinus*	*Fraxinus lanuginosa*	HM367466
Eudicotyledons	Oleaceae	*Fraxinus*	*Fraxinus uhdei*	HM367567
Eudicotyledons	Oleaceae	*Fraxinus*	*Fraxinus uhdei*	HM367568

Eudicotyledons	Oleaceae	*Fraxinus*	*Fraxinus uhdei*	HM367569
Eudicotyledons	Oleaceae	*Fraxinus*	*Fraxinus uhdei*	HM367570
Eudicotyledons	Loasaceae	*Mentzelia*	*Mentzelia jonesii*	FJ918158
Eudicotyledons	Loasaceae	*Mentzelia*	*Mentzelia jonesii*	FJ918159
Eudicotyledons	Loasaceae	*Mentzelia*	*Mentzelia jonesii*	FJ918160
Eudicotyledons	Loasaceae	*Mentzelia*	*Mentzelia jonesii*	FJ918161
Eudicotyledons	Loasaceae	*Mentzelia*	*Mentzelia jonesii*	FJ918162
Eudicotyledons	Loasaceae	*Mentzelia*	*Mentzelia thompsonii*	FJ918203
Eudicotyledons	Loasaceae	*Mentzelia*	*Mentzelia thompsonii*	FJ918204
Eudicotyledons	Loasaceae	*Mentzelia*	*Mentzelia thompsonii*	FJ918205
Eudicotyledons	Cucurbitaceae	*Momordica*	*Momordica camerounensis*	GQ162988
Eudicotyledons	Cucurbitaceae	*Momordica*	*Momordica camerounensis*	GQ162989
Eudicotyledons	Oleaceae	*Fraxinus*	*Fraxinus griffithii*	HM367434
Eudicotyledons	Oleaceae	*Fraxinus*	*Fraxinus griffithii*	HM367435
Eudicotyledons	Oleaceae	*Fraxinus*	*Fraxinus griffithii*	HM367436
Eudicotyledons	Oleaceae	*Fraxinus*	*Fraxinus malacophylla*	HM367485
Eudicotyledons	Oleaceae	*Fraxinus*	*Fraxinus malacophylla*	HM367486
Eudicotyledons	Lamiaceae	*Origanum*	*Origanum sp. MIB zpl(1)*	FR726126
Eudicotyledons	Lamiaceae	*Origanum*	*Origanum sp. MIB zpl(1)*	FR726127
Eudicotyledons	Lamiaceae	*Origanum*	*Origanum sp. MIB zpl(1)*	FR726128
Eudicotyledons	Brassicaceae	*Capsella*	*Capsella orientalis*	FR822347
Eudicotyledons	Brassicaceae	*Capsella*	*Capsella orientalis*	FR822348
Eudicotyledons	Brassicaceae	*Capsella*	*Capsella orientalis*	FR822349
Eudicotyledons	Ericaceae	*Rhododendron*	*Rhododendron traillianum*	JN046991
Eudicotyledons	Ericaceae	*Rhododendron*	*Rhododendron traillianum*	JN046992
Eudicotyledons	Ericaceae	*Rhododendron*	*Rhododendron traillianum*	JN046993
Eudicotyledons	Begoniaceae	*Begonia*	*Begonia purpureofolia*	JN044120
Eudicotyledons	Begoniaceae	*Begonia*	*Begonia purpureofolia*	JN044121
Eudicotyledons	Begoniaceae	*Begonia*	*Begonia purpureofolia*	JN044122
Eudicotyledons	Begoniaceae	*Begonia*	*Begonia purpureofolia*	JN044123
Eudicotyledons	Begoniaceae	*Begonia*	*Begonia purpureofolia*	JN044124
Eudicotyledons	Begoniaceae	*Begonia*	*Begonia purpureofolia*	JN044125
Eudicotyledons	Rubiaceae	*Oldenlandia*	*Oldenlandia chrysotricha*	JF699778
Eudicotyledons	Rubiaceae	*Oldenlandia*	*Oldenlandia chrysotricha*	JF699779
Eudicotyledons	Rubiaceae	*Oldenlandia*	*Oldenlandia chrysotricha*	JF699780
Eudicotyledons	Rubiaceae	*Oldenlandia*	*Oldenlandia chrysotricha*	JF699781
Eudicotyledons	Rubiaceae	*Oldenlandia*	*Oldenlandia chrysotricha*	JN044767
Eudicotyledons	Rubiaceae	*Oldenlandia*	*Oldenlandia chrysotricha*	JN044768
Eudicotyledons	Fabaceae	*Schizolobium*	*Schizolobium parahyba*	GQ167769
Eudicotyledons	Fabaceae	*Schizolobium*	*Schizolobium parahyba*	AF524985
Eudicotyledons	Fabaceae	*Schizolobium*	*Schizolobium parahyba*	FJ668606
Eudicotyledons	Fabaceae	*Schizolobium*	*Schizolobium parahyba*	FJ668607
Eudicotyledons	Fabaceae	*Schizolobium*	*Schizolobium parahyba*	FJ668610
Eudicotyledons	Fabaceae	*Schizolobium*	*Schizolobium parahyba*	FJ668611
Eudicotyledons	Fabaceae	*Schizolobium*	*Schizolobium parahyba*	FJ668612
Eudicotyledons	Fabaceae	*Schizolobium*	*Schizolobium parahyba*	FJ668613
Eudicotyledons	Fabaceae	*Schizolobium*	*Schizolobium parahyba*	GQ982357
Eudicotyledons	Gesneriaceae	*Cyrtandra*	*Cyrtandra hawaiensis*	GQ475133
Eudicotyledons	Gesneriaceae	*Cyrtandra*	*Cyrtandra hawaiensis*	GQ475158
Eudicotyledons	Gesneriaceae	*Cyrtandra*	*Cyrtandra hawaiensis*	GQ475159
Eudicotyledons	Gesneriaceae	*Cyrtandra*	*Cyrtandra hawaiensis*	EU920008
Eudicotyledons	Lamiaceae	*Thymus*	*Thymus vulgaris*	FR726148
Eudicotyledons	Lamiaceae	*Thymus*	*Thymus vulgaris*	FR726149

Eudicotyledons	Lamiaceae	*Thymus*	*Thymus vulgaris*	FR726150
Eudicotyledons	Lamiaceae	*Thymus*	*Thymus vulgaris*	AY679164
Eudicotyledons	Polygonaceae	*Polygonum*	*Polygonum chinense*	GQ435161
Eudicotyledons	Polygonaceae	*Polygonum*	*Polygonum chinense*	JN407039
Eudicotyledons	Polygonaceae	*Polygonum*	*Polygonum chinense*	JN407040
Eudicotyledons	Polygonaceae	*Polygonum*	*Polygonum chinense*	JN407041
Eudicotyledons	Polygonaceae	*Polygonum*	*Polygonum chinense*	JN407042
Eudicotyledons	Polygonaceae	*Polygonum*	*Polygonum chinense*	EU554053
Eudicotyledons	Apiaceae	*Osmorhiza*	*Osmorhiza occidentalis*	JN045666
Eudicotyledons	Apiaceae	*Osmorhiza*	*Osmorhiza occidentalis*	JN045667
Eudicotyledons	Apiaceae	*Osmorhiza*	*Osmorhiza occidentalis*	JN045668
Eudicotyledons	Apiaceae	*Osmorhiza*	*Osmorhiza occidentalis*	JN045669
Eudicotyledons	Gesneriaceae	*Cyrtandra*	*Cyrtandra compressa*	GQ475128
Eudicotyledons	Gesneriaceae	*Cyrtandra*	*Cyrtandra compressa*	EU920029
Eudicotyledons	Gentianaceae	*Gentianopsis*	*Gentianopsis crinita*	HM460854
Eudicotyledons	Gentianaceae	*Gentianopsis*	*Gentianopsis crinita*	HM460855
Eudicotyledons	Gentianaceae	*Gentianopsis*	*Gentianopsis crinita*	HM460856
Eudicotyledons	Ranunculaceae	*Aconitum*	*Aconitum carmichaelii*	GQ337742
Eudicotyledons	Ranunculaceae	*Aconitum*	*Aconitum carmichaelii*	GQ337743
Eudicotyledons	Ranunculaceae	*Aconitum*	*Aconitum carmichaelii*	GQ337744
Eudicotyledons	Ranunculaceae	*Aconitum*	*Aconitum carmichaelii*	GQ337745
Eudicotyledons	Ranunculaceae	*Aconitum*	*Aconitum carmichaelii*	GQ337746
Eudicotyledons	Ranunculaceae	*Aconitum*	*Aconitum carmichaelii*	GQ337747
Eudicotyledons	Ranunculaceae	*Aconitum*	*Aconitum carmichaelii*	GQ337748
Eudicotyledons	Ranunculaceae	*Aconitum*	*Aconitum carmichaelii*	GQ337749
Eudicotyledons	Ranunculaceae	*Aconitum*	*Aconitum carmichaelii*	GQ337750
Eudicotyledons	Ranunculaceae	*Aconitum*	*Aconitum carmichaelii*	GQ337765
Eudicotyledons	Ranunculaceae	*Aconitum*	*Aconitum carmichaelii*	GQ337766
Eudicotyledons	Ranunculaceae	*Aconitum*	*Aconitum carmichaelii*	GQ337767
Eudicotyledons	Ranunculaceae	*Aconitum*	*Aconitum carmichaelii*	GQ337768
Eudicotyledons	Ranunculaceae	*Aconitum*	*Aconitum carmichaelii*	GQ337769
Eudicotyledons	Ranunculaceae	*Aconitum*	*Aconitum carmichaelii*	GQ337770
Eudicotyledons	Ranunculaceae	*Aconitum*	*Aconitum carmichaelii*	GQ337771
Eudicotyledons	Ranunculaceae	*Aconitum*	*Aconitum carmichaelii*	GQ337772
Eudicotyledons	Ranunculaceae	*Aconitum*	*Aconitum carmichaelii*	GQ337773
Eudicotyledons	Ranunculaceae	*Aconitum*	*Aconitum carmichaelii*	GQ337778
Eudicotyledons	Ranunculaceae	*Aconitum*	*Aconitum carmichaelii*	GQ337810
Eudicotyledons	Ranunculaceae	*Aconitum*	*Aconitum carmichaelii*	GQ337811
Eudicotyledons	Ranunculaceae	*Aconitum*	*Aconitum carmichaelii*	GQ337823
Eudicotyledons	Ranunculaceae	*Aconitum*	*Aconitum carmichaelii*	GQ337850
Eudicotyledons	Ranunculaceae	*Aconitum*	*Aconitum carmichaelii*	GQ337851
Eudicotyledons	Ranunculaceae	*Aconitum*	*Aconitum carmichaelii*	GQ337852
Eudicotyledons	Ranunculaceae	*Aconitum*	*Aconitum carmichaelii*	GQ337853
Eudicotyledons	Ranunculaceae	*Aconitum*	*Aconitum carmichaelii*	FJ821166
Eudicotyledons	Ranunculaceae	*Aconitum*	*Aconitum carmichaelii*	FJ821167
Eudicotyledons	Ranunculaceae	*Aconitum*	*Aconitum lycoctonum*	AF216560
Eudicotyledons	Ranunculaceae	*Aconitum*	*Aconitum lycoctonum*	AF216561
Eudicotyledons	Ranunculaceae	*Aconitum*	*Aconitum lycoctonum*	AF216562
Eudicotyledons	Ranunculaceae	*Aconitum*	*Aconitum lycoctonum*	AF216563
Eudicotyledons	Ranunculaceae	*Aconitum*	*Aconitum lycoctonum*	AF216564
Eudicotyledons	Ranunculaceae	*Aconitum*	*Aconitum lycoctonum*	AF216565
Eudicotyledons	Ranunculaceae	*Aconitum*	*Aconitum lycoctonum*	AF216566
Eudicotyledons	Ranunculaceae	*Aconitum*	*Aconitum lycoctonum*	AF216568

Eudicotyledons	Ranunculaceae	*Aconitum*	*Aconitum lycoctonum*	AF216569
Eudicotyledons	Ranunculaceae	*Aconitum*	*Aconitum lycoctonum*	AF216571
Eudicotyledons	Ranunculaceae	*Aconitum*	*Aconitum lycoctonum*	AF216573
Eudicotyledons	Ranunculaceae	*Aconitum*	*Aconitum lycoctonum*	AF216574
Eudicotyledons	Ranunculaceae	*Aconitum*	*Aconitum lycoctonum*	FN675818
Eudicotyledons	Ranunculaceae	*Aconitum*	*Aconitum napellus*	AF216567
Eudicotyledons	Ranunculaceae	*Aconitum*	*Aconitum napellus*	FN675819
Eudicotyledons	Primulaceae	*Primula*	*Primula secundiflora*	HM018499
Eudicotyledons	Primulaceae	*Primula*	*Primula secundiflora*	HM018500
Eudicotyledons	Primulaceae	*Primula*	*Primula secundiflora*	HM018501
Eudicotyledons	Primulaceae	*Primula*	*Primula secundiflora*	HM018502
Eudicotyledons	Primulaceae	*Primula*	*Primula secundiflora*	HM018503
Eudicotyledons	Primulaceae	*Primula*	*Primula secundiflora*	HM018504
Eudicotyledons	Primulaceae	*Primula*	*Primula secundiflora*	HM018505
Eudicotyledons	Primulaceae	*Primula*	*Primula secundiflora*	HM018506
Eudicotyledons	Primulaceae	*Primula*	*Primula secundiflora*	HM018507
Eudicotyledons	Primulaceae	*Primula*	*Primula secundiflora*	HM018508
Eudicotyledons	Primulaceae	*Primula*	*Primula secundiflora*	HM018509
Eudicotyledons	Primulaceae	*Primula*	*Primula secundiflora*	HM018510
Eudicotyledons	Primulaceae	*Primula*	*Primula secundiflora*	HM018511
Eudicotyledons	Fabaceae	*Caesalpinia*	*Caesalpinia pannosa*	DQ501959
Eudicotyledons	Fabaceae	*Caesalpinia*	*Caesalpinia pannosa*	DQ208844
Eudicotyledons	Fabaceae	*Caesalpinia*	*Caesalpinia pannosa*	DQ208845
Eudicotyledons	Fabaceae	*Caesalpinia*	*Caesalpinia pannosa*	DQ208846
Eudicotyledons	Fabaceae	*Caesalpinia*	*Caesalpinia pannosa*	DQ208847
Eudicotyledons	Fabaceae	*Caesalpinia*	*Caesalpinia pannosa*	DQ208848
Eudicotyledons	Fabaceae	*Caesalpinia*	*Caesalpinia pannosa*	DQ208849
Eudicotyledons	Fabaceae	*Caesalpinia*	*Caesalpinia pannosa*	DQ208850
Eudicotyledons	Fabaceae	*Caesalpinia*	*Caesalpinia pannosa*	DQ208851
Eudicotyledons	Fabaceae	*Caesalpinia*	*Caesalpinia pannosa*	DQ208852
Eudicotyledons	Fabaceae	*Caesalpinia*	*Caesalpinia pannosa*	DQ208853
Eudicotyledons	Fabaceae	*Caesalpinia*	*Caesalpinia pannosa*	DQ208854
Eudicotyledons	Fabaceae	*Caesalpinia*	*Caesalpinia pannosa*	DQ208855
Eudicotyledons	Primulaceae	*Primula*	*Primula poissonii*	HM018485
Eudicotyledons	Primulaceae	*Primula*	*Primula poissonii*	HM018486
Eudicotyledons	Primulaceae	*Primula*	*Primula poissonii*	HM018487
Eudicotyledons	Primulaceae	*Primula*	*Primula poissonii*	HM018488
Eudicotyledons	Primulaceae	*Primula*	*Primula poissonii*	HM018489
Eudicotyledons	Primulaceae	*Primula*	*Primula poissonii*	HM018490
Eudicotyledons	Primulaceae	*Primula*	*Primula poissonii*	HM018491
Eudicotyledons	Primulaceae	*Primula*	*Primula poissonii*	HM018492
Eudicotyledons	Primulaceae	*Primula*	*Primula poissonii*	HM018493
Eudicotyledons	Primulaceae	*Primula*	*Primula poissonii*	HM018494
Eudicotyledons	Primulaceae	*Primula*	*Primula poissonii*	HM018495
Eudicotyledons	Primulaceae	*Primula*	*Primula poissonii*	HM018496
Eudicotyledons	Primulaceae	*Primula*	*Primula poissonii*	HM018497
Eudicotyledons	Primulaceae	*Primula*	*Primula poissonii*	HM018498
Eudicotyledons	Fabaceae	*Caesalpinia*	*Caesalpinia hintonii*	DQ208809
Eudicotyledons	Fabaceae	*Caesalpinia*	*Caesalpinia hintonii*	DQ208810
Eudicotyledons	Fabaceae	*Caesalpinia*	*Caesalpinia hintonii*	DQ208811
Eudicotyledons	Fabaceae	*Caesalpinia*	*Caesalpinia hintonii*	DQ208812
Eudicotyledons	Fabaceae	*Caesalpinia*	*Caesalpinia hintonii*	DQ208813
Eudicotyledons	Fabaceae	*Caesalpinia*	*Caesalpinia hintonii*	DQ208814

Eudicotyledons	Fabaceae	*Caesalpinia*	*Caesalpinia hintonii*	DQ208815
Eudicotyledons	Fabaceae	*Caesalpinia*	*Caesalpinia hintonii*	DQ208816
Eudicotyledons	Fabaceae	*Caesalpinia*	*Caesalpinia hintonii*	DQ208817
Eudicotyledons	Fabaceae	*Caesalpinia*	*Caesalpinia hintonii*	DQ208818
Eudicotyledons	Fabaceae	*Caesalpinia*	*Caesalpinia hintonii*	DQ208819
Eudicotyledons	Fabaceae	*Caesalpinia*	*Caesalpinia hintonii*	DQ208820
Eudicotyledons	Fabaceae	*Caesalpinia*	*Caesalpinia hintonii*	DQ208821
Eudicotyledons	Fabaceae	*Caesalpinia*	*Caesalpinia hintonii*	DQ208822
Eudicotyledons	Fabaceae	*Caesalpinia*	*Caesalpinia hintonii*	DQ208823
Eudicotyledons	Fabaceae	*Caesalpinia*	*Caesalpinia hintonii*	DQ208824
Eudicotyledons	Asteraceae	*Hypochaeris*	*Hypochaeris incana*	FM994954
Eudicotyledons	Asteraceae	*Hypochaeris*	*Hypochaeris incana*	FM994955
Eudicotyledons	Asteraceae	*Hypochaeris*	*Hypochaeris incana*	FM994956
Eudicotyledons	Asteraceae	*Hypochaeris*	*Hypochaeris incana*	FM994957
Eudicotyledons	Asteraceae	*Hypochaeris*	*Hypochaeris incana*	FM994958
Eudicotyledons	Asteraceae	*Hypochaeris*	*Hypochaeris incana*	FM994959
Eudicotyledons	Asteraceae	*Hypochaeris*	*Hypochaeris incana*	FM994960
Eudicotyledons	Asteraceae	*Hypochaeris*	*Hypochaeris incana*	FM994961
Eudicotyledons	Asteraceae	*Hypochaeris*	*Hypochaeris incana*	FM994962
Eudicotyledons	Asteraceae	*Hypochaeris*	*Hypochaeris incana*	FM994963
Eudicotyledons	Asteraceae	*Hypochaeris*	*Hypochaeris incana*	FM994964
Eudicotyledons	Asteraceae	*Hypochaeris*	*Hypochaeris incana*	FM994965
Eudicotyledons	Asteraceae	*Hypochaeris*	*Hypochaeris incana*	FM994966
Eudicotyledons	Asteraceae	*Hypochaeris*	*Hypochaeris incana*	FM994967
Eudicotyledons	Asteraceae	*Hypochaeris*	*Hypochaeris incana*	FM994968
Eudicotyledons	Asteraceae	*Hypochaeris*	*Hypochaeris incana*	FM994969
Eudicotyledons	Asteraceae	*Hypochaeris*	*Hypochaeris incana*	FM994970
Eudicotyledons	Asteraceae	*Hypochaeris*	*Hypochaeris incana*	FM994971
Eudicotyledons	Asteraceae	*Hypochaeris*	*Hypochaeris incana*	FM994972
Eudicotyledons	Asteraceae	*Hypochaeris*	*Hypochaeris incana*	FM994973
Eudicotyledons	Asteraceae	*Hypochaeris*	*Hypochaeris incana*	FM994974
Eudicotyledons	Asteraceae	*Hypochaeris*	*Hypochaeris incana*	FM994975
Eudicotyledons	Asteraceae	*Hypochaeris*	*Hypochaeris incana*	FM994976
Eudicotyledons	Asteraceae	*Hypochaeris*	*Hypochaeris incana*	FM994977
Eudicotyledons	Asteraceae	*Hypochaeris*	*Hypochaeris incana*	FM994978
Eudicotyledons	Asteraceae	*Hypochaeris*	*Hypochaeris incana*	FM994979
Eudicotyledons	Asteraceae	*Hypochaeris*	*Hypochaeris incana*	FM994980
Eudicotyledons	Asteraceae	*Hypochaeris*	*Hypochaeris incana*	FM994981
Eudicotyledons	Asteraceae	*Hypochaeris*	*Hypochaeris incana*	FM994982
Eudicotyledons	Asteraceae	*Hypochaeris*	*Hypochaeris incana*	FM994983
Eudicotyledons	Asteraceae	*Hypochaeris*	*Hypochaeris incana*	FM994984
Eudicotyledons	Asteraceae	*Hypochaeris*	*Hypochaeris incana*	FM994985
Eudicotyledons	Asteraceae	*Hypochaeris*	*Hypochaeris incana*	FM994986
Eudicotyledons	Asteraceae	*Hypochaeris*	*Hypochaeris incana*	FM994987
Eudicotyledons	Asteraceae	*Hypochaeris*	*Hypochaeris incana*	FM994988
Eudicotyledons	Asteraceae	*Hypochaeris*	*Hypochaeris incana*	FM994989
Eudicotyledons	Asteraceae	*Hypochaeris*	*Hypochaeris incana*	FM994990
Eudicotyledons	Asteraceae	*Hypochaeris*	*Hypochaeris incana*	FM994991
Eudicotyledons	Asteraceae	*Hypochaeris*	*Hypochaeris incana*	FM994992
Eudicotyledons	Asteraceae	*Hypochaeris*	*Hypochaeris incana*	FM994993
Eudicotyledons	Asteraceae	*Hypochaeris*	*Hypochaeris incana*	FM994994
Eudicotyledons	Asteraceae	*Hypochaeris*	*Hypochaeris incana*	FM994995
Eudicotyledons	Asteraceae	*Hypochaeris*	*Hypochaeris incana*	FN394275

Eudicotyledons	Asteraceae	*Hypochaeris*	*Hypochaeris incana*	FN394276
Eudicotyledons	Asteraceae	*Hypochaeris*	*Hypochaeris incana*	FN394277
Eudicotyledons	Asteraceae	*Hypochaeris*	*Hypochaeris incana*	FN394278
Eudicotyledons	Asteraceae	*Hypochaeris*	*Hypochaeris incana*	FN394279
Eudicotyledons	Asteraceae	*Hypochaeris*	*Hypochaeris incana*	FN394280
Eudicotyledons	Asteraceae	*Hypochaeris*	*Hypochaeris incana*	FN394281
Eudicotyledons	Asteraceae	*Hypochaeris*	*Hypochaeris incana*	FN394282
Eudicotyledons	Asteraceae	*Hypochaeris*	*Hypochaeris incana*	FN394283
Eudicotyledons	Asteraceae	*Hypochaeris*	*Hypochaeris incana*	FN394284
Eudicotyledons	Asteraceae	*Hypochaeris*	*Hypochaeris incana*	FN394285
Eudicotyledons	Asteraceae	*Hypochaeris*	*Hypochaeris incana*	FN394286
Eudicotyledons	Asteraceae	*Hypochaeris*	*Hypochaeris incana*	FN394287
Eudicotyledons	Asteraceae	*Hypochaeris*	*Hypochaeris incana*	FN394288
Eudicotyledons	Asteraceae	*Hypochaeris*	*Hypochaeris incana*	FN394289
Eudicotyledons	Asteraceae	*Hypochaeris*	*Hypochaeris incana*	FN394290
Eudicotyledons	Asteraceae	*Hypochaeris*	*Hypochaeris incana*	FN394291
Eudicotyledons	Asteraceae	*Hypochaeris*	*Hypochaeris incana*	FN394292
Eudicotyledons	Asteraceae	*Hypochaeris*	*Hypochaeris incana*	FN394293
Eudicotyledons	Asteraceae	*Hypochaeris*	*Hypochaeris incana*	FN394294
Eudicotyledons	Asteraceae	*Hypochaeris*	*Hypochaeris incana*	FN394295
Eudicotyledons	Asteraceae	*Hypochaeris*	*Hypochaeris incana*	FN394296
Eudicotyledons	Asteraceae	*Hypochaeris*	*Hypochaeris incana*	FN394297
Eudicotyledons	Asteraceae	*Hypochaeris*	*Hypochaeris incana*	FN394298
Eudicotyledons	Asteraceae	*Hypochaeris*	*Hypochaeris incana*	FN394299
Eudicotyledons	Asteraceae	*Hypochaeris*	*Hypochaeris incana*	FN394300
Eudicotyledons	Asteraceae	*Hypochaeris*	*Hypochaeris incana*	FN394301
Eudicotyledons	Asteraceae	*Hypochaeris*	*Hypochaeris incana*	FN394302
Eudicotyledons	Asteraceae	*Hypochaeris*	*Hypochaeris incana*	FN394303
Eudicotyledons	Asteraceae	*Hypochaeris*	*Hypochaeris incana*	FN394304
Eudicotyledons	Asteraceae	*Hypochaeris*	*Hypochaeris incana*	FN394305
Eudicotyledons	Asteraceae	*Hypochaeris*	*Hypochaeris incana*	FN394306
Eudicotyledons	Asteraceae	*Hypochaeris*	*Hypochaeris incana*	FN394307
Eudicotyledons	Ranunculaceae	*Aconitum*	*Aconitum scaposum*	GQ337739
Eudicotyledons	Ranunculaceae	*Aconitum*	*Aconitum scaposum*	GQ337740
Eudicotyledons	Ranunculaceae	*Aconitum*	*Aconitum scaposum*	GQ337741
Eudicotyledons	Ranunculaceae	*Aconitum*	*Aconitum scaposum*	JN043766
Eudicotyledons	Ranunculaceae	*Aconitum*	*Aconitum scaposum*	JN043767
Eudicotyledons	Ranunculaceae	*Aconitum*	*Aconitum scaposum*	JN043768
Eudicotyledons	Ranunculaceae	*Aconitum*	*Aconitum scaposum*	JN043769
Eudicotyledons	Ranunculaceae	*Aconitum*	*Aconitum gymnandrum*	FJ418149
Eudicotyledons	Ranunculaceae	*Aconitum*	*Aconitum gymnandrum*	FJ418150
Eudicotyledons	Ranunculaceae	*Aconitum*	*Aconitum gymnandrum*	FJ418151
Eudicotyledons	Ranunculaceae	*Aconitum*	*Aconitum gymnandrum*	GQ337734
Eudicotyledons	Ranunculaceae	*Aconitum*	*Aconitum gymnandrum*	GQ337735
Eudicotyledons	Ranunculaceae	*Aconitum*	*Aconitum gymnandrum*	GQ337736
Eudicotyledons	Lamiaceae	*Thymus*	*Thymus quinquecostatus*	AY281310
Eudicotyledons	Lamiaceae	*Thymus*	*Thymus quinquecostatus*	AY281311
Eudicotyledons	Lamiaceae	*Thymus*	*Thymus quinquecostatus*	AY281312
Eudicotyledons	Lamiaceae	*Thymus*	*Thymus quinquecostatus*	AY281313
Eudicotyledons	Lamiaceae	*Thymus*	*Thymus quinquecostatus*	AY281314
Eudicotyledons	Lamiaceae	*Thymus*	*Thymus quinquecostatus*	AY281315
Eudicotyledons	Lamiaceae	*Thymus*	*Thymus quinquecostatus*	AY443427
Eudicotyledons	Lamiaceae	*Thymus*	*Thymus quinquecostatus*	AY443428

Eudicotyledons	Lamiaceae	*Thymus*	*Thymus quinquecostatus*	AY443429
Eudicotyledons	Lamiaceae	*Thymus*	*Thymus quinquecostatus*	AY443430
Eudicotyledons	Lamiaceae	*Thymus*	*Thymus quinquecostatus*	AY443431
Eudicotyledons	Lamiaceae	*Thymus*	*Thymus quinquecostatus*	HM590114
Eudicotyledons	Lamiaceae	*Thymus*	*Thymus magnus*	AY281316
Eudicotyledons	Lamiaceae	*Thymus*	*Thymus magnus*	AY281317
Eudicotyledons	Lamiaceae	*Thymus*	*Thymus magnus*	AY443432
Eudicotyledons	Lamiaceae	*Thymus*	*Thymus magnus*	AY443433
Eudicotyledons	Ranunculaceae	*Aconitum*	*Aconitum kusnezoffii*	GQ337723
Eudicotyledons	Ranunculaceae	*Aconitum*	*Aconitum kusnezoffii*	GQ337724
Eudicotyledons	Ranunculaceae	*Aconitum*	*Aconitum kusnezoffii*	GQ337725
Eudicotyledons	Ranunculaceae	*Aconitum*	*Aconitum kusnezoffii*	GQ337789
Eudicotyledons	Ranunculaceae	*Aconitum*	*Aconitum kusnezoffii*	GQ337790
Eudicotyledons	Ranunculaceae	*Aconitum*	*Aconitum kusnezoffii*	GQ337798
Eudicotyledons	Ranunculaceae	*Aconitum*	*Aconitum kusnezoffii*	GQ337799
Eudicotyledons	Ranunculaceae	*Aconitum*	*Aconitum kusnezoffii*	GQ337812
Eudicotyledons	Ranunculaceae	*Aconitum*	*Aconitum kusnezoffii*	GQ337813
Eudicotyledons	Ranunculaceae	*Aconitum*	*Aconitum kusnezoffii*	GQ337814
Eudicotyledons	Ranunculaceae	*Aconitum*	*Aconitum kusnezoffii*	GQ337836
Eudicotyledons	Ranunculaceae	*Aconitum*	*Aconitum kusnezoffii*	GQ337837
Eudicotyledons	Ranunculaceae	*Aconitum*	*Aconitum kusnezoffii*	GQ337838
Eudicotyledons	Ranunculaceae	*Aconitum*	*Aconitum kusnezoffii*	GQ337839
Eudicotyledons	Ranunculaceae	*Aconitum*	*Aconitum kusnezoffii*	GQ337848
Eudicotyledons	Ranunculaceae	*Aconitum*	*Aconitum kusnezoffii*	GQ337849
Eudicotyledons	Ranunculaceae	*Aconitum*	*Aconitum kusnezoffii*	FJ821181
Eudicotyledons	Ranunculaceae	*Aconitum*	*Aconitum kusnezoffii*	FJ821182
Eudicotyledons	Ranunculaceae	*Aconitum*	*Aconitum kusnezoffii*	FJ821183
Eudicotyledons	Primulaceae	*Primula*	*Primula amethystina*	JN046466
Eudicotyledons	Primulaceae	*Primula*	*Primula amethystina*	JN046467
Eudicotyledons	Primulaceae	*Primula*	*Primula amethystina*	JN046468
Eudicotyledons	Orobanchaceae	*Agalinis*	*Agalinis acuta*	GU943532
Eudicotyledons	Orobanchaceae	*Agalinis*	*Agalinis acuta*	EU827882
Eudicotyledons	Orobanchaceae	*Agalinis*	*Agalinis acuta*	EU827883
Eudicotyledons	Orobanchaceae	*Agalinis*	*Agalinis acuta*	EU827884
Eudicotyledons	Orobanchaceae	*Agalinis*	*Agalinis acuta*	EU827885
Eudicotyledons	Orobanchaceae	*Agalinis*	*Agalinis acuta*	EU827886
Eudicotyledons	Orobanchaceae	*Agalinis*	*Agalinis acuta*	EU827887
Eudicotyledons	Orobanchaceae	*Agalinis*	*Agalinis acuta*	EU827888
Eudicotyledons	Orobanchaceae	*Agalinis*	*Agalinis acuta*	EU827889
Eudicotyledons	Orobanchaceae	*Agalinis*	*Agalinis acuta*	EU827890
Eudicotyledons	Orobanchaceae	*Agalinis*	*Agalinis fasciculata*	EU827904
Eudicotyledons	Orobanchaceae	*Agalinis*	*Agalinis fasciculata*	EU827905
Eudicotyledons	Orobanchaceae	*Agalinis*	*Agalinis fasciculata*	EU827906
Eudicotyledons	Orobanchaceae	*Agalinis*	*Agalinis obtusifolia*	GU943536
Eudicotyledons	Orobanchaceae	*Agalinis*	*Agalinis obtusifolia*	GU943537
Eudicotyledons	Orobanchaceae	*Agalinis*	*Agalinis obtusifolia*	GU943538
Eudicotyledons	Orobanchaceae	*Agalinis*	*Agalinis obtusifolia*	GU943539
Eudicotyledons	Orobanchaceae	*Agalinis*	*Agalinis obtusifolia*	EU827925
Eudicotyledons	Orobanchaceae	*Agalinis*	*Agalinis obtusifolia*	EU827926
Eudicotyledons	Orobanchaceae	*Agalinis*	*Agalinis obtusifolia*	EU827928
Eudicotyledons	Orobanchaceae	*Agalinis*	*Agalinis obtusifolia*	EU827929
Eudicotyledons	Orobanchaceae	*Agalinis*	*Agalinis obtusifolia*	EU827930
Eudicotyledons	Orobanchaceae	*Agalinis*	*Agalinis tenella*	GU943542

Eudicotyledons	Orobanchaceae	*Agalinis*	*Agalinis tenella*	GU943543
Eudicotyledons	Orobanchaceae	*Agalinis*	*Agalinis tenella*	EU827927
Eudicotyledons	Orobanchaceae	*Agalinis*	*Agalinis tenella*	EU827951
Eudicotyledons	Orobanchaceae	*Agalinis*	*Agalinis tenella*	EU827952
Eudicotyledons	Orobanchaceae	*Agalinis*	*Agalinis tenella*	EU827953
Eudicotyledons	Orobanchaceae	*Agalinis*	*Agalinis tenella*	EU827954
Eudicotyledons	Orobanchaceae	*Agalinis*	*Agalinis tenella*	EU827955
Eudicotyledons	Ranunculaceae	*Aconitum*	*Aconitum alboviolaceum*	GQ337824
Eudicotyledons	Ranunculaceae	*Aconitum*	*Aconitum alboviolaceum*	GQ337825
Eudicotyledons	Ranunculaceae	*Aconitum*	*Aconitum alboviolaceum*	GQ337826
Eudicotyledons	Ranunculaceae	*Aconitum*	*Aconitum alboviolaceum*	GQ337830
Eudicotyledons	Ranunculaceae	*Aconitum*	*Aconitum alboviolaceum*	GQ337831
Eudicotyledons	Ranunculaceae	*Aconitum*	*Aconitum alboviolaceum*	GQ337832
Eudicotyledons	Ranunculaceae	*Aconitum*	*Aconitum alboviolaceum*	GQ337833
Eudicotyledons	Ranunculaceae	*Aconitum*	*Aconitum alboviolaceum*	GQ337834
Eudicotyledons	Ranunculaceae	*Aconitum*	*Aconitum alboviolaceum*	GQ337835
Eudicotyledons	Ranunculaceae	*Aconitum*	*Aconitum alboviolaceum*	JN043731
Eudicotyledons	Ranunculaceae	*Aconitum*	*Aconitum alboviolaceum*	JN043732
Eudicotyledons	Ranunculaceae	*Aconitum*	*Aconitum alboviolaceum*	JN043733
Eudicotyledons	Ranunculaceae	*Aconitum*	*Aconitum alboviolaceum*	JN043734
Eudicotyledons	Ranunculaceae	*Aconitum*	*Aconitum alboviolaceum*	JN043735
Eudicotyledons	Phyllanthaceae	*Phyllanthus*	*Phyllanthus amarus*	GU598561
Eudicotyledons	Phyllanthaceae	*Phyllanthus*	*Phyllanthus amarus*	GU598562
Eudicotyledons	Phyllanthaceae	*Phyllanthus*	*Phyllanthus amarus*	GU598563
Eudicotyledons	Phyllanthaceae	*Phyllanthus*	*Phyllanthus amarus*	GU598564
Eudicotyledons	Phyllanthaceae	*Phyllanthus*	*Phyllanthus amarus*	GU598565
Eudicotyledons	Phyllanthaceae	*Phyllanthus*	*Phyllanthus amarus*	GU598577
Eudicotyledons	Phyllanthaceae	*Phyllanthus*	*Phyllanthus amarus*	GQ409816
Eudicotyledons	Phyllanthaceae	*Phyllanthus*	*Phyllanthus amarus*	GQ409819
Eudicotyledons	Phyllanthaceae	*Phyllanthus*	*Phyllanthus amarus*	GQ409820
Eudicotyledons	Gesneriaceae	*Cyrtandra*	*Cyrtandra laxiflora*	GQ475161
Eudicotyledons	Gesneriaceae	*Cyrtandra*	*Cyrtandra laxiflora*	EU920007
Eudicotyledons	Gesneriaceae	*Cyrtandra*	*Cyrtandra samoensis*	GQ475122
Eudicotyledons	Gesneriaceae	*Cyrtandra*	*Cyrtandra samoensis*	GQ475148
Eudicotyledons	Gesneriaceae	*Cyrtandra*	*Cyrtandra samoensis*	GQ475150
Eudicotyledons	Gesneriaceae	*Cyrtandra*	*Cyrtandra samoensis*	EU920001
Eudicotyledons	Gesneriaceae	*Cyrtandra*	*Cyrtandra samoensis*	EU920002
Eudicotyledons	Brassicaceae	*Cardamine*	*Cardamine nipponica*	AB365513
Eudicotyledons	Brassicaceae	*Cardamine*	*Cardamine nipponica*	AB365514
Eudicotyledons	Brassicaceae	*Cardamine*	*Cardamine nipponica*	AB365515
Eudicotyledons	Brassicaceae	*Cardamine*	*Cardamine nipponica*	AB365516
Eudicotyledons	Fabaceae	*Caesalpinia*	*Caesalpinia macvaughii*	DQ208836
Eudicotyledons	Fabaceae	*Caesalpinia*	*Caesalpinia macvaughii*	DQ208837
Eudicotyledons	Fabaceae	*Caesalpinia*	*Caesalpinia macvaughii*	DQ208838
Eudicotyledons	Fabaceae	*Caesalpinia*	*Caesalpinia macvaughii*	DQ208839
Eudicotyledons	Fabaceae	*Caesalpinia*	*Caesalpinia macvaughii*	DQ208840
Eudicotyledons	Fabaceae	*Caesalpinia*	*Caesalpinia macvaughii*	DQ208841
Eudicotyledons	Fabaceae	*Caesalpinia*	*Caesalpinia macvaughii*	DQ208842
Eudicotyledons	Fabaceae	*Caesalpinia*	*Caesalpinia macvaughii*	DQ208843
Eudicotyledons	Polygonaceae	*Persicaria*	*Persicaria nepalensis*	EF653742
Eudicotyledons	Polygonaceae	*Persicaria*	*Persicaria nepalensis*	JN046437
Eudicotyledons	Polygonaceae	*Persicaria*	*Persicaria nepalensis*	JN046438
Eudicotyledons	Polygonaceae	*Persicaria*	*Persicaria nepalensis*	JN046439

Eudicotyledons	Rubiaceae	*Hedyotis*	*Hedyotis auricularia*	JF699765
Eudicotyledons	Rubiaceae	*Hedyotis*	*Hedyotis auricularia*	JF699766
Eudicotyledons	Rubiaceae	*Hedyotis*	*Hedyotis auricularia*	JN044758
Eudicotyledons	Rubiaceae	*Hedyotis*	*Hedyotis auricularia*	JN044759
Eudicotyledons	Orobanchaceae	*Agalinis*	*Agalinis decemloba*	GU943533
Eudicotyledons	Orobanchaceae	*Agalinis*	*Agalinis decemloba*	GU943534
Eudicotyledons	Orobanchaceae	*Agalinis*	*Agalinis decemloba*	GU943535
Eudicotyledons	Orobanchaceae	*Agalinis*	*Agalinis decemloba*	EU827899
Eudicotyledons	Orobanchaceae	*Agalinis*	*Agalinis decemloba*	EU827900
Eudicotyledons	Orobanchaceae	*Agalinis*	*Agalinis skinneriana*	EU827947
Eudicotyledons	Orobanchaceae	*Agalinis*	*Agalinis skinneriana*	EU827948
Eudicotyledons	Orobanchaceae	*Agalinis*	*Agalinis skinneriana*	EU827949
Eudicotyledons	Brassicaceae	*Cardamine*	*Cardamine tangutorum*	JN044169
Eudicotyledons	Brassicaceae	*Cardamine*	*Cardamine tangutorum*	JN044170
Eudicotyledons	Brassicaceae	*Cardamine*	*Cardamine tangutorum*	JN044171
Eudicotyledons	Brassicaceae	*Cardamine*	*Cardamine tangutorum*	JN044172
Eudicotyledons	Gesneriaceae	*Cyrtandra*	*Cyrtandra anthropophagorum*	GQ475119
Eudicotyledons	Gesneriaceae	*Cyrtandra*	*Cyrtandra anthropophagorum*	GQ475120
Eudicotyledons	Gesneriaceae	*Cyrtandra*	*Cyrtandra anthropophagorum*	EU920042
Eudicotyledons	Gesneriaceae	*Cyrtandra*	*Cyrtandra munroi*	GQ475134
Eudicotyledons	Gesneriaceae	*Cyrtandra*	*Cyrtandra munroi*	GQ475155
Eudicotyledons	Primulaceae	*Primula*	*Primula moupinensis*	JN046538
Eudicotyledons	Primulaceae	*Primula*	*Primula moupinensis*	JN046539
Eudicotyledons	Primulaceae	*Primula*	*Primula moupinensis*	JN046540
Eudicotyledons	Primulaceae	*Primula*	*Primula moupinensis*	JN046541
Eudicotyledons	Primulaceae	*Primula*	*Primula moupinensis*	JN046542
Eudicotyledons	Gentianaceae	*Gentianopsis*	*Gentianopsis macrantha*	HM460859
Eudicotyledons	Gentianaceae	*Gentianopsis*	*Gentianopsis macrantha*	HM460860
Eudicotyledons	Gentianaceae	*Gentianopsis*	*Gentianopsis macrantha*	HM460861
Eudicotyledons	Gentianaceae	*Gentianopsis*	*Gentianopsis thermalis*	HM460862
Eudicotyledons	Gentianaceae	*Gentianopsis*	*Gentianopsis thermalis*	HM460863
Eudicotyledons	Gentianaceae	*Gentianopsis*	*Gentianopsis thermalis*	HM460864
Eudicotyledons	Apiaceae	*Peucedanum*	*Peucedanum medicum*	JN046225
Eudicotyledons	Apiaceae	*Peucedanum*	*Peucedanum medicum*	JN046226
Eudicotyledons	Apiaceae	*Peucedanum*	*Peucedanum medicum*	JN046227
Eudicotyledons	Apiaceae	*Peucedanum*	*Peucedanum medicum*	JN046228
Eudicotyledons	Loranthaceae	*Helixanthera*	*Helixanthera parasitica*	HQ317802
Eudicotyledons	Loranthaceae	*Helixanthera*	*Helixanthera parasitica*	HQ317803
Eudicotyledons	Loranthaceae	*Helixanthera*	*Helixanthera parasitica*	HQ317804
Eudicotyledons	Loranthaceae	*Helixanthera*	*Helixanthera parasitica*	HQ317805
Eudicotyledons	Loranthaceae	*Helixanthera*	*Helixanthera parasitica*	JN687573
Eudicotyledons	Melianthaceae	*Melianthus*	*Melianthus comosus*	DQ435427
Eudicotyledons	Melianthaceae	*Melianthus*	*Melianthus comosus*	DQ435428
Eudicotyledons	Melianthaceae	*Melianthus*	*Melianthus comosus*	DQ435429
Eudicotyledons	Melianthaceae	*Melianthus*	*Melianthus comosus*	DQ435430
Eudicotyledons	Melianthaceae	*Melianthus*	*Melianthus comosus*	DQ435431
Eudicotyledons	Melianthaceae	*Melianthus*	*Melianthus pectinatus*	DQ435434
Eudicotyledons	Melianthaceae	*Melianthus*	*Melianthus pectinatus*	DQ435435
Eudicotyledons	Asteraceae	*Blumea*	*Blumea fistulosa*	EU195589
Eudicotyledons	Asteraceae	*Blumea*	*Blumea fistulosa*	EF210984
Eudicotyledons	Malvaceae	*Malva*	*Malva alcea*	GQ248337
Eudicotyledons	Malvaceae	*Malva*	*Malva alcea*	EF419609
Eudicotyledons	Malvaceae	*Malva*	*Malva alcea*	EF419610

Eudicotyledons	Malvaceae	*Malva*	*Malva alcea*	EF419611
Eudicotyledons	Malvaceae	*Malva*	*Malva alcea*	EF590713
Eudicotyledons	Malvaceae	*Malva*	*Malva alcea*	HM214493
Eudicotyledons	Malvaceae	*Malva*	*Malva alcea*	HM214494
Eudicotyledons	Malvaceae	*Malva*	*Malva alcea*	HM214495
Eudicotyledons	Malvaceae	*Malva*	*Malva alcea*	HM214496
Eudicotyledons	Malvaceae	*Malva*	*Malva alcea*	HM214497
Eudicotyledons	Malvaceae	*Malva*	*Malva alcea*	HM214498
Eudicotyledons	Malvaceae	*Malva*	*Malva alcea*	HM214499
Eudicotyledons	Malvaceae	*Malva*	*Malva alcea*	HM214500
Eudicotyledons	Malvaceae	*Malva*	*Malva alcea*	HM214501
Eudicotyledons	Malvaceae	*Malva*	*Malva alcea*	HM214502
Eudicotyledons	Malvaceae	*Malva*	*Malva alcea*	HM214503
Eudicotyledons	Malvaceae	*Malva*	*Malva alcea*	HM214504
Eudicotyledons	Malvaceae	*Malva*	*Malva alcea*	HM214505
Eudicotyledons	Malvaceae	*Malva*	*Malva hispanica*	EF419606
Eudicotyledons	Malvaceae	*Malva*	*Malva hispanica*	EF419607
Eudicotyledons	Malvaceae	*Malva*	*Malva hispanica*	EF419608
Eudicotyledons	Loranthaceae	*Helixanthera*	*Helixanthera sampsonii*	HQ317806
Eudicotyledons	Loranthaceae	*Helixanthera*	*Helixanthera sampsonii*	HQ317807
Eudicotyledons	Asteraceae	*Sinosenecio*	*Sinosenecio cyclaminifolius*	JN047133
Eudicotyledons	Asteraceae	*Sinosenecio*	*Sinosenecio cyclaminifolius*	JN047134
Eudicotyledons	Asteraceae	*Sinosenecio*	*Sinosenecio cyclaminifolius*	JN047135
Gymnosperms	Pinaceae	*Picea*	*Picea abies*	FR832550
Gymnosperms	Pinaceae	*Picea*	*Picea abies*	FR832551
Gymnosperms	Pinaceae	*Picea*	*Picea abies*	FR832552
Gymnosperms	Pinaceae	*Picea*	*Picea abies*	FJ493294
Gymnosperms	Pinaceae	*Picea*	*Picea abies*	HQ114857
Gymnosperms	Pinaceae	*Picea*	*Picea abies*	HQ114858
Gymnosperms	Pinaceae	*Pinus*	*Pinus nigra*	EU531715
Gymnosperms	Pinaceae	*Pinus*	*Pinus nigra*	FJ493295
Gymnosperms	Pinaceae	*Pinus*	*Pinus nigra*	FN689385
Gymnosperms	Pinaceae	*Pinus*	*Pinus halepensis*	EU531714
Gymnosperms	Pinaceae	*Pinus*	*Pinus halepensis*	FR832528
Gymnosperms	Pinaceae	*Pinus*	*Pinus halepensis*	FR832529
Gymnosperms	Pinaceae	*Pinus*	*Pinus halepensis*	FR832530
Gymnosperms	Pinaceae	*Pinus*	*Pinus halepensis*	FR832531
Gymnosperms	Pinaceae	*Pinus*	*Pinus halepensis*	FN689388
Gymnosperms	Podocarpaceae	*Podocarpus*	*Podocarpus matudae*	HM036081
Gymnosperms	Podocarpaceae	*Podocarpus*	*Podocarpus matudae*	HM036082
Gymnosperms	Podocarpaceae	*Podocarpus*	*Podocarpus matudae*	HM036083
Gymnosperms	Podocarpaceae	*Podocarpus*	*Podocarpus matudae*	HM036084
Gymnosperms	Podocarpaceae	*Podocarpus*	*Podocarpus matudae*	HM036085
Gymnosperms	Podocarpaceae	*Podocarpus*	*Podocarpus matudae*	HM036086
Gymnosperms	Podocarpaceae	*Podocarpus*	*Podocarpus matudae*	HM036087
Gymnosperms	Podocarpaceae	*Podocarpus*	*Podocarpus matudae*	HM036088
Gymnosperms	Podocarpaceae	*Podocarpus*	*Podocarpus matudae*	HM036089
Gymnosperms	Podocarpaceae	*Podocarpus*	*Podocarpus matudae*	HM036090
Gymnosperms	Podocarpaceae	*Podocarpus*	*Podocarpus matudae*	HM036091
Gymnosperms	Cephalotaxaceae	*Cephalotaxus*	*Cephalotaxus mannii*	GQ463521
Gymnosperms	Cephalotaxaceae	*Cephalotaxus*	*Cephalotaxus mannii*	EF660675
Gymnosperms	Cephalotaxaceae	*Cephalotaxus*	*Cephalotaxus mannii*	JN044249
Gymnosperms	Cephalotaxaceae	*Cephalotaxus*	*Cephalotaxus mannii*	JN044250

Gymnosperms	Cephalotaxaceae	*Cephalotaxus*	*Cephalotaxus mannii*	JN044251
Gymnosperms	Cephalotaxaceae	*Cephalotaxus*	*Cephalotaxus mannii*	JN044252
Gymnosperms	Cephalotaxaceae	*Cephalotaxus*	*Cephalotaxus mannii*	JN044253
Gymnosperms	Cephalotaxaceae	*Cephalotaxus*	*Cephalotaxus mannii*	JN044254
Gymnosperms	Cephalotaxaceae	*Cephalotaxus*	*Cephalotaxus mannii*	JN044255
Gymnosperms	Cephalotaxaceae	*Cephalotaxus*	*Cephalotaxus mannii*	JN044256
Gymnosperms	Cephalotaxaceae	*Cephalotaxus*	*Cephalotaxus mannii*	JN044257
Monocotyledons	Potamogetonaceae	*Potamogeton*	*Potamogeton gramineus*	DQ786534
Monocotyledons	Potamogetonaceae	*Potamogeton*	*Potamogeton gramineus*	EF174574
Monocotyledons	Potamogetonaceae	*Potamogeton*	*Potamogeton gramineus*	FN668445
Monocotyledons	Orchidaceae	*Cymbidium*	*Cymbidium ensifolium*	FJ527765
Monocotyledons	Orchidaceae	*Cymbidium*	*Cymbidium ensifolium*	FJ527766
Monocotyledons	Asparagaceae	*Polygonatum*	*Polygonatum cirrhifolium*	EU850212
Monocotyledons	Asparagaceae	*Polygonatum*	*Polygonatum cirrhifolium*	JN046400
Monocotyledons	Asparagaceae	*Polygonatum*	*Polygonatum cirrhifolium*	JN046401
Monocotyledons	Asparagaceae	*Polygonatum*	*Polygonatum cirrhifolium*	JN046402
Monocotyledons	Asparagaceae	*Polygonatum*	*Polygonatum cirrhifolium*	JN046403
Monocotyledons	Asparagaceae	*Polygonatum*	*Polygonatum cirrhifolium*	JN046404
Monocotyledons	Asparagaceae	*Polygonatum*	*Polygonatum cirrhifolium*	JN046405
Monocotyledons	Asparagaceae	*Polygonatum*	*Polygonatum cirrhifolium*	JN046406
Monocotyledons	Asparagaceae	*Polygonatum*	*Polygonatum cirrhifolium*	JN046407
Monocotyledons	Asparagaceae	*Polygonatum*	*Polygonatum cirrhifolium*	JN046408
Monocotyledons	Asparagaceae	*Polygonatum*	*Polygonatum cirrhifolium*	JN046409
Monocotyledons	Asparagaceae	*Polygonatum*	*Polygonatum cirrhifolium*	JN046410
Monocotyledons	Asparagaceae	*Polygonatum*	*Polygonatum cirrhifolium*	JN046411
Monocotyledons	Asparagaceae	*Polygonatum*	*Polygonatum cirrhifolium*	JN046412
Monocotyledons	Arecaceae	*Wallichia*	*Wallichia disticha*	JF345073
Monocotyledons	Arecaceae	*Wallichia*	*Wallichia disticha*	JF345074
Monocotyledons	Arecaceae	*Arenga*	*Arenga caudata*	JF345007
Monocotyledons	Arecaceae	*Arenga*	*Arenga caudata*	JF345008
Monocotyledons	Arecaceae	*Arenga*	*Arenga caudata*	JF345009
Monocotyledons	Arecaceae	*Arenga*	*Arenga caudata*	JF345010
Monocotyledons	Arecaceae	*Arenga*	*Arenga caudata*	JF345011
Monocotyledons	Arecaceae	*Arenga*	*Arenga caudata*	JF345012
Monocotyledons	Arecaceae	*Arenga*	*Arenga caudata*	JF345013
Monocotyledons	Arecaceae	*Arenga*	*Arenga caudata*	JF345014
Monocotyledons	Arecaceae	*Arenga*	*Arenga caudata*	JF345015
Monocotyledons	Arecaceae	*Arenga*	*Arenga caudata*	JF345016
Monocotyledons	Arecaceae	*Arenga*	*Arenga undulatifolia*	JF345039
Monocotyledons	Arecaceae	*Arenga*	*Arenga undulatifolia*	JF345040
Monocotyledons	Araceae	*Wolffia*	*Wolffia brasiliensis*	GU454557
Monocotyledons	Araceae	*Wolffia*	*Wolffia brasiliensis*	GU454558
Monocotyledons	Araceae	*Wolffia*	*Wolffia brasiliensis*	GU454559
Monocotyledons	Araceae	*Wolffia*	*Wolffia columbiana*	GU454560
Monocotyledons	Araceae	*Wolffia*	*Wolffia columbiana*	GU454561
Monocotyledons	Araceae	*Wolffia*	*Wolffia columbiana*	GU454562
Monocotyledons	Araceae	*Wolffia*	*Wolffia columbiana*	GU454563
Monocotyledons	Araceae	*Wolffia*	*Wolffia columbiana*	GU454564
Monocotyledons	Araceae	*Wolffia*	*Wolffia columbiana*	HQ596901
Monocotyledons	Araceae	*Wolffiella*	*Wolffiella oblonga*	GU454545
Monocotyledons	Araceae	*Wolffiella*	*Wolffiella oblonga*	GU454546
Monocotyledons	Araceae	*Wolffiella*	*Wolffiella oblonga*	GU454547
Monocotyledons	Araceae	*Wolffiella*	*Wolffiella oblonga*	GU454548

Monocotyledons	Araceae	*Wolffiella*	*Wolffiella oblonga*	GU454549
Monocotyledons	Bromeliaceae	*Aechmea*	*Aechmea lingulata*	EF110667
Monocotyledons	Bromeliaceae	*Aechmea*	*Aechmea lingulata*	JN204616
Monocotyledons	Bromeliaceae	*Aechmea*	*Aechmea lingulata*	JN204617
Monocotyledons	Bromeliaceae	*Aechmea*	*Aechmea lingulata*	HQ913652
Monocotyledons	Hydrocharitaceae	*Najas*	*Najas graminea*	HQ687174
Monocotyledons	Hydrocharitaceae	*Najas*	*Najas graminea*	HQ687175
Monocotyledons	Hydrocharitaceae	*Najas*	*Najas graminea*	HQ687176
Monocotyledons	Hydrocharitaceae	*Najas*	*Najas graminea*	HQ687177
Monocotyledons	Hydrocharitaceae	*Najas*	*Najas graminea*	HQ687178
Monocotyledons	Hydrocharitaceae	*Najas*	*Najas graminea*	HQ687179
Monocotyledons	Bromeliaceae	*Billbergia*	*Billbergia euphemiae*	JN204630
Monocotyledons	Bromeliaceae	*Billbergia*	*Billbergia euphemiae*	JN204631
Monocotyledons	Bromeliaceae	*Billbergia*	*Billbergia euphemiae*	JN204632
Monocotyledons	Bromeliaceae	*Aechmea*	*Aechmea bromeliifolia*	JN204601
Monocotyledons	Bromeliaceae	*Aechmea*	*Aechmea bromeliifolia*	JN204602
Monocotyledons	Bromeliaceae	*Aechmea*	*Aechmea bromeliifolia*	JN204603
Monocotyledons	Bromeliaceae	*Aechmea*	*Aechmea bromeliifolia*	HQ913649
Monocotyledons	Bromeliaceae	*Aechmea*	*Aechmea coelestis*	JN204608
Monocotyledons	Bromeliaceae	*Aechmea*	*Aechmea coelestis*	JN204609
Monocotyledons	Bromeliaceae	*Aechmea*	*Aechmea recurvata*	JN204625
Monocotyledons	Bromeliaceae	*Aechmea*	*Aechmea recurvata*	JN204626
Monocotyledons	Bromeliaceae	*Aechmea*	*Aechmea recurvata*	JN204627
Monocotyledons	Arecaceae	*Arenga*	*Arenga engleri*	JF345020
Monocotyledons	Arecaceae	*Arenga*	*Arenga engleri*	JF345021
Monocotyledons	Arecaceae	*Arenga*	*Arenga engleri*	JF345022
Monocotyledons	Arecaceae	*Arenga*	*Arenga engleri*	JF345023
Monocotyledons	Arecaceae	*Arenga*	*Arenga obtusifolia*	JF345032
Monocotyledons	Arecaceae	*Arenga*	*Arenga obtusifolia*	JF345033
Monocotyledons	Arecaceae	*Wallichia*	*Wallichia gracilis*	JF345072
Monocotyledons	Arecaceae	*Wallichia*	*Wallichia gracilis*	JF345075
Monocotyledons	Arecaceae	*Wallichia*	*Wallichia gracilis*	JF345076
Monocotyledons	Bromeliaceae	*Aechmea*	*Aechmea caudata*	JN204605
Monocotyledons	Bromeliaceae	*Aechmea*	*Aechmea caudata*	JN204606
Monocotyledons	Bromeliaceae	*Aechmea*	*Aechmea caudata*	JN204607
Monocotyledons	Iridaceae	*Iris*	*Iris lactea*	JN044989
Monocotyledons	Iridaceae	*Iris*	*Iris lactea*	JN044990
Monocotyledons	Iridaceae	*Iris*	*Iris lactea*	JN044991
Monocotyledons	Iridaceae	*Iris*	*Iris lactea*	JN044992
Monocotyledons	Iridaceae	*Iris*	*Iris goniocarpa*	JN044981
Monocotyledons	Iridaceae	*Iris*	*Iris goniocarpa*	JN044982
Monocotyledons	Araceae	*Lemna*	*Lemna minor*	GU454511
Monocotyledons	Araceae	*Lemna*	*Lemna minor*	GU454512
Monocotyledons	Araceae	*Lemna*	*Lemna minor*	GU454513
Monocotyledons	Araceae	*Lemna*	*Lemna minor*	GU454514
Monocotyledons	Araceae	*Lemna*	*Lemna minor*	GU454515
Monocotyledons	Araceae	*Lemna*	*Lemna minor*	GU454516
Monocotyledons	Araceae	*Lemna*	*Lemna minor*	FJ395556
Monocotyledons	Araceae	*Lemna*	*Lemna minor*	GQ434996
Monocotyledons	Poaceae	*Thinopyrum*	*Thinopyrum intermedium*	HQ221771
Monocotyledons	Poaceae	*Thinopyrum*	*Thinopyrum intermedium*	HQ221772
Monocotyledons	Poaceae	*Thinopyrum*	*Thinopyrum intermedium*	HQ221773
Monocotyledons	Poaceae	*Thinopyrum*	*Thinopyrum intermedium*	HQ221774

Monocotyledons	Poaceae	*Thinopyrum*	*Thinopyrum intermedium*	HQ221775
Monocotyledons	Poaceae	*Thinopyrum*	*Thinopyrum intermedium*	HQ221776
Monocotyledons	Poaceae	*Thinopyrum*	*Thinopyrum intermedium*	HQ221777
Monocotyledons	Poaceae	*Thinopyrum*	*Thinopyrum intermedium*	HQ221778
Monocotyledons	Poaceae	*Thinopyrum*	*Thinopyrum intermedium*	HQ221779
Monocotyledons	Poaceae	*Thinopyrum*	*Thinopyrum intermedium*	HQ221780
Monocotyledons	Poaceae	*Thinopyrum*	*Thinopyrum intermedium*	HQ221781
Monocotyledons	Poaceae	*Thinopyrum*	*Thinopyrum intermedium*	HQ221782
Monocotyledons	Poaceae	*Thinopyrum*	*Thinopyrum intermedium*	HQ221783
Monocotyledons	Poaceae	*Thinopyrum*	*Thinopyrum intermedium*	HQ221784
Monocotyledons	Poaceae	*Thinopyrum*	*Thinopyrum intermedium*	HQ221785
Monocotyledons	Poaceae	*Thinopyrum*	*Thinopyrum intermedium*	HQ221786
Monocotyledons	Poaceae	*Thinopyrum*	*Thinopyrum intermedium*	HQ221787
Monocotyledons	Poaceae	*Thinopyrum*	*Thinopyrum intermedium*	HQ221788
Monocotyledons	Poaceae	*Thinopyrum*	*Thinopyrum intermedium*	HQ221790
Monocotyledons	Poaceae	*Thinopyrum*	*Thinopyrum intermedium*	HQ221792
Monocotyledons	Poaceae	*Thinopyrum*	*Thinopyrum intermedium*	HQ221793
Monocotyledons	Poaceae	*Thinopyrum*	*Thinopyrum intermedium*	HQ221794
Monocotyledons	Poaceae	*Thinopyrum*	*Thinopyrum intermedium*	HQ221795
Monocotyledons	Poaceae	*Thinopyrum*	*Thinopyrum intermedium*	HQ221796
Monocotyledons	Poaceae	*Thinopyrum*	*Thinopyrum intermedium*	HQ221797
Monocotyledons	Poaceae	*Thinopyrum*	*Thinopyrum intermedium*	HQ221798
Monocotyledons	Poaceae	*Thinopyrum*	*Thinopyrum intermedium*	HQ221799
Monocotyledons	Poaceae	*Thinopyrum*	*Thinopyrum intermedium*	HQ221800
Monocotyledons	Poaceae	*Thinopyrum*	*Thinopyrum intermedium*	HQ221801
Monocotyledons	Poaceae	*Thinopyrum*	*Thinopyrum intermedium*	HQ221802
Monocotyledons	Poaceae	*Thinopyrum*	*Thinopyrum intermedium*	HQ221803
Monocotyledons	Poaceae	*Thinopyrum*	*Thinopyrum intermedium*	HQ221804
Monocotyledons	Poaceae	*Thinopyrum*	*Thinopyrum intermedium*	HQ221805
Monocotyledons	Poaceae	*Thinopyrum*	*Thinopyrum intermedium*	HQ221806
Monocotyledons	Orchidaceae	*Oncidium*	*Oncidium poikilostalix*	FJ564029
Monocotyledons	Orchidaceae	*Oncidium*	*Oncidium poikilostalix*	FJ564660
Monocotyledons	Orchidaceae	*Oncidium*	*Oncidium cirrhosum*	FJ564032
Monocotyledons	Orchidaceae	*Oncidium*	*Oncidium cirrhosum*	FJ564180
Monocotyledons	Orchidaceae	*Oncidium*	*Oncidium cirrhosum*	FJ564569
Monocotyledons	Orchidaceae	*Oncidium*	*Oncidium hallii*	FJ563973
Monocotyledons	Orchidaceae	*Oncidium*	*Oncidium hallii*	FJ564159
Monocotyledons	Orchidaceae	*Oncidium*	*Oncidium retusum*	FJ564005
Monocotyledons	Orchidaceae	*Oncidium*	*Oncidium retusum*	FJ564215
Monocotyledons	Orchidaceae	*Oncidium*	*Oncidium retusum*	FJ564578
Monocotyledons	Orchidaceae	*Oncidium*	*Oncidium retusum*	FJ564580
Monocotyledons	Orchidaceae	*Oncidium*	*Oncidium tigroides*	FJ564548
Monocotyledons	Orchidaceae	*Oncidium*	*Oncidium tigroides*	FJ564570
Monocotyledons	Araceae	*Lemna*	*Lemna trisulca*	GU454521
Monocotyledons	Araceae	*Lemna*	*Lemna trisulca*	GU454522
Monocotyledons	Araceae	*Lemna*	*Lemna trisulca*	GU454523
Monocotyledons	Asparagaceae	*Asparagus*	*Asparagus officinalis*	HM990123
Monocotyledons	Asparagaceae	*Asparagus*	*Asparagus officinalis*	HM990126
Monocotyledons	Asparagaceae	*Asparagus*	*Asparagus officinalis*	HM990127
Monocotyledons	Asparagaceae	*Asparagus*	*Asparagus officinalis*	HM990129
Monocotyledons	Asparagaceae	*Asparagus*	*Asparagus officinalis*	HM990131
Monocotyledons	Asparagaceae	*Asparagus*	*Asparagus officinalis*	HM990132
Monocotyledons	Asparagaceae	*Asparagus*	*Asparagus officinalis*	HM990133

Monocotyledons	Asparagaceae	*Asparagus*	*Asparagus officinalis*	HM990134
Monocotyledons	Asparagaceae	*Asparagus*	*Asparagus officinalis*	HM990135
Monocotyledons	Asparagaceae	*Asparagus*	*Asparagus officinalis*	HM990136
Monocotyledons	Asparagaceae	*Asparagus*	*Asparagus officinalis*	HM990137
Monocotyledons	Asparagaceae	*Asparagus*	*Asparagus officinalis*	HM990139
Monocotyledons	Asparagaceae	*Asparagus*	*Asparagus officinalis*	HM990142
Monocotyledons	Asparagaceae	*Asparagus*	*Asparagus officinalis*	HM990143
Monocotyledons	Asparagaceae	*Asparagus*	*Asparagus officinalis*	HM990144
Monocotyledons	Asparagaceae	*Asparagus*	*Asparagus officinalis*	HM990146
Monocotyledons	Asparagaceae	*Asparagus*	*Asparagus officinalis*	HM990147
Monocotyledons	Poaceae	*Festuca*	*Festuca idahoensis*	DQ369764
Monocotyledons	Poaceae	*Festuca*	*Festuca idahoensis*	DQ369766
Monocotyledons	Poaceae	*Festuca*	*Festuca idahoensis*	DQ369770
Monocotyledons	Poaceae	*Festuca*	*Festuca idahoensis*	DQ369774
Monocotyledons	Poaceae	*Festuca*	*Festuca idahoensis*	DQ369778
Monocotyledons	Melanthiaceae	*Paris*	*Paris luquanensis*	GU178892
Monocotyledons	Melanthiaceae	*Paris*	*Paris luquanensis*	JN045726
Monocotyledons	Melanthiaceae	*Paris*	*Paris luquanensis*	JN045727
Monocotyledons	Melanthiaceae	*Paris*	*Paris luquanensis*	JN045728
Monocotyledons	Melanthiaceae	*Paris*	*Paris marmorata*	DQ404256
Monocotyledons	Melanthiaceae	*Paris*	*Paris marmorata*	GU178890
Monocotyledons	Melanthiaceae	*Paris*	*Paris marmorata*	JN045735
Monocotyledons	Melanthiaceae	*Paris*	*Paris marmorata*	JN045736
Monocotyledons	Melanthiaceae	*Paris*	*Paris marmorata*	JN045737
Monocotyledons	Melanthiaceae	*Paris*	*Paris marmorata*	JN045738
Monocotyledons	Melanthiaceae	*Paris*	*Paris marmorata*	JN045739
Monocotyledons	Poaceae	*Festuca*	*Festuca roemeri*	DQ369768
Monocotyledons	Poaceae	*Festuca*	*Festuca roemeri*	DQ369772
Monocotyledons	Poaceae	*Festuca*	*Festuca roemeri*	DQ369780
Monocotyledons	Poaceae	*Festuca*	*Festuca roemeri*	DQ369782
Monocotyledons	Poaceae	*Festuca*	*Festuca roemeri*	DQ369784
Monocotyledons	Poaceae	*Festuca*	*Festuca roemeri*	DQ369786
Monocotyledons	Poaceae	*Festuca*	*Festuca roemeri*	DQ369788
Monocotyledons	Poaceae	*Festuca*	*Festuca roemeri*	DQ369790
Monocotyledons	Melanthiaceae	*Paris*	*Paris delavayi*	GU178891
Monocotyledons	Melanthiaceae	*Paris*	*Paris delavayi*	JN045706
Monocotyledons	Melanthiaceae	*Paris*	*Paris delavayi*	JN045707
Monocotyledons	Melanthiaceae	*Paris*	*Paris delavayi*	JN045708
Monocotyledons	Melanthiaceae	*Paris*	*Paris delavayi*	JN045709
Monocotyledons	Melanthiaceae	*Paris*	*Paris delavayi*	JN045710
Monocotyledons	Melanthiaceae	*Paris*	*Paris delavayi*	JN045711
Monocotyledons	Melanthiaceae	*Paris*	*Paris mairei*	DQ404247
Monocotyledons	Melanthiaceae	*Paris*	*Paris mairei*	JN045729
Monocotyledons	Melanthiaceae	*Paris*	*Paris mairei*	JN045730
Monocotyledons	Melanthiaceae	*Paris*	*Paris mairei*	JN045731
Monocotyledons	Melanthiaceae	*Paris*	*Paris mairei*	JN045732
Monocotyledons	Melanthiaceae	*Paris*	*Paris mairei*	JN045733
Monocotyledons	Melanthiaceae	*Paris*	*Paris mairei*	JN045734
Monocotyledons	Melanthiaceae	*Paris*	*Paris vietnamensis*	DQ404246
Monocotyledons	Melanthiaceae	*Paris*	*Paris vietnamensis*	GU178893
Monocotyledons	Melanthiaceae	*Paris*	*Paris vietnamensis*	JN045775
Monocotyledons	Melanthiaceae	*Paris*	*Paris vietnamensis*	JN045776
Monocotyledons	Melanthiaceae	*Paris*	*Paris vietnamensis*	JN045777

Monocotyledons	Melanthiaceae	*Paris*	*Paris vietnamensis*	JN045778
Monocotyledons	Melanthiaceae	*Paris*	*Paris vietnamensis*	JN045779
Monocotyledons	Melanthiaceae	*Paris*	*Paris vietnamensis*	JN045780
Monocotyledons	Melanthiaceae	*Veratrum*	*Veratrum oxysepalum*	JF807759
Monocotyledons	Melanthiaceae	*Veratrum*	*Veratrum oxysepalum*	JF807760
Monocotyledons	Melanthiaceae	*Veratrum*	*Veratrum oxysepalum*	JF807761
Monocotyledons	Melanthiaceae	*Veratrum*	*Veratrum oxysepalum*	JF807762
Monocotyledons	Melanthiaceae	*Veratrum*	*Veratrum oxysepalum*	JF807763
Monocotyledons	Melanthiaceae	*Veratrum*	*Veratrum oxysepalum*	JF807764
Monocotyledons	Melanthiaceae	*Veratrum*	*Veratrum oxysepalum*	JF807765
Monocotyledons	Melanthiaceae	*Veratrum*	*Veratrum oxysepalum*	JF807766
Monocotyledons	Melanthiaceae	*Veratrum*	*Veratrum oxysepalum*	JF807767
Monocotyledons	Melanthiaceae	*Veratrum*	*Veratrum oxysepalum*	JF807768
Monocotyledons	Melanthiaceae	*Veratrum*	*Veratrum oxysepalum*	JF807769
Monocotyledons	Melanthiaceae	*Veratrum*	*Veratrum oxysepalum*	JF807770
Monocotyledons	Melanthiaceae	*Veratrum*	*Veratrum oxysepalum*	JF807771
Monocotyledons	Heliconiaceae	*Heliconia*	*Heliconia bihai*	GQ248311
Monocotyledons	Heliconiaceae	*Heliconia*	*Heliconia bihai*	EF590700
Monocotyledons	Heliconiaceae	*Heliconia*	*Heliconia caribaea*	GQ248312
Monocotyledons	Heliconiaceae	*Heliconia*	*Heliconia caribaea*	EF590701
Monocotyledons	Arecaceae	*Caryota*	*Caryota maxima*	HQ415573
Monocotyledons	Arecaceae	*Caryota*	*Caryota maxima*	JF345044
Monocotyledons	Arecaceae	*Caryota*	*Caryota maxima*	JF345045
Monocotyledons	Arecaceae	*Caryota*	*Caryota maxima*	JF345050
Monocotyledons	Arecaceae	*Caryota*	*Caryota maxima*	JF345051
Monocotyledons	Arecaceae	*Caryota*	*Caryota maxima*	JF345052
Monocotyledons	Arecaceae	*Caryota*	*Caryota maxima*	JF345053
Monocotyledons	Arecaceae	*Caryota*	*Caryota maxima*	JF345065
Monocotyledons	Arecaceae	*Caryota*	*Caryota obtusa*	JF345046
Monocotyledons	Arecaceae	*Caryota*	*Caryota obtusa*	JF345047
Monocotyledons	Arecaceae	*Caryota*	*Caryota obtusa*	JF345061
Monocotyledons	Arecaceae	*Caryota*	*Caryota obtusa*	JF345062
Monocotyledons	Arecaceae	*Caryota*	*Caryota obtusa*	JF345063
Monocotyledons	Arecaceae	*Caryota*	*Caryota obtusa*	JF345064
Monocotyledons	Bromeliaceae	*Pitcairnia*	*Pitcairnia flammea*	JN204659
Monocotyledons	Bromeliaceae	*Pitcairnia*	*Pitcairnia flammea*	JN204660
Monocotyledons	Hypoxidaceae	*Pauridia*	*Pauridia longituba*	FM206348
Monocotyledons	Hypoxidaceae	*Pauridia*	*Pauridia longituba*	FM206349
Monocotyledons	Hypoxidaceae	*Pauridia*	*Pauridia longituba*	FM206350
Monocotyledons	Hypoxidaceae	*Pauridia*	*Pauridia longituba*	FM206351
Monocotyledons	Hypoxidaceae	*Pauridia*	*Pauridia longituba*	FM206352
Monocotyledons	Hypoxidaceae	*Pauridia*	*Pauridia longituba*	FM206353
Monocotyledons	Hypoxidaceae	*Pauridia*	*Pauridia longituba*	FM206354
Monocotyledons	Hypoxidaceae	*Pauridia*	*Pauridia longituba*	FM206355
Monocotyledons	Hypoxidaceae	*Pauridia*	*Pauridia longituba*	FM206356
Monocotyledons	Hypoxidaceae	*Pauridia*	*Pauridia longituba*	FM206357
Monocotyledons	Hypoxidaceae	*Pauridia*	*Pauridia longituba*	FM206358
Monocotyledons	Hypoxidaceae	*Pauridia*	*Pauridia longituba*	FM206359
Monocotyledons	Hypoxidaceae	*Pauridia*	*Pauridia longituba*	FM206360
Monocotyledons	Hypoxidaceae	*Pauridia*	*Pauridia longituba*	FM206361
Monocotyledons	Hypoxidaceae	*Pauridia*	*Pauridia longituba*	FM206362
Monocotyledons	Hypoxidaceae	*Pauridia*	*Pauridia longituba*	FM206363
Monocotyledons	Hypoxidaceae	*Pauridia*	*Pauridia longituba*	FM206364

Monocotyledons	Hypoxidaceae	*Pauridia*	*Pauridia longituba*	FM206365
Monocotyledons	Hypoxidaceae	*Pauridia*	*Pauridia longituba*	FM206366
Monocotyledons	Hypoxidaceae	*Pauridia*	*Pauridia longituba*	FM206367
Monocotyledons	Hypoxidaceae	*Pauridia*	*Pauridia longituba*	FM206368
Monocotyledons	Hypoxidaceae	*Pauridia*	*Pauridia longituba*	FM206369
Monocotyledons	Hypoxidaceae	*Pauridia*	*Pauridia longituba*	FM206370
Monocotyledons	Hypoxidaceae	*Pauridia*	*Pauridia longituba*	FM206371
Monocotyledons	Hypoxidaceae	*Pauridia*	*Pauridia longituba*	FM206372
Monocotyledons	Hypoxidaceae	*Pauridia*	*Pauridia longituba*	FM206373
Monocotyledons	Hypoxidaceae	*Pauridia*	*Pauridia longituba*	FM206374
Monocotyledons	Hypoxidaceae	*Pauridia*	*Pauridia longituba*	FM206375
Monocotyledons	Hypoxidaceae	*Pauridia*	*Pauridia longituba*	FM206376
Monocotyledons	Hypoxidaceae	*Pauridia*	*Pauridia longituba*	FM206377
Monocotyledons	Hypoxidaceae	*Pauridia*	*Pauridia longituba*	FM206378
Monocotyledons	Hypoxidaceae	*Pauridia*	*Pauridia longituba*	FM206379
Monocotyledons	Hypoxidaceae	*Pauridia*	*Pauridia longituba*	FM206380
Monocotyledons	Hyacinthaceae	*Hyacinthoides*	*Hyacinthoides hispanica*	FJ423321
Monocotyledons	Hyacinthaceae	*Hyacinthoides*	*Hyacinthoides hispanica*	FJ423322
Monocotyledons	Hyacinthaceae	*Hyacinthoides*	*Hyacinthoides hispanica*	FJ423323
Monocotyledons	Hyacinthaceae	*Hyacinthoides*	*Hyacinthoides hispanica*	FJ423324
Monocotyledons	Hyacinthaceae	*Hyacinthoides*	*Hyacinthoides hispanica*	FJ423325
Monocotyledons	Hyacinthaceae	*Hyacinthoides*	*Hyacinthoides hispanica*	FJ423326
Monocotyledons	Hyacinthaceae	*Hyacinthoides*	*Hyacinthoides hispanica*	FJ423327
Monocotyledons	Hyacinthaceae	*Hyacinthoides*	*Hyacinthoides hispanica*	FJ423328
Monocotyledons	Hyacinthaceae	*Hyacinthoides*	*Hyacinthoides hispanica*	FJ423329
Monocotyledons	Hyacinthaceae	*Hyacinthoides*	*Hyacinthoides hispanica*	FJ423330
Monocotyledons	Hyacinthaceae	*Hyacinthoides*	*Hyacinthoides hispanica*	FJ423331
Monocotyledons	Hyacinthaceae	*Hyacinthoides*	*Hyacinthoides hispanica*	FJ423332
Monocotyledons	Hyacinthaceae	*Hyacinthoides*	*Hyacinthoides hispanica*	FJ423333
Monocotyledons	Hyacinthaceae	*Hyacinthoides*	*Hyacinthoides hispanica*	FJ423334
Monocotyledons	Hyacinthaceae	*Hyacinthoides*	*Hyacinthoides hispanica*	FJ423335
Monocotyledons	Hyacinthaceae	*Hyacinthoides*	*Hyacinthoides hispanica*	FJ423336
Monocotyledons	Hyacinthaceae	*Hyacinthoides*	*Hyacinthoides hispanica*	FJ423337
Monocotyledons	Hyacinthaceae	*Hyacinthoides*	*Hyacinthoides hispanica*	FJ423338
Monocotyledons	Hyacinthaceae	*Hyacinthoides*	*Hyacinthoides hispanica*	FJ423340
Monocotyledons	Hyacinthaceae	*Hyacinthoides*	*Hyacinthoides hispanica*	FJ423341
Monocotyledons	Hyacinthaceae	*Hyacinthoides*	*Hyacinthoides hispanica*	FJ423348
Monocotyledons	Hyacinthaceae	*Hyacinthoides*	*Hyacinthoides hispanica*	FJ423349
Monocotyledons	Hyacinthaceae	*Hyacinthoides*	*Hyacinthoides hispanica*	FJ423350
Monocotyledons	Hyacinthaceae	*Hyacinthoides*	*Hyacinthoides hispanica*	FJ423351
Monocotyledons	Hyacinthaceae	*Hyacinthoides*	*Hyacinthoides hispanica*	FJ423352
Monocotyledons	Hyacinthaceae	*Hyacinthoides*	*Hyacinthoides non-scripta*	FJ423316
Monocotyledons	Hyacinthaceae	*Hyacinthoides*	*Hyacinthoides non-scripta*	FJ423317
Monocotyledons	Hyacinthaceae	*Hyacinthoides*	*Hyacinthoides non-scripta*	FJ423318
Monocotyledons	Hyacinthaceae	*Hyacinthoides*	*Hyacinthoides non-scripta*	FJ423319
Monocotyledons	Hyacinthaceae	*Hyacinthoides*	*Hyacinthoides non-scripta*	FJ423320
Monocotyledons	Hyacinthaceae	*Hyacinthoides*	*Hyacinthoides reverchonii*	FJ423304
Monocotyledons	Hyacinthaceae	*Hyacinthoides*	*Hyacinthoides reverchonii*	FJ423305
Monocotyledons	Hyacinthaceae	*Hyacinthoides*	*Hyacinthoides reverchonii*	FJ423306
Monocotyledons	Hyacinthaceae	*Hyacinthoides*	*Hyacinthoides reverchonii*	FJ423307
Monocotyledons	Hyacinthaceae	*Hyacinthoides*	*Hyacinthoides reverchonii*	FJ423308
Monocotyledons	Stemonaceae	*Stemona*	*Stemona collinsae*	AB373194
Monocotyledons	Stemonaceae	*Stemona*	*Stemona collinsae*	AB373195

Mosses	Polytrichaceae	*Polytrichum*	*Polytrichum juniperinum*	GQ248374
Mosses	Polytrichaceae	*Polytrichum*	*Polytrichum juniperinum*	FJ572555
Mosses	Polytrichaceae	*Polytrichum*	*Polytrichum juniperinum*	FJ572554
Mosses	Polytrichaceae	*Polytrichum*	*Polytrichum juniperinum*	EU750675
Mosses	Polytrichaceae	*Polytrichum*	*Polytrichum juniperinum*	EU750673
Mosses	Polytrichaceae	*Polytrichum*	*Polytrichum juniperinum*	EU750674
Mosses	Polytrichaceae	*Polytrichum*	*Polytrichum juniperinum*	EF590731
