# Supplementary material for: Utility of the trnH–psbA Intergenic Spacer Region and Its Combinations as Plant DNA Barcodes: A Meta-Analysis
Source: PLoS One. 2012 Nov 14;7(11):e48833. doi: 10.1371/journal.pone.0048833 (PMC3498263; doi:10.1371/journal.pone.0048833)
Supplement: Table S3 — List of trnH – psbA samples used in this study. The names of the corresponding group, family, genus, and species, as well as the GenBank accession number for each sample are shown. (PDF) [file pone.0048833.s003.pdf]

Table S3. List of *trnH–psbA* samples used in this study. The names of the corresponding group, family, genus, and species, as well as the GenBank accession number for each sample are shown.

Taxonomy group	Family	Genus	Species	GenBank accesstion no.
Eudicotyledons	Betulaceae	*Carpinus*	*Carpinus betulus*	FJ395523
Eudicotyledons	Betulaceae	*Carpinus*	*Carpinus betulus*	AY211429
Eudicotyledons	Betulaceae	*Carpinus*	*Carpinus betulus*	FJ011833
Eudicotyledons	Betulaceae	*Carpinus*	*Carpinus caroliniana*	AY211430
Eudicotyledons	Betulaceae	*Carpinus*	*Carpinus caroliniana*	DQ006158
Eudicotyledons	Asteraceae	*Achillea*	*Achillea millefolium*	FJ395492
Eudicotyledons	Asteraceae	*Achillea*	*Achillea millefolium*	HQ451003
Eudicotyledons	Asteraceae	*Achillea*	*Achillea millefolium*	HQ451004
Eudicotyledons	Asteraceae	*Achillea*	*Achillea millefolium*	HQ451005
Eudicotyledons	Asteraceae	*Achillea*	*Achillea millefolium*	HQ451006
Eudicotyledons	Asteraceae	*Achillea*	*Achillea millefolium*	HQ451007
Eudicotyledons	Asteraceae	*Achillea*	*Achillea millefolium*	HQ451053
Eudicotyledons	Asteraceae	*Achillea*	*Achillea millefolium*	HQ451054
Eudicotyledons	Asteraceae	*Achillea*	*Achillea millefolium*	HQ596580
Eudicotyledons	Primulaceae	*Ardisia*	*Ardisia crenata*	HQ427113
Eudicotyledons	Primulaceae	*Ardisia*	*Ardisia crenata*	GU135312
Eudicotyledons	Primulaceae	*Ardisia*	*Ardisia crenata*	GU135441
Eudicotyledons	Betulaceae	*Corylus*	*Corylus avellana*	FJ011848
Eudicotyledons	Betulaceae	*Corylus*	*Corylus avellana*	FR865092
Eudicotyledons	Betulaceae	*Corylus*	*Corylus cornuta*	HQ596654
Eudicotyledons	Betulaceae	*Corylus*	*Corylus cornuta*	AY211450
Eudicotyledons	Betulaceae	*Corylus*	*Corylus cornuta*	FJ011850
Eudicotyledons	Oleaceae	*Ligustrum*	*Ligustrum vulgare*	FJ395550
Eudicotyledons	Oleaceae	*Ligustrum*	*Ligustrum vulgare*	FJ493288
Eudicotyledons	Oleaceae	*Ligustrum*	*Ligustrum vulgare*	FN675794
Eudicotyledons	Betulaceae	*Ostrya*	*Ostrya virginiana*	HQ596777
Eudicotyledons	Betulaceae	*Ostrya*	*Ostrya virginiana*	HQ596778
Eudicotyledons	Betulaceae	*Ostrya*	*Ostrya virginiana*	AY211456
Eudicotyledons	Betulaceae	*Ostrya*	*Ostrya virginiana*	FJ011865
Eudicotyledons	Asteraceae	*Tragopogon*	*Tragopogon porrifolius*	GQ435123
Eudicotyledons	Asteraceae	*Tragopogon*	*Tragopogon porrifolius*	EF374277
Eudicotyledons	Asteraceae	*Tragopogon*	*Tragopogon porrifolius*	EF374278
Eudicotyledons	Asteraceae	*Tragopogon*	*Tragopogon porrifolius*	EF374279
Eudicotyledons	Asteraceae	*Tragopogon*	*Tragopogon porrifolius*	EF374280
Eudicotyledons	Asteraceae	*Tragopogon*	*Tragopogon porrifolius*	HQ162037
Eudicotyledons	Juglandaceae	*Juglans*	*Juglans nigra*	HQ596736
Eudicotyledons	Juglandaceae	*Juglans*	*Juglans nigra*	AY293339
Eudicotyledons	Campanulaceae	*Cyananthus*	*Cyananthus lobatus*	JN044355
Eudicotyledons	Campanulaceae	*Cyananthus*	*Cyananthus lobatus*	JN044356
Eudicotyledons	Paeoniaceae	*Paeonia*	*Paeonia anomala*	DQ313731
Eudicotyledons	Paeoniaceae	*Paeonia*	*Paeonia anomala*	DQ313732
Eudicotyledons	Paeoniaceae	*Paeonia*	*Paeonia anomala*	DQ313733
Eudicotyledons	Paeoniaceae	*Paeonia*	*Paeonia anomala*	DQ313734
Eudicotyledons	Paeoniaceae	*Paeonia*	*Paeonia anomala*	DQ313735
Eudicotyledons	Paeoniaceae	*Paeonia*	*Paeonia anomala*	GQ435203
Eudicotyledons	Paeoniaceae	*Paeonia*	*Paeonia anomala*	GQ435204
Eudicotyledons	Paeoniaceae	*Paeonia*	*Paeonia delavayi*	GU367368
Eudicotyledons	Paeoniaceae	*Paeonia*	*Paeonia delavayi*	JF496834
Eudicotyledons	Asteraceae	*Chrysanthemum*	*Chrysanthemum x morifolium*	EF091620

Eudicotyledons	Asteraceae	*Chrysanthemum*	*Chrysanthemum x morifolium*	EF091621
Eudicotyledons	Asteraceae	*Chrysanthemum*	*Chrysanthemum x morifolium*	EF091623
Eudicotyledons	Asteraceae	*Chrysanthemum*	*Chrysanthemum x morifolium*	EF091625
Eudicotyledons	Asteraceae	*Chrysanthemum*	*Chrysanthemum x morifolium*	GU575275
Eudicotyledons	Asteraceae	*Chrysanthemum*	*Chrysanthemum x morifolium*	GQ435094
Eudicotyledons	Asteraceae	*Tragopogon*	*Tragopogon pratensis*	FJ395553
Eudicotyledons	Asteraceae	*Tragopogon*	*Tragopogon pratensis*	EF374281
Eudicotyledons	Asteraceae	*Tragopogon*	*Tragopogon dubius*	HQ596868
Eudicotyledons	Asteraceae	*Tragopogon*	*Tragopogon dubius*	EF374262
Eudicotyledons	Asteraceae	*Tragopogon*	*Tragopogon dubius*	HQ162036
Eudicotyledons	Oleaceae	*Ligustrum*	*Ligustrum japonicum*	FN675796
Eudicotyledons	Oleaceae	*Ligustrum*	*Ligustrum japonicum*	JF830322
Eudicotyledons	Oleaceae	*Ligustrum*	*Ligustrum japonicum*	JF830323
Eudicotyledons	Oleaceae	*Ligustrum*	*Ligustrum japonicum*	JF830324
Eudicotyledons	Oleaceae	*Ligustrum*	*Ligustrum japonicum*	JF830325
Eudicotyledons	Oleaceae	*Ligustrum*	*Ligustrum japonicum*	JF830326
Eudicotyledons	Juglandaceae	*Juglans*	*Juglans regia*	HE659564
Eudicotyledons	Juglandaceae	*Juglans*	*Juglans regia*	GQ435024
Eudicotyledons	Juglandaceae	*Juglans*	*Juglans regia*	AY293344
Eudicotyledons	Styracaceae	*Styrax*	*Styrax japonicus*	AB115400
Eudicotyledons	Styracaceae	*Styrax*	*Styrax japonicus*	AB237434
Eudicotyledons	Styracaceae	*Styrax*	*Styrax japonicus*	AB237435
Eudicotyledons	Paeoniaceae	*Paeonia*	*Paeonia rockii*	GU367372
Eudicotyledons	Paeoniaceae	*Paeonia*	*Paeonia rockii*	JF496836
Eudicotyledons	Paeoniaceae	*Paeonia*	*Paeonia rockii*	JF496837
Eudicotyledons	Paeoniaceae	*Paeonia*	*Paeonia rockii*	JF496838
Eudicotyledons	Paeoniaceae	*Paeonia*	*Paeonia rockii*	JF496839
Eudicotyledons	Paeoniaceae	*Paeonia*	*Paeonia rockii*	JF496840
Eudicotyledons	Paeoniaceae	*Paeonia*	*Paeonia rockii*	JF496841
Eudicotyledons	Paeoniaceae	*Paeonia*	*Paeonia rockii*	JF496842
Eudicotyledons	Paeoniaceae	*Paeonia*	*Paeonia rockii*	JF496843
Eudicotyledons	Paeoniaceae	*Paeonia*	*Paeonia rockii*	JF496844
Eudicotyledons	Paeoniaceae	*Paeonia*	*Paeonia rockii*	JF496845
Eudicotyledons	Paeoniaceae	*Paeonia*	*Paeonia rockii*	JF496846
Eudicotyledons	Paeoniaceae	*Paeonia*	*Paeonia rockii*	JF496847
Eudicotyledons	Paeoniaceae	*Paeonia*	*Paeonia rockii*	JF496848
Eudicotyledons	Paeoniaceae	*Paeonia*	*Paeonia rockii*	JF496849
Eudicotyledons	Paeoniaceae	*Paeonia*	*Paeonia rockii*	JF496850
Eudicotyledons	Paeoniaceae	*Paeonia*	*Paeonia rockii*	JF496851
Eudicotyledons	Paeoniaceae	*Paeonia*	*Paeonia rockii*	JF496852
Eudicotyledons	Paeoniaceae	*Paeonia*	*Paeonia rockii*	JF496853
Eudicotyledons	Paeoniaceae	*Paeonia*	*Paeonia rockii*	JF496854
Eudicotyledons	Paeoniaceae	*Paeonia*	*Paeonia rockii*	JF496855
Eudicotyledons	Paeoniaceae	*Paeonia*	*Paeonia jishanensis*	GU367369
Eudicotyledons	Paeoniaceae	*Paeonia*	*Paeonia jishanensis*	GU367370
Eudicotyledons	Betulaceae	*Carpinus*	*Carpinus laxiflora*	AY211434
Eudicotyledons	Betulaceae	*Carpinus*	*Carpinus laxiflora*	AY211435
Eudicotyledons	Betulaceae	*Carpinus*	*Carpinus laxiflora*	AB525361
Eudicotyledons	Betulaceae	*Carpinus*	*Carpinus laxiflora*	AB525362
Eudicotyledons	Betulaceae	*Carpinus*	*Carpinus laxiflora*	AB525363
Eudicotyledons	Betulaceae	*Carpinus*	*Carpinus laxiflora*	AB525364
Eudicotyledons	Betulaceae	*Carpinus*	*Carpinus laxiflora*	AB525365
Eudicotyledons	Betulaceae	*Carpinus*	*Carpinus laxiflora*	AB525366

Eudicotyledons	Betulaceae	*Carpinus*	*Carpinus laxiflora*	AB525367
Eudicotyledons	Betulaceae	*Carpinus*	*Carpinus laxiflora*	AB525368
Eudicotyledons	Betulaceae	*Carpinus*	*Carpinus laxiflora*	FJ011840
Eudicotyledons	Betulaceae	*Corylus*	*Corylus americana*	AY211448
Eudicotyledons	Betulaceae	*Corylus*	*Corylus americana*	FJ011847
Eudicotyledons	Betulaceae	*Carpinus*	*Carpinus turczaninovii*	AY211446
Eudicotyledons	Betulaceae	*Carpinus*	*Carpinus turczaninovii*	FJ011846
Eudicotyledons	Betulaceae	*Corylus*	*Corylus chinensis*	AY211449
Eudicotyledons	Betulaceae	*Corylus*	*Corylus chinensis*	FJ011849
Eudicotyledons	Betulaceae	*Ostrya*	*Ostrya rehderiana*	AY211454
Eudicotyledons	Betulaceae	*Ostrya*	*Ostrya rehderiana*	AY211455
Eudicotyledons	Betulaceae	*Ostrya*	*Ostrya rehderiana*	FJ011863
Eudicotyledons	Betulaceae	*Ostrya*	*Ostrya rehderiana*	FJ011864
Eudicotyledons	Betulaceae	*Carpinus*	*Carpinus cordata*	AY211431
Eudicotyledons	Betulaceae	*Carpinus*	*Carpinus cordata*	AY211432
Eudicotyledons	Betulaceae	*Carpinus*	*Carpinus cordata*	FJ011834
Eudicotyledons	Betulaceae	*Carpinus*	*Carpinus cordata*	FJ011835
Eudicotyledons	Juglandaceae	*Juglans*	*Juglans cathayensis*	AY293334
Eudicotyledons	Juglandaceae	*Juglans*	*Juglans cathayensis*	JF708229
Eudicotyledons	Juglandaceae	*Juglans*	*Juglans cinerea*	HQ596735
Eudicotyledons	Juglandaceae	*Juglans*	*Juglans cinerea*	AY293347
Eudicotyledons	Juglandaceae	*Juglans*	*Juglans olanchana*	AY293333
Eudicotyledons	Juglandaceae	*Juglans*	*Juglans olanchana*	AY293345
Eudicotyledons	Asteraceae	*Eupatorium*	*Eupatorium perfoliatum*	GU014469
Eudicotyledons	Asteraceae	*Eupatorium*	*Eupatorium perfoliatum*	EU750465
Eudicotyledons	Asteraceae	*Eupatorium*	*Eupatorium perfoliatum*	EU750466
Eudicotyledons	Asteraceae	*Eupatorium*	*Eupatorium serotinum*	GU818373
Eudicotyledons	Asteraceae	*Eupatorium*	*Eupatorium serotinum*	GU014470
Eudicotyledons	Primulaceae	*Omphalogramma*	*Omphalogramma delavayi*	JN045599
Eudicotyledons	Primulaceae	*Omphalogramma*	*Omphalogramma delavayi*	JN045600
Eudicotyledons	Primulaceae	*Omphalogramma*	*Omphalogramma delavayi*	JN045601
Eudicotyledons	Primulaceae	*Omphalogramma*	*Omphalogramma delavayi*	JN045602
Eudicotyledons	Primulaceae	*Omphalogramma*	*Omphalogramma delavayi*	JN045603
Eudicotyledons	Salicaceae	*Casearia*	*Casearia sylvestris*	GQ982170
Eudicotyledons	Salicaceae	*Casearia*	*Casearia sylvestris*	HM446898
Eudicotyledons	Fabaceae	*Anthyllis*	*Anthyllis circinnata*	HM468238
Eudicotyledons	Fabaceae	*Anthyllis*	*Anthyllis circinnata*	HQ646215
Eudicotyledons	Melastomataceae	*Melastoma*	*Melastoma candidum*	GQ435425
Eudicotyledons	Melastomataceae	*Melastoma*	*Melastoma candidum*	JN406998
Eudicotyledons	Melastomataceae	*Melastoma*	*Melastoma candidum*	JN406999
Eudicotyledons	Melastomataceae	*Melastoma*	*Melastoma candidum*	JN407000
Eudicotyledons	Melastomataceae	*Melastoma*	*Melastoma candidum*	JN407001
Eudicotyledons	Melastomataceae	*Melastoma*	*Melastoma candidum*	JF708230
Eudicotyledons	Myrtaceae	*Psidium*	*Psidium guajava*	GQ434986
Eudicotyledons	Myrtaceae	*Psidium*	*Psidium guajava*	GU135421
Eudicotyledons	Asteraceae	*Tolpis*	*Tolpis coronopifolia*	GU046621
Eudicotyledons	Asteraceae	*Tolpis*	*Tolpis coronopifolia*	GU046622
Eudicotyledons	Vitaceae	*Ampelopsis*	*Ampelopsis megalophylla*	JQ182521
Eudicotyledons	Vitaceae	*Ampelopsis*	*Ampelopsis megalophylla*	HQ656484
Eudicotyledons	Oleaceae	*Ligustrum*	*Ligustrum ovalifolium*	JF830304
Eudicotyledons	Oleaceae	*Ligustrum*	*Ligustrum ovalifolium*	JF830305
Eudicotyledons	Oleaceae	*Ligustrum*	*Ligustrum ovalifolium*	JF830306
Eudicotyledons	Oleaceae	*Ligustrum*	*Ligustrum ovalifolium*	JF830307

Eudicotyledons	Oleaceae	*Ligustrum*	*Ligustrum ovalifolium*	JF830308
Eudicotyledons	Oleaceae	*Ligustrum*	*Ligustrum ovalifolium*	JF830309
Eudicotyledons	Oleaceae	*Ligustrum*	*Ligustrum ovalifolium*	JF830310
Eudicotyledons	Oleaceae	*Ligustrum*	*Ligustrum ovalifolium*	JF830311
Eudicotyledons	Oleaceae	*Ligustrum*	*Ligustrum ovalifolium*	JF830312
Eudicotyledons	Oleaceae	*Ligustrum*	*Ligustrum sempervirens*	JF830258
Eudicotyledons	Oleaceae	*Ligustrum*	*Ligustrum sempervirens*	JF830259
Eudicotyledons	Oleaceae	*Ligustrum*	*Ligustrum sempervirens*	JF830260
Eudicotyledons	Oleaceae	*Picconia*	*Picconia excelsa*	EU854417
Eudicotyledons	Oleaceae	*Picconia*	*Picconia excelsa*	EU854418
Eudicotyledons	Melastomataceae	*Melastoma*	*Melastoma dodecandrum*	GQ435424
Eudicotyledons	Melastomataceae	*Melastoma*	*Melastoma dodecandrum*	JN040997
Eudicotyledons	Polygonaceae	*Oxyria*	*Oxyria sinensis*	JN045696
Eudicotyledons	Polygonaceae	*Oxyria*	*Oxyria sinensis*	JN045697
Eudicotyledons	Polygonaceae	*Oxyria*	*Oxyria sinensis*	JN045698
Eudicotyledons	Betulaceae	*Carpinus*	*Carpinus londoniana*	AY211436
Eudicotyledons	Betulaceae	*Carpinus*	*Carpinus londoniana*	AY211437
Eudicotyledons	Apocynaceae	*Caralluma*	*Caralluma indica*	HM475430
Eudicotyledons	Apocynaceae	*Caralluma*	*Caralluma indica*	HM475461
Eudicotyledons	Rosaceae	*Pyracantha*	*Pyracantha fortuneana*	JQ390761
Eudicotyledons	Rosaceae	*Pyracantha*	*Pyracantha fortuneana*	JN046710
Eudicotyledons	Rosaceae	*Pyracantha*	*Pyracantha fortuneana*	JN046711
Eudicotyledons	Rosaceae	*Pyracantha*	*Pyracantha fortuneana*	JN046712
Eudicotyledons	Rosaceae	*Pyracantha*	*Pyracantha fortuneana*	JN046713
Eudicotyledons	Rosaceae	*Pyracantha*	*Pyracantha fortuneana*	JN046714
Eudicotyledons	Rosaceae	*Pyracantha*	*Pyracantha fortuneana*	JN046715
Eudicotyledons	Rosaceae	*Pyracantha*	*Pyracantha fortuneana*	JN046716
Eudicotyledons	Rosaceae	*Pyracantha*	*Pyracantha fortuneana*	JN046717
Eudicotyledons	Rosaceae	*Pyracantha*	*Pyracantha fortuneana*	JN046718
Eudicotyledons	Malvaceae	*Althaea*	*Althaea officinalis*	EF679752
Eudicotyledons	Malvaceae	*Althaea*	*Althaea officinalis*	EF419652
Eudicotyledons	Malvaceae	*Althaea*	*Althaea officinalis*	EF419653
Eudicotyledons	Malvaceae	*Althaea*	*Althaea officinalis*	EF419654
Eudicotyledons	Malvaceae	*Althaea*	*Althaea officinalis*	EF419655
Eudicotyledons	Malvaceae	*Althaea*	*Althaea officinalis*	EF419656
Eudicotyledons	Asteraceae	*Chrysanthemum*	*Chrysanthemum indicum*	EF091608
Eudicotyledons	Asteraceae	*Chrysanthemum*	*Chrysanthemum indicum*	GQ435133
Eudicotyledons	Asteraceae	*Chrysanthemum*	*Chrysanthemum indicum*	AB234760
Eudicotyledons	Asteraceae	*Chrysanthemum*	*Chrysanthemum indicum*	AB234761
Eudicotyledons	Asteraceae	*Chrysanthemum*	*Chrysanthemum indicum*	AB234762
Eudicotyledons	Asteraceae	*Chrysanthemum*	*Chrysanthemum indicum*	AB234763
Eudicotyledons	Asteraceae	*Chrysanthemum*	*Chrysanthemum indicum*	AB234764
Eudicotyledons	Asteraceae	*Chrysanthemum*	*Chrysanthemum indicum*	AB234765
Eudicotyledons	Asteraceae	*Chrysanthemum*	*Chrysanthemum indicum*	AB234770
Eudicotyledons	Asteraceae	*Chrysanthemum*	*Chrysanthemum indicum*	AB234776
Eudicotyledons	Asteraceae	*Chrysanthemum*	*Chrysanthemum indicum*	AB234777
Eudicotyledons	Asteraceae	*Chrysanthemum*	*Chrysanthemum indicum*	AB234779
Eudicotyledons	Asteraceae	*Chrysanthemum*	*Chrysanthemum lavandulifolium*	EF091614
Eudicotyledons	Asteraceae	*Chrysanthemum*	*Chrysanthemum lavandulifolium*	GQ435132
Eudicotyledons	Asteraceae	*Chrysanthemum*	*Chrysanthemum zawadskii*	EF091627
Eudicotyledons	Asteraceae	*Chrysanthemum*	*Chrysanthemum zawadskii*	AB234743
Eudicotyledons	Asteraceae	*Chrysanthemum*	*Chrysanthemum zawadskii*	AB234744
Eudicotyledons	Asteraceae	*Chrysanthemum*	*Chrysanthemum zawadskii*	AB234746

Eudicotyledons	Asteraceae	*Chrysanthemum*	*Chrysanthemum zawadskii*	AB234747
Eudicotyledons	Asteraceae	*Chrysanthemum*	*Chrysanthemum zawadskii*	AB234766
Eudicotyledons	Asteraceae	*Chrysanthemum*	*Chrysanthemum zawadskii*	AB234767
Eudicotyledons	Asteraceae	*Chrysanthemum*	*Chrysanthemum zawadskii*	AB234768
Eudicotyledons	Vitaceae	*Cayratia*	*Cayratia japonica*	JQ182535
Eudicotyledons	Vitaceae	*Cayratia*	*Cayratia japonica*	HM585664
Eudicotyledons	Vitaceae	*Cayratia*	*Cayratia japonica*	JF437078
Eudicotyledons	Vitaceae	*Cayratia*	*Cayratia japonica*	JF437082
Eudicotyledons	Vitaceae	*Cayratia*	*Cayratia japonica*	JF437083
Eudicotyledons	Vitaceae	*Cayratia*	*Cayratia japonica*	JF437084
Eudicotyledons	Vitaceae	*Cayratia*	*Cayratia trifolia*	JQ182533
Eudicotyledons	Vitaceae	*Cayratia*	*Cayratia trifolia*	HM585666
Eudicotyledons	Vitaceae	*Cayratia*	*Cayratia trifolia*	JF437080
Eudicotyledons	Vitaceae	*Cayratia*	*Cayratia trifolia*	JF437081
Eudicotyledons	Vitaceae	*Cissus*	*Cissus repens*	GU372818
Eudicotyledons	Vitaceae	*Cissus*	*Cissus repens*	GU372819
Eudicotyledons	Vitaceae	*Cissus*	*Cissus repens*	HM585672
Eudicotyledons	Vitaceae	*Cissus*	*Cissus repens*	HQ656490
Eudicotyledons	Styracaceae	*Styrax*	*Styrax suberifolius*	HQ415402
Eudicotyledons	Styracaceae	*Styrax*	*Styrax suberifolius*	HQ426971
Eudicotyledons	Cactaceae	*Pfeiffera*	*Pfeiffera ianthothele*	FR716785
Eudicotyledons	Cactaceae	*Pfeiffera*	*Pfeiffera ianthothele*	FR716786
Eudicotyledons	Cactaceae	*Pfeiffera*	*Pfeiffera miyagawae*	FN995429
Eudicotyledons	Cactaceae	*Pfeiffera*	*Pfeiffera miyagawae*	FN995432
Eudicotyledons	Cactaceae	*Pfeiffera*	*Pfeiffera monacantha*	FN995430
Eudicotyledons	Cactaceae	*Pfeiffera*	*Pfeiffera monacantha*	FN995431
Eudicotyledons	Cactaceae	*Lepismium*	*Lepismium cruciforme*	FN669012
Eudicotyledons	Cactaceae	*Lepismium*	*Lepismium cruciforme*	FN669013
Eudicotyledons	Cactaceae	*Lepismium*	*Lepismium cruciforme*	FN669014
Eudicotyledons	Ericaceae	*Gaultheria*	*Gaultheria borneensis*	JN044550
Eudicotyledons	Ericaceae	*Gaultheria*	*Gaultheria borneensis*	JN044551
Eudicotyledons	Ericaceae	*Gaultheria*	*Gaultheria leucocarpa*	JN044630
Eudicotyledons	Ericaceae	*Gaultheria*	*Gaultheria leucocarpa*	JN044631
Eudicotyledons	Ericaceae	*Gaultheria*	*Gaultheria leucocarpa*	JN044632
Eudicotyledons	Ericaceae	*Gaultheria*	*Gaultheria leucocarpa*	JN044633
Eudicotyledons	Ericaceae	*Gaultheria*	*Gaultheria leucocarpa*	JN044634
Eudicotyledons	Ericaceae	*Gaultheria*	*Gaultheria leucocarpa*	JN044635
Eudicotyledons	Ericaceae	*Gaultheria*	*Gaultheria leucocarpa*	JN044636
Eudicotyledons	Ericaceae	*Gaultheria*	*Gaultheria leucocarpa*	JN044637
Eudicotyledons	Ericaceae	*Gaultheria*	*Gaultheria leucocarpa*	JN044638
Eudicotyledons	Ericaceae	*Gaultheria*	*Gaultheria leucocarpa*	JN044639
Eudicotyledons	Ericaceae	*Gaultheria*	*Gaultheria leucocarpa*	JN044640
Eudicotyledons	Ericaceae	*Gaultheria*	*Gaultheria leucocarpa*	JN044641
Eudicotyledons	Ericaceae	*Gaultheria*	*Gaultheria leucocarpa*	JN044642
Eudicotyledons	Ericaceae	*Gaultheria*	*Gaultheria leucocarpa*	JN044643
Eudicotyledons	Ericaceae	*Gaultheria*	*Gaultheria leucocarpa*	JN044644
Eudicotyledons	Ericaceae	*Gaultheria*	*Gaultheria leucocarpa*	JN044645
Eudicotyledons	Ericaceae	*Gaultheria*	*Gaultheria leucocarpa*	JN044646
Eudicotyledons	Ericaceae	*Gaultheria*	*Gaultheria leucocarpa*	JN044647
Eudicotyledons	Ericaceae	*Gaultheria*	*Gaultheria leucocarpa*	JN044648
Eudicotyledons	Ericaceae	*Gaultheria*	*Gaultheria leucocarpa*	JN044649
Eudicotyledons	Ericaceae	*Gaultheria*	*Gaultheria nummularioides*	JN044657
Eudicotyledons	Ericaceae	*Gaultheria*	*Gaultheria nummularioides*	JN044658

Eudicotyledons	Ericaceae	*Gaultheria*	*Gaultheria nummularioides*	JN044659
Eudicotyledons	Ericaceae	*Gaultheria*	*Gaultheria nummularioides*	JN044660
Eudicotyledons	Ericaceae	*Gaultheria*	*Gaultheria nummularioides*	JN044661
Eudicotyledons	Ericaceae	*Gaultheria*	*Gaultheria nummularioides*	JN044662
Eudicotyledons	Ericaceae	*Gaultheria*	*Gaultheria nummularioides*	JN044663
Eudicotyledons	Ericaceae	*Gaultheria*	*Gaultheria nummularioides*	JN044664
Eudicotyledons	Ericaceae	*Gaultheria*	*Gaultheria nummularioides*	JN044665
Eudicotyledons	Fabaceae	*Eperua*	*Eperua falcata*	GQ428689
Eudicotyledons	Fabaceae	*Eperua*	*Eperua falcata*	JN661742
Eudicotyledons	Fabaceae	*Eperua*	*Eperua falcata*	JN661752
Eudicotyledons	Fabaceae	*Eperua*	*Eperua falcata*	FJ038923
Eudicotyledons	Fabaceae	*Eperua*	*Eperua falcata*	FJ038924
Eudicotyledons	Fabaceae	*Eperua*	*Eperua grandiflora*	GQ428690
Eudicotyledons	Fabaceae	*Eperua*	*Eperua grandiflora*	FJ038925
Eudicotyledons	Fabaceae	*Eperua*	*Eperua grandiflora*	FJ038926
Eudicotyledons	Fagaceae	*Castanopsis*	*Castanopsis argyrophylla*	JN044187
Eudicotyledons	Fagaceae	*Castanopsis*	*Castanopsis argyrophylla*	JN044188
Eudicotyledons	Fagaceae	*Castanopsis*	*Castanopsis carlesii*	HQ427016
Eudicotyledons	Fagaceae	*Castanopsis*	*Castanopsis carlesii*	JN044189
Eudicotyledons	Fagaceae	*Castanopsis*	*Castanopsis carlesii*	JN044190
Eudicotyledons	Fagaceae	*Castanopsis*	*Castanopsis carlesii*	JN044191
Eudicotyledons	Fagaceae	*Castanopsis*	*Castanopsis carlesii*	JN044192
Eudicotyledons	Fagaceae	*Castanopsis*	*Castanopsis carlesii*	JN044193
Eudicotyledons	Fagaceae	*Castanopsis*	*Castanopsis ceratacantha*	JN044194
Eudicotyledons	Fagaceae	*Castanopsis*	*Castanopsis ceratacantha*	JN044195
Eudicotyledons	Fagaceae	*Castanopsis*	*Castanopsis ceratacantha*	JN044196
Eudicotyledons	Fagaceae	*Castanopsis*	*Castanopsis ceratacantha*	JN044197
Eudicotyledons	Fagaceae	*Castanopsis*	*Castanopsis fargesii*	HQ427014
Eudicotyledons	Fagaceae	*Castanopsis*	*Castanopsis fargesii*	JN044208
Eudicotyledons	Fagaceae	*Castanopsis*	*Castanopsis fargesii*	JN044209
Eudicotyledons	Fagaceae	*Castanopsis*	*Castanopsis fargesii*	JN044210
Eudicotyledons	Fagaceae	*Castanopsis*	*Castanopsis fissa*	HQ415580
Eudicotyledons	Fagaceae	*Castanopsis*	*Castanopsis fissa*	JN044211
Eudicotyledons	Fagaceae	*Castanopsis*	*Castanopsis fissa*	JN044212
Eudicotyledons	Fagaceae	*Castanopsis*	*Castanopsis fissa*	JN044213
Eudicotyledons	Fagaceae	*Castanopsis*	*Castanopsis rockii*	JN044231
Eudicotyledons	Fagaceae	*Castanopsis*	*Castanopsis rockii*	JN044232
Eudicotyledons	Fagaceae	*Castanopsis*	*Castanopsis rockii*	JN044233
Eudicotyledons	Fabaceae	*Anthyllis*	*Anthyllis montana*	HM468248
Eudicotyledons	Fabaceae	*Anthyllis*	*Anthyllis montana*	HQ646221
Eudicotyledons	Fabaceae	*Anthyllis*	*Anthyllis aurea*	HM468236
Eudicotyledons	Fabaceae	*Anthyllis*	*Anthyllis aurea*	HQ646211
Eudicotyledons	Fabaceae	*Anthyllis*	*Anthyllis barba-jovis*	HM468237
Eudicotyledons	Fabaceae	*Anthyllis*	*Anthyllis barba-jovis*	HQ646212
Eudicotyledons	Fabaceae	*Anthyllis*	*Anthyllis barba-jovis*	HQ646213
Eudicotyledons	Fabaceae	*Anthyllis*	*Anthyllis barba-jovis*	HQ646214
Eudicotyledons	Cactaceae	*Rhipsalis*	*Rhipsalis teres*	FN669077
Eudicotyledons	Cactaceae	*Rhipsalis*	*Rhipsalis teres*	FN669078
Eudicotyledons	Cactaceae	*Rhipsalis*	*Rhipsalis teres*	FN669080
Eudicotyledons	Cactaceae	*Rhipsalis*	*Rhipsalis teres*	FN669159
Eudicotyledons	Ericaceae	*Enkianthus*	*Enkianthus chinensis*	JN044451
Eudicotyledons	Ericaceae	*Enkianthus*	*Enkianthus chinensis*	JN044452
Eudicotyledons	Asteraceae	*Galinsoga*	*Galinsoga parviflora*	GQ435097

Eudicotyledons	Asteraceae	*Galinsoga*	*Galinsoga parviflora*	DQ006151
Eudicotyledons	Betulaceae	*Carpinus*	*Carpinus japonica*	FJ011836
Eudicotyledons	Betulaceae	*Carpinus*	*Carpinus japonica*	FJ011837
Eudicotyledons	Betulaceae	*Carpinus*	*Carpinus japonica*	FJ011838
Eudicotyledons	Betulaceae	*Carpinus*	*Carpinus monbeigiana*	AY211438
Eudicotyledons	Betulaceae	*Carpinus*	*Carpinus monbeigiana*	AY211439
Eudicotyledons	Betulaceae	*Carpinus*	*Carpinus monbeigiana*	AY211440
Eudicotyledons	Betulaceae	*Carpinus*	*Carpinus monbeigiana*	AY211441
Eudicotyledons	Betulaceae	*Ostrya*	*Ostrya carpinifolia*	AY211451
Eudicotyledons	Betulaceae	*Ostrya*	*Ostrya carpinifolia*	FJ011860
Eudicotyledons	Betulaceae	*Ostrya*	*Ostrya japonica*	AY211452
Eudicotyledons	Betulaceae	*Ostrya*	*Ostrya japonica*	FJ011861
Eudicotyledons	Betulaceae	*Ostrya*	*Ostrya knowltonii*	AY211453
Eudicotyledons	Betulaceae	*Ostrya*	*Ostrya knowltonii*	FJ011862
Eudicotyledons	Vitaceae	*Tetrastigma*	*Tetrastigma trifoliolatum*	HM585780
Eudicotyledons	Vitaceae	*Tetrastigma*	*Tetrastigma trifoliolatum*	HM585781
Eudicotyledons	Vitaceae	*Tetrastigma*	*Tetrastigma obovatum*	HM585746
Eudicotyledons	Vitaceae	*Tetrastigma*	*Tetrastigma obovatum*	HM585747
Eudicotyledons	Vitaceae	*Tetrastigma*	*Tetrastigma obovatum*	HM585748
Eudicotyledons	Fabaceae	*Anthyllis*	*Anthyllis cornicina*	HM468239
Eudicotyledons	Fabaceae	*Anthyllis*	*Anthyllis cornicina*	HQ646223
Eudicotyledons	Fabaceae	*Anthyllis*	*Anthyllis hermanniae*	HM468243
Eudicotyledons	Fabaceae	*Anthyllis*	*Anthyllis hermanniae*	HQ646217
Eudicotyledons	Fabaceae	*Anthyllis*	*Anthyllis hermanniae*	HQ646218
Eudicotyledons	Fabaceae	*Anthyllis*	*Anthyllis lotoides*	HM468247
Eudicotyledons	Fabaceae	*Anthyllis*	*Anthyllis lotoides*	HQ646220
Eudicotyledons	Asteraceae	*Galinsoga*	*Galinsoga quadriradiata*	GU818377
Eudicotyledons	Asteraceae	*Galinsoga*	*Galinsoga quadriradiata*	HQ596705
Eudicotyledons	Asteraceae	*Galinsoga*	*Galinsoga quadriradiata*	AY215550
Eudicotyledons	Ericaceae	*Cassiope*	*Cassiope fastigiata*	JN044175
Eudicotyledons	Ericaceae	*Cassiope*	*Cassiope fastigiata*	JN044176
Eudicotyledons	Ericaceae	*Cassiope*	*Cassiope fastigiata*	JN044177
Eudicotyledons	Ericaceae	*Cassiope*	*Cassiope fastigiata*	JN044178
Eudicotyledons	Rosaceae	*Pyracantha*	*Pyracantha coccinea*	JQ390762
Eudicotyledons	Rosaceae	*Pyracantha*	*Pyracantha coccinea*	GQ305327
Eudicotyledons	Apocynaceae	*Caralluma*	*Caralluma arabica*	HM475411
Eudicotyledons	Apocynaceae	*Caralluma*	*Caralluma arabica*	HM475412
Eudicotyledons	Apocynaceae	*Caralluma*	*Caralluma flava*	HM475425
Eudicotyledons	Apocynaceae	*Caralluma*	*Caralluma flava*	HM475426
Eudicotyledons	Apocynaceae	*Caralluma*	*Caralluma penicillata*	HM475436
Eudicotyledons	Apocynaceae	*Caralluma*	*Caralluma penicillata*	HM475437
Eudicotyledons	Fabaceae	*Calliandra*	*Calliandra juzepczukii*	AF524959
Eudicotyledons	Fabaceae	*Calliandra*	*Calliandra juzepczukii*	EU811999
Eudicotyledons	Fabaceae	*Inga*	*Inga edulis*	GQ118870
Eudicotyledons	Fabaceae	*Inga*	*Inga edulis*	AF524976
Eudicotyledons	Fabaceae	*Inga*	*Inga edulis*	AM922019
Eudicotyledons	Fabaceae	*Inga*	*Inga edulis*	AM922020
Eudicotyledons	Fabaceae	*Inga*	*Inga edulis*	AM922021
Eudicotyledons	Primulaceae	*Omphalogramma*	*Omphalogramma vinciflorum*	JN045628
Eudicotyledons	Primulaceae	*Omphalogramma*	*Omphalogramma vinciflorum*	JN045629
Eudicotyledons	Primulaceae	*Omphalogramma*	*Omphalogramma vinciflorum*	JN045630
Eudicotyledons	Primulaceae	*Omphalogramma*	*Omphalogramma vinciflorum*	JN045631
Eudicotyledons	Primulaceae	*Omphalogramma*	*Omphalogramma vinciflorum*	JN045632

Eudicotyledons	Primulaceae	*Omphalogramma*	*Omphalogramma vinciflorum*	JN045633
Eudicotyledons	Primulaceae	*Omphalogramma*	*Omphalogramma vinciflorum*	JN045634
Eudicotyledons	Primulaceae	*Omphalogramma*	*Omphalogramma vinciflorum*	JN045635
Eudicotyledons	Primulaceae	*Omphalogramma*	*Omphalogramma vinciflorum*	JN045636
Eudicotyledons	Primulaceae	*Omphalogramma*	*Omphalogramma vinciflorum*	JN045637
Eudicotyledons	Fabaceae	*Anthyllis*	*Anthyllis hamosa*	HM468241
Eudicotyledons	Fabaceae	*Anthyllis*	*Anthyllis hamosa*	HQ646216
Eudicotyledons	Sapotaceae	*Chrysophyllum*	*Chrysophyllum cainito*	DQ344096
Eudicotyledons	Sapotaceae	*Chrysophyllum*	*Chrysophyllum cainito*	GQ982186
Eudicotyledons	Sapotaceae	*Chrysophyllum*	*Chrysophyllum argenteum*	FJ039052
Eudicotyledons	Sapotaceae	*Chrysophyllum*	*Chrysophyllum argenteum*	FJ039053
Eudicotyledons	Sapotaceae	*Chrysophyllum*	*Chrysophyllum argenteum*	GQ982185
Eudicotyledons	Burseraceae	*Protium*	*Protium decandrum*	GQ428774
Eudicotyledons	Burseraceae	*Protium*	*Protium decandrum*	FJ038870
Eudicotyledons	Burseraceae	*Protium*	*Protium opacum*	GQ428755
Eudicotyledons	Burseraceae	*Protium*	*Protium opacum*	GQ428756
Eudicotyledons	Burseraceae	*Protium*	*Protium opacum*	FJ038879
Eudicotyledons	Burseraceae	*Protium*	*Protium sagotianum*	GQ428757
Eudicotyledons	Burseraceae	*Protium*	*Protium sagotianum*	GQ428758
Eudicotyledons	Burseraceae	*Protium*	*Protium sagotianum*	FJ038876
Eudicotyledons	Burseraceae	*Protium*	*Protium sagotianum*	FJ038877
Eudicotyledons	Burseraceae	*Protium*	*Protium trifoliolatum*	GQ428761
Eudicotyledons	Burseraceae	*Protium*	*Protium trifoliolatum*	FJ038875
Eudicotyledons	Burseraceae	*Protium*	*Protium trifoliolatum*	FJ038878
Eudicotyledons	Fabaceae	*Inga*	*Inga punctata*	GQ118882
Eudicotyledons	Fabaceae	*Inga*	*Inga punctata*	GQ982259
Eudicotyledons	Fabaceae	*Inga*	*Inga punctata*	AM922040
Eudicotyledons	Fabaceae	*Inga*	*Inga punctata*	AM922041
Eudicotyledons	Fabaceae	*Inga*	*Inga punctata*	AM922042
Eudicotyledons	Fabaceae	*Inga*	*Inga punctata*	AM922043
Eudicotyledons	Fabaceae	*Inga*	*Inga punctata*	AM922054
Eudicotyledons	Asteraceae	*Gazania*	*Gazania tenuifolia*	EF556405
Eudicotyledons	Asteraceae	*Gazania*	*Gazania tenuifolia*	EF556406
Eudicotyledons	Plantaginaceae	*Synthyris*	*Synthyris alpina*	EU242526
Eudicotyledons	Plantaginaceae	*Synthyris*	*Synthyris alpina*	EU242527
Eudicotyledons	Plantaginaceae	*Synthyris*	*Synthyris alpina*	EU242528
Eudicotyledons	Plantaginaceae	*Synthyris*	*Synthyris alpina*	EU242529
Eudicotyledons	Plantaginaceae	*Synthyris*	*Synthyris alpina*	EU242530
Eudicotyledons	Plantaginaceae	*Synthyris*	*Synthyris alpina*	EU242531
Eudicotyledons	Plantaginaceae	*Synthyris*	*Synthyris alpina*	EU242532
Eudicotyledons	Plantaginaceae	*Synthyris*	*Synthyris alpina*	EU242533
Eudicotyledons	Plantaginaceae	*Synthyris*	*Synthyris alpina*	EU242534
Eudicotyledons	Plantaginaceae	*Synthyris*	*Synthyris alpina*	EU242535
Eudicotyledons	Plantaginaceae	*Synthyris*	*Synthyris alpina*	EU242536
Eudicotyledons	Plantaginaceae	*Synthyris*	*Synthyris alpina*	EU242537
Eudicotyledons	Plantaginaceae	*Synthyris*	*Synthyris alpina*	EU242538
Eudicotyledons	Plantaginaceae	*Synthyris*	*Synthyris alpina*	EU242539
Eudicotyledons	Plantaginaceae	*Synthyris*	*Synthyris alpina*	EU242540
Eudicotyledons	Plantaginaceae	*Synthyris*	*Synthyris alpina*	EU242541
Eudicotyledons	Plantaginaceae	*Synthyris*	*Synthyris alpina*	EU242542
Eudicotyledons	Plantaginaceae	*Synthyris*	*Synthyris alpina*	EU242543
Eudicotyledons	Plantaginaceae	*Synthyris*	*Synthyris alpina*	EU242544
Eudicotyledons	Plantaginaceae	*Synthyris*	*Synthyris alpina*	EU242545

Eudicotyledons	Plantaginaceae	*Synthyris*	*Synthyris alpina*	EU242546
Eudicotyledons	Plantaginaceae	*Synthyris*	*Synthyris alpina*	EU242547
Eudicotyledons	Plantaginaceae	*Synthyris*	*Synthyris alpina*	EU242548
Eudicotyledons	Plantaginaceae	*Synthyris*	*Synthyris alpina*	EU242549
Eudicotyledons	Plantaginaceae	*Synthyris*	*Synthyris alpina*	EU242550
Eudicotyledons	Plantaginaceae	*Synthyris*	*Synthyris alpina*	EU242551
Eudicotyledons	Plantaginaceae	*Synthyris*	*Synthyris alpina*	EU242552
Eudicotyledons	Plantaginaceae	*Synthyris*	*Synthyris alpina*	EU242553
Eudicotyledons	Plantaginaceae	*Synthyris*	*Synthyris alpina*	EU242554
Eudicotyledons	Plantaginaceae	*Synthyris*	*Synthyris alpina*	EU242555
Eudicotyledons	Plantaginaceae	*Synthyris*	*Synthyris alpina*	EU242556
Eudicotyledons	Plantaginaceae	*Synthyris*	*Synthyris alpina*	EU242557
Eudicotyledons	Plantaginaceae	*Synthyris*	*Synthyris alpina*	EU242558
Eudicotyledons	Plantaginaceae	*Synthyris*	*Synthyris alpina*	EU242559
Eudicotyledons	Plantaginaceae	*Synthyris*	*Synthyris alpina*	EU242560
Eudicotyledons	Plantaginaceae	*Synthyris*	*Synthyris alpina*	EU242561
Eudicotyledons	Plantaginaceae	*Synthyris*	*Synthyris alpina*	EU242562
Eudicotyledons	Plantaginaceae	*Synthyris*	*Synthyris alpina*	EU242563
Eudicotyledons	Plantaginaceae	*Synthyris*	*Synthyris alpina*	EU242564
Eudicotyledons	Plantaginaceae	*Synthyris*	*Synthyris alpina*	EU242565
Eudicotyledons	Plantaginaceae	*Synthyris*	*Synthyris alpina*	EU242566
Eudicotyledons	Plantaginaceae	*Synthyris*	*Synthyris alpina*	EU242567
Eudicotyledons	Plantaginaceae	*Synthyris*	*Synthyris alpina*	EU242568
Eudicotyledons	Plantaginaceae	*Synthyris*	*Synthyris alpina*	EU242569
Eudicotyledons	Plantaginaceae	*Synthyris*	*Synthyris alpina*	EU242570
Eudicotyledons	Plantaginaceae	*Synthyris*	*Synthyris alpina*	EU242571
Eudicotyledons	Plantaginaceae	*Synthyris*	*Synthyris alpina*	EU242572
Eudicotyledons	Plantaginaceae	*Synthyris*	*Synthyris alpina*	EU242573
Eudicotyledons	Plantaginaceae	*Synthyris*	*Synthyris alpina*	EU242574
Eudicotyledons	Plantaginaceae	*Synthyris*	*Synthyris alpina*	EU242575
Eudicotyledons	Plantaginaceae	*Synthyris*	*Synthyris alpina*	EU242576
Eudicotyledons	Plantaginaceae	*Synthyris*	*Synthyris alpina*	EU242577
Eudicotyledons	Plantaginaceae	*Synthyris*	*Synthyris alpina*	EU242578
Eudicotyledons	Plantaginaceae	*Synthyris*	*Synthyris alpina*	EU242579
Eudicotyledons	Plantaginaceae	*Synthyris*	*Synthyris alpina*	EU242580
Eudicotyledons	Plantaginaceae	*Synthyris*	*Synthyris alpina*	EU242581
Eudicotyledons	Plantaginaceae	*Synthyris*	*Synthyris alpina*	EU242582
Eudicotyledons	Plantaginaceae	*Synthyris*	*Synthyris alpina*	EU242583
Eudicotyledons	Plantaginaceae	*Synthyris*	*Synthyris alpina*	EU242584
Eudicotyledons	Plantaginaceae	*Synthyris*	*Synthyris alpina*	EU242585
Eudicotyledons	Plantaginaceae	*Synthyris*	*Synthyris alpina*	EU242586
Eudicotyledons	Plantaginaceae	*Synthyris*	*Synthyris alpina*	EU242587
Eudicotyledons	Plantaginaceae	*Synthyris*	*Synthyris alpina*	EU242588
Eudicotyledons	Plantaginaceae	*Synthyris*	*Synthyris alpina*	EU242589
Eudicotyledons	Plantaginaceae	*Synthyris*	*Synthyris alpina*	EU242590
Eudicotyledons	Plantaginaceae	*Synthyris*	*Synthyris alpina*	EU242591
Eudicotyledons	Plantaginaceae	*Synthyris*	*Synthyris alpina*	EU242592
Eudicotyledons	Plantaginaceae	*Synthyris*	*Synthyris alpina*	EU242593
Eudicotyledons	Plantaginaceae	*Synthyris*	*Synthyris alpina*	EU242594
Eudicotyledons	Plantaginaceae	*Synthyris*	*Synthyris alpina*	EU242595
Eudicotyledons	Plantaginaceae	*Synthyris*	*Synthyris alpina*	EU242596
Eudicotyledons	Plantaginaceae	*Synthyris*	*Synthyris alpina*	EU242597
Eudicotyledons	Plantaginaceae	*Synthyris*	*Synthyris alpina*	EU242598

Eudicotyledons	Plantaginaceae	*Synthyris*	*Synthyris alpina*	EU242599
Eudicotyledons	Plantaginaceae	*Synthyris*	*Synthyris alpina*	EU242600
Eudicotyledons	Plantaginaceae	*Synthyris*	*Synthyris alpina*	EU242601
Eudicotyledons	Plantaginaceae	*Synthyris*	*Synthyris alpina*	EU242602
Eudicotyledons	Plantaginaceae	*Synthyris*	*Synthyris alpina*	EU242603
Eudicotyledons	Plantaginaceae	*Synthyris*	*Synthyris alpina*	EU242604
Eudicotyledons	Plantaginaceae	*Synthyris*	*Synthyris alpina*	EU242605
Eudicotyledons	Plantaginaceae	*Synthyris*	*Synthyris alpina*	EU242606
Eudicotyledons	Plantaginaceae	*Synthyris*	*Synthyris alpina*	EU242607
Eudicotyledons	Plantaginaceae	*Synthyris*	*Synthyris alpina*	EU242608
Eudicotyledons	Plantaginaceae	*Synthyris*	*Synthyris alpina*	EU242609
Eudicotyledons	Plantaginaceae	*Synthyris*	*Synthyris alpina*	EU242612
Eudicotyledons	Plantaginaceae	*Synthyris*	*Synthyris alpina*	EU242613
Eudicotyledons	Plantaginaceae	*Synthyris*	*Synthyris alpina*	EU242614
Eudicotyledons	Plantaginaceae	*Synthyris*	*Synthyris alpina*	EU242615
Eudicotyledons	Plantaginaceae	*Synthyris*	*Synthyris alpina*	EU242616
Eudicotyledons	Plantaginaceae	*Synthyris*	*Synthyris alpina*	EU242617
Eudicotyledons	Plantaginaceae	*Synthyris*	*Synthyris alpina*	EU242618
Eudicotyledons	Plantaginaceae	*Synthyris*	*Synthyris alpina*	EU242619
Eudicotyledons	Plantaginaceae	*Synthyris*	*Synthyris alpina*	EU242620
Eudicotyledons	Plantaginaceae	*Synthyris*	*Synthyris alpina*	EF550053
Eudicotyledons	Plantaginaceae	*Synthyris*	*Synthyris alpina*	EF550054
Eudicotyledons	Plantaginaceae	*Synthyris*	*Synthyris alpina*	EF550055
Eudicotyledons	Plantaginaceae	*Synthyris*	*Synthyris alpina*	EF550056
Eudicotyledons	Plantaginaceae	*Synthyris*	*Synthyris alpina*	EF550057
Eudicotyledons	Plantaginaceae	*Synthyris*	*Synthyris alpina*	EF550058
Eudicotyledons	Plantaginaceae	*Synthyris*	*Synthyris alpina*	EF550059
Eudicotyledons	Plantaginaceae	*Synthyris*	*Synthyris alpina*	EF550060
Eudicotyledons	Plantaginaceae	*Synthyris*	*Synthyris alpina*	EF550061
Eudicotyledons	Plantaginaceae	*Synthyris*	*Synthyris alpina*	EF550062
Eudicotyledons	Plantaginaceae	*Synthyris*	*Synthyris alpina*	EF550063
Eudicotyledons	Plantaginaceae	*Synthyris*	*Synthyris alpina*	EF550064
Eudicotyledons	Plantaginaceae	*Synthyris*	*Synthyris alpina*	EF550065
Eudicotyledons	Plantaginaceae	*Synthyris*	*Synthyris alpina*	EF550066
Eudicotyledons	Plantaginaceae	*Synthyris*	*Synthyris alpina*	EF550067
Eudicotyledons	Plantaginaceae	*Synthyris*	*Synthyris oblongifolia*	EU242621
Eudicotyledons	Plantaginaceae	*Synthyris*	*Synthyris oblongifolia*	EU242634
Eudicotyledons	Plantaginaceae	*Synthyris*	*Synthyris oblongifolia*	EU242635
Eudicotyledons	Plantaginaceae	*Synthyris*	*Synthyris oblongifolia*	EU242636
Eudicotyledons	Plantaginaceae	*Synthyris*	*Synthyris oblongifolia*	EU242637
Eudicotyledons	Plantaginaceae	*Synthyris*	*Synthyris oblongifolia*	EU242638
Eudicotyledons	Plantaginaceae	*Synthyris*	*Synthyris oblongifolia*	EU242639
Eudicotyledons	Plantaginaceae	*Synthyris*	*Synthyris oblongifolia*	EU242640
Eudicotyledons	Plantaginaceae	*Synthyris*	*Synthyris oblongifolia*	EU242641
Eudicotyledons	Plantaginaceae	*Synthyris*	*Synthyris oblongifolia*	EF550068
Eudicotyledons	Plantaginaceae	*Synthyris*	*Synthyris oblongifolia*	EF550069
Eudicotyledons	Plantaginaceae	*Synthyris*	*Synthyris oblongifolia*	EF550070
Eudicotyledons	Plantaginaceae	*Synthyris*	*Synthyris plantaginea*	EU242622
Eudicotyledons	Plantaginaceae	*Synthyris*	*Synthyris plantaginea*	EU242623
Eudicotyledons	Plantaginaceae	*Synthyris*	*Synthyris plantaginea*	EU242624
Eudicotyledons	Plantaginaceae	*Synthyris*	*Synthyris plantaginea*	EU242625
Eudicotyledons	Plantaginaceae	*Synthyris*	*Synthyris plantaginea*	EU242626
Eudicotyledons	Plantaginaceae	*Synthyris*	*Synthyris plantaginea*	EU242627

Eudicotyledons	Plantaginaceae	*Synthyris*	*Synthyris plantaginea*	EU242628
Eudicotyledons	Plantaginaceae	*Synthyris*	*Synthyris plantaginea*	EU242629
Eudicotyledons	Plantaginaceae	*Synthyris*	*Synthyris plantaginea*	EU242630
Eudicotyledons	Plantaginaceae	*Synthyris*	*Synthyris plantaginea*	EU242631
Eudicotyledons	Plantaginaceae	*Synthyris*	*Synthyris plantaginea*	EU242632
Eudicotyledons	Plantaginaceae	*Synthyris*	*Synthyris plantaginea*	EU242633
Eudicotyledons	Plantaginaceae	*Synthyris*	*Synthyris plantaginea*	EU242643
Eudicotyledons	Plantaginaceae	*Synthyris*	*Synthyris plantaginea*	EU242644
Eudicotyledons	Plantaginaceae	*Synthyris*	*Synthyris plantaginea*	EU242645
Eudicotyledons	Plantaginaceae	*Synthyris*	*Synthyris plantaginea*	EU242646
Eudicotyledons	Plantaginaceae	*Synthyris*	*Synthyris plantaginea*	EU242647
Eudicotyledons	Plantaginaceae	*Synthyris*	*Synthyris plantaginea*	EU242648
Eudicotyledons	Plantaginaceae	*Synthyris*	*Synthyris plantaginea*	EU242649
Eudicotyledons	Plantaginaceae	*Synthyris*	*Synthyris plantaginea*	EU242650
Eudicotyledons	Plantaginaceae	*Synthyris*	*Synthyris plantaginea*	EU242651
Eudicotyledons	Plantaginaceae	*Synthyris*	*Synthyris plantaginea*	EU242652
Eudicotyledons	Plantaginaceae	*Synthyris*	*Synthyris plantaginea*	EU242653
Eudicotyledons	Plantaginaceae	*Synthyris*	*Synthyris plantaginea*	EU242654
Eudicotyledons	Plantaginaceae	*Synthyris*	*Synthyris plantaginea*	EU242655
Eudicotyledons	Plantaginaceae	*Synthyris*	*Synthyris plantaginea*	EU242656
Eudicotyledons	Plantaginaceae	*Synthyris*	*Synthyris plantaginea*	EU242657
Eudicotyledons	Plantaginaceae	*Synthyris*	*Synthyris plantaginea*	EU242658
Eudicotyledons	Plantaginaceae	*Synthyris*	*Synthyris plantaginea*	EU242659
Eudicotyledons	Plantaginaceae	*Synthyris*	*Synthyris plantaginea*	EU242660
Eudicotyledons	Plantaginaceae	*Synthyris*	*Synthyris plantaginea*	EU242661
Eudicotyledons	Plantaginaceae	*Synthyris*	*Synthyris plantaginea*	EU242662
Eudicotyledons	Plantaginaceae	*Synthyris*	*Synthyris plantaginea*	EU242663
Eudicotyledons	Plantaginaceae	*Synthyris*	*Synthyris plantaginea*	EU242664
Eudicotyledons	Plantaginaceae	*Synthyris*	*Synthyris plantaginea*	EU242665
Eudicotyledons	Plantaginaceae	*Synthyris*	*Synthyris plantaginea*	EU242666
Eudicotyledons	Plantaginaceae	*Synthyris*	*Synthyris plantaginea*	EU242667
Eudicotyledons	Plantaginaceae	*Synthyris*	*Synthyris plantaginea*	EU242668
Eudicotyledons	Plantaginaceae	*Synthyris*	*Synthyris plantaginea*	EU242669
Eudicotyledons	Plantaginaceae	*Synthyris*	*Synthyris plantaginea*	EU242670
Eudicotyledons	Plantaginaceae	*Synthyris*	*Synthyris plantaginea*	EU242671
Eudicotyledons	Plantaginaceae	*Synthyris*	*Synthyris plantaginea*	EU242672
Eudicotyledons	Plantaginaceae	*Synthyris*	*Synthyris plantaginea*	EU242673
Eudicotyledons	Plantaginaceae	*Synthyris*	*Synthyris plantaginea*	EU242674
Eudicotyledons	Plantaginaceae	*Synthyris*	*Synthyris plantaginea*	EU242675
Eudicotyledons	Plantaginaceae	*Synthyris*	*Synthyris plantaginea*	EU242676
Eudicotyledons	Plantaginaceae	*Synthyris*	*Synthyris plantaginea*	EU242677
Eudicotyledons	Plantaginaceae	*Synthyris*	*Synthyris plantaginea*	EU242678
Eudicotyledons	Plantaginaceae	*Synthyris*	*Synthyris plantaginea*	EU242679
Eudicotyledons	Plantaginaceae	*Synthyris*	*Synthyris plantaginea*	EU242680
Eudicotyledons	Plantaginaceae	*Synthyris*	*Synthyris plantaginea*	EU242681
Eudicotyledons	Plantaginaceae	*Synthyris*	*Synthyris plantaginea*	EU242682
Eudicotyledons	Plantaginaceae	*Synthyris*	*Synthyris plantaginea*	EU242683
Eudicotyledons	Plantaginaceae	*Synthyris*	*Synthyris plantaginea*	EU242684
Eudicotyledons	Plantaginaceae	*Synthyris*	*Synthyris plantaginea*	EU242685
Eudicotyledons	Plantaginaceae	*Synthyris*	*Synthyris plantaginea*	EU242686
Eudicotyledons	Plantaginaceae	*Synthyris*	*Synthyris plantaginea*	EU242687
Eudicotyledons	Plantaginaceae	*Synthyris*	*Synthyris plantaginea*	EU242688
Eudicotyledons	Plantaginaceae	*Synthyris*	*Synthyris plantaginea*	EU242689

Eudicotyledons	Plantaginaceae	*Synthyris*	*Synthyris plantaginea*	EU242690
Eudicotyledons	Plantaginaceae	*Synthyris*	*Synthyris plantaginea*	EU242691
Eudicotyledons	Plantaginaceae	*Synthyris*	*Synthyris plantaginea*	EU242692
Eudicotyledons	Plantaginaceae	*Synthyris*	*Synthyris plantaginea*	EU242693
Eudicotyledons	Plantaginaceae	*Synthyris*	*Synthyris plantaginea*	EU242694
Eudicotyledons	Plantaginaceae	*Synthyris*	*Synthyris plantaginea*	EU242695
Eudicotyledons	Plantaginaceae	*Synthyris*	*Synthyris plantaginea*	EU242696
Eudicotyledons	Plantaginaceae	*Synthyris*	*Synthyris plantaginea*	EU242697
Eudicotyledons	Plantaginaceae	*Synthyris*	*Synthyris plantaginea*	EU242698
Eudicotyledons	Plantaginaceae	*Synthyris*	*Synthyris plantaginea*	EU242699
Eudicotyledons	Plantaginaceae	*Synthyris*	*Synthyris plantaginea*	EU242700
Eudicotyledons	Plantaginaceae	*Synthyris*	*Synthyris plantaginea*	EU242701
Eudicotyledons	Plantaginaceae	*Synthyris*	*Synthyris plantaginea*	EU242702
Eudicotyledons	Plantaginaceae	*Synthyris*	*Synthyris plantaginea*	EU242703
Eudicotyledons	Plantaginaceae	*Synthyris*	*Synthyris plantaginea*	EU242704
Eudicotyledons	Plantaginaceae	*Synthyris*	*Synthyris plantaginea*	EU242714
Eudicotyledons	Plantaginaceae	*Synthyris*	*Synthyris plantaginea*	EU242715
Eudicotyledons	Plantaginaceae	*Synthyris*	*Synthyris plantaginea*	EU242716
Eudicotyledons	Plantaginaceae	*Synthyris*	*Synthyris plantaginea*	EU242717
Eudicotyledons	Plantaginaceae	*Synthyris*	*Synthyris plantaginea*	EU242718
Eudicotyledons	Plantaginaceae	*Synthyris*	*Synthyris plantaginea*	EU242719
Eudicotyledons	Plantaginaceae	*Synthyris*	*Synthyris plantaginea*	EU242720
Eudicotyledons	Plantaginaceae	*Synthyris*	*Synthyris plantaginea*	EU242721
Eudicotyledons	Plantaginaceae	*Synthyris*	*Synthyris plantaginea*	EU242722
Eudicotyledons	Plantaginaceae	*Synthyris*	*Synthyris plantaginea*	EU242723
Eudicotyledons	Plantaginaceae	*Synthyris*	*Synthyris plantaginea*	EU242724
Eudicotyledons	Plantaginaceae	*Synthyris*	*Synthyris plantaginea*	EU242725
Eudicotyledons	Plantaginaceae	*Synthyris*	*Synthyris plantaginea*	EU242726
Eudicotyledons	Plantaginaceae	*Synthyris*	*Synthyris plantaginea*	EU242727
Eudicotyledons	Plantaginaceae	*Synthyris*	*Synthyris plantaginea*	EU242728
Eudicotyledons	Plantaginaceae	*Synthyris*	*Synthyris plantaginea*	EU242729
Eudicotyledons	Plantaginaceae	*Synthyris*	*Synthyris plantaginea*	EU242730
Eudicotyledons	Plantaginaceae	*Synthyris*	*Synthyris plantaginea*	EU242731
Eudicotyledons	Plantaginaceae	*Synthyris*	*Synthyris plantaginea*	EU242732
Eudicotyledons	Plantaginaceae	*Synthyris*	*Synthyris plantaginea*	EU242733
Eudicotyledons	Plantaginaceae	*Synthyris*	*Synthyris plantaginea*	EU242734
Eudicotyledons	Plantaginaceae	*Synthyris*	*Synthyris plantaginea*	EU242735
Eudicotyledons	Plantaginaceae	*Synthyris*	*Synthyris plantaginea*	EU242736
Eudicotyledons	Plantaginaceae	*Synthyris*	*Synthyris plantaginea*	EU242737
Eudicotyledons	Plantaginaceae	*Synthyris*	*Synthyris plantaginea*	EU242738
Eudicotyledons	Plantaginaceae	*Synthyris*	*Synthyris plantaginea*	EU242739
Eudicotyledons	Plantaginaceae	*Synthyris*	*Synthyris plantaginea*	EU242740
Eudicotyledons	Plantaginaceae	*Synthyris*	*Synthyris plantaginea*	EU242741
Eudicotyledons	Plantaginaceae	*Synthyris*	*Synthyris plantaginea*	EU242742
Eudicotyledons	Plantaginaceae	*Synthyris*	*Synthyris plantaginea*	EU242743
Eudicotyledons	Plantaginaceae	*Synthyris*	*Synthyris plantaginea*	EU242744
Eudicotyledons	Plantaginaceae	*Synthyris*	*Synthyris plantaginea*	EU242745
Eudicotyledons	Plantaginaceae	*Synthyris*	*Synthyris plantaginea*	EU242746
Eudicotyledons	Plantaginaceae	*Synthyris*	*Synthyris plantaginea*	EU242747
Eudicotyledons	Plantaginaceae	*Synthyris*	*Synthyris plantaginea*	EU242748
Eudicotyledons	Plantaginaceae	*Synthyris*	*Synthyris plantaginea*	EU242749
Eudicotyledons	Plantaginaceae	*Synthyris*	*Synthyris plantaginea*	EU242750
Eudicotyledons	Plantaginaceae	*Synthyris*	*Synthyris plantaginea*	EU242751

Eudicotyledons	Plantaginaceae	*Synthyris*	*Synthyris plantaginea*	EU242752
Eudicotyledons	Plantaginaceae	*Synthyris*	*Synthyris plantaginea*	EU242753
Eudicotyledons	Plantaginaceae	*Synthyris*	*Synthyris plantaginea*	EU242754
Eudicotyledons	Plantaginaceae	*Synthyris*	*Synthyris plantaginea*	EU242755
Eudicotyledons	Plantaginaceae	*Synthyris*	*Synthyris plantaginea*	EU242756
Eudicotyledons	Plantaginaceae	*Synthyris*	*Synthyris plantaginea*	EU242757
Eudicotyledons	Plantaginaceae	*Synthyris*	*Synthyris plantaginea*	EU242758
Eudicotyledons	Plantaginaceae	*Synthyris*	*Synthyris plantaginea*	EU242759
Eudicotyledons	Plantaginaceae	*Synthyris*	*Synthyris plantaginea*	EU242760
Eudicotyledons	Plantaginaceae	*Synthyris*	*Synthyris plantaginea*	EU242761
Eudicotyledons	Plantaginaceae	*Synthyris*	*Synthyris plantaginea*	EU242762
Eudicotyledons	Plantaginaceae	*Synthyris*	*Synthyris plantaginea*	EU242763
Eudicotyledons	Plantaginaceae	*Synthyris*	*Synthyris plantaginea*	EU242764
Eudicotyledons	Plantaginaceae	*Synthyris*	*Synthyris plantaginea*	EU242765
Eudicotyledons	Plantaginaceae	*Synthyris*	*Synthyris plantaginea*	EU242766
Eudicotyledons	Plantaginaceae	*Synthyris*	*Synthyris plantaginea*	EU242767
Eudicotyledons	Plantaginaceae	*Synthyris*	*Synthyris plantaginea*	EU242768
Eudicotyledons	Plantaginaceae	*Synthyris*	*Synthyris plantaginea*	EU242769
Eudicotyledons	Plantaginaceae	*Synthyris*	*Synthyris plantaginea*	EU242770
Eudicotyledons	Plantaginaceae	*Synthyris*	*Synthyris plantaginea*	EU242771
Eudicotyledons	Plantaginaceae	*Synthyris*	*Synthyris plantaginea*	EU242772
Eudicotyledons	Plantaginaceae	*Synthyris*	*Synthyris plantaginea*	EU242773
Eudicotyledons	Plantaginaceae	*Synthyris*	*Synthyris plantaginea*	EU242774
Eudicotyledons	Plantaginaceae	*Synthyris*	*Synthyris plantaginea*	EU242775
Eudicotyledons	Plantaginaceae	*Synthyris*	*Synthyris plantaginea*	EU242776
Eudicotyledons	Plantaginaceae	*Synthyris*	*Synthyris plantaginea*	EU242777
Eudicotyledons	Plantaginaceae	*Synthyris*	*Synthyris plantaginea*	EU242778
Eudicotyledons	Plantaginaceae	*Synthyris*	*Synthyris plantaginea*	EU242779
Eudicotyledons	Plantaginaceae	*Synthyris*	*Synthyris plantaginea*	EU242780
Eudicotyledons	Plantaginaceae	*Synthyris*	*Synthyris plantaginea*	EU242781
Eudicotyledons	Plantaginaceae	*Synthyris*	*Synthyris plantaginea*	EU242782
Eudicotyledons	Plantaginaceae	*Synthyris*	*Synthyris plantaginea*	EU242783
Eudicotyledons	Plantaginaceae	*Synthyris*	*Synthyris plantaginea*	EU242784
Eudicotyledons	Plantaginaceae	*Synthyris*	*Synthyris plantaginea*	EU242785
Eudicotyledons	Plantaginaceae	*Synthyris*	*Synthyris plantaginea*	EU242786
Eudicotyledons	Plantaginaceae	*Synthyris*	*Synthyris plantaginea*	EU242787
Eudicotyledons	Plantaginaceae	*Synthyris*	*Synthyris plantaginea*	EU242788
Eudicotyledons	Plantaginaceae	*Synthyris*	*Synthyris plantaginea*	EU242789
Eudicotyledons	Plantaginaceae	*Synthyris*	*Synthyris plantaginea*	EU242790
Eudicotyledons	Plantaginaceae	*Synthyris*	*Synthyris plantaginea*	EU242791
Eudicotyledons	Plantaginaceae	*Synthyris*	*Synthyris plantaginea*	EU242792
Eudicotyledons	Plantaginaceae	*Synthyris*	*Synthyris plantaginea*	EF550071
Eudicotyledons	Plantaginaceae	*Synthyris*	*Synthyris plantaginea*	EF550072
Eudicotyledons	Plantaginaceae	*Synthyris*	*Synthyris plantaginea*	EF550073
Eudicotyledons	Plantaginaceae	*Synthyris*	*Synthyris plantaginea*	EF550074
Eudicotyledons	Plantaginaceae	*Synthyris*	*Synthyris plantaginea*	EF550075
Eudicotyledons	Plantaginaceae	*Synthyris*	*Synthyris plantaginea*	EF550076
Eudicotyledons	Plantaginaceae	*Synthyris*	*Synthyris plantaginea*	EF550077
Eudicotyledons	Plantaginaceae	*Synthyris*	*Synthyris plantaginea*	EF550078
Eudicotyledons	Plantaginaceae	*Synthyris*	*Synthyris plantaginea*	EF550079
Eudicotyledons	Plantaginaceae	*Synthyris*	*Synthyris plantaginea*	EF550080
Eudicotyledons	Plantaginaceae	*Synthyris*	*Synthyris plantaginea*	EF550081
Eudicotyledons	Plantaginaceae	*Synthyris*	*Synthyris plantaginea*	EF550082

Eudicotyledons	Plantaginaceae	*Synthyris*	*Synthyris plantaginea*	EF550083
Eudicotyledons	Plantaginaceae	*Synthyris*	*Synthyris ritteriana*	EU242610
Eudicotyledons	Plantaginaceae	*Synthyris*	*Synthyris ritteriana*	EU242611
Eudicotyledons	Plantaginaceae	*Synthyris*	*Synthyris ritteriana*	EU242642
Eudicotyledons	Plantaginaceae	*Synthyris*	*Synthyris ritteriana*	EU242705
Eudicotyledons	Plantaginaceae	*Synthyris*	*Synthyris ritteriana*	EU242706
Eudicotyledons	Plantaginaceae	*Synthyris*	*Synthyris ritteriana*	EU242707
Eudicotyledons	Plantaginaceae	*Synthyris*	*Synthyris ritteriana*	EU242708
Eudicotyledons	Plantaginaceae	*Synthyris*	*Synthyris ritteriana*	EU242709
Eudicotyledons	Plantaginaceae	*Synthyris*	*Synthyris ritteriana*	EU242710
Eudicotyledons	Plantaginaceae	*Synthyris*	*Synthyris ritteriana*	EU242711
Eudicotyledons	Plantaginaceae	*Synthyris*	*Synthyris ritteriana*	EU242712
Eudicotyledons	Plantaginaceae	*Synthyris*	*Synthyris ritteriana*	EU242713
Eudicotyledons	Plantaginaceae	*Synthyris*	*Synthyris ritteriana*	EU242793
Eudicotyledons	Plantaginaceae	*Synthyris*	*Synthyris ritteriana*	EU242794
Eudicotyledons	Plantaginaceae	*Synthyris*	*Synthyris ritteriana*	EU242795
Eudicotyledons	Plantaginaceae	*Synthyris*	*Synthyris ritteriana*	EU242796
Eudicotyledons	Plantaginaceae	*Synthyris*	*Synthyris ritteriana*	EU242797
Eudicotyledons	Plantaginaceae	*Synthyris*	*Synthyris ritteriana*	EU242798
Eudicotyledons	Plantaginaceae	*Synthyris*	*Synthyris ritteriana*	EU242799
Eudicotyledons	Plantaginaceae	*Synthyris*	*Synthyris ritteriana*	EU242800
Eudicotyledons	Plantaginaceae	*Synthyris*	*Synthyris ritteriana*	EU242801
Eudicotyledons	Plantaginaceae	*Synthyris*	*Synthyris ritteriana*	EU242802
Eudicotyledons	Plantaginaceae	*Synthyris*	*Synthyris ritteriana*	EU242803
Eudicotyledons	Plantaginaceae	*Synthyris*	*Synthyris ritteriana*	EU242804
Eudicotyledons	Plantaginaceae	*Synthyris*	*Synthyris ritteriana*	EU242805
Eudicotyledons	Plantaginaceae	*Synthyris*	*Synthyris ritteriana*	EU242806
Eudicotyledons	Plantaginaceae	*Synthyris*	*Synthyris ritteriana*	EU242807
Eudicotyledons	Plantaginaceae	*Synthyris*	*Synthyris ritteriana*	EU242808
Eudicotyledons	Plantaginaceae	*Synthyris*	*Synthyris ritteriana*	EU242809
Eudicotyledons	Plantaginaceae	*Synthyris*	*Synthyris ritteriana*	EU242810
Eudicotyledons	Plantaginaceae	*Synthyris*	*Synthyris ritteriana*	EU242811
Eudicotyledons	Plantaginaceae	*Synthyris*	*Synthyris ritteriana*	EU242812
Eudicotyledons	Plantaginaceae	*Synthyris*	*Synthyris ritteriana*	EU242813
Eudicotyledons	Plantaginaceae	*Synthyris*	*Synthyris ritteriana*	EU242814
Eudicotyledons	Plantaginaceae	*Synthyris*	*Synthyris ritteriana*	EU242815
Eudicotyledons	Plantaginaceae	*Synthyris*	*Synthyris ritteriana*	EU242816
Eudicotyledons	Plantaginaceae	*Synthyris*	*Synthyris ritteriana*	EU242817
Eudicotyledons	Plantaginaceae	*Synthyris*	*Synthyris ritteriana*	EU242818
Eudicotyledons	Plantaginaceae	*Synthyris*	*Synthyris ritteriana*	EU242819
Eudicotyledons	Plantaginaceae	*Synthyris*	*Synthyris ritteriana*	EU242820
Eudicotyledons	Plantaginaceae	*Synthyris*	*Synthyris ritteriana*	EU242821
Eudicotyledons	Plantaginaceae	*Synthyris*	*Synthyris ritteriana*	EU242822
Eudicotyledons	Plantaginaceae	*Synthyris*	*Synthyris ritteriana*	EU242823
Eudicotyledons	Plantaginaceae	*Synthyris*	*Synthyris ritteriana*	EU242824
Eudicotyledons	Plantaginaceae	*Synthyris*	*Synthyris ritteriana*	EU242825
Eudicotyledons	Plantaginaceae	*Synthyris*	*Synthyris ritteriana*	EF550084
Eudicotyledons	Plantaginaceae	*Synthyris*	*Synthyris ritteriana*	EF550085
Eudicotyledons	Plantaginaceae	*Synthyris*	*Synthyris ritteriana*	EF550086
Eudicotyledons	Plantaginaceae	*Synthyris*	*Synthyris canbyi*	EU232407
Eudicotyledons	Plantaginaceae	*Synthyris*	*Synthyris canbyi*	EU232408
Eudicotyledons	Plantaginaceae	*Synthyris*	*Synthyris canbyi*	EU232409
Eudicotyledons	Plantaginaceae	*Synthyris*	*Synthyris canbyi*	EU232410

Eudicotyledons	Plantaginaceae	*Synthyris*	*Synthyris canbyi*	EU232411
Eudicotyledons	Plantaginaceae	*Synthyris*	*Synthyris canbyi*	EU232412
Eudicotyledons	Plantaginaceae	*Synthyris*	*Synthyris canbyi*	EU232413
Eudicotyledons	Plantaginaceae	*Synthyris*	*Synthyris canbyi*	EU232414
Eudicotyledons	Plantaginaceae	*Synthyris*	*Synthyris canbyi*	EU232415
Eudicotyledons	Plantaginaceae	*Synthyris*	*Synthyris canbyi*	EU232416
Eudicotyledons	Plantaginaceae	*Synthyris*	*Synthyris canbyi*	EU232417
Eudicotyledons	Plantaginaceae	*Synthyris*	*Synthyris canbyi*	EU232418
Eudicotyledons	Plantaginaceae	*Synthyris*	*Synthyris canbyi*	EU232419
Eudicotyledons	Plantaginaceae	*Synthyris*	*Synthyris canbyi*	EU232420
Eudicotyledons	Plantaginaceae	*Synthyris*	*Synthyris canbyi*	EU232421
Eudicotyledons	Plantaginaceae	*Synthyris*	*Synthyris canbyi*	EF550019
Eudicotyledons	Plantaginaceae	*Synthyris*	*Synthyris pinnatifida*	AY721961
Eudicotyledons	Plantaginaceae	*Synthyris*	*Synthyris pinnatifida*	AY721962
Eudicotyledons	Plantaginaceae	*Synthyris*	*Synthyris pinnatifida*	AY721963
Eudicotyledons	Plantaginaceae	*Synthyris*	*Synthyris pinnatifida*	AY721964
Eudicotyledons	Plantaginaceae	*Synthyris*	*Synthyris pinnatifida*	AY721965
Eudicotyledons	Plantaginaceae	*Synthyris*	*Synthyris pinnatifida*	AY721966
Eudicotyledons	Plantaginaceae	*Synthyris*	*Synthyris pinnatifida*	AY721967
Eudicotyledons	Plantaginaceae	*Synthyris*	*Synthyris pinnatifida*	AY721968
Eudicotyledons	Plantaginaceae	*Synthyris*	*Synthyris pinnatifida*	AY721969
Eudicotyledons	Plantaginaceae	*Synthyris*	*Synthyris pinnatifida*	AY721970
Eudicotyledons	Plantaginaceae	*Synthyris*	*Synthyris dissecta*	EU232348
Eudicotyledons	Plantaginaceae	*Synthyris*	*Synthyris dissecta*	EU232349
Eudicotyledons	Plantaginaceae	*Synthyris*	*Synthyris dissecta*	EU232350
Eudicotyledons	Plantaginaceae	*Synthyris*	*Synthyris dissecta*	EU232351
Eudicotyledons	Plantaginaceae	*Synthyris*	*Synthyris dissecta*	EU232352
Eudicotyledons	Plantaginaceae	*Synthyris*	*Synthyris dissecta*	EU232353
Eudicotyledons	Plantaginaceae	*Synthyris*	*Synthyris dissecta*	EU232354
Eudicotyledons	Plantaginaceae	*Synthyris*	*Synthyris dissecta*	EU232355
Eudicotyledons	Plantaginaceae	*Synthyris*	*Synthyris dissecta*	EU232356
Eudicotyledons	Plantaginaceae	*Synthyris*	*Synthyris dissecta*	EU232357
Eudicotyledons	Plantaginaceae	*Synthyris*	*Synthyris dissecta*	EU232358
Eudicotyledons	Plantaginaceae	*Synthyris*	*Synthyris dissecta*	EU232359
Eudicotyledons	Plantaginaceae	*Synthyris*	*Synthyris dissecta*	EU232360
Eudicotyledons	Plantaginaceae	*Synthyris*	*Synthyris dissecta*	EU232361
Eudicotyledons	Plantaginaceae	*Synthyris*	*Synthyris dissecta*	EU232362
Eudicotyledons	Plantaginaceae	*Synthyris*	*Synthyris dissecta*	EU232363
Eudicotyledons	Plantaginaceae	*Synthyris*	*Synthyris dissecta*	EU232364
Eudicotyledons	Plantaginaceae	*Synthyris*	*Synthyris dissecta*	EU232365
Eudicotyledons	Plantaginaceae	*Synthyris*	*Synthyris dissecta*	EU232366
Eudicotyledons	Plantaginaceae	*Synthyris*	*Synthyris dissecta*	EU232367
Eudicotyledons	Plantaginaceae	*Synthyris*	*Synthyris dissecta*	EU232368
Eudicotyledons	Plantaginaceae	*Synthyris*	*Synthyris dissecta*	EU232369
Eudicotyledons	Plantaginaceae	*Synthyris*	*Synthyris dissecta*	EU232370
Eudicotyledons	Plantaginaceae	*Synthyris*	*Synthyris dissecta*	EU232371
Eudicotyledons	Plantaginaceae	*Synthyris*	*Synthyris dissecta*	EU232372
Eudicotyledons	Plantaginaceae	*Synthyris*	*Synthyris dissecta*	EU232373
Eudicotyledons	Plantaginaceae	*Synthyris*	*Synthyris dissecta*	EU232374
Eudicotyledons	Plantaginaceae	*Synthyris*	*Synthyris dissecta*	EU232375
Eudicotyledons	Plantaginaceae	*Synthyris*	*Synthyris dissecta*	EU232376
Eudicotyledons	Plantaginaceae	*Synthyris*	*Synthyris dissecta*	EU232377
Eudicotyledons	Plantaginaceae	*Synthyris*	*Synthyris dissecta*	EU232378

Eudicotyledons	Plantaginaceae	*Synthyris*	*Synthyris dissecta*	EU232379
Eudicotyledons	Plantaginaceae	*Synthyris*	*Synthyris dissecta*	EU232380
Eudicotyledons	Plantaginaceae	*Synthyris*	*Synthyris dissecta*	EU232381
Eudicotyledons	Plantaginaceae	*Synthyris*	*Synthyris dissecta*	EU232382
Eudicotyledons	Plantaginaceae	*Synthyris*	*Synthyris dissecta*	EU232383
Eudicotyledons	Plantaginaceae	*Synthyris*	*Synthyris dissecta*	EU232384
Eudicotyledons	Plantaginaceae	*Synthyris*	*Synthyris dissecta*	EU232385
Eudicotyledons	Plantaginaceae	*Synthyris*	*Synthyris dissecta*	EU232386
Eudicotyledons	Plantaginaceae	*Synthyris*	*Synthyris dissecta*	EU232387
Eudicotyledons	Plantaginaceae	*Synthyris*	*Synthyris dissecta*	EU232388
Eudicotyledons	Plantaginaceae	*Synthyris*	*Synthyris dissecta*	EU232389
Eudicotyledons	Plantaginaceae	*Synthyris*	*Synthyris dissecta*	EU232390
Eudicotyledons	Plantaginaceae	*Synthyris*	*Synthyris dissecta*	EU232391
Eudicotyledons	Plantaginaceae	*Synthyris*	*Synthyris dissecta*	EU232392
Eudicotyledons	Plantaginaceae	*Synthyris*	*Synthyris dissecta*	EU232393
Eudicotyledons	Plantaginaceae	*Synthyris*	*Synthyris dissecta*	EU232394
Eudicotyledons	Plantaginaceae	*Synthyris*	*Synthyris dissecta*	EU232395
Eudicotyledons	Plantaginaceae	*Synthyris*	*Synthyris dissecta*	EU232396
Eudicotyledons	Plantaginaceae	*Synthyris*	*Synthyris dissecta*	EU232397
Eudicotyledons	Plantaginaceae	*Synthyris*	*Synthyris dissecta*	EU232398
Eudicotyledons	Plantaginaceae	*Synthyris*	*Synthyris dissecta*	EU232399
Eudicotyledons	Plantaginaceae	*Synthyris*	*Synthyris dissecta*	EU232400
Eudicotyledons	Plantaginaceae	*Synthyris*	*Synthyris dissecta*	EU232401
Eudicotyledons	Plantaginaceae	*Synthyris*	*Synthyris dissecta*	EU232402
Eudicotyledons	Plantaginaceae	*Synthyris*	*Synthyris dissecta*	EU232403
Eudicotyledons	Plantaginaceae	*Synthyris*	*Synthyris dissecta*	EU232404
Eudicotyledons	Plantaginaceae	*Synthyris*	*Synthyris dissecta*	EU232405
Eudicotyledons	Plantaginaceae	*Synthyris*	*Synthyris dissecta*	EU232406
Eudicotyledons	Plantaginaceae	*Synthyris*	*Synthyris dissecta*	EU232422
Eudicotyledons	Plantaginaceae	*Synthyris*	*Synthyris dissecta*	EU232423
Eudicotyledons	Plantaginaceae	*Synthyris*	*Synthyris dissecta*	EU232424
Eudicotyledons	Plantaginaceae	*Synthyris*	*Synthyris dissecta*	EU232425
Eudicotyledons	Plantaginaceae	*Synthyris*	*Synthyris dissecta*	EU232426
Eudicotyledons	Plantaginaceae	*Synthyris*	*Synthyris dissecta*	EU232427
Eudicotyledons	Plantaginaceae	*Synthyris*	*Synthyris dissecta*	EU232428
Eudicotyledons	Plantaginaceae	*Synthyris*	*Synthyris dissecta*	EU232429
Eudicotyledons	Plantaginaceae	*Synthyris*	*Synthyris dissecta*	EU232430
Eudicotyledons	Plantaginaceae	*Synthyris*	*Synthyris dissecta*	EU232431
Eudicotyledons	Plantaginaceae	*Synthyris*	*Synthyris dissecta*	EU232432
Eudicotyledons	Plantaginaceae	*Synthyris*	*Synthyris dissecta*	EU232433
Eudicotyledons	Plantaginaceae	*Synthyris*	*Synthyris dissecta*	EU232434
Eudicotyledons	Plantaginaceae	*Synthyris*	*Synthyris dissecta*	EU232435
Eudicotyledons	Plantaginaceae	*Synthyris*	*Synthyris dissecta*	EU232436
Eudicotyledons	Plantaginaceae	*Synthyris*	*Synthyris dissecta*	EU232438
Eudicotyledons	Plantaginaceae	*Synthyris*	*Synthyris dissecta*	EU232439
Eudicotyledons	Plantaginaceae	*Synthyris*	*Synthyris dissecta*	EU232440
Eudicotyledons	Plantaginaceae	*Synthyris*	*Synthyris dissecta*	EU232441
Eudicotyledons	Plantaginaceae	*Synthyris*	*Synthyris dissecta*	EU232442
Eudicotyledons	Plantaginaceae	*Synthyris*	*Synthyris dissecta*	EU232443
Eudicotyledons	Plantaginaceae	*Synthyris*	*Synthyris dissecta*	EU232444
Eudicotyledons	Plantaginaceae	*Synthyris*	*Synthyris dissecta*	EU232445
Eudicotyledons	Plantaginaceae	*Synthyris*	*Synthyris dissecta*	EU232446
Eudicotyledons	Plantaginaceae	*Synthyris*	*Synthyris dissecta*	EU232447

Eudicotyledons	Plantaginaceae	*Synthyris*	*Synthyris dissecta*	EU232448
Eudicotyledons	Plantaginaceae	*Synthyris*	*Synthyris dissecta*	EU232449
Eudicotyledons	Plantaginaceae	*Synthyris*	*Synthyris dissecta*	EU232450
Eudicotyledons	Plantaginaceae	*Synthyris*	*Synthyris dissecta*	EU232451
Eudicotyledons	Plantaginaceae	*Synthyris*	*Synthyris dissecta*	EU232452
Eudicotyledons	Plantaginaceae	*Synthyris*	*Synthyris dissecta*	EU232453
Eudicotyledons	Plantaginaceae	*Synthyris*	*Synthyris dissecta*	EU232454
Eudicotyledons	Plantaginaceae	*Synthyris*	*Synthyris dissecta*	EU232455
Eudicotyledons	Plantaginaceae	*Synthyris*	*Synthyris dissecta*	EU232456
Eudicotyledons	Plantaginaceae	*Synthyris*	*Synthyris dissecta*	EU232457
Eudicotyledons	Plantaginaceae	*Synthyris*	*Synthyris dissecta*	EU232458
Eudicotyledons	Plantaginaceae	*Synthyris*	*Synthyris dissecta*	EU232459
Eudicotyledons	Plantaginaceae	*Synthyris*	*Synthyris dissecta*	EU232460
Eudicotyledons	Plantaginaceae	*Synthyris*	*Synthyris dissecta*	EU232461
Eudicotyledons	Plantaginaceae	*Synthyris*	*Synthyris dissecta*	EU232462
Eudicotyledons	Plantaginaceae	*Synthyris*	*Synthyris dissecta*	EU232463
Eudicotyledons	Plantaginaceae	*Synthyris*	*Synthyris dissecta*	EU232464
Eudicotyledons	Plantaginaceae	*Synthyris*	*Synthyris dissecta*	EU232465
Eudicotyledons	Plantaginaceae	*Synthyris*	*Synthyris dissecta*	EU232466
Eudicotyledons	Plantaginaceae	*Synthyris*	*Synthyris dissecta*	EF550011
Eudicotyledons	Plantaginaceae	*Synthyris*	*Synthyris dissecta*	EF550012
Eudicotyledons	Plantaginaceae	*Synthyris*	*Synthyris dissecta*	EF550013
Eudicotyledons	Plantaginaceae	*Synthyris*	*Synthyris dissecta*	EF550014
Eudicotyledons	Plantaginaceae	*Synthyris*	*Synthyris dissecta*	EF550015
Eudicotyledons	Plantaginaceae	*Synthyris*	*Synthyris dissecta*	EF550016
Eudicotyledons	Plantaginaceae	*Synthyris*	*Synthyris dissecta*	EF550017
Eudicotyledons	Plantaginaceae	*Synthyris*	*Synthyris dissecta*	EF550018
Eudicotyledons	Plantaginaceae	*Synthyris*	*Synthyris dissecta*	EF550020
Eudicotyledons	Plantaginaceae	*Synthyris*	*Synthyris dissecta*	EF550022
Eudicotyledons	Plantaginaceae	*Synthyris*	*Synthyris dissecta*	EF550023
Eudicotyledons	Plantaginaceae	*Synthyris*	*Synthyris dissecta*	EF550024
Eudicotyledons	Plantaginaceae	*Synthyris*	*Synthyris dissecta*	EF550025
Eudicotyledons	Plantaginaceae	*Synthyris*	*Synthyris dissecta*	EF550026
Eudicotyledons	Plantaginaceae	*Synthyris*	*Synthyris dissecta*	EF550027
Eudicotyledons	Plantaginaceae	*Synthyris*	*Synthyris dissecta*	EF550028
Eudicotyledons	Plantaginaceae	*Synthyris*	*Synthyris dissecta*	EF550029
Eudicotyledons	Plantaginaceae	*Synthyris*	*Synthyris dissecta*	EF550030
Eudicotyledons	Plantaginaceae	*Synthyris*	*Synthyris laciniata*	AY721951
Eudicotyledons	Plantaginaceae	*Synthyris*	*Synthyris laciniata*	AY721952
Eudicotyledons	Plantaginaceae	*Synthyris*	*Synthyris laciniata*	AY721953
Eudicotyledons	Plantaginaceae	*Synthyris*	*Synthyris laciniata*	AY721954
Eudicotyledons	Plantaginaceae	*Synthyris*	*Synthyris laciniata*	AY721955
Eudicotyledons	Plantaginaceae	*Synthyris*	*Synthyris laciniata*	AY721956
Eudicotyledons	Plantaginaceae	*Synthyris*	*Synthyris laciniata*	AY721957
Eudicotyledons	Plantaginaceae	*Synthyris*	*Synthyris laciniata*	AY721958
Eudicotyledons	Plantaginaceae	*Synthyris*	*Synthyris laciniata*	AY721959
Eudicotyledons	Plantaginaceae	*Synthyris*	*Synthyris laciniata*	AY721960
Eudicotyledons	Plantaginaceae	*Synthyris*	*Synthyris ranunculina*	AY721971
Eudicotyledons	Plantaginaceae	*Synthyris*	*Synthyris ranunculina*	AY721972
Eudicotyledons	Plantaginaceae	*Synthyris*	*Synthyris ranunculina*	AY721973
Eudicotyledons	Plantaginaceae	*Synthyris*	*Synthyris ranunculina*	AY721974
Eudicotyledons	Plantaginaceae	*Synthyris*	*Synthyris ranunculina*	AY721975
Eudicotyledons	Plantaginaceae	*Synthyris*	*Synthyris ranunculina*	AY721976

Eudicotyledons	Plantaginaceae	*Synthyris*	*Synthyris ranunculina*	AY721977
Eudicotyledons	Plantaginaceae	*Synthyris*	*Synthyris ranunculina*	AY721978
Eudicotyledons	Plantaginaceae	*Synthyris*	*Synthyris ranunculina*	AY721979
Eudicotyledons	Plantaginaceae	*Synthyris*	*Synthyris ranunculina*	AY721980
Eudicotyledons	Plantaginaceae	*Synthyris*	*Synthyris lanuginosa*	EU232304
Eudicotyledons	Plantaginaceae	*Synthyris*	*Synthyris lanuginosa*	EU232305
Eudicotyledons	Plantaginaceae	*Synthyris*	*Synthyris lanuginosa*	EU232306
Eudicotyledons	Plantaginaceae	*Synthyris*	*Synthyris lanuginosa*	EU232307
Eudicotyledons	Plantaginaceae	*Synthyris*	*Synthyris lanuginosa*	EU232308
Eudicotyledons	Plantaginaceae	*Synthyris*	*Synthyris lanuginosa*	EU232309
Eudicotyledons	Plantaginaceae	*Synthyris*	*Synthyris lanuginosa*	EU232310
Eudicotyledons	Plantaginaceae	*Synthyris*	*Synthyris lanuginosa*	EU232311
Eudicotyledons	Plantaginaceae	*Synthyris*	*Synthyris lanuginosa*	EU232312
Eudicotyledons	Plantaginaceae	*Synthyris*	*Synthyris lanuginosa*	EU232313
Eudicotyledons	Plantaginaceae	*Synthyris*	*Synthyris lanuginosa*	EU232314
Eudicotyledons	Plantaginaceae	*Synthyris*	*Synthyris lanuginosa*	EU232315
Eudicotyledons	Plantaginaceae	*Synthyris*	*Synthyris lanuginosa*	EU232316
Eudicotyledons	Plantaginaceae	*Synthyris*	*Synthyris lanuginosa*	EU232317
Eudicotyledons	Plantaginaceae	*Synthyris*	*Synthyris lanuginosa*	EU232318
Eudicotyledons	Plantaginaceae	*Synthyris*	*Synthyris lanuginosa*	EU232319
Eudicotyledons	Plantaginaceae	*Synthyris*	*Synthyris lanuginosa*	EU232320
Eudicotyledons	Plantaginaceae	*Synthyris*	*Synthyris lanuginosa*	EU232321
Eudicotyledons	Plantaginaceae	*Synthyris*	*Synthyris lanuginosa*	EU232322
Eudicotyledons	Plantaginaceae	*Synthyris*	*Synthyris lanuginosa*	EU232323
Eudicotyledons	Plantaginaceae	*Synthyris*	*Synthyris lanuginosa*	EU232324
Eudicotyledons	Plantaginaceae	*Synthyris*	*Synthyris lanuginosa*	EU232325
Eudicotyledons	Plantaginaceae	*Synthyris*	*Synthyris lanuginosa*	EU232326
Eudicotyledons	Plantaginaceae	*Synthyris*	*Synthyris lanuginosa*	EU232327
Eudicotyledons	Plantaginaceae	*Synthyris*	*Synthyris lanuginosa*	EU232328
Eudicotyledons	Plantaginaceae	*Synthyris*	*Synthyris lanuginosa*	EU232329
Eudicotyledons	Plantaginaceae	*Synthyris*	*Synthyris lanuginosa*	EU232330
Eudicotyledons	Plantaginaceae	*Synthyris*	*Synthyris lanuginosa*	EU232331
Eudicotyledons	Plantaginaceae	*Synthyris*	*Synthyris lanuginosa*	EU232332
Eudicotyledons	Plantaginaceae	*Synthyris*	*Synthyris lanuginosa*	EU232333
Eudicotyledons	Plantaginaceae	*Synthyris*	*Synthyris lanuginosa*	EU232334
Eudicotyledons	Plantaginaceae	*Synthyris*	*Synthyris lanuginosa*	EU232335
Eudicotyledons	Plantaginaceae	*Synthyris*	*Synthyris lanuginosa*	EU232336
Eudicotyledons	Plantaginaceae	*Synthyris*	*Synthyris lanuginosa*	EU232337
Eudicotyledons	Plantaginaceae	*Synthyris*	*Synthyris lanuginosa*	EU232338
Eudicotyledons	Plantaginaceae	*Synthyris*	*Synthyris lanuginosa*	EU232339
Eudicotyledons	Plantaginaceae	*Synthyris*	*Synthyris lanuginosa*	EU232340
Eudicotyledons	Plantaginaceae	*Synthyris*	*Synthyris lanuginosa*	EU232341
Eudicotyledons	Plantaginaceae	*Synthyris*	*Synthyris lanuginosa*	EU232342
Eudicotyledons	Plantaginaceae	*Synthyris*	*Synthyris lanuginosa*	EU232343
Eudicotyledons	Plantaginaceae	*Synthyris*	*Synthyris lanuginosa*	EU232344
Eudicotyledons	Plantaginaceae	*Synthyris*	*Synthyris lanuginosa*	EU232345
Eudicotyledons	Plantaginaceae	*Synthyris*	*Synthyris lanuginosa*	EU232346
Eudicotyledons	Plantaginaceae	*Synthyris*	*Synthyris lanuginosa*	EU232347
Eudicotyledons	Plantaginaceae	*Synthyris*	*Synthyris lanuginosa*	EU232437
Eudicotyledons	Plantaginaceae	*Synthyris*	*Synthyris lanuginosa*	EF550009
Eudicotyledons	Plantaginaceae	*Synthyris*	*Synthyris lanuginosa*	EF550010
Eudicotyledons	Plantaginaceae	*Synthyris*	*Synthyris lanuginosa*	EF550021
Eudicotyledons	Ericaceae	*Erica*	*Erica arborea*	GU323801

Eudicotyledons	Ericaceae	*Erica*	*Erica arborea*	GU323802
Eudicotyledons	Ericaceae	*Erica*	*Erica arborea*	GU323803
Eudicotyledons	Ericaceae	*Erica*	*Erica arborea*	GU323804
Eudicotyledons	Ericaceae	*Erica*	*Erica arborea*	GU323805
Eudicotyledons	Ericaceae	*Erica*	*Erica arborea*	GU323806
Eudicotyledons	Ericaceae	*Erica*	*Erica arborea*	GU323807
Eudicotyledons	Ericaceae	*Erica*	*Erica arborea*	GU323808
Eudicotyledons	Ericaceae	*Erica*	*Erica arborea*	GU323809
Eudicotyledons	Ericaceae	*Erica*	*Erica arborea*	GU323810
Eudicotyledons	Ericaceae	*Erica*	*Erica arborea*	GU323811
Eudicotyledons	Ericaceae	*Erica*	*Erica arborea*	GU323812
Eudicotyledons	Ericaceae	*Erica*	*Erica arborea*	GU323813
Eudicotyledons	Ericaceae	*Erica*	*Erica arborea*	GU323814
Eudicotyledons	Ericaceae	*Erica*	*Erica arborea*	GU323815
Eudicotyledons	Ericaceae	*Erica*	*Erica arborea*	GU323816
Eudicotyledons	Ericaceae	*Erica*	*Erica arborea*	GU323817
Eudicotyledons	Ericaceae	*Erica*	*Erica arborea*	GU323818
Eudicotyledons	Ericaceae	*Erica*	*Erica arborea*	GU323819
Eudicotyledons	Ericaceae	*Erica*	*Erica arborea*	GU323820
Eudicotyledons	Ericaceae	*Erica*	*Erica arborea*	GU323821
Eudicotyledons	Ericaceae	*Erica*	*Erica arborea*	GU323822
Eudicotyledons	Ericaceae	*Erica*	*Erica arborea*	GU323823
Eudicotyledons	Ericaceae	*Erica*	*Erica arborea*	GU323824
Eudicotyledons	Ericaceae	*Erica*	*Erica arborea*	GU323825
Eudicotyledons	Ericaceae	*Erica*	*Erica arborea*	GU323826
Eudicotyledons	Ericaceae	*Erica*	*Erica arborea*	GU323827
Eudicotyledons	Ericaceae	*Erica*	*Erica arborea*	GU323828
Eudicotyledons	Ericaceae	*Erica*	*Erica arborea*	GU323829
Eudicotyledons	Ericaceae	*Erica*	*Erica arborea*	GU323830
Eudicotyledons	Ericaceae	*Erica*	*Erica arborea*	GU323831
Eudicotyledons	Ericaceae	*Erica*	*Erica arborea*	GU323832
Eudicotyledons	Ericaceae	*Erica*	*Erica arborea*	GU323833
Eudicotyledons	Ericaceae	*Erica*	*Erica arborea*	GU323834
Eudicotyledons	Ericaceae	*Erica*	*Erica arborea*	GU323835
Eudicotyledons	Ericaceae	*Erica*	*Erica arborea*	GU323836
Eudicotyledons	Ericaceae	*Erica*	*Erica arborea*	GU323837
Eudicotyledons	Ericaceae	*Erica*	*Erica arborea*	GU323838
Eudicotyledons	Ericaceae	*Erica*	*Erica arborea*	GU323839
Eudicotyledons	Ericaceae	*Erica*	*Erica arborea*	GU323840
Eudicotyledons	Ericaceae	*Erica*	*Erica arborea*	GU323841
Eudicotyledons	Ericaceae	*Erica*	*Erica arborea*	GU323842
Eudicotyledons	Ericaceae	*Erica*	*Erica arborea*	GU323843
Eudicotyledons	Ericaceae	*Erica*	*Erica arborea*	GU323844
Eudicotyledons	Ericaceae	*Erica*	*Erica arborea*	GU323845
Eudicotyledons	Ericaceae	*Erica*	*Erica arborea*	GU323846
Eudicotyledons	Ericaceae	*Erica*	*Erica arborea*	GU323847
Eudicotyledons	Ericaceae	*Erica*	*Erica arborea*	GU323848
Eudicotyledons	Ericaceae	*Erica*	*Erica arborea*	GU323849
Eudicotyledons	Ericaceae	*Erica*	*Erica arborea*	GU323850
Eudicotyledons	Ericaceae	*Erica*	*Erica arborea*	GU323851
Eudicotyledons	Ericaceae	*Erica*	*Erica arborea*	GU323852
Eudicotyledons	Ericaceae	*Erica*	*Erica arborea*	GU323853
Eudicotyledons	Ericaceae	*Erica*	*Erica arborea*	GU323854

Eudicotyledons	Ericaceae	*Erica*	*Erica arborea*	GU323855
Eudicotyledons	Ericaceae	*Erica*	*Erica arborea*	GU323856
Eudicotyledons	Ericaceae	*Erica*	*Erica arborea*	GU323857
Eudicotyledons	Ericaceae	*Erica*	*Erica arborea*	GU323858
Eudicotyledons	Ericaceae	*Erica*	*Erica arborea*	GU323859
Eudicotyledons	Ericaceae	*Erica*	*Erica arborea*	GU323860
Eudicotyledons	Ericaceae	*Erica*	*Erica arborea*	GU323861
Eudicotyledons	Ericaceae	*Erica*	*Erica arborea*	GU323862
Eudicotyledons	Ericaceae	*Erica*	*Erica arborea*	GU323863
Eudicotyledons	Ericaceae	*Erica*	*Erica arborea*	GU323864
Eudicotyledons	Ericaceae	*Erica*	*Erica arborea*	GU323865
Eudicotyledons	Ericaceae	*Erica*	*Erica arborea*	GU323866
Eudicotyledons	Ericaceae	*Erica*	*Erica arborea*	GU323867
Eudicotyledons	Ericaceae	*Erica*	*Erica arborea*	GU323868
Eudicotyledons	Ericaceae	*Erica*	*Erica arborea*	GU323869
Eudicotyledons	Ericaceae	*Erica*	*Erica arborea*	GU323870
Eudicotyledons	Ericaceae	*Erica*	*Erica arborea*	GU323871
Eudicotyledons	Ericaceae	*Erica*	*Erica arborea*	GU323872
Eudicotyledons	Ericaceae	*Erica*	*Erica arborea*	GU323873
Eudicotyledons	Ericaceae	*Erica*	*Erica arborea*	GU323874
Eudicotyledons	Ericaceae	*Erica*	*Erica arborea*	GU323875
Eudicotyledons	Ericaceae	*Erica*	*Erica arborea*	GU323876
Eudicotyledons	Ericaceae	*Erica*	*Erica arborea*	GU323877
Eudicotyledons	Ericaceae	*Erica*	*Erica arborea*	GU323878
Eudicotyledons	Ericaceae	*Erica*	*Erica arborea*	GU323879
Eudicotyledons	Ericaceae	*Erica*	*Erica arborea*	GU323880
Eudicotyledons	Ericaceae	*Erica*	*Erica arborea*	GU323881
Eudicotyledons	Ericaceae	*Erica*	*Erica arborea*	GU323882
Eudicotyledons	Ericaceae	*Erica*	*Erica arborea*	GU323883
Eudicotyledons	Ericaceae	*Erica*	*Erica arborea*	GU323884
Eudicotyledons	Ericaceae	*Erica*	*Erica arborea*	GU323885
Eudicotyledons	Ericaceae	*Erica*	*Erica arborea*	GU323886
Eudicotyledons	Ericaceae	*Erica*	*Erica arborea*	GU323887
Eudicotyledons	Ericaceae	*Erica*	*Erica arborea*	GU323888
Eudicotyledons	Ericaceae	*Erica*	*Erica arborea*	GU323889
Eudicotyledons	Ericaceae	*Erica*	*Erica arborea*	GU323890
Eudicotyledons	Ericaceae	*Erica*	*Erica arborea*	GU323891
Eudicotyledons	Ericaceae	*Erica*	*Erica arborea*	GU323892
Eudicotyledons	Ericaceae	*Erica*	*Erica arborea*	GU323893
Eudicotyledons	Ericaceae	*Erica*	*Erica arborea*	GU323894
Eudicotyledons	Ericaceae	*Erica*	*Erica arborea*	GU323895
Eudicotyledons	Ericaceae	*Erica*	*Erica arborea*	GU323896
Eudicotyledons	Ericaceae	*Erica*	*Erica arborea*	GU323897
Eudicotyledons	Ericaceae	*Erica*	*Erica arborea*	GU323898
Eudicotyledons	Ericaceae	*Erica*	*Erica arborea*	GU323899
Eudicotyledons	Ericaceae	*Erica*	*Erica arborea*	GU323900
Eudicotyledons	Ericaceae	*Erica*	*Erica arborea*	GU323901
Eudicotyledons	Ericaceae	*Erica*	*Erica arborea*	GU323902
Eudicotyledons	Ericaceae	*Erica*	*Erica arborea*	GU323903
Eudicotyledons	Ericaceae	*Erica*	*Erica arborea*	GU323904
Eudicotyledons	Ericaceae	*Erica*	*Erica arborea*	GU323905
Eudicotyledons	Ericaceae	*Erica*	*Erica scoparia*	JN605459
Eudicotyledons	Ericaceae	*Erica*	*Erica scoparia*	JN605460

Eudicotyledons	Ericaceae	*Erica*	*Erica scoparia*	JN605461
Eudicotyledons	Ericaceae	*Erica*	*Erica scoparia*	JN605462
Eudicotyledons	Ericaceae	*Erica*	*Erica scoparia*	JN605463
Eudicotyledons	Ericaceae	*Erica*	*Erica scoparia*	JN605464
Eudicotyledons	Ericaceae	*Erica*	*Erica scoparia*	JN605465
Eudicotyledons	Ericaceae	*Erica*	*Erica scoparia*	JN605466
Eudicotyledons	Ericaceae	*Erica*	*Erica scoparia*	JN605467
Eudicotyledons	Ericaceae	*Erica*	*Erica scoparia*	JN605468
Eudicotyledons	Ericaceae	*Erica*	*Erica scoparia*	JN605469
Eudicotyledons	Ericaceae	*Erica*	*Erica scoparia*	JN605470
Eudicotyledons	Ericaceae	*Erica*	*Erica scoparia*	JN605471
Eudicotyledons	Ericaceae	*Erica*	*Erica scoparia*	JN605472
Eudicotyledons	Ericaceae	*Erica*	*Erica scoparia*	JN605473
Eudicotyledons	Ericaceae	*Erica*	*Erica scoparia*	JN605474
Eudicotyledons	Ericaceae	*Erica*	*Erica scoparia*	JN605475
Eudicotyledons	Ericaceae	*Erica*	*Erica scoparia*	JN605476
Eudicotyledons	Ericaceae	*Erica*	*Erica scoparia*	JN605477
Eudicotyledons	Ericaceae	*Erica*	*Erica scoparia*	JN605478
Eudicotyledons	Ericaceae	*Erica*	*Erica scoparia*	JN605479
Eudicotyledons	Ericaceae	*Erica*	*Erica scoparia*	JN605480
Eudicotyledons	Ericaceae	*Erica*	*Erica scoparia*	JN605481
Eudicotyledons	Ericaceae	*Erica*	*Erica scoparia*	JN605482
Eudicotyledons	Ericaceae	*Erica*	*Erica scoparia*	JN605483
Eudicotyledons	Ericaceae	*Erica*	*Erica scoparia*	JN605484
Eudicotyledons	Ericaceae	*Erica*	*Erica scoparia*	JN605485
Eudicotyledons	Ericaceae	*Erica*	*Erica scoparia*	JN605486
Eudicotyledons	Ericaceae	*Erica*	*Erica scoparia*	JN605487
Eudicotyledons	Ericaceae	*Erica*	*Erica scoparia*	JN605488
Eudicotyledons	Ericaceae	*Erica*	*Erica scoparia*	JN605489
Eudicotyledons	Ericaceae	*Erica*	*Erica scoparia*	JN605490
Eudicotyledons	Ericaceae	*Erica*	*Erica scoparia*	JN605491
Eudicotyledons	Ericaceae	*Erica*	*Erica scoparia*	JN605492
Eudicotyledons	Ericaceae	*Erica*	*Erica scoparia*	JN605493
Eudicotyledons	Ericaceae	*Erica*	*Erica scoparia*	JN605494
Eudicotyledons	Ericaceae	*Erica*	*Erica scoparia*	JN605495
Eudicotyledons	Ericaceae	*Erica*	*Erica scoparia*	JN605496
Eudicotyledons	Ericaceae	*Erica*	*Erica scoparia*	JN605497
Eudicotyledons	Ericaceae	*Erica*	*Erica scoparia*	JN605498
Eudicotyledons	Ericaceae	*Erica*	*Erica scoparia*	JN605499
Eudicotyledons	Ericaceae	*Erica*	*Erica scoparia*	JN605500
Eudicotyledons	Ericaceae	*Erica*	*Erica scoparia*	JN605501
Eudicotyledons	Ericaceae	*Erica*	*Erica scoparia*	JN605502
Eudicotyledons	Ericaceae	*Erica*	*Erica scoparia*	JN605503
Eudicotyledons	Ericaceae	*Erica*	*Erica scoparia*	JN605504
Eudicotyledons	Ericaceae	*Erica*	*Erica scoparia*	JN605505
Eudicotyledons	Ericaceae	*Erica*	*Erica scoparia*	JN605506
Eudicotyledons	Ericaceae	*Erica*	*Erica scoparia*	JN605507
Eudicotyledons	Ericaceae	*Erica*	*Erica scoparia*	JN605508
Eudicotyledons	Ericaceae	*Erica*	*Erica scoparia*	JN605509
Eudicotyledons	Ericaceae	*Erica*	*Erica scoparia*	JN605510
Eudicotyledons	Ericaceae	*Erica*	*Erica scoparia*	JN605511
Eudicotyledons	Ericaceae	*Erica*	*Erica scoparia*	JN605512
Eudicotyledons	Ericaceae	*Erica*	*Erica scoparia*	JN605513

Eudicotyledons	Ericaceae	*Erica*	*Erica scoparia*	JN605514
Eudicotyledons	Ericaceae	*Erica*	*Erica scoparia*	JN605515
Eudicotyledons	Ericaceae	*Erica*	*Erica scoparia*	JN605516
Eudicotyledons	Ericaceae	*Erica*	*Erica scoparia*	JN605517
Eudicotyledons	Ericaceae	*Erica*	*Erica scoparia*	JN605518
Eudicotyledons	Ericaceae	*Erica*	*Erica scoparia*	JN605519
Eudicotyledons	Ericaceae	*Erica*	*Erica scoparia*	JN605520
Eudicotyledons	Ericaceae	*Erica*	*Erica scoparia*	JN605521
Eudicotyledons	Ericaceae	*Erica*	*Erica scoparia*	JN605522
Eudicotyledons	Ericaceae	*Erica*	*Erica scoparia*	JN605523
Eudicotyledons	Ericaceae	*Erica*	*Erica scoparia*	JN605524
Eudicotyledons	Ericaceae	*Erica*	*Erica scoparia*	JN605525
Eudicotyledons	Ericaceae	*Erica*	*Erica scoparia*	JN605526
Eudicotyledons	Ericaceae	*Erica*	*Erica scoparia*	JN605527
Eudicotyledons	Ericaceae	*Erica*	*Erica scoparia*	JN605528
Eudicotyledons	Ericaceae	*Erica*	*Erica scoparia*	JN605529
Eudicotyledons	Ericaceae	*Erica*	*Erica scoparia*	JN605530
Eudicotyledons	Ericaceae	*Erica*	*Erica scoparia*	JN605531
Eudicotyledons	Ericaceae	*Erica*	*Erica scoparia*	JN605532
Eudicotyledons	Ericaceae	*Erica*	*Erica scoparia*	JN605533
Eudicotyledons	Ericaceae	*Erica*	*Erica scoparia*	JN605534
Eudicotyledons	Ericaceae	*Erica*	*Erica scoparia*	JN605535
Eudicotyledons	Ericaceae	*Erica*	*Erica scoparia*	JN605536
Eudicotyledons	Ericaceae	*Erica*	*Erica scoparia*	JN605537
Eudicotyledons	Ericaceae	*Erica*	*Erica scoparia*	JN605538
Eudicotyledons	Ericaceae	*Erica*	*Erica scoparia*	JN605539
Eudicotyledons	Ericaceae	*Erica*	*Erica scoparia*	JN605540
Eudicotyledons	Ericaceae	*Erica*	*Erica scoparia*	JN605541
Eudicotyledons	Ericaceae	*Erica*	*Erica scoparia*	JN605542
Eudicotyledons	Ericaceae	*Erica*	*Erica scoparia*	JN605543
Eudicotyledons	Ericaceae	*Erica*	*Erica scoparia*	JN605544
Eudicotyledons	Ericaceae	*Erica*	*Erica scoparia*	JN605545
Eudicotyledons	Ericaceae	*Erica*	*Erica scoparia*	JN605546
Eudicotyledons	Ericaceae	*Erica*	*Erica scoparia*	JN605547
Eudicotyledons	Ericaceae	*Erica*	*Erica scoparia*	JN605548
Eudicotyledons	Ericaceae	*Erica*	*Erica scoparia*	JN605549
Eudicotyledons	Ericaceae	*Erica*	*Erica scoparia*	JN605550
Eudicotyledons	Ericaceae	*Erica*	*Erica scoparia*	JN605551
Eudicotyledons	Ericaceae	*Erica*	*Erica scoparia*	JN605552
Eudicotyledons	Ericaceae	*Erica*	*Erica scoparia*	JN605553
Eudicotyledons	Ericaceae	*Erica*	*Erica scoparia*	JN605554
Eudicotyledons	Ericaceae	*Erica*	*Erica scoparia*	JN605555
Eudicotyledons	Ericaceae	*Erica*	*Erica scoparia*	JN605556
Eudicotyledons	Ericaceae	*Erica*	*Erica scoparia*	JN605557
Eudicotyledons	Ericaceae	*Erica*	*Erica scoparia*	JN605558
Eudicotyledons	Ericaceae	*Erica*	*Erica scoparia*	JN605559
Eudicotyledons	Ericaceae	*Erica*	*Erica scoparia*	JN605560
Eudicotyledons	Ericaceae	*Erica*	*Erica scoparia*	JN605561
Eudicotyledons	Ericaceae	*Erica*	*Erica scoparia*	JN605562
Eudicotyledons	Cactaceae	*Mammillaria*	*Mammillaria crinita*	AY545367
Eudicotyledons	Cactaceae	*Mammillaria*	*Mammillaria crinita*	AY545368
Eudicotyledons	Cactaceae	*Mammillaria*	*Mammillaria mercadensis*	AY545409
Eudicotyledons	Cactaceae	*Mammillaria*	*Mammillaria mercadensis*	AY545410

Eudicotyledons	Rosaceae	*Cliffortia*	*Cliffortia odorata*	EU937596
Eudicotyledons	Rosaceae	*Cliffortia*	*Cliffortia odorata*	EU937597
Eudicotyledons	Asteraceae	*Achillea*	*Achillea asiatica*	HQ451008
Eudicotyledons	Asteraceae	*Achillea*	*Achillea asiatica*	HQ451009
Eudicotyledons	Asteraceae	*Achillea*	*Achillea asiatica*	HQ451010
Eudicotyledons	Asteraceae	*Achillea*	*Achillea asiatica*	HQ451011
Eudicotyledons	Asteraceae	*Achillea*	*Achillea asiatica*	HQ451012
Eudicotyledons	Asteraceae	*Achillea*	*Achillea asiatica*	HQ451013
Eudicotyledons	Asteraceae	*Achillea*	*Achillea asiatica*	HQ451014
Eudicotyledons	Asteraceae	*Achillea*	*Achillea asiatica*	HQ451015
Eudicotyledons	Asteraceae	*Achillea*	*Achillea asiatica*	HQ451016
Eudicotyledons	Asteraceae	*Achillea*	*Achillea asiatica*	HQ451017
Eudicotyledons	Asteraceae	*Achillea*	*Achillea asiatica*	HQ451018
Eudicotyledons	Asteraceae	*Achillea*	*Achillea asiatica*	HQ451019
Eudicotyledons	Asteraceae	*Achillea*	*Achillea asiatica*	HQ451020
Eudicotyledons	Asteraceae	*Achillea*	*Achillea asiatica*	HQ451021
Eudicotyledons	Asteraceae	*Achillea*	*Achillea asiatica*	HQ451022
Eudicotyledons	Asteraceae	*Achillea*	*Achillea asiatica*	HQ451023
Eudicotyledons	Asteraceae	*Achillea*	*Achillea asiatica*	HQ451024
Eudicotyledons	Asteraceae	*Achillea*	*Achillea asiatica*	HQ451025
Eudicotyledons	Asteraceae	*Achillea*	*Achillea asiatica*	HQ451026
Eudicotyledons	Asteraceae	*Achillea*	*Achillea asiatica*	HQ451027
Eudicotyledons	Asteraceae	*Achillea*	*Achillea aspleniifolia*	HQ451028
Eudicotyledons	Asteraceae	*Achillea*	*Achillea aspleniifolia*	HQ451029
Eudicotyledons	Asteraceae	*Achillea*	*Achillea aspleniifolia*	HQ451030
Eudicotyledons	Asteraceae	*Achillea*	*Achillea aspleniifolia*	HQ451031
Eudicotyledons	Asteraceae	*Achillea*	*Achillea aspleniifolia*	HQ451032
Eudicotyledons	Asteraceae	*Achillea*	*Achillea aspleniifolia*	HQ451033
Eudicotyledons	Asteraceae	*Achillea*	*Achillea aspleniifolia*	HQ451034
Eudicotyledons	Asteraceae	*Achillea*	*Achillea distans*	HQ451040
Eudicotyledons	Asteraceae	*Achillea*	*Achillea distans*	HQ451041
Eudicotyledons	Asteraceae	*Achillea*	*Achillea roseoalba*	HQ451055
Eudicotyledons	Asteraceae	*Achillea*	*Achillea roseoalba*	HQ451056
Eudicotyledons	Asteraceae	*Achillea*	*Achillea roseoalba*	HQ451057
Eudicotyledons	Asteraceae	*Achillea*	*Achillea roseoalba*	HQ451058
Eudicotyledons	Asteraceae	*Achillea*	*Achillea roseoalba*	HQ451059
Eudicotyledons	Asteraceae	*Achillea*	*Achillea roseoalba*	HQ451060
Eudicotyledons	Asteraceae	*Achillea*	*Achillea roseoalba*	HQ451061
Eudicotyledons	Asteraceae	*Achillea*	*Achillea schmakovii*	HQ451062
Eudicotyledons	Asteraceae	*Achillea*	*Achillea schmakovii*	HQ451063
Eudicotyledons	Asteraceae	*Achillea*	*Achillea setacea*	HQ451064
Eudicotyledons	Asteraceae	*Achillea*	*Achillea setacea*	HQ451065
Eudicotyledons	Asteraceae	*Achillea*	*Achillea setacea*	HQ451066
Eudicotyledons	Asteraceae	*Achillea*	*Achillea setacea*	HQ451067
Eudicotyledons	Asteraceae	*Achillea*	*Achillea setacea*	HQ451068
Eudicotyledons	Asteraceae	*Achillea*	*Achillea setacea*	HQ451069
Eudicotyledons	Asteraceae	*Achillea*	*Achillea setacea*	HQ451070
Eudicotyledons	Asteraceae	*Achillea*	*Achillea wilsoniana*	GU724266
Eudicotyledons	Asteraceae	*Achillea*	*Achillea wilsoniana*	GQ435138
Eudicotyledons	Asteraceae	*Achillea*	*Achillea wilsoniana*	GQ435139
Eudicotyledons	Primulaceae	*Omphalogramma*	*Omphalogramma souliei*	JN045619
Eudicotyledons	Primulaceae	*Omphalogramma*	*Omphalogramma souliei*	JN045620
Eudicotyledons	Primulaceae	*Omphalogramma*	*Omphalogramma souliei*	JN045621

Eudicotyledons	Primulaceae	*Omphalogramma*	*Omphalogramma souliei*	JN045622
Eudicotyledons	Primulaceae	*Omphalogramma*	*Omphalogramma souliei*	JN045623
Eudicotyledons	Polygonaceae	*Oxyria*	*Oxyria digyna*	JQ080918
Eudicotyledons	Polygonaceae	*Oxyria*	*Oxyria digyna*	JQ080919
Eudicotyledons	Polygonaceae	*Oxyria*	*Oxyria digyna*	JQ080920
Eudicotyledons	Polygonaceae	*Oxyria*	*Oxyria digyna*	JQ080921
Eudicotyledons	Polygonaceae	*Oxyria*	*Oxyria digyna*	JQ080922
Eudicotyledons	Polygonaceae	*Oxyria*	*Oxyria digyna*	JQ080923
Eudicotyledons	Polygonaceae	*Oxyria*	*Oxyria digyna*	JQ080924
Eudicotyledons	Polygonaceae	*Oxyria*	*Oxyria digyna*	JQ080925
Eudicotyledons	Polygonaceae	*Oxyria*	*Oxyria digyna*	JQ080926
Eudicotyledons	Polygonaceae	*Oxyria*	*Oxyria digyna*	JQ080927
Eudicotyledons	Polygonaceae	*Oxyria*	*Oxyria digyna*	JQ080928
Eudicotyledons	Polygonaceae	*Oxyria*	*Oxyria digyna*	JQ080929
Eudicotyledons	Polygonaceae	*Oxyria*	*Oxyria digyna*	JQ080930
Eudicotyledons	Polygonaceae	*Oxyria*	*Oxyria digyna*	JQ080931
Eudicotyledons	Polygonaceae	*Oxyria*	*Oxyria digyna*	JQ080932
Eudicotyledons	Polygonaceae	*Oxyria*	*Oxyria digyna*	JQ080933
Eudicotyledons	Polygonaceae	*Oxyria*	*Oxyria digyna*	JQ080934
Eudicotyledons	Polygonaceae	*Oxyria*	*Oxyria digyna*	JQ080935
Eudicotyledons	Polygonaceae	*Oxyria*	*Oxyria digyna*	JQ080936
Eudicotyledons	Polygonaceae	*Oxyria*	*Oxyria digyna*	JQ080937
Eudicotyledons	Polygonaceae	*Oxyria*	*Oxyria digyna*	JQ080938
Eudicotyledons	Polygonaceae	*Oxyria*	*Oxyria digyna*	JQ080939
Eudicotyledons	Polygonaceae	*Oxyria*	*Oxyria digyna*	JQ080940
Eudicotyledons	Polygonaceae	*Oxyria*	*Oxyria digyna*	JQ080941
Eudicotyledons	Polygonaceae	*Oxyria*	*Oxyria digyna*	JQ080942
Eudicotyledons	Polygonaceae	*Oxyria*	*Oxyria digyna*	JQ080943
Eudicotyledons	Polygonaceae	*Oxyria*	*Oxyria digyna*	JQ080944
Eudicotyledons	Polygonaceae	*Oxyria*	*Oxyria digyna*	JQ080945
Eudicotyledons	Polygonaceae	*Oxyria*	*Oxyria digyna*	JQ080946
Eudicotyledons	Polygonaceae	*Oxyria*	*Oxyria digyna*	JQ080947
Eudicotyledons	Polygonaceae	*Oxyria*	*Oxyria digyna*	JQ080948
Eudicotyledons	Polygonaceae	*Oxyria*	*Oxyria digyna*	JQ080949
Eudicotyledons	Polygonaceae	*Oxyria*	*Oxyria digyna*	JQ080950
Eudicotyledons	Polygonaceae	*Oxyria*	*Oxyria digyna*	JQ080951
Eudicotyledons	Polygonaceae	*Oxyria*	*Oxyria digyna*	JQ080952
Eudicotyledons	Polygonaceae	*Oxyria*	*Oxyria digyna*	JQ080953
Eudicotyledons	Polygonaceae	*Oxyria*	*Oxyria digyna*	JQ080954
Eudicotyledons	Polygonaceae	*Oxyria*	*Oxyria digyna*	JQ080955
Eudicotyledons	Polygonaceae	*Oxyria*	*Oxyria digyna*	JQ080956
Eudicotyledons	Polygonaceae	*Oxyria*	*Oxyria digyna*	JQ080957
Eudicotyledons	Polygonaceae	*Oxyria*	*Oxyria digyna*	JQ080958
Eudicotyledons	Polygonaceae	*Oxyria*	*Oxyria digyna*	JQ080959
Eudicotyledons	Polygonaceae	*Oxyria*	*Oxyria digyna*	JQ080960
Eudicotyledons	Polygonaceae	*Oxyria*	*Oxyria digyna*	JQ080961
Eudicotyledons	Polygonaceae	*Oxyria*	*Oxyria digyna*	JQ080962
Eudicotyledons	Polygonaceae	*Oxyria*	*Oxyria digyna*	GQ435162
Eudicotyledons	Polygonaceae	*Oxyria*	*Oxyria digyna*	EU554051
Eudicotyledons	Polygonaceae	*Oxyria*	*Oxyria digyna*	JN045690
Eudicotyledons	Polygonaceae	*Oxyria*	*Oxyria digyna*	JN045691
Eudicotyledons	Polygonaceae	*Oxyria*	*Oxyria digyna*	JN045692
Eudicotyledons	Polygonaceae	*Oxyria*	*Oxyria digyna*	JN045693

Eudicotyledons	Polygonaceae	*Oxyria*	*Oxyria digyna*	JN045694
Eudicotyledons	Polygonaceae	*Oxyria*	*Oxyria digyna*	JN045695
Eudicotyledons	Asteraceae	*Tragopogon*	*Tragopogon coloratus*	EU391970
Eudicotyledons	Asteraceae	*Tragopogon*	*Tragopogon coloratus*	EF374257
Eudicotyledons	Asteraceae	*Tragopogon*	*Tragopogon coloratus*	EF374258
Eudicotyledons	Asteraceae	*Tragopogon*	*Tragopogon dasyrhynchus*	EF374260
Eudicotyledons	Asteraceae	*Tragopogon*	*Tragopogon dasyrhynchus*	EF374261
Eudicotyledons	Asteraceae	*Tragopogon*	*Tragopogon latifolius*	EU391972
Eudicotyledons	Asteraceae	*Tragopogon*	*Tragopogon latifolius*	EF374269
Eudicotyledons	Vitaceae	*Cissus*	*Cissus verticillata*	JQ182495
Eudicotyledons	Vitaceae	*Cissus*	*Cissus verticillata*	HM585674
Eudicotyledons	Vitaceae	*Cissus*	*Cissus verticillata*	JF437101
Eudicotyledons	Violaceae	*Viola*	*Viola hondoensis*	GQ262596
Eudicotyledons	Violaceae	*Viola*	*Viola hondoensis*	GQ262633
Eudicotyledons	Violaceae	*Viola*	*Viola grypoceras*	GQ262603
Eudicotyledons	Violaceae	*Viola*	*Viola grypoceras*	HM483582
Eudicotyledons	Violaceae	*Viola*	*Viola verecunda*	GQ262606
Eudicotyledons	Violaceae	*Viola*	*Viola verecunda*	GQ262607
Eudicotyledons	Violaceae	*Viola*	*Viola rossii*	GQ262609
Eudicotyledons	Violaceae	*Viola*	*Viola rossii*	GQ262610
Eudicotyledons	Violaceae	*Viola*	*Viola philippica*	GQ262625
Eudicotyledons	Violaceae	*Viola*	*Viola philippica*	GQ435054
Eudicotyledons	Violaceae	*Viola*	*Viola violacea*	GQ262631
Eudicotyledons	Violaceae	*Viola*	*Viola violacea*	HM483596
Eudicotyledons	Euphorbiaceae	*Croton*	*Croton gratissimus*	EU213803
Eudicotyledons	Euphorbiaceae	*Croton*	*Croton gratissimus*	EU213804
Eudicotyledons	Euphorbiaceae	*Croton*	*Croton gratissimus*	EU213805
Eudicotyledons	Asteraceae	*Eupatorium*	*Eupatorium fortunei*	GU724260
Eudicotyledons	Asteraceae	*Eupatorium*	*Eupatorium fortunei*	GQ435109
Eudicotyledons	Asteraceae	*Eupatorium*	*Eupatorium fortunei*	GQ435110
Eudicotyledons	Asteraceae	*Eupatorium*	*Eupatorium fortunei*	GQ435111
Eudicotyledons	Asteraceae	*Chrysanthemum*	*Chrysanthemum boreale*	AB234748
Eudicotyledons	Asteraceae	*Chrysanthemum*	*Chrysanthemum boreale*	AB234749
Eudicotyledons	Asteraceae	*Chrysanthemum*	*Chrysanthemum boreale*	AB234750
Eudicotyledons	Asteraceae	*Chrysanthemum*	*Chrysanthemum boreale*	AB234751
Eudicotyledons	Asteraceae	*Chrysanthemum*	*Chrysanthemum boreale*	AB234752
Eudicotyledons	Asteraceae	*Chrysanthemum*	*Chrysanthemum boreale*	AB234753
Eudicotyledons	Asteraceae	*Chrysanthemum*	*Chrysanthemum boreale*	AB234754
Eudicotyledons	Asteraceae	*Chrysanthemum*	*Chrysanthemum boreale*	AB234755
Eudicotyledons	Asteraceae	*Chrysanthemum*	*Chrysanthemum boreale*	AB234756
Eudicotyledons	Asteraceae	*Chrysanthemum*	*Chrysanthemum boreale*	AB234757
Eudicotyledons	Asteraceae	*Chrysanthemum*	*Chrysanthemum boreale*	AB234758
Eudicotyledons	Asteraceae	*Chrysanthemum*	*Chrysanthemum boreale*	AB234759
Eudicotyledons	Asteraceae	*Chrysanthemum*	*Chrysanthemum boreale*	AB234769
Eudicotyledons	Asteraceae	*Chrysanthemum*	*Chrysanthemum boreale*	AB234778
Eudicotyledons	Asteraceae	*Chrysanthemum*	*Chrysanthemum japonense*	AB234771
Eudicotyledons	Asteraceae	*Chrysanthemum*	*Chrysanthemum japonense*	AB234772
Eudicotyledons	Asteraceae	*Chrysanthemum*	*Chrysanthemum arisanense*	AB234780
Eudicotyledons	Asteraceae	*Chrysanthemum*	*Chrysanthemum arisanense*	AB234781
Eudicotyledons	Vitaceae	*Ampelopsis*	*Ampelopsis bodinieri*	JQ182519
Eudicotyledons	Vitaceae	*Ampelopsis*	*Ampelopsis bodinieri*	JF437060
Eudicotyledons	Vitaceae	*Ampelopsis*	*Ampelopsis bodinieri*	JF437061
Eudicotyledons	Vitaceae	*Ampelopsis*	*Ampelopsis cantoniensis*	JQ182498

Eudicotyledons	Vitaceae	*Ampelopsis*	*Ampelopsis cantoniensis*	JN043979
Eudicotyledons	Vitaceae	*Ampelopsis*	*Ampelopsis cantoniensis*	JN043980
Eudicotyledons	Vitaceae	*Ampelopsis*	*Ampelopsis cantoniensis*	JN043981
Eudicotyledons	Vitaceae	*Ampelopsis*	*Ampelopsis cantoniensis*	JN043982
Eudicotyledons	Vitaceae	*Ampelopsis*	*Ampelopsis cantoniensis*	HM585660
Eudicotyledons	Vitaceae	*Ampelopsis*	*Ampelopsis cantoniensis*	JF437062
Eudicotyledons	Vitaceae	*Ampelopsis*	*Ampelopsis chaffanjonii*	JQ182528
Eudicotyledons	Vitaceae	*Ampelopsis*	*Ampelopsis chaffanjonii*	JN043983
Eudicotyledons	Vitaceae	*Ampelopsis*	*Ampelopsis chaffanjonii*	JN043984
Eudicotyledons	Vitaceae	*Ampelopsis*	*Ampelopsis chaffanjonii*	JN043985
Eudicotyledons	Vitaceae	*Ampelopsis*	*Ampelopsis chaffanjonii*	JN043986
Eudicotyledons	Vitaceae	*Ampelopsis*	*Ampelopsis chaffanjonii*	JF437063
Eudicotyledons	Vitaceae	*Ampelopsis*	*Ampelopsis cordata*	JQ182513
Eudicotyledons	Vitaceae	*Ampelopsis*	*Ampelopsis cordata*	JF437064
Eudicotyledons	Vitaceae	*Ampelopsis*	*Ampelopsis delavayana*	JN043987
Eudicotyledons	Vitaceae	*Ampelopsis*	*Ampelopsis delavayana*	JN043988
Eudicotyledons	Vitaceae	*Ampelopsis*	*Ampelopsis delavayana*	JF437065
Eudicotyledons	Vitaceae	*Ampelopsis*	*Ampelopsis glandulosa*	JQ182530
Eudicotyledons	Vitaceae	*Ampelopsis*	*Ampelopsis glandulosa*	JF437067
Eudicotyledons	Vitaceae	*Ampelopsis*	*Ampelopsis glandulosa*	JF437068
Eudicotyledons	Vitaceae	*Ampelopsis*	*Ampelopsis glandulosa*	JF437069
Eudicotyledons	Vitaceae	*Cissus*	*Cissus incisa*	HM585670
Eudicotyledons	Vitaceae	*Cissus*	*Cissus incisa*	JF437094
Eudicotyledons	Vitaceae	*Tetrastigma*	*Tetrastigma laoticum*	HM585732
Eudicotyledons	Vitaceae	*Tetrastigma*	*Tetrastigma laoticum*	HM585733
Eudicotyledons	Vitaceae	*Tetrastigma*	*Tetrastigma laoticum*	HM585734
Eudicotyledons	Vitaceae	*Tetrastigma*	*Tetrastigma laoticum*	HM585735
Eudicotyledons	Vitaceae	*Tetrastigma*	*Tetrastigma obtectum*	HM585749
Eudicotyledons	Vitaceae	*Tetrastigma*	*Tetrastigma obtectum*	HM585750
Eudicotyledons	Vitaceae	*Tetrastigma*	*Tetrastigma obtectum*	HM585751
Eudicotyledons	Vitaceae	*Tetrastigma*	*Tetrastigma obtectum*	JF437146
Eudicotyledons	Vitaceae	*Tetrastigma*	*Tetrastigma obtectum*	JF437154
Eudicotyledons	Vitaceae	*Tetrastigma*	*Tetrastigma serrulatum*	HM585764
Eudicotyledons	Vitaceae	*Tetrastigma*	*Tetrastigma serrulatum*	HM585765
Eudicotyledons	Vitaceae	*Tetrastigma*	*Tetrastigma serrulatum*	HM585766
Eudicotyledons	Vitaceae	*Tetrastigma*	*Tetrastigma serrulatum*	JF437149
Eudicotyledons	Vitaceae	*Tetrastigma*	*Tetrastigma siamense*	HM585767
Eudicotyledons	Vitaceae	*Tetrastigma*	*Tetrastigma siamense*	JF437150
Eudicotyledons	Vitaceae	*Tetrastigma*	*Tetrastigma triphyllum*	HM585782
Eudicotyledons	Vitaceae	*Tetrastigma*	*Tetrastigma triphyllum*	HM585783
Eudicotyledons	Vitaceae	*Tetrastigma*	*Tetrastigma triphyllum*	JF437151
Eudicotyledons	Vitaceae	*Tetrastigma*	*Tetrastigma triphyllum*	JF437152
Eudicotyledons	Convolvulaceae	*Jacquemontia*	*Jacquemontia havanensis*	DQ994737
Eudicotyledons	Convolvulaceae	*Jacquemontia*	*Jacquemontia havanensis*	DQ994738
Eudicotyledons	Convolvulaceae	*Jacquemontia*	*Jacquemontia havanensis*	DQ994741
Eudicotyledons	Convolvulaceae	*Jacquemontia*	*Jacquemontia havanensis*	DQ994742
Eudicotyledons	Convolvulaceae	*Jacquemontia*	*Jacquemontia verticillata*	DQ994732
Eudicotyledons	Convolvulaceae	*Jacquemontia*	*Jacquemontia verticillata*	DQ994733
Eudicotyledons	Asteraceae	*Achillea*	*Achillea latiloba*	HQ451049
Eudicotyledons	Asteraceae	*Achillea*	*Achillea latiloba*	HQ451050
Eudicotyledons	Asteraceae	*Achillea*	*Achillea latiloba*	HQ451051
Eudicotyledons	Paeoniaceae	*Paeonia*	*Paeonia intermedia*	DQ313729
Eudicotyledons	Paeoniaceae	*Paeonia*	*Paeonia intermedia*	DQ313730

Eudicotyledons	Polygonaceae	*Bistorta*	*Bistorta vivipara*	JN046447
Eudicotyledons	Polygonaceae	*Bistorta*	*Bistorta vivipara*	JN046448
Eudicotyledons	Polygonaceae	*Bistorta*	*Bistorta vivipara*	JN046449
Eudicotyledons	Polygonaceae	*Bistorta*	*Bistorta vivipara*	JN046450
Eudicotyledons	Cactaceae	*Opuntia*	*Opuntia ficus-indica*	FJ026612
Eudicotyledons	Cactaceae	*Opuntia*	*Opuntia ficus-indica*	FJ026613
Eudicotyledons	Cactaceae	*Opuntia*	*Opuntia ficus-indica*	EU930405
Eudicotyledons	Cactaceae	*Opuntia*	*Opuntia ficus-indica*	EU930414
Eudicotyledons	Cactaceae	*Opuntia*	*Opuntia ficus-indica*	EU930418
Eudicotyledons	Cactaceae	*Opuntia*	*Opuntia ficus-indica*	EU930420
Eudicotyledons	Convolvulaceae	*Jacquemontia*	*Jacquemontia ovalifolia*	DQ994743
Eudicotyledons	Convolvulaceae	*Jacquemontia*	*Jacquemontia ovalifolia*	FJ792543
Eudicotyledons	Myrtaceae	*Psidium*	*Psidium cattleyanum*	AM489878
Eudicotyledons	Myrtaceae	*Psidium*	*Psidium cattleyanum*	GU135360
Eudicotyledons	Cactaceae	*Opuntia*	*Opuntia albicarpa*	EU930410
Eudicotyledons	Cactaceae	*Opuntia*	*Opuntia albicarpa*	EU930411
Eudicotyledons	Cactaceae	*Opuntia*	*Opuntia albicarpa*	EU930412
Eudicotyledons	Cactaceae	*Opuntia*	*Opuntia albicarpa*	EU930419
Eudicotyledons	Vitaceae	*Ampelopsis*	*Ampelopsis grossedentata*	JQ182531
Eudicotyledons	Vitaceae	*Ampelopsis*	*Ampelopsis grossedentata*	JN043989
Eudicotyledons	Vitaceae	*Ampelopsis*	*Ampelopsis grossedentata*	JN043990
Eudicotyledons	Vitaceae	*Ampelopsis*	*Ampelopsis grossedentata*	JN043991
Eudicotyledons	Vitaceae	*Ampelopsis*	*Ampelopsis grossedentata*	JF437070
Eudicotyledons	Vitaceae	*Cissus*	*Cissus aralioides*	JQ182508
Eudicotyledons	Vitaceae	*Cissus*	*Cissus aralioides*	JF437088
Eudicotyledons	Vitaceae	*Cissus*	*Cissus erosa*	HM585668
Eudicotyledons	Vitaceae	*Cissus*	*Cissus erosa*	JF437093
Eudicotyledons	Vitaceae	*Cissus*	*Cissus rotundifolia*	JF437098
Eudicotyledons	Vitaceae	*Cissus*	*Cissus rotundifolia*	JF437099
Eudicotyledons	Fagaceae	*Castanopsis*	*Castanopsis eyrei*	HQ427008
Eudicotyledons	Fagaceae	*Castanopsis*	*Castanopsis eyrei*	JN044201
Eudicotyledons	Fagaceae	*Castanopsis*	*Castanopsis eyrei*	JN044202
Eudicotyledons	Fagaceae	*Castanopsis*	*Castanopsis eyrei*	JN044203
Eudicotyledons	Fagaceae	*Castanopsis*	*Castanopsis fabri*	JN044204
Eudicotyledons	Fagaceae	*Castanopsis*	*Castanopsis fabri*	JN044205
Eudicotyledons	Fagaceae	*Castanopsis*	*Castanopsis fabri*	JN044206
Eudicotyledons	Fagaceae	*Castanopsis*	*Castanopsis fabri*	JN044207
Eudicotyledons	Fagaceae	*Castanopsis*	*Castanopsis sclerophylla*	JN044234
Eudicotyledons	Fagaceae	*Castanopsis*	*Castanopsis sclerophylla*	JN044235
Eudicotyledons	Fagaceae	*Castanopsis*	*Castanopsis sclerophylla*	JN044236
Eudicotyledons	Euphorbiaceae	*Croton*	*Croton billbergianus*	HM044813
Eudicotyledons	Euphorbiaceae	*Croton*	*Croton billbergianus*	GQ982201
Eudicotyledons	Vitaceae	*Tetrastigma*	*Tetrastigma rumicispermum*	HM585763
Eudicotyledons	Vitaceae	*Tetrastigma*	*Tetrastigma rumicispermum*	HQ656488
Eudicotyledons	Acanthaceae	*Ruellia*	*Ruellia bourgaei*	GQ995636
Eudicotyledons	Acanthaceae	*Ruellia*	*Ruellia bourgaei*	GQ995637
Eudicotyledons	Acanthaceae	*Ruellia*	*Ruellia bourgaei*	GQ995638
Eudicotyledons	Acanthaceae	*Ruellia*	*Ruellia bourgaei*	GQ995639
Eudicotyledons	Acanthaceae	*Ruellia*	*Ruellia bourgaei*	GQ995640
Eudicotyledons	Acanthaceae	*Ruellia*	*Ruellia conzattii*	GQ995643
Eudicotyledons	Acanthaceae	*Ruellia*	*Ruellia conzattii*	GQ995644
Eudicotyledons	Acanthaceae	*Ruellia*	*Ruellia conzattii*	GQ995645
Eudicotyledons	Acanthaceae	*Ruellia*	*Ruellia elegans*	GQ995628

Eudicotyledons	Acanthaceae	*Ruellia*	*Ruellia elegans*	GU003917
Eudicotyledons	Acanthaceae	*Ruellia*	*Ruellia jaliscana*	GQ995652
Eudicotyledons	Acanthaceae	*Ruellia*	*Ruellia jaliscana*	GQ995653
Eudicotyledons	Acanthaceae	*Ruellia*	*Ruellia jaliscana*	GQ995654
Eudicotyledons	Acanthaceae	*Ruellia*	*Ruellia speciosa*	GQ995661
Eudicotyledons	Acanthaceae	*Ruellia*	*Ruellia speciosa*	GQ995662
Eudicotyledons	Acanthaceae	*Ruellia*	*Ruellia speciosa*	GQ995663
Eudicotyledons	Acanthaceae	*Ruellia*	*Ruellia speciosa*	GQ995664
Eudicotyledons	Acanthaceae	*Ruellia*	*Ruellia fruticosa*	GQ995646
Eudicotyledons	Acanthaceae	*Ruellia*	*Ruellia fruticosa*	GQ995647
Eudicotyledons	Acanthaceae	*Ruellia*	*Ruellia fruticosa*	GQ995648
Eudicotyledons	Acanthaceae	*Ruellia*	*Ruellia fruticosa*	GQ995649
Eudicotyledons	Acanthaceae	*Ruellia*	*Ruellia fruticosa*	GQ995650
Eudicotyledons	Malvaceae	*Althaea*	*Althaea armeniaca*	EF679753
Eudicotyledons	Malvaceae	*Althaea*	*Althaea armeniaca*	EF419660
Eudicotyledons	Malvaceae	*Althaea*	*Althaea cannabina*	EF419657
Eudicotyledons	Malvaceae	*Althaea*	*Althaea cannabina*	EF419658
Eudicotyledons	Malvaceae	*Althaea*	*Althaea cannabina*	EF419659
Eudicotyledons	Malvaceae	*Althaea*	*Althaea hirsuta*	EF419620
Eudicotyledons	Malvaceae	*Althaea*	*Althaea hirsuta*	EF419621
Eudicotyledons	Malvaceae	*Althaea*	*Althaea hirsuta*	EF419622
Eudicotyledons	Malvaceae	*Althaea*	*Althaea hirsuta*	EF419623
Eudicotyledons	Malvaceae	*Althaea*	*Althaea hirsuta*	EF419624
Eudicotyledons	Malvaceae	*Althaea*	*Althaea hirsuta*	EF419625
Eudicotyledons	Malvaceae	*Althaea*	*Althaea longiflora*	EF419638
Eudicotyledons	Malvaceae	*Althaea*	*Althaea longiflora*	EF419639
Eudicotyledons	Malvaceae	*Althaea*	*Althaea longiflora*	EF419640
Eudicotyledons	Malvaceae	*Althaea*	*Althaea ludwigii*	EF419641
Eudicotyledons	Malvaceae	*Althaea*	*Althaea ludwigii*	EF419642
Eudicotyledons	Malvaceae	*Althaea*	*Althaea ludwigii*	EF419643
Eudicotyledons	Oleaceae	*Ligustrum*	*Ligustrum lucidum*	GU135318
Eudicotyledons	Oleaceae	*Ligustrum*	*Ligustrum lucidum*	FN675795
Eudicotyledons	Oleaceae	*Ligustrum*	*Ligustrum lucidum*	JF830327
Eudicotyledons	Oleaceae	*Ligustrum*	*Ligustrum lucidum*	JF830328
Eudicotyledons	Oleaceae	*Ligustrum*	*Ligustrum lucidum*	JF830329
Eudicotyledons	Oleaceae	*Ligustrum*	*Ligustrum lucidum*	JF830330
Eudicotyledons	Oleaceae	*Ligustrum*	*Ligustrum lucidum*	JF830331
Eudicotyledons	Oleaceae	*Ligustrum*	*Ligustrum lucidum*	JF830332
Eudicotyledons	Oleaceae	*Ligustrum*	*Ligustrum lucidum*	JF830333
Eudicotyledons	Oleaceae	*Ligustrum*	*Ligustrum lucidum*	JF830334
Eudicotyledons	Oleaceae	*Ligustrum*	*Ligustrum lucidum*	JN045225
Eudicotyledons	Oleaceae	*Ligustrum*	*Ligustrum lucidum*	JN045226
Eudicotyledons	Oleaceae	*Ligustrum*	*Ligustrum lucidum*	JN045227
Eudicotyledons	Oleaceae	*Ligustrum*	*Ligustrum lucidum*	JN045228
Eudicotyledons	Oleaceae	*Ligustrum*	*Ligustrum lucidum*	JN045229
Eudicotyledons	Paeoniaceae	*Paeonia*	*Paeonia ludlowii*	GU367371
Eudicotyledons	Paeoniaceae	*Paeonia*	*Paeonia ludlowii*	JF496835
Eudicotyledons	Asteraceae	*Gazania*	*Gazania caespitosa*	EF556366
Eudicotyledons	Asteraceae	*Gazania*	*Gazania caespitosa*	EF556367
Eudicotyledons	Asteraceae	*Gazania*	*Gazania heterochaeta*	EF556370
Eudicotyledons	Asteraceae	*Gazania*	*Gazania heterochaeta*	EF556371
Eudicotyledons	Asteraceae	*Gazania*	*Gazania leiopoda*	EF556382
Eudicotyledons	Asteraceae	*Gazania*	*Gazania leiopoda*	EF556383

Eudicotyledons	Asteraceae	*Gazania*	*Gazania lichtensteinii*	EF556384
Eudicotyledons	Asteraceae	*Gazania*	*Gazania lichtensteinii*	EF556385
Eudicotyledons	Asteraceae	*Gazania*	*Gazania maritima*	EF556390
Eudicotyledons	Asteraceae	*Gazania*	*Gazania maritima*	EF556391
Eudicotyledons	Asteraceae	*Gazania*	*Gazania pectinata*	EF556392
Eudicotyledons	Asteraceae	*Gazania*	*Gazania pectinata*	EF556393
Eudicotyledons	Asteraceae	*Gazania*	*Gazania rigida*	EF556400
Eudicotyledons	Asteraceae	*Gazania*	*Gazania rigida*	EF556401
Eudicotyledons	Asteraceae	*Gazania*	*Gazania serrata*	EF556403
Eudicotyledons	Asteraceae	*Gazania*	*Gazania serrata*	EF556404
Eudicotyledons	Asteraceae	*Gazania*	*Gazania sp. NPB-2007*	EF556407
Eudicotyledons	Asteraceae	*Gazania*	*Gazania sp. NPB-2007*	EF556408
Eudicotyledons	Polygonaceae	*Bistorta*	*Bistorta macrophylla*	JN046427
Eudicotyledons	Polygonaceae	*Bistorta*	*Bistorta macrophylla*	JN046428
Eudicotyledons	Polygonaceae	*Bistorta*	*Bistorta macrophylla*	JN046429
Eudicotyledons	Polygonaceae	*Bistorta*	*Bistorta macrophylla*	JN046430
Eudicotyledons	Polygonaceae	*Bistorta*	*Bistorta macrophylla*	JN046431
Eudicotyledons	Polygonaceae	*Bistorta*	*Bistorta macrophylla*	JN046432
Eudicotyledons	Polygonaceae	*Bistorta*	*Bistorta macrophylla*	JN046433
Eudicotyledons	Polygonaceae	*Bistorta*	*Bistorta macrophylla*	JN046434
Eudicotyledons	Fabaceae	*Inga*	*Inga marginata*	GQ118873
Eudicotyledons	Fabaceae	*Inga*	*Inga marginata*	GQ982255
Eudicotyledons	Fabaceae	*Inga*	*Inga marginata*	AM922030
Eudicotyledons	Fabaceae	*Inga*	*Inga marginata*	AM922031
Eudicotyledons	Fabaceae	*Inga*	*Inga marginata*	AM922032
Eudicotyledons	Fabaceae	*Inga*	*Inga marginata*	AM922033
Eudicotyledons	Fabaceae	*Inga*	*Inga acreana*	GQ118861
Eudicotyledons	Fabaceae	*Inga*	*Inga acreana*	GQ428678
Eudicotyledons	Fabaceae	*Inga*	*Inga acreana*	FJ038935
Eudicotyledons	Fabaceae	*Inga*	*Inga acuminata*	GQ118862
Eudicotyledons	Fabaceae	*Inga*	*Inga acuminata*	GQ982251
Eudicotyledons	Fabaceae	*Inga*	*Inga acuminata*	AM922015
Eudicotyledons	Fabaceae	*Inga*	*Inga auristellae*	GQ118865
Eudicotyledons	Fabaceae	*Inga*	*Inga auristellae*	AM922016
Eudicotyledons	Fabaceae	*Inga*	*Inga bourgonii*	GQ118866
Eudicotyledons	Fabaceae	*Inga*	*Inga bourgonii*	AM922017
Eudicotyledons	Fabaceae	*Inga*	*Inga cocleensis*	GQ118869
Eudicotyledons	Fabaceae	*Inga*	*Inga cocleensis*	GQ982252
Eudicotyledons	Fabaceae	*Inga*	*Inga feuillei*	AM922022
Eudicotyledons	Fabaceae	*Inga*	*Inga feuillei*	AM922023
Eudicotyledons	Fabaceae	*Inga*	*Inga feuillei*	AM922024
Eudicotyledons	Fabaceae	*Inga*	*Inga goldmanii*	GQ118871
Eudicotyledons	Fabaceae	*Inga*	*Inga goldmanii*	GQ982253
Eudicotyledons	Fabaceae	*Inga*	*Inga goldmanii*	AM922025
Eudicotyledons	Fabaceae	*Inga*	*Inga leiocalycina*	FJ038941
Eudicotyledons	Fabaceae	*Inga*	*Inga leiocalycina*	AM922028
Eudicotyledons	Fabaceae	*Inga*	*Inga multijuga*	GQ118878
Eudicotyledons	Fabaceae	*Inga*	*Inga multijuga*	AM922034
Eudicotyledons	Fabaceae	*Inga*	*Inga multijuga*	AM922035
Eudicotyledons	Fabaceae	*Inga*	*Inga nobilis*	GQ118879
Eudicotyledons	Fabaceae	*Inga*	*Inga nobilis*	GQ982256
Eudicotyledons	Fabaceae	*Inga*	*Inga nobilis*	AM922036
Eudicotyledons	Fabaceae	*Inga*	*Inga nobilis*	AM922037

Eudicotyledons	Fabaceae	*Inga*	*Inga nobilis*	AM922038
Eudicotyledons	Fabaceae	*Inga*	*Inga pezizifera*	GQ118880
Eudicotyledons	Fabaceae	*Inga*	*Inga pezizifera*	GQ982258
Eudicotyledons	Fabaceae	*Inga*	*Inga ruiziana*	GQ118883
Eudicotyledons	Fabaceae	*Inga*	*Inga ruiziana*	GQ982260
Eudicotyledons	Fabaceae	*Inga*	*Inga sapindoides*	GQ118884
Eudicotyledons	Fabaceae	*Inga*	*Inga sapindoides*	GQ982261
Eudicotyledons	Fabaceae	*Inga*	*Inga sapindoides*	AM922047
Eudicotyledons	Fabaceae	*Inga*	*Inga sapindoides*	AM922049
Eudicotyledons	Fabaceae	*Inga*	*Inga sapindoides*	AM922050
Eudicotyledons	Fabaceae	*Inga*	*Inga spectabilis*	GQ982262
Eudicotyledons	Fabaceae	*Inga*	*Inga spectabilis*	AM922055
Eudicotyledons	Fabaceae	*Inga*	*Inga tenuistipula*	GQ118885
Eudicotyledons	Fabaceae	*Inga*	*Inga tenuistipula*	AM922056
Eudicotyledons	Fabaceae	*Inga*	*Inga thibaudiana*	GQ118886
Eudicotyledons	Fabaceae	*Inga*	*Inga thibaudiana*	GQ982263
Eudicotyledons	Fabaceae	*Inga*	*Inga umbellifera*	GQ118888
Eudicotyledons	Fabaceae	*Inga*	*Inga umbellifera*	GQ982264
Eudicotyledons	Fabaceae	*Inga*	*Inga umbellifera*	AM922057
Eudicotyledons	Fabaceae	*Inga*	*Inga umbellifera*	AM922058
Eudicotyledons	Fabaceae	*Inga*	*Inga vera*	GQ118890
Eudicotyledons	Fabaceae	*Inga*	*Inga vera*	HM446947
Eudicotyledons	Fabaceae	*Inga*	*Inga laurina*	GQ118877
Eudicotyledons	Fabaceae	*Inga*	*Inga laurina*	GQ982254
Eudicotyledons	Fabaceae	*Inga*	*Inga laurina*	AM922044
Eudicotyledons	Fabaceae	*Inga*	*Inga laurina*	AM922045
Eudicotyledons	Fabaceae	*Inga*	*Inga laurina*	AM922046
Eudicotyledons	Fabaceae	*Inga*	*Inga laurina*	HM446946
Eudicotyledons	Euphorbiaceae	*Croton*	*Croton megalobotrys*	EU213806
Eudicotyledons	Euphorbiaceae	*Croton*	*Croton megalobotrys*	EU213807
Eudicotyledons	Euphorbiaceae	*Croton*	*Croton megalobotrys*	EU213808
Eudicotyledons	Euphorbiaceae	*Croton*	*Croton pseudopulchellus*	EU213809
Eudicotyledons	Euphorbiaceae	*Croton*	*Croton pseudopulchellus*	EU213810
Eudicotyledons	Euphorbiaceae	*Croton*	*Croton pseudopulchellus*	EU213811
Eudicotyledons	Asteraceae	*Tragopogon*	*Tragopogon buphthalmoides*	EU391967
Eudicotyledons	Asteraceae	*Tragopogon*	*Tragopogon buphthalmoides*	EU391968
Eudicotyledons	Melastomataceae	*Melastoma*	*Melastoma tetramerum*	AB443945
Eudicotyledons	Melastomataceae	*Melastoma*	*Melastoma tetramerum*	AB443946
Eudicotyledons	Oleaceae	*Ligustrum*	*Ligustrum quihoui*	GQ435224
Eudicotyledons	Oleaceae	*Ligustrum*	*Ligustrum quihoui*	JF830270
Eudicotyledons	Oleaceae	*Ligustrum*	*Ligustrum quihoui*	JF830271
Eudicotyledons	Oleaceae	*Ligustrum*	*Ligustrum quihoui*	JF830272
Eudicotyledons	Oleaceae	*Ligustrum*	*Ligustrum quihoui*	JF830273
Eudicotyledons	Oleaceae	*Ligustrum*	*Ligustrum quihoui*	JF830274
Eudicotyledons	Oleaceae	*Ligustrum*	*Ligustrum quihoui*	JF830275
Eudicotyledons	Oleaceae	*Ligustrum*	*Ligustrum quihoui*	JN045234
Eudicotyledons	Oleaceae	*Ligustrum*	*Ligustrum quihoui*	JN045235
Eudicotyledons	Cactaceae	*Opuntia*	*Opuntia joconostle*	EU930406
Eudicotyledons	Cactaceae	*Opuntia*	*Opuntia joconostle*	EU930407
Eudicotyledons	Oleaceae	*Picconia*	*Picconia azorica*	EU854410
Eudicotyledons	Oleaceae	*Picconia*	*Picconia azorica*	EU854411
Eudicotyledons	Oleaceae	*Picconia*	*Picconia azorica*	EU854412
Eudicotyledons	Oleaceae	*Picconia*	*Picconia azorica*	EU854413

Eudicotyledons	Oleaceae	*Picconia*	*Picconia azorica*	EU854414
Eudicotyledons	Oleaceae	*Picconia*	*Picconia azorica*	EU854415
Eudicotyledons	Oleaceae	*Picconia*	*Picconia azorica*	EU854416
Eudicotyledons	Rosaceae	*Cliffortia*	*Cliffortia amplexistipula*	EU937660
Eudicotyledons	Rosaceae	*Cliffortia*	*Cliffortia amplexistipula*	EU937661
Eudicotyledons	Rosaceae	*Cliffortia*	*Cliffortia arborea*	EU937569
Eudicotyledons	Rosaceae	*Cliffortia*	*Cliffortia arborea*	EU937570
Eudicotyledons	Rosaceae	*Cliffortia*	*Cliffortia erectisepala*	EU937643
Eudicotyledons	Rosaceae	*Cliffortia*	*Cliffortia erectisepala*	EU937644
Eudicotyledons	Rosaceae	*Cliffortia*	*Cliffortia eriocephalina*	EU937573
Eudicotyledons	Rosaceae	*Cliffortia*	*Cliffortia eriocephalina*	EU937574
Eudicotyledons	Rosaceae	*Cliffortia*	*Cliffortia ferruginea*	EU937603
Eudicotyledons	Rosaceae	*Cliffortia*	*Cliffortia ferruginea*	EU937604
Eudicotyledons	Rosaceae	*Cliffortia*	*Cliffortia integerrima*	EU937629
Eudicotyledons	Rosaceae	*Cliffortia*	*Cliffortia integerrima*	EU937630
Eudicotyledons	Rosaceae	*Cliffortia*	*Cliffortia intermedia*	EU937624
Eudicotyledons	Rosaceae	*Cliffortia*	*Cliffortia intermedia*	EU937625
Eudicotyledons	Rosaceae	*Cliffortia*	*Cliffortia linearifolia*	EU937691
Eudicotyledons	Rosaceae	*Cliffortia*	*Cliffortia linearifolia*	EU937692
Eudicotyledons	Rosaceae	*Cliffortia*	*Cliffortia marginata*	EU937668
Eudicotyledons	Rosaceae	*Cliffortia*	*Cliffortia marginata*	EU937669
Eudicotyledons	Rosaceae	*Cliffortia*	*Cliffortia montana*	EU937576
Eudicotyledons	Rosaceae	*Cliffortia*	*Cliffortia montana*	EU937577
Eudicotyledons	Rosaceae	*Cliffortia*	*Cliffortia ramosissima*	EU937715
Eudicotyledons	Rosaceae	*Cliffortia*	*Cliffortia ramosissima*	EU937716
Eudicotyledons	Rosaceae	*Cliffortia*	*Cliffortia ruscifolia x Cliffortia teretifo*	EU937726
Eudicotyledons	Rosaceae	*Cliffortia*	*Cliffortia ruscifolia x Cliffortia teretifo*	EU937727
Eudicotyledons	Cactaceae	*Opuntia*	*Opuntia megacantha*	EU930403
Eudicotyledons	Cactaceae	*Opuntia*	*Opuntia megacantha*	EU930409
Eudicotyledons	Cactaceae	*Opuntia*	*Opuntia megacantha*	EU930413
Eudicotyledons	Cactaceae	*Opuntia*	*Opuntia megacantha*	EU930415
Eudicotyledons	Cactaceae	*Opuntia*	*Opuntia megacantha*	EU930416
Eudicotyledons	Ericaceae	*Gaultheria*	*Gaultheria dumicola*	JN044571
Eudicotyledons	Ericaceae	*Gaultheria*	*Gaultheria dumicola*	JN044572
Eudicotyledons	Ericaceae	*Gaultheria*	*Gaultheria dumicola*	JN044573
Eudicotyledons	Ericaceae	*Gaultheria*	*Gaultheria dumicola*	JN044574
Eudicotyledons	Ericaceae	*Gaultheria*	*Gaultheria dumicola*	JN044575
Eudicotyledons	Ericaceae	*Gaultheria*	*Gaultheria dumicola*	JN044576
Eudicotyledons	Ericaceae	*Gaultheria*	*Gaultheria dumicola*	JN044577
Eudicotyledons	Ericaceae	*Gaultheria*	*Gaultheria fragrantissima*	JN044580
Eudicotyledons	Ericaceae	*Gaultheria*	*Gaultheria fragrantissima*	JN044581
Eudicotyledons	Ericaceae	*Gaultheria*	*Gaultheria fragrantissima*	JN044582
Eudicotyledons	Ericaceae	*Gaultheria*	*Gaultheria fragrantissima*	JN044583
Eudicotyledons	Ericaceae	*Gaultheria*	*Gaultheria fragrantissima*	JN044584
Eudicotyledons	Ericaceae	*Gaultheria*	*Gaultheria fragrantissima*	JN044585
Eudicotyledons	Ericaceae	*Gaultheria*	*Gaultheria fragrantissima*	JN044586
Eudicotyledons	Ericaceae	*Gaultheria*	*Gaultheria fragrantissima*	JN044587
Eudicotyledons	Ericaceae	*Gaultheria*	*Gaultheria fragrantissima*	JN044588
Eudicotyledons	Ericaceae	*Gaultheria*	*Gaultheria fragrantissima*	JN044589
Eudicotyledons	Ericaceae	*Gaultheria*	*Gaultheria fragrantissima*	JN044590
Eudicotyledons	Ericaceae	*Gaultheria*	*Gaultheria fragrantissima*	JN044591
Eudicotyledons	Ericaceae	*Gaultheria*	*Gaultheria fragrantissima*	JN044592
Eudicotyledons	Ericaceae	*Gaultheria*	*Gaultheria fragrantissima*	JN044593

Eudicotyledons	Ericaceae	*Gaultheria*	*Gaultheria fragrantissima*	JN044594
Eudicotyledons	Ericaceae	*Gaultheria*	*Gaultheria fragrantissima*	JN044595
Eudicotyledons	Ericaceae	*Gaultheria*	*Gaultheria fragrantissima*	JN044596
Eudicotyledons	Ericaceae	*Gaultheria*	*Gaultheria griffithiana*	JN044597
Eudicotyledons	Ericaceae	*Gaultheria*	*Gaultheria griffithiana*	JN044598
Eudicotyledons	Ericaceae	*Gaultheria*	*Gaultheria griffithiana*	JN044599
Eudicotyledons	Ericaceae	*Gaultheria*	*Gaultheria griffithiana*	JN044600
Eudicotyledons	Ericaceae	*Gaultheria*	*Gaultheria griffithiana*	JN044601
Eudicotyledons	Ericaceae	*Gaultheria*	*Gaultheria griffithiana*	JN044602
Eudicotyledons	Ericaceae	*Gaultheria*	*Gaultheria griffithiana*	JN044603
Eudicotyledons	Ericaceae	*Gaultheria*	*Gaultheria griffithiana*	JN044604
Eudicotyledons	Ericaceae	*Gaultheria*	*Gaultheria griffithiana*	JN044605
Eudicotyledons	Ericaceae	*Gaultheria*	*Gaultheria griffithiana*	JN044606
Eudicotyledons	Ericaceae	*Gaultheria*	*Gaultheria griffithiana*	JN044607
Eudicotyledons	Ericaceae	*Gaultheria*	*Gaultheria griffithiana*	JN044608
Eudicotyledons	Ericaceae	*Gaultheria*	*Gaultheria hookeri*	JN044613
Eudicotyledons	Ericaceae	*Gaultheria*	*Gaultheria hookeri*	JN044614
Eudicotyledons	Ericaceae	*Gaultheria*	*Gaultheria hookeri*	JN044615
Eudicotyledons	Ericaceae	*Gaultheria*	*Gaultheria hookeri*	JN044616
Eudicotyledons	Ericaceae	*Gaultheria*	*Gaultheria hookeri*	JN044617
Eudicotyledons	Ericaceae	*Gaultheria*	*Gaultheria hookeri*	JN044618
Eudicotyledons	Ericaceae	*Gaultheria*	*Gaultheria hookeri*	JN044619
Eudicotyledons	Ericaceae	*Gaultheria*	*Gaultheria sinensis*	JN044690
Eudicotyledons	Ericaceae	*Gaultheria*	*Gaultheria sinensis*	JN044691
Eudicotyledons	Ericaceae	*Gaultheria*	*Gaultheria sinensis*	JN044692
Eudicotyledons	Ericaceae	*Gaultheria*	*Gaultheria sinensis*	JN044693
Eudicotyledons	Ericaceae	*Gaultheria*	*Gaultheria sinensis*	JN044694
Eudicotyledons	Ericaceae	*Gaultheria*	*Gaultheria sinensis*	JN044695
Eudicotyledons	Ericaceae	*Gaultheria*	*Gaultheria sinensis*	JN044696
Eudicotyledons	Ericaceae	*Gaultheria*	*Gaultheria sinensis*	JN044697
Eudicotyledons	Ericaceae	*Gaultheria*	*Gaultheria wardii*	JN044722
Eudicotyledons	Ericaceae	*Gaultheria*	*Gaultheria wardii*	JN044723
Eudicotyledons	Ericaceae	*Gaultheria*	*Gaultheria wardii*	JN044724
Eudicotyledons	Ericaceae	*Gaultheria*	*Gaultheria wardii*	JN044725
Eudicotyledons	Ericaceae	*Gaultheria*	*Gaultheria wardii*	JN044726
Eudicotyledons	Ericaceae	*Gaultheria*	*Gaultheria wardii*	JN044727
Eudicotyledons	Ericaceae	*Gaultheria*	*Gaultheria wardii*	JN044728
Eudicotyledons	Ericaceae	*Gaultheria*	*Gaultheria wardii*	JN044729
Eudicotyledons	Ericaceae	*Gaultheria*	*Gaultheria wardii*	JN044730
Eudicotyledons	Ericaceae	*Gaultheria*	*Gaultheria wardii*	JN044731
Eudicotyledons	Ericaceae	*Gaultheria*	*Gaultheria wardii*	JN044732
Eudicotyledons	Ericaceae	*Gaultheria*	*Gaultheria wardii*	JN044733
Eudicotyledons	Violaceae	*Viola*	*Viola prionantha*	GQ435053
Eudicotyledons	Violaceae	*Viola*	*Viola prionantha*	HM483589
Eudicotyledons	Sapotaceae	*Chrysophyllum*	*Chrysophyllum sanguinolentum*	FJ039055
Eudicotyledons	Sapotaceae	*Chrysophyllum*	*Chrysophyllum sanguinolentum*	FJ039056
Eudicotyledons	Ebenaceae	*Diospyros*	*Diospyros capreifolia*	GQ428662
Eudicotyledons	Ebenaceae	*Diospyros*	*Diospyros capreifolia*	GQ428664
Eudicotyledons	Ebenaceae	*Diospyros*	*Diospyros capreifolia*	FJ038911
Eudicotyledons	Ebenaceae	*Diospyros*	*Diospyros carbonaria*	GQ428663
Eudicotyledons	Ebenaceae	*Diospyros*	*Diospyros carbonaria*	FJ038912
Eudicotyledons	Fabaceae	*Inga*	*Inga brachystachys*	GQ428677
Eudicotyledons	Fabaceae	*Inga*	*Inga brachystachys*	FJ038939

Eudicotyledons	Fabaceae	*Inga*	*Inga huberi*	GQ428684
Eudicotyledons	Fabaceae	*Inga*	*Inga huberi*	FJ038940
Eudicotyledons	Fabaceae	*Inga*	*Inga nouragensis*	GQ428682
Eudicotyledons	Fabaceae	*Inga*	*Inga nouragensis*	GQ428683
Eudicotyledons	Fabaceae	*Inga*	*Inga paraensis*	GQ428676
Eudicotyledons	Fabaceae	*Inga*	*Inga paraensis*	FJ038937
Eudicotyledons	Fabaceae	*Inga*	*Inga paraensis*	FJ038938
Eudicotyledons	Burseraceae	*Protium*	*Protium gallicum*	GQ428759
Eudicotyledons	Burseraceae	*Protium*	*Protium gallicum*	FJ038873
Eudicotyledons	Burseraceae	*Protium*	*Protium gallicum*	FJ038874
Eudicotyledons	Ericaceae	*Enkianthus*	*Enkianthus quinqueflorus*	HQ415415
Eudicotyledons	Ericaceae	*Enkianthus*	*Enkianthus quinqueflorus*	JN044465
Eudicotyledons	Ericaceae	*Enkianthus*	*Enkianthus quinqueflorus*	JN044466
Eudicotyledons	Ericaceae	*Enkianthus*	*Enkianthus quinqueflorus*	JN044467
Eudicotyledons	Ericaceae	*Enkianthus*	*Enkianthus quinqueflorus*	JN044468
Eudicotyledons	Ericaceae	*Enkianthus*	*Enkianthus quinqueflorus*	JN044469
Eudicotyledons	Fabaceae	*Inga*	*Inga alba*	GQ118864
Eudicotyledons	Fabaceae	*Inga*	*Inga alba*	GQ428679
Eudicotyledons	Onagraceae	*Epilobium*	*Epilobium palustre*	JN044479
Eudicotyledons	Onagraceae	*Epilobium*	*Epilobium palustre*	JN129840
Eudicotyledons	Primulaceae	*Ardisia*	*Ardisia standleyana*	GQ982151
Eudicotyledons	Primulaceae	*Ardisia*	*Ardisia standleyana*	GQ982152
Eudicotyledons	Salicaceae	*Casearia*	*Casearia guianensis*	GQ982169
Eudicotyledons	Salicaceae	*Casearia*	*Casearia guianensis*	HM446897
Eudicotyledons	Asteraceae	*Tolpis*	*Tolpis glabrescens*	GU046625
Eudicotyledons	Asteraceae	*Tolpis*	*Tolpis glabrescens*	GU046626
Eudicotyledons	Asteraceae	*Tolpis*	*Tolpis lagopoda*	GU046628
Eudicotyledons	Asteraceae	*Tolpis*	*Tolpis lagopoda*	GU046629
Eudicotyledons	Asteraceae	*Tolpis*	*Tolpis sp. 5 MEM-2009*	GU046636
Eudicotyledons	Asteraceae	*Tolpis*	*Tolpis sp. 5 MEM-2009*	GU046637
Eudicotyledons	Asteraceae	*Tolpis*	*Tolpis webbii*	GU046631
Eudicotyledons	Asteraceae	*Tolpis*	*Tolpis webbii*	GU046632
Eudicotyledons	Rosaceae	*Pyracantha*	*Pyracantha angustifolia*	JQ390760
Eudicotyledons	Rosaceae	*Pyracantha*	*Pyracantha angustifolia*	JN046699
Eudicotyledons	Rosaceae	*Pyracantha*	*Pyracantha angustifolia*	JN046700
Eudicotyledons	Rosaceae	*Pyracantha*	*Pyracantha angustifolia*	JN046701
Eudicotyledons	Rosaceae	*Pyracantha*	*Pyracantha angustifolia*	JN046702
Eudicotyledons	Rosaceae	*Pyracantha*	*Pyracantha angustifolia*	JN046703
Eudicotyledons	Rosaceae	*Pyracantha*	*Pyracantha angustifolia*	JN046704
Eudicotyledons	Rosaceae	*Pyracantha*	*Pyracantha angustifolia*	JN046705
Eudicotyledons	Acanthaceae	*Ruellia*	*Ruellia tweediana*	GU135339
Eudicotyledons	Acanthaceae	*Ruellia*	*Ruellia tweediana*	GU135437
Eudicotyledons	Ericaceae	*Erica*	*Erica trimera*	GU323906
Eudicotyledons	Ericaceae	*Erica*	*Erica trimera*	GU323907
Eudicotyledons	Ericaceae	*Erica*	*Erica trimera*	GU323908
Eudicotyledons	Ericaceae	*Erica*	*Erica trimera*	GU323909
Eudicotyledons	Vitaceae	*Ampelopsis*	*Ampelopsis japonica*	JQ182491
Eudicotyledons	Vitaceae	*Ampelopsis*	*Ampelopsis japonica*	JF437071
Eudicotyledons	Cactaceae	*Lepismium*	*Lepismium lorentzianum*	FN669015
Eudicotyledons	Cactaceae	*Lepismium*	*Lepismium lorentzianum*	FN669017
Eudicotyledons	Cactaceae	*Lepismium*	*Lepismium lumbricoides*	FN669009
Eudicotyledons	Cactaceae	*Lepismium*	*Lepismium lumbricoides*	FN669010
Eudicotyledons	Cactaceae	*Lepismium*	*Lepismium lumbricoides*	FN669011

Eudicotyledons	Cactaceae	*Rhipsalis*	*Rhipsalis baccifera*	FN995435
Eudicotyledons	Cactaceae	*Rhipsalis*	*Rhipsalis baccifera*	FN669073
Eudicotyledons	Cactaceae	*Rhipsalis*	*Rhipsalis baccifera*	FN669079
Eudicotyledons	Cactaceae	*Rhipsalis*	*Rhipsalis elliptica*	FN669087
Eudicotyledons	Cactaceae	*Rhipsalis*	*Rhipsalis elliptica*	FN669089
Eudicotyledons	Cactaceae	*Rhipsalis*	*Rhipsalis grandiflora*	FN669040
Eudicotyledons	Cactaceae	*Rhipsalis*	*Rhipsalis grandiflora*	FN669041
Eudicotyledons	Cactaceae	*Rhipsalis*	*Rhipsalis grandiflora*	FN669042
Eudicotyledons	Cactaceae	*Rhipsalis*	*Rhipsalis grandiflora*	FN669081
Eudicotyledons	Cactaceae	*Rhipsalis*	*Rhipsalis micrantha*	FN669097
Eudicotyledons	Cactaceae	*Rhipsalis*	*Rhipsalis micrantha*	FN669098
Eudicotyledons	Cactaceae	*Rhipsalis*	*Rhipsalis oblonga*	FN669088
Eudicotyledons	Cactaceae	*Rhipsalis*	*Rhipsalis oblonga*	FN669100
Eudicotyledons	Cactaceae	*Rhipsalis*	*Rhipsalis occidentalis*	FN669090
Eudicotyledons	Cactaceae	*Rhipsalis*	*Rhipsalis occidentalis*	FN669091
Eudicotyledons	Cactaceae	*Rhipsalis*	*Rhipsalis pachyptera*	FN669055
Eudicotyledons	Cactaceae	*Rhipsalis*	*Rhipsalis pachyptera*	FN669057
Eudicotyledons	Cactaceae	*Rhipsalis*	*Rhipsalis pentaptera*	FN669102
Eudicotyledons	Cactaceae	*Rhipsalis*	*Rhipsalis pentaptera*	FN669103
Eudicotyledons	Cactaceae	*Rhipsalis*	*Rhipsalis pilocarpa*	FN669058
Eudicotyledons	Cactaceae	*Rhipsalis*	*Rhipsalis pilocarpa*	FN669059
Eudicotyledons	Cactaceae	*Rhipsalis*	*Rhipsalis puniceodiscus*	FN669108
Eudicotyledons	Cactaceae	*Rhipsalis*	*Rhipsalis puniceodiscus*	FN669109
Eudicotyledons	Ericaceae	*Cassiope*	*Cassiope dendrotricha*	JN044173
Eudicotyledons	Ericaceae	*Cassiope*	*Cassiope dendrotricha*	JN044174
Eudicotyledons	Ericaceae	*Cassiope*	*Cassiope pectinata*	JN044179
Eudicotyledons	Ericaceae	*Cassiope*	*Cassiope pectinata*	JN044180
Eudicotyledons	Ericaceae	*Cassiope*	*Cassiope selaginoides*	JN044181
Eudicotyledons	Ericaceae	*Cassiope*	*Cassiope selaginoides*	JN044182
Eudicotyledons	Ericaceae	*Cassiope*	*Cassiope selaginoides*	JN044183
Eudicotyledons	Ericaceae	*Cassiope*	*Cassiope selaginoides*	JN044184
Eudicotyledons	Ericaceae	*Cassiope*	*Cassiope selaginoides*	JN044185
Eudicotyledons	Ericaceae	*Cassiope*	*Cassiope selaginoides*	JN044186
Eudicotyledons	Euphorbiaceae	*Croton*	*Croton orinocensis*	HM044833
Eudicotyledons	Euphorbiaceae	*Croton*	*Croton orinocensis*	HM044834
Eudicotyledons	Campanulaceae	*Cyananthus*	*Cyananthus macrocalyx*	JN044357
Eudicotyledons	Campanulaceae	*Cyananthus*	*Cyananthus macrocalyx*	JN044358
Eudicotyledons	Campanulaceae	*Cyananthus*	*Cyananthus macrocalyx*	JN044359
Eudicotyledons	Campanulaceae	*Cyananthus*	*Cyananthus macrocalyx*	JN044360
Eudicotyledons	Ericaceae	*Gaultheria*	*Gaultheria brevistipes*	JN044552
Eudicotyledons	Ericaceae	*Gaultheria*	*Gaultheria brevistipes*	JN044553
Eudicotyledons	Ericaceae	*Gaultheria*	*Gaultheria brevistipes*	JN044554
Eudicotyledons	Ericaceae	*Gaultheria*	*Gaultheria brevistipes*	JN044555
Eudicotyledons	Ericaceae	*Gaultheria*	*Gaultheria cardiosepala*	JN044556
Eudicotyledons	Ericaceae	*Gaultheria*	*Gaultheria cardiosepala*	JN044557
Eudicotyledons	Ericaceae	*Gaultheria*	*Gaultheria cardiosepala*	JN044558
Eudicotyledons	Ericaceae	*Gaultheria*	*Gaultheria cardiosepala*	JN044559
Eudicotyledons	Ericaceae	*Gaultheria*	*Gaultheria cardiosepala*	JN044560
Eudicotyledons	Ericaceae	*Gaultheria*	*Gaultheria codonantha*	JN044561
Eudicotyledons	Ericaceae	*Gaultheria*	*Gaultheria codonantha*	JN044562
Eudicotyledons	Ericaceae	*Gaultheria*	*Gaultheria codonantha*	JN044563
Eudicotyledons	Ericaceae	*Gaultheria*	*Gaultheria codonantha*	JN044564
Eudicotyledons	Ericaceae	*Gaultheria*	*Gaultheria cuneata*	JN044565

Eudicotyledons	Ericaceae	*Gaultheria*	*Gaultheria cuneata*	JN044566
Eudicotyledons	Ericaceae	*Gaultheria*	*Gaultheria dolichopoda*	JN044567
Eudicotyledons	Ericaceae	*Gaultheria*	*Gaultheria dolichopoda*	JN044568
Eudicotyledons	Ericaceae	*Gaultheria*	*Gaultheria dolichopoda*	JN044569
Eudicotyledons	Ericaceae	*Gaultheria*	*Gaultheria dolichopoda*	JN044570
Eudicotyledons	Ericaceae	*Gaultheria*	*Gaultheria eciliata*	JN044578
Eudicotyledons	Ericaceae	*Gaultheria*	*Gaultheria eciliata*	JN044579
Eudicotyledons	Ericaceae	*Gaultheria*	*Gaultheria heteromera*	JN044609
Eudicotyledons	Ericaceae	*Gaultheria*	*Gaultheria heteromera*	JN044610
Eudicotyledons	Ericaceae	*Gaultheria*	*Gaultheria heteromera*	JN044611
Eudicotyledons	Ericaceae	*Gaultheria*	*Gaultheria heteromera*	JN044612
Eudicotyledons	Ericaceae	*Gaultheria*	*Gaultheria hypochlora*	JN044620
Eudicotyledons	Ericaceae	*Gaultheria*	*Gaultheria hypochlora*	JN044621
Eudicotyledons	Ericaceae	*Gaultheria*	*Gaultheria hypochlora*	JN044622
Eudicotyledons	Ericaceae	*Gaultheria*	*Gaultheria hypochlora*	JN044623
Eudicotyledons	Ericaceae	*Gaultheria*	*Gaultheria hypochlora*	JN044624
Eudicotyledons	Ericaceae	*Gaultheria*	*Gaultheria hypochlora*	JN044625
Eudicotyledons	Ericaceae	*Gaultheria*	*Gaultheria jingdongensis*	JN044626
Eudicotyledons	Ericaceae	*Gaultheria*	*Gaultheria jingdongensis*	JN044627
Eudicotyledons	Ericaceae	*Gaultheria*	*Gaultheria jingdongensis*	JN044628
Eudicotyledons	Ericaceae	*Gaultheria*	*Gaultheria jingdongensis*	JN044629
Eudicotyledons	Ericaceae	*Gaultheria*	*Gaultheria longibracteolata*	JN044650
Eudicotyledons	Ericaceae	*Gaultheria*	*Gaultheria longibracteolata*	JN044651
Eudicotyledons	Ericaceae	*Gaultheria*	*Gaultheria longibracteolata*	JN044652
Eudicotyledons	Ericaceae	*Gaultheria*	*Gaultheria notabilis*	JN044653
Eudicotyledons	Ericaceae	*Gaultheria*	*Gaultheria notabilis*	JN044654
Eudicotyledons	Ericaceae	*Gaultheria*	*Gaultheria notabilis*	JN044655
Eudicotyledons	Ericaceae	*Gaultheria*	*Gaultheria notabilis*	JN044656
Eudicotyledons	Ericaceae	*Gaultheria*	*Gaultheria praticola*	JN044666
Eudicotyledons	Ericaceae	*Gaultheria*	*Gaultheria praticola*	JN044667
Eudicotyledons	Ericaceae	*Gaultheria*	*Gaultheria praticola*	JN044668
Eudicotyledons	Ericaceae	*Gaultheria*	*Gaultheria praticola*	JN044669
Eudicotyledons	Ericaceae	*Gaultheria*	*Gaultheria praticola*	JN044670
Eudicotyledons	Ericaceae	*Gaultheria*	*Gaultheria praticola*	JN044671
Eudicotyledons	Ericaceae	*Gaultheria*	*Gaultheria praticola*	JN044672
Eudicotyledons	Ericaceae	*Gaultheria*	*Gaultheria praticola*	JN044673
Eudicotyledons	Ericaceae	*Gaultheria*	*Gaultheria pseudonotabilis*	JN044674
Eudicotyledons	Ericaceae	*Gaultheria*	*Gaultheria pseudonotabilis*	JN044675
Eudicotyledons	Ericaceae	*Gaultheria*	*Gaultheria pseudonotabilis*	JN044676
Eudicotyledons	Ericaceae	*Gaultheria*	*Gaultheria pseudonotabilis*	JN044677
Eudicotyledons	Ericaceae	*Gaultheria*	*Gaultheria pyrolifolia*	JN044678
Eudicotyledons	Ericaceae	*Gaultheria*	*Gaultheria pyrolifolia*	JN044679
Eudicotyledons	Ericaceae	*Gaultheria*	*Gaultheria pyrolifolia*	JN044680
Eudicotyledons	Ericaceae	*Gaultheria*	*Gaultheria pyrolifolia*	JN044681
Eudicotyledons	Ericaceae	*Gaultheria*	*Gaultheria semi-infera*	JN044682
Eudicotyledons	Ericaceae	*Gaultheria*	*Gaultheria semi-infera*	JN044683
Eudicotyledons	Ericaceae	*Gaultheria*	*Gaultheria semi-infera*	JN044684
Eudicotyledons	Ericaceae	*Gaultheria*	*Gaultheria semi-infera*	JN044685
Eudicotyledons	Ericaceae	*Gaultheria*	*Gaultheria semi-infera*	JN044686
Eudicotyledons	Ericaceae	*Gaultheria*	*Gaultheria semi-infera*	JN044687
Eudicotyledons	Ericaceae	*Gaultheria*	*Gaultheria semi-infera*	JN044688
Eudicotyledons	Ericaceae	*Gaultheria*	*Gaultheria semi-infera*	JN044689
Eudicotyledons	Ericaceae	*Gaultheria*	*Gaultheria straminea*	JN044698

Eudicotyledons	Ericaceae	*Gaultheria*	*Gaultheria straminea*	JN044699
Eudicotyledons	Ericaceae	*Gaultheria*	*Gaultheria straminea*	JN044700
Eudicotyledons	Ericaceae	*Gaultheria*	*Gaultheria straminea*	JN044701
Eudicotyledons	Ericaceae	*Gaultheria*	*Gaultheria trichophylla*	JN044710
Eudicotyledons	Ericaceae	*Gaultheria*	*Gaultheria trichophylla*	JN044711
Eudicotyledons	Ericaceae	*Gaultheria*	*Gaultheria trichophylla*	JN044712
Eudicotyledons	Ericaceae	*Gaultheria*	*Gaultheria trichophylla*	JN044713
Eudicotyledons	Ericaceae	*Gaultheria*	*Gaultheria trichophylla*	JN044714
Eudicotyledons	Ericaceae	*Gaultheria*	*Gaultheria trichophylla*	JN044715
Eudicotyledons	Ericaceae	*Gaultheria*	*Gaultheria trichophylla*	JN044716
Eudicotyledons	Ericaceae	*Gaultheria*	*Gaultheria trichophylla*	JN044717
Eudicotyledons	Ericaceae	*Gaultheria*	*Gaultheria trigonoclada*	JN044718
Eudicotyledons	Ericaceae	*Gaultheria*	*Gaultheria trigonoclada*	JN044719
Eudicotyledons	Ericaceae	*Gaultheria*	*Gaultheria trigonoclada*	JN044720
Eudicotyledons	Ericaceae	*Gaultheria*	*Gaultheria trigonoclada*	JN044721
Eudicotyledons	Cactaceae	*Pfeiffera*	*Pfeiffera asuntapatensis*	FR716781
Eudicotyledons	Cactaceae	*Pfeiffera*	*Pfeiffera asuntapatensis*	FR716782
Eudicotyledons	Cactaceae	*Pfeiffera*	*Pfeiffera boliviana*	FR716783
Eudicotyledons	Cactaceae	*Pfeiffera*	*Pfeiffera boliviana*	FR716784
Eudicotyledons	Cactaceae	*Pfeiffera*	*Pfeiffera paranganiensis*	FR716787
Eudicotyledons	Cactaceae	*Pfeiffera*	*Pfeiffera paranganiensis*	FR716788
Eudicotyledons	Cactaceae	*Pfeiffera*	*Pfeiffera paranganiensis*	FR716789
Eudicotyledons	Fabaceae	*Calliandra*	*Calliandra haematocephala*	GU396788
Eudicotyledons	Fabaceae	*Calliandra*	*Calliandra haematocephala*	GU396789
Eudicotyledons	Asteraceae	*Tragopogon*	*Tragopogon x mirabilis*	HQ456292
Eudicotyledons	Asteraceae	*Tragopogon*	*Tragopogon x mirabilis*	HQ456293
Eudicotyledons	Oleaceae	*Ligustrum*	*Ligustrum delavayanum*	JF830294
Eudicotyledons	Oleaceae	*Ligustrum*	*Ligustrum delavayanum*	JF830295
Eudicotyledons	Oleaceae	*Ligustrum*	*Ligustrum delavayanum*	JF830296
Eudicotyledons	Oleaceae	*Ligustrum*	*Ligustrum delavayanum*	JF830297
Eudicotyledons	Vitaceae	*Ampelocissus*	*Ampelocissus africana*	JQ182504
Eudicotyledons	Vitaceae	*Ampelocissus*	*Ampelocissus africana*	JQ182507
Eudicotyledons	Vitaceae	*Cayratia*	*Cayratia geniculata*	JQ182532
Eudicotyledons	Vitaceae	*Cayratia*	*Cayratia geniculata*	HM585662
Eudicotyledons	Vitaceae	*Cissus*	*Cissus subtetragona*	HM585673
Eudicotyledons	Vitaceae	*Cissus*	*Cissus subtetragona*	JF437102
Eudicotyledons	Vitaceae	*Tetrastigma*	*Tetrastigma annamense*	HM585682
Eudicotyledons	Vitaceae	*Tetrastigma*	*Tetrastigma annamense*	HM585683
Eudicotyledons	Vitaceae	*Tetrastigma*	*Tetrastigma apiculatum*	HM585684
Eudicotyledons	Vitaceae	*Tetrastigma*	*Tetrastigma apiculatum*	HM585685
Eudicotyledons	Vitaceae	*Tetrastigma*	*Tetrastigma apiculatum*	HM585686
Eudicotyledons	Vitaceae	*Tetrastigma*	*Tetrastigma bioritsense*	HM585688
Eudicotyledons	Vitaceae	*Tetrastigma*	*Tetrastigma bioritsense*	JF437140
Eudicotyledons	Vitaceae	*Tetrastigma*	*Tetrastigma ceratopetalum*	HM585693
Eudicotyledons	Vitaceae	*Tetrastigma*	*Tetrastigma ceratopetalum*	HM585694
Eudicotyledons	Vitaceae	*Tetrastigma*	*Tetrastigma ceratopetalum*	HM585695
Eudicotyledons	Vitaceae	*Tetrastigma*	*Tetrastigma ceratopetalum*	HM585696
Eudicotyledons	Vitaceae	*Tetrastigma*	*Tetrastigma ceratopetalum*	HM585697
Eudicotyledons	Vitaceae	*Tetrastigma*	*Tetrastigma ceratopetalum*	HM585698
Eudicotyledons	Vitaceae	*Tetrastigma*	*Tetrastigma cruciatum*	HM585700
Eudicotyledons	Vitaceae	*Tetrastigma*	*Tetrastigma cruciatum*	HM585701
Eudicotyledons	Vitaceae	*Tetrastigma*	*Tetrastigma cruciatum*	HM585702
Eudicotyledons	Vitaceae	*Tetrastigma*	*Tetrastigma delavayi*	HM585704

Eudicotyledons	Vitaceae	*Tetrastigma*	*Tetrastigma delavayi*	HM585705
Eudicotyledons	Vitaceae	*Tetrastigma*	*Tetrastigma delavayi*	HM585706
Eudicotyledons	Vitaceae	*Tetrastigma*	*Tetrastigma erubescens*	HM585710
Eudicotyledons	Vitaceae	*Tetrastigma*	*Tetrastigma erubescens*	HM585711
Eudicotyledons	Vitaceae	*Tetrastigma*	*Tetrastigma erubescens*	HM585712
Eudicotyledons	Vitaceae	*Tetrastigma*	*Tetrastigma erubescens*	JF437141
Eudicotyledons	Vitaceae	*Tetrastigma*	*Tetrastigma funingense*	HM585714
Eudicotyledons	Vitaceae	*Tetrastigma*	*Tetrastigma funingense*	HM585715
Eudicotyledons	Vitaceae	*Tetrastigma*	*Tetrastigma garrettii*	HM585716
Eudicotyledons	Vitaceae	*Tetrastigma*	*Tetrastigma garrettii*	HM585717
Eudicotyledons	Vitaceae	*Tetrastigma*	*Tetrastigma garrettii*	HM585718
Eudicotyledons	Vitaceae	*Tetrastigma*	*Tetrastigma garrettii*	JF437142
Eudicotyledons	Vitaceae	*Tetrastigma*	*Tetrastigma gaudichaudianum*	HM585720
Eudicotyledons	Vitaceae	*Tetrastigma*	*Tetrastigma gaudichaudianum*	HM585721
Eudicotyledons	Vitaceae	*Tetrastigma*	*Tetrastigma hemsleyanum*	HM585722
Eudicotyledons	Vitaceae	*Tetrastigma*	*Tetrastigma hemsleyanum*	HM585723
Eudicotyledons	Vitaceae	*Tetrastigma*	*Tetrastigma hemsleyanum*	JF437143
Eudicotyledons	Vitaceae	*Tetrastigma*	*Tetrastigma jinghongense*	HM585728
Eudicotyledons	Vitaceae	*Tetrastigma*	*Tetrastigma jinghongense*	JF437144
Eudicotyledons	Vitaceae	*Tetrastigma*	*Tetrastigma laevigatum*	HM585729
Eudicotyledons	Vitaceae	*Tetrastigma*	*Tetrastigma laevigatum*	HM585730
Eudicotyledons	Vitaceae	*Tetrastigma*	*Tetrastigma lanyuense*	HM585731
Eudicotyledons	Vitaceae	*Tetrastigma*	*Tetrastigma lanyuense*	JF437145
Eudicotyledons	Vitaceae	*Tetrastigma*	*Tetrastigma laxum*	HM585738
Eudicotyledons	Vitaceae	*Tetrastigma*	*Tetrastigma laxum*	HM585739
Eudicotyledons	Vitaceae	*Tetrastigma*	*Tetrastigma laxum*	HM585740
Eudicotyledons	Vitaceae	*Tetrastigma*	*Tetrastigma lenticellatum*	HM585741
Eudicotyledons	Vitaceae	*Tetrastigma*	*Tetrastigma lenticellatum*	HM585742
Eudicotyledons	Vitaceae	*Tetrastigma*	*Tetrastigma napaulense*	HM585744
Eudicotyledons	Vitaceae	*Tetrastigma*	*Tetrastigma napaulense*	HM585745
Eudicotyledons	Vitaceae	*Tetrastigma*	*Tetrastigma pachyphyllum*	HM585752
Eudicotyledons	Vitaceae	*Tetrastigma*	*Tetrastigma pachyphyllum*	HM585753
Eudicotyledons	Vitaceae	*Tetrastigma*	*Tetrastigma pachyphyllum*	JF437147
Eudicotyledons	Vitaceae	*Tetrastigma*	*Tetrastigma pedunculare*	HM585755
Eudicotyledons	Vitaceae	*Tetrastigma*	*Tetrastigma pedunculare*	HM585756
Eudicotyledons	Vitaceae	*Tetrastigma*	*Tetrastigma pedunculare*	HM585757
Eudicotyledons	Vitaceae	*Tetrastigma*	*Tetrastigma planicaule*	HM585759
Eudicotyledons	Vitaceae	*Tetrastigma*	*Tetrastigma planicaule*	HQ656487
Eudicotyledons	Vitaceae	*Tetrastigma*	*Tetrastigma planicaule*	JF437148
Eudicotyledons	Vitaceae	*Tetrastigma*	*Tetrastigma pyriforme*	HM585760
Eudicotyledons	Vitaceae	*Tetrastigma*	*Tetrastigma pyriforme*	HM585761
Eudicotyledons	Vitaceae	*Tetrastigma*	*Tetrastigma tuberculatum*	HM585784
Eudicotyledons	Vitaceae	*Tetrastigma*	*Tetrastigma tuberculatum*	HM585785
Eudicotyledons	Ebenaceae	*Diospyros*	*Diospyros morrisiana*	HQ415533
Eudicotyledons	Ebenaceae	*Diospyros*	*Diospyros morrisiana*	HQ427083
Eudicotyledons	Oleaceae	*Ligustrum*	*Ligustrum confusum*	JF830276
Eudicotyledons	Oleaceae	*Ligustrum*	*Ligustrum confusum*	JF830277
Eudicotyledons	Oleaceae	*Ligustrum*	*Ligustrum confusum*	JF830278
Eudicotyledons	Oleaceae	*Ligustrum*	*Ligustrum expansum*	JF830279
Eudicotyledons	Oleaceae	*Ligustrum*	*Ligustrum expansum*	JF830280
Eudicotyledons	Oleaceae	*Ligustrum*	*Ligustrum expansum*	JF830281
Eudicotyledons	Oleaceae	*Ligustrum*	*Ligustrum gracile*	JF830316
Eudicotyledons	Oleaceae	*Ligustrum*	*Ligustrum gracile*	JF830317

Eudicotyledons	Oleaceae	*Ligustrum*	*Ligustrum gracile*	JF830318
Eudicotyledons	Oleaceae	*Ligustrum*	*Ligustrum henryi*	JF830261
Eudicotyledons	Oleaceae	*Ligustrum*	*Ligustrum henryi*	JF830262
Eudicotyledons	Oleaceae	*Ligustrum*	*Ligustrum henryi*	JF830263
Eudicotyledons	Oleaceae	*Ligustrum*	*Ligustrum henryi*	JF830264
Eudicotyledons	Oleaceae	*Ligustrum*	*Ligustrum henryi*	JF830265
Eudicotyledons	Oleaceae	*Ligustrum*	*Ligustrum henryi*	JF830266
Eudicotyledons	Oleaceae	*Ligustrum*	*Ligustrum henryi*	JN045220
Eudicotyledons	Oleaceae	*Ligustrum*	*Ligustrum henryi*	JN045221
Eudicotyledons	Oleaceae	*Ligustrum*	*Ligustrum henryi*	JN045222
Eudicotyledons	Oleaceae	*Ligustrum*	*Ligustrum henryi*	JN045223
Eudicotyledons	Oleaceae	*Ligustrum*	*Ligustrum henryi*	JN045224
Eudicotyledons	Oleaceae	*Ligustrum*	*Ligustrum robustum*	JN045236
Eudicotyledons	Oleaceae	*Ligustrum*	*Ligustrum robustum*	JN045237
Eudicotyledons	Oleaceae	*Ligustrum*	*Ligustrum robustum*	JN045238
Eudicotyledons	Oleaceae	*Ligustrum*	*Ligustrum robustum*	JN045239
Eudicotyledons	Oleaceae	*Ligustrum*	*Ligustrum robustum*	JN045240
Eudicotyledons	Oleaceae	*Ligustrum*	*Ligustrum strongylophyllum*	JF830267
Eudicotyledons	Oleaceae	*Ligustrum*	*Ligustrum strongylophyllum*	JF830268
Eudicotyledons	Oleaceae	*Ligustrum*	*Ligustrum strongylophyllum*	JF830269
Eudicotyledons	Oleaceae	*Ligustrum*	*Ligustrum strongylophyllum*	JN045244
Eudicotyledons	Oleaceae	*Ligustrum*	*Ligustrum strongylophyllum*	JN045245
Eudicotyledons	Oleaceae	*Ligustrum*	*Ligustrum xingrenense*	JF830291
Eudicotyledons	Oleaceae	*Ligustrum*	*Ligustrum xingrenense*	JF830292
Eudicotyledons	Oleaceae	*Ligustrum*	*Ligustrum xingrenense*	JF830293
Eudicotyledons	Fabaceae	*Anthyllis*	*Anthyllis hystrix*	HM468244
Eudicotyledons	Fabaceae	*Anthyllis*	*Anthyllis hystrix*	HQ646219
Eudicotyledons	Oleaceae	*Ligustrum*	*Ligustrum pricei*	JN045230
Eudicotyledons	Oleaceae	*Ligustrum*	*Ligustrum pricei*	JN045231
Eudicotyledons	Oleaceae	*Ligustrum*	*Ligustrum pricei*	JN045232
Eudicotyledons	Oleaceae	*Ligustrum*	*Ligustrum pricei*	JN045233
Eudicotyledons	Primulaceae	*Omphalogramma*	*Omphalogramma elegans*	JN045604
Eudicotyledons	Primulaceae	*Omphalogramma*	*Omphalogramma elegans*	JN045605
Eudicotyledons	Primulaceae	*Omphalogramma*	*Omphalogramma elegans*	JN045606
Eudicotyledons	Primulaceae	*Omphalogramma*	*Omphalogramma elegans*	JN045607
Eudicotyledons	Primulaceae	*Omphalogramma*	*Omphalogramma elegans*	JN045608
Eudicotyledons	Primulaceae	*Omphalogramma*	*Omphalogramma forrestii*	JN045609
Eudicotyledons	Primulaceae	*Omphalogramma*	*Omphalogramma forrestii*	JN045610
Eudicotyledons	Primulaceae	*Omphalogramma*	*Omphalogramma forrestii*	JN045611
Eudicotyledons	Primulaceae	*Omphalogramma*	*Omphalogramma forrestii*	JN045612
Eudicotyledons	Primulaceae	*Omphalogramma*	*Omphalogramma forrestii*	JN045613
Eudicotyledons	Primulaceae	*Omphalogramma*	*Omphalogramma minus*	JN045614
Eudicotyledons	Primulaceae	*Omphalogramma*	*Omphalogramma minus*	JN045615
Eudicotyledons	Primulaceae	*Omphalogramma*	*Omphalogramma minus*	JN045616
Eudicotyledons	Primulaceae	*Omphalogramma*	*Omphalogramma minus*	JN045617
Eudicotyledons	Primulaceae	*Omphalogramma*	*Omphalogramma minus*	JN045618
Eudicotyledons	Primulaceae	*Omphalogramma*	*Omphalogramma tibeticum*	JN045624
Eudicotyledons	Primulaceae	*Omphalogramma*	*Omphalogramma tibeticum*	JN045625
Eudicotyledons	Primulaceae	*Omphalogramma*	*Omphalogramma tibeticum*	JN045626
Eudicotyledons	Primulaceae	*Omphalogramma*	*Omphalogramma tibeticum*	JN045627
Eudicotyledons	Rosaceae	*Pyracantha*	*Pyracantha crenulata*	JN046706
Eudicotyledons	Rosaceae	*Pyracantha*	*Pyracantha crenulata*	JN046707
Eudicotyledons	Rosaceae	*Pyracantha*	*Pyracantha crenulata*	JN046708

Eudicotyledons	Fagaceae	*Castanopsis*	*Castanopsis chunii*	JN044198
Eudicotyledons	Fagaceae	*Castanopsis*	*Castanopsis chunii*	JN044199
Eudicotyledons	Fagaceae	*Castanopsis*	*Castanopsis chunii*	JN044200
Eudicotyledons	Fagaceae	*Castanopsis*	*Castanopsis lamontii*	JN044214
Eudicotyledons	Fagaceae	*Castanopsis*	*Castanopsis lamontii*	JN044215
Eudicotyledons	Fagaceae	*Castanopsis*	*Castanopsis lamontii*	JN044216
Eudicotyledons	Fagaceae	*Castanopsis*	*Castanopsis lamontii*	JN044217
Eudicotyledons	Fagaceae	*Castanopsis*	*Castanopsis lamontii*	JN044218
Eudicotyledons	Fagaceae	*Castanopsis*	*Castanopsis megaphylla*	JN044219
Eudicotyledons	Fagaceae	*Castanopsis*	*Castanopsis megaphylla*	JN044220
Eudicotyledons	Fagaceae	*Castanopsis*	*Castanopsis orthacantha*	JN044221
Eudicotyledons	Fagaceae	*Castanopsis*	*Castanopsis orthacantha*	JN044222
Eudicotyledons	Fagaceae	*Castanopsis*	*Castanopsis orthacantha*	JN044223
Eudicotyledons	Fagaceae	*Castanopsis*	*Castanopsis platyacantha*	JN044224
Eudicotyledons	Fagaceae	*Castanopsis*	*Castanopsis platyacantha*	JN044225
Eudicotyledons	Fagaceae	*Castanopsis*	*Castanopsis platyacantha*	JN044226
Eudicotyledons	Fagaceae	*Castanopsis*	*Castanopsis platyacantha*	JN044227
Eudicotyledons	Fagaceae	*Castanopsis*	*Castanopsis remotidenticulata*	JN044228
Eudicotyledons	Fagaceae	*Castanopsis*	*Castanopsis remotidenticulata*	JN044229
Eudicotyledons	Fagaceae	*Castanopsis*	*Castanopsis remotidenticulata*	JN044230
Eudicotyledons	Campanulaceae	*Cyananthus*	*Cyananthus dolichosceles*	JN044331
Eudicotyledons	Campanulaceae	*Cyananthus*	*Cyananthus dolichosceles*	JN044332
Eudicotyledons	Campanulaceae	*Cyananthus*	*Cyananthus dolichosceles*	JN044333
Eudicotyledons	Campanulaceae	*Cyananthus*	*Cyananthus dolichosceles*	JN044334
Eudicotyledons	Campanulaceae	*Cyananthus*	*Cyananthus formosus*	JN044335
Eudicotyledons	Campanulaceae	*Cyananthus*	*Cyananthus formosus*	JN044336
Eudicotyledons	Campanulaceae	*Cyananthus*	*Cyananthus hookeri*	JN044337
Eudicotyledons	Campanulaceae	*Cyananthus*	*Cyananthus hookeri*	JN044338
Eudicotyledons	Campanulaceae	*Cyananthus*	*Cyananthus hookeri*	JN044339
Eudicotyledons	Campanulaceae	*Cyananthus*	*Cyananthus hookeri*	JN044340
Eudicotyledons	Campanulaceae	*Cyananthus*	*Cyananthus incanus*	JN044341
Eudicotyledons	Campanulaceae	*Cyananthus*	*Cyananthus incanus*	JN044342
Eudicotyledons	Campanulaceae	*Cyananthus*	*Cyananthus incanus*	JN044343
Eudicotyledons	Campanulaceae	*Cyananthus*	*Cyananthus incanus*	JN044344
Eudicotyledons	Campanulaceae	*Cyananthus*	*Cyananthus incanus*	JN044345
Eudicotyledons	Campanulaceae	*Cyananthus*	*Cyananthus inflatus*	JN044346
Eudicotyledons	Campanulaceae	*Cyananthus*	*Cyananthus inflatus*	JN044347
Eudicotyledons	Campanulaceae	*Cyananthus*	*Cyananthus inflatus*	JN044348
Eudicotyledons	Campanulaceae	*Cyananthus*	*Cyananthus inflatus*	JN044349
Eudicotyledons	Campanulaceae	*Cyananthus*	*Cyananthus inflatus*	JN044350
Eudicotyledons	Campanulaceae	*Cyananthus*	*Cyananthus lichiangensis*	JN044351
Eudicotyledons	Campanulaceae	*Cyananthus*	*Cyananthus lichiangensis*	JN044352
Eudicotyledons	Campanulaceae	*Cyananthus*	*Cyananthus lichiangensis*	JN044353
Eudicotyledons	Campanulaceae	*Cyananthus*	*Cyananthus lichiangensis*	JN044354
Eudicotyledons	Onagraceae	*Epilobium*	*Epilobium cylindricum*	JN129838
Eudicotyledons	Onagraceae	*Epilobium*	*Epilobium cylindricum*	JN129839
Eudicotyledons	Ericaceae	*Gaultheria*	*Gaultheria suborbicularis*	JN044702
Eudicotyledons	Ericaceae	*Gaultheria*	*Gaultheria suborbicularis*	JN044703
Eudicotyledons	Ericaceae	*Gaultheria*	*Gaultheria suborbicularis*	JN044704
Eudicotyledons	Ericaceae	*Gaultheria*	*Gaultheria suborbicularis*	JN044705
Eudicotyledons	Ericaceae	*Gaultheria*	*Gaultheria tetramera*	JN044706
Eudicotyledons	Ericaceae	*Gaultheria*	*Gaultheria tetramera*	JN044707
Eudicotyledons	Ericaceae	*Gaultheria*	*Gaultheria tetramera*	JN044708

Eudicotyledons	Ericaceae	*Gaultheria*	*Gaultheria tetramera*	JN044709
Eudicotyledons	Vitaceae	*Cayratia*	*Cayratia debilis*	HQ656482
Eudicotyledons	Vitaceae	*Cayratia*	*Cayratia debilis*	JF437075
Eudicotyledons	Vitaceae	*Cayratia*	*Cayratia debilis*	JF437076
Eudicotyledons	Asteraceae	*Mikania*	*Mikania laevigata*	JN052778
Eudicotyledons	Asteraceae	*Mikania*	*Mikania laevigata*	JN052779
Eudicotyledons	Asteraceae	*Mikania*	*Mikania laevigata*	JN052780
Eudicotyledons	Asteraceae	*Mikania*	*Mikania laevigata*	JN052781
Eudicotyledons	Asteraceae	*Mikania*	*Mikania laevigata*	JN052782
Eudicotyledons	Asteraceae	*Mikania*	*Mikania laevigata*	JN052783
Eudicotyledons	Vitaceae	*Ampelocissus*	*Ampelocissus erdvendbergiana*	JQ182527
Eudicotyledons	Vitaceae	*Ampelocissus*	*Ampelocissus erdvendbergiana*	JF437059
Eudicotyledons	Vitaceae	*Ampelopsis*	*Ampelopsis denudata*	JQ182534
Eudicotyledons	Vitaceae	*Ampelopsis*	*Ampelopsis denudata*	JF437066
Eudicotyledons	Vitaceae	*Cissus*	*Cissus assamica*	JF437089
Eudicotyledons	Vitaceae	*Cissus*	*Cissus assamica*	JF437090
Eudicotyledons	Asteraceae	*Mikania*	*Mikania glomerata*	JN052774
Eudicotyledons	Asteraceae	*Mikania*	*Mikania glomerata*	JN052775
Eudicotyledons	Asteraceae	*Mikania*	*Mikania glomerata*	JN052776
Eudicotyledons	Asteraceae	*Mikania*	*Mikania glomerata*	JN052777
Eudicotyledons	Asteraceae	*Achillea*	*Achillea inundata*	HQ451042
Eudicotyledons	Asteraceae	*Achillea*	*Achillea inundata*	HQ451043
Eudicotyledons	Rutaceae	*Citrus*	*Citrus limon*	GQ435450
Eudicotyledons	Rutaceae	*Citrus*	*Citrus limon*	JN315362
Eudicotyledons	Rutaceae	*Citrus*	*Citrus limon*	JN315363
Eudicotyledons	Ranunculaceae	*Ranunculus*	*Ranunculus acris*	FJ395535
Eudicotyledons	Ranunculaceae	*Ranunculus*	*Ranunculus acris*	HQ596809
Eudicotyledons	Moraceae	*Ficus*	*Ficus carica*	GU935094
Eudicotyledons	Moraceae	*Ficus*	*Ficus carica*	GU935095
Eudicotyledons	Betulaceae	*Betula*	*Betula pendula*	GQ248254
Eudicotyledons	Betulaceae	*Betula*	*Betula pendula*	JN247424
Eudicotyledons	Betulaceae	*Betula*	*Betula pendula*	FJ011881
Eudicotyledons	Betulaceae	*Betula*	*Betula papyrifera*	FJ011880
Eudicotyledons	Betulaceae	*Betula*	*Betula papyrifera*	EU750443
Eudicotyledons	Betulaceae	*Betula*	*Betula papyrifera*	EU750444
Eudicotyledons	Betulaceae	*Betula*	*Betula papyrifera*	EU750445
Eudicotyledons	Fagaceae	*Quercus*	*Quercus rubra*	HQ596807
Eudicotyledons	Fagaceae	*Quercus*	*Quercus rubra*	EU750513
Eudicotyledons	Fagaceae	*Quercus*	*Quercus rubra*	EU750514
Eudicotyledons	Fagaceae	*Quercus*	*Quercus rubra*	EU750515
Eudicotyledons	Fagaceae	*Quercus*	*Quercus alba*	EU750506
Eudicotyledons	Fagaceae	*Quercus*	*Quercus alba*	EU750507
Eudicotyledons	Fagaceae	*Quercus*	*Quercus alba*	EU750508
Eudicotyledons	Fagaceae	*Quercus*	*Quercus alba*	EU750509
Eudicotyledons	Betulaceae	*Alnus*	*Alnus incana*	FJ011870
Eudicotyledons	Betulaceae	*Alnus*	*Alnus incana*	FJ844532
Eudicotyledons	Betulaceae	*Alnus*	*Alnus incana*	FJ844533
Eudicotyledons	Betulaceae	*Alnus*	*Alnus incana*	FJ844534
Eudicotyledons	Betulaceae	*Alnus*	*Alnus glutinosa*	FJ011868
Eudicotyledons	Betulaceae	*Alnus*	*Alnus glutinosa*	FJ844535
Eudicotyledons	Betulaceae	*Alnus*	*Alnus glutinosa*	FJ844536
Eudicotyledons	Betulaceae	*Alnus*	*Alnus glutinosa*	FJ844537
Eudicotyledons	Betulaceae	*Alnus*	*Alnus glutinosa*	FJ844538

Eudicotyledons	Betulaceae	*Alnus*	*Alnus glutinosa*	FJ844539
Eudicotyledons	Onagraceae	*Chamerion*	*Chamerion angustifolium*	JN044476
Eudicotyledons	Onagraceae	*Chamerion*	*Chamerion angustifolium*	JN044477
Eudicotyledons	Onagraceae	*Chamerion*	*Chamerion angustifolium*	JN129841
Eudicotyledons	Onagraceae	*Chamerion*	*Chamerion angustifolium*	JN129842
Eudicotyledons	Onagraceae	*Chamerion*	*Chamerion angustifolium*	JN129843
Eudicotyledons	Fabaceae	*Schotia*	*Schotia brachypetala*	GQ405092
Eudicotyledons	Fabaceae	*Schotia*	*Schotia brachypetala*	GQ405098
Eudicotyledons	Fabaceae	*Schotia*	*Schotia brachypetala*	GQ405109
Eudicotyledons	Fabaceae	*Schotia*	*Schotia brachypetala*	GQ405121
Eudicotyledons	Fabaceae	*Schotia*	*Schotia brachypetala*	GQ405131
Eudicotyledons	Fabaceae	*Schotia*	*Schotia brachypetala*	GQ405132
Eudicotyledons	Fabaceae	*Astragalus*	*Astragalus adsurgens*	GU396749
Eudicotyledons	Fabaceae	*Astragalus*	*Astragalus adsurgens*	GU396750
Eudicotyledons	Fabaceae	*Astragalus*	*Astragalus adsurgens*	GU396751
Eudicotyledons	Betulaceae	*Betula*	*Betula alleghaniensis*	EU750441
Eudicotyledons	Betulaceae	*Betula*	*Betula alleghaniensis*	EU750442
Eudicotyledons	Hydrangeaceae	*Hydrangea*	*Hydrangea macrophylla*	GQ248319
Eudicotyledons	Hydrangeaceae	*Hydrangea*	*Hydrangea macrophylla*	EF590702
Eudicotyledons	Hydrangeaceae	*Hydrangea*	*Hydrangea macrophylla*	FN675801
Eudicotyledons	Hydrangeaceae	*Hydrangea*	*Hydrangea macrophylla*	HM216983
Eudicotyledons	Hydrangeaceae	*Hydrangea*	*Hydrangea macrophylla*	JF321258
Eudicotyledons	Lamiaceae	*Glechoma*	*Glechoma hederacea*	DQ667355
Eudicotyledons	Lamiaceae	*Glechoma*	*Glechoma hederacea*	FJ395524
Eudicotyledons	Lamiaceae	*Glechoma*	*Glechoma hederacea*	HQ596719
Eudicotyledons	Lamiaceae	*Glechoma*	*Glechoma hederacea*	DQ006196
Eudicotyledons	Lamiaceae	*Salvia*	*Salvia divinorum*	DQ667359
Eudicotyledons	Lamiaceae	*Salvia*	*Salvia divinorum*	HQ418913
Eudicotyledons	Loganiaceae	*Strychnos*	*Strychnos nux-vomica*	GQ435195
Eudicotyledons	Loganiaceae	*Strychnos*	*Strychnos nux-vomica*	GQ435196
Eudicotyledons	Loganiaceae	*Strychnos*	*Strychnos nux-vomica*	GQ435197
Eudicotyledons	Loganiaceae	*Strychnos*	*Strychnos nux-vomica*	GQ435198
Eudicotyledons	Nothofagaceae	*Nothofagus*	*Nothofagus antarctica*	GQ863325
Eudicotyledons	Nothofagaceae	*Nothofagus*	*Nothofagus antarctica*	GQ863328
Eudicotyledons	Nothofagaceae	*Nothofagus*	*Nothofagus antarctica*	GQ863336
Eudicotyledons	Nothofagaceae	*Nothofagus*	*Nothofagus antarctica*	GQ863339
Eudicotyledons	Nothofagaceae	*Nothofagus*	*Nothofagus antarctica*	GQ863341
Eudicotyledons	Nothofagaceae	*Nothofagus*	*Nothofagus antarctica*	GQ863342
Eudicotyledons	Nothofagaceae	*Nothofagus*	*Nothofagus antarctica*	GQ863346
Eudicotyledons	Nothofagaceae	*Nothofagus*	*Nothofagus antarctica*	GQ863348
Eudicotyledons	Nothofagaceae	*Nothofagus*	*Nothofagus antarctica*	GQ863349
Eudicotyledons	Nothofagaceae	*Nothofagus*	*Nothofagus antarctica*	GQ863351
Eudicotyledons	Nothofagaceae	*Nothofagus*	*Nothofagus betuloides*	GQ863332
Eudicotyledons	Nothofagaceae	*Nothofagus*	*Nothofagus betuloides*	GQ863337
Eudicotyledons	Nothofagaceae	*Nothofagus*	*Nothofagus betuloides*	GQ863343
Eudicotyledons	Nothofagaceae	*Nothofagus*	*Nothofagus betuloides*	GQ863350
Eudicotyledons	Nothofagaceae	*Nothofagus*	*Nothofagus betuloides*	GQ863352
Eudicotyledons	Nothofagaceae	*Nothofagus*	*Nothofagus cunninghamii*	JN247426
Eudicotyledons	Nothofagaceae	*Nothofagus*	*Nothofagus cunninghamii*	GQ863377
Eudicotyledons	Nothofagaceae	*Nothofagus*	*Nothofagus dombeyi*	GQ863326
Eudicotyledons	Nothofagaceae	*Nothofagus*	*Nothofagus dombeyi*	GQ863329
Eudicotyledons	Nothofagaceae	*Nothofagus*	*Nothofagus dombeyi*	GQ863333
Eudicotyledons	Nothofagaceae	*Nothofagus*	*Nothofagus dombeyi*	GQ863338

Eudicotyledons	Nothofagaceae	*Nothofagus*	*Nothofagus dombeyi*	GQ863340
Eudicotyledons	Nothofagaceae	*Nothofagus*	*Nothofagus dombeyi*	GQ863344
Eudicotyledons	Nothofagaceae	*Nothofagus*	*Nothofagus dombeyi*	GQ863347
Eudicotyledons	Nothofagaceae	*Nothofagus*	*Nothofagus nitida*	GQ863334
Eudicotyledons	Nothofagaceae	*Nothofagus*	*Nothofagus nitida*	GQ863345
Eudicotyledons	Nothofagaceae	*Nothofagus*	*Nothofagus obliqua*	GQ863331
Eudicotyledons	Nothofagaceae	*Nothofagus*	*Nothofagus obliqua*	GQ863335
Eudicotyledons	Nothofagaceae	*Nothofagus*	*Nothofagus pumilio*	GQ863388
Eudicotyledons	Nothofagaceae	*Nothofagus*	*Nothofagus pumilio*	GQ863389
Eudicotyledons	Nothofagaceae	*Nothofagus*	*Nothofagus pumilio*	GQ863390
Eudicotyledons	Nothofagaceae	*Nothofagus*	*Nothofagus pumilio*	GQ863391
Eudicotyledons	Nothofagaceae	*Nothofagus*	*Nothofagus pumilio*	GQ863392
Eudicotyledons	Nothofagaceae	*Nothofagus*	*Nothofagus pumilio*	GQ863393
Eudicotyledons	Nothofagaceae	*Nothofagus*	*Nothofagus pumilio*	GQ863394
Eudicotyledons	Nothofagaceae	*Nothofagus*	*Nothofagus pumilio*	GQ863395
Eudicotyledons	Nothofagaceae	*Nothofagus*	*Nothofagus pumilio*	GQ863396
Eudicotyledons	Nothofagaceae	*Nothofagus*	*Nothofagus pumilio*	GU152878
Eudicotyledons	Nothofagaceae	*Nothofagus*	*Nothofagus pumilio*	GU152879
Eudicotyledons	Nothofagaceae	*Nothofagus*	*Nothofagus pumilio*	GU152880
Eudicotyledons	Nothofagaceae	*Nothofagus*	*Nothofagus pumilio*	GU152881
Eudicotyledons	Nothofagaceae	*Nothofagus*	*Nothofagus pumilio*	GU152882
Eudicotyledons	Nothofagaceae	*Nothofagus*	*Nothofagus pumilio*	GU152883
Eudicotyledons	Nothofagaceae	*Nothofagus*	*Nothofagus pumilio*	GU152884
Eudicotyledons	Nothofagaceae	*Nothofagus*	*Nothofagus pumilio*	GU152885
Eudicotyledons	Lamiaceae	*Mentha*	*Mentha spicata*	DQ667354
Eudicotyledons	Lamiaceae	*Mentha*	*Mentha spicata*	EU585744
Eudicotyledons	Lamiaceae	*Mentha*	*Mentha spicata*	EU627573
Eudicotyledons	Lamiaceae	*Mentha*	*Mentha spicata*	EU627578
Eudicotyledons	Lamiaceae	*Mentha*	*Mentha spicata*	FR726101
Eudicotyledons	Lamiaceae	*Mentha*	*Mentha spicata*	FR726102
Eudicotyledons	Lamiaceae	*Mentha*	*Mentha spicata*	FJ513095
Eudicotyledons	Lamiaceae	*Mentha*	*Mentha spicata*	AY643666
Eudicotyledons	Lamiaceae	*Mentha*	*Mentha spicata*	AY643667
Eudicotyledons	Lamiaceae	*Mentha*	*Mentha spicata*	AY643668
Eudicotyledons	Lamiaceae	*Mentha*	*Mentha spicata*	AY643669
Eudicotyledons	Lamiaceae	*Mentha*	*Mentha spicata*	AY643670
Eudicotyledons	Lamiaceae	*Mentha*	*Mentha spicata*	AY643671
Eudicotyledons	Lamiaceae	*Mentha*	*Mentha spicata*	AY643672
Eudicotyledons	Lamiaceae	*Mentha*	*Mentha spicata*	AY643673
Eudicotyledons	Lamiaceae	*Mentha*	*Mentha spicata*	AY643674
Eudicotyledons	Lamiaceae	*Mentha*	*Mentha spicata*	AY643675
Eudicotyledons	Lamiaceae	*Mentha*	*Mentha spicata*	AY643676
Eudicotyledons	Lamiaceae	*Mentha*	*Mentha spicata*	AY643677
Eudicotyledons	Lamiaceae	*Mentha*	*Mentha spicata*	AY643678
Eudicotyledons	Lamiaceae	*Mentha*	*Mentha spicata*	HQ902826
Eudicotyledons	Lamiaceae	*Mentha*	*Mentha spicata*	HQ902827
Eudicotyledons	Lamiaceae	*Mentha*	*Mentha spicata*	HQ902854
Eudicotyledons	Lamiaceae	*Mentha*	*Mentha spicata*	HQ902865
Eudicotyledons	Rubiaceae	*Galium*	*Galium aparine*	HQ596706
Eudicotyledons	Rubiaceae	*Galium*	*Galium aparine*	HQ596707
Eudicotyledons	Rubiaceae	*Galium*	*Galium aparine*	DQ006223
Eudicotyledons	Plantaginaceae	*Plantago*	*Plantago major*	GQ248370
Eudicotyledons	Plantaginaceae	*Plantago*	*Plantago major*	JN407021

Eudicotyledons	Plantaginaceae	*Plantago*	*Plantago major*	JN407022
Eudicotyledons	Plantaginaceae	*Plantago*	*Plantago major*	JN407023
Eudicotyledons	Plantaginaceae	*Plantago*	*Plantago major*	JN407024
Eudicotyledons	Plantaginaceae	*Plantago*	*Plantago major*	JN407025
Eudicotyledons	Plantaginaceae	*Plantago*	*Plantago major*	EU750474
Eudicotyledons	Plantaginaceae	*Plantago*	*Plantago major*	EU750475
Eudicotyledons	Plantaginaceae	*Plantago*	*Plantago major*	EU750476
Eudicotyledons	Rosaceae	*Amelanchier*	*Amelanchier alnifolia*	JQ390632
Eudicotyledons	Rosaceae	*Amelanchier*	*Amelanchier alnifolia*	JQ390643
Eudicotyledons	Rosaceae	*Rubus*	*Rubus idaeus*	EU750527
Eudicotyledons	Rosaceae	*Rubus*	*Rubus idaeus*	EU750528
Eudicotyledons	Rosaceae	*Rubus*	*Rubus idaeus*	EU750529
Eudicotyledons	Rosaceae	*Rubus*	*Rubus idaeus*	EU750530
Eudicotyledons	Rosaceae	*Rubus*	*Rubus idaeus*	EU750531
Eudicotyledons	Rosaceae	*Sorbaria*	*Sorbaria arborea*	JN047232
Eudicotyledons	Rosaceae	*Sorbaria*	*Sorbaria arborea*	JN047233
Eudicotyledons	Rosaceae	*Sorbaria*	*Sorbaria arborea*	JN047234
Eudicotyledons	Rosaceae	*Sorbaria*	*Sorbaria arborea*	JN047235
Eudicotyledons	Solanaceae	*Mandragora*	*Mandragora officinarum*	HE659544
Eudicotyledons	Solanaceae	*Mandragora*	*Mandragora officinarum*	HQ216173
Eudicotyledons	Solanaceae	*Mandragora*	*Mandragora officinarum*	HQ216174
Eudicotyledons	Solanaceae	*Mandragora*	*Mandragora officinarum*	HQ216175
Eudicotyledons	Solanaceae	*Mandragora*	*Mandragora officinarum*	HQ216176
Eudicotyledons	Solanaceae	*Nolana*	*Nolana spathulata*	EU742495
Eudicotyledons	Solanaceae	*Nolana*	*Nolana spathulata*	FJ914073
Eudicotyledons	Papaveraceae	*Papaver*	*Papaver rhoeas*	GQ248360
Eudicotyledons	Papaveraceae	*Papaver*	*Papaver rhoeas*	JN584665
Eudicotyledons	Papaveraceae	*Papaver*	*Papaver rhoeas*	EF590721
Eudicotyledons	Lamiaceae	*Mentha*	*Mentha x piperita*	FR726096
Eudicotyledons	Lamiaceae	*Mentha*	*Mentha x piperita*	FR726097
Eudicotyledons	Lamiaceae	*Mentha*	*Mentha x piperita*	FR726098
Eudicotyledons	Lamiaceae	*Mentha*	*Mentha x piperita*	AY643698
Eudicotyledons	Lamiaceae	*Mentha*	*Mentha x piperita*	AY643699
Eudicotyledons	Lamiaceae	*Mentha*	*Mentha x piperita*	AY643700
Eudicotyledons	Lamiaceae	*Mentha*	*Mentha x piperita*	AY643701
Eudicotyledons	Lamiaceae	*Mentha*	*Mentha x piperita*	AY643702
Eudicotyledons	Lamiaceae	*Mentha*	*Mentha x piperita*	AY643703
Eudicotyledons	Lamiaceae	*Mentha*	*Mentha x piperita*	AY643704
Eudicotyledons	Lamiaceae	*Mentha*	*Mentha x piperita*	AY643705
Eudicotyledons	Lamiaceae	*Mentha*	*Mentha x piperita*	AY643706
Eudicotyledons	Lamiaceae	*Mentha*	*Mentha x piperita*	AY643707
Eudicotyledons	Rosaceae	*Amelanchier*	*Amelanchier bartramiana*	JQ390641
Eudicotyledons	Rosaceae	*Amelanchier*	*Amelanchier bartramiana*	EU500285
Eudicotyledons	Rosaceae	*Crataegus*	*Crataegus mollis*	EF127122
Eudicotyledons	Rosaceae	*Crataegus*	*Crataegus mollis*	GU562402
Eudicotyledons	Rosaceae	*Crataegus*	*Crataegus mollis*	HQ596656
Eudicotyledons	Rosaceae	*Crataegus*	*Crataegus mollis*	HQ596657
Eudicotyledons	Rosaceae	*Crataegus*	*Crataegus mollis*	HQ596658
Eudicotyledons	Rosaceae	*Aronia*	*Aronia arbutifolia*	JQ390630
Eudicotyledons	Rosaceae	*Aronia*	*Aronia arbutifolia*	GQ305339
Eudicotyledons	Rutaceae	*Citrus*	*Citrus maxima*	GQ267053
Eudicotyledons	Rutaceae	*Citrus*	*Citrus maxima*	GQ267054
Eudicotyledons	Rutaceae	*Citrus*	*Citrus maxima*	GQ267055

Eudicotyledons	Rutaceae	*Citrus*	*Citrus maxima*	GQ267056
Eudicotyledons	Rutaceae	*Citrus*	*Citrus maxima*	GQ267057
Eudicotyledons	Rutaceae	*Citrus*	*Citrus maxima*	GQ267058
Eudicotyledons	Rutaceae	*Citrus*	*Citrus maxima*	GQ267059
Eudicotyledons	Rutaceae	*Citrus*	*Citrus maxima*	GQ267060
Eudicotyledons	Rutaceae	*Citrus*	*Citrus maxima*	GQ267061
Eudicotyledons	Rutaceae	*Citrus*	*Citrus maxima*	GQ267065
Eudicotyledons	Rutaceae	*Citrus*	*Citrus maxima*	GQ435433
Eudicotyledons	Rutaceae	*Citrus*	*Citrus maxima*	GQ435434
Eudicotyledons	Rutaceae	*Citrus*	*Citrus maxima*	GQ435442
Eudicotyledons	Rutaceae	*Citrus*	*Citrus maxima*	GQ435443
Eudicotyledons	Rutaceae	*Citrus*	*Citrus maxima*	GQ435444
Eudicotyledons	Rutaceae	*Citrus*	*Citrus maxima*	GQ435445
Eudicotyledons	Rutaceae	*Citrus*	*Citrus maxima*	GQ435446
Eudicotyledons	Rutaceae	*Citrus*	*Citrus maxima*	JN315364
Eudicotyledons	Rutaceae	*Citrus*	*Citrus maxima*	JN315365
Eudicotyledons	Rutaceae	*Citrus*	*Citrus x paradisi*	JN315366
Eudicotyledons	Rutaceae	*Citrus*	*Citrus x paradisi*	JN315367
Eudicotyledons	Rutaceae	*Citrus*	*Citrus x paradisi*	HM446909
Eudicotyledons	Betulaceae	*Alnus*	*Alnus alnobetula*	FJ844483
Eudicotyledons	Betulaceae	*Alnus*	*Alnus alnobetula*	FJ844484
Eudicotyledons	Betulaceae	*Alnus*	*Alnus alnobetula*	FJ844487
Eudicotyledons	Betulaceae	*Alnus*	*Alnus alnobetula*	FJ844488
Eudicotyledons	Ranunculaceae	*Coptis*	*Coptis trifolia*	AB159539
Eudicotyledons	Ranunculaceae	*Coptis*	*Coptis trifolia*	AB159541
Eudicotyledons	Lamiaceae	*Mentha*	*Mentha longifolia*	AY643679
Eudicotyledons	Lamiaceae	*Mentha*	*Mentha longifolia*	AY643680
Eudicotyledons	Lamiaceae	*Mentha*	*Mentha longifolia*	AY643681
Eudicotyledons	Lamiaceae	*Mentha*	*Mentha longifolia*	AY643682
Eudicotyledons	Lamiaceae	*Mentha*	*Mentha longifolia*	AY643683
Eudicotyledons	Lamiaceae	*Mentha*	*Mentha longifolia*	AY643684
Eudicotyledons	Lamiaceae	*Mentha*	*Mentha longifolia*	AY643685
Eudicotyledons	Lamiaceae	*Mentha*	*Mentha longifolia*	AY643686
Eudicotyledons	Lamiaceae	*Mentha*	*Mentha longifolia*	HQ902825
Eudicotyledons	Lamiaceae	*Mentha*	*Mentha suaveolens*	AY643687
Eudicotyledons	Lamiaceae	*Mentha*	*Mentha suaveolens*	AY643688
Eudicotyledons	Lamiaceae	*Mentha*	*Mentha suaveolens*	AY643689
Eudicotyledons	Lamiaceae	*Mentha*	*Mentha suaveolens*	AY643690
Eudicotyledons	Lamiaceae	*Mentha*	*Mentha suaveolens*	AY643691
Eudicotyledons	Lamiaceae	*Mentha*	*Mentha suaveolens*	AY643692
Eudicotyledons	Lamiaceae	*Mentha*	*Mentha suaveolens*	AY643693
Eudicotyledons	Lamiaceae	*Mentha*	*Mentha suaveolens*	AY643694
Eudicotyledons	Lamiaceae	*Mentha*	*Mentha suaveolens*	AY643695
Eudicotyledons	Lamiaceae	*Mentha*	*Mentha suaveolens*	AY643696
Eudicotyledons	Lamiaceae	*Mentha*	*Mentha suaveolens*	AY643697
Eudicotyledons	Lamiaceae	*Mentha*	*Mentha suaveolens*	HQ902829
Eudicotyledons	Lamiaceae	*Mentha*	*Mentha suaveolens*	HQ902855
Eudicotyledons	Ranunculaceae	*Ranunculus*	*Ranunculus enysii*	FJ744171
Eudicotyledons	Ranunculaceae	*Ranunculus*	*Ranunculus enysii*	FJ744172
Eudicotyledons	Lamiaceae	*Salvia*	*Salvia officinalis*	JQ339263
Eudicotyledons	Lamiaceae	*Salvia*	*Salvia officinalis*	DQ667342
Eudicotyledons	Lamiaceae	*Salvia*	*Salvia officinalis*	FR726139
Eudicotyledons	Lamiaceae	*Salvia*	*Salvia officinalis*	FR726140

Eudicotyledons	Lamiaceae	*Salvia*	*Salvia officinalis*	FR726141
Eudicotyledons	Lamiaceae	*Salvia*	*Salvia officinalis*	FJ513122
Eudicotyledons	Lamiaceae	*Salvia*	*Salvia sclarea*	FR726143
Eudicotyledons	Lamiaceae	*Salvia*	*Salvia sclarea*	FJ513083
Eudicotyledons	Plantaginaceae	*Plantago*	*Plantago lanceolata*	FJ395538
Eudicotyledons	Plantaginaceae	*Plantago*	*Plantago lanceolata*	GQ248368
Eudicotyledons	Plantaginaceae	*Plantago*	*Plantago lanceolata*	GQ248369
Eudicotyledons	Plantaginaceae	*Plantago*	*Plantago lanceolata*	EU036257
Eudicotyledons	Plantaginaceae	*Plantago*	*Plantago lanceolata*	EU750471
Eudicotyledons	Plantaginaceae	*Plantago*	*Plantago lanceolata*	EU750472
Eudicotyledons	Plantaginaceae	*Plantago*	*Plantago lanceolata*	EU750473
Eudicotyledons	Rutaceae	*Citrus*	*Citrus aurantium*	GQ248267
Eudicotyledons	Rutaceae	*Citrus*	*Citrus aurantium*	EF590679
Eudicotyledons	Gesneriaceae	*Glossoloma*	*Glossoloma ichthyoderma*	DQ006190
Eudicotyledons	Gesneriaceae	*Glossoloma*	*Glossoloma ichthyoderma*	DQ211214
Eudicotyledons	Lamiaceae	*Salvia*	*Salvia pyrenaica*	HQ902842
Eudicotyledons	Lamiaceae	*Salvia*	*Salvia pyrenaica*	HQ902871
Eudicotyledons	Lamiaceae	*Salvia*	*Salvia uliginosa*	HQ418947
Eudicotyledons	Lamiaceae	*Salvia*	*Salvia uliginosa*	FR726144
Eudicotyledons	Lamiaceae	*Salvia*	*Salvia viridis*	HQ902843
Eudicotyledons	Lamiaceae	*Salvia*	*Salvia viridis*	HQ902861
Eudicotyledons	Gentianaceae	*Gentiana*	*Gentiana algida*	HM460868
Eudicotyledons	Gentianaceae	*Gentiana*	*Gentiana algida*	HM460869
Eudicotyledons	Gentianaceae	*Gentiana*	*Gentiana algida*	HM460870
Eudicotyledons	Gentianaceae	*Gentiana*	*Gentiana straminea*	GQ435167
Eudicotyledons	Gentianaceae	*Gentiana*	*Gentiana straminea*	GQ435168
Eudicotyledons	Rosaceae	*Amelanchier*	*Amelanchier arborea*	EF127152
Eudicotyledons	Rosaceae	*Amelanchier*	*Amelanchier arborea*	JQ390642
Eudicotyledons	Rosaceae	*Amelanchier*	*Amelanchier laevis*	JQ390635
Eudicotyledons	Rosaceae	*Amelanchier*	*Amelanchier laevis*	HQ596593
Eudicotyledons	Hydrangeaceae	*Hydrangea*	*Hydrangea paniculata*	HQ427003
Eudicotyledons	Hydrangeaceae	*Hydrangea*	*Hydrangea paniculata*	HM216984
Eudicotyledons	Gentianaceae	*Gentiana*	*Gentiana triflora*	GQ864031
Eudicotyledons	Gentianaceae	*Gentiana*	*Gentiana triflora*	GQ864032
Eudicotyledons	Rosaceae	*Potentilla*	*Potentilla anserina*	GQ384974
Eudicotyledons	Rosaceae	*Potentilla*	*Potentilla anserina*	FN668452
Eudicotyledons	Rosaceae	*Potentilla*	*Potentilla reptans*	FJ395525
Eudicotyledons	Rosaceae	*Potentilla*	*Potentilla reptans*	GQ384955
Eudicotyledons	Rosaceae	*Potentilla*	*Potentilla nivea*	DQ778819
Eudicotyledons	Rosaceae	*Potentilla*	*Potentilla nivea*	GQ384988
Eudicotyledons	Rosaceae	*Potentilla*	*Potentilla nivea*	GQ435275
Eudicotyledons	Asteraceae	*Senecio*	*Senecio inaequidens*	JN789928
Eudicotyledons	Asteraceae	*Senecio*	*Senecio inaequidens*	JN789929
Eudicotyledons	Asteraceae	*Senecio*	*Senecio inaequidens*	JN789930
Eudicotyledons	Asteraceae	*Senecio*	*Senecio madagascariensis*	JN789939
Eudicotyledons	Asteraceae	*Senecio*	*Senecio madagascariensis*	JN789949
Eudicotyledons	Caprifoliaceae	*Valeriana*	*Valeriana jatamansi*	GU477680
Eudicotyledons	Caprifoliaceae	*Valeriana*	*Valeriana jatamansi*	GU477681
Eudicotyledons	Caprifoliaceae	*Valeriana*	*Valeriana jatamansi*	GU477682
Eudicotyledons	Caprifoliaceae	*Valeriana*	*Valeriana jatamansi*	GU477683
Eudicotyledons	Caprifoliaceae	*Valeriana*	*Valeriana jatamansi*	GU477684
Eudicotyledons	Caprifoliaceae	*Valeriana*	*Valeriana jatamansi*	GU477685
Eudicotyledons	Caprifoliaceae	*Valeriana*	*Valeriana jatamansi*	GU477686

Eudicotyledons	Caprifoliaceae	*Valeriana*	*Valeriana jatamansi*	GU477687
Eudicotyledons	Caprifoliaceae	*Valeriana*	*Valeriana jatamansi*	GQ434936
Eudicotyledons	Rubiaceae	*Psychotria*	*Psychotria deflexa*	GQ982339
Eudicotyledons	Rubiaceae	*Psychotria*	*Psychotria deflexa*	HM446991
Eudicotyledons	Hydrangeaceae	*Hydrangea*	*Hydrangea quercifolia*	EF694837
Eudicotyledons	Hydrangeaceae	*Hydrangea*	*Hydrangea quercifolia*	HM216987
Eudicotyledons	Brassicaceae	*Lepidium*	*Lepidium campestre*	GU562410
Eudicotyledons	Brassicaceae	*Lepidium*	*Lepidium campestre*	HQ596746
Eudicotyledons	Moraceae	*Ficus*	*Ficus microcarpa*	JN407002
Eudicotyledons	Moraceae	*Ficus*	*Ficus microcarpa*	JN407003
Eudicotyledons	Moraceae	*Ficus*	*Ficus microcarpa*	JN407004
Eudicotyledons	Moraceae	*Ficus*	*Ficus microcarpa*	JN407005
Eudicotyledons	Moraceae	*Ficus*	*Ficus microcarpa*	GU135301
Eudicotyledons	Moraceae	*Ficus*	*Ficus religiosa*	GU935104
Eudicotyledons	Moraceae	*Ficus*	*Ficus religiosa*	GU935105
Eudicotyledons	Moraceae	*Ficus*	*Ficus religiosa*	GU935106
Eudicotyledons	Moraceae	*Ficus*	*Ficus religiosa*	GU935107
Eudicotyledons	Moraceae	*Ficus*	*Ficus religiosa*	GU935108
Eudicotyledons	Moraceae	*Ficus*	*Ficus religiosa*	GU935109
Eudicotyledons	Moraceae	*Ficus*	*Ficus religiosa*	GU935110
Eudicotyledons	Rosaceae	*Rubus*	*Rubus allegheniensis*	EU750524
Eudicotyledons	Rosaceae	*Rubus*	*Rubus allegheniensis*	EU750525
Eudicotyledons	Rosaceae	*Rubus*	*Rubus allegheniensis*	EU750526
Eudicotyledons	Rosaceae	*Rubus*	*Rubus crataegifolius*	GU980801
Eudicotyledons	Rosaceae	*Rubus*	*Rubus crataegifolius*	GU980802
Eudicotyledons	Rosaceae	*Rubus*	*Rubus crataegifolius*	GU980803
Eudicotyledons	Rosaceae	*Rubus*	*Rubus crataegifolius*	GU980804
Eudicotyledons	Rosaceae	*Rubus*	*Rubus crataegifolius*	GU980805
Eudicotyledons	Rosaceae	*Rubus*	*Rubus crataegifolius*	GU980806
Eudicotyledons	Rosaceae	*Rubus*	*Rubus crataegifolius*	GU980807
Eudicotyledons	Rosaceae	*Rubus*	*Rubus crataegifolius*	GU980808
Eudicotyledons	Rosaceae	*Rubus*	*Rubus crataegifolius*	GU980809
Eudicotyledons	Rosaceae	*Rubus*	*Rubus crataegifolius*	GU980810
Eudicotyledons	Rosaceae	*Rubus*	*Rubus crataegifolius*	GU980811
Eudicotyledons	Rosaceae	*Rubus*	*Rubus crataegifolius*	GU980812
Eudicotyledons	Rosaceae	*Rubus*	*Rubus crataegifolius*	GU980813
Eudicotyledons	Rosaceae	*Rubus*	*Rubus crataegifolius*	GU980814
Eudicotyledons	Rosaceae	*Rubus*	*Rubus crataegifolius*	GU980815
Eudicotyledons	Rosaceae	*Rubus*	*Rubus crataegifolius*	GQ435264
Eudicotyledons	Rosaceae	*Rubus*	*Rubus occidentalis*	HQ596824
Eudicotyledons	Rosaceae	*Rubus*	*Rubus occidentalis*	EU750532
Eudicotyledons	Rosaceae	*Rubus*	*Rubus occidentalis*	EU750533
Eudicotyledons	Rosaceae	*Rubus*	*Rubus occidentalis*	EU750534
Eudicotyledons	Rosaceae	*Rubus*	*Rubus occidentalis*	EU750535
Eudicotyledons	Rosaceae	*Rubus*	*Rubus odoratus*	EU750536
Eudicotyledons	Rosaceae	*Rubus*	*Rubus odoratus*	EU750537
Eudicotyledons	Asteraceae	*Senecio*	*Senecio vulgaris*	FJ395482
Eudicotyledons	Asteraceae	*Senecio*	*Senecio vulgaris*	EF538071
Eudicotyledons	Rubiaceae	*Psychotria*	*Psychotria rubra*	JN407051
Eudicotyledons	Rubiaceae	*Psychotria*	*Psychotria rubra*	JN407052
Eudicotyledons	Rubiaceae	*Psychotria*	*Psychotria rubra*	JN407053
Eudicotyledons	Rubiaceae	*Psychotria*	*Psychotria rubra*	JN407054
Eudicotyledons	Rubiaceae	*Psychotria*	*Psychotria graciliflora*	FJ208668

Eudicotyledons	Rubiaceae	*Psychotria*	*Psychotria graciliflora*	FJ208669
Eudicotyledons	Rubiaceae	*Psychotria*	*Psychotria graciliflora*	GQ982340
Eudicotyledons	Verbenaceae	*Verbena*	*Verbena officinalis*	GQ435187
Eudicotyledons	Verbenaceae	*Verbena*	*Verbena officinalis*	GQ435188
Eudicotyledons	Solanaceae	*Nolana*	*Nolana cerrateana*	EU742453
Eudicotyledons	Solanaceae	*Nolana*	*Nolana cerrateana*	FJ914079
Eudicotyledons	Solanaceae	*Nolana*	*Nolana humifusa*	EU742468
Eudicotyledons	Solanaceae	*Nolana*	*Nolana humifusa*	FJ914067
Eudicotyledons	Solanaceae	*Nolana*	*Nolana humifusa*	FJ914068
Eudicotyledons	Solanaceae	*Nolana*	*Nolana humifusa*	FJ914085
Eudicotyledons	Solanaceae	*Nolana*	*Nolana humifusa*	FJ914086
Eudicotyledons	Solanaceae	*Nolana*	*Nolana humifusa*	FJ914089
Eudicotyledons	Solanaceae	*Nolana*	*Nolana humifusa*	FJ914090
Eudicotyledons	Solanaceae	*Nolana*	*Nolana lycioides*	EU742479
Eudicotyledons	Solanaceae	*Nolana*	*Nolana lycioides*	FJ914087
Eudicotyledons	Solanaceae	*Nolana*	*Nolana peruviana*	EU742484
Eudicotyledons	Solanaceae	*Nolana*	*Nolana peruviana*	EU742485
Eudicotyledons	Solanaceae	*Nolana*	*Nolana stenophylla*	EU742497
Eudicotyledons	Solanaceae	*Nolana*	*Nolana stenophylla*	FJ914074
Eudicotyledons	Rutaceae	*Citrus*	*Citrus reticulata*	GQ248268
Eudicotyledons	Rutaceae	*Citrus*	*Citrus reticulata*	GQ267062
Eudicotyledons	Rutaceae	*Citrus*	*Citrus reticulata*	GQ267063
Eudicotyledons	Rutaceae	*Citrus*	*Citrus reticulata*	GQ435438
Eudicotyledons	Rutaceae	*Citrus*	*Citrus reticulata*	EF590680
Eudicotyledons	Fabaceae	*Astragalus*	*Astragalus chinensis*	GU396745
Eudicotyledons	Fabaceae	*Astragalus*	*Astragalus chinensis*	GQ434966
Eudicotyledons	Crassulaceae	*Aeonium*	*Aeonium percarneum*	AY082210
Eudicotyledons	Crassulaceae	*Aeonium*	*Aeonium percarneum*	AY082211
Eudicotyledons	Crassulaceae	*Aeonium*	*Aeonium aureum*	AY082186
Eudicotyledons	Crassulaceae	*Aeonium*	*Aeonium aureum*	AY082187
Eudicotyledons	Hydrangeaceae	*Hydrangea*	*Hydrangea arborescens*	DQ006194
Eudicotyledons	Hydrangeaceae	*Hydrangea*	*Hydrangea arborescens*	HM216966
Eudicotyledons	Hydrangeaceae	*Hydrangea*	*Hydrangea arborescens*	JF321259
Eudicotyledons	Fagaceae	*Quercus*	*Quercus phillyraeoides*	HQ427017
Eudicotyledons	Fagaceae	*Quercus*	*Quercus phillyraeoides*	AB650376
Eudicotyledons	Fagaceae	*Quercus*	*Quercus phillyraeoides*	AB650377
Eudicotyledons	Fagaceae	*Quercus*	*Quercus phillyraeoides*	AB650378
Eudicotyledons	Fagaceae	*Quercus*	*Quercus phillyraeoides*	AB650379
Eudicotyledons	Fagaceae	*Quercus*	*Quercus phillyraeoides*	AB650380
Eudicotyledons	Fagaceae	*Quercus*	*Quercus phillyraeoides*	AB650381
Eudicotyledons	Fagaceae	*Quercus*	*Quercus phillyraeoides*	AB650382
Eudicotyledons	Fagaceae	*Quercus*	*Quercus phillyraeoides*	AB650383
Eudicotyledons	Fagaceae	*Quercus*	*Quercus phillyraeoides*	AB650384
Eudicotyledons	Fagaceae	*Quercus*	*Quercus phillyraeoides*	AB650385
Eudicotyledons	Fagaceae	*Quercus*	*Quercus phillyraeoides*	AB650386
Eudicotyledons	Fagaceae	*Quercus*	*Quercus phillyraeoides*	AB650387
Eudicotyledons	Fagaceae	*Quercus*	*Quercus phillyraeoides*	AB650388
Eudicotyledons	Fagaceae	*Quercus*	*Quercus phillyraeoides*	AB650389
Eudicotyledons	Fagaceae	*Quercus*	*Quercus phillyraeoides*	AB650390
Eudicotyledons	Fagaceae	*Quercus*	*Quercus phillyraeoides*	AB650391
Eudicotyledons	Fagaceae	*Quercus*	*Quercus phillyraeoides*	AB650392
Eudicotyledons	Fagaceae	*Quercus*	*Quercus phillyraeoides*	AB650393
Eudicotyledons	Fagaceae	*Quercus*	*Quercus phillyraeoides*	AB650394

Eudicotyledons	Fagaceae	*Quercus*	*Quercus phillyraeoides*	AB650395
Eudicotyledons	Fagaceae	*Quercus*	*Quercus phillyraeoides*	AB650396
Eudicotyledons	Fagaceae	*Quercus*	*Quercus phillyraeoides*	AB650397
Eudicotyledons	Fagaceae	*Quercus*	*Quercus phillyraeoides*	AB650398
Eudicotyledons	Fagaceae	*Quercus*	*Quercus phillyraeoides*	AB650399
Eudicotyledons	Fagaceae	*Quercus*	*Quercus phillyraeoides*	AB650400
Eudicotyledons	Fagaceae	*Quercus*	*Quercus phillyraeoides*	AB650401
Eudicotyledons	Fagaceae	*Quercus*	*Quercus phillyraeoides*	AB650402
Eudicotyledons	Fagaceae	*Quercus*	*Quercus phillyraeoides*	AB650403
Eudicotyledons	Fagaceae	*Quercus*	*Quercus phillyraeoides*	AB650404
Eudicotyledons	Fagaceae	*Quercus*	*Quercus phillyraeoides*	AB650405
Eudicotyledons	Fagaceae	*Quercus*	*Quercus phillyraeoides*	AB650406
Eudicotyledons	Fagaceae	*Quercus*	*Quercus phillyraeoides*	AB650407
Eudicotyledons	Fagaceae	*Quercus*	*Quercus phillyraeoides*	AB650408
Eudicotyledons	Fagaceae	*Quercus*	*Quercus phillyraeoides*	AB650409
Eudicotyledons	Fagaceae	*Quercus*	*Quercus phillyraeoides*	AB650410
Eudicotyledons	Fagaceae	*Quercus*	*Quercus phillyraeoides*	AB650411
Eudicotyledons	Fagaceae	*Quercus*	*Quercus phillyraeoides*	AB650412
Eudicotyledons	Fagaceae	*Quercus*	*Quercus phillyraeoides*	AB650413
Eudicotyledons	Fagaceae	*Quercus*	*Quercus phillyraeoides*	AB650414
Eudicotyledons	Fagaceae	*Quercus*	*Quercus phillyraeoides*	AB650415
Eudicotyledons	Fagaceae	*Quercus*	*Quercus phillyraeoides*	AB650416
Eudicotyledons	Fagaceae	*Quercus*	*Quercus phillyraeoides*	AB650417
Eudicotyledons	Fagaceae	*Quercus*	*Quercus phillyraeoides*	AB650418
Eudicotyledons	Fagaceae	*Quercus*	*Quercus phillyraeoides*	AB650419
Eudicotyledons	Fagaceae	*Quercus*	*Quercus phillyraeoides*	AB650420
Eudicotyledons	Fagaceae	*Quercus*	*Quercus phillyraeoides*	AB650421
Eudicotyledons	Fagaceae	*Quercus*	*Quercus phillyraeoides*	AB650422
Eudicotyledons	Fagaceae	*Quercus*	*Quercus phillyraeoides*	AB650423
Eudicotyledons	Fagaceae	*Quercus*	*Quercus phillyraeoides*	AB650424
Eudicotyledons	Fagaceae	*Quercus*	*Quercus phillyraeoides*	AB650425
Eudicotyledons	Fagaceae	*Quercus*	*Quercus phillyraeoides*	AB650426
Eudicotyledons	Fagaceae	*Quercus*	*Quercus phillyraeoides*	AB650427
Eudicotyledons	Fagaceae	*Quercus*	*Quercus phillyraeoides*	AB650428
Eudicotyledons	Fagaceae	*Quercus*	*Quercus phillyraeoides*	AB650429
Eudicotyledons	Fagaceae	*Quercus*	*Quercus phillyraeoides*	AB650430
Eudicotyledons	Fagaceae	*Quercus*	*Quercus phillyraeoides*	AB650431
Eudicotyledons	Fagaceae	*Quercus*	*Quercus phillyraeoides*	AB650432
Eudicotyledons	Fagaceae	*Quercus*	*Quercus phillyraeoides*	AB650433
Eudicotyledons	Fagaceae	*Quercus*	*Quercus phillyraeoides*	AB650434
Eudicotyledons	Fagaceae	*Quercus*	*Quercus phillyraeoides*	AB650435
Eudicotyledons	Fagaceae	*Quercus*	*Quercus phillyraeoides*	AB650436
Eudicotyledons	Fagaceae	*Quercus*	*Quercus phillyraeoides*	AB650437
Eudicotyledons	Fagaceae	*Quercus*	*Quercus phillyraeoides*	AB650438
Eudicotyledons	Fagaceae	*Quercus*	*Quercus phillyraeoides*	AB650439
Eudicotyledons	Fagaceae	*Quercus*	*Quercus phillyraeoides*	AB650440
Eudicotyledons	Fagaceae	*Quercus*	*Quercus phillyraeoides*	AB650441
Eudicotyledons	Fagaceae	*Quercus*	*Quercus phillyraeoides*	AB650442
Eudicotyledons	Fagaceae	*Quercus*	*Quercus phillyraeoides*	AB650443
Eudicotyledons	Fagaceae	*Quercus*	*Quercus phillyraeoides*	AB650444
Eudicotyledons	Fagaceae	*Quercus*	*Quercus phillyraeoides*	AB650445
Eudicotyledons	Fagaceae	*Quercus*	*Quercus phillyraeoides*	AB650446
Eudicotyledons	Fagaceae	*Quercus*	*Quercus phillyraeoides*	AB650447

Eudicotyledons	Fagaceae	*Quercus*	*Quercus phillyraeoides*	AB650448
Eudicotyledons	Fagaceae	*Quercus*	*Quercus phillyraeoides*	AB650449
Eudicotyledons	Fagaceae	*Quercus*	*Quercus phillyraeoides*	AB650450
Eudicotyledons	Fagaceae	*Quercus*	*Quercus phillyraeoides*	AB650451
Eudicotyledons	Fagaceae	*Quercus*	*Quercus phillyraeoides*	AB650452
Eudicotyledons	Fagaceae	*Quercus*	*Quercus phillyraeoides*	AB650453
Eudicotyledons	Fagaceae	*Quercus*	*Quercus phillyraeoides*	AB650454
Eudicotyledons	Fagaceae	*Quercus*	*Quercus phillyraeoides*	AB650455
Eudicotyledons	Fagaceae	*Quercus*	*Quercus phillyraeoides*	AB650456
Eudicotyledons	Fagaceae	*Quercus*	*Quercus phillyraeoides*	AB650457
Eudicotyledons	Fagaceae	*Quercus*	*Quercus phillyraeoides*	AB650458
Eudicotyledons	Fagaceae	*Quercus*	*Quercus phillyraeoides*	AB650459
Eudicotyledons	Fagaceae	*Quercus*	*Quercus phillyraeoides*	AB650460
Eudicotyledons	Fagaceae	*Quercus*	*Quercus phillyraeoides*	AB650461
Eudicotyledons	Fagaceae	*Quercus*	*Quercus phillyraeoides*	AB650462
Eudicotyledons	Fagaceae	*Quercus*	*Quercus phillyraeoides*	AB650463
Eudicotyledons	Fagaceae	*Quercus*	*Quercus phillyraeoides*	AB650464
Eudicotyledons	Fagaceae	*Quercus*	*Quercus phillyraeoides*	AB650465
Eudicotyledons	Loganiaceae	*Strychnos*	*Strychnos spinosa*	EU213863
Eudicotyledons	Loganiaceae	*Strychnos*	*Strychnos spinosa*	EU213864
Eudicotyledons	Loganiaceae	*Strychnos*	*Strychnos spinosa*	EU213865
Eudicotyledons	Moraceae	*Ficus*	*Ficus insipida*	GQ438012
Eudicotyledons	Moraceae	*Ficus*	*Ficus insipida*	GQ438013
Eudicotyledons	Moraceae	*Ficus*	*Ficus insipida*	GQ438014
Eudicotyledons	Moraceae	*Ficus*	*Ficus insipida*	GQ438015
Eudicotyledons	Moraceae	*Ficus*	*Ficus insipida*	GQ438016
Eudicotyledons	Moraceae	*Ficus*	*Ficus insipida*	GQ438017
Eudicotyledons	Moraceae	*Ficus*	*Ficus insipida*	GQ438018
Eudicotyledons	Moraceae	*Ficus*	*Ficus insipida*	GQ438019
Eudicotyledons	Moraceae	*Ficus*	*Ficus insipida*	GQ438020
Eudicotyledons	Moraceae	*Ficus*	*Ficus insipida*	GQ438021
Eudicotyledons	Moraceae	*Ficus*	*Ficus insipida*	GQ438022
Eudicotyledons	Moraceae	*Ficus*	*Ficus insipida*	GQ438023
Eudicotyledons	Moraceae	*Ficus*	*Ficus insipida*	GQ438024
Eudicotyledons	Moraceae	*Ficus*	*Ficus insipida*	GQ438025
Eudicotyledons	Moraceae	*Ficus*	*Ficus insipida*	GQ438026
Eudicotyledons	Moraceae	*Ficus*	*Ficus insipida*	GQ438027
Eudicotyledons	Moraceae	*Ficus*	*Ficus insipida*	GQ438028
Eudicotyledons	Moraceae	*Ficus*	*Ficus insipida*	GQ438029
Eudicotyledons	Moraceae	*Ficus*	*Ficus insipida*	GQ438030
Eudicotyledons	Moraceae	*Ficus*	*Ficus insipida*	GQ438031
Eudicotyledons	Moraceae	*Ficus*	*Ficus insipida*	GQ438032
Eudicotyledons	Moraceae	*Ficus*	*Ficus insipida*	GQ438033
Eudicotyledons	Moraceae	*Ficus*	*Ficus insipida*	GQ438034
Eudicotyledons	Moraceae	*Ficus*	*Ficus insipida*	GQ438035
Eudicotyledons	Moraceae	*Ficus*	*Ficus insipida*	GQ438036
Eudicotyledons	Moraceae	*Ficus*	*Ficus insipida*	GQ438037
Eudicotyledons	Moraceae	*Ficus*	*Ficus insipida*	GQ438038
Eudicotyledons	Moraceae	*Ficus*	*Ficus insipida*	GQ438039
Eudicotyledons	Moraceae	*Ficus*	*Ficus insipida*	GQ438040
Eudicotyledons	Moraceae	*Ficus*	*Ficus insipida*	GQ438041
Eudicotyledons	Moraceae	*Ficus*	*Ficus insipida*	GQ438042
Eudicotyledons	Moraceae	*Ficus*	*Ficus insipida*	GQ438043

Eudicotyledons	Moraceae	*Ficus*	*Ficus insipida*	GQ438044
Eudicotyledons	Moraceae	*Ficus*	*Ficus insipida*	GQ438045
Eudicotyledons	Moraceae	*Ficus*	*Ficus insipida*	GQ438046
Eudicotyledons	Moraceae	*Ficus*	*Ficus insipida*	GQ438047
Eudicotyledons	Moraceae	*Ficus*	*Ficus insipida*	GQ438048
Eudicotyledons	Moraceae	*Ficus*	*Ficus insipida*	GQ438049
Eudicotyledons	Moraceae	*Ficus*	*Ficus insipida*	GQ438050
Eudicotyledons	Moraceae	*Ficus*	*Ficus insipida*	GQ438051
Eudicotyledons	Moraceae	*Ficus*	*Ficus insipida*	GQ438052
Eudicotyledons	Moraceae	*Ficus*	*Ficus insipida*	GQ438053
Eudicotyledons	Moraceae	*Ficus*	*Ficus insipida*	GQ438054
Eudicotyledons	Moraceae	*Ficus*	*Ficus insipida*	GQ438055
Eudicotyledons	Moraceae	*Ficus*	*Ficus insipida*	GQ438056
Eudicotyledons	Moraceae	*Ficus*	*Ficus insipida*	GQ438057
Eudicotyledons	Moraceae	*Ficus*	*Ficus insipida*	GQ438058
Eudicotyledons	Moraceae	*Ficus*	*Ficus insipida*	GQ438059
Eudicotyledons	Moraceae	*Ficus*	*Ficus insipida*	GQ438060
Eudicotyledons	Moraceae	*Ficus*	*Ficus insipida*	GQ438061
Eudicotyledons	Moraceae	*Ficus*	*Ficus insipida*	GQ438062
Eudicotyledons	Moraceae	*Ficus*	*Ficus insipida*	GQ438063
Eudicotyledons	Moraceae	*Ficus*	*Ficus insipida*	GQ438064
Eudicotyledons	Moraceae	*Ficus*	*Ficus insipida*	GQ438065
Eudicotyledons	Moraceae	*Ficus*	*Ficus insipida*	GQ438066
Eudicotyledons	Moraceae	*Ficus*	*Ficus insipida*	GQ438067
Eudicotyledons	Moraceae	*Ficus*	*Ficus insipida*	GQ438068
Eudicotyledons	Moraceae	*Ficus*	*Ficus insipida*	GQ438069
Eudicotyledons	Moraceae	*Ficus*	*Ficus insipida*	GQ438070
Eudicotyledons	Moraceae	*Ficus*	*Ficus insipida*	GQ438071
Eudicotyledons	Moraceae	*Ficus*	*Ficus insipida*	GQ438072
Eudicotyledons	Moraceae	*Ficus*	*Ficus insipida*	GQ438073
Eudicotyledons	Moraceae	*Ficus*	*Ficus insipida*	GQ438074
Eudicotyledons	Moraceae	*Ficus*	*Ficus insipida*	GQ438075
Eudicotyledons	Moraceae	*Ficus*	*Ficus insipida*	GQ438076
Eudicotyledons	Moraceae	*Ficus*	*Ficus insipida*	GQ438077
Eudicotyledons	Moraceae	*Ficus*	*Ficus insipida*	GQ438078
Eudicotyledons	Moraceae	*Ficus*	*Ficus insipida*	GQ438079
Eudicotyledons	Moraceae	*Ficus*	*Ficus insipida*	GQ438081
Eudicotyledons	Moraceae	*Ficus*	*Ficus insipida*	GQ438082
Eudicotyledons	Moraceae	*Ficus*	*Ficus insipida*	GQ438083
Eudicotyledons	Moraceae	*Ficus*	*Ficus insipida*	GQ438084
Eudicotyledons	Moraceae	*Ficus*	*Ficus insipida*	GQ438085
Eudicotyledons	Moraceae	*Ficus*	*Ficus insipida*	GQ438086
Eudicotyledons	Moraceae	*Ficus*	*Ficus insipida*	GQ438087
Eudicotyledons	Moraceae	*Ficus*	*Ficus insipida*	GQ438088
Eudicotyledons	Moraceae	*Ficus*	*Ficus insipida*	GQ438089
Eudicotyledons	Moraceae	*Ficus*	*Ficus insipida*	GQ438090
Eudicotyledons	Moraceae	*Ficus*	*Ficus insipida*	GQ438091
Eudicotyledons	Moraceae	*Ficus*	*Ficus insipida*	GQ438092
Eudicotyledons	Moraceae	*Ficus*	*Ficus insipida*	GQ438093
Eudicotyledons	Moraceae	*Ficus*	*Ficus insipida*	GQ438094
Eudicotyledons	Moraceae	*Ficus*	*Ficus insipida*	GQ438095
Eudicotyledons	Moraceae	*Ficus*	*Ficus insipida*	GQ438096
Eudicotyledons	Moraceae	*Ficus*	*Ficus insipida*	GQ438097

Eudicotyledons	Moraceae	*Ficus*	*Ficus insipida*	GQ438098
Eudicotyledons	Moraceae	*Ficus*	*Ficus insipida*	GQ438099
Eudicotyledons	Moraceae	*Ficus*	*Ficus insipida*	GQ438100
Eudicotyledons	Moraceae	*Ficus*	*Ficus insipida*	GQ438101
Eudicotyledons	Moraceae	*Ficus*	*Ficus insipida*	GQ438102
Eudicotyledons	Moraceae	*Ficus*	*Ficus insipida*	GQ438103
Eudicotyledons	Moraceae	*Ficus*	*Ficus insipida*	GQ438104
Eudicotyledons	Moraceae	*Ficus*	*Ficus insipida*	GQ438105
Eudicotyledons	Moraceae	*Ficus*	*Ficus insipida*	GQ438106
Eudicotyledons	Moraceae	*Ficus*	*Ficus insipida*	GQ438107
Eudicotyledons	Moraceae	*Ficus*	*Ficus insipida*	GQ438108
Eudicotyledons	Moraceae	*Ficus*	*Ficus insipida*	GQ438109
Eudicotyledons	Moraceae	*Ficus*	*Ficus insipida*	GQ438110
Eudicotyledons	Moraceae	*Ficus*	*Ficus insipida*	GQ438111
Eudicotyledons	Moraceae	*Ficus*	*Ficus insipida*	GQ438112
Eudicotyledons	Moraceae	*Ficus*	*Ficus insipida*	GQ438113
Eudicotyledons	Moraceae	*Ficus*	*Ficus insipida*	GQ438114
Eudicotyledons	Moraceae	*Ficus*	*Ficus insipida*	GQ438115
Eudicotyledons	Moraceae	*Ficus*	*Ficus insipida*	GQ438116
Eudicotyledons	Moraceae	*Ficus*	*Ficus insipida*	GQ438117
Eudicotyledons	Moraceae	*Ficus*	*Ficus insipida*	GQ438118
Eudicotyledons	Moraceae	*Ficus*	*Ficus insipida*	GQ438119
Eudicotyledons	Moraceae	*Ficus*	*Ficus insipida*	GQ438120
Eudicotyledons	Moraceae	*Ficus*	*Ficus insipida*	GQ438121
Eudicotyledons	Moraceae	*Ficus*	*Ficus insipida*	GQ438122
Eudicotyledons	Moraceae	*Ficus*	*Ficus insipida*	GQ438123
Eudicotyledons	Moraceae	*Ficus*	*Ficus insipida*	GQ438124
Eudicotyledons	Moraceae	*Ficus*	*Ficus insipida*	GQ438125
Eudicotyledons	Moraceae	*Ficus*	*Ficus insipida*	GQ438126
Eudicotyledons	Moraceae	*Ficus*	*Ficus insipida*	GQ438127
Eudicotyledons	Moraceae	*Ficus*	*Ficus insipida*	GQ438128
Eudicotyledons	Moraceae	*Ficus*	*Ficus insipida*	GQ438129
Eudicotyledons	Moraceae	*Ficus*	*Ficus insipida*	GQ438130
Eudicotyledons	Moraceae	*Ficus*	*Ficus insipida*	GQ438131
Eudicotyledons	Moraceae	*Ficus*	*Ficus insipida*	GQ438132
Eudicotyledons	Moraceae	*Ficus*	*Ficus insipida*	GQ438133
Eudicotyledons	Moraceae	*Ficus*	*Ficus insipida*	GQ438134
Eudicotyledons	Moraceae	*Ficus*	*Ficus insipida*	GQ438135
Eudicotyledons	Moraceae	*Ficus*	*Ficus insipida*	GQ438136
Eudicotyledons	Moraceae	*Ficus*	*Ficus insipida*	GQ438137
Eudicotyledons	Moraceae	*Ficus*	*Ficus insipida*	GQ438138
Eudicotyledons	Moraceae	*Ficus*	*Ficus insipida*	GQ438139
Eudicotyledons	Moraceae	*Ficus*	*Ficus insipida*	GQ438140
Eudicotyledons	Moraceae	*Ficus*	*Ficus insipida*	GQ438141
Eudicotyledons	Moraceae	*Ficus*	*Ficus insipida*	GQ438142
Eudicotyledons	Moraceae	*Ficus*	*Ficus insipida*	GQ438143
Eudicotyledons	Moraceae	*Ficus*	*Ficus insipida*	GQ438144
Eudicotyledons	Moraceae	*Ficus*	*Ficus insipida*	GQ438145
Eudicotyledons	Moraceae	*Ficus*	*Ficus insipida*	GQ438146
Eudicotyledons	Moraceae	*Ficus*	*Ficus insipida*	GQ438147
Eudicotyledons	Moraceae	*Ficus*	*Ficus insipida*	GQ438148
Eudicotyledons	Moraceae	*Ficus*	*Ficus insipida*	GQ438149
Eudicotyledons	Moraceae	*Ficus*	*Ficus insipida*	GQ438150

Eudicotyledons	Moraceae	*Ficus*	*Ficus insipida*	GQ438151
Eudicotyledons	Moraceae	*Ficus*	*Ficus insipida*	GQ438152
Eudicotyledons	Moraceae	*Ficus*	*Ficus insipida*	GQ438153
Eudicotyledons	Moraceae	*Ficus*	*Ficus insipida*	GQ438154
Eudicotyledons	Moraceae	*Ficus*	*Ficus insipida*	GQ438155
Eudicotyledons	Moraceae	*Ficus*	*Ficus insipida*	GQ438156
Eudicotyledons	Moraceae	*Ficus*	*Ficus insipida*	GQ438157
Eudicotyledons	Moraceae	*Ficus*	*Ficus insipida*	GQ438158
Eudicotyledons	Moraceae	*Ficus*	*Ficus insipida*	GQ438159
Eudicotyledons	Moraceae	*Ficus*	*Ficus insipida*	GQ438160
Eudicotyledons	Moraceae	*Ficus*	*Ficus insipida*	GQ438161
Eudicotyledons	Moraceae	*Ficus*	*Ficus insipida*	GQ438162
Eudicotyledons	Moraceae	*Ficus*	*Ficus insipida*	GQ438163
Eudicotyledons	Moraceae	*Ficus*	*Ficus insipida*	GQ438164
Eudicotyledons	Moraceae	*Ficus*	*Ficus insipida*	GQ438165
Eudicotyledons	Moraceae	*Ficus*	*Ficus insipida*	GQ438166
Eudicotyledons	Moraceae	*Ficus*	*Ficus insipida*	GQ438167
Eudicotyledons	Moraceae	*Ficus*	*Ficus insipida*	GQ438168
Eudicotyledons	Moraceae	*Ficus*	*Ficus insipida*	GQ438169
Eudicotyledons	Moraceae	*Ficus*	*Ficus insipida*	GQ438170
Eudicotyledons	Moraceae	*Ficus*	*Ficus insipida*	GQ438171
Eudicotyledons	Moraceae	*Ficus*	*Ficus insipida*	GQ438172
Eudicotyledons	Moraceae	*Ficus*	*Ficus insipida*	GQ438173
Eudicotyledons	Moraceae	*Ficus*	*Ficus insipida*	GQ438174
Eudicotyledons	Moraceae	*Ficus*	*Ficus insipida*	GQ438175
Eudicotyledons	Moraceae	*Ficus*	*Ficus insipida*	GQ438176
Eudicotyledons	Moraceae	*Ficus*	*Ficus insipida*	GQ438177
Eudicotyledons	Moraceae	*Ficus*	*Ficus insipida*	GQ438178
Eudicotyledons	Moraceae	*Ficus*	*Ficus insipida*	GQ438179
Eudicotyledons	Moraceae	*Ficus*	*Ficus insipida*	GQ438180
Eudicotyledons	Moraceae	*Ficus*	*Ficus insipida*	GQ438181
Eudicotyledons	Moraceae	*Ficus*	*Ficus insipida*	GQ438182
Eudicotyledons	Moraceae	*Ficus*	*Ficus insipida*	GQ438183
Eudicotyledons	Moraceae	*Ficus*	*Ficus insipida*	GQ438184
Eudicotyledons	Moraceae	*Ficus*	*Ficus insipida*	GQ438185
Eudicotyledons	Moraceae	*Ficus*	*Ficus insipida*	GQ438186
Eudicotyledons	Moraceae	*Ficus*	*Ficus insipida*	GQ438187
Eudicotyledons	Moraceae	*Ficus*	*Ficus insipida*	GQ438188
Eudicotyledons	Moraceae	*Ficus*	*Ficus insipida*	GQ438189
Eudicotyledons	Moraceae	*Ficus*	*Ficus insipida*	GQ438190
Eudicotyledons	Moraceae	*Ficus*	*Ficus insipida*	GQ438191
Eudicotyledons	Moraceae	*Ficus*	*Ficus insipida*	GQ438193
Eudicotyledons	Moraceae	*Ficus*	*Ficus insipida*	GQ438194
Eudicotyledons	Moraceae	*Ficus*	*Ficus insipida*	GQ438195
Eudicotyledons	Moraceae	*Ficus*	*Ficus insipida*	GQ438196
Eudicotyledons	Moraceae	*Ficus*	*Ficus insipida*	GQ438197
Eudicotyledons	Moraceae	*Ficus*	*Ficus insipida*	GQ438198
Eudicotyledons	Moraceae	*Ficus*	*Ficus insipida*	GQ438199
Eudicotyledons	Moraceae	*Ficus*	*Ficus insipida*	GQ438200
Eudicotyledons	Moraceae	*Ficus*	*Ficus insipida*	GQ438201
Eudicotyledons	Moraceae	*Ficus*	*Ficus insipida*	GQ438202
Eudicotyledons	Moraceae	*Ficus*	*Ficus insipida*	GQ438203
Eudicotyledons	Moraceae	*Ficus*	*Ficus insipida*	GQ438204

Eudicotyledons	Moraceae	*Ficus*	*Ficus insipida*	GQ438205
Eudicotyledons	Moraceae	*Ficus*	*Ficus insipida*	GQ438206
Eudicotyledons	Moraceae	*Ficus*	*Ficus insipida*	GQ438207
Eudicotyledons	Moraceae	*Ficus*	*Ficus insipida*	GQ438208
Eudicotyledons	Moraceae	*Ficus*	*Ficus insipida*	GQ438209
Eudicotyledons	Moraceae	*Ficus*	*Ficus insipida*	GQ438210
Eudicotyledons	Moraceae	*Ficus*	*Ficus insipida*	GQ438211
Eudicotyledons	Moraceae	*Ficus*	*Ficus insipida*	GQ982221
Eudicotyledons	Moraceae	*Ficus*	*Ficus racemosa*	GU935097
Eudicotyledons	Moraceae	*Ficus*	*Ficus racemosa*	GU935098
Eudicotyledons	Moraceae	*Ficus*	*Ficus racemosa*	GU935099
Eudicotyledons	Moraceae	*Ficus*	*Ficus racemosa*	GU935100
Eudicotyledons	Moraceae	*Ficus*	*Ficus racemosa*	JN044534
Eudicotyledons	Moraceae	*Ficus*	*Ficus racemosa*	JN044535
Eudicotyledons	Moraceae	*Ficus*	*Ficus tinctoria*	JN044542
Eudicotyledons	Moraceae	*Ficus*	*Ficus tinctoria*	JN044543
Eudicotyledons	Moraceae	*Ficus*	*Ficus tinctoria*	JN044544
Eudicotyledons	Moraceae	*Ficus*	*Ficus virens*	GU935116
Eudicotyledons	Moraceae	*Ficus*	*Ficus virens*	GU935117
Eudicotyledons	Plantaginaceae	*Plantago*	*Plantago rugelii*	EF590728
Eudicotyledons	Plantaginaceae	*Plantago*	*Plantago rugelii*	DQ006212
Eudicotyledons	Plantaginaceae	*Plantago*	*Plantago rugelii*	EU750477
Eudicotyledons	Plantaginaceae	*Plantago*	*Plantago rugelii*	EU750478
Eudicotyledons	Betulaceae	*Alnus*	*Alnus cordata*	FJ844542
Eudicotyledons	Betulaceae	*Alnus*	*Alnus cordata*	FJ844543
Eudicotyledons	Betulaceae	*Alnus*	*Alnus cordata*	FJ844544
Eudicotyledons	Betulaceae	*Alnus*	*Alnus firma*	FJ844493
Eudicotyledons	Betulaceae	*Alnus*	*Alnus firma*	FJ844494
Eudicotyledons	Betulaceae	*Alnus*	*Alnus firma*	FJ844495
Eudicotyledons	Betulaceae	*Alnus*	*Alnus firma*	FJ844496
Eudicotyledons	Betulaceae	*Alnus*	*Alnus firma*	FJ844497
Eudicotyledons	Betulaceae	*Alnus*	*Alnus formosana*	FJ844545
Eudicotyledons	Betulaceae	*Alnus*	*Alnus formosana*	FJ844546
Eudicotyledons	Betulaceae	*Alnus*	*Alnus hirsuta*	FJ011869
Eudicotyledons	Betulaceae	*Alnus*	*Alnus hirsuta*	FJ844520
Eudicotyledons	Betulaceae	*Alnus*	*Alnus hirsuta*	FJ844521
Eudicotyledons	Betulaceae	*Alnus*	*Alnus hirsuta*	FJ844523
Eudicotyledons	Betulaceae	*Alnus*	*Alnus hirsuta*	FJ844524
Eudicotyledons	Betulaceae	*Alnus*	*Alnus hirsuta*	FJ844525
Eudicotyledons	Betulaceae	*Alnus*	*Alnus hirsuta*	FJ844526
Eudicotyledons	Betulaceae	*Alnus*	*Alnus hirsuta*	FJ844527
Eudicotyledons	Betulaceae	*Alnus*	*Alnus hirsuta*	FJ844528
Eudicotyledons	Betulaceae	*Alnus*	*Alnus hirsuta*	FJ844529
Eudicotyledons	Betulaceae	*Alnus*	*Alnus hirsuta*	FJ844531
Eudicotyledons	Betulaceae	*Alnus*	*Alnus hirsuta*	JN043792
Eudicotyledons	Betulaceae	*Alnus*	*Alnus hirsuta*	JN043793
Eudicotyledons	Betulaceae	*Alnus*	*Alnus hirsuta*	JN043794
Eudicotyledons	Betulaceae	*Alnus*	*Alnus hirsuta*	JN043795
Eudicotyledons	Betulaceae	*Alnus*	*Alnus japonica*	AY211458
Eudicotyledons	Betulaceae	*Alnus*	*Alnus japonica*	FJ844522
Eudicotyledons	Betulaceae	*Alnus*	*Alnus japonica*	FJ844555
Eudicotyledons	Betulaceae	*Alnus*	*Alnus japonica*	FJ844556
Eudicotyledons	Betulaceae	*Alnus*	*Alnus japonica*	FJ844557

Eudicotyledons	Betulaceae	*Alnus*	*Alnus japonica*	FJ844558
Eudicotyledons	Betulaceae	*Alnus*	*Alnus japonica*	FJ844559
Eudicotyledons	Betulaceae	*Alnus*	*Alnus japonica*	FJ844560
Eudicotyledons	Betulaceae	*Alnus*	*Alnus japonica*	JN043796
Eudicotyledons	Betulaceae	*Alnus*	*Alnus japonica*	JN043797
Eudicotyledons	Betulaceae	*Alnus*	*Alnus japonica*	JN043798
Eudicotyledons	Betulaceae	*Alnus*	*Alnus japonica*	JN043799
Eudicotyledons	Betulaceae	*Alnus*	*Alnus japonica*	JN043800
Eudicotyledons	Betulaceae	*Alnus*	*Alnus japonica*	JN043801
Eudicotyledons	Betulaceae	*Alnus*	*Alnus japonica*	JN043802
Eudicotyledons	Betulaceae	*Alnus*	*Alnus japonica*	JN043803
Eudicotyledons	Betulaceae	*Alnus*	*Alnus japonica*	JN043804
Eudicotyledons	Betulaceae	*Alnus*	*Alnus matsumurae*	FJ844518
Eudicotyledons	Betulaceae	*Alnus*	*Alnus matsumurae*	FJ844519
Eudicotyledons	Betulaceae	*Alnus*	*Alnus nepalensis*	FJ011871
Eudicotyledons	Betulaceae	*Alnus*	*Alnus nepalensis*	FJ011872
Eudicotyledons	Betulaceae	*Alnus*	*Alnus nepalensis*	FJ844511
Eudicotyledons	Betulaceae	*Alnus*	*Alnus nepalensis*	FJ844512
Eudicotyledons	Betulaceae	*Alnus*	*Alnus nepalensis*	FJ844513
Eudicotyledons	Betulaceae	*Alnus*	*Alnus nepalensis*	FJ844514
Eudicotyledons	Betulaceae	*Alnus*	*Alnus nepalensis*	FJ844515
Eudicotyledons	Betulaceae	*Alnus*	*Alnus nepalensis*	JN043807
Eudicotyledons	Betulaceae	*Alnus*	*Alnus pendula*	FJ844489
Eudicotyledons	Betulaceae	*Alnus*	*Alnus pendula*	FJ844490
Eudicotyledons	Betulaceae	*Alnus*	*Alnus pendula*	FJ844491
Eudicotyledons	Betulaceae	*Alnus*	*Alnus pendula*	FJ844492
Eudicotyledons	Betulaceae	*Alnus*	*Alnus rhombifolia*	JN043808
Eudicotyledons	Betulaceae	*Alnus*	*Alnus rhombifolia*	JN043809
Eudicotyledons	Betulaceae	*Alnus*	*Alnus rubra*	FJ844562
Eudicotyledons	Betulaceae	*Alnus*	*Alnus rubra*	FJ844563
Eudicotyledons	Betulaceae	*Alnus*	*Alnus rubra*	JN043810
Eudicotyledons	Betulaceae	*Alnus*	*Alnus subcordata*	FJ844540
Eudicotyledons	Betulaceae	*Alnus*	*Alnus subcordata*	FJ844541
Eudicotyledons	Betulaceae	*Alnus*	*Alnus nitida*	FJ844508
Eudicotyledons	Betulaceae	*Alnus*	*Alnus nitida*	FJ844509
Eudicotyledons	Betulaceae	*Alnus*	*Alnus nitida*	FJ844510
Eudicotyledons	Crassulaceae	*Aeonium*	*Aeonium balsamiferum*	AY082192
Eudicotyledons	Crassulaceae	*Aeonium*	*Aeonium balsamiferum*	AY082202
Eudicotyledons	Solanaceae	*Lycium*	*Lycium americanum*	FJ189606
Eudicotyledons	Solanaceae	*Lycium*	*Lycium americanum*	EU742440
Eudicotyledons	Solanaceae	*Lycium*	*Lycium americanum*	HQ216167
Eudicotyledons	Solanaceae	*Lycium*	*Lycium barbarum*	HM195003
Eudicotyledons	Solanaceae	*Lycium*	*Lycium barbarum*	HM195004
Eudicotyledons	Solanaceae	*Lycium*	*Lycium barbarum*	JN045282
Eudicotyledons	Solanaceae	*Lycium*	*Lycium barbarum*	JN045283
Eudicotyledons	Solanaceae	*Lycium*	*Lycium pallidum*	JF284435
Eudicotyledons	Solanaceae	*Lycium*	*Lycium pallidum*	JF284436
Eudicotyledons	Solanaceae	*Lycium*	*Lycium ruthenicum*	HM195011
Eudicotyledons	Solanaceae	*Lycium*	*Lycium ruthenicum*	HM195012
Eudicotyledons	Solanaceae	*Lycium*	*Lycium ruthenicum*	JN045288
Eudicotyledons	Solanaceae	*Lycium*	*Lycium ruthenicum*	JN045289
Eudicotyledons	Solanaceae	*Lycium*	*Lycium ruthenicum*	JN045290
Eudicotyledons	Solanaceae	*Lycium*	*Lycium villosum*	HM195017

Eudicotyledons	Solanaceae	*Lycium*	*Lycium villosum*	FJ189626
Eudicotyledons	Solanaceae	*Lycium*	*Lycium chinense*	HM195006
Eudicotyledons	Solanaceae	*Lycium*	*Lycium chinense*	HM195007
Eudicotyledons	Solanaceae	*Lycium*	*Lycium chinense*	GQ435281
Eudicotyledons	Solanaceae	*Lycium*	*Lycium chinense*	JN045284
Eudicotyledons	Solanaceae	*Lycium*	*Lycium chinense*	JN045285
Eudicotyledons	Solanaceae	*Lycium*	*Lycium chinense*	JN045286
Eudicotyledons	Solanaceae	*Lycium*	*Lycium chinense*	JN045287
Eudicotyledons	Solanaceae	*Lycium*	*Lycium chinense*	HQ216168
Eudicotyledons	Fagaceae	*Fagus*	*Fagus hayatae*	JN044497
Eudicotyledons	Fagaceae	*Fagus*	*Fagus hayatae*	JN044498
Eudicotyledons	Fagaceae	*Fagus*	*Fagus hayatae*	JN044499
Eudicotyledons	Fagaceae	*Fagus*	*Fagus hayatae*	JN044500
Eudicotyledons	Verbenaceae	*Verbena*	*Verbena urticifolia*	HQ596882
Eudicotyledons	Verbenaceae	*Verbena*	*Verbena urticifolia*	DQ006231
Eudicotyledons	Rosaceae	*Crataegus*	*Crataegus monogyna*	EF127128
Eudicotyledons	Rosaceae	*Crataegus*	*Crataegus monogyna*	JQ390769
Eudicotyledons	Rosaceae	*Crataegus*	*Crataegus monogyna*	FJ395483
Eudicotyledons	Rosaceae	*Crataegus*	*Crataegus monogyna*	EU500288
Eudicotyledons	Rosaceae	*Crataegus*	*Crataegus monogyna*	FJ493304
Eudicotyledons	Rosaceae	*Photinia*	*Photinia serratifolia*	HQ427050
Eudicotyledons	Rosaceae	*Photinia*	*Photinia serratifolia*	GQ305350
Eudicotyledons	Rosaceae	*Sorbaria*	*Sorbaria sorbifolia*	GQ435278
Eudicotyledons	Rosaceae	*Sorbaria*	*Sorbaria sorbifolia*	JN047240
Eudicotyledons	Rosaceae	*Sorbaria*	*Sorbaria sorbifolia*	JN047241
Eudicotyledons	Apocynaceae	*Asclepias*	*Asclepias tuberosa*	GQ248251
Eudicotyledons	Apocynaceae	*Asclepias*	*Asclepias tuberosa*	EF590674
Eudicotyledons	Ranunculaceae	*Ranunculus*	*Ranunculus crithmifolius*	FJ744168
Eudicotyledons	Ranunculaceae	*Ranunculus*	*Ranunculus crithmifolius*	FJ744169
Eudicotyledons	Ranunculaceae	*Ranunculus*	*Ranunculus crithmifolius*	FJ744170
Eudicotyledons	Ranunculaceae	*Ranunculus*	*Ranunculus insignis*	FJ744174
Eudicotyledons	Ranunculaceae	*Ranunculus*	*Ranunculus insignis*	FJ744175
Eudicotyledons	Ranunculaceae	*Ranunculus*	*Ranunculus insignis*	FJ744176
Eudicotyledons	Ranunculaceae	*Ranunculus*	*Ranunculus lyallii*	FJ744177
Eudicotyledons	Ranunculaceae	*Ranunculus*	*Ranunculus lyallii*	FJ744178
Eudicotyledons	Ranunculaceae	*Ranunculus*	*Ranunculus lyallii*	FJ744179
Eudicotyledons	Ranunculaceae	*Ranunculus*	*Ranunculus sericophyllus*	FJ744180
Eudicotyledons	Ranunculaceae	*Ranunculus*	*Ranunculus sericophyllus*	FJ744181
Eudicotyledons	Hydrangeaceae	*Hydrangea*	*Hydrangea anomala*	JN044919
Eudicotyledons	Hydrangeaceae	*Hydrangea*	*Hydrangea anomala*	JN044920
Eudicotyledons	Hydrangeaceae	*Hydrangea*	*Hydrangea anomala*	JN044921
Eudicotyledons	Hydrangeaceae	*Hydrangea*	*Hydrangea anomala*	HM216965
Eudicotyledons	Hydrangeaceae	*Hydrangea*	*Hydrangea peruviana*	HM216985
Eudicotyledons	Hydrangeaceae	*Hydrangea*	*Hydrangea peruviana*	JF321260
Eudicotyledons	Brassicaceae	*Lepidium*	*Lepidium perfoliatum*	JN045142
Eudicotyledons	Brassicaceae	*Lepidium*	*Lepidium perfoliatum*	JN045143
Eudicotyledons	Brassicaceae	*Lepidium*	*Lepidium perfoliatum*	JN045144
Eudicotyledons	Brassicaceae	*Lepidium*	*Lepidium ruderale*	JN045145
Eudicotyledons	Brassicaceae	*Lepidium*	*Lepidium ruderale*	JN045146
Eudicotyledons	Brassicaceae	*Lepidium*	*Lepidium ruderale*	JN045147
Eudicotyledons	Brassicaceae	*Lepidium*	*Lepidium ruderale*	JN045148
Eudicotyledons	Brassicaceae	*Lepidium*	*Lepidium apetalum*	GQ435334
Eudicotyledons	Brassicaceae	*Lepidium*	*Lepidium apetalum*	JN045126

Eudicotyledons	Brassicaceae	*Lepidium*	*Lepidium apetalum*	JN045127
Eudicotyledons	Brassicaceae	*Lepidium*	*Lepidium apetalum*	JN045128
Eudicotyledons	Brassicaceae	*Lepidium*	*Lepidium apetalum*	JN045129
Eudicotyledons	Brassicaceae	*Lepidium*	*Lepidium apetalum*	JN045130
Eudicotyledons	Brassicaceae	*Lepidium*	*Lepidium apetalum*	JN045131
Eudicotyledons	Brassicaceae	*Lepidium*	*Lepidium apetalum*	JN045132
Eudicotyledons	Brassicaceae	*Lepidium*	*Lepidium apetalum*	JN045133
Eudicotyledons	Brassicaceae	*Lepidium*	*Lepidium apetalum*	JN045134
Eudicotyledons	Brassicaceae	*Lepidium*	*Lepidium apetalum*	JN045135
Eudicotyledons	Brassicaceae	*Lepidium*	*Lepidium apetalum*	JN045136
Eudicotyledons	Brassicaceae	*Lepidium*	*Lepidium apetalum*	JN045137
Eudicotyledons	Brassicaceae	*Lepidium*	*Lepidium apetalum*	JN045138
Eudicotyledons	Brassicaceae	*Lepidium*	*Lepidium apetalum*	JN045139
Eudicotyledons	Brassicaceae	*Lepidium*	*Lepidium apetalum*	JN045140
Eudicotyledons	Brassicaceae	*Lepidium*	*Lepidium apetalum*	JN045141
Eudicotyledons	Solanaceae	*Lycium*	*Lycium shawii*	HM195013
Eudicotyledons	Solanaceae	*Lycium*	*Lycium shawii*	HM195014
Eudicotyledons	Asteraceae	*Cota*	*Cota triumfettii*	AB683351
Eudicotyledons	Asteraceae	*Cota*	*Cota triumfettii*	FR689781
Eudicotyledons	Asteraceae	*Cota*	*Cota triumfettii*	FR689789
Eudicotyledons	Asteraceae	*Cota*	*Cota triumfettii*	FR689804
Eudicotyledons	Fabaceae	*Schotia*	*Schotia afra*	GQ405086
Eudicotyledons	Fabaceae	*Schotia*	*Schotia afra*	GQ405087
Eudicotyledons	Fabaceae	*Schotia*	*Schotia afra*	GQ405088
Eudicotyledons	Fabaceae	*Schotia*	*Schotia afra*	GQ405089
Eudicotyledons	Fabaceae	*Schotia*	*Schotia afra*	GQ405090
Eudicotyledons	Fabaceae	*Schotia*	*Schotia afra*	GQ405094
Eudicotyledons	Fabaceae	*Schotia*	*Schotia afra*	GQ405095
Eudicotyledons	Fabaceae	*Schotia*	*Schotia afra*	GQ405097
Eudicotyledons	Fabaceae	*Schotia*	*Schotia afra*	GQ405099
Eudicotyledons	Fabaceae	*Schotia*	*Schotia afra*	GQ405100
Eudicotyledons	Fabaceae	*Schotia*	*Schotia afra*	GQ405101
Eudicotyledons	Fabaceae	*Schotia*	*Schotia afra*	GQ405105
Eudicotyledons	Fabaceae	*Schotia*	*Schotia afra*	GQ405106
Eudicotyledons	Fabaceae	*Schotia*	*Schotia afra*	GQ405107
Eudicotyledons	Fabaceae	*Schotia*	*Schotia afra*	GQ405110
Eudicotyledons	Fabaceae	*Schotia*	*Schotia afra*	GQ405113
Eudicotyledons	Fabaceae	*Schotia*	*Schotia afra*	GQ405114
Eudicotyledons	Fabaceae	*Schotia*	*Schotia afra*	GQ405115
Eudicotyledons	Fabaceae	*Schotia*	*Schotia afra*	GQ405123
Eudicotyledons	Fabaceae	*Schotia*	*Schotia afra*	GQ405124
Eudicotyledons	Fabaceae	*Schotia*	*Schotia afra*	GQ405127
Eudicotyledons	Fabaceae	*Schotia*	*Schotia afra*	GQ405128
Eudicotyledons	Fabaceae	*Schotia*	*Schotia afra*	GQ405129
Eudicotyledons	Fabaceae	*Schotia*	*Schotia latifolia*	GQ405091
Eudicotyledons	Fabaceae	*Schotia*	*Schotia latifolia*	GQ405093
Eudicotyledons	Fabaceae	*Schotia*	*Schotia latifolia*	GQ405096
Eudicotyledons	Fabaceae	*Schotia*	*Schotia latifolia*	GQ405102
Eudicotyledons	Fabaceae	*Schotia*	*Schotia latifolia*	GQ405111
Eudicotyledons	Fabaceae	*Schotia*	*Schotia latifolia*	GQ405112
Eudicotyledons	Fabaceae	*Schotia*	*Schotia latifolia*	GQ405116
Eudicotyledons	Fabaceae	*Schotia*	*Schotia latifolia*	GQ405118
Eudicotyledons	Fabaceae	*Schotia*	*Schotia latifolia*	GQ405119

Eudicotyledons	Fabaceae	*Schotia*	*Schotia latifolia*	GQ405120
Eudicotyledons	Fabaceae	*Schotia*	*Schotia latifolia*	GQ405126
Eudicotyledons	Fagaceae	*Fagus*	*Fagus engleriana*	JN044492
Eudicotyledons	Fagaceae	*Fagus*	*Fagus engleriana*	JN044493
Eudicotyledons	Fagaceae	*Fagus*	*Fagus engleriana*	JN044494
Eudicotyledons	Fagaceae	*Fagus*	*Fagus engleriana*	JN044495
Eudicotyledons	Fagaceae	*Fagus*	*Fagus engleriana*	JN044496
Eudicotyledons	Fagaceae	*Fagus*	*Fagus lucida*	JN044504
Eudicotyledons	Fagaceae	*Fagus*	*Fagus lucida*	JN044505
Eudicotyledons	Fagaceae	*Fagus*	*Fagus lucida*	JN044506
Eudicotyledons	Fagaceae	*Fagus*	*Fagus lucida*	JN044507
Eudicotyledons	Fagaceae	*Fagus*	*Fagus lucida*	JN044508
Eudicotyledons	Fagaceae	*Fagus*	*Fagus longipetiolata*	JN044501
Eudicotyledons	Fagaceae	*Fagus*	*Fagus longipetiolata*	JN044502
Eudicotyledons	Fagaceae	*Fagus*	*Fagus longipetiolata*	JN044503
Eudicotyledons	Ranunculaceae	*Anemone*	*Anemone virginiana*	HQ596597
Eudicotyledons	Ranunculaceae	*Anemone*	*Anemone virginiana*	DQ006220
Eudicotyledons	Rutaceae	*Citrus*	*Citrus medica*	GQ435453
Eudicotyledons	Rutaceae	*Citrus*	*Citrus medica*	GQ435454
Eudicotyledons	Rosaceae	*Rubus*	*Rubus parvifolius*	GQ435257
Eudicotyledons	Rosaceae	*Rubus*	*Rubus parvifolius*	JN407048
Eudicotyledons	Rosaceae	*Rubus*	*Rubus parvifolius*	JN407049
Eudicotyledons	Lamiaceae	*Salvia*	*Salvia splendens*	JQ339257
Eudicotyledons	Lamiaceae	*Salvia*	*Salvia splendens*	FJ513111
Eudicotyledons	Moraceae	*Ficus*	*Ficus benjamina*	GU935092
Eudicotyledons	Moraceae	*Ficus*	*Ficus benjamina*	GU935093
Eudicotyledons	Moraceae	*Ficus*	*Ficus benjamina*	FN675798
Eudicotyledons	Moraceae	*Ficus*	*Ficus benjamina*	JN044511
Eudicotyledons	Moraceae	*Ficus*	*Ficus benjamina*	JN044512
Eudicotyledons	Moraceae	*Ficus*	*Ficus glumosa*	EU213822
Eudicotyledons	Moraceae	*Ficus*	*Ficus glumosa*	EU213823
Eudicotyledons	Moraceae	*Ficus*	*Ficus glumosa*	EU213824
Eudicotyledons	Moraceae	*Ficus*	*Ficus sycomorus*	EU213825
Eudicotyledons	Moraceae	*Ficus*	*Ficus sycomorus*	EU213826
Eudicotyledons	Moraceae	*Ficus*	*Ficus sycomorus*	EU213827
Eudicotyledons	Lamiaceae	*Clinopodium*	*Clinopodium coccineum*	DQ667344
Eudicotyledons	Lamiaceae	*Clinopodium*	*Clinopodium coccineum*	AY943555
Eudicotyledons	Lamiaceae	*Conradina*	*Conradina etonia*	AY943539
Eudicotyledons	Lamiaceae	*Conradina*	*Conradina etonia*	AY943540
Eudicotyledons	Asteraceae	*Encelia*	*Encelia californica*	DQ383890
Eudicotyledons	Asteraceae	*Encelia*	*Encelia californica*	AY215539
Eudicotyledons	Asteraceae	*Melampodium*	*Melampodium leucanthum*	AY215575
Eudicotyledons	Asteraceae	*Melampodium*	*Melampodium leucanthum*	FJ846087
Eudicotyledons	Asteraceae	*Melampodium*	*Melampodium leucanthum*	FJ846088
Eudicotyledons	Asteraceae	*Melampodium*	*Melampodium leucanthum*	FJ846089
Eudicotyledons	Asteraceae	*Melampodium*	*Melampodium leucanthum*	FJ846090
Eudicotyledons	Asteraceae	*Melampodium*	*Melampodium leucanthum*	FJ846091
Eudicotyledons	Asteraceae	*Melampodium*	*Melampodium leucanthum*	FJ846092
Eudicotyledons	Asteraceae	*Melampodium*	*Melampodium leucanthum*	FJ846093
Eudicotyledons	Asteraceae	*Melampodium*	*Melampodium leucanthum*	FJ846094
Eudicotyledons	Asteraceae	*Melampodium*	*Melampodium leucanthum*	FJ846095
Eudicotyledons	Asteraceae	*Melampodium*	*Melampodium leucanthum*	FJ846096
Eudicotyledons	Asteraceae	*Melampodium*	*Melampodium leucanthum*	FJ846097

Eudicotyledons	Asteraceae	*Melampodium*	*Melampodium leucanthum*	FJ846098
Eudicotyledons	Asteraceae	*Melampodium*	*Melampodium leucanthum*	FJ846099
Eudicotyledons	Asteraceae	*Melampodium*	*Melampodium leucanthum*	FJ846100
Eudicotyledons	Asteraceae	*Melampodium*	*Melampodium leucanthum*	FJ846101
Eudicotyledons	Asteraceae	*Melampodium*	*Melampodium leucanthum*	FJ846102
Eudicotyledons	Asteraceae	*Melampodium*	*Melampodium leucanthum*	FJ846103
Eudicotyledons	Asteraceae	*Melampodium*	*Melampodium leucanthum*	FJ846104
Eudicotyledons	Asteraceae	*Melampodium*	*Melampodium leucanthum*	FJ846105
Eudicotyledons	Asteraceae	*Melampodium*	*Melampodium leucanthum*	FJ846106
Eudicotyledons	Asteraceae	*Melampodium*	*Melampodium leucanthum*	FJ846107
Eudicotyledons	Asteraceae	*Melampodium*	*Melampodium leucanthum*	FJ846108
Eudicotyledons	Asteraceae	*Melampodium*	*Melampodium leucanthum*	FJ846109
Eudicotyledons	Asteraceae	*Melampodium*	*Melampodium leucanthum*	FJ846110
Eudicotyledons	Asteraceae	*Melampodium*	*Melampodium leucanthum*	FJ846111
Eudicotyledons	Asteraceae	*Melampodium*	*Melampodium leucanthum*	FJ846112
Eudicotyledons	Asteraceae	*Melampodium*	*Melampodium leucanthum*	FJ846113
Eudicotyledons	Asteraceae	*Melampodium*	*Melampodium leucanthum*	FJ846114
Eudicotyledons	Asteraceae	*Melampodium*	*Melampodium leucanthum*	FJ846115
Eudicotyledons	Asteraceae	*Melampodium*	*Melampodium leucanthum*	FJ846116
Eudicotyledons	Asteraceae	*Melampodium*	*Melampodium leucanthum*	FJ846117
Eudicotyledons	Asteraceae	*Melampodium*	*Melampodium leucanthum*	FJ846118
Eudicotyledons	Asteraceae	*Melampodium*	*Melampodium leucanthum*	FJ846119
Eudicotyledons	Asteraceae	*Melampodium*	*Melampodium leucanthum*	FJ846120
Eudicotyledons	Asteraceae	*Melampodium*	*Melampodium leucanthum*	FJ846121
Eudicotyledons	Asteraceae	*Melampodium*	*Melampodium leucanthum*	FJ846122
Eudicotyledons	Asteraceae	*Melampodium*	*Melampodium leucanthum*	FJ846123
Eudicotyledons	Asteraceae	*Melampodium*	*Melampodium leucanthum*	FJ846124
Eudicotyledons	Asteraceae	*Melampodium*	*Melampodium leucanthum*	FJ846125
Eudicotyledons	Asteraceae	*Melampodium*	*Melampodium leucanthum*	FJ846126
Eudicotyledons	Asteraceae	*Melampodium*	*Melampodium leucanthum*	FJ846127
Eudicotyledons	Asteraceae	*Melampodium*	*Melampodium leucanthum*	FJ846128
Eudicotyledons	Asteraceae	*Melampodium*	*Melampodium leucanthum*	FJ846129
Eudicotyledons	Asteraceae	*Melampodium*	*Melampodium leucanthum*	FJ846130
Eudicotyledons	Asteraceae	*Melampodium*	*Melampodium leucanthum*	FJ846131
Eudicotyledons	Asteraceae	*Melampodium*	*Melampodium leucanthum*	FJ846132
Eudicotyledons	Asteraceae	*Melampodium*	*Melampodium leucanthum*	FJ846133
Eudicotyledons	Asteraceae	*Melampodium*	*Melampodium leucanthum*	FJ846134
Eudicotyledons	Asteraceae	*Melampodium*	*Melampodium leucanthum*	FJ846135
Eudicotyledons	Asteraceae	*Melampodium*	*Melampodium leucanthum*	FJ846136
Eudicotyledons	Asteraceae	*Melampodium*	*Melampodium leucanthum*	FJ846137
Eudicotyledons	Asteraceae	*Melampodium*	*Melampodium leucanthum*	FJ846138
Eudicotyledons	Asteraceae	*Melampodium*	*Melampodium leucanthum*	FJ846139
Eudicotyledons	Asteraceae	*Melampodium*	*Melampodium leucanthum*	FJ846140
Eudicotyledons	Asteraceae	*Melampodium*	*Melampodium leucanthum*	FJ846141
Eudicotyledons	Asteraceae	*Melampodium*	*Melampodium leucanthum*	FJ846142
Eudicotyledons	Asteraceae	*Melampodium*	*Melampodium leucanthum*	FJ846143
Eudicotyledons	Asteraceae	*Melampodium*	*Melampodium leucanthum*	FJ846144
Eudicotyledons	Asteraceae	*Melampodium*	*Melampodium leucanthum*	FJ846145
Eudicotyledons	Asteraceae	*Melampodium*	*Melampodium leucanthum*	FJ846146
Eudicotyledons	Asteraceae	*Melampodium*	*Melampodium leucanthum*	FJ846147
Eudicotyledons	Asteraceae	*Melampodium*	*Melampodium leucanthum*	FJ846148
Eudicotyledons	Asteraceae	*Melampodium*	*Melampodium leucanthum*	FJ846149
Eudicotyledons	Asteraceae	*Melampodium*	*Melampodium leucanthum*	FJ846150

Eudicotyledons	Asteraceae	*Melampodium*	*Melampodium leucanthum*	FJ846151
Eudicotyledons	Asteraceae	*Melampodium*	*Melampodium leucanthum*	FJ846152
Eudicotyledons	Asteraceae	*Melampodium*	*Melampodium leucanthum*	FJ846153
Eudicotyledons	Asteraceae	*Melampodium*	*Melampodium leucanthum*	FJ846154
Eudicotyledons	Asteraceae	*Melampodium*	*Melampodium leucanthum*	FJ846155
Eudicotyledons	Asteraceae	*Melampodium*	*Melampodium leucanthum*	FJ846156
Eudicotyledons	Asteraceae	*Melampodium*	*Melampodium leucanthum*	FJ846157
Eudicotyledons	Asteraceae	*Melampodium*	*Melampodium leucanthum*	FJ846158
Eudicotyledons	Asteraceae	*Melampodium*	*Melampodium leucanthum*	FJ846159
Eudicotyledons	Asteraceae	*Melampodium*	*Melampodium leucanthum*	FJ846160
Eudicotyledons	Asteraceae	*Melampodium*	*Melampodium leucanthum*	FJ846161
Eudicotyledons	Asteraceae	*Melampodium*	*Melampodium leucanthum*	FJ846162
Eudicotyledons	Asteraceae	*Melampodium*	*Melampodium leucanthum*	FJ846163
Eudicotyledons	Asteraceae	*Melampodium*	*Melampodium leucanthum*	FJ846164
Eudicotyledons	Asteraceae	*Melampodium*	*Melampodium leucanthum*	FJ846165
Eudicotyledons	Asteraceae	*Melampodium*	*Melampodium leucanthum*	FJ846166
Eudicotyledons	Asteraceae	*Melampodium*	*Melampodium leucanthum*	FJ846167
Eudicotyledons	Asteraceae	*Melampodium*	*Melampodium leucanthum*	FJ846168
Eudicotyledons	Asteraceae	*Melampodium*	*Melampodium leucanthum*	FJ846169
Eudicotyledons	Asteraceae	*Melampodium*	*Melampodium leucanthum*	FJ846170
Eudicotyledons	Asteraceae	*Melampodium*	*Melampodium leucanthum*	FJ846171
Eudicotyledons	Asteraceae	*Melampodium*	*Melampodium leucanthum*	FJ846172
Eudicotyledons	Asteraceae	*Melampodium*	*Melampodium leucanthum*	FJ846173
Eudicotyledons	Asteraceae	*Melampodium*	*Melampodium leucanthum*	FJ846174
Eudicotyledons	Asteraceae	*Melampodium*	*Melampodium leucanthum*	FJ846175
Eudicotyledons	Asteraceae	*Melampodium*	*Melampodium leucanthum*	FJ846176
Eudicotyledons	Asteraceae	*Melampodium*	*Melampodium leucanthum*	FJ846177
Eudicotyledons	Asteraceae	*Melampodium*	*Melampodium leucanthum*	FJ846178
Eudicotyledons	Asteraceae	*Melampodium*	*Melampodium leucanthum*	FJ846179
Eudicotyledons	Asteraceae	*Melampodium*	*Melampodium leucanthum*	FJ846180
Eudicotyledons	Asteraceae	*Melampodium*	*Melampodium leucanthum*	FJ846181
Eudicotyledons	Asteraceae	*Melampodium*	*Melampodium leucanthum*	FJ846182
Eudicotyledons	Asteraceae	*Melampodium*	*Melampodium leucanthum*	FJ846183
Eudicotyledons	Asteraceae	*Melampodium*	*Melampodium leucanthum*	FJ846184
Eudicotyledons	Asteraceae	*Melampodium*	*Melampodium leucanthum*	FJ846185
Eudicotyledons	Asteraceae	*Melampodium*	*Melampodium leucanthum*	FJ846186
Eudicotyledons	Asteraceae	*Melampodium*	*Melampodium leucanthum*	FJ846187
Eudicotyledons	Asteraceae	*Melampodium*	*Melampodium leucanthum*	FJ846188
Eudicotyledons	Asteraceae	*Melampodium*	*Melampodium leucanthum*	FJ846189
Eudicotyledons	Asteraceae	*Melampodium*	*Melampodium leucanthum*	FJ846190
Eudicotyledons	Asteraceae	*Melampodium*	*Melampodium leucanthum*	FJ846191
Eudicotyledons	Asteraceae	*Melampodium*	*Melampodium leucanthum*	FJ846192
Eudicotyledons	Asteraceae	*Melampodium*	*Melampodium leucanthum*	FJ846193
Eudicotyledons	Asteraceae	*Melampodium*	*Melampodium leucanthum*	FJ846194
Eudicotyledons	Asteraceae	*Melampodium*	*Melampodium leucanthum*	FJ846195
Eudicotyledons	Asteraceae	*Melampodium*	*Melampodium leucanthum*	FJ846196
Eudicotyledons	Asteraceae	*Melampodium*	*Melampodium leucanthum*	FJ846197
Eudicotyledons	Asteraceae	*Melampodium*	*Melampodium leucanthum*	FJ846198
Eudicotyledons	Asteraceae	*Melampodium*	*Melampodium leucanthum*	FJ846199
Eudicotyledons	Asteraceae	*Melampodium*	*Melampodium leucanthum*	FJ846200
Eudicotyledons	Asteraceae	*Melampodium*	*Melampodium leucanthum*	FJ846201
Eudicotyledons	Asteraceae	*Melampodium*	*Melampodium leucanthum*	FJ846202
Eudicotyledons	Asteraceae	*Melampodium*	*Melampodium leucanthum*	FJ846203

Eudicotyledons	Asteraceae	*Melampodium*	*Melampodium leucanthum*	FJ846204
Eudicotyledons	Asteraceae	*Melampodium*	*Melampodium leucanthum*	FJ846205
Eudicotyledons	Asteraceae	*Melampodium*	*Melampodium leucanthum*	FJ846206
Eudicotyledons	Asteraceae	*Melampodium*	*Melampodium leucanthum*	FJ846207
Eudicotyledons	Asteraceae	*Melampodium*	*Melampodium leucanthum*	FJ846208
Eudicotyledons	Asteraceae	*Melampodium*	*Melampodium leucanthum*	FJ846209
Eudicotyledons	Asteraceae	*Melampodium*	*Melampodium leucanthum*	FJ846210
Eudicotyledons	Asteraceae	*Melampodium*	*Melampodium leucanthum*	FJ846211
Eudicotyledons	Asteraceae	*Melampodium*	*Melampodium leucanthum*	FJ846212
Eudicotyledons	Asteraceae	*Melampodium*	*Melampodium leucanthum*	FJ846213
Eudicotyledons	Asteraceae	*Melampodium*	*Melampodium leucanthum*	FJ846214
Eudicotyledons	Asteraceae	*Melampodium*	*Melampodium leucanthum*	FJ846215
Eudicotyledons	Asteraceae	*Melampodium*	*Melampodium leucanthum*	FJ846216
Eudicotyledons	Asteraceae	*Melampodium*	*Melampodium leucanthum*	FJ846217
Eudicotyledons	Asteraceae	*Melampodium*	*Melampodium leucanthum*	FJ846218
Eudicotyledons	Asteraceae	*Melampodium*	*Melampodium leucanthum*	FJ846219
Eudicotyledons	Asteraceae	*Melampodium*	*Melampodium leucanthum*	FJ846220
Eudicotyledons	Asteraceae	*Melampodium*	*Melampodium leucanthum*	FJ846221
Eudicotyledons	Asteraceae	*Melampodium*	*Melampodium leucanthum*	FJ846222
Eudicotyledons	Asteraceae	*Melampodium*	*Melampodium leucanthum*	FJ846223
Eudicotyledons	Asteraceae	*Melampodium*	*Melampodium leucanthum*	FJ846224
Eudicotyledons	Asteraceae	*Melampodium*	*Melampodium leucanthum*	FJ846225
Eudicotyledons	Asteraceae	*Melampodium*	*Melampodium leucanthum*	FJ846226
Eudicotyledons	Asteraceae	*Melampodium*	*Melampodium leucanthum*	FJ846227
Eudicotyledons	Asteraceae	*Melampodium*	*Melampodium leucanthum*	FJ846228
Eudicotyledons	Asteraceae	*Melampodium*	*Melampodium leucanthum*	FJ846229
Eudicotyledons	Asteraceae	*Melampodium*	*Melampodium leucanthum*	FJ846230
Eudicotyledons	Asteraceae	*Melampodium*	*Melampodium leucanthum*	FJ846231
Eudicotyledons	Asteraceae	*Melampodium*	*Melampodium leucanthum*	FJ846232
Eudicotyledons	Asteraceae	*Melampodium*	*Melampodium leucanthum*	FJ846233
Eudicotyledons	Asteraceae	*Melampodium*	*Melampodium leucanthum*	FJ846234
Eudicotyledons	Asteraceae	*Melampodium*	*Melampodium leucanthum*	FJ846235
Eudicotyledons	Asteraceae	*Melampodium*	*Melampodium leucanthum*	FJ846236
Eudicotyledons	Asteraceae	*Melampodium*	*Melampodium leucanthum*	FJ846237
Eudicotyledons	Asteraceae	*Melampodium*	*Melampodium leucanthum*	FJ846238
Eudicotyledons	Asteraceae	*Melampodium*	*Melampodium leucanthum*	FJ846239
Eudicotyledons	Asteraceae	*Melampodium*	*Melampodium leucanthum*	FJ846240
Eudicotyledons	Asteraceae	*Melampodium*	*Melampodium leucanthum*	FJ846241
Eudicotyledons	Asteraceae	*Melampodium*	*Melampodium leucanthum*	FJ846242
Eudicotyledons	Asteraceae	*Melampodium*	*Melampodium leucanthum*	FJ846243
Eudicotyledons	Asteraceae	*Melampodium*	*Melampodium leucanthum*	FJ846244
Eudicotyledons	Asteraceae	*Melampodium*	*Melampodium leucanthum*	FJ846245
Eudicotyledons	Asteraceae	*Melampodium*	*Melampodium leucanthum*	FJ846246
Eudicotyledons	Asteraceae	*Melampodium*	*Melampodium leucanthum*	FJ846247
Eudicotyledons	Asteraceae	*Melampodium*	*Melampodium leucanthum*	FJ846248
Eudicotyledons	Asteraceae	*Melampodium*	*Melampodium leucanthum*	FJ846249
Eudicotyledons	Asteraceae	*Melampodium*	*Melampodium leucanthum*	FJ846250
Eudicotyledons	Asteraceae	*Melampodium*	*Melampodium leucanthum*	FJ846251
Eudicotyledons	Asteraceae	*Melampodium*	*Melampodium leucanthum*	FJ846252
Eudicotyledons	Asteraceae	*Melampodium*	*Melampodium leucanthum*	FJ846253
Eudicotyledons	Asteraceae	*Melampodium*	*Melampodium leucanthum*	FJ846254
Eudicotyledons	Asteraceae	*Melampodium*	*Melampodium leucanthum*	FJ846255
Eudicotyledons	Asteraceae	*Melampodium*	*Melampodium leucanthum*	FJ846256

Eudicotyledons	Asteraceae	*Melampodium*	*Melampodium leucanthum*	FJ846257
Eudicotyledons	Asteraceae	*Melampodium*	*Melampodium leucanthum*	FJ846258
Eudicotyledons	Asteraceae	*Melampodium*	*Melampodium leucanthum*	FJ846259
Eudicotyledons	Asteraceae	*Melampodium*	*Melampodium leucanthum*	FJ846260
Eudicotyledons	Asteraceae	*Melampodium*	*Melampodium leucanthum*	FJ846261
Eudicotyledons	Asteraceae	*Melampodium*	*Melampodium leucanthum*	FJ846262
Eudicotyledons	Asteraceae	*Melampodium*	*Melampodium leucanthum*	FJ846263
Eudicotyledons	Asteraceae	*Melampodium*	*Melampodium leucanthum*	FJ846264
Eudicotyledons	Asteraceae	*Melampodium*	*Melampodium leucanthum*	FJ846265
Eudicotyledons	Asteraceae	*Melampodium*	*Melampodium leucanthum*	FJ846266
Eudicotyledons	Asteraceae	*Melampodium*	*Melampodium leucanthum*	FJ846267
Eudicotyledons	Asteraceae	*Melampodium*	*Melampodium leucanthum*	FJ846268
Eudicotyledons	Asteraceae	*Melampodium*	*Melampodium leucanthum*	FJ846269
Eudicotyledons	Asteraceae	*Melampodium*	*Melampodium leucanthum*	FJ846270
Eudicotyledons	Asteraceae	*Melampodium*	*Melampodium leucanthum*	FJ846271
Eudicotyledons	Asteraceae	*Melampodium*	*Melampodium leucanthum*	FJ846272
Eudicotyledons	Asteraceae	*Melampodium*	*Melampodium leucanthum*	FJ846273
Eudicotyledons	Asteraceae	*Melampodium*	*Melampodium leucanthum*	FJ846274
Eudicotyledons	Asteraceae	*Melampodium*	*Melampodium leucanthum*	FJ846275
Eudicotyledons	Asteraceae	*Melampodium*	*Melampodium leucanthum*	FJ846276
Eudicotyledons	Asteraceae	*Melampodium*	*Melampodium leucanthum*	FJ846277
Eudicotyledons	Asteraceae	*Melampodium*	*Melampodium leucanthum*	FJ846278
Eudicotyledons	Asteraceae	*Melampodium*	*Melampodium leucanthum*	FJ846279
Eudicotyledons	Asteraceae	*Melampodium*	*Melampodium leucanthum*	FJ846280
Eudicotyledons	Asteraceae	*Melampodium*	*Melampodium leucanthum*	FJ846281
Eudicotyledons	Asteraceae	*Melampodium*	*Melampodium leucanthum*	FJ846282
Eudicotyledons	Asteraceae	*Melampodium*	*Melampodium leucanthum*	FJ846283
Eudicotyledons	Asteraceae	*Melampodium*	*Melampodium leucanthum*	FJ846284
Eudicotyledons	Asteraceae	*Melampodium*	*Melampodium leucanthum*	FJ846285
Eudicotyledons	Asteraceae	*Melampodium*	*Melampodium leucanthum*	FJ846286
Eudicotyledons	Asteraceae	*Melampodium*	*Melampodium leucanthum*	FJ846287
Eudicotyledons	Asteraceae	*Melampodium*	*Melampodium leucanthum*	FJ846288
Eudicotyledons	Plantaginaceae	*Plantago*	*Plantago ovata*	GQ248371
Eudicotyledons	Plantaginaceae	*Plantago*	*Plantago ovata*	EU036243
Eudicotyledons	Plantaginaceae	*Plantago*	*Plantago ovata*	EU036244
Eudicotyledons	Plantaginaceae	*Plantago*	*Plantago ovata*	EU036245
Eudicotyledons	Plantaginaceae	*Plantago*	*Plantago ovata*	EU036246
Eudicotyledons	Plantaginaceae	*Plantago*	*Plantago ovata*	EU036247
Eudicotyledons	Plantaginaceae	*Plantago*	*Plantago ovata*	EU036248
Eudicotyledons	Plantaginaceae	*Plantago*	*Plantago ovata*	EU036249
Eudicotyledons	Plantaginaceae	*Plantago*	*Plantago ovata*	EU036250
Eudicotyledons	Plantaginaceae	*Plantago*	*Plantago ovata*	EU036251
Eudicotyledons	Plantaginaceae	*Plantago*	*Plantago ovata*	EU036252
Eudicotyledons	Plantaginaceae	*Plantago*	*Plantago ovata*	EU036253
Eudicotyledons	Plantaginaceae	*Plantago*	*Plantago ovata*	EU036254
Eudicotyledons	Plantaginaceae	*Plantago*	*Plantago ovata*	EU036255
Eudicotyledons	Plantaginaceae	*Plantago*	*Plantago ovata*	EU036256
Eudicotyledons	Plantaginaceae	*Plantago*	*Plantago ovata*	EF590729
Eudicotyledons	Asteraceae	*Melampodium*	*Melampodium americanum*	GU216556
Eudicotyledons	Asteraceae	*Melampodium*	*Melampodium americanum*	GU216557
Eudicotyledons	Asteraceae	*Melampodium*	*Melampodium pilosum*	GU216558
Eudicotyledons	Asteraceae	*Melampodium*	*Melampodium pilosum*	GU216559
Eudicotyledons	Hydrangeaceae	*Hydrangea*	*Hydrangea aspera*	JN044922

Eudicotyledons	Hydrangeaceae	*Hydrangea*	*Hydrangea aspera*	JN044923
Eudicotyledons	Hydrangeaceae	*Hydrangea*	*Hydrangea aspera*	HM216967
Eudicotyledons	Rubiaceae	*Psychotria*	*Psychotria grandis*	GQ982341
Eudicotyledons	Rubiaceae	*Psychotria*	*Psychotria grandis*	HM446992
Eudicotyledons	Asteraceae	*Senecio*	*Senecio scandens*	GQ435114
Eudicotyledons	Asteraceae	*Senecio*	*Senecio scandens*	GQ435115
Eudicotyledons	Asteraceae	*Senecio*	*Senecio scandens*	GQ435116
Eudicotyledons	Asteraceae	*Senecio*	*Senecio scandens*	GQ435117
Eudicotyledons	Asteraceae	*Senecio*	*Senecio scandens*	GQ435118
Eudicotyledons	Asteraceae	*Senecio*	*Senecio scandens*	EF538048
Eudicotyledons	Lamiaceae	*Mentha*	*Mentha aquatica*	FR726099
Eudicotyledons	Lamiaceae	*Mentha*	*Mentha aquatica*	FR726100
Eudicotyledons	Lamiaceae	*Mentha*	*Mentha aquatica*	AY643656
Eudicotyledons	Lamiaceae	*Mentha*	*Mentha aquatica*	AY643657
Eudicotyledons	Lamiaceae	*Mentha*	*Mentha aquatica*	AY643658
Eudicotyledons	Lamiaceae	*Mentha*	*Mentha aquatica*	AY643659
Eudicotyledons	Lamiaceae	*Mentha*	*Mentha aquatica*	AY643660
Eudicotyledons	Lamiaceae	*Mentha*	*Mentha aquatica*	AY643661
Eudicotyledons	Lamiaceae	*Mentha*	*Mentha aquatica*	AY643662
Eudicotyledons	Asteraceae	*Encelia*	*Encelia farinosa*	DQ661036
Eudicotyledons	Asteraceae	*Encelia*	*Encelia farinosa*	DQ661037
Eudicotyledons	Asteraceae	*Encelia*	*Encelia farinosa*	DQ661038
Eudicotyledons	Asteraceae	*Encelia*	*Encelia farinosa*	DQ661039
Eudicotyledons	Asteraceae	*Encelia*	*Encelia farinosa*	DQ661040
Eudicotyledons	Asteraceae	*Encelia*	*Encelia farinosa*	DQ661041
Eudicotyledons	Asteraceae	*Encelia*	*Encelia farinosa*	DQ661042
Eudicotyledons	Asteraceae	*Encelia*	*Encelia ventorum*	DQ383906
Eudicotyledons	Asteraceae	*Encelia*	*Encelia ventorum*	DQ383907
Eudicotyledons	Rosaceae	*Aronia*	*Aronia prunifolia*	JQ390629
Eudicotyledons	Rosaceae	*Aronia*	*Aronia prunifolia*	GQ305338
Eudicotyledons	Moraceae	*Brosimum*	*Brosimum alicastrum*	GQ436998
Eudicotyledons	Moraceae	*Brosimum*	*Brosimum alicastrum*	GQ436999
Eudicotyledons	Moraceae	*Brosimum*	*Brosimum alicastrum*	GQ437000
Eudicotyledons	Moraceae	*Brosimum*	*Brosimum alicastrum*	GQ437001
Eudicotyledons	Moraceae	*Brosimum*	*Brosimum alicastrum*	GQ437002
Eudicotyledons	Moraceae	*Brosimum*	*Brosimum alicastrum*	GQ437003
Eudicotyledons	Moraceae	*Brosimum*	*Brosimum alicastrum*	GQ437004
Eudicotyledons	Moraceae	*Brosimum*	*Brosimum alicastrum*	GQ437005
Eudicotyledons	Moraceae	*Brosimum*	*Brosimum alicastrum*	GQ437006
Eudicotyledons	Moraceae	*Brosimum*	*Brosimum alicastrum*	GQ437007
Eudicotyledons	Moraceae	*Brosimum*	*Brosimum alicastrum*	GQ437008
Eudicotyledons	Moraceae	*Brosimum*	*Brosimum alicastrum*	GQ437009
Eudicotyledons	Moraceae	*Brosimum*	*Brosimum alicastrum*	GQ437010
Eudicotyledons	Moraceae	*Brosimum*	*Brosimum alicastrum*	GQ437011
Eudicotyledons	Moraceae	*Brosimum*	*Brosimum alicastrum*	GQ437012
Eudicotyledons	Moraceae	*Brosimum*	*Brosimum alicastrum*	GQ437013
Eudicotyledons	Moraceae	*Brosimum*	*Brosimum alicastrum*	GQ437014
Eudicotyledons	Moraceae	*Brosimum*	*Brosimum alicastrum*	GQ437015
Eudicotyledons	Moraceae	*Brosimum*	*Brosimum alicastrum*	GQ437016
Eudicotyledons	Moraceae	*Brosimum*	*Brosimum alicastrum*	GQ437017
Eudicotyledons	Moraceae	*Brosimum*	*Brosimum alicastrum*	GQ437018
Eudicotyledons	Moraceae	*Brosimum*	*Brosimum alicastrum*	GQ437019
Eudicotyledons	Moraceae	*Brosimum*	*Brosimum alicastrum*	GQ437020

Eudicotyledons	Moraceae	*Brosimum*	*Brosimum alicastrum*	GQ437021
Eudicotyledons	Moraceae	*Brosimum*	*Brosimum alicastrum*	GQ437022
Eudicotyledons	Moraceae	*Brosimum*	*Brosimum alicastrum*	GQ437023
Eudicotyledons	Moraceae	*Brosimum*	*Brosimum alicastrum*	GQ437024
Eudicotyledons	Moraceae	*Brosimum*	*Brosimum alicastrum*	GQ437025
Eudicotyledons	Moraceae	*Brosimum*	*Brosimum alicastrum*	GQ437026
Eudicotyledons	Moraceae	*Brosimum*	*Brosimum alicastrum*	GQ437027
Eudicotyledons	Moraceae	*Brosimum*	*Brosimum alicastrum*	GQ437028
Eudicotyledons	Moraceae	*Brosimum*	*Brosimum alicastrum*	GQ437029
Eudicotyledons	Moraceae	*Brosimum*	*Brosimum alicastrum*	GQ437030
Eudicotyledons	Moraceae	*Brosimum*	*Brosimum alicastrum*	GQ437031
Eudicotyledons	Moraceae	*Brosimum*	*Brosimum alicastrum*	GQ437032
Eudicotyledons	Moraceae	*Brosimum*	*Brosimum alicastrum*	GQ437033
Eudicotyledons	Moraceae	*Brosimum*	*Brosimum alicastrum*	GQ437034
Eudicotyledons	Moraceae	*Brosimum*	*Brosimum alicastrum*	GQ437035
Eudicotyledons	Moraceae	*Brosimum*	*Brosimum alicastrum*	GQ437036
Eudicotyledons	Moraceae	*Brosimum*	*Brosimum alicastrum*	GQ437037
Eudicotyledons	Moraceae	*Brosimum*	*Brosimum alicastrum*	GQ437038
Eudicotyledons	Moraceae	*Brosimum*	*Brosimum alicastrum*	GQ437039
Eudicotyledons	Moraceae	*Brosimum*	*Brosimum alicastrum*	GQ437040
Eudicotyledons	Moraceae	*Brosimum*	*Brosimum alicastrum*	GQ437041
Eudicotyledons	Moraceae	*Brosimum*	*Brosimum alicastrum*	GQ437042
Eudicotyledons	Moraceae	*Brosimum*	*Brosimum alicastrum*	GQ437043
Eudicotyledons	Moraceae	*Brosimum*	*Brosimum alicastrum*	GQ437044
Eudicotyledons	Moraceae	*Brosimum*	*Brosimum alicastrum*	GQ437045
Eudicotyledons	Moraceae	*Brosimum*	*Brosimum alicastrum*	GQ437046
Eudicotyledons	Moraceae	*Brosimum*	*Brosimum alicastrum*	GQ437047
Eudicotyledons	Moraceae	*Brosimum*	*Brosimum alicastrum*	GQ437048
Eudicotyledons	Moraceae	*Brosimum*	*Brosimum alicastrum*	GQ437049
Eudicotyledons	Moraceae	*Brosimum*	*Brosimum alicastrum*	GQ437050
Eudicotyledons	Moraceae	*Brosimum*	*Brosimum alicastrum*	GQ437051
Eudicotyledons	Moraceae	*Brosimum*	*Brosimum alicastrum*	GQ437052
Eudicotyledons	Moraceae	*Brosimum*	*Brosimum alicastrum*	GQ437053
Eudicotyledons	Moraceae	*Brosimum*	*Brosimum alicastrum*	GQ437054
Eudicotyledons	Moraceae	*Brosimum*	*Brosimum alicastrum*	GQ437055
Eudicotyledons	Moraceae	*Brosimum*	*Brosimum alicastrum*	GQ437056
Eudicotyledons	Moraceae	*Brosimum*	*Brosimum alicastrum*	GQ437057
Eudicotyledons	Moraceae	*Brosimum*	*Brosimum alicastrum*	GQ437058
Eudicotyledons	Moraceae	*Brosimum*	*Brosimum alicastrum*	GQ437059
Eudicotyledons	Moraceae	*Brosimum*	*Brosimum alicastrum*	GQ437060
Eudicotyledons	Moraceae	*Brosimum*	*Brosimum alicastrum*	GQ437061
Eudicotyledons	Moraceae	*Brosimum*	*Brosimum alicastrum*	GQ437062
Eudicotyledons	Moraceae	*Brosimum*	*Brosimum alicastrum*	GQ437063
Eudicotyledons	Moraceae	*Brosimum*	*Brosimum alicastrum*	GQ437064
Eudicotyledons	Moraceae	*Brosimum*	*Brosimum alicastrum*	GQ437065
Eudicotyledons	Moraceae	*Brosimum*	*Brosimum alicastrum*	GQ437066
Eudicotyledons	Moraceae	*Brosimum*	*Brosimum alicastrum*	GQ437067
Eudicotyledons	Moraceae	*Brosimum*	*Brosimum alicastrum*	GQ437068
Eudicotyledons	Moraceae	*Brosimum*	*Brosimum alicastrum*	GQ437069
Eudicotyledons	Moraceae	*Brosimum*	*Brosimum alicastrum*	GQ437070
Eudicotyledons	Moraceae	*Brosimum*	*Brosimum alicastrum*	GQ437071
Eudicotyledons	Moraceae	*Brosimum*	*Brosimum alicastrum*	GQ437072
Eudicotyledons	Moraceae	*Brosimum*	*Brosimum alicastrum*	GQ437073

Eudicotyledons	Moraceae	*Brosimum*	*Brosimum alicastrum*	GQ437074
Eudicotyledons	Moraceae	*Brosimum*	*Brosimum alicastrum*	GQ437075
Eudicotyledons	Moraceae	*Brosimum*	*Brosimum alicastrum*	GQ437076
Eudicotyledons	Moraceae	*Brosimum*	*Brosimum alicastrum*	GQ437077
Eudicotyledons	Moraceae	*Brosimum*	*Brosimum alicastrum*	GQ437078
Eudicotyledons	Moraceae	*Brosimum*	*Brosimum alicastrum*	GQ437079
Eudicotyledons	Moraceae	*Brosimum*	*Brosimum alicastrum*	GQ437080
Eudicotyledons	Moraceae	*Brosimum*	*Brosimum alicastrum*	GQ437081
Eudicotyledons	Moraceae	*Brosimum*	*Brosimum alicastrum*	GQ437082
Eudicotyledons	Moraceae	*Brosimum*	*Brosimum alicastrum*	GQ437083
Eudicotyledons	Moraceae	*Brosimum*	*Brosimum alicastrum*	GQ437084
Eudicotyledons	Moraceae	*Brosimum*	*Brosimum alicastrum*	GQ437085
Eudicotyledons	Moraceae	*Brosimum*	*Brosimum alicastrum*	GQ437086
Eudicotyledons	Moraceae	*Brosimum*	*Brosimum alicastrum*	GQ437087
Eudicotyledons	Moraceae	*Brosimum*	*Brosimum alicastrum*	GQ437088
Eudicotyledons	Moraceae	*Brosimum*	*Brosimum alicastrum*	GQ437089
Eudicotyledons	Moraceae	*Brosimum*	*Brosimum alicastrum*	GQ437090
Eudicotyledons	Moraceae	*Brosimum*	*Brosimum alicastrum*	GQ437091
Eudicotyledons	Moraceae	*Brosimum*	*Brosimum alicastrum*	GQ437092
Eudicotyledons	Moraceae	*Brosimum*	*Brosimum alicastrum*	GQ437093
Eudicotyledons	Moraceae	*Brosimum*	*Brosimum alicastrum*	GQ437094
Eudicotyledons	Moraceae	*Brosimum*	*Brosimum alicastrum*	GQ437095
Eudicotyledons	Moraceae	*Brosimum*	*Brosimum alicastrum*	GQ437096
Eudicotyledons	Moraceae	*Brosimum*	*Brosimum alicastrum*	GQ437097
Eudicotyledons	Moraceae	*Brosimum*	*Brosimum alicastrum*	GQ437098
Eudicotyledons	Moraceae	*Brosimum*	*Brosimum alicastrum*	GQ437099
Eudicotyledons	Moraceae	*Brosimum*	*Brosimum alicastrum*	GQ437100
Eudicotyledons	Moraceae	*Brosimum*	*Brosimum alicastrum*	GQ437101
Eudicotyledons	Moraceae	*Brosimum*	*Brosimum alicastrum*	GQ437102
Eudicotyledons	Moraceae	*Brosimum*	*Brosimum alicastrum*	GQ437103
Eudicotyledons	Moraceae	*Brosimum*	*Brosimum alicastrum*	GQ437104
Eudicotyledons	Moraceae	*Brosimum*	*Brosimum alicastrum*	GQ437105
Eudicotyledons	Moraceae	*Brosimum*	*Brosimum alicastrum*	GQ437106
Eudicotyledons	Moraceae	*Brosimum*	*Brosimum alicastrum*	GQ437107
Eudicotyledons	Moraceae	*Brosimum*	*Brosimum alicastrum*	GQ437108
Eudicotyledons	Moraceae	*Brosimum*	*Brosimum alicastrum*	GQ437109
Eudicotyledons	Moraceae	*Brosimum*	*Brosimum alicastrum*	GQ437110
Eudicotyledons	Moraceae	*Brosimum*	*Brosimum alicastrum*	GQ437111
Eudicotyledons	Moraceae	*Brosimum*	*Brosimum alicastrum*	GQ437112
Eudicotyledons	Moraceae	*Brosimum*	*Brosimum alicastrum*	GQ437113
Eudicotyledons	Moraceae	*Brosimum*	*Brosimum alicastrum*	GQ437114
Eudicotyledons	Moraceae	*Brosimum*	*Brosimum alicastrum*	GQ437115
Eudicotyledons	Moraceae	*Brosimum*	*Brosimum alicastrum*	GQ437116
Eudicotyledons	Moraceae	*Brosimum*	*Brosimum alicastrum*	GQ437117
Eudicotyledons	Moraceae	*Brosimum*	*Brosimum alicastrum*	GQ437118
Eudicotyledons	Moraceae	*Brosimum*	*Brosimum alicastrum*	GQ437119
Eudicotyledons	Moraceae	*Brosimum*	*Brosimum alicastrum*	GQ437120
Eudicotyledons	Moraceae	*Brosimum*	*Brosimum alicastrum*	GQ437121
Eudicotyledons	Moraceae	*Brosimum*	*Brosimum alicastrum*	GQ437122
Eudicotyledons	Moraceae	*Brosimum*	*Brosimum alicastrum*	GQ437123
Eudicotyledons	Moraceae	*Brosimum*	*Brosimum alicastrum*	GQ437124
Eudicotyledons	Moraceae	*Brosimum*	*Brosimum alicastrum*	GQ437125
Eudicotyledons	Moraceae	*Brosimum*	*Brosimum alicastrum*	GQ437126

Eudicotyledons	Moraceae	*Brosimum*	*Brosimum alicastrum*	GQ437127
Eudicotyledons	Moraceae	*Brosimum*	*Brosimum alicastrum*	GQ437128
Eudicotyledons	Moraceae	*Brosimum*	*Brosimum alicastrum*	GQ437129
Eudicotyledons	Moraceae	*Brosimum*	*Brosimum alicastrum*	GQ437130
Eudicotyledons	Moraceae	*Brosimum*	*Brosimum alicastrum*	GQ437131
Eudicotyledons	Moraceae	*Brosimum*	*Brosimum alicastrum*	GQ437132
Eudicotyledons	Moraceae	*Brosimum*	*Brosimum alicastrum*	GQ437133
Eudicotyledons	Moraceae	*Brosimum*	*Brosimum alicastrum*	GQ437134
Eudicotyledons	Moraceae	*Brosimum*	*Brosimum alicastrum*	GQ437135
Eudicotyledons	Moraceae	*Brosimum*	*Brosimum alicastrum*	GQ437136
Eudicotyledons	Moraceae	*Brosimum*	*Brosimum alicastrum*	GQ437137
Eudicotyledons	Moraceae	*Brosimum*	*Brosimum alicastrum*	GQ437138
Eudicotyledons	Moraceae	*Brosimum*	*Brosimum alicastrum*	GQ437139
Eudicotyledons	Moraceae	*Brosimum*	*Brosimum alicastrum*	GQ437140
Eudicotyledons	Moraceae	*Brosimum*	*Brosimum alicastrum*	GQ437141
Eudicotyledons	Moraceae	*Brosimum*	*Brosimum alicastrum*	GQ437142
Eudicotyledons	Moraceae	*Brosimum*	*Brosimum alicastrum*	GQ437143
Eudicotyledons	Moraceae	*Brosimum*	*Brosimum alicastrum*	GQ437144
Eudicotyledons	Moraceae	*Brosimum*	*Brosimum alicastrum*	GQ437145
Eudicotyledons	Moraceae	*Brosimum*	*Brosimum alicastrum*	GQ437146
Eudicotyledons	Moraceae	*Brosimum*	*Brosimum alicastrum*	GQ437147
Eudicotyledons	Moraceae	*Brosimum*	*Brosimum alicastrum*	GQ437148
Eudicotyledons	Moraceae	*Brosimum*	*Brosimum alicastrum*	GQ437149
Eudicotyledons	Moraceae	*Brosimum*	*Brosimum alicastrum*	GQ437150
Eudicotyledons	Moraceae	*Brosimum*	*Brosimum alicastrum*	GQ437151
Eudicotyledons	Moraceae	*Brosimum*	*Brosimum alicastrum*	GQ437152
Eudicotyledons	Moraceae	*Brosimum*	*Brosimum alicastrum*	GQ437153
Eudicotyledons	Moraceae	*Brosimum*	*Brosimum alicastrum*	GQ437154
Eudicotyledons	Moraceae	*Brosimum*	*Brosimum alicastrum*	GQ437155
Eudicotyledons	Moraceae	*Brosimum*	*Brosimum alicastrum*	GQ437156
Eudicotyledons	Moraceae	*Brosimum*	*Brosimum alicastrum*	GQ437157
Eudicotyledons	Moraceae	*Brosimum*	*Brosimum alicastrum*	GQ437158
Eudicotyledons	Moraceae	*Brosimum*	*Brosimum alicastrum*	GQ437159
Eudicotyledons	Moraceae	*Brosimum*	*Brosimum alicastrum*	GQ437160
Eudicotyledons	Moraceae	*Brosimum*	*Brosimum alicastrum*	GQ437161
Eudicotyledons	Moraceae	*Brosimum*	*Brosimum alicastrum*	GQ437162
Eudicotyledons	Moraceae	*Brosimum*	*Brosimum alicastrum*	GQ437163
Eudicotyledons	Moraceae	*Brosimum*	*Brosimum alicastrum*	GQ437164
Eudicotyledons	Moraceae	*Brosimum*	*Brosimum alicastrum*	GQ437165
Eudicotyledons	Moraceae	*Brosimum*	*Brosimum alicastrum*	GQ437166
Eudicotyledons	Moraceae	*Brosimum*	*Brosimum alicastrum*	GQ437167
Eudicotyledons	Moraceae	*Brosimum*	*Brosimum alicastrum*	GQ437168
Eudicotyledons	Moraceae	*Brosimum*	*Brosimum alicastrum*	GQ437169
Eudicotyledons	Moraceae	*Brosimum*	*Brosimum alicastrum*	GQ437170
Eudicotyledons	Moraceae	*Brosimum*	*Brosimum alicastrum*	GQ437171
Eudicotyledons	Moraceae	*Brosimum*	*Brosimum alicastrum*	GQ437172
Eudicotyledons	Moraceae	*Brosimum*	*Brosimum alicastrum*	GQ437173
Eudicotyledons	Moraceae	*Brosimum*	*Brosimum alicastrum*	GQ437174
Eudicotyledons	Moraceae	*Brosimum*	*Brosimum alicastrum*	GQ437175
Eudicotyledons	Moraceae	*Brosimum*	*Brosimum alicastrum*	GQ437176
Eudicotyledons	Moraceae	*Brosimum*	*Brosimum alicastrum*	GQ437177
Eudicotyledons	Moraceae	*Brosimum*	*Brosimum alicastrum*	GQ437178
Eudicotyledons	Moraceae	*Brosimum*	*Brosimum alicastrum*	GQ437179

Eudicotyledons	Moraceae	*Brosimum*	*Brosimum alicastrum*	GQ437180
Eudicotyledons	Moraceae	*Brosimum*	*Brosimum alicastrum*	GQ437181
Eudicotyledons	Moraceae	*Brosimum*	*Brosimum alicastrum*	GQ437182
Eudicotyledons	Moraceae	*Brosimum*	*Brosimum alicastrum*	GQ437183
Eudicotyledons	Moraceae	*Brosimum*	*Brosimum alicastrum*	GQ437184
Eudicotyledons	Moraceae	*Brosimum*	*Brosimum alicastrum*	GQ437185
Eudicotyledons	Moraceae	*Brosimum*	*Brosimum alicastrum*	GQ437186
Eudicotyledons	Moraceae	*Brosimum*	*Brosimum alicastrum*	GQ437187
Eudicotyledons	Moraceae	*Brosimum*	*Brosimum alicastrum*	GQ437188
Eudicotyledons	Moraceae	*Brosimum*	*Brosimum alicastrum*	GQ437189
Eudicotyledons	Moraceae	*Brosimum*	*Brosimum alicastrum*	GQ437190
Eudicotyledons	Moraceae	*Brosimum*	*Brosimum alicastrum*	GQ437191
Eudicotyledons	Moraceae	*Brosimum*	*Brosimum alicastrum*	GQ437192
Eudicotyledons	Moraceae	*Brosimum*	*Brosimum alicastrum*	GQ437193
Eudicotyledons	Moraceae	*Brosimum*	*Brosimum alicastrum*	GQ437194
Eudicotyledons	Moraceae	*Brosimum*	*Brosimum alicastrum*	GQ437195
Eudicotyledons	Moraceae	*Brosimum*	*Brosimum alicastrum*	GQ437196
Eudicotyledons	Moraceae	*Brosimum*	*Brosimum alicastrum*	GQ437197
Eudicotyledons	Moraceae	*Brosimum*	*Brosimum alicastrum*	GQ437198
Eudicotyledons	Moraceae	*Brosimum*	*Brosimum alicastrum*	GQ437199
Eudicotyledons	Moraceae	*Brosimum*	*Brosimum alicastrum*	GQ437200
Eudicotyledons	Moraceae	*Brosimum*	*Brosimum alicastrum*	GQ437201
Eudicotyledons	Moraceae	*Brosimum*	*Brosimum alicastrum*	GQ437202
Eudicotyledons	Moraceae	*Brosimum*	*Brosimum alicastrum*	GQ437203
Eudicotyledons	Moraceae	*Brosimum*	*Brosimum alicastrum*	GQ437204
Eudicotyledons	Moraceae	*Brosimum*	*Brosimum alicastrum*	GQ437205
Eudicotyledons	Moraceae	*Brosimum*	*Brosimum alicastrum*	GQ437206
Eudicotyledons	Moraceae	*Brosimum*	*Brosimum alicastrum*	GQ982162
Eudicotyledons	Tamaricaceae	*Myricaria*	*Myricaria elegans*	EF394293
Eudicotyledons	Tamaricaceae	*Myricaria*	*Myricaria elegans*	EF394294
Eudicotyledons	Tamaricaceae	*Myricaria*	*Myricaria squamosa*	EU240617
Eudicotyledons	Tamaricaceae	*Myricaria*	*Myricaria squamosa*	EF394264
Eudicotyledons	Tamaricaceae	*Myricaria*	*Myricaria squamosa*	EF394265
Eudicotyledons	Tamaricaceae	*Myricaria*	*Myricaria squamosa*	EF394266
Eudicotyledons	Tamaricaceae	*Myricaria*	*Myricaria squamosa*	EF394267
Eudicotyledons	Tamaricaceae	*Myricaria*	*Myricaria bracteata*	EU240615
Eudicotyledons	Tamaricaceae	*Myricaria*	*Myricaria bracteata*	EF394268
Eudicotyledons	Tamaricaceae	*Myricaria*	*Myricaria bracteata*	EF394269
Eudicotyledons	Tamaricaceae	*Myricaria*	*Myricaria bracteata*	EF394270
Eudicotyledons	Tamaricaceae	*Myricaria*	*Myricaria bracteata*	EF394271
Eudicotyledons	Tamaricaceae	*Myricaria*	*Myricaria bracteata*	EF394272
Eudicotyledons	Tamaricaceae	*Myricaria*	*Myricaria bracteata*	EF394273
Eudicotyledons	Apocynaceae	*Hoodia*	*Hoodia gordonii*	FJ026605
Eudicotyledons	Apocynaceae	*Hoodia*	*Hoodia gordonii*	FJ026606
Eudicotyledons	Apocynaceae	*Hoodia*	*Hoodia gordonii*	FJ026607
Eudicotyledons	Apocynaceae	*Hoodia*	*Hoodia gordonii*	JN117261
Eudicotyledons	Apocynaceae	*Hoodia*	*Hoodia gordonii*	JN117262
Eudicotyledons	Apocynaceae	*Hoodia*	*Hoodia gordonii*	JN117263
Eudicotyledons	Meliaceae	*Carapa*	*Carapa guianensis*	JN122312
Eudicotyledons	Meliaceae	*Carapa*	*Carapa guianensis*	JN122313
Eudicotyledons	Meliaceae	*Carapa*	*Carapa guianensis*	JN122314
Eudicotyledons	Meliaceae	*Carapa*	*Carapa guianensis*	JN122315
Eudicotyledons	Meliaceae	*Carapa*	*Carapa guianensis*	JN122316

Eudicotyledons	Meliaceae	*Carapa*	*Carapa guianensis*	JN122317
Eudicotyledons	Meliaceae	*Carapa*	*Carapa guianensis*	JN122318
Eudicotyledons	Meliaceae	*Carapa*	*Carapa guianensis*	JN122319
Eudicotyledons	Meliaceae	*Carapa*	*Carapa guianensis*	JN122320
Eudicotyledons	Crassulaceae	*Rhodiola*	*Rhodiola bupleuroides*	JN046719
Eudicotyledons	Crassulaceae	*Rhodiola*	*Rhodiola bupleuroides*	JN046720
Eudicotyledons	Crassulaceae	*Rhodiola*	*Rhodiola sacra*	JN046729
Eudicotyledons	Crassulaceae	*Rhodiola*	*Rhodiola sacra*	JN046730
Eudicotyledons	Crassulaceae	*Rhodiola*	*Rhodiola dumulosa*	FJ794333
Eudicotyledons	Crassulaceae	*Rhodiola*	*Rhodiola dumulosa*	FJ794334
Eudicotyledons	Crassulaceae	*Rhodiola*	*Rhodiola dumulosa*	FJ794335
Eudicotyledons	Crassulaceae	*Rhodiola*	*Rhodiola dumulosa*	FJ794336
Eudicotyledons	Crassulaceae	*Rhodiola*	*Rhodiola dumulosa*	FJ794337
Eudicotyledons	Crassulaceae	*Rhodiola*	*Rhodiola dumulosa*	FJ794338
Eudicotyledons	Crassulaceae	*Rhodiola*	*Rhodiola dumulosa*	FJ794339
Eudicotyledons	Crassulaceae	*Rhodiola*	*Rhodiola dumulosa*	FJ794340
Eudicotyledons	Crassulaceae	*Rhodiola*	*Rhodiola dumulosa*	FJ794341
Eudicotyledons	Crassulaceae	*Rhodiola*	*Rhodiola dumulosa*	FJ794342
Eudicotyledons	Crassulaceae	*Rhodiola*	*Rhodiola dumulosa*	FJ794343
Eudicotyledons	Crassulaceae	*Rhodiola*	*Rhodiola dumulosa*	FJ794344
Eudicotyledons	Crassulaceae	*Rhodiola*	*Rhodiola fastigiata*	JN046723
Eudicotyledons	Crassulaceae	*Rhodiola*	*Rhodiola fastigiata*	JN046724
Eudicotyledons	Crassulaceae	*Rhodiola*	*Rhodiola fastigiata*	JN046725
Eudicotyledons	Crassulaceae	*Rhodiola*	*Rhodiola kirilowii*	FJ974030
Eudicotyledons	Crassulaceae	*Rhodiola*	*Rhodiola kirilowii*	JN046726
Eudicotyledons	Crassulaceae	*Rhodiola*	*Rhodiola kirilowii*	JN046727
Eudicotyledons	Crassulaceae	*Rhodiola*	*Rhodiola kirilowii*	JN046728
Eudicotyledons	Lamiaceae	*Isodon*	*Isodon lophanthoides*	JN045037
Eudicotyledons	Lamiaceae	*Isodon*	*Isodon lophanthoides*	JN045038
Eudicotyledons	Lamiaceae	*Clinopodium*	*Clinopodium vulgare*	DQ667409
Eudicotyledons	Lamiaceae	*Clinopodium*	*Clinopodium vulgare*	HQ596649
Eudicotyledons	Rosaceae	*Potentilla*	*Potentilla chinensis*	GQ435272
Eudicotyledons	Rosaceae	*Potentilla*	*Potentilla chinensis*	GQ435273
Eudicotyledons	Gesneriaceae	*Glossoloma*	*Glossoloma grandicalyx*	DQ006189
Eudicotyledons	Gesneriaceae	*Glossoloma*	*Glossoloma grandicalyx*	DQ211205
Eudicotyledons	Papaveraceae	*Papaver*	*Papaver bracteatum*	JN584661
Eudicotyledons	Papaveraceae	*Papaver*	*Papaver bracteatum*	EF590720
Eudicotyledons	Malvaceae	*Tarasa*	*Tarasa albertii*	AY184309
Eudicotyledons	Malvaceae	*Tarasa*	*Tarasa albertii*	AY232638
Eudicotyledons	Malvaceae	*Tarasa*	*Tarasa antofagastana*	AY184310
Eudicotyledons	Malvaceae	*Tarasa*	*Tarasa antofagastana*	AY184311
Eudicotyledons	Malvaceae	*Tarasa*	*Tarasa antofagastana*	AY184312
Eudicotyledons	Malvaceae	*Tarasa*	*Tarasa humilis*	AY184320
Eudicotyledons	Malvaceae	*Tarasa*	*Tarasa humilis*	AY232640
Eudicotyledons	Malvaceae	*Tarasa*	*Tarasa operculata*	AY184326
Eudicotyledons	Malvaceae	*Tarasa*	*Tarasa operculata*	AY232642
Eudicotyledons	Malvaceae	*Tarasa*	*Tarasa tenella*	AY184330
Eudicotyledons	Malvaceae	*Tarasa*	*Tarasa tenella*	AY232643
Eudicotyledons	Malvaceae	*Tarasa*	*Tarasa thyrsoidea*	AY184332
Eudicotyledons	Malvaceae	*Tarasa*	*Tarasa thyrsoidea*	AY232644
Eudicotyledons	Tamaricaceae	*Myricaria*	*Myricaria laxiflora*	EU240620
Eudicotyledons	Tamaricaceae	*Myricaria*	*Myricaria laxiflora*	EF394254
Eudicotyledons	Tamaricaceae	*Myricaria*	*Myricaria laxiflora*	EF394255

Eudicotyledons	Tamaricaceae	*Myricaria*	*Myricaria laxiflora*	EF394256
Eudicotyledons	Tamaricaceae	*Myricaria*	*Myricaria laxiflora*	EF394257
Eudicotyledons	Lamiaceae	*Clinopodium*	*Clinopodium alpinum*	HQ902815
Eudicotyledons	Lamiaceae	*Clinopodium*	*Clinopodium alpinum*	HQ902850
Eudicotyledons	Lamiaceae	*Salvia*	*Salvia miltiorrhiza*	JQ339249
Eudicotyledons	Lamiaceae	*Salvia*	*Salvia miltiorrhiza*	DQ667379
Eudicotyledons	Lamiaceae	*Salvia*	*Salvia miltiorrhiza*	DQ667419
Eudicotyledons	Lamiaceae	*Salvia*	*Salvia miltiorrhiza*	EU590856
Eudicotyledons	Lamiaceae	*Salvia*	*Salvia miltiorrhiza*	FJ513086
Eudicotyledons	Lamiaceae	*Salvia*	*Salvia miltiorrhiza*	FJ513087
Eudicotyledons	Loranthaceae	*Macrosolen*	*Macrosolen cochinchinensis*	HQ317809
Eudicotyledons	Loranthaceae	*Macrosolen*	*Macrosolen cochinchinensis*	HQ317810
Eudicotyledons	Loranthaceae	*Macrosolen*	*Macrosolen cochinchinensis*	HQ317811
Eudicotyledons	Loranthaceae	*Macrosolen*	*Macrosolen cochinchinensis*	JN687575
Eudicotyledons	Loranthaceae	*Scurrula*	*Scurrula chingii*	HQ317814
Eudicotyledons	Loranthaceae	*Scurrula*	*Scurrula chingii*	HQ317815
Eudicotyledons	Loranthaceae	*Scurrula*	*Scurrula parasitica*	HQ317816
Eudicotyledons	Loranthaceae	*Scurrula*	*Scurrula parasitica*	HQ317817
Eudicotyledons	Loranthaceae	*Scurrula*	*Scurrula parasitica*	HQ317818
Eudicotyledons	Loranthaceae	*Taxillus*	*Taxillus chinensis*	HQ317821
Eudicotyledons	Loranthaceae	*Taxillus*	*Taxillus chinensis*	HQ317822
Eudicotyledons	Loranthaceae	*Taxillus*	*Taxillus chinensis*	HQ317823
Eudicotyledons	Loranthaceae	*Taxillus*	*Taxillus chinensis*	HQ317824
Eudicotyledons	Loranthaceae	*Taxillus*	*Taxillus chinensis*	HQ317825
Eudicotyledons	Loranthaceae	*Taxillus*	*Taxillus chinensis*	HQ317826
Eudicotyledons	Loranthaceae	*Taxillus*	*Taxillus chinensis*	HQ317827
Eudicotyledons	Loranthaceae	*Taxillus*	*Taxillus chinensis*	HQ317828
Eudicotyledons	Loranthaceae	*Taxillus*	*Taxillus chinensis*	HQ317829
Eudicotyledons	Loranthaceae	*Taxillus*	*Taxillus chinensis*	HQ317830
Eudicotyledons	Loranthaceae	*Taxillus*	*Taxillus chinensis*	HQ317831
Eudicotyledons	Loranthaceae	*Taxillus*	*Taxillus chinensis*	GQ435467
Eudicotyledons	Loranthaceae	*Taxillus*	*Taxillus sutchuenensis*	HQ317837
Eudicotyledons	Loranthaceae	*Taxillus*	*Taxillus sutchuenensis*	HQ317838
Eudicotyledons	Sapotaceae	*Micropholis*	*Micropholis egensis*	DQ344115
Eudicotyledons	Sapotaceae	*Micropholis*	*Micropholis egensis*	GQ428709
Eudicotyledons	Sapotaceae	*Micropholis*	*Micropholis guyanensis*	DQ344116
Eudicotyledons	Sapotaceae	*Micropholis*	*Micropholis guyanensis*	FJ039064
Eudicotyledons	Sapotaceae	*Micropholis*	*Micropholis guyanensis*	FJ039065
Eudicotyledons	Sapotaceae	*Micropholis*	*Micropholis guyanensis*	HM446966
Eudicotyledons	Sapotaceae	*Micropholis*	*Micropholis venulosa*	DQ344117
Eudicotyledons	Sapotaceae	*Micropholis*	*Micropholis venulosa*	FJ039067
Eudicotyledons	Sapotaceae	*Micropholis*	*Micropholis venulosa*	FJ039068
Eudicotyledons	Moraceae	*Brosimum*	*Brosimum guianense*	FJ039011
Eudicotyledons	Moraceae	*Brosimum*	*Brosimum guianense*	GQ982163
Eudicotyledons	Caprifoliaceae	*Valeriana*	*Valeriana urticifolia*	AY794303
Eudicotyledons	Caprifoliaceae	*Valeriana*	*Valeriana urticifolia*	AY794304
Eudicotyledons	Crassulaceae	*Rhodiola*	*Rhodiola crenulata*	GQ435061
Eudicotyledons	Crassulaceae	*Rhodiola*	*Rhodiola crenulata*	JN046721
Eudicotyledons	Crassulaceae	*Rhodiola*	*Rhodiola crenulata*	JN046722
Eudicotyledons	Betulaceae	*Alnus*	*Alnus cremastogyne*	FJ844498
Eudicotyledons	Betulaceae	*Alnus*	*Alnus cremastogyne*	FJ844499
Eudicotyledons	Betulaceae	*Alnus*	*Alnus cremastogyne*	FJ844500
Eudicotyledons	Betulaceae	*Alnus*	*Alnus cremastogyne*	FJ844501

Eudicotyledons	Betulaceae	*Alnus*	*Alnus cremastogyne*	FJ844502
Eudicotyledons	Betulaceae	*Alnus*	*Alnus cremastogyne*	FJ844503
Eudicotyledons	Betulaceae	*Alnus*	*Alnus cremastogyne*	JN043787
Eudicotyledons	Betulaceae	*Alnus*	*Alnus cremastogyne*	JN043788
Eudicotyledons	Betulaceae	*Alnus*	*Alnus cremastogyne*	JN043789
Eudicotyledons	Betulaceae	*Alnus*	*Alnus cremastogyne*	JN043790
Eudicotyledons	Betulaceae	*Alnus*	*Alnus cremastogyne*	JN043791
Eudicotyledons	Betulaceae	*Alnus*	*Alnus ferdinandi-coburgii*	FJ844504
Eudicotyledons	Betulaceae	*Alnus*	*Alnus ferdinandi-coburgii*	FJ844505
Eudicotyledons	Betulaceae	*Alnus*	*Alnus ferdinandi-coburgii*	FJ844506
Eudicotyledons	Betulaceae	*Alnus*	*Alnus ferdinandi-coburgii*	FJ844507
Eudicotyledons	Betulaceae	*Alnus*	*Alnus mandshurica*	JN043805
Eudicotyledons	Betulaceae	*Alnus*	*Alnus mandshurica*	JN043806
Eudicotyledons	Betulaceae	*Alnus*	*Alnus orientalis*	FJ844551
Eudicotyledons	Betulaceae	*Alnus*	*Alnus orientalis*	FJ844552
Eudicotyledons	Betulaceae	*Alnus*	*Alnus serrulata*	FJ844566
Eudicotyledons	Betulaceae	*Alnus*	*Alnus serrulata*	FJ844567
Eudicotyledons	Betulaceae	*Alnus*	*Alnus trabeculosa*	FJ844547
Eudicotyledons	Betulaceae	*Alnus*	*Alnus trabeculosa*	FJ844548
Eudicotyledons	Betulaceae	*Alnus*	*Alnus trabeculosa*	FJ844549
Eudicotyledons	Betulaceae	*Alnus*	*Alnus trabeculosa*	FJ844550
Eudicotyledons	Betulaceae	*Alnus*	*Alnus trabeculosa*	JN043814
Eudicotyledons	Betulaceae	*Betula*	*Betula davurica*	FJ011874
Eudicotyledons	Betulaceae	*Betula*	*Betula davurica*	FJ011875
Eudicotyledons	Betulaceae	*Betula*	*Betula davurica*	FJ011876
Eudicotyledons	Betulaceae	*Betula*	*Betula davurica*	FJ011877
Eudicotyledons	Rubiaceae	*Galium*	*Galium mollugo*	FJ395515
Eudicotyledons	Rubiaceae	*Galium*	*Galium mollugo*	HQ596710
Eudicotyledons	Malvaceae	*Cristaria*	*Cristaria aspera*	AY371674
Eudicotyledons	Malvaceae	*Cristaria*	*Cristaria aspera*	AY371685
Eudicotyledons	Malvaceae	*Cristaria*	*Cristaria multifida*	AY371681
Eudicotyledons	Malvaceae	*Cristaria*	*Cristaria multifida*	AY371684
Eudicotyledons	Berberidaceae	*Berberis*	*Berberis insignis*	GU934951
Eudicotyledons	Berberidaceae	*Berberis*	*Berberis insignis*	GU934952
Eudicotyledons	Myrtaceae	*Syzygium*	*Syzygium cumini*	GU135329
Eudicotyledons	Myrtaceae	*Syzygium*	*Syzygium cumini*	GU135395
Eudicotyledons	Lamiaceae	*Clinopodium*	*Clinopodium ashei*	DQ667348
Eudicotyledons	Lamiaceae	*Clinopodium*	*Clinopodium ashei*	AY943551
Eudicotyledons	Lamiaceae	*Clinopodium*	*Clinopodium ashei*	AY943552
Eudicotyledons	Lamiaceae	*Clinopodium*	*Clinopodium ashei*	AY943553
Eudicotyledons	Lamiaceae	*Clinopodium*	*Clinopodium ashei*	AY943554
Eudicotyledons	Ranunculaceae	*Coptis*	*Coptis quinquefolia*	AB159523
Eudicotyledons	Ranunculaceae	*Coptis*	*Coptis quinquefolia*	AB159526
Eudicotyledons	Ranunculaceae	*Coptis*	*Coptis teeta*	JN862875
Eudicotyledons	Ranunculaceae	*Coptis*	*Coptis teeta*	JN862876
Eudicotyledons	Ranunculaceae	*Coptis*	*Coptis teeta*	JN862877
Eudicotyledons	Ranunculaceae	*Coptis*	*Coptis teeta*	AB163743
Eudicotyledons	Ranunculaceae	*Coptis*	*Coptis teeta*	HQ829539
Eudicotyledons	Ranunculaceae	*Coptis*	*Coptis teeta*	HQ829540
Eudicotyledons	Ranunculaceae	*Coptis*	*Coptis teeta*	HQ829541
Eudicotyledons	Ranunculaceae	*Coptis*	*Coptis teeta*	HQ829542
Eudicotyledons	Ranunculaceae	*Coptis*	*Coptis deltoidea*	GQ435210
Eudicotyledons	Ranunculaceae	*Coptis*	*Coptis deltoidea*	GQ435211

Eudicotyledons	Ranunculaceae	*Coptis*	*Coptis deltoidea*	GQ435212
Eudicotyledons	Ranunculaceae	*Coptis*	*Coptis deltoidea*	JN862873
Eudicotyledons	Ranunculaceae	*Coptis*	*Coptis deltoidea*	AB163744
Eudicotyledons	Ranunculaceae	*Coptis*	*Coptis deltoidea*	HQ829531
Eudicotyledons	Ranunculaceae	*Coptis*	*Coptis deltoidea*	HQ829532
Eudicotyledons	Ranunculaceae	*Coptis*	*Coptis deltoidea*	HQ829533
Eudicotyledons	Ranunculaceae	*Coptis*	*Coptis deltoidea*	HQ829534
Eudicotyledons	Ranunculaceae	*Coptis*	*Coptis deltoidea*	HQ829535
Eudicotyledons	Ranunculaceae	*Coptis*	*Coptis chinensis*	GQ435206
Eudicotyledons	Ranunculaceae	*Coptis*	*Coptis chinensis*	GQ435207
Eudicotyledons	Ranunculaceae	*Coptis*	*Coptis chinensis*	JN862866
Eudicotyledons	Ranunculaceae	*Coptis*	*Coptis chinensis*	JN862867
Eudicotyledons	Ranunculaceae	*Coptis*	*Coptis chinensis*	JN862868
Eudicotyledons	Ranunculaceae	*Coptis*	*Coptis chinensis*	JN862869
Eudicotyledons	Ranunculaceae	*Coptis*	*Coptis chinensis*	JN862870
Eudicotyledons	Ranunculaceae	*Coptis*	*Coptis chinensis*	JN862872
Eudicotyledons	Ranunculaceae	*Coptis*	*Coptis chinensis*	AB163745
Eudicotyledons	Ranunculaceae	*Coptis*	*Coptis chinensis*	HQ829527
Eudicotyledons	Ranunculaceae	*Coptis*	*Coptis chinensis*	HQ829528
Eudicotyledons	Ranunculaceae	*Coptis*	*Coptis chinensis*	HQ829529
Eudicotyledons	Ranunculaceae	*Coptis*	*Coptis omeiensis*	JN862874
Eudicotyledons	Ranunculaceae	*Coptis*	*Coptis omeiensis*	AB163746
Eudicotyledons	Ranunculaceae	*Coptis*	*Coptis omeiensis*	HQ829536
Eudicotyledons	Ranunculaceae	*Coptis*	*Coptis omeiensis*	HQ829537
Eudicotyledons	Ranunculaceae	*Coptis*	*Coptis omeiensis*	HQ829538
Eudicotyledons	Melastomataceae	*Miconia*	*Miconia impetiolaris*	GQ982289
Eudicotyledons	Melastomataceae	*Miconia*	*Miconia impetiolaris*	HM446959
Eudicotyledons	Lamiaceae	*Salvia*	*Salvia digitaloides*	DQ667363
Eudicotyledons	Lamiaceae	*Salvia*	*Salvia digitaloides*	FJ513124
Eudicotyledons	Lamiaceae	*Salvia*	*Salvia fruticosa*	EU627579
Eudicotyledons	Lamiaceae	*Salvia*	*Salvia fruticosa*	FJ513109
Eudicotyledons	Lamiaceae	*Salvia*	*Salvia fruticosa*	HQ902840
Eudicotyledons	Lamiaceae	*Salvia*	*Salvia fruticosa*	HQ902859
Eudicotyledons	Lamiaceae	*Salvia*	*Salvia greatae*	DQ667339
Eudicotyledons	Lamiaceae	*Salvia*	*Salvia greatae*	HQ418921
Eudicotyledons	Tamaricaceae	*Myricaria*	*Myricaria germanica*	EU914131
Eudicotyledons	Tamaricaceae	*Myricaria*	*Myricaria germanica*	EU914132
Eudicotyledons	Tamaricaceae	*Myricaria*	*Myricaria germanica*	HQ680684
Eudicotyledons	Tamaricaceae	*Myricaria*	*Myricaria germanica*	HQ680687
Eudicotyledons	Proteaceae	*Faurea*	*Faurea rochetiana*	EU213813
Eudicotyledons	Proteaceae	*Faurea*	*Faurea rochetiana*	EU213814
Eudicotyledons	Proteaceae	*Faurea*	*Faurea rochetiana*	EU213815
Eudicotyledons	Proteaceae	*Faurea*	*Faurea saligna*	EU213816
Eudicotyledons	Proteaceae	*Faurea*	*Faurea saligna*	EU213817
Eudicotyledons	Proteaceae	*Faurea*	*Faurea saligna*	EU213818
Eudicotyledons	Malvaceae	*Palaua*	*Palaua rhombifolia*	DQ826559
Eudicotyledons	Malvaceae	*Palaua*	*Palaua rhombifolia*	DQ156321
Eudicotyledons	Malvaceae	*Palaua*	*Palaua rhombifolia*	DQ156326
Eudicotyledons	Lamiaceae	*Mentha*	*Mentha arvensis*	DQ667410
Eudicotyledons	Lamiaceae	*Mentha*	*Mentha arvensis*	HQ596770
Eudicotyledons	Lamiaceae	*Mentha*	*Mentha arvensis*	AY643665
Eudicotyledons	Gentianaceae	*Gentiana*	*Gentiana scabra*	GQ864027
Eudicotyledons	Gentianaceae	*Gentiana*	*Gentiana scabra*	GQ864028

Eudicotyledons	Lamiaceae	*Mentha*	*Mentha canadensis*	GQ434942
Eudicotyledons	Lamiaceae	*Mentha*	*Mentha canadensis*	JN406990
Eudicotyledons	Lamiaceae	*Mentha*	*Mentha canadensis*	JN406991
Eudicotyledons	Lamiaceae	*Mentha*	*Mentha canadensis*	JN406992
Eudicotyledons	Lamiaceae	*Mentha*	*Mentha canadensis*	JN406993
Eudicotyledons	Lamiaceae	*Mentha*	*Mentha canadensis*	AY643663
Eudicotyledons	Lamiaceae	*Mentha*	*Mentha canadensis*	AY643664
Eudicotyledons	Lamiaceae	*Mentha*	*Mentha canadensis*	HM590119
Eudicotyledons	Lamiaceae	*Mentha*	*Mentha pulegium*	EU627577
Eudicotyledons	Lamiaceae	*Mentha*	*Mentha pulegium*	HQ902828
Eudicotyledons	Lamiaceae	*Mentha*	*Mentha pulegium*	HQ902869
Eudicotyledons	Rosaceae	*Crataegus*	*Crataegus laevigata*	EF127130
Eudicotyledons	Rosaceae	*Crataegus*	*Crataegus laevigata*	EU500297
Eudicotyledons	Anacardiaceae	*Searsia*	*Searsia leptodictya*	EU213845
Eudicotyledons	Anacardiaceae	*Searsia*	*Searsia leptodictya*	EU213846
Eudicotyledons	Anacardiaceae	*Searsia*	*Searsia leptodictya*	EU213847
Eudicotyledons	Caprifoliaceae	*Valeriana*	*Valeriana clematitis*	AY794250
Eudicotyledons	Caprifoliaceae	*Valeriana*	*Valeriana clematitis*	AY794251
Eudicotyledons	Moraceae	*Ficus*	*Ficus altissima*	GU135300
Eudicotyledons	Moraceae	*Ficus*	*Ficus altissima*	JN044509
Eudicotyledons	Moraceae	*Ficus*	*Ficus altissima*	JN044510
Eudicotyledons	Moraceae	*Ficus*	*Ficus benghalensis*	GU935087
Eudicotyledons	Moraceae	*Ficus*	*Ficus benghalensis*	GU935088
Eudicotyledons	Moraceae	*Ficus*	*Ficus benghalensis*	GU935089
Eudicotyledons	Moraceae	*Ficus*	*Ficus benghalensis*	GU935090
Eudicotyledons	Moraceae	*Ficus*	*Ficus benghalensis*	GU935091
Eudicotyledons	Moraceae	*Ficus*	*Ficus citrifolia*	GQ982219
Eudicotyledons	Moraceae	*Ficus*	*Ficus citrifolia*	HM446931
Eudicotyledons	Moraceae	*Ficus*	*Ficus abutilifolia*	EU213819
Eudicotyledons	Moraceae	*Ficus*	*Ficus abutilifolia*	EU213820
Eudicotyledons	Moraceae	*Ficus*	*Ficus abutilifolia*	EU213821
Eudicotyledons	Moraceae	*Ficus*	*Ficus lyrata*	JN044532
Eudicotyledons	Moraceae	*Ficus*	*Ficus lyrata*	JN044533
Eudicotyledons	Moraceae	*Ficus*	*Ficus ischnopoda*	JN044529
Eudicotyledons	Moraceae	*Ficus*	*Ficus ischnopoda*	JN044530
Eudicotyledons	Moraceae	*Ficus*	*Ficus ischnopoda*	JN044531
Eudicotyledons	Moraceae	*Ficus*	*Ficus hirta*	HQ415506
Eudicotyledons	Moraceae	*Ficus*	*Ficus hirta*	JN044524
Eudicotyledons	Moraceae	*Ficus*	*Ficus hirta*	JN044525
Eudicotyledons	Lamiaceae	*Phlomis*	*Phlomis lychnitis*	AY792624
Eudicotyledons	Lamiaceae	*Phlomis*	*Phlomis lychnitis*	AY792625
Eudicotyledons	Lamiaceae	*Phlomis*	*Phlomis lychnitis*	AY792626
Eudicotyledons	Lamiaceae	*Phlomis*	*Phlomis lychnitis*	AY792627
Eudicotyledons	Lamiaceae	*Phlomis*	*Phlomis lychnitis*	AY792628
Eudicotyledons	Lamiaceae	*Phlomis*	*Phlomis lychnitis*	AY792629
Eudicotyledons	Lamiaceae	*Phlomis*	*Phlomis lychnitis*	AY792630
Eudicotyledons	Lamiaceae	*Phlomis*	*Phlomis lychnitis*	AY792631
Eudicotyledons	Lamiaceae	*Phlomis*	*Phlomis lychnitis*	AY792632
Eudicotyledons	Lamiaceae	*Phlomis*	*Phlomis lychnitis*	AY792633
Eudicotyledons	Lamiaceae	*Phlomis*	*Phlomis lychnitis*	AY792634
Eudicotyledons	Lamiaceae	*Phlomis*	*Phlomis lychnitis*	AY792635
Eudicotyledons	Lamiaceae	*Phlomis*	*Phlomis lychnitis*	AY792636
Eudicotyledons	Lamiaceae	*Phlomis*	*Phlomis lychnitis*	AY792637

Eudicotyledons	Lamiaceae	*Phlomis*	*Phlomis lychnitis*	AY792638
Eudicotyledons	Lamiaceae	*Phlomis*	*Phlomis lychnitis*	AY792639
Eudicotyledons	Lamiaceae	*Phlomis*	*Phlomis lychnitis*	AY792640
Eudicotyledons	Lamiaceae	*Phlomis*	*Phlomis lychnitis*	AY792641
Eudicotyledons	Lamiaceae	*Phlomis*	*Phlomis lychnitis*	AY792642
Eudicotyledons	Lamiaceae	*Phlomis*	*Phlomis lychnitis*	AY792643
Eudicotyledons	Lamiaceae	*Phlomis*	*Phlomis lychnitis*	AY792644
Eudicotyledons	Lamiaceae	*Phlomis*	*Phlomis lychnitis*	AY792645
Eudicotyledons	Lamiaceae	*Phlomis*	*Phlomis lychnitis*	AY792646
Eudicotyledons	Asteraceae	*Elephantopus*	*Elephantopus mollis*	JN406939
Eudicotyledons	Asteraceae	*Elephantopus*	*Elephantopus mollis*	JN406940
Eudicotyledons	Asteraceae	*Elephantopus*	*Elephantopus mollis*	JN406941
Eudicotyledons	Asteraceae	*Elephantopus*	*Elephantopus mollis*	JN406942
Eudicotyledons	Lamiaceae	*Conradina*	*Conradina brevifolia*	AY943531
Eudicotyledons	Lamiaceae	*Conradina*	*Conradina brevifolia*	AY943532
Eudicotyledons	Lamiaceae	*Conradina*	*Conradina brevifolia*	AY943533
Eudicotyledons	Lamiaceae	*Conradina*	*Conradina brevifolia*	AY943534
Eudicotyledons	Lamiaceae	*Conradina*	*Conradina brevifolia*	AY943535
Eudicotyledons	Lamiaceae	*Conradina*	*Conradina canescens*	DQ667349
Eudicotyledons	Lamiaceae	*Conradina*	*Conradina canescens*	AY943536
Eudicotyledons	Lamiaceae	*Conradina*	*Conradina canescens*	AY943537
Eudicotyledons	Lamiaceae	*Conradina*	*Conradina canescens*	AY943538
Eudicotyledons	Lamiaceae	*Conradina*	*Conradina glabra*	AY943541
Eudicotyledons	Lamiaceae	*Conradina*	*Conradina glabra*	AY943542
Eudicotyledons	Lamiaceae	*Conradina*	*Conradina glabra*	AY943543
Eudicotyledons	Lamiaceae	*Conradina*	*Conradina grandiflora*	AY943544
Eudicotyledons	Lamiaceae	*Conradina*	*Conradina grandiflora*	AY943545
Eudicotyledons	Lamiaceae	*Conradina*	*Conradina grandiflora*	AY943546
Eudicotyledons	Lamiaceae	*Conradina*	*Conradina grandiflora*	AY943547
Eudicotyledons	Lamiaceae	*Conradina*	*Conradina sp. Edwards et al. 133*	AY943548
Eudicotyledons	Lamiaceae	*Conradina*	*Conradina sp. Edwards et al. 133*	AY943549
Eudicotyledons	Apocynaceae	*Asclepias*	*Asclepias incarnata*	HQ596607
Eudicotyledons	Apocynaceae	*Asclepias*	*Asclepias incarnata*	GQ248250
Eudicotyledons	Apocynaceae	*Asclepias*	*Asclepias incarnata*	EF590673
Eudicotyledons	Apocynaceae	*Asclepias*	*Asclepias incarnata*	DQ006139
Eudicotyledons	Myrtaceae	*Syzygium*	*Syzygium buxifolium*	HQ415488
Eudicotyledons	Myrtaceae	*Syzygium*	*Syzygium buxifolium*	HQ427087
Eudicotyledons	Myrtaceae	*Syzygium*	*Syzygium buxifolium*	GQ435378
Eudicotyledons	Myrtaceae	*Syzygium*	*Syzygium jambos*	HQ415491
Eudicotyledons	Myrtaceae	*Syzygium*	*Syzygium jambos*	AM489882
Eudicotyledons	Myrtaceae	*Syzygium*	*Syzygium jambos*	GU135387
Eudicotyledons	Myrtaceae	*Syzygium*	*Syzygium jambos*	HM447006
Eudicotyledons	Solanaceae	*Mandragora*	*Mandragora caulescens*	HQ216170
Eudicotyledons	Solanaceae	*Mandragora*	*Mandragora caulescens*	HQ216171
Eudicotyledons	Lamiaceae	*Salvia*	*Salvia przewalskii*	DQ667362
Eudicotyledons	Lamiaceae	*Salvia*	*Salvia przewalskii*	GQ435471
Eudicotyledons	Lamiaceae	*Salvia*	*Salvia przewalskii*	FJ513125
Eudicotyledons	Malvaceae	*Palaua*	*Palaua weberbaueri*	DQ826560
Eudicotyledons	Malvaceae	*Palaua*	*Palaua weberbaueri*	DQ156331
Eudicotyledons	Malvaceae	*Palaua*	*Palaua mollendoensis*	DQ826558
Eudicotyledons	Malvaceae	*Palaua*	*Palaua mollendoensis*	DQ156334
Eudicotyledons	Asteraceae	*Encelia*	*Encelia resinifera*	DQ383904
Eudicotyledons	Asteraceae	*Encelia*	*Encelia resinifera*	DQ383905

Eudicotyledons	Asteraceae	*Encelia*	*Encelia asperifolia*	DQ383888
Eudicotyledons	Asteraceae	*Encelia*	*Encelia asperifolia*	DQ383889
Eudicotyledons	Asteraceae	*Encelia*	*Encelia laciniata*	DQ383898
Eudicotyledons	Asteraceae	*Encelia*	*Encelia laciniata*	DQ383899
Eudicotyledons	Asteraceae	*Encelia*	*Encelia palmeri*	DQ383900
Eudicotyledons	Asteraceae	*Encelia*	*Encelia palmeri*	DQ383901
Eudicotyledons	Asteraceae	*Encelia*	*Encelia palmeri*	DQ383902
Eudicotyledons	Asteraceae	*Enceliopsis*	*Enceliopsis nudicaulis*	EF133507
Eudicotyledons	Asteraceae	*Enceliopsis*	*Enceliopsis nudicaulis*	DQ383914
Eudicotyledons	Asteraceae	*Enceliopsis*	*Enceliopsis nutans*	DQ383915
Eudicotyledons	Asteraceae	*Enceliopsis*	*Enceliopsis nutans*	DQ383916
Eudicotyledons	Moraceae	*Ficus*	*Ficus trigona*	GU935114
Eudicotyledons	Moraceae	*Ficus*	*Ficus trigona*	GU935115
Eudicotyledons	Loganiaceae	*Strychnos*	*Strychnos decussata*	EU213857
Eudicotyledons	Loganiaceae	*Strychnos*	*Strychnos decussata*	EU213858
Eudicotyledons	Loganiaceae	*Strychnos*	*Strychnos decussata*	EU213859
Eudicotyledons	Cucurbitaceae	*Thladiantha*	*Thladiantha hookeri*	JN047403
Eudicotyledons	Cucurbitaceae	*Thladiantha*	*Thladiantha hookeri*	JN047404
Eudicotyledons	Cucurbitaceae	*Thladiantha*	*Thladiantha hookeri*	JN047405
Eudicotyledons	Cucurbitaceae	*Thladiantha*	*Thladiantha hookeri*	JN047406
Eudicotyledons	Cucurbitaceae	*Thladiantha*	*Thladiantha hookeri*	JN047407
Eudicotyledons	Cucurbitaceae	*Thladiantha*	*Thladiantha davidii*	JN047384
Eudicotyledons	Cucurbitaceae	*Thladiantha*	*Thladiantha davidii*	JN047385
Eudicotyledons	Cucurbitaceae	*Thladiantha*	*Thladiantha davidii*	JN047386
Eudicotyledons	Cucurbitaceae	*Thladiantha*	*Thladiantha davidii*	JN047387
Eudicotyledons	Ranunculaceae	*Anemone*	*Anemone acutiloba*	HQ596595
Eudicotyledons	Ranunculaceae	*Anemone*	*Anemone acutiloba*	HQ596596
Eudicotyledons	Ranunculaceae	*Anemone*	*Anemone raddeana*	EF139280
Eudicotyledons	Ranunculaceae	*Anemone*	*Anemone raddeana*	EF139281
Eudicotyledons	Ranunculaceae	*Anemone*	*Anemone raddeana*	EF139282
Eudicotyledons	Ranunculaceae	*Anemone*	*Anemone raddeana*	EF139283
Eudicotyledons	Ranunculaceae	*Anemone*	*Anemone raddeana*	EF139284
Eudicotyledons	Ranunculaceae	*Anemone*	*Anemone raddeana*	EF139285
Eudicotyledons	Ranunculaceae	*Anemone*	*Anemone raddeana*	EF139286
Eudicotyledons	Ranunculaceae	*Anemone*	*Anemone raddeana*	EF139287
Eudicotyledons	Ranunculaceae	*Anemone*	*Anemone raddeana*	EF139288
Eudicotyledons	Lamiaceae	*Salvia*	*Salvia cacaliifolia*	DQ667367
Eudicotyledons	Lamiaceae	*Salvia*	*Salvia cacaliifolia*	HQ418903
Eudicotyledons	Lamiaceae	*Salvia*	*Salvia patens*	DQ667361
Eudicotyledons	Lamiaceae	*Salvia*	*Salvia patens*	HQ418937
Eudicotyledons	Lamiaceae	*Salvia*	*Salvia prunelloides*	DQ667371
Eudicotyledons	Lamiaceae	*Salvia*	*Salvia prunelloides*	HQ418938
Eudicotyledons	Lamiaceae	*Salvia*	*Salvia roborowskii*	DQ667384
Eudicotyledons	Lamiaceae	*Salvia*	*Salvia roborowskii*	FJ513126
Eudicotyledons	Asteraceae	*Elephantopus*	*Elephantopus scaber*	GQ435083
Eudicotyledons	Asteraceae	*Elephantopus*	*Elephantopus scaber*	JN406935
Eudicotyledons	Asteraceae	*Elephantopus*	*Elephantopus scaber*	JN406936
Eudicotyledons	Asteraceae	*Elephantopus*	*Elephantopus scaber*	JN406937
Eudicotyledons	Asteraceae	*Elephantopus*	*Elephantopus scaber*	JN406938
Eudicotyledons	Berberidaceae	*Berberis*	*Berberis chitria*	GU934930
Eudicotyledons	Berberidaceae	*Berberis*	*Berberis chitria*	GU934931
Eudicotyledons	Berberidaceae	*Berberis*	*Berberis chitria*	GU934932
Eudicotyledons	Berberidaceae	*Berberis*	*Berberis chitria*	GU934933

Eudicotyledons	Berberidaceae	*Berberis*	*Berberis chitria*	GU934935
Eudicotyledons	Berberidaceae	*Berberis*	*Berberis chitria*	GU934936
Eudicotyledons	Berberidaceae	*Berberis*	*Berberis chitria*	GU934937
Eudicotyledons	Rutaceae	*Citrus*	*Citrus limetta*	JN315368
Eudicotyledons	Rutaceae	*Citrus*	*Citrus limetta*	JN315369
Eudicotyledons	Rosaceae	*Crataegus*	*Crataegus brachyacantha*	EF127137
Eudicotyledons	Rosaceae	*Crataegus*	*Crataegus brachyacantha*	EU682691
Eudicotyledons	Rosaceae	*Crataegus*	*Crataegus calpodendron*	EF127120
Eudicotyledons	Rosaceae	*Crataegus*	*Crataegus calpodendron*	EU682690
Eudicotyledons	Rosaceae	*Crataegus*	*Crataegus chlorosarca*	EF127147
Eudicotyledons	Rosaceae	*Crataegus*	*Crataegus chlorosarca*	EU682698
Eudicotyledons	Rosaceae	*Crataegus*	*Crataegus heldreichii*	EF127127
Eudicotyledons	Rosaceae	*Crataegus*	*Crataegus heldreichii*	EU500295
Eudicotyledons	Rosaceae	*Crataegus*	*Crataegus marshallii*	EF127132
Eudicotyledons	Rosaceae	*Crataegus*	*Crataegus marshallii*	EU682703
Eudicotyledons	Rosaceae	*Crataegus*	*Crataegus maximowiczii*	EF127146
Eudicotyledons	Rosaceae	*Crataegus*	*Crataegus maximowiczii*	EU682697
Eudicotyledons	Rosaceae	*Crataegus*	*Crataegus opaca*	EF127125
Eudicotyledons	Rosaceae	*Crataegus*	*Crataegus opaca*	EU682699
Eudicotyledons	Rosaceae	*Crataegus*	*Crataegus pentagyna*	EF127131
Eudicotyledons	Rosaceae	*Crataegus*	*Crataegus pentagyna*	EU682702
Eudicotyledons	Rosaceae	*Crataegus*	*Crataegus phaenopyrum*	EF127133
Eudicotyledons	Rosaceae	*Crataegus*	*Crataegus phaenopyrum*	EU682700
Eudicotyledons	Rosaceae	*Crataegus*	*Crataegus punctata*	EF127123
Eudicotyledons	Rosaceae	*Crataegus*	*Crataegus punctata*	HQ596659
Eudicotyledons	Rosaceae	*Crataegus*	*Crataegus punctata*	EU682695
Eudicotyledons	Rosaceae	*Crataegus*	*Crataegus saligna*	EF127138
Eudicotyledons	Rosaceae	*Crataegus*	*Crataegus saligna*	EU682694
Eudicotyledons	Rosaceae	*Crataegus*	*Crataegus songarica*	EF127129
Eudicotyledons	Rosaceae	*Crataegus*	*Crataegus songarica*	EU500300
Eudicotyledons	Rosaceae	*Crataegus*	*Crataegus spathulata*	EF127134
Eudicotyledons	Rosaceae	*Crataegus*	*Crataegus spathulata*	EU682701
Eudicotyledons	Rosaceae	*Crataegus*	*Crataegus suksdorfii*	EF127139
Eudicotyledons	Rosaceae	*Crataegus*	*Crataegus suksdorfii*	EF127140
Eudicotyledons	Rosaceae	*Crataegus*	*Crataegus suksdorfii*	EU572741
Eudicotyledons	Rosaceae	*Crataegus*	*Crataegus suksdorfii*	EU572742
Eudicotyledons	Rosaceae	*Crataegus*	*Crataegus suksdorfii*	EU572743
Eudicotyledons	Rosaceae	*Crataegus*	*Crataegus suksdorfii*	EU572744
Eudicotyledons	Rosaceae	*Crataegus*	*Crataegus suksdorfii*	EU572745
Eudicotyledons	Rosaceae	*Crataegus*	*Crataegus suksdorfii*	EU572746
Eudicotyledons	Rosaceae	*Crataegus*	*Crataegus suksdorfii*	EU572747
Eudicotyledons	Rosaceae	*Crataegus*	*Crataegus suksdorfii*	EU572748
Eudicotyledons	Rosaceae	*Crataegus*	*Crataegus suksdorfii*	EU572749
Eudicotyledons	Rosaceae	*Crataegus*	*Crataegus suksdorfii*	EU572750
Eudicotyledons	Rosaceae	*Crataegus*	*Crataegus suksdorfii*	EU572751
Eudicotyledons	Rosaceae	*Crataegus*	*Crataegus suksdorfii*	EU572752
Eudicotyledons	Rosaceae	*Crataegus*	*Crataegus suksdorfii*	EU572753
Eudicotyledons	Rosaceae	*Crataegus*	*Crataegus suksdorfii*	EU572754
Eudicotyledons	Rosaceae	*Crataegus*	*Crataegus suksdorfii*	EU572755
Eudicotyledons	Rosaceae	*Crataegus*	*Crataegus suksdorfii*	EU572756
Eudicotyledons	Rosaceae	*Crataegus*	*Crataegus suksdorfii*	EU572757
Eudicotyledons	Rosaceae	*Crataegus*	*Crataegus suksdorfii*	EU572758
Eudicotyledons	Rosaceae	*Crataegus*	*Crataegus suksdorfii*	EU572759

Eudicotyledons	Rosaceae	*Crataegus*	*Crataegus suksdorfii*	EU572760
Eudicotyledons	Rosaceae	*Crataegus*	*Crataegus suksdorfii*	EU572761
Eudicotyledons	Rosaceae	*Crataegus*	*Crataegus suksdorfii*	EU572762
Eudicotyledons	Rosaceae	*Crataegus*	*Crataegus suksdorfii*	EU572763
Eudicotyledons	Rosaceae	*Crataegus*	*Crataegus suksdorfii*	EU572764
Eudicotyledons	Rosaceae	*Crataegus*	*Crataegus suksdorfii*	EU572765
Eudicotyledons	Rosaceae	*Crataegus*	*Crataegus suksdorfii*	EU572766
Eudicotyledons	Rosaceae	*Crataegus*	*Crataegus suksdorfii*	EU572767
Eudicotyledons	Rosaceae	*Crataegus*	*Crataegus suksdorfii*	EU572768
Eudicotyledons	Rosaceae	*Crataegus*	*Crataegus suksdorfii*	EU572769
Eudicotyledons	Rosaceae	*Crataegus*	*Crataegus suksdorfii*	EU572770
Eudicotyledons	Rosaceae	*Crataegus*	*Crataegus suksdorfii*	EU572771
Eudicotyledons	Rosaceae	*Crataegus*	*Crataegus suksdorfii*	EU572772
Eudicotyledons	Rosaceae	*Crataegus*	*Crataegus suksdorfii*	EU572773
Eudicotyledons	Rosaceae	*Crataegus*	*Crataegus suksdorfii*	EU572774
Eudicotyledons	Rosaceae	*Crataegus*	*Crataegus suksdorfii*	EU572775
Eudicotyledons	Rosaceae	*Crataegus*	*Crataegus suksdorfii*	EU572776
Eudicotyledons	Rosaceae	*Crataegus*	*Crataegus suksdorfii*	EU572777
Eudicotyledons	Rosaceae	*Crataegus*	*Crataegus suksdorfii*	EU572778
Eudicotyledons	Rosaceae	*Crataegus*	*Crataegus suksdorfii*	EU572779
Eudicotyledons	Rosaceae	*Crataegus*	*Crataegus suksdorfii*	EU572780
Eudicotyledons	Rosaceae	*Crataegus*	*Crataegus suksdorfii*	EU572781
Eudicotyledons	Rosaceae	*Crataegus*	*Crataegus suksdorfii*	EU572782
Eudicotyledons	Rosaceae	*Crataegus*	*Crataegus suksdorfii*	EU572783
Eudicotyledons	Rosaceae	*Crataegus*	*Crataegus suksdorfii*	EU572822
Eudicotyledons	Rosaceae	*Crataegus*	*Crataegus suksdorfii*	EU572823
Eudicotyledons	Rosaceae	*Crataegus*	*Crataegus suksdorfii*	EU572824
Eudicotyledons	Rosaceae	*Crataegus*	*Crataegus suksdorfii*	EU572825
Eudicotyledons	Rosaceae	*Crataegus*	*Crataegus suksdorfii*	EU572826
Eudicotyledons	Rosaceae	*Crataegus*	*Crataegus suksdorfii*	EU572827
Eudicotyledons	Rosaceae	*Crataegus*	*Crataegus suksdorfii*	EU572828
Eudicotyledons	Rosaceae	*Crataegus*	*Crataegus suksdorfii*	EU572829
Eudicotyledons	Rosaceae	*Crataegus*	*Crataegus suksdorfii*	EU572830
Eudicotyledons	Rosaceae	*Crataegus*	*Crataegus suksdorfii*	EU572831
Eudicotyledons	Rosaceae	*Crataegus*	*Crataegus suksdorfii*	EU572832
Eudicotyledons	Rosaceae	*Crataegus*	*Crataegus suksdorfii*	EU572833
Eudicotyledons	Rosaceae	*Crataegus*	*Crataegus suksdorfii*	EU572834
Eudicotyledons	Rosaceae	*Crataegus*	*Crataegus suksdorfii*	EU572835
Eudicotyledons	Rosaceae	*Crataegus*	*Crataegus suksdorfii*	EU572836
Eudicotyledons	Rosaceae	*Crataegus*	*Crataegus suksdorfii*	EU572837
Eudicotyledons	Rosaceae	*Crataegus*	*Crataegus suksdorfii*	EU572850
Eudicotyledons	Rosaceae	*Crataegus*	*Crataegus suksdorfii*	EU572851
Eudicotyledons	Rosaceae	*Crataegus*	*Crataegus suksdorfii*	EU572852
Eudicotyledons	Rosaceae	*Crataegus*	*Crataegus suksdorfii*	EU572853
Eudicotyledons	Rosaceae	*Crataegus*	*Crataegus suksdorfii*	EU572854
Eudicotyledons	Rosaceae	*Crataegus*	*Crataegus suksdorfii*	EU572855
Eudicotyledons	Rosaceae	*Crataegus*	*Crataegus suksdorfii*	EU572856
Eudicotyledons	Rosaceae	*Crataegus*	*Crataegus suksdorfii*	EU572857
Eudicotyledons	Rosaceae	*Crataegus*	*Crataegus suksdorfii*	EU572858
Eudicotyledons	Rosaceae	*Crataegus*	*Crataegus suksdorfii*	EU572859
Eudicotyledons	Rosaceae	*Crataegus*	*Crataegus suksdorfii*	EU572860
Eudicotyledons	Rosaceae	*Crataegus*	*Crataegus suksdorfii*	EU572861
Eudicotyledons	Rosaceae	*Crataegus*	*Crataegus suksdorfii*	EU572862

Eudicotyledons	Rosaceae	*Crataegus*	*Crataegus suksdorfii*	EU682692
Eudicotyledons	Rosaceae	*Crataegus*	*Crataegus suksdorfii*	EU682693
Eudicotyledons	Rosaceae	*Crataegus*	*Crataegus triflora*	EF127121
Eudicotyledons	Rosaceae	*Crataegus*	*Crataegus triflora*	EU500260
Eudicotyledons	Rosaceae	*Crataegus*	*Crataegus uniflora*	EF127149
Eudicotyledons	Rosaceae	*Crataegus*	*Crataegus uniflora*	EU682689
Eudicotyledons	Rosaceae	*Crataegus*	*Crataegus viridis*	EF127150
Eudicotyledons	Rosaceae	*Crataegus*	*Crataegus viridis*	EU682688
Eudicotyledons	Solanaceae	*Lycium*	*Lycium pumilum*	HM195010
Eudicotyledons	Solanaceae	*Lycium*	*Lycium pumilum*	FJ189621
Eudicotyledons	Solanaceae	*Nolana*	*Nolana coelestis*	FJ189605
Eudicotyledons	Solanaceae	*Nolana*	*Nolana coelestis*	EU742457
Eudicotyledons	Solanaceae	*Nolana*	*Nolana werdermannii*	FJ189604
Eudicotyledons	Solanaceae	*Nolana*	*Nolana werdermannii*	EU742506
Eudicotyledons	Ranunculaceae	*Anemone*	*Anemone amurensis*	EF139299
Eudicotyledons	Ranunculaceae	*Anemone*	*Anemone amurensis*	EF139300
Eudicotyledons	Ranunculaceae	*Anemone*	*Anemone amurensis*	EF139301
Eudicotyledons	Ranunculaceae	*Anemone*	*Anemone amurensis*	EF139302
Eudicotyledons	Ranunculaceae	*Anemone*	*Anemone amurensis*	EF139303
Eudicotyledons	Ranunculaceae	*Anemone*	*Anemone amurensis*	EF139304
Eudicotyledons	Ranunculaceae	*Anemone*	*Anemone amurensis*	EF139305
Eudicotyledons	Ranunculaceae	*Anemone*	*Anemone amurensis*	EF139306
Eudicotyledons	Ranunculaceae	*Anemone*	*Anemone amurensis*	EF139307
Eudicotyledons	Ranunculaceae	*Anemone*	*Anemone pendulisepala*	EF139289
Eudicotyledons	Ranunculaceae	*Anemone*	*Anemone pendulisepala*	EF139290
Eudicotyledons	Ranunculaceae	*Anemone*	*Anemone pendulisepala*	EF139291
Eudicotyledons	Ranunculaceae	*Anemone*	*Anemone pendulisepala*	EF139292
Eudicotyledons	Ranunculaceae	*Anemone*	*Anemone pendulisepala*	EF139293
Eudicotyledons	Ranunculaceae	*Anemone*	*Anemone pendulisepala*	EF139294
Eudicotyledons	Ranunculaceae	*Anemone*	*Anemone pendulisepala*	EF139295
Eudicotyledons	Ranunculaceae	*Anemone*	*Anemone pendulisepala*	EF139296
Eudicotyledons	Ranunculaceae	*Anemone*	*Anemone pendulisepala*	EF139297
Eudicotyledons	Ranunculaceae	*Anemone*	*Anemone pendulisepala*	EF139298
Eudicotyledons	Ranunculaceae	*Anemone*	*Anemone reflexa*	EF139308
Eudicotyledons	Ranunculaceae	*Anemone*	*Anemone reflexa*	EF139309
Eudicotyledons	Ranunculaceae	*Anemone*	*Anemone reflexa*	EF139310
Eudicotyledons	Ranunculaceae	*Anemone*	*Anemone reflexa*	EF139311
Eudicotyledons	Ranunculaceae	*Anemone*	*Anemone reflexa*	EF139312
Eudicotyledons	Ranunculaceae	*Anemone*	*Anemone reflexa*	EF139313
Eudicotyledons	Ranunculaceae	*Anemone*	*Anemone stolonifera*	EF139278
Eudicotyledons	Ranunculaceae	*Anemone*	*Anemone stolonifera*	EF139279
Eudicotyledons	Lamiaceae	*Salvia*	*Salvia plebeia*	JQ339261
Eudicotyledons	Lamiaceae	*Salvia*	*Salvia plebeia*	JQ339262
Eudicotyledons	Lamiaceae	*Salvia*	*Salvia plebeia*	FJ513114
Eudicotyledons	Lamiaceae	*Isodon*	*Isodon japonicus*	AB446238
Eudicotyledons	Lamiaceae	*Isodon*	*Isodon japonicus*	AB446239
Eudicotyledons	Lamiaceae	*Isodon*	*Isodon japonicus*	AB446240
Eudicotyledons	Lamiaceae	*Isodon*	*Isodon japonicus*	AB556804
Eudicotyledons	Lamiaceae	*Isodon*	*Isodon japonicus*	AB556815
Eudicotyledons	Lamiaceae	*Isodon*	*Isodon japonicus*	FJ513115
Eudicotyledons	Lamiaceae	*Isodon*	*Isodon trichocarpus*	AB556805
Eudicotyledons	Lamiaceae	*Isodon*	*Isodon trichocarpus*	AB556810
Eudicotyledons	Tamaricaceae	*Myricaria*	*Myricaria paniculata*	EU240616

Eudicotyledons	Tamaricaceae	*Myricaria*	*Myricaria paniculata*	EF394258
Eudicotyledons	Tamaricaceae	*Myricaria*	*Myricaria paniculata*	EF394259
Eudicotyledons	Tamaricaceae	*Myricaria*	*Myricaria paniculata*	EF394260
Eudicotyledons	Tamaricaceae	*Myricaria*	*Myricaria paniculata*	EF394261
Eudicotyledons	Tamaricaceae	*Myricaria*	*Myricaria paniculata*	EF394262
Eudicotyledons	Tamaricaceae	*Myricaria*	*Myricaria paniculata*	EF394263
Eudicotyledons	Tamaricaceae	*Myricaria*	*Myricaria platyphylla*	EU240618
Eudicotyledons	Tamaricaceae	*Myricaria*	*Myricaria platyphylla*	EF394284
Eudicotyledons	Tamaricaceae	*Myricaria*	*Myricaria platyphylla*	EF394285
Eudicotyledons	Tamaricaceae	*Myricaria*	*Myricaria prostrata*	EU240624
Eudicotyledons	Tamaricaceae	*Myricaria*	*Myricaria prostrata*	EF394292
Eudicotyledons	Tamaricaceae	*Myricaria*	*Myricaria pulcherrima*	EU240619
Eudicotyledons	Tamaricaceae	*Myricaria*	*Myricaria pulcherrima*	EF394286
Eudicotyledons	Tamaricaceae	*Myricaria*	*Myricaria pulcherrima*	EF394287
Eudicotyledons	Tamaricaceae	*Myricaria*	*Myricaria pulcherrima*	EF394288
Eudicotyledons	Tamaricaceae	*Myricaria*	*Myricaria pulcherrima*	EF394289
Eudicotyledons	Tamaricaceae	*Myricaria*	*Myricaria pulcherrima*	EF394290
Eudicotyledons	Tamaricaceae	*Myricaria*	*Myricaria rosea*	EU240621
Eudicotyledons	Tamaricaceae	*Myricaria*	*Myricaria rosea*	EF394291
Eudicotyledons	Tamaricaceae	*Myricaria*	*Myricaria wardii*	EU240622
Eudicotyledons	Tamaricaceae	*Myricaria*	*Myricaria wardii*	EU240623
Eudicotyledons	Tamaricaceae	*Myricaria*	*Myricaria wardii*	EF394274
Eudicotyledons	Tamaricaceae	*Myricaria*	*Myricaria wardii*	EF394275
Eudicotyledons	Tamaricaceae	*Myricaria*	*Myricaria wardii*	EF394276
Eudicotyledons	Tamaricaceae	*Myricaria*	*Myricaria wardii*	EF394277
Eudicotyledons	Tamaricaceae	*Myricaria*	*Myricaria wardii*	EF394278
Eudicotyledons	Tamaricaceae	*Myricaria*	*Myricaria wardii*	EF394279
Eudicotyledons	Tamaricaceae	*Myricaria*	*Myricaria wardii*	EF394280
Eudicotyledons	Tamaricaceae	*Myricaria*	*Myricaria wardii*	EF394281
Eudicotyledons	Tamaricaceae	*Myricaria*	*Myricaria wardii*	EF394282
Eudicotyledons	Tamaricaceae	*Myricaria*	*Myricaria wardii*	EF394283
Eudicotyledons	Tamaricaceae	*Myricaria*	*Myricaria wardii*	EF394296
Eudicotyledons	Hydrangeaceae	*Hydrangea*	*Hydrangea xanthoneura*	GQ248320
Eudicotyledons	Hydrangeaceae	*Hydrangea*	*Hydrangea xanthoneura*	EF590703
Eudicotyledons	Hydrangeaceae	*Hydrangea*	*Hydrangea xanthoneura*	HM216996
Eudicotyledons	Rosaceae	*Photinia*	*Photinia beauverdiana*	JQ390759
Eudicotyledons	Rosaceae	*Photinia*	*Photinia beauverdiana*	HQ427047
Eudicotyledons	Rosaceae	*Photinia*	*Photinia beauverdiana*	GQ305321
Eudicotyledons	Rosaceae	*Photinia*	*Photinia parvifolia*	HQ427049
Eudicotyledons	Rosaceae	*Photinia*	*Photinia parvifolia*	GQ305319
Eudicotyledons	Rosaceae	*Potentilla*	*Potentilla pensylvanica*	GQ384994
Eudicotyledons	Rosaceae	*Potentilla*	*Potentilla pensylvanica*	GQ385023
Eudicotyledons	Solanaceae	*Nolana*	*Nolana lezamae*	EU742477
Eudicotyledons	Solanaceae	*Nolana*	*Nolana lezamae*	FJ914093
Eudicotyledons	Solanaceae	*Nolana*	*Nolana arequipensis*	EU742448
Eudicotyledons	Solanaceae	*Nolana*	*Nolana arequipensis*	FJ914082
Eudicotyledons	Solanaceae	*Nolana*	*Nolana plicata*	EU742486
Eudicotyledons	Solanaceae	*Nolana*	*Nolana plicata*	FJ914083
Eudicotyledons	Solanaceae	*Nolana*	*Nolana sessiliflora*	EU742494
Eudicotyledons	Solanaceae	*Nolana*	*Nolana sessiliflora*	FJ914065
Eudicotyledons	Moraceae	*Ficus*	*Ficus hispida*	GU935101
Eudicotyledons	Moraceae	*Ficus*	*Ficus hispida*	GU935102
Eudicotyledons	Moraceae	*Ficus*	*Ficus hispida*	GU935103

Eudicotyledons	Moraceae	*Ficus*	*Ficus hispida*	JN044527
Eudicotyledons	Moraceae	*Ficus*	*Ficus hispida*	JN044528
Eudicotyledons	Moraceae	*Ficus*	*Ficus cyrtophylla*	JN044513
Eudicotyledons	Moraceae	*Ficus*	*Ficus cyrtophylla*	JN044514
Eudicotyledons	Moraceae	*Ficus*	*Ficus cyrtophylla*	JN044515
Eudicotyledons	Moraceae	*Ficus*	*Ficus semicordata*	JN044536
Eudicotyledons	Moraceae	*Ficus*	*Ficus semicordata*	JN044537
Eudicotyledons	Moraceae	*Ficus*	*Ficus semicordata*	JN044538
Eudicotyledons	Moraceae	*Ficus*	*Ficus semicordata*	JN044539
Eudicotyledons	Moraceae	*Ficus*	*Ficus tikoua*	JN044540
Eudicotyledons	Moraceae	*Ficus*	*Ficus tikoua*	JN044541
Eudicotyledons	Moraceae	*Ficus*	*Ficus vasculosa*	HQ415499
Eudicotyledons	Moraceae	*Ficus*	*Ficus vasculosa*	JN044548
Eudicotyledons	Moraceae	*Ficus*	*Ficus vasculosa*	JN044549
Eudicotyledons	Melastomataceae	*Miconia*	*Miconia longispicata*	JN661731
Eudicotyledons	Melastomataceae	*Miconia*	*Miconia longispicata*	JN661747
Eudicotyledons	Melastomataceae	*Miconia*	*Miconia longispicata*	JN661759
Eudicotyledons	Moraceae	*Ficus*	*Ficus glaberrima*	JN044518
Eudicotyledons	Moraceae	*Ficus*	*Ficus glaberrima*	JN044519
Eudicotyledons	Moraceae	*Ficus*	*Ficus glaberrima*	JN044520
Eudicotyledons	Moraceae	*Ficus*	*Ficus glaberrima*	JN044521
Eudicotyledons	Anacardiaceae	*Searsia*	*Searsia gueinzii*	EU213842
Eudicotyledons	Anacardiaceae	*Searsia*	*Searsia gueinzii*	EU213843
Eudicotyledons	Anacardiaceae	*Searsia*	*Searsia gueinzii*	EU213844
Eudicotyledons	Anacardiaceae	*Searsia*	*Searsia transvaalensis*	EU213848
Eudicotyledons	Anacardiaceae	*Searsia*	*Searsia transvaalensis*	EU213849
Eudicotyledons	Anacardiaceae	*Searsia*	*Searsia transvaalensis*	EU213850
Eudicotyledons	Loganiaceae	*Strychnos*	*Strychnos madagascariensis*	EU213860
Eudicotyledons	Loganiaceae	*Strychnos*	*Strychnos madagascariensis*	EU213861
Eudicotyledons	Loganiaceae	*Strychnos*	*Strychnos madagascariensis*	EU213862
Eudicotyledons	Rosaceae	*Crataegus*	*Crataegus castlegarensis*	EU500278
Eudicotyledons	Rosaceae	*Crataegus*	*Crataegus castlegarensis*	EU572868
Eudicotyledons	Rosaceae	*Crataegus*	*Crataegus castlegarensis*	EU572869
Eudicotyledons	Rosaceae	*Crataegus*	*Crataegus castlegarensis*	EU572870
Eudicotyledons	Rosaceae	*Crataegus*	*Crataegus castlegarensis*	EU572871
Eudicotyledons	Rosaceae	*Crataegus*	*Crataegus castlegarensis*	EU572872
Eudicotyledons	Rosaceae	*Crataegus*	*Crataegus castlegarensis*	EU572873
Eudicotyledons	Rosaceae	*Crataegus*	*Crataegus chrysocarpa*	GU562399
Eudicotyledons	Rosaceae	*Crataegus*	*Crataegus chrysocarpa*	EU500261
Eudicotyledons	Rosaceae	*Crataegus*	*Crataegus douglasii*	EU500280
Eudicotyledons	Rosaceae	*Crataegus*	*Crataegus douglasii*	EU572784
Eudicotyledons	Rosaceae	*Crataegus*	*Crataegus douglasii*	EU572785
Eudicotyledons	Rosaceae	*Crataegus*	*Crataegus douglasii*	EU572786
Eudicotyledons	Rosaceae	*Crataegus*	*Crataegus douglasii*	EU572787
Eudicotyledons	Rosaceae	*Crataegus*	*Crataegus douglasii*	EU572788
Eudicotyledons	Rosaceae	*Crataegus*	*Crataegus douglasii*	EU572789
Eudicotyledons	Rosaceae	*Crataegus*	*Crataegus douglasii*	EU572790
Eudicotyledons	Rosaceae	*Crataegus*	*Crataegus douglasii*	EU572791
Eudicotyledons	Rosaceae	*Crataegus*	*Crataegus douglasii*	EU572792
Eudicotyledons	Rosaceae	*Crataegus*	*Crataegus douglasii*	EU572793
Eudicotyledons	Rosaceae	*Crataegus*	*Crataegus douglasii*	EU572794
Eudicotyledons	Rosaceae	*Crataegus*	*Crataegus douglasii*	EU572795
Eudicotyledons	Rosaceae	*Crataegus*	*Crataegus douglasii*	EU572796

Eudicotyledons	Rosaceae	*Crataegus*	*Crataegus douglasii*	EU572797
Eudicotyledons	Rosaceae	*Crataegus*	*Crataegus douglasii*	EU572798
Eudicotyledons	Rosaceae	*Crataegus*	*Crataegus douglasii*	EU572799
Eudicotyledons	Rosaceae	*Crataegus*	*Crataegus douglasii*	EU572800
Eudicotyledons	Rosaceae	*Crataegus*	*Crataegus douglasii*	EU572801
Eudicotyledons	Rosaceae	*Crataegus*	*Crataegus douglasii*	EU572802
Eudicotyledons	Rosaceae	*Crataegus*	*Crataegus douglasii*	EU572803
Eudicotyledons	Rosaceae	*Crataegus*	*Crataegus douglasii*	EU572804
Eudicotyledons	Rosaceae	*Crataegus*	*Crataegus douglasii*	EU572805
Eudicotyledons	Rosaceae	*Crataegus*	*Crataegus douglasii*	EU572806
Eudicotyledons	Rosaceae	*Crataegus*	*Crataegus douglasii*	EU572807
Eudicotyledons	Rosaceae	*Crataegus*	*Crataegus douglasii*	EU572808
Eudicotyledons	Rosaceae	*Crataegus*	*Crataegus douglasii*	EU572809
Eudicotyledons	Rosaceae	*Crataegus*	*Crataegus douglasii*	EU572810
Eudicotyledons	Rosaceae	*Crataegus*	*Crataegus douglasii*	EU572811
Eudicotyledons	Rosaceae	*Crataegus*	*Crataegus douglasii*	EU572812
Eudicotyledons	Rosaceae	*Crataegus*	*Crataegus douglasii*	EU572813
Eudicotyledons	Rosaceae	*Crataegus*	*Crataegus douglasii*	EU572814
Eudicotyledons	Rosaceae	*Crataegus*	*Crataegus douglasii*	EU572815
Eudicotyledons	Rosaceae	*Crataegus*	*Crataegus douglasii*	EU572816
Eudicotyledons	Rosaceae	*Crataegus*	*Crataegus douglasii*	EU572817
Eudicotyledons	Rosaceae	*Crataegus*	*Crataegus douglasii*	EU572818
Eudicotyledons	Rosaceae	*Crataegus*	*Crataegus douglasii*	EU572819
Eudicotyledons	Rosaceae	*Crataegus*	*Crataegus douglasii*	EU572820
Eudicotyledons	Rosaceae	*Crataegus*	*Crataegus douglasii*	EU572821
Eudicotyledons	Rosaceae	*Crataegus*	*Crataegus douglasii*	EU572838
Eudicotyledons	Rosaceae	*Crataegus*	*Crataegus douglasii*	EU572839
Eudicotyledons	Rosaceae	*Crataegus*	*Crataegus douglasii*	EU572840
Eudicotyledons	Rosaceae	*Crataegus*	*Crataegus douglasii*	EU572841
Eudicotyledons	Rosaceae	*Crataegus*	*Crataegus douglasii*	EU572842
Eudicotyledons	Rosaceae	*Crataegus*	*Crataegus douglasii*	EU572843
Eudicotyledons	Rosaceae	*Crataegus*	*Crataegus douglasii*	EU572844
Eudicotyledons	Rosaceae	*Crataegus*	*Crataegus douglasii*	EU572845
Eudicotyledons	Rosaceae	*Crataegus*	*Crataegus douglasii*	EU572846
Eudicotyledons	Rosaceae	*Crataegus*	*Crataegus douglasii*	EU572847
Eudicotyledons	Rosaceae	*Crataegus*	*Crataegus douglasii*	EU572848
Eudicotyledons	Rosaceae	*Crataegus*	*Crataegus douglasii*	EU572849
Eudicotyledons	Rosaceae	*Crataegus*	*Crataegus macracantha*	GU562392
Eudicotyledons	Rosaceae	*Crataegus*	*Crataegus macracantha*	HQ596655
Eudicotyledons	Rosaceae	*Crataegus*	*Crataegus macracantha*	EU500259
Eudicotyledons	Rosaceae	*Crataegus*	*Crataegus okennonii*	EU500279
Eudicotyledons	Rosaceae	*Crataegus*	*Crataegus okennonii*	EU572863
Eudicotyledons	Rosaceae	*Crataegus*	*Crataegus okennonii*	EU572864
Eudicotyledons	Rosaceae	*Crataegus*	*Crataegus okennonii*	EU572865
Eudicotyledons	Rosaceae	*Crataegus*	*Crataegus okennonii*	EU572866
Eudicotyledons	Rosaceae	*Crataegus*	*Crataegus okennonii*	EU572867
Eudicotyledons	Rosaceae	*Crataegus*	*Crataegus pinnatifida*	EU500276
Eudicotyledons	Rosaceae	*Crataegus*	*Crataegus pinnatifida*	EU500292
Eudicotyledons	Lamiaceae	*Clinopodium*	*Clinopodium chinense*	EU590865
Eudicotyledons	Lamiaceae	*Clinopodium*	*Clinopodium chinense*	FJ513089
Eudicotyledons	Lamiaceae	*Glechoma*	*Glechoma longituba*	EU590860
Eudicotyledons	Lamiaceae	*Glechoma*	*Glechoma longituba*	JF708216
Eudicotyledons	Fagaceae	*Quercus*	*Quercus macrocarpa*	HQ596806

Eudicotyledons	Fagaceae	*Quercus*	*Quercus macrocarpa*	EU750511
Eudicotyledons	Fagaceae	*Quercus*	*Quercus macrocarpa*	EU750512
Eudicotyledons	Rosaceae	*Potentilla*	*Potentilla recta*	HQ596800
Eudicotyledons	Rosaceae	*Potentilla*	*Potentilla recta*	GQ384960
Eudicotyledons	Fabaceae	*Astragalus*	*Astragalus trichopodus*	GU396746
Eudicotyledons	Fabaceae	*Astragalus*	*Astragalus trichopodus*	GU396747
Eudicotyledons	Fabaceae	*Astragalus*	*Astragalus trichopodus*	GU396748
Eudicotyledons	Lamiaceae	*Phlomis*	*Phlomis fruticosa*	EU627580
Eudicotyledons	Lamiaceae	*Phlomis*	*Phlomis fruticosa*	EU627581
Eudicotyledons	Lamiaceae	*Phlomis*	*Phlomis fruticosa*	EU627582
Eudicotyledons	Lamiaceae	*Phlomis*	*Phlomis fruticosa*	HQ902857
Eudicotyledons	Lamiaceae	*Isodon*	*Isodon longitubus*	AB446204
Eudicotyledons	Lamiaceae	*Isodon*	*Isodon longitubus*	AB446205
Eudicotyledons	Lamiaceae	*Isodon*	*Isodon longitubus*	AB446206
Eudicotyledons	Lamiaceae	*Isodon*	*Isodon longitubus*	AB446207
Eudicotyledons	Lamiaceae	*Isodon*	*Isodon longitubus*	AB446208
Eudicotyledons	Lamiaceae	*Isodon*	*Isodon longitubus*	AB446209
Eudicotyledons	Lamiaceae	*Isodon*	*Isodon longitubus*	AB446210
Eudicotyledons	Lamiaceae	*Isodon*	*Isodon longitubus*	AB446211
Eudicotyledons	Lamiaceae	*Isodon*	*Isodon longitubus*	AB446212
Eudicotyledons	Lamiaceae	*Isodon*	*Isodon longitubus*	AB446213
Eudicotyledons	Lamiaceae	*Isodon*	*Isodon longitubus*	AB446214
Eudicotyledons	Lamiaceae	*Isodon*	*Isodon longitubus*	AB446215
Eudicotyledons	Lamiaceae	*Isodon*	*Isodon longitubus*	AB446216
Eudicotyledons	Lamiaceae	*Isodon*	*Isodon longitubus*	AB446217
Eudicotyledons	Lamiaceae	*Isodon*	*Isodon longitubus*	AB446218
Eudicotyledons	Lamiaceae	*Isodon*	*Isodon longitubus*	AB446219
Eudicotyledons	Lamiaceae	*Isodon*	*Isodon longitubus*	AB446220
Eudicotyledons	Lamiaceae	*Isodon*	*Isodon longitubus*	AB446221
Eudicotyledons	Lamiaceae	*Isodon*	*Isodon longitubus*	AB446222
Eudicotyledons	Lamiaceae	*Isodon*	*Isodon longitubus*	AB556814
Eudicotyledons	Lamiaceae	*Isodon*	*Isodon longitubus*	AB556817
Eudicotyledons	Lamiaceae	*Isodon*	*Isodon inflexus*	AB446223
Eudicotyledons	Lamiaceae	*Isodon*	*Isodon inflexus*	AB446224
Eudicotyledons	Lamiaceae	*Isodon*	*Isodon inflexus*	AB446225
Eudicotyledons	Lamiaceae	*Isodon*	*Isodon inflexus*	AB446226
Eudicotyledons	Lamiaceae	*Isodon*	*Isodon inflexus*	AB446227
Eudicotyledons	Lamiaceae	*Isodon*	*Isodon inflexus*	AB446228
Eudicotyledons	Lamiaceae	*Isodon*	*Isodon inflexus*	AB556811
Eudicotyledons	Lamiaceae	*Isodon*	*Isodon inflexus*	AB556812
Eudicotyledons	Lamiaceae	*Isodon*	*Isodon inflexus*	FJ513099
Eudicotyledons	Gentianaceae	*Gentiana*	*Gentiana rigescens*	GQ435170
Eudicotyledons	Gentianaceae	*Gentiana*	*Gentiana rigescens*	GQ864033
Eudicotyledons	Gentianaceae	*Gentiana*	*Gentiana rigescens*	GQ864034
Eudicotyledons	Rubiaceae	*Psychotria*	*Psychotria mexiae*	FJ208689
Eudicotyledons	Rubiaceae	*Psychotria*	*Psychotria mexiae*	FJ208703
Eudicotyledons	Rubiaceae	*Psychotria*	*Psychotria nubiphila*	FJ208682
Eudicotyledons	Rubiaceae	*Psychotria*	*Psychotria nubiphila*	FJ208684
Eudicotyledons	Rubiaceae	*Psychotria*	*Psychotria psychotriifolia*	FJ208686
Eudicotyledons	Rubiaceae	*Psychotria*	*Psychotria psychotriifolia*	FJ208690
Eudicotyledons	Rubiaceae	*Psychotria*	*Psychotria psychotriifolia*	GQ982345
Eudicotyledons	Rosaceae	*Potentilla*	*Potentilla aurea*	GQ384978
Eudicotyledons	Rosaceae	*Potentilla*	*Potentilla aurea*	GQ384984

Eudicotyledons	Meliaceae	*Carapa*	*Carapa procera*	GQ428752
Eudicotyledons	Meliaceae	*Carapa*	*Carapa procera*	FJ039000
Eudicotyledons	Meliaceae	*Carapa*	*Carapa procera*	FJ039001
Eudicotyledons	Meliaceae	*Carapa*	*Carapa procera*	JF288754
Eudicotyledons	Meliaceae	*Carapa*	*Carapa surinamensis*	JN122307
Eudicotyledons	Meliaceae	*Carapa*	*Carapa surinamensis*	JN122308
Eudicotyledons	Meliaceae	*Carapa*	*Carapa surinamensis*	JN122309
Eudicotyledons	Meliaceae	*Carapa*	*Carapa surinamensis*	JN122310
Eudicotyledons	Meliaceae	*Carapa*	*Carapa surinamensis*	JN122311
Eudicotyledons	Lamiaceae	*Teucrium*	*Teucrium viscidum*	FJ513102
Eudicotyledons	Lamiaceae	*Teucrium*	*Teucrium viscidum*	HM590116
Eudicotyledons	Asteraceae	*Cota*	*Cota pestalozzae*	FR689835
Eudicotyledons	Asteraceae	*Cota*	*Cota pestalozzae*	FR689877
Eudicotyledons	Apocynaceae	*Hoodia*	*Hoodia parviflora*	FJ026608
Eudicotyledons	Apocynaceae	*Hoodia*	*Hoodia parviflora*	FJ026609
Eudicotyledons	Apocynaceae	*Hoodia*	*Hoodia parviflora*	JN117264
Eudicotyledons	Apocynaceae	*Hoodia*	*Hoodia ruschii*	FJ026610
Eudicotyledons	Apocynaceae	*Hoodia*	*Hoodia ruschii*	FJ026611
Eudicotyledons	Apocynaceae	*Hoodia*	*Hoodia ruschii*	JN117265
Eudicotyledons	Chrysobalanaceae	*Licania*	*Licania alba*	GQ428727
Eudicotyledons	Chrysobalanaceae	*Licania*	*Licania alba*	FJ038897
Eudicotyledons	Chrysobalanaceae	*Licania*	*Licania alba*	FJ038898
Eudicotyledons	Chrysobalanaceae	*Licania*	*Licania canescens*	FJ038899
Eudicotyledons	Chrysobalanaceae	*Licania*	*Licania canescens*	FJ038900
Eudicotyledons	Chrysobalanaceae	*Licania*	*Licania canescens*	FJ038901
Eudicotyledons	Chrysobalanaceae	*Licania*	*Licania majuscula*	FJ038902
Eudicotyledons	Chrysobalanaceae	*Licania*	*Licania majuscula*	FJ038903
Eudicotyledons	Chrysobalanaceae	*Licania*	*Licania membranacea*	GQ428772
Eudicotyledons	Chrysobalanaceae	*Licania*	*Licania membranacea*	FJ038904
Eudicotyledons	Sapotaceae	*Micropholis*	*Micropholis cayennensis*	GQ428707
Eudicotyledons	Sapotaceae	*Micropholis*	*Micropholis cayennensis*	FJ039062
Eudicotyledons	Sapotaceae	*Micropholis*	*Micropholis cayennensis*	FJ039063
Eudicotyledons	Asteraceae	*Melampodium*	*Melampodium argophyllum*	FJ845873
Eudicotyledons	Asteraceae	*Melampodium*	*Melampodium argophyllum*	FJ845874
Eudicotyledons	Asteraceae	*Melampodium*	*Melampodium argophyllum*	FJ845875
Eudicotyledons	Asteraceae	*Melampodium*	*Melampodium argophyllum*	FJ845876
Eudicotyledons	Asteraceae	*Melampodium*	*Melampodium argophyllum*	FJ845877
Eudicotyledons	Asteraceae	*Melampodium*	*Melampodium argophyllum*	FJ845878
Eudicotyledons	Asteraceae	*Melampodium*	*Melampodium cupulatum*	GU216580
Eudicotyledons	Asteraceae	*Melampodium*	*Melampodium cupulatum*	GU216581
Eudicotyledons	Asteraceae	*Melampodium*	*Melampodium diffusum*	GU216554
Eudicotyledons	Asteraceae	*Melampodium*	*Melampodium diffusum*	GU216555
Eudicotyledons	Asteraceae	*Melampodium*	*Melampodium linearilobum*	GU216574
Eudicotyledons	Asteraceae	*Melampodium*	*Melampodium linearilobum*	GU216575
Eudicotyledons	Asteraceae	*Melampodium*	*Melampodium longicorne*	GU216569
Eudicotyledons	Asteraceae	*Melampodium*	*Melampodium longicorne*	GU216570
Eudicotyledons	Asteraceae	*Melampodium*	*Melampodium longipes*	GU216560
Eudicotyledons	Asteraceae	*Melampodium*	*Melampodium longipes*	GU216561
Eudicotyledons	Asteraceae	*Melampodium*	*Melampodium mayfieldii*	GU216563
Eudicotyledons	Asteraceae	*Melampodium*	*Melampodium mayfieldii*	GU216564
Eudicotyledons	Asteraceae	*Melampodium*	*Melampodium nayaritense*	GU216571
Eudicotyledons	Asteraceae	*Melampodium*	*Melampodium nayaritense*	GU216572
Eudicotyledons	Asteraceae	*Melampodium*	*Melampodium nayaritense*	GU216573

Eudicotyledons	Asteraceae	*Melampodium*	*Melampodium rosei*	GU216582
Eudicotyledons	Asteraceae	*Melampodium*	*Melampodium rosei*	GU216583
Eudicotyledons	Asteraceae	*Melampodium*	*Melampodium strigosum*	GU216567
Eudicotyledons	Asteraceae	*Melampodium*	*Melampodium strigosum*	GU216568
Eudicotyledons	Asteraceae	*Melampodium*	*Melampodium tenellum*	GU216577
Eudicotyledons	Asteraceae	*Melampodium*	*Melampodium tenellum*	GU216578
Eudicotyledons	Solanaceae	*Nolana*	*Nolana gracillima*	FJ914088
Eudicotyledons	Solanaceae	*Nolana*	*Nolana gracillima*	FJ914092
Eudicotyledons	Solanaceae	*Nolana*	*Nolana pallidula*	FJ914069
Eudicotyledons	Solanaceae	*Nolana*	*Nolana pallidula*	FJ914076
Eudicotyledons	Solanaceae	*Nolana*	*Nolana tovariana*	FJ914071
Eudicotyledons	Solanaceae	*Nolana*	*Nolana tovariana*	FJ914075
Eudicotyledons	Solanaceae	*Nolana*	*Nolana tovariana*	FJ914077
Eudicotyledons	Solanaceae	*Nolana*	*Nolana tovariana*	FJ914080
Eudicotyledons	Solanaceae	*Nolana*	*Nolana tovariana*	FJ914081
Eudicotyledons	Fabaceae	*Astragalus*	*Astragalus membranaceus*	GU396752
Eudicotyledons	Fabaceae	*Astragalus*	*Astragalus membranaceus*	GU396753
Eudicotyledons	Fabaceae	*Astragalus*	*Astragalus membranaceus*	GQ139475
Eudicotyledons	Fabaceae	*Astragalus*	*Astragalus membranaceus*	GQ139476
Eudicotyledons	Fabaceae	*Astragalus*	*Astragalus membranaceus*	GQ139477
Eudicotyledons	Fabaceae	*Astragalus*	*Astragalus membranaceus*	GQ139478
Eudicotyledons	Fabaceae	*Astragalus*	*Astragalus membranaceus*	GQ139479
Eudicotyledons	Fabaceae	*Astragalus*	*Astragalus membranaceus*	GQ139480
Eudicotyledons	Fabaceae	*Astragalus*	*Astragalus membranaceus*	GQ139481
Eudicotyledons	Fabaceae	*Astragalus*	*Astragalus membranaceus*	GQ139482
Eudicotyledons	Fabaceae	*Astragalus*	*Astragalus membranaceus*	GQ139483
Eudicotyledons	Rosaceae	*Potentilla*	*Potentilla neumaniana*	GQ384967
Eudicotyledons	Rosaceae	*Potentilla*	*Potentilla neumaniana*	GQ384973
Eudicotyledons	Rosaceae	*Potentilla*	*Potentilla argentea*	GQ384965
Eudicotyledons	Rosaceae	*Potentilla*	*Potentilla argentea*	GQ384977
Eudicotyledons	Rosaceae	*Potentilla*	*Potentilla argentea*	GQ384986
Eudicotyledons	Rosaceae	*Potentilla*	*Potentilla argentea*	HM776523
Eudicotyledons	Rosaceae	*Potentilla*	*Potentilla argentea*	HM776524
Eudicotyledons	Rosaceae	*Potentilla*	*Potentilla argentea*	HM776525
Eudicotyledons	Rosaceae	*Potentilla*	*Potentilla argentea*	HM776526
Eudicotyledons	Rosaceae	*Potentilla*	*Potentilla argentea*	HM776527
Eudicotyledons	Rosaceae	*Potentilla*	*Potentilla argentea*	HM776528
Eudicotyledons	Rosaceae	*Potentilla*	*Potentilla argentea*	HM776529
Eudicotyledons	Rosaceae	*Potentilla*	*Potentilla argentea*	HM776530
Eudicotyledons	Rosaceae	*Potentilla*	*Potentilla argentea*	HM776531
Eudicotyledons	Rosaceae	*Potentilla*	*Potentilla argentea*	HM776532
Eudicotyledons	Rosaceae	*Potentilla*	*Potentilla argentea*	HM776533
Eudicotyledons	Rosaceae	*Potentilla*	*Potentilla argentea*	HM776534
Eudicotyledons	Rosaceae	*Potentilla*	*Potentilla argentea*	HM776535
Eudicotyledons	Rosaceae	*Potentilla*	*Potentilla argentea*	HM776536
Eudicotyledons	Rosaceae	*Potentilla*	*Potentilla argentea*	HM776537
Eudicotyledons	Rosaceae	*Potentilla*	*Potentilla argentea*	HM776538
Eudicotyledons	Rosaceae	*Potentilla*	*Potentilla argentea*	HM776539
Eudicotyledons	Rosaceae	*Potentilla*	*Potentilla argentea*	HM776540
Eudicotyledons	Rosaceae	*Potentilla*	*Potentilla argentea*	HM776541
Eudicotyledons	Rosaceae	*Potentilla*	*Potentilla argentea*	HM776542
Eudicotyledons	Rosaceae	*Potentilla*	*Potentilla argentea*	HM776543
Eudicotyledons	Rosaceae	*Potentilla*	*Potentilla argentea*	HM776544

Eudicotyledons	Rosaceae	*Potentilla*	*Potentilla argentea*	HM776545
Eudicotyledons	Rosaceae	*Potentilla*	*Potentilla argentea*	HM776546
Eudicotyledons	Rosaceae	*Potentilla*	*Potentilla argentea*	HM776547
Eudicotyledons	Rosaceae	*Potentilla*	*Potentilla argentea*	HM776548
Eudicotyledons	Rosaceae	*Potentilla*	*Potentilla argentea*	HM776549
Eudicotyledons	Rosaceae	*Potentilla*	*Potentilla argentea*	HM776550
Eudicotyledons	Rosaceae	*Potentilla*	*Potentilla argentea*	HM776551
Eudicotyledons	Rosaceae	*Potentilla*	*Potentilla argentea*	HM776552
Eudicotyledons	Rosaceae	*Potentilla*	*Potentilla argentea*	HM776553
Eudicotyledons	Rosaceae	*Potentilla*	*Potentilla argentea*	HM776554
Eudicotyledons	Rosaceae	*Potentilla*	*Potentilla argentea*	HM776555
Eudicotyledons	Rosaceae	*Potentilla*	*Potentilla argentea*	HM776556
Eudicotyledons	Rosaceae	*Potentilla*	*Potentilla argentea*	HM776557
Eudicotyledons	Rosaceae	*Potentilla*	*Potentilla argentea*	HM776558
Eudicotyledons	Rosaceae	*Potentilla*	*Potentilla argentea*	HM776559
Eudicotyledons	Rosaceae	*Potentilla*	*Potentilla argentea*	HM776560
Eudicotyledons	Rosaceae	*Potentilla*	*Potentilla argentea*	HM776561
Eudicotyledons	Rosaceae	*Potentilla*	*Potentilla argentea*	HM776562
Eudicotyledons	Rosaceae	*Potentilla*	*Potentilla argentea*	HM776563
Eudicotyledons	Rosaceae	*Potentilla*	*Potentilla argentea*	HM776564
Eudicotyledons	Rosaceae	*Potentilla*	*Potentilla argentea*	HM776565
Eudicotyledons	Rosaceae	*Potentilla*	*Potentilla argentea*	HM776566
Eudicotyledons	Rosaceae	*Potentilla*	*Potentilla argentea*	HM776567
Eudicotyledons	Rosaceae	*Potentilla*	*Potentilla argentea*	HM776568
Eudicotyledons	Rosaceae	*Potentilla*	*Potentilla argentea*	HM776573
Eudicotyledons	Rosaceae	*Potentilla*	*Potentilla argentea*	HM776574
Eudicotyledons	Rosaceae	*Potentilla*	*Potentilla argentea*	HM776575
Eudicotyledons	Berberidaceae	*Berberis*	*Berberis aristata*	GU934917
Eudicotyledons	Berberidaceae	*Berberis*	*Berberis aristata*	GU934918
Eudicotyledons	Berberidaceae	*Berberis*	*Berberis aristata*	GU934919
Eudicotyledons	Berberidaceae	*Berberis*	*Berberis aristata*	GU934920
Eudicotyledons	Berberidaceae	*Berberis*	*Berberis aristata*	GU934921
Eudicotyledons	Berberidaceae	*Berberis*	*Berberis asiatica*	GU934922
Eudicotyledons	Berberidaceae	*Berberis*	*Berberis asiatica*	GU934923
Eudicotyledons	Berberidaceae	*Berberis*	*Berberis asiatica*	GU934924
Eudicotyledons	Berberidaceae	*Berberis*	*Berberis asiatica*	GU934925
Eudicotyledons	Berberidaceae	*Berberis*	*Berberis asiatica*	GU934926
Eudicotyledons	Berberidaceae	*Berberis*	*Berberis asiatica*	GU934927
Eudicotyledons	Berberidaceae	*Berberis*	*Berberis asiatica*	GU934928
Eudicotyledons	Berberidaceae	*Berberis*	*Berberis asiatica*	GU934929
Eudicotyledons	Berberidaceae	*Berberis*	*Berberis asiatica*	GU934934
Eudicotyledons	Berberidaceae	*Berberis*	*Berberis lycium*	GU934957
Eudicotyledons	Berberidaceae	*Berberis*	*Berberis lycium*	GU934958
Eudicotyledons	Berberidaceae	*Berberis*	*Berberis lycium*	GU934959
Eudicotyledons	Berberidaceae	*Berberis*	*Berberis lycium*	GU934960
Eudicotyledons	Berberidaceae	*Berberis*	*Berberis lycium*	GU934961
Eudicotyledons	Berberidaceae	*Berberis*	*Berberis lycium*	GU934962
Eudicotyledons	Berberidaceae	*Berberis*	*Berberis lycium*	GU934963
Eudicotyledons	Berberidaceae	*Berberis*	*Berberis lycium*	GU934964
Eudicotyledons	Berberidaceae	*Berberis*	*Berberis lycium*	GU934965
Eudicotyledons	Berberidaceae	*Berberis*	*Berberis lycium*	GU934966
Eudicotyledons	Berberidaceae	*Berberis*	*Berberis lycium*	GU934967
Eudicotyledons	Berberidaceae	*Berberis*	*Berberis lycium*	GU934968

Eudicotyledons	Berberidaceae	*Berberis*	*Berberis lycium*	GU934969
Eudicotyledons	Berberidaceae	*Berberis*	*Berberis lycium*	GU934970
Eudicotyledons	Berberidaceae	*Berberis*	*Berberis lycium*	GU934971
Eudicotyledons	Berberidaceae	*Berberis*	*Berberis lycium*	GU934972
Eudicotyledons	Rosaceae	*Aronia*	*Aronia melanocarpa*	JQ390631
Eudicotyledons	Rosaceae	*Aronia*	*Aronia melanocarpa*	GQ305340
Eudicotyledons	Rosaceae	*Photinia*	*Photinia prunifolia*	HQ415532
Eudicotyledons	Rosaceae	*Photinia*	*Photinia prunifolia*	GQ305342
Eudicotyledons	Rosaceae	*Photinia*	*Photinia raupingensis*	GQ305341
Eudicotyledons	Rosaceae	*Photinia*	*Photinia raupingensis*	GQ305355
Eudicotyledons	Lamiaceae	*Isodon*	*Isodon bulleyanus*	JN045032
Eudicotyledons	Lamiaceae	*Isodon*	*Isodon bulleyanus*	JN045033
Eudicotyledons	Lamiaceae	*Isodon*	*Isodon bulleyanus*	JN045034
Eudicotyledons	Lamiaceae	*Isodon*	*Isodon flabelliformis*	JN045035
Eudicotyledons	Lamiaceae	*Isodon*	*Isodon flabelliformis*	JN045036
Eudicotyledons	Lamiaceae	*Isodon*	*Isodon loxothyrsus*	JN045039
Eudicotyledons	Lamiaceae	*Isodon*	*Isodon loxothyrsus*	JN045040
Eudicotyledons	Lamiaceae	*Isodon*	*Isodon melissoides*	JN045041
Eudicotyledons	Lamiaceae	*Isodon*	*Isodon melissoides*	JN045042
Eudicotyledons	Lamiaceae	*Isodon*	*Isodon sculponeatus*	JN045043
Eudicotyledons	Lamiaceae	*Isodon*	*Isodon sculponeatus*	JN045044
Eudicotyledons	Lamiaceae	*Isodon*	*Isodon yuennanensis*	JN045045
Eudicotyledons	Lamiaceae	*Isodon*	*Isodon yuennanensis*	JN045046
Eudicotyledons	Moraceae	*Ficus*	*Ficus esquiroliana*	HQ415502
Eudicotyledons	Moraceae	*Ficus*	*Ficus esquiroliana*	JN044516
Eudicotyledons	Moraceae	*Ficus*	*Ficus esquiroliana*	JN044517
Eudicotyledons	Rosaceae	*Potentilla*	*Potentilla alpicola*	HQ433263
Eudicotyledons	Rosaceae	*Potentilla*	*Potentilla alpicola*	GQ384980
Eudicotyledons	Rosaceae	*Potentilla*	*Potentilla incana*	GQ384971
Eudicotyledons	Rosaceae	*Potentilla*	*Potentilla incana*	GQ384972
Eudicotyledons	Rosaceae	*Potentilla*	*Potentilla pulcherrima*	GQ384995
Eudicotyledons	Rosaceae	*Potentilla*	*Potentilla pulcherrima*	GQ385014
Eudicotyledons	Rosaceae	*Potentilla*	*Potentilla velutina*	GQ384966
Eudicotyledons	Rosaceae	*Potentilla*	*Potentilla velutina*	GQ384969
Eudicotyledons	Lamiaceae	*Isodon*	*Isodon effusus*	AB556806
Eudicotyledons	Lamiaceae	*Isodon*	*Isodon effusus*	AB556808
Eudicotyledons	Gentianaceae	*Gentiana*	*Gentiana manshurica*	GQ864029
Eudicotyledons	Gentianaceae	*Gentiana*	*Gentiana manshurica*	GQ864030
Eudicotyledons	Betulaceae	*Alnus*	*Alnus fauriei*	FJ844516
Eudicotyledons	Betulaceae	*Alnus*	*Alnus fauriei*	FJ844517
Eudicotyledons	Betulaceae	*Alnus*	*Alnus serrulatoides*	FJ844553
Eudicotyledons	Betulaceae	*Alnus*	*Alnus serrulatoides*	FJ844554
Eudicotyledons	Sapotaceae	*Micropholis*	*Micropholis sanctae-rosae*	GQ428710
Eudicotyledons	Sapotaceae	*Micropholis*	*Micropholis sanctae-rosae*	GQ428711
Eudicotyledons	Nothofagaceae	*Nothofagus*	*Nothofagus nervosa*	GQ863327
Eudicotyledons	Nothofagaceae	*Nothofagus*	*Nothofagus nervosa*	GQ863330
Eudicotyledons	Cucurbitaceae	*Thladiantha*	*Thladiantha cordifolia*	GQ163094
Eudicotyledons	Cucurbitaceae	*Thladiantha*	*Thladiantha cordifolia*	JN047372
Eudicotyledons	Cucurbitaceae	*Thladiantha*	*Thladiantha cordifolia*	JN047373
Eudicotyledons	Cucurbitaceae	*Thladiantha*	*Thladiantha cordifolia*	JN047374
Eudicotyledons	Cucurbitaceae	*Thladiantha*	*Thladiantha cordifolia*	JN047375
Eudicotyledons	Cucurbitaceae	*Thladiantha*	*Thladiantha cordifolia*	JN047376
Eudicotyledons	Cucurbitaceae	*Thladiantha*	*Thladiantha cordifolia*	JN047377

Eudicotyledons	Cucurbitaceae	*Thladiantha*	*Thladiantha cordifolia*	JN047378
Eudicotyledons	Cucurbitaceae	*Thladiantha*	*Thladiantha cordifolia*	JN047379
Eudicotyledons	Cucurbitaceae	*Thladiantha*	*Thladiantha cordifolia*	JN047380
Eudicotyledons	Cucurbitaceae	*Thladiantha*	*Thladiantha cordifolia*	JN047381
Eudicotyledons	Cucurbitaceae	*Thladiantha*	*Thladiantha cordifolia*	JN047382
Eudicotyledons	Cucurbitaceae	*Thladiantha*	*Thladiantha cordifolia*	JN047383
Eudicotyledons	Cucurbitaceae	*Thladiantha*	*Thladiantha nudiflora*	GQ163095
Eudicotyledons	Cucurbitaceae	*Thladiantha*	*Thladiantha nudiflora*	JN047429
Eudicotyledons	Cucurbitaceae	*Thladiantha*	*Thladiantha nudiflora*	JN047430
Eudicotyledons	Cucurbitaceae	*Thladiantha*	*Thladiantha nudiflora*	JN047431
Eudicotyledons	Cucurbitaceae	*Thladiantha*	*Thladiantha nudiflora*	JN047432
Eudicotyledons	Cucurbitaceae	*Thladiantha*	*Thladiantha nudiflora*	JN047433
Eudicotyledons	Cucurbitaceae	*Thladiantha*	*Thladiantha nudiflora*	JN047434
Eudicotyledons	Cucurbitaceae	*Thladiantha*	*Thladiantha nudiflora*	JN047435
Eudicotyledons	Cucurbitaceae	*Thladiantha*	*Thladiantha nudiflora*	JN047436
Eudicotyledons	Cucurbitaceae	*Thladiantha*	*Thladiantha nudiflora*	JN047437
Eudicotyledons	Cucurbitaceae	*Thladiantha*	*Thladiantha nudiflora*	JN047438
Eudicotyledons	Rosaceae	*Sorbaria*	*Sorbaria kirilowii*	JN047238
Eudicotyledons	Rosaceae	*Sorbaria*	*Sorbaria kirilowii*	JN047239
Eudicotyledons	Berberidaceae	*Berberis*	*Berberis angulosa*	GU934914
Eudicotyledons	Berberidaceae	*Berberis*	*Berberis angulosa*	GU934915
Eudicotyledons	Berberidaceae	*Berberis*	*Berberis angulosa*	GU934916
Eudicotyledons	Berberidaceae	*Berberis*	*Berberis glaucocarpa*	GU934938
Eudicotyledons	Berberidaceae	*Berberis*	*Berberis glaucocarpa*	GU934939
Eudicotyledons	Berberidaceae	*Berberis*	*Berberis glaucocarpa*	GU934940
Eudicotyledons	Berberidaceae	*Berberis*	*Berberis glaucocarpa*	GU934941
Eudicotyledons	Berberidaceae	*Berberis*	*Berberis glaucocarpa*	GU934942
Eudicotyledons	Berberidaceae	*Berberis*	*Berberis glaucocarpa*	GU934943
Eudicotyledons	Berberidaceae	*Berberis*	*Berberis glaucocarpa*	GU934944
Eudicotyledons	Berberidaceae	*Berberis*	*Berberis glaucocarpa*	GU934945
Eudicotyledons	Berberidaceae	*Berberis*	*Berberis hainesii*	GU934946
Eudicotyledons	Berberidaceae	*Berberis*	*Berberis hainesii*	GU934947
Eudicotyledons	Berberidaceae	*Berberis*	*Berberis hainesii*	GU934948
Eudicotyledons	Berberidaceae	*Berberis*	*Berberis hainesii*	GU934949
Eudicotyledons	Berberidaceae	*Berberis*	*Berberis hainesii*	GU934950
Eudicotyledons	Berberidaceae	*Berberis*	*Berberis jaeschkeana*	GU934953
Eudicotyledons	Berberidaceae	*Berberis*	*Berberis jaeschkeana*	GU934954
Eudicotyledons	Berberidaceae	*Berberis*	*Berberis jaeschkeana*	GU934955
Eudicotyledons	Berberidaceae	*Berberis*	*Berberis jaeschkeana*	GU934956
Eudicotyledons	Berberidaceae	*Berberis*	*Berberis pachyacantha*	GU934973
Eudicotyledons	Berberidaceae	*Berberis*	*Berberis pachyacantha*	GU934974
Eudicotyledons	Berberidaceae	*Berberis*	*Berberis pachyacantha*	GU934975
Eudicotyledons	Berberidaceae	*Berberis*	*Berberis pachyacantha*	GU934976
Eudicotyledons	Berberidaceae	*Berberis*	*Berberis replicata*	GU934977
Eudicotyledons	Berberidaceae	*Berberis*	*Berberis replicata*	GU934978
Eudicotyledons	Berberidaceae	*Berberis*	*Berberis replicata*	GU934979
Eudicotyledons	Berberidaceae	*Berberis*	*Berberis replicata*	GU934980
Eudicotyledons	Berberidaceae	*Berberis*	*Berberis replicata*	GU934981
Eudicotyledons	Berberidaceae	*Berberis*	*Berberis replicata*	GU934982
Eudicotyledons	Berberidaceae	*Berberis*	*Berberis tinctoria*	GU934983
Eudicotyledons	Berberidaceae	*Berberis*	*Berberis tinctoria*	GU934984
Eudicotyledons	Berberidaceae	*Berberis*	*Berberis tinctoria*	GU934985
Eudicotyledons	Berberidaceae	*Berberis*	*Berberis tinctoria*	GU934986

Eudicotyledons	Berberidaceae	*Berberis*	*Berberis tinctoria*	GU934987
Eudicotyledons	Berberidaceae	*Berberis*	*Berberis tinctoria*	GU934988
Eudicotyledons	Berberidaceae	*Berberis*	*Berberis tinctoria*	GU934989
Eudicotyledons	Berberidaceae	*Berberis*	*Berberis tinctoria*	GU934990
Eudicotyledons	Berberidaceae	*Berberis*	*Berberis umbellata*	GU934991
Eudicotyledons	Berberidaceae	*Berberis*	*Berberis umbellata*	GU934992
Eudicotyledons	Berberidaceae	*Berberis*	*Berberis umbellata*	GU934993
Eudicotyledons	Berberidaceae	*Berberis*	*Berberis wightiana*	GU934994
Eudicotyledons	Berberidaceae	*Berberis*	*Berberis wightiana*	GU934995
Eudicotyledons	Berberidaceae	*Berberis*	*Berberis wightiana*	GU934996
Eudicotyledons	Moraceae	*Ficus*	*Ficus retusa*	GU935111
Eudicotyledons	Moraceae	*Ficus*	*Ficus retusa*	GU935112
Eudicotyledons	Fabaceae	*Schotia*	*Schotia aff. latifolia SR-2010*	GQ405103
Eudicotyledons	Fabaceae	*Schotia*	*Schotia aff. latifolia SR-2010*	GQ405104
Eudicotyledons	Fabaceae	*Schotia*	*Schotia aff. latifolia SR-2010*	GQ405122
Eudicotyledons	Fabaceae	*Schotia*	*Schotia aff. latifolia SR-2010*	GQ405133
Eudicotyledons	Fabaceae	*Schotia*	*Schotia aff. brachypetala SR-2010*	GQ405108
Eudicotyledons	Fabaceae	*Schotia*	*Schotia aff. brachypetala SR-2010*	GQ405130
Eudicotyledons	Fabaceae	*Schotia*	*Schotia latifolia x Schotia afra*	GQ405125
Eudicotyledons	Fabaceae	*Schotia*	*Schotia latifolia x Schotia afra*	GQ405134
Eudicotyledons	Fabaceae	*Schotia*	*Schotia latifolia x Schotia afra*	GQ405135
Eudicotyledons	Hydrangeaceae	*Hydrangea*	*Hydrangea heteromalla*	JN044926
Eudicotyledons	Hydrangeaceae	*Hydrangea*	*Hydrangea heteromalla*	JN044927
Eudicotyledons	Hydrangeaceae	*Hydrangea*	*Hydrangea heteromalla*	HM216975
Eudicotyledons	Cucurbitaceae	*Thladiantha*	*Thladiantha lijiangensis*	JN047408
Eudicotyledons	Cucurbitaceae	*Thladiantha*	*Thladiantha lijiangensis*	JN047409
Eudicotyledons	Cucurbitaceae	*Thladiantha*	*Thladiantha lijiangensis*	JN047410
Eudicotyledons	Cucurbitaceae	*Thladiantha*	*Thladiantha lijiangensis*	JN047411
Eudicotyledons	Cucurbitaceae	*Thladiantha*	*Thladiantha lijiangensis*	JN047412
Eudicotyledons	Cucurbitaceae	*Thladiantha*	*Thladiantha lijiangensis*	JN047413
Eudicotyledons	Cucurbitaceae	*Thladiantha*	*Thladiantha lijiangensis*	JN047414
Eudicotyledons	Rosaceae	*Potentilla*	*Potentilla calabra*	HM776569
Eudicotyledons	Rosaceae	*Potentilla*	*Potentilla calabra*	HM776570
Eudicotyledons	Rosaceae	*Potentilla*	*Potentilla calabra*	HM776571
Eudicotyledons	Asteraceae	*Senecio*	*Senecio cadiscus*	GU818346
Eudicotyledons	Asteraceae	*Senecio*	*Senecio cadiscus*	GU818347
Eudicotyledons	Lamiaceae	*Mentha*	*Mentha sp. MIB zpl*	FR726103
Eudicotyledons	Lamiaceae	*Mentha*	*Mentha sp. MIB zpl*	FR726104
Eudicotyledons	Lamiaceae	*Mentha*	*Mentha sp. MIB zpl*	FR726105
Eudicotyledons	Lamiaceae	*Salvia*	*Salvia sp. MIB zpl*	FR726145
Eudicotyledons	Lamiaceae	*Salvia*	*Salvia sp. MIB zpl*	FR726146
Eudicotyledons	Lamiaceae	*Salvia*	*Salvia sp. MIB zpl*	FR726147
Eudicotyledons	Solanaceae	*Lycium*	*Lycium truncatum*	HM195015
Eudicotyledons	Solanaceae	*Lycium*	*Lycium truncatum*	HM195016
Eudicotyledons	Solanaceae	*Lycium*	*Lycium yunnanense*	HM195018
Eudicotyledons	Solanaceae	*Lycium*	*Lycium yunnanense*	HM195019
Eudicotyledons	Hydrangeaceae	*Hydrangea*	*Hydrangea bretschneideri*	HM216969
Eudicotyledons	Hydrangeaceae	*Hydrangea*	*Hydrangea bretschneideri*	JF321261
Eudicotyledons	Lamiaceae	*Teucrium*	*Teucrium montanum*	FR865074
Eudicotyledons	Lamiaceae	*Teucrium*	*Teucrium montanum*	HQ902877
Eudicotyledons	Elaeocarpaceae	*Elaeocarpus*	*Elaeocarpus decipiens*	HQ415428
Eudicotyledons	Elaeocarpaceae	*Elaeocarpus*	*Elaeocarpus decipiens*	HQ426998
Eudicotyledons	Elaeocarpaceae	*Elaeocarpus*	*Elaeocarpus japonicus*	HQ415431

Eudicotyledons	Elaeocarpaceae	*Elaeocarpus*	*Elaeocarpus japonicus*	HQ426999
Eudicotyledons	Moraceae	*Ficus*	*Ficus variolosa*	HQ415501
Eudicotyledons	Moraceae	*Ficus*	*Ficus variolosa*	JN044545
Eudicotyledons	Moraceae	*Ficus*	*Ficus variolosa*	JN044546
Eudicotyledons	Moraceae	*Ficus*	*Ficus variolosa*	JN044547
Eudicotyledons	Loranthaceae	*Macrosolen*	*Macrosolen tricolor*	HQ317812
Eudicotyledons	Loranthaceae	*Macrosolen*	*Macrosolen tricolor*	HQ317813
Eudicotyledons	Loranthaceae	*Taxillus*	*Taxillus limprichtii*	HQ317832
Eudicotyledons	Loranthaceae	*Taxillus*	*Taxillus limprichtii*	HQ317833
Eudicotyledons	Loranthaceae	*Taxillus*	*Taxillus limprichtii*	HQ317834
Eudicotyledons	Loranthaceae	*Taxillus*	*Taxillus nigrans*	HQ317835
Eudicotyledons	Loranthaceae	*Taxillus*	*Taxillus nigrans*	HQ317836
Eudicotyledons	Hydrangeaceae	*Hydrangea*	*Hydrangea davidii*	JN044924
Eudicotyledons	Hydrangeaceae	*Hydrangea*	*Hydrangea davidii*	JN044925
Eudicotyledons	Hydrangeaceae	*Hydrangea*	*Hydrangea davidii*	HM216974
Eudicotyledons	Cucurbitaceae	*Thladiantha*	*Thladiantha capitata*	JN047367
Eudicotyledons	Cucurbitaceae	*Thladiantha*	*Thladiantha capitata*	JN047368
Eudicotyledons	Cucurbitaceae	*Thladiantha*	*Thladiantha capitata*	JN047369
Eudicotyledons	Cucurbitaceae	*Thladiantha*	*Thladiantha capitata*	JN047370
Eudicotyledons	Cucurbitaceae	*Thladiantha*	*Thladiantha capitata*	JN047371
Eudicotyledons	Cucurbitaceae	*Thladiantha*	*Thladiantha dentata*	JN047388
Eudicotyledons	Cucurbitaceae	*Thladiantha*	*Thladiantha dentata*	JN047389
Eudicotyledons	Cucurbitaceae	*Thladiantha*	*Thladiantha dentata*	JN047390
Eudicotyledons	Cucurbitaceae	*Thladiantha*	*Thladiantha dentata*	JN047391
Eudicotyledons	Cucurbitaceae	*Thladiantha*	*Thladiantha grandisepala*	JN047392
Eudicotyledons	Cucurbitaceae	*Thladiantha*	*Thladiantha grandisepala*	JN047393
Eudicotyledons	Cucurbitaceae	*Thladiantha*	*Thladiantha grandisepala*	JN047394
Eudicotyledons	Cucurbitaceae	*Thladiantha*	*Thladiantha grandisepala*	JN047395
Eudicotyledons	Cucurbitaceae	*Thladiantha*	*Thladiantha grandisepala*	JN047396
Eudicotyledons	Cucurbitaceae	*Thladiantha*	*Thladiantha grandisepala*	JN047397
Eudicotyledons	Cucurbitaceae	*Thladiantha*	*Thladiantha grandisepala*	JN047398
Eudicotyledons	Cucurbitaceae	*Thladiantha*	*Thladiantha grandisepala*	JN047399
Eudicotyledons	Cucurbitaceae	*Thladiantha*	*Thladiantha henryi*	JN047400
Eudicotyledons	Cucurbitaceae	*Thladiantha*	*Thladiantha henryi*	JN047401
Eudicotyledons	Cucurbitaceae	*Thladiantha*	*Thladiantha henryi*	JN047402
Eudicotyledons	Cucurbitaceae	*Thladiantha*	*Thladiantha longisepala*	JN047415
Eudicotyledons	Cucurbitaceae	*Thladiantha*	*Thladiantha longisepala*	JN047416
Eudicotyledons	Cucurbitaceae	*Thladiantha*	*Thladiantha longisepala*	JN047417
Eudicotyledons	Cucurbitaceae	*Thladiantha*	*Thladiantha maculata*	JN047418
Eudicotyledons	Cucurbitaceae	*Thladiantha*	*Thladiantha maculata*	JN047419
Eudicotyledons	Cucurbitaceae	*Thladiantha*	*Thladiantha maculata*	JN047420
Eudicotyledons	Cucurbitaceae	*Thladiantha*	*Thladiantha maculata*	JN047421
Eudicotyledons	Cucurbitaceae	*Thladiantha*	*Thladiantha maculata*	JN047422
Eudicotyledons	Cucurbitaceae	*Thladiantha*	*Thladiantha maculata*	JN047423
Eudicotyledons	Cucurbitaceae	*Thladiantha*	*Thladiantha montana*	JN047424
Eudicotyledons	Cucurbitaceae	*Thladiantha*	*Thladiantha montana*	JN047425
Eudicotyledons	Cucurbitaceae	*Thladiantha*	*Thladiantha montana*	JN047426
Eudicotyledons	Cucurbitaceae	*Thladiantha*	*Thladiantha montana*	JN047427
Eudicotyledons	Cucurbitaceae	*Thladiantha*	*Thladiantha montana*	JN047428
Eudicotyledons	Cucurbitaceae	*Thladiantha*	*Thladiantha oliveri*	JN047439
Eudicotyledons	Cucurbitaceae	*Thladiantha*	*Thladiantha oliveri*	JN047440
Eudicotyledons	Cucurbitaceae	*Thladiantha*	*Thladiantha oliveri*	JN047441
Eudicotyledons	Cucurbitaceae	*Thladiantha*	*Thladiantha oliveri*	JN047442

Eudicotyledons	Cucurbitaceae	*Thladiantha*	*Thladiantha oliveri*	JN047443
Eudicotyledons	Cucurbitaceae	*Thladiantha*	*Thladiantha oliveri*	JN047444
Eudicotyledons	Cucurbitaceae	*Thladiantha*	*Thladiantha villosula*	JN047445
Eudicotyledons	Cucurbitaceae	*Thladiantha*	*Thladiantha villosula*	JN047446
Eudicotyledons	Cucurbitaceae	*Thladiantha*	*Thladiantha villosula*	JN047447
Eudicotyledons	Cucurbitaceae	*Thladiantha*	*Thladiantha villosula*	JN047448
Eudicotyledons	Cucurbitaceae	*Thladiantha*	*Thladiantha villosula*	JN047449
Eudicotyledons	Cucurbitaceae	*Thladiantha*	*Thladiantha villosula*	JN047450
Eudicotyledons	Rosaceae	*Potentilla*	*Potentilla parvifolia*	JN044380
Eudicotyledons	Rosaceae	*Potentilla*	*Potentilla parvifolia*	JN044381
Eudicotyledons	Rosaceae	*Potentilla*	*Potentilla parvifolia*	JN044382
Eudicotyledons	Rosaceae	*Potentilla*	*Potentilla parvifolia*	JN044383
Eudicotyledons	Rosaceae	*Potentilla*	*Potentilla parvifolia*	JN044384
Eudicotyledons	Rosaceae	*Potentilla*	*Potentilla parvifolia*	JN044385
Eudicotyledons	Rosaceae	*Potentilla*	*Potentilla parvifolia*	JN044386
Eudicotyledons	Rosaceae	*Potentilla*	*Potentilla parvifolia*	JN044387
Eudicotyledons	Rosaceae	*Potentilla*	*Potentilla parvifolia*	JN044388
Eudicotyledons	Onagraceae	*Chamerion*	*Chamerion conspersum*	JN044478
Eudicotyledons	Onagraceae	*Chamerion*	*Chamerion conspersum*	JN129844
Eudicotyledons	Moraceae	*Ficus*	*Ficus heteromorpha*	JN044522
Eudicotyledons	Moraceae	*Ficus*	*Ficus heteromorpha*	JN044523
Eudicotyledons	Rosaceae	*Rubus*	*Rubus reflexus*	JN407044
Eudicotyledons	Rosaceae	*Rubus*	*Rubus reflexus*	JN407045
Eudicotyledons	Rosaceae	*Rubus*	*Rubus reflexus*	JN407046
Eudicotyledons	Rosaceae	*Rubus*	*Rubus reflexus*	JN407047
Eudicotyledons	Rosaceae	*Rubus*	*Rubus reflexus*	JN407050
Eudicotyledons	Lamiaceae	*Teucrium*	*Teucrium polium*	HQ902847
Eudicotyledons	Lamiaceae	*Teucrium*	*Teucrium polium*	HQ902867
Eudicotyledons	Asteraceae	*Senecio*	*Senecio massaicus*	JN789941
Eudicotyledons	Asteraceae	*Senecio*	*Senecio massaicus*	JN789942
Eudicotyledons	Asteraceae	*Senecio*	*Senecio massaicus*	JN789943
Eudicotyledons	Lamiaceae	*Teucrium*	*Teucrium divaricatum*	HQ902846
Eudicotyledons	Lamiaceae	*Teucrium*	*Teucrium divaricatum*	HQ902863
Eudicotyledons	Amaranthaceae	*Chenopodium*	*Chenopodium album*	HQ596639
Eudicotyledons	Amaranthaceae	*Chenopodium*	*Chenopodium album*	GQ435157
Eudicotyledons	Amaranthaceae	*Chenopodium*	*Chenopodium album*	JN044272
Eudicotyledons	Amaranthaceae	*Chenopodium*	*Chenopodium album*	JN044273
Eudicotyledons	Amaranthaceae	*Chenopodium*	*Chenopodium album*	JN044274
Eudicotyledons	Amaranthaceae	*Chenopodium*	*Chenopodium album*	JN044275
Eudicotyledons	Amaranthaceae	*Chenopodium*	*Chenopodium album*	JN044276
Eudicotyledons	Amaranthaceae	*Chenopodium*	*Chenopodium album*	JN044277
Eudicotyledons	Amaranthaceae	*Chenopodium*	*Chenopodium album*	JN044278
Eudicotyledons	Amaranthaceae	*Chenopodium*	*Chenopodium album*	JN044279
Eudicotyledons	Amaranthaceae	*Chenopodium*	*Chenopodium album*	JN044280
Eudicotyledons	Amaranthaceae	*Chenopodium*	*Chenopodium album*	JN044281
Eudicotyledons	Amaranthaceae	*Chenopodium*	*Chenopodium album*	JN044282
Eudicotyledons	Amaranthaceae	*Chenopodium*	*Chenopodium album*	JN044283
Eudicotyledons	Amaranthaceae	*Chenopodium*	*Chenopodium album*	JN044284
Eudicotyledons	Amaranthaceae	*Chenopodium*	*Chenopodium album*	JN044285
Eudicotyledons	Amaranthaceae	*Chenopodium*	*Chenopodium album*	JN044286
Eudicotyledons	Amaranthaceae	*Chenopodium*	*Chenopodium album*	JN044287
Eudicotyledons	Amaranthaceae	*Chenopodium*	*Chenopodium album*	JN044288
Eudicotyledons	Amaranthaceae	*Chenopodium*	*Chenopodium album*	JN044289

Eudicotyledons	Amaranthaceae	*Chenopodium*	*Chenopodium album*	JN044290
Eudicotyledons	Amaranthaceae	*Chenopodium*	*Chenopodium album*	JN044291
Eudicotyledons	Amaranthaceae	*Chenopodium*	*Chenopodium album*	JN044292
Eudicotyledons	Amaranthaceae	*Amaranthus*	*Amaranthus hybridus*	GQ248242
Eudicotyledons	Amaranthaceae	*Amaranthus*	*Amaranthus hybridus*	DQ006131
Eudicotyledons	Amaranthaceae	*Amaranthus*	*Amaranthus hybridus*	JN043883
Eudicotyledons	Amaranthaceae	*Amaranthus*	*Amaranthus hybridus*	JN043884
Eudicotyledons	Amaranthaceae	*Amaranthus*	*Amaranthus hybridus*	JN043885
Eudicotyledons	Vitaceae	*Vitis*	*Vitis aestivalis*	JQ182515
Eudicotyledons	Vitaceae	*Vitis*	*Vitis aestivalis*	HM585788
Eudicotyledons	Vitaceae	*Vitis*	*Vitis aestivalis*	HQ108268
Eudicotyledons	Vitaceae	*Vitis*	*Vitis aestivalis*	HQ656411
Eudicotyledons	Vitaceae	*Vitis*	*Vitis aestivalis*	HQ656425
Eudicotyledons	Vitaceae	*Vitis*	*Vitis aestivalis*	HQ656426
Eudicotyledons	Vitaceae	*Vitis*	*Vitis aestivalis*	HQ656427
Eudicotyledons	Vitaceae	*Parthenocissus*	*Parthenocissus quinquefolia*	JQ182514
Eudicotyledons	Vitaceae	*Parthenocissus*	*Parthenocissus quinquefolia*	HM585680
Eudicotyledons	Vitaceae	*Parthenocissus*	*Parthenocissus quinquefolia*	HQ108338
Eudicotyledons	Vitaceae	*Parthenocissus*	*Parthenocissus quinquefolia*	HQ656485
Eudicotyledons	Vitaceae	*Parthenocissus*	*Parthenocissus quinquefolia*	JF437133
Eudicotyledons	Rhamnaceae	*Rhamnus*	*Rhamnus cathartica*	HQ596812
Eudicotyledons	Rhamnaceae	*Rhamnus*	*Rhamnus cathartica*	EU750518
Eudicotyledons	Rhamnaceae	*Rhamnus*	*Rhamnus cathartica*	EU750519
Eudicotyledons	Rhamnaceae	*Rhamnus*	*Rhamnus cathartica*	EU750520
Eudicotyledons	Rhamnaceae	*Rhamnus*	*Rhamnus cathartica*	EU750521
Eudicotyledons	Malvaceae	*Gossypium*	*Gossypium barbadense*	HM437878
Eudicotyledons	Malvaceae	*Gossypium*	*Gossypium barbadense*	HM437879
Eudicotyledons	Malvaceae	*Gossypium*	*Gossypium barbadense*	HM437880
Eudicotyledons	Malvaceae	*Gossypium*	*Gossypium barbadense*	HM437881
Eudicotyledons	Malvaceae	*Gossypium*	*Gossypium barbadense*	HM437882
Eudicotyledons	Malvaceae	*Gossypium*	*Gossypium barbadense*	HM437883
Eudicotyledons	Malvaceae	*Gossypium*	*Gossypium barbadense*	HM437884
Eudicotyledons	Malvaceae	*Gossypium*	*Gossypium barbadense*	HM437885
Eudicotyledons	Malvaceae	*Gossypium*	*Gossypium hirsutum*	HM437901
Eudicotyledons	Malvaceae	*Gossypium*	*Gossypium hirsutum*	HM437902
Eudicotyledons	Malvaceae	*Gossypium*	*Gossypium hirsutum*	HM437903
Eudicotyledons	Malvaceae	*Gossypium*	*Gossypium hirsutum*	HM437904
Eudicotyledons	Malvaceae	*Gossypium*	*Gossypium hirsutum*	HM437905
Eudicotyledons	Malvaceae	*Gossypium*	*Gossypium hirsutum*	HM437906
Eudicotyledons	Malvaceae	*Gossypium*	*Gossypium hirsutum*	HM437907
Eudicotyledons	Cucurbitaceae	*Bryonia*	*Bryonia dioica*	FJ009144
Eudicotyledons	Cucurbitaceae	*Bryonia*	*Bryonia dioica*	FJ009145
Eudicotyledons	Cucurbitaceae	*Bryonia*	*Bryonia dioica*	FJ009146
Eudicotyledons	Cucurbitaceae	*Bryonia*	*Bryonia dioica*	FJ009147
Eudicotyledons	Cucurbitaceae	*Bryonia*	*Bryonia dioica*	FJ009148
Eudicotyledons	Cucurbitaceae	*Bryonia*	*Bryonia dioica*	FJ009149
Eudicotyledons	Cucurbitaceae	*Bryonia*	*Bryonia dioica*	FJ009150
Eudicotyledons	Cucurbitaceae	*Bryonia*	*Bryonia dioica*	FJ009151
Eudicotyledons	Cucurbitaceae	*Bryonia*	*Bryonia dioica*	FJ009152
Eudicotyledons	Cucurbitaceae	*Bryonia*	*Bryonia dioica*	FJ009153
Eudicotyledons	Cucurbitaceae	*Bryonia*	*Bryonia dioica*	FJ009154
Eudicotyledons	Cucurbitaceae	*Bryonia*	*Bryonia dioica*	FJ009155
Eudicotyledons	Cucurbitaceae	*Bryonia*	*Bryonia dioica*	FJ009156

Eudicotyledons	Cucurbitaceae	*Bryonia*	*Bryonia dioica*	FJ009157
Eudicotyledons	Cucurbitaceae	*Bryonia*	*Bryonia dioica*	EU531705
Eudicotyledons	Cucurbitaceae	*Bryonia*	*Bryonia dioica*	EU683739
Eudicotyledons	Cucurbitaceae	*Bryonia*	*Bryonia dioica*	EU096357
Eudicotyledons	Cucurbitaceae	*Bryonia*	*Bryonia dioica*	EU096358
Eudicotyledons	Cucurbitaceae	*Bryonia*	*Bryonia dioica*	EU096359
Eudicotyledons	Cucurbitaceae	*Bryonia*	*Bryonia dioica*	EU096360
Eudicotyledons	Cucurbitaceae	*Bryonia*	*Bryonia dioica*	EU096361
Eudicotyledons	Cucurbitaceae	*Bryonia*	*Bryonia dioica*	EU096362
Eudicotyledons	Cucurbitaceae	*Bryonia*	*Bryonia dioica*	EU096363
Eudicotyledons	Cucurbitaceae	*Bryonia*	*Bryonia dioica*	EU096364
Eudicotyledons	Cucurbitaceae	*Bryonia*	*Bryonia dioica*	EU096365
Eudicotyledons	Cucurbitaceae	*Bryonia*	*Bryonia dioica*	EU096366
Eudicotyledons	Cucurbitaceae	*Bryonia*	*Bryonia dioica*	EU096367
Eudicotyledons	Cucurbitaceae	*Bryonia*	*Bryonia dioica*	EU096368
Eudicotyledons	Cucurbitaceae	*Bryonia*	*Bryonia dioica*	EU096369
Eudicotyledons	Cucurbitaceae	*Bryonia*	*Bryonia dioica*	EU096370
Eudicotyledons	Cucurbitaceae	*Bryonia*	*Bryonia dioica*	EU096371
Eudicotyledons	Cucurbitaceae	*Bryonia*	*Bryonia dioica*	EU096372
Eudicotyledons	Cucurbitaceae	*Bryonia*	*Bryonia dioica*	EU096373
Eudicotyledons	Cucurbitaceae	*Bryonia*	*Bryonia dioica*	EU096374
Eudicotyledons	Cucurbitaceae	*Bryonia*	*Bryonia dioica*	EU096375
Eudicotyledons	Cucurbitaceae	*Bryonia*	*Bryonia dioica*	EU096376
Eudicotyledons	Cucurbitaceae	*Bryonia*	*Bryonia dioica*	EU096377
Eudicotyledons	Cucurbitaceae	*Bryonia*	*Bryonia dioica*	EU096378
Eudicotyledons	Cucurbitaceae	*Bryonia*	*Bryonia dioica*	EU096379
Eudicotyledons	Cucurbitaceae	*Bryonia*	*Bryonia dioica*	EU096380
Eudicotyledons	Cucurbitaceae	*Bryonia*	*Bryonia dioica*	EU096381
Eudicotyledons	Cucurbitaceae	*Bryonia*	*Bryonia dioica*	EU096382
Eudicotyledons	Cucurbitaceae	*Bryonia*	*Bryonia dioica*	EU096383
Eudicotyledons	Cucurbitaceae	*Bryonia*	*Bryonia dioica*	EU096384
Eudicotyledons	Cucurbitaceae	*Bryonia*	*Bryonia dioica*	EU096385
Eudicotyledons	Cucurbitaceae	*Bryonia*	*Bryonia dioica*	EU096386
Eudicotyledons	Cucurbitaceae	*Bryonia*	*Bryonia dioica*	EU096387
Eudicotyledons	Cucurbitaceae	*Bryonia*	*Bryonia dioica*	EU096388
Eudicotyledons	Cucurbitaceae	*Bryonia*	*Bryonia dioica*	EU096389
Eudicotyledons	Cucurbitaceae	*Bryonia*	*Bryonia dioica*	EU096390
Eudicotyledons	Cucurbitaceae	*Bryonia*	*Bryonia dioica*	EU096391
Eudicotyledons	Cucurbitaceae	*Bryonia*	*Bryonia dioica*	EU096392
Eudicotyledons	Cucurbitaceae	*Bryonia*	*Bryonia dioica*	EU096393
Eudicotyledons	Cucurbitaceae	*Bryonia*	*Bryonia dioica*	EU096394
Eudicotyledons	Cucurbitaceae	*Bryonia*	*Bryonia dioica*	EU096395
Eudicotyledons	Cucurbitaceae	*Bryonia*	*Bryonia dioica*	EU096396
Eudicotyledons	Cucurbitaceae	*Bryonia*	*Bryonia dioica*	EU096397
Eudicotyledons	Cucurbitaceae	*Bryonia*	*Bryonia dioica*	EU096398
Eudicotyledons	Cucurbitaceae	*Bryonia*	*Bryonia dioica*	EU096399
Eudicotyledons	Cucurbitaceae	*Bryonia*	*Bryonia dioica*	EU096402
Eudicotyledons	Cucurbitaceae	*Bryonia*	*Bryonia dioica*	EU096403
Eudicotyledons	Cucurbitaceae	*Bryonia*	*Bryonia dioica*	EU096404
Eudicotyledons	Cucurbitaceae	*Momordica*	*Momordica balsamina*	GQ162974
Eudicotyledons	Cucurbitaceae	*Momordica*	*Momordica balsamina*	GQ162975
Eudicotyledons	Cucurbitaceae	*Momordica*	*Momordica balsamina*	GQ162976
Eudicotyledons	Cucurbitaceae	*Momordica*	*Momordica balsamina*	GQ162977

Eudicotyledons	Cucurbitaceae	*Momordica*	*Momordica charantia*	GQ162992
Eudicotyledons	Cucurbitaceae	*Momordica*	*Momordica charantia*	GQ162993
Eudicotyledons	Cucurbitaceae	*Momordica*	*Momordica charantia*	GQ162994
Eudicotyledons	Cucurbitaceae	*Momordica*	*Momordica charantia*	GQ162995
Eudicotyledons	Cucurbitaceae	*Momordica*	*Momordica charantia*	GQ162996
Eudicotyledons	Cucurbitaceae	*Momordica*	*Momordica charantia*	GQ162997
Eudicotyledons	Cucurbitaceae	*Momordica*	*Momordica charantia*	GQ162998
Eudicotyledons	Cucurbitaceae	*Momordica*	*Momordica charantia*	JN406960
Eudicotyledons	Cucurbitaceae	*Momordica*	*Momordica charantia*	JN406961
Eudicotyledons	Cucurbitaceae	*Momordica*	*Momordica charantia*	JN406962
Eudicotyledons	Cucurbitaceae	*Momordica*	*Momordica charantia*	GQ845138
Eudicotyledons	Cucurbitaceae	*Momordica*	*Momordica cochinchinensis*	GQ163004
Eudicotyledons	Cucurbitaceae	*Momordica*	*Momordica cochinchinensis*	GQ163005
Eudicotyledons	Cucurbitaceae	*Momordica*	*Momordica cochinchinensis*	GQ163006
Eudicotyledons	Cucurbitaceae	*Momordica*	*Momordica cochinchinensis*	GQ163007
Eudicotyledons	Cucurbitaceae	*Momordica*	*Momordica cochinchinensis*	GQ435030
Eudicotyledons	Cucurbitaceae	*Momordica*	*Momordica cochinchinensis*	GQ435031
Eudicotyledons	Cucurbitaceae	*Momordica*	*Momordica cochinchinensis*	GQ845134
Eudicotyledons	Cucurbitaceae	*Momordica*	*Momordica cochinchinensis*	GQ845135
Eudicotyledons	Cucurbitaceae	*Momordica*	*Momordica cochinchinensis*	GQ845136
Eudicotyledons	Cucurbitaceae	*Momordica*	*Momordica repens*	GQ163068
Eudicotyledons	Cucurbitaceae	*Momordica*	*Momordica repens*	GQ163069
Eudicotyledons	Cucurbitaceae	*Trichosanthes*	*Trichosanthes kirilowii*	GQ435028
Eudicotyledons	Cucurbitaceae	*Trichosanthes*	*Trichosanthes kirilowii*	GQ845127
Eudicotyledons	Cucurbitaceae	*Trichosanthes*	*Trichosanthes kirilowii*	GQ845132
Eudicotyledons	Cucurbitaceae	*Trichosanthes*	*Trichosanthes kirilowii*	GQ845133
Eudicotyledons	Passifloraceae	*Passiflora*	*Passiflora quadrangularis*	AY032809
Eudicotyledons	Passifloraceae	*Passiflora*	*Passiflora quadrangularis*	GQ248362
Eudicotyledons	Passifloraceae	*Passiflora*	*Passiflora quadrangularis*	EF590723
Eudicotyledons	Salicaceae	*Populus*	*Populus tremuloides*	EU750503
Eudicotyledons	Salicaceae	*Populus*	*Populus tremuloides*	EU750504
Eudicotyledons	Salicaceae	*Populus*	*Populus tremuloides*	EU750505
Eudicotyledons	Salicaceae	*Populus*	*Populus deltoides*	GU562412
Eudicotyledons	Salicaceae	*Populus*	*Populus deltoides*	HQ596797
Eudicotyledons	Brassicaceae	*Brassica*	*Brassica juncea*	GQ435335
Eudicotyledons	Brassicaceae	*Brassica*	*Brassica juncea*	GQ435336
Eudicotyledons	Brassicaceae	*Brassica*	*Brassica juncea*	AB669926
Eudicotyledons	Brassicaceae	*Capsella*	*Capsella bursa-pastoris*	FJ395502
Eudicotyledons	Brassicaceae	*Capsella*	*Capsella bursa-pastoris*	HQ596620
Eudicotyledons	Brassicaceae	*Capsella*	*Capsella bursa-pastoris*	FR822344
Eudicotyledons	Brassicaceae	*Capsella*	*Capsella bursa-pastoris*	FR822345
Eudicotyledons	Brassicaceae	*Capsella*	*Capsella bursa-pastoris*	FR822346
Eudicotyledons	Brassicaceae	*Capsella*	*Capsella bursa-pastoris*	FJ493269
Eudicotyledons	Brassicaceae	*Raphanus*	*Raphanus sativus*	GQ184362
Eudicotyledons	Brassicaceae	*Raphanus*	*Raphanus sativus*	GQ184363
Eudicotyledons	Brassicaceae	*Raphanus*	*Raphanus sativus*	GQ248384
Eudicotyledons	Brassicaceae	*Raphanus*	*Raphanus sativus*	GQ435337
Eudicotyledons	Brassicaceae	*Raphanus*	*Raphanus sativus*	AB669929
Eudicotyledons	Brassicaceae	*Raphanus*	*Raphanus sativus*	AB669930
Eudicotyledons	Brassicaceae	*Raphanus*	*Raphanus sativus*	EF590737
Eudicotyledons	Sapotaceae	*Manilkara*	*Manilkara zapota*	GU135342
Eudicotyledons	Sapotaceae	*Manilkara*	*Manilkara zapota*	AM179738
Eudicotyledons	Rosaceae	*Prunus*	*Prunus domestica*	AY500614

Eudicotyledons	Rosaceae	*Prunus*	*Prunus domestica*	FN675833
Eudicotyledons	Rosaceae	*Prunus*	*Prunus x yedoensis*	GQ248382
Eudicotyledons	Rosaceae	*Prunus*	*Prunus x yedoensis*	EF590735
Eudicotyledons	Rosaceae	*Prunus*	*Prunus persica*	GQ435268
Eudicotyledons	Rosaceae	*Prunus*	*Prunus persica*	GQ435269
Eudicotyledons	Rosaceae	*Prunus*	*Prunus persica*	AY500628
Eudicotyledons	Rosaceae	*Prunus*	*Prunus persica*	FN675835
Eudicotyledons	Rosaceae	*Prunus*	*Prunus persica*	JN046652
Eudicotyledons	Rosaceae	*Prunus*	*Prunus persica*	JN046653
Eudicotyledons	Rosaceae	*Prunus*	*Prunus persica*	JN046654
Eudicotyledons	Rosaceae	*Prunus*	*Prunus persica*	JN046655
Eudicotyledons	Rosaceae	*Prunus*	*Prunus persica*	JN046656
Eudicotyledons	Rosaceae	*Prunus*	*Prunus persica*	JN046657
Eudicotyledons	Rosaceae	*Prunus*	*Prunus persica*	JN046658
Eudicotyledons	Rosaceae	*Prunus*	*Prunus persica*	JN046660
Eudicotyledons	Grossulariaceae	*Ribes*	*Ribes americanum*	HQ596814
Eudicotyledons	Grossulariaceae	*Ribes*	*Ribes americanum*	HQ596815
Eudicotyledons	Fabaceae	*Acacia*	*Acacia confusa*	GU396784
Eudicotyledons	Fabaceae	*Acacia*	*Acacia confusa*	GU396785
Eudicotyledons	Fabaceae	*Acacia*	*Acacia confusa*	GQ435006
Eudicotyledons	Fabaceae	*Albizia*	*Albizia julibrissin*	GU396765
Eudicotyledons	Fabaceae	*Albizia*	*Albizia julibrissin*	GU135433
Eudicotyledons	Fabaceae	*Albizia*	*Albizia julibrissin*	EU811952
Eudicotyledons	Fabaceae	*Albizia*	*Albizia julibrissin*	JF733773
Eudicotyledons	Fabaceae	*Canavalia*	*Canavalia rosea*	HQ707421
Eudicotyledons	Fabaceae	*Canavalia*	*Canavalia rosea*	HQ707422
Eudicotyledons	Fabaceae	*Canavalia*	*Canavalia rosea*	HQ707423
Eudicotyledons	Fabaceae	*Canavalia*	*Canavalia rosea*	HQ707424
Eudicotyledons	Fabaceae	*Canavalia*	*Canavalia rosea*	HQ707425
Eudicotyledons	Fabaceae	*Canavalia*	*Canavalia rosea*	HQ707426
Eudicotyledons	Fabaceae	*Canavalia*	*Canavalia rosea*	HQ707427
Eudicotyledons	Fabaceae	*Canavalia*	*Canavalia rosea*	HQ707428
Eudicotyledons	Fabaceae	*Leucaena*	*Leucaena leucocephala*	GU396787
Eudicotyledons	Fabaceae	*Leucaena*	*Leucaena leucocephala*	AF524962
Eudicotyledons	Fabaceae	*Leucaena*	*Leucaena leucocephala*	GU135371
Eudicotyledons	Fabaceae	*Leucaena*	*Leucaena leucocephala*	EU811958
Eudicotyledons	Fabaceae	*Trifolium*	*Trifolium repens*	FJ395537
Eudicotyledons	Fabaceae	*Trifolium*	*Trifolium repens*	EU750600
Eudicotyledons	Fabaceae	*Trifolium*	*Trifolium repens*	EU750601
Eudicotyledons	Fabaceae	*Trifolium*	*Trifolium repens*	EU750602
Eudicotyledons	Fabaceae	*Wisteria*	*Wisteria floribunda*	EU424096
Eudicotyledons	Fabaceae	*Wisteria*	*Wisteria floribunda*	EU424097
Eudicotyledons	Fabaceae	*Wisteria*	*Wisteria floribunda*	EU424098
Eudicotyledons	Linaceae	*Linum*	*Linum usitatissimum*	GQ845295
Eudicotyledons	Linaceae	*Linum*	*Linum usitatissimum*	GQ845296
Eudicotyledons	Linaceae	*Linum*	*Linum usitatissimum*	GQ845297
Eudicotyledons	Linaceae	*Linum*	*Linum usitatissimum*	GQ845298
Eudicotyledons	Linaceae	*Linum*	*Linum usitatissimum*	GQ845299
Eudicotyledons	Linaceae	*Linum*	*Linum usitatissimum*	GQ845300
Eudicotyledons	Linaceae	*Linum*	*Linum usitatissimum*	GQ845301
Eudicotyledons	Linaceae	*Linum*	*Linum usitatissimum*	GQ845302
Eudicotyledons	Linaceae	*Linum*	*Linum usitatissimum*	GQ845303
Eudicotyledons	Linaceae	*Linum*	*Linum usitatissimum*	GQ845304

Eudicotyledons	Burseraceae	*Bursera*	*Bursera inaguensis*	GQ377880
Eudicotyledons	Burseraceae	*Bursera*	*Bursera inaguensis*	GQ377881
Eudicotyledons	Burseraceae	*Bursera*	*Bursera inaguensis*	AY309393
Eudicotyledons	Aceraceae	*Acer*	*Acer negundo*	DQ978611
Eudicotyledons	Aceraceae	*Acer*	*Acer negundo*	HQ596578
Eudicotyledons	Aceraceae	*Acer*	*Acer negundo*	DQ006227
Eudicotyledons	Aceraceae	*Acer*	*Acer negundo*	EU750427
Eudicotyledons	Aceraceae	*Acer*	*Acer negundo*	EU750428
Eudicotyledons	Aceraceae	*Acer*	*Acer saccharum*	DQ887747
Eudicotyledons	Aceraceae	*Acer*	*Acer saccharum*	DQ887748
Eudicotyledons	Aceraceae	*Acer*	*Acer saccharum*	EU750438
Eudicotyledons	Aceraceae	*Acer*	*Acer saccharum*	EU750439
Eudicotyledons	Aceraceae	*Acer*	*Acer saccharum*	EU750440
Eudicotyledons	Aceraceae	*Acer*	*Acer platanoides*	DQ978619
Eudicotyledons	Aceraceae	*Acer*	*Acer platanoides*	EU750429
Eudicotyledons	Aceraceae	*Acer*	*Acer platanoides*	EU750430
Eudicotyledons	Aceraceae	*Acer*	*Acer pseudoplatanus*	DQ978620
Eudicotyledons	Aceraceae	*Acer*	*Acer pseudoplatanus*	DQ978621
Eudicotyledons	Aceraceae	*Acer*	*Acer pseudoplatanus*	FJ395512
Eudicotyledons	Araliaceae	*Hedera*	*Hedera helix*	FJ395451
Eudicotyledons	Araliaceae	*Hedera*	*Hedera helix*	FN675793
Eudicotyledons	Araliaceae	*Panax*	*Panax ginseng*	GQ435398
Eudicotyledons	Araliaceae	*Panax*	*Panax ginseng*	HQ112863
Eudicotyledons	Araliaceae	*Panax*	*Panax ginseng*	HQ112864
Eudicotyledons	Solanaceae	*Capsicum*	*Capsicum annuum*	JQ087870
Eudicotyledons	Solanaceae	*Capsicum*	*Capsicum annuum*	GU575282
Eudicotyledons	Solanaceae	*Capsicum*	*Capsicum frutescens*	JQ087871
Eudicotyledons	Solanaceae	*Capsicum*	*Capsicum frutescens*	EF537185
Eudicotyledons	Solanaceae	*Capsicum*	*Capsicum frutescens*	EF537186
Eudicotyledons	Solanaceae	*Capsicum*	*Capsicum frutescens*	EF537187
Eudicotyledons	Solanaceae	*Datura*	*Datura inoxia*	GQ274314
Eudicotyledons	Solanaceae	*Datura*	*Datura inoxia*	GQ435286
Eudicotyledons	Solanaceae	*Datura*	*Datura inoxia*	JN244375
Eudicotyledons	Solanaceae	*Datura*	*Datura inoxia*	JN244376
Eudicotyledons	Solanaceae	*Datura*	*Datura inoxia*	JN244377
Eudicotyledons	Solanaceae	*Datura*	*Datura stramonium*	GQ248285
Eudicotyledons	Solanaceae	*Datura*	*Datura stramonium*	JN244368
Eudicotyledons	Solanaceae	*Datura*	*Datura stramonium*	JN244369
Eudicotyledons	Solanaceae	*Datura*	*Datura stramonium*	JN244370
Eudicotyledons	Solanaceae	*Datura*	*Datura stramonium*	JN244371
Eudicotyledons	Solanaceae	*Datura*	*Datura stramonium*	JN244372
Eudicotyledons	Solanaceae	*Datura*	*Datura stramonium*	JN244373
Eudicotyledons	Solanaceae	*Datura*	*Datura stramonium*	JN244374
Eudicotyledons	Solanaceae	*Datura*	*Datura stramonium*	EF590686
Eudicotyledons	Solanaceae	*Datura*	*Datura stramonium*	DQ006229
Eudicotyledons	Solanaceae	*Hyoscyamus*	*Hyoscyamus niger*	HQ216161
Eudicotyledons	Solanaceae	*Hyoscyamus*	*Hyoscyamus niger*	HQ216162
Eudicotyledons	Solanaceae	*Solanum*	*Solanum lycopersicum*	GU562406
Eudicotyledons	Solanaceae	*Solanum*	*Solanum lycopersicum*	GU575280
Eudicotyledons	Solanaceae	*Solanum*	*Solanum lycopersicum*	FN675828
Eudicotyledons	Solanaceae	*Solanum*	*Solanum lycopersicum*	HQ856112
Eudicotyledons	Solanaceae	*Petunia*	*Petunia integrifolia*	AY772904
Eudicotyledons	Solanaceae	*Petunia*	*Petunia integrifolia*	AY772905

Eudicotyledons	Solanaceae	*Petunia*	*Petunia integrifolia*	AY772906
Eudicotyledons	Solanaceae	*Petunia*	*Petunia integrifolia*	AY772907
Eudicotyledons	Solanaceae	*Petunia*	*Petunia integrifolia*	AY772908
Eudicotyledons	Solanaceae	*Petunia*	*Petunia integrifolia*	AY772909
Eudicotyledons	Solanaceae	*Petunia*	*Petunia integrifolia*	AY772910
Eudicotyledons	Solanaceae	*Petunia*	*Petunia integrifolia*	DQ208094
Eudicotyledons	Solanaceae	*Petunia*	*Petunia integrifolia*	DQ208095
Eudicotyledons	Solanaceae	*Petunia*	*Petunia integrifolia*	DQ208096
Eudicotyledons	Solanaceae	*Petunia*	*Petunia integrifolia*	DQ208097
Eudicotyledons	Solanaceae	*Petunia*	*Petunia integrifolia*	DQ208098
Eudicotyledons	Solanaceae	*Petunia*	*Petunia integrifolia*	DQ208099
Eudicotyledons	Solanaceae	*Petunia*	*Petunia integrifolia*	DQ208100
Eudicotyledons	Solanaceae	*Petunia*	*Petunia integrifolia*	DQ208101
Eudicotyledons	Solanaceae	*Petunia*	*Petunia integrifolia*	DQ208102
Eudicotyledons	Solanaceae	*Petunia*	*Petunia integrifolia*	DQ208103
Eudicotyledons	Solanaceae	*Petunia*	*Petunia integrifolia*	DQ208104
Eudicotyledons	Solanaceae	*Petunia*	*Petunia integrifolia*	DQ208105
Eudicotyledons	Solanaceae	*Petunia*	*Petunia integrifolia*	DQ208106
Eudicotyledons	Solanaceae	*Petunia*	*Petunia integrifolia*	DQ208107
Eudicotyledons	Solanaceae	*Petunia*	*Petunia integrifolia*	DQ208108
Eudicotyledons	Solanaceae	*Petunia*	*Petunia integrifolia*	DQ208109
Eudicotyledons	Solanaceae	*Petunia*	*Petunia integrifolia*	DQ208110
Eudicotyledons	Solanaceae	*Petunia*	*Petunia integrifolia*	DQ208111
Eudicotyledons	Solanaceae	*Petunia*	*Petunia integrifolia*	DQ208112
Eudicotyledons	Solanaceae	*Petunia*	*Petunia integrifolia*	DQ208113
Eudicotyledons	Solanaceae	*Petunia*	*Petunia integrifolia*	DQ208114
Eudicotyledons	Solanaceae	*Petunia*	*Petunia integrifolia*	DQ208115
Eudicotyledons	Solanaceae	*Petunia*	*Petunia integrifolia*	DQ208116
Eudicotyledons	Solanaceae	*Petunia*	*Petunia integrifolia*	DQ208117
Eudicotyledons	Solanaceae	*Petunia*	*Petunia integrifolia*	DQ208118
Eudicotyledons	Solanaceae	*Petunia*	*Petunia integrifolia*	DQ208119
Eudicotyledons	Solanaceae	*Petunia*	*Petunia integrifolia*	DQ208120
Eudicotyledons	Solanaceae	*Petunia*	*Petunia integrifolia*	DQ208121
Eudicotyledons	Solanaceae	*Petunia*	*Petunia integrifolia*	DQ208122
Eudicotyledons	Solanaceae	*Petunia*	*Petunia integrifolia*	DQ208123
Eudicotyledons	Solanaceae	*Petunia*	*Petunia integrifolia*	DQ208124
Eudicotyledons	Solanaceae	*Petunia*	*Petunia integrifolia*	DQ208125
Eudicotyledons	Solanaceae	*Petunia*	*Petunia integrifolia*	DQ208126
Eudicotyledons	Solanaceae	*Petunia*	*Petunia integrifolia*	DQ208127
Eudicotyledons	Solanaceae	*Petunia*	*Petunia integrifolia*	DQ208128
Eudicotyledons	Solanaceae	*Petunia*	*Petunia integrifolia*	DQ208129
Eudicotyledons	Solanaceae	*Petunia*	*Petunia integrifolia*	DQ208130
Eudicotyledons	Solanaceae	*Petunia*	*Petunia integrifolia*	DQ208131
Eudicotyledons	Solanaceae	*Petunia*	*Petunia integrifolia*	DQ208132
Eudicotyledons	Solanaceae	*Petunia*	*Petunia integrifolia*	DQ208133
Eudicotyledons	Solanaceae	*Petunia*	*Petunia integrifolia*	DQ208134
Eudicotyledons	Solanaceae	*Petunia*	*Petunia integrifolia*	DQ208135
Eudicotyledons	Solanaceae	*Petunia*	*Petunia integrifolia*	DQ208136
Eudicotyledons	Solanaceae	*Petunia*	*Petunia integrifolia*	DQ208137
Eudicotyledons	Solanaceae	*Petunia*	*Petunia integrifolia*	DQ208138
Eudicotyledons	Solanaceae	*Petunia*	*Petunia integrifolia*	DQ208139
Eudicotyledons	Solanaceae	*Petunia*	*Petunia integrifolia*	DQ208140
Eudicotyledons	Solanaceae	*Petunia*	*Petunia integrifolia*	DQ208141

Eudicotyledons	Solanaceae	*Petunia*	*Petunia integrifolia*	DQ208142
Eudicotyledons	Solanaceae	*Petunia*	*Petunia integrifolia*	DQ208143
Eudicotyledons	Solanaceae	*Petunia*	*Petunia integrifolia*	DQ208144
Eudicotyledons	Solanaceae	*Petunia*	*Petunia integrifolia*	DQ208145
Eudicotyledons	Solanaceae	*Petunia*	*Petunia integrifolia*	DQ208146
Eudicotyledons	Solanaceae	*Petunia*	*Petunia integrifolia*	DQ208147
Eudicotyledons	Solanaceae	*Petunia*	*Petunia integrifolia*	DQ208148
Eudicotyledons	Solanaceae	*Petunia*	*Petunia integrifolia*	DQ208149
Eudicotyledons	Solanaceae	*Petunia*	*Petunia integrifolia*	DQ208150
Eudicotyledons	Solanaceae	*Petunia*	*Petunia integrifolia*	DQ208151
Eudicotyledons	Solanaceae	*Petunia*	*Petunia integrifolia*	DQ208152
Eudicotyledons	Solanaceae	*Petunia*	*Petunia integrifolia*	DQ208153
Eudicotyledons	Solanaceae	*Petunia*	*Petunia integrifolia*	DQ208154
Eudicotyledons	Solanaceae	*Petunia*	*Petunia integrifolia*	DQ208162
Eudicotyledons	Solanaceae	*Solanum*	*Solanum melongena*	HM016405
Eudicotyledons	Solanaceae	*Solanum*	*Solanum melongena*	HM016406
Eudicotyledons	Solanaceae	*Solanum*	*Solanum melongena*	HM016407
Eudicotyledons	Solanaceae	*Solanum*	*Solanum melongena*	HM016408
Eudicotyledons	Solanaceae	*Solanum*	*Solanum melongena*	HM016409
Eudicotyledons	Solanaceae	*Solanum*	*Solanum melongena*	HM016410
Eudicotyledons	Solanaceae	*Solanum*	*Solanum nigrum*	EU750554
Eudicotyledons	Solanaceae	*Solanum*	*Solanum nigrum*	EU750555
Eudicotyledons	Solanaceae	*Solanum*	*Solanum nigrum*	EU750556
Eudicotyledons	Solanaceae	*Solanum*	*Solanum nigrum*	FN675826
Eudicotyledons	Solanaceae	*Solanum*	*Solanum nigrum*	JN047220
Eudicotyledons	Solanaceae	*Solanum*	*Solanum nigrum*	JN047221
Eudicotyledons	Solanaceae	*Solanum*	*Solanum nigrum*	JN047222
Eudicotyledons	Solanaceae	*Solanum*	*Solanum nigrum*	JN047223
Eudicotyledons	Solanaceae	*Solanum*	*Solanum nigrum*	JN047224
Eudicotyledons	Solanaceae	*Solanum*	*Solanum nigrum*	JN047225
Eudicotyledons	Convolvulaceae	*Convolvulus*	*Convolvulus arvensis*	FJ395486
Eudicotyledons	Convolvulaceae	*Convolvulus*	*Convolvulus arvensis*	HQ596651
Eudicotyledons	Lamiaceae	*Leonurus*	*Leonurus japonicus*	JQ339258
Eudicotyledons	Lamiaceae	*Leonurus*	*Leonurus japonicus*	JQ339259
Eudicotyledons	Lamiaceae	*Leonurus*	*Leonurus japonicus*	EU590862
Eudicotyledons	Lamiaceae	*Leonurus*	*Leonurus japonicus*	FJ513112
Eudicotyledons	Plantaginaceae	*Antirrhinum*	*Antirrhinum majus*	HM152920
Eudicotyledons	Plantaginaceae	*Antirrhinum*	*Antirrhinum majus*	HM152921
Eudicotyledons	Plantaginaceae	*Antirrhinum*	*Antirrhinum majus*	HM152922
Eudicotyledons	Plantaginaceae	*Antirrhinum*	*Antirrhinum majus*	HM152923
Eudicotyledons	Plantaginaceae	*Antirrhinum*	*Antirrhinum majus*	HM152924
Eudicotyledons	Plantaginaceae	*Antirrhinum*	*Antirrhinum majus*	HM152925
Eudicotyledons	Adoxaceae	*Sambucus*	*Sambucus nigra*	FJ395531
Eudicotyledons	Adoxaceae	*Sambucus*	*Sambucus nigra*	FJ395532
Eudicotyledons	Adoxaceae	*Sambucus*	*Sambucus nigra*	FN675824
Eudicotyledons	Adoxaceae	*Sambucus*	*Sambucus racemosa*	HQ596834
Eudicotyledons	Adoxaceae	*Sambucus*	*Sambucus racemosa*	FN675823
Eudicotyledons	Adoxaceae	*Viburnum*	*Viburnum acerifolium*	AY627384
Eudicotyledons	Adoxaceae	*Viburnum*	*Viburnum acerifolium*	DQ006166
Eudicotyledons	Adoxaceae	*Viburnum*	*Viburnum acerifolium*	EU750607
Eudicotyledons	Adoxaceae	*Viburnum*	*Viburnum acerifolium*	EU750608
Eudicotyledons	Asteraceae	*Ambrosia*	*Ambrosia artemisiifolia*	HQ596592
Eudicotyledons	Asteraceae	*Ambrosia*	*Ambrosia artemisiifolia*	GQ248244

Eudicotyledons	Asteraceae	*Ambrosia*	*Ambrosia artemisiifolia*	EF590669
Eudicotyledons	Asteraceae	*Ambrosia*	*Ambrosia artemisiifolia*	DQ006141
Eudicotyledons	Asteraceae	*Ambrosia*	*Ambrosia trifida*	GQ248245
Eudicotyledons	Asteraceae	*Ambrosia*	*Ambrosia trifida*	AY215512
Eudicotyledons	Asteraceae	*Ambrosia*	*Ambrosia trifida*	EF590670
Eudicotyledons	Asteraceae	*Ambrosia*	*Ambrosia trifida*	DQ006142
Eudicotyledons	Asteraceae	*Arctium*	*Arctium lappa*	GU724258
Eudicotyledons	Asteraceae	*Arctium*	*Arctium lappa*	GQ435104
Eudicotyledons	Asteraceae	*Arctium*	*Arctium lappa*	GQ435105
Eudicotyledons	Asteraceae	*Arctium*	*Arctium lappa*	AY914834
Eudicotyledons	Asteraceae	*Artemisia*	*Artemisia vulgaris*	HQ596606
Eudicotyledons	Asteraceae	*Artemisia*	*Artemisia vulgaris*	AB683349
Eudicotyledons	Asteraceae	*Helianthus*	*Helianthus annuus*	AM490227
Eudicotyledons	Asteraceae	*Helianthus*	*Helianthus annuus*	AY215554
Eudicotyledons	Asteraceae	*Helianthus*	*Helianthus tuberosus*	GU818386
Eudicotyledons	Asteraceae	*Helianthus*	*Helianthus tuberosus*	AM490226
Eudicotyledons	Asteraceae	*Helianthus*	*Helianthus tuberosus*	HQ688937
Eudicotyledons	Asteraceae	*Arnica*	*Arnica mollis*	AM690568
Eudicotyledons	Asteraceae	*Arnica*	*Arnica mollis*	AY215514
Eudicotyledons	Cornaceae	*Cornus*	*Cornus racemosa*	EU750448
Eudicotyledons	Cornaceae	*Cornus*	*Cornus racemosa*	EU750449
Eudicotyledons	Cornaceae	*Cornus*	*Cornus racemosa*	JF321216
Eudicotyledons	Cornaceae	*Nyssa*	*Nyssa sylvatica*	EU734456
Eudicotyledons	Cornaceae	*Nyssa*	*Nyssa sylvatica*	JN045594
Eudicotyledons	Cornaceae	*Nyssa*	*Nyssa sylvatica*	JN045595
Eudicotyledons	Cornaceae	*Nyssa*	*Nyssa sylvatica*	JN045596
Eudicotyledons	Cornaceae	*Nyssa*	*Nyssa sylvatica*	JF321236
Eudicotyledons	Aquifoliaceae	*Ilex*	*Ilex aquifolium*	FJ395543
Eudicotyledons	Aquifoliaceae	*Ilex*	*Ilex aquifolium*	EU359337
Eudicotyledons	Aquifoliaceae	*Ilex*	*Ilex aquifolium*	FN675791
Eudicotyledons	Ericaceae	*Rhododendron*	*Rhododendron hippophaeoides*	JN046840
Eudicotyledons	Ericaceae	*Rhododendron*	*Rhododendron hippophaeoides*	JN046841
Eudicotyledons	Hamamelidaceae	*Hamamelis*	*Hamamelis mollis*	GU576760
Eudicotyledons	Hamamelidaceae	*Hamamelis*	*Hamamelis mollis*	GU576761
Eudicotyledons	Hamamelidaceae	*Hamamelis*	*Hamamelis mollis*	GU576762
Eudicotyledons	Hamamelidaceae	*Hamamelis*	*Hamamelis virginiana*	GU576769
Eudicotyledons	Hamamelidaceae	*Hamamelis*	*Hamamelis virginiana*	GU576770
Eudicotyledons	Hamamelidaceae	*Hamamelis*	*Hamamelis virginiana*	EU595863
Eudicotyledons	Altingiaceae	*Liquidambar*	*Liquidambar styraciflua*	GU576773
Eudicotyledons	Altingiaceae	*Liquidambar*	*Liquidambar styraciflua*	AB445366
Eudicotyledons	Altingiaceae	*Liquidambar*	*Liquidambar styraciflua*	EF138707
Eudicotyledons	Altingiaceae	*Liquidambar*	*Liquidambar styraciflua*	EF138708
Eudicotyledons	Altingiaceae	*Liquidambar*	*Liquidambar styraciflua*	EF138709
Eudicotyledons	Altingiaceae	*Liquidambar*	*Liquidambar styraciflua*	EF138710
Eudicotyledons	Altingiaceae	*Liquidambar*	*Liquidambar styraciflua*	EF138711
Eudicotyledons	Altingiaceae	*Liquidambar*	*Liquidambar styraciflua*	EF138712
Eudicotyledons	Altingiaceae	*Liquidambar*	*Liquidambar styraciflua*	EF138713
Eudicotyledons	Altingiaceae	*Liquidambar*	*Liquidambar styraciflua*	EF138714
Eudicotyledons	Altingiaceae	*Liquidambar*	*Liquidambar styraciflua*	EF138715
Eudicotyledons	Altingiaceae	*Liquidambar*	*Liquidambar styraciflua*	EF138716
Eudicotyledons	Altingiaceae	*Liquidambar*	*Liquidambar styraciflua*	EF138717
Eudicotyledons	Altingiaceae	*Liquidambar*	*Liquidambar styraciflua*	EF138718
Eudicotyledons	Altingiaceae	*Liquidambar*	*Liquidambar styraciflua*	EF138719

Eudicotyledons	Altingiaceae	*Liquidambar*	*Liquidambar styraciflua*	EF138720
Eudicotyledons	Altingiaceae	*Liquidambar*	*Liquidambar styraciflua*	EF138721
Eudicotyledons	Altingiaceae	*Liquidambar*	*Liquidambar styraciflua*	EF138722
Eudicotyledons	Altingiaceae	*Liquidambar*	*Liquidambar styraciflua*	EF138723
Eudicotyledons	Altingiaceae	*Liquidambar*	*Liquidambar styraciflua*	EF138724
Eudicotyledons	Altingiaceae	*Liquidambar*	*Liquidambar styraciflua*	EF138725
Eudicotyledons	Altingiaceae	*Liquidambar*	*Liquidambar styraciflua*	EF138726
Eudicotyledons	Altingiaceae	*Liquidambar*	*Liquidambar styraciflua*	EF138727
Eudicotyledons	Altingiaceae	*Liquidambar*	*Liquidambar styraciflua*	EF138728
Eudicotyledons	Cornaceae	*Cornus*	*Cornus alternifolia*	EU750446
Eudicotyledons	Cornaceae	*Cornus*	*Cornus alternifolia*	EU750447
Eudicotyledons	Cornaceae	*Cornus*	*Cornus alternifolia*	JF321212
Eudicotyledons	Cornaceae	*Cornus*	*Cornus officinalis*	GQ435328
Eudicotyledons	Cornaceae	*Cornus*	*Cornus officinalis*	JF321222
Eudicotyledons	Fabaceae	*Wisteria*	*Wisteria sinensis*	GU396782
Eudicotyledons	Fabaceae	*Wisteria*	*Wisteria sinensis*	EU424102
Eudicotyledons	Fabaceae	*Wisteria*	*Wisteria sinensis*	EU424103
Eudicotyledons	Fabaceae	*Wisteria*	*Wisteria sinensis*	EU424104
Eudicotyledons	Fabaceae	*Wisteria*	*Wisteria sinensis*	GU135426
Eudicotyledons	Fabaceae	*Wisteria*	*Wisteria sinensis*	FN675802
Eudicotyledons	Rosaceae	*Prunus*	*Prunus serotina*	HQ596803
Eudicotyledons	Rosaceae	*Prunus*	*Prunus serotina*	DQ006222
Eudicotyledons	Rosaceae	*Prunus*	*Prunus serotina*	AY500633
Eudicotyledons	Fabaceae	*Canavalia*	*Canavalia cathartica*	GU396816
Eudicotyledons	Fabaceae	*Canavalia*	*Canavalia cathartica*	HQ707430
Eudicotyledons	Fabaceae	*Canavalia*	*Canavalia cathartica*	HQ707432
Eudicotyledons	Amaranthaceae	*Amaranthus*	*Amaranthus tricolor*	JN043902
Eudicotyledons	Amaranthaceae	*Amaranthus*	*Amaranthus tricolor*	JN043903
Eudicotyledons	Amaranthaceae	*Amaranthus*	*Amaranthus tricolor*	JN043904
Eudicotyledons	Amaranthaceae	*Amaranthus*	*Amaranthus tricolor*	JN043905
Eudicotyledons	Amaranthaceae	*Amaranthus*	*Amaranthus tricolor*	JN043906
Eudicotyledons	Amaranthaceae	*Amaranthus*	*Amaranthus tricolor*	JN043907
Eudicotyledons	Amaranthaceae	*Amaranthus*	*Amaranthus tricolor*	JN043908
Eudicotyledons	Amaranthaceae	*Amaranthus*	*Amaranthus tricolor*	JN043909
Eudicotyledons	Malvaceae	*Gossypium*	*Gossypium arboreum*	HM437871
Eudicotyledons	Malvaceae	*Gossypium*	*Gossypium arboreum*	HM437872
Eudicotyledons	Malvaceae	*Gossypium*	*Gossypium arboreum*	HM437873
Eudicotyledons	Malvaceae	*Gossypium*	*Gossypium arboreum*	HM437874
Eudicotyledons	Malvaceae	*Gossypium*	*Gossypium arboreum*	HM437875
Eudicotyledons	Malvaceae	*Gossypium*	*Gossypium arboreum*	HM437876
Eudicotyledons	Malvaceae	*Gossypium*	*Gossypium arboreum*	HM437877
Eudicotyledons	Vitaceae	*Vitis*	*Vitis vinifera*	FR725983
Eudicotyledons	Vitaceae	*Vitis*	*Vitis vinifera*	FR725984
Eudicotyledons	Vitaceae	*Vitis*	*Vitis vinifera*	FR725985
Eudicotyledons	Vitaceae	*Vitis*	*Vitis vinifera*	FR725986
Eudicotyledons	Vitaceae	*Vitis*	*Vitis vinifera*	FR725987
Eudicotyledons	Vitaceae	*Vitis*	*Vitis vinifera*	FR725988
Eudicotyledons	Vitaceae	*Vitis*	*Vitis vinifera*	FR725989
Eudicotyledons	Vitaceae	*Vitis*	*Vitis vinifera*	FR725990
Eudicotyledons	Vitaceae	*Vitis*	*Vitis vinifera*	FR725991
Eudicotyledons	Vitaceae	*Vitis*	*Vitis vinifera*	FR725992
Eudicotyledons	Vitaceae	*Vitis*	*Vitis vinifera*	FR725993
Eudicotyledons	Vitaceae	*Vitis*	*Vitis vinifera*	FR725994

Eudicotyledons	Vitaceae	*Vitis*	*Vitis vinifera*	FR725995
Eudicotyledons	Vitaceae	*Vitis*	*Vitis vinifera*	FR725996
Eudicotyledons	Vitaceae	*Vitis*	*Vitis vinifera*	FR725997
Eudicotyledons	Vitaceae	*Vitis*	*Vitis vinifera*	FR725998
Eudicotyledons	Vitaceae	*Vitis*	*Vitis vinifera*	FR725999
Eudicotyledons	Vitaceae	*Vitis*	*Vitis vinifera*	FR726000
Eudicotyledons	Vitaceae	*Vitis*	*Vitis vinifera*	FR726001
Eudicotyledons	Vitaceae	*Vitis*	*Vitis vinifera*	FR726002
Eudicotyledons	Vitaceae	*Vitis*	*Vitis vinifera*	FR726003
Eudicotyledons	Vitaceae	*Vitis*	*Vitis vinifera*	FR726004
Eudicotyledons	Vitaceae	*Vitis*	*Vitis vinifera*	FR726005
Eudicotyledons	Vitaceae	*Vitis*	*Vitis vinifera*	FR726006
Eudicotyledons	Vitaceae	*Vitis*	*Vitis vinifera*	FR726007
Eudicotyledons	Vitaceae	*Vitis*	*Vitis vinifera*	FR726008
Eudicotyledons	Vitaceae	*Vitis*	*Vitis vinifera*	FR726009
Eudicotyledons	Vitaceae	*Vitis*	*Vitis vinifera*	FR726010
Eudicotyledons	Vitaceae	*Vitis*	*Vitis vinifera*	FR726011
Eudicotyledons	Vitaceae	*Vitis*	*Vitis vinifera*	FR726012
Eudicotyledons	Vitaceae	*Vitis*	*Vitis vinifera*	FR726013
Eudicotyledons	Vitaceae	*Vitis*	*Vitis vinifera*	FR726014
Eudicotyledons	Vitaceae	*Vitis*	*Vitis vinifera*	FR726015
Eudicotyledons	Vitaceae	*Vitis*	*Vitis vinifera*	FR726016
Eudicotyledons	Vitaceae	*Vitis*	*Vitis vinifera*	FR726017
Eudicotyledons	Vitaceae	*Vitis*	*Vitis vinifera*	FR726018
Eudicotyledons	Vitaceae	*Vitis*	*Vitis vinifera*	FR726019
Eudicotyledons	Vitaceae	*Vitis*	*Vitis vinifera*	FR726020
Eudicotyledons	Vitaceae	*Vitis*	*Vitis vinifera*	FR726021
Eudicotyledons	Vitaceae	*Vitis*	*Vitis vinifera*	FR726022
Eudicotyledons	Vitaceae	*Vitis*	*Vitis vinifera*	FR726023
Eudicotyledons	Vitaceae	*Vitis*	*Vitis vinifera*	FR726024
Eudicotyledons	Vitaceae	*Vitis*	*Vitis vinifera*	FR726025
Eudicotyledons	Vitaceae	*Vitis*	*Vitis vinifera*	FR726026
Eudicotyledons	Vitaceae	*Vitis*	*Vitis vinifera*	FR726027
Eudicotyledons	Vitaceae	*Vitis*	*Vitis vinifera*	FR726028
Eudicotyledons	Vitaceae	*Vitis*	*Vitis vinifera*	FR726029
Eudicotyledons	Vitaceae	*Vitis*	*Vitis vinifera*	FR726030
Eudicotyledons	Vitaceae	*Vitis*	*Vitis vinifera*	FR726031
Eudicotyledons	Vitaceae	*Vitis*	*Vitis vinifera*	FR726032
Eudicotyledons	Vitaceae	*Vitis*	*Vitis vinifera*	FR726033
Eudicotyledons	Vitaceae	*Vitis*	*Vitis vinifera*	FR726034
Eudicotyledons	Vitaceae	*Vitis*	*Vitis vinifera*	FR726035
Eudicotyledons	Vitaceae	*Vitis*	*Vitis vinifera*	FR726036
Eudicotyledons	Vitaceae	*Vitis*	*Vitis vinifera*	FR726037
Eudicotyledons	Vitaceae	*Vitis*	*Vitis vinifera*	FR726038
Eudicotyledons	Vitaceae	*Vitis*	*Vitis vinifera*	FR726039
Eudicotyledons	Vitaceae	*Vitis*	*Vitis vinifera*	FR726040
Eudicotyledons	Vitaceae	*Vitis*	*Vitis vinifera*	FR726041
Eudicotyledons	Vitaceae	*Vitis*	*Vitis vinifera*	FR726042
Eudicotyledons	Vitaceae	*Vitis*	*Vitis vinifera*	FR726043
Eudicotyledons	Vitaceae	*Vitis*	*Vitis vinifera*	FR726044
Eudicotyledons	Vitaceae	*Vitis*	*Vitis vinifera*	FR726045
Eudicotyledons	Vitaceae	*Vitis*	*Vitis vinifera*	FR726046
Eudicotyledons	Vitaceae	*Vitis*	*Vitis vinifera*	FR726047

Eudicotyledons	Vitaceae	*Vitis*	*Vitis vinifera*	FR726048
Eudicotyledons	Vitaceae	*Vitis*	*Vitis vinifera*	FR726049
Eudicotyledons	Vitaceae	*Vitis*	*Vitis vinifera*	FR726050
Eudicotyledons	Vitaceae	*Vitis*	*Vitis vinifera*	FR726051
Eudicotyledons	Vitaceae	*Vitis*	*Vitis vinifera*	FR726052
Eudicotyledons	Vitaceae	*Vitis*	*Vitis vinifera*	FR726053
Eudicotyledons	Vitaceae	*Vitis*	*Vitis vinifera*	FR726054
Eudicotyledons	Vitaceae	*Vitis*	*Vitis vinifera*	FR726055
Eudicotyledons	Vitaceae	*Vitis*	*Vitis vinifera*	FR726056
Eudicotyledons	Vitaceae	*Vitis*	*Vitis vinifera*	FR726057
Eudicotyledons	Vitaceae	*Vitis*	*Vitis vinifera*	FR726058
Eudicotyledons	Vitaceae	*Vitis*	*Vitis vinifera*	FR726059
Eudicotyledons	Vitaceae	*Vitis*	*Vitis vinifera*	FR726060
Eudicotyledons	Vitaceae	*Vitis*	*Vitis vinifera*	FR726061
Eudicotyledons	Vitaceae	*Vitis*	*Vitis vinifera*	FR726062
Eudicotyledons	Vitaceae	*Vitis*	*Vitis vinifera*	FR726063
Eudicotyledons	Vitaceae	*Vitis*	*Vitis vinifera*	FR726064
Eudicotyledons	Vitaceae	*Vitis*	*Vitis vinifera*	FR726065
Eudicotyledons	Vitaceae	*Vitis*	*Vitis vinifera*	FR726066
Eudicotyledons	Vitaceae	*Vitis*	*Vitis vinifera*	FR726067
Eudicotyledons	Vitaceae	*Vitis*	*Vitis vinifera*	FR726068
Eudicotyledons	Vitaceae	*Vitis*	*Vitis vinifera*	FR726069
Eudicotyledons	Vitaceae	*Vitis*	*Vitis vinifera*	FR726070
Eudicotyledons	Vitaceae	*Vitis*	*Vitis vinifera*	FR726071
Eudicotyledons	Vitaceae	*Vitis*	*Vitis vinifera*	FR726072
Eudicotyledons	Vitaceae	*Vitis*	*Vitis vinifera*	FR726073
Eudicotyledons	Vitaceae	*Vitis*	*Vitis vinifera*	FR726074
Eudicotyledons	Vitaceae	*Vitis*	*Vitis vinifera*	FR726075
Eudicotyledons	Vitaceae	*Vitis*	*Vitis vinifera*	FR726076
Eudicotyledons	Vitaceae	*Vitis*	*Vitis vinifera*	FR726077
Eudicotyledons	Vitaceae	*Vitis*	*Vitis vinifera*	FR726078
Eudicotyledons	Vitaceae	*Vitis*	*Vitis vinifera*	FR726079
Eudicotyledons	Vitaceae	*Vitis*	*Vitis vinifera*	FR726080
Eudicotyledons	Vitaceae	*Vitis*	*Vitis vinifera*	FR726081
Eudicotyledons	Vitaceae	*Vitis*	*Vitis vinifera*	FR726082
Eudicotyledons	Vitaceae	*Vitis*	*Vitis vinifera*	FR726083
Eudicotyledons	Vitaceae	*Vitis*	*Vitis vinifera*	FR726084
Eudicotyledons	Vitaceae	*Vitis*	*Vitis vinifera*	FR726085
Eudicotyledons	Vitaceae	*Vitis*	*Vitis vinifera*	FR726086
Eudicotyledons	Vitaceae	*Vitis*	*Vitis vinifera*	FR726087
Eudicotyledons	Vitaceae	*Vitis*	*Vitis vinifera*	FR726088
Eudicotyledons	Vitaceae	*Vitis*	*Vitis vinifera*	FR726089
Eudicotyledons	Vitaceae	*Vitis*	*Vitis vinifera*	FR726090
Eudicotyledons	Vitaceae	*Vitis*	*Vitis vinifera*	FR726091
Eudicotyledons	Vitaceae	*Vitis*	*Vitis vinifera*	FR726092
Eudicotyledons	Vitaceae	*Vitis*	*Vitis vinifera*	FR726093
Eudicotyledons	Vitaceae	*Vitis*	*Vitis vinifera*	FR726094
Eudicotyledons	Vitaceae	*Vitis*	*Vitis vinifera*	FR726095
Eudicotyledons	Vitaceae	*Vitis*	*Vitis vinifera*	HQ108320
Eudicotyledons	Vitaceae	*Vitis*	*Vitis vinifera*	HQ108321
Eudicotyledons	Vitaceae	*Vitis*	*Vitis vinifera*	HQ108322
Eudicotyledons	Vitaceae	*Vitis*	*Vitis vinifera*	HQ108323
Eudicotyledons	Vitaceae	*Vitis*	*Vitis vinifera*	HQ108324

Eudicotyledons	Vitaceae	*Vitis*	*Vitis vinifera*	HQ108325
Eudicotyledons	Vitaceae	*Vitis*	*Vitis vinifera*	HQ108326
Eudicotyledons	Vitaceae	*Vitis*	*Vitis vinifera*	HQ108327
Eudicotyledons	Vitaceae	*Vitis*	*Vitis vinifera*	HQ108328
Eudicotyledons	Vitaceae	*Vitis*	*Vitis vinifera*	HQ108329
Eudicotyledons	Vitaceae	*Vitis*	*Vitis vinifera*	HQ108330
Eudicotyledons	Vitaceae	*Vitis*	*Vitis vinifera*	HQ108331
Eudicotyledons	Vitaceae	*Vitis*	*Vitis vinifera*	HQ108332
Eudicotyledons	Vitaceae	*Vitis*	*Vitis vinifera*	HQ108333
Eudicotyledons	Vitaceae	*Vitis*	*Vitis vinifera*	HQ108334
Eudicotyledons	Vitaceae	*Vitis*	*Vitis vinifera*	HQ108335
Eudicotyledons	Rosaceae	*Prunus*	*Prunus laurocerasus*	AY500635
Eudicotyledons	Rosaceae	*Prunus*	*Prunus laurocerasus*	FN675834
Eudicotyledons	Rosaceae	*Rosa*	*Rosa woodsii*	DQ778814
Eudicotyledons	Rosaceae	*Rosa*	*Rosa woodsii*	DQ778815
Eudicotyledons	Solanaceae	*Petunia*	*Petunia axillaris*	AY772884
Eudicotyledons	Solanaceae	*Petunia*	*Petunia axillaris*	AY772885
Eudicotyledons	Solanaceae	*Petunia*	*Petunia axillaris*	AY772886
Eudicotyledons	Solanaceae	*Petunia*	*Petunia axillaris*	DQ225609
Eudicotyledons	Solanaceae	*Petunia*	*Petunia axillaris*	DQ225610
Eudicotyledons	Solanaceae	*Petunia*	*Petunia axillaris*	DQ225611
Eudicotyledons	Solanaceae	*Petunia*	*Petunia axillaris*	DQ225612
Eudicotyledons	Solanaceae	*Petunia*	*Petunia axillaris*	DQ225613
Eudicotyledons	Solanaceae	*Petunia*	*Petunia axillaris*	DQ225614
Eudicotyledons	Solanaceae	*Petunia*	*Petunia axillaris*	DQ225615
Eudicotyledons	Solanaceae	*Petunia*	*Petunia axillaris*	DQ225616
Eudicotyledons	Solanaceae	*Petunia*	*Petunia axillaris*	DQ225617
Eudicotyledons	Solanaceae	*Petunia*	*Petunia axillaris*	DQ225618
Eudicotyledons	Solanaceae	*Petunia*	*Petunia axillaris*	DQ225619
Eudicotyledons	Solanaceae	*Petunia*	*Petunia axillaris*	DQ225620
Eudicotyledons	Solanaceae	*Petunia*	*Petunia axillaris*	DQ225621
Eudicotyledons	Solanaceae	*Petunia*	*Petunia axillaris*	DQ225622
Eudicotyledons	Solanaceae	*Petunia*	*Petunia axillaris*	DQ225623
Eudicotyledons	Solanaceae	*Petunia*	*Petunia axillaris*	DQ225624
Eudicotyledons	Solanaceae	*Petunia*	*Petunia axillaris*	DQ225625
Eudicotyledons	Solanaceae	*Petunia*	*Petunia axillaris*	DQ225626
Eudicotyledons	Solanaceae	*Petunia*	*Petunia axillaris*	DQ225627
Eudicotyledons	Solanaceae	*Petunia*	*Petunia axillaris*	DQ225628
Eudicotyledons	Solanaceae	*Petunia*	*Petunia axillaris*	DQ225629
Eudicotyledons	Solanaceae	*Petunia*	*Petunia axillaris*	DQ225630
Eudicotyledons	Solanaceae	*Petunia*	*Petunia axillaris*	DQ225631
Eudicotyledons	Solanaceae	*Petunia*	*Petunia axillaris*	DQ225632
Eudicotyledons	Solanaceae	*Petunia*	*Petunia axillaris*	DQ225633
Eudicotyledons	Solanaceae	*Petunia*	*Petunia axillaris*	DQ225634
Eudicotyledons	Solanaceae	*Petunia*	*Petunia axillaris*	DQ225635
Eudicotyledons	Solanaceae	*Petunia*	*Petunia axillaris*	DQ225636
Eudicotyledons	Solanaceae	*Petunia*	*Petunia axillaris*	DQ225637
Eudicotyledons	Solanaceae	*Petunia*	*Petunia axillaris*	DQ225638
Eudicotyledons	Solanaceae	*Petunia*	*Petunia axillaris*	DQ225639
Eudicotyledons	Solanaceae	*Petunia*	*Petunia axillaris*	DQ225640
Eudicotyledons	Solanaceae	*Petunia*	*Petunia axillaris*	DQ225641
Eudicotyledons	Solanaceae	*Petunia*	*Petunia axillaris*	DQ225642
Eudicotyledons	Solanaceae	*Petunia*	*Petunia axillaris*	DQ225643

Eudicotyledons	Solanaceae	*Petunia*	*Petunia axillaris*	DQ225644
Eudicotyledons	Solanaceae	*Petunia*	*Petunia axillaris*	DQ225645
Eudicotyledons	Solanaceae	*Petunia*	*Petunia axillaris*	DQ225646
Eudicotyledons	Solanaceae	*Petunia*	*Petunia axillaris*	DQ225647
Eudicotyledons	Solanaceae	*Petunia*	*Petunia axillaris*	DQ225648
Eudicotyledons	Solanaceae	*Petunia*	*Petunia axillaris*	DQ225649
Eudicotyledons	Solanaceae	*Petunia*	*Petunia axillaris*	DQ225650
Eudicotyledons	Solanaceae	*Petunia*	*Petunia axillaris*	DQ225651
Eudicotyledons	Solanaceae	*Petunia*	*Petunia axillaris*	DQ225652
Eudicotyledons	Solanaceae	*Petunia*	*Petunia axillaris*	DQ225653
Eudicotyledons	Solanaceae	*Petunia*	*Petunia axillaris*	DQ225654
Eudicotyledons	Solanaceae	*Petunia*	*Petunia axillaris*	DQ225655
Eudicotyledons	Solanaceae	*Petunia*	*Petunia axillaris*	DQ225656
Eudicotyledons	Solanaceae	*Petunia*	*Petunia axillaris*	DQ225657
Eudicotyledons	Solanaceae	*Petunia*	*Petunia axillaris*	DQ225658
Eudicotyledons	Solanaceae	*Petunia*	*Petunia axillaris*	DQ225659
Eudicotyledons	Solanaceae	*Petunia*	*Petunia axillaris*	DQ225660
Eudicotyledons	Solanaceae	*Petunia*	*Petunia axillaris*	DQ225661
Eudicotyledons	Solanaceae	*Petunia*	*Petunia axillaris*	DQ225662
Eudicotyledons	Solanaceae	*Petunia*	*Petunia axillaris*	DQ225663
Eudicotyledons	Solanaceae	*Petunia*	*Petunia axillaris*	DQ225664
Eudicotyledons	Solanaceae	*Petunia*	*Petunia axillaris*	DQ225665
Eudicotyledons	Malvaceae	*Gossypium*	*Gossypium herbaceum*	HM437886
Eudicotyledons	Malvaceae	*Gossypium*	*Gossypium herbaceum*	HM437887
Eudicotyledons	Malvaceae	*Gossypium*	*Gossypium herbaceum*	HM437888
Eudicotyledons	Malvaceae	*Gossypium*	*Gossypium herbaceum*	HM437889
Eudicotyledons	Malvaceae	*Gossypium*	*Gossypium herbaceum*	HM437890
Eudicotyledons	Malvaceae	*Gossypium*	*Gossypium herbaceum*	HM437891
Eudicotyledons	Malvaceae	*Gossypium*	*Gossypium herbaceum*	HM437892
Eudicotyledons	Malvaceae	*Gossypium*	*Gossypium herbaceum*	HM437893
Eudicotyledons	Malvaceae	*Gossypium*	*Gossypium herbaceum*	HM437894
Eudicotyledons	Malvaceae	*Gossypium*	*Gossypium herbaceum*	HM437895
Eudicotyledons	Malvaceae	*Gossypium*	*Gossypium herbaceum*	HM437896
Eudicotyledons	Malvaceae	*Gossypium*	*Gossypium herbaceum*	HM437897
Eudicotyledons	Malvaceae	*Gossypium*	*Gossypium herbaceum*	HM437898
Eudicotyledons	Malvaceae	*Gossypium*	*Gossypium herbaceum*	HM437899
Eudicotyledons	Malvaceae	*Gossypium*	*Gossypium herbaceum*	HM437900
Eudicotyledons	Myrtaceae	*Eucalyptus*	*Eucalyptus globulus*	GQ248303
Eudicotyledons	Myrtaceae	*Eucalyptus*	*Eucalyptus globulus*	EF434409
Eudicotyledons	Myrtaceae	*Eucalyptus*	*Eucalyptus globulus*	EF590698
Eudicotyledons	Asteraceae	*Artemisia*	*Artemisia annua*	FJ418749
Eudicotyledons	Asteraceae	*Artemisia*	*Artemisia annua*	GQ435090
Eudicotyledons	Asteraceae	*Artemisia*	*Artemisia annua*	GQ435091
Eudicotyledons	Asteraceae	*Artemisia*	*Artemisia annua*	DQ006143
Eudicotyledons	Solanaceae	*Datura*	*Datura metel*	GQ435287
Eudicotyledons	Solanaceae	*Datura*	*Datura metel*	JN244381
Eudicotyledons	Solanaceae	*Datura*	*Datura metel*	JN244382
Eudicotyledons	Solanaceae	*Datura*	*Datura metel*	JN244383
Eudicotyledons	Solanaceae	*Datura*	*Datura metel*	EF590685
Eudicotyledons	Linaceae	*Linum*	*Linum perenne*	HQ596750
Eudicotyledons	Linaceae	*Linum*	*Linum perenne*	GQ845287
Eudicotyledons	Santalaceae	*Santalum*	*Santalum album*	GQ435374
Eudicotyledons	Santalaceae	*Santalum*	*Santalum album*	GQ435375

Eudicotyledons	Santalaceae	*Santalum*	*Santalum album*	GQ435376
Eudicotyledons	Santalaceae	*Santalum*	*Santalum album*	GQ435377
Eudicotyledons	Rosaceae	*Prunus*	*Prunus armeniaca*	AY500620
Eudicotyledons	Rosaceae	*Prunus*	*Prunus armeniaca*	FN675830
Eudicotyledons	Rosaceae	*Prunus*	*Prunus armeniaca*	JN046602
Eudicotyledons	Rosaceae	*Prunus*	*Prunus armeniaca*	JN046603
Eudicotyledons	Rosaceae	*Prunus*	*Prunus armeniaca*	JN046604
Eudicotyledons	Rosaceae	*Prunus*	*Prunus armeniaca*	JN046605
Eudicotyledons	Rosaceae	*Prunus*	*Prunus armeniaca*	JN046606
Eudicotyledons	Rosaceae	*Prunus*	*Prunus armeniaca*	JN046607
Eudicotyledons	Rosaceae	*Prunus*	*Prunus armeniaca*	JN046635
Eudicotyledons	Rosaceae	*Prunus*	*Prunus armeniaca*	JN046636
Eudicotyledons	Rosaceae	*Prunus*	*Prunus armeniaca*	JN046637
Eudicotyledons	Rosaceae	*Prunus*	*Prunus armeniaca*	JN046638
Eudicotyledons	Rosaceae	*Chaenomeles*	*Chaenomeles cathayensis*	JQ390648
Eudicotyledons	Rosaceae	*Chaenomeles*	*Chaenomeles cathayensis*	JQ390652
Eudicotyledons	Rosaceae	*Chaenomeles*	*Chaenomeles sinensis*	JQ390755
Eudicotyledons	Rosaceae	*Chaenomeles*	*Chaenomeles sinensis*	JQ390756
Eudicotyledons	Rosaceae	*Chaenomeles*	*Chaenomeles sinensis*	JQ390757
Eudicotyledons	Caryophyllaceae	*Silene*	*Silene latifolia*	EF091495
Eudicotyledons	Caryophyllaceae	*Silene*	*Silene latifolia*	EF091496
Eudicotyledons	Caryophyllaceae	*Silene*	*Silene latifolia*	EF091497
Eudicotyledons	Caryophyllaceae	*Silene*	*Silene latifolia*	EF091498
Eudicotyledons	Caryophyllaceae	*Silene*	*Silene latifolia*	EF091499
Eudicotyledons	Caryophyllaceae	*Silene*	*Silene latifolia*	EF091500
Eudicotyledons	Caryophyllaceae	*Silene*	*Silene latifolia*	EF091501
Eudicotyledons	Caryophyllaceae	*Silene*	*Silene latifolia*	EF091502
Eudicotyledons	Caryophyllaceae	*Silene*	*Silene latifolia*	EF091503
Eudicotyledons	Caryophyllaceae	*Silene*	*Silene latifolia*	EF091504
Eudicotyledons	Caryophyllaceae	*Silene*	*Silene latifolia*	EF091505
Eudicotyledons	Caryophyllaceae	*Silene*	*Silene latifolia*	EF091506
Eudicotyledons	Caryophyllaceae	*Silene*	*Silene latifolia*	EF091507
Eudicotyledons	Caryophyllaceae	*Silene*	*Silene latifolia*	EF091508
Eudicotyledons	Caryophyllaceae	*Silene*	*Silene latifolia*	EF091509
Eudicotyledons	Caryophyllaceae	*Silene*	*Silene latifolia*	EF091510
Eudicotyledons	Caryophyllaceae	*Silene*	*Silene latifolia*	EF091511
Eudicotyledons	Caryophyllaceae	*Silene*	*Silene latifolia*	EF091512
Eudicotyledons	Caryophyllaceae	*Silene*	*Silene latifolia*	EF091513
Eudicotyledons	Caryophyllaceae	*Silene*	*Silene latifolia*	EF091514
Eudicotyledons	Caryophyllaceae	*Silene*	*Silene latifolia*	EF091515
Eudicotyledons	Caryophyllaceae	*Silene*	*Silene latifolia*	EF091516
Eudicotyledons	Caryophyllaceae	*Silene*	*Silene latifolia*	EF091517
Eudicotyledons	Caryophyllaceae	*Silene*	*Silene latifolia*	EF091518
Eudicotyledons	Caryophyllaceae	*Silene*	*Silene latifolia*	EF091519
Eudicotyledons	Caryophyllaceae	*Silene*	*Silene latifolia*	EF091520
Eudicotyledons	Caryophyllaceae	*Silene*	*Silene latifolia*	EF091521
Eudicotyledons	Caryophyllaceae	*Silene*	*Silene latifolia*	EF091522
Eudicotyledons	Caryophyllaceae	*Silene*	*Silene latifolia*	EF091524
Eudicotyledons	Caryophyllaceae	*Silene*	*Silene latifolia*	EF091525
Eudicotyledons	Caryophyllaceae	*Silene*	*Silene latifolia*	EF091526
Eudicotyledons	Caryophyllaceae	*Silene*	*Silene latifolia*	EF091528
Eudicotyledons	Caryophyllaceae	*Silene*	*Silene latifolia*	EF091529
Eudicotyledons	Caryophyllaceae	*Silene*	*Silene latifolia*	EF091530

Eudicotyledons	Caryophyllaceae	*Silene*	*Silene latifolia*	EF091531
Eudicotyledons	Caryophyllaceae	*Silene*	*Silene latifolia*	EF091532
Eudicotyledons	Caryophyllaceae	*Silene*	*Silene latifolia*	GU562391
Eudicotyledons	Caryophyllaceae	*Silene*	*Silene latifolia*	AF518904
Eudicotyledons	Caryophyllaceae	*Silene*	*Silene latifolia*	AF518905
Eudicotyledons	Caryophyllaceae	*Silene*	*Silene latifolia*	AF518906
Eudicotyledons	Caryophyllaceae	*Silene*	*Silene latifolia*	AF518907
Eudicotyledons	Caryophyllaceae	*Silene*	*Silene latifolia*	AF518908
Eudicotyledons	Caryophyllaceae	*Silene*	*Silene latifolia*	AF518909
Eudicotyledons	Caryophyllaceae	*Silene*	*Silene latifolia*	AF518910
Eudicotyledons	Caryophyllaceae	*Silene*	*Silene latifolia*	AF518911
Eudicotyledons	Caryophyllaceae	*Silene*	*Silene latifolia*	AF518912
Eudicotyledons	Caryophyllaceae	*Silene*	*Silene latifolia*	AF518913
Eudicotyledons	Caryophyllaceae	*Silene*	*Silene latifolia*	AF518914
Eudicotyledons	Caryophyllaceae	*Silene*	*Silene latifolia*	AF518915
Eudicotyledons	Caryophyllaceae	*Silene*	*Silene latifolia*	AF518916
Eudicotyledons	Caryophyllaceae	*Silene*	*Silene latifolia*	AF518917
Eudicotyledons	Caryophyllaceae	*Silene*	*Silene latifolia*	AF518918
Eudicotyledons	Caryophyllaceae	*Silene*	*Silene latifolia*	AF518919
Eudicotyledons	Caryophyllaceae	*Silene*	*Silene latifolia*	AF518920
Eudicotyledons	Caryophyllaceae	*Silene*	*Silene latifolia*	AF518921
Eudicotyledons	Caryophyllaceae	*Silene*	*Silene latifolia*	AF518922
Eudicotyledons	Caryophyllaceae	*Silene*	*Silene latifolia*	AF518923
Eudicotyledons	Caryophyllaceae	*Silene*	*Silene latifolia*	AF518924
Eudicotyledons	Caryophyllaceae	*Silene*	*Silene latifolia*	AF518925
Eudicotyledons	Caryophyllaceae	*Silene*	*Silene latifolia*	AF518926
Eudicotyledons	Caryophyllaceae	*Silene*	*Silene latifolia*	AF518927
Eudicotyledons	Caryophyllaceae	*Silene*	*Silene latifolia*	AF518928
Eudicotyledons	Caryophyllaceae	*Silene*	*Silene latifolia*	EU750546
Eudicotyledons	Caryophyllaceae	*Silene*	*Silene latifolia*	EU750547
Eudicotyledons	Caryophyllaceae	*Silene*	*Silene latifolia*	FN821213
Eudicotyledons	Caryophyllaceae	*Silene*	*Silene latifolia*	FN821214
Eudicotyledons	Caryophyllaceae	*Silene*	*Silene latifolia*	FN821215
Eudicotyledons	Caryophyllaceae	*Silene*	*Silene latifolia*	FN821216
Eudicotyledons	Caryophyllaceae	*Silene*	*Silene latifolia*	FN821217
Eudicotyledons	Caryophyllaceae	*Silene*	*Silene latifolia*	FN821218
Eudicotyledons	Caryophyllaceae	*Silene*	*Silene latifolia*	FN821219
Eudicotyledons	Caryophyllaceae	*Silene*	*Silene latifolia*	FN821220
Eudicotyledons	Caryophyllaceae	*Silene*	*Silene latifolia*	FN821221
Eudicotyledons	Caryophyllaceae	*Silene*	*Silene latifolia*	FN821222
Eudicotyledons	Caryophyllaceae	*Silene*	*Silene latifolia*	FN821223
Eudicotyledons	Caryophyllaceae	*Silene*	*Silene latifolia*	FN821224
Eudicotyledons	Caryophyllaceae	*Silene*	*Silene latifolia*	FN821225
Eudicotyledons	Celastraceae	*Parnassia*	*Parnassia palustris*	JF802362
Eudicotyledons	Celastraceae	*Parnassia*	*Parnassia palustris*	JF802363
Eudicotyledons	Celastraceae	*Parnassia*	*Parnassia palustris*	JF802364
Eudicotyledons	Celastraceae	*Parnassia*	*Parnassia palustris*	JF802365
Eudicotyledons	Celastraceae	*Parnassia*	*Parnassia palustris*	JN045842
Eudicotyledons	Celastraceae	*Parnassia*	*Parnassia palustris*	JN045843
Eudicotyledons	Celastraceae	*Parnassia*	*Parnassia palustris*	JN045844
Eudicotyledons	Celastraceae	*Parnassia*	*Parnassia palustris*	JN045845
Eudicotyledons	Celastraceae	*Parnassia*	*Parnassia palustris*	JN045846
Eudicotyledons	Oleaceae	*Fraxinus*	*Fraxinus americana*	HQ596701

Eudicotyledons	Oleaceae	*Fraxinus*	*Fraxinus americana*	HM367360
Eudicotyledons	Oleaceae	*Fraxinus*	*Fraxinus americana*	HM367361
Eudicotyledons	Oleaceae	*Fraxinus*	*Fraxinus americana*	HM367362
Eudicotyledons	Oleaceae	*Fraxinus*	*Fraxinus americana*	HM367363
Eudicotyledons	Oleaceae	*Fraxinus*	*Fraxinus americana*	HM367364
Eudicotyledons	Oleaceae	*Fraxinus*	*Fraxinus excelsior*	FJ395513
Eudicotyledons	Oleaceae	*Fraxinus*	*Fraxinus excelsior*	HM367417
Eudicotyledons	Oleaceae	*Fraxinus*	*Fraxinus excelsior*	HM367418
Eudicotyledons	Oleaceae	*Fraxinus*	*Fraxinus excelsior*	HM367419
Eudicotyledons	Oleaceae	*Fraxinus*	*Fraxinus excelsior*	HM367420
Eudicotyledons	Oleaceae	*Fraxinus*	*Fraxinus excelsior*	HM367421
Eudicotyledons	Oleaceae	*Fraxinus*	*Fraxinus excelsior*	HM367422
Eudicotyledons	Oleaceae	*Fraxinus*	*Fraxinus excelsior*	HM367423
Eudicotyledons	Oleaceae	*Fraxinus*	*Fraxinus ornus*	HM367500
Eudicotyledons	Oleaceae	*Fraxinus*	*Fraxinus ornus*	HM367501
Eudicotyledons	Oleaceae	*Fraxinus*	*Fraxinus ornus*	HM367502
Eudicotyledons	Oleaceae	*Fraxinus*	*Fraxinus ornus*	HM367503
Eudicotyledons	Oleaceae	*Fraxinus*	*Fraxinus ornus*	HM367504
Eudicotyledons	Oleaceae	*Fraxinus*	*Fraxinus ornus*	HM367505
Eudicotyledons	Vitaceae	*Leea*	*Leea guineensis*	JQ182503
Eudicotyledons	Vitaceae	*Leea*	*Leea guineensis*	JF437121
Eudicotyledons	Vitaceae	*Leea*	*Leea guineensis*	JF437122
Eudicotyledons	Lamiaceae	*Ocimum*	*Ocimum basilicum*	JQ339256
Eudicotyledons	Lamiaceae	*Ocimum*	*Ocimum basilicum*	DQ667350
Eudicotyledons	Lamiaceae	*Ocimum*	*Ocimum basilicum*	FR726106
Eudicotyledons	Lamiaceae	*Ocimum*	*Ocimum basilicum*	FR726107
Eudicotyledons	Lamiaceae	*Ocimum*	*Ocimum basilicum*	FR726108
Eudicotyledons	Lamiaceae	*Ocimum*	*Ocimum basilicum*	FR726114
Eudicotyledons	Lamiaceae	*Ocimum*	*Ocimum basilicum*	FR726115
Eudicotyledons	Lamiaceae	*Ocimum*	*Ocimum basilicum*	FR726116
Eudicotyledons	Lamiaceae	*Ocimum*	*Ocimum basilicum*	FR726117
Eudicotyledons	Lamiaceae	*Ocimum*	*Ocimum basilicum*	FR726118
Eudicotyledons	Lamiaceae	*Ocimum*	*Ocimum basilicum*	FR726119
Eudicotyledons	Lamiaceae	*Ocimum*	*Ocimum basilicum*	FR726120
Eudicotyledons	Lamiaceae	*Ocimum*	*Ocimum basilicum*	FR726121
Eudicotyledons	Lamiaceae	*Ocimum*	*Ocimum basilicum*	HM590118
Eudicotyledons	Lamiaceae	*Origanum*	*Origanum vulgare*	DQ667353
Eudicotyledons	Lamiaceae	*Origanum*	*Origanum vulgare*	HQ596773
Eudicotyledons	Lamiaceae	*Origanum*	*Origanum vulgare*	FR726129
Eudicotyledons	Lamiaceae	*Origanum*	*Origanum vulgare*	FR726130
Eudicotyledons	Lamiaceae	*Origanum*	*Origanum vulgare*	FR726131
Eudicotyledons	Lamiaceae	*Origanum*	*Origanum vulgare*	FR726132
Eudicotyledons	Lamiaceae	*Origanum*	*Origanum vulgare*	FR726133
Eudicotyledons	Lamiaceae	*Origanum*	*Origanum vulgare*	FR865080
Eudicotyledons	Lamiaceae	*Origanum*	*Origanum vulgare*	FJ513100
Eudicotyledons	Lamiaceae	*Rosmarinus*	*Rosmarinus officinalis*	DQ667351
Eudicotyledons	Lamiaceae	*Rosmarinus*	*Rosmarinus officinalis*	FR726154
Eudicotyledons	Lamiaceae	*Rosmarinus*	*Rosmarinus officinalis*	FR726155
Eudicotyledons	Lamiaceae	*Rosmarinus*	*Rosmarinus officinalis*	FR726156
Eudicotyledons	Lamiaceae	*Rosmarinus*	*Rosmarinus officinalis*	FJ513141
Eudicotyledons	Caryophyllaceae	*Silene*	*Silene acaulis*	AY949839
Eudicotyledons	Caryophyllaceae	*Silene*	*Silene acaulis*	AY949840
Eudicotyledons	Caryophyllaceae	*Silene*	*Silene acaulis*	AY949841

Eudicotyledons	Caryophyllaceae	*Silene*	*Silene acaulis*	AY949842
Eudicotyledons	Caryophyllaceae	*Silene*	*Silene dioica*	FN821205
Eudicotyledons	Caryophyllaceae	*Silene*	*Silene dioica*	FN821206
Eudicotyledons	Caryophyllaceae	*Silene*	*Silene dioica*	FN821207
Eudicotyledons	Caryophyllaceae	*Silene*	*Silene dioica*	FN821208
Eudicotyledons	Caryophyllaceae	*Silene*	*Silene dioica*	FN821209
Eudicotyledons	Caryophyllaceae	*Silene*	*Silene dioica*	FN821210
Eudicotyledons	Caryophyllaceae	*Silene*	*Silene dioica*	FN821211
Eudicotyledons	Salicaceae	*Salix*	*Salix viminalis*	GU373302
Eudicotyledons	Salicaceae	*Salix*	*Salix viminalis*	GU373303
Eudicotyledons	Saxifragaceae	*Mitella*	*Mitella diphylla*	GU562388
Eudicotyledons	Saxifragaceae	*Mitella*	*Mitella diphylla*	AB492499
Eudicotyledons	Saxifragaceae	*Mitella*	*Mitella diphylla*	AB492500
Eudicotyledons	Saxifragaceae	*Mitella*	*Mitella nuda*	GU562394
Eudicotyledons	Saxifragaceae	*Mitella*	*Mitella nuda*	AB492501
Eudicotyledons	Apiaceae	*Angelica*	*Angelica archangelica*	GQ248246
Eudicotyledons	Apiaceae	*Angelica*	*Angelica archangelica*	EF590671
Eudicotyledons	Polygonaceae	*Rumex*	*Rumex acetosa*	FJ395473
Eudicotyledons	Polygonaceae	*Rumex*	*Rumex acetosa*	EU554050
Eudicotyledons	Asteraceae	*Arctotis*	*Arctotis stoechadifolia*	EU846451
Eudicotyledons	Asteraceae	*Arctotis*	*Arctotis stoechadifolia*	EU846452
Eudicotyledons	Asteraceae	*Centaurea*	*Centaurea toletana*	FJ459727
Eudicotyledons	Asteraceae	*Centaurea*	*Centaurea toletana*	FJ459728
Eudicotyledons	Asteraceae	*Centaurea*	*Centaurea toletana*	FJ459729
Eudicotyledons	Asteraceae	*Cirsium*	*Cirsium arvense*	FJ395528
Eudicotyledons	Asteraceae	*Cirsium*	*Cirsium arvense*	HQ596645
Eudicotyledons	Asteraceae	*Doniophyton*	*Doniophyton anomalum*	EU841296
Eudicotyledons	Asteraceae	*Doniophyton*	*Doniophyton anomalum*	EU841297
Eudicotyledons	Asteraceae	*Haplocarpha*	*Haplocarpha scaposa*	DQ444780
Eudicotyledons	Asteraceae	*Haplocarpha*	*Haplocarpha scaposa*	EU846396
Eudicotyledons	Asteraceae	*Santolina*	*Santolina chamaecyparissus*	JF345726
Eudicotyledons	Asteraceae	*Santolina*	*Santolina chamaecyparissus*	JF345740
Eudicotyledons	Asteraceae	*Santolina*	*Santolina chamaecyparissus*	JF345749
Eudicotyledons	Asteraceae	*Santolina*	*Santolina chamaecyparissus*	JF345757
Eudicotyledons	Asteraceae	*Santolina*	*Santolina chamaecyparissus*	JF345758
Eudicotyledons	Caryophyllaceae	*Silene*	*Silene vulgaris*	EF094072
Eudicotyledons	Caryophyllaceae	*Silene*	*Silene vulgaris*	EF094073
Eudicotyledons	Caryophyllaceae	*Silene*	*Silene vulgaris*	EF094074
Eudicotyledons	Caryophyllaceae	*Silene*	*Silene vulgaris*	EF094075
Eudicotyledons	Caryophyllaceae	*Silene*	*Silene vulgaris*	EF094076
Eudicotyledons	Caryophyllaceae	*Silene*	*Silene vulgaris*	EF094077
Eudicotyledons	Caryophyllaceae	*Silene*	*Silene vulgaris*	EF094078
Eudicotyledons	Caryophyllaceae	*Silene*	*Silene vulgaris*	EF094079
Eudicotyledons	Caryophyllaceae	*Silene*	*Silene vulgaris*	EF094080
Eudicotyledons	Caryophyllaceae	*Silene*	*Silene vulgaris*	EF094081
Eudicotyledons	Caryophyllaceae	*Silene*	*Silene vulgaris*	EF094082
Eudicotyledons	Caryophyllaceae	*Silene*	*Silene vulgaris*	EF094083
Eudicotyledons	Caryophyllaceae	*Silene*	*Silene vulgaris*	EF094084
Eudicotyledons	Caryophyllaceae	*Silene*	*Silene vulgaris*	EF094085
Eudicotyledons	Caryophyllaceae	*Silene*	*Silene vulgaris*	EF094086
Eudicotyledons	Caryophyllaceae	*Silene*	*Silene vulgaris*	EF094087
Eudicotyledons	Caryophyllaceae	*Silene*	*Silene vulgaris*	EF094088
Eudicotyledons	Caryophyllaceae	*Silene*	*Silene vulgaris*	EF094089

Eudicotyledons	Caryophyllaceae	*Silene*	*Silene vulgaris*	EF094090
Eudicotyledons	Caryophyllaceae	*Silene*	*Silene vulgaris*	EF094091
Eudicotyledons	Caryophyllaceae	*Silene*	*Silene vulgaris*	EF094092
Eudicotyledons	Caryophyllaceae	*Silene*	*Silene vulgaris*	EF094093
Eudicotyledons	Caryophyllaceae	*Silene*	*Silene vulgaris*	HE602491
Eudicotyledons	Caryophyllaceae	*Silene*	*Silene vulgaris*	AF518957
Eudicotyledons	Caryophyllaceae	*Silene*	*Silene vulgaris*	AF518958
Eudicotyledons	Caryophyllaceae	*Silene*	*Silene vulgaris*	AF518959
Eudicotyledons	Caryophyllaceae	*Silene*	*Silene vulgaris*	AF518960
Eudicotyledons	Caryophyllaceae	*Silene*	*Silene vulgaris*	AF518961
Eudicotyledons	Caryophyllaceae	*Silene*	*Silene vulgaris*	AF518962
Eudicotyledons	Caryophyllaceae	*Silene*	*Silene vulgaris*	AF518963
Eudicotyledons	Caryophyllaceae	*Silene*	*Silene vulgaris*	AF518964
Eudicotyledons	Caryophyllaceae	*Silene*	*Silene vulgaris*	AF518965
Eudicotyledons	Caryophyllaceae	*Silene*	*Silene vulgaris*	AF518966
Eudicotyledons	Caryophyllaceae	*Silene*	*Silene vulgaris*	AF518967
Eudicotyledons	Caryophyllaceae	*Silene*	*Silene vulgaris*	AF518968
Eudicotyledons	Caryophyllaceae	*Silene*	*Silene vulgaris*	AF518969
Eudicotyledons	Caryophyllaceae	*Silene*	*Silene vulgaris*	AF518970
Eudicotyledons	Caryophyllaceae	*Silene*	*Silene vulgaris*	AF518971
Eudicotyledons	Caryophyllaceae	*Silene*	*Silene vulgaris*	AF518972
Eudicotyledons	Caryophyllaceae	*Silene*	*Silene vulgaris*	AF518973
Eudicotyledons	Caryophyllaceae	*Silene*	*Silene vulgaris*	AF518974
Eudicotyledons	Caryophyllaceae	*Silene*	*Silene vulgaris*	AF518975
Eudicotyledons	Caryophyllaceae	*Silene*	*Silene vulgaris*	AF518976
Eudicotyledons	Caryophyllaceae	*Silene*	*Silene vulgaris*	AF518977
Eudicotyledons	Caryophyllaceae	*Silene*	*Silene vulgaris*	AF518978
Eudicotyledons	Caryophyllaceae	*Silene*	*Silene vulgaris*	AF518979
Eudicotyledons	Caryophyllaceae	*Silene*	*Silene vulgaris*	AF518980
Eudicotyledons	Caryophyllaceae	*Silene*	*Silene vulgaris*	AF518981
Eudicotyledons	Caryophyllaceae	*Silene*	*Silene vulgaris*	AF518982
Eudicotyledons	Caryophyllaceae	*Silene*	*Silene vulgaris*	AF518983
Eudicotyledons	Caryophyllaceae	*Silene*	*Silene vulgaris*	AF518984
Eudicotyledons	Caryophyllaceae	*Silene*	*Silene vulgaris*	AY629274
Eudicotyledons	Caryophyllaceae	*Silene*	*Silene vulgaris*	AY629275
Eudicotyledons	Caryophyllaceae	*Silene*	*Silene vulgaris*	AY629276
Eudicotyledons	Caryophyllaceae	*Silene*	*Silene vulgaris*	AY629277
Eudicotyledons	Caryophyllaceae	*Silene*	*Silene vulgaris*	AY629278
Eudicotyledons	Caryophyllaceae	*Silene*	*Silene vulgaris*	AY629279
Eudicotyledons	Caryophyllaceae	*Silene*	*Silene vulgaris*	AY629280
Eudicotyledons	Caryophyllaceae	*Silene*	*Silene vulgaris*	AY629281
Eudicotyledons	Caryophyllaceae	*Silene*	*Silene vulgaris*	AY629282
Eudicotyledons	Caryophyllaceae	*Silene*	*Silene vulgaris*	AY629283
Eudicotyledons	Caryophyllaceae	*Silene*	*Silene vulgaris*	AY629284
Eudicotyledons	Caryophyllaceae	*Silene*	*Silene vulgaris*	AY629285
Eudicotyledons	Caryophyllaceae	*Silene*	*Silene vulgaris*	AY629286
Eudicotyledons	Caryophyllaceae	*Silene*	*Silene vulgaris*	AY629287
Eudicotyledons	Caryophyllaceae	*Silene*	*Silene vulgaris*	AY629288
Eudicotyledons	Caryophyllaceae	*Silene*	*Silene vulgaris*	AY629289
Eudicotyledons	Caryophyllaceae	*Silene*	*Silene vulgaris*	AY629290
Eudicotyledons	Caryophyllaceae	*Silene*	*Silene vulgaris*	AY629291
Eudicotyledons	Caryophyllaceae	*Silene*	*Silene vulgaris*	AY629292
Eudicotyledons	Caryophyllaceae	*Silene*	*Silene vulgaris*	AY629294

Eudicotyledons	Caryophyllaceae	*Silene*	*Silene vulgaris*	AY629295
Eudicotyledons	Caryophyllaceae	*Silene*	*Silene vulgaris*	AY629296
Eudicotyledons	Caryophyllaceae	*Silene*	*Silene vulgaris*	AY629297
Eudicotyledons	Caryophyllaceae	*Silene*	*Silene vulgaris*	EU750548
Eudicotyledons	Caryophyllaceae	*Silene*	*Silene vulgaris*	EU750549
Eudicotyledons	Caryophyllaceae	*Silene*	*Silene vulgaris*	EU750550
Eudicotyledons	Caryophyllaceae	*Silene*	*Silene vulgaris*	EU805570
Eudicotyledons	Caryophyllaceae	*Silene*	*Silene vulgaris*	EU805571
Eudicotyledons	Caryophyllaceae	*Silene*	*Silene vulgaris*	EU805572
Eudicotyledons	Caryophyllaceae	*Silene*	*Silene vulgaris*	EU805573
Eudicotyledons	Caryophyllaceae	*Silene*	*Silene vulgaris*	EU805574
Eudicotyledons	Rosaceae	*Prunus*	*Prunus avium*	FJ395551
Eudicotyledons	Rosaceae	*Prunus*	*Prunus avium*	FN675831
Eudicotyledons	Salicaceae	*Populus*	*Populus alba*	HE659540
Eudicotyledons	Salicaceae	*Populus*	*Populus alba*	GQ248375
Eudicotyledons	Rubiaceae	*Oldenlandia*	*Oldenlandia corymbosa*	JN044771
Eudicotyledons	Rubiaceae	*Oldenlandia*	*Oldenlandia corymbosa*	JN044772
Eudicotyledons	Nitrariaceae	*Peganum*	*Peganum harmala*	FJ752656
Eudicotyledons	Nitrariaceae	*Peganum*	*Peganum harmala*	FJ752657
Eudicotyledons	Nitrariaceae	*Peganum*	*Peganum harmala*	FJ752661
Eudicotyledons	Nitrariaceae	*Peganum*	*Peganum harmala*	FJ752664
Eudicotyledons	Nitrariaceae	*Peganum*	*Peganum harmala*	FJ752666
Eudicotyledons	Meliaceae	*Cipadessa*	*Cipadessa baccifera*	JN044299
Eudicotyledons	Meliaceae	*Cipadessa*	*Cipadessa baccifera*	JN044300
Eudicotyledons	Meliaceae	*Cipadessa*	*Cipadessa baccifera*	JN044301
Eudicotyledons	Meliaceae	*Cipadessa*	*Cipadessa baccifera*	JN044302
Eudicotyledons	Meliaceae	*Cipadessa*	*Cipadessa baccifera*	JN044303
Eudicotyledons	Araliaceae	*Panax*	*Panax notoginseng*	GQ435399
Eudicotyledons	Araliaceae	*Panax*	*Panax notoginseng*	GQ435400
Eudicotyledons	Araliaceae	*Panax*	*Panax notoginseng*	GQ435401
Eudicotyledons	Araliaceae	*Panax*	*Panax notoginseng*	HQ112882
Eudicotyledons	Araliaceae	*Panax*	*Panax notoginseng*	HQ112883
Eudicotyledons	Araliaceae	*Panax*	*Panax notoginseng*	HQ112884
Eudicotyledons	Araliaceae	*Panax*	*Panax quinquefolius*	GQ435403
Eudicotyledons	Araliaceae	*Panax*	*Panax quinquefolius*	HQ112887
Eudicotyledons	Araliaceae	*Panax*	*Panax quinquefolius*	HQ112888
Eudicotyledons	Araliaceae	*Panax*	*Panax pseudoginseng*	HQ112885
Eudicotyledons	Araliaceae	*Panax*	*Panax pseudoginseng*	HQ112886
Eudicotyledons	Araliaceae	*Panax*	*Panax stipuleanatus*	HQ112889
Eudicotyledons	Araliaceae	*Panax*	*Panax stipuleanatus*	HQ112890
Eudicotyledons	Araliaceae	*Panax*	*Panax stipuleanatus*	HQ112891
Eudicotyledons	Araliaceae	*Panax*	*Panax stipuleanatus*	HQ112892
Eudicotyledons	Araliaceae	*Panax*	*Panax trifolius*	GU054891
Eudicotyledons	Araliaceae	*Panax*	*Panax trifolius*	HQ112893
Eudicotyledons	Araliaceae	*Panax*	*Panax trifolius*	HQ112894
Eudicotyledons	Araliaceae	*Panax*	*Panax japonicus*	GQ435405
Eudicotyledons	Araliaceae	*Panax*	*Panax japonicus*	GQ435406
Eudicotyledons	Araliaceae	*Panax*	*Panax japonicus*	GQ435407
Eudicotyledons	Araliaceae	*Panax*	*Panax japonicus*	HQ112873
Eudicotyledons	Araliaceae	*Panax*	*Panax japonicus*	HQ112874
Eudicotyledons	Araliaceae	*Panax*	*Panax japonicus*	HQ112875
Eudicotyledons	Araliaceae	*Panax*	*Panax japonicus*	HQ112876
Eudicotyledons	Araliaceae	*Panax*	*Panax japonicus*	HQ112877

Eudicotyledons	Araliaceae	*Panax*	*Panax japonicus*	HQ112878
Eudicotyledons	Araliaceae	*Panax*	*Panax japonicus*	HQ112879
Eudicotyledons	Araliaceae	*Panax*	*Panax japonicus*	HQ112880
Eudicotyledons	Araliaceae	*Panax*	*Panax japonicus*	HQ112881
Eudicotyledons	Aceraceae	*Acer*	*Acer rubrum*	DQ978624
Eudicotyledons	Aceraceae	*Acer*	*Acer rubrum*	DQ528758
Eudicotyledons	Aceraceae	*Acer*	*Acer rubrum*	DQ528759
Eudicotyledons	Aceraceae	*Acer*	*Acer rubrum*	HQ266067
Eudicotyledons	Aceraceae	*Acer*	*Acer rubrum*	HQ266068
Eudicotyledons	Aceraceae	*Acer*	*Acer rubrum*	HQ266069
Eudicotyledons	Aceraceae	*Acer*	*Acer rubrum*	HQ266070
Eudicotyledons	Aceraceae	*Acer*	*Acer rubrum*	HQ266071
Eudicotyledons	Aceraceae	*Acer*	*Acer rubrum*	HQ266072
Eudicotyledons	Aceraceae	*Acer*	*Acer rubrum*	HQ266073
Eudicotyledons	Aceraceae	*Acer*	*Acer rubrum*	HQ266074
Eudicotyledons	Aceraceae	*Acer*	*Acer rubrum*	HQ266075
Eudicotyledons	Aceraceae	*Acer*	*Acer rubrum*	HQ266076
Eudicotyledons	Aceraceae	*Acer*	*Acer rubrum*	HQ266077
Eudicotyledons	Aceraceae	*Acer*	*Acer rubrum*	HQ266078
Eudicotyledons	Aceraceae	*Acer*	*Acer rubrum*	HQ266079
Eudicotyledons	Aceraceae	*Acer*	*Acer rubrum*	HQ266080
Eudicotyledons	Aceraceae	*Acer*	*Acer rubrum*	HQ266081
Eudicotyledons	Aceraceae	*Acer*	*Acer rubrum*	HQ266082
Eudicotyledons	Aceraceae	*Acer*	*Acer rubrum*	HQ266083
Eudicotyledons	Aceraceae	*Acer*	*Acer rubrum*	HQ266084
Eudicotyledons	Aceraceae	*Acer*	*Acer rubrum*	HQ266085
Eudicotyledons	Aceraceae	*Acer*	*Acer rubrum*	HQ266086
Eudicotyledons	Aceraceae	*Acer*	*Acer rubrum*	HQ266087
Eudicotyledons	Aceraceae	*Acer*	*Acer rubrum*	HQ266088
Eudicotyledons	Aceraceae	*Acer*	*Acer rubrum*	HQ266089
Eudicotyledons	Aceraceae	*Acer*	*Acer rubrum*	HQ266090
Eudicotyledons	Aceraceae	*Acer*	*Acer rubrum*	HQ266091
Eudicotyledons	Aceraceae	*Acer*	*Acer rubrum*	HQ266092
Eudicotyledons	Aceraceae	*Acer*	*Acer rubrum*	HQ266093
Eudicotyledons	Aceraceae	*Acer*	*Acer rubrum*	HQ266094
Eudicotyledons	Aceraceae	*Acer*	*Acer rubrum*	HQ266095
Eudicotyledons	Aceraceae	*Acer*	*Acer rubrum*	HQ266096
Eudicotyledons	Aceraceae	*Acer*	*Acer rubrum*	HQ266097
Eudicotyledons	Aceraceae	*Acer*	*Acer rubrum*	HQ266098
Eudicotyledons	Aceraceae	*Acer*	*Acer rubrum*	HQ266099
Eudicotyledons	Aceraceae	*Acer*	*Acer rubrum*	HQ266100
Eudicotyledons	Aceraceae	*Acer*	*Acer rubrum*	HQ266101
Eudicotyledons	Aceraceae	*Acer*	*Acer rubrum*	HQ266102
Eudicotyledons	Aceraceae	*Acer*	*Acer rubrum*	HQ266103
Eudicotyledons	Aceraceae	*Acer*	*Acer rubrum*	HQ266104
Eudicotyledons	Aceraceae	*Acer*	*Acer rubrum*	HQ266105
Eudicotyledons	Aceraceae	*Acer*	*Acer rubrum*	HQ266106
Eudicotyledons	Aceraceae	*Acer*	*Acer rubrum*	HQ266107
Eudicotyledons	Aceraceae	*Acer*	*Acer rubrum*	HQ266108
Eudicotyledons	Aceraceae	*Acer*	*Acer rubrum*	HQ266109
Eudicotyledons	Aceraceae	*Acer*	*Acer rubrum*	HQ266110
Eudicotyledons	Aceraceae	*Acer*	*Acer rubrum*	HQ266111
Eudicotyledons	Aceraceae	*Acer*	*Acer rubrum*	HQ266112

Eudicotyledons	Aceraceae	*Acer*	*Acer rubrum*	HQ266113
Eudicotyledons	Aceraceae	*Acer*	*Acer rubrum*	HQ266114
Eudicotyledons	Aceraceae	*Acer*	*Acer rubrum*	HQ266115
Eudicotyledons	Aceraceae	*Acer*	*Acer rubrum*	HQ266116
Eudicotyledons	Aceraceae	*Acer*	*Acer rubrum*	HQ266117
Eudicotyledons	Aceraceae	*Acer*	*Acer rubrum*	HQ266118
Eudicotyledons	Aceraceae	*Acer*	*Acer rubrum*	HQ266119
Eudicotyledons	Aceraceae	*Acer*	*Acer rubrum*	HQ266120
Eudicotyledons	Aceraceae	*Acer*	*Acer rubrum*	HQ266121
Eudicotyledons	Aceraceae	*Acer*	*Acer rubrum*	HQ266122
Eudicotyledons	Aceraceae	*Acer*	*Acer rubrum*	HQ266123
Eudicotyledons	Aceraceae	*Acer*	*Acer rubrum*	HQ266124
Eudicotyledons	Aceraceae	*Acer*	*Acer rubrum*	HQ266125
Eudicotyledons	Aceraceae	*Acer*	*Acer rubrum*	HQ266126
Eudicotyledons	Aceraceae	*Acer*	*Acer rubrum*	HQ266127
Eudicotyledons	Aceraceae	*Acer*	*Acer rubrum*	HQ266128
Eudicotyledons	Aceraceae	*Acer*	*Acer rubrum*	HQ266129
Eudicotyledons	Aceraceae	*Acer*	*Acer rubrum*	HQ266130
Eudicotyledons	Aceraceae	*Acer*	*Acer rubrum*	HQ266131
Eudicotyledons	Aceraceae	*Acer*	*Acer rubrum*	HQ266132
Eudicotyledons	Aceraceae	*Acer*	*Acer rubrum*	HQ266133
Eudicotyledons	Aceraceae	*Acer*	*Acer rubrum*	HQ266134
Eudicotyledons	Aceraceae	*Acer*	*Acer rubrum*	HQ266135
Eudicotyledons	Aceraceae	*Acer*	*Acer rubrum*	HQ266136
Eudicotyledons	Aceraceae	*Acer*	*Acer rubrum*	HQ266137
Eudicotyledons	Aceraceae	*Acer*	*Acer rubrum*	HQ266138
Eudicotyledons	Aceraceae	*Acer*	*Acer rubrum*	HQ266139
Eudicotyledons	Aceraceae	*Acer*	*Acer rubrum*	HQ266140
Eudicotyledons	Aceraceae	*Acer*	*Acer rubrum*	HQ266141
Eudicotyledons	Aceraceae	*Acer*	*Acer rubrum*	HQ266142
Eudicotyledons	Aceraceae	*Acer*	*Acer rubrum*	HQ266143
Eudicotyledons	Aceraceae	*Acer*	*Acer rubrum*	HQ266144
Eudicotyledons	Aceraceae	*Acer*	*Acer rubrum*	HQ266145
Eudicotyledons	Aceraceae	*Acer*	*Acer rubrum*	HQ266146
Eudicotyledons	Aceraceae	*Acer*	*Acer rubrum*	HQ266147
Eudicotyledons	Aceraceae	*Acer*	*Acer rubrum*	HQ266148
Eudicotyledons	Aceraceae	*Acer*	*Acer rubrum*	HQ266149
Eudicotyledons	Aceraceae	*Acer*	*Acer rubrum*	HQ266150
Eudicotyledons	Aceraceae	*Acer*	*Acer rubrum*	HQ266151
Eudicotyledons	Aceraceae	*Acer*	*Acer rubrum*	HQ266152
Eudicotyledons	Aceraceae	*Acer*	*Acer rubrum*	HQ266153
Eudicotyledons	Aceraceae	*Acer*	*Acer rubrum*	HQ266154
Eudicotyledons	Aceraceae	*Acer*	*Acer rubrum*	HQ266155
Eudicotyledons	Aceraceae	*Acer*	*Acer rubrum*	HQ266156
Eudicotyledons	Aceraceae	*Acer*	*Acer rubrum*	HQ266157
Eudicotyledons	Aceraceae	*Acer*	*Acer rubrum*	HQ266158
Eudicotyledons	Aceraceae	*Acer*	*Acer rubrum*	HQ266159
Eudicotyledons	Aceraceae	*Acer*	*Acer rubrum*	HQ266160
Eudicotyledons	Aceraceae	*Acer*	*Acer rubrum*	HQ266161
Eudicotyledons	Aceraceae	*Acer*	*Acer rubrum*	HQ266162
Eudicotyledons	Aceraceae	*Acer*	*Acer rubrum*	HQ266163
Eudicotyledons	Aceraceae	*Acer*	*Acer rubrum*	HQ266164
Eudicotyledons	Aceraceae	*Acer*	*Acer rubrum*	HQ266165

Eudicotyledons	Aceraceae	*Acer*	*Acer rubrum*	HQ266166
Eudicotyledons	Aceraceae	*Acer*	*Acer rubrum*	HQ266167
Eudicotyledons	Aceraceae	*Acer*	*Acer rubrum*	HQ266168
Eudicotyledons	Aceraceae	*Acer*	*Acer rubrum*	HQ266169
Eudicotyledons	Aceraceae	*Acer*	*Acer rubrum*	HQ266170
Eudicotyledons	Aceraceae	*Acer*	*Acer rubrum*	HQ266171
Eudicotyledons	Aceraceae	*Acer*	*Acer rubrum*	HQ266172
Eudicotyledons	Aceraceae	*Acer*	*Acer rubrum*	HQ266173
Eudicotyledons	Aceraceae	*Acer*	*Acer rubrum*	HQ266174
Eudicotyledons	Aceraceae	*Acer*	*Acer rubrum*	HQ266175
Eudicotyledons	Aceraceae	*Acer*	*Acer rubrum*	HQ266176
Eudicotyledons	Aceraceae	*Acer*	*Acer rubrum*	HQ266177
Eudicotyledons	Aceraceae	*Acer*	*Acer rubrum*	HQ266178
Eudicotyledons	Aceraceae	*Acer*	*Acer rubrum*	HQ266179
Eudicotyledons	Aceraceae	*Acer*	*Acer rubrum*	HQ266180
Eudicotyledons	Aceraceae	*Acer*	*Acer rubrum*	HQ266181
Eudicotyledons	Aceraceae	*Acer*	*Acer rubrum*	HQ266182
Eudicotyledons	Aceraceae	*Acer*	*Acer rubrum*	HQ266183
Eudicotyledons	Aceraceae	*Acer*	*Acer rubrum*	HQ266184
Eudicotyledons	Aceraceae	*Acer*	*Acer rubrum*	HQ266185
Eudicotyledons	Aceraceae	*Acer*	*Acer rubrum*	HQ266186
Eudicotyledons	Aceraceae	*Acer*	*Acer rubrum*	HQ266187
Eudicotyledons	Aceraceae	*Acer*	*Acer rubrum*	HQ266188
Eudicotyledons	Aceraceae	*Acer*	*Acer rubrum*	HQ266189
Eudicotyledons	Aceraceae	*Acer*	*Acer rubrum*	HQ266190
Eudicotyledons	Aceraceae	*Acer*	*Acer rubrum*	HQ266191
Eudicotyledons	Aceraceae	*Acer*	*Acer rubrum*	HQ266192
Eudicotyledons	Aceraceae	*Acer*	*Acer rubrum*	HQ266193
Eudicotyledons	Aceraceae	*Acer*	*Acer rubrum*	HQ266194
Eudicotyledons	Aceraceae	*Acer*	*Acer rubrum*	HQ266195
Eudicotyledons	Aceraceae	*Acer*	*Acer rubrum*	HQ266196
Eudicotyledons	Aceraceae	*Acer*	*Acer rubrum*	HQ266197
Eudicotyledons	Aceraceae	*Acer*	*Acer rubrum*	HQ266198
Eudicotyledons	Aceraceae	*Acer*	*Acer rubrum*	HQ266199
Eudicotyledons	Aceraceae	*Acer*	*Acer rubrum*	HQ266200
Eudicotyledons	Aceraceae	*Acer*	*Acer rubrum*	HQ266201
Eudicotyledons	Aceraceae	*Acer*	*Acer rubrum*	HQ266202
Eudicotyledons	Aceraceae	*Acer*	*Acer rubrum*	HQ266203
Eudicotyledons	Aceraceae	*Acer*	*Acer rubrum*	HQ266204
Eudicotyledons	Aceraceae	*Acer*	*Acer rubrum*	HQ266205
Eudicotyledons	Aceraceae	*Acer*	*Acer rubrum*	HQ266206
Eudicotyledons	Aceraceae	*Acer*	*Acer rubrum*	HQ266207
Eudicotyledons	Aceraceae	*Acer*	*Acer rubrum*	HQ266208
Eudicotyledons	Aceraceae	*Acer*	*Acer rubrum*	HQ266209
Eudicotyledons	Aceraceae	*Acer*	*Acer rubrum*	HQ266210
Eudicotyledons	Aceraceae	*Acer*	*Acer rubrum*	HQ266211
Eudicotyledons	Aceraceae	*Acer*	*Acer rubrum*	HQ266212
Eudicotyledons	Aceraceae	*Acer*	*Acer rubrum*	HQ266213
Eudicotyledons	Aceraceae	*Acer*	*Acer rubrum*	HQ266214
Eudicotyledons	Aceraceae	*Acer*	*Acer rubrum*	HQ266215
Eudicotyledons	Aceraceae	*Acer*	*Acer rubrum*	HQ266216
Eudicotyledons	Aceraceae	*Acer*	*Acer rubrum*	HQ266217
Eudicotyledons	Aceraceae	*Acer*	*Acer rubrum*	HQ266218

Eudicotyledons	Aceraceae	*Acer*	*Acer rubrum*	HQ266219
Eudicotyledons	Aceraceae	*Acer*	*Acer rubrum*	HQ266220
Eudicotyledons	Aceraceae	*Acer*	*Acer rubrum*	HQ266221
Eudicotyledons	Aceraceae	*Acer*	*Acer rubrum*	HQ266222
Eudicotyledons	Aceraceae	*Acer*	*Acer rubrum*	HQ266223
Eudicotyledons	Aceraceae	*Acer*	*Acer rubrum*	HQ266224
Eudicotyledons	Aceraceae	*Acer*	*Acer rubrum*	HQ266225
Eudicotyledons	Aceraceae	*Acer*	*Acer rubrum*	HQ266226
Eudicotyledons	Aceraceae	*Acer*	*Acer rubrum*	HQ266227
Eudicotyledons	Aceraceae	*Acer*	*Acer rubrum*	HQ266228
Eudicotyledons	Aceraceae	*Acer*	*Acer rubrum*	HQ266229
Eudicotyledons	Aceraceae	*Acer*	*Acer rubrum*	HQ266230
Eudicotyledons	Aceraceae	*Acer*	*Acer rubrum*	HQ266231
Eudicotyledons	Aceraceae	*Acer*	*Acer rubrum*	HQ266232
Eudicotyledons	Aceraceae	*Acer*	*Acer rubrum*	HQ266233
Eudicotyledons	Aceraceae	*Acer*	*Acer rubrum*	HQ266234
Eudicotyledons	Aceraceae	*Acer*	*Acer rubrum*	HQ266235
Eudicotyledons	Aceraceae	*Acer*	*Acer rubrum*	HQ266236
Eudicotyledons	Aceraceae	*Acer*	*Acer rubrum*	HQ266237
Eudicotyledons	Aceraceae	*Acer*	*Acer rubrum*	HQ266238
Eudicotyledons	Aceraceae	*Acer*	*Acer rubrum*	HQ266239
Eudicotyledons	Aceraceae	*Acer*	*Acer rubrum*	HQ266240
Eudicotyledons	Aceraceae	*Acer*	*Acer rubrum*	HQ266241
Eudicotyledons	Aceraceae	*Acer*	*Acer rubrum*	HQ266242
Eudicotyledons	Aceraceae	*Acer*	*Acer rubrum*	HQ266243
Eudicotyledons	Aceraceae	*Acer*	*Acer rubrum*	HQ266244
Eudicotyledons	Aceraceae	*Acer*	*Acer rubrum*	HQ266245
Eudicotyledons	Aceraceae	*Acer*	*Acer rubrum*	HQ266246
Eudicotyledons	Aceraceae	*Acer*	*Acer rubrum*	HQ266247
Eudicotyledons	Aceraceae	*Acer*	*Acer rubrum*	HQ266248
Eudicotyledons	Aceraceae	*Acer*	*Acer rubrum*	HQ266249
Eudicotyledons	Aceraceae	*Acer*	*Acer rubrum*	HQ266250
Eudicotyledons	Aceraceae	*Acer*	*Acer rubrum*	HQ266251
Eudicotyledons	Aceraceae	*Acer*	*Acer rubrum*	HQ266252
Eudicotyledons	Aceraceae	*Acer*	*Acer rubrum*	HQ266253
Eudicotyledons	Aceraceae	*Acer*	*Acer rubrum*	HQ266254
Eudicotyledons	Aceraceae	*Acer*	*Acer rubrum*	HQ266255
Eudicotyledons	Aceraceae	*Acer*	*Acer rubrum*	HQ266256
Eudicotyledons	Aceraceae	*Acer*	*Acer rubrum*	HQ266257
Eudicotyledons	Aceraceae	*Acer*	*Acer rubrum*	HQ266258
Eudicotyledons	Aceraceae	*Acer*	*Acer rubrum*	HQ266259
Eudicotyledons	Aceraceae	*Acer*	*Acer rubrum*	HQ266260
Eudicotyledons	Aceraceae	*Acer*	*Acer rubrum*	HQ266261
Eudicotyledons	Aceraceae	*Acer*	*Acer rubrum*	HQ266262
Eudicotyledons	Aceraceae	*Acer*	*Acer rubrum*	HQ266263
Eudicotyledons	Aceraceae	*Acer*	*Acer rubrum*	HQ266264
Eudicotyledons	Aceraceae	*Acer*	*Acer rubrum*	HQ266265
Eudicotyledons	Aceraceae	*Acer*	*Acer rubrum*	HQ266266
Eudicotyledons	Aceraceae	*Acer*	*Acer rubrum*	HQ266267
Eudicotyledons	Aceraceae	*Acer*	*Acer rubrum*	HQ266268
Eudicotyledons	Aceraceae	*Acer*	*Acer rubrum*	HQ266269
Eudicotyledons	Aceraceae	*Acer*	*Acer rubrum*	HQ266270
Eudicotyledons	Aceraceae	*Acer*	*Acer rubrum*	HQ266271

Eudicotyledons	Aceraceae	*Acer*	*Acer rubrum*	HQ266272
Eudicotyledons	Aceraceae	*Acer*	*Acer rubrum*	HQ266273
Eudicotyledons	Aceraceae	*Acer*	*Acer rubrum*	HQ266274
Eudicotyledons	Aceraceae	*Acer*	*Acer rubrum*	HQ266275
Eudicotyledons	Aceraceae	*Acer*	*Acer rubrum*	HQ266276
Eudicotyledons	Aceraceae	*Acer*	*Acer rubrum*	HQ266277
Eudicotyledons	Aceraceae	*Acer*	*Acer rubrum*	HQ266278
Eudicotyledons	Aceraceae	*Acer*	*Acer rubrum*	HQ266279
Eudicotyledons	Aceraceae	*Acer*	*Acer rubrum*	HQ266280
Eudicotyledons	Aceraceae	*Acer*	*Acer rubrum*	HQ266281
Eudicotyledons	Aceraceae	*Acer*	*Acer rubrum*	HQ266282
Eudicotyledons	Aceraceae	*Acer*	*Acer rubrum*	HQ266283
Eudicotyledons	Aceraceae	*Acer*	*Acer rubrum*	HQ266284
Eudicotyledons	Aceraceae	*Acer*	*Acer rubrum*	HQ266285
Eudicotyledons	Aceraceae	*Acer*	*Acer rubrum*	HQ266286
Eudicotyledons	Aceraceae	*Acer*	*Acer rubrum*	HQ266287
Eudicotyledons	Aceraceae	*Acer*	*Acer rubrum*	HQ266288
Eudicotyledons	Aceraceae	*Acer*	*Acer rubrum*	HQ266289
Eudicotyledons	Aceraceae	*Acer*	*Acer rubrum*	HQ266290
Eudicotyledons	Aceraceae	*Acer*	*Acer rubrum*	HQ266291
Eudicotyledons	Aceraceae	*Acer*	*Acer rubrum*	HQ266292
Eudicotyledons	Aceraceae	*Acer*	*Acer rubrum*	HQ266293
Eudicotyledons	Aceraceae	*Acer*	*Acer rubrum*	HQ266294
Eudicotyledons	Aceraceae	*Acer*	*Acer rubrum*	HQ266295
Eudicotyledons	Aceraceae	*Acer*	*Acer rubrum*	HQ266296
Eudicotyledons	Aceraceae	*Acer*	*Acer rubrum*	HQ266297
Eudicotyledons	Aceraceae	*Acer*	*Acer rubrum*	HQ266298
Eudicotyledons	Aceraceae	*Acer*	*Acer rubrum*	HQ266299
Eudicotyledons	Aceraceae	*Acer*	*Acer rubrum*	HQ266300
Eudicotyledons	Aceraceae	*Acer*	*Acer rubrum*	HQ266301
Eudicotyledons	Aceraceae	*Acer*	*Acer rubrum*	HQ266302
Eudicotyledons	Aceraceae	*Acer*	*Acer rubrum*	HQ266303
Eudicotyledons	Aceraceae	*Acer*	*Acer rubrum*	HQ266304
Eudicotyledons	Aceraceae	*Acer*	*Acer rubrum*	HQ266305
Eudicotyledons	Aceraceae	*Acer*	*Acer rubrum*	HQ266306
Eudicotyledons	Aceraceae	*Acer*	*Acer rubrum*	HQ266307
Eudicotyledons	Aceraceae	*Acer*	*Acer rubrum*	HQ266308
Eudicotyledons	Aceraceae	*Acer*	*Acer rubrum*	HQ266309
Eudicotyledons	Aceraceae	*Acer*	*Acer rubrum*	HQ266310
Eudicotyledons	Aceraceae	*Acer*	*Acer rubrum*	HQ266311
Eudicotyledons	Aceraceae	*Acer*	*Acer rubrum*	HQ266312
Eudicotyledons	Aceraceae	*Acer*	*Acer rubrum*	HQ266313
Eudicotyledons	Aceraceae	*Acer*	*Acer rubrum*	HQ266314
Eudicotyledons	Aceraceae	*Acer*	*Acer rubrum*	HQ266315
Eudicotyledons	Aceraceae	*Acer*	*Acer rubrum*	HQ266316
Eudicotyledons	Aceraceae	*Acer*	*Acer rubrum*	HQ266317
Eudicotyledons	Aceraceae	*Acer*	*Acer rubrum*	HQ266318
Eudicotyledons	Aceraceae	*Acer*	*Acer rubrum*	HQ266319
Eudicotyledons	Aceraceae	*Acer*	*Acer rubrum*	HQ266320
Eudicotyledons	Aceraceae	*Acer*	*Acer rubrum*	HQ266321
Eudicotyledons	Aceraceae	*Acer*	*Acer rubrum*	HQ266322
Eudicotyledons	Aceraceae	*Acer*	*Acer rubrum*	HQ266323
Eudicotyledons	Aceraceae	*Acer*	*Acer rubrum*	HQ266324

Eudicotyledons	Aceraceae	*Acer*	*Acer rubrum*	AY853842
Eudicotyledons	Aceraceae	*Acer*	*Acer rubrum*	AY853843
Eudicotyledons	Aceraceae	*Acer*	*Acer rubrum*	AY853844
Eudicotyledons	Aceraceae	*Acer*	*Acer rubrum*	AY853845
Eudicotyledons	Aceraceae	*Acer*	*Acer rubrum*	AY853846
Eudicotyledons	Aceraceae	*Acer*	*Acer rubrum*	AY853847
Eudicotyledons	Aceraceae	*Acer*	*Acer rubrum*	AY853848
Eudicotyledons	Aceraceae	*Acer*	*Acer rubrum*	AY853849
Eudicotyledons	Aceraceae	*Acer*	*Acer rubrum*	AY853850
Eudicotyledons	Aceraceae	*Acer*	*Acer rubrum*	AY853851
Eudicotyledons	Aceraceae	*Acer*	*Acer rubrum*	AY853852
Eudicotyledons	Aceraceae	*Acer*	*Acer rubrum*	AY853853
Eudicotyledons	Aceraceae	*Acer*	*Acer rubrum*	AY853854
Eudicotyledons	Aceraceae	*Acer*	*Acer rubrum*	AY853855
Eudicotyledons	Aceraceae	*Acer*	*Acer rubrum*	AY853856
Eudicotyledons	Aceraceae	*Acer*	*Acer rubrum*	AY853857
Eudicotyledons	Aceraceae	*Acer*	*Acer rubrum*	AY853858
Eudicotyledons	Aceraceae	*Acer*	*Acer rubrum*	AY853859
Eudicotyledons	Aceraceae	*Acer*	*Acer rubrum*	AY853860
Eudicotyledons	Aceraceae	*Acer*	*Acer rubrum*	AY853861
Eudicotyledons	Aceraceae	*Acer*	*Acer rubrum*	AY853862
Eudicotyledons	Aceraceae	*Acer*	*Acer rubrum*	AY853863
Eudicotyledons	Aceraceae	*Acer*	*Acer rubrum*	AY853864
Eudicotyledons	Aceraceae	*Acer*	*Acer rubrum*	AY853865
Eudicotyledons	Aceraceae	*Acer*	*Acer rubrum*	AY853866
Eudicotyledons	Aceraceae	*Acer*	*Acer rubrum*	AY853867
Eudicotyledons	Aceraceae	*Acer*	*Acer rubrum*	AY853868
Eudicotyledons	Aceraceae	*Acer*	*Acer rubrum*	AY853869
Eudicotyledons	Aceraceae	*Acer*	*Acer rubrum*	AY853870
Eudicotyledons	Aceraceae	*Acer*	*Acer rubrum*	AY853871
Eudicotyledons	Aceraceae	*Acer*	*Acer rubrum*	AY853872
Eudicotyledons	Aceraceae	*Acer*	*Acer rubrum*	AY853873
Eudicotyledons	Aceraceae	*Acer*	*Acer rubrum*	AY853874
Eudicotyledons	Aceraceae	*Acer*	*Acer rubrum*	AY853875
Eudicotyledons	Aceraceae	*Acer*	*Acer rubrum*	AY853876
Eudicotyledons	Aceraceae	*Acer*	*Acer rubrum*	AY853877
Eudicotyledons	Aceraceae	*Acer*	*Acer rubrum*	AY853878
Eudicotyledons	Aceraceae	*Acer*	*Acer rubrum*	EU750431
Eudicotyledons	Aceraceae	*Acer*	*Acer rubrum*	EU750432
Eudicotyledons	Aceraceae	*Acer*	*Acer rubrum*	EU750433
Eudicotyledons	Solanaceae	*Solanum*	*Solanum dulcamara*	FJ395554
Eudicotyledons	Solanaceae	*Solanum*	*Solanum dulcamara*	HQ596850
Eudicotyledons	Solanaceae	*Solanum*	*Solanum dulcamara*	EU750551
Eudicotyledons	Solanaceae	*Solanum*	*Solanum dulcamara*	EU750552
Eudicotyledons	Solanaceae	*Solanum*	*Solanum dulcamara*	EU750553
Eudicotyledons	Solanaceae	*Solanum*	*Solanum dulcamara*	FN675825
Eudicotyledons	Araliaceae	*Hydrocotyle*	*Hydrocotyle verticillata*	FM207074
Eudicotyledons	Araliaceae	*Hydrocotyle*	*Hydrocotyle verticillata*	FM207075
Eudicotyledons	Araliaceae	*Hydrocotyle*	*Hydrocotyle verticillata*	FM207076
Eudicotyledons	Araliaceae	*Hydrocotyle*	*Hydrocotyle verticillata*	FM207077
Eudicotyledons	Araliaceae	*Hydrocotyle*	*Hydrocotyle verticillata*	FM207078
Eudicotyledons	Fabaceae	*Wisteria*	*Wisteria frutescens*	EU424099
Eudicotyledons	Fabaceae	*Wisteria*	*Wisteria frutescens*	EU424100

Eudicotyledons	Aceraceae	*Acer*	*Acer takesimense*	DQ978634
Eudicotyledons	Aceraceae	*Acer*	*Acer takesimense*	HM352727
Eudicotyledons	Apiaceae	*Angelica*	*Angelica dahurica*	GU967808
Eudicotyledons	Apiaceae	*Angelica*	*Angelica dahurica*	GQ248247
Eudicotyledons	Apiaceae	*Angelica*	*Angelica dahurica*	GQ435303
Eudicotyledons	Apiaceae	*Angelica*	*Angelica dahurica*	GQ435304
Eudicotyledons	Apiaceae	*Angelica*	*Angelica dahurica*	EF590672
Eudicotyledons	Plantaginaceae	*Antirrhinum*	*Antirrhinum hispanicum*	HM152904
Eudicotyledons	Plantaginaceae	*Antirrhinum*	*Antirrhinum hispanicum*	HM152905
Eudicotyledons	Plantaginaceae	*Antirrhinum*	*Antirrhinum hispanicum*	HM152906
Eudicotyledons	Ericaceae	*Rhododendron*	*Rhododendron edgeworthii*	JN046798
Eudicotyledons	Ericaceae	*Rhododendron*	*Rhododendron edgeworthii*	JN046799
Eudicotyledons	Ericaceae	*Rhododendron*	*Rhododendron edgeworthii*	JN046800
Eudicotyledons	Ericaceae	*Rhododendron*	*Rhododendron edgeworthii*	JN046801
Eudicotyledons	Ericaceae	*Rhododendron*	*Rhododendron edgeworthii*	JN046802
Eudicotyledons	Ericaceae	*Rhododendron*	*Rhododendron edgeworthii*	HQ706962
Eudicotyledons	Ericaceae	*Rhododendron*	*Rhododendron edgeworthii*	HQ706963
Eudicotyledons	Ericaceae	*Rhododendron*	*Rhododendron molle*	HQ707000
Eudicotyledons	Ericaceae	*Rhododendron*	*Rhododendron molle*	HQ707001
Eudicotyledons	Ericaceae	*Rhododendron*	*Rhododendron molle*	HQ707002
Eudicotyledons	Ericaceae	*Rhododendron*	*Rhododendron molle*	HQ707003
Eudicotyledons	Ericaceae	*Rhododendron*	*Rhododendron ovatum*	HQ426989
Eudicotyledons	Ericaceae	*Rhododendron*	*Rhododendron ovatum*	JN046915
Eudicotyledons	Ericaceae	*Rhododendron*	*Rhododendron ovatum*	JN046916
Eudicotyledons	Ericaceae	*Rhododendron*	*Rhododendron ferrugineum*	HE585249
Eudicotyledons	Ericaceae	*Rhododendron*	*Rhododendron ferrugineum*	HE585250
Eudicotyledons	Ericaceae	*Rhododendron*	*Rhododendron ferrugineum*	HE585251
Eudicotyledons	Ericaceae	*Rhododendron*	*Rhododendron moulmainense*	JN046890
Eudicotyledons	Ericaceae	*Rhododendron*	*Rhododendron moulmainense*	JN046891
Eudicotyledons	Ericaceae	*Rhododendron*	*Rhododendron moulmainense*	JN046892
Eudicotyledons	Ericaceae	*Rhododendron*	*Rhododendron moulmainense*	JN046893
Eudicotyledons	Ericaceae	*Rhododendron*	*Rhododendron moulmainense*	HQ707004
Eudicotyledons	Gentianaceae	*Swertia*	*Swertia franchetiana*	JN047284
Eudicotyledons	Gentianaceae	*Swertia*	*Swertia franchetiana*	JN047285
Eudicotyledons	Gentianaceae	*Swertia*	*Swertia franchetiana*	JN047286
Eudicotyledons	Gentianaceae	*Swertia*	*Swertia tetraptera*	JN047299
Eudicotyledons	Gentianaceae	*Swertia*	*Swertia tetraptera*	JN047300
Eudicotyledons	Apiaceae	*Angelica*	*Angelica decursiva*	GQ435318
Eudicotyledons	Apiaceae	*Angelica*	*Angelica decursiva*	GQ435319
Eudicotyledons	Aquifoliaceae	*Ilex*	*Ilex latifolia*	HQ426980
Eudicotyledons	Aquifoliaceae	*Ilex*	*Ilex latifolia*	EU359318
Eudicotyledons	Aquifoliaceae	*Ilex*	*Ilex latifolia*	JN044947
Eudicotyledons	Aquifoliaceae	*Ilex*	*Ilex latifolia*	JN044948
Eudicotyledons	Aquifoliaceae	*Ilex*	*Ilex latifolia*	JN044949
Eudicotyledons	Aquifoliaceae	*Ilex*	*Ilex latifolia*	JN044950
Eudicotyledons	Aquifoliaceae	*Ilex*	*Ilex rotunda*	HQ415422
Eudicotyledons	Aquifoliaceae	*Ilex*	*Ilex rotunda*	JN406924
Eudicotyledons	Geraniaceae	*Geranium*	*Geranium nepalense*	JN044739
Eudicotyledons	Geraniaceae	*Geranium*	*Geranium nepalense*	JN044740
Eudicotyledons	Geraniaceae	*Geranium*	*Geranium nepalense*	JN044741
Eudicotyledons	Caryophyllaceae	*Silene*	*Silene baccifera*	JN047113
Eudicotyledons	Caryophyllaceae	*Silene*	*Silene baccifera*	JN047114
Eudicotyledons	Caryophyllaceae	*Silene*	*Silene baccifera*	JN047115

Eudicotyledons	Caryophyllaceae	*Silene*	*Silene baccifera*	JN047116
Eudicotyledons	Caryophyllaceae	*Silene*	*Silene baccifera*	JN047117
Eudicotyledons	Caryophyllaceae	*Silene*	*Silene aprica*	JN047101
Eudicotyledons	Caryophyllaceae	*Silene*	*Silene aprica*	JN047102
Eudicotyledons	Caryophyllaceae	*Silene*	*Silene aprica*	JN047103
Eudicotyledons	Caryophyllaceae	*Silene*	*Silene aprica*	JN047104
Eudicotyledons	Caryophyllaceae	*Silene*	*Silene aprica*	JN047105
Eudicotyledons	Caryophyllaceae	*Silene*	*Silene aprica*	JN047106
Eudicotyledons	Caryophyllaceae	*Silene*	*Silene aprica*	JN047107
Eudicotyledons	Caryophyllaceae	*Silene*	*Silene aprica*	JN047108
Eudicotyledons	Caryophyllaceae	*Silene*	*Silene aprica*	JN047109
Eudicotyledons	Caryophyllaceae	*Silene*	*Silene aprica*	JN047110
Eudicotyledons	Caryophyllaceae	*Silene*	*Silene aprica*	JN047111
Eudicotyledons	Caryophyllaceae	*Silene*	*Silene aprica*	JN047112
Eudicotyledons	Balsaminaceae	*Impatiens*	*Impatiens capensis*	HQ596732
Eudicotyledons	Balsaminaceae	*Impatiens*	*Impatiens capensis*	DQ006157
Eudicotyledons	Asteraceae	*Inula*	*Inula helenium*	HQ596733
Eudicotyledons	Asteraceae	*Inula*	*Inula helenium*	HQ596734
Eudicotyledons	Asteraceae	*Inula*	*Inula helenium*	FM998672
Eudicotyledons	Oleaceae	*Fraxinus*	*Fraxinus mandshurica*	HM367487
Eudicotyledons	Oleaceae	*Fraxinus*	*Fraxinus mandshurica*	HM367488
Eudicotyledons	Oleaceae	*Fraxinus*	*Fraxinus mandshurica*	HM367489
Eudicotyledons	Oleaceae	*Fraxinus*	*Fraxinus mandshurica*	HM367490
Eudicotyledons	Oleaceae	*Fraxinus*	*Fraxinus mandshurica*	HM367491
Eudicotyledons	Oleaceae	*Fraxinus*	*Fraxinus mandshurica*	HM367492
Eudicotyledons	Oleaceae	*Fraxinus*	*Fraxinus mandshurica*	HM367493
Eudicotyledons	Oleaceae	*Fraxinus*	*Fraxinus mandshurica*	HM367494
Eudicotyledons	Oleaceae	*Fraxinus*	*Fraxinus mandshurica*	HM367495
Eudicotyledons	Oleaceae	*Fraxinus*	*Fraxinus platypoda*	HM367513
Eudicotyledons	Oleaceae	*Fraxinus*	*Fraxinus platypoda*	HM367514
Eudicotyledons	Oleaceae	*Fraxinus*	*Fraxinus platypoda*	HM367515
Eudicotyledons	Oleaceae	*Fraxinus*	*Fraxinus platypoda*	HM367516
Eudicotyledons	Oleaceae	*Fraxinus*	*Fraxinus platypoda*	HM367517
Eudicotyledons	Oleaceae	*Fraxinus*	*Fraxinus platypoda*	HM367518
Eudicotyledons	Oleaceae	*Fraxinus*	*Fraxinus platypoda*	HM367519
Eudicotyledons	Oleaceae	*Fraxinus*	*Fraxinus platypoda*	HM367520
Eudicotyledons	Oleaceae	*Fraxinus*	*Fraxinus platypoda*	HM367521
Eudicotyledons	Oleaceae	*Fraxinus*	*Fraxinus platypoda*	HM367522
Eudicotyledons	Oleaceae	*Fraxinus*	*Fraxinus platypoda*	HM367523
Eudicotyledons	Oleaceae	*Fraxinus*	*Fraxinus platypoda*	HM367524
Eudicotyledons	Oleaceae	*Fraxinus*	*Fraxinus nigra*	HQ596702
Eudicotyledons	Oleaceae	*Fraxinus*	*Fraxinus nigra*	HM367497
Eudicotyledons	Oleaceae	*Fraxinus*	*Fraxinus nigra*	HM367498
Eudicotyledons	Oleaceae	*Fraxinus*	*Fraxinus quadrangulata*	HM367531
Eudicotyledons	Oleaceae	*Fraxinus*	*Fraxinus quadrangulata*	HM367532
Eudicotyledons	Oleaceae	*Fraxinus*	*Fraxinus chinensis*	GQ435221
Eudicotyledons	Oleaceae	*Fraxinus*	*Fraxinus chinensis*	HM367402
Eudicotyledons	Oleaceae	*Fraxinus*	*Fraxinus chinensis*	HM367403
Eudicotyledons	Oleaceae	*Fraxinus*	*Fraxinus chinensis*	HM367404
Eudicotyledons	Oleaceae	*Fraxinus*	*Fraxinus chinensis*	HM367405
Eudicotyledons	Oleaceae	*Fraxinus*	*Fraxinus chinensis*	HM367406
Eudicotyledons	Oleaceae	*Fraxinus*	*Fraxinus chinensis*	HM367407
Eudicotyledons	Oleaceae	*Fraxinus*	*Fraxinus chinensis*	HM367408

Eudicotyledons	Oleaceae	*Fraxinus*	*Fraxinus chinensis*	HM367409
Eudicotyledons	Oleaceae	*Fraxinus*	*Fraxinus longicuspis*	HM367472
Eudicotyledons	Oleaceae	*Fraxinus*	*Fraxinus longicuspis*	HM367473
Eudicotyledons	Oleaceae	*Fraxinus*	*Fraxinus longicuspis*	HM367474
Eudicotyledons	Oleaceae	*Fraxinus*	*Fraxinus longicuspis*	HM367475
Eudicotyledons	Oleaceae	*Fraxinus*	*Fraxinus longicuspis*	HM367476
Eudicotyledons	Oleaceae	*Fraxinus*	*Fraxinus longicuspis*	HM367477
Eudicotyledons	Oleaceae	*Fraxinus*	*Fraxinus longicuspis*	HM367478
Eudicotyledons	Oleaceae	*Fraxinus*	*Fraxinus longicuspis*	HM367479
Eudicotyledons	Oleaceae	*Fraxinus*	*Fraxinus longicuspis*	HM367480
Eudicotyledons	Oleaceae	*Fraxinus*	*Fraxinus longicuspis*	HM367481
Eudicotyledons	Oleaceae	*Fraxinus*	*Fraxinus longicuspis*	HM367482
Eudicotyledons	Oleaceae	*Fraxinus*	*Fraxinus longicuspis*	HM367483
Eudicotyledons	Oleaceae	*Fraxinus*	*Fraxinus longicuspis*	HM367484
Eudicotyledons	Oleaceae	*Fraxinus*	*Fraxinus pennsylvanica*	HQ596703
Eudicotyledons	Oleaceae	*Fraxinus*	*Fraxinus pennsylvanica*	HM367511
Eudicotyledons	Oleaceae	*Fraxinus*	*Fraxinus pennsylvanica*	HM367512
Eudicotyledons	Oleaceae	*Fraxinus*	*Fraxinus tomentosa*	HM367560
Eudicotyledons	Oleaceae	*Fraxinus*	*Fraxinus tomentosa*	HM367561
Eudicotyledons	Oleaceae	*Fraxinus*	*Fraxinus tomentosa*	HM367562
Eudicotyledons	Oleaceae	*Fraxinus*	*Fraxinus tomentosa*	HM367563
Eudicotyledons	Oleaceae	*Fraxinus*	*Fraxinus tomentosa*	HM367564
Eudicotyledons	Oleaceae	*Fraxinus*	*Fraxinus velutina*	HM367571
Eudicotyledons	Oleaceae	*Fraxinus*	*Fraxinus velutina*	HM367572
Eudicotyledons	Oleaceae	*Fraxinus*	*Fraxinus velutina*	HM367573
Eudicotyledons	Oleaceae	*Fraxinus*	*Fraxinus velutina*	HM367574
Eudicotyledons	Oleaceae	*Fraxinus*	*Fraxinus velutina*	HM367575
Eudicotyledons	Oleaceae	*Fraxinus*	*Fraxinus velutina*	HM367576
Eudicotyledons	Oleaceae	*Fraxinus*	*Fraxinus velutina*	HM367577
Eudicotyledons	Oleaceae	*Fraxinus*	*Fraxinus velutina*	HM367578
Eudicotyledons	Oleaceae	*Fraxinus*	*Fraxinus velutina*	HM367579
Eudicotyledons	Oleaceae	*Fraxinus*	*Fraxinus biltmoreana*	HM367392
Eudicotyledons	Oleaceae	*Fraxinus*	*Fraxinus biltmoreana*	HM367393
Eudicotyledons	Oleaceae	*Fraxinus*	*Fraxinus latifolia*	HM367467
Eudicotyledons	Oleaceae	*Fraxinus*	*Fraxinus latifolia*	HM367468
Eudicotyledons	Oleaceae	*Fraxinus*	*Fraxinus latifolia*	HM367469
Eudicotyledons	Oleaceae	*Fraxinus*	*Fraxinus latifolia*	HM367470
Eudicotyledons	Oleaceae	*Fraxinus*	*Fraxinus latifolia*	HM367471
Eudicotyledons	Oleaceae	*Fraxinus*	*Fraxinus anomala*	HM367379
Eudicotyledons	Oleaceae	*Fraxinus*	*Fraxinus anomala*	HM367380
Eudicotyledons	Oleaceae	*Fraxinus*	*Fraxinus cuspidata*	HM367410
Eudicotyledons	Oleaceae	*Fraxinus*	*Fraxinus cuspidata*	HM367411
Eudicotyledons	Oleaceae	*Fraxinus*	*Fraxinus cuspidata*	HM367412
Eudicotyledons	Oleaceae	*Fraxinus*	*Fraxinus cuspidata*	HM367413
Eudicotyledons	Oleaceae	*Fraxinus*	*Fraxinus cuspidata*	HM367414
Eudicotyledons	Asteraceae	*Inula*	*Inula salicina*	FR865070
Eudicotyledons	Asteraceae	*Inula*	*Inula salicina*	FM998675
Eudicotyledons	Adoxaceae	*Sambucus*	*Sambucus canadensis*	HQ596833
Eudicotyledons	Adoxaceae	*Sambucus*	*Sambucus canadensis*	AY627425
Eudicotyledons	Adoxaceae	*Sambucus*	*Sambucus adnata*	JN047058
Eudicotyledons	Adoxaceae	*Sambucus*	*Sambucus adnata*	JN047059
Eudicotyledons	Adoxaceae	*Sambucus*	*Sambucus adnata*	JN047060
Eudicotyledons	Adoxaceae	*Sambucus*	*Sambucus adnata*	JN047061

Eudicotyledons	Adoxaceae	*Sambucus*	*Sambucus adnata*	JN047062
Eudicotyledons	Adoxaceae	*Sambucus*	*Sambucus adnata*	JN047063
Eudicotyledons	Adoxaceae	*Sambucus*	*Sambucus adnata*	JN047064
Eudicotyledons	Adoxaceae	*Sambucus*	*Sambucus adnata*	JN047065
Eudicotyledons	Adoxaceae	*Sambucus*	*Sambucus adnata*	JN047066
Eudicotyledons	Adoxaceae	*Sambucus*	*Sambucus adnata*	JN047067
Eudicotyledons	Adoxaceae	*Sambucus*	*Sambucus adnata*	JN047068
Eudicotyledons	Fabaceae	*Trifolium*	*Trifolium pratense*	FJ395480
Eudicotyledons	Fabaceae	*Trifolium*	*Trifolium pratense*	EU750597
Eudicotyledons	Fabaceae	*Trifolium*	*Trifolium pratense*	EU750598
Eudicotyledons	Fabaceae	*Trifolium*	*Trifolium pratense*	EU750599
Eudicotyledons	Aceraceae	*Acer*	*Acer tataricum*	JN043729
Eudicotyledons	Aceraceae	*Acer*	*Acer tataricum*	JN043730
Eudicotyledons	Aceraceae	*Acer*	*Acer spicatum*	DQ978632
Eudicotyledons	Aceraceae	*Acer*	*Acer spicatum*	HQ596579
Eudicotyledons	Aceraceae	*Acer*	*Acer tegmentosum*	DQ978637
Eudicotyledons	Aceraceae	*Acer*	*Acer tegmentosum*	HM008584
Eudicotyledons	Cornaceae	*Cornus*	*Cornus nuttallii*	FJ541997
Eudicotyledons	Cornaceae	*Cornus*	*Cornus nuttallii*	JF321205
Eudicotyledons	Adoxaceae	*Viburnum*	*Viburnum lentago*	HQ596888
Eudicotyledons	Adoxaceae	*Viburnum*	*Viburnum lentago*	AY627406
Eudicotyledons	Adoxaceae	*Viburnum*	*Viburnum lentago*	EU750609
Eudicotyledons	Adoxaceae	*Viburnum*	*Viburnum lentago*	EU750610
Eudicotyledons	Cucurbitaceae	*Bryonia*	*Bryonia cretica*	EU096340
Eudicotyledons	Cucurbitaceae	*Bryonia*	*Bryonia cretica*	EU096341
Eudicotyledons	Cucurbitaceae	*Bryonia*	*Bryonia cretica*	EU096342
Eudicotyledons	Cucurbitaceae	*Bryonia*	*Bryonia cretica*	EU096343
Eudicotyledons	Cucurbitaceae	*Bryonia*	*Bryonia cretica*	EU096344
Eudicotyledons	Cucurbitaceae	*Bryonia*	*Bryonia cretica*	EU096345
Eudicotyledons	Cucurbitaceae	*Bryonia*	*Bryonia cretica*	EU096346
Eudicotyledons	Cucurbitaceae	*Bryonia*	*Bryonia cretica*	EU096347
Eudicotyledons	Cucurbitaceae	*Bryonia*	*Bryonia cretica*	EU096405
Eudicotyledons	Cucurbitaceae	*Bryonia*	*Bryonia cretica*	EU096406
Eudicotyledons	Hamamelidaceae	*Hamamelis*	*Hamamelis japonica*	GU576755
Eudicotyledons	Hamamelidaceae	*Hamamelis*	*Hamamelis japonica*	GU576756
Eudicotyledons	Hamamelidaceae	*Hamamelis*	*Hamamelis japonica*	GU576757
Eudicotyledons	Hamamelidaceae	*Hamamelis*	*Hamamelis japonica*	GU576758
Eudicotyledons	Hamamelidaceae	*Hamamelis*	*Hamamelis vernalis*	GU576764
Eudicotyledons	Hamamelidaceae	*Hamamelis*	*Hamamelis vernalis*	GU576765
Eudicotyledons	Hamamelidaceae	*Hamamelis*	*Hamamelis vernalis*	GU576766
Eudicotyledons	Hamamelidaceae	*Hamamelis*	*Hamamelis vernalis*	GU576767
Eudicotyledons	Hamamelidaceae	*Hamamelis*	*Hamamelis vernalis*	GU576768
Eudicotyledons	Altingiaceae	*Liquidambar*	*Liquidambar acalycina*	GU576771
Eudicotyledons	Altingiaceae	*Liquidambar*	*Liquidambar acalycina*	EU595860
Eudicotyledons	Altingiaceae	*Liquidambar*	*Liquidambar formosana*	GU576772
Eudicotyledons	Altingiaceae	*Liquidambar*	*Liquidambar formosana*	HQ427006
Eudicotyledons	Altingiaceae	*Liquidambar*	*Liquidambar formosana*	GQ435052
Eudicotyledons	Altingiaceae	*Liquidambar*	*Liquidambar formosana*	EU595861
Eudicotyledons	Altingiaceae	*Liquidambar*	*Liquidambar formosana*	EF138730
Eudicotyledons	Altingiaceae	*Liquidambar*	*Liquidambar orientalis*	EU595855
Eudicotyledons	Altingiaceae	*Liquidambar*	*Liquidambar orientalis*	EF138729
Eudicotyledons	Balsaminaceae	*Impatiens*	*Impatiens balsamina*	GQ434995
Eudicotyledons	Balsaminaceae	*Impatiens*	*Impatiens balsamina*	EF590706

Eudicotyledons	Rosaceae	*Fragaria*	*Fragaria moschata*	GQ476762
Eudicotyledons	Rosaceae	*Fragaria*	*Fragaria moschata*	GQ476763
Eudicotyledons	Rosaceae	*Fragaria*	*Fragaria moschata*	GQ476764
Eudicotyledons	Orobanchaceae	*Pedicularis*	*Pedicularis resupinata*	JN046091
Eudicotyledons	Orobanchaceae	*Pedicularis*	*Pedicularis resupinata*	JN046092
Eudicotyledons	Orobanchaceae	*Pedicularis*	*Pedicularis resupinata*	JN046093
Eudicotyledons	Lamiaceae	*Scutellaria*	*Scutellaria baicalensis*	GQ374140
Eudicotyledons	Lamiaceae	*Scutellaria*	*Scutellaria baicalensis*	GQ374141
Eudicotyledons	Lamiaceae	*Scutellaria*	*Scutellaria baicalensis*	GQ374142
Eudicotyledons	Lamiaceae	*Scutellaria*	*Scutellaria baicalensis*	GQ374143
Eudicotyledons	Lamiaceae	*Scutellaria*	*Scutellaria baicalensis*	GQ374144
Eudicotyledons	Lamiaceae	*Scutellaria*	*Scutellaria baicalensis*	GQ374145
Eudicotyledons	Lamiaceae	*Scutellaria*	*Scutellaria baicalensis*	GQ374146
Eudicotyledons	Lamiaceae	*Scutellaria*	*Scutellaria baicalensis*	GQ374147
Eudicotyledons	Lamiaceae	*Scutellaria*	*Scutellaria baicalensis*	GQ374148
Eudicotyledons	Lamiaceae	*Scutellaria*	*Scutellaria baicalensis*	GQ374149
Eudicotyledons	Lamiaceae	*Scutellaria*	*Scutellaria baicalensis*	GQ374150
Eudicotyledons	Lamiaceae	*Scutellaria*	*Scutellaria baicalensis*	GQ374151
Eudicotyledons	Lamiaceae	*Scutellaria*	*Scutellaria baicalensis*	EU590864
Eudicotyledons	Lamiaceae	*Scutellaria*	*Scutellaria baicalensis*	HQ680364
Eudicotyledons	Lamiaceae	*Scutellaria*	*Scutellaria baicalensis*	HQ680365
Eudicotyledons	Lamiaceae	*Scutellaria*	*Scutellaria baicalensis*	HQ680366
Eudicotyledons	Aceraceae	*Acer*	*Acer palmatum*	DQ978615
Eudicotyledons	Aceraceae	*Acer*	*Acer palmatum*	HM352723
Eudicotyledons	Aceraceae	*Acer*	*Acer crataegifolium*	DQ978593
Eudicotyledons	Aceraceae	*Acer*	*Acer crataegifolium*	HM008572
Eudicotyledons	Aceraceae	*Acer*	*Acer tschonoskii*	DQ978641
Eudicotyledons	Aceraceae	*Acer*	*Acer tschonoskii*	HM008585
Eudicotyledons	Asteraceae	*Centaurea*	*Centaurea vallesiaca*	DQ846171
Eudicotyledons	Asteraceae	*Centaurea*	*Centaurea vallesiaca*	DQ846172
Eudicotyledons	Asteraceae	*Centaurea*	*Centaurea vallesiaca*	DQ846173
Eudicotyledons	Asteraceae	*Centaurea*	*Centaurea vallesiaca*	DQ846174
Eudicotyledons	Asteraceae	*Centaurea*	*Centaurea vallesiaca*	DQ846175
Eudicotyledons	Asteraceae	*Centaurea*	*Centaurea vallesiaca*	DQ846176
Eudicotyledons	Asteraceae	*Centaurea*	*Centaurea vallesiaca*	DQ846177
Eudicotyledons	Asteraceae	*Centaurea*	*Centaurea vallesiaca*	DQ846178
Eudicotyledons	Asteraceae	*Centaurea*	*Centaurea vallesiaca*	DQ846179
Eudicotyledons	Asteraceae	*Centaurea*	*Centaurea vallesiaca*	DQ846207
Eudicotyledons	Asteraceae	*Centaurea*	*Centaurea vallesiaca*	DQ846208
Eudicotyledons	Asteraceae	*Centaurea*	*Centaurea vallesiaca*	DQ846209
Eudicotyledons	Asteraceae	*Centaurea*	*Centaurea vallesiaca*	DQ846267
Eudicotyledons	Asteraceae	*Centaurea*	*Centaurea vallesiaca*	DQ846268
Eudicotyledons	Asteraceae	*Centaurea*	*Centaurea vallesiaca*	DQ846269
Eudicotyledons	Asteraceae	*Centaurea*	*Centaurea vallesiaca*	DQ846270
Eudicotyledons	Asteraceae	*Centaurea*	*Centaurea vallesiaca*	DQ846271
Eudicotyledons	Asteraceae	*Centaurea*	*Centaurea vallesiaca*	DQ846272
Eudicotyledons	Asteraceae	*Erigeron*	*Erigeron philadelphicus*	HQ596689
Eudicotyledons	Asteraceae	*Erigeron*	*Erigeron philadelphicus*	HQ596690
Eudicotyledons	Salicaceae	*Populus*	*Populus balsamifera*	GU562411
Eudicotyledons	Salicaceae	*Populus*	*Populus balsamifera*	EU750495
Eudicotyledons	Salicaceae	*Populus*	*Populus balsamifera*	EU750496
Eudicotyledons	Rosaceae	*Rosa*	*Rosa gallica*	DQ778773
Eudicotyledons	Rosaceae	*Rosa*	*Rosa gallica*	AB043947

Eudicotyledons	Rosaceae	*Rosa*	*Rosa canina*	DQ778759
Eudicotyledons	Rosaceae	*Rosa*	*Rosa canina*	FJ395548
Eudicotyledons	Rosaceae	*Rosa*	*Rosa carolina*	DQ778760
Eudicotyledons	Rosaceae	*Rosa*	*Rosa carolina*	DQ778761
Eudicotyledons	Rosaceae	*Rosa*	*Rosa blanda*	DQ778753
Eudicotyledons	Rosaceae	*Rosa*	*Rosa blanda*	DQ778779
Eudicotyledons	Rosaceae	*Rosa*	*Rosa blanda*	DQ778803
Eudicotyledons	Rosaceae	*Rosa*	*Rosa blanda*	HQ596822
Eudicotyledons	Rosaceae	*Rosa*	*Rosa californica*	DQ778757
Eudicotyledons	Rosaceae	*Rosa*	*Rosa californica*	DQ778758
Eudicotyledons	Rosaceae	*Rosa*	*Rosa moschata*	DQ778789
Eudicotyledons	Rosaceae	*Rosa*	*Rosa moschata*	AB043948
Eudicotyledons	Rosaceae	*Rosa*	*Rosa moschata*	AB043949
Eudicotyledons	Rosaceae	*Rosa*	*Rosa moschata*	AB043951
Eudicotyledons	Rosaceae	*Rosa*	*Rosa moschata*	AB043952
Eudicotyledons	Rosaceae	*Rosa*	*Rosa lucieae*	DQ778811
Eudicotyledons	Rosaceae	*Rosa*	*Rosa lucieae*	DQ778812
Eudicotyledons	Plantaginaceae	*Veronica*	*Veronica densifolia*	FJ848125
Eudicotyledons	Plantaginaceae	*Veronica*	*Veronica densifolia*	FJ848126
Eudicotyledons	Plantaginaceae	*Veronica*	*Veronica macrantha*	FJ848136
Eudicotyledons	Plantaginaceae	*Veronica*	*Veronica macrantha*	FJ848137
Eudicotyledons	Plantaginaceae	*Veronica*	*Veronica salicifolia*	FJ848141
Eudicotyledons	Plantaginaceae	*Veronica*	*Veronica salicifolia*	FJ848142
Eudicotyledons	Ericaceae	*Rhododendron*	*Rhododendron stamineum*	JN046976
Eudicotyledons	Ericaceae	*Rhododendron*	*Rhododendron stamineum*	JN046977
Eudicotyledons	Ericaceae	*Rhododendron*	*Rhododendron stamineum*	HQ707025
Eudicotyledons	Ericaceae	*Rhododendron*	*Rhododendron stamineum*	HQ707026
Eudicotyledons	Ericaceae	*Rhododendron*	*Rhododendron primuliflorum*	JN046929
Eudicotyledons	Ericaceae	*Rhododendron*	*Rhododendron primuliflorum*	JN046930
Eudicotyledons	Ericaceae	*Rhododendron*	*Rhododendron primuliflorum*	JN046931
Eudicotyledons	Ericaceae	*Rhododendron*	*Rhododendron farrerae*	HQ706967
Eudicotyledons	Ericaceae	*Rhododendron*	*Rhododendron farrerae*	HQ706968
Eudicotyledons	Ericaceae	*Rhododendron*	*Rhododendron indicum*	HQ706982
Eudicotyledons	Ericaceae	*Rhododendron*	*Rhododendron indicum*	HQ706983
Eudicotyledons	Ericaceae	*Rhododendron*	*Rhododendron indicum*	HQ706984
Eudicotyledons	Ericaceae	*Rhododendron*	*Rhododendron indicum*	HQ706985
Eudicotyledons	Salicaceae	*Salix*	*Salix alba*	GU373307
Eudicotyledons	Salicaceae	*Salix*	*Salix alba*	HQ596830
Eudicotyledons	Salicaceae	*Salix*	*Salix babylonica*	EU750538
Eudicotyledons	Salicaceae	*Salix*	*Salix babylonica*	EU750539
Eudicotyledons	Salicaceae	*Salix*	*Salix bebbiana*	GU562403
Eudicotyledons	Salicaceae	*Salix*	*Salix bebbiana*	HQ596831
Eudicotyledons	Aceraceae	*Acer*	*Acer saccharinum*	HQ266408
Eudicotyledons	Aceraceae	*Acer*	*Acer saccharinum*	HQ266409
Eudicotyledons	Aceraceae	*Acer*	*Acer saccharinum*	HQ266410
Eudicotyledons	Aceraceae	*Acer*	*Acer saccharinum*	HQ266411
Eudicotyledons	Aceraceae	*Acer*	*Acer saccharinum*	HQ266412
Eudicotyledons	Aceraceae	*Acer*	*Acer saccharinum*	HQ266413
Eudicotyledons	Aceraceae	*Acer*	*Acer saccharinum*	HQ266414
Eudicotyledons	Aceraceae	*Acer*	*Acer saccharinum*	HQ266415
Eudicotyledons	Aceraceae	*Acer*	*Acer saccharinum*	HQ266416
Eudicotyledons	Aceraceae	*Acer*	*Acer saccharinum*	HQ266417
Eudicotyledons	Aceraceae	*Acer*	*Acer saccharinum*	HQ266418

Eudicotyledons	Aceraceae	*Acer*	*Acer saccharinum*	HQ266419
Eudicotyledons	Aceraceae	*Acer*	*Acer saccharinum*	HQ266420
Eudicotyledons	Aceraceae	*Acer*	*Acer saccharinum*	HQ266421
Eudicotyledons	Aceraceae	*Acer*	*Acer saccharinum*	HQ266422
Eudicotyledons	Aceraceae	*Acer*	*Acer saccharinum*	HQ266423
Eudicotyledons	Aceraceae	*Acer*	*Acer saccharinum*	HQ266424
Eudicotyledons	Aceraceae	*Acer*	*Acer saccharinum*	HQ266425
Eudicotyledons	Aceraceae	*Acer*	*Acer saccharinum*	HQ266426
Eudicotyledons	Aceraceae	*Acer*	*Acer saccharinum*	HQ266427
Eudicotyledons	Aceraceae	*Acer*	*Acer saccharinum*	HQ266428
Eudicotyledons	Aceraceae	*Acer*	*Acer saccharinum*	HQ266429
Eudicotyledons	Aceraceae	*Acer*	*Acer saccharinum*	HQ266430
Eudicotyledons	Aceraceae	*Acer*	*Acer saccharinum*	HQ266431
Eudicotyledons	Aceraceae	*Acer*	*Acer saccharinum*	HQ266432
Eudicotyledons	Aceraceae	*Acer*	*Acer saccharinum*	HQ266433
Eudicotyledons	Aceraceae	*Acer*	*Acer saccharinum*	HQ266434
Eudicotyledons	Aceraceae	*Acer*	*Acer saccharinum*	HQ266435
Eudicotyledons	Aceraceae	*Acer*	*Acer saccharinum*	HQ266436
Eudicotyledons	Aceraceae	*Acer*	*Acer saccharinum*	HQ266437
Eudicotyledons	Aceraceae	*Acer*	*Acer saccharinum*	HQ266438
Eudicotyledons	Aceraceae	*Acer*	*Acer saccharinum*	HQ266439
Eudicotyledons	Aceraceae	*Acer*	*Acer saccharinum*	HQ266440
Eudicotyledons	Aceraceae	*Acer*	*Acer saccharinum*	HQ266441
Eudicotyledons	Aceraceae	*Acer*	*Acer saccharinum*	HQ266442
Eudicotyledons	Aceraceae	*Acer*	*Acer saccharinum*	HQ266443
Eudicotyledons	Aceraceae	*Acer*	*Acer saccharinum*	HQ266444
Eudicotyledons	Aceraceae	*Acer*	*Acer saccharinum*	HQ266445
Eudicotyledons	Aceraceae	*Acer*	*Acer saccharinum*	HQ266446
Eudicotyledons	Aceraceae	*Acer*	*Acer saccharinum*	HQ266447
Eudicotyledons	Aceraceae	*Acer*	*Acer saccharinum*	HQ266448
Eudicotyledons	Aceraceae	*Acer*	*Acer saccharinum*	HQ266449
Eudicotyledons	Aceraceae	*Acer*	*Acer saccharinum*	HQ266450
Eudicotyledons	Aceraceae	*Acer*	*Acer saccharinum*	HQ266451
Eudicotyledons	Aceraceae	*Acer*	*Acer saccharinum*	HQ266452
Eudicotyledons	Aceraceae	*Acer*	*Acer saccharinum*	HQ266453
Eudicotyledons	Aceraceae	*Acer*	*Acer saccharinum*	HQ266454
Eudicotyledons	Aceraceae	*Acer*	*Acer saccharinum*	HQ266455
Eudicotyledons	Aceraceae	*Acer*	*Acer saccharinum*	HQ266456
Eudicotyledons	Aceraceae	*Acer*	*Acer saccharinum*	HQ266457
Eudicotyledons	Aceraceae	*Acer*	*Acer saccharinum*	HQ266458
Eudicotyledons	Aceraceae	*Acer*	*Acer saccharinum*	HQ266459
Eudicotyledons	Aceraceae	*Acer*	*Acer saccharinum*	HQ266460
Eudicotyledons	Aceraceae	*Acer*	*Acer saccharinum*	HQ266461
Eudicotyledons	Aceraceae	*Acer*	*Acer saccharinum*	HQ266462
Eudicotyledons	Aceraceae	*Acer*	*Acer saccharinum*	HQ266463
Eudicotyledons	Aceraceae	*Acer*	*Acer saccharinum*	HQ266464
Eudicotyledons	Aceraceae	*Acer*	*Acer saccharinum*	HQ266465
Eudicotyledons	Aceraceae	*Acer*	*Acer saccharinum*	HQ266466
Eudicotyledons	Aceraceae	*Acer*	*Acer saccharinum*	HQ266467
Eudicotyledons	Aceraceae	*Acer*	*Acer saccharinum*	HQ266468
Eudicotyledons	Aceraceae	*Acer*	*Acer saccharinum*	HQ266469
Eudicotyledons	Aceraceae	*Acer*	*Acer saccharinum*	HQ266470
Eudicotyledons	Aceraceae	*Acer*	*Acer saccharinum*	HQ266471

Eudicotyledons	Aceraceae	*Acer*	*Acer saccharinum*	HQ266472
Eudicotyledons	Aceraceae	*Acer*	*Acer saccharinum*	HQ266473
Eudicotyledons	Aceraceae	*Acer*	*Acer saccharinum*	HQ266474
Eudicotyledons	Aceraceae	*Acer*	*Acer saccharinum*	HQ266475
Eudicotyledons	Aceraceae	*Acer*	*Acer saccharinum*	HQ266476
Eudicotyledons	Aceraceae	*Acer*	*Acer saccharinum*	HQ266477
Eudicotyledons	Aceraceae	*Acer*	*Acer saccharinum*	HQ266478
Eudicotyledons	Aceraceae	*Acer*	*Acer saccharinum*	HQ266479
Eudicotyledons	Aceraceae	*Acer*	*Acer saccharinum*	HQ266480
Eudicotyledons	Aceraceae	*Acer*	*Acer saccharinum*	HQ266481
Eudicotyledons	Aceraceae	*Acer*	*Acer saccharinum*	HQ266482
Eudicotyledons	Aceraceae	*Acer*	*Acer saccharinum*	HQ266483
Eudicotyledons	Aceraceae	*Acer*	*Acer saccharinum*	HQ266484
Eudicotyledons	Aceraceae	*Acer*	*Acer saccharinum*	HQ266485
Eudicotyledons	Aceraceae	*Acer*	*Acer saccharinum*	HQ266486
Eudicotyledons	Aceraceae	*Acer*	*Acer saccharinum*	HQ266487
Eudicotyledons	Aceraceae	*Acer*	*Acer saccharinum*	HQ266488
Eudicotyledons	Aceraceae	*Acer*	*Acer saccharinum*	HQ266489
Eudicotyledons	Aceraceae	*Acer*	*Acer saccharinum*	HQ266490
Eudicotyledons	Aceraceae	*Acer*	*Acer saccharinum*	EU750434
Eudicotyledons	Aceraceae	*Acer*	*Acer saccharinum*	EU750435
Eudicotyledons	Aceraceae	*Acer*	*Acer saccharinum*	EU750436
Eudicotyledons	Aceraceae	*Acer*	*Acer saccharinum*	EU750437
Eudicotyledons	Asteraceae	*Lactuca*	*Lactuca serriola*	HQ596742
Eudicotyledons	Asteraceae	*Lactuca*	*Lactuca serriola*	GU109332
Eudicotyledons	Asteraceae	*Lactuca*	*Lactuca serriola*	EU750469
Eudicotyledons	Asteraceae	*Lactuca*	*Lactuca serriola*	EU750470
Eudicotyledons	Asteraceae	*Lactuca*	*Lactuca indica*	GU109317
Eudicotyledons	Asteraceae	*Lactuca*	*Lactuca indica*	GU109320
Eudicotyledons	Campanulaceae	*Lobelia*	*Lobelia cardinalis*	DQ272722
Eudicotyledons	Campanulaceae	*Lobelia*	*Lobelia cardinalis*	GQ248331
Eudicotyledons	Campanulaceae	*Lobelia*	*Lobelia cardinalis*	EF590709
Eudicotyledons	Salicaceae	*Salix*	*Salix exigua*	EU750544
Eudicotyledons	Salicaceae	*Salix*	*Salix exigua*	EU750545
Eudicotyledons	Salicaceae	*Salix*	*Salix herbacea*	GU373280
Eudicotyledons	Salicaceae	*Salix*	*Salix herbacea*	GU373281
Eudicotyledons	Burseraceae	*Bursera*	*Bursera attenuata*	GQ377871
Eudicotyledons	Burseraceae	*Bursera*	*Bursera attenuata*	GQ377872
Eudicotyledons	Burseraceae	*Bursera*	*Bursera attenuata*	GQ377873
Eudicotyledons	Burseraceae	*Bursera*	*Bursera grandifolia*	GQ377876
Eudicotyledons	Burseraceae	*Bursera*	*Bursera grandifolia*	GQ377877
Eudicotyledons	Burseraceae	*Bursera*	*Bursera grandifolia*	GQ377878
Eudicotyledons	Burseraceae	*Bursera*	*Bursera grandifolia*	GQ377879
Eudicotyledons	Burseraceae	*Bursera*	*Bursera instabilis*	GQ377882
Eudicotyledons	Burseraceae	*Bursera*	*Bursera instabilis*	GQ377883
Eudicotyledons	Burseraceae	*Bursera*	*Bursera instabilis*	GQ377884
Eudicotyledons	Burseraceae	*Bursera*	*Bursera instabilis*	GQ377885
Eudicotyledons	Burseraceae	*Bursera*	*Bursera longipes*	GQ377890
Eudicotyledons	Burseraceae	*Bursera*	*Bursera longipes*	GQ377891
Eudicotyledons	Burseraceae	*Bursera*	*Bursera longipes*	GQ377892
Eudicotyledons	Burseraceae	*Bursera*	*Bursera longipes*	GQ377893
Eudicotyledons	Burseraceae	*Bursera*	*Bursera longipes*	AY309395
Eudicotyledons	Burseraceae	*Bursera*	*Bursera simaruba*	GQ377897

Eudicotyledons	Burseraceae	*Bursera*	*Bursera simaruba*	GQ377898
Eudicotyledons	Burseraceae	*Bursera*	*Bursera simaruba*	GQ377901
Eudicotyledons	Burseraceae	*Bursera*	*Bursera simaruba*	GQ377902
Eudicotyledons	Burseraceae	*Bursera*	*Bursera simaruba*	GQ377903
Eudicotyledons	Burseraceae	*Bursera*	*Bursera simaruba*	GQ377904
Eudicotyledons	Burseraceae	*Bursera*	*Bursera simaruba*	GQ377905
Eudicotyledons	Burseraceae	*Bursera*	*Bursera simaruba*	GQ377906
Eudicotyledons	Burseraceae	*Bursera*	*Bursera simaruba*	GQ377907
Eudicotyledons	Burseraceae	*Bursera*	*Bursera simaruba*	GQ377908
Eudicotyledons	Burseraceae	*Bursera*	*Bursera simaruba*	GQ377909
Eudicotyledons	Burseraceae	*Bursera*	*Bursera simaruba*	GQ377910
Eudicotyledons	Burseraceae	*Bursera*	*Bursera simaruba*	GQ377911
Eudicotyledons	Burseraceae	*Bursera*	*Bursera simaruba*	GQ377912
Eudicotyledons	Burseraceae	*Bursera*	*Bursera simaruba*	GQ377913
Eudicotyledons	Burseraceae	*Bursera*	*Bursera simaruba*	GQ377914
Eudicotyledons	Burseraceae	*Bursera*	*Bursera simaruba*	GQ377915
Eudicotyledons	Burseraceae	*Bursera*	*Bursera simaruba*	GQ377916
Eudicotyledons	Burseraceae	*Bursera*	*Bursera simaruba*	GQ377917
Eudicotyledons	Burseraceae	*Bursera*	*Bursera simaruba*	GQ377918
Eudicotyledons	Burseraceae	*Bursera*	*Bursera simaruba*	GQ377919
Eudicotyledons	Burseraceae	*Bursera*	*Bursera simaruba*	GQ377920
Eudicotyledons	Burseraceae	*Bursera*	*Bursera simaruba*	GQ377921
Eudicotyledons	Burseraceae	*Bursera*	*Bursera simaruba*	GQ377922
Eudicotyledons	Burseraceae	*Bursera*	*Bursera simaruba*	GQ377923
Eudicotyledons	Burseraceae	*Bursera*	*Bursera simaruba*	GQ377924
Eudicotyledons	Burseraceae	*Bursera*	*Bursera simaruba*	GQ377925
Eudicotyledons	Burseraceae	*Bursera*	*Bursera simaruba*	GQ377926
Eudicotyledons	Burseraceae	*Bursera*	*Bursera simaruba*	GQ377927
Eudicotyledons	Burseraceae	*Bursera*	*Bursera simaruba*	GQ437532
Eudicotyledons	Burseraceae	*Bursera*	*Bursera simaruba*	GQ437533
Eudicotyledons	Burseraceae	*Bursera*	*Bursera simaruba*	GQ437534
Eudicotyledons	Burseraceae	*Bursera*	*Bursera simaruba*	GQ437535
Eudicotyledons	Burseraceae	*Bursera*	*Bursera simaruba*	GQ437536
Eudicotyledons	Burseraceae	*Bursera*	*Bursera simaruba*	GQ437537
Eudicotyledons	Burseraceae	*Bursera*	*Bursera simaruba*	GQ437538
Eudicotyledons	Burseraceae	*Bursera*	*Bursera simaruba*	GQ437539
Eudicotyledons	Burseraceae	*Bursera*	*Bursera simaruba*	GQ437540
Eudicotyledons	Burseraceae	*Bursera*	*Bursera simaruba*	GQ437541
Eudicotyledons	Burseraceae	*Bursera*	*Bursera simaruba*	GQ437542
Eudicotyledons	Burseraceae	*Bursera*	*Bursera simaruba*	GQ437543
Eudicotyledons	Burseraceae	*Bursera*	*Bursera simaruba*	GQ437544
Eudicotyledons	Burseraceae	*Bursera*	*Bursera simaruba*	GQ437545
Eudicotyledons	Burseraceae	*Bursera*	*Bursera simaruba*	GQ437546
Eudicotyledons	Burseraceae	*Bursera*	*Bursera simaruba*	GQ437547
Eudicotyledons	Burseraceae	*Bursera*	*Bursera simaruba*	GQ437548
Eudicotyledons	Burseraceae	*Bursera*	*Bursera simaruba*	GQ437549
Eudicotyledons	Burseraceae	*Bursera*	*Bursera simaruba*	GQ437550
Eudicotyledons	Burseraceae	*Bursera*	*Bursera simaruba*	GQ437551
Eudicotyledons	Burseraceae	*Bursera*	*Bursera simaruba*	GQ437552
Eudicotyledons	Burseraceae	*Bursera*	*Bursera simaruba*	GQ437553
Eudicotyledons	Burseraceae	*Bursera*	*Bursera simaruba*	GQ437554
Eudicotyledons	Burseraceae	*Bursera*	*Bursera simaruba*	GQ437555
Eudicotyledons	Burseraceae	*Bursera*	*Bursera simaruba*	GQ437556

Eudicotyledons	Burseraceae	*Bursera*	*Bursera simaruba*	GQ437557
Eudicotyledons	Burseraceae	*Bursera*	*Bursera simaruba*	GQ437558
Eudicotyledons	Burseraceae	*Bursera*	*Bursera simaruba*	GQ437559
Eudicotyledons	Burseraceae	*Bursera*	*Bursera simaruba*	GQ437560
Eudicotyledons	Burseraceae	*Bursera*	*Bursera simaruba*	GQ437561
Eudicotyledons	Burseraceae	*Bursera*	*Bursera simaruba*	GQ437562
Eudicotyledons	Burseraceae	*Bursera*	*Bursera simaruba*	GQ437563
Eudicotyledons	Burseraceae	*Bursera*	*Bursera simaruba*	GQ437564
Eudicotyledons	Burseraceae	*Bursera*	*Bursera simaruba*	GQ437565
Eudicotyledons	Burseraceae	*Bursera*	*Bursera simaruba*	GQ437566
Eudicotyledons	Burseraceae	*Bursera*	*Bursera simaruba*	GQ437567
Eudicotyledons	Burseraceae	*Bursera*	*Bursera simaruba*	GQ437568
Eudicotyledons	Burseraceae	*Bursera*	*Bursera simaruba*	GQ437569
Eudicotyledons	Burseraceae	*Bursera*	*Bursera simaruba*	GQ437570
Eudicotyledons	Burseraceae	*Bursera*	*Bursera simaruba*	GQ437571
Eudicotyledons	Burseraceae	*Bursera*	*Bursera simaruba*	GQ437572
Eudicotyledons	Burseraceae	*Bursera*	*Bursera simaruba*	GQ437573
Eudicotyledons	Burseraceae	*Bursera*	*Bursera simaruba*	GQ437574
Eudicotyledons	Burseraceae	*Bursera*	*Bursera simaruba*	GQ437575
Eudicotyledons	Burseraceae	*Bursera*	*Bursera simaruba*	GQ437576
Eudicotyledons	Burseraceae	*Bursera*	*Bursera simaruba*	GQ437577
Eudicotyledons	Burseraceae	*Bursera*	*Bursera simaruba*	GQ437578
Eudicotyledons	Burseraceae	*Bursera*	*Bursera simaruba*	GQ437579
Eudicotyledons	Burseraceae	*Bursera*	*Bursera simaruba*	GQ437580
Eudicotyledons	Burseraceae	*Bursera*	*Bursera simaruba*	GQ437581
Eudicotyledons	Burseraceae	*Bursera*	*Bursera simaruba*	GQ437582
Eudicotyledons	Burseraceae	*Bursera*	*Bursera simaruba*	GQ437583
Eudicotyledons	Burseraceae	*Bursera*	*Bursera simaruba*	GQ437584
Eudicotyledons	Burseraceae	*Bursera*	*Bursera simaruba*	GQ437585
Eudicotyledons	Burseraceae	*Bursera*	*Bursera simaruba*	GQ437586
Eudicotyledons	Burseraceae	*Bursera*	*Bursera simaruba*	GQ437587
Eudicotyledons	Burseraceae	*Bursera*	*Bursera simaruba*	GQ437588
Eudicotyledons	Burseraceae	*Bursera*	*Bursera simaruba*	GQ437589
Eudicotyledons	Burseraceae	*Bursera*	*Bursera simaruba*	GQ437590
Eudicotyledons	Burseraceae	*Bursera*	*Bursera simaruba*	GQ437591
Eudicotyledons	Burseraceae	*Bursera*	*Bursera simaruba*	GQ437592
Eudicotyledons	Burseraceae	*Bursera*	*Bursera simaruba*	GQ437593
Eudicotyledons	Burseraceae	*Bursera*	*Bursera simaruba*	GQ437594
Eudicotyledons	Burseraceae	*Bursera*	*Bursera simaruba*	GQ437595
Eudicotyledons	Burseraceae	*Bursera*	*Bursera simaruba*	GQ437596
Eudicotyledons	Burseraceae	*Bursera*	*Bursera simaruba*	GQ437597
Eudicotyledons	Burseraceae	*Bursera*	*Bursera simaruba*	GQ437598
Eudicotyledons	Burseraceae	*Bursera*	*Bursera simaruba*	GQ437599
Eudicotyledons	Burseraceae	*Bursera*	*Bursera simaruba*	GQ437600
Eudicotyledons	Burseraceae	*Bursera*	*Bursera simaruba*	GQ437601
Eudicotyledons	Burseraceae	*Bursera*	*Bursera simaruba*	GQ437602
Eudicotyledons	Burseraceae	*Bursera*	*Bursera simaruba*	GQ437603
Eudicotyledons	Burseraceae	*Bursera*	*Bursera simaruba*	GQ437604
Eudicotyledons	Burseraceae	*Bursera*	*Bursera simaruba*	GQ437605
Eudicotyledons	Burseraceae	*Bursera*	*Bursera simaruba*	GQ437606
Eudicotyledons	Burseraceae	*Bursera*	*Bursera simaruba*	GQ437607
Eudicotyledons	Burseraceae	*Bursera*	*Bursera simaruba*	GQ437608
Eudicotyledons	Burseraceae	*Bursera*	*Bursera simaruba*	GQ437609

Eudicotyledons	Burseraceae	*Bursera*	*Bursera simaruba*	GQ437610
Eudicotyledons	Burseraceae	*Bursera*	*Bursera simaruba*	GQ437611
Eudicotyledons	Burseraceae	*Bursera*	*Bursera simaruba*	GQ437612
Eudicotyledons	Burseraceae	*Bursera*	*Bursera simaruba*	GQ437613
Eudicotyledons	Burseraceae	*Bursera*	*Bursera simaruba*	GQ437614
Eudicotyledons	Burseraceae	*Bursera*	*Bursera simaruba*	GQ437615
Eudicotyledons	Burseraceae	*Bursera*	*Bursera simaruba*	GQ437616
Eudicotyledons	Burseraceae	*Bursera*	*Bursera simaruba*	GQ437617
Eudicotyledons	Burseraceae	*Bursera*	*Bursera simaruba*	GQ437618
Eudicotyledons	Burseraceae	*Bursera*	*Bursera simaruba*	GQ437619
Eudicotyledons	Burseraceae	*Bursera*	*Bursera simaruba*	GQ437620
Eudicotyledons	Burseraceae	*Bursera*	*Bursera simaruba*	GQ437621
Eudicotyledons	Burseraceae	*Bursera*	*Bursera simaruba*	GQ437622
Eudicotyledons	Burseraceae	*Bursera*	*Bursera simaruba*	GQ437623
Eudicotyledons	Burseraceae	*Bursera*	*Bursera simaruba*	GQ437624
Eudicotyledons	Burseraceae	*Bursera*	*Bursera simaruba*	GQ437625
Eudicotyledons	Burseraceae	*Bursera*	*Bursera simaruba*	GQ437626
Eudicotyledons	Burseraceae	*Bursera*	*Bursera simaruba*	GQ437627
Eudicotyledons	Burseraceae	*Bursera*	*Bursera simaruba*	GQ437628
Eudicotyledons	Burseraceae	*Bursera*	*Bursera simaruba*	GQ437629
Eudicotyledons	Burseraceae	*Bursera*	*Bursera simaruba*	GQ437630
Eudicotyledons	Burseraceae	*Bursera*	*Bursera simaruba*	GQ437631
Eudicotyledons	Burseraceae	*Bursera*	*Bursera simaruba*	GQ437632
Eudicotyledons	Burseraceae	*Bursera*	*Bursera simaruba*	GQ437633
Eudicotyledons	Burseraceae	*Bursera*	*Bursera simaruba*	GQ437634
Eudicotyledons	Burseraceae	*Bursera*	*Bursera simaruba*	GQ437635
Eudicotyledons	Burseraceae	*Bursera*	*Bursera simaruba*	GQ437636
Eudicotyledons	Burseraceae	*Bursera*	*Bursera simaruba*	GQ437637
Eudicotyledons	Burseraceae	*Bursera*	*Bursera simaruba*	GQ437638
Eudicotyledons	Burseraceae	*Bursera*	*Bursera simaruba*	GQ437639
Eudicotyledons	Burseraceae	*Bursera*	*Bursera simaruba*	GQ437640
Eudicotyledons	Burseraceae	*Bursera*	*Bursera simaruba*	GQ437641
Eudicotyledons	Burseraceae	*Bursera*	*Bursera simaruba*	GQ437642
Eudicotyledons	Burseraceae	*Bursera*	*Bursera simaruba*	GQ437643
Eudicotyledons	Burseraceae	*Bursera*	*Bursera simaruba*	GQ437644
Eudicotyledons	Burseraceae	*Bursera*	*Bursera simaruba*	GQ437645
Eudicotyledons	Burseraceae	*Bursera*	*Bursera simaruba*	GQ437646
Eudicotyledons	Burseraceae	*Bursera*	*Bursera simaruba*	GQ437647
Eudicotyledons	Burseraceae	*Bursera*	*Bursera simaruba*	GQ437648
Eudicotyledons	Burseraceae	*Bursera*	*Bursera simaruba*	GQ437649
Eudicotyledons	Burseraceae	*Bursera*	*Bursera simaruba*	GQ437650
Eudicotyledons	Burseraceae	*Bursera*	*Bursera simaruba*	GQ437651
Eudicotyledons	Burseraceae	*Bursera*	*Bursera simaruba*	GQ437652
Eudicotyledons	Burseraceae	*Bursera*	*Bursera simaruba*	GQ437653
Eudicotyledons	Burseraceae	*Bursera*	*Bursera simaruba*	GQ437654
Eudicotyledons	Burseraceae	*Bursera*	*Bursera simaruba*	GQ437655
Eudicotyledons	Burseraceae	*Bursera*	*Bursera simaruba*	GQ437656
Eudicotyledons	Burseraceae	*Bursera*	*Bursera simaruba*	GQ437657
Eudicotyledons	Burseraceae	*Bursera*	*Bursera simaruba*	GQ437658
Eudicotyledons	Burseraceae	*Bursera*	*Bursera simaruba*	GQ437659
Eudicotyledons	Burseraceae	*Bursera*	*Bursera simaruba*	GQ437660
Eudicotyledons	Burseraceae	*Bursera*	*Bursera simaruba*	GQ437661
Eudicotyledons	Burseraceae	*Bursera*	*Bursera simaruba*	GQ437662

Eudicotyledons	Burseraceae	*Bursera*	*Bursera simaruba*	GQ437663
Eudicotyledons	Burseraceae	*Bursera*	*Bursera simaruba*	GQ437664
Eudicotyledons	Burseraceae	*Bursera*	*Bursera simaruba*	GQ437665
Eudicotyledons	Burseraceae	*Bursera*	*Bursera simaruba*	GQ437666
Eudicotyledons	Burseraceae	*Bursera*	*Bursera simaruba*	GQ437667
Eudicotyledons	Burseraceae	*Bursera*	*Bursera simaruba*	GQ437668
Eudicotyledons	Burseraceae	*Bursera*	*Bursera simaruba*	GQ437669
Eudicotyledons	Burseraceae	*Bursera*	*Bursera simaruba*	GQ437670
Eudicotyledons	Burseraceae	*Bursera*	*Bursera simaruba*	GQ437671
Eudicotyledons	Burseraceae	*Bursera*	*Bursera simaruba*	GQ437672
Eudicotyledons	Burseraceae	*Bursera*	*Bursera simaruba*	GQ437673
Eudicotyledons	Burseraceae	*Bursera*	*Bursera simaruba*	GQ437674
Eudicotyledons	Burseraceae	*Bursera*	*Bursera simaruba*	GQ437675
Eudicotyledons	Burseraceae	*Bursera*	*Bursera simaruba*	GQ437676
Eudicotyledons	Burseraceae	*Bursera*	*Bursera simaruba*	GQ437677
Eudicotyledons	Burseraceae	*Bursera*	*Bursera simaruba*	GQ437678
Eudicotyledons	Burseraceae	*Bursera*	*Bursera simaruba*	GQ437679
Eudicotyledons	Burseraceae	*Bursera*	*Bursera simaruba*	GQ437680
Eudicotyledons	Burseraceae	*Bursera*	*Bursera simaruba*	GQ437681
Eudicotyledons	Burseraceae	*Bursera*	*Bursera simaruba*	GQ437682
Eudicotyledons	Burseraceae	*Bursera*	*Bursera simaruba*	GQ437683
Eudicotyledons	Burseraceae	*Bursera*	*Bursera simaruba*	GQ437684
Eudicotyledons	Burseraceae	*Bursera*	*Bursera simaruba*	GQ437685
Eudicotyledons	Burseraceae	*Bursera*	*Bursera simaruba*	GQ437686
Eudicotyledons	Burseraceae	*Bursera*	*Bursera simaruba*	GQ437687
Eudicotyledons	Burseraceae	*Bursera*	*Bursera simaruba*	GQ437688
Eudicotyledons	Burseraceae	*Bursera*	*Bursera simaruba*	GQ437689
Eudicotyledons	Burseraceae	*Bursera*	*Bursera simaruba*	GQ437690
Eudicotyledons	Burseraceae	*Bursera*	*Bursera simaruba*	GQ437691
Eudicotyledons	Burseraceae	*Bursera*	*Bursera simaruba*	GQ437692
Eudicotyledons	Burseraceae	*Bursera*	*Bursera simaruba*	GQ437693
Eudicotyledons	Burseraceae	*Bursera*	*Bursera simaruba*	GQ437694
Eudicotyledons	Burseraceae	*Bursera*	*Bursera simaruba*	GQ437695
Eudicotyledons	Burseraceae	*Bursera*	*Bursera simaruba*	GQ437696
Eudicotyledons	Burseraceae	*Bursera*	*Bursera simaruba*	GQ437697
Eudicotyledons	Burseraceae	*Bursera*	*Bursera simaruba*	GQ437698
Eudicotyledons	Burseraceae	*Bursera*	*Bursera simaruba*	GQ437699
Eudicotyledons	Burseraceae	*Bursera*	*Bursera simaruba*	GQ437700
Eudicotyledons	Burseraceae	*Bursera*	*Bursera simaruba*	GQ437701
Eudicotyledons	Burseraceae	*Bursera*	*Bursera simaruba*	GQ437702
Eudicotyledons	Burseraceae	*Bursera*	*Bursera simaruba*	GQ437703
Eudicotyledons	Burseraceae	*Bursera*	*Bursera simaruba*	GQ437704
Eudicotyledons	Burseraceae	*Bursera*	*Bursera simaruba*	GQ437705
Eudicotyledons	Burseraceae	*Bursera*	*Bursera simaruba*	GQ437706
Eudicotyledons	Burseraceae	*Bursera*	*Bursera simaruba*	GQ437707
Eudicotyledons	Burseraceae	*Bursera*	*Bursera simaruba*	GQ437708
Eudicotyledons	Burseraceae	*Bursera*	*Bursera simaruba*	GQ437709
Eudicotyledons	Burseraceae	*Bursera*	*Bursera simaruba*	GQ437710
Eudicotyledons	Burseraceae	*Bursera*	*Bursera simaruba*	GQ437711
Eudicotyledons	Burseraceae	*Bursera*	*Bursera simaruba*	GQ437712
Eudicotyledons	Burseraceae	*Bursera*	*Bursera simaruba*	GQ437713
Eudicotyledons	Burseraceae	*Bursera*	*Bursera simaruba*	GQ437714
Eudicotyledons	Burseraceae	*Bursera*	*Bursera simaruba*	GQ437715

Eudicotyledons	Burseraceae	*Bursera*	*Bursera simaruba*	GQ437716
Eudicotyledons	Burseraceae	*Bursera*	*Bursera simaruba*	GQ437717
Eudicotyledons	Burseraceae	*Bursera*	*Bursera simaruba*	GQ437718
Eudicotyledons	Burseraceae	*Bursera*	*Bursera simaruba*	GQ437719
Eudicotyledons	Burseraceae	*Bursera*	*Bursera simaruba*	GQ437720
Eudicotyledons	Burseraceae	*Bursera*	*Bursera simaruba*	GQ437721
Eudicotyledons	Burseraceae	*Bursera*	*Bursera simaruba*	GQ437722
Eudicotyledons	Burseraceae	*Bursera*	*Bursera simaruba*	GQ437723
Eudicotyledons	Burseraceae	*Bursera*	*Bursera simaruba*	GQ437724
Eudicotyledons	Burseraceae	*Bursera*	*Bursera simaruba*	GQ437725
Eudicotyledons	Burseraceae	*Bursera*	*Bursera simaruba*	GQ437726
Eudicotyledons	Burseraceae	*Bursera*	*Bursera simaruba*	GQ437727
Eudicotyledons	Burseraceae	*Bursera*	*Bursera simaruba*	GQ437728
Eudicotyledons	Burseraceae	*Bursera*	*Bursera simaruba*	GQ437729
Eudicotyledons	Burseraceae	*Bursera*	*Bursera simaruba*	GQ437730
Eudicotyledons	Burseraceae	*Bursera*	*Bursera simaruba*	GQ437731
Eudicotyledons	Burseraceae	*Bursera*	*Bursera simaruba*	GQ437732
Eudicotyledons	Burseraceae	*Bursera*	*Bursera simaruba*	GQ437733
Eudicotyledons	Burseraceae	*Bursera*	*Bursera simaruba*	GQ437734
Eudicotyledons	Burseraceae	*Bursera*	*Bursera simaruba*	GQ437735
Eudicotyledons	Burseraceae	*Bursera*	*Bursera simaruba*	GQ437736
Eudicotyledons	Burseraceae	*Bursera*	*Bursera simaruba*	GQ437737
Eudicotyledons	Burseraceae	*Bursera*	*Bursera simaruba*	GQ437738
Eudicotyledons	Burseraceae	*Bursera*	*Bursera simaruba*	GQ437739
Eudicotyledons	Burseraceae	*Bursera*	*Bursera simaruba*	GQ437740
Eudicotyledons	Burseraceae	*Bursera*	*Bursera simaruba*	GQ437741
Eudicotyledons	Burseraceae	*Bursera*	*Bursera simaruba*	GQ437742
Eudicotyledons	Burseraceae	*Bursera*	*Bursera simaruba*	GQ437743
Eudicotyledons	Burseraceae	*Bursera*	*Bursera simaruba*	GQ437744
Eudicotyledons	Burseraceae	*Bursera*	*Bursera simaruba*	GQ437745
Eudicotyledons	Burseraceae	*Bursera*	*Bursera simaruba*	GQ437746
Eudicotyledons	Burseraceae	*Bursera*	*Bursera simaruba*	GQ437747
Eudicotyledons	Burseraceae	*Bursera*	*Bursera simaruba*	GQ437748
Eudicotyledons	Burseraceae	*Bursera*	*Bursera simaruba*	GQ437749
Eudicotyledons	Burseraceae	*Bursera*	*Bursera simaruba*	GQ437750
Eudicotyledons	Burseraceae	*Bursera*	*Bursera simaruba*	GQ437751
Eudicotyledons	Burseraceae	*Bursera*	*Bursera simaruba*	GQ437752
Eudicotyledons	Burseraceae	*Bursera*	*Bursera simaruba*	GQ437753
Eudicotyledons	Burseraceae	*Bursera*	*Bursera simaruba*	GQ437754
Eudicotyledons	Burseraceae	*Bursera*	*Bursera simaruba*	GQ437755
Eudicotyledons	Burseraceae	*Bursera*	*Bursera simaruba*	GQ437756
Eudicotyledons	Burseraceae	*Bursera*	*Bursera simaruba*	GQ437757
Eudicotyledons	Burseraceae	*Bursera*	*Bursera simaruba*	GQ437758
Eudicotyledons	Burseraceae	*Bursera*	*Bursera simaruba*	GQ437759
Eudicotyledons	Burseraceae	*Bursera*	*Bursera simaruba*	GQ437760
Eudicotyledons	Burseraceae	*Bursera*	*Bursera simaruba*	GQ437761
Eudicotyledons	Burseraceae	*Bursera*	*Bursera simaruba*	GQ437762
Eudicotyledons	Burseraceae	*Bursera*	*Bursera simaruba*	GQ437763
Eudicotyledons	Burseraceae	*Bursera*	*Bursera simaruba*	GQ437764
Eudicotyledons	Burseraceae	*Bursera*	*Bursera simaruba*	GQ437765
Eudicotyledons	Burseraceae	*Bursera*	*Bursera simaruba*	GQ437766
Eudicotyledons	Burseraceae	*Bursera*	*Bursera simaruba*	GQ437767
Eudicotyledons	Burseraceae	*Bursera*	*Bursera simaruba*	GQ437768

Eudicotyledons	Burseraceae	*Bursera*	*Bursera simaruba*	GQ437769
Eudicotyledons	Burseraceae	*Bursera*	*Bursera simaruba*	GQ437770
Eudicotyledons	Burseraceae	*Bursera*	*Bursera simaruba*	GQ437771
Eudicotyledons	Burseraceae	*Bursera*	*Bursera simaruba*	GQ437772
Eudicotyledons	Burseraceae	*Bursera*	*Bursera simaruba*	GQ437773
Eudicotyledons	Burseraceae	*Bursera*	*Bursera simaruba*	GQ437774
Eudicotyledons	Burseraceae	*Bursera*	*Bursera simaruba*	GQ437775
Eudicotyledons	Burseraceae	*Bursera*	*Bursera simaruba*	GQ437776
Eudicotyledons	Burseraceae	*Bursera*	*Bursera simaruba*	GQ437777
Eudicotyledons	Burseraceae	*Bursera*	*Bursera simaruba*	GQ437778
Eudicotyledons	Burseraceae	*Bursera*	*Bursera simaruba*	GQ437779
Eudicotyledons	Burseraceae	*Bursera*	*Bursera simaruba*	GQ437780
Eudicotyledons	Burseraceae	*Bursera*	*Bursera simaruba*	GQ437781
Eudicotyledons	Burseraceae	*Bursera*	*Bursera simaruba*	GQ437782
Eudicotyledons	Burseraceae	*Bursera*	*Bursera simaruba*	GQ437783
Eudicotyledons	Burseraceae	*Bursera*	*Bursera simaruba*	GQ437784
Eudicotyledons	Burseraceae	*Bursera*	*Bursera simaruba*	GQ437785
Eudicotyledons	Burseraceae	*Bursera*	*Bursera simaruba*	GQ437786
Eudicotyledons	Burseraceae	*Bursera*	*Bursera simaruba*	GQ437787
Eudicotyledons	Burseraceae	*Bursera*	*Bursera simaruba*	GQ437788
Eudicotyledons	Burseraceae	*Bursera*	*Bursera simaruba*	GQ437789
Eudicotyledons	Burseraceae	*Bursera*	*Bursera simaruba*	GQ437790
Eudicotyledons	Burseraceae	*Bursera*	*Bursera simaruba*	GQ437791
Eudicotyledons	Burseraceae	*Bursera*	*Bursera simaruba*	GQ437792
Eudicotyledons	Burseraceae	*Bursera*	*Bursera simaruba*	GQ437793
Eudicotyledons	Burseraceae	*Bursera*	*Bursera simaruba*	GQ437794
Eudicotyledons	Burseraceae	*Bursera*	*Bursera simaruba*	GQ437795
Eudicotyledons	Burseraceae	*Bursera*	*Bursera simaruba*	GQ437796
Eudicotyledons	Burseraceae	*Bursera*	*Bursera simaruba*	GQ437797
Eudicotyledons	Burseraceae	*Bursera*	*Bursera simaruba*	GQ437798
Eudicotyledons	Burseraceae	*Bursera*	*Bursera simaruba*	GQ437799
Eudicotyledons	Burseraceae	*Bursera*	*Bursera simaruba*	GQ437800
Eudicotyledons	Burseraceae	*Bursera*	*Bursera simaruba*	GQ437801
Eudicotyledons	Burseraceae	*Bursera*	*Bursera simaruba*	GQ437802
Eudicotyledons	Burseraceae	*Bursera*	*Bursera simaruba*	GQ437803
Eudicotyledons	Burseraceae	*Bursera*	*Bursera simaruba*	GQ437804
Eudicotyledons	Burseraceae	*Bursera*	*Bursera simaruba*	GQ437805
Eudicotyledons	Burseraceae	*Bursera*	*Bursera simaruba*	GQ437806
Eudicotyledons	Burseraceae	*Bursera*	*Bursera simaruba*	GQ437807
Eudicotyledons	Burseraceae	*Bursera*	*Bursera simaruba*	GQ437808
Eudicotyledons	Burseraceae	*Bursera*	*Bursera simaruba*	GQ437809
Eudicotyledons	Burseraceae	*Bursera*	*Bursera simaruba*	GQ437810
Eudicotyledons	Burseraceae	*Bursera*	*Bursera simaruba*	GQ437811
Eudicotyledons	Burseraceae	*Bursera*	*Bursera simaruba*	AY309401
Eudicotyledons	Burseraceae	*Bursera*	*Bursera simaruba*	AY309402
Eudicotyledons	Solanaceae	*Capsicum*	*Capsicum chinense*	EF537224
Eudicotyledons	Solanaceae	*Capsicum*	*Capsicum chinense*	EF537225
Eudicotyledons	Solanaceae	*Capsicum*	*Capsicum chinense*	EF537226
Eudicotyledons	Solanaceae	*Capsicum*	*Capsicum chinense*	JF276755
Eudicotyledons	Solanaceae	*Capsicum*	*Capsicum chinense*	JF276756
Eudicotyledons	Solanaceae	*Capsicum*	*Capsicum chinense*	JF276757
Eudicotyledons	Brassicaceae	*Capsella*	*Capsella rubella*	FR822350
Eudicotyledons	Brassicaceae	*Capsella*	*Capsella rubella*	FR822351

Eudicotyledons	Araliaceae	*Hydrocotyle*	*Hydrocotyle vulgaris*	FM207079
Eudicotyledons	Araliaceae	*Hydrocotyle*	*Hydrocotyle vulgaris*	FM207080
Eudicotyledons	Araliaceae	*Hydrocotyle*	*Hydrocotyle vulgaris*	FM207081
Eudicotyledons	Araliaceae	*Hydrocotyle*	*Hydrocotyle vulgaris*	FM207082
Eudicotyledons	Araliaceae	*Hydrocotyle*	*Hydrocotyle vulgaris*	FM207083
Eudicotyledons	Araliaceae	*Hydrocotyle*	*Hydrocotyle vulgaris*	FM207084
Eudicotyledons	Adoxaceae	*Viburnum*	*Viburnum opulus*	GU562393
Eudicotyledons	Adoxaceae	*Viburnum*	*Viburnum opulus*	EU750611
Eudicotyledons	Adoxaceae	*Viburnum*	*Viburnum opulus*	EU750612
Eudicotyledons	Araliaceae	*Hedera*	*Hedera algeriensis*	AY163508
Eudicotyledons	Araliaceae	*Hedera*	*Hedera algeriensis*	GU054838
Eudicotyledons	Araliaceae	*Hedera*	*Hedera hibernica*	AY163520
Eudicotyledons	Araliaceae	*Hedera*	*Hedera hibernica*	AY163521
Eudicotyledons	Araliaceae	*Hedera*	*Hedera hibernica*	FN675789
Eudicotyledons	Araliaceae	*Hedera*	*Hedera pastuchovii*	AY163509
Eudicotyledons	Araliaceae	*Hedera*	*Hedera pastuchovii*	GU054799
Eudicotyledons	Araliaceae	*Hedera*	*Hedera rhombea*	AY163510
Eudicotyledons	Araliaceae	*Hedera*	*Hedera rhombea*	GU054798
Eudicotyledons	Loasaceae	*Mentzelia*	*Mentzelia lindleyi*	FJ918163
Eudicotyledons	Loasaceae	*Mentzelia*	*Mentzelia lindleyi*	FJ918164
Eudicotyledons	Loasaceae	*Mentzelia*	*Mentzelia lindleyi*	FJ918165
Eudicotyledons	Loasaceae	*Mentzelia*	*Mentzelia lindleyi*	JF321278
Eudicotyledons	Apiaceae	*Eryngium*	*Eryngium alpinum*	GQ385229
Eudicotyledons	Apiaceae	*Eryngium*	*Eryngium alpinum*	GQ385230
Eudicotyledons	Apiaceae	*Eryngium*	*Eryngium alpinum*	GQ385231
Eudicotyledons	Apiaceae	*Eryngium*	*Eryngium alpinum*	GQ385232
Eudicotyledons	Apiaceae	*Eryngium*	*Eryngium alpinum*	GQ385233
Eudicotyledons	Apiaceae	*Eryngium*	*Eryngium alpinum*	GQ385234
Eudicotyledons	Apiaceae	*Eryngium*	*Eryngium alpinum*	GQ385235
Eudicotyledons	Apiaceae	*Eryngium*	*Eryngium alpinum*	GQ385236
Eudicotyledons	Apiaceae	*Eryngium*	*Eryngium alpinum*	GQ385237
Eudicotyledons	Apiaceae	*Eryngium*	*Eryngium alpinum*	GQ385238
Eudicotyledons	Apiaceae	*Eryngium*	*Eryngium alpinum*	GQ385239
Eudicotyledons	Asteraceae	*Erigeron*	*Erigeron annuus*	GU724265
Eudicotyledons	Asteraceae	*Erigeron*	*Erigeron annuus*	HQ596688
Eudicotyledons	Asteraceae	*Erigeron*	*Erigeron annuus*	EU337691
Eudicotyledons	Asteraceae	*Erigeron*	*Erigeron annuus*	EU337692
Eudicotyledons	Asteraceae	*Erigeron*	*Erigeron annuus*	EU337693
Eudicotyledons	Asteraceae	*Erigeron*	*Erigeron annuus*	EU750456
Eudicotyledons	Asteraceae	*Erigeron*	*Erigeron annuus*	EU750457
Eudicotyledons	Asteraceae	*Erigeron*	*Erigeron annuus*	EU750458
Eudicotyledons	Asteraceae	*Erigeron*	*Erigeron strigosus*	HQ596691
Eudicotyledons	Asteraceae	*Erigeron*	*Erigeron strigosus*	EU750459
Eudicotyledons	Asteraceae	*Erigeron*	*Erigeron strigosus*	EU750460
Eudicotyledons	Asteraceae	*Erigeron*	*Erigeron strigosus*	EU750461
Eudicotyledons	Asteraceae	*Erigeron*	*Erigeron strigosus*	EU750462
Eudicotyledons	Asteraceae	*Cirsium*	*Cirsium vulgare*	HQ596646
Eudicotyledons	Asteraceae	*Cirsium*	*Cirsium vulgare*	HQ596647
Eudicotyledons	Ranunculaceae	*Eranthis*	*Eranthis byunsanensis*	JF505810
Eudicotyledons	Ranunculaceae	*Eranthis*	*Eranthis byunsanensis*	JF505811
Eudicotyledons	Ranunculaceae	*Eranthis*	*Eranthis byunsanensis*	JF505812
Eudicotyledons	Ranunculaceae	*Eranthis*	*Eranthis byunsanensis*	JF505813
Eudicotyledons	Ranunculaceae	*Eranthis*	*Eranthis byunsanensis*	JF505814

Eudicotyledons	Ranunculaceae	*Eranthis*	*Eranthis byunsanensis*	JF505815
Eudicotyledons	Ranunculaceae	*Eranthis*	*Eranthis byunsanensis*	JF505816
Eudicotyledons	Ranunculaceae	*Eranthis*	*Eranthis byunsanensis*	JF505817
Eudicotyledons	Ranunculaceae	*Eranthis*	*Eranthis byunsanensis*	JF505818
Eudicotyledons	Ranunculaceae	*Eranthis*	*Eranthis byunsanensis*	JF505819
Eudicotyledons	Ranunculaceae	*Eranthis*	*Eranthis byunsanensis*	JF505820
Eudicotyledons	Ranunculaceae	*Eranthis*	*Eranthis byunsanensis*	JF505821
Eudicotyledons	Ranunculaceae	*Eranthis*	*Eranthis byunsanensis*	JF505822
Eudicotyledons	Ranunculaceae	*Eranthis*	*Eranthis byunsanensis*	JF505823
Eudicotyledons	Ranunculaceae	*Eranthis*	*Eranthis byunsanensis*	JF505824
Eudicotyledons	Ranunculaceae	*Eranthis*	*Eranthis byunsanensis*	JF505825
Eudicotyledons	Ranunculaceae	*Eranthis*	*Eranthis byunsanensis*	JF505826
Eudicotyledons	Ranunculaceae	*Eranthis*	*Eranthis byunsanensis*	JF505827
Eudicotyledons	Ranunculaceae	*Eranthis*	*Eranthis byunsanensis*	JF505828
Eudicotyledons	Ranunculaceae	*Eranthis*	*Eranthis byunsanensis*	JF505829
Eudicotyledons	Ranunculaceae	*Eranthis*	*Eranthis byunsanensis*	JF505830
Eudicotyledons	Ranunculaceae	*Eranthis*	*Eranthis byunsanensis*	JF505831
Eudicotyledons	Ranunculaceae	*Eranthis*	*Eranthis byunsanensis*	JF505832
Eudicotyledons	Ranunculaceae	*Eranthis*	*Eranthis byunsanensis*	JF505833
Eudicotyledons	Ranunculaceae	*Eranthis*	*Eranthis byunsanensis*	JF505834
Eudicotyledons	Ranunculaceae	*Eranthis*	*Eranthis stellata*	JF505808
Eudicotyledons	Ranunculaceae	*Eranthis*	*Eranthis stellata*	JF505809
Eudicotyledons	Vitaceae	*Vitis*	*Vitis riparia*	HQ596898
Eudicotyledons	Vitaceae	*Vitis*	*Vitis riparia*	HQ596899
Eudicotyledons	Vitaceae	*Vitis*	*Vitis riparia*	HQ108294
Eudicotyledons	Vitaceae	*Vitis*	*Vitis riparia*	HQ108295
Eudicotyledons	Vitaceae	*Vitis*	*Vitis riparia*	HQ108296
Eudicotyledons	Vitaceae	*Vitis*	*Vitis riparia*	HQ108298
Eudicotyledons	Vitaceae	*Vitis*	*Vitis riparia*	HQ108299
Eudicotyledons	Vitaceae	*Vitis*	*Vitis riparia*	HQ108300
Eudicotyledons	Vitaceae	*Vitis*	*Vitis riparia*	HQ108301
Eudicotyledons	Vitaceae	*Vitis*	*Vitis riparia*	HQ108302
Eudicotyledons	Vitaceae	*Vitis*	*Vitis riparia*	HQ656407
Eudicotyledons	Vitaceae	*Vitis*	*Vitis riparia*	HQ656409
Eudicotyledons	Vitaceae	*Vitis*	*Vitis riparia*	HQ656491
Eudicotyledons	Vitaceae	*Vitis*	*Vitis riparia*	JF437165
Eudicotyledons	Asteraceae	*Jacobaea*	*Jacobaea vulgaris*	FJ395449
Eudicotyledons	Asteraceae	*Jacobaea*	*Jacobaea vulgaris*	AY155657
Eudicotyledons	Asteraceae	*Santolina*	*Santolina rosmarinifolia*	JF345737
Eudicotyledons	Asteraceae	*Santolina*	*Santolina rosmarinifolia*	JF345751
Eudicotyledons	Asteraceae	*Santolina*	*Santolina rosmarinifolia*	JF345752
Eudicotyledons	Asteraceae	*Santolina*	*Santolina rosmarinifolia*	JF345753
Eudicotyledons	Asteraceae	*Santolina*	*Santolina rosmarinifolia*	JF345759
Eudicotyledons	Asteraceae	*Santolina*	*Santolina rosmarinifolia*	JF345760
Eudicotyledons	Asteraceae	*Santolina*	*Santolina rosmarinifolia*	JF345770
Eudicotyledons	Asteraceae	*Santolina*	*Santolina rosmarinifolia*	JF345771
Eudicotyledons	Aceraceae	*Acer*	*Acer fabri*	EF186780
Eudicotyledons	Aceraceae	*Acer*	*Acer fabri*	JN043722
Eudicotyledons	Aceraceae	*Acer*	*Acer fabri*	JN043723
Eudicotyledons	Aceraceae	*Acer*	*Acer fabri*	JN043724
Eudicotyledons	Aceraceae	*Acer*	*Acer fabri*	JN043725
Eudicotyledons	Aceraceae	*Acer*	*Acer fabri*	HM352719
Eudicotyledons	Apiaceae	*Heracleum*	*Heracleum moellendorffii*	GU967830

Eudicotyledons	Apiaceae	*Heracleum*	*Heracleum moellendorffii*	EU594940
Eudicotyledons	Saxifragaceae	*Mitella*	*Mitella acerina*	AB492622
Eudicotyledons	Saxifragaceae	*Mitella*	*Mitella acerina*	AB492623
Eudicotyledons	Saxifragaceae	*Mitella*	*Mitella acerina*	AB492624
Eudicotyledons	Saxifragaceae	*Mitella*	*Mitella acerina*	AB492625
Eudicotyledons	Saxifragaceae	*Mitella*	*Mitella acerina*	AB492626
Eudicotyledons	Saxifragaceae	*Mitella*	*Mitella acerina*	AB492627
Eudicotyledons	Saxifragaceae	*Mitella*	*Mitella japonica*	AB492506
Eudicotyledons	Saxifragaceae	*Mitella*	*Mitella japonica*	AB492507
Eudicotyledons	Saxifragaceae	*Mitella*	*Mitella japonica*	AB492508
Eudicotyledons	Saxifragaceae	*Mitella*	*Mitella japonica*	AB492509
Eudicotyledons	Saxifragaceae	*Mitella*	*Mitella japonica*	AB492510
Eudicotyledons	Saxifragaceae	*Mitella*	*Mitella japonica*	AB492511
Eudicotyledons	Saxifragaceae	*Mitella*	*Mitella japonica*	AB492512
Eudicotyledons	Saxifragaceae	*Mitella*	*Mitella japonica*	AB492513
Eudicotyledons	Saxifragaceae	*Mitella*	*Mitella japonica*	AB492514
Eudicotyledons	Saxifragaceae	*Mitella*	*Mitella japonica*	AB492515
Eudicotyledons	Saxifragaceae	*Mitella*	*Mitella japonica*	AB492516
Eudicotyledons	Saxifragaceae	*Mitella*	*Mitella japonica*	AB492517
Eudicotyledons	Saxifragaceae	*Mitella*	*Mitella japonica*	AB492518
Eudicotyledons	Saxifragaceae	*Mitella*	*Mitella japonica*	AB492519
Eudicotyledons	Saxifragaceae	*Mitella*	*Mitella japonica*	AB492520
Eudicotyledons	Saxifragaceae	*Mitella*	*Mitella japonica*	AB492521
Eudicotyledons	Saxifragaceae	*Mitella*	*Mitella japonica*	AB492522
Eudicotyledons	Saxifragaceae	*Mitella*	*Mitella japonica*	AB492523
Eudicotyledons	Saxifragaceae	*Mitella*	*Mitella japonica*	AB492524
Eudicotyledons	Saxifragaceae	*Mitella*	*Mitella japonica*	AB492525
Eudicotyledons	Saxifragaceae	*Mitella*	*Mitella japonica*	AB492526
Eudicotyledons	Saxifragaceae	*Mitella*	*Mitella japonica*	AB492527
Eudicotyledons	Saxifragaceae	*Mitella*	*Mitella japonica*	AB492528
Eudicotyledons	Saxifragaceae	*Mitella*	*Mitella japonica*	AB492529
Eudicotyledons	Saxifragaceae	*Mitella*	*Mitella japonica*	AB492530
Eudicotyledons	Saxifragaceae	*Mitella*	*Mitella japonica*	AB492531
Eudicotyledons	Saxifragaceae	*Mitella*	*Mitella japonica*	AB492532
Eudicotyledons	Saxifragaceae	*Mitella*	*Mitella japonica*	AB492533
Eudicotyledons	Saxifragaceae	*Mitella*	*Mitella japonica*	AB492534
Eudicotyledons	Saxifragaceae	*Mitella*	*Mitella pauciflora*	AB492591
Eudicotyledons	Saxifragaceae	*Mitella*	*Mitella pauciflora*	AB492592
Eudicotyledons	Saxifragaceae	*Mitella*	*Mitella pauciflora*	AB492593
Eudicotyledons	Saxifragaceae	*Mitella*	*Mitella pauciflora*	AB492594
Eudicotyledons	Saxifragaceae	*Mitella*	*Mitella pauciflora*	AB492595
Eudicotyledons	Saxifragaceae	*Mitella*	*Mitella pauciflora*	AB492596
Eudicotyledons	Saxifragaceae	*Mitella*	*Mitella pauciflora*	AB492597
Eudicotyledons	Saxifragaceae	*Mitella*	*Mitella pauciflora*	AB492598
Eudicotyledons	Saxifragaceae	*Mitella*	*Mitella pauciflora*	AB492599
Eudicotyledons	Saxifragaceae	*Mitella*	*Mitella pauciflora*	AB492600
Eudicotyledons	Saxifragaceae	*Mitella*	*Mitella pauciflora*	AB492601
Eudicotyledons	Saxifragaceae	*Mitella*	*Mitella pauciflora*	AB492602
Eudicotyledons	Saxifragaceae	*Mitella*	*Mitella pauciflora*	AB492603
Eudicotyledons	Saxifragaceae	*Mitella*	*Mitella pauciflora*	AB492604
Eudicotyledons	Saxifragaceae	*Mitella*	*Mitella pauciflora*	AB492605
Eudicotyledons	Saxifragaceae	*Mitella*	*Mitella pauciflora*	AB492606
Eudicotyledons	Saxifragaceae	*Mitella*	*Mitella pauciflora*	AB492607

Eudicotyledons	Saxifragaceae	*Mitella*	*Mitella pauciflora*	AB492608
Eudicotyledons	Saxifragaceae	*Mitella*	*Mitella pauciflora*	AB492609
Eudicotyledons	Saxifragaceae	*Mitella*	*Mitella pauciflora*	AB492610
Eudicotyledons	Saxifragaceae	*Mitella*	*Mitella pauciflora*	AB492611
Eudicotyledons	Saxifragaceae	*Mitella*	*Mitella yoshinagae*	AB492535
Eudicotyledons	Saxifragaceae	*Mitella*	*Mitella yoshinagae*	AB492536
Eudicotyledons	Saxifragaceae	*Mitella*	*Mitella yoshinagae*	AB492537
Eudicotyledons	Saxifragaceae	*Mitella*	*Mitella yoshinagae*	AB492538
Eudicotyledons	Saxifragaceae	*Mitella*	*Mitella yoshinagae*	AB492539
Eudicotyledons	Saxifragaceae	*Mitella*	*Mitella yoshinagae*	AB492540
Eudicotyledons	Saxifragaceae	*Mitella*	*Mitella yoshinagae*	AB492541
Eudicotyledons	Saxifragaceae	*Mitella*	*Mitella yoshinagae*	AB492542
Eudicotyledons	Saxifragaceae	*Mitella*	*Mitella yoshinagae*	AB492543
Eudicotyledons	Saxifragaceae	*Mitella*	*Mitella yoshinagae*	AB492544
Eudicotyledons	Saxifragaceae	*Mitella*	*Mitella yoshinagae*	AB492545
Eudicotyledons	Saxifragaceae	*Mitella*	*Mitella yoshinagae*	AB492546
Eudicotyledons	Saxifragaceae	*Mitella*	*Mitella yoshinagae*	AB492547
Eudicotyledons	Saxifragaceae	*Mitella*	*Mitella yoshinagae*	AB492548
Eudicotyledons	Rosaceae	*Fragaria*	*Fragaria orientalis*	GQ476767
Eudicotyledons	Rosaceae	*Fragaria*	*Fragaria orientalis*	GQ476768
Eudicotyledons	Rosaceae	*Fragaria*	*Fragaria orientalis*	GQ476769
Eudicotyledons	Aquifoliaceae	*Ilex*	*Ilex opaca*	EU359322
Eudicotyledons	Aquifoliaceae	*Ilex*	*Ilex opaca*	EF590704
Eudicotyledons	Rosaceae	*Prunus*	*Prunus mume*	AY500621
Eudicotyledons	Rosaceae	*Prunus*	*Prunus mume*	JN046609
Eudicotyledons	Rosaceae	*Prunus*	*Prunus mume*	JN046647
Eudicotyledons	Rosaceae	*Prunus*	*Prunus mume*	JN046648
Eudicotyledons	Rosaceae	*Prunus*	*Prunus mume*	JN046649
Eudicotyledons	Rosaceae	*Prunus*	*Prunus mume*	JN046650
Eudicotyledons	Rosaceae	*Prunus*	*Prunus mume*	JN046651
Eudicotyledons	Plantaginaceae	*Antirrhinum*	*Antirrhinum braun-blanquetii*	HM152884
Eudicotyledons	Plantaginaceae	*Antirrhinum*	*Antirrhinum braun-blanquetii*	HM152885
Eudicotyledons	Plantaginaceae	*Antirrhinum*	*Antirrhinum graniticum*	HM152899
Eudicotyledons	Plantaginaceae	*Antirrhinum*	*Antirrhinum graniticum*	HM152900
Eudicotyledons	Plantaginaceae	*Antirrhinum*	*Antirrhinum graniticum*	HM152901
Eudicotyledons	Plantaginaceae	*Antirrhinum*	*Antirrhinum molle*	HM152931
Eudicotyledons	Plantaginaceae	*Antirrhinum*	*Antirrhinum molle*	HM152932
Eudicotyledons	Plantaginaceae	*Antirrhinum*	*Antirrhinum siculum*	HM152948
Eudicotyledons	Plantaginaceae	*Antirrhinum*	*Antirrhinum siculum*	HM152949
Eudicotyledons	Plantaginaceae	*Antirrhinum*	*Antirrhinum valentinum*	HM152961
Eudicotyledons	Plantaginaceae	*Antirrhinum*	*Antirrhinum valentinum*	HM152962
Eudicotyledons	Plantaginaceae	*Antirrhinum*	*Antirrhinum valentinum*	HM152963
Eudicotyledons	Plantaginaceae	*Antirrhinum*	*Antirrhinum valentinum*	HM152964
Eudicotyledons	Plantaginaceae	*Antirrhinum*	*Antirrhinum valentinum*	HM152965
Eudicotyledons	Oleaceae	*Fraxinus*	*Fraxinus texensis*	HM367558
Eudicotyledons	Oleaceae	*Fraxinus*	*Fraxinus texensis*	HM367559
Eudicotyledons	Vitaceae	*Vitis*	*Vitis rotundifolia*	HM585791
Eudicotyledons	Vitaceae	*Vitis*	*Vitis rotundifolia*	HQ108337
Eudicotyledons	Vitaceae	*Vitis*	*Vitis rotundifolia*	HQ656476
Eudicotyledons	Vitaceae	*Vitis*	*Vitis rotundifolia*	HQ656477
Eudicotyledons	Vitaceae	*Vitis*	*Vitis rotundifolia*	HQ656478
Eudicotyledons	Vitaceae	*Vitis*	*Vitis rotundifolia*	JF437166
Eudicotyledons	Vitaceae	*Vitis*	*Vitis amurensis*	HQ108309

Eudicotyledons	Vitaceae	*Vitis*	*Vitis amurensis*	HQ656454
Eudicotyledons	Vitaceae	*Vitis*	*Vitis amurensis*	HQ656455
Eudicotyledons	Vitaceae	*Vitis*	*Vitis amurensis*	HQ656456
Eudicotyledons	Vitaceae	*Vitis*	*Vitis rupestris*	HQ108303
Eudicotyledons	Vitaceae	*Vitis*	*Vitis rupestris*	HQ108304
Eudicotyledons	Vitaceae	*Vitis*	*Vitis rupestris*	HQ108305
Eudicotyledons	Vitaceae	*Vitis*	*Vitis rupestris*	HQ108306
Eudicotyledons	Vitaceae	*Vitis*	*Vitis rupestris*	HQ108307
Eudicotyledons	Vitaceae	*Vitis*	*Vitis rupestris*	HQ108308
Eudicotyledons	Vitaceae	*Vitis*	*Vitis rupestris*	HQ656417
Eudicotyledons	Vitaceae	*Vitis*	*Vitis arizonica*	HQ108269
Eudicotyledons	Vitaceae	*Vitis*	*Vitis arizonica*	HQ656408
Eudicotyledons	Vitaceae	*Vitis*	*Vitis arizonica*	HQ656480
Eudicotyledons	Vitaceae	*Vitis*	*Vitis davidii*	JN802331
Eudicotyledons	Vitaceae	*Vitis*	*Vitis davidii*	HQ108316
Eudicotyledons	Vitaceae	*Vitis*	*Vitis davidii*	HQ108317
Eudicotyledons	Vitaceae	*Vitis*	*Vitis davidii*	HQ656440
Eudicotyledons	Vitaceae	*Vitis*	*Vitis davidii*	HQ656441
Eudicotyledons	Vitaceae	*Vitis*	*Vitis davidii*	HQ656450
Eudicotyledons	Vitaceae	*Vitis*	*Vitis labrusca*	HQ108293
Eudicotyledons	Vitaceae	*Vitis*	*Vitis labrusca*	HQ108297
Eudicotyledons	Vitaceae	*Vitis*	*Vitis labrusca*	HQ656421
Eudicotyledons	Vitaceae	*Vitis*	*Vitis labrusca*	HQ656481
Eudicotyledons	Rosaceae	*Chaenomeles*	*Chaenomeles speciosa*	JQ390646
Eudicotyledons	Rosaceae	*Chaenomeles*	*Chaenomeles speciosa*	JQ390649
Eudicotyledons	Rosaceae	*Chaenomeles*	*Chaenomeles speciosa*	JQ390650
Eudicotyledons	Rosaceae	*Chaenomeles*	*Chaenomeles speciosa*	JQ390651
Eudicotyledons	Rosaceae	*Chaenomeles*	*Chaenomeles speciosa*	JQ390653
Eudicotyledons	Rosaceae	*Malus*	*Malus angustifolia*	EF127153
Eudicotyledons	Rosaceae	*Malus*	*Malus angustifolia*	JQ390733
Eudicotyledons	Rosaceae	*Malus*	*Malus sargentii*	JQ390722
Eudicotyledons	Rosaceae	*Malus*	*Malus sargentii*	JQ390723
Eudicotyledons	Solanaceae	*Capsicum*	*Capsicum chacoense*	EF537208
Eudicotyledons	Solanaceae	*Capsicum*	*Capsicum chacoense*	EF537209
Eudicotyledons	Solanaceae	*Capsicum*	*Capsicum chacoense*	EF537210
Eudicotyledons	Brassicaceae	*Raphanus*	*Raphanus raphanistrum*	GQ248383
Eudicotyledons	Brassicaceae	*Raphanus*	*Raphanus raphanistrum*	EF590736
Eudicotyledons	Solanaceae	*Capsicum*	*Capsicum pubescens*	EF537221
Eudicotyledons	Solanaceae	*Capsicum*	*Capsicum pubescens*	EF537222
Eudicotyledons	Solanaceae	*Capsicum*	*Capsicum pubescens*	EF537223
Eudicotyledons	Rosaceae	*Prunus*	*Prunus spinosa*	FJ395549
Eudicotyledons	Rosaceae	*Prunus*	*Prunus spinosa*	FR865110
Eudicotyledons	Rosaceae	*Prunus*	*Prunus spinosa*	AY500615
Eudicotyledons	Rosaceae	*Rosa*	*Rosa acicularis*	DQ778741
Eudicotyledons	Rosaceae	*Rosa*	*Rosa acicularis*	DQ778742
Eudicotyledons	Rosaceae	*Rosa*	*Rosa acicularis*	DQ778743
Eudicotyledons	Ericaceae	*Rhododendron*	*Rhododendron mariesii*	HQ426992
Eudicotyledons	Ericaceae	*Rhododendron*	*Rhododendron mariesii*	JN046878
Eudicotyledons	Ericaceae	*Rhododendron*	*Rhododendron mariesii*	JN046879
Eudicotyledons	Ericaceae	*Rhododendron*	*Rhododendron mariesii*	HQ706995
Eudicotyledons	Ericaceae	*Rhododendron*	*Rhododendron simsii*	HQ415427
Eudicotyledons	Ericaceae	*Rhododendron*	*Rhododendron simsii*	HQ426991
Eudicotyledons	Ericaceae	*Rhododendron*	*Rhododendron simsii*	JN046961

Eudicotyledons	Ericaceae	*Rhododendron*	*Rhododendron simsii*	JN046962
Eudicotyledons	Ericaceae	*Rhododendron*	*Rhododendron simsii*	JN046963
Eudicotyledons	Ericaceae	*Rhododendron*	*Rhododendron simsii*	HQ707022
Eudicotyledons	Ericaceae	*Rhododendron*	*Rhododendron oldhamii*	HQ707008
Eudicotyledons	Ericaceae	*Rhododendron*	*Rhododendron oldhamii*	HQ707009
Eudicotyledons	Ericaceae	*Rhododendron*	*Rhododendron micranthum*	HQ706998
Eudicotyledons	Ericaceae	*Rhododendron*	*Rhododendron micranthum*	HQ706999
Eudicotyledons	Asteraceae	*Inula*	*Inula britannica*	GU724259
Eudicotyledons	Asteraceae	*Inula*	*Inula britannica*	GQ435106
Eudicotyledons	Asteraceae	*Inula*	*Inula britannica*	GQ435107
Eudicotyledons	Asteraceae	*Inula*	*Inula britannica*	AY215561
Eudicotyledons	Solanaceae	*Solanum*	*Solanum torvum*	JN407061
Eudicotyledons	Solanaceae	*Solanum*	*Solanum torvum*	JN407062
Eudicotyledons	Solanaceae	*Solanum*	*Solanum torvum*	JN407063
Eudicotyledons	Solanaceae	*Solanum*	*Solanum torvum*	JN407064
Eudicotyledons	Solanaceae	*Solanum*	*Solanum torvum*	GU135422
Eudicotyledons	Solanaceae	*Solanum*	*Solanum torvum*	JN047230
Eudicotyledons	Solanaceae	*Solanum*	*Solanum torvum*	JN047231
Eudicotyledons	Asteraceae	*Centaurea*	*Centaurea corymbosa*	DQ846180
Eudicotyledons	Asteraceae	*Centaurea*	*Centaurea corymbosa*	DQ846181
Eudicotyledons	Asteraceae	*Centaurea*	*Centaurea corymbosa*	DQ846182
Eudicotyledons	Asteraceae	*Centaurea*	*Centaurea corymbosa*	DQ846195
Eudicotyledons	Asteraceae	*Centaurea*	*Centaurea corymbosa*	DQ846196
Eudicotyledons	Asteraceae	*Centaurea*	*Centaurea corymbosa*	DQ846197
Eudicotyledons	Asteraceae	*Centaurea*	*Centaurea corymbosa*	DQ846198
Eudicotyledons	Asteraceae	*Centaurea*	*Centaurea corymbosa*	DQ846199
Eudicotyledons	Asteraceae	*Centaurea*	*Centaurea corymbosa*	DQ846200
Eudicotyledons	Asteraceae	*Centaurea*	*Centaurea corymbosa*	DQ846201
Eudicotyledons	Asteraceae	*Centaurea*	*Centaurea corymbosa*	DQ846202
Eudicotyledons	Asteraceae	*Centaurea*	*Centaurea corymbosa*	DQ846203
Eudicotyledons	Asteraceae	*Centaurea*	*Centaurea corymbosa*	DQ846231
Eudicotyledons	Asteraceae	*Centaurea*	*Centaurea corymbosa*	DQ846232
Eudicotyledons	Asteraceae	*Centaurea*	*Centaurea corymbosa*	DQ846233
Eudicotyledons	Asteraceae	*Centaurea*	*Centaurea corymbosa*	DQ846234
Eudicotyledons	Asteraceae	*Centaurea*	*Centaurea corymbosa*	DQ846235
Eudicotyledons	Asteraceae	*Centaurea*	*Centaurea corymbosa*	DQ846236
Eudicotyledons	Rosaceae	*Prunus*	*Prunus mandshurica*	AY500619
Eudicotyledons	Rosaceae	*Prunus*	*Prunus mandshurica*	JN046639
Eudicotyledons	Rosaceae	*Prunus*	*Prunus mandshurica*	JN046640
Eudicotyledons	Rosaceae	*Prunus*	*Prunus mandshurica*	JN046641
Eudicotyledons	Platanaceae	*Platanus*	*Platanus orientalis*	HE661218
Eudicotyledons	Platanaceae	*Platanus*	*Platanus orientalis*	HE661219
Eudicotyledons	Platanaceae	*Platanus*	*Platanus orientalis*	HE661220
Eudicotyledons	Platanaceae	*Platanus*	*Platanus orientalis*	HE661221
Eudicotyledons	Platanaceae	*Platanus*	*Platanus orientalis*	HE661222
Eudicotyledons	Plantaginaceae	*Veronica*	*Veronica thomsonii*	FJ848128
Eudicotyledons	Plantaginaceae	*Veronica*	*Veronica thomsonii*	FJ848130
Eudicotyledons	Amaranthaceae	*Amaranthus*	*Amaranthus albus*	JN043879
Eudicotyledons	Amaranthaceae	*Amaranthus*	*Amaranthus albus*	JN043880
Eudicotyledons	Amaranthaceae	*Amaranthus*	*Amaranthus albus*	JN043881
Eudicotyledons	Amaranthaceae	*Amaranthus*	*Amaranthus albus*	JN043882
Eudicotyledons	Amaranthaceae	*Amaranthus*	*Amaranthus retroflexus*	HQ596591
Eudicotyledons	Amaranthaceae	*Amaranthus*	*Amaranthus retroflexus*	JN043886

Eudicotyledons	Amaranthaceae	*Amaranthus*	*Amaranthus retroflexus*	JN043887
Eudicotyledons	Amaranthaceae	*Amaranthus*	*Amaranthus retroflexus*	JN043888
Eudicotyledons	Amaranthaceae	*Amaranthus*	*Amaranthus retroflexus*	JN043889
Eudicotyledons	Amaranthaceae	*Amaranthus*	*Amaranthus retroflexus*	JN043890
Eudicotyledons	Amaranthaceae	*Amaranthus*	*Amaranthus retroflexus*	JN043891
Eudicotyledons	Amaranthaceae	*Amaranthus*	*Amaranthus retroflexus*	JN043892
Eudicotyledons	Amaranthaceae	*Amaranthus*	*Amaranthus retroflexus*	JN043893
Eudicotyledons	Amaranthaceae	*Amaranthus*	*Amaranthus spinosus*	GQ248243
Eudicotyledons	Amaranthaceae	*Amaranthus*	*Amaranthus spinosus*	DQ006132
Eudicotyledons	Amaranthaceae	*Amaranthus*	*Amaranthus spinosus*	DQ006133
Eudicotyledons	Amaranthaceae	*Amaranthus*	*Amaranthus spinosus*	JN043894
Eudicotyledons	Amaranthaceae	*Amaranthus*	*Amaranthus spinosus*	JN043895
Eudicotyledons	Amaranthaceae	*Amaranthus*	*Amaranthus spinosus*	JN043896
Eudicotyledons	Amaranthaceae	*Amaranthus*	*Amaranthus spinosus*	JN043897
Eudicotyledons	Amaranthaceae	*Amaranthus*	*Amaranthus spinosus*	JN043898
Eudicotyledons	Amaranthaceae	*Amaranthus*	*Amaranthus spinosus*	JN043899
Eudicotyledons	Amaranthaceae	*Amaranthus*	*Amaranthus spinosus*	JN043900
Eudicotyledons	Amaranthaceae	*Amaranthus*	*Amaranthus spinosus*	JN043901
Eudicotyledons	Asteraceae	*Centaurea*	*Centaurea paniculata*	DQ846189
Eudicotyledons	Asteraceae	*Centaurea*	*Centaurea paniculata*	DQ846190
Eudicotyledons	Asteraceae	*Centaurea*	*Centaurea paniculata*	DQ846191
Eudicotyledons	Oleaceae	*Fraxinus*	*Fraxinus dipetala*	HM367415
Eudicotyledons	Oleaceae	*Fraxinus*	*Fraxinus dipetala*	HM367416
Eudicotyledons	Oleaceae	*Fraxinus*	*Fraxinus greggii*	HM367428
Eudicotyledons	Oleaceae	*Fraxinus*	*Fraxinus greggii*	HM367429
Eudicotyledons	Oleaceae	*Fraxinus*	*Fraxinus greggii*	HM367430
Eudicotyledons	Oleaceae	*Fraxinus*	*Fraxinus greggii*	HM367431
Eudicotyledons	Oleaceae	*Fraxinus*	*Fraxinus greggii*	HM367432
Eudicotyledons	Oleaceae	*Fraxinus*	*Fraxinus greggii*	HM367433
Eudicotyledons	Oleaceae	*Fraxinus*	*Fraxinus xanthoxyloides*	HM367580
Eudicotyledons	Oleaceae	*Fraxinus*	*Fraxinus xanthoxyloides*	HM367581
Eudicotyledons	Oleaceae	*Fraxinus*	*Fraxinus xanthoxyloides*	HM367582
Eudicotyledons	Oleaceae	*Fraxinus*	*Fraxinus xanthoxyloides*	HM367583
Eudicotyledons	Oleaceae	*Fraxinus*	*Fraxinus xanthoxyloides*	HM367584
Eudicotyledons	Oleaceae	*Fraxinus*	*Fraxinus xanthoxyloides*	HM367585
Eudicotyledons	Oleaceae	*Fraxinus*	*Fraxinus xanthoxyloides*	HM367586
Eudicotyledons	Rosaceae	*Rosa*	*Rosa phoenicia*	AB043944
Eudicotyledons	Rosaceae	*Rosa*	*Rosa phoenicia*	AB043945
Eudicotyledons	Rosaceae	*Rosa*	*Rosa phoenicia*	AB043946
Eudicotyledons	Rosaceae	*Prunus*	*Prunus virginiana*	GU562409
Eudicotyledons	Rosaceae	*Prunus*	*Prunus virginiana*	HQ596804
Eudicotyledons	Rosaceae	*Prunus*	*Prunus virginiana*	GQ248381
Eudicotyledons	Rosaceae	*Prunus*	*Prunus virginiana*	EF590734
Eudicotyledons	Rosaceae	*Prunus*	*Prunus virginiana*	AY500634
Eudicotyledons	Grossulariaceae	*Ribes*	*Ribes alpinum*	HQ596813
Eudicotyledons	Grossulariaceae	*Ribes*	*Ribes alpinum*	AY138094
Eudicotyledons	Grossulariaceae	*Ribes*	*Ribes uva-crispa*	GU361913
Eudicotyledons	Grossulariaceae	*Ribes*	*Ribes uva-crispa*	AY138077
Eudicotyledons	Rosaceae	*Rosa*	*Rosa arvensis*	DQ778748
Eudicotyledons	Rosaceae	*Rosa*	*Rosa arvensis*	FJ395545
Eudicotyledons	Gentianaceae	*Swertia*	*Swertia macrosperma*	JN047287
Eudicotyledons	Gentianaceae	*Swertia*	*Swertia macrosperma*	JN047288
Eudicotyledons	Gentianaceae	*Swertia*	*Swertia macrosperma*	JN047289

Eudicotyledons	Gentianaceae	*Swertia*	*Swertia macrosperma*	JN047290
Eudicotyledons	Gentianaceae	*Swertia*	*Swertia macrosperma*	JN047291
Eudicotyledons	Gentianaceae	*Swertia*	*Swertia punicea*	JN047292
Eudicotyledons	Gentianaceae	*Swertia*	*Swertia punicea*	JN047293
Eudicotyledons	Gentianaceae	*Swertia*	*Swertia punicea*	JN047294
Eudicotyledons	Gentianaceae	*Swertia*	*Swertia punicea*	JN047295
Eudicotyledons	Gentianaceae	*Swertia*	*Swertia punicea*	JN047296
Eudicotyledons	Polygonaceae	*Rheum*	*Rheum officinale*	GQ435476
Eudicotyledons	Polygonaceae	*Rheum*	*Rheum officinale*	GQ435477
Eudicotyledons	Polygonaceae	*Rheum*	*Rheum officinale*	GQ435478
Eudicotyledons	Polygonaceae	*Rheum*	*Rheum officinale*	EU554041
Eudicotyledons	Polygonaceae	*Rheum*	*Rheum palmatum*	GQ435479
Eudicotyledons	Polygonaceae	*Rheum*	*Rheum palmatum*	EU554042
Eudicotyledons	Polygonaceae	*Rheum*	*Rheum tanguticum*	GQ435480
Eudicotyledons	Polygonaceae	*Rheum*	*Rheum tanguticum*	GQ435481
Eudicotyledons	Polygonaceae	*Rheum*	*Rheum tanguticum*	GQ435482
Eudicotyledons	Polygonaceae	*Rheum*	*Rheum tanguticum*	EU554043
Eudicotyledons	Polygonaceae	*Rumex*	*Rumex patientia*	JN047055
Eudicotyledons	Polygonaceae	*Rumex*	*Rumex patientia*	JN047056
Eudicotyledons	Polygonaceae	*Rumex*	*Rumex patientia*	JN047057
Eudicotyledons	Gentianaceae	*Swertia*	*Swertia erythrosticta*	JN047282
Eudicotyledons	Gentianaceae	*Swertia*	*Swertia erythrosticta*	JN047283
Eudicotyledons	Fabaceae	*Acacia*	*Acacia catechu*	GQ434968
Eudicotyledons	Fabaceae	*Acacia*	*Acacia catechu*	GQ434969
Eudicotyledons	Fabaceae	*Acacia*	*Acacia catechu*	GQ434970
Eudicotyledons	Fabaceae	*Acacia*	*Acacia melanoxylon*	AF195712
Eudicotyledons	Fabaceae	*Acacia*	*Acacia melanoxylon*	FJ808525
Eudicotyledons	Fabaceae	*Acacia*	*Acacia melanoxylon*	FJ808526
Eudicotyledons	Fabaceae	*Acacia*	*Acacia melanoxylon*	FJ808527
Eudicotyledons	Fabaceae	*Acacia*	*Acacia melanoxylon*	FJ808528
Eudicotyledons	Fabaceae	*Acacia*	*Acacia melanoxylon*	FJ808532
Eudicotyledons	Fabaceae	*Acacia*	*Acacia melanoxylon*	FJ808533
Eudicotyledons	Fabaceae	*Acacia*	*Acacia melanoxylon*	FJ808534
Eudicotyledons	Fabaceae	*Acacia*	*Acacia nilotica*	AF524993
Eudicotyledons	Fabaceae	*Acacia*	*Acacia nilotica*	FJ808555
Eudicotyledons	Fabaceae	*Acacia*	*Acacia nilotica*	FJ808557
Eudicotyledons	Fabaceae	*Acacia*	*Acacia nilotica*	FJ808558
Eudicotyledons	Fabaceae	*Acacia*	*Acacia nilotica*	FJ808559
Eudicotyledons	Fabaceae	*Acacia*	*Acacia nilotica*	FJ808562
Eudicotyledons	Fabaceae	*Acacia*	*Acacia nilotica*	FJ808563
Eudicotyledons	Fabaceae	*Acacia*	*Acacia nilotica*	FJ808565
Eudicotyledons	Fabaceae	*Acacia*	*Acacia nilotica*	FJ808566
Eudicotyledons	Fabaceae	*Acacia*	*Acacia nilotica*	GQ872326
Eudicotyledons	Fabaceae	*Acacia*	*Acacia senegal*	AF195718
Eudicotyledons	Fabaceae	*Acacia*	*Acacia senegal*	AF524996
Eudicotyledons	Fabaceae	*Acacia*	*Acacia tortilis*	EU213786
Eudicotyledons	Fabaceae	*Acacia*	*Acacia tortilis*	EU213787
Eudicotyledons	Fabaceae	*Acacia*	*Acacia tortilis*	EU213788
Eudicotyledons	Fabaceae	*Acacia*	*Acacia tortilis*	AF524994
Eudicotyledons	Fabaceae	*Acacia*	*Acacia tortilis*	GQ872341
Eudicotyledons	Fabaceae	*Acacia*	*Acacia schweinfurthii*	AF524999
Eudicotyledons	Fabaceae	*Acacia*	*Acacia schweinfurthii*	GQ872333
Eudicotyledons	Fabaceae	*Acacia*	*Acacia schweinfurthii*	EU811944

Eudicotyledons	Fabaceae	*Acacia*	*Acacia longifolia*	AF195722
Eudicotyledons	Fabaceae	*Acacia*	*Acacia longifolia*	FJ808515
Eudicotyledons	Fabaceae	*Acacia*	*Acacia longifolia*	FJ808516
Eudicotyledons	Fabaceae	*Acacia*	*Acacia longifolia*	FJ808517
Eudicotyledons	Fabaceae	*Acacia*	*Acacia longifolia*	FJ808518
Eudicotyledons	Fabaceae	*Acacia*	*Acacia longifolia*	FJ808519
Eudicotyledons	Fabaceae	*Acacia*	*Acacia longifolia*	FJ808520
Eudicotyledons	Fabaceae	*Acacia*	*Acacia longifolia*	FJ808521
Eudicotyledons	Fabaceae	*Acacia*	*Acacia longifolia*	FJ808522
Eudicotyledons	Fabaceae	*Acacia*	*Acacia longifolia*	FJ808523
Eudicotyledons	Asteraceae	*Arctium*	*Arctium minus*	FJ395501
Eudicotyledons	Asteraceae	*Arctium*	*Arctium minus*	HQ596603
Eudicotyledons	Asteraceae	*Carlina*	*Carlina gummifera*	HE659555
Eudicotyledons	Asteraceae	*Carlina*	*Carlina gummifera*	EU571447
Eudicotyledons	Asteraceae	*Carlina*	*Carlina lanata*	HE602472
Eudicotyledons	Asteraceae	*Carlina*	*Carlina lanata*	EU571446
Eudicotyledons	Plantaginaceae	*Antirrhinum*	*Antirrhinum meonanthum*	HM152926
Eudicotyledons	Plantaginaceae	*Antirrhinum*	*Antirrhinum meonanthum*	HM152927
Eudicotyledons	Asteraceae	*Centaurea*	*Centaurea prolongi*	FJ459721
Eudicotyledons	Asteraceae	*Centaurea*	*Centaurea prolongi*	FJ459722
Eudicotyledons	Aquifoliaceae	*Ilex*	*Ilex cornuta*	GQ434981
Eudicotyledons	Aquifoliaceae	*Ilex*	*Ilex cornuta*	GQ434982
Eudicotyledons	Aquifoliaceae	*Ilex*	*Ilex cornuta*	EU359378
Eudicotyledons	Aquifoliaceae	*Ilex*	*Ilex cornuta*	JN044938
Eudicotyledons	Aquifoliaceae	*Ilex*	*Ilex cornuta*	JN044939
Eudicotyledons	Aquifoliaceae	*Ilex*	*Ilex cornuta*	JN044940
Eudicotyledons	Aquifoliaceae	*Ilex*	*Ilex cornuta*	JN044941
Eudicotyledons	Aquifoliaceae	*Ilex*	*Ilex cornuta*	JN044942
Eudicotyledons	Aquifoliaceae	*Ilex*	*Ilex cornuta*	JN044943
Eudicotyledons	Gentianaceae	*Swertia*	*Swertia bimaculata*	JN047270
Eudicotyledons	Gentianaceae	*Swertia*	*Swertia bimaculata*	JN047271
Eudicotyledons	Gentianaceae	*Swertia*	*Swertia cincta*	JN047272
Eudicotyledons	Gentianaceae	*Swertia*	*Swertia cincta*	JN047273
Eudicotyledons	Gentianaceae	*Swertia*	*Swertia cincta*	JN047274
Eudicotyledons	Gentianaceae	*Swertia*	*Swertia cincta*	JN047275
Eudicotyledons	Gentianaceae	*Swertia*	*Swertia cincta*	JN047276
Eudicotyledons	Gentianaceae	*Swertia*	*Swertia cincta*	JN047277
Eudicotyledons	Asteraceae	*Arnica*	*Arnica dealbata*	AM690595
Eudicotyledons	Asteraceae	*Arnica*	*Arnica dealbata*	AY215626
Eudicotyledons	Asteraceae	*Arnica*	*Arnica longifolia*	AM690563
Eudicotyledons	Asteraceae	*Arnica*	*Arnica longifolia*	AM690586
Eudicotyledons	Rosaceae	*Prunus*	*Prunus mira*	AY500627
Eudicotyledons	Rosaceae	*Prunus*	*Prunus mira*	JN046642
Eudicotyledons	Rosaceae	*Prunus*	*Prunus mira*	JN046643
Eudicotyledons	Rosaceae	*Prunus*	*Prunus mira*	JN046644
Eudicotyledons	Rosaceae	*Prunus*	*Prunus mira*	JN046645
Eudicotyledons	Rosaceae	*Prunus*	*Prunus mira*	JN046646
Eudicotyledons	Rosaceae	*Prunus*	*Prunus davidiana*	AY500626
Eudicotyledons	Rosaceae	*Prunus*	*Prunus davidiana*	JN046610
Eudicotyledons	Rosaceae	*Prunus*	*Prunus davidiana*	JN046611
Eudicotyledons	Rosaceae	*Prunus*	*Prunus davidiana*	JN046612
Eudicotyledons	Rosaceae	*Prunus*	*Prunus davidiana*	JN046613
Eudicotyledons	Rosaceae	*Prunus*	*Prunus davidiana*	JN046614

Eudicotyledons	Rosaceae	*Prunus*	*Prunus mexicana*	AY500596
Eudicotyledons	Rosaceae	*Prunus*	*Prunus mexicana*	AY500599
Eudicotyledons	Rosaceae	*Prunus*	*Prunus sibirica*	GQ435274
Eudicotyledons	Rosaceae	*Prunus*	*Prunus sibirica*	JN046662
Eudicotyledons	Rosaceae	*Prunus*	*Prunus sibirica*	JN046663
Eudicotyledons	Rosaceae	*Prunus*	*Prunus sibirica*	JN046664
Eudicotyledons	Rosaceae	*Prunus*	*Prunus sibirica*	JN046665
Eudicotyledons	Rosaceae	*Prunus*	*Prunus sibirica*	JN046666
Eudicotyledons	Rosaceae	*Prunus*	*Prunus sibirica*	JN046667
Eudicotyledons	Vitaceae	*Vitis*	*Vitis flexuosa*	JQ182524
Eudicotyledons	Vitaceae	*Vitis*	*Vitis flexuosa*	HM585789
Eudicotyledons	Vitaceae	*Vitis*	*Vitis flexuosa*	HQ108312
Eudicotyledons	Vitaceae	*Vitis*	*Vitis flexuosa*	HQ656442
Eudicotyledons	Vitaceae	*Vitis*	*Vitis flexuosa*	HQ656453
Eudicotyledons	Vitaceae	*Vitis*	*Vitis flexuosa*	HQ656463
Eudicotyledons	Vitaceae	*Vitis*	*Vitis flexuosa*	HQ656466
Eudicotyledons	Vitaceae	*Vitis*	*Vitis thunbergii*	HQ108315
Eudicotyledons	Vitaceae	*Vitis*	*Vitis thunbergii*	JF437167
Eudicotyledons	Apiaceae	*Eryngium*	*Eryngium giganteum*	GQ385241
Eudicotyledons	Apiaceae	*Eryngium*	*Eryngium giganteum*	FJ475136
Eudicotyledons	Passifloraceae	*Passiflora*	*Passiflora foetida*	AY032814
Eudicotyledons	Passifloraceae	*Passiflora*	*Passiflora foetida*	AY220136
Eudicotyledons	Passifloraceae	*Passiflora*	*Passiflora foetida*	DQ238759
Eudicotyledons	Passifloraceae	*Passiflora*	*Passiflora elegans*	AY032806
Eudicotyledons	Passifloraceae	*Passiflora*	*Passiflora elegans*	AY219300
Eudicotyledons	Passifloraceae	*Passiflora*	*Passiflora elegans*	AY219301
Eudicotyledons	Passifloraceae	*Passiflora*	*Passiflora elegans*	AY219302
Eudicotyledons	Passifloraceae	*Passiflora*	*Passiflora elegans*	AY219303
Eudicotyledons	Passifloraceae	*Passiflora*	*Passiflora elegans*	AY219304
Eudicotyledons	Passifloraceae	*Passiflora*	*Passiflora elegans*	AY219305
Eudicotyledons	Passifloraceae	*Passiflora*	*Passiflora elegans*	AY219306
Eudicotyledons	Passifloraceae	*Passiflora*	*Passiflora elegans*	AY219307
Eudicotyledons	Passifloraceae	*Passiflora*	*Passiflora elegans*	AY219308
Eudicotyledons	Passifloraceae	*Passiflora*	*Passiflora elegans*	AY219309
Eudicotyledons	Passifloraceae	*Passiflora*	*Passiflora elegans*	AY219310
Eudicotyledons	Passifloraceae	*Passiflora*	*Passiflora actinia*	AY032807
Eudicotyledons	Passifloraceae	*Passiflora*	*Passiflora actinia*	AY219288
Eudicotyledons	Passifloraceae	*Passiflora*	*Passiflora actinia*	AY219289
Eudicotyledons	Passifloraceae	*Passiflora*	*Passiflora actinia*	AY219290
Eudicotyledons	Passifloraceae	*Passiflora*	*Passiflora actinia*	AY219291
Eudicotyledons	Passifloraceae	*Passiflora*	*Passiflora actinia*	AY219292
Eudicotyledons	Passifloraceae	*Passiflora*	*Passiflora actinia*	AY219293
Eudicotyledons	Passifloraceae	*Passiflora*	*Passiflora actinia*	AY219294
Eudicotyledons	Passifloraceae	*Passiflora*	*Passiflora actinia*	AY219295
Eudicotyledons	Passifloraceae	*Passiflora*	*Passiflora actinia*	AY219296
Eudicotyledons	Passifloraceae	*Passiflora*	*Passiflora actinia*	AY219297
Eudicotyledons	Passifloraceae	*Passiflora*	*Passiflora actinia*	AY219298
Eudicotyledons	Passifloraceae	*Passiflora*	*Passiflora actinia*	AY219299
Eudicotyledons	Passifloraceae	*Passiflora*	*Passiflora incarnata*	AY032810
Eudicotyledons	Passifloraceae	*Passiflora*	*Passiflora incarnata*	GQ248361
Eudicotyledons	Passifloraceae	*Passiflora*	*Passiflora incarnata*	EF590722
Eudicotyledons	Passifloraceae	*Passiflora*	*Passiflora incarnata*	DQ238757
Eudicotyledons	Passifloraceae	*Passiflora*	*Passiflora galbana*	AY032817

Eudicotyledons	Passifloraceae	*Passiflora*	*Passiflora galbana*	AY220137
Eudicotyledons	Passifloraceae	*Passiflora*	*Passiflora tenuifila*	AY032813
Eudicotyledons	Passifloraceae	*Passiflora*	*Passiflora tenuifila*	AY220140
Eudicotyledons	Passifloraceae	*Passiflora*	*Passiflora caerulea*	AY032816
Eudicotyledons	Passifloraceae	*Passiflora*	*Passiflora caerulea*	AY220135
Eudicotyledons	Cornaceae	*Nyssa*	*Nyssa yunnanensis*	EU734469
Eudicotyledons	Cornaceae	*Nyssa*	*Nyssa yunnanensis*	EU734470
Eudicotyledons	Plantaginaceae	*Veronica*	*Veronica serpyllifolia*	HQ596886
Eudicotyledons	Plantaginaceae	*Veronica*	*Veronica serpyllifolia*	HQ596887
Eudicotyledons	Vitaceae	*Leea*	*Leea indica*	HM585679
Eudicotyledons	Vitaceae	*Leea*	*Leea indica*	JF437123
Eudicotyledons	Plantaginaceae	*Veronica*	*Veronica spicata*	HQ327970
Eudicotyledons	Plantaginaceae	*Veronica*	*Veronica spicata*	HQ327971
Eudicotyledons	Plantaginaceae	*Veronica*	*Veronica spicata*	HQ327980
Eudicotyledons	Plantaginaceae	*Veronica*	*Veronica spicata*	HQ327983
Eudicotyledons	Plantaginaceae	*Veronica*	*Veronica spicata*	HQ327984
Eudicotyledons	Plantaginaceae	*Veronica*	*Veronica chamaedrys*	HM370828
Eudicotyledons	Plantaginaceae	*Veronica*	*Veronica chamaedrys*	HM370841
Eudicotyledons	Plantaginaceae	*Veronica*	*Veronica chamaedrys*	HM370842
Eudicotyledons	Plantaginaceae	*Veronica*	*Veronica chamaedrys*	HM370850
Eudicotyledons	Plantaginaceae	*Veronica*	*Veronica chamaedrys*	HM370861
Eudicotyledons	Plantaginaceae	*Veronica*	*Veronica chamaedrys*	HM370868
Eudicotyledons	Plantaginaceae	*Veronica*	*Veronica chamaedrys*	HM370871
Eudicotyledons	Plantaginaceae	*Veronica*	*Veronica chamaedrys*	HM370878
Eudicotyledons	Plantaginaceae	*Veronica*	*Veronica chamaedrys*	HM370882
Eudicotyledons	Plantaginaceae	*Veronica*	*Veronica chamaedrys*	HM370902
Eudicotyledons	Plantaginaceae	*Veronica*	*Veronica chamaedrys*	HM370948
Eudicotyledons	Plantaginaceae	*Veronica*	*Veronica chamaedrys*	HM370952
Eudicotyledons	Plantaginaceae	*Veronica*	*Veronica chamaedrys*	HM370954
Eudicotyledons	Plantaginaceae	*Veronica*	*Veronica chamaedrys*	HM370955
Eudicotyledons	Plantaginaceae	*Veronica*	*Veronica chamaedrys*	HM370956
Eudicotyledons	Solanaceae	*Capsicum*	*Capsicum tovarii*	EF537211
Eudicotyledons	Solanaceae	*Capsicum*	*Capsicum tovarii*	EF537212
Eudicotyledons	Solanaceae	*Capsicum*	*Capsicum tovarii*	EF537213
Eudicotyledons	Solanaceae	*Capsicum*	*Capsicum tovarii*	EF537214
Eudicotyledons	Solanaceae	*Capsicum*	*Capsicum tovarii*	EF537215
Eudicotyledons	Solanaceae	*Capsicum*	*Capsicum tovarii*	EF537216
Eudicotyledons	Rosaceae	*Prunus*	*Prunus ferganensis*	JN046620
Eudicotyledons	Rosaceae	*Prunus*	*Prunus ferganensis*	JN046621
Eudicotyledons	Rosaceae	*Prunus*	*Prunus ferganensis*	JN046622
Eudicotyledons	Rosaceae	*Prunus*	*Prunus ferganensis*	JN046623
Eudicotyledons	Rosaceae	*Prunus*	*Prunus ferganensis*	JN046624
Eudicotyledons	Oleaceae	*Fraxinus*	*Fraxinus angustifolia*	HE659569
Eudicotyledons	Oleaceae	*Fraxinus*	*Fraxinus angustifolia*	HM367365
Eudicotyledons	Oleaceae	*Fraxinus*	*Fraxinus angustifolia*	HM367366
Eudicotyledons	Oleaceae	*Fraxinus*	*Fraxinus angustifolia*	HM367367
Eudicotyledons	Oleaceae	*Fraxinus*	*Fraxinus angustifolia*	HM367368
Eudicotyledons	Oleaceae	*Fraxinus*	*Fraxinus angustifolia*	HM367369
Eudicotyledons	Oleaceae	*Fraxinus*	*Fraxinus angustifolia*	HM367370
Eudicotyledons	Oleaceae	*Fraxinus*	*Fraxinus angustifolia*	HM367371
Eudicotyledons	Oleaceae	*Fraxinus*	*Fraxinus angustifolia*	HM367372
Eudicotyledons	Oleaceae	*Fraxinus*	*Fraxinus angustifolia*	HM367373
Eudicotyledons	Oleaceae	*Fraxinus*	*Fraxinus angustifolia*	HM367374

Eudicotyledons	Oleaceae	*Fraxinus*	*Fraxinus angustifolia*	HM367375
Eudicotyledons	Oleaceae	*Fraxinus*	*Fraxinus angustifolia*	HM367376
Eudicotyledons	Oleaceae	*Fraxinus*	*Fraxinus angustifolia*	HM367377
Eudicotyledons	Oleaceae	*Fraxinus*	*Fraxinus angustifolia*	HM367378
Eudicotyledons	Gentianaceae	*Swertia*	*Swertia decora*	JN047278
Eudicotyledons	Gentianaceae	*Swertia*	*Swertia decora*	JN047279
Eudicotyledons	Gentianaceae	*Swertia*	*Swertia decora*	JN047280
Eudicotyledons	Gentianaceae	*Swertia*	*Swertia decora*	JN047281
Eudicotyledons	Gentianaceae	*Swertia*	*Swertia yunnanensis*	JN047301
Eudicotyledons	Gentianaceae	*Swertia*	*Swertia yunnanensis*	JN047302
Eudicotyledons	Aceraceae	*Acer*	*Acer davidii*	HM008573
Eudicotyledons	Aceraceae	*Acer*	*Acer davidii*	JN043713
Eudicotyledons	Aceraceae	*Acer*	*Acer davidii*	JN043714
Eudicotyledons	Aceraceae	*Acer*	*Acer davidii*	JN043715
Eudicotyledons	Aceraceae	*Acer*	*Acer davidii*	JN043716
Eudicotyledons	Aceraceae	*Acer*	*Acer davidii*	JN043717
Eudicotyledons	Aceraceae	*Acer*	*Acer davidii*	JN043718
Eudicotyledons	Aceraceae	*Acer*	*Acer davidii*	JN043719
Eudicotyledons	Aceraceae	*Acer*	*Acer davidii*	JN043720
Eudicotyledons	Aceraceae	*Acer*	*Acer davidii*	JN043721
Eudicotyledons	Rubiaceae	*Luculia*	*Luculia pinceana*	JN045267
Eudicotyledons	Rubiaceae	*Luculia*	*Luculia pinceana*	JN045268
Eudicotyledons	Rubiaceae	*Luculia*	*Luculia pinceana*	JN045269
Eudicotyledons	Rubiaceae	*Luculia*	*Luculia pinceana*	JN045270
Eudicotyledons	Rubiaceae	*Luculia*	*Luculia pinceana*	JN045271
Eudicotyledons	Aceraceae	*Acer*	*Acer erythranthum*	HM352717
Eudicotyledons	Aceraceae	*Acer*	*Acer erythranthum*	HM352718
Eudicotyledons	Begoniaceae	*Begonia*	*Begonia cirrosa*	JN044017
Eudicotyledons	Begoniaceae	*Begonia*	*Begonia cirrosa*	JN044018
Eudicotyledons	Begoniaceae	*Begonia*	*Begonia longicarpa*	JN044076
Eudicotyledons	Begoniaceae	*Begonia*	*Begonia longicarpa*	JN044077
Eudicotyledons	Begoniaceae	*Begonia*	*Begonia longicarpa*	JN044078
Eudicotyledons	Begoniaceae	*Begonia*	*Begonia longicarpa*	JN044079
Eudicotyledons	Begoniaceae	*Begonia*	*Begonia longicarpa*	JN044080
Eudicotyledons	Begoniaceae	*Begonia*	*Begonia ruboides*	JN044126
Eudicotyledons	Begoniaceae	*Begonia*	*Begonia ruboides*	JN044127
Eudicotyledons	Begoniaceae	*Begonia*	*Begonia ruboides*	JN044128
Eudicotyledons	Begoniaceae	*Begonia*	*Begonia ruboides*	JN044129
Eudicotyledons	Begoniaceae	*Begonia*	*Begonia ruboides*	JN044130
Eudicotyledons	Begoniaceae	*Begonia*	*Begonia ruboides*	JN044131
Eudicotyledons	Polygonaceae	*Rumex*	*Rumex crispus*	FJ395508
Eudicotyledons	Polygonaceae	*Rumex*	*Rumex crispus*	HQ596828
Eudicotyledons	Polygonaceae	*Rumex*	*Rumex crispus*	JN047048
Eudicotyledons	Polygonaceae	*Rumex*	*Rumex crispus*	JN047049
Eudicotyledons	Polygonaceae	*Rumex*	*Rumex nepalensis*	JN047050
Eudicotyledons	Polygonaceae	*Rumex*	*Rumex nepalensis*	JN047051
Eudicotyledons	Polygonaceae	*Rumex*	*Rumex nepalensis*	JN047052
Eudicotyledons	Grossulariaceae	*Ribes*	*Ribes alpestre*	AY138059
Eudicotyledons	Grossulariaceae	*Ribes*	*Ribes alpestre*	JN047033
Eudicotyledons	Grossulariaceae	*Ribes*	*Ribes alpestre*	JN047034
Eudicotyledons	Grossulariaceae	*Ribes*	*Ribes alpestre*	JN047035
Eudicotyledons	Grossulariaceae	*Ribes*	*Ribes alpestre*	JN047036
Eudicotyledons	Grossulariaceae	*Ribes*	*Ribes alpestre*	JN047037

Eudicotyledons	Grossulariaceae	*Ribes*	*Ribes alpestre*	JN047038
Eudicotyledons	Grossulariaceae	*Ribes*	*Ribes alpestre*	JN047039
Eudicotyledons	Grossulariaceae	*Ribes*	*Ribes lacustre*	HQ596817
Eudicotyledons	Grossulariaceae	*Ribes*	*Ribes lacustre*	AY138092
Eudicotyledons	Grossulariaceae	*Ribes*	*Ribes rubrum*	FJ395461
Eudicotyledons	Grossulariaceae	*Ribes*	*Ribes rubrum*	HQ596818
Eudicotyledons	Grossulariaceae	*Ribes*	*Ribes rubrum*	AY138108
Eudicotyledons	Ericaceae	*Rhododendron*	*Rhododendron arboreum*	JN046748
Eudicotyledons	Ericaceae	*Rhododendron*	*Rhododendron arboreum*	JN046749
Eudicotyledons	Ericaceae	*Rhododendron*	*Rhododendron arboreum*	JN046750
Eudicotyledons	Ericaceae	*Rhododendron*	*Rhododendron arboreum*	JN046751
Eudicotyledons	Fabaceae	*Acacia*	*Acacia acuminata*	HQ170465
Eudicotyledons	Fabaceae	*Acacia*	*Acacia acuminata*	HQ170466
Eudicotyledons	Fabaceae	*Acacia*	*Acacia ampliceps*	AF525003
Eudicotyledons	Fabaceae	*Acacia*	*Acacia ampliceps*	EU811962
Eudicotyledons	Cornaceae	*Cornus*	*Cornus sericea*	EU750452
Eudicotyledons	Cornaceae	*Cornus*	*Cornus sericea*	EU750453
Eudicotyledons	Cornaceae	*Cornus*	*Cornus sericea*	EU750454
Eudicotyledons	Cornaceae	*Cornus*	*Cornus sericea*	EU750455
Eudicotyledons	Asteraceae	*Santolina*	*Santolina africana*	JF345721
Eudicotyledons	Asteraceae	*Santolina*	*Santolina africana*	JF345722
Eudicotyledons	Asteraceae	*Santolina*	*Santolina africana*	JF345724
Eudicotyledons	Brassicaceae	*Brassica*	*Brassica maurorum*	AB669905
Eudicotyledons	Brassicaceae	*Brassica*	*Brassica maurorum*	AB669906
Eudicotyledons	Burseraceae	*Bursera*	*Bursera cinerea*	GQ377874
Eudicotyledons	Burseraceae	*Bursera*	*Bursera cinerea*	GQ377875
Eudicotyledons	Ericaceae	*Rhododendron*	*Rhododendron leptothrium*	JN046865
Eudicotyledons	Ericaceae	*Rhododendron*	*Rhododendron leptothrium*	JN046866
Eudicotyledons	Ericaceae	*Rhododendron*	*Rhododendron leptothrium*	JN046867
Eudicotyledons	Ericaceae	*Rhododendron*	*Rhododendron leptothrium*	JN046868
Eudicotyledons	Ericaceae	*Rhododendron*	*Rhododendron mackenzianum*	JN046872
Eudicotyledons	Ericaceae	*Rhododendron*	*Rhododendron mackenzianum*	JN046873
Eudicotyledons	Ericaceae	*Rhododendron*	*Rhododendron mackenzianum*	JN046874
Eudicotyledons	Ericaceae	*Rhododendron*	*Rhododendron spinuliferum*	JN046974
Eudicotyledons	Ericaceae	*Rhododendron*	*Rhododendron spinuliferum*	JN046975
Eudicotyledons	Ericaceae	*Rhododendron*	*Rhododendron vialii*	HQ707031
Eudicotyledons	Ericaceae	*Rhododendron*	*Rhododendron vialii*	HQ707032
Eudicotyledons	Myrtaceae	*Eucalyptus*	*Eucalyptus melliodora*	GQ248304
Eudicotyledons	Myrtaceae	*Eucalyptus*	*Eucalyptus melliodora*	EF590699
Eudicotyledons	Ericaceae	*Rhododendron*	*Rhododendron fortunei*	HQ706969
Eudicotyledons	Ericaceae	*Rhododendron*	*Rhododendron fortunei*	HQ706970
Eudicotyledons	Ericaceae	*Rhododendron*	*Rhododendron fortunei*	HQ706971
Eudicotyledons	Ericaceae	*Rhododendron*	*Rhododendron fortunei*	HQ706972
Eudicotyledons	Ericaceae	*Rhododendron*	*Rhododendron latoucheae*	HQ426990
Eudicotyledons	Ericaceae	*Rhododendron*	*Rhododendron latoucheae*	HQ706992
Eudicotyledons	Ericaceae	*Rhododendron*	*Rhododendron pachypodum*	JN046917
Eudicotyledons	Ericaceae	*Rhododendron*	*Rhododendron pachypodum*	JN046918
Eudicotyledons	Ericaceae	*Rhododendron*	*Rhododendron pachypodum*	JN046919
Eudicotyledons	Ericaceae	*Rhododendron*	*Rhododendron racemosum*	JN046932
Eudicotyledons	Ericaceae	*Rhododendron*	*Rhododendron racemosum*	JN046933
Eudicotyledons	Ericaceae	*Rhododendron*	*Rhododendron racemosum*	JN046934
Eudicotyledons	Ericaceae	*Rhododendron*	*Rhododendron trichocladum*	JN046996
Eudicotyledons	Ericaceae	*Rhododendron*	*Rhododendron trichocladum*	JN046997

Eudicotyledons	Ericaceae	*Rhododendron*	*Rhododendron trichocladum*	JN046998
Eudicotyledons	Aquifoliaceae	*Ilex*	*Ilex asprella*	JN406922
Eudicotyledons	Aquifoliaceae	*Ilex*	*Ilex asprella*	JN406923
Eudicotyledons	Aquifoliaceae	*Ilex*	*Ilex paraguariensis*	GQ248322
Eudicotyledons	Aquifoliaceae	*Ilex*	*Ilex paraguariensis*	EU359321
Eudicotyledons	Aquifoliaceae	*Ilex*	*Ilex paraguariensis*	EF590705
Eudicotyledons	Aquifoliaceae	*Ilex*	*Ilex pubescens*	HQ415418
Eudicotyledons	Aquifoliaceae	*Ilex*	*Ilex pubescens*	HQ426982
Eudicotyledons	Aquifoliaceae	*Ilex*	*Ilex purpurea*	HQ426983
Eudicotyledons	Aquifoliaceae	*Ilex*	*Ilex purpurea*	EU359331
Eudicotyledons	Fabaceae	*Wisteria*	*Wisteria brachybotrys*	EU424093
Eudicotyledons	Fabaceae	*Wisteria*	*Wisteria brachybotrys*	EU424094
Eudicotyledons	Asteraceae	*Ligularia*	*Ligularia hodgsonii*	GQ435102
Eudicotyledons	Asteraceae	*Ligularia*	*Ligularia hodgsonii*	AB369628
Eudicotyledons	Asteraceae	*Ligularia*	*Ligularia virgaurea*	JN045216
Eudicotyledons	Asteraceae	*Ligularia*	*Ligularia virgaurea*	JN045217
Eudicotyledons	Asteraceae	*Ligularia*	*Ligularia virgaurea*	JN045218
Eudicotyledons	Asteraceae	*Ligularia*	*Ligularia virgaurea*	JN045219
Eudicotyledons	Asteraceae	*Ligularia*	*Ligularia stenocephala*	AB369632
Eudicotyledons	Asteraceae	*Ligularia*	*Ligularia stenocephala*	EF538078
Eudicotyledons	Asteraceae	*Jacobaea*	*Jacobaea erucifolia*	FJ395460
Eudicotyledons	Asteraceae	*Jacobaea*	*Jacobaea erucifolia*	AY155652
Eudicotyledons	Fabaceae	*Leucaena*	*Leucaena diversifolia*	EF643867
Eudicotyledons	Fabaceae	*Leucaena*	*Leucaena diversifolia*	EF643869
Eudicotyledons	Fabaceae	*Leucaena*	*Leucaena diversifolia*	EF643881
Eudicotyledons	Fabaceae	*Leucaena*	*Leucaena diversifolia*	EF643882
Eudicotyledons	Fabaceae	*Leucaena*	*Leucaena pulverulenta*	EF643865
Eudicotyledons	Fabaceae	*Leucaena*	*Leucaena pulverulenta*	EF643872
Eudicotyledons	Ericaceae	*Rhododendron*	*Rhododendron pendulum*	JN046920
Eudicotyledons	Ericaceae	*Rhododendron*	*Rhododendron pendulum*	JN046921
Eudicotyledons	Ericaceae	*Rhododendron*	*Rhododendron virgatum*	JN047012
Eudicotyledons	Ericaceae	*Rhododendron*	*Rhododendron virgatum*	JN047013
Eudicotyledons	Ericaceae	*Rhododendron*	*Rhododendron virgatum*	HQ707033
Eudicotyledons	Ericaceae	*Rhododendron*	*Rhododendron virgatum*	HQ707034
Eudicotyledons	Loasaceae	*Mentzelia*	*Mentzelia albicaulis*	FJ918127
Eudicotyledons	Loasaceae	*Mentzelia*	*Mentzelia albicaulis*	FJ918128
Eudicotyledons	Loasaceae	*Mentzelia*	*Mentzelia albicaulis*	FJ918129
Eudicotyledons	Loasaceae	*Mentzelia*	*Mentzelia albicaulis*	FJ918130
Eudicotyledons	Loasaceae	*Mentzelia*	*Mentzelia dispersa*	FJ918150
Eudicotyledons	Loasaceae	*Mentzelia*	*Mentzelia dispersa*	FJ918151
Eudicotyledons	Loasaceae	*Mentzelia*	*Mentzelia dispersa*	JF321280
Eudicotyledons	Loasaceae	*Mentzelia*	*Mentzelia gracilenta*	FJ918155
Eudicotyledons	Loasaceae	*Mentzelia*	*Mentzelia gracilenta*	FJ918156
Eudicotyledons	Loasaceae	*Mentzelia*	*Mentzelia gracilenta*	FJ918157
Eudicotyledons	Begoniaceae	*Begonia*	*Begonia masoniana*	JN044096
Eudicotyledons	Begoniaceae	*Begonia*	*Begonia masoniana*	JN044097
Eudicotyledons	Begoniaceae	*Begonia*	*Begonia masoniana*	JN044098
Eudicotyledons	Fabaceae	*Acacia*	*Acacia galpinii*	AF525010
Eudicotyledons	Fabaceae	*Acacia*	*Acacia galpinii*	GQ872313
Eudicotyledons	Fabaceae	*Acacia*	*Acacia galpinii*	EU811968
Eudicotyledons	Fabaceae	*Albizia*	*Albizia kalkora*	HQ426986
Eudicotyledons	Fabaceae	*Albizia*	*Albizia kalkora*	AF524965
Eudicotyledons	Lamiaceae	*Ajuga*	*Ajuga ciliata*	JQ339253

Eudicotyledons	Lamiaceae	*Ajuga*	*Ajuga ciliata*	JQ339254
Eudicotyledons	Lamiaceae	*Ajuga*	*Ajuga ciliata*	JQ339255
Eudicotyledons	Lamiaceae	*Ajuga*	*Ajuga ciliata*	FJ513092
Eudicotyledons	Asteraceae	*Saussurea*	*Saussurea involucrata*	GQ435128
Eudicotyledons	Asteraceae	*Saussurea*	*Saussurea involucrata*	EF420893
Eudicotyledons	Plantaginaceae	*Veronica*	*Veronica vindobonensis*	HM370833
Eudicotyledons	Plantaginaceae	*Veronica*	*Veronica vindobonensis*	HM370855
Eudicotyledons	Plantaginaceae	*Veronica*	*Veronica vindobonensis*	HM370857
Eudicotyledons	Plantaginaceae	*Veronica*	*Veronica vindobonensis*	HM370858
Eudicotyledons	Plantaginaceae	*Veronica*	*Veronica vindobonensis*	HM370859
Eudicotyledons	Plantaginaceae	*Veronica*	*Veronica vindobonensis*	HM370864
Eudicotyledons	Plantaginaceae	*Veronica*	*Veronica vindobonensis*	HM370865
Eudicotyledons	Plantaginaceae	*Veronica*	*Veronica vindobonensis*	HM370866
Eudicotyledons	Plantaginaceae	*Veronica*	*Veronica vindobonensis*	HM370872
Eudicotyledons	Plantaginaceae	*Veronica*	*Veronica vindobonensis*	HM370874
Eudicotyledons	Plantaginaceae	*Veronica*	*Veronica vindobonensis*	HM370877
Eudicotyledons	Plantaginaceae	*Veronica*	*Veronica vindobonensis*	HM370879
Eudicotyledons	Plantaginaceae	*Veronica*	*Veronica vindobonensis*	HM370880
Eudicotyledons	Plantaginaceae	*Veronica*	*Veronica vindobonensis*	HM370881
Eudicotyledons	Plantaginaceae	*Veronica*	*Veronica vindobonensis*	HM370883
Eudicotyledons	Plantaginaceae	*Veronica*	*Veronica vindobonensis*	HM370910
Eudicotyledons	Plantaginaceae	*Veronica*	*Veronica vindobonensis*	HM370913
Eudicotyledons	Plantaginaceae	*Veronica*	*Veronica vindobonensis*	HM370914
Eudicotyledons	Plantaginaceae	*Veronica*	*Veronica vindobonensis*	HM370922
Eudicotyledons	Plantaginaceae	*Veronica*	*Veronica vindobonensis*	HM370923
Eudicotyledons	Plantaginaceae	*Veronica*	*Veronica vindobonensis*	HM370924
Eudicotyledons	Plantaginaceae	*Veronica*	*Veronica vindobonensis*	HM370926
Eudicotyledons	Plantaginaceae	*Veronica*	*Veronica vindobonensis*	HM370929
Eudicotyledons	Plantaginaceae	*Veronica*	*Veronica vindobonensis*	HM370934
Eudicotyledons	Plantaginaceae	*Veronica*	*Veronica vindobonensis*	HM370935
Eudicotyledons	Plantaginaceae	*Veronica*	*Veronica vindobonensis*	HM370941
Eudicotyledons	Ranunculaceae	*Eranthis*	*Eranthis pinnatifida*	JF505843
Eudicotyledons	Ranunculaceae	*Eranthis*	*Eranthis pinnatifida*	JF505844
Eudicotyledons	Ranunculaceae	*Eranthis*	*Eranthis pinnatifida*	JF505845
Eudicotyledons	Ranunculaceae	*Eranthis*	*Eranthis pinnatifida*	JF505846
Eudicotyledons	Ranunculaceae	*Eranthis*	*Eranthis pinnatifida*	JF505847
Eudicotyledons	Ranunculaceae	*Eranthis*	*Eranthis pinnatifida*	JF505848
Eudicotyledons	Solanaceae	*Solanum*	*Solanum aethiopicum*	HM016414
Eudicotyledons	Solanaceae	*Solanum*	*Solanum aethiopicum*	HM016415
Eudicotyledons	Solanaceae	*Solanum*	*Solanum aethiopicum*	HM016416
Eudicotyledons	Solanaceae	*Solanum*	*Solanum evolvulifolium*	HQ856093
Eudicotyledons	Solanaceae	*Solanum*	*Solanum evolvulifolium*	HQ856094
Eudicotyledons	Solanaceae	*Solanum*	*Solanum evolvulifolium*	HQ856095
Eudicotyledons	Solanaceae	*Solanum*	*Solanum evolvulifolium*	HQ856096
Eudicotyledons	Solanaceae	*Solanum*	*Solanum phaseoloides*	HQ856097
Eudicotyledons	Solanaceae	*Solanum*	*Solanum phaseoloides*	HQ856098
Eudicotyledons	Grossulariaceae	*Ribes*	*Ribes glaciale*	AY138097
Eudicotyledons	Grossulariaceae	*Ribes*	*Ribes glaciale*	JN047040
Eudicotyledons	Grossulariaceae	*Ribes*	*Ribes glaciale*	JN047041
Eudicotyledons	Grossulariaceae	*Ribes*	*Ribes cynosbati*	HQ596816
Eudicotyledons	Grossulariaceae	*Ribes*	*Ribes cynosbati*	AY138063
Eudicotyledons	Grossulariaceae	*Ribes*	*Ribes orientale*	AY138099
Eudicotyledons	Grossulariaceae	*Ribes*	*Ribes orientale*	JN047045

Eudicotyledons	Grossulariaceae	*Ribes*	*Ribes orientale*	JN047046
Eudicotyledons	Grossulariaceae	*Ribes*	*Ribes orientale*	JN047047
Eudicotyledons	Grossulariaceae	*Ribes*	*Ribes himalense*	AY138106
Eudicotyledons	Grossulariaceae	*Ribes*	*Ribes himalense*	JN047042
Eudicotyledons	Grossulariaceae	*Ribes*	*Ribes himalense*	JN047043
Eudicotyledons	Grossulariaceae	*Ribes*	*Ribes himalense*	JN047044
Eudicotyledons	Burseraceae	*Canarium*	*Canarium tramdenum*	HQ415435
Eudicotyledons	Burseraceae	*Canarium*	*Canarium tramdenum*	AY635380
Eudicotyledons	Oxalidaceae	*Oxalis*	*Oxalis corniculata*	JN407013
Eudicotyledons	Oxalidaceae	*Oxalis*	*Oxalis corniculata*	JN407014
Eudicotyledons	Oxalidaceae	*Oxalis*	*Oxalis corniculata*	JN407015
Eudicotyledons	Oxalidaceae	*Oxalis*	*Oxalis corniculata*	JN602087
Eudicotyledons	Solanaceae	*Solanum*	*Solanum viarum*	GU135370
Eudicotyledons	Solanaceae	*Solanum*	*Solanum viarum*	GU135408
Eudicotyledons	Araliaceae	*Hedera*	*Hedera sinensis*	GQ435394
Eudicotyledons	Araliaceae	*Hedera*	*Hedera sinensis*	AY163522
Eudicotyledons	Araliaceae	*Hedera*	*Hedera sinensis*	GU054813
Eudicotyledons	Araliaceae	*Hedera*	*Hedera sinensis*	HM755916
Eudicotyledons	Orobanchaceae	*Pedicularis*	*Pedicularis anas*	JN045888
Eudicotyledons	Orobanchaceae	*Pedicularis*	*Pedicularis anas*	JN045889
Eudicotyledons	Orobanchaceae	*Pedicularis*	*Pedicularis anas*	JN045890
Eudicotyledons	Orobanchaceae	*Pedicularis*	*Pedicularis anas*	JN045891
Eudicotyledons	Orobanchaceae	*Pedicularis*	*Pedicularis anas*	JN045892
Eudicotyledons	Orobanchaceae	*Pedicularis*	*Pedicularis axillaris*	JN045893
Eudicotyledons	Orobanchaceae	*Pedicularis*	*Pedicularis axillaris*	JN045894
Eudicotyledons	Orobanchaceae	*Pedicularis*	*Pedicularis axillaris*	JN045895
Eudicotyledons	Orobanchaceae	*Pedicularis*	*Pedicularis axillaris*	JN045896
Eudicotyledons	Orobanchaceae	*Pedicularis*	*Pedicularis brevilabris*	JN045901
Eudicotyledons	Orobanchaceae	*Pedicularis*	*Pedicularis brevilabris*	JN045902
Eudicotyledons	Orobanchaceae	*Pedicularis*	*Pedicularis brevilabris*	JN045903
Eudicotyledons	Orobanchaceae	*Pedicularis*	*Pedicularis brevilabris*	JN045904
Eudicotyledons	Orobanchaceae	*Pedicularis*	*Pedicularis cinerascens*	JN045911
Eudicotyledons	Orobanchaceae	*Pedicularis*	*Pedicularis cinerascens*	JN045912
Eudicotyledons	Orobanchaceae	*Pedicularis*	*Pedicularis confertiflora*	JN045917
Eudicotyledons	Orobanchaceae	*Pedicularis*	*Pedicularis confertiflora*	JN045918
Eudicotyledons	Orobanchaceae	*Pedicularis*	*Pedicularis confertiflora*	JN045919
Eudicotyledons	Orobanchaceae	*Pedicularis*	*Pedicularis confertiflora*	JN045920
Eudicotyledons	Orobanchaceae	*Pedicularis*	*Pedicularis confertiflora*	JN045921
Eudicotyledons	Orobanchaceae	*Pedicularis*	*Pedicularis cyathophylla*	JN045933
Eudicotyledons	Orobanchaceae	*Pedicularis*	*Pedicularis cyathophylla*	JN045934
Eudicotyledons	Orobanchaceae	*Pedicularis*	*Pedicularis cyathophylla*	JN045935
Eudicotyledons	Orobanchaceae	*Pedicularis*	*Pedicularis cyathophylla*	JN045936
Eudicotyledons	Orobanchaceae	*Pedicularis*	*Pedicularis cyathophylla*	JN045937
Eudicotyledons	Orobanchaceae	*Pedicularis*	*Pedicularis cyathophylloides*	JN045938
Eudicotyledons	Orobanchaceae	*Pedicularis*	*Pedicularis cyathophylloides*	JN045939
Eudicotyledons	Orobanchaceae	*Pedicularis*	*Pedicularis cyathophylloides*	JN045940
Eudicotyledons	Orobanchaceae	*Pedicularis*	*Pedicularis cyathophylloides*	JN045941
Eudicotyledons	Orobanchaceae	*Pedicularis*	*Pedicularis cyathophylloides*	JN045942
Eudicotyledons	Orobanchaceae	*Pedicularis*	*Pedicularis debilis*	JN045950
Eudicotyledons	Orobanchaceae	*Pedicularis*	*Pedicularis debilis*	JN045951
Eudicotyledons	Orobanchaceae	*Pedicularis*	*Pedicularis debilis*	JN045952
Eudicotyledons	Orobanchaceae	*Pedicularis*	*Pedicularis densispica*	JN045959
Eudicotyledons	Orobanchaceae	*Pedicularis*	*Pedicularis densispica*	JN045960

Eudicotyledons	Orobanchaceae	*Pedicularis*	*Pedicularis densispica*	JN045961
Eudicotyledons	Orobanchaceae	*Pedicularis*	*Pedicularis densispica*	JN045962
Eudicotyledons	Orobanchaceae	*Pedicularis*	*Pedicularis densispica*	JN045963
Eudicotyledons	Orobanchaceae	*Pedicularis*	*Pedicularis gyrorhyncha*	JN045999
Eudicotyledons	Orobanchaceae	*Pedicularis*	*Pedicularis gyrorhyncha*	JN046000
Eudicotyledons	Orobanchaceae	*Pedicularis*	*Pedicularis gyrorhyncha*	JN046001
Eudicotyledons	Orobanchaceae	*Pedicularis*	*Pedicularis integrifolia*	JN046004
Eudicotyledons	Orobanchaceae	*Pedicularis*	*Pedicularis integrifolia*	JN046005
Eudicotyledons	Orobanchaceae	*Pedicularis*	*Pedicularis integrifolia*	JN046006
Eudicotyledons	Orobanchaceae	*Pedicularis*	*Pedicularis integrifolia*	JN046007
Eudicotyledons	Orobanchaceae	*Pedicularis*	*Pedicularis lachnoglossa*	JN046018
Eudicotyledons	Orobanchaceae	*Pedicularis*	*Pedicularis lachnoglossa*	JN046019
Eudicotyledons	Orobanchaceae	*Pedicularis*	*Pedicularis lachnoglossa*	JN046020
Eudicotyledons	Orobanchaceae	*Pedicularis*	*Pedicularis lachnoglossa*	JN046021
Eudicotyledons	Orobanchaceae	*Pedicularis*	*Pedicularis lachnoglossa*	JN046022
Eudicotyledons	Orobanchaceae	*Pedicularis*	*Pedicularis longicaulis*	JN046029
Eudicotyledons	Orobanchaceae	*Pedicularis*	*Pedicularis longicaulis*	JN046030
Eudicotyledons	Orobanchaceae	*Pedicularis*	*Pedicularis longicaulis*	JN046031
Eudicotyledons	Orobanchaceae	*Pedicularis*	*Pedicularis longicaulis*	JN046032
Eudicotyledons	Orobanchaceae	*Pedicularis*	*Pedicularis lutescens*	JN046040
Eudicotyledons	Orobanchaceae	*Pedicularis*	*Pedicularis lutescens*	JN046041
Eudicotyledons	Orobanchaceae	*Pedicularis*	*Pedicularis lutescens*	JN046042
Eudicotyledons	Orobanchaceae	*Pedicularis*	*Pedicularis lutescens*	JN046043
Eudicotyledons	Orobanchaceae	*Pedicularis*	*Pedicularis lutescens*	JN046044
Eudicotyledons	Orobanchaceae	*Pedicularis*	*Pedicularis mussotii*	JN046061
Eudicotyledons	Orobanchaceae	*Pedicularis*	*Pedicularis mussotii*	JN046062
Eudicotyledons	Orobanchaceae	*Pedicularis*	*Pedicularis mussotii*	JN046063
Eudicotyledons	Orobanchaceae	*Pedicularis*	*Pedicularis mussotii*	JN046064
Eudicotyledons	Orobanchaceae	*Pedicularis*	*Pedicularis mussotii*	JN046065
Eudicotyledons	Orobanchaceae	*Pedicularis*	*Pedicularis rhodotricha*	JN046104
Eudicotyledons	Orobanchaceae	*Pedicularis*	*Pedicularis rhodotricha*	JN046105
Eudicotyledons	Orobanchaceae	*Pedicularis*	*Pedicularis rhodotricha*	JN046106
Eudicotyledons	Orobanchaceae	*Pedicularis*	*Pedicularis rhodotricha*	JN046107
Eudicotyledons	Orobanchaceae	*Pedicularis*	*Pedicularis rhodotricha*	JN046108
Eudicotyledons	Orobanchaceae	*Pedicularis*	*Pedicularis rhynchodonta*	JN046109
Eudicotyledons	Orobanchaceae	*Pedicularis*	*Pedicularis rhynchodonta*	JN046110
Eudicotyledons	Orobanchaceae	*Pedicularis*	*Pedicularis rhynchodonta*	JN046111
Eudicotyledons	Orobanchaceae	*Pedicularis*	*Pedicularis rhynchodonta*	JN046112
Eudicotyledons	Orobanchaceae	*Pedicularis*	*Pedicularis rhynchodonta*	JN046113
Eudicotyledons	Orobanchaceae	*Pedicularis*	*Pedicularis roylei*	JN046120
Eudicotyledons	Orobanchaceae	*Pedicularis*	*Pedicularis roylei*	JN046121
Eudicotyledons	Orobanchaceae	*Pedicularis*	*Pedicularis rupicola*	JN046122
Eudicotyledons	Orobanchaceae	*Pedicularis*	*Pedicularis rupicola*	JN046123
Eudicotyledons	Orobanchaceae	*Pedicularis*	*Pedicularis rupicola*	JN046124
Eudicotyledons	Orobanchaceae	*Pedicularis*	*Pedicularis rupicola*	JN046125
Eudicotyledons	Orobanchaceae	*Pedicularis*	*Pedicularis rupicola*	JN046126
Eudicotyledons	Orobanchaceae	*Pedicularis*	*Pedicularis rupicola*	JN046127
Eudicotyledons	Orobanchaceae	*Pedicularis*	*Pedicularis rupicola*	JN046128
Eudicotyledons	Orobanchaceae	*Pedicularis*	*Pedicularis sigmoidea*	JN046132
Eudicotyledons	Orobanchaceae	*Pedicularis*	*Pedicularis sigmoidea*	JN046133
Eudicotyledons	Orobanchaceae	*Pedicularis*	*Pedicularis superba*	JN046153
Eudicotyledons	Orobanchaceae	*Pedicularis*	*Pedicularis superba*	JN046154
Eudicotyledons	Orobanchaceae	*Pedicularis*	*Pedicularis superba*	JN046155

Eudicotyledons	Orobanchaceae	*Pedicularis*	*Pedicularis superba*	JN046156
Eudicotyledons	Orobanchaceae	*Pedicularis*	*Pedicularis trichoglossa*	JN046178
Eudicotyledons	Orobanchaceae	*Pedicularis*	*Pedicularis trichoglossa*	JN046179
Eudicotyledons	Orobanchaceae	*Pedicularis*	*Pedicularis urceolata*	JN046188
Eudicotyledons	Orobanchaceae	*Pedicularis*	*Pedicularis urceolata*	JN046189
Eudicotyledons	Orobanchaceae	*Pedicularis*	*Pedicularis urceolata*	JN046190
Eudicotyledons	Orobanchaceae	*Pedicularis*	*Pedicularis urceolata*	JN046191
Eudicotyledons	Orobanchaceae	*Pedicularis*	*Pedicularis urceolata*	JN046192
Eudicotyledons	Asteraceae	*Ligularia*	*Ligularia dentata*	AB369645
Eudicotyledons	Asteraceae	*Ligularia*	*Ligularia dentata*	DQ131856
Eudicotyledons	Ericaceae	*Rhododendron*	*Rhododendron obtusum*	HQ707006
Eudicotyledons	Ericaceae	*Rhododendron*	*Rhododendron obtusum*	HQ707007
Eudicotyledons	Adoxaceae	*Viburnum*	*Viburnum betulifolium*	HQ592061
Eudicotyledons	Adoxaceae	*Viburnum*	*Viburnum betulifolium*	JN047484
Eudicotyledons	Adoxaceae	*Viburnum*	*Viburnum betulifolium*	JN047485
Eudicotyledons	Adoxaceae	*Viburnum*	*Viburnum betulifolium*	JN047486
Eudicotyledons	Adoxaceae	*Viburnum*	*Viburnum betulifolium*	JN047487
Eudicotyledons	Adoxaceae	*Viburnum*	*Viburnum betulifolium*	JN047488
Eudicotyledons	Adoxaceae	*Viburnum*	*Viburnum betulifolium*	JN047489
Eudicotyledons	Vitaceae	*Vitis*	*Vitis cinerea*	HQ108287
Eudicotyledons	Vitaceae	*Vitis*	*Vitis cinerea*	HQ108288
Eudicotyledons	Vitaceae	*Vitis*	*Vitis cinerea*	HQ108289
Eudicotyledons	Vitaceae	*Vitis*	*Vitis cinerea*	HQ656413
Eudicotyledons	Vitaceae	*Vitis*	*Vitis cinerea*	HQ656414
Eudicotyledons	Loasaceae	*Mentzelia*	*Mentzelia congesta*	FJ918139
Eudicotyledons	Loasaceae	*Mentzelia*	*Mentzelia congesta*	FJ918140
Eudicotyledons	Loasaceae	*Mentzelia*	*Mentzelia congesta*	FJ918141
Eudicotyledons	Loasaceae	*Mentzelia*	*Mentzelia micrantha*	FJ918166
Eudicotyledons	Loasaceae	*Mentzelia*	*Mentzelia micrantha*	FJ918167
Eudicotyledons	Loasaceae	*Mentzelia*	*Mentzelia nitens*	FJ918183
Eudicotyledons	Loasaceae	*Mentzelia*	*Mentzelia nitens*	FJ918184
Eudicotyledons	Loasaceae	*Mentzelia*	*Mentzelia nitens*	FJ918185
Eudicotyledons	Loasaceae	*Mentzelia*	*Mentzelia nitens*	FJ918186
Eudicotyledons	Loasaceae	*Mentzelia*	*Mentzelia nitens*	FJ918187
Eudicotyledons	Ericaceae	*Rhododendron*	*Rhododendron vaccinioides*	AY196031
Eudicotyledons	Ericaceae	*Rhododendron*	*Rhododendron vaccinioides*	JN047008
Eudicotyledons	Ericaceae	*Rhododendron*	*Rhododendron vaccinioides*	JN047009
Eudicotyledons	Vitaceae	*Vitis*	*Vitis piasezkii*	HQ108314
Eudicotyledons	Vitaceae	*Vitis*	*Vitis piasezkii*	HQ656444
Eudicotyledons	Solanaceae	*Solanum*	*Solanum lyratum*	GQ435280
Eudicotyledons	Solanaceae	*Solanum*	*Solanum lyratum*	JN047213
Eudicotyledons	Solanaceae	*Solanum*	*Solanum lyratum*	JN047214
Eudicotyledons	Solanaceae	*Solanum*	*Solanum lyratum*	JN047215
Eudicotyledons	Solanaceae	*Solanum*	*Solanum lyratum*	JN047216
Eudicotyledons	Solanaceae	*Solanum*	*Solanum lyratum*	JN047217
Eudicotyledons	Solanaceae	*Solanum*	*Solanum lyratum*	JN047218
Eudicotyledons	Solanaceae	*Solanum*	*Solanum lyratum*	JN047219
Eudicotyledons	Caryophyllaceae	*Cerastium*	*Cerastium fontanum*	FJ395474
Eudicotyledons	Caryophyllaceae	*Cerastium*	*Cerastium fontanum*	HQ596636
Eudicotyledons	Vitaceae	*Vitis*	*Vitis pseudoreticulata*	HQ656437
Eudicotyledons	Vitaceae	*Vitis*	*Vitis pseudoreticulata*	HQ656438
Eudicotyledons	Vitaceae	*Vitis*	*Vitis pseudoreticulata*	HQ656467
Eudicotyledons	Lamiaceae	*Scutellaria*	*Scutellaria lateriflora*	HQ596839

Eudicotyledons	Lamiaceae	*Scutellaria*	*Scutellaria lateriflora*	HQ596840
Eudicotyledons	Plantaginaceae	*Antirrhinum*	*Antirrhinum australe*	HM152881
Eudicotyledons	Plantaginaceae	*Antirrhinum*	*Antirrhinum australe*	HM152882
Eudicotyledons	Plantaginaceae	*Antirrhinum*	*Antirrhinum australe*	HM152883
Eudicotyledons	Plantaginaceae	*Antirrhinum*	*Antirrhinum charidemi*	HM152886
Eudicotyledons	Plantaginaceae	*Antirrhinum*	*Antirrhinum charidemi*	HM152887
Eudicotyledons	Plantaginaceae	*Antirrhinum*	*Antirrhinum charidemi*	HM152888
Eudicotyledons	Plantaginaceae	*Antirrhinum*	*Antirrhinum charidemi*	HM152889
Eudicotyledons	Plantaginaceae	*Antirrhinum*	*Antirrhinum latifolium*	HM152907
Eudicotyledons	Plantaginaceae	*Antirrhinum*	*Antirrhinum latifolium*	HM152911
Eudicotyledons	Plantaginaceae	*Antirrhinum*	*Antirrhinum lopesianum*	HM152917
Eudicotyledons	Plantaginaceae	*Antirrhinum*	*Antirrhinum lopesianum*	HM152918
Eudicotyledons	Plantaginaceae	*Antirrhinum*	*Antirrhinum lopesianum*	HM152919
Eudicotyledons	Plantaginaceae	*Antirrhinum*	*Antirrhinum microphyllum*	HM152928
Eudicotyledons	Plantaginaceae	*Antirrhinum*	*Antirrhinum microphyllum*	HM152929
Eudicotyledons	Plantaginaceae	*Antirrhinum*	*Antirrhinum microphyllum*	HM152930
Eudicotyledons	Plantaginaceae	*Antirrhinum*	*Antirrhinum sempervirens*	HM152943
Eudicotyledons	Plantaginaceae	*Antirrhinum*	*Antirrhinum sempervirens*	HM152944
Eudicotyledons	Plantaginaceae	*Antirrhinum*	*Antirrhinum sempervirens*	HM152945
Eudicotyledons	Adoxaceae	*Viburnum*	*Viburnum cylindricum*	AY627389
Eudicotyledons	Adoxaceae	*Viburnum*	*Viburnum cylindricum*	JN047490
Eudicotyledons	Adoxaceae	*Viburnum*	*Viburnum cylindricum*	JN047491
Eudicotyledons	Adoxaceae	*Viburnum*	*Viburnum cylindricum*	JN047492
Eudicotyledons	Adoxaceae	*Viburnum*	*Viburnum cylindricum*	JN047493
Eudicotyledons	Adoxaceae	*Viburnum*	*Viburnum cylindricum*	JN047494
Eudicotyledons	Adoxaceae	*Viburnum*	*Viburnum cylindricum*	JN047495
Eudicotyledons	Adoxaceae	*Viburnum*	*Viburnum dilatatum*	AY627392
Eudicotyledons	Adoxaceae	*Viburnum*	*Viburnum dilatatum*	JN047496
Eudicotyledons	Adoxaceae	*Viburnum*	*Viburnum dilatatum*	JN047497
Eudicotyledons	Adoxaceae	*Viburnum*	*Viburnum dilatatum*	JN047498
Eudicotyledons	Adoxaceae	*Viburnum*	*Viburnum dilatatum*	JN047499
Eudicotyledons	Adoxaceae	*Viburnum*	*Viburnum dilatatum*	JN047500
Eudicotyledons	Adoxaceae	*Viburnum*	*Viburnum dilatatum*	JN047501
Eudicotyledons	Adoxaceae	*Viburnum*	*Viburnum erosum*	HQ427059
Eudicotyledons	Adoxaceae	*Viburnum*	*Viburnum erosum*	AY627396
Eudicotyledons	Adoxaceae	*Viburnum*	*Viburnum erosum*	JN047502
Eudicotyledons	Adoxaceae	*Viburnum*	*Viburnum erosum*	JN047503
Eudicotyledons	Adoxaceae	*Viburnum*	*Viburnum erosum*	JN047504
Eudicotyledons	Adoxaceae	*Viburnum*	*Viburnum erosum*	JN047505
Eudicotyledons	Adoxaceae	*Viburnum*	*Viburnum erosum*	JN047506
Eudicotyledons	Adoxaceae	*Viburnum*	*Viburnum odoratissimum*	HQ415464
Eudicotyledons	Adoxaceae	*Viburnum*	*Viburnum odoratissimum*	AY627411
Eudicotyledons	Adoxaceae	*Viburnum*	*Viburnum prunifolium*	AY627413
Eudicotyledons	Adoxaceae	*Viburnum*	*Viburnum prunifolium*	DQ006167
Eudicotyledons	Adoxaceae	*Viburnum*	*Viburnum stenocalyx*	HQ592097
Eudicotyledons	Adoxaceae	*Viburnum*	*Viburnum stenocalyx*	AY627418
Eudicotyledons	Adoxaceae	*Viburnum*	*Viburnum triphyllum*	HQ592105
Eudicotyledons	Adoxaceae	*Viburnum*	*Viburnum triphyllum*	AY627422
Eudicotyledons	Adoxaceae	*Viburnum*	*Viburnum utile*	AY627424
Eudicotyledons	Adoxaceae	*Viburnum*	*Viburnum utile*	JN047526
Eudicotyledons	Adoxaceae	*Viburnum*	*Viburnum utile*	JN047527
Eudicotyledons	Asteraceae	*Saussurea*	*Saussurea katochaete*	EF420899
Eudicotyledons	Asteraceae	*Saussurea*	*Saussurea katochaete*	EF420900

Eudicotyledons	Amaranthaceae	*Chenopodium*	*Chenopodium foliosum*	JN044293
Eudicotyledons	Amaranthaceae	*Chenopodium*	*Chenopodium foliosum*	JN044294
Eudicotyledons	Solanaceae	*Capsicum*	*Capsicum flexuosum*	EF537229
Eudicotyledons	Solanaceae	*Capsicum*	*Capsicum flexuosum*	EF537230
Eudicotyledons	Solanaceae	*Capsicum*	*Capsicum flexuosum*	EF537231
Eudicotyledons	Solanaceae	*Capsicum*	*Capsicum flexuosum*	EF537232
Eudicotyledons	Saxifragaceae	*Mitella*	*Mitella doiana*	AB492557
Eudicotyledons	Saxifragaceae	*Mitella*	*Mitella doiana*	AB492558
Eudicotyledons	Saxifragaceae	*Mitella*	*Mitella doiana*	AB492559
Eudicotyledons	Saxifragaceae	*Mitella*	*Mitella kiusiana*	AB492580
Eudicotyledons	Saxifragaceae	*Mitella*	*Mitella kiusiana*	AB492581
Eudicotyledons	Saxifragaceae	*Mitella*	*Mitella kiusiana*	AB492582
Eudicotyledons	Saxifragaceae	*Mitella*	*Mitella kiusiana*	AB492583
Eudicotyledons	Saxifragaceae	*Mitella*	*Mitella kiusiana*	AB492584
Eudicotyledons	Saxifragaceae	*Mitella*	*Mitella kiusiana*	AB492585
Eudicotyledons	Saxifragaceae	*Mitella*	*Mitella kiusiana*	AB492586
Eudicotyledons	Saxifragaceae	*Mitella*	*Mitella kiusiana*	AB492587
Eudicotyledons	Saxifragaceae	*Mitella*	*Mitella kiusiana*	AB492588
Eudicotyledons	Saxifragaceae	*Mitella*	*Mitella kiusiana*	AB492589
Eudicotyledons	Saxifragaceae	*Mitella*	*Mitella kiusiana*	AB492590
Eudicotyledons	Saxifragaceae	*Mitella*	*Mitella koshiensis*	AB492612
Eudicotyledons	Saxifragaceae	*Mitella*	*Mitella koshiensis*	AB492613
Eudicotyledons	Saxifragaceae	*Mitella*	*Mitella koshiensis*	AB492614
Eudicotyledons	Saxifragaceae	*Mitella*	*Mitella koshiensis*	AB492615
Eudicotyledons	Saxifragaceae	*Mitella*	*Mitella koshiensis*	AB492616
Eudicotyledons	Saxifragaceae	*Mitella*	*Mitella formosana*	AB492549
Eudicotyledons	Saxifragaceae	*Mitella*	*Mitella formosana*	AB492550
Eudicotyledons	Saxifragaceae	*Mitella*	*Mitella formosana*	AB492551
Eudicotyledons	Saxifragaceae	*Mitella*	*Mitella formosana*	AB492552
Eudicotyledons	Saxifragaceae	*Mitella*	*Mitella formosana*	AB492553
Eudicotyledons	Saxifragaceae	*Mitella*	*Mitella formosana*	AB492554
Eudicotyledons	Saxifragaceae	*Mitella*	*Mitella formosana*	AB492555
Eudicotyledons	Saxifragaceae	*Mitella*	*Mitella formosana*	AB492556
Eudicotyledons	Cucurbitaceae	*Bryonia*	*Bryonia alba*	EU096317
Eudicotyledons	Cucurbitaceae	*Bryonia*	*Bryonia alba*	EU096318
Eudicotyledons	Cucurbitaceae	*Bryonia*	*Bryonia alba*	EU096319
Eudicotyledons	Cucurbitaceae	*Bryonia*	*Bryonia alba*	EU096320
Eudicotyledons	Cucurbitaceae	*Bryonia*	*Bryonia alba*	EU096321
Eudicotyledons	Cucurbitaceae	*Bryonia*	*Bryonia alba*	EU096322
Eudicotyledons	Cucurbitaceae	*Bryonia*	*Bryonia alba*	EU096323
Eudicotyledons	Cucurbitaceae	*Bryonia*	*Bryonia alba*	EU096324
Eudicotyledons	Cucurbitaceae	*Bryonia*	*Bryonia alba*	EU096325
Eudicotyledons	Cucurbitaceae	*Bryonia*	*Bryonia alba*	EU096326
Eudicotyledons	Cucurbitaceae	*Bryonia*	*Bryonia alba*	EU096327
Eudicotyledons	Cucurbitaceae	*Bryonia*	*Bryonia alba*	EU096328
Eudicotyledons	Cucurbitaceae	*Bryonia*	*Bryonia alba*	EU096329
Eudicotyledons	Cucurbitaceae	*Bryonia*	*Bryonia alba*	EU096330
Eudicotyledons	Cucurbitaceae	*Bryonia*	*Bryonia alba*	EU096331
Eudicotyledons	Cucurbitaceae	*Bryonia*	*Bryonia alba*	EU096332
Eudicotyledons	Cucurbitaceae	*Bryonia*	*Bryonia alba*	EU096333
Eudicotyledons	Cucurbitaceae	*Bryonia*	*Bryonia alba*	EU096334
Eudicotyledons	Cucurbitaceae	*Bryonia*	*Bryonia alba*	EU096335
Eudicotyledons	Cucurbitaceae	*Bryonia*	*Bryonia alba*	EU096336

Eudicotyledons	Cucurbitaceae	*Bryonia*	*Bryonia alba*	EU096337
Eudicotyledons	Cucurbitaceae	*Bryonia*	*Bryonia alba*	EU096338
Eudicotyledons	Cucurbitaceae	*Bryonia*	*Bryonia alba*	EU096339
Eudicotyledons	Amaranthaceae	*Chenopodium*	*Chenopodium glaucum*	JN044295
Eudicotyledons	Amaranthaceae	*Chenopodium*	*Chenopodium glaucum*	JN044296
Eudicotyledons	Amaranthaceae	*Chenopodium*	*Chenopodium glaucum*	JN044297
Eudicotyledons	Amaranthaceae	*Chenopodium*	*Chenopodium glaucum*	JN044298
Eudicotyledons	Oxalidaceae	*Oxalis*	*Oxalis namaquana*	JN983505
Eudicotyledons	Oxalidaceae	*Oxalis*	*Oxalis namaquana*	JN983506
Eudicotyledons	Oxalidaceae	*Oxalis*	*Oxalis namaquana*	JN983507
Eudicotyledons	Oxalidaceae	*Oxalis*	*Oxalis namaquana*	JN983508
Eudicotyledons	Burseraceae	*Bursera*	*Bursera brunea*	AY309404
Eudicotyledons	Burseraceae	*Bursera*	*Bursera brunea*	AY309405
Eudicotyledons	Burseraceae	*Bursera*	*Bursera brunea*	AY309406
Eudicotyledons	Burseraceae	*Bursera*	*Bursera gracilipes*	AY309407
Eudicotyledons	Burseraceae	*Bursera*	*Bursera gracilipes*	AY309408
Eudicotyledons	Burseraceae	*Canarium*	*Canarium pilosum*	AY635378
Eudicotyledons	Burseraceae	*Canarium*	*Canarium pilosum*	AY831938
Eudicotyledons	Rubiaceae	*Oldenlandia*	*Oldenlandia tenelliflora*	JF699810
Eudicotyledons	Rubiaceae	*Oldenlandia*	*Oldenlandia tenelliflora*	JF699811
Eudicotyledons	Rubiaceae	*Oldenlandia*	*Oldenlandia tenelliflora*	JN044785
Eudicotyledons	Rubiaceae	*Oldenlandia*	*Oldenlandia tenelliflora*	JN044786
Eudicotyledons	Rubiaceae	*Oldenlandia*	*Oldenlandia diffusa*	GQ435231
Eudicotyledons	Rubiaceae	*Oldenlandia*	*Oldenlandia diffusa*	JF699788
Eudicotyledons	Rubiaceae	*Oldenlandia*	*Oldenlandia diffusa*	JF699789
Eudicotyledons	Asteraceae	*Ligularia*	*Ligularia tongolensis*	JN045209
Eudicotyledons	Asteraceae	*Ligularia*	*Ligularia tongolensis*	JN045210
Eudicotyledons	Asteraceae	*Ligularia*	*Ligularia tongolensis*	JN045211
Eudicotyledons	Asteraceae	*Ligularia*	*Ligularia tongolensis*	JN045212
Eudicotyledons	Asteraceae	*Arctotis*	*Arctotis bellidifolia*	EU846421
Eudicotyledons	Asteraceae	*Arctotis*	*Arctotis bellidifolia*	EU846422
Eudicotyledons	Rosaceae	*Prunus*	*Prunus pensylvanica*	HQ596802
Eudicotyledons	Rosaceae	*Prunus*	*Prunus pensylvanica*	AY500632
Eudicotyledons	Asteraceae	*Ligularia*	*Ligularia nelumbifolia*	JN045199
Eudicotyledons	Asteraceae	*Ligularia*	*Ligularia nelumbifolia*	JN045200
Eudicotyledons	Asteraceae	*Ligularia*	*Ligularia pleurocaulis*	JN045201
Eudicotyledons	Asteraceae	*Ligularia*	*Ligularia pleurocaulis*	JN045202
Eudicotyledons	Asteraceae	*Ligularia*	*Ligularia subspicata*	JN045203
Eudicotyledons	Asteraceae	*Ligularia*	*Ligularia subspicata*	JN045204
Eudicotyledons	Asteraceae	*Ligularia*	*Ligularia subspicata*	JN045205
Eudicotyledons	Asteraceae	*Ligularia*	*Ligularia subspicata*	JN045206
Eudicotyledons	Asteraceae	*Ligularia*	*Ligularia subspicata*	JN045207
Eudicotyledons	Asteraceae	*Ligularia*	*Ligularia subspicata*	JN045208
Eudicotyledons	Asteraceae	*Ligularia*	*Ligularia vellerea*	JN045213
Eudicotyledons	Asteraceae	*Ligularia*	*Ligularia vellerea*	JN045214
Eudicotyledons	Asteraceae	*Ligularia*	*Ligularia vellerea*	JN045215
Eudicotyledons	Ericaceae	*Rhododendron*	*Rhododendron hybrid cultivar*	HQ707035
Eudicotyledons	Ericaceae	*Rhododendron*	*Rhododendron hybrid cultivar*	HQ707036
Eudicotyledons	Brassicaceae	*Capsella*	*Capsella grandiflora*	FR822352
Eudicotyledons	Brassicaceae	*Capsella*	*Capsella grandiflora*	FR822353
Eudicotyledons	Rubiaceae	*Morinda*	*Morinda officinalis*	JN045486
Eudicotyledons	Rubiaceae	*Morinda*	*Morinda officinalis*	JN045487
Eudicotyledons	Rubiaceae	*Morinda*	*Morinda officinalis*	JN045488

Eudicotyledons	Rosaceae	*Rosa*	*Rosa palustris*	DQ778798
Eudicotyledons	Rosaceae	*Rosa*	*Rosa palustris*	HQ596823
Eudicotyledons	Rosaceae	*Rosa*	*Rosa xanthina*	DQ778816
Eudicotyledons	Rosaceae	*Rosa*	*Rosa xanthina*	DQ778817
Eudicotyledons	Solanaceae	*Solanum*	*Solanum aculeatissimum*	JN047208
Eudicotyledons	Solanaceae	*Solanum*	*Solanum aculeatissimum*	JN047209
Eudicotyledons	Solanaceae	*Solanum*	*Solanum aculeatissimum*	JN047210
Eudicotyledons	Solanaceae	*Solanum*	*Solanum aculeatissimum*	JN047211
Eudicotyledons	Solanaceae	*Solanum*	*Solanum aculeatissimum*	JN047212
Eudicotyledons	Convolvulaceae	*Convolvulus*	*Convolvulus floridus*	EF375896
Eudicotyledons	Convolvulaceae	*Convolvulus*	*Convolvulus floridus*	EF375897
Eudicotyledons	Convolvulaceae	*Convolvulus*	*Convolvulus floridus*	EF375898
Eudicotyledons	Convolvulaceae	*Convolvulus*	*Convolvulus floridus*	EF375899
Eudicotyledons	Convolvulaceae	*Convolvulus*	*Convolvulus floridus*	EF375900
Eudicotyledons	Convolvulaceae	*Convolvulus*	*Convolvulus floridus*	EF375901
Eudicotyledons	Convolvulaceae	*Convolvulus*	*Convolvulus floridus*	EF375902
Eudicotyledons	Convolvulaceae	*Convolvulus*	*Convolvulus floridus*	EF375903
Eudicotyledons	Convolvulaceae	*Convolvulus*	*Convolvulus floridus*	EF375904
Eudicotyledons	Convolvulaceae	*Convolvulus*	*Convolvulus floridus*	EF375905
Eudicotyledons	Convolvulaceae	*Convolvulus*	*Convolvulus floridus*	EF375906
Eudicotyledons	Convolvulaceae	*Convolvulus*	*Convolvulus floridus*	EF375907
Eudicotyledons	Convolvulaceae	*Convolvulus*	*Convolvulus floridus*	EF375908
Eudicotyledons	Convolvulaceae	*Convolvulus*	*Convolvulus floridus*	EF375909
Eudicotyledons	Convolvulaceae	*Convolvulus*	*Convolvulus floridus*	EF375910
Eudicotyledons	Convolvulaceae	*Convolvulus*	*Convolvulus floridus*	EF375911
Eudicotyledons	Convolvulaceae	*Convolvulus*	*Convolvulus floridus*	EF375912
Eudicotyledons	Convolvulaceae	*Convolvulus*	*Convolvulus floridus*	EF375913
Eudicotyledons	Convolvulaceae	*Convolvulus*	*Convolvulus floridus*	EF375914
Eudicotyledons	Convolvulaceae	*Convolvulus*	*Convolvulus scoparius*	EF375915
Eudicotyledons	Convolvulaceae	*Convolvulus*	*Convolvulus scoparius*	EF375916
Eudicotyledons	Convolvulaceae	*Convolvulus*	*Convolvulus scoparius*	EF375917
Eudicotyledons	Convolvulaceae	*Convolvulus*	*Convolvulus scoparius*	EF375918
Eudicotyledons	Convolvulaceae	*Convolvulus*	*Convolvulus scoparius*	EF375919
Eudicotyledons	Convolvulaceae	*Convolvulus*	*Convolvulus scoparius*	EF375920
Eudicotyledons	Convolvulaceae	*Convolvulus*	*Convolvulus scoparius*	EF375921
Eudicotyledons	Convolvulaceae	*Convolvulus*	*Convolvulus scoparius*	EF375922
Eudicotyledons	Convolvulaceae	*Convolvulus*	*Convolvulus scoparius*	EF375923
Eudicotyledons	Convolvulaceae	*Convolvulus*	*Convolvulus scoparius*	EF375924
Eudicotyledons	Convolvulaceae	*Convolvulus*	*Convolvulus scoparius*	EF375925
Eudicotyledons	Convolvulaceae	*Convolvulus*	*Convolvulus scoparius*	EF375926
Eudicotyledons	Convolvulaceae	*Convolvulus*	*Convolvulus scoparius*	EF375927
Eudicotyledons	Convolvulaceae	*Convolvulus*	*Convolvulus scoparius*	EF375928
Eudicotyledons	Convolvulaceae	*Convolvulus*	*Convolvulus scoparius*	EF375929
Eudicotyledons	Asteraceae	*Ligularia*	*Ligularia lankongensis*	JN045197
Eudicotyledons	Asteraceae	*Ligularia*	*Ligularia lankongensis*	JN045198
Eudicotyledons	Rubiaceae	*Morinda*	*Morinda umbellata*	JN045492
Eudicotyledons	Rubiaceae	*Morinda*	*Morinda umbellata*	JN045493
Eudicotyledons	Rubiaceae	*Morinda*	*Morinda umbellata*	JN045494
Eudicotyledons	Rubiaceae	*Morinda*	*Morinda umbellata*	JN045495
Eudicotyledons	Rubiaceae	*Morinda*	*Morinda umbellata*	JN045496
Eudicotyledons	Lamiaceae	*Origanum*	*Origanum majorana*	FR726122
Eudicotyledons	Lamiaceae	*Origanum*	*Origanum majorana*	FR726123
Eudicotyledons	Lamiaceae	*Origanum*	*Origanum majorana*	FR726124

Eudicotyledons	Lamiaceae	*Origanum*	*Origanum majorana*	FR726125
Eudicotyledons	Lamiaceae	*Origanum*	*Origanum majorana*	HQ902835
Eudicotyledons	Sapotaceae	*Manilkara*	*Manilkara huberi*	FJ039060
Eudicotyledons	Sapotaceae	*Manilkara*	*Manilkara huberi*	FJ039061
Eudicotyledons	Sapotaceae	*Manilkara*	*Manilkara huberi*	AM179733
Eudicotyledons	Rubiaceae	*Mussaenda*	*Mussaenda pubescens*	JN045520
Eudicotyledons	Rubiaceae	*Mussaenda*	*Mussaenda pubescens*	JN045521
Eudicotyledons	Rubiaceae	*Mussaenda*	*Mussaenda pubescens*	JN045522
Eudicotyledons	Rubiaceae	*Mussaenda*	*Mussaenda pubescens*	JN045523
Eudicotyledons	Rubiaceae	*Mussaenda*	*Mussaenda pubescens*	JN045524
Eudicotyledons	Rubiaceae	*Mussaenda*	*Mussaenda pubescens*	JN045525
Eudicotyledons	Rubiaceae	*Mussaenda*	*Mussaenda pubescens*	JN045526
Eudicotyledons	Rubiaceae	*Mussaenda*	*Mussaenda pubescens*	JN045527
Eudicotyledons	Caryophyllaceae	*Cerastium*	*Cerastium eriophorum*	AY521416
Eudicotyledons	Caryophyllaceae	*Cerastium*	*Cerastium eriophorum*	AY521438
Eudicotyledons	Cucurbitaceae	*Trichosanthes*	*Trichosanthes lepiniana*	HQ829555
Eudicotyledons	Cucurbitaceae	*Trichosanthes*	*Trichosanthes lepiniana*	HQ829556
Eudicotyledons	Rosaceae	*Malus*	*Malus pumila*	JQ390737
Eudicotyledons	Rosaceae	*Malus*	*Malus pumila*	GU562389
Eudicotyledons	Rosaceae	*Malus*	*Malus pumila*	HQ596764
Eudicotyledons	Salicaceae	*Salix*	*Salix eriocephala*	EU750540
Eudicotyledons	Salicaceae	*Salix*	*Salix eriocephala*	EU750541
Eudicotyledons	Salicaceae	*Salix*	*Salix eriocephala*	EU750542
Eudicotyledons	Salicaceae	*Salix*	*Salix eriocephala*	EU750543
Eudicotyledons	Caryophyllaceae	*Dianthus*	*Dianthus superbus*	GU441090
Eudicotyledons	Caryophyllaceae	*Dianthus*	*Dianthus superbus*	GU441091
Eudicotyledons	Caryophyllaceae	*Dianthus*	*Dianthus superbus*	GQ435348
Eudicotyledons	Caryophyllaceae	*Dianthus*	*Dianthus sylvestris*	GU441092
Eudicotyledons	Caryophyllaceae	*Dianthus*	*Dianthus sylvestris*	GU441093
Eudicotyledons	Caryophyllaceae	*Dianthus*	*Dianthus sylvestris*	GU441094
Eudicotyledons	Aceraceae	*Acer*	*Acer grandidentatum*	DQ887749
Eudicotyledons	Aceraceae	*Acer*	*Acer grandidentatum*	DQ978627
Eudicotyledons	Plantaginaceae	*Antirrhinum*	*Antirrhinum pulverulentum*	HM152937
Eudicotyledons	Plantaginaceae	*Antirrhinum*	*Antirrhinum pulverulentum*	HM152938
Eudicotyledons	Plantaginaceae	*Antirrhinum*	*Antirrhinum pulverulentum*	HM152939
Eudicotyledons	Plantaginaceae	*Antirrhinum*	*Antirrhinum pulverulentum*	HM152940
Eudicotyledons	Plantaginaceae	*Antirrhinum*	*Antirrhinum pulverulentum*	HM152941
Eudicotyledons	Plantaginaceae	*Antirrhinum*	*Antirrhinum pulverulentum*	HM152942
Eudicotyledons	Plantaginaceae	*Antirrhinum*	*Antirrhinum grosii*	HM152902
Eudicotyledons	Plantaginaceae	*Antirrhinum*	*Antirrhinum grosii*	HM152903
Eudicotyledons	Plantaginaceae	*Antirrhinum*	*Antirrhinum subbaeticum*	HM152950
Eudicotyledons	Plantaginaceae	*Antirrhinum*	*Antirrhinum subbaeticum*	HM152951
Eudicotyledons	Plantaginaceae	*Antirrhinum*	*Antirrhinum subbaeticum*	HM152952
Eudicotyledons	Plantaginaceae	*Antirrhinum*	*Antirrhinum subbaeticum*	HM152953
Eudicotyledons	Burseraceae	*Canarium*	*Canarium album*	HQ415434
Eudicotyledons	Burseraceae	*Canarium*	*Canarium album*	AY635371
Eudicotyledons	Burseraceae	*Bursera*	*Bursera graveolens*	GQ505924
Eudicotyledons	Burseraceae	*Bursera*	*Bursera graveolens*	GQ505927
Eudicotyledons	Burseraceae	*Bursera*	*Bursera graveolens*	GQ505928
Eudicotyledons	Burseraceae	*Bursera*	*Bursera graveolens*	GQ505929
Eudicotyledons	Burseraceae	*Bursera*	*Bursera graveolens*	GQ505930
Eudicotyledons	Burseraceae	*Bursera*	*Bursera graveolens*	JF919186
Eudicotyledons	Burseraceae	*Bursera*	*Bursera malacophylla*	GQ505925

Eudicotyledons	Burseraceae	*Bursera*	*Bursera malacophylla*	GQ505926
Eudicotyledons	Plantaginaceae	*Veronica*	*Veronica krumovii*	HM370885
Eudicotyledons	Plantaginaceae	*Veronica*	*Veronica krumovii*	HM370886
Eudicotyledons	Plantaginaceae	*Veronica*	*Veronica krumovii*	HM370905
Eudicotyledons	Plantaginaceae	*Veronica*	*Veronica krumovii*	HM370906
Eudicotyledons	Plantaginaceae	*Veronica*	*Veronica krumovii*	HM370939
Eudicotyledons	Plantaginaceae	*Veronica*	*Veronica krumovii*	HM370946
Eudicotyledons	Fabaceae	*Acacia*	*Acacia brevispica*	GQ872304
Eudicotyledons	Fabaceae	*Acacia*	*Acacia brevispica*	EU811977
Eudicotyledons	Platanaceae	*Platanus*	*Platanus rzedowskii*	HE661211
Eudicotyledons	Platanaceae	*Platanus*	*Platanus rzedowskii*	HE661212
Eudicotyledons	Platanaceae	*Platanus*	*Platanus rzedowskii*	HE661213
Eudicotyledons	Platanaceae	*Platanus*	*Platanus rzedowskii*	HE661214
Eudicotyledons	Platanaceae	*Platanus*	*Platanus rzedowskii*	HE661215
Eudicotyledons	Platanaceae	*Platanus*	*Platanus rzedowskii*	HE661216
Eudicotyledons	Platanaceae	*Platanus*	*Platanus rzedowskii*	HE661217
Eudicotyledons	Platanaceae	*Platanus*	*Platanus wrightii*	HE661226
Eudicotyledons	Platanaceae	*Platanus*	*Platanus wrightii*	HE661227
Eudicotyledons	Platanaceae	*Platanus*	*Platanus wrightii*	HE661228
Eudicotyledons	Platanaceae	*Platanus*	*Platanus wrightii*	HE661229
Eudicotyledons	Asteraceae	*Ligularia*	*Ligularia cymbulifera*	JN045182
Eudicotyledons	Asteraceae	*Ligularia*	*Ligularia cymbulifera*	JN045183
Eudicotyledons	Asteraceae	*Ligularia*	*Ligularia cymbulifera*	JN045184
Eudicotyledons	Asteraceae	*Ligularia*	*Ligularia cymbulifera*	JN045185
Eudicotyledons	Asteraceae	*Ligularia*	*Ligularia cymbulifera*	JN045186
Eudicotyledons	Asteraceae	*Ligularia*	*Ligularia cymbulifera*	JN045187
Eudicotyledons	Asteraceae	*Ligularia*	*Ligularia cymbulifera*	JN045188
Eudicotyledons	Ericaceae	*Rhododendron*	*Rhododendron impeditum*	JN046842
Eudicotyledons	Ericaceae	*Rhododendron*	*Rhododendron impeditum*	JN046843
Eudicotyledons	Ericaceae	*Rhododendron*	*Rhododendron impeditum*	JN046844
Eudicotyledons	Ericaceae	*Rhododendron*	*Rhododendron impeditum*	JN046845
Eudicotyledons	Campanulaceae	*Lobelia*	*Lobelia inflata*	HQ596751
Eudicotyledons	Campanulaceae	*Lobelia*	*Lobelia inflata*	GQ248332
Eudicotyledons	Campanulaceae	*Lobelia*	*Lobelia inflata*	EF590710
Eudicotyledons	Solanaceae	*Hyoscyamus*	*Hyoscyamus albus*	HQ216158
Eudicotyledons	Solanaceae	*Hyoscyamus*	*Hyoscyamus albus*	HQ216159
Eudicotyledons	Ericaceae	*Rhododendron*	*Rhododendron excellens*	HQ706964
Eudicotyledons	Ericaceae	*Rhododendron*	*Rhododendron excellens*	HQ706965
Eudicotyledons	Ericaceae	*Rhododendron*	*Rhododendron excellens*	HQ706966
Eudicotyledons	Ericaceae	*Rhododendron*	*Rhododendron forrestii*	JN046818
Eudicotyledons	Ericaceae	*Rhododendron*	*Rhododendron forrestii*	JN046819
Eudicotyledons	Ericaceae	*Rhododendron*	*Rhododendron genestierianum*	JN046826
Eudicotyledons	Ericaceae	*Rhododendron*	*Rhododendron genestierianum*	JN046827
Eudicotyledons	Ericaceae	*Rhododendron*	*Rhododendron mekongense*	JN046880
Eudicotyledons	Ericaceae	*Rhododendron*	*Rhododendron mekongense*	JN046881
Eudicotyledons	Ericaceae	*Rhododendron*	*Rhododendron mekongense*	HQ706996
Eudicotyledons	Ericaceae	*Rhododendron*	*Rhododendron mekongense*	HQ706997
Eudicotyledons	Ericaceae	*Rhododendron*	*Rhododendron triflorum*	JN047002
Eudicotyledons	Ericaceae	*Rhododendron*	*Rhododendron triflorum*	JN047003
Eudicotyledons	Ericaceae	*Rhododendron*	*Rhododendron wardii*	JN047016
Eudicotyledons	Ericaceae	*Rhododendron*	*Rhododendron wardii*	JN047017
Eudicotyledons	Ericaceae	*Rhododendron*	*Rhododendron wardii*	JN047018
Eudicotyledons	Ericaceae	*Rhododendron*	*Rhododendron wardii*	JN047019

Eudicotyledons	Ericaceae	*Rhododendron*	*Rhododendron wardii*	JN047020
Eudicotyledons	Ericaceae	*Rhododendron*	*Rhododendron wardii*	HQ707037
Eudicotyledons	Ericaceae	*Rhododendron*	*Rhododendron wardii*	HQ707038
Eudicotyledons	Ericaceae	*Rhododendron*	*Rhododendron xanthostephanum*	JN047021
Eudicotyledons	Ericaceae	*Rhododendron*	*Rhododendron xanthostephanum*	JN047022
Eudicotyledons	Ericaceae	*Rhododendron*	*Rhododendron xanthostephanum*	JN047023
Eudicotyledons	Ericaceae	*Rhododendron*	*Rhododendron xanthostephanum*	JN047024
Eudicotyledons	Ericaceae	*Rhododendron*	*Rhododendron maddenii*	JN046875
Eudicotyledons	Ericaceae	*Rhododendron*	*Rhododendron maddenii*	JN046876
Eudicotyledons	Ericaceae	*Rhododendron*	*Rhododendron maddenii*	JN046877
Eudicotyledons	Ericaceae	*Rhododendron*	*Rhododendron auriculatum*	HQ706955
Eudicotyledons	Ericaceae	*Rhododendron*	*Rhododendron auriculatum*	HQ706956
Eudicotyledons	Ericaceae	*Rhododendron*	*Rhododendron delavayi*	JN046793
Eudicotyledons	Ericaceae	*Rhododendron*	*Rhododendron delavayi*	JN046794
Eudicotyledons	Ericaceae	*Rhododendron*	*Rhododendron delavayi*	JN046795
Eudicotyledons	Ericaceae	*Rhododendron*	*Rhododendron delavayi*	JN046796
Eudicotyledons	Ericaceae	*Rhododendron*	*Rhododendron delavayi*	JN046797
Eudicotyledons	Ericaceae	*Rhododendron*	*Rhododendron delavayi*	HM636525
Eudicotyledons	Ericaceae	*Rhododendron*	*Rhododendron delavayi*	HQ706960
Eudicotyledons	Ericaceae	*Rhododendron*	*Rhododendron delavayi*	HQ706961
Eudicotyledons	Ericaceae	*Rhododendron*	*Rhododendron jingangshanicum*	HQ706989
Eudicotyledons	Ericaceae	*Rhododendron*	*Rhododendron jingangshanicum*	HQ706990
Eudicotyledons	Ericaceae	*Rhododendron*	*Rhododendron irroratum*	JN046846
Eudicotyledons	Ericaceae	*Rhododendron*	*Rhododendron irroratum*	JN046847
Eudicotyledons	Ericaceae	*Rhododendron*	*Rhododendron irroratum*	JN046848
Eudicotyledons	Ericaceae	*Rhododendron*	*Rhododendron irroratum*	JN046849
Eudicotyledons	Ericaceae	*Rhododendron*	*Rhododendron irroratum*	HQ706986
Eudicotyledons	Ericaceae	*Rhododendron*	*Rhododendron irroratum*	HQ706987
Eudicotyledons	Ericaceae	*Rhododendron*	*Rhododendron irroratum*	HQ706988
Eudicotyledons	Ericaceae	*Rhododendron*	*Rhododendron annae*	HQ706951
Eudicotyledons	Ericaceae	*Rhododendron*	*Rhododendron annae*	HQ706952
Eudicotyledons	Ericaceae	*Rhododendron*	*Rhododendron annae*	HQ706953
Eudicotyledons	Orobanchaceae	*Pedicularis*	*Pedicularis kansuensis*	JN046008
Eudicotyledons	Orobanchaceae	*Pedicularis*	*Pedicularis kansuensis*	JN046009
Eudicotyledons	Orobanchaceae	*Pedicularis*	*Pedicularis kansuensis*	JN046010
Eudicotyledons	Orobanchaceae	*Pedicularis*	*Pedicularis kansuensis*	JN046011
Eudicotyledons	Orobanchaceae	*Pedicularis*	*Pedicularis kansuensis*	JN046012
Eudicotyledons	Orobanchaceae	*Pedicularis*	*Pedicularis rex*	JN046094
Eudicotyledons	Orobanchaceae	*Pedicularis*	*Pedicularis rex*	JN046095
Eudicotyledons	Orobanchaceae	*Pedicularis*	*Pedicularis rex*	JN046096
Eudicotyledons	Orobanchaceae	*Pedicularis*	*Pedicularis rex*	JN046097
Eudicotyledons	Orobanchaceae	*Pedicularis*	*Pedicularis rex*	JN046098
Eudicotyledons	Orobanchaceae	*Pedicularis*	*Pedicularis salviiflora*	JN046129
Eudicotyledons	Orobanchaceae	*Pedicularis*	*Pedicularis salviiflora*	JN046130
Eudicotyledons	Orobanchaceae	*Pedicularis*	*Pedicularis salviiflora*	JN046131
Eudicotyledons	Solanaceae	*Petunia*	*Petunia altiplana*	DQ791909
Eudicotyledons	Solanaceae	*Petunia*	*Petunia altiplana*	DQ791910
Eudicotyledons	Solanaceae	*Petunia*	*Petunia altiplana*	DQ791911
Eudicotyledons	Solanaceae	*Petunia*	*Petunia altiplana*	DQ791912
Eudicotyledons	Solanaceae	*Petunia*	*Petunia altiplana*	DQ791913
Eudicotyledons	Solanaceae	*Petunia*	*Petunia altiplana*	DQ791914
Eudicotyledons	Solanaceae	*Petunia*	*Petunia altiplana*	DQ791915
Eudicotyledons	Solanaceae	*Petunia*	*Petunia altiplana*	DQ791916

Eudicotyledons	Solanaceae	*Petunia*	*Petunia altiplana*	DQ791917
Eudicotyledons	Solanaceae	*Petunia*	*Petunia altiplana*	DQ791918
Eudicotyledons	Solanaceae	*Petunia*	*Petunia altiplana*	DQ791919
Eudicotyledons	Solanaceae	*Petunia*	*Petunia altiplana*	DQ791920
Eudicotyledons	Solanaceae	*Petunia*	*Petunia altiplana*	DQ791921
Eudicotyledons	Solanaceae	*Petunia*	*Petunia altiplana*	DQ791922
Eudicotyledons	Solanaceae	*Petunia*	*Petunia altiplana*	DQ791923
Eudicotyledons	Solanaceae	*Petunia*	*Petunia altiplana*	DQ791924
Eudicotyledons	Solanaceae	*Petunia*	*Petunia altiplana*	DQ791925
Eudicotyledons	Solanaceae	*Petunia*	*Petunia altiplana*	DQ791926
Eudicotyledons	Solanaceae	*Petunia*	*Petunia altiplana*	DQ791927
Eudicotyledons	Solanaceae	*Petunia*	*Petunia altiplana*	DQ791928
Eudicotyledons	Solanaceae	*Petunia*	*Petunia altiplana*	DQ791929
Eudicotyledons	Solanaceae	*Petunia*	*Petunia altiplana*	DQ791930
Eudicotyledons	Solanaceae	*Petunia*	*Petunia altiplana*	DQ791931
Eudicotyledons	Solanaceae	*Petunia*	*Petunia altiplana*	DQ791932
Eudicotyledons	Solanaceae	*Petunia*	*Petunia altiplana*	DQ791933
Eudicotyledons	Solanaceae	*Petunia*	*Petunia altiplana*	DQ791934
Eudicotyledons	Solanaceae	*Petunia*	*Petunia altiplana*	DQ791935
Eudicotyledons	Solanaceae	*Petunia*	*Petunia altiplana*	DQ791936
Eudicotyledons	Solanaceae	*Petunia*	*Petunia altiplana*	DQ791937
Eudicotyledons	Solanaceae	*Petunia*	*Petunia altiplana*	DQ791938
Eudicotyledons	Solanaceae	*Petunia*	*Petunia altiplana*	DQ791939
Eudicotyledons	Solanaceae	*Petunia*	*Petunia altiplana*	DQ791940
Eudicotyledons	Solanaceae	*Petunia*	*Petunia altiplana*	DQ791941
Eudicotyledons	Solanaceae	*Petunia*	*Petunia altiplana*	DQ791942
Eudicotyledons	Solanaceae	*Petunia*	*Petunia altiplana*	DQ791943
Eudicotyledons	Solanaceae	*Petunia*	*Petunia altiplana*	DQ791944
Eudicotyledons	Solanaceae	*Petunia*	*Petunia altiplana*	DQ791945
Eudicotyledons	Solanaceae	*Petunia*	*Petunia altiplana*	DQ791946
Eudicotyledons	Solanaceae	*Petunia*	*Petunia altiplana*	DQ791947
Eudicotyledons	Solanaceae	*Petunia*	*Petunia altiplana*	DQ791948
Eudicotyledons	Solanaceae	*Petunia*	*Petunia altiplana*	DQ791949
Eudicotyledons	Solanaceae	*Petunia*	*Petunia altiplana*	DQ791950
Eudicotyledons	Solanaceae	*Petunia*	*Petunia altiplana*	DQ791951
Eudicotyledons	Solanaceae	*Petunia*	*Petunia altiplana*	DQ791952
Eudicotyledons	Solanaceae	*Petunia*	*Petunia altiplana*	DQ791953
Eudicotyledons	Solanaceae	*Petunia*	*Petunia altiplana*	DQ791954
Eudicotyledons	Solanaceae	*Petunia*	*Petunia altiplana*	DQ791955
Eudicotyledons	Solanaceae	*Petunia*	*Petunia altiplana*	DQ791956
Eudicotyledons	Solanaceae	*Petunia*	*Petunia altiplana*	DQ791957
Eudicotyledons	Solanaceae	*Petunia*	*Petunia altiplana*	DQ791958
Eudicotyledons	Solanaceae	*Petunia*	*Petunia altiplana*	DQ791959
Eudicotyledons	Solanaceae	*Petunia*	*Petunia altiplana*	DQ791960
Eudicotyledons	Solanaceae	*Petunia*	*Petunia altiplana*	DQ791961
Eudicotyledons	Solanaceae	*Petunia*	*Petunia altiplana*	DQ791962
Eudicotyledons	Solanaceae	*Petunia*	*Petunia altiplana*	DQ791963
Eudicotyledons	Solanaceae	*Petunia*	*Petunia altiplana*	DQ791964
Eudicotyledons	Solanaceae	*Petunia*	*Petunia altiplana*	DQ791965
Eudicotyledons	Solanaceae	*Petunia*	*Petunia altiplana*	DQ791966
Eudicotyledons	Solanaceae	*Petunia*	*Petunia altiplana*	DQ791967
Eudicotyledons	Solanaceae	*Petunia*	*Petunia altiplana*	DQ791968
Eudicotyledons	Solanaceae	*Petunia*	*Petunia altiplana*	DQ791969

Eudicotyledons	Solanaceae	*Petunia*	*Petunia altiplana*	DQ791970
Eudicotyledons	Solanaceae	*Petunia*	*Petunia altiplana*	DQ791971
Eudicotyledons	Solanaceae	*Petunia*	*Petunia altiplana*	DQ791972
Eudicotyledons	Solanaceae	*Petunia*	*Petunia altiplana*	DQ791973
Eudicotyledons	Solanaceae	*Petunia*	*Petunia altiplana*	DQ791974
Eudicotyledons	Solanaceae	*Petunia*	*Petunia altiplana*	DQ791975
Eudicotyledons	Solanaceae	*Petunia*	*Petunia altiplana*	DQ791976
Eudicotyledons	Solanaceae	*Petunia*	*Petunia altiplana*	DQ791977
Eudicotyledons	Solanaceae	*Petunia*	*Petunia altiplana*	DQ791978
Eudicotyledons	Solanaceae	*Petunia*	*Petunia altiplana*	DQ791979
Eudicotyledons	Solanaceae	*Petunia*	*Petunia altiplana*	DQ791980
Eudicotyledons	Solanaceae	*Petunia*	*Petunia altiplana*	DQ791981
Eudicotyledons	Solanaceae	*Petunia*	*Petunia altiplana*	DQ791982
Eudicotyledons	Solanaceae	*Petunia*	*Petunia altiplana*	DQ791983
Eudicotyledons	Solanaceae	*Petunia*	*Petunia altiplana*	DQ791984
Eudicotyledons	Solanaceae	*Petunia*	*Petunia altiplana*	DQ791985
Eudicotyledons	Solanaceae	*Petunia*	*Petunia altiplana*	DQ791986
Eudicotyledons	Solanaceae	*Petunia*	*Petunia altiplana*	DQ791987
Eudicotyledons	Solanaceae	*Petunia*	*Petunia altiplana*	DQ791988
Eudicotyledons	Solanaceae	*Petunia*	*Petunia altiplana*	DQ791989
Eudicotyledons	Solanaceae	*Petunia*	*Petunia altiplana*	DQ791990
Eudicotyledons	Solanaceae	*Petunia*	*Petunia altiplana*	DQ791991
Eudicotyledons	Solanaceae	*Petunia*	*Petunia altiplana*	DQ791992
Eudicotyledons	Solanaceae	*Petunia*	*Petunia altiplana*	DQ791993
Eudicotyledons	Solanaceae	*Petunia*	*Petunia altiplana*	DQ791994
Eudicotyledons	Solanaceae	*Petunia*	*Petunia altiplana*	DQ791995
Eudicotyledons	Solanaceae	*Petunia*	*Petunia altiplana*	DQ791996
Eudicotyledons	Solanaceae	*Petunia*	*Petunia altiplana*	DQ791997
Eudicotyledons	Solanaceae	*Petunia*	*Petunia altiplana*	DQ791998
Eudicotyledons	Solanaceae	*Petunia*	*Petunia altiplana*	AY772887
Eudicotyledons	Solanaceae	*Petunia*	*Petunia altiplana*	AY772888
Eudicotyledons	Solanaceae	*Petunia*	*Petunia altiplana*	AY772889
Eudicotyledons	Solanaceae	*Petunia*	*Petunia altiplana*	AY772890
Eudicotyledons	Solanaceae	*Petunia*	*Petunia altiplana*	AY772891
Eudicotyledons	Solanaceae	*Petunia*	*Petunia bonjardinensis*	DQ791999
Eudicotyledons	Solanaceae	*Petunia*	*Petunia bonjardinensis*	DQ792000
Eudicotyledons	Solanaceae	*Petunia*	*Petunia bonjardinensis*	DQ792001
Eudicotyledons	Solanaceae	*Petunia*	*Petunia bonjardinensis*	DQ792002
Eudicotyledons	Solanaceae	*Petunia*	*Petunia bonjardinensis*	DQ792003
Eudicotyledons	Solanaceae	*Petunia*	*Petunia bonjardinensis*	DQ792004
Eudicotyledons	Solanaceae	*Petunia*	*Petunia bonjardinensis*	DQ792005
Eudicotyledons	Solanaceae	*Petunia*	*Petunia bonjardinensis*	DQ792006
Eudicotyledons	Solanaceae	*Petunia*	*Petunia bonjardinensis*	DQ792007
Eudicotyledons	Solanaceae	*Petunia*	*Petunia bonjardinensis*	DQ792008
Eudicotyledons	Solanaceae	*Petunia*	*Petunia bonjardinensis*	DQ792009
Eudicotyledons	Solanaceae	*Petunia*	*Petunia bonjardinensis*	DQ792010
Eudicotyledons	Solanaceae	*Petunia*	*Petunia bonjardinensis*	DQ792011
Eudicotyledons	Solanaceae	*Petunia*	*Petunia bonjardinensis*	DQ792012
Eudicotyledons	Solanaceae	*Petunia*	*Petunia bonjardinensis*	DQ792013
Eudicotyledons	Solanaceae	*Petunia*	*Petunia bonjardinensis*	DQ792014
Eudicotyledons	Solanaceae	*Petunia*	*Petunia bonjardinensis*	DQ792015
Eudicotyledons	Solanaceae	*Petunia*	*Petunia bonjardinensis*	DQ792016
Eudicotyledons	Solanaceae	*Petunia*	*Petunia bonjardinensis*	DQ792017

Eudicotyledons	Solanaceae	*Petunia*	*Petunia bonjardinensis*	DQ792018
Eudicotyledons	Solanaceae	*Petunia*	*Petunia bonjardinensis*	DQ792019
Eudicotyledons	Solanaceae	*Petunia*	*Petunia bonjardinensis*	DQ792020
Eudicotyledons	Solanaceae	*Petunia*	*Petunia bonjardinensis*	DQ792021
Eudicotyledons	Solanaceae	*Petunia*	*Petunia bonjardinensis*	DQ792022
Eudicotyledons	Solanaceae	*Petunia*	*Petunia bonjardinensis*	DQ792023
Eudicotyledons	Solanaceae	*Petunia*	*Petunia bonjardinensis*	DQ792024
Eudicotyledons	Solanaceae	*Petunia*	*Petunia bonjardinensis*	DQ792025
Eudicotyledons	Solanaceae	*Petunia*	*Petunia bonjardinensis*	DQ792026
Eudicotyledons	Solanaceae	*Petunia*	*Petunia bonjardinensis*	DQ792027
Eudicotyledons	Solanaceae	*Petunia*	*Petunia bonjardinensis*	DQ792028
Eudicotyledons	Solanaceae	*Petunia*	*Petunia bonjardinensis*	DQ792029
Eudicotyledons	Solanaceae	*Petunia*	*Petunia bonjardinensis*	DQ792030
Eudicotyledons	Solanaceae	*Petunia*	*Petunia bonjardinensis*	DQ792031
Eudicotyledons	Solanaceae	*Petunia*	*Petunia bonjardinensis*	DQ792032
Eudicotyledons	Solanaceae	*Petunia*	*Petunia bonjardinensis*	DQ792033
Eudicotyledons	Solanaceae	*Petunia*	*Petunia bonjardinensis*	DQ792034
Eudicotyledons	Solanaceae	*Petunia*	*Petunia bonjardinensis*	DQ792035
Eudicotyledons	Solanaceae	*Petunia*	*Petunia bonjardinensis*	DQ792036
Eudicotyledons	Solanaceae	*Petunia*	*Petunia bonjardinensis*	DQ792037
Eudicotyledons	Solanaceae	*Petunia*	*Petunia bonjardinensis*	DQ792038
Eudicotyledons	Solanaceae	*Petunia*	*Petunia bonjardinensis*	DQ792039
Eudicotyledons	Solanaceae	*Petunia*	*Petunia bonjardinensis*	DQ792040
Eudicotyledons	Solanaceae	*Petunia*	*Petunia bonjardinensis*	DQ792041
Eudicotyledons	Solanaceae	*Petunia*	*Petunia bonjardinensis*	DQ792042
Eudicotyledons	Solanaceae	*Petunia*	*Petunia bonjardinensis*	DQ792043
Eudicotyledons	Solanaceae	*Petunia*	*Petunia bonjardinensis*	DQ792044
Eudicotyledons	Solanaceae	*Petunia*	*Petunia bonjardinensis*	DQ792045
Eudicotyledons	Solanaceae	*Petunia*	*Petunia bonjardinensis*	DQ792046
Eudicotyledons	Solanaceae	*Petunia*	*Petunia bonjardinensis*	DQ792047
Eudicotyledons	Solanaceae	*Petunia*	*Petunia bonjardinensis*	DQ792048
Eudicotyledons	Solanaceae	*Petunia*	*Petunia bonjardinensis*	DQ792049
Eudicotyledons	Solanaceae	*Petunia*	*Petunia bonjardinensis*	DQ792050
Eudicotyledons	Solanaceae	*Petunia*	*Petunia bonjardinensis*	DQ792051
Eudicotyledons	Solanaceae	*Petunia*	*Petunia bonjardinensis*	DQ792052
Eudicotyledons	Solanaceae	*Petunia*	*Petunia bonjardinensis*	DQ792053
Eudicotyledons	Solanaceae	*Petunia*	*Petunia bonjardinensis*	DQ792054
Eudicotyledons	Solanaceae	*Petunia*	*Petunia bonjardinensis*	DQ792055
Eudicotyledons	Solanaceae	*Petunia*	*Petunia bonjardinensis*	DQ792056
Eudicotyledons	Solanaceae	*Petunia*	*Petunia bonjardinensis*	DQ792057
Eudicotyledons	Solanaceae	*Petunia*	*Petunia bonjardinensis*	DQ792058
Eudicotyledons	Solanaceae	*Petunia*	*Petunia bonjardinensis*	AY772911
Eudicotyledons	Solanaceae	*Petunia*	*Petunia bonjardinensis*	AY772912
Eudicotyledons	Solanaceae	*Petunia*	*Petunia bonjardinensis*	AY772913
Eudicotyledons	Solanaceae	*Petunia*	*Petunia bonjardinensis*	AY772914
Eudicotyledons	Solanaceae	*Petunia*	*Petunia bonjardinensis*	AY772915
Eudicotyledons	Solanaceae	*Petunia*	*Petunia mantiqueirensis*	DQ792059
Eudicotyledons	Solanaceae	*Petunia*	*Petunia mantiqueirensis*	DQ792060
Eudicotyledons	Solanaceae	*Petunia*	*Petunia mantiqueirensis*	DQ792061
Eudicotyledons	Solanaceae	*Petunia*	*Petunia mantiqueirensis*	DQ792062
Eudicotyledons	Solanaceae	*Petunia*	*Petunia mantiqueirensis*	DQ792063
Eudicotyledons	Solanaceae	*Petunia*	*Petunia mantiqueirensis*	DQ792064
Eudicotyledons	Solanaceae	*Petunia*	*Petunia mantiqueirensis*	DQ792065

Eudicotyledons	Solanaceae	*Petunia*	*Petunia mantiqueirensis*	DQ792066
Eudicotyledons	Solanaceae	*Petunia*	*Petunia mantiqueirensis*	DQ792067
Eudicotyledons	Solanaceae	*Petunia*	*Petunia mantiqueirensis*	DQ792068
Eudicotyledons	Solanaceae	*Petunia*	*Petunia mantiqueirensis*	DQ792069
Eudicotyledons	Solanaceae	*Petunia*	*Petunia mantiqueirensis*	DQ792070
Eudicotyledons	Solanaceae	*Petunia*	*Petunia mantiqueirensis*	DQ792071
Eudicotyledons	Solanaceae	*Petunia*	*Petunia mantiqueirensis*	DQ792072
Eudicotyledons	Solanaceae	*Petunia*	*Petunia mantiqueirensis*	DQ792073
Eudicotyledons	Solanaceae	*Petunia*	*Petunia mantiqueirensis*	DQ792074
Eudicotyledons	Solanaceae	*Petunia*	*Petunia mantiqueirensis*	DQ792075
Eudicotyledons	Solanaceae	*Petunia*	*Petunia mantiqueirensis*	DQ792076
Eudicotyledons	Solanaceae	*Petunia*	*Petunia mantiqueirensis*	DQ792077
Eudicotyledons	Solanaceae	*Petunia*	*Petunia mantiqueirensis*	DQ792078
Eudicotyledons	Solanaceae	*Petunia*	*Petunia mantiqueirensis*	DQ792079
Eudicotyledons	Solanaceae	*Petunia*	*Petunia mantiqueirensis*	AY772920
Eudicotyledons	Solanaceae	*Petunia*	*Petunia mantiqueirensis*	AY772921
Eudicotyledons	Solanaceae	*Petunia*	*Petunia mantiqueirensis*	AY772922
Eudicotyledons	Solanaceae	*Petunia*	*Petunia mantiqueirensis*	AY772923
Eudicotyledons	Solanaceae	*Petunia*	*Petunia reitzii*	DQ792080
Eudicotyledons	Solanaceae	*Petunia*	*Petunia reitzii*	DQ792081
Eudicotyledons	Solanaceae	*Petunia*	*Petunia reitzii*	DQ792082
Eudicotyledons	Solanaceae	*Petunia*	*Petunia reitzii*	DQ792083
Eudicotyledons	Solanaceae	*Petunia*	*Petunia reitzii*	DQ792084
Eudicotyledons	Solanaceae	*Petunia*	*Petunia reitzii*	DQ792085
Eudicotyledons	Solanaceae	*Petunia*	*Petunia reitzii*	DQ792086
Eudicotyledons	Solanaceae	*Petunia*	*Petunia reitzii*	DQ792087
Eudicotyledons	Solanaceae	*Petunia*	*Petunia reitzii*	DQ792088
Eudicotyledons	Solanaceae	*Petunia*	*Petunia reitzii*	DQ792089
Eudicotyledons	Solanaceae	*Petunia*	*Petunia reitzii*	DQ792090
Eudicotyledons	Solanaceae	*Petunia*	*Petunia reitzii*	DQ792091
Eudicotyledons	Solanaceae	*Petunia*	*Petunia reitzii*	DQ792092
Eudicotyledons	Solanaceae	*Petunia*	*Petunia reitzii*	DQ792093
Eudicotyledons	Solanaceae	*Petunia*	*Petunia reitzii*	DQ792094
Eudicotyledons	Solanaceae	*Petunia*	*Petunia reitzii*	DQ792095
Eudicotyledons	Solanaceae	*Petunia*	*Petunia reitzii*	DQ792096
Eudicotyledons	Solanaceae	*Petunia*	*Petunia reitzii*	DQ792097
Eudicotyledons	Solanaceae	*Petunia*	*Petunia reitzii*	DQ792098
Eudicotyledons	Solanaceae	*Petunia*	*Petunia reitzii*	DQ792099
Eudicotyledons	Solanaceae	*Petunia*	*Petunia reitzii*	DQ792100
Eudicotyledons	Solanaceae	*Petunia*	*Petunia reitzii*	DQ792101
Eudicotyledons	Solanaceae	*Petunia*	*Petunia reitzii*	AY772916
Eudicotyledons	Solanaceae	*Petunia*	*Petunia reitzii*	AY772917
Eudicotyledons	Solanaceae	*Petunia*	*Petunia reitzii*	AY772918
Eudicotyledons	Solanaceae	*Petunia*	*Petunia reitzii*	AY772919
Eudicotyledons	Solanaceae	*Petunia*	*Petunia saxicola*	DQ792102
Eudicotyledons	Solanaceae	*Petunia*	*Petunia saxicola*	DQ792103
Eudicotyledons	Solanaceae	*Petunia*	*Petunia saxicola*	DQ792104
Eudicotyledons	Solanaceae	*Petunia*	*Petunia saxicola*	DQ792105
Eudicotyledons	Solanaceae	*Petunia*	*Petunia saxicola*	DQ792106
Eudicotyledons	Solanaceae	*Petunia*	*Petunia saxicola*	DQ792107
Eudicotyledons	Solanaceae	*Petunia*	*Petunia saxicola*	DQ792108
Eudicotyledons	Solanaceae	*Petunia*	*Petunia saxicola*	DQ792109
Eudicotyledons	Solanaceae	*Petunia*	*Petunia saxicola*	DQ792110

Eudicotyledons	Solanaceae	*Petunia*	*Petunia saxicola*	DQ792111
Eudicotyledons	Solanaceae	*Petunia*	*Petunia saxicola*	DQ792112
Eudicotyledons	Solanaceae	*Petunia*	*Petunia saxicola*	DQ792113
Eudicotyledons	Solanaceae	*Petunia*	*Petunia saxicola*	DQ792114
Eudicotyledons	Solanaceae	*Petunia*	*Petunia saxicola*	DQ792115
Eudicotyledons	Solanaceae	*Petunia*	*Petunia saxicola*	DQ792116
Eudicotyledons	Solanaceae	*Petunia*	*Petunia saxicola*	DQ792117
Eudicotyledons	Solanaceae	*Petunia*	*Petunia saxicola*	DQ792118
Eudicotyledons	Solanaceae	*Petunia*	*Petunia saxicola*	DQ792119
Eudicotyledons	Solanaceae	*Petunia*	*Petunia saxicola*	DQ792120
Eudicotyledons	Solanaceae	*Petunia*	*Petunia saxicola*	DQ792121
Eudicotyledons	Solanaceae	*Petunia*	*Petunia saxicola*	DQ792122
Eudicotyledons	Solanaceae	*Petunia*	*Petunia saxicola*	DQ792123
Eudicotyledons	Solanaceae	*Petunia*	*Petunia saxicola*	DQ792124
Eudicotyledons	Solanaceae	*Petunia*	*Petunia saxicola*	DQ792125
Eudicotyledons	Solanaceae	*Petunia*	*Petunia saxicola*	AY772902
Eudicotyledons	Solanaceae	*Petunia*	*Petunia saxicola*	AY772903
Eudicotyledons	Solanaceae	*Petunia*	*Petunia secreta*	AY772895
Eudicotyledons	Solanaceae	*Petunia*	*Petunia secreta*	AY772896
Eudicotyledons	Solanaceae	*Petunia*	*Petunia secreta*	AY772897
Eudicotyledons	Solanaceae	*Petunia*	*Petunia littoralis*	AY772898
Eudicotyledons	Solanaceae	*Petunia*	*Petunia littoralis*	AY772899
Eudicotyledons	Solanaceae	*Petunia*	*Petunia littoralis*	AY772900
Eudicotyledons	Solanaceae	*Petunia*	*Petunia littoralis*	AY772901
Eudicotyledons	Solanaceae	*Petunia*	*Petunia littoralis*	DQ208155
Eudicotyledons	Solanaceae	*Petunia*	*Petunia littoralis*	DQ208156
Eudicotyledons	Solanaceae	*Petunia*	*Petunia littoralis*	DQ208157
Eudicotyledons	Solanaceae	*Petunia*	*Petunia littoralis*	DQ208158
Eudicotyledons	Solanaceae	*Petunia*	*Petunia littoralis*	DQ208159
Eudicotyledons	Solanaceae	*Petunia*	*Petunia littoralis*	DQ208160
Eudicotyledons	Solanaceae	*Petunia*	*Petunia littoralis*	DQ208161
Eudicotyledons	Solanaceae	*Petunia*	*Petunia scheideana*	DQ792126
Eudicotyledons	Solanaceae	*Petunia*	*Petunia scheideana*	DQ792127
Eudicotyledons	Solanaceae	*Petunia*	*Petunia scheideana*	DQ792128
Eudicotyledons	Solanaceae	*Petunia*	*Petunia scheideana*	DQ792129
Eudicotyledons	Solanaceae	*Petunia*	*Petunia scheideana*	DQ792130
Eudicotyledons	Solanaceae	*Petunia*	*Petunia scheideana*	DQ792131
Eudicotyledons	Solanaceae	*Petunia*	*Petunia scheideana*	DQ792132
Eudicotyledons	Solanaceae	*Petunia*	*Petunia scheideana*	DQ792133
Eudicotyledons	Solanaceae	*Petunia*	*Petunia scheideana*	DQ792134
Eudicotyledons	Solanaceae	*Petunia*	*Petunia scheideana*	DQ792135
Eudicotyledons	Solanaceae	*Petunia*	*Petunia scheideana*	DQ792136
Eudicotyledons	Solanaceae	*Petunia*	*Petunia scheideana*	DQ792137
Eudicotyledons	Solanaceae	*Petunia*	*Petunia scheideana*	DQ792138
Eudicotyledons	Solanaceae	*Petunia*	*Petunia scheideana*	DQ792139
Eudicotyledons	Solanaceae	*Petunia*	*Petunia scheideana*	DQ792140
Eudicotyledons	Solanaceae	*Petunia*	*Petunia scheideana*	DQ792141
Eudicotyledons	Solanaceae	*Petunia*	*Petunia scheideana*	DQ792142
Eudicotyledons	Solanaceae	*Petunia*	*Petunia scheideana*	DQ792143
Eudicotyledons	Solanaceae	*Petunia*	*Petunia scheideana*	DQ792144
Eudicotyledons	Solanaceae	*Petunia*	*Petunia scheideana*	DQ792145
Eudicotyledons	Solanaceae	*Petunia*	*Petunia scheideana*	DQ792146
Eudicotyledons	Solanaceae	*Petunia*	*Petunia scheideana*	DQ792147

Eudicotyledons	Solanaceae	*Petunia*	*Petunia scheideana*	DQ792148
Eudicotyledons	Solanaceae	*Petunia*	*Petunia scheideana*	DQ792149
Eudicotyledons	Solanaceae	*Petunia*	*Petunia scheideana*	DQ792150
Eudicotyledons	Solanaceae	*Petunia*	*Petunia scheideana*	DQ792151
Eudicotyledons	Solanaceae	*Petunia*	*Petunia scheideana*	DQ792152
Eudicotyledons	Solanaceae	*Petunia*	*Petunia scheideana*	DQ792153
Eudicotyledons	Solanaceae	*Petunia*	*Petunia scheideana*	DQ792154
Eudicotyledons	Solanaceae	*Petunia*	*Petunia scheideana*	DQ792155
Eudicotyledons	Solanaceae	*Petunia*	*Petunia scheideana*	DQ792156
Eudicotyledons	Solanaceae	*Petunia*	*Petunia scheideana*	DQ792157
Eudicotyledons	Solanaceae	*Petunia*	*Petunia scheideana*	DQ792158
Eudicotyledons	Solanaceae	*Petunia*	*Petunia scheideana*	DQ792159
Eudicotyledons	Solanaceae	*Petunia*	*Petunia scheideana*	DQ792160
Eudicotyledons	Solanaceae	*Petunia*	*Petunia scheideana*	DQ792161
Eudicotyledons	Solanaceae	*Petunia*	*Petunia scheideana*	DQ792162
Eudicotyledons	Solanaceae	*Petunia*	*Petunia scheideana*	DQ792163
Eudicotyledons	Solanaceae	*Petunia*	*Petunia scheideana*	DQ792164
Eudicotyledons	Solanaceae	*Petunia*	*Petunia scheideana*	DQ792165
Eudicotyledons	Solanaceae	*Petunia*	*Petunia scheideana*	DQ792166
Eudicotyledons	Solanaceae	*Petunia*	*Petunia scheideana*	DQ792167
Eudicotyledons	Solanaceae	*Petunia*	*Petunia scheideana*	DQ792168
Eudicotyledons	Solanaceae	*Petunia*	*Petunia scheideana*	DQ792169
Eudicotyledons	Solanaceae	*Petunia*	*Petunia scheideana*	DQ792170
Eudicotyledons	Solanaceae	*Petunia*	*Petunia scheideana*	DQ792171
Eudicotyledons	Solanaceae	*Petunia*	*Petunia scheideana*	DQ792172
Eudicotyledons	Solanaceae	*Petunia*	*Petunia scheideana*	DQ792173
Eudicotyledons	Solanaceae	*Petunia*	*Petunia scheideana*	DQ792174
Eudicotyledons	Solanaceae	*Petunia*	*Petunia scheideana*	DQ792175
Eudicotyledons	Solanaceae	*Petunia*	*Petunia scheideana*	DQ792176
Eudicotyledons	Solanaceae	*Petunia*	*Petunia scheideana*	DQ792177
Eudicotyledons	Solanaceae	*Petunia*	*Petunia scheideana*	DQ792178
Eudicotyledons	Solanaceae	*Petunia*	*Petunia scheideana*	DQ792179
Eudicotyledons	Solanaceae	*Petunia*	*Petunia scheideana*	DQ792180
Eudicotyledons	Solanaceae	*Petunia*	*Petunia scheideana*	DQ792181
Eudicotyledons	Solanaceae	*Petunia*	*Petunia scheideana*	DQ792182
Eudicotyledons	Solanaceae	*Petunia*	*Petunia scheideana*	DQ792183
Eudicotyledons	Solanaceae	*Petunia*	*Petunia scheideana*	AY772924
Eudicotyledons	Solanaceae	*Petunia*	*Petunia scheideana*	AY772925
Eudicotyledons	Solanaceae	*Petunia*	*Petunia exserta*	AY772892
Eudicotyledons	Solanaceae	*Petunia*	*Petunia exserta*	AY772893
Eudicotyledons	Solanaceae	*Petunia*	*Petunia exserta*	AY772894
Eudicotyledons	Solanaceae	*Petunia*	*Petunia exserta*	DQ225666
Eudicotyledons	Solanaceae	*Petunia*	*Petunia exserta*	DQ225667
Eudicotyledons	Solanaceae	*Petunia*	*Petunia exserta*	DQ225668
Eudicotyledons	Solanaceae	*Petunia*	*Petunia exserta*	DQ225669
Eudicotyledons	Solanaceae	*Petunia*	*Petunia exserta*	DQ225670
Eudicotyledons	Solanaceae	*Petunia*	*Petunia exserta*	DQ225671
Eudicotyledons	Solanaceae	*Petunia*	*Petunia exserta*	DQ225672
Eudicotyledons	Solanaceae	*Petunia*	*Petunia exserta*	DQ225673
Eudicotyledons	Solanaceae	*Petunia*	*Petunia exserta*	DQ225674
Eudicotyledons	Solanaceae	*Petunia*	*Petunia exserta*	DQ225675
Eudicotyledons	Solanaceae	*Petunia*	*Petunia exserta*	DQ225676
Eudicotyledons	Solanaceae	*Petunia*	*Petunia exserta*	DQ225677

Eudicotyledons	Solanaceae	*Petunia*	*Petunia exserta*	DQ225678
Eudicotyledons	Solanaceae	*Petunia*	*Petunia exserta*	DQ225679
Eudicotyledons	Solanaceae	*Petunia*	*Petunia exserta*	DQ225680
Eudicotyledons	Solanaceae	*Petunia*	*Petunia exserta*	DQ225681
Eudicotyledons	Solanaceae	*Petunia*	*Petunia exserta*	DQ225682
Eudicotyledons	Solanaceae	*Petunia*	*Petunia exserta*	DQ225683
Eudicotyledons	Solanaceae	*Petunia*	*Petunia exserta*	DQ225684
Eudicotyledons	Solanaceae	*Petunia*	*Petunia exserta*	DQ225685
Eudicotyledons	Solanaceae	*Petunia*	*Petunia exserta*	DQ225686
Eudicotyledons	Solanaceae	*Petunia*	*Petunia exserta*	DQ225687
Eudicotyledons	Solanaceae	*Petunia*	*Petunia exserta*	DQ225688
Eudicotyledons	Solanaceae	*Petunia*	*Petunia exserta*	DQ225689
Eudicotyledons	Solanaceae	*Petunia*	*Petunia exserta*	DQ225690
Eudicotyledons	Solanaceae	*Petunia*	*Petunia exserta*	DQ225691
Eudicotyledons	Solanaceae	*Petunia*	*Petunia exserta*	DQ225692
Eudicotyledons	Solanaceae	*Petunia*	*Petunia exserta*	DQ225693
Eudicotyledons	Solanaceae	*Petunia*	*Petunia exserta*	DQ225694
Eudicotyledons	Solanaceae	*Petunia*	*Petunia exserta*	DQ225695
Eudicotyledons	Solanaceae	*Petunia*	*Petunia exserta*	DQ225696
Eudicotyledons	Solanaceae	*Petunia*	*Petunia exserta*	DQ225697
Eudicotyledons	Solanaceae	*Petunia*	*Petunia exserta*	DQ225698
Eudicotyledons	Solanaceae	*Petunia*	*Petunia exserta*	DQ225699
Eudicotyledons	Solanaceae	*Petunia*	*Petunia exserta*	DQ225700
Eudicotyledons	Solanaceae	*Petunia*	*Petunia exserta*	DQ225701
Eudicotyledons	Solanaceae	*Petunia*	*Petunia exserta*	DQ225702
Eudicotyledons	Solanaceae	*Petunia*	*Petunia exserta*	DQ225703
Eudicotyledons	Solanaceae	*Petunia*	*Petunia exserta*	DQ225704
Eudicotyledons	Solanaceae	*Petunia*	*Petunia exserta*	DQ225705
Eudicotyledons	Solanaceae	*Petunia*	*Petunia exserta*	DQ225706
Eudicotyledons	Solanaceae	*Petunia*	*Petunia exserta*	DQ225707
Eudicotyledons	Solanaceae	*Petunia*	*Petunia exserta*	DQ225708
Eudicotyledons	Solanaceae	*Petunia*	*Petunia exserta*	DQ225709
Eudicotyledons	Solanaceae	*Petunia*	*Petunia exserta*	DQ225710
Eudicotyledons	Celastraceae	*Parnassia*	*Parnassia faberi*	JF802324
Eudicotyledons	Celastraceae	*Parnassia*	*Parnassia faberi*	JF802325
Eudicotyledons	Celastraceae	*Parnassia*	*Parnassia faberi*	JF802326
Eudicotyledons	Celastraceae	*Parnassia*	*Parnassia faberi*	JF802327
Eudicotyledons	Celastraceae	*Parnassia*	*Parnassia faberi*	JF802328
Eudicotyledons	Celastraceae	*Parnassia*	*Parnassia faberi*	JF802329
Eudicotyledons	Celastraceae	*Parnassia*	*Parnassia faberi*	JN045806
Eudicotyledons	Celastraceae	*Parnassia*	*Parnassia faberi*	JN045807
Eudicotyledons	Celastraceae	*Parnassia*	*Parnassia faberi*	JN045808
Eudicotyledons	Celastraceae	*Parnassia*	*Parnassia faberi*	JN045809
Eudicotyledons	Celastraceae	*Parnassia*	*Parnassia laxmannii*	JN045825
Eudicotyledons	Celastraceae	*Parnassia*	*Parnassia laxmannii*	JN045826
Eudicotyledons	Celastraceae	*Parnassia*	*Parnassia laxmannii*	JN045827
Eudicotyledons	Celastraceae	*Parnassia*	*Parnassia laxmannii*	JN045828
Eudicotyledons	Celastraceae	*Parnassia*	*Parnassia tenella*	JF802371
Eudicotyledons	Celastraceae	*Parnassia*	*Parnassia tenella*	JF802372
Eudicotyledons	Celastraceae	*Parnassia*	*Parnassia tenella*	JF802373
Eudicotyledons	Celastraceae	*Parnassia*	*Parnassia tenella*	JF802374
Eudicotyledons	Celastraceae	*Parnassia*	*Parnassia tenella*	JF802375
Eudicotyledons	Celastraceae	*Parnassia*	*Parnassia tenella*	JN045852

Eudicotyledons	Celastraceae	*Parnassia*	*Parnassia tenella*	JN045853
Eudicotyledons	Celastraceae	*Parnassia*	*Parnassia tenella*	JN045854
Eudicotyledons	Celastraceae	*Parnassia*	*Parnassia trinervis*	JF802376
Eudicotyledons	Celastraceae	*Parnassia*	*Parnassia trinervis*	JF802377
Eudicotyledons	Celastraceae	*Parnassia*	*Parnassia trinervis*	JF802378
Eudicotyledons	Celastraceae	*Parnassia*	*Parnassia trinervis*	JF802379
Eudicotyledons	Celastraceae	*Parnassia*	*Parnassia trinervis*	JF802380
Eudicotyledons	Celastraceae	*Parnassia*	*Parnassia trinervis*	JN045855
Eudicotyledons	Celastraceae	*Parnassia*	*Parnassia trinervis*	JN045856
Eudicotyledons	Celastraceae	*Parnassia*	*Parnassia trinervis*	JN045857
Eudicotyledons	Celastraceae	*Parnassia*	*Parnassia trinervis*	JN045858
Eudicotyledons	Celastraceae	*Parnassia*	*Parnassia trinervis*	JN045859
Eudicotyledons	Celastraceae	*Parnassia*	*Parnassia trinervis*	JN045860
Eudicotyledons	Celastraceae	*Parnassia*	*Parnassia trinervis*	JN045861
Eudicotyledons	Orobanchaceae	*Pedicularis*	*Pedicularis alaschanica*	JN045877
Eudicotyledons	Orobanchaceae	*Pedicularis*	*Pedicularis alaschanica*	JN045878
Eudicotyledons	Orobanchaceae	*Pedicularis*	*Pedicularis alaschanica*	JN045879
Eudicotyledons	Orobanchaceae	*Pedicularis*	*Pedicularis cranolopha*	JN045922
Eudicotyledons	Orobanchaceae	*Pedicularis*	*Pedicularis cranolopha*	JN045923
Eudicotyledons	Orobanchaceae	*Pedicularis*	*Pedicularis cranolopha*	JN045924
Eudicotyledons	Orobanchaceae	*Pedicularis*	*Pedicularis cranolopha*	JN045925
Eudicotyledons	Orobanchaceae	*Pedicularis*	*Pedicularis cranolopha*	JN045926
Eudicotyledons	Orobanchaceae	*Pedicularis*	*Pedicularis cranolopha*	JN045927
Eudicotyledons	Orobanchaceae	*Pedicularis*	*Pedicularis cranolopha*	JN045928
Eudicotyledons	Orobanchaceae	*Pedicularis*	*Pedicularis davidii*	JN045947
Eudicotyledons	Orobanchaceae	*Pedicularis*	*Pedicularis davidii*	JN045948
Eudicotyledons	Orobanchaceae	*Pedicularis*	*Pedicularis davidii*	JN045949
Eudicotyledons	Orobanchaceae	*Pedicularis*	*Pedicularis decorissima*	JN045955
Eudicotyledons	Orobanchaceae	*Pedicularis*	*Pedicularis decorissima*	JN045956
Eudicotyledons	Orobanchaceae	*Pedicularis*	*Pedicularis dolichocymba*	JN045970
Eudicotyledons	Orobanchaceae	*Pedicularis*	*Pedicularis dolichocymba*	JN045971
Eudicotyledons	Orobanchaceae	*Pedicularis*	*Pedicularis elwesii*	JN045975
Eudicotyledons	Orobanchaceae	*Pedicularis*	*Pedicularis elwesii*	JN045976
Eudicotyledons	Orobanchaceae	*Pedicularis*	*Pedicularis elwesii*	JN045977
Eudicotyledons	Orobanchaceae	*Pedicularis*	*Pedicularis elwesii*	JN045978
Eudicotyledons	Orobanchaceae	*Pedicularis*	*Pedicularis gracilis*	JN045988
Eudicotyledons	Orobanchaceae	*Pedicularis*	*Pedicularis gracilis*	JN045989
Eudicotyledons	Orobanchaceae	*Pedicularis*	*Pedicularis gracilis*	JN045990
Eudicotyledons	Orobanchaceae	*Pedicularis*	*Pedicularis gracilis*	JN045991
Eudicotyledons	Orobanchaceae	*Pedicularis*	*Pedicularis lyrata*	JN046045
Eudicotyledons	Orobanchaceae	*Pedicularis*	*Pedicularis lyrata*	JN046046
Eudicotyledons	Orobanchaceae	*Pedicularis*	*Pedicularis lyrata*	JN046047
Eudicotyledons	Orobanchaceae	*Pedicularis*	*Pedicularis lyrata*	JN046048
Eudicotyledons	Orobanchaceae	*Pedicularis*	*Pedicularis lyrata*	JN046049
Eudicotyledons	Orobanchaceae	*Pedicularis*	*Pedicularis lyrata*	JN046050
Eudicotyledons	Orobanchaceae	*Pedicularis*	*Pedicularis lyrata*	JN046051
Eudicotyledons	Orobanchaceae	*Pedicularis*	*Pedicularis megalantha*	JN046059
Eudicotyledons	Orobanchaceae	*Pedicularis*	*Pedicularis megalantha*	JN046060
Eudicotyledons	Orobanchaceae	*Pedicularis*	*Pedicularis oederi*	JN046068
Eudicotyledons	Orobanchaceae	*Pedicularis*	*Pedicularis oederi*	JN046069
Eudicotyledons	Orobanchaceae	*Pedicularis*	*Pedicularis oederi*	JN046070
Eudicotyledons	Orobanchaceae	*Pedicularis*	*Pedicularis oederi*	JN046071
Eudicotyledons	Orobanchaceae	*Pedicularis*	*Pedicularis przewalskii*	JN046080

Eudicotyledons	Orobanchaceae	*Pedicularis*	*Pedicularis przewalskii*	JN046081
Eudicotyledons	Orobanchaceae	*Pedicularis*	*Pedicularis przewalskii*	JN046082
Eudicotyledons	Orobanchaceae	*Pedicularis*	*Pedicularis rhinanthoides*	JN046099
Eudicotyledons	Orobanchaceae	*Pedicularis*	*Pedicularis rhinanthoides*	JN046100
Eudicotyledons	Orobanchaceae	*Pedicularis*	*Pedicularis rhinanthoides*	JN046101
Eudicotyledons	Orobanchaceae	*Pedicularis*	*Pedicularis rhinanthoides*	JN046102
Eudicotyledons	Orobanchaceae	*Pedicularis*	*Pedicularis rhinanthoides*	JN046103
Eudicotyledons	Orobanchaceae	*Pedicularis*	*Pedicularis thamnophila*	JN046164
Eudicotyledons	Orobanchaceae	*Pedicularis*	*Pedicularis thamnophila*	JN046165
Eudicotyledons	Orobanchaceae	*Pedicularis*	*Pedicularis thamnophila*	JN046166
Eudicotyledons	Orobanchaceae	*Pedicularis*	*Pedicularis thamnophila*	JN046167
Eudicotyledons	Orobanchaceae	*Pedicularis*	*Pedicularis thamnophila*	JN046168
Eudicotyledons	Orobanchaceae	*Pedicularis*	*Pedicularis tibetica*	JN046169
Eudicotyledons	Orobanchaceae	*Pedicularis*	*Pedicularis tibetica*	JN046170
Eudicotyledons	Orobanchaceae	*Pedicularis*	*Pedicularis tibetica*	JN046171
Eudicotyledons	Orobanchaceae	*Pedicularis*	*Pedicularis tibetica*	JN046172
Eudicotyledons	Solanaceae	*Solanum*	*Solanum campylacanthum*	HM016419
Eudicotyledons	Solanaceae	*Solanum*	*Solanum campylacanthum*	HM016420
Eudicotyledons	Solanaceae	*Solanum*	*Solanum campylacanthum*	HM016424
Eudicotyledons	Solanaceae	*Solanum*	*Solanum linnaeanum*	HM016425
Eudicotyledons	Solanaceae	*Solanum*	*Solanum linnaeanum*	HM016429
Eudicotyledons	Solanaceae	*Solanum*	*Solanum incanum*	HM016427
Eudicotyledons	Solanaceae	*Solanum*	*Solanum incanum*	HM016430
Eudicotyledons	Solanaceae	*Solanum*	*Solanum panduriforme*	HM016417
Eudicotyledons	Solanaceae	*Solanum*	*Solanum panduriforme*	HM016428
Eudicotyledons	Solanaceae	*Solanum*	*Solanum panduriforme*	EU213851
Eudicotyledons	Solanaceae	*Solanum*	*Solanum panduriforme*	EU213852
Eudicotyledons	Solanaceae	*Solanum*	*Solanum panduriforme*	EU213853
Eudicotyledons	Rosaceae	*Prunus*	*Prunus kansuensis*	JN046630
Eudicotyledons	Rosaceae	*Prunus*	*Prunus kansuensis*	JN046631
Eudicotyledons	Rosaceae	*Prunus*	*Prunus kansuensis*	JN046632
Eudicotyledons	Rosaceae	*Prunus*	*Prunus kansuensis*	JN046633
Eudicotyledons	Rosaceae	*Prunus*	*Prunus kansuensis*	JN046634
Eudicotyledons	Campanulaceae	*Lobelia*	*Lobelia siphilitica*	HQ596752
Eudicotyledons	Campanulaceae	*Lobelia*	*Lobelia siphilitica*	DQ006198
Eudicotyledons	Caryophyllaceae	*Silene*	*Silene patula*	FJ890524
Eudicotyledons	Caryophyllaceae	*Silene*	*Silene patula*	FJ890525
Eudicotyledons	Caryophyllaceae	*Silene*	*Silene patula*	FJ890526
Eudicotyledons	Caryophyllaceae	*Silene*	*Silene patula*	FJ890527
Eudicotyledons	Caryophyllaceae	*Silene*	*Silene patula*	FJ890528
Eudicotyledons	Caryophyllaceae	*Silene*	*Silene patula*	FJ890529
Eudicotyledons	Caryophyllaceae	*Silene*	*Silene patula*	FJ890530
Eudicotyledons	Plantaginaceae	*Antirrhinum*	*Antirrhinum pertegasii*	HM152935
Eudicotyledons	Plantaginaceae	*Antirrhinum*	*Antirrhinum pertegasii*	HM152936
Eudicotyledons	Ericaceae	*Rhododendron*	*Rhododendron decorum*	JN046786
Eudicotyledons	Ericaceae	*Rhododendron*	*Rhododendron decorum*	JN046787
Eudicotyledons	Ericaceae	*Rhododendron*	*Rhododendron decorum*	JN046788
Eudicotyledons	Ericaceae	*Rhododendron*	*Rhododendron decorum*	JN046789
Eudicotyledons	Ericaceae	*Rhododendron*	*Rhododendron decorum*	JN046790
Eudicotyledons	Ericaceae	*Rhododendron*	*Rhododendron decorum*	JN046791
Eudicotyledons	Ericaceae	*Rhododendron*	*Rhododendron decorum*	JN046792
Eudicotyledons	Ericaceae	*Rhododendron*	*Rhododendron decorum*	HQ706959
Eudicotyledons	Vitaceae	*Parthenocissus*	*Parthenocissus tricuspidata*	HQ108339

Eudicotyledons	Vitaceae	*Parthenocissus*	*Parthenocissus tricuspidata*	HQ656486
Eudicotyledons	Vitaceae	*Parthenocissus*	*Parthenocissus tricuspidata*	JF437135
Eudicotyledons	Vitaceae	*Vitis*	*Vitis betulifolia*	HQ108311
Eudicotyledons	Vitaceae	*Vitis*	*Vitis betulifolia*	HQ656451
Eudicotyledons	Vitaceae	*Vitis*	*Vitis betulifolia*	HQ656462
Eudicotyledons	Vitaceae	*Vitis*	*Vitis betulifolia*	JF437157
Eudicotyledons	Vitaceae	*Vitis*	*Vitis heyneana*	JN802332
Eudicotyledons	Vitaceae	*Vitis*	*Vitis heyneana*	HQ656457
Eudicotyledons	Vitaceae	*Vitis*	*Vitis heyneana*	HQ656464
Eudicotyledons	Vitaceae	*Vitis*	*Vitis heyneana*	HQ656465
Eudicotyledons	Vitaceae	*Vitis*	*Vitis heyneana*	JF437160
Eudicotyledons	Vitaceae	*Vitis*	*Vitis heyneana*	JF437161
Eudicotyledons	Vitaceae	*Vitis*	*Vitis tiliifolia*	HQ656479
Eudicotyledons	Vitaceae	*Vitis*	*Vitis tiliifolia*	JF437168
Eudicotyledons	Geraniaceae	*Geranium*	*Geranium sibiricum*	JN044742
Eudicotyledons	Geraniaceae	*Geranium*	*Geranium sibiricum*	JN044743
Eudicotyledons	Linaceae	*Linum*	*Linum bienne*	GQ845292
Eudicotyledons	Linaceae	*Linum*	*Linum bienne*	GQ845293
Eudicotyledons	Linaceae	*Linum*	*Linum bienne*	GQ845294
Eudicotyledons	Celastraceae	*Euonymus*	*Euonymus oxyphyllus*	AB525283
Eudicotyledons	Celastraceae	*Euonymus*	*Euonymus oxyphyllus*	AB525284
Eudicotyledons	Celastraceae	*Euonymus*	*Euonymus oxyphyllus*	AB525285
Eudicotyledons	Celastraceae	*Euonymus*	*Euonymus oxyphyllus*	AB525286
Eudicotyledons	Celastraceae	*Euonymus*	*Euonymus oxyphyllus*	AB525287
Eudicotyledons	Celastraceae	*Euonymus*	*Euonymus oxyphyllus*	AB525288
Eudicotyledons	Celastraceae	*Euonymus*	*Euonymus oxyphyllus*	AB525289
Eudicotyledons	Celastraceae	*Euonymus*	*Euonymus oxyphyllus*	AB525290
Eudicotyledons	Celastraceae	*Euonymus*	*Euonymus oxyphyllus*	AB525291
Eudicotyledons	Celastraceae	*Euonymus*	*Euonymus oxyphyllus*	AB525292
Eudicotyledons	Celastraceae	*Euonymus*	*Euonymus oxyphyllus*	AB525293
Eudicotyledons	Celastraceae	*Euonymus*	*Euonymus oxyphyllus*	AB525294
Eudicotyledons	Celastraceae	*Euonymus*	*Euonymus oxyphyllus*	AB525295
Eudicotyledons	Celastraceae	*Euonymus*	*Euonymus oxyphyllus*	AB525296
Eudicotyledons	Celastraceae	*Euonymus*	*Euonymus oxyphyllus*	AB525297
Eudicotyledons	Celastraceae	*Euonymus*	*Euonymus oxyphyllus*	AB525298
Eudicotyledons	Celastraceae	*Euonymus*	*Euonymus oxyphyllus*	AB525299
Eudicotyledons	Celastraceae	*Euonymus*	*Euonymus oxyphyllus*	AB525300
Eudicotyledons	Celastraceae	*Euonymus*	*Euonymus oxyphyllus*	AB525301
Eudicotyledons	Celastraceae	*Euonymus*	*Euonymus oxyphyllus*	AB525302
Eudicotyledons	Celastraceae	*Euonymus*	*Euonymus oxyphyllus*	AB525303
Eudicotyledons	Celastraceae	*Euonymus*	*Euonymus oxyphyllus*	AB525304
Eudicotyledons	Celastraceae	*Euonymus*	*Euonymus oxyphyllus*	AB525305
Eudicotyledons	Celastraceae	*Euonymus*	*Euonymus oxyphyllus*	AB525306
Eudicotyledons	Celastraceae	*Euonymus*	*Euonymus oxyphyllus*	AB525307
Eudicotyledons	Celastraceae	*Euonymus*	*Euonymus oxyphyllus*	AB525308
Eudicotyledons	Celastraceae	*Euonymus*	*Euonymus oxyphyllus*	AB525309
Eudicotyledons	Aceraceae	*Acer*	*Acer pycnanthum*	DQ978623
Eudicotyledons	Aceraceae	*Acer*	*Acer pycnanthum*	GQ855775
Eudicotyledons	Aceraceae	*Acer*	*Acer pycnanthum*	GQ855776
Eudicotyledons	Asteraceae	*Centaurea*	*Centaurea exarata*	DQ846216
Eudicotyledons	Asteraceae	*Centaurea*	*Centaurea exarata*	DQ846217
Eudicotyledons	Asteraceae	*Centaurea*	*Centaurea exarata*	DQ846218
Eudicotyledons	Asteraceae	*Centaurea*	*Centaurea exarata*	DQ846228

Eudicotyledons	Asteraceae	*Centaurea*	*Centaurea exarata*	DQ846229
Eudicotyledons	Asteraceae	*Centaurea*	*Centaurea exarata*	DQ846230
Eudicotyledons	Asteraceae	*Centaurea*	*Centaurea exarata*	DQ846246
Eudicotyledons	Asteraceae	*Centaurea*	*Centaurea exarata*	DQ846247
Eudicotyledons	Asteraceae	*Centaurea*	*Centaurea exarata*	DQ846248
Eudicotyledons	Nitrariaceae	*Nitraria*	*Nitraria sphaerocarpa*	JN045584
Eudicotyledons	Nitrariaceae	*Nitraria*	*Nitraria sphaerocarpa*	JN045585
Eudicotyledons	Nitrariaceae	*Nitraria*	*Nitraria sphaerocarpa*	JN045586
Eudicotyledons	Nitrariaceae	*Nitraria*	*Nitraria sibirica*	JN045581
Eudicotyledons	Nitrariaceae	*Nitraria*	*Nitraria sibirica*	JN045582
Eudicotyledons	Nitrariaceae	*Nitraria*	*Nitraria sibirica*	JN045583
Eudicotyledons	Nitrariaceae	*Nitraria*	*Nitraria roborowskii*	JN045579
Eudicotyledons	Nitrariaceae	*Nitraria*	*Nitraria roborowskii*	JN045580
Eudicotyledons	Asteraceae	*Ligularia*	*Ligularia hookeri*	JN045192
Eudicotyledons	Asteraceae	*Ligularia*	*Ligularia hookeri*	JN045193
Eudicotyledons	Apiaceae	*Heracleum*	*Heracleum antasiaticum*	EU594936
Eudicotyledons	Apiaceae	*Heracleum*	*Heracleum antasiaticum*	EU594937
Eudicotyledons	Apiaceae	*Heracleum*	*Heracleum antasiaticum*	EU594938
Eudicotyledons	Solanaceae	*Petunia*	*Petunia axillaris x Petunia exserta*	DQ225711
Eudicotyledons	Solanaceae	*Petunia*	*Petunia axillaris x Petunia exserta*	DQ225712
Eudicotyledons	Solanaceae	*Petunia*	*Petunia axillaris x Petunia exserta*	DQ225713
Eudicotyledons	Solanaceae	*Petunia*	*Petunia axillaris x Petunia exserta*	DQ225714
Eudicotyledons	Solanaceae	*Petunia*	*Petunia axillaris x Petunia exserta*	DQ225715
Eudicotyledons	Solanaceae	*Petunia*	*Petunia axillaris x Petunia exserta*	DQ225716
Eudicotyledons	Solanaceae	*Petunia*	*Petunia axillaris x Petunia exserta*	DQ225717
Eudicotyledons	Solanaceae	*Petunia*	*Petunia axillaris x Petunia exserta*	DQ225718
Eudicotyledons	Solanaceae	*Petunia*	*Petunia axillaris x Petunia exserta*	DQ225719
Eudicotyledons	Solanaceae	*Petunia*	*Petunia axillaris x Petunia exserta*	DQ225720
Eudicotyledons	Solanaceae	*Petunia*	*Petunia axillaris x Petunia exserta*	DQ225721
Eudicotyledons	Solanaceae	*Petunia*	*Petunia axillaris x Petunia exserta*	DQ225722
Eudicotyledons	Solanaceae	*Petunia*	*Petunia axillaris x Petunia exserta*	DQ225723
Eudicotyledons	Solanaceae	*Petunia*	*Petunia axillaris x Petunia exserta*	DQ225724
Eudicotyledons	Solanaceae	*Petunia*	*Petunia axillaris x Petunia exserta*	DQ225725
Eudicotyledons	Solanaceae	*Petunia*	*Petunia axillaris x Petunia exserta*	DQ225726
Eudicotyledons	Solanaceae	*Petunia*	*Petunia axillaris x Petunia exserta*	DQ225727
Eudicotyledons	Solanaceae	*Petunia*	*Petunia axillaris x Petunia exserta*	DQ225728
Eudicotyledons	Solanaceae	*Petunia*	*Petunia axillaris x Petunia exserta*	DQ225729
Eudicotyledons	Gentianaceae	*Swertia*	*Swertia tenuis*	JN047297
Eudicotyledons	Gentianaceae	*Swertia*	*Swertia tenuis*	JN047298
Eudicotyledons	Cornaceae	*Cornus*	*Cornus rugosa*	EU750450
Eudicotyledons	Cornaceae	*Cornus*	*Cornus rugosa*	EU750451
Eudicotyledons	Campanulaceae	*Lobelia*	*Lobelia chinensis*	GQ435063
Eudicotyledons	Campanulaceae	*Lobelia*	*Lobelia chinensis*	JN040996
Eudicotyledons	Fabaceae	*Albizia*	*Albizia lebbeck*	GU135326
Eudicotyledons	Fabaceae	*Albizia*	*Albizia lebbeck*	EU811983
Eudicotyledons	Myrtaceae	*Myrcia*	*Myrcia coumeta*	AM489864
Eudicotyledons	Myrtaceae	*Myrcia*	*Myrcia coumeta*	GQ248348
Eudicotyledons	Myrtaceae	*Myrcia*	*Myrcia fallax*	AM489865
Eudicotyledons	Myrtaceae	*Myrcia*	*Myrcia fallax*	GQ248349
Eudicotyledons	Myrtaceae	*Myrcia*	*Myrcia fallax*	HM446968
Eudicotyledons	Asteraceae	*Lychnophora*	*Lychnophora salicifolia*	FJ031849
Eudicotyledons	Asteraceae	*Lychnophora*	*Lychnophora salicifolia*	FJ031850
Eudicotyledons	Asteraceae	*Lychnophora*	*Lychnophora salicifolia*	FJ031851

Eudicotyledons	Asteraceae	*Lychnophora*	*Lychnophora salicifolia*	FJ031852
Eudicotyledons	Asteraceae	*Lychnophora*	*Lychnophora salicifolia*	FJ031853
Eudicotyledons	Asteraceae	*Lychnophora*	*Lychnophora salicifolia*	FJ031854
Eudicotyledons	Asteraceae	*Lychnophora*	*Lychnophora salicifolia*	FJ031855
Eudicotyledons	Asteraceae	*Lychnophora*	*Lychnophora salicifolia*	FJ031856
Eudicotyledons	Asteraceae	*Lychnophora*	*Lychnophora salicifolia*	FJ031857
Eudicotyledons	Asteraceae	*Lychnophora*	*Lychnophora salicifolia*	FJ031858
Eudicotyledons	Asteraceae	*Lychnophora*	*Lychnophora salicifolia*	FJ031859
Eudicotyledons	Asteraceae	*Lychnophora*	*Lychnophora salicifolia*	FJ031860
Eudicotyledons	Asteraceae	*Lychnophora*	*Lychnophora salicifolia*	FJ031861
Eudicotyledons	Asteraceae	*Lychnophora*	*Lychnophora salicifolia*	FJ031862
Eudicotyledons	Asteraceae	*Lychnophora*	*Lychnophora salicifolia*	FJ031863
Eudicotyledons	Asteraceae	*Lychnophora*	*Lychnophora salicifolia*	FJ031864
Eudicotyledons	Asteraceae	*Lychnophora*	*Lychnophora salicifolia*	FJ031865
Eudicotyledons	Asteraceae	*Lychnophora*	*Lychnophora salicifolia*	FJ031866
Eudicotyledons	Asteraceae	*Arctotis*	*Arctotis acaulis*	DQ444765
Eudicotyledons	Asteraceae	*Arctotis*	*Arctotis acaulis*	EU846414
Eudicotyledons	Asteraceae	*Arctotis*	*Arctotis arctotoides*	DQ444766
Eudicotyledons	Asteraceae	*Arctotis*	*Arctotis arctotoides*	EU846405
Eudicotyledons	Asteraceae	*Arctotis*	*Arctotis aspera*	DQ444767
Eudicotyledons	Asteraceae	*Arctotis*	*Arctotis aspera*	EU846418
Eudicotyledons	Asteraceae	*Arctotis*	*Arctotis dregei*	DQ444769
Eudicotyledons	Asteraceae	*Arctotis*	*Arctotis dregei*	EU846403
Eudicotyledons	Asteraceae	*Arctotis*	*Arctotis perfoliata*	DQ444770
Eudicotyledons	Asteraceae	*Arctotis*	*Arctotis perfoliata*	EU846411
Eudicotyledons	Asteraceae	*Haplocarpha*	*Haplocarpha nervosa*	DQ444778
Eudicotyledons	Asteraceae	*Haplocarpha*	*Haplocarpha nervosa*	EU846394
Eudicotyledons	Asteraceae	*Haplocarpha*	*Haplocarpha rueppellii*	DQ444779
Eudicotyledons	Asteraceae	*Haplocarpha*	*Haplocarpha rueppellii*	EU846395
Eudicotyledons	Asteraceae	*Haplocarpha*	*Haplocarpha schimperi*	DQ444781
Eudicotyledons	Asteraceae	*Haplocarpha*	*Haplocarpha schimperi*	EU846397
Eudicotyledons	Apiaceae	*Heracleum*	*Heracleum dissectum*	EF042117
Eudicotyledons	Apiaceae	*Heracleum*	*Heracleum dissectum*	GU967815
Eudicotyledons	Apiaceae	*Heracleum*	*Heracleum leskovii*	DQ869371
Eudicotyledons	Apiaceae	*Heracleum*	*Heracleum leskovii*	DQ927299
Eudicotyledons	Cucurbitaceae	*Trichosanthes*	*Trichosanthes ovigera*	HQ829557
Eudicotyledons	Cucurbitaceae	*Trichosanthes*	*Trichosanthes ovigera*	HQ829558
Eudicotyledons	Cucurbitaceae	*Trichosanthes*	*Trichosanthes ovigera*	HQ829559
Eudicotyledons	Cucurbitaceae	*Trichosanthes*	*Trichosanthes ovigera*	HQ829560
Eudicotyledons	Cucurbitaceae	*Trichosanthes*	*Trichosanthes ovigera*	HQ829561
Eudicotyledons	Cucurbitaceae	*Trichosanthes*	*Trichosanthes ovigera*	HQ829562
Eudicotyledons	Cucurbitaceae	*Trichosanthes*	*Trichosanthes ovigera*	HQ829563
Eudicotyledons	Cucurbitaceae	*Trichosanthes*	*Trichosanthes ovigera*	HQ829564
Eudicotyledons	Cucurbitaceae	*Trichosanthes*	*Trichosanthes ovigera*	HQ829565
Eudicotyledons	Vitaceae	*Vitis*	*Vitis bryoniifolia*	HQ656445
Eudicotyledons	Vitaceae	*Vitis*	*Vitis bryoniifolia*	HQ656460
Eudicotyledons	Vitaceae	*Vitis*	*Vitis bryoniifolia*	HQ656461
Eudicotyledons	Aceraceae	*Acer*	*Acer pectinatum*	HM008586
Eudicotyledons	Aceraceae	*Acer*	*Acer pectinatum*	JN043726
Eudicotyledons	Aceraceae	*Acer*	*Acer pectinatum*	JN043727
Eudicotyledons	Aceraceae	*Acer*	*Acer pectinatum*	JN043728
Eudicotyledons	Ericaceae	*Rhododendron*	*Rhododendron agastum*	JN046735
Eudicotyledons	Ericaceae	*Rhododendron*	*Rhododendron agastum*	JN046736

Eudicotyledons	Ericaceae	*Rhododendron*	*Rhododendron agastum*	HQ706949
Eudicotyledons	Ericaceae	*Rhododendron*	*Rhododendron agastum*	HQ706950
Eudicotyledons	Celastraceae	*Parnassia*	*Parnassia kotzebuei*	JF802344
Eudicotyledons	Celastraceae	*Parnassia*	*Parnassia kotzebuei*	JF802345
Eudicotyledons	Celastraceae	*Parnassia*	*Parnassia kotzebuei*	JN045822
Eudicotyledons	Celastraceae	*Parnassia*	*Parnassia kotzebuei*	JN045823
Eudicotyledons	Celastraceae	*Parnassia*	*Parnassia kotzebuei*	JN045824
Eudicotyledons	Salicaceae	*Salix*	*Salix polaris*	GU373291
Eudicotyledons	Salicaceae	*Salix*	*Salix polaris*	GU373292
Eudicotyledons	Lamiaceae	*Scutellaria*	*Scutellaria barbata*	JQ339246
Eudicotyledons	Lamiaceae	*Scutellaria*	*Scutellaria barbata*	JQ339247
Eudicotyledons	Lamiaceae	*Scutellaria*	*Scutellaria barbata*	JQ339248
Eudicotyledons	Lamiaceae	*Scutellaria*	*Scutellaria barbata*	EU590859
Eudicotyledons	Rosaceae	*Rosa*	*Rosa bella*	DQ778752
Eudicotyledons	Rosaceae	*Rosa*	*Rosa bella*	GQ435258
Eudicotyledons	Rosaceae	*Rosa*	*Rosa bridgesii*	DQ778755
Eudicotyledons	Rosaceae	*Rosa*	*Rosa bridgesii*	DQ778818
Eudicotyledons	Rosaceae	*Rosa*	*Rosa minutifolia*	DQ778786
Eudicotyledons	Rosaceae	*Rosa*	*Rosa minutifolia*	DQ778787
Eudicotyledons	Asteraceae	*Centaurea*	*Centaurea filiformis*	DQ846186
Eudicotyledons	Asteraceae	*Centaurea*	*Centaurea filiformis*	DQ846187
Eudicotyledons	Asteraceae	*Centaurea*	*Centaurea filiformis*	DQ846188
Eudicotyledons	Asteraceae	*Centaurea*	*Centaurea filiformis*	DQ846252
Eudicotyledons	Asteraceae	*Centaurea*	*Centaurea filiformis*	DQ846253
Eudicotyledons	Asteraceae	*Centaurea*	*Centaurea filiformis*	DQ846254
Eudicotyledons	Asteraceae	*Centaurea*	*Centaurea filiformis*	DQ846255
Eudicotyledons	Asteraceae	*Centaurea*	*Centaurea filiformis*	DQ846256
Eudicotyledons	Asteraceae	*Centaurea*	*Centaurea filiformis*	DQ846257
Eudicotyledons	Asteraceae	*Centaurea*	*Centaurea subtilis*	DQ846219
Eudicotyledons	Asteraceae	*Centaurea*	*Centaurea subtilis*	DQ846220
Eudicotyledons	Asteraceae	*Centaurea*	*Centaurea subtilis*	DQ846221
Eudicotyledons	Orobanchaceae	*Pedicularis*	*Pedicularis dichotoma*	JN045964
Eudicotyledons	Orobanchaceae	*Pedicularis*	*Pedicularis dichotoma*	JN045965
Eudicotyledons	Orobanchaceae	*Pedicularis*	*Pedicularis dichotoma*	JN045966
Eudicotyledons	Orobanchaceae	*Pedicularis*	*Pedicularis dichotoma*	JN045967
Eudicotyledons	Orobanchaceae	*Pedicularis*	*Pedicularis glabrescens*	JN045984
Eudicotyledons	Orobanchaceae	*Pedicularis*	*Pedicularis glabrescens*	JN045985
Eudicotyledons	Orobanchaceae	*Pedicularis*	*Pedicularis glabrescens*	JN045986
Eudicotyledons	Orobanchaceae	*Pedicularis*	*Pedicularis glabrescens*	JN045987
Eudicotyledons	Ericaceae	*Rhododendron*	*Rhododendron aberconwayi*	JN046731
Eudicotyledons	Ericaceae	*Rhododendron*	*Rhododendron aberconwayi*	JN046732
Eudicotyledons	Ericaceae	*Rhododendron*	*Rhododendron anthosphaerum*	JN046739
Eudicotyledons	Ericaceae	*Rhododendron*	*Rhododendron anthosphaerum*	JN046740
Eudicotyledons	Ericaceae	*Rhododendron*	*Rhododendron anthosphaerum*	JN046741
Eudicotyledons	Ericaceae	*Rhododendron*	*Rhododendron anthosphaerum*	JN046742
Eudicotyledons	Ericaceae	*Rhododendron*	*Rhododendron anthosphaerum*	HQ706954
Eudicotyledons	Ericaceae	*Rhododendron*	*Rhododendron fastigiatum*	JN046808
Eudicotyledons	Ericaceae	*Rhododendron*	*Rhododendron fastigiatum*	JN046809
Eudicotyledons	Ericaceae	*Rhododendron*	*Rhododendron fastigiatum*	JN046810
Eudicotyledons	Ericaceae	*Rhododendron*	*Rhododendron fastigiatum*	JN046811
Eudicotyledons	Ericaceae	*Rhododendron*	*Rhododendron sinogrande*	JN046964
Eudicotyledons	Ericaceae	*Rhododendron*	*Rhododendron sinogrande*	JN046965
Eudicotyledons	Ericaceae	*Rhododendron*	*Rhododendron sinogrande*	JN046966

Eudicotyledons	Ericaceae	*Rhododendron*	*Rhododendron sinogrande*	JN046967
Eudicotyledons	Ericaceae	*Rhododendron*	*Rhododendron sinogrande*	JN046968
Eudicotyledons	Ericaceae	*Rhododendron*	*Rhododendron lukiangense*	JN046869
Eudicotyledons	Ericaceae	*Rhododendron*	*Rhododendron lukiangense*	JN046870
Eudicotyledons	Ericaceae	*Rhododendron*	*Rhododendron lukiangense*	JN046871
Eudicotyledons	Ericaceae	*Rhododendron*	*Rhododendron rubiginosum*	JN046937
Eudicotyledons	Ericaceae	*Rhododendron*	*Rhododendron rubiginosum*	JN046938
Eudicotyledons	Ericaceae	*Rhododendron*	*Rhododendron rubiginosum*	JN046939
Eudicotyledons	Ericaceae	*Rhododendron*	*Rhododendron rubiginosum*	JN046940
Eudicotyledons	Ericaceae	*Rhododendron*	*Rhododendron rubiginosum*	JN046941
Eudicotyledons	Ericaceae	*Rhododendron*	*Rhododendron rubiginosum*	JN046942
Eudicotyledons	Ericaceae	*Rhododendron*	*Rhododendron rubiginosum*	JN046943
Eudicotyledons	Ericaceae	*Rhododendron*	*Rhododendron rubiginosum*	HQ707017
Eudicotyledons	Vitaceae	*Parthenocissus*	*Parthenocissus vitacea*	HM585681
Eudicotyledons	Vitaceae	*Parthenocissus*	*Parthenocissus vitacea*	JF437136
Eudicotyledons	Vitaceae	*Parthenocissus*	*Parthenocissus chinensis*	JQ182502
Eudicotyledons	Vitaceae	*Parthenocissus*	*Parthenocissus chinensis*	JF437127
Eudicotyledons	Vitaceae	*Parthenocissus*	*Parthenocissus chinensis*	JF437128
Eudicotyledons	Vitaceae	*Parthenocissus*	*Parthenocissus dalzielii*	JF437129
Eudicotyledons	Vitaceae	*Parthenocissus*	*Parthenocissus dalzielii*	JF437130
Eudicotyledons	Vitaceae	*Vitis*	*Vitis popenoei*	HM585790
Eudicotyledons	Vitaceae	*Vitis*	*Vitis popenoei*	JF437164
Eudicotyledons	Ericaceae	*Rhododendron*	*Rhododendron x pulchrum*	HQ707011
Eudicotyledons	Ericaceae	*Rhododendron*	*Rhododendron x pulchrum*	HQ707012
Eudicotyledons	Ericaceae	*Rhododendron*	*Rhododendron x pulchrum*	HQ707013
Eudicotyledons	Fabaceae	*Acacia*	*Acacia saligna*	FJ808535
Eudicotyledons	Fabaceae	*Acacia*	*Acacia saligna*	FJ808536
Eudicotyledons	Fabaceae	*Acacia*	*Acacia saligna*	FJ808537
Eudicotyledons	Fabaceae	*Acacia*	*Acacia saligna*	FJ808538
Eudicotyledons	Oxalidaceae	*Oxalis*	*Oxalis sp. JZ-2007*	EF040587
Eudicotyledons	Oxalidaceae	*Oxalis*	*Oxalis sp. JZ-2007*	EF040588
Eudicotyledons	Oxalidaceae	*Oxalis*	*Oxalis sp. JZ-2007*	EF040589
Eudicotyledons	Oxalidaceae	*Oxalis*	*Oxalis sp. JZ-2007*	EF040590
Eudicotyledons	Oxalidaceae	*Oxalis*	*Oxalis sp. JZ-2007*	EF040591
Eudicotyledons	Oxalidaceae	*Oxalis*	*Oxalis sp. JZ-2007*	EF040592
Eudicotyledons	Oxalidaceae	*Oxalis*	*Oxalis sp. JZ-2007*	EF040593
Eudicotyledons	Oxalidaceae	*Oxalis*	*Oxalis sp. JZ-2007*	EF040594
Eudicotyledons	Oxalidaceae	*Oxalis*	*Oxalis sp. JZ-2007*	EF040595
Eudicotyledons	Oxalidaceae	*Oxalis*	*Oxalis sp. JZ-2007*	EF040596
Eudicotyledons	Oxalidaceae	*Oxalis*	*Oxalis sp. JZ-2007*	EF040597
Eudicotyledons	Convolvulaceae	*Convolvulus*	*Convolvulus x despreauxii*	EF375930
Eudicotyledons	Convolvulaceae	*Convolvulus*	*Convolvulus x despreauxii*	EF375931
Eudicotyledons	Convolvulaceae	*Convolvulus*	*Convolvulus x despreauxii*	EF375932
Eudicotyledons	Convolvulaceae	*Convolvulus*	*Convolvulus x despreauxii*	EF375933
Eudicotyledons	Convolvulaceae	*Convolvulus*	*Convolvulus x despreauxii*	EF375934
Eudicotyledons	Convolvulaceae	*Convolvulus*	*Convolvulus x despreauxii*	EF375935
Eudicotyledons	Convolvulaceae	*Convolvulus*	*Convolvulus x despreauxii*	EF375936
Eudicotyledons	Convolvulaceae	*Convolvulus*	*Convolvulus x despreauxii*	EF375937
Eudicotyledons	Convolvulaceae	*Convolvulus*	*Convolvulus x despreauxii*	EF375938
Eudicotyledons	Convolvulaceae	*Convolvulus*	*Convolvulus x despreauxii*	EF375939
Eudicotyledons	Convolvulaceae	*Convolvulus*	*Convolvulus x despreauxii*	EF375940
Eudicotyledons	Convolvulaceae	*Convolvulus*	*Convolvulus x despreauxii*	EF375941
Eudicotyledons	Celastraceae	*Parnassia*	*Parnassia grandifolia*	JF802337

Eudicotyledons	Celastraceae	*Parnassia*	*Parnassia grandifolia*	JF802338
Eudicotyledons	Asteraceae	*Ligularia*	*Ligularia dictyoneura*	JN045189
Eudicotyledons	Asteraceae	*Ligularia*	*Ligularia dictyoneura*	JN045190
Eudicotyledons	Asteraceae	*Ligularia*	*Ligularia dictyoneura*	JN045191
Eudicotyledons	Asteraceae	*Arnica*	*Arnica gracilis*	AM690558
Eudicotyledons	Asteraceae	*Arnica*	*Arnica gracilis*	AM690584
Eudicotyledons	Asteraceae	*Arnica*	*Arnica montana*	AM690567
Eudicotyledons	Asteraceae	*Arnica*	*Arnica montana*	AM690582
Eudicotyledons	Asteraceae	*Arnica*	*Arnica nevadensis*	AM690557
Eudicotyledons	Asteraceae	*Arnica*	*Arnica nevadensis*	AM690561
Eudicotyledons	Asteraceae	*Arnica*	*Arnica ovata*	AM690564
Eudicotyledons	Asteraceae	*Arnica*	*Arnica ovata*	AM690576
Eudicotyledons	Asteraceae	*Arnica*	*Arnica spathulata*	AM690560
Eudicotyledons	Asteraceae	*Arnica*	*Arnica spathulata*	AM690583
Eudicotyledons	Asteraceae	*Arnica*	*Arnica acaulis*	AM690559
Eudicotyledons	Asteraceae	*Arnica*	*Arnica acaulis*	AM690587
Eudicotyledons	Adoxaceae	*Viburnum*	*Viburnum foetidum*	HQ592075
Eudicotyledons	Adoxaceae	*Viburnum*	*Viburnum foetidum*	JN047509
Eudicotyledons	Adoxaceae	*Viburnum*	*Viburnum foetidum*	JN047510
Eudicotyledons	Adoxaceae	*Viburnum*	*Viburnum foetidum*	JN047511
Eudicotyledons	Adoxaceae	*Viburnum*	*Viburnum foetidum*	JN047512
Eudicotyledons	Adoxaceae	*Viburnum*	*Viburnum foetidum*	JN047513
Eudicotyledons	Adoxaceae	*Viburnum*	*Viburnum setigerum*	HQ592096
Eudicotyledons	Adoxaceae	*Viburnum*	*Viburnum setigerum*	EF590749
Eudicotyledons	Begoniaceae	*Begonia*	*Begonia baviensis*	JN044003
Eudicotyledons	Begoniaceae	*Begonia*	*Begonia baviensis*	JN044004
Eudicotyledons	Begoniaceae	*Begonia*	*Begonia baviensis*	JN044005
Eudicotyledons	Begoniaceae	*Begonia*	*Begonia baviensis*	JN044006
Eudicotyledons	Begoniaceae	*Begonia*	*Begonia baviensis*	JN044007
Eudicotyledons	Begoniaceae	*Begonia*	*Begonia baviensis*	JN044008
Eudicotyledons	Adoxaceae	*Viburnum*	*Viburnum cotinifolium*	GQ248401
Eudicotyledons	Adoxaceae	*Viburnum*	*Viburnum cotinifolium*	EF590748
Eudicotyledons	Fabaceae	*Leucaena*	*Leucaena x mixtec*	EF643862
Eudicotyledons	Fabaceae	*Leucaena*	*Leucaena x mixtec*	EF643863
Eudicotyledons	Fabaceae	*Leucaena*	*Leucaena x mixtec*	EF643873
Eudicotyledons	Fabaceae	*Leucaena*	*Leucaena x mixtec*	EF643892
Eudicotyledons	Fabaceae	*Leucaena*	*Leucaena x spontanea*	EF643860
Eudicotyledons	Fabaceae	*Leucaena*	*Leucaena x spontanea*	EF643864
Eudicotyledons	Fabaceae	*Leucaena*	*Leucaena x spontanea*	EF643866
Eudicotyledons	Fabaceae	*Leucaena*	*Leucaena x spontanea*	EF643870
Eudicotyledons	Fabaceae	*Leucaena*	*Leucaena x spontanea*	EF643871
Eudicotyledons	Lamiaceae	*Origanum*	*Origanum onites*	HQ902834
Eudicotyledons	Lamiaceae	*Origanum*	*Origanum onites*	HQ902856
Eudicotyledons	Santalaceae	*Santalum*	*Santalum spicatum*	HQ170461
Eudicotyledons	Santalaceae	*Santalum*	*Santalum spicatum*	HQ170462
Eudicotyledons	Santalaceae	*Santalum*	*Santalum spicatum*	HQ170463
Eudicotyledons	Santalaceae	*Santalum*	*Santalum spicatum*	HQ170464
Eudicotyledons	Adoxaceae	*Viburnum*	*Viburnum awabuki*	HQ592060
Eudicotyledons	Adoxaceae	*Viburnum*	*Viburnum awabuki*	JN047523
Eudicotyledons	Adoxaceae	*Viburnum*	*Viburnum awabuki*	JN047524
Eudicotyledons	Adoxaceae	*Viburnum*	*Viburnum awabuki*	JN047525
Eudicotyledons	Asteraceae	*Inula*	*Inula japonica*	GU724261
Eudicotyledons	Asteraceae	*Inula*	*Inula japonica*	GU724262

Eudicotyledons	Fabaceae	*Acacia*	*Acacia nigrescens*	EU213784
Eudicotyledons	Fabaceae	*Acacia*	*Acacia nigrescens*	EU213785
Eudicotyledons	Solanaceae	*Capsicum*	*Capsicum campylopodium*	EF537235
Eudicotyledons	Solanaceae	*Capsicum*	*Capsicum campylopodium*	EF537236
Eudicotyledons	Solanaceae	*Capsicum*	*Capsicum hunzikerianum*	EF537244
Eudicotyledons	Solanaceae	*Capsicum*	*Capsicum hunzikerianum*	EF537245
Eudicotyledons	Solanaceae	*Capsicum*	*Capsicum parvifolium*	EF537217
Eudicotyledons	Solanaceae	*Capsicum*	*Capsicum parvifolium*	EF537218
Eudicotyledons	Solanaceae	*Capsicum*	*Capsicum parvifolium*	EF537219
Eudicotyledons	Solanaceae	*Capsicum*	*Capsicum recurvatum*	EF537240
Eudicotyledons	Solanaceae	*Capsicum*	*Capsicum recurvatum*	EF537241
Eudicotyledons	Solanaceae	*Capsicum*	*Capsicum recurvatum*	EF537242
Eudicotyledons	Solanaceae	*Capsicum*	*Capsicum recurvatum*	EF537243
Eudicotyledons	Solanaceae	*Capsicum*	*Capsicum rhomboideum*	EF537237
Eudicotyledons	Solanaceae	*Capsicum*	*Capsicum rhomboideum*	EF537238
Eudicotyledons	Solanaceae	*Capsicum*	*Capsicum rhomboideum*	EF537239
Eudicotyledons	Rubiaceae	*Oldenlandia*	*Oldenlandia consanguinea*	JF699782
Eudicotyledons	Rubiaceae	*Oldenlandia*	*Oldenlandia consanguinea*	JF699783
Eudicotyledons	Rubiaceae	*Oldenlandia*	*Oldenlandia consanguinea*	JN044769
Eudicotyledons	Rubiaceae	*Oldenlandia*	*Oldenlandia consanguinea*	JN044770
Eudicotyledons	Cucurbitaceae	*Bryonia*	*Bryonia acuta*	EU096433
Eudicotyledons	Cucurbitaceae	*Bryonia*	*Bryonia acuta*	EU096434
Eudicotyledons	Cucurbitaceae	*Bryonia*	*Bryonia aspera*	EU683740
Eudicotyledons	Cucurbitaceae	*Bryonia*	*Bryonia aspera*	EU096423
Eudicotyledons	Cucurbitaceae	*Bryonia*	*Bryonia aspera*	EU096424
Eudicotyledons	Cucurbitaceae	*Bryonia*	*Bryonia aspera*	EU096425
Eudicotyledons	Cucurbitaceae	*Bryonia*	*Bryonia aspera*	EU096426
Eudicotyledons	Cucurbitaceae	*Bryonia*	*Bryonia aspera*	EU096427
Eudicotyledons	Cucurbitaceae	*Bryonia*	*Bryonia aspera*	EU096428
Eudicotyledons	Cucurbitaceae	*Bryonia*	*Bryonia aspera*	EU096429
Eudicotyledons	Cucurbitaceae	*Bryonia*	*Bryonia aspera*	EU096430
Eudicotyledons	Cucurbitaceae	*Bryonia*	*Bryonia aspera*	EU096431
Eudicotyledons	Cucurbitaceae	*Bryonia*	*Bryonia aspera*	EU096432
Eudicotyledons	Cucurbitaceae	*Bryonia*	*Bryonia aspera*	EU096435
Eudicotyledons	Cucurbitaceae	*Bryonia*	*Bryonia aspera*	EU096437
Eudicotyledons	Cucurbitaceae	*Bryonia*	*Bryonia aspera*	EU096438
Eudicotyledons	Cucurbitaceae	*Bryonia*	*Bryonia aspera*	EU096439
Eudicotyledons	Cucurbitaceae	*Bryonia*	*Bryonia aspera*	EU096440
Eudicotyledons	Cucurbitaceae	*Bryonia*	*Bryonia aspera*	EU096441
Eudicotyledons	Cucurbitaceae	*Bryonia*	*Bryonia aspera*	EU096442
Eudicotyledons	Cucurbitaceae	*Bryonia*	*Bryonia marmorata*	EU096400
Eudicotyledons	Cucurbitaceae	*Bryonia*	*Bryonia marmorata*	EU096401
Eudicotyledons	Cucurbitaceae	*Bryonia*	*Bryonia monoica*	EU096407
Eudicotyledons	Cucurbitaceae	*Bryonia*	*Bryonia monoica*	EU096408
Eudicotyledons	Cucurbitaceae	*Bryonia*	*Bryonia monoica*	EU096409
Eudicotyledons	Cucurbitaceae	*Bryonia*	*Bryonia monoica*	EU096410
Eudicotyledons	Cucurbitaceae	*Bryonia*	*Bryonia monoica*	EU096411
Eudicotyledons	Cucurbitaceae	*Bryonia*	*Bryonia monoica*	EU096412
Eudicotyledons	Cucurbitaceae	*Bryonia*	*Bryonia monoica*	EU096413
Eudicotyledons	Cucurbitaceae	*Bryonia*	*Bryonia monoica*	EU096414
Eudicotyledons	Cucurbitaceae	*Bryonia*	*Bryonia monoica*	EU096415
Eudicotyledons	Cucurbitaceae	*Bryonia*	*Bryonia monoica*	EU096416
Eudicotyledons	Cucurbitaceae	*Bryonia*	*Bryonia monoica*	EU096417

Eudicotyledons	Cucurbitaceae	*Bryonia*	*Bryonia monoica*	EU096418
Eudicotyledons	Cucurbitaceae	*Bryonia*	*Bryonia monoica*	EU096419
Eudicotyledons	Cucurbitaceae	*Bryonia*	*Bryonia monoica*	EU096420
Eudicotyledons	Cucurbitaceae	*Bryonia*	*Bryonia monoica*	EU096421
Eudicotyledons	Cucurbitaceae	*Bryonia*	*Bryonia monoica*	EU096422
Eudicotyledons	Cucurbitaceae	*Bryonia*	*Bryonia multiflora*	EU096348
Eudicotyledons	Cucurbitaceae	*Bryonia*	*Bryonia multiflora*	EU096349
Eudicotyledons	Cucurbitaceae	*Bryonia*	*Bryonia syriaca*	EU096350
Eudicotyledons	Cucurbitaceae	*Bryonia*	*Bryonia syriaca*	EU096351
Eudicotyledons	Cucurbitaceae	*Bryonia*	*Bryonia syriaca*	EU096352
Eudicotyledons	Cucurbitaceae	*Bryonia*	*Bryonia syriaca*	EU096353
Eudicotyledons	Cucurbitaceae	*Bryonia*	*Bryonia syriaca*	EU096354
Eudicotyledons	Cucurbitaceae	*Bryonia*	*Bryonia syriaca*	EU096355
Eudicotyledons	Cucurbitaceae	*Bryonia*	*Bryonia syriaca*	EU096356
Eudicotyledons	Cucurbitaceae	*Bryonia*	*Bryonia verrucosa*	EU096443
Eudicotyledons	Cucurbitaceae	*Bryonia*	*Bryonia verrucosa*	EU096444
Eudicotyledons	Cucurbitaceae	*Bryonia*	*Bryonia verrucosa*	EU096445
Eudicotyledons	Salicaceae	*Salix*	*Salix phylicifolia*	GU373289
Eudicotyledons	Salicaceae	*Salix*	*Salix phylicifolia*	GU373290
Eudicotyledons	Salicaceae	*Salix*	*Salix aurita*	GU373275
Eudicotyledons	Salicaceae	*Salix*	*Salix aurita*	GU373276
Eudicotyledons	Salicaceae	*Salix*	*Salix aurita*	GU373277
Eudicotyledons	Salicaceae	*Salix*	*Salix lapponum*	GU373282
Eudicotyledons	Salicaceae	*Salix*	*Salix lapponum*	GU373283
Eudicotyledons	Salicaceae	*Salix*	*Salix lapponum*	GU373322
Eudicotyledons	Salicaceae	*Salix*	*Salix myrsinites*	GU373284
Eudicotyledons	Salicaceae	*Salix*	*Salix myrsinites*	GU373285
Eudicotyledons	Salicaceae	*Salix*	*Salix myrsinites*	GU373286
Eudicotyledons	Salicaceae	*Salix*	*Salix cinerea*	GU373278
Eudicotyledons	Salicaceae	*Salix*	*Salix cinerea*	GU373279
Eudicotyledons	Apiaceae	*Heracleum*	*Heracleum hemsleyanum*	GU967822
Eudicotyledons	Apiaceae	*Heracleum*	*Heracleum hemsleyanum*	GU967823
Eudicotyledons	Apiaceae	*Heracleum*	*Heracleum hemsleyanum*	GU967824
Eudicotyledons	Apiaceae	*Heracleum*	*Heracleum hemsleyanum*	GU967825
Eudicotyledons	Cucurbitaceae	*Trichosanthes*	*Trichosanthes villosa*	HQ829574
Eudicotyledons	Cucurbitaceae	*Trichosanthes*	*Trichosanthes villosa*	HQ829575
Eudicotyledons	Cucurbitaceae	*Trichosanthes*	*Trichosanthes villosa*	HQ829576
Eudicotyledons	Ericaceae	*Rhododendron*	*Rhododendron aganniphum*	JN046733
Eudicotyledons	Ericaceae	*Rhododendron*	*Rhododendron aganniphum*	JN046734
Eudicotyledons	Ericaceae	*Rhododendron*	*Rhododendron aganniphum*	HQ706948
Eudicotyledons	Ericaceae	*Rhododendron*	*Rhododendron alutaceum*	JN046737
Eudicotyledons	Ericaceae	*Rhododendron*	*Rhododendron alutaceum*	JN046738
Eudicotyledons	Ericaceae	*Rhododendron*	*Rhododendron beesianum*	JN046758
Eudicotyledons	Ericaceae	*Rhododendron*	*Rhododendron beesianum*	JN046759
Eudicotyledons	Ericaceae	*Rhododendron*	*Rhododendron beesianum*	JN046760
Eudicotyledons	Ericaceae	*Rhododendron*	*Rhododendron beesianum*	JN046761
Eudicotyledons	Ericaceae	*Rhododendron*	*Rhododendron beesianum*	JN046762
Eudicotyledons	Ericaceae	*Rhododendron*	*Rhododendron bureavii*	JN046763
Eudicotyledons	Ericaceae	*Rhododendron*	*Rhododendron bureavii*	JN046764
Eudicotyledons	Ericaceae	*Rhododendron*	*Rhododendron bureavii*	JN046765
Eudicotyledons	Ericaceae	*Rhododendron*	*Rhododendron campylocarpum*	JN046770
Eudicotyledons	Ericaceae	*Rhododendron*	*Rhododendron campylocarpum*	JN046771
Eudicotyledons	Ericaceae	*Rhododendron*	*Rhododendron campylocarpum*	JN046772

Eudicotyledons	Ericaceae	*Rhododendron*	*Rhododendron campylocarpum*	JN046773
Eudicotyledons	Ericaceae	*Rhododendron*	*Rhododendron campylocarpum*	JN046774
Eudicotyledons	Ericaceae	*Rhododendron*	*Rhododendron floccigerum*	JN046814
Eudicotyledons	Ericaceae	*Rhododendron*	*Rhododendron floccigerum*	JN046815
Eudicotyledons	Ericaceae	*Rhododendron*	*Rhododendron floccigerum*	JN046816
Eudicotyledons	Ericaceae	*Rhododendron*	*Rhododendron floccigerum*	JN046817
Eudicotyledons	Ericaceae	*Rhododendron*	*Rhododendron fulvum*	JN046822
Eudicotyledons	Ericaceae	*Rhododendron*	*Rhododendron fulvum*	JN046823
Eudicotyledons	Ericaceae	*Rhododendron*	*Rhododendron glischrum*	JN046828
Eudicotyledons	Ericaceae	*Rhododendron*	*Rhododendron glischrum*	JN046829
Eudicotyledons	Ericaceae	*Rhododendron*	*Rhododendron selense*	JN046955
Eudicotyledons	Ericaceae	*Rhododendron*	*Rhododendron selense*	JN046956
Eudicotyledons	Ericaceae	*Rhododendron*	*Rhododendron selense*	JN046957
Eudicotyledons	Ericaceae	*Rhododendron*	*Rhododendron selense*	JN046958
Eudicotyledons	Ericaceae	*Rhododendron*	*Rhododendron vernicosum*	JN047010
Eudicotyledons	Ericaceae	*Rhododendron*	*Rhododendron vernicosum*	JN047011
Eudicotyledons	Ericaceae	*Rhododendron*	*Rhododendron vernicosum*	HQ707029
Eudicotyledons	Ericaceae	*Rhododendron*	*Rhododendron vernicosum*	HQ707030
Eudicotyledons	Salicaceae	*Populus*	*Populus grandidentata*	EU750500
Eudicotyledons	Salicaceae	*Populus*	*Populus grandidentata*	EU750501
Eudicotyledons	Salicaceae	*Populus*	*Populus grandidentata*	EU750502
Eudicotyledons	Solanaceae	*Solanum*	*Solanum lichtensteinii*	HM016421
Eudicotyledons	Solanaceae	*Solanum*	*Solanum lichtensteinii*	HM016426
Eudicotyledons	Oleaceae	*Fraxinus*	*Fraxinus apertisquamifera*	HM367381
Eudicotyledons	Oleaceae	*Fraxinus*	*Fraxinus apertisquamifera*	HM367382
Eudicotyledons	Oleaceae	*Fraxinus*	*Fraxinus apertisquamifera*	HM367383
Eudicotyledons	Oleaceae	*Fraxinus*	*Fraxinus apertisquamifera*	HM367384
Eudicotyledons	Oleaceae	*Fraxinus*	*Fraxinus apertisquamifera*	HM367385
Eudicotyledons	Oleaceae	*Fraxinus*	*Fraxinus apertisquamifera*	HM367386
Eudicotyledons	Oleaceae	*Fraxinus*	*Fraxinus apertisquamifera*	HM367387
Eudicotyledons	Oleaceae	*Fraxinus*	*Fraxinus apertisquamifera*	HM367388
Eudicotyledons	Oleaceae	*Fraxinus*	*Fraxinus berlandieriana*	HM367389
Eudicotyledons	Oleaceae	*Fraxinus*	*Fraxinus berlandieriana*	HM367390
Eudicotyledons	Oleaceae	*Fraxinus*	*Fraxinus berlandieriana*	HM367391
Eudicotyledons	Oleaceae	*Fraxinus*	*Fraxinus bungeana*	HM367394
Eudicotyledons	Oleaceae	*Fraxinus*	*Fraxinus bungeana*	HM367395
Eudicotyledons	Oleaceae	*Fraxinus*	*Fraxinus bungeana*	HM367396
Eudicotyledons	Oleaceae	*Fraxinus*	*Fraxinus caroliniana*	HM367397
Eudicotyledons	Oleaceae	*Fraxinus*	*Fraxinus caroliniana*	HM367398
Eudicotyledons	Oleaceae	*Fraxinus*	*Fraxinus chiisanensis*	HM367399
Eudicotyledons	Oleaceae	*Fraxinus*	*Fraxinus chiisanensis*	HM367400
Eudicotyledons	Oleaceae	*Fraxinus*	*Fraxinus chiisanensis*	HM367401
Eudicotyledons	Oleaceae	*Fraxinus*	*Fraxinus floribunda*	HM367424
Eudicotyledons	Oleaceae	*Fraxinus*	*Fraxinus floribunda*	HM367425
Eudicotyledons	Oleaceae	*Fraxinus*	*Fraxinus lanuginosa*	HM367443
Eudicotyledons	Oleaceae	*Fraxinus*	*Fraxinus lanuginosa*	HM367444
Eudicotyledons	Oleaceae	*Fraxinus*	*Fraxinus lanuginosa*	HM367445
Eudicotyledons	Oleaceae	*Fraxinus*	*Fraxinus lanuginosa*	HM367446
Eudicotyledons	Oleaceae	*Fraxinus*	*Fraxinus lanuginosa*	HM367447
Eudicotyledons	Oleaceae	*Fraxinus*	*Fraxinus lanuginosa*	HM367448
Eudicotyledons	Oleaceae	*Fraxinus*	*Fraxinus lanuginosa*	HM367449
Eudicotyledons	Oleaceae	*Fraxinus*	*Fraxinus lanuginosa*	HM367450
Eudicotyledons	Oleaceae	*Fraxinus*	*Fraxinus lanuginosa*	HM367451

Eudicotyledons	Oleaceae	*Fraxinus*	*Fraxinus lanuginosa*	HM367452
Eudicotyledons	Oleaceae	*Fraxinus*	*Fraxinus lanuginosa*	HM367453
Eudicotyledons	Oleaceae	*Fraxinus*	*Fraxinus lanuginosa*	HM367454
Eudicotyledons	Oleaceae	*Fraxinus*	*Fraxinus lanuginosa*	HM367455
Eudicotyledons	Oleaceae	*Fraxinus*	*Fraxinus lanuginosa*	HM367456
Eudicotyledons	Oleaceae	*Fraxinus*	*Fraxinus lanuginosa*	HM367457
Eudicotyledons	Oleaceae	*Fraxinus*	*Fraxinus lanuginosa*	HM367458
Eudicotyledons	Oleaceae	*Fraxinus*	*Fraxinus lanuginosa*	HM367460
Eudicotyledons	Oleaceae	*Fraxinus*	*Fraxinus lanuginosa*	HM367461
Eudicotyledons	Oleaceae	*Fraxinus*	*Fraxinus lanuginosa*	HM367462
Eudicotyledons	Oleaceae	*Fraxinus*	*Fraxinus lanuginosa*	HM367463
Eudicotyledons	Oleaceae	*Fraxinus*	*Fraxinus lanuginosa*	HM367464
Eudicotyledons	Oleaceae	*Fraxinus*	*Fraxinus lanuginosa*	HM367465
Eudicotyledons	Oleaceae	*Fraxinus*	*Fraxinus lanuginosa*	HM367466
Eudicotyledons	Oleaceae	*Fraxinus*	*Fraxinus paxiana*	HM367508
Eudicotyledons	Oleaceae	*Fraxinus*	*Fraxinus paxiana*	HM367509
Eudicotyledons	Oleaceae	*Fraxinus*	*Fraxinus paxiana*	HM367510
Eudicotyledons	Oleaceae	*Fraxinus*	*Fraxinus sieboldiana*	HM367537
Eudicotyledons	Oleaceae	*Fraxinus*	*Fraxinus sieboldiana*	HM367538
Eudicotyledons	Oleaceae	*Fraxinus*	*Fraxinus sieboldiana*	HM367539
Eudicotyledons	Oleaceae	*Fraxinus*	*Fraxinus sieboldiana*	HM367540
Eudicotyledons	Oleaceae	*Fraxinus*	*Fraxinus sieboldiana*	HM367541
Eudicotyledons	Oleaceae	*Fraxinus*	*Fraxinus sieboldiana*	HM367542
Eudicotyledons	Oleaceae	*Fraxinus*	*Fraxinus sieboldiana*	HM367543
Eudicotyledons	Oleaceae	*Fraxinus*	*Fraxinus sieboldiana*	HM367544
Eudicotyledons	Oleaceae	*Fraxinus*	*Fraxinus sieboldiana*	HM367545
Eudicotyledons	Oleaceae	*Fraxinus*	*Fraxinus sieboldiana*	HM367546
Eudicotyledons	Oleaceae	*Fraxinus*	*Fraxinus sieboldiana*	HM367547
Eudicotyledons	Oleaceae	*Fraxinus*	*Fraxinus spaethiana*	HM367554
Eudicotyledons	Oleaceae	*Fraxinus*	*Fraxinus spaethiana*	HM367555
Eudicotyledons	Oleaceae	*Fraxinus*	*Fraxinus spaethiana*	HM367556
Eudicotyledons	Oleaceae	*Fraxinus*	*Fraxinus spaethiana*	HM367557
Eudicotyledons	Oleaceae	*Fraxinus*	*Fraxinus uhdei*	HM367567
Eudicotyledons	Oleaceae	*Fraxinus*	*Fraxinus uhdei*	HM367568
Eudicotyledons	Oleaceae	*Fraxinus*	*Fraxinus uhdei*	HM367569
Eudicotyledons	Oleaceae	*Fraxinus*	*Fraxinus uhdei*	HM367570
Eudicotyledons	Apiaceae	*Heracleum*	*Heracleum franchetii*	GU967818
Eudicotyledons	Apiaceae	*Heracleum*	*Heracleum franchetii*	GU967819
Eudicotyledons	Apiaceae	*Heracleum*	*Heracleum franchetii*	GU967820
Eudicotyledons	Apiaceae	*Heracleum*	*Heracleum franchetii*	GU967821
Eudicotyledons	Fabaceae	*Acacia*	*Acacia exuvialis*	EU213781
Eudicotyledons	Fabaceae	*Acacia*	*Acacia exuvialis*	EU213782
Eudicotyledons	Fabaceae	*Acacia*	*Acacia exuvialis*	EU213783
Eudicotyledons	Fabaceae	*Acacia*	*Acacia exuvialis*	GQ872312
Eudicotyledons	Aquifoliaceae	*Ilex*	*Ilex ficoidea*	HQ415419
Eudicotyledons	Aquifoliaceae	*Ilex*	*Ilex ficoidea*	HQ426979
Eudicotyledons	Vitaceae	*Vitis*	*Vitis acerifolia*	HQ656432
Eudicotyledons	Vitaceae	*Vitis*	*Vitis acerifolia*	HQ656433
Eudicotyledons	Asteraceae	*Carlina*	*Carlina brachylepis*	EU531703
Eudicotyledons	Asteraceae	*Carlina*	*Carlina brachylepis*	EU571413
Eudicotyledons	Asteraceae	*Carlina*	*Carlina macrophylla*	EU531704
Eudicotyledons	Asteraceae	*Carlina*	*Carlina macrophylla*	EU571448
Eudicotyledons	Ericaceae	*Rhododendron*	*Rhododendron hirsutum*	HE585252

Eudicotyledons	Ericaceae	*Rhododendron*	*Rhododendron hirsutum*	HE585253
Eudicotyledons	Lamiaceae	*Scutellaria*	*Scutellaria rehderiana*	GQ374155
Eudicotyledons	Lamiaceae	*Scutellaria*	*Scutellaria rehderiana*	FJ528994
Eudicotyledons	Lamiaceae	*Scutellaria*	*Scutellaria rehderiana*	HQ680367
Eudicotyledons	Lamiaceae	*Scutellaria*	*Scutellaria rehderiana*	HQ680368
Eudicotyledons	Fabaceae	*Wisteria*	*Wisteria villosa*	EU424105
Eudicotyledons	Fabaceae	*Wisteria*	*Wisteria villosa*	EU424106
Eudicotyledons	Fabaceae	*Wisteria*	*Wisteria villosa*	EU424107
Eudicotyledons	Asteraceae	*Cirsium*	*Cirsium japonicum*	GU724249
Eudicotyledons	Asteraceae	*Cirsium*	*Cirsium japonicum*	GQ435079
Eudicotyledons	Asteraceae	*Cirsium*	*Cirsium japonicum*	GQ435080
Eudicotyledons	Asteraceae	*Cirsium*	*Cirsium japonicum*	GQ435081
Eudicotyledons	Asteraceae	*Doniophyton*	*Doniophyton weddellii*	EU841294
Eudicotyledons	Asteraceae	*Doniophyton*	*Doniophyton weddellii*	EU841295
Eudicotyledons	Salicaceae	*Salix*	*Salix x rubens*	GU373310
Eudicotyledons	Salicaceae	*Salix*	*Salix x rubens*	GU373311
Eudicotyledons	Salicaceae	*Salix*	*Salix x rubens*	GU373312
Eudicotyledons	Oxalidaceae	*Oxalis*	*Oxalis dines*	JN983509
Eudicotyledons	Oxalidaceae	*Oxalis*	*Oxalis dines*	JN983518
Eudicotyledons	Oxalidaceae	*Oxalis*	*Oxalis dines*	JN983519
Eudicotyledons	Oxalidaceae	*Oxalis*	*Oxalis dines*	JN983520
Eudicotyledons	Oxalidaceae	*Oxalis*	*Oxalis disticha*	JN983510
Eudicotyledons	Oxalidaceae	*Oxalis*	*Oxalis disticha*	JN983511
Eudicotyledons	Oxalidaceae	*Oxalis*	*Oxalis disticha*	JN983512
Eudicotyledons	Oxalidaceae	*Oxalis*	*Oxalis disticha*	JN983513
Eudicotyledons	Oxalidaceae	*Oxalis*	*Oxalis disticha*	JN983514
Eudicotyledons	Oxalidaceae	*Oxalis*	*Oxalis disticha*	JN983515
Eudicotyledons	Oxalidaceae	*Oxalis*	*Oxalis disticha*	JN983516
Eudicotyledons	Oxalidaceae	*Oxalis*	*Oxalis disticha*	JN983517
Eudicotyledons	Cucurbitaceae	*Momordica*	*Momordica angustisepala*	GQ162969
Eudicotyledons	Cucurbitaceae	*Momordica*	*Momordica angustisepala*	GQ162970
Eudicotyledons	Cucurbitaceae	*Momordica*	*Momordica angustisepala*	GQ162971
Eudicotyledons	Cucurbitaceae	*Momordica*	*Momordica cissoides*	GQ162999
Eudicotyledons	Cucurbitaceae	*Momordica*	*Momordica cissoides*	GQ163000
Eudicotyledons	Cucurbitaceae	*Momordica*	*Momordica cissoides*	GQ163001
Eudicotyledons	Fabaceae	*Acacia*	*Acacia grandicornuta*	GQ872316
Eudicotyledons	Fabaceae	*Acacia*	*Acacia grandicornuta*	EU811985
Eudicotyledons	Asteraceae	*Lactuca*	*Lactuca canadensis*	GU818401
Eudicotyledons	Asteraceae	*Lactuca*	*Lactuca canadensis*	EU750467
Eudicotyledons	Asteraceae	*Lactuca*	*Lactuca canadensis*	EU750468
Eudicotyledons	Rhamnaceae	*Rhamnus*	*Rhamnus alnifolia*	EU750516
Eudicotyledons	Rhamnaceae	*Rhamnus*	*Rhamnus alnifolia*	EU750517
Eudicotyledons	Rosaceae	*Fragaria*	*Fragaria corymbosa*	GQ476759
Eudicotyledons	Rosaceae	*Fragaria*	*Fragaria corymbosa*	GQ476760
Eudicotyledons	Plantaginaceae	*Antirrhinum*	*Antirrhinum controversum*	HM152891
Eudicotyledons	Plantaginaceae	*Antirrhinum*	*Antirrhinum controversum*	HM152892
Eudicotyledons	Plantaginaceae	*Antirrhinum*	*Antirrhinum controversum*	HM152893
Eudicotyledons	Plantaginaceae	*Antirrhinum*	*Antirrhinum controversum*	HM152894
Eudicotyledons	Plantaginaceae	*Antirrhinum*	*Antirrhinum controversum*	HM152895
Eudicotyledons	Plantaginaceae	*Antirrhinum*	*Antirrhinum controversum*	HM152896
Eudicotyledons	Plantaginaceae	*Antirrhinum*	*Antirrhinum controversum*	HM152897
Eudicotyledons	Plantaginaceae	*Antirrhinum*	*Antirrhinum controversum*	HM152898
Eudicotyledons	Araliaceae	*Hydrocotyle*	*Hydrocotyle ranunculoides*	FM207049

Eudicotyledons	Araliaceae	*Hydrocotyle*	*Hydrocotyle ranunculoides*	FM207050
Eudicotyledons	Araliaceae	*Hydrocotyle*	*Hydrocotyle ranunculoides*	FM207051
Eudicotyledons	Araliaceae	*Hydrocotyle*	*Hydrocotyle ranunculoides*	FM207052
Eudicotyledons	Araliaceae	*Hydrocotyle*	*Hydrocotyle ranunculoides*	FM207053
Eudicotyledons	Araliaceae	*Hydrocotyle*	*Hydrocotyle ranunculoides*	FM207054
Eudicotyledons	Araliaceae	*Hydrocotyle*	*Hydrocotyle ranunculoides*	FM207055
Eudicotyledons	Araliaceae	*Hydrocotyle*	*Hydrocotyle ranunculoides*	FM207056
Eudicotyledons	Araliaceae	*Hydrocotyle*	*Hydrocotyle ranunculoides*	FM207057
Eudicotyledons	Araliaceae	*Hydrocotyle*	*Hydrocotyle ranunculoides*	FM207058
Eudicotyledons	Araliaceae	*Hydrocotyle*	*Hydrocotyle ranunculoides*	FM207059
Eudicotyledons	Araliaceae	*Hydrocotyle*	*Hydrocotyle ranunculoides*	FM207060
Eudicotyledons	Araliaceae	*Hydrocotyle*	*Hydrocotyle ranunculoides*	FM207061
Eudicotyledons	Araliaceae	*Hydrocotyle*	*Hydrocotyle leucocephala*	FM207062
Eudicotyledons	Araliaceae	*Hydrocotyle*	*Hydrocotyle leucocephala*	FM207063
Eudicotyledons	Araliaceae	*Hydrocotyle*	*Hydrocotyle leucocephala*	FM207064
Eudicotyledons	Araliaceae	*Hydrocotyle*	*Hydrocotyle leucocephala*	FM207065
Eudicotyledons	Araliaceae	*Hydrocotyle*	*Hydrocotyle leucocephala*	FM207066
Eudicotyledons	Geraniaceae	*Geranium*	*Geranium carolinianum*	JN044734
Eudicotyledons	Geraniaceae	*Geranium*	*Geranium carolinianum*	JN044735
Eudicotyledons	Geraniaceae	*Geranium*	*Geranium carolinianum*	JN044736
Eudicotyledons	Cornaceae	*Nyssa*	*Nyssa javanica*	EU734467
Eudicotyledons	Cornaceae	*Nyssa*	*Nyssa javanica*	EU734468
Eudicotyledons	Cornaceae	*Nyssa*	*Nyssa shangszeensis*	EU734457
Eudicotyledons	Cornaceae	*Nyssa*	*Nyssa shangszeensis*	EU734458
Eudicotyledons	Cornaceae	*Nyssa*	*Nyssa shangszeensis*	JN045587
Eudicotyledons	Cornaceae	*Nyssa*	*Nyssa shangszeensis*	JN045588
Eudicotyledons	Cornaceae	*Nyssa*	*Nyssa shweliensis*	EU734465
Eudicotyledons	Cornaceae	*Nyssa*	*Nyssa shweliensis*	EU734466
Eudicotyledons	Cornaceae	*Nyssa*	*Nyssa shweliensis*	JN045589
Eudicotyledons	Cornaceae	*Nyssa*	*Nyssa shweliensis*	JN045590
Eudicotyledons	Cornaceae	*Nyssa*	*Nyssa shweliensis*	JN045591
Eudicotyledons	Cornaceae	*Nyssa*	*Nyssa sinensis*	HQ427019
Eudicotyledons	Cornaceae	*Nyssa*	*Nyssa sinensis*	EU734459
Eudicotyledons	Cornaceae	*Nyssa*	*Nyssa sinensis*	EU734460
Eudicotyledons	Cornaceae	*Nyssa*	*Nyssa sinensis*	EU734461
Eudicotyledons	Cornaceae	*Nyssa*	*Nyssa sinensis*	EU734462
Eudicotyledons	Cornaceae	*Nyssa*	*Nyssa sinensis*	JN045592
Eudicotyledons	Cornaceae	*Nyssa*	*Nyssa sinensis*	JN045593
Eudicotyledons	Cornaceae	*Nyssa*	*Nyssa sinensis*	JF321237
Eudicotyledons	Cornaceae	*Nyssa*	*Nyssa wenshanensis*	EU734463
Eudicotyledons	Cornaceae	*Nyssa*	*Nyssa wenshanensis*	EU734464
Eudicotyledons	Cornaceae	*Nyssa*	*Nyssa wenshanensis*	JN045597
Eudicotyledons	Cornaceae	*Nyssa*	*Nyssa wenshanensis*	JN045598
Eudicotyledons	Asteraceae	*Arctotis*	*Arctotis debensis*	EU846406
Eudicotyledons	Asteraceae	*Arctotis*	*Arctotis debensis*	EU846407
Eudicotyledons	Asteraceae	*Arctotis*	*Arctotis erosa*	EU846427
Eudicotyledons	Asteraceae	*Arctotis*	*Arctotis erosa*	EU846428
Eudicotyledons	Asteraceae	*Arctotis*	*Arctotis erosa*	EU846429
Eudicotyledons	Asteraceae	*Arctotis*	*Arctotis erosa*	EU846430
Eudicotyledons	Asteraceae	*Arctotis*	*Arctotis flaccida*	EU846432
Eudicotyledons	Asteraceae	*Arctotis*	*Arctotis flaccida*	EU846433
Eudicotyledons	Asteraceae	*Arctotis*	*Arctotis incisa*	EU846436
Eudicotyledons	Asteraceae	*Arctotis*	*Arctotis incisa*	EU846437

Eudicotyledons	Asteraceae	*Arctotis*	*Arctotis lanceolata*	EU846439
Eudicotyledons	Asteraceae	*Arctotis*	*Arctotis lanceolata*	EU846440
Eudicotyledons	Asteraceae	*Arctotis*	*Arctotis microcephala*	EU846409
Eudicotyledons	Asteraceae	*Arctotis*	*Arctotis microcephala*	EU846410
Eudicotyledons	Asteraceae	*Arctotis*	*Arctotis revoluta*	EU846446
Eudicotyledons	Asteraceae	*Arctotis*	*Arctotis revoluta*	EU846447
Eudicotyledons	Asteraceae	*Arctotis*	*Arctotis scapiformis*	EU846412
Eudicotyledons	Asteraceae	*Arctotis*	*Arctotis scapiformis*	EU846413
Eudicotyledons	Asteraceae	*Arctotis*	*Arctotis semipapposa*	EU846449
Eudicotyledons	Asteraceae	*Arctotis*	*Arctotis semipapposa*	EU846450
Eudicotyledons	Asteraceae	*Arctotis*	*Arctotis verbascifolia*	EU846453
Eudicotyledons	Asteraceae	*Arctotis*	*Arctotis verbascifolia*	EU846454
Eudicotyledons	Asteraceae	*Arctotis*	*Arctotis sp. C RJM-2008*	EU846458
Eudicotyledons	Asteraceae	*Arctotis*	*Arctotis sp. C RJM-2008*	EU846459
Eudicotyledons	Asteraceae	*Cirsium*	*Cirsium setosum*	GQ435076
Eudicotyledons	Asteraceae	*Cirsium*	*Cirsium setosum*	GQ435077
Eudicotyledons	Asteraceae	*Cirsium*	*Cirsium setosum*	GQ435078
Eudicotyledons	Lamiaceae	*Leonurus*	*Leonurus cardiaca*	HQ596745
Eudicotyledons	Lamiaceae	*Leonurus*	*Leonurus cardiaca*	FJ513116
Eudicotyledons	Asteraceae	*Lychnophora*	*Lychnophora ericoides*	FJ031669
Eudicotyledons	Asteraceae	*Lychnophora*	*Lychnophora ericoides*	FJ031670
Eudicotyledons	Asteraceae	*Lychnophora*	*Lychnophora ericoides*	FJ031671
Eudicotyledons	Asteraceae	*Lychnophora*	*Lychnophora ericoides*	FJ031672
Eudicotyledons	Asteraceae	*Lychnophora*	*Lychnophora ericoides*	FJ031673
Eudicotyledons	Asteraceae	*Lychnophora*	*Lychnophora ericoides*	FJ031674
Eudicotyledons	Asteraceae	*Lychnophora*	*Lychnophora ericoides*	FJ031675
Eudicotyledons	Asteraceae	*Lychnophora*	*Lychnophora ericoides*	FJ031676
Eudicotyledons	Asteraceae	*Lychnophora*	*Lychnophora ericoides*	FJ031677
Eudicotyledons	Asteraceae	*Lychnophora*	*Lychnophora ericoides*	FJ031678
Eudicotyledons	Asteraceae	*Lychnophora*	*Lychnophora ericoides*	FJ031679
Eudicotyledons	Asteraceae	*Lychnophora*	*Lychnophora ericoides*	FJ031680
Eudicotyledons	Asteraceae	*Lychnophora*	*Lychnophora ericoides*	FJ031681
Eudicotyledons	Asteraceae	*Lychnophora*	*Lychnophora ericoides*	FJ031682
Eudicotyledons	Asteraceae	*Lychnophora*	*Lychnophora ericoides*	FJ031683
Eudicotyledons	Asteraceae	*Lychnophora*	*Lychnophora ericoides*	FJ031684
Eudicotyledons	Asteraceae	*Lychnophora*	*Lychnophora ericoides*	FJ031685
Eudicotyledons	Asteraceae	*Lychnophora*	*Lychnophora ericoides*	FJ031686
Eudicotyledons	Asteraceae	*Lychnophora*	*Lychnophora ericoides*	FJ031687
Eudicotyledons	Asteraceae	*Lychnophora*	*Lychnophora ericoides*	FJ031688
Eudicotyledons	Asteraceae	*Lychnophora*	*Lychnophora ericoides*	FJ031689
Eudicotyledons	Asteraceae	*Lychnophora*	*Lychnophora ericoides*	FJ031690
Eudicotyledons	Asteraceae	*Lychnophora*	*Lychnophora ericoides*	FJ031691
Eudicotyledons	Asteraceae	*Lychnophora*	*Lychnophora ericoides*	FJ031692
Eudicotyledons	Asteraceae	*Lychnophora*	*Lychnophora ericoides*	FJ031693
Eudicotyledons	Asteraceae	*Lychnophora*	*Lychnophora ericoides*	FJ031694
Eudicotyledons	Asteraceae	*Lychnophora*	*Lychnophora ericoides*	FJ031695
Eudicotyledons	Asteraceae	*Lychnophora*	*Lychnophora ericoides*	FJ031696
Eudicotyledons	Asteraceae	*Lychnophora*	*Lychnophora ericoides*	FJ031697
Eudicotyledons	Asteraceae	*Lychnophora*	*Lychnophora ericoides*	FJ031698
Eudicotyledons	Asteraceae	*Lychnophora*	*Lychnophora ericoides*	FJ031699
Eudicotyledons	Asteraceae	*Lychnophora*	*Lychnophora ericoides*	FJ031700
Eudicotyledons	Asteraceae	*Lychnophora*	*Lychnophora ericoides*	FJ031701
Eudicotyledons	Asteraceae	*Lychnophora*	*Lychnophora ericoides*	FJ031702

Eudicotyledons	Asteraceae	*Lychnophora*	*Lychnophora ericoides*	FJ031703
Eudicotyledons	Asteraceae	*Lychnophora*	*Lychnophora ericoides*	FJ031704
Eudicotyledons	Asteraceae	*Lychnophora*	*Lychnophora ericoides*	FJ031705
Eudicotyledons	Asteraceae	*Lychnophora*	*Lychnophora ericoides*	FJ031706
Eudicotyledons	Asteraceae	*Lychnophora*	*Lychnophora ericoides*	FJ031707
Eudicotyledons	Asteraceae	*Lychnophora*	*Lychnophora ericoides*	FJ031708
Eudicotyledons	Asteraceae	*Lychnophora*	*Lychnophora ericoides*	FJ031709
Eudicotyledons	Asteraceae	*Lychnophora*	*Lychnophora ericoides*	FJ031710
Eudicotyledons	Asteraceae	*Lychnophora*	*Lychnophora ericoides*	FJ031711
Eudicotyledons	Asteraceae	*Lychnophora*	*Lychnophora ericoides*	FJ031712
Eudicotyledons	Asteraceae	*Lychnophora*	*Lychnophora ericoides*	FJ031713
Eudicotyledons	Asteraceae	*Lychnophora*	*Lychnophora ericoides*	FJ031714
Eudicotyledons	Asteraceae	*Lychnophora*	*Lychnophora ericoides*	FJ031715
Eudicotyledons	Asteraceae	*Lychnophora*	*Lychnophora ericoides*	FJ031716
Eudicotyledons	Asteraceae	*Lychnophora*	*Lychnophora ericoides*	FJ031717
Eudicotyledons	Asteraceae	*Lychnophora*	*Lychnophora ericoides*	FJ031718
Eudicotyledons	Asteraceae	*Lychnophora*	*Lychnophora ericoides*	FJ031719
Eudicotyledons	Asteraceae	*Lychnophora*	*Lychnophora ericoides*	FJ031720
Eudicotyledons	Asteraceae	*Lychnophora*	*Lychnophora ericoides*	FJ031721
Eudicotyledons	Asteraceae	*Lychnophora*	*Lychnophora ericoides*	FJ031722
Eudicotyledons	Asteraceae	*Lychnophora*	*Lychnophora ericoides*	FJ031723
Eudicotyledons	Asteraceae	*Lychnophora*	*Lychnophora ericoides*	FJ031724
Eudicotyledons	Asteraceae	*Lychnophora*	*Lychnophora ericoides*	FJ031725
Eudicotyledons	Asteraceae	*Lychnophora*	*Lychnophora ericoides*	FJ031726
Eudicotyledons	Asteraceae	*Lychnophora*	*Lychnophora ericoides*	FJ031727
Eudicotyledons	Asteraceae	*Lychnophora*	*Lychnophora ericoides*	FJ031728
Eudicotyledons	Asteraceae	*Lychnophora*	*Lychnophora ericoides*	FJ031729
Eudicotyledons	Asteraceae	*Lychnophora*	*Lychnophora ericoides*	FJ031730
Eudicotyledons	Asteraceae	*Lychnophora*	*Lychnophora ericoides*	FJ031731
Eudicotyledons	Asteraceae	*Lychnophora*	*Lychnophora ericoides*	FJ031732
Eudicotyledons	Asteraceae	*Lychnophora*	*Lychnophora ericoides*	FJ031733
Eudicotyledons	Asteraceae	*Lychnophora*	*Lychnophora ericoides*	FJ031734
Eudicotyledons	Asteraceae	*Lychnophora*	*Lychnophora ericoides*	FJ031735
Eudicotyledons	Asteraceae	*Lychnophora*	*Lychnophora ericoides*	FJ031736
Eudicotyledons	Asteraceae	*Lychnophora*	*Lychnophora ericoides*	FJ031737
Eudicotyledons	Asteraceae	*Lychnophora*	*Lychnophora ericoides*	FJ031738
Eudicotyledons	Asteraceae	*Lychnophora*	*Lychnophora ericoides*	FJ031739
Eudicotyledons	Asteraceae	*Lychnophora*	*Lychnophora ericoides*	FJ031740
Eudicotyledons	Asteraceae	*Lychnophora*	*Lychnophora ericoides*	FJ031741
Eudicotyledons	Asteraceae	*Lychnophora*	*Lychnophora ericoides*	FJ031742
Eudicotyledons	Asteraceae	*Lychnophora*	*Lychnophora ericoides*	FJ031743
Eudicotyledons	Asteraceae	*Lychnophora*	*Lychnophora ericoides*	FJ031744
Eudicotyledons	Asteraceae	*Lychnophora*	*Lychnophora ericoides*	FJ031745
Eudicotyledons	Asteraceae	*Lychnophora*	*Lychnophora ericoides*	FJ031746
Eudicotyledons	Asteraceae	*Lychnophora*	*Lychnophora ericoides*	FJ031747
Eudicotyledons	Asteraceae	*Lychnophora*	*Lychnophora ericoides*	FJ031748
Eudicotyledons	Asteraceae	*Lychnophora*	*Lychnophora ericoides*	FJ031749
Eudicotyledons	Asteraceae	*Lychnophora*	*Lychnophora ericoides*	FJ031750
Eudicotyledons	Asteraceae	*Lychnophora*	*Lychnophora ericoides*	FJ031751
Eudicotyledons	Asteraceae	*Lychnophora*	*Lychnophora ericoides*	FJ031752
Eudicotyledons	Asteraceae	*Lychnophora*	*Lychnophora ericoides*	FJ031753
Eudicotyledons	Asteraceae	*Lychnophora*	*Lychnophora ericoides*	FJ031754
Eudicotyledons	Asteraceae	*Lychnophora*	*Lychnophora ericoides*	FJ031755

Eudicotyledons	Asteraceae	*Lychnophora*	*Lychnophora ericoides*	FJ031756
Eudicotyledons	Asteraceae	*Lychnophora*	*Lychnophora ericoides*	FJ031757
Eudicotyledons	Asteraceae	*Lychnophora*	*Lychnophora ericoides*	FJ031758
Eudicotyledons	Asteraceae	*Lychnophora*	*Lychnophora ericoides*	FJ031759
Eudicotyledons	Asteraceae	*Lychnophora*	*Lychnophora ericoides*	FJ031760
Eudicotyledons	Asteraceae	*Lychnophora*	*Lychnophora ericoides*	FJ031761
Eudicotyledons	Asteraceae	*Lychnophora*	*Lychnophora ericoides*	FJ031762
Eudicotyledons	Asteraceae	*Lychnophora*	*Lychnophora ericoides*	FJ031763
Eudicotyledons	Asteraceae	*Lychnophora*	*Lychnophora ericoides*	FJ031764
Eudicotyledons	Asteraceae	*Lychnophora*	*Lychnophora ericoides*	FJ031765
Eudicotyledons	Asteraceae	*Lychnophora*	*Lychnophora ericoides*	FJ031766
Eudicotyledons	Asteraceae	*Lychnophora*	*Lychnophora ericoides*	FJ031767
Eudicotyledons	Asteraceae	*Lychnophora*	*Lychnophora ericoides*	FJ031768
Eudicotyledons	Asteraceae	*Lychnophora*	*Lychnophora ericoides*	FJ031769
Eudicotyledons	Asteraceae	*Lychnophora*	*Lychnophora ericoides*	FJ031770
Eudicotyledons	Asteraceae	*Lychnophora*	*Lychnophora ericoides*	FJ031771
Eudicotyledons	Asteraceae	*Lychnophora*	*Lychnophora ericoides*	FJ031772
Eudicotyledons	Asteraceae	*Lychnophora*	*Lychnophora ericoides*	FJ031773
Eudicotyledons	Asteraceae	*Lychnophora*	*Lychnophora ericoides*	FJ031774
Eudicotyledons	Asteraceae	*Lychnophora*	*Lychnophora ericoides*	FJ031775
Eudicotyledons	Asteraceae	*Lychnophora*	*Lychnophora ericoides*	FJ031776
Eudicotyledons	Asteraceae	*Lychnophora*	*Lychnophora ericoides*	FJ031777
Eudicotyledons	Asteraceae	*Lychnophora*	*Lychnophora ericoides*	FJ031778
Eudicotyledons	Asteraceae	*Lychnophora*	*Lychnophora ericoides*	FJ031779
Eudicotyledons	Asteraceae	*Lychnophora*	*Lychnophora ericoides*	FJ031780
Eudicotyledons	Asteraceae	*Lychnophora*	*Lychnophora ericoides*	FJ031781
Eudicotyledons	Asteraceae	*Lychnophora*	*Lychnophora ericoides*	FJ031782
Eudicotyledons	Asteraceae	*Lychnophora*	*Lychnophora ericoides*	FJ031783
Eudicotyledons	Asteraceae	*Lychnophora*	*Lychnophora ericoides*	FJ031784
Eudicotyledons	Asteraceae	*Lychnophora*	*Lychnophora ericoides*	FJ031785
Eudicotyledons	Asteraceae	*Lychnophora*	*Lychnophora ericoides*	FJ031786
Eudicotyledons	Asteraceae	*Lychnophora*	*Lychnophora ericoides*	FJ031787
Eudicotyledons	Asteraceae	*Lychnophora*	*Lychnophora ericoides*	FJ031788
Eudicotyledons	Asteraceae	*Lychnophora*	*Lychnophora ericoides*	FJ031789
Eudicotyledons	Asteraceae	*Lychnophora*	*Lychnophora ericoides*	FJ031790
Eudicotyledons	Asteraceae	*Lychnophora*	*Lychnophora ericoides*	FJ031791
Eudicotyledons	Asteraceae	*Lychnophora*	*Lychnophora ericoides*	FJ031792
Eudicotyledons	Asteraceae	*Lychnophora*	*Lychnophora ericoides*	FJ031793
Eudicotyledons	Asteraceae	*Lychnophora*	*Lychnophora ericoides*	FJ031794
Eudicotyledons	Asteraceae	*Lychnophora*	*Lychnophora ericoides*	FJ031795
Eudicotyledons	Asteraceae	*Lychnophora*	*Lychnophora ericoides*	FJ031796
Eudicotyledons	Asteraceae	*Lychnophora*	*Lychnophora ericoides*	FJ031797
Eudicotyledons	Asteraceae	*Lychnophora*	*Lychnophora ericoides*	FJ031798
Eudicotyledons	Asteraceae	*Lychnophora*	*Lychnophora ericoides*	FJ031799
Eudicotyledons	Asteraceae	*Lychnophora*	*Lychnophora ericoides*	FJ031800
Eudicotyledons	Asteraceae	*Lychnophora*	*Lychnophora ericoides*	FJ031801
Eudicotyledons	Asteraceae	*Lychnophora*	*Lychnophora ericoides*	FJ031802
Eudicotyledons	Asteraceae	*Lychnophora*	*Lychnophora ericoides*	FJ031803
Eudicotyledons	Asteraceae	*Lychnophora*	*Lychnophora ericoides*	FJ031804
Eudicotyledons	Asteraceae	*Lychnophora*	*Lychnophora ericoides*	FJ031805
Eudicotyledons	Asteraceae	*Lychnophora*	*Lychnophora ericoides*	FJ031806
Eudicotyledons	Asteraceae	*Lychnophora*	*Lychnophora ericoides*	FJ031807
Eudicotyledons	Asteraceae	*Lychnophora*	*Lychnophora ericoides*	FJ031808

Eudicotyledons	Asteraceae	*Lychnophora*	*Lychnophora ericoides*	FJ031809
Eudicotyledons	Asteraceae	*Lychnophora*	*Lychnophora ericoides*	FJ031810
Eudicotyledons	Asteraceae	*Lychnophora*	*Lychnophora ericoides*	FJ031811
Eudicotyledons	Asteraceae	*Lychnophora*	*Lychnophora ericoides*	FJ031812
Eudicotyledons	Asteraceae	*Lychnophora*	*Lychnophora ericoides*	FJ031813
Eudicotyledons	Asteraceae	*Lychnophora*	*Lychnophora ericoides*	FJ031814
Eudicotyledons	Asteraceae	*Lychnophora*	*Lychnophora ericoides*	FJ031815
Eudicotyledons	Asteraceae	*Lychnophora*	*Lychnophora ericoides*	FJ031816
Eudicotyledons	Asteraceae	*Lychnophora*	*Lychnophora ericoides*	FJ031817
Eudicotyledons	Asteraceae	*Lychnophora*	*Lychnophora ericoides*	FJ031818
Eudicotyledons	Asteraceae	*Lychnophora*	*Lychnophora ericoides*	FJ031819
Eudicotyledons	Asteraceae	*Lychnophora*	*Lychnophora ericoides*	FJ031820
Eudicotyledons	Asteraceae	*Lychnophora*	*Lychnophora ericoides*	FJ031821
Eudicotyledons	Asteraceae	*Lychnophora*	*Lychnophora ericoides*	FJ031822
Eudicotyledons	Asteraceae	*Lychnophora*	*Lychnophora ericoides*	FJ031823
Eudicotyledons	Asteraceae	*Lychnophora*	*Lychnophora ericoides*	FJ031824
Eudicotyledons	Asteraceae	*Lychnophora*	*Lychnophora ericoides*	FJ031825
Eudicotyledons	Asteraceae	*Lychnophora*	*Lychnophora ericoides*	FJ031826
Eudicotyledons	Asteraceae	*Lychnophora*	*Lychnophora ericoides*	FJ031827
Eudicotyledons	Asteraceae	*Lychnophora*	*Lychnophora ericoides*	FJ031828
Eudicotyledons	Asteraceae	*Lychnophora*	*Lychnophora ericoides*	FJ031829
Eudicotyledons	Asteraceae	*Lychnophora*	*Lychnophora ericoides*	FJ031830
Eudicotyledons	Asteraceae	*Lychnophora*	*Lychnophora ericoides*	FJ031831
Eudicotyledons	Asteraceae	*Lychnophora*	*Lychnophora ericoides*	FJ031832
Eudicotyledons	Asteraceae	*Lychnophora*	*Lychnophora ericoides*	FJ031833
Eudicotyledons	Asteraceae	*Lychnophora*	*Lychnophora ericoides*	FJ031834
Eudicotyledons	Asteraceae	*Lychnophora*	*Lychnophora ericoides*	FJ031835
Eudicotyledons	Asteraceae	*Lychnophora*	*Lychnophora ericoides*	FJ031836
Eudicotyledons	Asteraceae	*Lychnophora*	*Lychnophora ericoides*	FJ031837
Eudicotyledons	Asteraceae	*Lychnophora*	*Lychnophora ericoides*	FJ031838
Eudicotyledons	Asteraceae	*Lychnophora*	*Lychnophora ericoides*	FJ031839
Eudicotyledons	Asteraceae	*Lychnophora*	*Lychnophora ericoides*	FJ031840
Eudicotyledons	Asteraceae	*Lychnophora*	*Lychnophora ericoides*	FJ031841
Eudicotyledons	Asteraceae	*Lychnophora*	*Lychnophora ericoides*	FJ031842
Eudicotyledons	Asteraceae	*Lychnophora*	*Lychnophora ericoides*	FJ031843
Eudicotyledons	Asteraceae	*Lychnophora*	*Lychnophora ericoides*	FJ031844
Eudicotyledons	Asteraceae	*Lychnophora*	*Lychnophora ericoides*	FJ031867
Eudicotyledons	Asteraceae	*Lychnophora*	*Lychnophora ericoides*	FJ031868
Eudicotyledons	Asteraceae	*Lychnophora*	*Lychnophora ericoides*	FJ031869
Eudicotyledons	Asteraceae	*Lychnophora*	*Lychnophora ericoides*	FJ031870
Eudicotyledons	Asteraceae	*Lychnophora*	*Lychnophora ericoides*	FJ031871
Eudicotyledons	Asteraceae	*Lychnophora*	*Lychnophora ericoides*	FJ031872
Eudicotyledons	Asteraceae	*Lychnophora*	*Lychnophora ericoides*	FJ031873
Eudicotyledons	Asteraceae	*Lychnophora*	*Lychnophora ericoides*	FJ031874
Eudicotyledons	Asteraceae	*Lychnophora*	*Lychnophora ericoides*	FJ031875
Eudicotyledons	Asteraceae	*Lychnophora*	*Lychnophora ericoides*	FJ031876
Eudicotyledons	Asteraceae	*Lychnophora*	*Lychnophora ericoides*	FJ031877
Eudicotyledons	Asteraceae	*Lychnophora*	*Lychnophora ericoides*	FJ031878
Eudicotyledons	Asteraceae	*Lychnophora*	*Lychnophora ericoides*	FJ031879
Eudicotyledons	Asteraceae	*Lychnophora*	*Lychnophora ericoides*	FJ031880
Eudicotyledons	Asteraceae	*Lychnophora*	*Lychnophora ericoides*	FJ031881
Eudicotyledons	Asteraceae	*Lychnophora*	*Lychnophora ericoides*	FJ031882
Eudicotyledons	Asteraceae	*Lychnophora*	*Lychnophora diamantinana*	FJ031845

Eudicotyledons	Asteraceae	*Lychnophora*	*Lychnophora diamantinana*	FJ031846
Eudicotyledons	Asteraceae	*Lychnophora*	*Lychnophora diamantinana*	FJ031847
Eudicotyledons	Asteraceae	*Lychnophora*	*Lychnophora diamantinana*	FJ031848
Eudicotyledons	Sapotaceae	*Manilkara*	*Manilkara bidentata*	GQ428717
Eudicotyledons	Sapotaceae	*Manilkara*	*Manilkara bidentata*	FJ039059
Eudicotyledons	Sapotaceae	*Manilkara*	*Manilkara bidentata*	HM446954
Eudicotyledons	Meliaceae	*Trichilia*	*Trichilia cipo*	FJ039003
Eudicotyledons	Meliaceae	*Trichilia*	*Trichilia cipo*	FJ039004
Eudicotyledons	Meliaceae	*Trichilia*	*Trichilia pallida*	GQ428732
Eudicotyledons	Meliaceae	*Trichilia*	*Trichilia pallida*	FJ039005
Eudicotyledons	Meliaceae	*Trichilia*	*Trichilia pallida*	FJ039006
Eudicotyledons	Meliaceae	*Trichilia*	*Trichilia pallida*	GQ982392
Eudicotyledons	Meliaceae	*Trichilia*	*Trichilia pallida*	HM447009
Eudicotyledons	Loasaceae	*Mentzelia*	*Mentzelia affinis*	FJ918124
Eudicotyledons	Loasaceae	*Mentzelia*	*Mentzelia affinis*	FJ918125
Eudicotyledons	Loasaceae	*Mentzelia*	*Mentzelia affinis*	FJ918126
Eudicotyledons	Loasaceae	*Mentzelia*	*Mentzelia sp. JMB-2009a*	FJ918131
Eudicotyledons	Loasaceae	*Mentzelia*	*Mentzelia sp. JMB-2009a*	FJ918132
Eudicotyledons	Loasaceae	*Mentzelia*	*Mentzelia sp. JMB-2009a*	FJ918133
Eudicotyledons	Loasaceae	*Mentzelia*	*Mentzelia sp. JMB-2009a*	FJ918134
Eudicotyledons	Loasaceae	*Mentzelia*	*Mentzelia sp. JMB-2009a*	FJ918135
Eudicotyledons	Loasaceae	*Mentzelia*	*Mentzelia californica*	FJ918136
Eudicotyledons	Loasaceae	*Mentzelia*	*Mentzelia californica*	FJ918137
Eudicotyledons	Loasaceae	*Mentzelia*	*Mentzelia californica*	FJ918138
Eudicotyledons	Loasaceae	*Mentzelia*	*Mentzelia crocea*	FJ918142
Eudicotyledons	Loasaceae	*Mentzelia*	*Mentzelia crocea*	FJ918143
Eudicotyledons	Loasaceae	*Mentzelia*	*Mentzelia crocea*	FJ918144
Eudicotyledons	Loasaceae	*Mentzelia*	*Mentzelia desertorum*	FJ918145
Eudicotyledons	Loasaceae	*Mentzelia*	*Mentzelia desertorum*	FJ918146
Eudicotyledons	Loasaceae	*Mentzelia*	*Mentzelia desertorum*	FJ918147
Eudicotyledons	Loasaceae	*Mentzelia*	*Mentzelia desertorum*	FJ918148
Eudicotyledons	Loasaceae	*Mentzelia*	*Mentzelia desertorum*	FJ918149
Eudicotyledons	Loasaceae	*Mentzelia*	*Mentzelia eremophila*	FJ918152
Eudicotyledons	Loasaceae	*Mentzelia*	*Mentzelia eremophila*	FJ918153
Eudicotyledons	Loasaceae	*Mentzelia*	*Mentzelia eremophila*	FJ918154
Eudicotyledons	Loasaceae	*Mentzelia*	*Mentzelia jonesii*	FJ918158
Eudicotyledons	Loasaceae	*Mentzelia*	*Mentzelia jonesii*	FJ918159
Eudicotyledons	Loasaceae	*Mentzelia*	*Mentzelia jonesii*	FJ918160
Eudicotyledons	Loasaceae	*Mentzelia*	*Mentzelia jonesii*	FJ918161
Eudicotyledons	Loasaceae	*Mentzelia*	*Mentzelia jonesii*	FJ918162
Eudicotyledons	Loasaceae	*Mentzelia*	*Mentzelia mojavensis*	FJ918168
Eudicotyledons	Loasaceae	*Mentzelia*	*Mentzelia mojavensis*	FJ918169
Eudicotyledons	Loasaceae	*Mentzelia*	*Mentzelia mojavensis*	FJ918170
Eudicotyledons	Loasaceae	*Mentzelia*	*Mentzelia mojavensis*	FJ918171
Eudicotyledons	Loasaceae	*Mentzelia*	*Mentzelia mollis*	FJ918172
Eudicotyledons	Loasaceae	*Mentzelia*	*Mentzelia mollis*	FJ918173
Eudicotyledons	Loasaceae	*Mentzelia*	*Mentzelia mollis*	FJ918174
Eudicotyledons	Loasaceae	*Mentzelia*	*Mentzelia mollis*	FJ918175
Eudicotyledons	Loasaceae	*Mentzelia*	*Mentzelia mollis*	FJ918176
Eudicotyledons	Loasaceae	*Mentzelia*	*Mentzelia mollis*	FJ918177
Eudicotyledons	Loasaceae	*Mentzelia*	*Mentzelia sp. JMB-2009b*	FJ918178
Eudicotyledons	Loasaceae	*Mentzelia*	*Mentzelia sp. JMB-2009b*	FJ918179
Eudicotyledons	Loasaceae	*Mentzelia*	*Mentzelia sp. JMB-2009b*	FJ918180

Eudicotyledons	Loasaceae	*Mentzelia*	*Mentzelia montana*	FJ918181
Eudicotyledons	Loasaceae	*Mentzelia*	*Mentzelia montana*	FJ918182
Eudicotyledons	Loasaceae	*Mentzelia*	*Mentzelia montana*	FJ918201
Eudicotyledons	Loasaceae	*Mentzelia*	*Mentzelia montana*	FJ918202
Eudicotyledons	Loasaceae	*Mentzelia*	*Mentzelia obscura*	FJ918188
Eudicotyledons	Loasaceae	*Mentzelia*	*Mentzelia obscura*	FJ918189
Eudicotyledons	Loasaceae	*Mentzelia*	*Mentzelia obscura*	FJ918190
Eudicotyledons	Loasaceae	*Mentzelia*	*Mentzelia packardiae*	FJ918191
Eudicotyledons	Loasaceae	*Mentzelia*	*Mentzelia packardiae*	FJ918192
Eudicotyledons	Loasaceae	*Mentzelia*	*Mentzelia packardiae*	FJ918193
Eudicotyledons	Loasaceae	*Mentzelia*	*Mentzelia packardiae*	FJ918194
Eudicotyledons	Loasaceae	*Mentzelia*	*Mentzelia pectinata*	FJ918195
Eudicotyledons	Loasaceae	*Mentzelia*	*Mentzelia pectinata*	FJ918196
Eudicotyledons	Loasaceae	*Mentzelia*	*Mentzelia pectinata*	FJ918197
Eudicotyledons	Loasaceae	*Mentzelia*	*Mentzelia ravenii*	FJ918198
Eudicotyledons	Loasaceae	*Mentzelia*	*Mentzelia ravenii*	FJ918199
Eudicotyledons	Loasaceae	*Mentzelia*	*Mentzelia ravenii*	FJ918200
Eudicotyledons	Loasaceae	*Mentzelia*	*Mentzelia thompsonii*	FJ918203
Eudicotyledons	Loasaceae	*Mentzelia*	*Mentzelia thompsonii*	FJ918204
Eudicotyledons	Loasaceae	*Mentzelia*	*Mentzelia thompsonii*	FJ918205
Eudicotyledons	Loasaceae	*Mentzelia*	*Mentzelia veatchiana*	FJ918206
Eudicotyledons	Loasaceae	*Mentzelia*	*Mentzelia veatchiana*	FJ918207
Eudicotyledons	Loasaceae	*Mentzelia*	*Mentzelia veatchiana*	FJ918208
Eudicotyledons	Loasaceae	*Mentzelia*	*Mentzelia veatchiana*	FJ918209
Eudicotyledons	Caryophyllaceae	*Dianthus*	*Dianthus armeria*	GU441005
Eudicotyledons	Caryophyllaceae	*Dianthus*	*Dianthus armeria*	GU441006
Eudicotyledons	Rosaceae	*Rosa*	*Rosa longicuspis*	GU575154
Eudicotyledons	Rosaceae	*Rosa*	*Rosa longicuspis*	GU575155
Eudicotyledons	Cucurbitaceae	*Momordica*	*Momordica cymbalaria*	GQ163010
Eudicotyledons	Cucurbitaceae	*Momordica*	*Momordica cymbalaria*	GQ163011
Eudicotyledons	Cucurbitaceae	*Momordica*	*Momordica cymbalaria*	GQ163012
Eudicotyledons	Cucurbitaceae	*Momordica*	*Momordica dioica*	GQ163017
Eudicotyledons	Cucurbitaceae	*Momordica*	*Momordica dioica*	GQ163018
Eudicotyledons	Cucurbitaceae	*Momordica*	*Momordica dioica*	GQ163019
Eudicotyledons	Fabaceae	*Acacia*	*Acacia arabica*	FJ808539
Eudicotyledons	Fabaceae	*Acacia*	*Acacia arabica*	FJ808540
Eudicotyledons	Asteraceae	*Artemisia*	*Artemisia lactiflora*	GU724245
Eudicotyledons	Asteraceae	*Artemisia*	*Artemisia lactiflora*	GU724246
Eudicotyledons	Nitrariaceae	*Peganum*	*Peganum multisectum*	FJ752658
Eudicotyledons	Nitrariaceae	*Peganum*	*Peganum multisectum*	FJ752659
Eudicotyledons	Nitrariaceae	*Peganum*	*Peganum multisectum*	FJ752660
Eudicotyledons	Nitrariaceae	*Peganum*	*Peganum multisectum*	FJ752662
Eudicotyledons	Nitrariaceae	*Peganum*	*Peganum multisectum*	FJ752665
Eudicotyledons	Nitrariaceae	*Peganum*	*Peganum nigellastrum*	FJ752654
Eudicotyledons	Nitrariaceae	*Peganum*	*Peganum nigellastrum*	FJ752655
Eudicotyledons	Nitrariaceae	*Peganum*	*Peganum nigellastrum*	FJ752663
Eudicotyledons	Nitrariaceae	*Peganum*	*Peganum nigellastrum*	FJ752667
Eudicotyledons	Nitrariaceae	*Peganum*	*Peganum nigellastrum*	FJ752668
Eudicotyledons	Rosaceae	*Fragaria*	*Fragaria chinensis*	GQ476757
Eudicotyledons	Rosaceae	*Fragaria*	*Fragaria chinensis*	GQ476758
Eudicotyledons	Cucurbitaceae	*Trichosanthes*	*Trichosanthes rosthornii*	GQ845128
Eudicotyledons	Cucurbitaceae	*Trichosanthes*	*Trichosanthes rosthornii*	GQ845129
Eudicotyledons	Cucurbitaceae	*Trichosanthes*	*Trichosanthes rosthornii*	GQ845130

Eudicotyledons	Cucurbitaceae	*Trichosanthes*	*Trichosanthes cucumeroides*	HQ829550
Eudicotyledons	Cucurbitaceae	*Trichosanthes*	*Trichosanthes cucumeroides*	HQ829551
Eudicotyledons	Cucurbitaceae	*Trichosanthes*	*Trichosanthes cucumeroides*	HQ829552
Eudicotyledons	Cucurbitaceae	*Trichosanthes*	*Trichosanthes truncata*	HQ829570
Eudicotyledons	Cucurbitaceae	*Trichosanthes*	*Trichosanthes truncata*	HQ829571
Eudicotyledons	Cucurbitaceae	*Trichosanthes*	*Trichosanthes truncata*	HQ829572
Eudicotyledons	Cucurbitaceae	*Trichosanthes*	*Trichosanthes truncata*	HQ829573
Eudicotyledons	Cucurbitaceae	*Trichosanthes*	*Trichosanthes truncata*	HQ829577
Eudicotyledons	Solanaceae	*Solanum*	*Solanum diphyllum*	GU135297
Eudicotyledons	Solanaceae	*Solanum*	*Solanum diphyllum*	GU135375
Eudicotyledons	Vitaceae	*Vitis*	*Vitis coignetiae*	HQ108319
Eudicotyledons	Vitaceae	*Vitis*	*Vitis coignetiae*	HQ656468
Eudicotyledons	Cucurbitaceae	*Momordica*	*Momordica anigosantha*	GQ162972
Eudicotyledons	Cucurbitaceae	*Momordica*	*Momordica anigosantha*	GQ162973
Eudicotyledons	Cucurbitaceae	*Momordica*	*Momordica boivinii*	GQ162978
Eudicotyledons	Cucurbitaceae	*Momordica*	*Momordica boivinii*	GQ162979
Eudicotyledons	Cucurbitaceae	*Momordica*	*Momordica boivinii*	GQ162980
Eudicotyledons	Cucurbitaceae	*Momordica*	*Momordica camerounensis*	GQ162988
Eudicotyledons	Cucurbitaceae	*Momordica*	*Momordica camerounensis*	GQ162989
Eudicotyledons	Cucurbitaceae	*Momordica*	*Momordica cardiospermoides*	GQ162990
Eudicotyledons	Cucurbitaceae	*Momordica*	*Momordica cardiospermoides*	GQ162991
Eudicotyledons	Cucurbitaceae	*Momordica*	*Momordica clarkeana*	GQ163002
Eudicotyledons	Cucurbitaceae	*Momordica*	*Momordica clarkeana*	GQ163003
Eudicotyledons	Cucurbitaceae	*Momordica*	*Momordica corymbifera*	GQ163008
Eudicotyledons	Cucurbitaceae	*Momordica*	*Momordica corymbifera*	GQ163009
Eudicotyledons	Cucurbitaceae	*Momordica*	*Momordica denticulata*	GQ163013
Eudicotyledons	Cucurbitaceae	*Momordica*	*Momordica denticulata*	GQ163014
Eudicotyledons	Cucurbitaceae	*Momordica*	*Momordica denudata*	GQ163015
Eudicotyledons	Cucurbitaceae	*Momordica*	*Momordica denudata*	GQ163016
Eudicotyledons	Cucurbitaceae	*Momordica*	*Momordica enneaphylla*	GQ163021
Eudicotyledons	Cucurbitaceae	*Momordica*	*Momordica enneaphylla*	GQ163022
Eudicotyledons	Cucurbitaceae	*Momordica*	*Momordica enneaphylla*	GQ163023
Eudicotyledons	Cucurbitaceae	*Momordica*	*Momordica enneaphylla*	GQ163024
Eudicotyledons	Cucurbitaceae	*Momordica*	*Momordica enneaphylla*	GQ163025
Eudicotyledons	Cucurbitaceae	*Momordica*	*Momordica enneaphylla*	GQ163026
Eudicotyledons	Cucurbitaceae	*Momordica*	*Momordica enneaphylla*	GQ163027
Eudicotyledons	Cucurbitaceae	*Momordica*	*Momordica enneaphylla*	GQ163028
Eudicotyledons	Cucurbitaceae	*Momordica*	*Momordica friesiorum*	GQ163033
Eudicotyledons	Cucurbitaceae	*Momordica*	*Momordica friesiorum*	GQ163034
Eudicotyledons	Cucurbitaceae	*Momordica*	*Momordica glabra*	GQ163036
Eudicotyledons	Cucurbitaceae	*Momordica*	*Momordica glabra*	GQ163037
Eudicotyledons	Cucurbitaceae	*Momordica*	*Momordica henriquesii*	GQ163038
Eudicotyledons	Cucurbitaceae	*Momordica*	*Momordica henriquesii*	GQ163039
Eudicotyledons	Cucurbitaceae	*Momordica*	*Momordica humilis*	GQ163040
Eudicotyledons	Cucurbitaceae	*Momordica*	*Momordica humilis*	GQ163041
Eudicotyledons	Cucurbitaceae	*Momordica*	*Momordica macrophylla*	GQ163049
Eudicotyledons	Cucurbitaceae	*Momordica*	*Momordica macrophylla*	GQ163050
Eudicotyledons	Cucurbitaceae	*Momordica*	*Momordica multiflora*	GQ163052
Eudicotyledons	Cucurbitaceae	*Momordica*	*Momordica multiflora*	GQ163053
Eudicotyledons	Cucurbitaceae	*Momordica*	*Momordica multiflora*	GQ163054
Eudicotyledons	Cucurbitaceae	*Momordica*	*Momordica parvifolia*	GQ163058
Eudicotyledons	Cucurbitaceae	*Momordica*	*Momordica parvifolia*	GQ163059
Eudicotyledons	Cucurbitaceae	*Momordica*	*Momordica parvifolia*	GQ163060

Eudicotyledons	Cucurbitaceae	*Momordica*	*Momordica parvifolia*	GQ163061
Eudicotyledons	Cucurbitaceae	*Momordica*	*Momordica pterocarpa*	GQ163063
Eudicotyledons	Cucurbitaceae	*Momordica*	*Momordica pterocarpa*	GQ163064
Eudicotyledons	Cucurbitaceae	*Momordica*	*Momordica rostrata*	GQ163070
Eudicotyledons	Cucurbitaceae	*Momordica*	*Momordica rostrata*	GQ163071
Eudicotyledons	Cucurbitaceae	*Momordica*	*Momordica sessilifolia*	GQ163072
Eudicotyledons	Cucurbitaceae	*Momordica*	*Momordica sessilifolia*	GQ163073
Eudicotyledons	Cucurbitaceae	*Momordica*	*Momordica sphaeroidea*	GQ163081
Eudicotyledons	Cucurbitaceae	*Momordica*	*Momordica sphaeroidea*	GQ163082
Eudicotyledons	Cucurbitaceae	*Momordica*	*Momordica sphaeroidea*	GQ163083
Eudicotyledons	Cucurbitaceae	*Momordica*	*Momordica subangulata*	GQ163085
Eudicotyledons	Cucurbitaceae	*Momordica*	*Momordica subangulata*	GQ163086
Eudicotyledons	Cucurbitaceae	*Momordica*	*Momordica subangulata*	GQ163087
Eudicotyledons	Cucurbitaceae	*Momordica*	*Momordica trifoliolata*	GQ163090
Eudicotyledons	Cucurbitaceae	*Momordica*	*Momordica trifoliolata*	GQ163091
Eudicotyledons	Salicaceae	*Salix*	*Salix alba x Salix pentandra*	GU373308
Eudicotyledons	Salicaceae	*Salix*	*Salix alba x Salix pentandra*	GU373309
Eudicotyledons	Salicaceae	*Salix*	*Salix aurita x Salix myrtilloides*	GU373315
Eudicotyledons	Salicaceae	*Salix*	*Salix aurita x Salix myrtilloides*	GU373323
Eudicotyledons	Salicaceae	*Salix*	*Salix aurita x Salix repens subsp. reper*	GU373316
Eudicotyledons	Salicaceae	*Salix*	*Salix aurita x Salix repens subsp. reper*	GU373317
Eudicotyledons	Salicaceae	*Salix*	*Salix myrsinifolia x Salix phylicifolia*	GU373319
Eudicotyledons	Salicaceae	*Salix*	*Salix myrsinifolia x Salix phylicifolia*	GU373320
Eudicotyledons	Solanaceae	*Solanum*	*Solanum anceps*	HQ856107
Eudicotyledons	Solanaceae	*Solanum*	*Solanum anceps*	HQ856108
Eudicotyledons	Caryophyllaceae	*Dianthus*	*Dianthus broteri*	GU441012
Eudicotyledons	Caryophyllaceae	*Dianthus*	*Dianthus broteri*	GU574182
Eudicotyledons	Caryophyllaceae	*Dianthus*	*Dianthus broteri*	GU574183
Eudicotyledons	Caryophyllaceae	*Dianthus*	*Dianthus broteri*	GU574184
Eudicotyledons	Caryophyllaceae	*Dianthus*	*Dianthus broteri*	GU574185
Eudicotyledons	Caryophyllaceae	*Dianthus*	*Dianthus broteri*	GU574186
Eudicotyledons	Caryophyllaceae	*Dianthus*	*Dianthus broteri*	GU574187
Eudicotyledons	Caryophyllaceae	*Dianthus*	*Dianthus broteri*	GU070815
Eudicotyledons	Caryophyllaceae	*Dianthus*	*Dianthus broteri*	GU070816
Eudicotyledons	Caryophyllaceae	*Dianthus*	*Dianthus broteri*	GU070819
Eudicotyledons	Caryophyllaceae	*Dianthus*	*Dianthus broteri*	GU070820
Eudicotyledons	Caryophyllaceae	*Dianthus*	*Dianthus broteri*	GU070821
Eudicotyledons	Caryophyllaceae	*Dianthus*	*Dianthus broteri*	GU070822
Eudicotyledons	Caryophyllaceae	*Dianthus*	*Dianthus broteri*	GU070823
Eudicotyledons	Caryophyllaceae	*Dianthus*	*Dianthus broteri*	GU070825
Eudicotyledons	Caryophyllaceae	*Dianthus*	*Dianthus broteri*	GU070826
Eudicotyledons	Caryophyllaceae	*Dianthus*	*Dianthus broteri*	GU070827
Eudicotyledons	Caryophyllaceae	*Dianthus*	*Dianthus broteri*	GU070828
Eudicotyledons	Caryophyllaceae	*Dianthus*	*Dianthus broteri*	GU070829
Eudicotyledons	Caryophyllaceae	*Dianthus*	*Dianthus broteri*	GU070830
Eudicotyledons	Caryophyllaceae	*Dianthus*	*Dianthus broteri*	GU070831
Eudicotyledons	Caryophyllaceae	*Dianthus*	*Dianthus broteri*	GU070832
Eudicotyledons	Caryophyllaceae	*Dianthus*	*Dianthus broteri*	GU070833
Eudicotyledons	Caryophyllaceae	*Dianthus*	*Dianthus broteri*	GU070835
Eudicotyledons	Caryophyllaceae	*Dianthus*	*Dianthus broteri*	GU070836
Eudicotyledons	Caryophyllaceae	*Dianthus*	*Dianthus broteri*	GU070837
Eudicotyledons	Caryophyllaceae	*Dianthus*	*Dianthus broteri*	GU070838
Eudicotyledons	Caryophyllaceae	*Dianthus*	*Dianthus broteri*	GU070839

Eudicotyledons	Solanaceae	*Solanum*	*Solanum delagoense*	HM016422
Eudicotyledons	Solanaceae	*Solanum*	*Solanum delagoense*	HM016423
Eudicotyledons	Caryophyllaceae	*Dianthus*	*Dianthus caespitosus*	GU441013
Eudicotyledons	Caryophyllaceae	*Dianthus*	*Dianthus caespitosus*	GU441014
Eudicotyledons	Caryophyllaceae	*Dianthus*	*Dianthus diffusus*	GU441036
Eudicotyledons	Caryophyllaceae	*Dianthus*	*Dianthus diffusus*	GU441037
Eudicotyledons	Caryophyllaceae	*Dianthus*	*Dianthus giganteus*	GU441043
Eudicotyledons	Caryophyllaceae	*Dianthus*	*Dianthus giganteus*	GU441044
Eudicotyledons	Caryophyllaceae	*Dianthus*	*Dianthus lusitanus*	GU441060
Eudicotyledons	Caryophyllaceae	*Dianthus*	*Dianthus lusitanus*	GU441061
Eudicotyledons	Caryophyllaceae	*Dianthus*	*Dianthus serrulatus*	GU441083
Eudicotyledons	Caryophyllaceae	*Dianthus*	*Dianthus serrulatus*	GU441084
Eudicotyledons	Vitaceae	*Vitis*	*Vitis girdiana*	HQ108270
Eudicotyledons	Vitaceae	*Vitis*	*Vitis girdiana*	HQ656406
Eudicotyledons	Vitaceae	*Vitis*	*Vitis vulpina*	JQ182523
Eudicotyledons	Vitaceae	*Vitis*	*Vitis vulpina*	HQ656416
Eudicotyledons	Vitaceae	*Vitis*	*Vitis vulpina*	HQ656429
Eudicotyledons	Oleaceae	*Fraxinus*	*Fraxinus holotricha*	HM367438
Eudicotyledons	Oleaceae	*Fraxinus*	*Fraxinus holotricha*	HM367439
Eudicotyledons	Oleaceae	*Fraxinus*	*Fraxinus holotricha*	HM367440
Eudicotyledons	Oleaceae	*Fraxinus*	*Fraxinus holotricha*	HM367441
Eudicotyledons	Oleaceae	*Fraxinus*	*Fraxinus profunda*	HM367525
Eudicotyledons	Oleaceae	*Fraxinus*	*Fraxinus profunda*	HM367526
Eudicotyledons	Oleaceae	*Fraxinus*	*Fraxinus profunda*	HM367527
Eudicotyledons	Oleaceae	*Fraxinus*	*Fraxinus profunda*	HM367528
Eudicotyledons	Oleaceae	*Fraxinus*	*Fraxinus profunda*	HM367529
Eudicotyledons	Asteraceae	*Santolina*	*Santolina insularis*	JF345741
Eudicotyledons	Asteraceae	*Santolina*	*Santolina insularis*	JF345750
Eudicotyledons	Burseraceae	*Bursera*	*Bursera itzae*	GQ377886
Eudicotyledons	Burseraceae	*Bursera*	*Bursera itzae*	GQ377887
Eudicotyledons	Burseraceae	*Bursera*	*Bursera ovalifolia*	GQ377894
Eudicotyledons	Burseraceae	*Bursera*	*Bursera ovalifolia*	GQ377895
Eudicotyledons	Burseraceae	*Bursera*	*Bursera ovalifolia*	JF919200
Eudicotyledons	Burseraceae	*Bursera*	*Bursera roseana*	GQ377899
Eudicotyledons	Burseraceae	*Bursera*	*Bursera roseana*	GQ377900
Eudicotyledons	Burseraceae	*Bursera*	*Bursera shaferi*	GQ377928
Eudicotyledons	Burseraceae	*Bursera*	*Bursera shaferi*	GQ377929
Eudicotyledons	Celastraceae	*Parnassia*	*Parnassia mysorensis*	JF802352
Eudicotyledons	Celastraceae	*Parnassia*	*Parnassia mysorensis*	JF802353
Eudicotyledons	Celastraceae	*Parnassia*	*Parnassia mysorensis*	JF802354
Eudicotyledons	Celastraceae	*Parnassia*	*Parnassia mysorensis*	JF802355
Eudicotyledons	Celastraceae	*Parnassia*	*Parnassia mysorensis*	JF802356
Eudicotyledons	Celastraceae	*Parnassia*	*Parnassia mysorensis*	JN045834
Eudicotyledons	Celastraceae	*Parnassia*	*Parnassia mysorensis*	JN045835
Eudicotyledons	Celastraceae	*Parnassia*	*Parnassia mysorensis*	JN045836
Eudicotyledons	Celastraceae	*Parnassia*	*Parnassia mysorensis*	JN045837
Eudicotyledons	Oleaceae	*Fraxinus*	*Fraxinus retusa*	HM367534
Eudicotyledons	Oleaceae	*Fraxinus*	*Fraxinus retusa*	HM367535
Eudicotyledons	Oleaceae	*Fraxinus*	*Fraxinus griffithii*	HM367434
Eudicotyledons	Oleaceae	*Fraxinus*	*Fraxinus griffithii*	HM367435
Eudicotyledons	Oleaceae	*Fraxinus*	*Fraxinus griffithii*	HM367436
Eudicotyledons	Oleaceae	*Fraxinus*	*Fraxinus malacophylla*	HM367485
Eudicotyledons	Oleaceae	*Fraxinus*	*Fraxinus malacophylla*	HM367486

Eudicotyledons	Vitaceae	*Vitis*	*Vitis romanetii*	HQ108318
Eudicotyledons	Vitaceae	*Vitis*	*Vitis romanetii*	HQ656446
Eudicotyledons	Myrtaceae	*Myrcia*	*Myrcia splendens*	JN091433
Eudicotyledons	Myrtaceae	*Myrcia*	*Myrcia splendens*	JN091434
Eudicotyledons	Myrtaceae	*Myrcia*	*Myrcia splendens*	HM446970
Eudicotyledons	Vitaceae	*Vitis*	*Vitis x doaniana*	HQ108272
Eudicotyledons	Vitaceae	*Vitis*	*Vitis x doaniana*	HQ108273
Eudicotyledons	Vitaceae	*Vitis*	*Vitis balansana*	HQ108310
Eudicotyledons	Vitaceae	*Vitis*	*Vitis balansana*	HQ656443
Eudicotyledons	Vitaceae	*Vitis*	*Vitis balansana*	HQ656458
Eudicotyledons	Vitaceae	*Vitis*	*Vitis balansana*	HQ656459
Eudicotyledons	Lamiaceae	*Ocimum*	*Ocimum sp. MIB zpl*	FR726111
Eudicotyledons	Lamiaceae	*Ocimum*	*Ocimum sp. MIB zpl*	FR726112
Eudicotyledons	Lamiaceae	*Ocimum*	*Ocimum sp. MIB zpl*	FR726113
Eudicotyledons	Lamiaceae	*Origanum*	*Origanum sp. MIB zpl(1)*	FR726126
Eudicotyledons	Lamiaceae	*Origanum*	*Origanum sp. MIB zpl(1)*	FR726127
Eudicotyledons	Lamiaceae	*Origanum*	*Origanum sp. MIB zpl(1)*	FR726128
Eudicotyledons	Lamiaceae	*Rosmarinus*	*Rosmarinus sp. MIB zpl*	FR726157
Eudicotyledons	Lamiaceae	*Rosmarinus*	*Rosmarinus sp. MIB zpl*	FR726158
Eudicotyledons	Lamiaceae	*Rosmarinus*	*Rosmarinus sp. MIB zpl*	FR726159
Eudicotyledons	Lamiaceae	*Origanum*	*Origanum sp. MIB zpl(2)*	FR726134
Eudicotyledons	Lamiaceae	*Origanum*	*Origanum sp. MIB zpl(2)*	FR726135
Eudicotyledons	Lamiaceae	*Origanum*	*Origanum sp. MIB zpl(2)*	FR726136
Eudicotyledons	Amaranthaceae	*Chenopodium*	*Chenopodium simplex*	HQ596640
Eudicotyledons	Amaranthaceae	*Chenopodium*	*Chenopodium simplex*	HQ596641
Eudicotyledons	Ericaceae	*Rhododendron*	*Rhododendron emarginatum*	JN046803
Eudicotyledons	Ericaceae	*Rhododendron*	*Rhododendron emarginatum*	JN046804
Eudicotyledons	Ericaceae	*Rhododendron*	*Rhododendron emarginatum*	JN046805
Eudicotyledons	Brassicaceae	*Capsella*	*Capsella orientalis*	FR822347
Eudicotyledons	Brassicaceae	*Capsella*	*Capsella orientalis*	FR822348
Eudicotyledons	Brassicaceae	*Capsella*	*Capsella orientalis*	FR822349
Eudicotyledons	Plantaginaceae	*Veronica*	*Veronica barrelieri*	HQ327851
Eudicotyledons	Plantaginaceae	*Veronica*	*Veronica barrelieri*	HQ327852
Eudicotyledons	Plantaginaceae	*Veronica*	*Veronica barrelieri*	HQ327861
Eudicotyledons	Plantaginaceae	*Veronica*	*Veronica barrelieri*	HQ327862
Eudicotyledons	Plantaginaceae	*Veronica*	*Veronica barrelieri*	HQ327875
Eudicotyledons	Plantaginaceae	*Veronica*	*Veronica barrelieri*	HQ327876
Eudicotyledons	Plantaginaceae	*Veronica*	*Veronica barrelieri*	HQ327899
Eudicotyledons	Plantaginaceae	*Veronica*	*Veronica barrelieri*	HQ327900
Eudicotyledons	Aquifoliaceae	*Ilex*	*Ilex kaushue*	JN044944
Eudicotyledons	Aquifoliaceae	*Ilex*	*Ilex kaushue*	JN044945
Eudicotyledons	Aquifoliaceae	*Ilex*	*Ilex kaushue*	JN044946
Eudicotyledons	Aquifoliaceae	*Ilex*	*Ilex pentagona*	JN044951
Eudicotyledons	Aquifoliaceae	*Ilex*	*Ilex pentagona*	JN044952
Eudicotyledons	Aquifoliaceae	*Ilex*	*Ilex pentagona*	JN044953
Eudicotyledons	Vitaceae	*Vitis*	*Vitis lawsonii*	HM585736
Eudicotyledons	Vitaceae	*Vitis*	*Vitis lawsonii*	HM585737
Eudicotyledons	Celastraceae	*Euonymus*	*Euonymus nitidus*	HQ415545
Eudicotyledons	Celastraceae	*Euonymus*	*Euonymus nitidus*	HQ427091
Eudicotyledons	Convolvulaceae	*Convolvulus*	*Convolvulus valentinus*	HQ616177
Eudicotyledons	Convolvulaceae	*Convolvulus*	*Convolvulus valentinus*	HQ616178
Eudicotyledons	Convolvulaceae	*Convolvulus*	*Convolvulus valentinus*	HQ616179
Eudicotyledons	Convolvulaceae	*Convolvulus*	*Convolvulus valentinus*	HQ616183

Eudicotyledons	Convolvulaceae	*Convolvulus*	*Convolvulus valentinus*	HQ616184
Eudicotyledons	Convolvulaceae	*Convolvulus*	*Convolvulus valentinus*	HQ616185
Eudicotyledons	Convolvulaceae	*Convolvulus*	*Convolvulus valentinus*	HQ616186
Eudicotyledons	Convolvulaceae	*Convolvulus*	*Convolvulus valentinus*	HQ616187
Eudicotyledons	Convolvulaceae	*Convolvulus*	*Convolvulus valentinus*	HQ616188
Eudicotyledons	Convolvulaceae	*Convolvulus*	*Convolvulus valentinus*	HQ616189
Eudicotyledons	Convolvulaceae	*Convolvulus*	*Convolvulus valentinus*	HQ616190
Eudicotyledons	Convolvulaceae	*Convolvulus*	*Convolvulus valentinus*	HQ616205
Eudicotyledons	Convolvulaceae	*Convolvulus*	*Convolvulus valentinus*	HQ616206
Eudicotyledons	Celastraceae	*Parnassia*	*Parnassia asarifolia*	JF802307
Eudicotyledons	Celastraceae	*Parnassia*	*Parnassia asarifolia*	JF802308
Eudicotyledons	Celastraceae	*Parnassia*	*Parnassia asarifolia*	JF802309
Eudicotyledons	Celastraceae	*Parnassia*	*Parnassia asarifolia*	JN045781
Eudicotyledons	Celastraceae	*Parnassia*	*Parnassia asarifolia*	JN045782
Eudicotyledons	Celastraceae	*Parnassia*	*Parnassia asarifolia*	JN045783
Eudicotyledons	Celastraceae	*Parnassia*	*Parnassia delavayi*	JF795377
Eudicotyledons	Celastraceae	*Parnassia*	*Parnassia delavayi*	JF802310
Eudicotyledons	Celastraceae	*Parnassia*	*Parnassia delavayi*	JF802311
Eudicotyledons	Celastraceae	*Parnassia*	*Parnassia delavayi*	JF802312
Eudicotyledons	Celastraceae	*Parnassia*	*Parnassia delavayi*	JF802313
Eudicotyledons	Celastraceae	*Parnassia*	*Parnassia delavayi*	JF802314
Eudicotyledons	Celastraceae	*Parnassia*	*Parnassia delavayi*	JN045796
Eudicotyledons	Celastraceae	*Parnassia*	*Parnassia delavayi*	JN045797
Eudicotyledons	Celastraceae	*Parnassia*	*Parnassia delavayi*	JN045798
Eudicotyledons	Celastraceae	*Parnassia*	*Parnassia delavayi*	JN045799
Eudicotyledons	Celastraceae	*Parnassia*	*Parnassia delavayi*	JN045800
Eudicotyledons	Celastraceae	*Parnassia*	*Parnassia deqenensis*	JF802315
Eudicotyledons	Celastraceae	*Parnassia*	*Parnassia deqenensis*	JF802316
Eudicotyledons	Celastraceae	*Parnassia*	*Parnassia deqenensis*	JN105083
Eudicotyledons	Celastraceae	*Parnassia*	*Parnassia dilatata*	JF802317
Eudicotyledons	Celastraceae	*Parnassia*	*Parnassia dilatata*	JF802318
Eudicotyledons	Celastraceae	*Parnassia*	*Parnassia dilatata*	JF802319
Eudicotyledons	Celastraceae	*Parnassia*	*Parnassia dilatata*	JN045801
Eudicotyledons	Celastraceae	*Parnassia*	*Parnassia dilatata*	JN045802
Eudicotyledons	Celastraceae	*Parnassia*	*Parnassia dilatata*	JN045803
Eudicotyledons	Celastraceae	*Parnassia*	*Parnassia epunctulata*	JF802320
Eudicotyledons	Celastraceae	*Parnassia*	*Parnassia epunctulata*	JF802321
Eudicotyledons	Celastraceae	*Parnassia*	*Parnassia epunctulata*	JN045804
Eudicotyledons	Celastraceae	*Parnassia*	*Parnassia epunctulata*	JN045805
Eudicotyledons	Celastraceae	*Parnassia*	*Parnassia esquirolii*	JF802322
Eudicotyledons	Celastraceae	*Parnassia*	*Parnassia esquirolii*	JF802323
Eudicotyledons	Celastraceae	*Parnassia*	*Parnassia farreri*	JF802330
Eudicotyledons	Celastraceae	*Parnassia*	*Parnassia farreri*	JF802331
Eudicotyledons	Celastraceae	*Parnassia*	*Parnassia farreri*	JF802332
Eudicotyledons	Celastraceae	*Parnassia*	*Parnassia farreri*	JN045810
Eudicotyledons	Celastraceae	*Parnassia*	*Parnassia farreri*	JN045811
Eudicotyledons	Celastraceae	*Parnassia*	*Parnassia farreri*	JN045812
Eudicotyledons	Celastraceae	*Parnassia*	*Parnassia foliosa*	JF802333
Eudicotyledons	Celastraceae	*Parnassia*	*Parnassia foliosa*	JF802334
Eudicotyledons	Celastraceae	*Parnassia*	*Parnassia foliosa*	JF802335
Eudicotyledons	Celastraceae	*Parnassia*	*Parnassia foliosa*	JF802336
Eudicotyledons	Celastraceae	*Parnassia*	*Parnassia foliosa*	JN045813
Eudicotyledons	Celastraceae	*Parnassia*	*Parnassia foliosa*	JN045814

Eudicotyledons	Celastraceae	*Parnassia*	*Parnassia foliosa*	JN045815
Eudicotyledons	Celastraceae	*Parnassia*	*Parnassia foliosa*	JN045816
Eudicotyledons	Celastraceae	*Parnassia*	*Parnassia guilinensis*	JF802339
Eudicotyledons	Celastraceae	*Parnassia*	*Parnassia guilinensis*	JF802340
Eudicotyledons	Celastraceae	*Parnassia*	*Parnassia guilinensis*	JF802341
Eudicotyledons	Celastraceae	*Parnassia*	*Parnassia guilinensis*	JN045817
Eudicotyledons	Celastraceae	*Parnassia*	*Parnassia guilinensis*	JN045818
Eudicotyledons	Celastraceae	*Parnassia*	*Parnassia guilinensis*	JN045819
Eudicotyledons	Celastraceae	*Parnassia*	*Parnassia kangdingensis*	JF802342
Eudicotyledons	Celastraceae	*Parnassia*	*Parnassia kangdingensis*	JF802343
Eudicotyledons	Celastraceae	*Parnassia*	*Parnassia kangdingensis*	JN045820
Eudicotyledons	Celastraceae	*Parnassia*	*Parnassia kangdingensis*	JN045821
Eudicotyledons	Celastraceae	*Parnassia*	*Parnassia leptophylla*	JF802346
Eudicotyledons	Celastraceae	*Parnassia*	*Parnassia leptophylla*	JF802347
Eudicotyledons	Celastraceae	*Parnassia*	*Parnassia leptophylla*	JN045829
Eudicotyledons	Celastraceae	*Parnassia*	*Parnassia leptophylla*	JN045830
Eudicotyledons	Celastraceae	*Parnassia*	*Parnassia leptophylla*	JN045831
Eudicotyledons	Celastraceae	*Parnassia*	*Parnassia longipetala*	JF802348
Eudicotyledons	Celastraceae	*Parnassia*	*Parnassia longipetala*	JF802349
Eudicotyledons	Celastraceae	*Parnassia*	*Parnassia longipetala*	JN045832
Eudicotyledons	Celastraceae	*Parnassia*	*Parnassia longipetala*	JN045833
Eudicotyledons	Celastraceae	*Parnassia*	*Parnassia lutea*	JF802350
Eudicotyledons	Celastraceae	*Parnassia*	*Parnassia lutea*	JF802351
Eudicotyledons	Celastraceae	*Parnassia*	*Parnassia noemiae*	JN188290
Eudicotyledons	Celastraceae	*Parnassia*	*Parnassia noemiae*	JF802357
Eudicotyledons	Celastraceae	*Parnassia*	*Parnassia noemiae*	JF802358
Eudicotyledons	Celastraceae	*Parnassia*	*Parnassia noemiae*	JF802361
Eudicotyledons	Celastraceae	*Parnassia*	*Parnassia noemiae*	JN045838
Eudicotyledons	Celastraceae	*Parnassia*	*Parnassia noemiae*	JN045839
Eudicotyledons	Celastraceae	*Parnassia*	*Parnassia nubicola*	JF802359
Eudicotyledons	Celastraceae	*Parnassia*	*Parnassia nubicola*	JF802360
Eudicotyledons	Celastraceae	*Parnassia*	*Parnassia nubicola*	JN045840
Eudicotyledons	Celastraceae	*Parnassia*	*Parnassia nubicola*	JN045841
Eudicotyledons	Celastraceae	*Parnassia*	*Parnassia perciliata*	JF802366
Eudicotyledons	Celastraceae	*Parnassia*	*Parnassia perciliata*	JF802367
Eudicotyledons	Celastraceae	*Parnassia*	*Parnassia perciliata*	JF802368
Eudicotyledons	Celastraceae	*Parnassia*	*Parnassia perciliata*	JN045847
Eudicotyledons	Celastraceae	*Parnassia*	*Parnassia perciliata*	JN045848
Eudicotyledons	Celastraceae	*Parnassia*	*Parnassia perciliata*	JN045849
Eudicotyledons	Celastraceae	*Parnassia*	*Parnassia perciliata*	JN045850
Eudicotyledons	Celastraceae	*Parnassia*	*Parnassia perciliata*	JN045851
Eudicotyledons	Celastraceae	*Parnassia*	*Parnassia submysorensis*	JF802369
Eudicotyledons	Celastraceae	*Parnassia*	*Parnassia submysorensis*	JF802370
Eudicotyledons	Celastraceae	*Parnassia*	*Parnassia venusta*	JF802381
Eudicotyledons	Celastraceae	*Parnassia*	*Parnassia venusta*	JF802382
Eudicotyledons	Celastraceae	*Parnassia*	*Parnassia venusta*	JF802383
Eudicotyledons	Celastraceae	*Parnassia*	*Parnassia venusta*	JN045862
Eudicotyledons	Celastraceae	*Parnassia*	*Parnassia venusta*	JN045863
Eudicotyledons	Celastraceae	*Parnassia*	*Parnassia viridiflora*	JF802384
Eudicotyledons	Celastraceae	*Parnassia*	*Parnassia viridiflora*	JF802385
Eudicotyledons	Celastraceae	*Parnassia*	*Parnassia viridiflora*	JN045864
Eudicotyledons	Celastraceae	*Parnassia*	*Parnassia viridiflora*	JN045865
Eudicotyledons	Celastraceae	*Parnassia*	*Parnassia wightiana*	JF802386

Eudicotyledons	Celastraceae	*Parnassia*	*Parnassia wightiana*	JF802387
Eudicotyledons	Celastraceae	*Parnassia*	*Parnassia wightiana*	JF802388
Eudicotyledons	Celastraceae	*Parnassia*	*Parnassia wightiana*	JF802389
Eudicotyledons	Celastraceae	*Parnassia*	*Parnassia wightiana*	JF802390
Eudicotyledons	Celastraceae	*Parnassia*	*Parnassia wightiana*	JF802391
Eudicotyledons	Celastraceae	*Parnassia*	*Parnassia wightiana*	JF802392
Eudicotyledons	Celastraceae	*Parnassia*	*Parnassia wightiana*	JF802393
Eudicotyledons	Celastraceae	*Parnassia*	*Parnassia wightiana*	JF802394
Eudicotyledons	Celastraceae	*Parnassia*	*Parnassia wightiana*	JF802395
Eudicotyledons	Celastraceae	*Parnassia*	*Parnassia wightiana*	JF802396
Eudicotyledons	Celastraceae	*Parnassia*	*Parnassia wightiana*	JN045866
Eudicotyledons	Celastraceae	*Parnassia*	*Parnassia wightiana*	JN045867
Eudicotyledons	Celastraceae	*Parnassia*	*Parnassia wightiana*	JN045868
Eudicotyledons	Celastraceae	*Parnassia*	*Parnassia wightiana*	JN045869
Eudicotyledons	Celastraceae	*Parnassia*	*Parnassia wightiana*	JN045870
Eudicotyledons	Celastraceae	*Parnassia*	*Parnassia wightiana*	JN045871
Eudicotyledons	Celastraceae	*Parnassia*	*Parnassia yunnanensis*	JF802397
Eudicotyledons	Celastraceae	*Parnassia*	*Parnassia yunnanensis*	JF802398
Eudicotyledons	Celastraceae	*Parnassia*	*Parnassia yunnanensis*	JF802399
Eudicotyledons	Celastraceae	*Parnassia*	*Parnassia yunnanensis*	JF802400
Eudicotyledons	Celastraceae	*Parnassia*	*Parnassia yunnanensis*	JF802401
Eudicotyledons	Celastraceae	*Parnassia*	*Parnassia yunnanensis*	JN045872
Eudicotyledons	Celastraceae	*Parnassia*	*Parnassia yunnanensis*	JN045873
Eudicotyledons	Celastraceae	*Parnassia*	*Parnassia yunnanensis*	JN045874
Eudicotyledons	Celastraceae	*Parnassia*	*Parnassia yunnanensis*	JN045875
Eudicotyledons	Celastraceae	*Parnassia*	*Parnassia yunnanensis*	JN045876
Eudicotyledons	Aquifoliaceae	*Ilex*	*Ilex chinensis*	JN044933
Eudicotyledons	Aquifoliaceae	*Ilex*	*Ilex chinensis*	JN044934
Eudicotyledons	Aquifoliaceae	*Ilex*	*Ilex chinensis*	JN044935
Eudicotyledons	Aquifoliaceae	*Ilex*	*Ilex chinensis*	JN044936
Eudicotyledons	Aquifoliaceae	*Ilex*	*Ilex chinensis*	JN044937
Eudicotyledons	Rubiaceae	*Luculia*	*Luculia yunnanensis*	JN045277
Eudicotyledons	Rubiaceae	*Luculia*	*Luculia yunnanensis*	JN045278
Eudicotyledons	Rubiaceae	*Luculia*	*Luculia yunnanensis*	JN045279
Eudicotyledons	Rubiaceae	*Luculia*	*Luculia yunnanensis*	JN045280
Eudicotyledons	Rubiaceae	*Luculia*	*Luculia yunnanensis*	JN045281
Eudicotyledons	Rubiaceae	*Morinda*	*Morinda badia*	JN045476
Eudicotyledons	Rubiaceae	*Morinda*	*Morinda badia*	JN045477
Eudicotyledons	Rubiaceae	*Morinda*	*Morinda brevipes*	JN045478
Eudicotyledons	Rubiaceae	*Morinda*	*Morinda brevipes*	JN045479
Eudicotyledons	Rubiaceae	*Morinda*	*Morinda brevipes*	JN045480
Eudicotyledons	Rubiaceae	*Morinda*	*Morinda callicarpifolia*	JN045481
Eudicotyledons	Rubiaceae	*Morinda*	*Morinda callicarpifolia*	JN045482
Eudicotyledons	Rubiaceae	*Morinda*	*Morinda hainanensis*	JN045483
Eudicotyledons	Rubiaceae	*Morinda*	*Morinda hainanensis*	JN045484
Eudicotyledons	Rubiaceae	*Morinda*	*Morinda hainanensis*	JN045485
Eudicotyledons	Rubiaceae	*Mussaenda*	*Mussaenda divaricata*	JN045500
Eudicotyledons	Rubiaceae	*Mussaenda*	*Mussaenda divaricata*	JN045501
Eudicotyledons	Rubiaceae	*Mussaenda*	*Mussaenda divaricata*	JN045502
Eudicotyledons	Rubiaceae	*Mussaenda*	*Mussaenda esquirolii*	JN045503
Eudicotyledons	Rubiaceae	*Mussaenda*	*Mussaenda esquirolii*	JN045504
Eudicotyledons	Rubiaceae	*Mussaenda*	*Mussaenda frondosa*	JN045505
Eudicotyledons	Rubiaceae	*Mussaenda*	*Mussaenda frondosa*	JN045506

Eudicotyledons	Rubiaceae	*Mussaenda*	*Mussaenda hossei*	JN045507
Eudicotyledons	Rubiaceae	*Mussaenda*	*Mussaenda hossei*	JN045508
Eudicotyledons	Rubiaceae	*Mussaenda*	*Mussaenda kwangtungensis*	JN045509
Eudicotyledons	Rubiaceae	*Mussaenda*	*Mussaenda kwangtungensis*	JN045510
Eudicotyledons	Rubiaceae	*Mussaenda*	*Mussaenda kwangtungensis*	JN045511
Eudicotyledons	Rubiaceae	*Mussaenda*	*Mussaenda laxiflora*	JN045512
Eudicotyledons	Rubiaceae	*Mussaenda*	*Mussaenda laxiflora*	JN045513
Eudicotyledons	Rubiaceae	*Mussaenda*	*Mussaenda laxiflora*	JN045514
Eudicotyledons	Rubiaceae	*Mussaenda*	*Mussaenda macrophylla*	JN045515
Eudicotyledons	Rubiaceae	*Mussaenda*	*Mussaenda macrophylla*	JN045516
Eudicotyledons	Rubiaceae	*Mussaenda*	*Mussaenda macrophylla*	JN045517
Eudicotyledons	Rubiaceae	*Mussaenda*	*Mussaenda pingbianensis*	JN045518
Eudicotyledons	Rubiaceae	*Mussaenda*	*Mussaenda pingbianensis*	JN045519
Eudicotyledons	Rubiaceae	*Mussaenda*	*Mussaenda treutleri*	JN045533
Eudicotyledons	Rubiaceae	*Mussaenda*	*Mussaenda treutleri*	JN045534
Eudicotyledons	Celastraceae	*Parnassia*	*Parnassia brevistyla*	JF795368
Eudicotyledons	Celastraceae	*Parnassia*	*Parnassia brevistyla*	JF795369
Eudicotyledons	Celastraceae	*Parnassia*	*Parnassia brevistyla*	JF795370
Eudicotyledons	Celastraceae	*Parnassia*	*Parnassia brevistyla*	JF795371
Eudicotyledons	Celastraceae	*Parnassia*	*Parnassia brevistyla*	JN045786
Eudicotyledons	Celastraceae	*Parnassia*	*Parnassia brevistyla*	JN045787
Eudicotyledons	Celastraceae	*Parnassia*	*Parnassia brevistyla*	JN045788
Eudicotyledons	Celastraceae	*Parnassia*	*Parnassia brevistyla*	JN045789
Eudicotyledons	Celastraceae	*Parnassia*	*Parnassia brevistyla*	JN045790
Eudicotyledons	Celastraceae	*Parnassia*	*Parnassia cacuminum*	JN045791
Eudicotyledons	Celastraceae	*Parnassia*	*Parnassia cacuminum*	JN045792
Eudicotyledons	Celastraceae	*Parnassia*	*Parnassia chinensis*	JF795372
Eudicotyledons	Celastraceae	*Parnassia*	*Parnassia chinensis*	JF795373
Eudicotyledons	Celastraceae	*Parnassia*	*Parnassia chinensis*	JF795374
Eudicotyledons	Celastraceae	*Parnassia*	*Parnassia chinensis*	JF795375
Eudicotyledons	Celastraceae	*Parnassia*	*Parnassia chinensis*	JF795376
Eudicotyledons	Celastraceae	*Parnassia*	*Parnassia chinensis*	JN045793
Eudicotyledons	Celastraceae	*Parnassia*	*Parnassia chinensis*	JN045794
Eudicotyledons	Celastraceae	*Parnassia*	*Parnassia chinensis*	JN045795
Eudicotyledons	Orobanchaceae	*Pedicularis*	*Pedicularis alopecuros*	JN045880
Eudicotyledons	Orobanchaceae	*Pedicularis*	*Pedicularis alopecuros*	JN045881
Eudicotyledons	Orobanchaceae	*Pedicularis*	*Pedicularis alopecuros*	JN045882
Eudicotyledons	Orobanchaceae	*Pedicularis*	*Pedicularis alopecuros*	JN045883
Eudicotyledons	Orobanchaceae	*Pedicularis*	*Pedicularis alopecuros*	JN045884
Eudicotyledons	Orobanchaceae	*Pedicularis*	*Pedicularis alopecuros*	JN045885
Eudicotyledons	Orobanchaceae	*Pedicularis*	*Pedicularis amplituba*	JN045886
Eudicotyledons	Orobanchaceae	*Pedicularis*	*Pedicularis amplituba*	JN045887
Eudicotyledons	Orobanchaceae	*Pedicularis*	*Pedicularis batangensis*	JN045897
Eudicotyledons	Orobanchaceae	*Pedicularis*	*Pedicularis batangensis*	JN045898
Eudicotyledons	Orobanchaceae	*Pedicularis*	*Pedicularis batangensis*	JN045899
Eudicotyledons	Orobanchaceae	*Pedicularis*	*Pedicularis batangensis*	JN045900
Eudicotyledons	Orobanchaceae	*Pedicularis*	*Pedicularis cephalantha*	JN045905
Eudicotyledons	Orobanchaceae	*Pedicularis*	*Pedicularis cephalantha*	JN045906
Eudicotyledons	Orobanchaceae	*Pedicularis*	*Pedicularis cephalantha*	JN045907
Eudicotyledons	Orobanchaceae	*Pedicularis*	*Pedicularis cephalantha*	JN045908
Eudicotyledons	Orobanchaceae	*Pedicularis*	*Pedicularis cephalantha*	JN045909
Eudicotyledons	Orobanchaceae	*Pedicularis*	*Pedicularis cephalantha*	JN045910
Eudicotyledons	Orobanchaceae	*Pedicularis*	*Pedicularis clarkei*	JN045913

Eudicotyledons	Orobanchaceae	*Pedicularis*	*Pedicularis clarkei*	JN045914
Eudicotyledons	Orobanchaceae	*Pedicularis*	*Pedicularis comptoniifolia*	JN045915
Eudicotyledons	Orobanchaceae	*Pedicularis*	*Pedicularis comptoniifolia*	JN045916
Eudicotyledons	Orobanchaceae	*Pedicularis*	*Pedicularis crenata*	JN045929
Eudicotyledons	Orobanchaceae	*Pedicularis*	*Pedicularis crenata*	JN045930
Eudicotyledons	Orobanchaceae	*Pedicularis*	*Pedicularis crenata*	JN045931
Eudicotyledons	Orobanchaceae	*Pedicularis*	*Pedicularis crenata*	JN045932
Eudicotyledons	Orobanchaceae	*Pedicularis*	*Pedicularis cymbalaria*	JN045943
Eudicotyledons	Orobanchaceae	*Pedicularis*	*Pedicularis cymbalaria*	JN045944
Eudicotyledons	Orobanchaceae	*Pedicularis*	*Pedicularis cymbalaria*	JN045945
Eudicotyledons	Orobanchaceae	*Pedicularis*	*Pedicularis cymbalaria*	JN045946
Eudicotyledons	Orobanchaceae	*Pedicularis*	*Pedicularis decora*	JN045953
Eudicotyledons	Orobanchaceae	*Pedicularis*	*Pedicularis decora*	JN045954
Eudicotyledons	Orobanchaceae	*Pedicularis*	*Pedicularis deltoidea*	JN045957
Eudicotyledons	Orobanchaceae	*Pedicularis*	*Pedicularis deltoidea*	JN045958
Eudicotyledons	Orobanchaceae	*Pedicularis*	*Pedicularis dissectifolia*	JN045968
Eudicotyledons	Orobanchaceae	*Pedicularis*	*Pedicularis dissectifolia*	JN045969
Eudicotyledons	Orobanchaceae	*Pedicularis*	*Pedicularis dunniana*	JN045972
Eudicotyledons	Orobanchaceae	*Pedicularis*	*Pedicularis dunniana*	JN045973
Eudicotyledons	Orobanchaceae	*Pedicularis*	*Pedicularis dunniana*	JN045974
Eudicotyledons	Orobanchaceae	*Pedicularis*	*Pedicularis fengii*	JN045979
Eudicotyledons	Orobanchaceae	*Pedicularis*	*Pedicularis fengii*	JN045980
Eudicotyledons	Orobanchaceae	*Pedicularis*	*Pedicularis fengii*	JN045981
Eudicotyledons	Orobanchaceae	*Pedicularis*	*Pedicularis floribunda*	JN045982
Eudicotyledons	Orobanchaceae	*Pedicularis*	*Pedicularis floribunda*	JN045983
Eudicotyledons	Orobanchaceae	*Pedicularis*	*Pedicularis gruina*	JN045993
Eudicotyledons	Orobanchaceae	*Pedicularis*	*Pedicularis gruina*	JN045994
Eudicotyledons	Orobanchaceae	*Pedicularis*	*Pedicularis gruina*	JN045995
Eudicotyledons	Orobanchaceae	*Pedicularis*	*Pedicularis gruina*	JN045996
Eudicotyledons	Orobanchaceae	*Pedicularis*	*Pedicularis gruina*	JN045997
Eudicotyledons	Orobanchaceae	*Pedicularis*	*Pedicularis gruina*	JN045998
Eudicotyledons	Orobanchaceae	*Pedicularis*	*Pedicularis ingens*	JN046002
Eudicotyledons	Orobanchaceae	*Pedicularis*	*Pedicularis ingens*	JN046003
Eudicotyledons	Orobanchaceae	*Pedicularis*	*Pedicularis labordei*	JN046013
Eudicotyledons	Orobanchaceae	*Pedicularis*	*Pedicularis labordei*	JN046014
Eudicotyledons	Orobanchaceae	*Pedicularis*	*Pedicularis labordei*	JN046015
Eudicotyledons	Orobanchaceae	*Pedicularis*	*Pedicularis labordei*	JN046016
Eudicotyledons	Orobanchaceae	*Pedicularis*	*Pedicularis labordei*	JN046017
Eudicotyledons	Orobanchaceae	*Pedicularis*	*Pedicularis latituba*	JN046023
Eudicotyledons	Orobanchaceae	*Pedicularis*	*Pedicularis latituba*	JN046024
Eudicotyledons	Orobanchaceae	*Pedicularis*	*Pedicularis latituba*	JN046025
Eudicotyledons	Orobanchaceae	*Pedicularis*	*Pedicularis lecomtei*	JN046026
Eudicotyledons	Orobanchaceae	*Pedicularis*	*Pedicularis lecomtei*	JN046027
Eudicotyledons	Orobanchaceae	*Pedicularis*	*Pedicularis lecomtei*	JN046028
Eudicotyledons	Orobanchaceae	*Pedicularis*	*Pedicularis longipes*	JN046038
Eudicotyledons	Orobanchaceae	*Pedicularis*	*Pedicularis longipes*	JN046039
Eudicotyledons	Orobanchaceae	*Pedicularis*	*Pedicularis macrorhyncha*	JN046052
Eudicotyledons	Orobanchaceae	*Pedicularis*	*Pedicularis macrorhyncha*	JN046053
Eudicotyledons	Orobanchaceae	*Pedicularis*	*Pedicularis macrosiphon*	JN046054
Eudicotyledons	Orobanchaceae	*Pedicularis*	*Pedicularis macrosiphon*	JN046055
Eudicotyledons	Orobanchaceae	*Pedicularis*	*Pedicularis macrosiphon*	JN046056
Eudicotyledons	Orobanchaceae	*Pedicularis*	*Pedicularis maxonii*	JN046057
Eudicotyledons	Orobanchaceae	*Pedicularis*	*Pedicularis maxonii*	JN046058

Eudicotyledons	Orobanchaceae	*Pedicularis*	*Pedicularis nigra*	JN046066
Eudicotyledons	Orobanchaceae	*Pedicularis*	*Pedicularis nigra*	JN046067
Eudicotyledons	Orobanchaceae	*Pedicularis*	*Pedicularis orthocoryne*	JN046072
Eudicotyledons	Orobanchaceae	*Pedicularis*	*Pedicularis orthocoryne*	JN046073
Eudicotyledons	Orobanchaceae	*Pedicularis*	*Pedicularis oxycarpa*	JN046074
Eudicotyledons	Orobanchaceae	*Pedicularis*	*Pedicularis oxycarpa*	JN046075
Eudicotyledons	Orobanchaceae	*Pedicularis*	*Pedicularis oxycarpa*	JN046076
Eudicotyledons	Orobanchaceae	*Pedicularis*	*Pedicularis oxycarpa*	JN046077
Eudicotyledons	Orobanchaceae	*Pedicularis*	*Pedicularis oxycarpa*	JN046078
Eudicotyledons	Orobanchaceae	*Pedicularis*	*Pedicularis oxycarpa*	JN046079
Eudicotyledons	Orobanchaceae	*Pedicularis*	*Pedicularis pseudomelampyriflora*	JN046083
Eudicotyledons	Orobanchaceae	*Pedicularis*	*Pedicularis pseudomelampyriflora*	JN046084
Eudicotyledons	Orobanchaceae	*Pedicularis*	*Pedicularis pseudomelampyriflora*	JN046085
Eudicotyledons	Orobanchaceae	*Pedicularis*	*Pedicularis pseudomelampyriflora*	JN046086
Eudicotyledons	Orobanchaceae	*Pedicularis*	*Pedicularis pseudomelampyriflora*	JN046087
Eudicotyledons	Orobanchaceae	*Pedicularis*	*Pedicularis pseudoversicolor*	JN046088
Eudicotyledons	Orobanchaceae	*Pedicularis*	*Pedicularis pseudoversicolor*	JN046089
Eudicotyledons	Orobanchaceae	*Pedicularis*	*Pedicularis pseudoversicolor*	JN046090
Eudicotyledons	Orobanchaceae	*Pedicularis*	*Pedicularis rigida*	JN046114
Eudicotyledons	Orobanchaceae	*Pedicularis*	*Pedicularis rigida*	JN046115
Eudicotyledons	Orobanchaceae	*Pedicularis*	*Pedicularis rigida*	JN046116
Eudicotyledons	Orobanchaceae	*Pedicularis*	*Pedicularis rigida*	JN046117
Eudicotyledons	Orobanchaceae	*Pedicularis*	*Pedicularis rizhaoensis*	JN046118
Eudicotyledons	Orobanchaceae	*Pedicularis*	*Pedicularis rizhaoensis*	JN046119
Eudicotyledons	Orobanchaceae	*Pedicularis*	*Pedicularis sikangensis*	JN046134
Eudicotyledons	Orobanchaceae	*Pedicularis*	*Pedicularis sikangensis*	JN046135
Eudicotyledons	Orobanchaceae	*Pedicularis*	*Pedicularis sikangensis*	JN046136
Eudicotyledons	Orobanchaceae	*Pedicularis*	*Pedicularis sorbifolia*	JN046145
Eudicotyledons	Orobanchaceae	*Pedicularis*	*Pedicularis sorbifolia*	JN046146
Eudicotyledons	Orobanchaceae	*Pedicularis*	*Pedicularis sorbifolia*	JN046147
Eudicotyledons	Orobanchaceae	*Pedicularis*	*Pedicularis tachanensis*	JN046157
Eudicotyledons	Orobanchaceae	*Pedicularis*	*Pedicularis tachanensis*	JN046158
Eudicotyledons	Orobanchaceae	*Pedicularis*	*Pedicularis tachanensis*	JN046159
Eudicotyledons	Orobanchaceae	*Pedicularis*	*Pedicularis tachanensis*	JN046160
Eudicotyledons	Orobanchaceae	*Pedicularis*	*Pedicularis tenuisecta*	JN046161
Eudicotyledons	Orobanchaceae	*Pedicularis*	*Pedicularis tenuisecta*	JN046162
Eudicotyledons	Orobanchaceae	*Pedicularis*	*Pedicularis tenuisecta*	JN046163
Eudicotyledons	Orobanchaceae	*Pedicularis*	*Pedicularis tongolensis*	JN046173
Eudicotyledons	Orobanchaceae	*Pedicularis*	*Pedicularis tongolensis*	JN046174
Eudicotyledons	Orobanchaceae	*Pedicularis*	*Pedicularis tongolensis*	JN046175
Eudicotyledons	Orobanchaceae	*Pedicularis*	*Pedicularis tongolensis*	JN046176
Eudicotyledons	Orobanchaceae	*Pedicularis*	*Pedicularis tongolensis*	JN046177
Eudicotyledons	Orobanchaceae	*Pedicularis*	*Pedicularis tricolor*	JN046180
Eudicotyledons	Orobanchaceae	*Pedicularis*	*Pedicularis tricolor*	JN046181
Eudicotyledons	Orobanchaceae	*Pedicularis*	*Pedicularis tricolor*	JN046182
Eudicotyledons	Orobanchaceae	*Pedicularis*	*Pedicularis tsekouensis*	JN046183
Eudicotyledons	Orobanchaceae	*Pedicularis*	*Pedicularis tsekouensis*	JN046184
Eudicotyledons	Orobanchaceae	*Pedicularis*	*Pedicularis tsekouensis*	JN046185
Eudicotyledons	Orobanchaceae	*Pedicularis*	*Pedicularis umbelliformis*	JN046186
Eudicotyledons	Orobanchaceae	*Pedicularis*	*Pedicularis umbelliformis*	JN046187
Eudicotyledons	Orobanchaceae	*Pedicularis*	*Pedicularis variegata*	JN046193
Eudicotyledons	Orobanchaceae	*Pedicularis*	*Pedicularis variegata*	JN046194
Eudicotyledons	Orobanchaceae	*Pedicularis*	*Pedicularis vialii*	JN046195

Eudicotyledons	Orobanchaceae	*Pedicularis*	*Pedicularis vialii*	JN046196
Eudicotyledons	Ericaceae	*Rhododendron*	*Rhododendron aperantum*	JN046743
Eudicotyledons	Ericaceae	*Rhododendron*	*Rhododendron aperantum*	JN046744
Eudicotyledons	Ericaceae	*Rhododendron*	*Rhododendron aperantum*	JN046745
Eudicotyledons	Ericaceae	*Rhododendron*	*Rhododendron araiophyllum*	JN046746
Eudicotyledons	Ericaceae	*Rhododendron*	*Rhododendron araiophyllum*	JN046747
Eudicotyledons	Ericaceae	*Rhododendron*	*Rhododendron arizelum*	JN046752
Eudicotyledons	Ericaceae	*Rhododendron*	*Rhododendron arizelum*	JN046753
Eudicotyledons	Ericaceae	*Rhododendron*	*Rhododendron arizelum*	JN046754
Eudicotyledons	Ericaceae	*Rhododendron*	*Rhododendron arizelum*	JN046755
Eudicotyledons	Ericaceae	*Rhododendron*	*Rhododendron bainbridgeanum*	JN046756
Eudicotyledons	Ericaceae	*Rhododendron*	*Rhododendron bainbridgeanum*	JN046757
Eudicotyledons	Ericaceae	*Rhododendron*	*Rhododendron cephalanthum*	JN046775
Eudicotyledons	Ericaceae	*Rhododendron*	*Rhododendron cephalanthum*	JN046776
Eudicotyledons	Ericaceae	*Rhododendron*	*Rhododendron cephalanthum*	JN046777
Eudicotyledons	Ericaceae	*Rhododendron*	*Rhododendron cephalanthum*	JN046778
Eudicotyledons	Ericaceae	*Rhododendron*	*Rhododendron cinnabarinum*	JN046779
Eudicotyledons	Ericaceae	*Rhododendron*	*Rhododendron cinnabarinum*	JN046780
Eudicotyledons	Ericaceae	*Rhododendron*	*Rhododendron cinnabarinum*	JN046781
Eudicotyledons	Ericaceae	*Rhododendron*	*Rhododendron complexum*	JN046784
Eudicotyledons	Ericaceae	*Rhododendron*	*Rhododendron complexum*	JN046785
Eudicotyledons	Ericaceae	*Rhododendron*	*Rhododendron fragariiflorum*	JN046820
Eudicotyledons	Ericaceae	*Rhododendron*	*Rhododendron fragariiflorum*	JN046821
Eudicotyledons	Ericaceae	*Rhododendron*	*Rhododendron fuyuanense*	JN046824
Eudicotyledons	Ericaceae	*Rhododendron*	*Rhododendron fuyuanense*	JN046825
Eudicotyledons	Ericaceae	*Rhododendron*	*Rhododendron haematodes*	JN046830
Eudicotyledons	Ericaceae	*Rhododendron*	*Rhododendron haematodes*	JN046831
Eudicotyledons	Ericaceae	*Rhododendron*	*Rhododendron haematodes*	JN046832
Eudicotyledons	Ericaceae	*Rhododendron*	*Rhododendron haematodes*	JN046833
Eudicotyledons	Ericaceae	*Rhododendron*	*Rhododendron haematodes*	JN046834
Eudicotyledons	Ericaceae	*Rhododendron*	*Rhododendron haematodes*	JN046835
Eudicotyledons	Ericaceae	*Rhododendron*	*Rhododendron heliolepis*	JN046836
Eudicotyledons	Ericaceae	*Rhododendron*	*Rhododendron heliolepis*	JN046837
Eudicotyledons	Ericaceae	*Rhododendron*	*Rhododendron heliolepis*	JN046838
Eudicotyledons	Ericaceae	*Rhododendron*	*Rhododendron heliolepis*	JN046839
Eudicotyledons	Ericaceae	*Rhododendron*	*Rhododendron keleticum*	JN046850
Eudicotyledons	Ericaceae	*Rhododendron*	*Rhododendron keleticum*	JN046851
Eudicotyledons	Ericaceae	*Rhododendron*	*Rhododendron lacteum*	JN046852
Eudicotyledons	Ericaceae	*Rhododendron*	*Rhododendron lacteum*	JN046853
Eudicotyledons	Ericaceae	*Rhododendron*	*Rhododendron lacteum*	JN046854
Eudicotyledons	Ericaceae	*Rhododendron*	*Rhododendron lepidotum*	JN046855
Eudicotyledons	Ericaceae	*Rhododendron*	*Rhododendron lepidotum*	JN046856
Eudicotyledons	Ericaceae	*Rhododendron*	*Rhododendron lepidotum*	JN046857
Eudicotyledons	Ericaceae	*Rhododendron*	*Rhododendron lepidotum*	JN046858
Eudicotyledons	Ericaceae	*Rhododendron*	*Rhododendron lepidotum*	JN046859
Eudicotyledons	Ericaceae	*Rhododendron*	*Rhododendron lepidotum*	JN046860
Eudicotyledons	Ericaceae	*Rhododendron*	*Rhododendron lepidotum*	JN046861
Eudicotyledons	Ericaceae	*Rhododendron*	*Rhododendron lepidotum*	JN046862
Eudicotyledons	Ericaceae	*Rhododendron*	*Rhododendron lepidotum*	JN046863
Eudicotyledons	Ericaceae	*Rhododendron*	*Rhododendron lepidotum*	JN046864
Eudicotyledons	Ericaceae	*Rhododendron*	*Rhododendron microphyton*	JN046883
Eudicotyledons	Ericaceae	*Rhododendron*	*Rhododendron microphyton*	JN046884
Eudicotyledons	Ericaceae	*Rhododendron*	*Rhododendron microphyton*	JN046885

Eudicotyledons	Ericaceae	*Rhododendron*	*Rhododendron microphyton*	JN046886
Eudicotyledons	Ericaceae	*Rhododendron*	*Rhododendron monanthum*	JN046887
Eudicotyledons	Ericaceae	*Rhododendron*	*Rhododendron monanthum*	JN046888
Eudicotyledons	Ericaceae	*Rhododendron*	*Rhododendron monanthum*	JN046889
Eudicotyledons	Ericaceae	*Rhododendron*	*Rhododendron nivale*	JN046896
Eudicotyledons	Ericaceae	*Rhododendron*	*Rhododendron nivale*	JN046897
Eudicotyledons	Ericaceae	*Rhododendron*	*Rhododendron nivale*	JN046898
Eudicotyledons	Ericaceae	*Rhododendron*	*Rhododendron nivale*	JN046899
Eudicotyledons	Ericaceae	*Rhododendron*	*Rhododendron nivale*	JN046900
Eudicotyledons	Ericaceae	*Rhododendron*	*Rhododendron nivale*	JN046901
Eudicotyledons	Ericaceae	*Rhododendron*	*Rhododendron nivale*	JN046902
Eudicotyledons	Ericaceae	*Rhododendron*	*Rhododendron nivale*	JN046903
Eudicotyledons	Ericaceae	*Rhododendron*	*Rhododendron nivale*	JN046904
Eudicotyledons	Ericaceae	*Rhododendron*	*Rhododendron nivale*	JN046905
Eudicotyledons	Ericaceae	*Rhododendron*	*Rhododendron nyingchiense*	JN046906
Eudicotyledons	Ericaceae	*Rhododendron*	*Rhododendron nyingchiense*	JN046907
Eudicotyledons	Ericaceae	*Rhododendron*	*Rhododendron oreotrephes*	JN046908
Eudicotyledons	Ericaceae	*Rhododendron*	*Rhododendron oreotrephes*	JN046909
Eudicotyledons	Ericaceae	*Rhododendron*	*Rhododendron oreotrephes*	JN046910
Eudicotyledons	Ericaceae	*Rhododendron*	*Rhododendron oreotrephes*	JN046911
Eudicotyledons	Ericaceae	*Rhododendron*	*Rhododendron oreotrephes*	JN046912
Eudicotyledons	Ericaceae	*Rhododendron*	*Rhododendron oreotrephes*	JN046913
Eudicotyledons	Ericaceae	*Rhododendron*	*Rhododendron oreotrephes*	JN046914
Eudicotyledons	Ericaceae	*Rhododendron*	*Rhododendron sinonuttallii*	JN046969
Eudicotyledons	Ericaceae	*Rhododendron*	*Rhododendron sinonuttallii*	JN046970
Eudicotyledons	Ericaceae	*Rhododendron*	*Rhododendron sphaeroblastum*	JN046971
Eudicotyledons	Ericaceae	*Rhododendron*	*Rhododendron sphaeroblastum*	JN046972
Eudicotyledons	Ericaceae	*Rhododendron*	*Rhododendron stewartianum*	JN046978
Eudicotyledons	Ericaceae	*Rhododendron*	*Rhododendron stewartianum*	JN046979
Eudicotyledons	Ericaceae	*Rhododendron*	*Rhododendron stewartianum*	JN046980
Eudicotyledons	Ericaceae	*Rhododendron*	*Rhododendron stewartianum*	JN046981
Eudicotyledons	Ericaceae	*Rhododendron*	*Rhododendron stewartianum*	JN046982
Eudicotyledons	Ericaceae	*Rhododendron*	*Rhododendron stewartianum*	JN046983
Eudicotyledons	Ericaceae	*Rhododendron*	*Rhododendron tapetiforme*	JN046984
Eudicotyledons	Ericaceae	*Rhododendron*	*Rhododendron tapetiforme*	JN046985
Eudicotyledons	Ericaceae	*Rhododendron*	*Rhododendron telmateium*	JN046986
Eudicotyledons	Ericaceae	*Rhododendron*	*Rhododendron telmateium*	JN046987
Eudicotyledons	Ericaceae	*Rhododendron*	*Rhododendron telmateium*	JN046988
Eudicotyledons	Ericaceae	*Rhododendron*	*Rhododendron traillianum*	JN046991
Eudicotyledons	Ericaceae	*Rhododendron*	*Rhododendron traillianum*	JN046992
Eudicotyledons	Ericaceae	*Rhododendron*	*Rhododendron traillianum*	JN046993
Eudicotyledons	Ericaceae	*Rhododendron*	*Rhododendron trichostomum*	JN046999
Eudicotyledons	Ericaceae	*Rhododendron*	*Rhododendron trichostomum*	JN047000
Eudicotyledons	Ericaceae	*Rhododendron*	*Rhododendron trichostomum*	JN047001
Eudicotyledons	Ericaceae	*Rhododendron*	*Rhododendron uvariifolium*	JN047004
Eudicotyledons	Ericaceae	*Rhododendron*	*Rhododendron uvariifolium*	JN047005
Eudicotyledons	Ericaceae	*Rhododendron*	*Rhododendron uvariifolium*	JN047006
Eudicotyledons	Ericaceae	*Rhododendron*	*Rhododendron uvariifolium*	JN047007
Eudicotyledons	Ericaceae	*Rhododendron*	*Rhododendron wallichii*	JN047014
Eudicotyledons	Ericaceae	*Rhododendron*	*Rhododendron wallichii*	JN047015
Eudicotyledons	Ericaceae	*Rhododendron*	*Rhododendron yunnanense*	JN047025
Eudicotyledons	Ericaceae	*Rhododendron*	*Rhododendron yunnanense*	JN047026
Eudicotyledons	Ericaceae	*Rhododendron*	*Rhododendron yunnanense*	JN047027

Eudicotyledons	Ericaceae	*Rhododendron*	*Rhododendron yunnanense*	JN047028
Eudicotyledons	Ericaceae	*Rhododendron*	*Rhododendron yunnanense*	JN047029
Eudicotyledons	Polygonaceae	*Rumex*	*Rumex pamiricus*	JN047053
Eudicotyledons	Polygonaceae	*Rumex*	*Rumex pamiricus*	JN047054
Eudicotyledons	Adoxaceae	*Sambucus*	*Sambucus chinensis*	JN047069
Eudicotyledons	Adoxaceae	*Sambucus*	*Sambucus chinensis*	JN047070
Eudicotyledons	Adoxaceae	*Sambucus*	*Sambucus chinensis*	JN047071
Eudicotyledons	Adoxaceae	*Sambucus*	*Sambucus chinensis*	JN047072
Eudicotyledons	Adoxaceae	*Sambucus*	*Sambucus chinensis*	JN047073
Eudicotyledons	Adoxaceae	*Sambucus*	*Sambucus chinensis*	JN047074
Eudicotyledons	Adoxaceae	*Sambucus*	*Sambucus chinensis*	JN047075
Eudicotyledons	Adoxaceae	*Sambucus*	*Sambucus chinensis*	JN047076
Eudicotyledons	Adoxaceae	*Sambucus*	*Sambucus chinensis*	JN047077
Eudicotyledons	Adoxaceae	*Sambucus*	*Sambucus chinensis*	JN047078
Eudicotyledons	Adoxaceae	*Sambucus*	*Sambucus chinensis*	JN047079
Eudicotyledons	Caryophyllaceae	*Silene*	*Silene firma*	JN047118
Eudicotyledons	Caryophyllaceae	*Silene*	*Silene firma*	JN047119
Eudicotyledons	Caryophyllaceae	*Silene*	*Silene firma*	JN047120
Eudicotyledons	Solanaceae	*Solanum*	*Solanum pittosporifolium*	JN047226
Eudicotyledons	Solanaceae	*Solanum*	*Solanum pittosporifolium*	JN047227
Eudicotyledons	Solanaceae	*Solanum*	*Solanum spirale*	JN047228
Eudicotyledons	Solanaceae	*Solanum*	*Solanum spirale*	JN047229
Eudicotyledons	Adoxaceae	*Viburnum*	*Viburnum fordiae*	JN047517
Eudicotyledons	Adoxaceae	*Viburnum*	*Viburnum fordiae*	JN047518
Eudicotyledons	Adoxaceae	*Viburnum*	*Viburnum fordiae*	JN047519
Eudicotyledons	Adoxaceae	*Viburnum*	*Viburnum fordiae*	JN047520
Eudicotyledons	Adoxaceae	*Viburnum*	*Viburnum fordiae*	JN047521
Eudicotyledons	Adoxaceae	*Viburnum*	*Viburnum fordiae*	JN047522
Eudicotyledons	Begoniaceae	*Begonia*	*Begonia biflora*	JN044009
Eudicotyledons	Begoniaceae	*Begonia*	*Begonia biflora*	JN044010
Eudicotyledons	Begoniaceae	*Begonia*	*Begonia biflora*	JN044011
Eudicotyledons	Begoniaceae	*Begonia*	*Begonia biflora*	JN044012
Eudicotyledons	Begoniaceae	*Begonia*	*Begonia biflora*	JN044013
Eudicotyledons	Begoniaceae	*Begonia*	*Begonia biflora*	JN044014
Eudicotyledons	Begoniaceae	*Begonia*	*Begonia biflora*	JN044015
Eudicotyledons	Begoniaceae	*Begonia*	*Begonia biflora*	JN044016
Eudicotyledons	Begoniaceae	*Begonia*	*Begonia crystallina*	JN044019
Eudicotyledons	Begoniaceae	*Begonia*	*Begonia crystallina*	JN044020
Eudicotyledons	Begoniaceae	*Begonia*	*Begonia crystallina*	JN044021
Eudicotyledons	Begoniaceae	*Begonia*	*Begonia crystallina*	JN044022
Eudicotyledons	Begoniaceae	*Begonia*	*Begonia crystallina*	JN044023
Eudicotyledons	Begoniaceae	*Begonia*	*Begonia crystallina*	JN044024
Eudicotyledons	Begoniaceae	*Begonia*	*Begonia crystallina*	JN044025
Eudicotyledons	Begoniaceae	*Begonia*	*Begonia cucurbitifolia*	JN044026
Eudicotyledons	Begoniaceae	*Begonia*	*Begonia cucurbitifolia*	JN044027
Eudicotyledons	Begoniaceae	*Begonia*	*Begonia cucurbitifolia*	JN044028
Eudicotyledons	Begoniaceae	*Begonia*	*Begonia cucurbitifolia*	JN044029
Eudicotyledons	Begoniaceae	*Begonia*	*Begonia cylindrica*	JN044030
Eudicotyledons	Begoniaceae	*Begonia*	*Begonia cylindrica*	JN044031
Eudicotyledons	Begoniaceae	*Begonia*	*Begonia cylindrica*	JN044032
Eudicotyledons	Begoniaceae	*Begonia*	*Begonia daweishanensis*	JN044033
Eudicotyledons	Begoniaceae	*Begonia*	*Begonia daweishanensis*	JN044034
Eudicotyledons	Begoniaceae	*Begonia*	*Begonia daweishanensis*	JN044035

Eudicotyledons	Begoniaceae	*Begonia*	*Begonia daweishanensis*	JN044036
Eudicotyledons	Begoniaceae	*Begonia*	*Begonia daweishanensis*	JN044037
Eudicotyledons	Begoniaceae	*Begonia*	*Begonia daweishanensis*	JN044038
Eudicotyledons	Begoniaceae	*Begonia*	*Begonia daxinensis*	JN044039
Eudicotyledons	Begoniaceae	*Begonia*	*Begonia daxinensis*	JN044040
Eudicotyledons	Begoniaceae	*Begonia*	*Begonia daxinensis*	JN044041
Eudicotyledons	Begoniaceae	*Begonia*	*Begonia daxinensis*	JN044042
Eudicotyledons	Begoniaceae	*Begonia*	*Begonia daxinensis*	JN044043
Eudicotyledons	Begoniaceae	*Begonia*	*Begonia daxinensis*	JN044044
Eudicotyledons	Begoniaceae	*Begonia*	*Begonia fangii*	JN044045
Eudicotyledons	Begoniaceae	*Begonia*	*Begonia fangii*	JN044046
Eudicotyledons	Begoniaceae	*Begonia*	*Begonia fangii*	JN044047
Eudicotyledons	Begoniaceae	*Begonia*	*Begonia fangii*	JN044048
Eudicotyledons	Begoniaceae	*Begonia*	*Begonia filiformis*	JN044049
Eudicotyledons	Begoniaceae	*Begonia*	*Begonia filiformis*	JN044050
Eudicotyledons	Begoniaceae	*Begonia*	*Begonia filiformis*	JN044051
Eudicotyledons	Begoniaceae	*Begonia*	*Begonia filiformis*	JN044052
Eudicotyledons	Begoniaceae	*Begonia*	*Begonia filiformis*	JN044053
Eudicotyledons	Begoniaceae	*Begonia*	*Begonia guangxiensis*	JN044059
Eudicotyledons	Begoniaceae	*Begonia*	*Begonia guangxiensis*	JN044060
Eudicotyledons	Begoniaceae	*Begonia*	*Begonia guangxiensis*	JN044061
Eudicotyledons	Begoniaceae	*Begonia*	*Begonia hekouensis*	JN044062
Eudicotyledons	Begoniaceae	*Begonia*	*Begonia hekouensis*	JN044063
Eudicotyledons	Begoniaceae	*Begonia*	*Begonia hekouensis*	JN044064
Eudicotyledons	Begoniaceae	*Begonia*	*Begonia hekouensis*	JN044065
Eudicotyledons	Begoniaceae	*Begonia*	*Begonia hekouensis*	JN044066
Eudicotyledons	Begoniaceae	*Begonia*	*Begonia hekouensis*	JN044067
Eudicotyledons	Begoniaceae	*Begonia*	*Begonia huangii*	JN044068
Eudicotyledons	Begoniaceae	*Begonia*	*Begonia huangii*	JN044069
Eudicotyledons	Begoniaceae	*Begonia*	*Begonia huangii*	JN044070
Eudicotyledons	Begoniaceae	*Begonia*	*Begonia lanternaria*	JN044071
Eudicotyledons	Begoniaceae	*Begonia*	*Begonia lanternaria*	JN044072
Eudicotyledons	Begoniaceae	*Begonia*	*Begonia lanternaria*	JN044073
Eudicotyledons	Begoniaceae	*Begonia*	*Begonia lanternaria*	JN044074
Eudicotyledons	Begoniaceae	*Begonia*	*Begonia lanternaria*	JN044075
Eudicotyledons	Begoniaceae	*Begonia*	*Begonia longistyla*	JN044081
Eudicotyledons	Begoniaceae	*Begonia*	*Begonia longistyla*	JN044082
Eudicotyledons	Begoniaceae	*Begonia*	*Begonia longistyla*	JN044083
Eudicotyledons	Begoniaceae	*Begonia*	*Begonia longistyla*	JN044084
Eudicotyledons	Begoniaceae	*Begonia*	*Begonia longistyla*	JN044085
Eudicotyledons	Begoniaceae	*Begonia*	*Begonia longistyla*	JN044086
Eudicotyledons	Begoniaceae	*Begonia*	*Begonia longistyla*	JN044087
Eudicotyledons	Begoniaceae	*Begonia*	*Begonia longistyla*	JN044088
Eudicotyledons	Begoniaceae	*Begonia*	*Begonia longistyla*	JN044089
Eudicotyledons	Begoniaceae	*Begonia*	*Begonia longistyla*	JN044090
Eudicotyledons	Begoniaceae	*Begonia*	*Begonia longistyla*	JN044091
Eudicotyledons	Begoniaceae	*Begonia*	*Begonia longistyla*	JN044092
Eudicotyledons	Begoniaceae	*Begonia*	*Begonia luzhaiensis*	JN044093
Eudicotyledons	Begoniaceae	*Begonia*	*Begonia luzhaiensis*	JN044094
Eudicotyledons	Begoniaceae	*Begonia*	*Begonia luzhaiensis*	JN044095
Eudicotyledons	Begoniaceae	*Begonia*	*Begonia megalophyllaria*	JN044099
Eudicotyledons	Begoniaceae	*Begonia*	*Begonia megalophyllaria*	JN044100
Eudicotyledons	Begoniaceae	*Begonia*	*Begonia megalophyllaria*	JN044101

Eudicotyledons	Begoniaceae	*Begonia*	*Begonia megalophyllaria*	JN044102
Eudicotyledons	Begoniaceae	*Begonia*	*Begonia megalophyllaria*	JN044103
Eudicotyledons	Begoniaceae	*Begonia*	*Begonia megalophyllaria*	JN044104
Eudicotyledons	Begoniaceae	*Begonia*	*Begonia mengtzeana*	JN044105
Eudicotyledons	Begoniaceae	*Begonia*	*Begonia mengtzeana*	JN044106
Eudicotyledons	Begoniaceae	*Begonia*	*Begonia mengtzeana*	JN044107
Eudicotyledons	Begoniaceae	*Begonia*	*Begonia mengtzeana*	JN044108
Eudicotyledons	Begoniaceae	*Begonia*	*Begonia mengtzeana*	JN044109
Eudicotyledons	Begoniaceae	*Begonia*	*Begonia ornithophylla*	JN044113
Eudicotyledons	Begoniaceae	*Begonia*	*Begonia ornithophylla*	JN044114
Eudicotyledons	Begoniaceae	*Begonia*	*Begonia platycarpa*	JN044115
Eudicotyledons	Begoniaceae	*Begonia*	*Begonia platycarpa*	JN044116
Eudicotyledons	Begoniaceae	*Begonia*	*Begonia platycarpa*	JN044117
Eudicotyledons	Begoniaceae	*Begonia*	*Begonia platycarpa*	JN044118
Eudicotyledons	Begoniaceae	*Begonia*	*Begonia platycarpa*	JN044119
Eudicotyledons	Begoniaceae	*Begonia*	*Begonia purpureofolia*	JN044120
Eudicotyledons	Begoniaceae	*Begonia*	*Begonia purpureofolia*	JN044121
Eudicotyledons	Begoniaceae	*Begonia*	*Begonia purpureofolia*	JN044122
Eudicotyledons	Begoniaceae	*Begonia*	*Begonia purpureofolia*	JN044123
Eudicotyledons	Begoniaceae	*Begonia*	*Begonia purpureofolia*	JN044124
Eudicotyledons	Begoniaceae	*Begonia*	*Begonia purpureofolia*	JN044125
Eudicotyledons	Begoniaceae	*Begonia*	*Begonia umbraculifolia*	JN044132
Eudicotyledons	Begoniaceae	*Begonia*	*Begonia umbraculifolia*	JN044133
Eudicotyledons	Begoniaceae	*Begonia*	*Begonia umbraculifolia*	JN044134
Eudicotyledons	Begoniaceae	*Begonia*	*Begonia umbraculifolia*	JN044135
Eudicotyledons	Begoniaceae	*Begonia*	*Begonia villifolia*	JN044139
Eudicotyledons	Begoniaceae	*Begonia*	*Begonia villifolia*	JN044140
Eudicotyledons	Begoniaceae	*Begonia*	*Begonia villifolia*	JN044141
Eudicotyledons	Begoniaceae	*Begonia*	*Begonia villifolia*	JN044142
Eudicotyledons	Begoniaceae	*Begonia*	*Begonia villifolia*	JN044143
Eudicotyledons	Begoniaceae	*Begonia*	*Begonia villifolia*	JN044144
Eudicotyledons	Begoniaceae	*Begonia*	*Begonia wangii*	JN044145
Eudicotyledons	Begoniaceae	*Begonia*	*Begonia wangii*	JN044146
Eudicotyledons	Begoniaceae	*Begonia*	*Begonia wangii*	JN044147
Eudicotyledons	Begoniaceae	*Begonia*	*Begonia wangii*	JN044148
Eudicotyledons	Begoniaceae	*Begonia*	*Begonia wangii*	JN044149
Eudicotyledons	Begoniaceae	*Begonia*	*Begonia wangii*	JN044150
Eudicotyledons	Meliaceae	*Cipadessa*	*Cipadessa cinerascens*	JN044304
Eudicotyledons	Meliaceae	*Cipadessa*	*Cipadessa cinerascens*	JN044305
Eudicotyledons	Rubiaceae	*Oldenlandia*	*Oldenlandia bodinieri*	JF699770
Eudicotyledons	Rubiaceae	*Oldenlandia*	*Oldenlandia bodinieri*	JF699771
Eudicotyledons	Rubiaceae	*Oldenlandia*	*Oldenlandia bodinieri*	JF699772
Eudicotyledons	Rubiaceae	*Oldenlandia*	*Oldenlandia bodinieri*	JN044760
Eudicotyledons	Rubiaceae	*Oldenlandia*	*Oldenlandia bodinieri*	JN044761
Eudicotyledons	Rubiaceae	*Oldenlandia*	*Oldenlandia bodinieri*	JN044762
Eudicotyledons	Rubiaceae	*Oldenlandia*	*Oldenlandia chrysotricha*	JF699778
Eudicotyledons	Rubiaceae	*Oldenlandia*	*Oldenlandia chrysotricha*	JF699779
Eudicotyledons	Rubiaceae	*Oldenlandia*	*Oldenlandia chrysotricha*	JF699780
Eudicotyledons	Rubiaceae	*Oldenlandia*	*Oldenlandia chrysotricha*	JF699781
Eudicotyledons	Rubiaceae	*Oldenlandia*	*Oldenlandia chrysotricha*	JN044767
Eudicotyledons	Rubiaceae	*Oldenlandia*	*Oldenlandia chrysotricha*	JN044768
Eudicotyledons	Rubiaceae	*Oldenlandia*	*Oldenlandia ovatifolia*	JF699793
Eudicotyledons	Rubiaceae	*Oldenlandia*	*Oldenlandia ovatifolia*	JF699794

Eudicotyledons	Rubiaceae	*Oldenlandia*	*Oldenlandia ovatifolia*	JF699795
Eudicotyledons	Rubiaceae	*Oldenlandia*	*Oldenlandia ovatifolia*	JF699796
Eudicotyledons	Rubiaceae	*Oldenlandia*	*Oldenlandia ovatifolia*	JN044777
Eudicotyledons	Rubiaceae	*Oldenlandia*	*Oldenlandia ovatifolia*	JN044778
Eudicotyledons	Rubiaceae	*Oldenlandia*	*Oldenlandia tenuipes*	JF699812
Eudicotyledons	Rubiaceae	*Oldenlandia*	*Oldenlandia tenuipes*	JF699813
Eudicotyledons	Rubiaceae	*Oldenlandia*	*Oldenlandia tenuipes*	JN044787
Eudicotyledons	Rubiaceae	*Oldenlandia*	*Oldenlandia tenuipes*	JN044788
Eudicotyledons	Geraniaceae	*Geranium*	*Geranium melanandrum*	JN044737
Eudicotyledons	Geraniaceae	*Geranium*	*Geranium melanandrum*	JN044738
Eudicotyledons	Rubiaceae	*Oldenlandia*	*Oldenlandia assimilis*	JF699761
Eudicotyledons	Rubiaceae	*Oldenlandia*	*Oldenlandia assimilis*	JF699762
Eudicotyledons	Rubiaceae	*Oldenlandia*	*Oldenlandia assimilis*	JF699763
Eudicotyledons	Rubiaceae	*Oldenlandia*	*Oldenlandia assimilis*	JF699764
Eudicotyledons	Vitaceae	*Vitis*	*Vitis biformis*	HQ656418
Eudicotyledons	Vitaceae	*Vitis*	*Vitis biformis*	HQ656419
Eudicotyledons	Vitaceae	*Vitis*	*Vitis chunganensis*	HQ656431
Eudicotyledons	Vitaceae	*Vitis*	*Vitis chunganensis*	JF437159
Eudicotyledons	Vitaceae	*Vitis*	*Vitis retordii*	HQ656428
Eudicotyledons	Vitaceae	*Vitis*	*Vitis retordii*	HQ656435
Eudicotyledons	Fabaceae	*Canavalia*	*Canavalia hawaiiensis*	HQ707433
Eudicotyledons	Fabaceae	*Canavalia*	*Canavalia hawaiiensis*	HQ707440
Eudicotyledons	Fabaceae	*Canavalia*	*Canavalia galeata*	HQ707436
Eudicotyledons	Fabaceae	*Canavalia*	*Canavalia galeata*	HQ707439
Eudicotyledons	Vitaceae	*Vitis*	*Vitis lanata*	JF437162
Eudicotyledons	Vitaceae	*Vitis*	*Vitis lanata*	JF437163
Eudicotyledons	Begoniaceae	*Begonia*	*Begonia liuyanii*	JN044054
Eudicotyledons	Begoniaceae	*Begonia*	*Begonia liuyanii*	JN044055
Eudicotyledons	Begoniaceae	*Begonia*	*Begonia liuyanii*	JN044056
Eudicotyledons	Begoniaceae	*Begonia*	*Begonia liuyanii*	JN044057
Eudicotyledons	Begoniaceae	*Begonia*	*Begonia liuyanii*	JN044058
Eudicotyledons	Begoniaceae	*Begonia*	*Begonia zhengyiana*	JN044151
Eudicotyledons	Begoniaceae	*Begonia*	*Begonia zhengyiana*	JN044152
Eudicotyledons	Celastraceae	*Parnassia*	*Parnassia bifolia*	JN045784
Eudicotyledons	Celastraceae	*Parnassia*	*Parnassia bifolia*	JN045785
Eudicotyledons	Rosaceae	*Prunus*	*Armeniaca zhengheensis*	JN046668
Eudicotyledons	Rosaceae	*Prunus*	*Armeniaca zhengheensis*	JN046669
Eudicotyledons	Lamiaceae	*Scutellaria*	*Scutellaria amoena*	HQ680370
Eudicotyledons	Lamiaceae	*Scutellaria*	*Scutellaria amoena*	HQ680371
Eudicotyledons	Cucurbitaceae	*Trichosanthes*	*Trichosanthes baviensis*	HQ829543
Eudicotyledons	Cucurbitaceae	*Trichosanthes*	*Trichosanthes baviensis*	HQ829544
Eudicotyledons	Cucurbitaceae	*Trichosanthes*	*Trichosanthes baviensis*	HQ829545
Eudicotyledons	Cucurbitaceae	*Trichosanthes*	*Trichosanthes baviensis*	HQ829546
Eudicotyledons	Cucurbitaceae	*Trichosanthes*	*Trichosanthes baviensis*	HQ829547
Eudicotyledons	Cucurbitaceae	*Trichosanthes*	*Trichosanthes baviensis*	HQ829548
Eudicotyledons	Cucurbitaceae	*Trichosanthes*	*Trichosanthes baviensis*	HQ829549
Eudicotyledons	Cucurbitaceae	*Trichosanthes*	*Trichosanthes dunniana*	HQ829553
Eudicotyledons	Cucurbitaceae	*Trichosanthes*	*Trichosanthes dunniana*	HQ829554
Eudicotyledons	Myrtaceae	*Eucalyptus*	*Eucalyptus loxophleba*	HQ170467
Eudicotyledons	Myrtaceae	*Eucalyptus*	*Eucalyptus loxophleba*	HQ170468
Eudicotyledons	Ericaceae	*Rhododendron*	*Rhododendron glanduliferum*	HQ706975
Eudicotyledons	Ericaceae	*Rhododendron*	*Rhododendron glanduliferum*	HQ706976
Eudicotyledons	Ericaceae	*Rhododendron*	*Rhododendron hunanense*	HQ706978

Eudicotyledons	Ericaceae	*Rhododendron*	*Rhododendron hunanense*	HQ706979
Eudicotyledons	Ericaceae	*Rhododendron*	*Rhododendron hypoblematosum*	HQ706980
Eudicotyledons	Ericaceae	*Rhododendron*	*Rhododendron hypoblematosum*	HQ706981
Eudicotyledons	Ericaceae	*Rhododendron*	*Rhododendron rhuyuenense*	HQ707014
Eudicotyledons	Ericaceae	*Rhododendron*	*Rhododendron rhuyuenense*	HQ707015
Eudicotyledons	Ericaceae	*Rhododendron*	*Rhododendron simiarum*	HQ707020
Eudicotyledons	Ericaceae	*Rhododendron*	*Rhododendron simiarum*	HQ707021
Eudicotyledons	Ericaceae	*Rhododendron*	*Rhododendron spanotrichum*	HQ707023
Eudicotyledons	Ericaceae	*Rhododendron*	*Rhododendron spanotrichum*	HQ707024
Eudicotyledons	Ericaceae	*Rhododendron*	*Rhododendron zhangjiajieense*	HQ707040
Eudicotyledons	Ericaceae	*Rhododendron*	*Rhododendron zhangjiajieense*	HQ707041
Eudicotyledons	Ericaceae	*Rhododendron*	*Rhododendron x fragrans*	HQ706973
Eudicotyledons	Ericaceae	*Rhododendron*	*Rhododendron x fragrans*	HQ706974
Eudicotyledons	Solanaceae	*Solanum*	*Solanum limoncochaense*	HQ856102
Eudicotyledons	Solanaceae	*Solanum*	*Solanum limoncochaense*	HQ856103
Eudicotyledons	Solanaceae	*Solanum*	*Solanum loxophyllum*	HQ856099
Eudicotyledons	Solanaceae	*Solanum*	*Solanum loxophyllum*	HQ856100
Eudicotyledons	Lamiaceae	*Ajuga*	*Ajuga orientalis*	HQ902817
Eudicotyledons	Lamiaceae	*Ajuga*	*Ajuga orientalis*	HQ902870
Eudicotyledons	Myrtaceae	*Myrcia*	*Myrcia amazonica*	JN091403
Eudicotyledons	Myrtaceae	*Myrcia*	*Myrcia amazonica*	JN091404
Eudicotyledons	Myrtaceae	*Myrcia*	*Myrcia amazonica*	JN091405
Eudicotyledons	Myrtaceae	*Myrcia*	*Myrcia amazonica*	JN091406
Eudicotyledons	Myrtaceae	*Myrcia*	*Myrcia guianensis*	JN091415
Eudicotyledons	Myrtaceae	*Myrcia*	*Myrcia guianensis*	JN091416
Eudicotyledons	Asteraceae	*Santolina*	*Santolina benthamiana*	JF345734
Eudicotyledons	Asteraceae	*Santolina*	*Santolina benthamiana*	JF345735
Eudicotyledons	Asteraceae	*Santolina*	*Santolina corsica*	JF345747
Eudicotyledons	Asteraceae	*Santolina*	*Santolina corsica*	JF345748
Eudicotyledons	Asteraceae	*Santolina*	*Santolina pectinata*	JF345754
Eudicotyledons	Asteraceae	*Santolina*	*Santolina pectinata*	JF345755
Eudicotyledons	Asteraceae	*Santolina*	*Santolina pectinata*	JF345756
Eudicotyledons	Asteraceae	*Santolina*	*Santolina pectinata*	JF345761
Eudicotyledons	Asteraceae	*Santolina*	*Santolina pectinata*	JF345774
Eudicotyledons	Asteraceae	*Santolina*	*Santolina semidentata*	JF345738
Eudicotyledons	Asteraceae	*Santolina*	*Santolina semidentata*	JF345766
Eudicotyledons	Asteraceae	*Santolina*	*Santolina semidentata*	JF345772
Eudicotyledons	Asteraceae	*Santolina*	*Santolina semidentata*	JF345773
Eudicotyledons	Asteraceae	*Santolina*	*Santolina villosa*	JF345727
Eudicotyledons	Asteraceae	*Santolina*	*Santolina villosa*	JF345728
Eudicotyledons	Asteraceae	*Santolina*	*Santolina villosa*	JF345729
Eudicotyledons	Asteraceae	*Santolina*	*Santolina villosa*	JF345730
Eudicotyledons	Asteraceae	*Santolina*	*Santolina villosa*	JF345731
Eudicotyledons	Asteraceae	*Santolina*	*Santolina villosa*	JF345732
Eudicotyledons	Asteraceae	*Santolina*	*Santolina virens*	JF345764
Eudicotyledons	Asteraceae	*Santolina*	*Santolina virens*	JF345765
Eudicotyledons	Ranunculaceae	*Eranthis*	*Eranthis pungdoensis*	JF505835
Eudicotyledons	Ranunculaceae	*Eranthis*	*Eranthis pungdoensis*	JF505836
Eudicotyledons	Ranunculaceae	*Eranthis*	*Eranthis pungdoensis*	JF505837
Eudicotyledons	Ranunculaceae	*Eranthis*	*Eranthis pungdoensis*	JF505838
Eudicotyledons	Ranunculaceae	*Eranthis*	*Eranthis pungdoensis*	JF505839
Eudicotyledons	Ranunculaceae	*Eranthis*	*Eranthis pungdoensis*	JF505840
Eudicotyledons	Ranunculaceae	*Eranthis*	*Eranthis pungdoensis*	JF505841

Eudicotyledons	Ranunculaceae	*Eranthis*	*Eranthis pungdoensis*	JF505842
Eudicotyledons	Orobanchaceae	*Agalinis*	*Agalinis auriculata*	EU827893
Eudicotyledons	Orobanchaceae	*Agalinis*	*Agalinis auriculata*	EU827894
Eudicotyledons	Rosaceae	*Dasiphora*	*Dasiphora fruticosa*	JN044373
Eudicotyledons	Rosaceae	*Dasiphora*	*Dasiphora fruticosa*	JN044374
Eudicotyledons	Rosaceae	*Dasiphora*	*Dasiphora fruticosa*	JN044375
Eudicotyledons	Rosaceae	*Dasiphora*	*Dasiphora fruticosa*	JN044376
Eudicotyledons	Rosaceae	*Dasiphora*	*Dasiphora fruticosa*	JN044377
Eudicotyledons	Rosaceae	*Dasiphora*	*Dasiphora fruticosa*	JN044378
Eudicotyledons	Rosaceae	*Dasiphora*	*Dasiphora fruticosa*	JN044379
Eudicotyledons	Araliaceae	*Dendropanax*	*Dendropanax arboreus*	GQ982206
Eudicotyledons	Araliaceae	*Dendropanax*	*Dendropanax arboreus*	GU054860
Eudicotyledons	Araliaceae	*Dendropanax*	*Dendropanax arboreus*	GU054874
Eudicotyledons	Araliaceae	*Dendropanax*	*Dendropanax arboreus*	GU054882
Eudicotyledons	Araliaceae	*Dendropanax*	*Dendropanax arboreus*	HM446923
Eudicotyledons	Araliaceae	*Schefflera*	*Schefflera arboricola*	GU054816
Eudicotyledons	Araliaceae	*Schefflera*	*Schefflera arboricola*	FN675800
Eudicotyledons	Fabaceae	*Schizolobium*	*Schizolobium parahyba*	GQ167769
Eudicotyledons	Fabaceae	*Schizolobium*	*Schizolobium parahyba*	AF524985
Eudicotyledons	Fabaceae	*Schizolobium*	*Schizolobium parahyba*	FJ668606
Eudicotyledons	Fabaceae	*Schizolobium*	*Schizolobium parahyba*	FJ668607
Eudicotyledons	Fabaceae	*Schizolobium*	*Schizolobium parahyba*	FJ668610
Eudicotyledons	Fabaceae	*Schizolobium*	*Schizolobium parahyba*	FJ668611
Eudicotyledons	Fabaceae	*Schizolobium*	*Schizolobium parahyba*	FJ668612
Eudicotyledons	Fabaceae	*Schizolobium*	*Schizolobium parahyba*	FJ668613
Eudicotyledons	Fabaceae	*Schizolobium*	*Schizolobium parahyba*	GQ982357
Eudicotyledons	Polygonaceae	*Persicaria*	*Persicaria hydropiper*	HQ596796
Eudicotyledons	Polygonaceae	*Persicaria*	*Persicaria hydropiper*	EF653753
Eudicotyledons	Polygonaceae	*Persicaria*	*Persicaria hydropiper*	EF653754
Eudicotyledons	Polygonaceae	*Persicaria*	*Persicaria hydropiper*	EU750490
Eudicotyledons	Polygonaceae	*Persicaria*	*Persicaria hydropiper*	EU750491
Eudicotyledons	Polygonaceae	*Persicaria*	*Persicaria hydropiper*	JN046425
Eudicotyledons	Polygonaceae	*Persicaria*	*Persicaria hydropiper*	JN046426
Eudicotyledons	Elaeagnaceae	*Hippophae*	*Hippophae salicifolia*	JN044909
Eudicotyledons	Elaeagnaceae	*Hippophae*	*Hippophae salicifolia*	JN044910
Eudicotyledons	Elaeagnaceae	*Hippophae*	*Hippophae salicifolia*	JN044911
Eudicotyledons	Elaeagnaceae	*Hippophae*	*Hippophae salicifolia*	JN044912
Eudicotyledons	Elaeagnaceae	*Hippophae*	*Hippophae salicifolia*	JN044913
Eudicotyledons	Orobanchaceae	*Castilleja*	*Castilleja miniata*	FJ765679
Eudicotyledons	Orobanchaceae	*Castilleja*	*Castilleja miniata*	FJ765680
Eudicotyledons	Orobanchaceae	*Castilleja*	*Castilleja miniata*	FJ765681
Eudicotyledons	Orobanchaceae	*Castilleja*	*Castilleja miniata*	FJ765682
Eudicotyledons	Orobanchaceae	*Castilleja*	*Castilleja miniata*	FJ765683
Eudicotyledons	Orobanchaceae	*Castilleja*	*Castilleja miniata*	FJ765684
Eudicotyledons	Orobanchaceae	*Castilleja*	*Castilleja miniata*	FJ765685
Eudicotyledons	Orobanchaceae	*Castilleja*	*Castilleja miniata*	FJ765686
Eudicotyledons	Orobanchaceae	*Castilleja*	*Castilleja miniata*	FJ765687
Eudicotyledons	Orobanchaceae	*Castilleja*	*Castilleja miniata*	FJ765688
Eudicotyledons	Orobanchaceae	*Castilleja*	*Castilleja miniata*	FJ765689
Eudicotyledons	Orobanchaceae	*Castilleja*	*Castilleja miniata*	FJ765690
Eudicotyledons	Orobanchaceae	*Castilleja*	*Castilleja miniata*	FJ765691
Eudicotyledons	Orobanchaceae	*Castilleja*	*Castilleja miniata*	FJ765692
Eudicotyledons	Orobanchaceae	*Castilleja*	*Castilleja miniata*	FJ765693

Eudicotyledons	Orobanchaceae	*Castilleja*	*Castilleja miniata*	FJ765694
Eudicotyledons	Gesneriaceae	*Cyrtandra*	*Cyrtandra hawaiensis*	GQ475133
Eudicotyledons	Gesneriaceae	*Cyrtandra*	*Cyrtandra hawaiensis*	GQ475158
Eudicotyledons	Gesneriaceae	*Cyrtandra*	*Cyrtandra hawaiensis*	GQ475159
Eudicotyledons	Gesneriaceae	*Cyrtandra*	*Cyrtandra hawaiensis*	EU920008
Eudicotyledons	Ericaceae	*Pieris*	*Pieris floribunda*	EU547720
Eudicotyledons	Ericaceae	*Pieris*	*Pieris floribunda*	AB206629
Eudicotyledons	Ericaceae	*Pieris*	*Pieris formosa*	HQ426993
Eudicotyledons	Ericaceae	*Pieris*	*Pieris formosa*	EU547718
Eudicotyledons	Ericaceae	*Pieris*	*Pieris formosa*	AB206627
Eudicotyledons	Ericaceae	*Pieris*	*Pieris nana*	EU547716
Eudicotyledons	Ericaceae	*Pieris*	*Pieris nana*	AB206630
Eudicotyledons	Ericaceae	*Pieris*	*Pieris nana*	AB259356
Eudicotyledons	Ericaceae	*Pieris*	*Pieris phillyreifolia*	EU547721
Eudicotyledons	Ericaceae	*Pieris*	*Pieris phillyreifolia*	AB206628
Eudicotyledons	Apiaceae	*Osmorhiza*	*Osmorhiza aristata*	JN045638
Eudicotyledons	Apiaceae	*Osmorhiza*	*Osmorhiza aristata*	JN045639
Eudicotyledons	Apiaceae	*Osmorhiza*	*Osmorhiza aristata*	JN045640
Eudicotyledons	Apiaceae	*Peucedanum*	*Peucedanum japonicum*	JN046219
Eudicotyledons	Apiaceae	*Peucedanum*	*Peucedanum japonicum*	JN046220
Eudicotyledons	Apiaceae	*Peucedanum*	*Peucedanum japonicum*	JN046221
Eudicotyledons	Fabaceae	*Glycyrrhiza*	*Glycyrrhiza glabra*	HE659562
Eudicotyledons	Fabaceae	*Glycyrrhiza*	*Glycyrrhiza glabra*	GU396809
Eudicotyledons	Fabaceae	*Glycyrrhiza*	*Glycyrrhiza glabra*	GU396810
Eudicotyledons	Lamiaceae	*Thymus*	*Thymus vulgaris*	FR726148
Eudicotyledons	Lamiaceae	*Thymus*	*Thymus vulgaris*	FR726149
Eudicotyledons	Lamiaceae	*Thymus*	*Thymus vulgaris*	FR726150
Eudicotyledons	Lamiaceae	*Thymus*	*Thymus vulgaris*	AY679164
Eudicotyledons	Asteraceae	*Sonchus*	*Sonchus asper*	FJ395510
Eudicotyledons	Asteraceae	*Sonchus*	*Sonchus asper*	EU750573
Eudicotyledons	Asteraceae	*Sonchus*	*Sonchus asper*	EU750574
Eudicotyledons	Asteraceae	*Sonchus*	*Sonchus asper*	EU750575
Eudicotyledons	Asteraceae	*Sonchus*	*Sonchus asper*	AY457989
Eudicotyledons	Asteraceae	*Sonchus*	*Sonchus oleraceus*	HQ596853
Eudicotyledons	Asteraceae	*Sonchus*	*Sonchus oleraceus*	EU750576
Eudicotyledons	Asteraceae	*Sonchus*	*Sonchus oleraceus*	EU750577
Eudicotyledons	Asteraceae	*Sonchus*	*Sonchus oleraceus*	AY457987
Eudicotyledons	Asteraceae	*Sonchus*	*Sonchus oleraceus*	AY457988
Eudicotyledons	Asteraceae	*Taraxacum*	*Taraxacum officinale*	FJ395471
Eudicotyledons	Asteraceae	*Taraxacum*	*Taraxacum officinale*	HQ162035
Eudicotyledons	Ranunculaceae	*Aconitum*	*Aconitum nagarum*	GQ337841
Eudicotyledons	Ranunculaceae	*Aconitum*	*Aconitum nagarum*	GQ337842
Eudicotyledons	Ranunculaceae	*Aconitum*	*Aconitum nagarum*	FJ821186
Eudicotyledons	Hamamelidaceae	*Corylopsis*	*Corylopsis pauciflora*	AB237062
Eudicotyledons	Hamamelidaceae	*Corylopsis*	*Corylopsis pauciflora*	AB237065
Eudicotyledons	Hamamelidaceae	*Corylopsis*	*Corylopsis pauciflora*	AB237066
Eudicotyledons	Hamamelidaceae	*Corylopsis*	*Corylopsis pauciflora*	AB237077
Eudicotyledons	Hamamelidaceae	*Corylopsis*	*Corylopsis spicata*	AB237063
Eudicotyledons	Hamamelidaceae	*Corylopsis*	*Corylopsis spicata*	AB237075
Eudicotyledons	Apocynaceae	*Alstonia*	*Alstonia scholaris*	GQ435037
Eudicotyledons	Apocynaceae	*Alstonia*	*Alstonia scholaris*	GQ435038
Eudicotyledons	Apocynaceae	*Alstonia*	*Alstonia scholaris*	JN245985
Eudicotyledons	Lamiaceae	*Lamium*	*Lamium amplexicaule*	AY281318

Eudicotyledons	Lamiaceae	*Lamium*	*Lamium amplexicaule*	HQ902820
Eudicotyledons	Lamiaceae	*Lamium*	*Lamium amplexicaule*	HQ902852
Eudicotyledons	Lamiaceae	*Lamium*	*Lamium maculatum*	JF780167
Eudicotyledons	Lamiaceae	*Lamium*	*Lamium maculatum*	JF780168
Eudicotyledons	Lamiaceae	*Lamium*	*Lamium purpureum*	JF780180
Eudicotyledons	Lamiaceae	*Lamium*	*Lamium purpureum*	JF780181
Eudicotyledons	Lamiaceae	*Lamium*	*Lamium purpureum*	JF780182
Eudicotyledons	Lamiaceae	*Lamium*	*Lamium purpureum*	JF780183
Eudicotyledons	Fabaceae	*Andira*	*Andira inermis*	GQ429134
Eudicotyledons	Fabaceae	*Andira*	*Andira inermis*	GQ982145
Eudicotyledons	Fabaceae	*Andira*	*Andira inermis*	HM446886
Eudicotyledons	Fabaceae	*Chamaecrista*	*Chamaecrista fasciculata*	GQ248261
Eudicotyledons	Fabaceae	*Chamaecrista*	*Chamaecrista fasciculata*	EF590677
Eudicotyledons	Fabaceae	*Dalbergia*	*Dalbergia hupeana*	GU396817
Eudicotyledons	Fabaceae	*Dalbergia*	*Dalbergia hupeana*	HQ426987
Eudicotyledons	Fabaceae	*Senna*	*Senna alata*	GU396790
Eudicotyledons	Fabaceae	*Senna*	*Senna alata*	GU969278
Eudicotyledons	Fabaceae	*Senna*	*Senna alata*	GQ435366
Eudicotyledons	Fabaceae	*Senna*	*Senna alata*	HQ161753
Eudicotyledons	Polygonaceae	*Polygonum*	*Polygonum chinense*	GQ435161
Eudicotyledons	Polygonaceae	*Polygonum*	*Polygonum chinense*	JN407039
Eudicotyledons	Polygonaceae	*Polygonum*	*Polygonum chinense*	JN407040
Eudicotyledons	Polygonaceae	*Polygonum*	*Polygonum chinense*	JN407041
Eudicotyledons	Polygonaceae	*Polygonum*	*Polygonum chinense*	JN407042
Eudicotyledons	Polygonaceae	*Polygonum*	*Polygonum chinense*	EU554053
Eudicotyledons	Apiaceae	*Osmorhiza*	*Osmorhiza longistylis*	DQ006137
Eudicotyledons	Apiaceae	*Osmorhiza*	*Osmorhiza longistylis*	JN045660
Eudicotyledons	Apiaceae	*Osmorhiza*	*Osmorhiza longistylis*	JN045661
Eudicotyledons	Apiaceae	*Osmorhiza*	*Osmorhiza longistylis*	JN045662
Eudicotyledons	Apiaceae	*Osmorhiza*	*Osmorhiza occidentalis*	JN045666
Eudicotyledons	Apiaceae	*Osmorhiza*	*Osmorhiza occidentalis*	JN045667
Eudicotyledons	Apiaceae	*Osmorhiza*	*Osmorhiza occidentalis*	JN045668
Eudicotyledons	Apiaceae	*Osmorhiza*	*Osmorhiza occidentalis*	JN045669
Eudicotyledons	Symplocaceae	*Symplocos*	*Symplocos paniculata*	HQ427077
Eudicotyledons	Symplocaceae	*Symplocos*	*Symplocos paniculata*	AB115370
Eudicotyledons	Asteraceae	*Solidago*	*Solidago canadensis*	EU337694
Eudicotyledons	Asteraceae	*Solidago*	*Solidago canadensis*	EU337695
Eudicotyledons	Asteraceae	*Solidago*	*Solidago canadensis*	EU337696
Eudicotyledons	Asteraceae	*Solidago*	*Solidago canadensis*	EU337697
Eudicotyledons	Asteraceae	*Solidago*	*Solidago canadensis*	EU337698
Eudicotyledons	Asteraceae	*Solidago*	*Solidago canadensis*	EU337699
Eudicotyledons	Asteraceae	*Solidago*	*Solidago canadensis*	EU337700
Eudicotyledons	Asteraceae	*Solidago*	*Solidago canadensis*	EU750563
Eudicotyledons	Asteraceae	*Solidago*	*Solidago canadensis*	EU750564
Eudicotyledons	Asteraceae	*Solidago*	*Solidago canadensis*	HQ142550
Eudicotyledons	Asteraceae	*Leucogenes*	*Leucogenes leontopodium*	EU007663
Eudicotyledons	Asteraceae	*Leucogenes*	*Leucogenes leontopodium*	EU007664
Eudicotyledons	Asteraceae	*Leucogenes*	*Leucogenes leontopodium*	AY611227
Eudicotyledons	Theaceae	*Schima*	*Schima superba*	HM100541
Eudicotyledons	Theaceae	*Schima*	*Schima superba*	HM100542
Eudicotyledons	Theaceae	*Schima*	*Schima superba*	HQ415477
Eudicotyledons	Theaceae	*Schima*	*Schima superba*	HQ427073
Eudicotyledons	Theaceae	*Schima*	*Schima superba*	GQ435327

Eudicotyledons	Theaceae	*Stewartia*	*Stewartia pseudocamellia*	HM100572
Eudicotyledons	Theaceae	*Stewartia*	*Stewartia pseudocamellia*	HM100573
Eudicotyledons	Theaceae	*Stewartia*	*Stewartia pseudocamellia*	HM100574
Eudicotyledons	Theaceae	*Stewartia*	*Stewartia pseudocamellia*	HM100575
Eudicotyledons	Primulaceae	*Primula*	*Primula meadia*	GU066767
Eudicotyledons	Primulaceae	*Primula*	*Primula meadia*	GU066768
Eudicotyledons	Primulaceae	*Primula*	*Primula meadia*	GU066769
Eudicotyledons	Primulaceae	*Primula*	*Primula meadia*	GU066770
Eudicotyledons	Primulaceae	*Primula*	*Primula meadia*	GU066771
Eudicotyledons	Primulaceae	*Primula*	*Primula meadia*	GU066772
Eudicotyledons	Primulaceae	*Primula*	*Primula meadia*	HM778141
Eudicotyledons	Primulaceae	*Primula*	*Primula meadia*	HM778142
Eudicotyledons	Primulaceae	*Primula*	*Primula meadia*	HM778143
Eudicotyledons	Primulaceae	*Primula*	*Primula meadia*	HM778144
Eudicotyledons	Primulaceae	*Primula*	*Primula meadia*	HM778146
Eudicotyledons	Primulaceae	*Primula*	*Primula meadia*	HM778147
Eudicotyledons	Primulaceae	*Primula*	*Primula meadia*	HM778148
Eudicotyledons	Primulaceae	*Primula*	*Primula meadia*	HM778150
Eudicotyledons	Primulaceae	*Primula*	*Primula sieboldii*	AB161492
Eudicotyledons	Primulaceae	*Primula*	*Primula sieboldii*	AB161493
Eudicotyledons	Primulaceae	*Primula*	*Primula sieboldii*	AB161494
Eudicotyledons	Primulaceae	*Primula*	*Primula sieboldii*	AB161495
Eudicotyledons	Primulaceae	*Primula*	*Primula sieboldii*	AB161496
Eudicotyledons	Primulaceae	*Primula*	*Primula sieboldii*	AB277089
Eudicotyledons	Primulaceae	*Primula*	*Primula sieboldii*	AB277090
Eudicotyledons	Primulaceae	*Primula*	*Primula sieboldii*	AB381925
Eudicotyledons	Hydrangeaceae	*Schizophragma*	*Schizophragma hydrangeoides*	HM217001
Eudicotyledons	Hydrangeaceae	*Schizophragma*	*Schizophragma hydrangeoides*	JF321271
Eudicotyledons	Gesneriaceae	*Cyrtandra*	*Cyrtandra falcifolia*	GQ475147
Eudicotyledons	Gesneriaceae	*Cyrtandra*	*Cyrtandra falcifolia*	GQ475149
Eudicotyledons	Gesneriaceae	*Cyrtandra*	*Cyrtandra compressa*	GQ475128
Eudicotyledons	Gesneriaceae	*Cyrtandra*	*Cyrtandra compressa*	EU920029
Eudicotyledons	Gesneriaceae	*Cyrtandra*	*Cyrtandra pogonantha*	GQ475125
Eudicotyledons	Gesneriaceae	*Cyrtandra*	*Cyrtandra pogonantha*	GQ475129
Eudicotyledons	Gesneriaceae	*Cyrtandra*	*Cyrtandra pogonantha*	EU920027
Eudicotyledons	Apiaceae	*Pleurospermum*	*Pleurospermum uralense*	FJ475169
Eudicotyledons	Apiaceae	*Pleurospermum*	*Pleurospermum uralense*	JN046393
Eudicotyledons	Apiaceae	*Pleurospermum*	*Pleurospermum uralense*	JN046394
Eudicotyledons	Apiaceae	*Pleurospermum*	*Pleurospermum uralense*	JN046395
Eudicotyledons	Apiaceae	*Pleurospermum*	*Pleurospermum uralense*	JN046396
Eudicotyledons	Apiaceae	*Pleurospermum*	*Pleurospermum uralense*	JN046397
Eudicotyledons	Hamamelidaceae	*Loropetalum*	*Loropetalum chinense*	GU576774
Eudicotyledons	Hamamelidaceae	*Loropetalum*	*Loropetalum chinense*	GU576775
Eudicotyledons	Hamamelidaceae	*Loropetalum*	*Loropetalum chinense*	HQ427005
Eudicotyledons	Hamamelidaceae	*Loropetalum*	*Loropetalum chinense*	JN542801
Eudicotyledons	Hamamelidaceae	*Loropetalum*	*Loropetalum chinense*	JN542802
Eudicotyledons	Hamamelidaceae	*Loropetalum*	*Loropetalum chinense*	JN542803
Eudicotyledons	Hamamelidaceae	*Loropetalum*	*Loropetalum chinense*	JN542804
Eudicotyledons	Hamamelidaceae	*Loropetalum*	*Loropetalum chinense*	JN542805
Eudicotyledons	Hamamelidaceae	*Loropetalum*	*Loropetalum chinense*	JN542806
Eudicotyledons	Hamamelidaceae	*Loropetalum*	*Loropetalum chinense*	JN542807
Eudicotyledons	Hamamelidaceae	*Loropetalum*	*Loropetalum chinense*	JN542808
Eudicotyledons	Hamamelidaceae	*Loropetalum*	*Loropetalum chinense*	JN542809

Eudicotyledons	Hamamelidaceae	*Loropetalum*	*Loropetalum chinense*	JN542810
Eudicotyledons	Hamamelidaceae	*Loropetalum*	*Loropetalum chinense*	JN542811
Eudicotyledons	Hamamelidaceae	*Loropetalum*	*Loropetalum chinense*	JN542812
Eudicotyledons	Hamamelidaceae	*Loropetalum*	*Loropetalum chinense*	JN542813
Eudicotyledons	Hamamelidaceae	*Loropetalum*	*Loropetalum chinense*	JN542814
Eudicotyledons	Hamamelidaceae	*Loropetalum*	*Loropetalum chinense*	JN542815
Eudicotyledons	Hamamelidaceae	*Loropetalum*	*Loropetalum chinense*	JN542816
Eudicotyledons	Hamamelidaceae	*Loropetalum*	*Loropetalum chinense*	JN542817
Eudicotyledons	Hamamelidaceae	*Loropetalum*	*Loropetalum chinense*	JN542818
Eudicotyledons	Hamamelidaceae	*Loropetalum*	*Loropetalum chinense*	JN542819
Eudicotyledons	Hamamelidaceae	*Loropetalum*	*Loropetalum chinense*	JN542820
Eudicotyledons	Hamamelidaceae	*Loropetalum*	*Loropetalum chinense*	JN542821
Eudicotyledons	Hamamelidaceae	*Loropetalum*	*Loropetalum chinense*	JN542822
Eudicotyledons	Hamamelidaceae	*Loropetalum*	*Loropetalum chinense*	JN542823
Eudicotyledons	Hamamelidaceae	*Loropetalum*	*Loropetalum chinense*	JN542824
Eudicotyledons	Hamamelidaceae	*Loropetalum*	*Loropetalum chinense*	JN542825
Eudicotyledons	Hamamelidaceae	*Loropetalum*	*Loropetalum chinense*	JN542826
Eudicotyledons	Hamamelidaceae	*Loropetalum*	*Loropetalum chinense*	JN542827
Eudicotyledons	Hamamelidaceae	*Loropetalum*	*Loropetalum chinense*	JN542828
Eudicotyledons	Hamamelidaceae	*Loropetalum*	*Loropetalum chinense*	AB237078
Eudicotyledons	Fabaceae	*Vachellia*	*Vachellia farnesiana*	AF195723
Eudicotyledons	Fabaceae	*Vachellia*	*Vachellia farnesiana*	FJ808541
Eudicotyledons	Fabaceae	*Vachellia*	*Vachellia farnesiana*	FJ808542
Eudicotyledons	Fabaceae	*Vachellia*	*Vachellia farnesiana*	FJ808543
Eudicotyledons	Fabaceae	*Vachellia*	*Vachellia farnesiana*	FJ808544
Eudicotyledons	Fabaceae	*Vachellia*	*Vachellia farnesiana*	FJ808545
Eudicotyledons	Fabaceae	*Vachellia*	*Vachellia farnesiana*	FJ808546
Eudicotyledons	Fabaceae	*Vachellia*	*Vachellia farnesiana*	FJ808547
Eudicotyledons	Fabaceae	*Vachellia*	*Vachellia farnesiana*	FJ808548
Eudicotyledons	Fabaceae	*Vachellia*	*Vachellia farnesiana*	FJ808549
Eudicotyledons	Fabaceae	*Vachellia*	*Vachellia farnesiana*	FJ808550
Eudicotyledons	Fabaceae	*Vachellia*	*Vachellia farnesiana*	FJ808551
Eudicotyledons	Fabaceae	*Vachellia*	*Vachellia farnesiana*	FJ808552
Eudicotyledons	Fabaceae	*Vachellia*	*Vachellia farnesiana*	FJ808553
Eudicotyledons	Fabaceae	*Vachellia*	*Vachellia farnesiana*	FJ808554
Eudicotyledons	Fabaceae	*Senna*	*Senna alexandrina*	JF838365
Eudicotyledons	Fabaceae	*Senna*	*Senna alexandrina*	HQ161771
Eudicotyledons	Fabaceae	*Glycyrrhiza*	*Glycyrrhiza uralensis*	GU396733
Eudicotyledons	Fabaceae	*Glycyrrhiza*	*Glycyrrhiza uralensis*	GU396734
Eudicotyledons	Fabaceae	*Glycyrrhiza*	*Glycyrrhiza uralensis*	GU396735
Eudicotyledons	Fabaceae	*Glycyrrhiza*	*Glycyrrhiza uralensis*	AB649776
Eudicotyledons	Brassicaceae	*Cardamine*	*Cardamine impatiens*	JN044162
Eudicotyledons	Brassicaceae	*Cardamine*	*Cardamine impatiens*	JN044163
Eudicotyledons	Brassicaceae	*Cardamine*	*Cardamine impatiens*	JN044164
Eudicotyledons	Brassicaceae	*Cardamine*	*Cardamine impatiens*	JN044165
Eudicotyledons	Brassicaceae	*Cardamine*	*Cardamine impatiens*	JN044166
Eudicotyledons	Gentianaceae	*Gentianopsis*	*Gentianopsis crinita*	HM460854
Eudicotyledons	Gentianaceae	*Gentianopsis*	*Gentianopsis crinita*	HM460855
Eudicotyledons	Gentianaceae	*Gentianopsis*	*Gentianopsis crinita*	HM460856
Eudicotyledons	Ranunculaceae	*Aconitum*	*Aconitum carmichaelii*	GQ337742
Eudicotyledons	Ranunculaceae	*Aconitum*	*Aconitum carmichaelii*	GQ337743
Eudicotyledons	Ranunculaceae	*Aconitum*	*Aconitum carmichaelii*	GQ337744
Eudicotyledons	Ranunculaceae	*Aconitum*	*Aconitum carmichaelii*	GQ337745

Eudicotyledons	Ranunculaceae	*Aconitum*	*Aconitum carmichaelii*	GQ337746
Eudicotyledons	Ranunculaceae	*Aconitum*	*Aconitum carmichaelii*	GQ337747
Eudicotyledons	Ranunculaceae	*Aconitum*	*Aconitum carmichaelii*	GQ337748
Eudicotyledons	Ranunculaceae	*Aconitum*	*Aconitum carmichaelii*	GQ337749
Eudicotyledons	Ranunculaceae	*Aconitum*	*Aconitum carmichaelii*	GQ337750
Eudicotyledons	Ranunculaceae	*Aconitum*	*Aconitum carmichaelii*	GQ337765
Eudicotyledons	Ranunculaceae	*Aconitum*	*Aconitum carmichaelii*	GQ337766
Eudicotyledons	Ranunculaceae	*Aconitum*	*Aconitum carmichaelii*	GQ337767
Eudicotyledons	Ranunculaceae	*Aconitum*	*Aconitum carmichaelii*	GQ337768
Eudicotyledons	Ranunculaceae	*Aconitum*	*Aconitum carmichaelii*	GQ337769
Eudicotyledons	Ranunculaceae	*Aconitum*	*Aconitum carmichaelii*	GQ337770
Eudicotyledons	Ranunculaceae	*Aconitum*	*Aconitum carmichaelii*	GQ337771
Eudicotyledons	Ranunculaceae	*Aconitum*	*Aconitum carmichaelii*	GQ337772
Eudicotyledons	Ranunculaceae	*Aconitum*	*Aconitum carmichaelii*	GQ337773
Eudicotyledons	Ranunculaceae	*Aconitum*	*Aconitum carmichaelii*	GQ337778
Eudicotyledons	Ranunculaceae	*Aconitum*	*Aconitum carmichaelii*	GQ337810
Eudicotyledons	Ranunculaceae	*Aconitum*	*Aconitum carmichaelii*	GQ337811
Eudicotyledons	Ranunculaceae	*Aconitum*	*Aconitum carmichaelii*	GQ337823
Eudicotyledons	Ranunculaceae	*Aconitum*	*Aconitum carmichaelii*	GQ337850
Eudicotyledons	Ranunculaceae	*Aconitum*	*Aconitum carmichaelii*	GQ337851
Eudicotyledons	Ranunculaceae	*Aconitum*	*Aconitum carmichaelii*	GQ337852
Eudicotyledons	Ranunculaceae	*Aconitum*	*Aconitum carmichaelii*	GQ337853
Eudicotyledons	Ranunculaceae	*Aconitum*	*Aconitum carmichaelii*	FJ821166
Eudicotyledons	Ranunculaceae	*Aconitum*	*Aconitum carmichaelii*	FJ821167
Eudicotyledons	Asteraceae	*Taraxacum*	*Taraxacum mongolicum*	HQ436189
Eudicotyledons	Asteraceae	*Taraxacum*	*Taraxacum mongolicum*	JN406943
Eudicotyledons	Asteraceae	*Taraxacum*	*Taraxacum mongolicum*	JN406944
Eudicotyledons	Asteraceae	*Taraxacum*	*Taraxacum mongolicum*	JN406945
Eudicotyledons	Asteraceae	*Taraxacum*	*Taraxacum mongolicum*	JN406946
Eudicotyledons	Asteraceae	*Craspedia*	*Craspedia aurantia*	EF187709
Eudicotyledons	Asteraceae	*Craspedia*	*Craspedia aurantia*	EF187716
Eudicotyledons	Asteraceae	*Leucogenes*	*Leucogenes grandiceps*	EU007658
Eudicotyledons	Asteraceae	*Leucogenes*	*Leucogenes grandiceps*	EU007659
Eudicotyledons	Asteraceae	*Leucogenes*	*Leucogenes grandiceps*	EU007661
Eudicotyledons	Asteraceae	*Leucogenes*	*Leucogenes grandiceps*	EU007662
Eudicotyledons	Asteraceae	*Leucogenes*	*Leucogenes grandiceps*	EU007669
Eudicotyledons	Asteraceae	*Leucogenes*	*Leucogenes grandiceps*	EU007670
Eudicotyledons	Asteraceae	*Leucogenes*	*Leucogenes grandiceps*	EU007671
Eudicotyledons	Asteraceae	*Leucogenes*	*Leucogenes grandiceps*	AY611224
Eudicotyledons	Polygonaceae	*Persicaria*	*Persicaria tinctoria*	EU554055
Eudicotyledons	Polygonaceae	*Persicaria*	*Persicaria tinctoria*	EU197005
Eudicotyledons	Caprifoliaceae	*Lonicera*	*Lonicera japonica*	GU135313
Eudicotyledons	Caprifoliaceae	*Lonicera*	*Lonicera japonica*	JN045252
Eudicotyledons	Caprifoliaceae	*Lonicera*	*Lonicera japonica*	JN045253
Eudicotyledons	Caprifoliaceae	*Lonicera*	*Lonicera japonica*	JN045254
Eudicotyledons	Caprifoliaceae	*Lonicera*	*Lonicera japonica*	HM228536
Eudicotyledons	Caprifoliaceae	*Lonicera*	*Lonicera japonica*	HM228537
Eudicotyledons	Caprifoliaceae	*Lonicera*	*Lonicera japonica*	HM228538
Eudicotyledons	Caprifoliaceae	*Lonicera*	*Lonicera japonica*	HM228539
Eudicotyledons	Caprifoliaceae	*Lonicera*	*Lonicera japonica*	HM228540
Eudicotyledons	Caprifoliaceae	*Lonicera*	*Lonicera japonica*	HM228541
Eudicotyledons	Caprifoliaceae	*Lonicera*	*Lonicera japonica*	HM228542
Eudicotyledons	Caprifoliaceae	*Lonicera*	*Lonicera japonica*	HM228543

Eudicotyledons	Apiaceae	*Osmorhiza*	*Osmorhiza claytonii*	HQ596776
Eudicotyledons	Apiaceae	*Osmorhiza*	*Osmorhiza claytonii*	JN045650
Eudicotyledons	Apiaceae	*Osmorhiza*	*Osmorhiza claytonii*	JN045651
Eudicotyledons	Fabaceae	*Dalbergia*	*Dalbergia cochinchinensis*	FR854148
Eudicotyledons	Fabaceae	*Dalbergia*	*Dalbergia cochinchinensis*	FR854149
Eudicotyledons	Fabaceae	*Dalbergia*	*Dalbergia cochinchinensis*	FR854150
Eudicotyledons	Fabaceae	*Dalbergia*	*Dalbergia cochinchinensis*	FR854151
Eudicotyledons	Fabaceae	*Dalbergia*	*Dalbergia cochinchinensis*	FR854152
Eudicotyledons	Fabaceae	*Dalbergia*	*Dalbergia sissoo*	GU396738
Eudicotyledons	Fabaceae	*Dalbergia*	*Dalbergia sissoo*	GU135327
Eudicotyledons	Apiaceae	*Osmorhiza*	*Osmorhiza brachypoda*	JN045648
Eudicotyledons	Apiaceae	*Osmorhiza*	*Osmorhiza brachypoda*	JN045649
Eudicotyledons	Apiaceae	*Osmorhiza*	*Osmorhiza depauperata*	JN045652
Eudicotyledons	Apiaceae	*Osmorhiza*	*Osmorhiza depauperata*	JN045653
Eudicotyledons	Apiaceae	*Osmorhiza*	*Osmorhiza depauperata*	JN045654
Eudicotyledons	Apiaceae	*Osmorhiza*	*Osmorhiza depauperata*	JN045655
Eudicotyledons	Brassicaceae	*Cardamine*	*Cardamine macrophylla*	JN044167
Eudicotyledons	Brassicaceae	*Cardamine*	*Cardamine macrophylla*	JN044168
Eudicotyledons	Primulaceae	*Primula*	*Primula sikkimensis*	JN046577
Eudicotyledons	Primulaceae	*Primula*	*Primula sikkimensis*	JN046578
Eudicotyledons	Primulaceae	*Primula*	*Primula sikkimensis*	JN046579
Eudicotyledons	Primulaceae	*Primula*	*Primula sikkimensis*	JN046580
Eudicotyledons	Primulaceae	*Primula*	*Primula sikkimensis*	JN046581
Eudicotyledons	Primulaceae	*Lysimachia*	*Lysimachia congestiflora*	JN045311
Eudicotyledons	Primulaceae	*Lysimachia*	*Lysimachia congestiflora*	JN045312
Eudicotyledons	Primulaceae	*Lysimachia*	*Lysimachia congestiflora*	JN045313
Eudicotyledons	Primulaceae	*Lysimachia*	*Lysimachia congestiflora*	JN045314
Eudicotyledons	Primulaceae	*Lysimachia*	*Lysimachia congestiflora*	JN045315
Eudicotyledons	Caprifoliaceae	*Lonicera*	*Lonicera dasystyla*	GQ435289
Eudicotyledons	Caprifoliaceae	*Lonicera*	*Lonicera dasystyla*	HM228544
Eudicotyledons	Caprifoliaceae	*Lonicera*	*Lonicera dasystyla*	HM228545
Eudicotyledons	Caprifoliaceae	*Lonicera*	*Lonicera similis*	HM228546
Eudicotyledons	Caprifoliaceae	*Lonicera*	*Lonicera similis*	HM228547
Eudicotyledons	Caprifoliaceae	*Lonicera*	*Lonicera similis*	HM228548
Eudicotyledons	Caprifoliaceae	*Lonicera*	*Lonicera similis*	HM228549
Eudicotyledons	Ranunculaceae	*Aconitum*	*Aconitum anthora*	AF216570
Eudicotyledons	Ranunculaceae	*Aconitum*	*Aconitum anthora*	FN675821
Eudicotyledons	Ranunculaceae	*Aconitum*	*Aconitum lycoctonum*	AF216560
Eudicotyledons	Ranunculaceae	*Aconitum*	*Aconitum lycoctonum*	AF216561
Eudicotyledons	Ranunculaceae	*Aconitum*	*Aconitum lycoctonum*	AF216562
Eudicotyledons	Ranunculaceae	*Aconitum*	*Aconitum lycoctonum*	AF216563
Eudicotyledons	Ranunculaceae	*Aconitum*	*Aconitum lycoctonum*	AF216564
Eudicotyledons	Ranunculaceae	*Aconitum*	*Aconitum lycoctonum*	AF216565
Eudicotyledons	Ranunculaceae	*Aconitum*	*Aconitum lycoctonum*	AF216566
Eudicotyledons	Ranunculaceae	*Aconitum*	*Aconitum lycoctonum*	AF216568
Eudicotyledons	Ranunculaceae	*Aconitum*	*Aconitum lycoctonum*	AF216569
Eudicotyledons	Ranunculaceae	*Aconitum*	*Aconitum lycoctonum*	AF216571
Eudicotyledons	Ranunculaceae	*Aconitum*	*Aconitum lycoctonum*	AF216573
Eudicotyledons	Ranunculaceae	*Aconitum*	*Aconitum lycoctonum*	AF216574
Eudicotyledons	Ranunculaceae	*Aconitum*	*Aconitum lycoctonum*	FN675818
Eudicotyledons	Ranunculaceae	*Aconitum*	*Aconitum moldavicum*	AF216558
Eudicotyledons	Ranunculaceae	*Aconitum*	*Aconitum moldavicum*	AF216577
Eudicotyledons	Ranunculaceae	*Aconitum*	*Aconitum napellus*	AF216567

Eudicotyledons	Ranunculaceae	*Aconitum*	*Aconitum napellus*	FN675819
Eudicotyledons	Ranunculaceae	*Aconitum*	*Aconitum septentrionale*	AF216575
Eudicotyledons	Ranunculaceae	*Aconitum*	*Aconitum septentrionale*	AF216576
Eudicotyledons	Fabaceae	*Andira*	*Andira aubletii*	GQ428688
Eudicotyledons	Fabaceae	*Andira*	*Andira aubletii*	GQ428766
Eudicotyledons	Fabaceae	*Andira*	*Andira aubletii*	FJ038928
Eudicotyledons	Asteraceae	*Craspedia*	*Craspedia variabilis*	EF187694
Eudicotyledons	Asteraceae	*Craspedia*	*Craspedia variabilis*	EF187708
Eudicotyledons	Fabaceae	*Senna*	*Senna occidentalis*	GU396776
Eudicotyledons	Fabaceae	*Senna*	*Senna occidentalis*	GU969280
Eudicotyledons	Fabaceae	*Senna*	*Senna occidentalis*	HQ161765
Eudicotyledons	Ranunculaceae	*Delphinium*	*Delphinium delavayi*	JN044402
Eudicotyledons	Ranunculaceae	*Delphinium*	*Delphinium delavayi*	JN044403
Eudicotyledons	Rosaceae	*Sanguisorba*	*Sanguisorba minor*	FR865111
Eudicotyledons	Rosaceae	*Sanguisorba*	*Sanguisorba minor*	EU937564
Eudicotyledons	Rosaceae	*Sanguisorba*	*Sanguisorba officinalis*	GQ435245
Eudicotyledons	Rosaceae	*Sanguisorba*	*Sanguisorba officinalis*	GQ435246
Eudicotyledons	Rosaceae	*Sanguisorba*	*Sanguisorba officinalis*	GQ435247
Eudicotyledons	Rosaceae	*Sanguisorba*	*Sanguisorba officinalis*	EU937565
Eudicotyledons	Polygonaceae	*Persicaria*	*Persicaria posumbu*	EF653752
Eudicotyledons	Polygonaceae	*Persicaria*	*Persicaria posumbu*	EU196996
Eudicotyledons	Polygonaceae	*Persicaria*	*Persicaria posumbu*	DQ006216
Eudicotyledons	Polygonaceae	*Persicaria*	*Persicaria punctata*	EF653757
Eudicotyledons	Polygonaceae	*Persicaria*	*Persicaria punctata*	EU196998
Eudicotyledons	Polygonaceae	*Polygonum*	*Polygonum aviculare*	FJ395458
Eudicotyledons	Polygonaceae	*Polygonum*	*Polygonum aviculare*	EF653735
Eudicotyledons	Polygonaceae	*Polygonum*	*Polygonum aviculare*	FJ503034
Eudicotyledons	Polygonaceae	*Polygonum*	*Polygonum aviculare*	EU750484
Eudicotyledons	Polygonaceae	*Polygonum*	*Polygonum aviculare*	EU750485
Eudicotyledons	Polygonaceae	*Polygonum*	*Polygonum aviculare*	EU750486
Eudicotyledons	Polygonaceae	*Polygonum*	*Polygonum aviculare*	EU750487
Eudicotyledons	Fabaceae	*Vachellia*	*Vachellia constricta*	AF524989
Eudicotyledons	Fabaceae	*Vachellia*	*Vachellia constricta*	EU811987
Eudicotyledons	Malvaceae	*Lavatera*	*Lavatera olbia*	EF419561
Eudicotyledons	Malvaceae	*Lavatera*	*Lavatera olbia*	EF419562
Eudicotyledons	Malvaceae	*Lavatera*	*Lavatera phoenicea*	EF419644
Eudicotyledons	Malvaceae	*Lavatera*	*Lavatera phoenicea*	EF419645
Eudicotyledons	Malvaceae	*Lavatera*	*Lavatera phoenicea*	EF419646
Eudicotyledons	Malvaceae	*Lavatera*	*Lavatera trimestris*	EF419568
Eudicotyledons	Malvaceae	*Lavatera*	*Lavatera trimestris*	EF419569
Eudicotyledons	Malvaceae	*Lavatera*	*Lavatera trimestris*	EF419570
Eudicotyledons	Malvaceae	*Lavatera*	*Lavatera trimestris*	EF419571
Eudicotyledons	Primulaceae	*Primula*	*Primula bulleyana*	HM018480
Eudicotyledons	Primulaceae	*Primula*	*Primula bulleyana*	HM018481
Eudicotyledons	Primulaceae	*Primula*	*Primula bulleyana*	HM018482
Eudicotyledons	Primulaceae	*Primula*	*Primula bulleyana*	HM018483
Eudicotyledons	Primulaceae	*Primula*	*Primula bulleyana*	HM018484
Eudicotyledons	Theaceae	*Stewartia*	*Stewartia ovata*	HM100567
Eudicotyledons	Theaceae	*Stewartia*	*Stewartia ovata*	HM100568
Eudicotyledons	Theaceae	*Stewartia*	*Stewartia ovata*	HM100569
Eudicotyledons	Theaceae	*Stewartia*	*Stewartia ovata*	HM100570
Eudicotyledons	Apocynaceae	*Vincetoxicum*	*Vincetoxicum atratum*	GQ435173
Eudicotyledons	Apocynaceae	*Vincetoxicum*	*Vincetoxicum atratum*	AB109148

Eudicotyledons	Primulaceae	*Primula*	*Primula secundiflora*	HM018499
Eudicotyledons	Primulaceae	*Primula*	*Primula secundiflora*	HM018500
Eudicotyledons	Primulaceae	*Primula*	*Primula secundiflora*	HM018501
Eudicotyledons	Primulaceae	*Primula*	*Primula secundiflora*	HM018502
Eudicotyledons	Primulaceae	*Primula*	*Primula secundiflora*	HM018503
Eudicotyledons	Primulaceae	*Primula*	*Primula secundiflora*	HM018504
Eudicotyledons	Primulaceae	*Primula*	*Primula secundiflora*	HM018505
Eudicotyledons	Primulaceae	*Primula*	*Primula secundiflora*	HM018506
Eudicotyledons	Primulaceae	*Primula*	*Primula secundiflora*	HM018507
Eudicotyledons	Primulaceae	*Primula*	*Primula secundiflora*	HM018508
Eudicotyledons	Primulaceae	*Primula*	*Primula secundiflora*	HM018509
Eudicotyledons	Primulaceae	*Primula*	*Primula secundiflora*	HM018510
Eudicotyledons	Primulaceae	*Primula*	*Primula secundiflora*	HM018511
Eudicotyledons	Fabaceae	*Caesalpinia*	*Caesalpinia pannosa*	DQ501959
Eudicotyledons	Fabaceae	*Caesalpinia*	*Caesalpinia pannosa*	DQ208844
Eudicotyledons	Fabaceae	*Caesalpinia*	*Caesalpinia pannosa*	DQ208845
Eudicotyledons	Fabaceae	*Caesalpinia*	*Caesalpinia pannosa*	DQ208846
Eudicotyledons	Fabaceae	*Caesalpinia*	*Caesalpinia pannosa*	DQ208847
Eudicotyledons	Fabaceae	*Caesalpinia*	*Caesalpinia pannosa*	DQ208848
Eudicotyledons	Fabaceae	*Caesalpinia*	*Caesalpinia pannosa*	DQ208849
Eudicotyledons	Fabaceae	*Caesalpinia*	*Caesalpinia pannosa*	DQ208850
Eudicotyledons	Fabaceae	*Caesalpinia*	*Caesalpinia pannosa*	DQ208851
Eudicotyledons	Fabaceae	*Caesalpinia*	*Caesalpinia pannosa*	DQ208852
Eudicotyledons	Fabaceae	*Caesalpinia*	*Caesalpinia pannosa*	DQ208853
Eudicotyledons	Fabaceae	*Caesalpinia*	*Caesalpinia pannosa*	DQ208854
Eudicotyledons	Fabaceae	*Caesalpinia*	*Caesalpinia pannosa*	DQ208855
Eudicotyledons	Fabaceae	*Senna*	*Senna hirsuta*	JF838362
Eudicotyledons	Fabaceae	*Senna*	*Senna hirsuta*	HQ161764
Eudicotyledons	Primulaceae	*Primula*	*Primula poissonii*	HM018485
Eudicotyledons	Primulaceae	*Primula*	*Primula poissonii*	HM018486
Eudicotyledons	Primulaceae	*Primula*	*Primula poissonii*	HM018487
Eudicotyledons	Primulaceae	*Primula*	*Primula poissonii*	HM018488
Eudicotyledons	Primulaceae	*Primula*	*Primula poissonii*	HM018489
Eudicotyledons	Primulaceae	*Primula*	*Primula poissonii*	HM018490
Eudicotyledons	Primulaceae	*Primula*	*Primula poissonii*	HM018491
Eudicotyledons	Primulaceae	*Primula*	*Primula poissonii*	HM018492
Eudicotyledons	Primulaceae	*Primula*	*Primula poissonii*	HM018493
Eudicotyledons	Primulaceae	*Primula*	*Primula poissonii*	HM018494
Eudicotyledons	Primulaceae	*Primula*	*Primula poissonii*	HM018495
Eudicotyledons	Primulaceae	*Primula*	*Primula poissonii*	HM018496
Eudicotyledons	Primulaceae	*Primula*	*Primula poissonii*	HM018497
Eudicotyledons	Primulaceae	*Primula*	*Primula poissonii*	HM018498
Eudicotyledons	Primulaceae	*Lysimachia*	*Lysimachia clethroides*	JN045306
Eudicotyledons	Primulaceae	*Lysimachia*	*Lysimachia clethroides*	JN045307
Eudicotyledons	Primulaceae	*Lysimachia*	*Lysimachia clethroides*	JN045308
Eudicotyledons	Primulaceae	*Lysimachia*	*Lysimachia clethroides*	JN045309
Eudicotyledons	Primulaceae	*Lysimachia*	*Lysimachia clethroides*	JN045310
Eudicotyledons	Primulaceae	*Primula*	*Primula obconica*	JN046547
Eudicotyledons	Primulaceae	*Primula*	*Primula obconica*	JN046548
Eudicotyledons	Primulaceae	*Primula*	*Primula obconica*	JN046549
Eudicotyledons	Primulaceae	*Primula*	*Primula sinensis*	JN046582
Eudicotyledons	Primulaceae	*Primula*	*Primula sinensis*	JN046583
Eudicotyledons	Primulaceae	*Primula*	*Primula sinensis*	JN046584

Eudicotyledons	Apiaceae	*Osmorhiza*	*Osmorhiza berteroi*	JN045641
Eudicotyledons	Apiaceae	*Osmorhiza*	*Osmorhiza berteroi*	JN045642
Eudicotyledons	Apiaceae	*Osmorhiza*	*Osmorhiza berteroi*	JN045643
Eudicotyledons	Apiaceae	*Osmorhiza*	*Osmorhiza berteroi*	JN045644
Eudicotyledons	Apiaceae	*Osmorhiza*	*Osmorhiza berteroi*	JN045645
Eudicotyledons	Apiaceae	*Osmorhiza*	*Osmorhiza berteroi*	JN045646
Eudicotyledons	Apiaceae	*Osmorhiza*	*Osmorhiza berteroi*	JN045647
Eudicotyledons	Apiaceae	*Osmorhiza*	*Osmorhiza glabrata*	JN045656
Eudicotyledons	Apiaceae	*Osmorhiza*	*Osmorhiza glabrata*	JN045657
Eudicotyledons	Apiaceae	*Osmorhiza*	*Osmorhiza glabrata*	JN045658
Eudicotyledons	Apiaceae	*Osmorhiza*	*Osmorhiza glabrata*	JN045659
Eudicotyledons	Primulaceae	*Primula*	*Primula bellidifolia*	JN046474
Eudicotyledons	Primulaceae	*Primula*	*Primula bellidifolia*	JN046475
Eudicotyledons	Primulaceae	*Primula*	*Primula bellidifolia*	JN046476
Eudicotyledons	Primulaceae	*Primula*	*Primula calderiana*	JN046486
Eudicotyledons	Primulaceae	*Primula*	*Primula calderiana*	JN046487
Eudicotyledons	Primulaceae	*Primula*	*Primula calderiana*	JN046488
Eudicotyledons	Primulaceae	*Primula*	*Primula calderiana*	JN046489
Eudicotyledons	Primulaceae	*Primula*	*Primula chungensis*	HM018470
Eudicotyledons	Primulaceae	*Primula*	*Primula chungensis*	HM018471
Eudicotyledons	Primulaceae	*Primula*	*Primula denticulata*	JN046504
Eudicotyledons	Primulaceae	*Primula*	*Primula denticulata*	JN046505
Eudicotyledons	Primulaceae	*Primula*	*Primula denticulata*	JN046506
Eudicotyledons	Primulaceae	*Primula*	*Primula denticulata*	JN046507
Eudicotyledons	Primulaceae	*Primula*	*Primula gemmifera*	JN046522
Eudicotyledons	Primulaceae	*Primula*	*Primula gemmifera*	JN046523
Eudicotyledons	Primulaceae	*Primula*	*Primula gemmifera*	JN046524
Eudicotyledons	Primulaceae	*Primula*	*Primula gemmifera*	JN046525
Eudicotyledons	Primulaceae	*Primula*	*Primula membranifolia*	JN046536
Eudicotyledons	Primulaceae	*Primula*	*Primula membranifolia*	JN046537
Eudicotyledons	Primulaceae	*Primula*	*Primula pulchella*	JN046564
Eudicotyledons	Primulaceae	*Primula*	*Primula pulchella*	JN046565
Eudicotyledons	Primulaceae	*Primula*	*Primula pulchella*	JN046566
Eudicotyledons	Primulaceae	*Primula*	*Primula pulchella*	JN046567
Eudicotyledons	Primulaceae	*Primula*	*Primula pulchella*	JN046568
Eudicotyledons	Primulaceae	*Primula*	*Primula septemloba*	JN046575
Eudicotyledons	Primulaceae	*Primula*	*Primula septemloba*	JN046576
Eudicotyledons	Primulaceae	*Primula*	*Primula sonchifolia*	JN046585
Eudicotyledons	Primulaceae	*Primula*	*Primula sonchifolia*	JN046586
Eudicotyledons	Primulaceae	*Primula*	*Primula sonchifolia*	JN046587
Eudicotyledons	Primulaceae	*Primula*	*Primula sonchifolia*	JN046588
Eudicotyledons	Primulaceae	*Primula*	*Primula sonchifolia*	JN046589
Eudicotyledons	Primulaceae	*Primula*	*Primula yunnanensis*	JN046600
Eudicotyledons	Primulaceae	*Primula*	*Primula yunnanensis*	JN046601
Eudicotyledons	Hamamelidaceae	*Corylopsis*	*Corylopsis glabrescens*	GU576749
Eudicotyledons	Hamamelidaceae	*Corylopsis*	*Corylopsis glabrescens*	AB237064
Eudicotyledons	Hamamelidaceae	*Corylopsis*	*Corylopsis glabrescens*	AB237069
Eudicotyledons	Hamamelidaceae	*Corylopsis*	*Corylopsis glabrescens*	AB237070
Eudicotyledons	Theaceae	*Stewartia*	*Stewartia pteropetiolata*	HM100528
Eudicotyledons	Theaceae	*Stewartia*	*Stewartia pteropetiolata*	HM100529
Eudicotyledons	Theaceae	*Stewartia*	*Stewartia villosa*	HM100523
Eudicotyledons	Theaceae	*Stewartia*	*Stewartia villosa*	HM100534
Eudicotyledons	Theaceae	*Schima*	*Schima argentea*	HM100535

Eudicotyledons	Theaceae	*Schima*	*Schima argentea*	HM100536
Eudicotyledons	Theaceae	*Stewartia*	*Stewartia malacodendron*	HM100556
Eudicotyledons	Theaceae	*Stewartia*	*Stewartia malacodendron*	HM100557
Eudicotyledons	Theaceae	*Stewartia*	*Stewartia malacodendron*	HM100558
Eudicotyledons	Theaceae	*Stewartia*	*Stewartia malacodendron*	HM100559
Eudicotyledons	Theaceae	*Stewartia*	*Stewartia monadelpha*	HM100560
Eudicotyledons	Theaceae	*Stewartia*	*Stewartia monadelpha*	HM100561
Eudicotyledons	Theaceae	*Stewartia*	*Stewartia monadelpha*	HM100562
Eudicotyledons	Theaceae	*Stewartia*	*Stewartia monadelpha*	HM100563
Eudicotyledons	Theaceae	*Stewartia*	*Stewartia monadelpha*	HM100564
Eudicotyledons	Theaceae	*Stewartia*	*Stewartia sinensis*	HM100547
Eudicotyledons	Theaceae	*Stewartia*	*Stewartia sinensis*	HM100548
Eudicotyledons	Theaceae	*Stewartia*	*Stewartia sinensis*	HM100549
Eudicotyledons	Theaceae	*Stewartia*	*Stewartia sinensis*	HM100550
Eudicotyledons	Theaceae	*Stewartia*	*Stewartia sinensis*	HM100551
Eudicotyledons	Theaceae	*Stewartia*	*Stewartia sinensis*	HM100552
Eudicotyledons	Theaceae	*Stewartia*	*Stewartia sinensis*	HM100553
Eudicotyledons	Theaceae	*Stewartia*	*Stewartia sinensis*	HM100554
Eudicotyledons	Theaceae	*Stewartia*	*Stewartia sinensis*	HM100555
Eudicotyledons	Theaceae	*Stewartia*	*Stewartia sinensis*	HM100566
Eudicotyledons	Theaceae	*Stewartia*	*Stewartia sinensis*	HM100588
Eudicotyledons	Theaceae	*Stewartia*	*Stewartia sinensis*	HM100589
Eudicotyledons	Theaceae	*Stewartia*	*Stewartia rostrata*	HM100576
Eudicotyledons	Theaceae	*Stewartia*	*Stewartia rostrata*	HM100577
Eudicotyledons	Theaceae	*Stewartia*	*Stewartia rostrata*	HM100578
Eudicotyledons	Theaceae	*Stewartia*	*Stewartia rostrata*	HM100579
Eudicotyledons	Theaceae	*Stewartia*	*Stewartia rostrata*	HM100586
Eudicotyledons	Primulaceae	*Primula*	*Primula alpicola*	JN046461
Eudicotyledons	Primulaceae	*Primula*	*Primula alpicola*	JN046462
Eudicotyledons	Primulaceae	*Primula*	*Primula alpicola*	JN046463
Eudicotyledons	Primulaceae	*Primula*	*Primula alpicola*	JN046464
Eudicotyledons	Primulaceae	*Primula*	*Primula alpicola*	JN046465
Eudicotyledons	Apocynaceae	*Fockea*	*Fockea multiflora*	AM231766
Eudicotyledons	Apocynaceae	*Fockea*	*Fockea multiflora*	AM231767
Eudicotyledons	Apocynaceae	*Fockea*	*Fockea multiflora*	AM231768
Eudicotyledons	Orobanchaceae	*Castilleja*	*Castilleja sulphurea*	FJ765709
Eudicotyledons	Orobanchaceae	*Castilleja*	*Castilleja sulphurea*	FJ765710
Eudicotyledons	Orobanchaceae	*Castilleja*	*Castilleja sulphurea*	FJ765711
Eudicotyledons	Orobanchaceae	*Castilleja*	*Castilleja sulphurea*	FJ765712
Eudicotyledons	Orobanchaceae	*Castilleja*	*Castilleja sulphurea*	FJ765713
Eudicotyledons	Orobanchaceae	*Castilleja*	*Castilleja sulphurea*	FJ765714
Eudicotyledons	Orobanchaceae	*Castilleja*	*Castilleja sulphurea*	FJ765715
Eudicotyledons	Orobanchaceae	*Castilleja*	*Castilleja sulphurea*	FJ765716
Eudicotyledons	Orobanchaceae	*Castilleja*	*Castilleja sulphurea*	FJ765717
Eudicotyledons	Orobanchaceae	*Castilleja*	*Castilleja sulphurea*	FJ765718
Eudicotyledons	Orobanchaceae	*Castilleja*	*Castilleja sulphurea*	FJ765719
Eudicotyledons	Orobanchaceae	*Castilleja*	*Castilleja sulphurea*	FJ765720
Eudicotyledons	Orobanchaceae	*Castilleja*	*Castilleja sulphurea*	FJ765721
Eudicotyledons	Orobanchaceae	*Castilleja*	*Castilleja sulphurea*	FJ765722
Eudicotyledons	Orobanchaceae	*Castilleja*	*Castilleja sulphurea*	FJ765723
Eudicotyledons	Orobanchaceae	*Castilleja*	*Castilleja sulphurea*	FJ765724
Eudicotyledons	Orobanchaceae	*Castilleja*	*Castilleja sulphurea*	FJ765725
Eudicotyledons	Fabaceae	*Caesalpinia*	*Caesalpinia hintonii*	DQ208809

Eudicotyledons	Fabaceae	*Caesalpinia*	*Caesalpinia hintonii*	DQ208810
Eudicotyledons	Fabaceae	*Caesalpinia*	*Caesalpinia hintonii*	DQ208811
Eudicotyledons	Fabaceae	*Caesalpinia*	*Caesalpinia hintonii*	DQ208812
Eudicotyledons	Fabaceae	*Caesalpinia*	*Caesalpinia hintonii*	DQ208813
Eudicotyledons	Fabaceae	*Caesalpinia*	*Caesalpinia hintonii*	DQ208814
Eudicotyledons	Fabaceae	*Caesalpinia*	*Caesalpinia hintonii*	DQ208815
Eudicotyledons	Fabaceae	*Caesalpinia*	*Caesalpinia hintonii*	DQ208816
Eudicotyledons	Fabaceae	*Caesalpinia*	*Caesalpinia hintonii*	DQ208817
Eudicotyledons	Fabaceae	*Caesalpinia*	*Caesalpinia hintonii*	DQ208818
Eudicotyledons	Fabaceae	*Caesalpinia*	*Caesalpinia hintonii*	DQ208819
Eudicotyledons	Fabaceae	*Caesalpinia*	*Caesalpinia hintonii*	DQ208820
Eudicotyledons	Fabaceae	*Caesalpinia*	*Caesalpinia hintonii*	DQ208821
Eudicotyledons	Fabaceae	*Caesalpinia*	*Caesalpinia hintonii*	DQ208822
Eudicotyledons	Fabaceae	*Caesalpinia*	*Caesalpinia hintonii*	DQ208823
Eudicotyledons	Fabaceae	*Caesalpinia*	*Caesalpinia hintonii*	DQ208824
Eudicotyledons	Fabaceae	*Schizolobium*	*Schizolobium amazonicum*	FJ668605
Eudicotyledons	Fabaceae	*Schizolobium*	*Schizolobium amazonicum*	FJ668608
Eudicotyledons	Fabaceae	*Schizolobium*	*Schizolobium amazonicum*	FJ668609
Eudicotyledons	Elaeagnaceae	*Hippophae*	*Hippophae tibetana*	JN044914
Eudicotyledons	Elaeagnaceae	*Hippophae*	*Hippophae tibetana*	JN044915
Eudicotyledons	Elaeagnaceae	*Hippophae*	*Hippophae tibetana*	JN044916
Eudicotyledons	Elaeagnaceae	*Hippophae*	*Hippophae tibetana*	JN044917
Eudicotyledons	Elaeagnaceae	*Hippophae*	*Hippophae tibetana*	JN044918
Eudicotyledons	Theaceae	*Schima*	*Schima khasiana*	HM100537
Eudicotyledons	Theaceae	*Schima*	*Schima khasiana*	HM100538
Eudicotyledons	Fabaceae	*Mariosousa*	*Mariosousa coulteri*	AF525008
Eudicotyledons	Fabaceae	*Mariosousa*	*Mariosousa coulteri*	EU811993
Eudicotyledons	Fabaceae	*Mariosousa*	*Mariosousa dolichostachya*	AF525009
Eudicotyledons	Fabaceae	*Mariosousa*	*Mariosousa dolichostachya*	EU811992
Eudicotyledons	Lamiaceae	*Vitex*	*Vitex trifolia*	JQ319675
Eudicotyledons	Lamiaceae	*Vitex*	*Vitex trifolia*	GQ435191
Eudicotyledons	Asteraceae	*Raoulia*	*Raoulia eximia*	AY611207
Eudicotyledons	Asteraceae	*Raoulia*	*Raoulia eximia*	AY611208
Eudicotyledons	Asteraceae	*Raoulia*	*Raoulia eximia*	AY611209
Eudicotyledons	Asteraceae	*Raoulia*	*Raoulia eximia*	AY611210
Eudicotyledons	Asteraceae	*Hypochaeris*	*Hypochaeris incana*	FM994954
Eudicotyledons	Asteraceae	*Hypochaeris*	*Hypochaeris incana*	FM994955
Eudicotyledons	Asteraceae	*Hypochaeris*	*Hypochaeris incana*	FM994956
Eudicotyledons	Asteraceae	*Hypochaeris*	*Hypochaeris incana*	FM994957
Eudicotyledons	Asteraceae	*Hypochaeris*	*Hypochaeris incana*	FM994958
Eudicotyledons	Asteraceae	*Hypochaeris*	*Hypochaeris incana*	FM994959
Eudicotyledons	Asteraceae	*Hypochaeris*	*Hypochaeris incana*	FM994960
Eudicotyledons	Asteraceae	*Hypochaeris*	*Hypochaeris incana*	FM994961
Eudicotyledons	Asteraceae	*Hypochaeris*	*Hypochaeris incana*	FM994962
Eudicotyledons	Asteraceae	*Hypochaeris*	*Hypochaeris incana*	FM994963
Eudicotyledons	Asteraceae	*Hypochaeris*	*Hypochaeris incana*	FM994964
Eudicotyledons	Asteraceae	*Hypochaeris*	*Hypochaeris incana*	FM994965
Eudicotyledons	Asteraceae	*Hypochaeris*	*Hypochaeris incana*	FM994966
Eudicotyledons	Asteraceae	*Hypochaeris*	*Hypochaeris incana*	FM994967
Eudicotyledons	Asteraceae	*Hypochaeris*	*Hypochaeris incana*	FM994968
Eudicotyledons	Asteraceae	*Hypochaeris*	*Hypochaeris incana*	FM994969
Eudicotyledons	Asteraceae	*Hypochaeris*	*Hypochaeris incana*	FM994970
Eudicotyledons	Asteraceae	*Hypochaeris*	*Hypochaeris incana*	FM994971

Eudicotyledons	Asteraceae	*Hypochaeris*	*Hypochaeris incana*	FM994972
Eudicotyledons	Asteraceae	*Hypochaeris*	*Hypochaeris incana*	FM994973
Eudicotyledons	Asteraceae	*Hypochaeris*	*Hypochaeris incana*	FM994974
Eudicotyledons	Asteraceae	*Hypochaeris*	*Hypochaeris incana*	FM994975
Eudicotyledons	Asteraceae	*Hypochaeris*	*Hypochaeris incana*	FM994976
Eudicotyledons	Asteraceae	*Hypochaeris*	*Hypochaeris incana*	FM994977
Eudicotyledons	Asteraceae	*Hypochaeris*	*Hypochaeris incana*	FM994978
Eudicotyledons	Asteraceae	*Hypochaeris*	*Hypochaeris incana*	FM994979
Eudicotyledons	Asteraceae	*Hypochaeris*	*Hypochaeris incana*	FM994980
Eudicotyledons	Asteraceae	*Hypochaeris*	*Hypochaeris incana*	FM994981
Eudicotyledons	Asteraceae	*Hypochaeris*	*Hypochaeris incana*	FM994982
Eudicotyledons	Asteraceae	*Hypochaeris*	*Hypochaeris incana*	FM994983
Eudicotyledons	Asteraceae	*Hypochaeris*	*Hypochaeris incana*	FM994984
Eudicotyledons	Asteraceae	*Hypochaeris*	*Hypochaeris incana*	FM994985
Eudicotyledons	Asteraceae	*Hypochaeris*	*Hypochaeris incana*	FM994986
Eudicotyledons	Asteraceae	*Hypochaeris*	*Hypochaeris incana*	FM994987
Eudicotyledons	Asteraceae	*Hypochaeris*	*Hypochaeris incana*	FM994988
Eudicotyledons	Asteraceae	*Hypochaeris*	*Hypochaeris incana*	FM994989
Eudicotyledons	Asteraceae	*Hypochaeris*	*Hypochaeris incana*	FM994990
Eudicotyledons	Asteraceae	*Hypochaeris*	*Hypochaeris incana*	FM994991
Eudicotyledons	Asteraceae	*Hypochaeris*	*Hypochaeris incana*	FM994992
Eudicotyledons	Asteraceae	*Hypochaeris*	*Hypochaeris incana*	FM994993
Eudicotyledons	Asteraceae	*Hypochaeris*	*Hypochaeris incana*	FM994994
Eudicotyledons	Asteraceae	*Hypochaeris*	*Hypochaeris incana*	FM994995
Eudicotyledons	Asteraceae	*Hypochaeris*	*Hypochaeris incana*	FN394275
Eudicotyledons	Asteraceae	*Hypochaeris*	*Hypochaeris incana*	FN394276
Eudicotyledons	Asteraceae	*Hypochaeris*	*Hypochaeris incana*	FN394277
Eudicotyledons	Asteraceae	*Hypochaeris*	*Hypochaeris incana*	FN394278
Eudicotyledons	Asteraceae	*Hypochaeris*	*Hypochaeris incana*	FN394279
Eudicotyledons	Asteraceae	*Hypochaeris*	*Hypochaeris incana*	FN394280
Eudicotyledons	Asteraceae	*Hypochaeris*	*Hypochaeris incana*	FN394281
Eudicotyledons	Asteraceae	*Hypochaeris*	*Hypochaeris incana*	FN394282
Eudicotyledons	Asteraceae	*Hypochaeris*	*Hypochaeris incana*	FN394283
Eudicotyledons	Asteraceae	*Hypochaeris*	*Hypochaeris incana*	FN394284
Eudicotyledons	Asteraceae	*Hypochaeris*	*Hypochaeris incana*	FN394285
Eudicotyledons	Asteraceae	*Hypochaeris*	*Hypochaeris incana*	FN394286
Eudicotyledons	Asteraceae	*Hypochaeris*	*Hypochaeris incana*	FN394287
Eudicotyledons	Asteraceae	*Hypochaeris*	*Hypochaeris incana*	FN394288
Eudicotyledons	Asteraceae	*Hypochaeris*	*Hypochaeris incana*	FN394289
Eudicotyledons	Asteraceae	*Hypochaeris*	*Hypochaeris incana*	FN394290
Eudicotyledons	Asteraceae	*Hypochaeris*	*Hypochaeris incana*	FN394291
Eudicotyledons	Asteraceae	*Hypochaeris*	*Hypochaeris incana*	FN394292
Eudicotyledons	Asteraceae	*Hypochaeris*	*Hypochaeris incana*	FN394293
Eudicotyledons	Asteraceae	*Hypochaeris*	*Hypochaeris incana*	FN394294
Eudicotyledons	Asteraceae	*Hypochaeris*	*Hypochaeris incana*	FN394295
Eudicotyledons	Asteraceae	*Hypochaeris*	*Hypochaeris incana*	FN394296
Eudicotyledons	Asteraceae	*Hypochaeris*	*Hypochaeris incana*	FN394297
Eudicotyledons	Asteraceae	*Hypochaeris*	*Hypochaeris incana*	FN394298
Eudicotyledons	Asteraceae	*Hypochaeris*	*Hypochaeris incana*	FN394299
Eudicotyledons	Asteraceae	*Hypochaeris*	*Hypochaeris incana*	FN394300
Eudicotyledons	Asteraceae	*Hypochaeris*	*Hypochaeris incana*	FN394301
Eudicotyledons	Asteraceae	*Hypochaeris*	*Hypochaeris incana*	FN394302
Eudicotyledons	Asteraceae	*Hypochaeris*	*Hypochaeris incana*	FN394303

Eudicotyledons	Asteraceae	*Hypochaeris*	*Hypochaeris incana*	FN394304
Eudicotyledons	Asteraceae	*Hypochaeris*	*Hypochaeris incana*	FN394305
Eudicotyledons	Asteraceae	*Hypochaeris*	*Hypochaeris incana*	FN394306
Eudicotyledons	Asteraceae	*Hypochaeris*	*Hypochaeris incana*	FN394307
Eudicotyledons	Asteraceae	*Hypochaeris*	*Hypochaeris tenuifolia*	FM994947
Eudicotyledons	Asteraceae	*Hypochaeris*	*Hypochaeris tenuifolia*	FM994948
Eudicotyledons	Asteraceae	*Hypochaeris*	*Hypochaeris tenuifolia*	FM994949
Eudicotyledons	Asteraceae	*Hypochaeris*	*Hypochaeris tenuifolia*	FM994950
Eudicotyledons	Asteraceae	*Hypochaeris*	*Hypochaeris tenuifolia*	FM994951
Eudicotyledons	Asteraceae	*Hypochaeris*	*Hypochaeris tenuifolia*	FM994952
Eudicotyledons	Asteraceae	*Hypochaeris*	*Hypochaeris tenuifolia*	FM994953
Eudicotyledons	Primulaceae	*Lysimachia*	*Lysimachia christinae*	GQ434938
Eudicotyledons	Primulaceae	*Lysimachia*	*Lysimachia christinae*	JN045301
Eudicotyledons	Primulaceae	*Lysimachia*	*Lysimachia christinae*	JN045302
Eudicotyledons	Primulaceae	*Lysimachia*	*Lysimachia christinae*	JN045303
Eudicotyledons	Primulaceae	*Lysimachia*	*Lysimachia christinae*	JN045304
Eudicotyledons	Primulaceae	*Lysimachia*	*Lysimachia christinae*	JN045305
Eudicotyledons	Primulaceae	*Lysimachia*	*Lysimachia ciliata*	HQ596760
Eudicotyledons	Primulaceae	*Lysimachia*	*Lysimachia ciliata*	DQ006219
Eudicotyledons	Primulaceae	*Lysimachia*	*Lysimachia crispidens*	JN045316
Eudicotyledons	Primulaceae	*Lysimachia*	*Lysimachia crispidens*	JN045317
Eudicotyledons	Primulaceae	*Lysimachia*	*Lysimachia decurrens*	GQ257964
Eudicotyledons	Primulaceae	*Lysimachia*	*Lysimachia decurrens*	JN045318
Eudicotyledons	Primulaceae	*Lysimachia*	*Lysimachia decurrens*	JN045319
Eudicotyledons	Primulaceae	*Lysimachia*	*Lysimachia fistulosa*	JN045327
Eudicotyledons	Primulaceae	*Lysimachia*	*Lysimachia fistulosa*	JN045328
Eudicotyledons	Primulaceae	*Lysimachia*	*Lysimachia fistulosa*	JN045329
Eudicotyledons	Primulaceae	*Lysimachia*	*Lysimachia fistulosa*	JN045330
Eudicotyledons	Primulaceae	*Lysimachia*	*Lysimachia fordiana*	JN045331
Eudicotyledons	Primulaceae	*Lysimachia*	*Lysimachia fordiana*	JN045332
Eudicotyledons	Primulaceae	*Lysimachia*	*Lysimachia fortunei*	JN045333
Eudicotyledons	Primulaceae	*Lysimachia*	*Lysimachia fortunei*	JN045334
Eudicotyledons	Primulaceae	*Lysimachia*	*Lysimachia fortunei*	JN045335
Eudicotyledons	Primulaceae	*Lysimachia*	*Lysimachia fortunei*	JN045336
Eudicotyledons	Primulaceae	*Lysimachia*	*Lysimachia fortunei*	JN045337
Eudicotyledons	Primulaceae	*Lysimachia*	*Lysimachia glutinosa*	GQ257967
Eudicotyledons	Primulaceae	*Lysimachia*	*Lysimachia glutinosa*	GQ257968
Eudicotyledons	Primulaceae	*Lysimachia*	*Lysimachia grammica*	JN045340
Eudicotyledons	Primulaceae	*Lysimachia*	*Lysimachia grammica*	JN045341
Eudicotyledons	Primulaceae	*Lysimachia*	*Lysimachia heterogenea*	JN045353
Eudicotyledons	Primulaceae	*Lysimachia*	*Lysimachia heterogenea*	JN045354
Eudicotyledons	Primulaceae	*Lysimachia*	*Lysimachia heterogenea*	JN045355
Eudicotyledons	Primulaceae	*Lysimachia*	*Lysimachia iniki*	GQ257970
Eudicotyledons	Primulaceae	*Lysimachia*	*Lysimachia iniki*	GQ257971
Eudicotyledons	Primulaceae	*Lysimachia*	*Lysimachia insignis*	JN045356
Eudicotyledons	Primulaceae	*Lysimachia*	*Lysimachia insignis*	JN045357
Eudicotyledons	Primulaceae	*Lysimachia*	*Lysimachia insignis*	JN045358
Eudicotyledons	Primulaceae	*Lysimachia*	*Lysimachia insignis*	JN045359
Eudicotyledons	Primulaceae	*Lysimachia*	*Lysimachia insignis*	JN045360
Eudicotyledons	Primulaceae	*Lysimachia*	*Lysimachia insignis*	JN045361
Eudicotyledons	Primulaceae	*Lysimachia*	*Lysimachia laxa*	JN045364
Eudicotyledons	Primulaceae	*Lysimachia*	*Lysimachia laxa*	JN045365
Eudicotyledons	Primulaceae	*Lysimachia*	*Lysimachia lobelioides*	GQ257975

Eudicotyledons	Primulaceae	*Lysimachia*	*Lysimachia lobelioides*	JN045366
Eudicotyledons	Primulaceae	*Lysimachia*	*Lysimachia lobelioides*	JN045367
Eudicotyledons	Primulaceae	*Lysimachia*	*Lysimachia longipes*	JN045368
Eudicotyledons	Primulaceae	*Lysimachia*	*Lysimachia longipes*	JN045369
Eudicotyledons	Primulaceae	*Lysimachia*	*Lysimachia longipes*	JN045370
Eudicotyledons	Primulaceae	*Lysimachia*	*Lysimachia melampyroides*	JN045371
Eudicotyledons	Primulaceae	*Lysimachia*	*Lysimachia melampyroides*	JN045372
Eudicotyledons	Primulaceae	*Lysimachia*	*Lysimachia omeiensis*	JN045373
Eudicotyledons	Primulaceae	*Lysimachia*	*Lysimachia omeiensis*	JN045374
Eudicotyledons	Primulaceae	*Lysimachia*	*Lysimachia paridiformis*	JN045375
Eudicotyledons	Primulaceae	*Lysimachia*	*Lysimachia paridiformis*	JN045376
Eudicotyledons	Primulaceae	*Lysimachia*	*Lysimachia paridiformis*	JN045377
Eudicotyledons	Primulaceae	*Lysimachia*	*Lysimachia paridiformis*	JN045378
Eudicotyledons	Primulaceae	*Lysimachia*	*Lysimachia paridiformis*	JN045379
Eudicotyledons	Primulaceae	*Lysimachia*	*Lysimachia phyllocephala*	JN045386
Eudicotyledons	Primulaceae	*Lysimachia*	*Lysimachia phyllocephala*	JN045387
Eudicotyledons	Primulaceae	*Lysimachia*	*Lysimachia pittosporoides*	JN045388
Eudicotyledons	Primulaceae	*Lysimachia*	*Lysimachia pittosporoides*	JN045389
Eudicotyledons	Primulaceae	*Lysimachia*	*Lysimachia rubiginosa*	JN045390
Eudicotyledons	Primulaceae	*Lysimachia*	*Lysimachia rubiginosa*	JN045391
Eudicotyledons	Primulaceae	*Lysimachia*	*Lysimachia rubiginosa*	JN045392
Eudicotyledons	Primulaceae	*Lysimachia*	*Lysimachia candida*	JN045293
Eudicotyledons	Primulaceae	*Lysimachia*	*Lysimachia candida*	JN045294
Eudicotyledons	Asteraceae	*Hypochaeris*	*Hypochaeris hookeri*	FM994941
Eudicotyledons	Asteraceae	*Hypochaeris*	*Hypochaeris hookeri*	FM994942
Eudicotyledons	Asteraceae	*Hypochaeris*	*Hypochaeris hookeri*	FM994943
Eudicotyledons	Asteraceae	*Hypochaeris*	*Hypochaeris hookeri*	FM994944
Eudicotyledons	Asteraceae	*Hypochaeris*	*Hypochaeris hookeri*	FM994945
Eudicotyledons	Asteraceae	*Hypochaeris*	*Hypochaeris hookeri*	FM994946
Eudicotyledons	Ranunculaceae	*Aconitum*	*Aconitum scaposum*	GQ337739
Eudicotyledons	Ranunculaceae	*Aconitum*	*Aconitum scaposum*	GQ337740
Eudicotyledons	Ranunculaceae	*Aconitum*	*Aconitum scaposum*	GQ337741
Eudicotyledons	Ranunculaceae	*Aconitum*	*Aconitum scaposum*	JN043766
Eudicotyledons	Ranunculaceae	*Aconitum*	*Aconitum scaposum*	JN043767
Eudicotyledons	Ranunculaceae	*Aconitum*	*Aconitum scaposum*	JN043768
Eudicotyledons	Ranunculaceae	*Aconitum*	*Aconitum scaposum*	JN043769
Eudicotyledons	Ranunculaceae	*Aconitum*	*Aconitum sinomontanum*	JN043772
Eudicotyledons	Ranunculaceae	*Aconitum*	*Aconitum sinomontanum*	JN043773
Eudicotyledons	Ranunculaceae	*Aconitum*	*Aconitum sinomontanum*	JN043774
Eudicotyledons	Ranunculaceae	*Aconitum*	*Aconitum sinomontanum*	JN043775
Eudicotyledons	Ranunculaceae	*Aconitum*	*Aconitum hemsleyanum*	GQ337729
Eudicotyledons	Ranunculaceae	*Aconitum*	*Aconitum hemsleyanum*	GQ337730
Eudicotyledons	Ranunculaceae	*Aconitum*	*Aconitum hemsleyanum*	GQ337731
Eudicotyledons	Ranunculaceae	*Aconitum*	*Aconitum hemsleyanum*	GQ337732
Eudicotyledons	Ranunculaceae	*Aconitum*	*Aconitum hemsleyanum*	GQ337733
Eudicotyledons	Ranunculaceae	*Aconitum*	*Aconitum hemsleyanum*	GQ337809
Eudicotyledons	Ranunculaceae	*Aconitum*	*Aconitum hemsleyanum*	GQ337843
Eudicotyledons	Ranunculaceae	*Aconitum*	*Aconitum gymnandrum*	FJ418149
Eudicotyledons	Ranunculaceae	*Aconitum*	*Aconitum gymnandrum*	FJ418150
Eudicotyledons	Ranunculaceae	*Aconitum*	*Aconitum gymnandrum*	FJ418151
Eudicotyledons	Ranunculaceae	*Aconitum*	*Aconitum gymnandrum*	GQ337734
Eudicotyledons	Ranunculaceae	*Aconitum*	*Aconitum gymnandrum*	GQ337735
Eudicotyledons	Ranunculaceae	*Aconitum*	*Aconitum gymnandrum*	GQ337736

Eudicotyledons	Ranunculaceae	*Delphinium*	*Delphinium forrestii*	JN044406
Eudicotyledons	Ranunculaceae	*Delphinium*	*Delphinium forrestii*	JN044407
Eudicotyledons	Amaranthaceae	*Suaeda*	*Suaeda acuminata*	JN047261
Eudicotyledons	Amaranthaceae	*Suaeda*	*Suaeda acuminata*	JN047262
Eudicotyledons	Amaranthaceae	*Suaeda*	*Suaeda acuminata*	JN047263
Eudicotyledons	Amaranthaceae	*Suaeda*	*Suaeda corniculata*	JN047264
Eudicotyledons	Amaranthaceae	*Suaeda*	*Suaeda corniculata*	JN047265
Eudicotyledons	Fabaceae	*Chamaecrista*	*Chamaecrista nictitans*	GQ248262
Eudicotyledons	Fabaceae	*Chamaecrista*	*Chamaecrista nictitans*	EF590678
Eudicotyledons	Ranunculaceae	*Aconitum*	*Aconitum vilmorinianum*	GQ337751
Eudicotyledons	Ranunculaceae	*Aconitum*	*Aconitum vilmorinianum*	GQ337752
Eudicotyledons	Ranunculaceae	*Aconitum*	*Aconitum vilmorinianum*	GQ337756
Eudicotyledons	Ranunculaceae	*Aconitum*	*Aconitum vilmorinianum*	GQ337757
Eudicotyledons	Ranunculaceae	*Aconitum*	*Aconitum vilmorinianum*	GQ337758
Eudicotyledons	Ranunculaceae	*Aconitum*	*Aconitum vilmorinianum*	GQ337759
Eudicotyledons	Ranunculaceae	*Aconitum*	*Aconitum vilmorinianum*	GQ337760
Eudicotyledons	Ranunculaceae	*Aconitum*	*Aconitum vilmorinianum*	GQ337761
Eudicotyledons	Ranunculaceae	*Aconitum*	*Aconitum vilmorinianum*	GQ337762
Eudicotyledons	Ranunculaceae	*Aconitum*	*Aconitum vilmorinianum*	GQ337763
Eudicotyledons	Ranunculaceae	*Aconitum*	*Aconitum vilmorinianum*	GQ337764
Eudicotyledons	Ranunculaceae	*Aconitum*	*Aconitum vilmorinianum*	GQ337844
Eudicotyledons	Ranunculaceae	*Aconitum*	*Aconitum vilmorinianum*	GQ337845
Eudicotyledons	Ranunculaceae	*Aconitum*	*Aconitum vilmorinianum*	GQ337846
Eudicotyledons	Ranunculaceae	*Aconitum*	*Aconitum vilmorinianum*	GQ337847
Eudicotyledons	Ranunculaceae	*Aconitum*	*Aconitum contortum*	GQ337791
Eudicotyledons	Ranunculaceae	*Aconitum*	*Aconitum contortum*	GQ337792
Eudicotyledons	Ranunculaceae	*Aconitum*	*Aconitum contortum*	GQ337793
Eudicotyledons	Ranunculaceae	*Aconitum*	*Aconitum contortum*	GQ337794
Eudicotyledons	Ranunculaceae	*Aconitum*	*Aconitum brachypodum*	GQ337726
Eudicotyledons	Ranunculaceae	*Aconitum*	*Aconitum brachypodum*	GQ337727
Eudicotyledons	Ranunculaceae	*Aconitum*	*Aconitum brachypodum*	GQ337728
Eudicotyledons	Apocynaceae	*Vincetoxicum*	*Vincetoxicum macrophyllum*	AB109154
Eudicotyledons	Apocynaceae	*Vincetoxicum*	*Vincetoxicum macrophyllum*	AB109155
Eudicotyledons	Apocynaceae	*Vincetoxicum*	*Vincetoxicum nipponicum*	AB109157
Eudicotyledons	Apocynaceae	*Vincetoxicum*	*Vincetoxicum nipponicum*	AB109158
Eudicotyledons	Lamiaceae	*Thymus*	*Thymus quinquecostatus*	AY281310
Eudicotyledons	Lamiaceae	*Thymus*	*Thymus quinquecostatus*	AY281311
Eudicotyledons	Lamiaceae	*Thymus*	*Thymus quinquecostatus*	AY281312
Eudicotyledons	Lamiaceae	*Thymus*	*Thymus quinquecostatus*	AY281313
Eudicotyledons	Lamiaceae	*Thymus*	*Thymus quinquecostatus*	AY281314
Eudicotyledons	Lamiaceae	*Thymus*	*Thymus quinquecostatus*	AY281315
Eudicotyledons	Lamiaceae	*Thymus*	*Thymus quinquecostatus*	AY443427
Eudicotyledons	Lamiaceae	*Thymus*	*Thymus quinquecostatus*	AY443428
Eudicotyledons	Lamiaceae	*Thymus*	*Thymus quinquecostatus*	AY443429
Eudicotyledons	Lamiaceae	*Thymus*	*Thymus quinquecostatus*	AY443430
Eudicotyledons	Lamiaceae	*Thymus*	*Thymus quinquecostatus*	AY443431
Eudicotyledons	Lamiaceae	*Thymus*	*Thymus quinquecostatus*	HM590114
Eudicotyledons	Lamiaceae	*Thymus*	*Thymus magnus*	AY281316
Eudicotyledons	Lamiaceae	*Thymus*	*Thymus magnus*	AY281317
Eudicotyledons	Lamiaceae	*Thymus*	*Thymus magnus*	AY443432
Eudicotyledons	Lamiaceae	*Thymus*	*Thymus magnus*	AY443433
Eudicotyledons	Araliaceae	*Dendropanax*	*Dendropanax burmanicus*	GU054806
Eudicotyledons	Araliaceae	*Dendropanax*	*Dendropanax burmanicus*	GU054876

Eudicotyledons	Ranunculaceae	*Aconitum*	*Aconitum kusnezoffii*	GQ337723
Eudicotyledons	Ranunculaceae	*Aconitum*	*Aconitum kusnezoffii*	GQ337724
Eudicotyledons	Ranunculaceae	*Aconitum*	*Aconitum kusnezoffii*	GQ337725
Eudicotyledons	Ranunculaceae	*Aconitum*	*Aconitum kusnezoffii*	GQ337789
Eudicotyledons	Ranunculaceae	*Aconitum*	*Aconitum kusnezoffii*	GQ337790
Eudicotyledons	Ranunculaceae	*Aconitum*	*Aconitum kusnezoffii*	GQ337798
Eudicotyledons	Ranunculaceae	*Aconitum*	*Aconitum kusnezoffii*	GQ337799
Eudicotyledons	Ranunculaceae	*Aconitum*	*Aconitum kusnezoffii*	GQ337812
Eudicotyledons	Ranunculaceae	*Aconitum*	*Aconitum kusnezoffii*	GQ337813
Eudicotyledons	Ranunculaceae	*Aconitum*	*Aconitum kusnezoffii*	GQ337814
Eudicotyledons	Ranunculaceae	*Aconitum*	*Aconitum kusnezoffii*	GQ337836
Eudicotyledons	Ranunculaceae	*Aconitum*	*Aconitum kusnezoffii*	GQ337837
Eudicotyledons	Ranunculaceae	*Aconitum*	*Aconitum kusnezoffii*	GQ337838
Eudicotyledons	Ranunculaceae	*Aconitum*	*Aconitum kusnezoffii*	GQ337839
Eudicotyledons	Ranunculaceae	*Aconitum*	*Aconitum kusnezoffii*	GQ337848
Eudicotyledons	Ranunculaceae	*Aconitum*	*Aconitum kusnezoffii*	GQ337849
Eudicotyledons	Ranunculaceae	*Aconitum*	*Aconitum kusnezoffii*	FJ821181
Eudicotyledons	Ranunculaceae	*Aconitum*	*Aconitum kusnezoffii*	FJ821182
Eudicotyledons	Ranunculaceae	*Aconitum*	*Aconitum kusnezoffii*	FJ821183
Eudicotyledons	Symplocaceae	*Symplocos*	*Symplocos cochinchinensis*	HQ415519
Eudicotyledons	Symplocaceae	*Symplocos*	*Symplocos cochinchinensis*	AB115382
Eudicotyledons	Symplocaceae	*Symplocos*	*Symplocos lancifolia*	HQ415516
Eudicotyledons	Symplocaceae	*Symplocos*	*Symplocos lancifolia*	AB115386
Eudicotyledons	Symplocaceae	*Symplocos*	*Symplocos stellaris*	HQ427079
Eudicotyledons	Symplocaceae	*Symplocos*	*Symplocos stellaris*	AB115389
Eudicotyledons	Araliaceae	*Dendropanax*	*Dendropanax chevalieri*	GU054858
Eudicotyledons	Araliaceae	*Dendropanax*	*Dendropanax chevalieri*	GU054878
Eudicotyledons	Symplocaceae	*Symplocos*	*Symplocos laurina*	EU769757
Eudicotyledons	Symplocaceae	*Symplocos*	*Symplocos laurina*	EU769758
Eudicotyledons	Symplocaceae	*Symplocos*	*Symplocos laurina*	EU769759
Eudicotyledons	Symplocaceae	*Symplocos*	*Symplocos laurina*	EU769760
Eudicotyledons	Symplocaceae	*Symplocos*	*Symplocos laurina*	EU769761
Eudicotyledons	Symplocaceae	*Symplocos*	*Symplocos laurina*	EU769762
Eudicotyledons	Symplocaceae	*Symplocos*	*Symplocos laurina*	EU769763
Eudicotyledons	Symplocaceae	*Symplocos*	*Symplocos laurina*	EU769764
Eudicotyledons	Symplocaceae	*Symplocos*	*Symplocos laurina*	EU769765
Eudicotyledons	Symplocaceae	*Symplocos*	*Symplocos laurina*	EU769766
Eudicotyledons	Symplocaceae	*Symplocos*	*Symplocos laurina*	EU769767
Eudicotyledons	Symplocaceae	*Symplocos*	*Symplocos laurina*	EU769768
Eudicotyledons	Symplocaceae	*Symplocos*	*Symplocos laurina*	EU769769
Eudicotyledons	Symplocaceae	*Symplocos*	*Symplocos laurina*	EU769770
Eudicotyledons	Symplocaceae	*Symplocos*	*Symplocos laurina*	EU769771
Eudicotyledons	Symplocaceae	*Symplocos*	*Symplocos laurina*	EU769772
Eudicotyledons	Symplocaceae	*Symplocos*	*Symplocos laurina*	EU769773
Eudicotyledons	Symplocaceae	*Symplocos*	*Symplocos laurina*	EU769774
Eudicotyledons	Symplocaceae	*Symplocos*	*Symplocos laurina*	EU769775
Eudicotyledons	Symplocaceae	*Symplocos*	*Symplocos laurina*	EU769776
Eudicotyledons	Symplocaceae	*Symplocos*	*Symplocos laurina*	EU769777
Eudicotyledons	Symplocaceae	*Symplocos*	*Symplocos laurina*	EU769778
Eudicotyledons	Symplocaceae	*Symplocos*	*Symplocos laurina*	EU769779
Eudicotyledons	Symplocaceae	*Symplocos*	*Symplocos laurina*	EU769780
Eudicotyledons	Symplocaceae	*Symplocos*	*Symplocos laurina*	EU769781
Eudicotyledons	Symplocaceae	*Symplocos*	*Symplocos laurina*	EU769782

Eudicotyledons	Symplocaceae	*Symplocos*	*Symplocos laurina*	EU769783
Eudicotyledons	Symplocaceae	*Symplocos*	*Symplocos laurina*	EU769784
Eudicotyledons	Symplocaceae	*Symplocos*	*Symplocos laurina*	EU769785
Eudicotyledons	Symplocaceae	*Symplocos*	*Symplocos laurina*	EU769786
Eudicotyledons	Symplocaceae	*Symplocos*	*Symplocos laurina*	EU769787
Eudicotyledons	Symplocaceae	*Symplocos*	*Symplocos laurina*	EU769788
Eudicotyledons	Symplocaceae	*Symplocos*	*Symplocos laurina*	EU769789
Eudicotyledons	Symplocaceae	*Symplocos*	*Symplocos laurina*	EU769790
Eudicotyledons	Symplocaceae	*Symplocos*	*Symplocos laurina*	EU769791
Eudicotyledons	Symplocaceae	*Symplocos*	*Symplocos laurina*	EU769792
Eudicotyledons	Symplocaceae	*Symplocos*	*Symplocos laurina*	EU769793
Eudicotyledons	Symplocaceae	*Symplocos*	*Symplocos laurina*	EU769794
Eudicotyledons	Symplocaceae	*Symplocos*	*Symplocos laurina*	EU769795
Eudicotyledons	Symplocaceae	*Symplocos*	*Symplocos laurina*	EU769796
Eudicotyledons	Symplocaceae	*Symplocos*	*Symplocos laurina*	EU769797
Eudicotyledons	Symplocaceae	*Symplocos*	*Symplocos laurina*	EU769798
Eudicotyledons	Symplocaceae	*Symplocos*	*Symplocos laurina*	EU769799
Eudicotyledons	Symplocaceae	*Symplocos*	*Symplocos laurina*	EU769800
Eudicotyledons	Symplocaceae	*Symplocos*	*Symplocos laurina*	EU769801
Eudicotyledons	Symplocaceae	*Symplocos*	*Symplocos laurina*	EU769802
Eudicotyledons	Symplocaceae	*Symplocos*	*Symplocos laurina*	EU769803
Eudicotyledons	Symplocaceae	*Symplocos*	*Symplocos laurina*	EU769804
Eudicotyledons	Symplocaceae	*Symplocos*	*Symplocos laurina*	EU769805
Eudicotyledons	Symplocaceae	*Symplocos*	*Symplocos laurina*	EU769806
Eudicotyledons	Symplocaceae	*Symplocos*	*Symplocos laurina*	EU769807
Eudicotyledons	Symplocaceae	*Symplocos*	*Symplocos laurina*	EU769808
Eudicotyledons	Symplocaceae	*Symplocos*	*Symplocos laurina*	EU769809
Eudicotyledons	Symplocaceae	*Symplocos*	*Symplocos laurina*	EU769810
Eudicotyledons	Symplocaceae	*Symplocos*	*Symplocos laurina*	EU769811
Eudicotyledons	Symplocaceae	*Symplocos*	*Symplocos laurina*	EU769812
Eudicotyledons	Symplocaceae	*Symplocos*	*Symplocos laurina*	EU769813
Eudicotyledons	Symplocaceae	*Symplocos*	*Symplocos laurina*	EU769814
Eudicotyledons	Symplocaceae	*Symplocos*	*Symplocos laurina*	EU769815
Eudicotyledons	Symplocaceae	*Symplocos*	*Symplocos laurina*	EU769816
Eudicotyledons	Symplocaceae	*Symplocos*	*Symplocos laurina*	EU769817
Eudicotyledons	Symplocaceae	*Symplocos*	*Symplocos laurina*	EU769818
Eudicotyledons	Symplocaceae	*Symplocos*	*Symplocos laurina*	EU769819
Eudicotyledons	Symplocaceae	*Symplocos*	*Symplocos laurina*	EU769820
Eudicotyledons	Symplocaceae	*Symplocos*	*Symplocos laurina*	EU769821
Eudicotyledons	Symplocaceae	*Symplocos*	*Symplocos laurina*	EU769822
Eudicotyledons	Symplocaceae	*Symplocos*	*Symplocos laurina*	EU769823
Eudicotyledons	Symplocaceae	*Symplocos*	*Symplocos laurina*	EU769824
Eudicotyledons	Symplocaceae	*Symplocos*	*Symplocos laurina*	EU769825
Eudicotyledons	Symplocaceae	*Symplocos*	*Symplocos laurina*	EU769826
Eudicotyledons	Symplocaceae	*Symplocos*	*Symplocos laurina*	EU769827
Eudicotyledons	Symplocaceae	*Symplocos*	*Symplocos laurina*	EU769828
Eudicotyledons	Symplocaceae	*Symplocos*	*Symplocos laurina*	EU769829
Eudicotyledons	Symplocaceae	*Symplocos*	*Symplocos laurina*	EU769830
Eudicotyledons	Symplocaceae	*Symplocos*	*Symplocos laurina*	EU769831
Eudicotyledons	Symplocaceae	*Symplocos*	*Symplocos laurina*	EU769832
Eudicotyledons	Symplocaceae	*Symplocos*	*Symplocos laurina*	EU769833
Eudicotyledons	Symplocaceae	*Symplocos*	*Symplocos laurina*	EU769834
Eudicotyledons	Symplocaceae	*Symplocos*	*Symplocos laurina*	EU769835

Eudicotyledons	Symplocaceae	*Symplocos*	*Symplocos laurina*	EU769836
Eudicotyledons	Symplocaceae	*Symplocos*	*Symplocos laurina*	EU769837
Eudicotyledons	Symplocaceae	*Symplocos*	*Symplocos laurina*	EU769838
Eudicotyledons	Symplocaceae	*Symplocos*	*Symplocos laurina*	EU769839
Eudicotyledons	Symplocaceae	*Symplocos*	*Symplocos laurina*	EU769840
Eudicotyledons	Symplocaceae	*Symplocos*	*Symplocos laurina*	EU769841
Eudicotyledons	Symplocaceae	*Symplocos*	*Symplocos laurina*	EU769842
Eudicotyledons	Symplocaceae	*Symplocos*	*Symplocos laurina*	EU769843
Eudicotyledons	Symplocaceae	*Symplocos*	*Symplocos laurina*	EU769844
Eudicotyledons	Symplocaceae	*Symplocos*	*Symplocos laurina*	EU769845
Eudicotyledons	Symplocaceae	*Symplocos*	*Symplocos laurina*	EU769846
Eudicotyledons	Symplocaceae	*Symplocos*	*Symplocos laurina*	EU769847
Eudicotyledons	Symplocaceae	*Symplocos*	*Symplocos laurina*	EU769848
Eudicotyledons	Symplocaceae	*Symplocos*	*Symplocos laurina*	EU769849
Eudicotyledons	Symplocaceae	*Symplocos*	*Symplocos laurina*	EU769850
Eudicotyledons	Symplocaceae	*Symplocos*	*Symplocos laurina*	EU769851
Eudicotyledons	Symplocaceae	*Symplocos*	*Symplocos laurina*	EU769852
Eudicotyledons	Symplocaceae	*Symplocos*	*Symplocos laurina*	EU769853
Eudicotyledons	Symplocaceae	*Symplocos*	*Symplocos laurina*	EU769854
Eudicotyledons	Symplocaceae	*Symplocos*	*Symplocos laurina*	EU769855
Eudicotyledons	Symplocaceae	*Symplocos*	*Symplocos laurina*	EU769856
Eudicotyledons	Symplocaceae	*Symplocos*	*Symplocos laurina*	EU769857
Eudicotyledons	Symplocaceae	*Symplocos*	*Symplocos laurina*	EU769858
Eudicotyledons	Symplocaceae	*Symplocos*	*Symplocos laurina*	EU769859
Eudicotyledons	Symplocaceae	*Symplocos*	*Symplocos laurina*	EU769860
Eudicotyledons	Symplocaceae	*Symplocos*	*Symplocos laurina*	EU769861
Eudicotyledons	Symplocaceae	*Symplocos*	*Symplocos laurina*	EU769862
Eudicotyledons	Symplocaceae	*Symplocos*	*Symplocos laurina*	EU769863
Eudicotyledons	Symplocaceae	*Symplocos*	*Symplocos laurina*	EU769864
Eudicotyledons	Symplocaceae	*Symplocos*	*Symplocos laurina*	EU769865
Eudicotyledons	Symplocaceae	*Symplocos*	*Symplocos laurina*	EU769866
Eudicotyledons	Symplocaceae	*Symplocos*	*Symplocos laurina*	EU769867
Eudicotyledons	Symplocaceae	*Symplocos*	*Symplocos laurina*	EU769868
Eudicotyledons	Symplocaceae	*Symplocos*	*Symplocos laurina*	EU769869
Eudicotyledons	Symplocaceae	*Symplocos*	*Symplocos laurina*	EU769870
Eudicotyledons	Symplocaceae	*Symplocos*	*Symplocos laurina*	EU769871
Eudicotyledons	Symplocaceae	*Symplocos*	*Symplocos laurina*	EU769872
Eudicotyledons	Symplocaceae	*Symplocos*	*Symplocos laurina*	EU769873
Eudicotyledons	Symplocaceae	*Symplocos*	*Symplocos laurina*	EU769874
Eudicotyledons	Symplocaceae	*Symplocos*	*Symplocos laurina*	EU769875
Eudicotyledons	Symplocaceae	*Symplocos*	*Symplocos laurina*	EU769876
Eudicotyledons	Symplocaceae	*Symplocos*	*Symplocos laurina*	EU769877
Eudicotyledons	Symplocaceae	*Symplocos*	*Symplocos laurina*	EU769878
Eudicotyledons	Symplocaceae	*Symplocos*	*Symplocos laurina*	EU769879
Eudicotyledons	Symplocaceae	*Symplocos*	*Symplocos laurina*	EU769880
Eudicotyledons	Symplocaceae	*Symplocos*	*Symplocos laurina*	EU769881
Eudicotyledons	Symplocaceae	*Symplocos*	*Symplocos laurina*	EU769882
Eudicotyledons	Symplocaceae	*Symplocos*	*Symplocos laurina*	EU769883
Eudicotyledons	Symplocaceae	*Symplocos*	*Symplocos laurina*	EU769884
Eudicotyledons	Symplocaceae	*Symplocos*	*Symplocos laurina*	EU769885
Eudicotyledons	Symplocaceae	*Symplocos*	*Symplocos laurina*	EU769886
Eudicotyledons	Symplocaceae	*Symplocos*	*Symplocos laurina*	EU769887
Eudicotyledons	Symplocaceae	*Symplocos*	*Symplocos laurina*	EU769888

Eudicotyledons	Symplocaceae	*Symplocos*	*Symplocos laurina*	EU769889
Eudicotyledons	Symplocaceae	*Symplocos*	*Symplocos laurina*	EU769890
Eudicotyledons	Symplocaceae	*Symplocos*	*Symplocos laurina*	EU769891
Eudicotyledons	Symplocaceae	*Symplocos*	*Symplocos laurina*	EU769892
Eudicotyledons	Symplocaceae	*Symplocos*	*Symplocos laurina*	EU769893
Eudicotyledons	Symplocaceae	*Symplocos*	*Symplocos laurina*	EU769894
Eudicotyledons	Symplocaceae	*Symplocos*	*Symplocos laurina*	EU769895
Eudicotyledons	Symplocaceae	*Symplocos*	*Symplocos laurina*	EU769896
Eudicotyledons	Symplocaceae	*Symplocos*	*Symplocos laurina*	EU769897
Eudicotyledons	Symplocaceae	*Symplocos*	*Symplocos laurina*	EU769898
Eudicotyledons	Symplocaceae	*Symplocos*	*Symplocos laurina*	EU769899
Eudicotyledons	Symplocaceae	*Symplocos*	*Symplocos laurina*	EU769900
Eudicotyledons	Symplocaceae	*Symplocos*	*Symplocos laurina*	EU769901
Eudicotyledons	Symplocaceae	*Symplocos*	*Symplocos laurina*	EU769902
Eudicotyledons	Symplocaceae	*Symplocos*	*Symplocos laurina*	EU769903
Eudicotyledons	Symplocaceae	*Symplocos*	*Symplocos laurina*	EU769904
Eudicotyledons	Symplocaceae	*Symplocos*	*Symplocos laurina*	EU769905
Eudicotyledons	Symplocaceae	*Symplocos*	*Symplocos laurina*	EU769906
Eudicotyledons	Symplocaceae	*Symplocos*	*Symplocos laurina*	EU769907
Eudicotyledons	Symplocaceae	*Symplocos*	*Symplocos laurina*	EU769908
Eudicotyledons	Symplocaceae	*Symplocos*	*Symplocos laurina*	EU769909
Eudicotyledons	Symplocaceae	*Symplocos*	*Symplocos laurina*	EU769910
Eudicotyledons	Symplocaceae	*Symplocos*	*Symplocos laurina*	EU769911
Eudicotyledons	Symplocaceae	*Symplocos*	*Symplocos laurina*	EU769912
Eudicotyledons	Symplocaceae	*Symplocos*	*Symplocos laurina*	EU769913
Eudicotyledons	Symplocaceae	*Symplocos*	*Symplocos laurina*	EU769914
Eudicotyledons	Symplocaceae	*Symplocos*	*Symplocos laurina*	EU769915
Eudicotyledons	Symplocaceae	*Symplocos*	*Symplocos laurina*	EU769916
Eudicotyledons	Symplocaceae	*Symplocos*	*Symplocos laurina*	EU769917
Eudicotyledons	Symplocaceae	*Symplocos*	*Symplocos laurina*	EU769918
Eudicotyledons	Symplocaceae	*Symplocos*	*Symplocos laurina*	EU769919
Eudicotyledons	Symplocaceae	*Symplocos*	*Symplocos laurina*	EU769920
Eudicotyledons	Symplocaceae	*Symplocos*	*Symplocos laurina*	EU769921
Eudicotyledons	Symplocaceae	*Symplocos*	*Symplocos laurina*	EU769922
Eudicotyledons	Symplocaceae	*Symplocos*	*Symplocos laurina*	EU769923
Eudicotyledons	Symplocaceae	*Symplocos*	*Symplocos laurina*	EU769924
Eudicotyledons	Symplocaceae	*Symplocos*	*Symplocos laurina*	EU769925
Eudicotyledons	Symplocaceae	*Symplocos*	*Symplocos laurina*	EU769926
Eudicotyledons	Symplocaceae	*Symplocos*	*Symplocos laurina*	EU769927
Eudicotyledons	Symplocaceae	*Symplocos*	*Symplocos laurina*	EU769928
Eudicotyledons	Symplocaceae	*Symplocos*	*Symplocos laurina*	EU769929
Eudicotyledons	Symplocaceae	*Symplocos*	*Symplocos laurina*	EU769930
Eudicotyledons	Symplocaceae	*Symplocos*	*Symplocos laurina*	EU769931
Eudicotyledons	Symplocaceae	*Symplocos*	*Symplocos laurina*	EU769932
Eudicotyledons	Symplocaceae	*Symplocos*	*Symplocos laurina*	EU769933
Eudicotyledons	Symplocaceae	*Symplocos*	*Symplocos laurina*	EU769934
Eudicotyledons	Symplocaceae	*Symplocos*	*Symplocos laurina*	EU769935
Eudicotyledons	Symplocaceae	*Symplocos*	*Symplocos laurina*	EU769936
Eudicotyledons	Symplocaceae	*Symplocos*	*Symplocos laurina*	EU769937
Eudicotyledons	Symplocaceae	*Symplocos*	*Symplocos laurina*	EU769938
Eudicotyledons	Symplocaceae	*Symplocos*	*Symplocos laurina*	EU769939
Eudicotyledons	Symplocaceae	*Symplocos*	*Symplocos laurina*	EU769940
Eudicotyledons	Symplocaceae	*Symplocos*	*Symplocos laurina*	EU769941

Eudicotyledons	Symplocaceae	*Symplocos*	*Symplocos laurina*	EU769942
Eudicotyledons	Symplocaceae	*Symplocos*	*Symplocos laurina*	EU769943
Eudicotyledons	Symplocaceae	*Symplocos*	*Symplocos laurina*	EU769944
Eudicotyledons	Symplocaceae	*Symplocos*	*Symplocos laurina*	EU769945
Eudicotyledons	Symplocaceae	*Symplocos*	*Symplocos laurina*	EU769946
Eudicotyledons	Symplocaceae	*Symplocos*	*Symplocos laurina*	EU769947
Eudicotyledons	Symplocaceae	*Symplocos*	*Symplocos laurina*	EU769948
Eudicotyledons	Symplocaceae	*Symplocos*	*Symplocos laurina*	EU769949
Eudicotyledons	Symplocaceae	*Symplocos*	*Symplocos laurina*	EU769950
Eudicotyledons	Symplocaceae	*Symplocos*	*Symplocos laurina*	EU769951
Eudicotyledons	Symplocaceae	*Symplocos*	*Symplocos laurina*	EU769952
Eudicotyledons	Polygonaceae	*Polygonum*	*Polygonum amphibium*	EF653750
Eudicotyledons	Polygonaceae	*Polygonum*	*Polygonum amphibium*	EF653751
Eudicotyledons	Asteraceae	*Sonchus*	*Sonchus hydrophilus*	AY457991
Eudicotyledons	Asteraceae	*Sonchus*	*Sonchus hydrophilus*	AY457992
Eudicotyledons	Asteraceae	*Sonchus*	*Sonchus hydrophilus*	AY457993
Eudicotyledons	Asteraceae	*Sonchus*	*Sonchus hydrophilus*	AY457994
Eudicotyledons	Asteraceae	*Sonchus*	*Sonchus hydrophilus*	AY457995
Eudicotyledons	Phyllanthaceae	*Phyllanthus*	*Phyllanthus acidus*	GU598541
Eudicotyledons	Phyllanthaceae	*Phyllanthus*	*Phyllanthus acidus*	GU598542
Eudicotyledons	Phyllanthaceae	*Phyllanthus*	*Phyllanthus acidus*	GU598543
Eudicotyledons	Primulaceae	*Primula*	*Primula amethystina*	JN046466
Eudicotyledons	Primulaceae	*Primula*	*Primula amethystina*	JN046467
Eudicotyledons	Primulaceae	*Primula*	*Primula amethystina*	JN046468
Eudicotyledons	Primulaceae	*Primula*	*Primula faberi*	JN046517
Eudicotyledons	Primulaceae	*Primula*	*Primula faberi*	JN046518
Eudicotyledons	Asteraceae	*Raoulia*	*Raoulia grandiflora*	AY611231
Eudicotyledons	Asteraceae	*Raoulia*	*Raoulia grandiflora*	AY611232
Eudicotyledons	Orobanchaceae	*Agalinis*	*Agalinis acuta*	GU943532
Eudicotyledons	Orobanchaceae	*Agalinis*	*Agalinis acuta*	EU827882
Eudicotyledons	Orobanchaceae	*Agalinis*	*Agalinis acuta*	EU827883
Eudicotyledons	Orobanchaceae	*Agalinis*	*Agalinis acuta*	EU827884
Eudicotyledons	Orobanchaceae	*Agalinis*	*Agalinis acuta*	EU827885
Eudicotyledons	Orobanchaceae	*Agalinis*	*Agalinis acuta*	EU827886
Eudicotyledons	Orobanchaceae	*Agalinis*	*Agalinis acuta*	EU827887
Eudicotyledons	Orobanchaceae	*Agalinis*	*Agalinis acuta*	EU827888
Eudicotyledons	Orobanchaceae	*Agalinis*	*Agalinis acuta*	EU827889
Eudicotyledons	Orobanchaceae	*Agalinis*	*Agalinis acuta*	EU827890
Eudicotyledons	Orobanchaceae	*Agalinis*	*Agalinis aphylla*	EU827891
Eudicotyledons	Orobanchaceae	*Agalinis*	*Agalinis aphylla*	EU827892
Eudicotyledons	Orobanchaceae	*Agalinis*	*Agalinis divaricata*	EU827901
Eudicotyledons	Orobanchaceae	*Agalinis*	*Agalinis divaricata*	EU827902
Eudicotyledons	Orobanchaceae	*Agalinis*	*Agalinis fasciculata*	EU827904
Eudicotyledons	Orobanchaceae	*Agalinis*	*Agalinis fasciculata*	EU827905
Eudicotyledons	Orobanchaceae	*Agalinis*	*Agalinis fasciculata*	EU827906
Eudicotyledons	Orobanchaceae	*Agalinis*	*Agalinis filicaulis*	EU827907
Eudicotyledons	Orobanchaceae	*Agalinis*	*Agalinis filicaulis*	EU827908
Eudicotyledons	Orobanchaceae	*Agalinis*	*Agalinis heterophylla*	EU827896
Eudicotyledons	Orobanchaceae	*Agalinis*	*Agalinis heterophylla*	EU827914
Eudicotyledons	Orobanchaceae	*Agalinis*	*Agalinis heterophylla*	EU827915
Eudicotyledons	Orobanchaceae	*Agalinis*	*Agalinis linifolia*	EU827920
Eudicotyledons	Orobanchaceae	*Agalinis*	*Agalinis linifolia*	EU827921
Eudicotyledons	Orobanchaceae	*Agalinis*	*Agalinis obtusifolia*	GU943536

Eudicotyledons	Orobanchaceae	*Agalinis*	*Agalinis obtusifolia*	GU943537
Eudicotyledons	Orobanchaceae	*Agalinis*	*Agalinis obtusifolia*	GU943538
Eudicotyledons	Orobanchaceae	*Agalinis*	*Agalinis obtusifolia*	GU943539
Eudicotyledons	Orobanchaceae	*Agalinis*	*Agalinis obtusifolia*	EU827925
Eudicotyledons	Orobanchaceae	*Agalinis*	*Agalinis obtusifolia*	EU827926
Eudicotyledons	Orobanchaceae	*Agalinis*	*Agalinis obtusifolia*	EU827928
Eudicotyledons	Orobanchaceae	*Agalinis*	*Agalinis obtusifolia*	EU827929
Eudicotyledons	Orobanchaceae	*Agalinis*	*Agalinis obtusifolia*	EU827930
Eudicotyledons	Orobanchaceae	*Agalinis*	*Agalinis plukenetii*	EU827937
Eudicotyledons	Orobanchaceae	*Agalinis*	*Agalinis plukenetii*	EU827938
Eudicotyledons	Orobanchaceae	*Agalinis*	*Agalinis pulchella*	EU827939
Eudicotyledons	Orobanchaceae	*Agalinis*	*Agalinis pulchella*	EU827940
Eudicotyledons	Orobanchaceae	*Agalinis*	*Agalinis setacea*	GU943540
Eudicotyledons	Orobanchaceae	*Agalinis*	*Agalinis setacea*	GU943541
Eudicotyledons	Orobanchaceae	*Agalinis*	*Agalinis setacea*	EU827945
Eudicotyledons	Orobanchaceae	*Agalinis*	*Agalinis setacea*	EU827946
Eudicotyledons	Orobanchaceae	*Agalinis*	*Agalinis strictifolia*	EU827898
Eudicotyledons	Orobanchaceae	*Agalinis*	*Agalinis strictifolia*	EU827950
Eudicotyledons	Orobanchaceae	*Agalinis*	*Agalinis tenella*	GU943542
Eudicotyledons	Orobanchaceae	*Agalinis*	*Agalinis tenella*	GU943543
Eudicotyledons	Orobanchaceae	*Agalinis*	*Agalinis tenella*	EU827927
Eudicotyledons	Orobanchaceae	*Agalinis*	*Agalinis tenella*	EU827951
Eudicotyledons	Orobanchaceae	*Agalinis*	*Agalinis tenella*	EU827952
Eudicotyledons	Orobanchaceae	*Agalinis*	*Agalinis tenella*	EU827953
Eudicotyledons	Orobanchaceae	*Agalinis*	*Agalinis tenella*	EU827954
Eudicotyledons	Orobanchaceae	*Agalinis*	*Agalinis tenella*	EU827955
Eudicotyledons	Orobanchaceae	*Agalinis*	*Agalinis tenuifolia*	GU943544
Eudicotyledons	Orobanchaceae	*Agalinis*	*Agalinis tenuifolia*	EU827956
Eudicotyledons	Orobanchaceae	*Agalinis*	*Agalinis tenuifolia*	EU827957
Eudicotyledons	Orobanchaceae	*Agalinis*	*Agalinis tenuifolia*	EU827958
Eudicotyledons	Ranunculaceae	*Aconitum*	*Aconitum alboviolaceum*	GQ337824
Eudicotyledons	Ranunculaceae	*Aconitum*	*Aconitum alboviolaceum*	GQ337825
Eudicotyledons	Ranunculaceae	*Aconitum*	*Aconitum alboviolaceum*	GQ337826
Eudicotyledons	Ranunculaceae	*Aconitum*	*Aconitum alboviolaceum*	GQ337830
Eudicotyledons	Ranunculaceae	*Aconitum*	*Aconitum alboviolaceum*	GQ337831
Eudicotyledons	Ranunculaceae	*Aconitum*	*Aconitum alboviolaceum*	GQ337832
Eudicotyledons	Ranunculaceae	*Aconitum*	*Aconitum alboviolaceum*	GQ337833
Eudicotyledons	Ranunculaceae	*Aconitum*	*Aconitum alboviolaceum*	GQ337834
Eudicotyledons	Ranunculaceae	*Aconitum*	*Aconitum alboviolaceum*	GQ337835
Eudicotyledons	Ranunculaceae	*Aconitum*	*Aconitum alboviolaceum*	JN043731
Eudicotyledons	Ranunculaceae	*Aconitum*	*Aconitum alboviolaceum*	JN043732
Eudicotyledons	Ranunculaceae	*Aconitum*	*Aconitum alboviolaceum*	JN043733
Eudicotyledons	Ranunculaceae	*Aconitum*	*Aconitum alboviolaceum*	JN043734
Eudicotyledons	Ranunculaceae	*Aconitum*	*Aconitum alboviolaceum*	JN043735
Eudicotyledons	Phyllanthaceae	*Phyllanthus*	*Phyllanthus debilis*	GU598567
Eudicotyledons	Phyllanthaceae	*Phyllanthus*	*Phyllanthus debilis*	GU598568
Eudicotyledons	Phyllanthaceae	*Phyllanthus*	*Phyllanthus debilis*	GQ409810
Eudicotyledons	Phyllanthaceae	*Phyllanthus*	*Phyllanthus debilis*	GQ409811
Eudicotyledons	Phyllanthaceae	*Phyllanthus*	*Phyllanthus debilis*	GQ409812
Eudicotyledons	Phyllanthaceae	*Phyllanthus*	*Phyllanthus amarus*	GU598561
Eudicotyledons	Phyllanthaceae	*Phyllanthus*	*Phyllanthus amarus*	GU598562
Eudicotyledons	Phyllanthaceae	*Phyllanthus*	*Phyllanthus amarus*	GU598563
Eudicotyledons	Phyllanthaceae	*Phyllanthus*	*Phyllanthus amarus*	GU598564

Eudicotyledons	Phyllanthaceae	*Phyllanthus*	*Phyllanthus amarus*	GU598565
Eudicotyledons	Phyllanthaceae	*Phyllanthus*	*Phyllanthus amarus*	GU598577
Eudicotyledons	Phyllanthaceae	*Phyllanthus*	*Phyllanthus amarus*	GQ409816
Eudicotyledons	Phyllanthaceae	*Phyllanthus*	*Phyllanthus amarus*	GQ409819
Eudicotyledons	Phyllanthaceae	*Phyllanthus*	*Phyllanthus amarus*	GQ409820
Eudicotyledons	Plumbaginaceae	*Limonium*	*Limonium wrightii*	AB450048
Eudicotyledons	Plumbaginaceae	*Limonium*	*Limonium wrightii*	AB450051
Eudicotyledons	Plumbaginaceae	*Limonium*	*Limonium wrightii*	AB450054
Eudicotyledons	Plumbaginaceae	*Limonium*	*Limonium wrightii*	AB450057
Eudicotyledons	Plumbaginaceae	*Limonium*	*Limonium wrightii*	AB450060
Eudicotyledons	Plumbaginaceae	*Limonium*	*Limonium wrightii*	AB450063
Eudicotyledons	Plumbaginaceae	*Limonium*	*Limonium wrightii*	AB450066
Eudicotyledons	Plumbaginaceae	*Limonium*	*Limonium wrightii*	AB450069
Eudicotyledons	Plumbaginaceae	*Limonium*	*Limonium wrightii*	AB450072
Eudicotyledons	Plumbaginaceae	*Limonium*	*Limonium wrightii*	AB450075
Eudicotyledons	Plumbaginaceae	*Limonium*	*Limonium wrightii*	AB450078
Eudicotyledons	Plumbaginaceae	*Limonium*	*Limonium wrightii*	AB450081
Eudicotyledons	Plumbaginaceae	*Limonium*	*Limonium wrightii*	AB450084
Eudicotyledons	Plumbaginaceae	*Limonium*	*Limonium wrightii*	AB450087
Eudicotyledons	Plumbaginaceae	*Limonium*	*Limonium wrightii*	AB450090
Eudicotyledons	Plumbaginaceae	*Limonium*	*Limonium wrightii*	AB450093
Eudicotyledons	Plumbaginaceae	*Limonium*	*Limonium wrightii*	AB450096
Eudicotyledons	Plumbaginaceae	*Limonium*	*Limonium wrightii*	AB450099
Eudicotyledons	Plumbaginaceae	*Limonium*	*Limonium wrightii*	AB450102
Eudicotyledons	Plumbaginaceae	*Limonium*	*Limonium wrightii*	AB450105
Eudicotyledons	Plumbaginaceae	*Limonium*	*Limonium wrightii*	AB450108
Eudicotyledons	Plumbaginaceae	*Limonium*	*Limonium wrightii*	AB450111
Eudicotyledons	Plumbaginaceae	*Limonium*	*Limonium wrightii*	AB450114
Eudicotyledons	Plumbaginaceae	*Limonium*	*Limonium wrightii*	AB450117
Eudicotyledons	Plumbaginaceae	*Limonium*	*Limonium wrightii*	AB450120
Eudicotyledons	Plumbaginaceae	*Limonium*	*Limonium wrightii*	AB450123
Eudicotyledons	Plumbaginaceae	*Limonium*	*Limonium wrightii*	AB450126
Eudicotyledons	Plumbaginaceae	*Limonium*	*Limonium wrightii*	AB450129
Eudicotyledons	Plumbaginaceae	*Limonium*	*Limonium wrightii*	AB450132
Eudicotyledons	Plumbaginaceae	*Limonium*	*Limonium wrightii*	AB450135
Eudicotyledons	Plumbaginaceae	*Limonium*	*Limonium wrightii*	AB450138
Eudicotyledons	Plumbaginaceae	*Limonium*	*Limonium wrightii*	AB450141
Eudicotyledons	Plumbaginaceae	*Limonium*	*Limonium wrightii*	AB450144
Eudicotyledons	Plumbaginaceae	*Limonium*	*Limonium wrightii*	AB450147
Eudicotyledons	Plumbaginaceae	*Limonium*	*Limonium wrightii*	AB450150
Eudicotyledons	Plumbaginaceae	*Limonium*	*Limonium wrightii*	AB450153
Eudicotyledons	Plumbaginaceae	*Limonium*	*Limonium wrightii*	AB450156
Eudicotyledons	Plumbaginaceae	*Limonium*	*Limonium wrightii*	AB450159
Eudicotyledons	Plumbaginaceae	*Limonium*	*Limonium wrightii*	AB450162
Eudicotyledons	Plumbaginaceae	*Limonium*	*Limonium wrightii*	AB450165
Eudicotyledons	Plumbaginaceae	*Limonium*	*Limonium wrightii*	AB450168
Eudicotyledons	Plumbaginaceae	*Limonium*	*Limonium wrightii*	AB450171
Eudicotyledons	Plumbaginaceae	*Limonium*	*Limonium wrightii*	AB450174
Eudicotyledons	Plumbaginaceae	*Limonium*	*Limonium wrightii*	AB450177
Eudicotyledons	Plumbaginaceae	*Limonium*	*Limonium wrightii*	AB450180
Eudicotyledons	Plumbaginaceae	*Limonium*	*Limonium wrightii*	AB450183
Eudicotyledons	Plumbaginaceae	*Limonium*	*Limonium wrightii*	AB450186
Eudicotyledons	Plumbaginaceae	*Limonium*	*Limonium wrightii*	AB450189

Eudicotyledons	Plumbaginaceae	*Limonium*	*Limonium wrightii*	AB450192
Eudicotyledons	Plumbaginaceae	*Limonium*	*Limonium sinense*	AB450198
Eudicotyledons	Plumbaginaceae	*Limonium*	*Limonium sinense*	AB450201
Eudicotyledons	Plumbaginaceae	*Limonium*	*Limonium sinense*	AB450204
Eudicotyledons	Plumbaginaceae	*Limonium*	*Limonium sinense*	AB450207
Eudicotyledons	Plumbaginaceae	*Limonium*	*Limonium sinense*	AB450210
Eudicotyledons	Plumbaginaceae	*Limonium*	*Limonium tetragonum*	AB450216
Eudicotyledons	Plumbaginaceae	*Limonium*	*Limonium tetragonum*	AB450219
Eudicotyledons	Plumbaginaceae	*Limonium*	*Limonium tetragonum*	AB450222
Eudicotyledons	Phyllanthaceae	*Phyllanthus*	*Phyllanthus urinaria*	GU598573
Eudicotyledons	Phyllanthaceae	*Phyllanthus*	*Phyllanthus urinaria*	GU598574
Eudicotyledons	Phyllanthaceae	*Phyllanthus*	*Phyllanthus urinaria*	GQ409813
Eudicotyledons	Phyllanthaceae	*Phyllanthus*	*Phyllanthus urinaria*	GQ409815
Eudicotyledons	Fabaceae	*Dalbergia*	*Dalbergia nigrescens*	FR854158
Eudicotyledons	Fabaceae	*Dalbergia*	*Dalbergia nigrescens*	FR854159
Eudicotyledons	Fabaceae	*Dalbergia*	*Dalbergia nigrescens*	FR854160
Eudicotyledons	Fabaceae	*Dalbergia*	*Dalbergia nigrescens*	FR854161
Eudicotyledons	Fabaceae	*Dalbergia*	*Dalbergia nigrescens*	FR854162
Eudicotyledons	Phyllanthaceae	*Phyllanthus*	*Phyllanthus polyphyllus*	GU598544
Eudicotyledons	Phyllanthaceae	*Phyllanthus*	*Phyllanthus polyphyllus*	GU598545
Eudicotyledons	Phyllanthaceae	*Phyllanthus*	*Phyllanthus polyphyllus*	GU598546
Eudicotyledons	Gesneriaceae	*Cyrtandra*	*Cyrtandra kauaiensis*	GQ475121
Eudicotyledons	Gesneriaceae	*Cyrtandra*	*Cyrtandra kauaiensis*	EU919999
Eudicotyledons	Gesneriaceae	*Cyrtandra*	*Cyrtandra laxiflora*	GQ475161
Eudicotyledons	Gesneriaceae	*Cyrtandra*	*Cyrtandra laxiflora*	EU920007
Eudicotyledons	Gesneriaceae	*Cyrtandra*	*Cyrtandra samoensis*	GQ475122
Eudicotyledons	Gesneriaceae	*Cyrtandra*	*Cyrtandra samoensis*	GQ475148
Eudicotyledons	Gesneriaceae	*Cyrtandra*	*Cyrtandra samoensis*	GQ475150
Eudicotyledons	Gesneriaceae	*Cyrtandra*	*Cyrtandra samoensis*	EU920001
Eudicotyledons	Gesneriaceae	*Cyrtandra*	*Cyrtandra samoensis*	EU920002
Eudicotyledons	Primulaceae	*Lysimachia*	*Lysimachia alfredii*	JN045291
Eudicotyledons	Primulaceae	*Lysimachia*	*Lysimachia alfredii*	JN045292
Eudicotyledons	Primulaceae	*Lysimachia*	*Lysimachia capillipes*	JN045295
Eudicotyledons	Primulaceae	*Lysimachia*	*Lysimachia capillipes*	JN045296
Eudicotyledons	Ericaceae	*Pieris*	*Pieris japonica*	HQ426995
Eudicotyledons	Ericaceae	*Pieris*	*Pieris japonica*	EU547719
Eudicotyledons	Ericaceae	*Pieris*	*Pieris japonica*	AB206621
Eudicotyledons	Ericaceae	*Pieris*	*Pieris koidzumiana*	AB206623
Eudicotyledons	Ericaceae	*Pieris*	*Pieris koidzumiana*	AB206624
Eudicotyledons	Phyllanthaceae	*Phyllanthus*	*Phyllanthus maderaspatensis*	GU598536
Eudicotyledons	Phyllanthaceae	*Phyllanthus*	*Phyllanthus maderaspatensis*	GU598537
Eudicotyledons	Phyllanthaceae	*Phyllanthus*	*Phyllanthus maderaspatensis*	GU598538
Eudicotyledons	Phyllanthaceae	*Phyllanthus*	*Phyllanthus maderaspatensis*	GU598575
Eudicotyledons	Phyllanthaceae	*Phyllanthus*	*Phyllanthus maderaspatensis*	GU598576
Eudicotyledons	Phyllanthaceae	*Phyllanthus*	*Phyllanthus rheedii*	GQ409807
Eudicotyledons	Phyllanthaceae	*Phyllanthus*	*Phyllanthus rheedii*	GQ409809
Eudicotyledons	Araliaceae	*Schefflera*	*Schefflera heptaphylla*	HQ415433
Eudicotyledons	Araliaceae	*Schefflera*	*Schefflera heptaphylla*	JN406930
Eudicotyledons	Araliaceae	*Schefflera*	*Schefflera heptaphylla*	JN406931
Eudicotyledons	Araliaceae	*Schefflera*	*Schefflera heptaphylla*	JN406932
Eudicotyledons	Araliaceae	*Schefflera*	*Schefflera heptaphylla*	JN406933
Eudicotyledons	Araliaceae	*Schefflera*	*Schefflera heptaphylla*	JN406934
Eudicotyledons	Araliaceae	*Schefflera*	*Schefflera heptaphylla*	GU054831

Eudicotyledons	Araliaceae	*Schefflera*	*Schefflera morototoni*	GU054837
Eudicotyledons	Araliaceae	*Schefflera*	*Schefflera morototoni*	HM447000
Eudicotyledons	Asteraceae	*Solidago*	*Solidago flexicaulis*	DQ006152
Eudicotyledons	Asteraceae	*Solidago*	*Solidago flexicaulis*	EU750565
Eudicotyledons	Asteraceae	*Solidago*	*Solidago flexicaulis*	EU750566
Eudicotyledons	Asteraceae	*Solidago*	*Solidago flexicaulis*	HQ142547
Eudicotyledons	Asteraceae	*Solidago*	*Solidago gigantea*	HQ596851
Eudicotyledons	Asteraceae	*Solidago*	*Solidago gigantea*	EU337125
Eudicotyledons	Asteraceae	*Solidago*	*Solidago gigantea*	EU337126
Eudicotyledons	Asteraceae	*Solidago*	*Solidago gigantea*	EU337127
Eudicotyledons	Asteraceae	*Solidago*	*Solidago gigantea*	EU337128
Eudicotyledons	Asteraceae	*Solidago*	*Solidago gigantea*	EU337129
Eudicotyledons	Asteraceae	*Solidago*	*Solidago gigantea*	EU337130
Eudicotyledons	Asteraceae	*Solidago*	*Solidago gigantea*	EU337131
Eudicotyledons	Asteraceae	*Solidago*	*Solidago gigantea*	EU337132
Eudicotyledons	Asteraceae	*Solidago*	*Solidago gigantea*	EU337133
Eudicotyledons	Asteraceae	*Solidago*	*Solidago gigantea*	EU337134
Eudicotyledons	Asteraceae	*Solidago*	*Solidago gigantea*	EU337135
Eudicotyledons	Asteraceae	*Solidago*	*Solidago gigantea*	EU337136
Eudicotyledons	Asteraceae	*Solidago*	*Solidago gigantea*	EU337137
Eudicotyledons	Asteraceae	*Solidago*	*Solidago gigantea*	EU337138
Eudicotyledons	Asteraceae	*Solidago*	*Solidago gigantea*	EU337139
Eudicotyledons	Asteraceae	*Solidago*	*Solidago gigantea*	EU337140
Eudicotyledons	Asteraceae	*Solidago*	*Solidago gigantea*	EU337141
Eudicotyledons	Asteraceae	*Solidago*	*Solidago gigantea*	EU337142
Eudicotyledons	Asteraceae	*Solidago*	*Solidago gigantea*	EU337143
Eudicotyledons	Asteraceae	*Solidago*	*Solidago gigantea*	EU337144
Eudicotyledons	Asteraceae	*Solidago*	*Solidago gigantea*	EU337145
Eudicotyledons	Asteraceae	*Solidago*	*Solidago gigantea*	EU337146
Eudicotyledons	Asteraceae	*Solidago*	*Solidago gigantea*	EU337147
Eudicotyledons	Asteraceae	*Solidago*	*Solidago gigantea*	EU337148
Eudicotyledons	Asteraceae	*Solidago*	*Solidago gigantea*	EU337149
Eudicotyledons	Asteraceae	*Solidago*	*Solidago gigantea*	EU337150
Eudicotyledons	Asteraceae	*Solidago*	*Solidago gigantea*	EU337151
Eudicotyledons	Asteraceae	*Solidago*	*Solidago gigantea*	EU337152
Eudicotyledons	Asteraceae	*Solidago*	*Solidago gigantea*	EU337153
Eudicotyledons	Asteraceae	*Solidago*	*Solidago gigantea*	EU337154
Eudicotyledons	Asteraceae	*Solidago*	*Solidago gigantea*	EU337155
Eudicotyledons	Asteraceae	*Solidago*	*Solidago gigantea*	EU337156
Eudicotyledons	Asteraceae	*Solidago*	*Solidago gigantea*	EU337157
Eudicotyledons	Asteraceae	*Solidago*	*Solidago gigantea*	EU337158
Eudicotyledons	Asteraceae	*Solidago*	*Solidago gigantea*	EU337159
Eudicotyledons	Asteraceae	*Solidago*	*Solidago gigantea*	EU337160
Eudicotyledons	Asteraceae	*Solidago*	*Solidago gigantea*	EU337161
Eudicotyledons	Asteraceae	*Solidago*	*Solidago gigantea*	EU337162
Eudicotyledons	Asteraceae	*Solidago*	*Solidago gigantea*	EU337163
Eudicotyledons	Asteraceae	*Solidago*	*Solidago gigantea*	EU337164
Eudicotyledons	Asteraceae	*Solidago*	*Solidago gigantea*	EU337165
Eudicotyledons	Asteraceae	*Solidago*	*Solidago gigantea*	EU337166
Eudicotyledons	Asteraceae	*Solidago*	*Solidago gigantea*	EU337167
Eudicotyledons	Asteraceae	*Solidago*	*Solidago gigantea*	EU337168
Eudicotyledons	Asteraceae	*Solidago*	*Solidago gigantea*	EU337169
Eudicotyledons	Asteraceae	*Solidago*	*Solidago gigantea*	EU337170

Eudicotyledons	Asteraceae	*Solidago*	*Solidago gigantea*	EU337171
Eudicotyledons	Asteraceae	*Solidago*	*Solidago gigantea*	EU337172
Eudicotyledons	Asteraceae	*Solidago*	*Solidago gigantea*	EU337173
Eudicotyledons	Asteraceae	*Solidago*	*Solidago gigantea*	EU337174
Eudicotyledons	Asteraceae	*Solidago*	*Solidago gigantea*	EU337175
Eudicotyledons	Asteraceae	*Solidago*	*Solidago gigantea*	EU337176
Eudicotyledons	Asteraceae	*Solidago*	*Solidago gigantea*	EU337177
Eudicotyledons	Asteraceae	*Solidago*	*Solidago gigantea*	EU337178
Eudicotyledons	Asteraceae	*Solidago*	*Solidago gigantea*	EU337179
Eudicotyledons	Asteraceae	*Solidago*	*Solidago gigantea*	EU337180
Eudicotyledons	Asteraceae	*Solidago*	*Solidago gigantea*	EU337181
Eudicotyledons	Asteraceae	*Solidago*	*Solidago gigantea*	EU337182
Eudicotyledons	Asteraceae	*Solidago*	*Solidago gigantea*	EU337183
Eudicotyledons	Asteraceae	*Solidago*	*Solidago gigantea*	EU337184
Eudicotyledons	Asteraceae	*Solidago*	*Solidago gigantea*	EU337185
Eudicotyledons	Asteraceae	*Solidago*	*Solidago gigantea*	EU337186
Eudicotyledons	Asteraceae	*Solidago*	*Solidago gigantea*	EU337187
Eudicotyledons	Asteraceae	*Solidago*	*Solidago gigantea*	EU337188
Eudicotyledons	Asteraceae	*Solidago*	*Solidago gigantea*	EU337189
Eudicotyledons	Asteraceae	*Solidago*	*Solidago gigantea*	EU337190
Eudicotyledons	Asteraceae	*Solidago*	*Solidago gigantea*	EU337191
Eudicotyledons	Asteraceae	*Solidago*	*Solidago gigantea*	EU337192
Eudicotyledons	Asteraceae	*Solidago*	*Solidago gigantea*	EU337193
Eudicotyledons	Asteraceae	*Solidago*	*Solidago gigantea*	EU337194
Eudicotyledons	Asteraceae	*Solidago*	*Solidago gigantea*	EU337195
Eudicotyledons	Asteraceae	*Solidago*	*Solidago gigantea*	EU337196
Eudicotyledons	Asteraceae	*Solidago*	*Solidago gigantea*	EU337197
Eudicotyledons	Asteraceae	*Solidago*	*Solidago gigantea*	EU337198
Eudicotyledons	Asteraceae	*Solidago*	*Solidago gigantea*	EU337199
Eudicotyledons	Asteraceae	*Solidago*	*Solidago gigantea*	EU337200
Eudicotyledons	Asteraceae	*Solidago*	*Solidago gigantea*	EU337201
Eudicotyledons	Asteraceae	*Solidago*	*Solidago gigantea*	EU337202
Eudicotyledons	Asteraceae	*Solidago*	*Solidago gigantea*	EU337203
Eudicotyledons	Asteraceae	*Solidago*	*Solidago gigantea*	EU337204
Eudicotyledons	Asteraceae	*Solidago*	*Solidago gigantea*	EU337205
Eudicotyledons	Asteraceae	*Solidago*	*Solidago gigantea*	EU337206
Eudicotyledons	Asteraceae	*Solidago*	*Solidago gigantea*	EU337207
Eudicotyledons	Asteraceae	*Solidago*	*Solidago gigantea*	EU337208
Eudicotyledons	Asteraceae	*Solidago*	*Solidago gigantea*	EU337209
Eudicotyledons	Asteraceae	*Solidago*	*Solidago gigantea*	EU337210
Eudicotyledons	Asteraceae	*Solidago*	*Solidago gigantea*	EU337211
Eudicotyledons	Asteraceae	*Solidago*	*Solidago gigantea*	EU337212
Eudicotyledons	Asteraceae	*Solidago*	*Solidago gigantea*	EU337213
Eudicotyledons	Asteraceae	*Solidago*	*Solidago gigantea*	EU337214
Eudicotyledons	Asteraceae	*Solidago*	*Solidago gigantea*	EU337215
Eudicotyledons	Asteraceae	*Solidago*	*Solidago gigantea*	EU337216
Eudicotyledons	Asteraceae	*Solidago*	*Solidago gigantea*	EU337217
Eudicotyledons	Asteraceae	*Solidago*	*Solidago gigantea*	EU337218
Eudicotyledons	Asteraceae	*Solidago*	*Solidago gigantea*	EU337219
Eudicotyledons	Asteraceae	*Solidago*	*Solidago gigantea*	EU337220
Eudicotyledons	Asteraceae	*Solidago*	*Solidago gigantea*	EU337221
Eudicotyledons	Asteraceae	*Solidago*	*Solidago gigantea*	EU337222
Eudicotyledons	Asteraceae	*Solidago*	*Solidago gigantea*	EU337223

Eudicotyledons	Asteraceae	*Solidago*	*Solidago gigantea*	EU337224
Eudicotyledons	Asteraceae	*Solidago*	*Solidago gigantea*	EU337225
Eudicotyledons	Asteraceae	*Solidago*	*Solidago gigantea*	EU337226
Eudicotyledons	Asteraceae	*Solidago*	*Solidago gigantea*	EU337227
Eudicotyledons	Asteraceae	*Solidago*	*Solidago gigantea*	EU337228
Eudicotyledons	Asteraceae	*Solidago*	*Solidago gigantea*	EU337229
Eudicotyledons	Asteraceae	*Solidago*	*Solidago gigantea*	EU337230
Eudicotyledons	Asteraceae	*Solidago*	*Solidago gigantea*	EU337231
Eudicotyledons	Asteraceae	*Solidago*	*Solidago gigantea*	EU337232
Eudicotyledons	Asteraceae	*Solidago*	*Solidago gigantea*	EU337233
Eudicotyledons	Asteraceae	*Solidago*	*Solidago gigantea*	EU337234
Eudicotyledons	Asteraceae	*Solidago*	*Solidago gigantea*	EU337235
Eudicotyledons	Asteraceae	*Solidago*	*Solidago gigantea*	EU337236
Eudicotyledons	Asteraceae	*Solidago*	*Solidago gigantea*	EU337237
Eudicotyledons	Asteraceae	*Solidago*	*Solidago gigantea*	EU337238
Eudicotyledons	Asteraceae	*Solidago*	*Solidago gigantea*	EU337239
Eudicotyledons	Asteraceae	*Solidago*	*Solidago gigantea*	EU337240
Eudicotyledons	Asteraceae	*Solidago*	*Solidago gigantea*	EU337241
Eudicotyledons	Asteraceae	*Solidago*	*Solidago gigantea*	EU337242
Eudicotyledons	Asteraceae	*Solidago*	*Solidago gigantea*	EU337243
Eudicotyledons	Asteraceae	*Solidago*	*Solidago gigantea*	EU337244
Eudicotyledons	Asteraceae	*Solidago*	*Solidago gigantea*	EU337245
Eudicotyledons	Asteraceae	*Solidago*	*Solidago gigantea*	EU337246
Eudicotyledons	Asteraceae	*Solidago*	*Solidago gigantea*	EU337247
Eudicotyledons	Asteraceae	*Solidago*	*Solidago gigantea*	EU337248
Eudicotyledons	Asteraceae	*Solidago*	*Solidago gigantea*	EU337249
Eudicotyledons	Asteraceae	*Solidago*	*Solidago gigantea*	EU337250
Eudicotyledons	Asteraceae	*Solidago*	*Solidago gigantea*	EU337251
Eudicotyledons	Asteraceae	*Solidago*	*Solidago gigantea*	EU337252
Eudicotyledons	Asteraceae	*Solidago*	*Solidago gigantea*	EU337253
Eudicotyledons	Asteraceae	*Solidago*	*Solidago gigantea*	EU337254
Eudicotyledons	Asteraceae	*Solidago*	*Solidago gigantea*	EU337255
Eudicotyledons	Asteraceae	*Solidago*	*Solidago gigantea*	EU337256
Eudicotyledons	Asteraceae	*Solidago*	*Solidago gigantea*	EU337257
Eudicotyledons	Asteraceae	*Solidago*	*Solidago gigantea*	EU337258
Eudicotyledons	Asteraceae	*Solidago*	*Solidago gigantea*	EU337259
Eudicotyledons	Asteraceae	*Solidago*	*Solidago gigantea*	EU337260
Eudicotyledons	Asteraceae	*Solidago*	*Solidago gigantea*	EU337261
Eudicotyledons	Asteraceae	*Solidago*	*Solidago gigantea*	EU337262
Eudicotyledons	Asteraceae	*Solidago*	*Solidago gigantea*	EU337263
Eudicotyledons	Asteraceae	*Solidago*	*Solidago gigantea*	EU337264
Eudicotyledons	Asteraceae	*Solidago*	*Solidago gigantea*	EU337265
Eudicotyledons	Asteraceae	*Solidago*	*Solidago gigantea*	EU337266
Eudicotyledons	Asteraceae	*Solidago*	*Solidago gigantea*	EU337267
Eudicotyledons	Asteraceae	*Solidago*	*Solidago gigantea*	EU337268
Eudicotyledons	Asteraceae	*Solidago*	*Solidago gigantea*	EU337269
Eudicotyledons	Asteraceae	*Solidago*	*Solidago gigantea*	EU337270
Eudicotyledons	Asteraceae	*Solidago*	*Solidago gigantea*	EU337271
Eudicotyledons	Asteraceae	*Solidago*	*Solidago gigantea*	EU337272
Eudicotyledons	Asteraceae	*Solidago*	*Solidago gigantea*	EU337273
Eudicotyledons	Asteraceae	*Solidago*	*Solidago gigantea*	EU337274
Eudicotyledons	Asteraceae	*Solidago*	*Solidago gigantea*	EU337275
Eudicotyledons	Asteraceae	*Solidago*	*Solidago gigantea*	EU337276

Eudicotyledons	Asteraceae	*Solidago*	*Solidago gigantea*	EU337277
Eudicotyledons	Asteraceae	*Solidago*	*Solidago gigantea*	EU337278
Eudicotyledons	Asteraceae	*Solidago*	*Solidago gigantea*	EU337279
Eudicotyledons	Asteraceae	*Solidago*	*Solidago gigantea*	EU337280
Eudicotyledons	Asteraceae	*Solidago*	*Solidago gigantea*	EU337281
Eudicotyledons	Asteraceae	*Solidago*	*Solidago gigantea*	EU337282
Eudicotyledons	Asteraceae	*Solidago*	*Solidago gigantea*	EU337283
Eudicotyledons	Asteraceae	*Solidago*	*Solidago gigantea*	EU337284
Eudicotyledons	Asteraceae	*Solidago*	*Solidago gigantea*	EU337285
Eudicotyledons	Asteraceae	*Solidago*	*Solidago gigantea*	EU337286
Eudicotyledons	Asteraceae	*Solidago*	*Solidago gigantea*	EU337287
Eudicotyledons	Asteraceae	*Solidago*	*Solidago gigantea*	EU337288
Eudicotyledons	Asteraceae	*Solidago*	*Solidago gigantea*	EU337289
Eudicotyledons	Asteraceae	*Solidago*	*Solidago gigantea*	EU337290
Eudicotyledons	Asteraceae	*Solidago*	*Solidago gigantea*	EU337291
Eudicotyledons	Asteraceae	*Solidago*	*Solidago gigantea*	EU337292
Eudicotyledons	Asteraceae	*Solidago*	*Solidago gigantea*	EU337293
Eudicotyledons	Asteraceae	*Solidago*	*Solidago gigantea*	EU337294
Eudicotyledons	Asteraceae	*Solidago*	*Solidago gigantea*	EU337295
Eudicotyledons	Asteraceae	*Solidago*	*Solidago gigantea*	EU337296
Eudicotyledons	Asteraceae	*Solidago*	*Solidago gigantea*	EU337297
Eudicotyledons	Asteraceae	*Solidago*	*Solidago gigantea*	EU337298
Eudicotyledons	Asteraceae	*Solidago*	*Solidago gigantea*	EU337299
Eudicotyledons	Asteraceae	*Solidago*	*Solidago gigantea*	EU337300
Eudicotyledons	Asteraceae	*Solidago*	*Solidago gigantea*	EU337301
Eudicotyledons	Asteraceae	*Solidago*	*Solidago gigantea*	EU337302
Eudicotyledons	Asteraceae	*Solidago*	*Solidago gigantea*	EU337303
Eudicotyledons	Asteraceae	*Solidago*	*Solidago gigantea*	EU337304
Eudicotyledons	Asteraceae	*Solidago*	*Solidago gigantea*	EU337305
Eudicotyledons	Asteraceae	*Solidago*	*Solidago gigantea*	EU337306
Eudicotyledons	Asteraceae	*Solidago*	*Solidago gigantea*	EU337307
Eudicotyledons	Asteraceae	*Solidago*	*Solidago gigantea*	EU337308
Eudicotyledons	Asteraceae	*Solidago*	*Solidago gigantea*	EU337309
Eudicotyledons	Asteraceae	*Solidago*	*Solidago gigantea*	EU337310
Eudicotyledons	Asteraceae	*Solidago*	*Solidago gigantea*	EU337311
Eudicotyledons	Asteraceae	*Solidago*	*Solidago gigantea*	EU337312
Eudicotyledons	Asteraceae	*Solidago*	*Solidago gigantea*	EU337313
Eudicotyledons	Asteraceae	*Solidago*	*Solidago gigantea*	EU337314
Eudicotyledons	Asteraceae	*Solidago*	*Solidago gigantea*	EU337315
Eudicotyledons	Asteraceae	*Solidago*	*Solidago gigantea*	EU337316
Eudicotyledons	Asteraceae	*Solidago*	*Solidago gigantea*	EU337317
Eudicotyledons	Asteraceae	*Solidago*	*Solidago gigantea*	EU337318
Eudicotyledons	Asteraceae	*Solidago*	*Solidago gigantea*	EU337319
Eudicotyledons	Asteraceae	*Solidago*	*Solidago gigantea*	EU337320
Eudicotyledons	Asteraceae	*Solidago*	*Solidago gigantea*	EU337321
Eudicotyledons	Asteraceae	*Solidago*	*Solidago gigantea*	EU337322
Eudicotyledons	Asteraceae	*Solidago*	*Solidago gigantea*	EU337323
Eudicotyledons	Asteraceae	*Solidago*	*Solidago gigantea*	EU337324
Eudicotyledons	Asteraceae	*Solidago*	*Solidago gigantea*	EU337325
Eudicotyledons	Asteraceae	*Solidago*	*Solidago gigantea*	EU337326
Eudicotyledons	Asteraceae	*Solidago*	*Solidago gigantea*	EU337327
Eudicotyledons	Asteraceae	*Solidago*	*Solidago gigantea*	EU337328
Eudicotyledons	Asteraceae	*Solidago*	*Solidago gigantea*	EU337329

Eudicotyledons	Asteraceae	*Solidago*	*Solidago gigantea*	EU337330
Eudicotyledons	Asteraceae	*Solidago*	*Solidago gigantea*	EU337331
Eudicotyledons	Asteraceae	*Solidago*	*Solidago gigantea*	EU337332
Eudicotyledons	Asteraceae	*Solidago*	*Solidago gigantea*	EU337333
Eudicotyledons	Asteraceae	*Solidago*	*Solidago gigantea*	EU337334
Eudicotyledons	Asteraceae	*Solidago*	*Solidago gigantea*	EU337335
Eudicotyledons	Asteraceae	*Solidago*	*Solidago gigantea*	EU337336
Eudicotyledons	Asteraceae	*Solidago*	*Solidago gigantea*	EU337337
Eudicotyledons	Asteraceae	*Solidago*	*Solidago gigantea*	EU337338
Eudicotyledons	Asteraceae	*Solidago*	*Solidago gigantea*	EU337339
Eudicotyledons	Asteraceae	*Solidago*	*Solidago gigantea*	EU337340
Eudicotyledons	Asteraceae	*Solidago*	*Solidago gigantea*	EU337341
Eudicotyledons	Asteraceae	*Solidago*	*Solidago gigantea*	EU337342
Eudicotyledons	Asteraceae	*Solidago*	*Solidago gigantea*	EU337343
Eudicotyledons	Asteraceae	*Solidago*	*Solidago gigantea*	EU337344
Eudicotyledons	Asteraceae	*Solidago*	*Solidago gigantea*	EU337345
Eudicotyledons	Asteraceae	*Solidago*	*Solidago gigantea*	EU337346
Eudicotyledons	Asteraceae	*Solidago*	*Solidago gigantea*	EU337347
Eudicotyledons	Asteraceae	*Solidago*	*Solidago gigantea*	EU337348
Eudicotyledons	Asteraceae	*Solidago*	*Solidago gigantea*	EU337349
Eudicotyledons	Asteraceae	*Solidago*	*Solidago gigantea*	EU337350
Eudicotyledons	Asteraceae	*Solidago*	*Solidago gigantea*	EU337351
Eudicotyledons	Asteraceae	*Solidago*	*Solidago gigantea*	EU337352
Eudicotyledons	Asteraceae	*Solidago*	*Solidago gigantea*	EU337353
Eudicotyledons	Asteraceae	*Solidago*	*Solidago gigantea*	EU337354
Eudicotyledons	Asteraceae	*Solidago*	*Solidago gigantea*	EU337355
Eudicotyledons	Asteraceae	*Solidago*	*Solidago gigantea*	EU337356
Eudicotyledons	Asteraceae	*Solidago*	*Solidago gigantea*	EU337357
Eudicotyledons	Asteraceae	*Solidago*	*Solidago gigantea*	EU337358
Eudicotyledons	Asteraceae	*Solidago*	*Solidago gigantea*	EU337359
Eudicotyledons	Asteraceae	*Solidago*	*Solidago gigantea*	EU337360
Eudicotyledons	Asteraceae	*Solidago*	*Solidago gigantea*	EU337361
Eudicotyledons	Asteraceae	*Solidago*	*Solidago gigantea*	EU337362
Eudicotyledons	Asteraceae	*Solidago*	*Solidago gigantea*	EU337363
Eudicotyledons	Asteraceae	*Solidago*	*Solidago gigantea*	EU337364
Eudicotyledons	Asteraceae	*Solidago*	*Solidago gigantea*	EU337365
Eudicotyledons	Asteraceae	*Solidago*	*Solidago gigantea*	EU337366
Eudicotyledons	Asteraceae	*Solidago*	*Solidago gigantea*	EU337367
Eudicotyledons	Asteraceae	*Solidago*	*Solidago gigantea*	EU337368
Eudicotyledons	Asteraceae	*Solidago*	*Solidago gigantea*	EU337369
Eudicotyledons	Asteraceae	*Solidago*	*Solidago gigantea*	EU337370
Eudicotyledons	Asteraceae	*Solidago*	*Solidago gigantea*	EU337371
Eudicotyledons	Asteraceae	*Solidago*	*Solidago gigantea*	EU337372
Eudicotyledons	Asteraceae	*Solidago*	*Solidago gigantea*	EU337373
Eudicotyledons	Asteraceae	*Solidago*	*Solidago gigantea*	EU337374
Eudicotyledons	Asteraceae	*Solidago*	*Solidago gigantea*	EU337375
Eudicotyledons	Asteraceae	*Solidago*	*Solidago gigantea*	EU337376
Eudicotyledons	Asteraceae	*Solidago*	*Solidago gigantea*	EU337377
Eudicotyledons	Asteraceae	*Solidago*	*Solidago gigantea*	EU337378
Eudicotyledons	Asteraceae	*Solidago*	*Solidago gigantea*	EU337379
Eudicotyledons	Asteraceae	*Solidago*	*Solidago gigantea*	EU337380
Eudicotyledons	Asteraceae	*Solidago*	*Solidago gigantea*	EU337381
Eudicotyledons	Asteraceae	*Solidago*	*Solidago gigantea*	EU337382

Eudicotyledons	Asteraceae	*Solidago*	*Solidago gigantea*	EU337383
Eudicotyledons	Asteraceae	*Solidago*	*Solidago gigantea*	EU337384
Eudicotyledons	Asteraceae	*Solidago*	*Solidago gigantea*	EU337385
Eudicotyledons	Asteraceae	*Solidago*	*Solidago gigantea*	EU337386
Eudicotyledons	Asteraceae	*Solidago*	*Solidago gigantea*	EU337387
Eudicotyledons	Asteraceae	*Solidago*	*Solidago gigantea*	EU337388
Eudicotyledons	Asteraceae	*Solidago*	*Solidago gigantea*	EU337389
Eudicotyledons	Asteraceae	*Solidago*	*Solidago gigantea*	EU337390
Eudicotyledons	Asteraceae	*Solidago*	*Solidago gigantea*	EU337391
Eudicotyledons	Asteraceae	*Solidago*	*Solidago gigantea*	EU337392
Eudicotyledons	Asteraceae	*Solidago*	*Solidago gigantea*	DQ006153
Eudicotyledons	Asteraceae	*Solidago*	*Solidago gigantea*	HQ142551
Eudicotyledons	Asteraceae	*Solidago*	*Solidago gigantea*	HQ142552
Eudicotyledons	Asteraceae	*Solidago*	*Solidago gigantea*	HQ142553
Eudicotyledons	Asteraceae	*Solidago*	*Solidago gigantea*	HQ142554
Eudicotyledons	Asteraceae	*Solidago*	*Solidago juncea*	DQ006154
Eudicotyledons	Asteraceae	*Solidago*	*Solidago juncea*	HQ142560
Eudicotyledons	Asteraceae	*Solidago*	*Solidago simplex*	DQ006155
Eudicotyledons	Asteraceae	*Solidago*	*Solidago simplex*	HQ142543
Eudicotyledons	Araliaceae	*Dendropanax*	*Dendropanax sessiliflorus*	GU054812
Eudicotyledons	Araliaceae	*Dendropanax*	*Dendropanax sessiliflorus*	GU054826
Eudicotyledons	Araliaceae	*Dendropanax*	*Dendropanax sessiliflorus*	GU054881
Eudicotyledons	Brassicaceae	*Cardamine*	*Cardamine nipponica*	AB365513
Eudicotyledons	Brassicaceae	*Cardamine*	*Cardamine nipponica*	AB365514
Eudicotyledons	Brassicaceae	*Cardamine*	*Cardamine nipponica*	AB365515
Eudicotyledons	Brassicaceae	*Cardamine*	*Cardamine nipponica*	AB365516
Eudicotyledons	Fabaceae	*Senna*	*Senna obtusifolia*	GU396802
Eudicotyledons	Fabaceae	*Senna*	*Senna obtusifolia*	GU396803
Eudicotyledons	Fabaceae	*Senna*	*Senna obtusifolia*	GU396804
Eudicotyledons	Fabaceae	*Senna*	*Senna obtusifolia*	GU396805
Eudicotyledons	Fabaceae	*Senna*	*Senna obtusifolia*	GQ435431
Eudicotyledons	Fabaceae	*Senna*	*Senna obtusifolia*	GQ435432
Eudicotyledons	Fabaceae	*Senna*	*Senna pallida*	JF838364
Eudicotyledons	Fabaceae	*Senna*	*Senna pallida*	HQ161754
Eudicotyledons	Fabaceae	*Senna*	*Senna siamea*	GU969279
Eudicotyledons	Fabaceae	*Senna*	*Senna siamea*	HQ161767
Eudicotyledons	Fabaceae	*Senna*	*Senna spectabilis*	JF838360
Eudicotyledons	Fabaceae	*Senna*	*Senna spectabilis*	HQ161761
Eudicotyledons	Fabaceae	*Caesalpinia*	*Caesalpinia oyamae*	DQ208785
Eudicotyledons	Fabaceae	*Caesalpinia*	*Caesalpinia oyamae*	DQ208786
Eudicotyledons	Fabaceae	*Caesalpinia*	*Caesalpinia oyamae*	DQ208787
Eudicotyledons	Fabaceae	*Caesalpinia*	*Caesalpinia oyamae*	DQ208788
Eudicotyledons	Fabaceae	*Caesalpinia*	*Caesalpinia oyamae*	DQ208789
Eudicotyledons	Fabaceae	*Caesalpinia*	*Caesalpinia oyamae*	DQ208790
Eudicotyledons	Fabaceae	*Caesalpinia*	*Caesalpinia oyamae*	DQ208791
Eudicotyledons	Fabaceae	*Caesalpinia*	*Caesalpinia oyamae*	DQ208792
Eudicotyledons	Fabaceae	*Caesalpinia*	*Caesalpinia oyamae*	DQ208793
Eudicotyledons	Fabaceae	*Caesalpinia*	*Caesalpinia oyamae*	DQ208794
Eudicotyledons	Fabaceae	*Caesalpinia*	*Caesalpinia oyamae*	DQ208795
Eudicotyledons	Fabaceae	*Caesalpinia*	*Caesalpinia oyamae*	DQ208796
Eudicotyledons	Fabaceae	*Caesalpinia*	*Caesalpinia oyamae*	DQ208797
Eudicotyledons	Fabaceae	*Caesalpinia*	*Caesalpinia oyamae*	DQ208798
Eudicotyledons	Fabaceae	*Caesalpinia*	*Caesalpinia oyamae*	DQ208799

Eudicotyledons	Fabaceae	*Caesalpinia*	*Caesalpinia oyamae*	DQ208800
Eudicotyledons	Fabaceae	*Caesalpinia*	*Caesalpinia oyamae*	DQ208801
Eudicotyledons	Fabaceae	*Caesalpinia*	*Caesalpinia oyamae*	DQ208802
Eudicotyledons	Fabaceae	*Caesalpinia*	*Caesalpinia oyamae*	DQ208803
Eudicotyledons	Fabaceae	*Caesalpinia*	*Caesalpinia oyamae*	DQ208804
Eudicotyledons	Fabaceae	*Caesalpinia*	*Caesalpinia oyamae*	DQ208805
Eudicotyledons	Fabaceae	*Caesalpinia*	*Caesalpinia oyamae*	DQ208806
Eudicotyledons	Fabaceae	*Caesalpinia*	*Caesalpinia oyamae*	DQ208807
Eudicotyledons	Fabaceae	*Caesalpinia*	*Caesalpinia oyamae*	DQ208808
Eudicotyledons	Fabaceae	*Caesalpinia*	*Caesalpinia epifanioi*	DQ208825
Eudicotyledons	Fabaceae	*Caesalpinia*	*Caesalpinia epifanioi*	DQ208826
Eudicotyledons	Fabaceae	*Caesalpinia*	*Caesalpinia epifanioi*	DQ208827
Eudicotyledons	Fabaceae	*Caesalpinia*	*Caesalpinia epifanioi*	DQ208828
Eudicotyledons	Fabaceae	*Caesalpinia*	*Caesalpinia epifanioi*	DQ208829
Eudicotyledons	Fabaceae	*Caesalpinia*	*Caesalpinia epifanioi*	DQ208830
Eudicotyledons	Fabaceae	*Caesalpinia*	*Caesalpinia melanadenia*	DQ208831
Eudicotyledons	Fabaceae	*Caesalpinia*	*Caesalpinia melanadenia*	DQ208832
Eudicotyledons	Fabaceae	*Caesalpinia*	*Caesalpinia melanadenia*	DQ208833
Eudicotyledons	Fabaceae	*Caesalpinia*	*Caesalpinia melanadenia*	DQ208834
Eudicotyledons	Fabaceae	*Caesalpinia*	*Caesalpinia melanadenia*	DQ208835
Eudicotyledons	Fabaceae	*Caesalpinia*	*Caesalpinia macvaughii*	DQ208836
Eudicotyledons	Fabaceae	*Caesalpinia*	*Caesalpinia macvaughii*	DQ208837
Eudicotyledons	Fabaceae	*Caesalpinia*	*Caesalpinia macvaughii*	DQ208838
Eudicotyledons	Fabaceae	*Caesalpinia*	*Caesalpinia macvaughii*	DQ208839
Eudicotyledons	Fabaceae	*Caesalpinia*	*Caesalpinia macvaughii*	DQ208840
Eudicotyledons	Fabaceae	*Caesalpinia*	*Caesalpinia macvaughii*	DQ208841
Eudicotyledons	Fabaceae	*Caesalpinia*	*Caesalpinia macvaughii*	DQ208842
Eudicotyledons	Fabaceae	*Caesalpinia*	*Caesalpinia macvaughii*	DQ208843
Eudicotyledons	Apiaceae	*Peucedanum*	*Peucedanum ledebourielloides*	JN046222
Eudicotyledons	Apiaceae	*Peucedanum*	*Peucedanum ledebourielloides*	JN046223
Eudicotyledons	Apiaceae	*Peucedanum*	*Peucedanum ledebourielloides*	JN046224
Eudicotyledons	Lamiaceae	*Vitex*	*Vitex negundo*	JQ319673
Eudicotyledons	Lamiaceae	*Vitex*	*Vitex negundo*	DQ304781
Eudicotyledons	Lamiaceae	*Vitex*	*Vitex negundo*	DQ304782
Eudicotyledons	Lamiaceae	*Vitex*	*Vitex negundo*	DQ304783
Eudicotyledons	Fabaceae	*Senna*	*Senna tora*	GU969277
Eudicotyledons	Fabaceae	*Senna*	*Senna tora*	HQ161762
Eudicotyledons	Caprifoliaceae	*Lonicera*	*Lonicera confusa*	HM228523
Eudicotyledons	Caprifoliaceae	*Lonicera*	*Lonicera confusa*	HM228524
Eudicotyledons	Caprifoliaceae	*Lonicera*	*Lonicera confusa*	HM228525
Eudicotyledons	Caprifoliaceae	*Lonicera*	*Lonicera confusa*	HM228526
Eudicotyledons	Caprifoliaceae	*Lonicera*	*Lonicera confusa*	HM228527
Eudicotyledons	Caprifoliaceae	*Lonicera*	*Lonicera confusa*	HM228528
Eudicotyledons	Caprifoliaceae	*Lonicera*	*Lonicera confusa*	HM228529
Eudicotyledons	Primulaceae	*Primula*	*Primula calliantha*	JN046490
Eudicotyledons	Primulaceae	*Primula*	*Primula calliantha*	JN046491
Eudicotyledons	Primulaceae	*Primula*	*Primula calliantha*	JN046492
Eudicotyledons	Primulaceae	*Primula*	*Primula chionantha*	JN046495
Eudicotyledons	Primulaceae	*Primula*	*Primula chionantha*	JN046496
Eudicotyledons	Primulaceae	*Primula*	*Primula chionantha*	JN046497
Eudicotyledons	Primulaceae	*Primula*	*Primula chionantha*	JN046498
Eudicotyledons	Primulaceae	*Primula*	*Primula chionantha*	JN046499
Eudicotyledons	Primulaceae	*Primula*	*Primula deflexa*	JN046500

Eudicotyledons	Primulaceae	*Primula*	*Primula deflexa*	JN046501
Eudicotyledons	Primulaceae	*Primula*	*Primula deflexa*	JN046502
Eudicotyledons	Primulaceae	*Primula*	*Primula deflexa*	JN046503
Eudicotyledons	Primulaceae	*Primula*	*Primula fasciculata*	JN046519
Eudicotyledons	Primulaceae	*Primula*	*Primula fasciculata*	JN046520
Eudicotyledons	Primulaceae	*Primula*	*Primula fasciculata*	JN046521
Eudicotyledons	Primulaceae	*Primula*	*Primula heucherifolia*	JN046526
Eudicotyledons	Primulaceae	*Primula*	*Primula heucherifolia*	JN046527
Eudicotyledons	Apocynaceae	*Fockea*	*Fockea angustifolia*	AM231758
Eudicotyledons	Apocynaceae	*Fockea*	*Fockea angustifolia*	AM231759
Eudicotyledons	Apocynaceae	*Fockea*	*Fockea angustifolia*	AM231760
Eudicotyledons	Apocynaceae	*Fockea*	*Fockea angustifolia*	AM231761
Eudicotyledons	Apocynaceae	*Fockea*	*Fockea comaru*	AM231763
Eudicotyledons	Apocynaceae	*Fockea*	*Fockea comaru*	AM231764
Eudicotyledons	Orobanchaceae	*Agalinis*	*Agalinis purpurea*	EU827941
Eudicotyledons	Orobanchaceae	*Agalinis*	*Agalinis purpurea*	EU827942
Eudicotyledons	Orobanchaceae	*Agalinis*	*Agalinis purpurea*	EU827943
Eudicotyledons	Orobanchaceae	*Agalinis*	*Agalinis purpurea*	EU827944
Eudicotyledons	Amaranthaceae	*Suaeda*	*Suaeda glauca*	JN047266
Eudicotyledons	Amaranthaceae	*Suaeda*	*Suaeda glauca*	JN047267
Eudicotyledons	Amaranthaceae	*Suaeda*	*Suaeda glauca*	JN047268
Eudicotyledons	Amaranthaceae	*Suaeda*	*Suaeda glauca*	JN047269
Eudicotyledons	Lamiaceae	*Vitex*	*Vitex rotundifolia*	JQ319674
Eudicotyledons	Lamiaceae	*Vitex*	*Vitex rotundifolia*	GQ435184
Eudicotyledons	Phyllanthaceae	*Phyllanthus*	*Phyllanthus fraternus*	GU598566
Eudicotyledons	Phyllanthaceae	*Phyllanthus*	*Phyllanthus fraternus*	GU598569
Eudicotyledons	Polygonaceae	*Persicaria*	*Persicaria lapathifolia*	EF653755
Eudicotyledons	Polygonaceae	*Persicaria*	*Persicaria lapathifolia*	EU196983
Eudicotyledons	Polygonaceae	*Persicaria*	*Persicaria lapathifolia*	EU196984
Eudicotyledons	Polygonaceae	*Persicaria*	*Persicaria lapathifolia*	FJ503036
Eudicotyledons	Ranunculaceae	*Pulsatilla*	*Pulsatilla vernalis*	EF597126
Eudicotyledons	Ranunculaceae	*Pulsatilla*	*Pulsatilla vernalis*	EF597127
Eudicotyledons	Ranunculaceae	*Pulsatilla*	*Pulsatilla vernalis*	EF597128
Eudicotyledons	Ranunculaceae	*Pulsatilla*	*Pulsatilla vernalis*	EF597129
Eudicotyledons	Ranunculaceae	*Pulsatilla*	*Pulsatilla vernalis*	EF597130
Eudicotyledons	Ranunculaceae	*Pulsatilla*	*Pulsatilla vernalis*	EF597131
Eudicotyledons	Ranunculaceae	*Pulsatilla*	*Pulsatilla vernalis*	EF597132
Eudicotyledons	Ranunculaceae	*Pulsatilla*	*Pulsatilla vernalis*	EF597133
Eudicotyledons	Ranunculaceae	*Pulsatilla*	*Pulsatilla vernalis*	EF597144
Eudicotyledons	Ranunculaceae	*Pulsatilla*	*Pulsatilla vernalis*	EF597145
Eudicotyledons	Ranunculaceae	*Pulsatilla*	*Pulsatilla vulgaris*	EF597134
Eudicotyledons	Ranunculaceae	*Pulsatilla*	*Pulsatilla vulgaris*	EF597136
Eudicotyledons	Malvaceae	*Lavatera*	*Lavatera flava*	EF419551
Eudicotyledons	Malvaceae	*Lavatera*	*Lavatera flava*	EF419552
Eudicotyledons	Malvaceae	*Lavatera*	*Lavatera maroccana*	EF419563
Eudicotyledons	Malvaceae	*Lavatera*	*Lavatera maroccana*	EF419564
Eudicotyledons	Malvaceae	*Lavatera*	*Lavatera mauritanica*	EF419581
Eudicotyledons	Malvaceae	*Lavatera*	*Lavatera mauritanica*	EF419582
Eudicotyledons	Malvaceae	*Lavatera*	*Lavatera mauritanica*	EF419583
Eudicotyledons	Malvaceae	*Lavatera*	*Lavatera mauritanica*	EF419584
Eudicotyledons	Malvaceae	*Lavatera*	*Lavatera punctata*	EF419565
Eudicotyledons	Malvaceae	*Lavatera*	*Lavatera punctata*	EF419566
Eudicotyledons	Caprifoliaceae	*Lonicera*	*Lonicera hispida*	JN045246

Eudicotyledons	Caprifoliaceae	*Lonicera*	*Lonicera hispida*	JN045247
Eudicotyledons	Caprifoliaceae	*Lonicera*	*Lonicera hispida*	JN045248
Eudicotyledons	Caprifoliaceae	*Lonicera*	*Lonicera hispida*	JN045249
Eudicotyledons	Caprifoliaceae	*Lonicera*	*Lonicera hispida*	JN045250
Eudicotyledons	Caprifoliaceae	*Lonicera*	*Lonicera rupicola*	JN045255
Eudicotyledons	Caprifoliaceae	*Lonicera*	*Lonicera rupicola*	JN045256
Eudicotyledons	Caprifoliaceae	*Lonicera*	*Lonicera rupicola*	JN045257
Eudicotyledons	Caprifoliaceae	*Lonicera*	*Lonicera rupicola*	JN045258
Eudicotyledons	Caprifoliaceae	*Lonicera*	*Lonicera rupicola*	JN045259
Eudicotyledons	Polygonaceae	*Persicaria*	*Persicaria nepalensis*	EF653742
Eudicotyledons	Polygonaceae	*Persicaria*	*Persicaria nepalensis*	JN046437
Eudicotyledons	Polygonaceae	*Persicaria*	*Persicaria nepalensis*	JN046438
Eudicotyledons	Polygonaceae	*Persicaria*	*Persicaria nepalensis*	JN046439
Eudicotyledons	Asteraceae	*Leucogenes*	*Leucogenes neglecta*	EU007665
Eudicotyledons	Asteraceae	*Leucogenes*	*Leucogenes neglecta*	EU007666
Eudicotyledons	Asteraceae	*Leucogenes*	*Leucogenes neglecta*	EU007667
Eudicotyledons	Asteraceae	*Leucogenes*	*Leucogenes neglecta*	EU007668
Eudicotyledons	Lamiaceae	*Lamium*	*Lamium tomentosum*	JF780184
Eudicotyledons	Lamiaceae	*Lamium*	*Lamium tomentosum*	JF780185
Eudicotyledons	Lamiaceae	*Lamium*	*Lamium tomentosum*	JF780186
Eudicotyledons	Lamiaceae	*Lamium*	*Lamium tomentosum*	JF780187
Eudicotyledons	Polygonaceae	*Persicaria*	*Persicaria maculosa*	EU196988
Eudicotyledons	Polygonaceae	*Persicaria*	*Persicaria maculosa*	EU750492
Eudicotyledons	Polygonaceae	*Persicaria*	*Persicaria maculosa*	EU750493
Eudicotyledons	Polygonaceae	*Persicaria*	*Persicaria maculosa*	EU750494
Eudicotyledons	Rubiaceae	*Hedyotis*	*Hedyotis auricularia*	JF699765
Eudicotyledons	Rubiaceae	*Hedyotis*	*Hedyotis auricularia*	JF699766
Eudicotyledons	Rubiaceae	*Hedyotis*	*Hedyotis auricularia*	JN044758
Eudicotyledons	Rubiaceae	*Hedyotis*	*Hedyotis auricularia*	JN044759
Eudicotyledons	Rubiaceae	*Hedyotis*	*Hedyotis biflora*	JF699767
Eudicotyledons	Rubiaceae	*Hedyotis*	*Hedyotis biflora*	JF699768
Eudicotyledons	Rubiaceae	*Hedyotis*	*Hedyotis biflora*	JF699769
Eudicotyledons	Rubiaceae	*Hedyotis*	*Hedyotis costata*	JF699787
Eudicotyledons	Rubiaceae	*Hedyotis*	*Hedyotis costata*	JN044773
Eudicotyledons	Rubiaceae	*Hedyotis*	*Hedyotis costata*	JN044774
Eudicotyledons	Rubiaceae	*Hedyotis*	*Hedyotis effusa*	JF699790
Eudicotyledons	Rubiaceae	*Hedyotis*	*Hedyotis effusa*	JF699791
Eudicotyledons	Rubiaceae	*Hedyotis*	*Hedyotis effusa*	JN044775
Eudicotyledons	Rubiaceae	*Hedyotis*	*Hedyotis effusa*	JN044776
Eudicotyledons	Rubiaceae	*Hedyotis*	*Hedyotis verticillata*	JF699816
Eudicotyledons	Rubiaceae	*Hedyotis*	*Hedyotis verticillata*	JF699817
Eudicotyledons	Rubiaceae	*Hedyotis*	*Hedyotis verticillata*	JF699818
Eudicotyledons	Rubiaceae	*Hedyotis*	*Hedyotis verticillata*	JF699819
Eudicotyledons	Rubiaceae	*Hedyotis*	*Hedyotis yangchunensis*	JF699821
Eudicotyledons	Rubiaceae	*Hedyotis*	*Hedyotis yangchunensis*	JF699822
Eudicotyledons	Rubiaceae	*Hedyotis*	*Hedyotis yangchunensis*	JF699823
Eudicotyledons	Rubiaceae	*Hedyotis*	*Hedyotis yangchunensis*	JN044791
Eudicotyledons	Rubiaceae	*Hedyotis*	*Hedyotis yangchunensis*	JN044792
Eudicotyledons	Asteraceae	*Solidago*	*Solidago virgaurea*	EU337703
Eudicotyledons	Asteraceae	*Solidago*	*Solidago virgaurea*	EU337704
Eudicotyledons	Asteraceae	*Solidago*	*Solidago virgaurea*	EU337705
Eudicotyledons	Polygonaceae	*Persicaria*	*Persicaria glabra*	EU196973
Eudicotyledons	Polygonaceae	*Persicaria*	*Persicaria glabra*	EU196974

Eudicotyledons	Asteraceae	*Solidago*	*Solidago caesia*	EU750561
Eudicotyledons	Asteraceae	*Solidago*	*Solidago caesia*	EU750562
Eudicotyledons	Asteraceae	*Solidago*	*Solidago caesia*	HQ142546
Eudicotyledons	Asteraceae	*Solidago*	*Solidago nemoralis*	EU750567
Eudicotyledons	Asteraceae	*Solidago*	*Solidago nemoralis*	EU750568
Eudicotyledons	Asteraceae	*Solidago*	*Solidago nemoralis*	EU750569
Eudicotyledons	Asteraceae	*Solidago*	*Solidago nemoralis*	HQ142555
Eudicotyledons	Brassicaceae	*Pugionium*	*Pugionium dolabratum*	JN046694
Eudicotyledons	Brassicaceae	*Pugionium*	*Pugionium dolabratum*	JN046695
Eudicotyledons	Brassicaceae	*Pugionium*	*Pugionium dolabratum*	JN046696
Eudicotyledons	Brassicaceae	*Pugionium*	*Pugionium dolabratum*	JN046697
Eudicotyledons	Brassicaceae	*Pugionium*	*Pugionium dolabratum*	JN046698
Eudicotyledons	Fabaceae	*Caesalpinia*	*Caesalpinia sappan*	GU396795
Eudicotyledons	Fabaceae	*Caesalpinia*	*Caesalpinia sappan*	GQ435369
Eudicotyledons	Fabaceae	*Caesalpinia*	*Caesalpinia sappan*	GQ435370
Eudicotyledons	Polygonaceae	*Persicaria*	*Persicaria hirsuta*	EU196975
Eudicotyledons	Polygonaceae	*Persicaria*	*Persicaria hirsuta*	EU196976
Eudicotyledons	Polygonaceae	*Persicaria*	*Persicaria hydropiperoides*	EU196978
Eudicotyledons	Polygonaceae	*Persicaria*	*Persicaria hydropiperoides*	EU196979
Eudicotyledons	Polygonaceae	*Persicaria*	*Persicaria hydropiperoides*	EU196980
Eudicotyledons	Polygonaceae	*Persicaria*	*Persicaria minor*	EU196990
Eudicotyledons	Polygonaceae	*Persicaria*	*Persicaria minor*	EU196991
Eudicotyledons	Polygonaceae	*Persicaria*	*Persicaria viscosa*	EU197008
Eudicotyledons	Polygonaceae	*Persicaria*	*Persicaria viscosa*	JF708227
Eudicotyledons	Apiaceae	*Pleurospermum*	*Pleurospermum franchetianum*	JN046387
Eudicotyledons	Apiaceae	*Pleurospermum*	*Pleurospermum franchetianum*	JN046388
Eudicotyledons	Fabaceae	*Dalbergia*	*Dalbergia odorifera*	GU396739
Eudicotyledons	Fabaceae	*Dalbergia*	*Dalbergia odorifera*	GQ434964
Eudicotyledons	Asteraceae	*Solidago*	*Solidago lepida*	EU337701
Eudicotyledons	Asteraceae	*Solidago*	*Solidago lepida*	EU337702
Eudicotyledons	Asteraceae	*Soroseris*	*Soroseris glomerata*	HQ436182
Eudicotyledons	Asteraceae	*Soroseris*	*Soroseris glomerata*	HQ436183
Eudicotyledons	Asteraceae	*Soroseris*	*Soroseris glomerata*	HQ436184
Eudicotyledons	Asteraceae	*Soroseris*	*Soroseris glomerata*	JN047247
Eudicotyledons	Asteraceae	*Soroseris*	*Soroseris glomerata*	JN047248
Eudicotyledons	Asteraceae	*Solidago*	*Solidago rugosa*	JQ246439
Eudicotyledons	Asteraceae	*Solidago*	*Solidago rugosa*	HQ596852
Eudicotyledons	Asteraceae	*Solidago*	*Solidago rugosa*	EU750570
Eudicotyledons	Asteraceae	*Solidago*	*Solidago rugosa*	EU750571
Eudicotyledons	Asteraceae	*Solidago*	*Solidago rugosa*	EU750572
Eudicotyledons	Asteraceae	*Solidago*	*Solidago rugosa*	HQ142557
Eudicotyledons	Polygonaceae	*Polygonum*	*Polygonum sinomontanum*	JN046445
Eudicotyledons	Polygonaceae	*Polygonum*	*Polygonum sinomontanum*	JN046446
Eudicotyledons	Orobanchaceae	*Agalinis*	*Agalinis decemloba*	GU943533
Eudicotyledons	Orobanchaceae	*Agalinis*	*Agalinis decemloba*	GU943534
Eudicotyledons	Orobanchaceae	*Agalinis*	*Agalinis decemloba*	GU943535
Eudicotyledons	Orobanchaceae	*Agalinis*	*Agalinis decemloba*	EU827899
Eudicotyledons	Orobanchaceae	*Agalinis*	*Agalinis decemloba*	EU827900
Eudicotyledons	Orobanchaceae	*Agalinis*	*Agalinis gattingeri*	EU827909
Eudicotyledons	Orobanchaceae	*Agalinis*	*Agalinis gattingeri*	EU827910
Eudicotyledons	Orobanchaceae	*Agalinis*	*Agalinis gattingeri*	EU827911
Eudicotyledons	Orobanchaceae	*Agalinis*	*Agalinis harperi*	EU827912
Eudicotyledons	Orobanchaceae	*Agalinis*	*Agalinis harperi*	EU827913

Eudicotyledons	Orobanchaceae	*Agalinis*	*Agalinis homalantha*	EU827916
Eudicotyledons	Orobanchaceae	*Agalinis*	*Agalinis homalantha*	EU827917
Eudicotyledons	Orobanchaceae	*Agalinis*	*Agalinis laxa*	EU827918
Eudicotyledons	Orobanchaceae	*Agalinis*	*Agalinis laxa*	EU827919
Eudicotyledons	Orobanchaceae	*Agalinis*	*Agalinis maritima*	EU827897
Eudicotyledons	Orobanchaceae	*Agalinis*	*Agalinis maritima*	EU827922
Eudicotyledons	Orobanchaceae	*Agalinis*	*Agalinis navasotensis*	EU827923
Eudicotyledons	Orobanchaceae	*Agalinis*	*Agalinis navasotensis*	EU827924
Eudicotyledons	Orobanchaceae	*Agalinis*	*Agalinis oligophylla*	EU827931
Eudicotyledons	Orobanchaceae	*Agalinis*	*Agalinis oligophylla*	EU827932
Eudicotyledons	Orobanchaceae	*Agalinis*	*Agalinis oligophylla*	EU827933
Eudicotyledons	Orobanchaceae	*Agalinis*	*Agalinis oligophylla*	EU827934
Eudicotyledons	Orobanchaceae	*Agalinis*	*Agalinis paupercula*	EU827935
Eudicotyledons	Orobanchaceae	*Agalinis*	*Agalinis paupercula*	EU827936
Eudicotyledons	Orobanchaceae	*Agalinis*	*Agalinis skinneriana*	EU827947
Eudicotyledons	Orobanchaceae	*Agalinis*	*Agalinis skinneriana*	EU827948
Eudicotyledons	Orobanchaceae	*Agalinis*	*Agalinis skinneriana*	EU827949
Eudicotyledons	Orobanchaceae	*Agalinis*	*Agalinis viridis*	EU827959
Eudicotyledons	Orobanchaceae	*Agalinis*	*Agalinis viridis*	EU827960
Eudicotyledons	Fabaceae	*Lespedeza*	*Lespedeza bicolor*	GU396760
Eudicotyledons	Fabaceae	*Lespedeza*	*Lespedeza bicolor*	GU572290
Eudicotyledons	Brassicaceae	*Pugionium*	*Pugionium cornutum*	JN046689
Eudicotyledons	Brassicaceae	*Pugionium*	*Pugionium cornutum*	JN046690
Eudicotyledons	Brassicaceae	*Pugionium*	*Pugionium cornutum*	JN046691
Eudicotyledons	Brassicaceae	*Pugionium*	*Pugionium cornutum*	JN046692
Eudicotyledons	Brassicaceae	*Pugionium*	*Pugionium cornutum*	JN046693
Eudicotyledons	Brassicaceae	*Cardamine*	*Cardamine tangutorum*	JN044169
Eudicotyledons	Brassicaceae	*Cardamine*	*Cardamine tangutorum*	JN044170
Eudicotyledons	Brassicaceae	*Cardamine*	*Cardamine tangutorum*	JN044171
Eudicotyledons	Brassicaceae	*Cardamine*	*Cardamine tangutorum*	JN044172
Eudicotyledons	Caprifoliaceae	*Lonicera*	*Lonicera acuminata*	HM228516
Eudicotyledons	Caprifoliaceae	*Lonicera*	*Lonicera acuminata*	HM228517
Eudicotyledons	Caprifoliaceae	*Lonicera*	*Lonicera trichosantha*	JN045260
Eudicotyledons	Caprifoliaceae	*Lonicera*	*Lonicera trichosantha*	JN045261
Eudicotyledons	Gesneriaceae	*Cyrtandra*	*Cyrtandra anthropophagorum*	GQ475119
Eudicotyledons	Gesneriaceae	*Cyrtandra*	*Cyrtandra anthropophagorum*	GQ475120
Eudicotyledons	Gesneriaceae	*Cyrtandra*	*Cyrtandra anthropophagorum*	EU920042
Eudicotyledons	Gesneriaceae	*Cyrtandra*	*Cyrtandra calpidicarpa*	GQ475123
Eudicotyledons	Gesneriaceae	*Cyrtandra*	*Cyrtandra calpidicarpa*	GQ475164
Eudicotyledons	Gesneriaceae	*Cyrtandra*	*Cyrtandra calpidicarpa*	EU920010
Eudicotyledons	Gesneriaceae	*Cyrtandra*	*Cyrtandra feaniana*	GQ475130
Eudicotyledons	Gesneriaceae	*Cyrtandra*	*Cyrtandra feaniana*	EU920019
Eudicotyledons	Gesneriaceae	*Cyrtandra*	*Cyrtandra kaulantha*	GQ475160
Eudicotyledons	Gesneriaceae	*Cyrtandra*	*Cyrtandra kaulantha*	EU920011
Eudicotyledons	Gesneriaceae	*Cyrtandra*	*Cyrtandra munroi*	GQ475134
Eudicotyledons	Gesneriaceae	*Cyrtandra*	*Cyrtandra munroi*	GQ475155
Eudicotyledons	Gesneriaceae	*Cyrtandra*	*Cyrtandra richii*	GQ475127
Eudicotyledons	Gesneriaceae	*Cyrtandra*	*Cyrtandra richii*	EU920028
Eudicotyledons	Gesneriaceae	*Cyrtandra*	*Cyrtandra sp. Plunkett 1843*	GQ475118
Eudicotyledons	Gesneriaceae	*Cyrtandra*	*Cyrtandra sp. Plunkett 1843*	EU919993
Eudicotyledons	Gesneriaceae	*Cyrtandra*	*Cyrtandra wagneri*	GQ475115
Eudicotyledons	Gesneriaceae	*Cyrtandra*	*Cyrtandra wagneri*	GQ475116
Eudicotyledons	Gesneriaceae	*Cyrtandra*	*Cyrtandra wagneri*	EU919991

Eudicotyledons	Fabaceae	*Lespedeza*	*Lespedeza floribunda*	GU396761
Eudicotyledons	Fabaceae	*Lespedeza*	*Lespedeza floribunda*	GU572299
Eudicotyledons	Hydrangeaceae	*Schizophragma*	*Schizophragma integrifolium*	FJ647143
Eudicotyledons	Hydrangeaceae	*Schizophragma*	*Schizophragma integrifolium*	HM217002
Eudicotyledons	Fabaceae	*Dalbergia*	*Dalbergia tonkinensis*	FR854163
Eudicotyledons	Fabaceae	*Dalbergia*	*Dalbergia tonkinensis*	FR854164
Eudicotyledons	Fabaceae	*Dalbergia*	*Dalbergia tonkinensis*	FR854165
Eudicotyledons	Fabaceae	*Dalbergia*	*Dalbergia tonkinensis*	FR854166
Eudicotyledons	Fabaceae	*Dalbergia*	*Dalbergia tonkinensis*	FR854167
Eudicotyledons	Orobanchaceae	*Castilleja*	*Castilleja rhexiifolia*	FJ765695
Eudicotyledons	Orobanchaceae	*Castilleja*	*Castilleja rhexiifolia*	FJ765696
Eudicotyledons	Orobanchaceae	*Castilleja*	*Castilleja rhexiifolia*	FJ765697
Eudicotyledons	Orobanchaceae	*Castilleja*	*Castilleja rhexiifolia*	FJ765698
Eudicotyledons	Orobanchaceae	*Castilleja*	*Castilleja rhexiifolia*	FJ765699
Eudicotyledons	Orobanchaceae	*Castilleja*	*Castilleja rhexiifolia*	FJ765700
Eudicotyledons	Orobanchaceae	*Castilleja*	*Castilleja rhexiifolia*	FJ765701
Eudicotyledons	Orobanchaceae	*Castilleja*	*Castilleja rhexiifolia*	FJ765702
Eudicotyledons	Orobanchaceae	*Castilleja*	*Castilleja rhexiifolia*	FJ765703
Eudicotyledons	Orobanchaceae	*Castilleja*	*Castilleja rhexiifolia*	FJ765704
Eudicotyledons	Orobanchaceae	*Castilleja*	*Castilleja rhexiifolia*	FJ765705
Eudicotyledons	Orobanchaceae	*Castilleja*	*Castilleja rhexiifolia*	FJ765706
Eudicotyledons	Orobanchaceae	*Castilleja*	*Castilleja rhexiifolia*	FJ765707
Eudicotyledons	Orobanchaceae	*Castilleja*	*Castilleja rhexiifolia*	FJ765708
Eudicotyledons	Rosaceae	*Dasiphora*	*Dasiphora davurica*	JN044366
Eudicotyledons	Rosaceae	*Dasiphora*	*Dasiphora davurica*	JN044367
Eudicotyledons	Rosaceae	*Dasiphora*	*Dasiphora davurica*	JN044368
Eudicotyledons	Rosaceae	*Dasiphora*	*Dasiphora davurica*	JN044369
Eudicotyledons	Rosaceae	*Dasiphora*	*Dasiphora davurica*	JN044370
Eudicotyledons	Rosaceae	*Dasiphora*	*Dasiphora davurica*	JN044371
Eudicotyledons	Rosaceae	*Dasiphora*	*Dasiphora davurica*	JN044372
Eudicotyledons	Caprifoliaceae	*Lonicera*	*Lonicera hypoglauca*	JN045251
Eudicotyledons	Caprifoliaceae	*Lonicera*	*Lonicera hypoglauca*	HM228518
Eudicotyledons	Caprifoliaceae	*Lonicera*	*Lonicera hypoglauca*	HM228519
Eudicotyledons	Caprifoliaceae	*Lonicera*	*Lonicera hypoglauca*	HM228520
Eudicotyledons	Caprifoliaceae	*Lonicera*	*Lonicera hypoglauca*	HM228521
Eudicotyledons	Caprifoliaceae	*Lonicera*	*Lonicera hypoglauca*	HM228522
Eudicotyledons	Caprifoliaceae	*Lonicera*	*Lonicera macranthoides*	GQ435288
Eudicotyledons	Caprifoliaceae	*Lonicera*	*Lonicera macranthoides*	HM228530
Eudicotyledons	Caprifoliaceae	*Lonicera*	*Lonicera macranthoides*	HM228531
Eudicotyledons	Caprifoliaceae	*Lonicera*	*Lonicera macranthoides*	HM228532
Eudicotyledons	Caprifoliaceae	*Lonicera*	*Lonicera macranthoides*	HM228533
Eudicotyledons	Caprifoliaceae	*Lonicera*	*Lonicera macranthoides*	HM228534
Eudicotyledons	Caprifoliaceae	*Lonicera*	*Lonicera macranthoides*	HM228535
Eudicotyledons	Caprifoliaceae	*Lonicera*	*Lonicera fulvotomentosa*	GQ435472
Eudicotyledons	Caprifoliaceae	*Lonicera*	*Lonicera fulvotomentosa*	HM228550
Eudicotyledons	Caprifoliaceae	*Lonicera*	*Lonicera fulvotomentosa*	HM228551
Eudicotyledons	Caprifoliaceae	*Lonicera*	*Lonicera fulvotomentosa*	HM228552
Eudicotyledons	Gesneriaceae	*Cyrtandra*	*Cyrtandra platyphylla*	GQ475154
Eudicotyledons	Gesneriaceae	*Cyrtandra*	*Cyrtandra platyphylla*	GQ475156
Eudicotyledons	Ranunculaceae	*Aconitum*	*Aconitum coreanum*	GQ337795
Eudicotyledons	Ranunculaceae	*Aconitum*	*Aconitum coreanum*	GQ337796
Eudicotyledons	Ranunculaceae	*Aconitum*	*Aconitum coreanum*	GQ337797
Eudicotyledons	Ranunculaceae	*Aconitum*	*Aconitum coreanum*	GQ337802

Eudicotyledons	Ranunculaceae	*Aconitum*	*Aconitum coreanum*	GQ337803
Eudicotyledons	Ranunculaceae	*Aconitum*	*Aconitum coreanum*	GQ337804
Eudicotyledons	Ranunculaceae	*Aconitum*	*Aconitum coreanum*	GQ337827
Eudicotyledons	Ranunculaceae	*Aconitum*	*Aconitum coreanum*	GQ337828
Eudicotyledons	Ranunculaceae	*Aconitum*	*Aconitum coreanum*	GQ337829
Eudicotyledons	Ranunculaceae	*Aconitum*	*Aconitum coreanum*	GQ337840
Eudicotyledons	Hypericaceae	*Hypericum*	*Hypericum monogynum*	JN044928
Eudicotyledons	Hypericaceae	*Hypericum*	*Hypericum monogynum*	JN044929
Eudicotyledons	Fabaceae	*Lespedeza*	*Lespedeza chinensis*	GU396815
Eudicotyledons	Fabaceae	*Lespedeza*	*Lespedeza chinensis*	GU572294
Eudicotyledons	Fabaceae	*Lespedeza*	*Lespedeza davurica*	GU396762
Eudicotyledons	Fabaceae	*Lespedeza*	*Lespedeza davurica*	GU572297
Eudicotyledons	Primulaceae	*Primula*	*Primula aromatica*	JN046469
Eudicotyledons	Primulaceae	*Primula*	*Primula aromatica*	JN046470
Eudicotyledons	Primulaceae	*Primula*	*Primula bella*	JN046471
Eudicotyledons	Primulaceae	*Primula*	*Primula bella*	JN046472
Eudicotyledons	Primulaceae	*Primula*	*Primula bella*	JN046473
Eudicotyledons	Primulaceae	*Primula*	*Primula blattariformis*	JN046477
Eudicotyledons	Primulaceae	*Primula*	*Primula blattariformis*	JN046478
Eudicotyledons	Primulaceae	*Primula*	*Primula blattariformis*	JN046479
Eudicotyledons	Primulaceae	*Primula*	*Primula blinii*	JN046480
Eudicotyledons	Primulaceae	*Primula*	*Primula blinii*	JN046481
Eudicotyledons	Primulaceae	*Primula*	*Primula blinii*	JN046482
Eudicotyledons	Primulaceae	*Primula*	*Primula blinii*	JN046483
Eudicotyledons	Primulaceae	*Primula*	*Primula boreiocalliantha*	JN046484
Eudicotyledons	Primulaceae	*Primula*	*Primula boreiocalliantha*	JN046485
Eudicotyledons	Primulaceae	*Primula*	*Primula chapaensis*	JN046493
Eudicotyledons	Primulaceae	*Primula*	*Primula chapaensis*	JN046494
Eudicotyledons	Primulaceae	*Primula*	*Primula helodoxa*	HM018472
Eudicotyledons	Primulaceae	*Primula*	*Primula helodoxa*	HM018473
Eudicotyledons	Primulaceae	*Primula*	*Primula helodoxa*	HM018474
Eudicotyledons	Primulaceae	*Primula*	*Primula helodoxa*	HM018475
Eudicotyledons	Primulaceae	*Primula*	*Primula malvacea*	JN046530
Eudicotyledons	Primulaceae	*Primula*	*Primula malvacea*	JN046531
Eudicotyledons	Primulaceae	*Primula*	*Primula malvacea*	JN046532
Eudicotyledons	Primulaceae	*Primula*	*Primula malvacea*	JN046533
Eudicotyledons	Primulaceae	*Primula*	*Primula moupinensis*	JN046538
Eudicotyledons	Primulaceae	*Primula*	*Primula moupinensis*	JN046539
Eudicotyledons	Primulaceae	*Primula*	*Primula moupinensis*	JN046540
Eudicotyledons	Primulaceae	*Primula*	*Primula moupinensis*	JN046541
Eudicotyledons	Primulaceae	*Primula*	*Primula moupinensis*	JN046542
Eudicotyledons	Primulaceae	*Primula*	*Primula ovalifolia*	HM018512
Eudicotyledons	Primulaceae	*Primula*	*Primula ovalifolia*	JN046552
Eudicotyledons	Primulaceae	*Primula*	*Primula ovalifolia*	JN046553
Eudicotyledons	Primulaceae	*Primula*	*Primula ovalifolia*	JN046554
Eudicotyledons	Primulaceae	*Primula*	*Primula ovalifolia*	JN046555
Eudicotyledons	Primulaceae	*Primula*	*Primula ovalifolia*	JN046556
Eudicotyledons	Primulaceae	*Primula*	*Primula partschiana*	JN046557
Eudicotyledons	Primulaceae	*Primula*	*Primula partschiana*	JN046558
Eudicotyledons	Primulaceae	*Primula*	*Primula polyneura*	JN046559
Eudicotyledons	Primulaceae	*Primula*	*Primula polyneura*	JN046560
Eudicotyledons	Primulaceae	*Primula*	*Primula polyneura*	JN046561
Eudicotyledons	Primulaceae	*Primula*	*Primula pycnoloba*	JN046569

Eudicotyledons	Primulaceae	*Primula*	*Primula pycnoloba*	JN046570
Eudicotyledons	Primulaceae	*Primula*	*Primula pycnoloba*	JN046571
Eudicotyledons	Primulaceae	*Primula*	*Primula rugosa*	JN046572
Eudicotyledons	Primulaceae	*Primula*	*Primula rugosa*	JN046573
Eudicotyledons	Primulaceae	*Primula*	*Primula rugosa*	JN046574
Eudicotyledons	Primulaceae	*Primula*	*Primula wangii*	JN046598
Eudicotyledons	Primulaceae	*Primula*	*Primula wangii*	JN046599
Eudicotyledons	Apocynaceae	*Alstonia*	*Alstonia macrophylla*	GU135391
Eudicotyledons	Apocynaceae	*Alstonia*	*Alstonia macrophylla*	GU135392
Eudicotyledons	Apocynaceae	*Alstonia*	*Alstonia macrophylla*	GU135394
Eudicotyledons	Rubiaceae	*Hedyotis*	*Hedyotis shiuyingiae*	JF699808
Eudicotyledons	Rubiaceae	*Hedyotis*	*Hedyotis shiuyingiae*	JF699809
Eudicotyledons	Rubiaceae	*Hedyotis*	*Hedyotis shiuyingiae*	JN044783
Eudicotyledons	Rubiaceae	*Hedyotis*	*Hedyotis shiuyingiae*	JN044784
Eudicotyledons	Fabaceae	*Dalbergia*	*Dalbergia assamica*	FR854143
Eudicotyledons	Fabaceae	*Dalbergia*	*Dalbergia assamica*	FR854144
Eudicotyledons	Fabaceae	*Dalbergia*	*Dalbergia assamica*	FR854145
Eudicotyledons	Fabaceae	*Dalbergia*	*Dalbergia assamica*	FR854146
Eudicotyledons	Fabaceae	*Dalbergia*	*Dalbergia assamica*	FR854147
Eudicotyledons	Phyllanthaceae	*Phyllanthus*	*Phyllanthus indofischeri*	GU598558
Eudicotyledons	Phyllanthaceae	*Phyllanthus*	*Phyllanthus indofischeri*	GU598559
Eudicotyledons	Phyllanthaceae	*Phyllanthus*	*Phyllanthus indofischeri*	GU598560
Eudicotyledons	Primulaceae	*Lysimachia*	*Lysimachia kalalauensis*	GQ257972
Eudicotyledons	Primulaceae	*Lysimachia*	*Lysimachia kalalauensis*	GQ257973
Eudicotyledons	Primulaceae	*Lysimachia*	*Lysimachia scopulensis*	GQ257983
Eudicotyledons	Primulaceae	*Lysimachia*	*Lysimachia scopulensis*	GQ257984
Eudicotyledons	Phyllanthaceae	*Phyllanthus*	*Phyllanthus kozhikodianus*	GU598570
Eudicotyledons	Phyllanthaceae	*Phyllanthus*	*Phyllanthus kozhikodianus*	GU598571
Eudicotyledons	Phyllanthaceae	*Phyllanthus*	*Phyllanthus kozhikodianus*	GU598572
Eudicotyledons	Phyllanthaceae	*Phyllanthus*	*Phyllanthus kozhikodianus*	GU598578
Eudicotyledons	Phyllanthaceae	*Phyllanthus*	*Phyllanthus kozhikodianus*	GQ409804
Eudicotyledons	Phyllanthaceae	*Phyllanthus*	*Phyllanthus kozhikodianus*	GQ409805
Eudicotyledons	Phyllanthaceae	*Phyllanthus*	*Phyllanthus kozhikodianus*	GQ409806
Eudicotyledons	Phyllanthaceae	*Phyllanthus*	*Phyllanthus lawii*	GU598556
Eudicotyledons	Phyllanthaceae	*Phyllanthus*	*Phyllanthus lawii*	GU598557
Eudicotyledons	Phyllanthaceae	*Phyllanthus*	*Phyllanthus missionis*	GU598553
Eudicotyledons	Phyllanthaceae	*Phyllanthus*	*Phyllanthus missionis*	GU598554
Eudicotyledons	Phyllanthaceae	*Phyllanthus*	*Phyllanthus missionis*	GU598555
Eudicotyledons	Phyllanthaceae	*Phyllanthus*	*Phyllanthus rotundifolius*	GU598548
Eudicotyledons	Phyllanthaceae	*Phyllanthus*	*Phyllanthus rotundifolius*	GU598549
Eudicotyledons	Phyllanthaceae	*Phyllanthus*	*Phyllanthus rotundifolius*	GU598550
Eudicotyledons	Phyllanthaceae	*Phyllanthus*	*Phyllanthus talbotii*	GU598551
Eudicotyledons	Phyllanthaceae	*Phyllanthus*	*Phyllanthus talbotii*	GU598552
Eudicotyledons	Polygonaceae	*Polygonum*	*Polygonum filicaule*	JN046423
Eudicotyledons	Polygonaceae	*Polygonum*	*Polygonum filicaule*	JN046424
Eudicotyledons	Gentianaceae	*Gentianopsis*	*Gentianopsis macrantha*	HM460859
Eudicotyledons	Gentianaceae	*Gentianopsis*	*Gentianopsis macrantha*	HM460860
Eudicotyledons	Gentianaceae	*Gentianopsis*	*Gentianopsis macrantha*	HM460861
Eudicotyledons	Gentianaceae	*Gentianopsis*	*Gentianopsis thermalis*	HM460862
Eudicotyledons	Gentianaceae	*Gentianopsis*	*Gentianopsis thermalis*	HM460863
Eudicotyledons	Gentianaceae	*Gentianopsis*	*Gentianopsis thermalis*	HM460864
Eudicotyledons	Hamamelidaceae	*Loropetalum*	*Loropetalum subcordatum*	JN542799
Eudicotyledons	Hamamelidaceae	*Loropetalum*	*Loropetalum subcordatum*	JN542800

Eudicotyledons	Hamamelidaceae	*Loropetalum*	*Loropetalum subcordatum*	HM369153
Eudicotyledons	Hamamelidaceae	*Loropetalum*	*Loropetalum subcordatum*	HM369154
Eudicotyledons	Araliaceae	*Dendropanax*	*Dendropanax dentiger*	HQ427094
Eudicotyledons	Araliaceae	*Dendropanax*	*Dendropanax dentiger*	GU054804
Eudicotyledons	Araliaceae	*Dendropanax*	*Dendropanax dentiger*	GU054808
Eudicotyledons	Araliaceae	*Dendropanax*	*Dendropanax dentiger*	GU054844
Eudicotyledons	Araliaceae	*Dendropanax*	*Dendropanax dentiger*	GU054884
Eudicotyledons	Araliaceae	*Dendropanax*	*Dendropanax caucanus*	GU054820
Eudicotyledons	Araliaceae	*Dendropanax*	*Dendropanax caucanus*	GU054821
Eudicotyledons	Araliaceae	*Dendropanax*	*Dendropanax gonatopodus*	GU054822
Eudicotyledons	Araliaceae	*Dendropanax*	*Dendropanax gonatopodus*	GU054871
Eudicotyledons	Araliaceae	*Dendropanax*	*Dendropanax gonatopodus*	GU054880
Eudicotyledons	Araliaceae	*Dendropanax*	*Dendropanax oliganthus*	GU054824
Eudicotyledons	Araliaceae	*Dendropanax*	*Dendropanax oliganthus*	GU054863
Eudicotyledons	Araliaceae	*Dendropanax*	*Dendropanax praestans*	GU054847
Eudicotyledons	Araliaceae	*Dendropanax*	*Dendropanax praestans*	GU054849
Eudicotyledons	Araliaceae	*Dendropanax*	*Dendropanax bolivianus*	GU054861
Eudicotyledons	Araliaceae	*Dendropanax*	*Dendropanax bolivianus*	GU054862
Eudicotyledons	Araliaceae	*Dendropanax*	*Dendropanax cuneatus*	GU054864
Eudicotyledons	Araliaceae	*Dendropanax*	*Dendropanax cuneatus*	GU054870
Eudicotyledons	Araliaceae	*Dendropanax*	*Dendropanax poilanei*	GU054866
Eudicotyledons	Araliaceae	*Dendropanax*	*Dendropanax poilanei*	GU054867
Eudicotyledons	Araliaceae	*Dendropanax*	*Dendropanax oligodontus*	GU054879
Eudicotyledons	Araliaceae	*Dendropanax*	*Dendropanax oligodontus*	GU054885
Eudicotyledons	Araliaceae	*Dendropanax*	*Dendropanax palustris*	GU054842
Eudicotyledons	Araliaceae	*Dendropanax*	*Dendropanax palustris*	GU054848
Eudicotyledons	Lamiaceae	*Thymus*	*Thymus sp. MIB zpl*	FR726151
Eudicotyledons	Lamiaceae	*Thymus*	*Thymus sp. MIB zpl*	FR726152
Eudicotyledons	Lamiaceae	*Thymus*	*Thymus sp. MIB zpl*	FR726153
Eudicotyledons	Asteraceae	*Soroseris*	*Soroseris gillii*	HQ436181
Eudicotyledons	Asteraceae	*Soroseris*	*Soroseris gillii*	JN047245
Eudicotyledons	Asteraceae	*Soroseris*	*Soroseris gillii*	JN047246
Eudicotyledons	Theaceae	*Stewartia*	*Stewartia cordifolia*	HM100516
Eudicotyledons	Theaceae	*Stewartia*	*Stewartia cordifolia*	HM100517
Eudicotyledons	Theaceae	*Stewartia*	*Stewartia laotica*	HM100519
Eudicotyledons	Theaceae	*Stewartia*	*Stewartia laotica*	HM100520
Eudicotyledons	Theaceae	*Stewartia*	*Stewartia micrantha*	HM100521
Eudicotyledons	Theaceae	*Stewartia*	*Stewartia micrantha*	HM100522
Eudicotyledons	Theaceae	*Stewartia*	*Stewartia obovata*	HM100524
Eudicotyledons	Theaceae	*Stewartia*	*Stewartia obovata*	HM100525
Eudicotyledons	Theaceae	*Stewartia*	*Stewartia sichuanensis*	HM100526
Eudicotyledons	Theaceae	*Stewartia*	*Stewartia sichuanensis*	HM100527
Eudicotyledons	Theaceae	*Stewartia*	*Stewartia sinii*	HM100530
Eudicotyledons	Theaceae	*Stewartia*	*Stewartia sinii*	HM100531
Eudicotyledons	Theaceae	*Stewartia*	*Stewartia tonkinensis*	HM100532
Eudicotyledons	Theaceae	*Stewartia*	*Stewartia tonkinensis*	HM100533
Eudicotyledons	Theaceae	*Stewartia*	*Stewartia rubiginosa*	HM100565
Eudicotyledons	Theaceae	*Stewartia*	*Stewartia rubiginosa*	HM100580
Eudicotyledons	Theaceae	*Stewartia*	*Stewartia rubiginosa*	HM100581
Eudicotyledons	Theaceae	*Stewartia*	*Stewartia rubiginosa*	HM100582
Eudicotyledons	Fabaceae	*Senna*	*Senna sulfurea*	JF838361
Eudicotyledons	Fabaceae	*Senna*	*Senna sulfurea*	HQ161758
Eudicotyledons	Fabaceae	*Senna*	*Senna sophera*	JF838363

Eudicotyledons	Fabaceae	*Senna*	*Senna sophera*	HQ161760
Eudicotyledons	Asteraceae	*Solidago*	*Solidago houghtonii*	HQ142563
Eudicotyledons	Asteraceae	*Solidago*	*Solidago houghtonii*	HQ142564
Eudicotyledons	Asteraceae	*Solidago*	*Solidago houghtonii*	HQ142565
Eudicotyledons	Asteraceae	*Solidago*	*Solidago houghtonii*	HQ142566
Eudicotyledons	Asteraceae	*Soroseris*	*Soroseris erysimoides*	HQ436179
Eudicotyledons	Asteraceae	*Soroseris*	*Soroseris erysimoides*	HQ436180
Eudicotyledons	Asteraceae	*Soroseris*	*Soroseris erysimoides*	JN047243
Eudicotyledons	Asteraceae	*Soroseris*	*Soroseris erysimoides*	JN047244
Eudicotyledons	Asteraceae	*Soroseris*	*Soroseris hirsuta*	HQ436185
Eudicotyledons	Asteraceae	*Soroseris*	*Soroseris hirsuta*	HQ436186
Eudicotyledons	Asteraceae	*Soroseris*	*Soroseris hirsuta*	JN047249
Eudicotyledons	Asteraceae	*Soroseris*	*Soroseris hirsuta*	JN047250
Eudicotyledons	Fabaceae	*Dalbergia*	*Dalbergia oliveri*	FR854153
Eudicotyledons	Fabaceae	*Dalbergia*	*Dalbergia oliveri*	FR854154
Eudicotyledons	Fabaceae	*Dalbergia*	*Dalbergia oliveri*	FR854155
Eudicotyledons	Fabaceae	*Dalbergia*	*Dalbergia oliveri*	FR854156
Eudicotyledons	Fabaceae	*Dalbergia*	*Dalbergia oliveri*	FR854157
Eudicotyledons	Rubiaceae	*Hedyotis*	*Hedyotis pterita*	JF699797
Eudicotyledons	Rubiaceae	*Hedyotis*	*Hedyotis pterita*	JF699798
Eudicotyledons	Rubiaceae	*Hedyotis*	*Hedyotis pterita*	JF699799
Eudicotyledons	Rubiaceae	*Hedyotis*	*Hedyotis pterita*	JF699800
Eudicotyledons	Hypericaceae	*Hypericum*	*Hypericum przewalskii*	JN044930
Eudicotyledons	Hypericaceae	*Hypericum*	*Hypericum przewalskii*	JN044931
Eudicotyledons	Hypericaceae	*Hypericum*	*Hypericum przewalskii*	JN044932
Eudicotyledons	Rubiaceae	*Leptodermis*	*Leptodermis buxifolia*	JN045149
Eudicotyledons	Rubiaceae	*Leptodermis*	*Leptodermis buxifolia*	JN045150
Eudicotyledons	Rubiaceae	*Leptodermis*	*Leptodermis hirsutiflora*	JN045153
Eudicotyledons	Rubiaceae	*Leptodermis*	*Leptodermis hirsutiflora*	JN045154
Eudicotyledons	Rubiaceae	*Leptodermis*	*Leptodermis ovata*	JN045155
Eudicotyledons	Rubiaceae	*Leptodermis*	*Leptodermis ovata*	JN045156
Eudicotyledons	Rubiaceae	*Leptodermis*	*Leptodermis pilosa*	JN045157
Eudicotyledons	Rubiaceae	*Leptodermis*	*Leptodermis pilosa*	JN045158
Eudicotyledons	Rubiaceae	*Leptodermis*	*Leptodermis pilosa*	JN045159
Eudicotyledons	Rubiaceae	*Leptodermis*	*Leptodermis scabrida*	JN045170
Eudicotyledons	Rubiaceae	*Leptodermis*	*Leptodermis scabrida*	JN045171
Eudicotyledons	Rubiaceae	*Leptodermis*	*Leptodermis scabrida*	JN045172
Eudicotyledons	Rubiaceae	*Leptodermis*	*Leptodermis scabrida*	JN045173
Eudicotyledons	Rubiaceae	*Leptodermis*	*Leptodermis ludlowii*	JN045174
Eudicotyledons	Rubiaceae	*Leptodermis*	*Leptodermis ludlowii*	JN045175
Eudicotyledons	Rubiaceae	*Leptodermis*	*Leptodermis vestita*	JN045178
Eudicotyledons	Rubiaceae	*Leptodermis*	*Leptodermis vestita*	JN045179
Eudicotyledons	Caprifoliaceae	*Lonicera*	*Lonicera webbiana*	JN045264
Eudicotyledons	Caprifoliaceae	*Lonicera*	*Lonicera webbiana*	JN045265
Eudicotyledons	Caprifoliaceae	*Lonicera*	*Lonicera webbiana*	JN045266
Eudicotyledons	Primulaceae	*Lysimachia*	*Lysimachia chapaensis*	JN045297
Eudicotyledons	Primulaceae	*Lysimachia*	*Lysimachia chapaensis*	JN045298
Eudicotyledons	Primulaceae	*Lysimachia*	*Lysimachia chekiangensis*	JN045299
Eudicotyledons	Primulaceae	*Lysimachia*	*Lysimachia chekiangensis*	JN045300
Eudicotyledons	Primulaceae	*Lysimachia*	*Lysimachia dextrorsiflora*	JN045323
Eudicotyledons	Primulaceae	*Lysimachia*	*Lysimachia dextrorsiflora*	JN045324
Eudicotyledons	Primulaceae	*Lysimachia*	*Lysimachia erosipetala*	JN045325
Eudicotyledons	Primulaceae	*Lysimachia*	*Lysimachia erosipetala*	JN045326

Eudicotyledons	Primulaceae	*Lysimachia*	*Lysimachia gesnerioides*	JN045338
Eudicotyledons	Primulaceae	*Lysimachia*	*Lysimachia gesnerioides*	JN045339
Eudicotyledons	Primulaceae	*Lysimachia*	*Lysimachia hemsleyana*	JN045342
Eudicotyledons	Primulaceae	*Lysimachia*	*Lysimachia hemsleyana*	JN045343
Eudicotyledons	Primulaceae	*Lysimachia*	*Lysimachia hemsleyana*	JN045344
Eudicotyledons	Primulaceae	*Lysimachia*	*Lysimachia hemsleyana*	JN045345
Eudicotyledons	Primulaceae	*Lysimachia*	*Lysimachia hemsleyana*	JN045346
Eudicotyledons	Primulaceae	*Lysimachia*	*Lysimachia hemsleyi*	JN045347
Eudicotyledons	Primulaceae	*Lysimachia*	*Lysimachia hemsleyi*	JN045348
Eudicotyledons	Primulaceae	*Lysimachia*	*Lysimachia hemsleyi*	JN045349
Eudicotyledons	Primulaceae	*Lysimachia*	*Lysimachia hemsleyi*	JN045350
Eudicotyledons	Primulaceae	*Lysimachia*	*Lysimachia heterobotrys*	JN045351
Eudicotyledons	Primulaceae	*Lysimachia*	*Lysimachia heterobotrys*	JN045352
Eudicotyledons	Primulaceae	*Lysimachia*	*Lysimachia klattiana*	JN045362
Eudicotyledons	Primulaceae	*Lysimachia*	*Lysimachia klattiana*	JN045363
Eudicotyledons	Primulaceae	*Lysimachia*	*Lysimachia patungensis*	JN045380
Eudicotyledons	Primulaceae	*Lysimachia*	*Lysimachia patungensis*	JN045381
Eudicotyledons	Primulaceae	*Lysimachia*	*Lysimachia patungensis*	JN045382
Eudicotyledons	Primulaceae	*Lysimachia*	*Lysimachia patungensis*	JN045383
Eudicotyledons	Primulaceae	*Lysimachia*	*Lysimachia pentapetala*	JN045384
Eudicotyledons	Primulaceae	*Lysimachia*	*Lysimachia pentapetala*	JN045385
Eudicotyledons	Apiaceae	*Peucedanum*	*Peucedanum ampliatum*	JN046213
Eudicotyledons	Apiaceae	*Peucedanum*	*Peucedanum ampliatum*	JN046214
Eudicotyledons	Apiaceae	*Peucedanum*	*Peucedanum ampliatum*	JN046215
Eudicotyledons	Apiaceae	*Peucedanum*	*Peucedanum caespitosum*	JN046216
Eudicotyledons	Apiaceae	*Peucedanum*	*Peucedanum caespitosum*	JN046217
Eudicotyledons	Apiaceae	*Peucedanum*	*Peucedanum caespitosum*	JN046218
Eudicotyledons	Apiaceae	*Peucedanum*	*Peucedanum medicum*	JN046225
Eudicotyledons	Apiaceae	*Peucedanum*	*Peucedanum medicum*	JN046226
Eudicotyledons	Apiaceae	*Peucedanum*	*Peucedanum medicum*	JN046227
Eudicotyledons	Apiaceae	*Peucedanum*	*Peucedanum medicum*	JN046228
Eudicotyledons	Apiaceae	*Peucedanum*	*Peucedanum wawrae*	JN046236
Eudicotyledons	Apiaceae	*Peucedanum*	*Peucedanum wawrae*	JN046237
Eudicotyledons	Apiaceae	*Peucedanum*	*Peucedanum wawrae*	JN046238
Eudicotyledons	Apiaceae	*Pleurospermum*	*Pleurospermum cristatum*	JN046385
Eudicotyledons	Apiaceae	*Pleurospermum*	*Pleurospermum cristatum*	JN046386
Eudicotyledons	Apiaceae	*Pleurospermum*	*Pleurospermum giraldii*	JN046389
Eudicotyledons	Apiaceae	*Pleurospermum*	*Pleurospermum giraldii*	JN046390
Eudicotyledons	Apiaceae	*Pleurospermum*	*Pleurospermum giraldii*	JN046391
Eudicotyledons	Apiaceae	*Pleurospermum*	*Pleurospermum giraldii*	JN046392
Eudicotyledons	Primulaceae	*Primula*	*Primula agleniana*	JN046458
Eudicotyledons	Primulaceae	*Primula*	*Primula agleniana*	JN046459
Eudicotyledons	Primulaceae	*Primula*	*Primula agleniana*	JN046460
Eudicotyledons	Primulaceae	*Primula*	*Primula diantha*	JN046508
Eudicotyledons	Primulaceae	*Primula*	*Primula diantha*	JN046509
Eudicotyledons	Primulaceae	*Primula*	*Primula diantha*	JN046510
Eudicotyledons	Primulaceae	*Primula*	*Primula diantha*	JN046511
Eudicotyledons	Primulaceae	*Primula*	*Primula duclouxii*	JN046512
Eudicotyledons	Primulaceae	*Primula*	*Primula duclouxii*	JN046513
Eudicotyledons	Primulaceae	*Primula*	*Primula epilosa*	JN046514
Eudicotyledons	Primulaceae	*Primula*	*Primula epilosa*	JN046515
Eudicotyledons	Primulaceae	*Primula*	*Primula epilosa*	JN046516
Eudicotyledons	Primulaceae	*Primula*	*Primula kialensis*	JN046528

Eudicotyledons	Primulaceae	*Primula*	*Primula kialensis*	JN046529
Eudicotyledons	Primulaceae	*Primula*	*Primula melanops*	JN046534
Eudicotyledons	Primulaceae	*Primula*	*Primula melanops*	JN046535
Eudicotyledons	Primulaceae	*Primula*	*Primula munroi*	JN046543
Eudicotyledons	Primulaceae	*Primula*	*Primula munroi*	JN046544
Eudicotyledons	Primulaceae	*Primula*	*Primula munroi*	JN046545
Eudicotyledons	Primulaceae	*Primula*	*Primula munroi*	JN046546
Eudicotyledons	Primulaceae	*Primula*	*Primula oreodoxa*	JN046550
Eudicotyledons	Primulaceae	*Primula*	*Primula oreodoxa*	JN046551
Eudicotyledons	Primulaceae	*Primula*	*Primula prattii*	JN046562
Eudicotyledons	Primulaceae	*Primula*	*Primula prattii*	JN046563
Eudicotyledons	Primulaceae	*Primula*	*Primula spicata*	JN046590
Eudicotyledons	Primulaceae	*Primula*	*Primula spicata*	JN046591
Eudicotyledons	Primulaceae	*Primula*	*Primula szechuanica*	JN046592
Eudicotyledons	Primulaceae	*Primula*	*Primula szechuanica*	JN046593
Eudicotyledons	Primulaceae	*Primula*	*Primula tardiflora*	JN046594
Eudicotyledons	Primulaceae	*Primula*	*Primula tardiflora*	JN046595
Eudicotyledons	Primulaceae	*Primula*	*Primula virginis*	JN046596
Eudicotyledons	Primulaceae	*Primula*	*Primula virginis*	JN046597
Eudicotyledons	Ranunculaceae	*Aconitum*	*Aconitum angustius*	JN043736
Eudicotyledons	Ranunculaceae	*Aconitum*	*Aconitum angustius*	JN043737
Eudicotyledons	Ranunculaceae	*Aconitum*	*Aconitum angustius*	JN043738
Eudicotyledons	Ranunculaceae	*Aconitum*	*Aconitum angustius*	JN043739
Eudicotyledons	Ranunculaceae	*Aconitum*	*Aconitum angustius*	JN043740
Eudicotyledons	Ranunculaceae	*Aconitum*	*Aconitum angustius*	JN043741
Eudicotyledons	Ranunculaceae	*Aconitum*	*Aconitum angustius*	JN043742
Eudicotyledons	Ranunculaceae	*Aconitum*	*Aconitum angustius*	JN043743
Eudicotyledons	Ranunculaceae	*Aconitum*	*Aconitum angustius*	JN043744
Eudicotyledons	Ranunculaceae	*Aconitum*	*Aconitum angustius*	JN043745
Eudicotyledons	Ranunculaceae	*Aconitum*	*Aconitum angustius*	JN043746
Eudicotyledons	Ranunculaceae	*Aconitum*	*Aconitum barbatum*	JN043747
Eudicotyledons	Ranunculaceae	*Aconitum*	*Aconitum barbatum*	JN043748
Eudicotyledons	Ranunculaceae	*Aconitum*	*Aconitum barbatum*	JN043749
Eudicotyledons	Ranunculaceae	*Aconitum*	*Aconitum barbatum*	JN043750
Eudicotyledons	Ranunculaceae	*Aconitum*	*Aconitum barbatum*	JN043751
Eudicotyledons	Ranunculaceae	*Aconitum*	*Aconitum barbatum*	JN043752
Eudicotyledons	Ranunculaceae	*Aconitum*	*Aconitum longecassidatum*	JN043756
Eudicotyledons	Ranunculaceae	*Aconitum*	*Aconitum longecassidatum*	JN043757
Eudicotyledons	Ranunculaceae	*Aconitum*	*Aconitum longecassidatum*	JN043758
Eudicotyledons	Ranunculaceae	*Aconitum*	*Aconitum longecassidatum*	JN043759
Eudicotyledons	Ranunculaceae	*Aconitum*	*Aconitum longecassidatum*	JN043760
Eudicotyledons	Ranunculaceae	*Aconitum*	*Aconitum longecassidatum*	JN043761
Eudicotyledons	Ranunculaceae	*Aconitum*	*Aconitum longecassidatum*	JN043762
Eudicotyledons	Ranunculaceae	*Aconitum*	*Aconitum monticola*	JN043763
Eudicotyledons	Ranunculaceae	*Aconitum*	*Aconitum monticola*	JN043764
Eudicotyledons	Ranunculaceae	*Aconitum*	*Aconitum monticola*	JN043765
Eudicotyledons	Ranunculaceae	*Delphinium*	*Delphinium batangense*	JN044396
Eudicotyledons	Ranunculaceae	*Delphinium*	*Delphinium batangense*	JN044397
Eudicotyledons	Ranunculaceae	*Delphinium*	*Delphinium beesianum*	JN044398
Eudicotyledons	Ranunculaceae	*Delphinium*	*Delphinium beesianum*	JN044399
Eudicotyledons	Ranunculaceae	*Delphinium*	*Delphinium ceratophorum*	JN044400
Eudicotyledons	Ranunculaceae	*Delphinium*	*Delphinium ceratophorum*	JN044401
Eudicotyledons	Ranunculaceae	*Delphinium*	*Delphinium dolichocentroides*	JN044404

Eudicotyledons	Ranunculaceae	*Delphinium*	*Delphinium dolichocentroides*	JN044405
Eudicotyledons	Ranunculaceae	*Delphinium*	*Delphinium kamaonense*	JN044409
Eudicotyledons	Ranunculaceae	*Delphinium*	*Delphinium kamaonense*	JN044410
Eudicotyledons	Ranunculaceae	*Delphinium*	*Delphinium muliense*	JN044411
Eudicotyledons	Ranunculaceae	*Delphinium*	*Delphinium muliense*	JN044412
Eudicotyledons	Ranunculaceae	*Delphinium*	*Delphinium omeiense*	JN044413
Eudicotyledons	Ranunculaceae	*Delphinium*	*Delphinium omeiense*	JN044414
Eudicotyledons	Ranunculaceae	*Delphinium*	*Delphinium omeiense*	JN044415
Eudicotyledons	Ranunculaceae	*Delphinium*	*Delphinium omeiense*	JN044416
Eudicotyledons	Ranunculaceae	*Delphinium*	*Delphinium omeiense*	JN044417
Eudicotyledons	Ranunculaceae	*Delphinium*	*Delphinium pseudohamatum*	JN044420
Eudicotyledons	Ranunculaceae	*Delphinium*	*Delphinium pseudohamatum*	JN044421
Eudicotyledons	Ranunculaceae	*Delphinium*	*Delphinium pycnocentrum*	JN044422
Eudicotyledons	Ranunculaceae	*Delphinium*	*Delphinium pycnocentrum*	JN044423
Eudicotyledons	Ranunculaceae	*Delphinium*	*Delphinium smithianum*	JN044424
Eudicotyledons	Ranunculaceae	*Delphinium*	*Delphinium smithianum*	JN044425
Eudicotyledons	Ranunculaceae	*Delphinium*	*Delphinium spirocentrum*	JN044426
Eudicotyledons	Ranunculaceae	*Delphinium*	*Delphinium spirocentrum*	JN044427
Eudicotyledons	Ranunculaceae	*Delphinium*	*Delphinium taliense*	JN044428
Eudicotyledons	Ranunculaceae	*Delphinium*	*Delphinium taliense*	JN044429
Eudicotyledons	Ranunculaceae	*Delphinium*	*Delphinium taliense*	JN044430
Eudicotyledons	Ranunculaceae	*Delphinium*	*Delphinium taliense*	JN044431
Eudicotyledons	Ranunculaceae	*Delphinium*	*Delphinium tatsienense*	JN044432
Eudicotyledons	Ranunculaceae	*Delphinium*	*Delphinium tatsienense*	JN044433
Eudicotyledons	Ranunculaceae	*Delphinium*	*Delphinium tatsienense*	JN044434
Eudicotyledons	Ranunculaceae	*Delphinium*	*Delphinium tatsienense*	JN044435
Eudicotyledons	Ranunculaceae	*Delphinium*	*Delphinium tatsienense*	JN044436
Eudicotyledons	Ranunculaceae	*Delphinium*	*Delphinium tatsienense*	JN044437
Eudicotyledons	Ranunculaceae	*Delphinium*	*Delphinium thibeticum*	JN044438
Eudicotyledons	Ranunculaceae	*Delphinium*	*Delphinium thibeticum*	JN044439
Eudicotyledons	Ranunculaceae	*Delphinium*	*Delphinium thibeticum*	JN044440
Eudicotyledons	Ranunculaceae	*Delphinium*	*Delphinium tongolense*	JN044441
Eudicotyledons	Ranunculaceae	*Delphinium*	*Delphinium tongolense*	JN044442
Eudicotyledons	Ranunculaceae	*Delphinium*	*Delphinium trichophorum*	JN044443
Eudicotyledons	Ranunculaceae	*Delphinium*	*Delphinium trichophorum*	JN044444
Eudicotyledons	Rubiaceae	*Hedyotis*	*Hedyotis cantoniensis*	JF699773
Eudicotyledons	Rubiaceae	*Hedyotis*	*Hedyotis cantoniensis*	JF699774
Eudicotyledons	Rubiaceae	*Hedyotis*	*Hedyotis cantoniensis*	JF699775
Eudicotyledons	Rubiaceae	*Hedyotis*	*Hedyotis cantoniensis*	JN044763
Eudicotyledons	Rubiaceae	*Hedyotis*	*Hedyotis cantoniensis*	JN044764
Eudicotyledons	Rubiaceae	*Hedyotis*	*Hedyotis caudatifolia*	JF699776
Eudicotyledons	Rubiaceae	*Hedyotis*	*Hedyotis caudatifolia*	JF699777
Eudicotyledons	Rubiaceae	*Hedyotis*	*Hedyotis caudatifolia*	JN044765
Eudicotyledons	Rubiaceae	*Hedyotis*	*Hedyotis caudatifolia*	JN044766
Eudicotyledons	Rubiaceae	*Hedyotis*	*Hedyotis pulcherrima*	JF699801
Eudicotyledons	Rubiaceae	*Hedyotis*	*Hedyotis pulcherrima*	JF699802
Eudicotyledons	Rubiaceae	*Hedyotis*	*Hedyotis pulcherrima*	JF699803
Eudicotyledons	Rubiaceae	*Hedyotis*	*Hedyotis pulcherrima*	JN044779
Eudicotyledons	Rubiaceae	*Hedyotis*	*Hedyotis pulcherrima*	JN044780
Eudicotyledons	Rubiaceae	*Hedyotis*	*Hedyotis shenzhenensis*	JF699805
Eudicotyledons	Rubiaceae	*Hedyotis*	*Hedyotis shenzhenensis*	JF699806
Eudicotyledons	Rubiaceae	*Hedyotis*	*Hedyotis shenzhenensis*	JF699807
Eudicotyledons	Rubiaceae	*Hedyotis*	*Hedyotis shenzhenensis*	JN044781

Eudicotyledons	Rubiaceae	*Hedyotis*	*Hedyotis shenzhenensis*	JN044782
Eudicotyledons	Rubiaceae	*Hedyotis*	*Hedyotis uncinella*	JF699814
Eudicotyledons	Rubiaceae	*Hedyotis*	*Hedyotis uncinella*	JF699815
Eudicotyledons	Rubiaceae	*Hedyotis*	*Hedyotis uncinella*	JN044789
Eudicotyledons	Rubiaceae	*Hedyotis*	*Hedyotis uncinella*	JN044790
Eudicotyledons	Lamiaceae	*Lamium*	*Lamium barbatum*	JF780113
Eudicotyledons	Lamiaceae	*Lamium*	*Lamium barbatum*	JF780114
Eudicotyledons	Lamiaceae	*Lamium*	*Lamium barbatum*	JF780115
Eudicotyledons	Lamiaceae	*Lamium*	*Lamium barbatum*	HM590111
Eudicotyledons	Ranunculaceae	*Aconitum*	*Aconitum shennongjiaense*	JN043770
Eudicotyledons	Ranunculaceae	*Aconitum*	*Aconitum shennongjiaense*	JN043771
Eudicotyledons	Rubiaceae	*Leptodermis*	*Leptodermis forrestii*	JN045151
Eudicotyledons	Rubiaceae	*Leptodermis*	*Leptodermis forrestii*	JN045152
Eudicotyledons	Lamiaceae	*Lamium*	*Lamium x holsaticum*	JF780157
Eudicotyledons	Lamiaceae	*Lamium*	*Lamium x holsaticum*	JF780158
Eudicotyledons	Lamiaceae	*Lamium*	*Lamium bifidum*	JF780128
Eudicotyledons	Lamiaceae	*Lamium*	*Lamium bifidum*	JF780129
Eudicotyledons	Lamiaceae	*Lamium*	*Lamium bifidum*	JF780130
Eudicotyledons	Lamiaceae	*Lamium*	*Lamium confertum*	JF780131
Eudicotyledons	Lamiaceae	*Lamium*	*Lamium confertum*	JF780132
Eudicotyledons	Lamiaceae	*Lamium*	*Lamium confertum*	JF780133
Eudicotyledons	Lamiaceae	*Lamium*	*Lamium eriocephalum*	JF780135
Eudicotyledons	Lamiaceae	*Lamium*	*Lamium eriocephalum*	JF780136
Eudicotyledons	Lamiaceae	*Lamium*	*Lamium flexuosum*	JF780137
Eudicotyledons	Lamiaceae	*Lamium*	*Lamium flexuosum*	JF780138
Eudicotyledons	Lamiaceae	*Lamium*	*Lamium flexuosum*	JF780139
Eudicotyledons	Lamiaceae	*Lamium*	*Lamium galactophyllum*	JF780140
Eudicotyledons	Lamiaceae	*Lamium*	*Lamium galactophyllum*	JF780141
Eudicotyledons	Lamiaceae	*Lamium*	*Lamium macrodon*	JF780164
Eudicotyledons	Lamiaceae	*Lamium*	*Lamium macrodon*	JF780165
Eudicotyledons	Lamiaceae	*Lamium*	*Lamium macrodon*	JF780166
Eudicotyledons	Lamiaceae	*Lamium*	*Lamium moschatum*	JF780169
Eudicotyledons	Lamiaceae	*Lamium*	*Lamium moschatum*	JF780171
Eudicotyledons	Lamiaceae	*Lamium*	*Lamium moschatum*	HQ902821
Eudicotyledons	Lamiaceae	*Lamium*	*Lamium multifidum*	JF780172
Eudicotyledons	Lamiaceae	*Lamium*	*Lamium multifidum*	JF780173
Eudicotyledons	Lamiaceae	*Lamium*	*Lamium orientale*	JF780174
Eudicotyledons	Lamiaceae	*Lamium*	*Lamium orientale*	JF780175
Eudicotyledons	Lamiaceae	*Lamium*	*Lamium orvala*	JF780176
Eudicotyledons	Lamiaceae	*Lamium*	*Lamium orvala*	JF780177
Eudicotyledons	Lamiaceae	*Lamium*	*Lamium orvala*	JF780178
Eudicotyledons	Lamiaceae	*Lamium*	*Lamium orvala*	JF780179
Eudicotyledons	Lamiaceae	*Thymus*	*Thymus sipyleus*	HQ902849
Eudicotyledons	Lamiaceae	*Thymus*	*Thymus sipyleus*	HQ902878
Eudicotyledons	Asteraceae	*Symphyotrichum*	*Symphyotrichum cordifolium*	HQ596857
Eudicotyledons	Asteraceae	*Symphyotrichum*	*Symphyotrichum cordifolium*	DQ006144
Eudicotyledons	Asteraceae	*Nabalus*	*Nabalus altissimus*	HQ596801
Eudicotyledons	Asteraceae	*Nabalus*	*Nabalus altissimus*	HQ162010
Eudicotyledons	Asteraceae	*Symphyotrichum*	*Symphyotrichum novae-angliae*	GU818335
Eudicotyledons	Asteraceae	*Symphyotrichum*	*Symphyotrichum novae-angliae*	EU750589
Eudicotyledons	Asteraceae	*Symphyotrichum*	*Symphyotrichum novae-angliae*	EU750590
Eudicotyledons	Asteraceae	*Symphyotrichum*	*Symphyotrichum novae-angliae*	EU750591
Eudicotyledons	Asteraceae	*Symphyotrichum*	*Symphyotrichum novae-angliae*	EU750592

Eudicotyledons	Malvaceae	*Alcea*	*Alcea rosea*	EF679744
Eudicotyledons	Malvaceae	*Alcea*	*Alcea rosea*	EF419662
Eudicotyledons	Zygophyllaceae	*Larrea*	*Larrea tridentata*	JF266708
Eudicotyledons	Zygophyllaceae	*Larrea*	*Larrea tridentata*	JF266709
Eudicotyledons	Zygophyllaceae	*Larrea*	*Larrea tridentata*	JF266710
Eudicotyledons	Zygophyllaceae	*Larrea*	*Larrea tridentata*	JF266711
Eudicotyledons	Zygophyllaceae	*Larrea*	*Larrea tridentata*	JF266712
Eudicotyledons	Zygophyllaceae	*Larrea*	*Larrea tridentata*	JF266713
Eudicotyledons	Zygophyllaceae	*Larrea*	*Larrea tridentata*	JF266714
Eudicotyledons	Zygophyllaceae	*Larrea*	*Larrea tridentata*	JF266715
Eudicotyledons	Zygophyllaceae	*Larrea*	*Larrea tridentata*	JF266716
Eudicotyledons	Zygophyllaceae	*Larrea*	*Larrea tridentata*	JF266717
Eudicotyledons	Zygophyllaceae	*Larrea*	*Larrea tridentata*	JF266718
Eudicotyledons	Zygophyllaceae	*Larrea*	*Larrea tridentata*	JF266719
Eudicotyledons	Zygophyllaceae	*Larrea*	*Larrea tridentata*	JF266720
Eudicotyledons	Zygophyllaceae	*Larrea*	*Larrea tridentata*	JF266721
Eudicotyledons	Zygophyllaceae	*Larrea*	*Larrea tridentata*	JF266722
Eudicotyledons	Zygophyllaceae	*Larrea*	*Larrea tridentata*	JF266723
Eudicotyledons	Zygophyllaceae	*Larrea*	*Larrea tridentata*	JF266724
Eudicotyledons	Zygophyllaceae	*Larrea*	*Larrea tridentata*	JF266725
Eudicotyledons	Zygophyllaceae	*Larrea*	*Larrea tridentata*	JF266726
Eudicotyledons	Zygophyllaceae	*Larrea*	*Larrea tridentata*	JF266727
Eudicotyledons	Zygophyllaceae	*Larrea*	*Larrea tridentata*	JF266728
Eudicotyledons	Zygophyllaceae	*Larrea*	*Larrea tridentata*	JF266729
Eudicotyledons	Zygophyllaceae	*Larrea*	*Larrea tridentata*	JF266730
Eudicotyledons	Zygophyllaceae	*Larrea*	*Larrea tridentata*	JF266731
Eudicotyledons	Zygophyllaceae	*Larrea*	*Larrea tridentata*	JF266732
Eudicotyledons	Zygophyllaceae	*Larrea*	*Larrea tridentata*	JF266733
Eudicotyledons	Zygophyllaceae	*Larrea*	*Larrea tridentata*	JF266734
Eudicotyledons	Zygophyllaceae	*Larrea*	*Larrea tridentata*	JF266735
Eudicotyledons	Zygophyllaceae	*Larrea*	*Larrea tridentata*	JF266736
Eudicotyledons	Zygophyllaceae	*Larrea*	*Larrea tridentata*	JF266737
Eudicotyledons	Zygophyllaceae	*Larrea*	*Larrea tridentata*	JF266738
Eudicotyledons	Zygophyllaceae	*Larrea*	*Larrea tridentata*	JF266739
Eudicotyledons	Zygophyllaceae	*Larrea*	*Larrea tridentata*	JF266740
Eudicotyledons	Zygophyllaceae	*Larrea*	*Larrea tridentata*	JF266741
Eudicotyledons	Zygophyllaceae	*Larrea*	*Larrea tridentata*	JF266742
Eudicotyledons	Zygophyllaceae	*Larrea*	*Larrea tridentata*	JF266743
Eudicotyledons	Zygophyllaceae	*Larrea*	*Larrea tridentata*	JF266744
Eudicotyledons	Zygophyllaceae	*Larrea*	*Larrea tridentata*	JF266745
Eudicotyledons	Zygophyllaceae	*Larrea*	*Larrea tridentata*	JF266746
Eudicotyledons	Zygophyllaceae	*Larrea*	*Larrea tridentata*	JF266747
Eudicotyledons	Zygophyllaceae	*Larrea*	*Larrea tridentata*	JF266748
Eudicotyledons	Zygophyllaceae	*Larrea*	*Larrea tridentata*	JF266749
Eudicotyledons	Zygophyllaceae	*Larrea*	*Larrea tridentata*	JF266750
Eudicotyledons	Zygophyllaceae	*Larrea*	*Larrea tridentata*	JF266751
Eudicotyledons	Zygophyllaceae	*Larrea*	*Larrea tridentata*	JF266752
Eudicotyledons	Zygophyllaceae	*Larrea*	*Larrea tridentata*	JF266753
Eudicotyledons	Zygophyllaceae	*Larrea*	*Larrea tridentata*	JF266754
Eudicotyledons	Zygophyllaceae	*Larrea*	*Larrea tridentata*	JF266755
Eudicotyledons	Zygophyllaceae	*Larrea*	*Larrea tridentata*	JF266756
Eudicotyledons	Zygophyllaceae	*Larrea*	*Larrea tridentata*	JF266757
Eudicotyledons	Zygophyllaceae	*Larrea*	*Larrea tridentata*	JF266758

Eudicotyledons	Zygophyllaceae	*Larrea*	*Larrea tridentata*	JF266759
Eudicotyledons	Zygophyllaceae	*Larrea*	*Larrea tridentata*	JF266760
Eudicotyledons	Zygophyllaceae	*Larrea*	*Larrea tridentata*	JF266763
Eudicotyledons	Zygophyllaceae	*Larrea*	*Larrea tridentata*	JF266764
Eudicotyledons	Zygophyllaceae	*Larrea*	*Larrea tridentata*	JF266765
Eudicotyledons	Zygophyllaceae	*Larrea*	*Larrea tridentata*	JF266766
Eudicotyledons	Zygophyllaceae	*Larrea*	*Larrea tridentata*	JF266767
Eudicotyledons	Zygophyllaceae	*Larrea*	*Larrea tridentata*	JF266768
Eudicotyledons	Zygophyllaceae	*Larrea*	*Larrea tridentata*	JF266769
Eudicotyledons	Zygophyllaceae	*Larrea*	*Larrea tridentata*	JF266770
Eudicotyledons	Zygophyllaceae	*Larrea*	*Larrea tridentata*	JF266771
Eudicotyledons	Zygophyllaceae	*Larrea*	*Larrea tridentata*	JF266772
Eudicotyledons	Zygophyllaceae	*Larrea*	*Larrea tridentata*	JF266773
Eudicotyledons	Zygophyllaceae	*Larrea*	*Larrea tridentata*	JF266774
Eudicotyledons	Zygophyllaceae	*Larrea*	*Larrea tridentata*	JF266775
Eudicotyledons	Zygophyllaceae	*Larrea*	*Larrea tridentata*	JF266779
Eudicotyledons	Zygophyllaceae	*Larrea*	*Larrea tridentata*	JF266780
Eudicotyledons	Zygophyllaceae	*Larrea*	*Larrea tridentata*	JF266781
Eudicotyledons	Zygophyllaceae	*Larrea*	*Larrea tridentata*	JF266782
Eudicotyledons	Zygophyllaceae	*Larrea*	*Larrea tridentata*	JF266783
Eudicotyledons	Zygophyllaceae	*Larrea*	*Larrea tridentata*	JF266784
Eudicotyledons	Zygophyllaceae	*Larrea*	*Larrea tridentata*	JF266785
Eudicotyledons	Zygophyllaceae	*Larrea*	*Larrea tridentata*	JF266786
Eudicotyledons	Zygophyllaceae	*Larrea*	*Larrea tridentata*	JF266787
Eudicotyledons	Zygophyllaceae	*Larrea*	*Larrea tridentata*	JF266788
Eudicotyledons	Zygophyllaceae	*Larrea*	*Larrea tridentata*	JF266789
Eudicotyledons	Zygophyllaceae	*Larrea*	*Larrea tridentata*	JF266790
Eudicotyledons	Zygophyllaceae	*Larrea*	*Larrea tridentata*	JF266791
Eudicotyledons	Zygophyllaceae	*Larrea*	*Larrea tridentata*	JF266792
Eudicotyledons	Zygophyllaceae	*Larrea*	*Larrea tridentata*	JF266793
Eudicotyledons	Zygophyllaceae	*Larrea*	*Larrea tridentata*	JF266794
Eudicotyledons	Zygophyllaceae	*Larrea*	*Larrea tridentata*	JF266795
Eudicotyledons	Zygophyllaceae	*Larrea*	*Larrea tridentata*	JF266796
Eudicotyledons	Zygophyllaceae	*Larrea*	*Larrea tridentata*	JF266797
Eudicotyledons	Zygophyllaceae	*Larrea*	*Larrea tridentata*	JF266798
Eudicotyledons	Zygophyllaceae	*Larrea*	*Larrea tridentata*	JF266799
Eudicotyledons	Zygophyllaceae	*Larrea*	*Larrea tridentata*	JF266800
Eudicotyledons	Zygophyllaceae	*Larrea*	*Larrea tridentata*	JF266801
Eudicotyledons	Zygophyllaceae	*Larrea*	*Larrea tridentata*	JF266802
Eudicotyledons	Zygophyllaceae	*Larrea*	*Larrea tridentata*	JF266803
Eudicotyledons	Zygophyllaceae	*Larrea*	*Larrea tridentata*	JF266804
Eudicotyledons	Zygophyllaceae	*Larrea*	*Larrea tridentata*	JF266805
Eudicotyledons	Zygophyllaceae	*Larrea*	*Larrea tridentata*	JF266806
Eudicotyledons	Zygophyllaceae	*Larrea*	*Larrea tridentata*	JF266807
Eudicotyledons	Zygophyllaceae	*Larrea*	*Larrea tridentata*	JF266808
Eudicotyledons	Zygophyllaceae	*Larrea*	*Larrea tridentata*	JF266809
Eudicotyledons	Zygophyllaceae	*Larrea*	*Larrea tridentata*	JF266810
Eudicotyledons	Zygophyllaceae	*Larrea*	*Larrea tridentata*	JF266811
Eudicotyledons	Zygophyllaceae	*Larrea*	*Larrea tridentata*	JF266812
Eudicotyledons	Zygophyllaceae	*Larrea*	*Larrea tridentata*	JF266813
Eudicotyledons	Zygophyllaceae	*Larrea*	*Larrea tridentata*	JF266814
Eudicotyledons	Zygophyllaceae	*Larrea*	*Larrea tridentata*	JF266815
Eudicotyledons	Zygophyllaceae	*Larrea*	*Larrea tridentata*	JF266816

Eudicotyledons	Zygophyllaceae	*Larrea*	*Larrea tridentata*	JF266817
Eudicotyledons	Zygophyllaceae	*Larrea*	*Larrea tridentata*	JF266818
Eudicotyledons	Zygophyllaceae	*Larrea*	*Larrea tridentata*	JF266819
Eudicotyledons	Zygophyllaceae	*Zygophyllum*	*Zygophyllum fabago*	JN047533
Eudicotyledons	Zygophyllaceae	*Zygophyllum*	*Zygophyllum fabago*	JN047534
Eudicotyledons	Zygophyllaceae	*Zygophyllum*	*Zygophyllum fabago*	JN047535
Eudicotyledons	Caryocaraceae	*Caryocar*	*Caryocar glabrum*	GQ428701
Eudicotyledons	Caryocaraceae	*Caryocar*	*Caryocar glabrum*	FJ038884
Eudicotyledons	Caryocaraceae	*Caryocar*	*Caryocar glabrum*	FJ038885
Eudicotyledons	Betulaceae	*Ostryopsis*	*Ostryopsis davidiana*	EU852626
Eudicotyledons	Betulaceae	*Ostryopsis*	*Ostryopsis davidiana*	EU852627
Eudicotyledons	Betulaceae	*Ostryopsis*	*Ostryopsis davidiana*	EU852628
Eudicotyledons	Betulaceae	*Ostryopsis*	*Ostryopsis davidiana*	EU852629
Eudicotyledons	Betulaceae	*Ostryopsis*	*Ostryopsis davidiana*	EU852630
Eudicotyledons	Betulaceae	*Ostryopsis*	*Ostryopsis davidiana*	EU852631
Eudicotyledons	Betulaceae	*Ostryopsis*	*Ostryopsis davidiana*	JN045670
Eudicotyledons	Betulaceae	*Ostryopsis*	*Ostryopsis davidiana*	JN045671
Eudicotyledons	Betulaceae	*Ostryopsis*	*Ostryopsis davidiana*	JN045672
Eudicotyledons	Betulaceae	*Ostryopsis*	*Ostryopsis davidiana*	JN045673
Eudicotyledons	Betulaceae	*Ostryopsis*	*Ostryopsis davidiana*	JN045674
Eudicotyledons	Betulaceae	*Ostryopsis*	*Ostryopsis davidiana*	JN045675
Eudicotyledons	Betulaceae	*Ostryopsis*	*Ostryopsis davidiana*	JN045676
Eudicotyledons	Betulaceae	*Ostryopsis*	*Ostryopsis davidiana*	JN045677
Eudicotyledons	Betulaceae	*Ostryopsis*	*Ostryopsis davidiana*	JN045678
Eudicotyledons	Betulaceae	*Ostryopsis*	*Ostryopsis davidiana*	JN045679
Eudicotyledons	Melianthaceae	*Melianthus*	*Melianthus major*	DQ435423
Eudicotyledons	Melianthaceae	*Melianthus*	*Melianthus major*	DQ435424
Eudicotyledons	Melianthaceae	*Melianthus*	*Melianthus major*	DQ435425
Eudicotyledons	Malvaceae	*Malope*	*Malope trifida*	EF679751
Eudicotyledons	Malvaceae	*Malope*	*Malope trifida*	EF419647
Eudicotyledons	Malvaceae	*Malope*	*Malope trifida*	EF419648
Eudicotyledons	Malvaceae	*Malope*	*Malope trifida*	EF419649
Eudicotyledons	Zygophyllaceae	*Larrea*	*Larrea nitida*	JF266822
Eudicotyledons	Zygophyllaceae	*Larrea*	*Larrea nitida*	JF266824
Eudicotyledons	Malvaceae	*Malva*	*Malva nicaeensis*	EF419592
Eudicotyledons	Malvaceae	*Malva*	*Malva nicaeensis*	EF419593
Eudicotyledons	Malvaceae	*Malva*	*Malva nicaeensis*	EF419594
Eudicotyledons	Malvaceae	*Malva*	*Malva nicaeensis*	EF419595
Eudicotyledons	Malvaceae	*Malva*	*Malva nicaeensis*	EF419596
Eudicotyledons	Asteraceae	*Blumea*	*Blumea mollis*	EU195594
Eudicotyledons	Asteraceae	*Blumea*	*Blumea mollis*	EU195595
Eudicotyledons	Asteraceae	*Blumea*	*Blumea mollis*	EU195597
Eudicotyledons	Asteraceae	*Blumea*	*Blumea mollis*	EF210991
Eudicotyledons	Asteraceae	*Blumea*	*Blumea riparia*	EU195600
Eudicotyledons	Asteraceae	*Blumea*	*Blumea riparia*	EF210993
Eudicotyledons	Solanaceae	*Grabowskia*	*Grabowskia boerhaviifolia*	HM195022
Eudicotyledons	Solanaceae	*Grabowskia*	*Grabowskia boerhaviifolia*	HQ216156
Eudicotyledons	Solanaceae	*Grabowskia*	*Grabowskia boerhaviifolia*	JF284422
Eudicotyledons	Solanaceae	*Grabowskia*	*Grabowskia boerhaviifolia*	JF284423
Eudicotyledons	Solanaceae	*Grabowskia*	*Grabowskia boerhaviifolia*	JF284424
Eudicotyledons	Solanaceae	*Grabowskia*	*Grabowskia boerhaviifolia*	JF284425
Eudicotyledons	Solanaceae	*Grabowskia*	*Grabowskia boerhaviifolia*	JF284426
Eudicotyledons	Solanaceae	*Grabowskia*	*Grabowskia duplicata*	JF284427

Eudicotyledons	Solanaceae	*Grabowskia*	*Grabowskia duplicata*	JF284428
Eudicotyledons	Asteraceae	*Tanacetum*	*Tanacetum corymbosum*	AB683392
Eudicotyledons	Asteraceae	*Tanacetum*	*Tanacetum corymbosum*	FR865113
Eudicotyledons	Asteraceae	*Tanacetum*	*Tanacetum parthenium*	AB683414
Eudicotyledons	Asteraceae	*Tanacetum*	*Tanacetum parthenium*	AB683415
Eudicotyledons	Malvaceae	*Malva*	*Malva dendromorpha*	EF419585
Eudicotyledons	Malvaceae	*Malva*	*Malva dendromorpha*	EF419586
Eudicotyledons	Malvaceae	*Malva*	*Malva dendromorpha*	EF419587
Eudicotyledons	Malvaceae	*Malva*	*Malva linnaei*	EF419588
Eudicotyledons	Malvaceae	*Malva*	*Malva linnaei*	EF419589
Eudicotyledons	Malvaceae	*Malva*	*Malva linnaei*	EF419590
Eudicotyledons	Malvaceae	*Malva*	*Malva linnaei*	EF419591
Eudicotyledons	Malvaceae	*Malva*	*Malva parviflora*	EF419601
Eudicotyledons	Malvaceae	*Malva*	*Malva parviflora*	EF419602
Eudicotyledons	Malvaceae	*Malva*	*Malva parviflora*	EF419603
Eudicotyledons	Malvaceae	*Malva*	*Malva parviflora*	EF419604
Eudicotyledons	Malvaceae	*Malva*	*Malva sylvestris*	EF419599
Eudicotyledons	Malvaceae	*Malva*	*Malva sylvestris*	EF419600
Eudicotyledons	Malvaceae	*Malva*	*Malva sylvestris*	EF590714
Eudicotyledons	Malvaceae	*Malva*	*Malva wigandii*	EF419572
Eudicotyledons	Malvaceae	*Malva*	*Malva wigandii*	EF419573
Eudicotyledons	Malvaceae	*Malva*	*Malva wigandii*	EF419574
Eudicotyledons	Malvaceae	*Malva*	*Malva wigandii*	EF419575
Eudicotyledons	Malvaceae	*Malva*	*Malva wigandii*	EF419576
Eudicotyledons	Asteraceae	*Sinosenecio*	*Sinosenecio koreanus*	JN047167
Eudicotyledons	Asteraceae	*Sinosenecio*	*Sinosenecio koreanus*	JN047168
Eudicotyledons	Asteraceae	*Sinosenecio*	*Sinosenecio koreanus*	JN047169
Eudicotyledons	Orobanchaceae	*Cistanche*	*Cistanche deserticola*	GQ435468
Eudicotyledons	Orobanchaceae	*Cistanche*	*Cistanche deserticola*	GQ435469
Eudicotyledons	Orobanchaceae	*Cistanche*	*Cistanche deserticola*	FJ914368
Eudicotyledons	Orobanchaceae	*Cistanche*	*Cistanche deserticola*	FJ914369
Eudicotyledons	Orobanchaceae	*Cistanche*	*Cistanche deserticola*	FJ914370
Eudicotyledons	Orobanchaceae	*Cistanche*	*Cistanche deserticola*	FJ914371
Eudicotyledons	Orobanchaceae	*Cistanche*	*Cistanche deserticola*	FJ914372
Eudicotyledons	Orobanchaceae	*Cistanche*	*Cistanche deserticola*	FJ914373
Eudicotyledons	Orobanchaceae	*Cistanche*	*Cistanche deserticola*	FJ914374
Eudicotyledons	Orobanchaceae	*Cistanche*	*Cistanche deserticola*	FJ914375
Eudicotyledons	Orobanchaceae	*Cistanche*	*Cistanche salsa*	FJ914376
Eudicotyledons	Orobanchaceae	*Cistanche*	*Cistanche salsa*	FJ914377
Eudicotyledons	Orobanchaceae	*Cistanche*	*Cistanche salsa*	FJ914378
Eudicotyledons	Orobanchaceae	*Cistanche*	*Cistanche tubulosa*	GQ435163
Eudicotyledons	Orobanchaceae	*Cistanche*	*Cistanche tubulosa*	GQ435164
Eudicotyledons	Orobanchaceae	*Cistanche*	*Cistanche tubulosa*	FJ914381
Eudicotyledons	Orobanchaceae	*Cistanche*	*Cistanche tubulosa*	FJ914382
Eudicotyledons	Orobanchaceae	*Cistanche*	*Cistanche tubulosa*	FJ914383
Eudicotyledons	Orobanchaceae	*Cistanche*	*Cistanche tubulosa*	FJ914384
Eudicotyledons	Orobanchaceae	*Cistanche*	*Cistanche tubulosa*	FJ914385
Eudicotyledons	Orobanchaceae	*Cistanche*	*Cistanche tubulosa*	FJ914386
Eudicotyledons	Stachyuraceae	*Stachyurus*	*Stachyurus himalaicus*	JN047255
Eudicotyledons	Stachyuraceae	*Stachyurus*	*Stachyurus himalaicus*	JN047256
Eudicotyledons	Stachyuraceae	*Stachyurus*	*Stachyurus himalaicus*	JN047257
Eudicotyledons	Stachyuraceae	*Stachyurus*	*Stachyurus chinensis*	JN047251
Eudicotyledons	Stachyuraceae	*Stachyurus*	*Stachyurus chinensis*	JN047252

Eudicotyledons	Stachyuraceae	*Stachyurus*	*Stachyurus chinensis*	JN047253
Eudicotyledons	Stachyuraceae	*Stachyurus*	*Stachyurus chinensis*	JN047254
Eudicotyledons	Brassicaceae	*Draba*	*Draba nemorosa*	JN044449
Eudicotyledons	Brassicaceae	*Draba*	*Draba nemorosa*	JN044450
Eudicotyledons	Betulaceae	*Ostryopsis*	*Ostryopsis nobilis*	AY211457
Eudicotyledons	Betulaceae	*Ostryopsis*	*Ostryopsis nobilis*	FJ011866
Eudicotyledons	Betulaceae	*Ostryopsis*	*Ostryopsis nobilis*	JN045685
Eudicotyledons	Betulaceae	*Ostryopsis*	*Ostryopsis nobilis*	JN045686
Eudicotyledons	Betulaceae	*Ostryopsis*	*Ostryopsis nobilis*	JN045687
Eudicotyledons	Betulaceae	*Ostryopsis*	*Ostryopsis nobilis*	JN045688
Eudicotyledons	Betulaceae	*Ostryopsis*	*Ostryopsis nobilis*	JN045689
Eudicotyledons	Malvaceae	*Talipariti*	*Talipariti macrophyllum*	AB181124
Eudicotyledons	Malvaceae	*Talipariti*	*Talipariti macrophyllum*	AY727165
Eudicotyledons	Malvaceae	*Talipariti*	*Talipariti tiliaceum*	AB181110
Eudicotyledons	Malvaceae	*Talipariti*	*Talipariti tiliaceum*	AB181111
Eudicotyledons	Malvaceae	*Talipariti*	*Talipariti tiliaceum*	AB181112
Eudicotyledons	Malvaceae	*Talipariti*	*Talipariti tiliaceum*	AB181113
Eudicotyledons	Malvaceae	*Talipariti*	*Talipariti tiliaceum*	AB181114
Eudicotyledons	Malvaceae	*Talipariti*	*Talipariti tiliaceum*	AB181115
Eudicotyledons	Malvaceae	*Talipariti*	*Talipariti tiliaceum*	AB181116
Eudicotyledons	Malvaceae	*Talipariti*	*Talipariti tiliaceum*	AB181117
Eudicotyledons	Malvaceae	*Talipariti*	*Talipariti tiliaceum*	AB181118
Eudicotyledons	Malvaceae	*Talipariti*	*Talipariti tiliaceum*	AB181119
Eudicotyledons	Malvaceae	*Talipariti*	*Talipariti tiliaceum*	AB181120
Eudicotyledons	Malvaceae	*Talipariti*	*Talipariti tiliaceum*	AB181121
Eudicotyledons	Malvaceae	*Talipariti*	*Talipariti tiliaceum*	AB181122
Eudicotyledons	Malvaceae	*Talipariti*	*Talipariti tiliaceum*	GU135393
Eudicotyledons	Malvaceae	*Malva*	*Malva neglecta*	HQ596765
Eudicotyledons	Malvaceae	*Malva*	*Malva neglecta*	EF419597
Eudicotyledons	Malvaceae	*Malva*	*Malva neglecta*	EF419598
Eudicotyledons	Asteraceae	*Sinosenecio*	*Sinosenecio globiger*	JN047149
Eudicotyledons	Asteraceae	*Sinosenecio*	*Sinosenecio globiger*	JN047150
Eudicotyledons	Asteraceae	*Sinosenecio*	*Sinosenecio globiger*	JN047151
Eudicotyledons	Asteraceae	*Sinosenecio*	*Sinosenecio globiger*	JN047152
Eudicotyledons	Asteraceae	*Sinosenecio*	*Sinosenecio oldhamianus*	JN047177
Eudicotyledons	Asteraceae	*Sinosenecio*	*Sinosenecio oldhamianus*	JN047178
Eudicotyledons	Asteraceae	*Sinosenecio*	*Sinosenecio oldhamianus*	JN047179
Eudicotyledons	Asteraceae	*Sinosenecio*	*Sinosenecio subcoriaceus*	JN047190
Eudicotyledons	Asteraceae	*Sinosenecio*	*Sinosenecio subcoriaceus*	JN047191
Eudicotyledons	Asteraceae	*Sinosenecio*	*Sinosenecio subcoriaceus*	JN047192
Eudicotyledons	Asteraceae	*Sinosenecio*	*Sinosenecio subcoriaceus*	JN047193
Eudicotyledons	Zygophyllaceae	*Zygophyllum*	*Zygophyllum pterocarpum*	JN047540
Eudicotyledons	Zygophyllaceae	*Zygophyllum*	*Zygophyllum pterocarpum*	JN047541
Eudicotyledons	Brassicaceae	*Draba*	*Draba lanceolata*	JN044445
Eudicotyledons	Brassicaceae	*Draba*	*Draba lanceolata*	JN044446
Eudicotyledons	Brassicaceae	*Draba*	*Draba lanceolata*	JN044447
Eudicotyledons	Brassicaceae	*Draba*	*Draba lanceolata*	JN044448
Eudicotyledons	Cucurbitaceae	*Hemsleya*	*Hemsleya lijiangensis*	EF424070
Eudicotyledons	Cucurbitaceae	*Hemsleya*	*Hemsleya lijiangensis*	JN044845
Eudicotyledons	Cucurbitaceae	*Hemsleya*	*Hemsleya lijiangensis*	JN044846
Eudicotyledons	Cucurbitaceae	*Hemsleya*	*Hemsleya lijiangensis*	JN044847
Eudicotyledons	Cucurbitaceae	*Hemsleya*	*Hemsleya lijiangensis*	JN044848
Eudicotyledons	Cucurbitaceae	*Hemsleya*	*Hemsleya lijiangensis*	JN044849

Eudicotyledons	Asteraceae	*Sinosenecio*	*Sinosenecio bodinieri*	JN047129
Eudicotyledons	Asteraceae	*Sinosenecio*	*Sinosenecio bodinieri*	JN047130
Eudicotyledons	Asteraceae	*Sinosenecio*	*Sinosenecio septilobus*	JN047185
Eudicotyledons	Asteraceae	*Sinosenecio*	*Sinosenecio septilobus*	JN047186
Eudicotyledons	Asteraceae	*Sinosenecio*	*Sinosenecio septilobus*	JN047187
Eudicotyledons	Thymelaeaceae	*Daphne*	*Daphne laureola*	GQ167445
Eudicotyledons	Thymelaeaceae	*Daphne*	*Daphne laureola*	GQ167446
Eudicotyledons	Thymelaeaceae	*Daphne*	*Daphne laureola*	GQ167447
Eudicotyledons	Thymelaeaceae	*Daphne*	*Daphne laureola*	GQ167448
Eudicotyledons	Thymelaeaceae	*Daphne*	*Daphne laureola*	GQ167449
Eudicotyledons	Thymelaeaceae	*Daphne*	*Daphne laureola*	GQ167450
Eudicotyledons	Thymelaeaceae	*Daphne*	*Daphne laureola*	GQ167451
Eudicotyledons	Thymelaeaceae	*Daphne*	*Daphne laureola*	GQ167452
Eudicotyledons	Thymelaeaceae	*Daphne*	*Daphne laureola*	GQ167453
Eudicotyledons	Thymelaeaceae	*Daphne*	*Daphne laureola*	GQ167454
Eudicotyledons	Thymelaeaceae	*Daphne*	*Daphne laureola*	GQ167455
Eudicotyledons	Thymelaeaceae	*Daphne*	*Daphne laureola*	GQ167456
Eudicotyledons	Thymelaeaceae	*Daphne*	*Daphne laureola*	GQ167457
Eudicotyledons	Thymelaeaceae	*Daphne*	*Daphne laureola*	GQ167458
Eudicotyledons	Thymelaeaceae	*Daphne*	*Daphne laureola*	GQ167459
Eudicotyledons	Thymelaeaceae	*Daphne*	*Daphne laureola*	GQ167460
Eudicotyledons	Thymelaeaceae	*Daphne*	*Daphne laureola*	GQ167461
Eudicotyledons	Thymelaeaceae	*Daphne*	*Daphne laureola*	GQ167462
Eudicotyledons	Thymelaeaceae	*Daphne*	*Daphne laureola*	GQ167463
Eudicotyledons	Thymelaeaceae	*Daphne*	*Daphne laureola*	GQ167464
Eudicotyledons	Thymelaeaceae	*Daphne*	*Daphne laureola*	GQ167465
Eudicotyledons	Thymelaeaceae	*Daphne*	*Daphne laureola*	GQ167466
Eudicotyledons	Thymelaeaceae	*Daphne*	*Daphne laureola*	GQ167467
Eudicotyledons	Thymelaeaceae	*Daphne*	*Daphne laureola*	GQ167468
Eudicotyledons	Thymelaeaceae	*Daphne*	*Daphne laureola*	GQ167469
Eudicotyledons	Thymelaeaceae	*Daphne*	*Daphne laureola*	GQ167470
Eudicotyledons	Thymelaeaceae	*Daphne*	*Daphne laureola*	GQ167471
Eudicotyledons	Thymelaeaceae	*Daphne*	*Daphne laureola*	GQ167472
Eudicotyledons	Thymelaeaceae	*Daphne*	*Daphne laureola*	GQ167473
Eudicotyledons	Thymelaeaceae	*Daphne*	*Daphne laureola*	GQ167474
Eudicotyledons	Thymelaeaceae	*Daphne*	*Daphne laureola*	GQ167475
Eudicotyledons	Thymelaeaceae	*Daphne*	*Daphne laureola*	GQ167476
Eudicotyledons	Thymelaeaceae	*Daphne*	*Daphne laureola*	GQ167477
Eudicotyledons	Thymelaeaceae	*Daphne*	*Daphne laureola*	GQ167478
Eudicotyledons	Thymelaeaceae	*Daphne*	*Daphne laureola*	GQ167479
Eudicotyledons	Thymelaeaceae	*Daphne*	*Daphne laureola*	GQ167480
Eudicotyledons	Thymelaeaceae	*Daphne*	*Daphne laureola*	GQ167481
Eudicotyledons	Thymelaeaceae	*Daphne*	*Daphne laureola*	GQ167482
Eudicotyledons	Thymelaeaceae	*Daphne*	*Daphne laureola*	GQ167483
Eudicotyledons	Thymelaeaceae	*Daphne*	*Daphne laureola*	GQ167484
Eudicotyledons	Thymelaeaceae	*Daphne*	*Daphne laureola*	GQ167485
Eudicotyledons	Thymelaeaceae	*Daphne*	*Daphne laureola*	GQ167486
Eudicotyledons	Thymelaeaceae	*Daphne*	*Daphne cneorum*	GQ167435
Eudicotyledons	Thymelaeaceae	*Daphne*	*Daphne cneorum*	GQ167436
Eudicotyledons	Thymelaeaceae	*Daphne*	*Daphne cneorum*	GQ167437
Eudicotyledons	Thymelaeaceae	*Daphne*	*Daphne cneorum*	GQ167438
Eudicotyledons	Thymelaeaceae	*Daphne*	*Daphne gnidium*	GQ167439
Eudicotyledons	Thymelaeaceae	*Daphne*	*Daphne gnidium*	GQ167440

Eudicotyledons	Thymelaeaceae	*Daphne*	*Daphne gnidium*	GQ167441
Eudicotyledons	Thymelaeaceae	*Daphne*	*Daphne gnidium*	GQ167442
Eudicotyledons	Thymelaeaceae	*Daphne*	*Daphne gnidium*	GQ167443
Eudicotyledons	Thymelaeaceae	*Daphne*	*Daphne gnidium*	GQ167444
Eudicotyledons	Loranthaceae	*Helixanthera*	*Helixanthera parasitica*	HQ317802
Eudicotyledons	Loranthaceae	*Helixanthera*	*Helixanthera parasitica*	HQ317803
Eudicotyledons	Loranthaceae	*Helixanthera*	*Helixanthera parasitica*	HQ317804
Eudicotyledons	Loranthaceae	*Helixanthera*	*Helixanthera parasitica*	HQ317805
Eudicotyledons	Loranthaceae	*Helixanthera*	*Helixanthera parasitica*	JN687573
Eudicotyledons	Orobanchaceae	*Cistanche*	*Cistanche sinensis*	FJ914379
Eudicotyledons	Orobanchaceae	*Cistanche*	*Cistanche sinensis*	FJ914380
Eudicotyledons	Zygophyllaceae	*Zygophyllum*	*Zygophyllum rosowii*	JN047542
Eudicotyledons	Zygophyllaceae	*Zygophyllum*	*Zygophyllum rosowii*	JN047543
Eudicotyledons	Boraginaceae	*Cordia*	*Cordia alliodora*	JQ710508
Eudicotyledons	Boraginaceae	*Cordia*	*Cordia alliodora*	JQ710509
Eudicotyledons	Boraginaceae	*Cordia*	*Cordia alliodora*	JQ710510
Eudicotyledons	Boraginaceae	*Cordia*	*Cordia alliodora*	JQ710511
Eudicotyledons	Boraginaceae	*Cordia*	*Cordia alliodora*	JQ710512
Eudicotyledons	Boraginaceae	*Cordia*	*Cordia alliodora*	JQ710513
Eudicotyledons	Boraginaceae	*Cordia*	*Cordia alliodora*	JQ710514
Eudicotyledons	Boraginaceae	*Cordia*	*Cordia alliodora*	JQ710515
Eudicotyledons	Boraginaceae	*Cordia*	*Cordia alliodora*	JQ710516
Eudicotyledons	Boraginaceae	*Cordia*	*Cordia alliodora*	JQ710517
Eudicotyledons	Boraginaceae	*Cordia*	*Cordia alliodora*	JQ710518
Eudicotyledons	Boraginaceae	*Cordia*	*Cordia alliodora*	JQ710519
Eudicotyledons	Boraginaceae	*Cordia*	*Cordia alliodora*	JQ710520
Eudicotyledons	Boraginaceae	*Cordia*	*Cordia alliodora*	JQ710521
Eudicotyledons	Boraginaceae	*Cordia*	*Cordia alliodora*	JQ710522
Eudicotyledons	Boraginaceae	*Cordia*	*Cordia alliodora*	JQ710523
Eudicotyledons	Boraginaceae	*Cordia*	*Cordia alliodora*	JQ710524
Eudicotyledons	Boraginaceae	*Cordia*	*Cordia alliodora*	JQ710525
Eudicotyledons	Boraginaceae	*Cordia*	*Cordia alliodora*	JQ710526
Eudicotyledons	Boraginaceae	*Cordia*	*Cordia alliodora*	JQ710527
Eudicotyledons	Boraginaceae	*Cordia*	*Cordia alliodora*	JQ710528
Eudicotyledons	Boraginaceae	*Cordia*	*Cordia alliodora*	JQ710529
Eudicotyledons	Boraginaceae	*Cordia*	*Cordia alliodora*	JQ710530
Eudicotyledons	Boraginaceae	*Cordia*	*Cordia alliodora*	JQ710531
Eudicotyledons	Boraginaceae	*Cordia*	*Cordia alliodora*	JQ710532
Eudicotyledons	Boraginaceae	*Cordia*	*Cordia alliodora*	JQ710533
Eudicotyledons	Boraginaceae	*Cordia*	*Cordia alliodora*	JQ710534
Eudicotyledons	Boraginaceae	*Cordia*	*Cordia alliodora*	JQ710535
Eudicotyledons	Boraginaceae	*Cordia*	*Cordia alliodora*	JQ710536
Eudicotyledons	Boraginaceae	*Cordia*	*Cordia alliodora*	JQ710537
Eudicotyledons	Boraginaceae	*Cordia*	*Cordia alliodora*	JQ710538
Eudicotyledons	Boraginaceae	*Cordia*	*Cordia alliodora*	JQ710539
Eudicotyledons	Boraginaceae	*Cordia*	*Cordia alliodora*	JQ710540
Eudicotyledons	Boraginaceae	*Cordia*	*Cordia alliodora*	JQ710541
Eudicotyledons	Boraginaceae	*Cordia*	*Cordia alliodora*	JQ710542
Eudicotyledons	Boraginaceae	*Cordia*	*Cordia alliodora*	JQ710543
Eudicotyledons	Boraginaceae	*Cordia*	*Cordia alliodora*	JQ710544
Eudicotyledons	Boraginaceae	*Cordia*	*Cordia alliodora*	JQ710545
Eudicotyledons	Boraginaceae	*Cordia*	*Cordia alliodora*	JQ710546
Eudicotyledons	Boraginaceae	*Cordia*	*Cordia alliodora*	JQ710547

Eudicotyledons	Boraginaceae	*Cordia*	*Cordia alliodora*	JQ710548
Eudicotyledons	Boraginaceae	*Cordia*	*Cordia alliodora*	JQ710549
Eudicotyledons	Boraginaceae	*Cordia*	*Cordia alliodora*	JQ710550
Eudicotyledons	Boraginaceae	*Cordia*	*Cordia alliodora*	JQ710551
Eudicotyledons	Boraginaceae	*Cordia*	*Cordia alliodora*	GQ982196
Eudicotyledons	Malvaceae	*Talipariti*	*Talipariti glabrum*	AB181101
Eudicotyledons	Malvaceae	*Talipariti*	*Talipariti glabrum*	AB181102
Eudicotyledons	Malvaceae	*Talipariti*	*Talipariti glabrum*	AB181103
Eudicotyledons	Malvaceae	*Talipariti*	*Talipariti glabrum*	AB181104
Eudicotyledons	Malvaceae	*Talipariti*	*Talipariti glabrum*	AB181105
Eudicotyledons	Malvaceae	*Talipariti*	*Talipariti glabrum*	AB181106
Eudicotyledons	Malvaceae	*Talipariti*	*Talipariti glabrum*	AB181107
Eudicotyledons	Malvaceae	*Talipariti*	*Talipariti glabrum*	AB181108
Eudicotyledons	Malvaceae	*Talipariti*	*Talipariti glabrum*	AB181109
Eudicotyledons	Asteraceae	*Tanacetum*	*Tanacetum abrotanifolium*	AB683373
Eudicotyledons	Asteraceae	*Tanacetum*	*Tanacetum abrotanifolium*	AB683374
Eudicotyledons	Asteraceae	*Tanacetum*	*Tanacetum balsamita*	AB683382
Eudicotyledons	Asteraceae	*Tanacetum*	*Tanacetum balsamita*	AB683383
Eudicotyledons	Asteraceae	*Tanacetum*	*Tanacetum coccineum*	AB683391
Eudicotyledons	Asteraceae	*Tanacetum*	*Tanacetum coccineum*	FR689920
Eudicotyledons	Asteraceae	*Tanacetum*	*Tanacetum pinnatum*	AB683416
Eudicotyledons	Asteraceae	*Tanacetum*	*Tanacetum pinnatum*	AB683417
Eudicotyledons	Asteraceae	*Tanacetum*	*Tanacetum punctatum*	AB683424
Eudicotyledons	Asteraceae	*Tanacetum*	*Tanacetum punctatum*	AB683425
Eudicotyledons	Asteraceae	*Tanacetum*	*Tanacetum kotschyi*	AB683405
Eudicotyledons	Asteraceae	*Tanacetum*	*Tanacetum kotschyi*	AB683406
Eudicotyledons	Rubiaceae	*Razafimandimbisonia*	*Razafimandimbisonia minor*	AM939390
Eudicotyledons	Rubiaceae	*Razafimandimbisonia*	*Razafimandimbisonia minor*	AM939391
Eudicotyledons	Rubiaceae	*Razafimandimbisonia*	*Razafimandimbisonia minor*	AM939392
Eudicotyledons	Rubiaceae	*Razafimandimbisonia*	*Razafimandimbisonia minor*	AM939393
Eudicotyledons	Solanaceae	*Grabowskia*	*Grabowskia obtusa*	JF284429
Eudicotyledons	Solanaceae	*Grabowskia*	*Grabowskia obtusa*	JF284430
Eudicotyledons	Sapotaceae	*Pouteria*	*Pouteria guianensis*	DQ344134
Eudicotyledons	Sapotaceae	*Pouteria*	*Pouteria guianensis*	GQ428712
Eudicotyledons	Sapotaceae	*Pouteria*	*Pouteria guianensis*	GQ428713
Eudicotyledons	Stachyuraceae	*Stachyurus*	*Stachyurus yunnanensis*	JN047258
Eudicotyledons	Stachyuraceae	*Stachyurus*	*Stachyurus yunnanensis*	JN047259
Eudicotyledons	Stachyuraceae	*Stachyurus*	*Stachyurus yunnanensis*	JN047260
Eudicotyledons	Melianthaceae	*Melianthus*	*Melianthus comosus*	DQ435427
Eudicotyledons	Melianthaceae	*Melianthus*	*Melianthus comosus*	DQ435428
Eudicotyledons	Melianthaceae	*Melianthus*	*Melianthus comosus*	DQ435429
Eudicotyledons	Melianthaceae	*Melianthus*	*Melianthus comosus*	DQ435430
Eudicotyledons	Melianthaceae	*Melianthus*	*Melianthus comosus*	DQ435431
Eudicotyledons	Melianthaceae	*Melianthus*	*Melianthus pectinatus*	DQ435434
Eudicotyledons	Melianthaceae	*Melianthus*	*Melianthus pectinatus*	DQ435435
Eudicotyledons	Sapotaceae	*Pouteria*	*Pouteria torta*	FJ039081
Eudicotyledons	Sapotaceae	*Pouteria*	*Pouteria torta*	FJ039082
Eudicotyledons	Asteraceae	*Blumea*	*Blumea aromatica*	EU195586
Eudicotyledons	Asteraceae	*Blumea*	*Blumea aromatica*	EF210998
Eudicotyledons	Asteraceae	*Blumea*	*Blumea clarkei*	EU195587
Eudicotyledons	Asteraceae	*Blumea*	*Blumea clarkei*	EF211020
Eudicotyledons	Asteraceae	*Blumea*	*Blumea densiflora*	EU195588
Eudicotyledons	Asteraceae	*Blumea*	*Blumea densiflora*	EF210983

Eudicotyledons	Asteraceae	*Blumea*	*Blumea fistulosa*	EU195589
Eudicotyledons	Asteraceae	*Blumea*	*Blumea fistulosa*	EF210984
Eudicotyledons	Asteraceae	*Blumea*	*Blumea megacephala*	EU195593
Eudicotyledons	Asteraceae	*Blumea*	*Blumea megacephala*	EF211010
Eudicotyledons	Asteraceae	*Blumea*	*Blumea paniculata*	EU195599
Eudicotyledons	Asteraceae	*Blumea*	*Blumea paniculata*	EF210990
Eudicotyledons	Asteraceae	*Blumea*	*Blumea sinuata*	EU195601
Eudicotyledons	Asteraceae	*Blumea*	*Blumea sinuata*	EF210996
Eudicotyledons	Asteraceae	*Blumea*	*Blumea virens*	EU195602
Eudicotyledons	Asteraceae	*Blumea*	*Blumea virens*	EF211005
Eudicotyledons	Malvaceae	*Malva*	*Malva alcea*	GQ248337
Eudicotyledons	Malvaceae	*Malva*	*Malva alcea*	EF419609
Eudicotyledons	Malvaceae	*Malva*	*Malva alcea*	EF419610
Eudicotyledons	Malvaceae	*Malva*	*Malva alcea*	EF419611
Eudicotyledons	Malvaceae	*Malva*	*Malva alcea*	EF590713
Eudicotyledons	Malvaceae	*Malva*	*Malva alcea*	HM214493
Eudicotyledons	Malvaceae	*Malva*	*Malva alcea*	HM214494
Eudicotyledons	Malvaceae	*Malva*	*Malva alcea*	HM214495
Eudicotyledons	Malvaceae	*Malva*	*Malva alcea*	HM214496
Eudicotyledons	Malvaceae	*Malva*	*Malva alcea*	HM214497
Eudicotyledons	Malvaceae	*Malva*	*Malva alcea*	HM214498
Eudicotyledons	Malvaceae	*Malva*	*Malva alcea*	HM214499
Eudicotyledons	Malvaceae	*Malva*	*Malva alcea*	HM214500
Eudicotyledons	Malvaceae	*Malva*	*Malva alcea*	HM214501
Eudicotyledons	Malvaceae	*Malva*	*Malva alcea*	HM214502
Eudicotyledons	Malvaceae	*Malva*	*Malva alcea*	HM214503
Eudicotyledons	Malvaceae	*Malva*	*Malva alcea*	HM214504
Eudicotyledons	Malvaceae	*Malva*	*Malva alcea*	HM214505
Eudicotyledons	Malvaceae	*Alcea*	*Alcea angulata*	GU166775
Eudicotyledons	Malvaceae	*Alcea*	*Alcea angulata*	GU166776
Eudicotyledons	Malvaceae	*Alcea*	*Alcea pallida*	EF679740
Eudicotyledons	Malvaceae	*Alcea*	*Alcea pallida*	EF419661
Eudicotyledons	Malvaceae	*Malope*	*Malope malacoides*	EF419650
Eudicotyledons	Malvaceae	*Malope*	*Malope malacoides*	EF419651
Eudicotyledons	Malvaceae	*Malva*	*Malva aegyptia*	EF419630
Eudicotyledons	Malvaceae	*Malva*	*Malva aegyptia*	EF419631
Eudicotyledons	Malvaceae	*Malva*	*Malva aegyptia*	EF419632
Eudicotyledons	Malvaceae	*Malva*	*Malva hispanica*	EF419606
Eudicotyledons	Malvaceae	*Malva*	*Malva hispanica*	EF419607
Eudicotyledons	Malvaceae	*Malva*	*Malva hispanica*	EF419608
Eudicotyledons	Malvaceae	*Malva*	*Malva moschata*	EF419615
Eudicotyledons	Malvaceae	*Malva*	*Malva moschata*	EF419616
Eudicotyledons	Malvaceae	*Malva*	*Malva moschata*	EF419617
Eudicotyledons	Malvaceae	*Malva*	*Malva moschata*	EF419618
Eudicotyledons	Malvaceae	*Malva*	*Malva moschata*	EF419619
Eudicotyledons	Malvaceae	*Malva*	*Malva moschata*	HM214513
Eudicotyledons	Malvaceae	*Malva*	*Malva moschata*	HM214514
Eudicotyledons	Malvaceae	*Malva*	*Malva tournefortiana*	EF419612
Eudicotyledons	Malvaceae	*Malva*	*Malva tournefortiana*	EF419613
Eudicotyledons	Malvaceae	*Malva*	*Malva tournefortiana*	EF419614
Eudicotyledons	Malvaceae	*Malva*	*Malva trifida*	EF419633
Eudicotyledons	Malvaceae	*Malva*	*Malva trifida*	EF419634
Eudicotyledons	Malvaceae	*Malva*	*Malva trifida*	EF419635

Eudicotyledons	Malvaceae	*Malva*	*Malva trifida*	EF419636
Eudicotyledons	Cucurbitaceae	*Hemsleya*	*Hemsleya carnosiflora*	EF621685
Eudicotyledons	Cucurbitaceae	*Hemsleya*	*Hemsleya carnosiflora*	JN044807
Eudicotyledons	Cucurbitaceae	*Hemsleya*	*Hemsleya carnosiflora*	JN044808
Eudicotyledons	Cucurbitaceae	*Hemsleya*	*Hemsleya carnosiflora*	JN044809
Eudicotyledons	Cucurbitaceae	*Hemsleya*	*Hemsleya carnosiflora*	JN044810
Eudicotyledons	Cucurbitaceae	*Hemsleya*	*Hemsleya chengyihana*	EF621705
Eudicotyledons	Cucurbitaceae	*Hemsleya*	*Hemsleya chengyihana*	JN044811
Eudicotyledons	Cucurbitaceae	*Hemsleya*	*Hemsleya chengyihana*	JN044812
Eudicotyledons	Cucurbitaceae	*Hemsleya*	*Hemsleya chengyihana*	JN044813
Eudicotyledons	Cucurbitaceae	*Hemsleya*	*Hemsleya chengyihana*	JN044814
Eudicotyledons	Cucurbitaceae	*Hemsleya*	*Hemsleya chengyihana*	JN044815
Eudicotyledons	Cucurbitaceae	*Hemsleya*	*Hemsleya dipterygia*	EF621704
Eudicotyledons	Cucurbitaceae	*Hemsleya*	*Hemsleya dipterygia*	JN044824
Eudicotyledons	Cucurbitaceae	*Hemsleya*	*Hemsleya dipterygia*	JN044825
Eudicotyledons	Cucurbitaceae	*Hemsleya*	*Hemsleya dipterygia*	JN044826
Eudicotyledons	Cucurbitaceae	*Hemsleya*	*Hemsleya ellipsoidea*	EF621703
Eudicotyledons	Cucurbitaceae	*Hemsleya*	*Hemsleya ellipsoidea*	JN044827
Eudicotyledons	Cucurbitaceae	*Hemsleya*	*Hemsleya ellipsoidea*	JN044828
Eudicotyledons	Cucurbitaceae	*Hemsleya*	*Hemsleya ellipsoidea*	JN044829
Eudicotyledons	Cucurbitaceae	*Hemsleya*	*Hemsleya ellipsoidea*	JN044830
Eudicotyledons	Cucurbitaceae	*Hemsleya*	*Hemsleya ellipsoidea*	JN044831
Eudicotyledons	Cucurbitaceae	*Hemsleya*	*Hemsleya endecaphylla*	EF621702
Eudicotyledons	Cucurbitaceae	*Hemsleya*	*Hemsleya endecaphylla*	JN044832
Eudicotyledons	Cucurbitaceae	*Hemsleya*	*Hemsleya endecaphylla*	JN044833
Eudicotyledons	Cucurbitaceae	*Hemsleya*	*Hemsleya gigantha*	EF621701
Eudicotyledons	Cucurbitaceae	*Hemsleya*	*Hemsleya gigantha*	JN044834
Eudicotyledons	Cucurbitaceae	*Hemsleya*	*Hemsleya gigantha*	JN044835
Eudicotyledons	Cucurbitaceae	*Hemsleya*	*Hemsleya gigantha*	JN044836
Eudicotyledons	Cucurbitaceae	*Hemsleya*	*Hemsleya gigantha*	JN044837
Eudicotyledons	Cucurbitaceae	*Hemsleya*	*Hemsleya graciliflora*	EF621700
Eudicotyledons	Cucurbitaceae	*Hemsleya*	*Hemsleya graciliflora*	JN044838
Eudicotyledons	Cucurbitaceae	*Hemsleya*	*Hemsleya graciliflora*	JN044839
Eudicotyledons	Cucurbitaceae	*Hemsleya*	*Hemsleya graciliflora*	JN044840
Eudicotyledons	Cucurbitaceae	*Hemsleya*	*Hemsleya graciliflora*	JN044841
Eudicotyledons	Cucurbitaceae	*Hemsleya*	*Hemsleya longicarpa*	EF621699
Eudicotyledons	Cucurbitaceae	*Hemsleya*	*Hemsleya longicarpa*	JN044850
Eudicotyledons	Cucurbitaceae	*Hemsleya*	*Hemsleya longicarpa*	JN044851
Eudicotyledons	Cucurbitaceae	*Hemsleya*	*Hemsleya macrosperma*	EF621697
Eudicotyledons	Cucurbitaceae	*Hemsleya*	*Hemsleya macrosperma*	JN044858
Eudicotyledons	Cucurbitaceae	*Hemsleya*	*Hemsleya macrosperma*	JN044859
Eudicotyledons	Cucurbitaceae	*Hemsleya*	*Hemsleya macrosperma*	JN044860
Eudicotyledons	Cucurbitaceae	*Hemsleya*	*Hemsleya macrosperma*	JN044861
Eudicotyledons	Cucurbitaceae	*Hemsleya*	*Hemsleya mitrata*	EF621696
Eudicotyledons	Cucurbitaceae	*Hemsleya*	*Hemsleya mitrata*	JN044862
Eudicotyledons	Cucurbitaceae	*Hemsleya*	*Hemsleya mitrata*	JN044863
Eudicotyledons	Cucurbitaceae	*Hemsleya*	*Hemsleya mitrata*	JN044864
Eudicotyledons	Cucurbitaceae	*Hemsleya*	*Hemsleya mitrata*	JN044865
Eudicotyledons	Cucurbitaceae	*Hemsleya*	*Hemsleya omeiensis*	EF621695
Eudicotyledons	Cucurbitaceae	*Hemsleya*	*Hemsleya omeiensis*	JN044866
Eudicotyledons	Cucurbitaceae	*Hemsleya*	*Hemsleya omeiensis*	JN044867
Eudicotyledons	Cucurbitaceae	*Hemsleya*	*Hemsleya omeiensis*	JN044868
Eudicotyledons	Cucurbitaceae	*Hemsleya*	*Hemsleya panacis-scandens*	EF621694

Eudicotyledons	Cucurbitaceae	*Hemsleya*	*Hemsleya panacis-scandens*	JN044869
Eudicotyledons	Cucurbitaceae	*Hemsleya*	*Hemsleya panacis-scandens*	JN044870
Eudicotyledons	Cucurbitaceae	*Hemsleya*	*Hemsleya panacis-scandens*	JN044871
Eudicotyledons	Cucurbitaceae	*Hemsleya*	*Hemsleya panacis-scandens*	JN044872
Eudicotyledons	Cucurbitaceae	*Hemsleya*	*Hemsleya panlongqi*	EF621693
Eudicotyledons	Cucurbitaceae	*Hemsleya*	*Hemsleya panlongqi*	JN044873
Eudicotyledons	Cucurbitaceae	*Hemsleya*	*Hemsleya panlongqi*	JN044874
Eudicotyledons	Cucurbitaceae	*Hemsleya*	*Hemsleya panlongqi*	JN044875
Eudicotyledons	Cucurbitaceae	*Hemsleya*	*Hemsleya pengxianensis*	EF621692
Eudicotyledons	Cucurbitaceae	*Hemsleya*	*Hemsleya pengxianensis*	JN044876
Eudicotyledons	Cucurbitaceae	*Hemsleya*	*Hemsleya pengxianensis*	JN044877
Eudicotyledons	Cucurbitaceae	*Hemsleya*	*Hemsleya pengxianensis*	JN044878
Eudicotyledons	Cucurbitaceae	*Hemsleya*	*Hemsleya turbinata*	EF621690
Eudicotyledons	Cucurbitaceae	*Hemsleya*	*Hemsleya turbinata*	JN044879
Eudicotyledons	Cucurbitaceae	*Hemsleya*	*Hemsleya turbinata*	JN044880
Eudicotyledons	Cucurbitaceae	*Hemsleya*	*Hemsleya turbinata*	JN044881
Eudicotyledons	Cucurbitaceae	*Hemsleya*	*Hemsleya turbinata*	JN044882
Eudicotyledons	Cucurbitaceae	*Hemsleya*	*Hemsleya zhejiangensis*	EF621689
Eudicotyledons	Cucurbitaceae	*Hemsleya*	*Hemsleya zhejiangensis*	JN044883
Eudicotyledons	Cucurbitaceae	*Hemsleya*	*Hemsleya zhejiangensis*	JN044884
Eudicotyledons	Cucurbitaceae	*Hemsleya*	*Hemsleya zhejiangensis*	JN044885
Eudicotyledons	Cucurbitaceae	*Hemsleya*	*Hemsleya macrocarpa*	EF621698
Eudicotyledons	Cucurbitaceae	*Hemsleya*	*Hemsleya macrocarpa*	JN044852
Eudicotyledons	Cucurbitaceae	*Hemsleya*	*Hemsleya macrocarpa*	JN044853
Eudicotyledons	Cucurbitaceae	*Hemsleya*	*Hemsleya macrocarpa*	JN044854
Eudicotyledons	Cucurbitaceae	*Hemsleya*	*Hemsleya macrocarpa*	JN044855
Eudicotyledons	Cucurbitaceae	*Hemsleya*	*Hemsleya macrocarpa*	JN044856
Eudicotyledons	Cucurbitaceae	*Hemsleya*	*Hemsleya macrocarpa*	JN044857
Eudicotyledons	Cucurbitaceae	*Hemsleya*	*Hemsleya amabilis*	EF424071
Eudicotyledons	Cucurbitaceae	*Hemsleya*	*Hemsleya amabilis*	JN044795
Eudicotyledons	Cucurbitaceae	*Hemsleya*	*Hemsleya amabilis*	JN044796
Eudicotyledons	Cucurbitaceae	*Hemsleya*	*Hemsleya amabilis*	JN044797
Eudicotyledons	Cucurbitaceae	*Hemsleya*	*Hemsleya amabilis*	JN044798
Eudicotyledons	Cucurbitaceae	*Hemsleya*	*Hemsleya amabilis*	JN044799
Eudicotyledons	Cucurbitaceae	*Hemsleya*	*Hemsleya amabilis*	JN044800
Eudicotyledons	Cucurbitaceae	*Hemsleya*	*Hemsleya amabilis*	JN044801
Eudicotyledons	Cucurbitaceae	*Hemsleya*	*Hemsleya amabilis*	JN044802
Eudicotyledons	Cucurbitaceae	*Hemsleya*	*Hemsleya amabilis*	JN044803
Eudicotyledons	Cucurbitaceae	*Hemsleya*	*Hemsleya amabilis*	JN044804
Eudicotyledons	Cucurbitaceae	*Hemsleya*	*Hemsleya amabilis*	JN044805
Eudicotyledons	Cucurbitaceae	*Hemsleya*	*Hemsleya amabilis*	JN044806
Eudicotyledons	Cucurbitaceae	*Hemsleya*	*Hemsleya chinensis*	EF424069
Eudicotyledons	Cucurbitaceae	*Hemsleya*	*Hemsleya chinensis*	JN044816
Eudicotyledons	Cucurbitaceae	*Hemsleya*	*Hemsleya chinensis*	JN044817
Eudicotyledons	Cucurbitaceae	*Hemsleya*	*Hemsleya chinensis*	JN044818
Eudicotyledons	Cucurbitaceae	*Hemsleya*	*Hemsleya chinensis*	JN044819
Eudicotyledons	Cucurbitaceae	*Hemsleya*	*Hemsleya chinensis*	JN044820
Eudicotyledons	Cucurbitaceae	*Hemsleya*	*Hemsleya chinensis*	JN044821
Eudicotyledons	Cucurbitaceae	*Hemsleya*	*Hemsleya chinensis*	JN044822
Eudicotyledons	Cucurbitaceae	*Hemsleya*	*Hemsleya chinensis*	JN044823
Eudicotyledons	Cucurbitaceae	*Hemsleya*	*Hemsleya sp. HTL-2007*	EF424067
Eudicotyledons	Cucurbitaceae	*Hemsleya*	*Hemsleya sp. HTL-2007*	JN044842
Eudicotyledons	Cucurbitaceae	*Hemsleya*	*Hemsleya sp. HTL-2007*	JN044843

Eudicotyledons	Cucurbitaceae	*Hemsleya*	*Hemsleya sp. HTL-2007*	JN044844
Eudicotyledons	Fabaceae	*Aspalathus*	*Aspalathus costulata*	EU000772
Eudicotyledons	Fabaceae	*Aspalathus*	*Aspalathus costulata*	EU000773
Eudicotyledons	Fabaceae	*Aspalathus*	*Aspalathus crenata*	EU000774
Eudicotyledons	Fabaceae	*Aspalathus*	*Aspalathus crenata*	EU000775
Eudicotyledons	Malvaceae	*Alcea*	*Alcea glabrata*	GU166780
Eudicotyledons	Malvaceae	*Alcea*	*Alcea glabrata*	GU166781
Eudicotyledons	Asteraceae	*Sinosenecio*	*Sinosenecio chienii*	JN047131
Eudicotyledons	Asteraceae	*Sinosenecio*	*Sinosenecio chienii*	JN047132
Eudicotyledons	Asteraceae	*Sinosenecio*	*Sinosenecio denticulatus*	JN047136
Eudicotyledons	Asteraceae	*Sinosenecio*	*Sinosenecio denticulatus*	JN047137
Eudicotyledons	Asteraceae	*Sinosenecio*	*Sinosenecio denticulatus*	JN047138
Eudicotyledons	Asteraceae	*Sinosenecio*	*Sinosenecio denticulatus*	JN047139
Eudicotyledons	Asteraceae	*Sinosenecio*	*Sinosenecio euosmus*	GU818473
Eudicotyledons	Asteraceae	*Sinosenecio*	*Sinosenecio euosmus*	JN047143
Eudicotyledons	Asteraceae	*Sinosenecio*	*Sinosenecio euosmus*	JN047144
Eudicotyledons	Asteraceae	*Sinosenecio*	*Sinosenecio euosmus*	JN047145
Eudicotyledons	Asteraceae	*Sinosenecio*	*Sinosenecio euosmus*	JN047146
Eudicotyledons	Asteraceae	*Sinosenecio*	*Sinosenecio euosmus*	JN047147
Eudicotyledons	Asteraceae	*Sinosenecio*	*Sinosenecio euosmus*	JN047148
Eudicotyledons	Asteraceae	*Sinosenecio*	*Sinosenecio guangxiensis*	JN047156
Eudicotyledons	Asteraceae	*Sinosenecio*	*Sinosenecio guangxiensis*	JN047157
Eudicotyledons	Asteraceae	*Sinosenecio*	*Sinosenecio guangxiensis*	JN047158
Eudicotyledons	Asteraceae	*Sinosenecio*	*Sinosenecio homogyniphyllus*	JN047159
Eudicotyledons	Asteraceae	*Sinosenecio*	*Sinosenecio homogyniphyllus*	JN047160
Eudicotyledons	Asteraceae	*Sinosenecio*	*Sinosenecio homogyniphyllus*	JN047161
Eudicotyledons	Asteraceae	*Sinosenecio*	*Sinosenecio homogyniphyllus*	JN047162
Eudicotyledons	Asteraceae	*Sinosenecio*	*Sinosenecio homogyniphyllus*	JN047163
Eudicotyledons	Caryocaraceae	*Caryocar*	*Caryocar brasiliense*	EU350258
Eudicotyledons	Caryocaraceae	*Caryocar*	*Caryocar brasiliense*	EU350259
Eudicotyledons	Caryocaraceae	*Caryocar*	*Caryocar brasiliense*	EU350260
Eudicotyledons	Caryocaraceae	*Caryocar*	*Caryocar brasiliense*	EU350261
Eudicotyledons	Caryocaraceae	*Caryocar*	*Caryocar brasiliense*	EU350262
Eudicotyledons	Caryocaraceae	*Caryocar*	*Caryocar brasiliense*	EU350263
Eudicotyledons	Caryocaraceae	*Caryocar*	*Caryocar brasiliense*	EU350264
Eudicotyledons	Caryocaraceae	*Caryocar*	*Caryocar brasiliense*	EU350265
Eudicotyledons	Caryocaraceae	*Caryocar*	*Caryocar brasiliense*	EU350266
Eudicotyledons	Caryocaraceae	*Caryocar*	*Caryocar brasiliense*	EU350267
Eudicotyledons	Asteraceae	*Symphyotrichum*	*Symphyotrichum ericoides*	EU750581
Eudicotyledons	Asteraceae	*Symphyotrichum*	*Symphyotrichum ericoides*	EU750582
Eudicotyledons	Combretaceae	*Combretum*	*Combretum apiculatum*	EU213794
Eudicotyledons	Combretaceae	*Combretum*	*Combretum apiculatum*	EU213795
Eudicotyledons	Combretaceae	*Combretum*	*Combretum apiculatum*	EU213796
Eudicotyledons	Combretaceae	*Combretum*	*Combretum collinum*	EU213797
Eudicotyledons	Combretaceae	*Combretum*	*Combretum collinum*	EU213798
Eudicotyledons	Combretaceae	*Combretum*	*Combretum collinum*	EU213799
Eudicotyledons	Combretaceae	*Combretum*	*Combretum hereroense*	EU338193
Eudicotyledons	Combretaceae	*Combretum*	*Combretum hereroense*	EU213800
Eudicotyledons	Combretaceae	*Combretum*	*Combretum hereroense*	EU213801
Eudicotyledons	Combretaceae	*Combretum*	*Combretum hereroense*	EU213802
Eudicotyledons	Malvaceae	*Grewia*	*Grewia bicolor*	EU213828
Eudicotyledons	Malvaceae	*Grewia*	*Grewia bicolor*	EU213829
Eudicotyledons	Malvaceae	*Grewia*	*Grewia bicolor*	EU213830

Eudicotyledons	Malvaceae	*Grewia*	*Grewia flavescens*	EU213831
Eudicotyledons	Malvaceae	*Grewia*	*Grewia flavescens*	EU213832
Eudicotyledons	Malvaceae	*Grewia*	*Grewia villosa*	EU213833
Eudicotyledons	Malvaceae	*Grewia*	*Grewia villosa*	EU213834
Eudicotyledons	Malvaceae	*Grewia*	*Grewia villosa*	EU213835
Eudicotyledons	Rubiaceae	*Razafimandimbisonia*	*Razafimandimbisonia sambiranensis*	AM939394
Eudicotyledons	Rubiaceae	*Razafimandimbisonia*	*Razafimandimbisonia sambiranensis*	AM939395
Eudicotyledons	Combretaceae	*Combretum*	*Combretum imberbe*	EU338208
Eudicotyledons	Combretaceae	*Combretum*	*Combretum imberbe*	EU338209
Eudicotyledons	Combretaceae	*Combretum*	*Combretum molle*	EU338200
Eudicotyledons	Combretaceae	*Combretum*	*Combretum molle*	EU338201
Eudicotyledons	Combretaceae	*Combretum*	*Combretum platypetalum*	EU338179
Eudicotyledons	Combretaceae	*Combretum*	*Combretum platypetalum*	EU338180
Eudicotyledons	Caryocaraceae	*Caryocar*	*Caryocar microcarpum*	EU350268
Eudicotyledons	Caryocaraceae	*Caryocar*	*Caryocar microcarpum*	EU350269
Eudicotyledons	Caryocaraceae	*Caryocar*	*Caryocar microcarpum*	EU350270
Eudicotyledons	Caryocaraceae	*Caryocar*	*Caryocar microcarpum*	EU350271
Eudicotyledons	Caryocaraceae	*Caryocar*	*Caryocar microcarpum*	EU350272
Eudicotyledons	Caryocaraceae	*Caryocar*	*Caryocar microcarpum*	EU350273
Eudicotyledons	Caryocaraceae	*Caryocar*	*Caryocar microcarpum*	EU350274
Eudicotyledons	Caryocaraceae	*Caryocar*	*Caryocar microcarpum*	EU350275
Eudicotyledons	Caryocaraceae	*Caryocar*	*Caryocar microcarpum*	EU350276
Eudicotyledons	Caryocaraceae	*Caryocar*	*Caryocar microcarpum*	EU350277
Eudicotyledons	Caryocaraceae	*Caryocar*	*Caryocar microcarpum*	EU350278
Eudicotyledons	Caryocaraceae	*Caryocar*	*Caryocar microcarpum*	EU350279
Eudicotyledons	Caryocaraceae	*Caryocar*	*Caryocar microcarpum*	EU350280
Eudicotyledons	Caryocaraceae	*Caryocar*	*Caryocar microcarpum*	EU350281
Eudicotyledons	Caryocaraceae	*Caryocar*	*Caryocar microcarpum*	EU350282
Eudicotyledons	Caryocaraceae	*Caryocar*	*Caryocar microcarpum*	EU350283
Eudicotyledons	Caryocaraceae	*Caryocar*	*Caryocar microcarpum*	EU350284
Eudicotyledons	Caryocaraceae	*Caryocar*	*Caryocar microcarpum*	EU350285
Eudicotyledons	Caryocaraceae	*Caryocar*	*Caryocar microcarpum*	EU350286
Eudicotyledons	Caryocaraceae	*Caryocar*	*Caryocar microcarpum*	EU350287
Eudicotyledons	Caryocaraceae	*Caryocar*	*Caryocar microcarpum*	EU350288
Eudicotyledons	Caryocaraceae	*Caryocar*	*Caryocar microcarpum*	EU350289
Eudicotyledons	Caryocaraceae	*Caryocar*	*Caryocar microcarpum*	EU350290
Eudicotyledons	Caryocaraceae	*Caryocar*	*Caryocar microcarpum*	EU350291
Eudicotyledons	Caryocaraceae	*Caryocar*	*Caryocar microcarpum*	EU350292
Eudicotyledons	Caryocaraceae	*Caryocar*	*Caryocar microcarpum*	EU350293
Eudicotyledons	Caryocaraceae	*Caryocar*	*Caryocar microcarpum*	EU350294
Eudicotyledons	Caryocaraceae	*Caryocar*	*Caryocar microcarpum*	EU350295
Eudicotyledons	Caryocaraceae	*Caryocar*	*Caryocar microcarpum*	EU350296
Eudicotyledons	Caryocaraceae	*Caryocar*	*Caryocar microcarpum*	EU350297
Eudicotyledons	Caryocaraceae	*Caryocar*	*Caryocar microcarpum*	EU350298
Eudicotyledons	Caryocaraceae	*Caryocar*	*Caryocar microcarpum*	EU350299
Eudicotyledons	Caryocaraceae	*Caryocar*	*Caryocar microcarpum*	EU350300
Eudicotyledons	Caryocaraceae	*Caryocar*	*Caryocar microcarpum*	EU350301
Eudicotyledons	Caryocaraceae	*Caryocar*	*Caryocar microcarpum*	EU350302
Eudicotyledons	Caryocaraceae	*Caryocar*	*Caryocar microcarpum*	EU350303
Eudicotyledons	Caryocaraceae	*Caryocar*	*Caryocar microcarpum*	EU350304
Eudicotyledons	Caryocaraceae	*Caryocar*	*Caryocar microcarpum*	EU350305
Eudicotyledons	Caryocaraceae	*Caryocar*	*Caryocar microcarpum*	EU350306
Eudicotyledons	Caryocaraceae	*Caryocar*	*Caryocar microcarpum*	EU350307

Eudicotyledons	Caryocaraceae	*Caryocar*	*Caryocar microcarpum*	EU350308
Eudicotyledons	Caryocaraceae	*Caryocar*	*Caryocar villosum*	EU350309
Eudicotyledons	Caryocaraceae	*Caryocar*	*Caryocar villosum*	EU350310
Eudicotyledons	Caryocaraceae	*Caryocar*	*Caryocar villosum*	EU350311
Eudicotyledons	Caryocaraceae	*Caryocar*	*Caryocar villosum*	EU350312
Eudicotyledons	Caryocaraceae	*Caryocar*	*Caryocar villosum*	EU350313
Eudicotyledons	Caryocaraceae	*Caryocar*	*Caryocar villosum*	EU350314
Eudicotyledons	Caryocaraceae	*Caryocar*	*Caryocar villosum*	EU350315
Eudicotyledons	Caryocaraceae	*Caryocar*	*Caryocar villosum*	EU350316
Eudicotyledons	Caryocaraceae	*Caryocar*	*Caryocar villosum*	EU350317
Eudicotyledons	Caryocaraceae	*Caryocar*	*Caryocar villosum*	EU350318
Eudicotyledons	Caryocaraceae	*Caryocar*	*Caryocar villosum*	EU350319
Eudicotyledons	Caryocaraceae	*Caryocar*	*Caryocar villosum*	EU350320
Eudicotyledons	Caryocaraceae	*Caryocar*	*Caryocar villosum*	EU350321
Eudicotyledons	Caryocaraceae	*Caryocar*	*Caryocar villosum*	EU350322
Eudicotyledons	Caryocaraceae	*Caryocar*	*Caryocar villosum*	EU350323
Eudicotyledons	Caryocaraceae	*Caryocar*	*Caryocar villosum*	EU350324
Eudicotyledons	Caryocaraceae	*Caryocar*	*Caryocar villosum*	EU350325
Eudicotyledons	Caryocaraceae	*Caryocar*	*Caryocar villosum*	EU350326
Eudicotyledons	Caryocaraceae	*Caryocar*	*Caryocar villosum*	EU350327
Eudicotyledons	Caryocaraceae	*Caryocar*	*Caryocar villosum*	EU350328
Eudicotyledons	Caryocaraceae	*Caryocar*	*Caryocar villosum*	EU350329
Eudicotyledons	Caryocaraceae	*Caryocar*	*Caryocar villosum*	EU350330
Eudicotyledons	Caryocaraceae	*Caryocar*	*Caryocar villosum*	EU350331
Eudicotyledons	Caryocaraceae	*Caryocar*	*Caryocar villosum*	EU350332
Eudicotyledons	Caryocaraceae	*Caryocar*	*Caryocar villosum*	EU350333
Eudicotyledons	Caryocaraceae	*Caryocar*	*Caryocar villosum*	EU350334
Eudicotyledons	Caryocaraceae	*Caryocar*	*Caryocar villosum*	EU350335
Eudicotyledons	Caryocaraceae	*Caryocar*	*Caryocar villosum*	EU350336
Eudicotyledons	Caryocaraceae	*Caryocar*	*Caryocar villosum*	EU350337
Eudicotyledons	Caryocaraceae	*Caryocar*	*Caryocar villosum*	EU350338
Eudicotyledons	Caryocaraceae	*Caryocar*	*Caryocar villosum*	EU350339
Eudicotyledons	Caryocaraceae	*Caryocar*	*Caryocar villosum*	EU350340
Eudicotyledons	Caryocaraceae	*Caryocar*	*Caryocar villosum*	EU350341
Eudicotyledons	Caryocaraceae	*Caryocar*	*Caryocar villosum*	EU350342
Eudicotyledons	Caryocaraceae	*Caryocar*	*Caryocar villosum*	EU350343
Eudicotyledons	Caryocaraceae	*Caryocar*	*Caryocar villosum*	EU350344
Eudicotyledons	Caryocaraceae	*Caryocar*	*Caryocar villosum*	EU350345
Eudicotyledons	Caryocaraceae	*Caryocar*	*Caryocar villosum*	EU350346
Eudicotyledons	Caryocaraceae	*Caryocar*	*Caryocar villosum*	EU350347
Eudicotyledons	Caryocaraceae	*Caryocar*	*Caryocar villosum*	EU350348
Eudicotyledons	Caryocaraceae	*Caryocar*	*Caryocar villosum*	EU350349
Eudicotyledons	Caryocaraceae	*Caryocar*	*Caryocar villosum*	EU350350
Eudicotyledons	Caryocaraceae	*Caryocar*	*Caryocar villosum*	EU350351
Eudicotyledons	Caryocaraceae	*Caryocar*	*Caryocar villosum*	EU350352
Eudicotyledons	Caryocaraceae	*Caryocar*	*Caryocar villosum*	EU350353
Eudicotyledons	Caryocaraceae	*Caryocar*	*Caryocar villosum*	EU350354
Eudicotyledons	Caryocaraceae	*Caryocar*	*Caryocar villosum*	EU350355
Eudicotyledons	Caryocaraceae	*Caryocar*	*Caryocar villosum*	EU350356
Eudicotyledons	Caryocaraceae	*Caryocar*	*Caryocar villosum*	EU350357
Eudicotyledons	Caryocaraceae	*Caryocar*	*Caryocar villosum*	EU350358
Eudicotyledons	Asteraceae	*Symphyotrichum*	*Symphyotrichum ciliolatum*	EU750578
Eudicotyledons	Asteraceae	*Symphyotrichum*	*Symphyotrichum ciliolatum*	EU750579

Eudicotyledons	Asteraceae	*Symphyotrichum*	*Symphyotrichum ciliolatum*	EU750580
Eudicotyledons	Asteraceae	*Symphyotrichum*	*Symphyotrichum lanceolatum*	EU750583
Eudicotyledons	Asteraceae	*Symphyotrichum*	*Symphyotrichum lanceolatum*	EU750584
Eudicotyledons	Asteraceae	*Symphyotrichum*	*Symphyotrichum lateriflorum*	EU750585
Eudicotyledons	Asteraceae	*Symphyotrichum*	*Symphyotrichum lateriflorum*	EU750586
Eudicotyledons	Asteraceae	*Symphyotrichum*	*Symphyotrichum lateriflorum*	EU750587
Eudicotyledons	Asteraceae	*Symphyotrichum*	*Symphyotrichum lateriflorum*	EU750588
Eudicotyledons	Asteraceae	*Symphyotrichum*	*Symphyotrichum pilosum*	EU750593
Eudicotyledons	Asteraceae	*Symphyotrichum*	*Symphyotrichum pilosum*	EU750594
Eudicotyledons	Asteraceae	*Symphyotrichum*	*Symphyotrichum urophyllum*	EU750595
Eudicotyledons	Asteraceae	*Symphyotrichum*	*Symphyotrichum urophyllum*	EU750596
Eudicotyledons	Asteraceae	*Symphyotrichum*	*Symphyotrichum puniceum*	HQ596858
Eudicotyledons	Asteraceae	*Symphyotrichum*	*Symphyotrichum puniceum*	HQ596859
Eudicotyledons	Asteraceae	*Anthemis*	*Anthemis cupaniana*	FR689891
Eudicotyledons	Asteraceae	*Anthemis*	*Anthemis cupaniana*	FR689901
Eudicotyledons	Asteraceae	*Anthemis*	*Anthemis yemensis*	FR689841
Eudicotyledons	Asteraceae	*Anthemis*	*Anthemis yemensis*	FR689892
Eudicotyledons	Apocynaceae	*Aspidosperma*	*Aspidosperma marcgravianum*	FJ038857
Eudicotyledons	Apocynaceae	*Aspidosperma*	*Aspidosperma marcgravianum*	FJ038858
Eudicotyledons	Boraginaceae	*Cordia*	*Cordia sagotii*	FJ038862
Eudicotyledons	Boraginaceae	*Cordia*	*Cordia sagotii*	FJ038863
Eudicotyledons	Boraginaceae	*Cordia*	*Cordia sagotii*	FJ038864
Eudicotyledons	Chrysobalanaceae	*Couepia*	*Couepia bracteosa*	FJ038891
Eudicotyledons	Chrysobalanaceae	*Couepia*	*Couepia bracteosa*	FJ038892
Eudicotyledons	Chrysobalanaceae	*Couepia*	*Couepia guianensis*	FJ038889
Eudicotyledons	Chrysobalanaceae	*Couepia*	*Couepia guianensis*	FJ038890
Eudicotyledons	Sapotaceae	*Pouteria*	*Pouteria decorticans*	FJ039069
Eudicotyledons	Sapotaceae	*Pouteria*	*Pouteria decorticans*	FJ039070
Eudicotyledons	Sapotaceae	*Pouteria*	*Pouteria decorticans*	FJ039071
Eudicotyledons	Sapotaceae	*Pouteria*	*Pouteria decorticans*	FJ039072
Eudicotyledons	Sapotaceae	*Pouteria*	*Pouteria filipes*	GQ428714
Eudicotyledons	Sapotaceae	*Pouteria*	*Pouteria filipes*	FJ039076
Eudicotyledons	Sapotaceae	*Pouteria*	*Pouteria gongrijpii*	FJ039073
Eudicotyledons	Sapotaceae	*Pouteria*	*Pouteria gongrijpii*	FJ039074
Eudicotyledons	Sapotaceae	*Pouteria*	*Pouteria sp. MAG-2009*	FJ039077
Eudicotyledons	Sapotaceae	*Pouteria*	*Pouteria sp. MAG-2009*	FJ039078
Eudicotyledons	Sapotaceae	*Pouteria*	*Pouteria sp. MAG-2009*	FJ039080
Eudicotyledons	Thymelaeaceae	*Daphne*	*Daphne blagayana*	GQ167433
Eudicotyledons	Thymelaeaceae	*Daphne*	*Daphne blagayana*	GQ167434
Eudicotyledons	Thymelaeaceae	*Daphne*	*Daphne rodriguezii*	GQ167489
Eudicotyledons	Thymelaeaceae	*Daphne*	*Daphne rodriguezii*	GQ167490
Eudicotyledons	Asteraceae	*Pityopsis*	*Pityopsis aspera*	GQ892627
Eudicotyledons	Asteraceae	*Pityopsis*	*Pityopsis aspera*	GQ892628
Eudicotyledons	Asteraceae	*Pityopsis*	*Pityopsis falcata*	GQ892616
Eudicotyledons	Asteraceae	*Pityopsis*	*Pityopsis falcata*	GQ892617
Eudicotyledons	Asteraceae	*Pityopsis*	*Pityopsis falcata*	GQ892618
Eudicotyledons	Asteraceae	*Pityopsis*	*Pityopsis oligantha*	GQ892625
Eudicotyledons	Asteraceae	*Pityopsis*	*Pityopsis oligantha*	GQ892626
Eudicotyledons	Asteraceae	*Pityopsis*	*Pityopsis pinifolia*	GQ892619
Eudicotyledons	Asteraceae	*Pityopsis*	*Pityopsis pinifolia*	GQ892620
Eudicotyledons	Asteraceae	*Pityopsis*	*Pityopsis pinifolia*	GQ892621
Eudicotyledons	Asteraceae	*Pityopsis*	*Pityopsis ruthii*	GQ892622
Eudicotyledons	Asteraceae	*Pityopsis*	*Pityopsis ruthii*	GQ892623

Eudicotyledons	Apocynaceae	*Aspidosperma*	*Aspidosperma spruceanum*	GQ428748
Eudicotyledons	Apocynaceae	*Aspidosperma*	*Aspidosperma spruceanum*	GQ982153
Eudicotyledons	Asteraceae	*Chaetoseris*	*Chaetoseris cyanea*	GU109326
Eudicotyledons	Asteraceae	*Chaetoseris*	*Chaetoseris cyanea*	GU109329
Eudicotyledons	Malvaceae	*Alcea*	*Alcea acaulis*	GU166773
Eudicotyledons	Malvaceae	*Alcea*	*Alcea acaulis*	GU166774
Eudicotyledons	Malvaceae	*Alcea*	*Alcea apterocarpa*	GU166777
Eudicotyledons	Malvaceae	*Alcea*	*Alcea apterocarpa*	GU166778
Eudicotyledons	Malvaceae	*Alcea*	*Alcea hohenackeri*	GU166783
Eudicotyledons	Malvaceae	*Alcea*	*Alcea hohenackeri*	GU166784
Eudicotyledons	Malvaceae	*Alcea*	*Alcea lavateriflora*	GU166785
Eudicotyledons	Malvaceae	*Alcea*	*Alcea lavateriflora*	GU166786
Eudicotyledons	Malvaceae	*Alcea*	*Alcea rufescens*	GU166789
Eudicotyledons	Malvaceae	*Alcea*	*Alcea rufescens*	GU166790
Eudicotyledons	Orobanchaceae	*Pterygiella*	*Pterygiella cylindrica*	JN416386
Eudicotyledons	Orobanchaceae	*Pterygiella*	*Pterygiella cylindrica*	JN416387
Eudicotyledons	Orobanchaceae	*Pterygiella*	*Pterygiella cylindrica*	JN416388
Eudicotyledons	Orobanchaceae	*Pterygiella*	*Pterygiella cylindrica*	JN046672
Eudicotyledons	Orobanchaceae	*Pterygiella*	*Pterygiella cylindrica*	JN046673
Eudicotyledons	Orobanchaceae	*Pterygiella*	*Pterygiella cylindrica*	JN046674
Eudicotyledons	Orobanchaceae	*Pterygiella*	*Pterygiella duclouxii*	JN416389
Eudicotyledons	Orobanchaceae	*Pterygiella*	*Pterygiella duclouxii*	JN416390
Eudicotyledons	Orobanchaceae	*Pterygiella*	*Pterygiella duclouxii*	JN416391
Eudicotyledons	Orobanchaceae	*Pterygiella*	*Pterygiella duclouxii*	JN416392
Eudicotyledons	Orobanchaceae	*Pterygiella*	*Pterygiella duclouxii*	JN416393
Eudicotyledons	Orobanchaceae	*Pterygiella*	*Pterygiella duclouxii*	JN416394
Eudicotyledons	Orobanchaceae	*Pterygiella*	*Pterygiella duclouxii*	JN416395
Eudicotyledons	Orobanchaceae	*Pterygiella*	*Pterygiella duclouxii*	JN416396
Eudicotyledons	Orobanchaceae	*Pterygiella*	*Pterygiella duclouxii*	JN416397
Eudicotyledons	Orobanchaceae	*Pterygiella*	*Pterygiella duclouxii*	JN416398
Eudicotyledons	Orobanchaceae	*Pterygiella*	*Pterygiella duclouxii*	JN416399
Eudicotyledons	Orobanchaceae	*Pterygiella*	*Pterygiella duclouxii*	JN416400
Eudicotyledons	Orobanchaceae	*Pterygiella*	*Pterygiella duclouxii*	JN416401
Eudicotyledons	Orobanchaceae	*Pterygiella*	*Pterygiella duclouxii*	JN416402
Eudicotyledons	Orobanchaceae	*Pterygiella*	*Pterygiella duclouxii*	JN416403
Eudicotyledons	Orobanchaceae	*Pterygiella*	*Pterygiella duclouxii*	JN416404
Eudicotyledons	Orobanchaceae	*Pterygiella*	*Pterygiella duclouxii*	JN416405
Eudicotyledons	Orobanchaceae	*Pterygiella*	*Pterygiella duclouxii*	JN416406
Eudicotyledons	Orobanchaceae	*Pterygiella*	*Pterygiella duclouxii*	JN416407
Eudicotyledons	Orobanchaceae	*Pterygiella*	*Pterygiella duclouxii*	JN416408
Eudicotyledons	Orobanchaceae	*Pterygiella*	*Pterygiella duclouxii*	JN416409
Eudicotyledons	Orobanchaceae	*Pterygiella*	*Pterygiella duclouxii*	JN046675
Eudicotyledons	Orobanchaceae	*Pterygiella*	*Pterygiella duclouxii*	JN046676
Eudicotyledons	Orobanchaceae	*Pterygiella*	*Pterygiella duclouxii*	JN046677
Eudicotyledons	Orobanchaceae	*Pterygiella*	*Pterygiella duclouxii*	JN046678
Eudicotyledons	Orobanchaceae	*Pterygiella*	*Pterygiella duclouxii*	JN046679
Eudicotyledons	Orobanchaceae	*Pterygiella*	*Pterygiella duclouxii*	JN046680
Eudicotyledons	Orobanchaceae	*Pterygiella*	*Pterygiella nigrescens*	JN416410
Eudicotyledons	Orobanchaceae	*Pterygiella*	*Pterygiella nigrescens*	JN416411
Eudicotyledons	Orobanchaceae	*Pterygiella*	*Pterygiella nigrescens*	JN046681
Eudicotyledons	Orobanchaceae	*Pterygiella*	*Pterygiella nigrescens*	JN046682
Eudicotyledons	Orobanchaceae	*Pterygiella*	*Pterygiella nigrescens*	JN046683
Eudicotyledons	Orobanchaceae	*Pterygiella*	*Pterygiella suffruticosa*	JN416412

Eudicotyledons	Orobanchaceae	*Pterygiella*	*Pterygiella suffruticosa*	JN416413
Eudicotyledons	Orobanchaceae	*Pterygiella*	*Pterygiella suffruticosa*	JN416414
Eudicotyledons	Orobanchaceae	*Pterygiella*	*Pterygiella suffruticosa*	JN416415
Eudicotyledons	Orobanchaceae	*Pterygiella*	*Pterygiella suffruticosa*	JN046684
Eudicotyledons	Orobanchaceae	*Pterygiella*	*Pterygiella suffruticosa*	JN046685
Eudicotyledons	Orobanchaceae	*Pterygiella*	*Pterygiella suffruticosa*	JN046686
Eudicotyledons	Orobanchaceae	*Pterygiella*	*Pterygiella suffruticosa*	JN046687
Eudicotyledons	Orobanchaceae	*Pterygiella*	*Pterygiella suffruticosa*	JN046688
Eudicotyledons	Asteraceae	*Nabalus*	*Nabalus alatus*	HQ162026
Eudicotyledons	Asteraceae	*Nabalus*	*Nabalus alatus*	HQ162027
Eudicotyledons	Asteraceae	*Nabalus*	*Nabalus asper*	HQ162020
Eudicotyledons	Asteraceae	*Nabalus*	*Nabalus asper*	HQ162021
Eudicotyledons	Asteraceae	*Nabalus*	*Nabalus asper*	HQ162025
Eudicotyledons	Asteraceae	*Nabalus*	*Nabalus barbatus*	HQ162011
Eudicotyledons	Asteraceae	*Nabalus*	*Nabalus barbatus*	HQ162023
Eudicotyledons	Asteraceae	*Nabalus*	*Nabalus barbatus*	HQ162024
Eudicotyledons	Asteraceae	*Nabalus*	*Nabalus racemosus*	HQ162022
Eudicotyledons	Asteraceae	*Nabalus*	*Nabalus racemosus*	HQ162030
Eudicotyledons	Asteraceae	*Nabalus*	*Nabalus roanensis*	HQ162015
Eudicotyledons	Asteraceae	*Nabalus*	*Nabalus roanensis*	HQ162016
Eudicotyledons	Cactaceae	*Pilosocereus*	*Pilosocereus machrisii*	JN035466
Eudicotyledons	Cactaceae	*Pilosocereus*	*Pilosocereus machrisii*	JN035467
Eudicotyledons	Cactaceae	*Pilosocereus*	*Pilosocereus machrisii*	JN035468
Eudicotyledons	Cactaceae	*Pilosocereus*	*Pilosocereus machrisii*	JN035469
Eudicotyledons	Cactaceae	*Pilosocereus*	*Pilosocereus machrisii*	JN035470
Eudicotyledons	Cactaceae	*Pilosocereus*	*Pilosocereus machrisii*	JN035471
Eudicotyledons	Cactaceae	*Pilosocereus*	*Pilosocereus machrisii*	JN035472
Eudicotyledons	Cactaceae	*Pilosocereus*	*Pilosocereus machrisii*	JN035473
Eudicotyledons	Cactaceae	*Pilosocereus*	*Pilosocereus machrisii*	JN035474
Eudicotyledons	Cactaceae	*Pilosocereus*	*Pilosocereus machrisii*	JN035475
Eudicotyledons	Cactaceae	*Pilosocereus*	*Pilosocereus machrisii*	JN035476
Eudicotyledons	Cactaceae	*Pilosocereus*	*Pilosocereus machrisii*	JN035477
Eudicotyledons	Cactaceae	*Pilosocereus*	*Pilosocereus machrisii*	JN035478
Eudicotyledons	Cactaceae	*Pilosocereus*	*Pilosocereus machrisii*	JN035479
Eudicotyledons	Cactaceae	*Pilosocereus*	*Pilosocereus machrisii*	JN035480
Eudicotyledons	Cactaceae	*Pilosocereus*	*Pilosocereus machrisii*	JN035491
Eudicotyledons	Cactaceae	*Pilosocereus*	*Pilosocereus machrisii*	JN035492
Eudicotyledons	Cactaceae	*Pilosocereus*	*Pilosocereus machrisii*	JN035493
Eudicotyledons	Cactaceae	*Pilosocereus*	*Pilosocereus machrisii*	JN035494
Eudicotyledons	Cactaceae	*Pilosocereus*	*Pilosocereus machrisii*	JN035495
Eudicotyledons	Cactaceae	*Pilosocereus*	*Pilosocereus machrisii*	JN035496
Eudicotyledons	Cactaceae	*Pilosocereus*	*Pilosocereus machrisii*	JN035497
Eudicotyledons	Cactaceae	*Pilosocereus*	*Pilosocereus machrisii*	JN035498
Eudicotyledons	Cactaceae	*Pilosocereus*	*Pilosocereus machrisii*	JN035499
Eudicotyledons	Cactaceae	*Pilosocereus*	*Pilosocereus machrisii*	JN035500
Eudicotyledons	Cactaceae	*Pilosocereus*	*Pilosocereus machrisii*	JN035501
Eudicotyledons	Cactaceae	*Pilosocereus*	*Pilosocereus machrisii*	JN035502
Eudicotyledons	Cactaceae	*Pilosocereus*	*Pilosocereus machrisii*	JN035503
Eudicotyledons	Rutaceae	*Spathelia*	*Spathelia sorbifolia*	FR747986
Eudicotyledons	Rutaceae	*Spathelia*	*Spathelia sorbifolia*	FR747989
Eudicotyledons	Rutaceae	*Spathelia*	*Spathelia splendens*	FR747991
Eudicotyledons	Rutaceae	*Spathelia*	*Spathelia splendens*	FR747995
Eudicotyledons	Rutaceae	*Spathelia*	*Spathelia splendens*	FR747998

Eudicotyledons	Rutaceae	*Spathelia*	*Spathelia wrightii*	FR747992
Eudicotyledons	Rutaceae	*Spathelia*	*Spathelia wrightii*	FR747999
Eudicotyledons	Rutaceae	*Spathelia*	*Spathelia wrightii*	FR748000
Eudicotyledons	Rutaceae	*Spathelia*	*Spathelia vernicosa*	FR747996
Eudicotyledons	Rutaceae	*Spathelia*	*Spathelia vernicosa*	FR747997
Eudicotyledons	Rutaceae	*Spathelia*	*Spathelia vernicosa*	FR748001
Eudicotyledons	Malvaceae	*Malva*	*Malva excisa*	HM214506
Eudicotyledons	Malvaceae	*Malva*	*Malva excisa*	HM214507
Eudicotyledons	Malvaceae	*Malva*	*Malva excisa*	HM214508
Eudicotyledons	Malvaceae	*Malva*	*Malva excisa*	HM214509
Eudicotyledons	Malvaceae	*Malva*	*Malva excisa*	HM214510
Eudicotyledons	Malvaceae	*Malva*	*Malva excisa*	HM214511
Eudicotyledons	Malvaceae	*Malva*	*Malva excisa*	HM214512
Eudicotyledons	Asteraceae	*Chaetoseris*	*Chaetoseris grandiflora*	HQ436157
Eudicotyledons	Asteraceae	*Chaetoseris*	*Chaetoseris grandiflora*	HQ436158
Eudicotyledons	Asteraceae	*Syncalathium*	*Syncalathium chrysocephalum*	HQ436166
Eudicotyledons	Asteraceae	*Syncalathium*	*Syncalathium chrysocephalum*	JN047303
Eudicotyledons	Asteraceae	*Syncalathium*	*Syncalathium chrysocephalum*	JN047304
Eudicotyledons	Asteraceae	*Syncalathium*	*Syncalathium chrysocephalum*	JN047305
Eudicotyledons	Asteraceae	*Syncalathium*	*Syncalathium disciforme*	HQ436167
Eudicotyledons	Asteraceae	*Syncalathium*	*Syncalathium disciforme*	JN047306
Eudicotyledons	Asteraceae	*Syncalathium*	*Syncalathium disciforme*	JN047307
Eudicotyledons	Asteraceae	*Syncalathium*	*Syncalathium disciforme*	JN047308
Eudicotyledons	Asteraceae	*Syncalathium*	*Syncalathium kawaguchii*	HQ436168
Eudicotyledons	Asteraceae	*Syncalathium*	*Syncalathium kawaguchii*	JN047309
Eudicotyledons	Asteraceae	*Syncalathium*	*Syncalathium kawaguchii*	JN047310
Eudicotyledons	Asteraceae	*Syncalathium*	*Syncalathium kawaguchii*	JN047311
Eudicotyledons	Asteraceae	*Syncalathium*	*Syncalathium pilosum*	HQ436169
Eudicotyledons	Asteraceae	*Syncalathium*	*Syncalathium pilosum*	JN047312
Eudicotyledons	Asteraceae	*Syncalathium*	*Syncalathium qinghaiense*	HQ436170
Eudicotyledons	Asteraceae	*Syncalathium*	*Syncalathium qinghaiense*	HQ436171
Eudicotyledons	Asteraceae	*Syncalathium*	*Syncalathium roseum*	HQ436172
Eudicotyledons	Asteraceae	*Syncalathium*	*Syncalathium roseum*	JN047313
Eudicotyledons	Asteraceae	*Syncalathium*	*Syncalathium souliei*	HQ436173
Eudicotyledons	Asteraceae	*Syncalathium*	*Syncalathium souliei*	HQ436174
Eudicotyledons	Asteraceae	*Syncalathium*	*Syncalathium souliei*	HQ436175
Eudicotyledons	Asteraceae	*Syncalathium*	*Syncalathium souliei*	HQ436176
Eudicotyledons	Asteraceae	*Syncalathium*	*Syncalathium souliei*	HQ436177
Eudicotyledons	Asteraceae	*Syncalathium*	*Syncalathium souliei*	HQ436178
Eudicotyledons	Asteraceae	*Nabalus*	*Nabalus tatarinowii*	HQ436162
Eudicotyledons	Asteraceae	*Nabalus*	*Nabalus tatarinowii*	HQ436163
Eudicotyledons	Loranthaceae	*Helixanthera*	*Helixanthera sampsonii*	HQ317806
Eudicotyledons	Loranthaceae	*Helixanthera*	*Helixanthera sampsonii*	HQ317807
Eudicotyledons	Asteraceae	*Sinosenecio*	*Sinosenecio sp. DZL-2011*	JN047126
Eudicotyledons	Asteraceae	*Sinosenecio*	*Sinosenecio sp. DZL-2011*	JN047127
Eudicotyledons	Asteraceae	*Sinosenecio*	*Sinosenecio sp. DZL-2011*	JN047128
Eudicotyledons	Asteraceae	*Sinosenecio*	*Sinosenecio cyclaminifolius*	JN047133
Eudicotyledons	Asteraceae	*Sinosenecio*	*Sinosenecio cyclaminifolius*	JN047134
Eudicotyledons	Asteraceae	*Sinosenecio*	*Sinosenecio cyclaminifolius*	JN047135
Eudicotyledons	Asteraceae	*Sinosenecio*	*Sinosenecio dryas*	JN047140
Eudicotyledons	Asteraceae	*Sinosenecio*	*Sinosenecio dryas*	JN047141
Eudicotyledons	Asteraceae	*Sinosenecio*	*Sinosenecio dryas*	JN047142
Eudicotyledons	Asteraceae	*Sinosenecio*	*Sinosenecio jiuhuashanicus*	JN047164

Eudicotyledons	Asteraceae	*Sinosenecio*	*Sinosenecio jiuhuashanicus*	JN047165
Eudicotyledons	Asteraceae	*Sinosenecio*	*Sinosenecio jiuhuashanicus*	JN047166
Eudicotyledons	Asteraceae	*Sinosenecio*	*Sinosenecio latouchei*	JN047170
Eudicotyledons	Asteraceae	*Sinosenecio*	*Sinosenecio latouchei*	JN047171
Eudicotyledons	Asteraceae	*Sinosenecio*	*Sinosenecio latouchei*	JN047172
Eudicotyledons	Asteraceae	*Sinosenecio*	*Sinosenecio latouchei*	JN047173
Eudicotyledons	Asteraceae	*Sinosenecio*	*Sinosenecio ligularioides*	JN047174
Eudicotyledons	Asteraceae	*Sinosenecio*	*Sinosenecio ligularioides*	JN047175
Eudicotyledons	Asteraceae	*Sinosenecio*	*Sinosenecio ligularioides*	JN047176
Eudicotyledons	Asteraceae	*Sinosenecio*	*Sinosenecio palmatisectus*	JN047180
Eudicotyledons	Asteraceae	*Sinosenecio*	*Sinosenecio palmatisectus*	JN047181
Eudicotyledons	Asteraceae	*Sinosenecio*	*Sinosenecio sichuanicus*	JN047188
Eudicotyledons	Asteraceae	*Sinosenecio*	*Sinosenecio sichuanicus*	JN047189
Eudicotyledons	Asteraceae	*Sinosenecio*	*Sinosenecio subrosulatus*	JN047194
Eudicotyledons	Asteraceae	*Sinosenecio*	*Sinosenecio subrosulatus*	JN047195
Eudicotyledons	Asteraceae	*Sinosenecio*	*Sinosenecio sungpanensis*	JN047196
Eudicotyledons	Asteraceae	*Sinosenecio*	*Sinosenecio sungpanensis*	JN047197
Eudicotyledons	Asteraceae	*Sinosenecio*	*Sinosenecio villifer*	JN047198
Eudicotyledons	Asteraceae	*Sinosenecio*	*Sinosenecio villifer*	JN047199
Eudicotyledons	Asteraceae	*Sinosenecio*	*Sinosenecio villifer*	JN047200
Eudicotyledons	Asteraceae	*Sinosenecio*	*Sinosenecio yilingii*	JN047201
Eudicotyledons	Asteraceae	*Sinosenecio*	*Sinosenecio yilingii*	JN047202
Eudicotyledons	Asteraceae	*Sinosenecio*	*Sinosenecio yilingii*	JN047203
Eudicotyledons	Zygophyllaceae	*Zygophyllum*	*Zygophyllum brachypterum*	JN047531
Eudicotyledons	Zygophyllaceae	*Zygophyllum*	*Zygophyllum brachypterum*	JN047532
Eudicotyledons	Zygophyllaceae	*Zygophyllum*	*Zygophyllum macropodum*	JN047536
Eudicotyledons	Zygophyllaceae	*Zygophyllum*	*Zygophyllum macropodum*	JN047537
Eudicotyledons	Zygophyllaceae	*Zygophyllum*	*Zygophyllum obliquum*	JN047538
Eudicotyledons	Zygophyllaceae	*Zygophyllum*	*Zygophyllum obliquum*	JN047539
Eudicotyledons	Betulaceae	*Ostryopsis*	*Ostryopsis intermedia*	JN045680
Eudicotyledons	Betulaceae	*Ostryopsis*	*Ostryopsis intermedia*	JN045681
Eudicotyledons	Betulaceae	*Ostryopsis*	*Ostryopsis intermedia*	JN045682
Eudicotyledons	Betulaceae	*Ostryopsis*	*Ostryopsis intermedia*	JN045683
Eudicotyledons	Betulaceae	*Ostryopsis*	*Ostryopsis intermedia*	JN045684
Eudicotyledons	Asteraceae	*Sinosenecio*	*Sinosenecio palmatilobus*	JN047182
Eudicotyledons	Asteraceae	*Sinosenecio*	*Sinosenecio palmatilobus*	JN047183
Eudicotyledons	Asteraceae	*Sinosenecio*	*Sinosenecio palmatilobus*	JN047184
Eudicotyledons	Cactaceae	*Pilosocereus*	*Pilosocereus aureispinus*	JN035509
Eudicotyledons	Cactaceae	*Pilosocereus*	*Pilosocereus aureispinus*	JN035510
Eudicotyledons	Cactaceae	*Pilosocereus*	*Pilosocereus aureispinus*	JN035511
Eudicotyledons	Cactaceae	*Pilosocereus*	*Pilosocereus aureispinus*	JN035512
Eudicotyledons	Cactaceae	*Pilosocereus*	*Pilosocereus aureispinus*	JN035513
Eudicotyledons	Cactaceae	*Pilosocereus*	*Pilosocereus aurisetus*	JN035481
Eudicotyledons	Cactaceae	*Pilosocereus*	*Pilosocereus aurisetus*	JN035482
Eudicotyledons	Cactaceae	*Pilosocereus*	*Pilosocereus aurisetus*	JN035483
Eudicotyledons	Cactaceae	*Pilosocereus*	*Pilosocereus aurisetus*	JN035484
Eudicotyledons	Cactaceae	*Pilosocereus*	*Pilosocereus aurisetus*	JN035485
Eudicotyledons	Cactaceae	*Pilosocereus*	*Pilosocereus aurisetus*	JN035486
Eudicotyledons	Cactaceae	*Pilosocereus*	*Pilosocereus aurisetus*	JN035487
Eudicotyledons	Cactaceae	*Pilosocereus*	*Pilosocereus aurisetus*	JN035488
Eudicotyledons	Cactaceae	*Pilosocereus*	*Pilosocereus aurisetus*	JN035489
Eudicotyledons	Cactaceae	*Pilosocereus*	*Pilosocereus aurisetus*	JN035490
Eudicotyledons	Cactaceae	*Pilosocereus*	*Pilosocereus vilaboensis*	JN035504

Eudicotyledons	Cactaceae	*Pilosocereus*	*Pilosocereus vilaboensis*	JN035505
Eudicotyledons	Cactaceae	*Pilosocereus*	*Pilosocereus vilaboensis*	JN035506
Eudicotyledons	Cactaceae	*Pilosocereus*	*Pilosocereus vilaboensis*	JN035507
Eudicotyledons	Cactaceae	*Pilosocereus*	*Pilosocereus vilaboensis*	JN035508
Ferns	Pteridaceae	*Pteris*	*Pteris vittata*	GU592490
Ferns	Pteridaceae	*Pteris*	*Pteris vittata*	AB575497
Ferns	Pteridaceae	*Pteris*	*Pteris vittata*	GU135302
Ferns	Lygodiaceae	*Lygodium*	*Lygodium japonicum*	GU592479
Ferns	Lygodiaceae	*Lygodium*	*Lygodium japonicum*	GU592480
Ferns	Lygodiaceae	*Lygodium*	*Lygodium japonicum*	GU592481
Ferns	Lygodiaceae	*Lygodium*	*Lygodium japonicum*	GU592482
Ferns	Lygodiaceae	*Lygodium*	*Lygodium japonicum*	GQ435002
Ferns	Lygodiaceae	*Lygodium*	*Lygodium japonicum*	GQ435003
Ferns	Lygodiaceae	*Lygodium*	*Lygodium japonicum*	JN406994
Ferns	Lygodiaceae	*Lygodium*	*Lygodium japonicum*	JN406995
Ferns	Lygodiaceae	*Lygodium*	*Lygodium japonicum*	JN406996
Ferns	Lygodiaceae	*Lygodium*	*Lygodium japonicum*	JN406997
Ferns	Lygodiaceae	*Lygodium*	*Lygodium japonicum*	AB575389
Ferns	Lygodiaceae	*Lygodium*	*Lygodium japonicum*	GU135305
Ferns	Pteridaceae	*Adiantum*	*Adiantum pedatum*	HQ596582
Ferns	Pteridaceae	*Adiantum*	*Adiantum pedatum*	AB575455
Ferns	Lygodiaceae	*Lygodium*	*Lygodium microphyllum*	AB575390
Ferns	Lygodiaceae	*Lygodium*	*Lygodium microphyllum*	GU135294
Ferns	Lygodiaceae	*Lygodium*	*Lygodium microphyllum*	GU135402
Ferns	Pteridaceae	*Pteris*	*Pteris multifida*	GU592489
Ferns	Pteridaceae	*Pteris*	*Pteris multifida*	GQ434994
Ferns	Pteridaceae	*Pteris*	*Pteris multifida*	AB575488
Ferns	Polypodiaceae	*Pyrrosia*	*Pyrrosia lingua*	GU592455
Ferns	Polypodiaceae	*Pyrrosia*	*Pyrrosia lingua*	GU592456
Ferns	Polypodiaceae	*Pyrrosia*	*Pyrrosia lingua*	AB575909
Ferns	Pteridaceae	*Pteris*	*Pteris semipinnata*	GU592488
Ferns	Pteridaceae	*Pteris*	*Pteris semipinnata*	GQ435141
Ferns	Pteridaceae	*Pteris*	*Pteris semipinnata*	AB575494
Ferns	Pteridaceae	*Adiantum*	*Adiantum hispidulum*	GQ248237
Ferns	Pteridaceae	*Adiantum*	*Adiantum hispidulum*	EF590667
Ferns	Pteridaceae	*Adiantum*	*Adiantum venustum*	GQ248238
Ferns	Pteridaceae	*Adiantum*	*Adiantum venustum*	EF590668
Ferns	Lygodiaceae	*Lygodium*	*Lygodium scandens*	GU592477
Ferns	Lygodiaceae	*Lygodium*	*Lygodium scandens*	GU592478
Ferns	Polypodiaceae	*Pyrrosia*	*Pyrrosia petiolosa*	GU592452
Ferns	Polypodiaceae	*Pyrrosia*	*Pyrrosia petiolosa*	GU592453
Ferns	Polypodiaceae	*Pyrrosia*	*Pyrrosia petiolosa*	GU592454
Ferns	Polypodiaceae	*Pyrrosia*	*Pyrrosia sheareri*	GU592458
Ferns	Polypodiaceae	*Pyrrosia*	*Pyrrosia sheareri*	GU592459
Ferns	Polypodiaceae	*Pyrrosia*	*Pyrrosia sheareri*	GU592460
Ferns	Dryopteridaceae	*Dryopteris*	*Dryopteris filix-mas*	GQ428025
Ferns	Dryopteridaceae	*Dryopteris*	*Dryopteris filix-mas*	JN189398
Ferns	Hymenophyllaceae	*Crepidomanes*	*Crepidomanes minutum*	AB575367
Ferns	Hymenophyllaceae	*Crepidomanes*	*Crepidomanes minutum*	EU338484
Ferns	Hymenophyllaceae	*Crepidomanes*	*Crepidomanes minutum*	EU123004
Ferns	Hymenophyllaceae	*Crepidomanes*	*Crepidomanes minutum*	EU123005
Ferns	Lindsaeaceae	*Lindsaea*	*Lindsaea ensifolia*	GU478521
Ferns	Lindsaeaceae	*Lindsaea*	*Lindsaea ensifolia*	GU478522

Ferns	Lindsaeaceae	*Lindsaea*	*Lindsaea ensifolia*	AB575416
Ferns	Lindsaeaceae	*Lindsaea*	*Lindsaea odorata*	GU478431
Ferns	Lindsaeaceae	*Lindsaea*	*Lindsaea odorata*	GU478432
Ferns	Lindsaeaceae	*Odontosoria*	*Odontosoria chinensis*	GU478452
Ferns	Lindsaeaceae	*Odontosoria*	*Odontosoria chinensis*	AB575428
Ferns	Lomariopsidaceae	*Nephrolepis*	*Nephrolepis cordifolia*	AB575842
Ferns	Lomariopsidaceae	*Nephrolepis*	*Nephrolepis cordifolia*	GU135431
Ferns	Aspleniaceae	*Asplenium*	*Asplenium antiquum*	AB575502
Ferns	Aspleniaceae	*Asplenium*	*Asplenium antiquum*	EU240017
Ferns	Lindsaeaceae	*Odontosoria*	*Odontosoria biflora*	GU478453
Ferns	Lindsaeaceae	*Odontosoria*	*Odontosoria biflora*	AB575427
Ferns	Hymenophyllaceae	*Crepidomanes*	*Crepidomanes bipunctatum*	EU338483
Ferns	Hymenophyllaceae	*Crepidomanes*	*Crepidomanes bipunctatum*	EU122997
Ferns	Hymenophyllaceae	*Crepidomanes*	*Crepidomanes bipunctatum*	EU122998
Ferns	Aspleniaceae	*Asplenium*	*Asplenium trichomanes*	GQ428019
Ferns	Aspleniaceae	*Asplenium*	*Asplenium trichomanes*	AB575525
Ferns	Aspleniaceae	*Asplenium*	*Asplenium trichomanes*	EU125554
Ferns	Dryopteridaceae	*Dryopteris*	*Dryopteris crassirhizoma*	GU592467
Ferns	Dryopteridaceae	*Dryopteris*	*Dryopteris crassirhizoma*	GU592468
Ferns	Dryopteridaceae	*Dryopteris*	*Dryopteris crassirhizoma*	GU592469
Ferns	Dryopteridaceae	*Dryopteris*	*Dryopteris crassirhizoma*	JN189435
Ferns	Dryopteridaceae	*Dryopteris*	*Dryopteris crassirhizoma*	AB575746
Ferns	Aspleniaceae	*Asplenium*	*Asplenium dalhousiae*	EU125563
Ferns	Aspleniaceae	*Asplenium*	*Asplenium dalhousiae*	EU125564
Ferns	Aspleniaceae	*Asplenium*	*Asplenium ceterach*	EU125557
Ferns	Aspleniaceae	*Asplenium*	*Asplenium ceterach*	EU125558
Ferns	Aspleniaceae	*Asplenium*	*Asplenium ceterach*	EU125559
Ferns	Aspleniaceae	*Asplenium*	*Asplenium ceterach*	EU125560
Ferns	Aspleniaceae	*Asplenium*	*Asplenium ceterach*	EU125561
Ferns	Dryopteridaceae	*Dryopteris*	*Dryopteris goldieana*	JN189396
Ferns	Dryopteridaceae	*Dryopteris*	*Dryopteris goldieana*	EF590693
Ferns	Dryopteridaceae	*Dryopteris*	*Dryopteris intermedia*	JN189394
Ferns	Dryopteridaceae	*Dryopteris*	*Dryopteris intermedia*	AB575765
Ferns	Dryopteridaceae	*Dryopteris*	*Dryopteris intermedia*	EU750638
Ferns	Dryopteridaceae	*Dryopteris*	*Dryopteris intermedia*	EU750639
Ferns	Dryopteridaceae	*Dryopteris*	*Dryopteris intermedia*	EU750640
Ferns	Dryopteridaceae	*Dryopteris*	*Dryopteris bissetiana*	JN189479
Ferns	Dryopteridaceae	*Dryopteris*	*Dryopteris bissetiana*	AB575740
Ferns	Dryopteridaceae	*Dryopteris*	*Dryopteris carthusiana*	JN189402
Ferns	Dryopteridaceae	*Dryopteris*	*Dryopteris carthusiana*	EU750635
Ferns	Dryopteridaceae	*Dryopteris*	*Dryopteris carthusiana*	EU750636
Ferns	Dryopteridaceae	*Dryopteris*	*Dryopteris carthusiana*	EU750637
Ferns	Dryopteridaceae	*Dryopteris*	*Dryopteris championii*	JN189480
Ferns	Dryopteridaceae	*Dryopteris*	*Dryopteris championii*	AB575742
Ferns	Dryopteridaceae	*Dryopteris*	*Dryopteris erythrosora*	JN189473
Ferns	Dryopteridaceae	*Dryopteris*	*Dryopteris erythrosora*	AB575749
Ferns	Dryopteridaceae	*Dryopteris*	*Dryopteris erythrosora*	EF590692
Ferns	Dryopteridaceae	*Dryopteris*	*Dryopteris expansa*	JN189397
Ferns	Dryopteridaceae	*Dryopteris*	*Dryopteris expansa*	AB575750
Ferns	Dryopteridaceae	*Dryopteris*	*Dryopteris formosana*	JN189437
Ferns	Dryopteridaceae	*Dryopteris*	*Dryopteris formosana*	AB575751
Ferns	Dryopteridaceae	*Dryopteris*	*Dryopteris hondoensis*	JN189475
Ferns	Dryopteridaceae	*Dryopteris*	*Dryopteris hondoensis*	AB575761

Ferns	Dryopteridaceae	*Dryopteris*	*Dryopteris lacera*	JN189477
Ferns	Dryopteridaceae	*Dryopteris*	*Dryopteris lacera*	AB575770
Ferns	Dryopteridaceae	*Dryopteris*	*Dryopteris pacifica*	JN189486
Ferns	Dryopteridaceae	*Dryopteris*	*Dryopteris pacifica*	AB575778
Ferns	Dryopteridaceae	*Dryopteris*	*Dryopteris polylepis*	JN189481
Ferns	Dryopteridaceae	*Dryopteris*	*Dryopteris polylepis*	AB575780
Ferns	Dryopteridaceae	*Dryopteris*	*Dryopteris pycnopteroides*	JN189476
Ferns	Dryopteridaceae	*Dryopteris*	*Dryopteris pycnopteroides*	AB575781
Ferns	Dryopteridaceae	*Dryopteris*	*Dryopteris sacrosancta*	JN189484
Ferns	Dryopteridaceae	*Dryopteris*	*Dryopteris sacrosancta*	AB575784
Ferns	Dryopteridaceae	*Dryopteris*	*Dryopteris tokyoensis*	JN189468
Ferns	Dryopteridaceae	*Dryopteris*	*Dryopteris tokyoensis*	AB575795
Ferns	Dryopteridaceae	*Dryopteris*	*Dryopteris uniformis*	JN189447
Ferns	Dryopteridaceae	*Dryopteris*	*Dryopteris uniformis*	AB575797
Ferns	Aspleniaceae	*Asplenium*	*Asplenium hookerianum*	EF418420
Ferns	Aspleniaceae	*Asplenium*	*Asplenium hookerianum*	EF418421
Ferns	Aspleniaceae	*Asplenium*	*Asplenium hookerianum*	EF418422
Ferns	Aspleniaceae	*Asplenium*	*Asplenium shuttleworthianum*	EU240014
Ferns	Aspleniaceae	*Asplenium*	*Asplenium shuttleworthianum*	EU240015
Ferns	Aspleniaceae	*Asplenium*	*Asplenium shuttleworthianum*	EU240016
Ferns	Dryopteridaceae	*Dryopteris*	*Dryopteris sparsa*	JN189491
Ferns	Dryopteridaceae	*Dryopteris*	*Dryopteris sparsa*	AB575794
Ferns	Lomariopsidaceae	*Nephrolepis*	*Nephrolepis auriculata*	GU592450
Ferns	Lomariopsidaceae	*Nephrolepis*	*Nephrolepis auriculata*	GU592451
Ferns	Dryopteridaceae	*Dryopteris*	*Dryopteris hendersonii*	JN189439
Ferns	Dryopteridaceae	*Dryopteris*	*Dryopteris hendersonii*	AB575760
Ferns	Dryopteridaceae	*Dryopteris*	*Dryopteris gymnosora*	JN189438
Ferns	Dryopteridaceae	*Dryopteris*	*Dryopteris gymnosora*	AB575754
Ferns	Dryopteridaceae	*Dryopteris*	*Dryopteris polita*	JN189496
Ferns	Dryopteridaceae	*Dryopteris*	*Dryopteris polita*	AB575779
Ferns	Hymenophyllaceae	*Crepidomanes*	*Crepidomanes humile*	EU122999
Ferns	Hymenophyllaceae	*Crepidomanes*	*Crepidomanes humile*	EU123000
Ferns	Hymenophyllaceae	*Crepidomanes*	*Crepidomanes humile*	EU123001
Ferns	Hymenophyllaceae	*Crepidomanes*	*Crepidomanes kurzii*	EU123002
Ferns	Hymenophyllaceae	*Crepidomanes*	*Crepidomanes kurzii*	EU123003
Ferns	Dryopteridaceae	*Dryopteris*	*Dryopteris marginalis*	JN189393
Ferns	Dryopteridaceae	*Dryopteris*	*Dryopteris marginalis*	EU750641
Ferns	Dryopteridaceae	*Dryopteris*	*Dryopteris marginalis*	EU750642
Ferns	Dryopteridaceae	*Dryopteris*	*Dryopteris marginalis*	EU750643
Ferns	Dryopteridaceae	*Dryopteris*	*Dryopteris marginalis*	EU750644
Ferns	Lindsaeaceae	*Lindsaea*	*Lindsaea blotiana*	GU478510
Ferns	Lindsaeaceae	*Lindsaea*	*Lindsaea blotiana*	GU478511
Ferns	Lindsaeaceae	*Lindsaea*	*Lindsaea divaricata*	EU146032
Ferns	Lindsaeaceae	*Lindsaea*	*Lindsaea divaricata*	EU146033
Ferns	Lindsaeaceae	*Lindsaea*	*Lindsaea divaricata*	EU146034
Ferns	Lindsaeaceae	*Lindsaea*	*Lindsaea divaricata*	EU146036
Ferns	Lindsaeaceae	*Lindsaea*	*Lindsaea divaricata*	EU146037
Ferns	Lindsaeaceae	*Lindsaea*	*Lindsaea divaricata*	EU146040
Ferns	Lindsaeaceae	*Lindsaea*	*Lindsaea digitata*	EU146035
Ferns	Lindsaeaceae	*Lindsaea*	*Lindsaea digitata*	EU146038
Ferns	Lindsaeaceae	*Lindsaea*	*Lindsaea digitata*	EU146039
Ferns	Lindsaeaceae	*Lindsaea*	*Lindsaea digitata*	EU146041
Ferns	Lindsaeaceae	*Lindsaea*	*Lindsaea bifida*	GU478485

Ferns	Lindsaeaceae	*Lindsaea*	*Lindsaea bifida*	FJ360901
Ferns	Lindsaeaceae	*Lindsaea*	*Lindsaea chienii*	FJ360905
Ferns	Lindsaeaceae	*Lindsaea*	*Lindsaea chienii*	AB575415
Ferns	Lindsaeaceae	*Lindsaea*	*Lindsaea coarctata*	GU478507
Ferns	Lindsaeaceae	*Lindsaea*	*Lindsaea coarctata*	FJ360906
Ferns	Lindsaeaceae	*Lindsaea*	*Lindsaea hemiglossa*	GU478487
Ferns	Lindsaeaceae	*Lindsaea*	*Lindsaea hemiglossa*	FJ360910
Ferns	Lindsaeaceae	*Lindsaea*	*Lindsaea javanensis*	FJ360911
Ferns	Lindsaeaceae	*Lindsaea*	*Lindsaea javanensis*	FJ360912
Ferns	Lindsaeaceae	*Lindsaea*	*Lindsaea javanensis*	AB575418
Ferns	Lindsaeaceae	*Lindsaea*	*Lindsaea lapeyrousei*	GU478556
Ferns	Lindsaeaceae	*Lindsaea*	*Lindsaea lapeyrousei*	FJ360916
Ferns	Lindsaeaceae	*Lindsaea*	*Lindsaea portoricensis*	FJ360928
Ferns	Lindsaeaceae	*Lindsaea*	*Lindsaea portoricensis*	FJ360937
Ferns	Lindsaeaceae	*Lindsaea*	*Lindsaea diplosora*	GU478535
Ferns	Lindsaeaceae	*Lindsaea*	*Lindsaea diplosora*	GU478565
Ferns	Lindsaeaceae	*Lindsaea*	*Lindsaea doryphora*	GU478539
Ferns	Lindsaeaceae	*Lindsaea*	*Lindsaea doryphora*	GU478540
Ferns	Lindsaeaceae	*Lindsaea*	*Lindsaea imrayana*	GU478435
Ferns	Lindsaeaceae	*Lindsaea*	*Lindsaea imrayana*	GU478436
Ferns	Lindsaeaceae	*Lindsaea*	*Lindsaea integra*	GU478531
Ferns	Lindsaeaceae	*Lindsaea*	*Lindsaea integra*	GU478532
Ferns	Lindsaeaceae	*Lindsaea*	*Lindsaea kawabatae*	GU478499
Ferns	Lindsaeaceae	*Lindsaea*	*Lindsaea kawabatae*	AB575419
Ferns	Lindsaeaceae	*Lindsaea*	*Lindsaea lucida*	GU478542
Ferns	Lindsaeaceae	*Lindsaea*	*Lindsaea lucida*	AB575420
Ferns	Lindsaeaceae	*Lindsaea*	*Lindsaea malayensis*	GU478530
Ferns	Lindsaeaceae	*Lindsaea*	*Lindsaea malayensis*	GU478545
Ferns	Lindsaeaceae	*Lindsaea*	*Lindsaea media*	GU478515
Ferns	Lindsaeaceae	*Lindsaea*	*Lindsaea media*	GU478516
Ferns	Lindsaeaceae	*Lindsaea*	*Lindsaea obtusa*	GU478547
Ferns	Lindsaeaceae	*Lindsaea*	*Lindsaea obtusa*	GU478548
Ferns	Lindsaeaceae	*Lindsaea*	*Lindsaea rigidiuscula*	GU478475
Ferns	Lindsaeaceae	*Lindsaea*	*Lindsaea rigidiuscula*	GU478476
Ferns	Dryopteridaceae	*Dryopteris*	*Dryopteris chinensis*	JN189433
Ferns	Dryopteridaceae	*Dryopteris*	*Dryopteris chinensis*	AB575743
Ferns	Dryopteridaceae	*Dryopteris*	*Dryopteris monticola*	JN189482
Ferns	Dryopteridaceae	*Dryopteris*	*Dryopteris monticola*	AB575775
Ferns	Dryopteridaceae	*Dryopteris*	*Dryopteris sordidipes*	JN189495
Ferns	Dryopteridaceae	*Dryopteris*	*Dryopteris sordidipes*	AB575793
Ferns	Hymenophyllaceae	*Hymenophyllum*	*Hymenophyllum polyanthos*	AB575375
Ferns	Hymenophyllaceae	*Hymenophyllum*	*Hymenophyllum polyanthos*	EU338480
Ferns	Hymenophyllaceae	*Hymenophyllum*	*Hymenophyllum polyanthos*	EU123016
Ferns	Hymenophyllaceae	*Hymenophyllum*	*Hymenophyllum polyanthos*	EU123017
Ferns	Hymenophyllaceae	*Hymenophyllum*	*Hymenophyllum polyanthos*	EU123018
Ferns	Hymenophyllaceae	*Hymenophyllum*	*Hymenophyllum digitatum*	EU338481
Ferns	Hymenophyllaceae	*Hymenophyllum*	*Hymenophyllum digitatum*	EU338482
Ferns	Hymenophyllaceae	*Hymenophyllum*	*Hymenophyllum digitatum*	EU123008
Ferns	Hymenophyllaceae	*Hymenophyllum*	*Hymenophyllum digitatum*	EU123009
Ferns	Hymenophyllaceae	*Polyphlebium*	*Polyphlebium borbonicum*	EU338477
Ferns	Hymenophyllaceae	*Polyphlebium*	*Polyphlebium borbonicum*	EU123019
Ferns	Hymenophyllaceae	*Polyphlebium*	*Polyphlebium borbonicum*	EU123020
Ferns	Hymenophyllaceae	*Polyphlebium*	*Polyphlebium borbonicum*	EU123021

Ferns	Hymenophyllaceae	*Polyphlebium*	*Polyphlebium endlicherianum*	EU338478
Ferns	Hymenophyllaceae	*Polyphlebium*	*Polyphlebium endlicherianum*	EU123022
Ferns	Hymenophyllaceae	*Polyphlebium*	*Polyphlebium endlicherianum*	EU123023
Ferns	Hymenophyllaceae	*Hymenophyllum*	*Hymenophyllum pallidum*	EU338479
Ferns	Hymenophyllaceae	*Hymenophyllum*	*Hymenophyllum pallidum*	EU123011
Ferns	Hymenophyllaceae	*Hymenophyllum*	*Hymenophyllum pallidum*	EU123012
Ferns	Hymenophyllaceae	*Hymenophyllum*	*Hymenophyllum pallidum*	EU123013
Ferns	Hymenophyllaceae	*Hymenophyllum*	*Hymenophyllum pallidum*	EU123014
Ferns	Hymenophyllaceae	*Hymenophyllum*	*Hymenophyllum pallidum*	EU123015
Ferns	Pteridaceae	*Cheilanthes*	*Cheilanthes arizonica*	JN647842
Ferns	Pteridaceae	*Cheilanthes*	*Cheilanthes arizonica*	JN647843
Ferns	Pteridaceae	*Cheilanthes*	*Cheilanthes arizonica*	JN647844
Ferns	Pteridaceae	*Cheilanthes*	*Cheilanthes arizonica*	JN647845
Ferns	Pteridaceae	*Cheilanthes*	*Cheilanthes angustifolia*	JN647835
Ferns	Pteridaceae	*Cheilanthes*	*Cheilanthes angustifolia*	JN647836
Ferns	Pteridaceae	*Cheilanthes*	*Cheilanthes angustifolia*	JN647837
Ferns	Pteridaceae	*Cheilanthes*	*Cheilanthes angustifolia*	JN647838
Ferns	Pteridaceae	*Cheilanthes*	*Cheilanthes angustifolia*	JN647839
Ferns	Pteridaceae	*Cheilanthes*	*Cheilanthes angustifolia*	JN647840
Ferns	Pteridaceae	*Cheilanthes*	*Cheilanthes chaerophylla*	JN647846
Ferns	Pteridaceae	*Cheilanthes*	*Cheilanthes chaerophylla*	JN647847
Ferns	Pteridaceae	*Cheilanthes*	*Cheilanthes chaerophylla*	JN647848
Ferns	Pteridaceae	*Cheilanthes*	*Cheilanthes chaerophylla*	JN647849
Ferns	Pteridaceae	*Cheilanthes*	*Cheilanthes complanata*	JN647850
Ferns	Pteridaceae	*Cheilanthes*	*Cheilanthes complanata*	JN647851
Ferns	Pteridaceae	*Cheilanthes*	*Cheilanthes cuneata*	JN647852
Ferns	Pteridaceae	*Cheilanthes*	*Cheilanthes cuneata*	JN647853
Ferns	Pteridaceae	*Cheilanthes*	*Cheilanthes decomposita*	JN647854
Ferns	Pteridaceae	*Cheilanthes*	*Cheilanthes decomposita*	JN647855
Ferns	Pteridaceae	*Cheilanthes*	*Cheilanthes decomposita*	JN647856
Ferns	Pteridaceae	*Cheilanthes*	*Cheilanthes decomposita*	JN647857
Ferns	Pteridaceae	*Cheilanthes*	*Cheilanthes decurrens*	JN647858
Ferns	Pteridaceae	*Cheilanthes*	*Cheilanthes decurrens*	JN647859
Ferns	Pteridaceae	*Cheilanthes*	*Cheilanthes harrisii*	JN647860
Ferns	Pteridaceae	*Cheilanthes*	*Cheilanthes harrisii*	JN647861
Ferns	Pteridaceae	*Cheilanthes*	*Cheilanthes hintoniorum*	JN647863
Ferns	Pteridaceae	*Cheilanthes*	*Cheilanthes hintoniorum*	JN647864
Ferns	Pteridaceae	*Cheilanthes*	*Cheilanthes kaulfussii*	JN647865
Ferns	Pteridaceae	*Cheilanthes*	*Cheilanthes kaulfussii*	JN647866
Ferns	Pteridaceae	*Cheilanthes*	*Cheilanthes kaulfussii*	JN647867
Ferns	Pteridaceae	*Cheilanthes*	*Cheilanthes kaulfussii*	JN647868
Ferns	Pteridaceae	*Cheilanthes*	*Cheilanthes kaulfussii*	JN647869
Ferns	Pteridaceae	*Cheilanthes*	*Cheilanthes marginata*	JN647870
Ferns	Pteridaceae	*Cheilanthes*	*Cheilanthes marginata*	JN647871
Ferns	Pteridaceae	*Cheilanthes*	*Cheilanthes marginata*	JN647872
Ferns	Pteridaceae	*Cheilanthes*	*Cheilanthes marginata*	JN647873
Ferns	Pteridaceae	*Cheilanthes*	*Cheilanthes marginata*	JN647874
Ferns	Pteridaceae	*Cheilanthes*	*Cheilanthes marginata*	JN647875
Ferns	Pteridaceae	*Cheilanthes*	*Cheilanthes marginata*	JN647876
Ferns	Pteridaceae	*Cheilanthes*	*Cheilanthes marginata*	JN647877
Ferns	Pteridaceae	*Cheilanthes*	*Cheilanthes marginata*	JN647878
Ferns	Pteridaceae	*Cheilanthes*	*Cheilanthes membranacea*	JN647879
Ferns	Pteridaceae	*Cheilanthes*	*Cheilanthes membranacea*	JN647880

Ferns	Pteridaceae	*Cheilanthes*	*Cheilanthes pellaeopsis*	JN647881
Ferns	Pteridaceae	*Cheilanthes*	*Cheilanthes pellaeopsis*	JN647882
Ferns	Pteridaceae	*Cheilanthes*	*Cheilanthes purpusii*	JN647883
Ferns	Pteridaceae	*Cheilanthes*	*Cheilanthes purpusii*	JN647884
Ferns	Pteridaceae	*Cheilanthes*	*Cheilanthes pyramidalis*	JN647885
Ferns	Pteridaceae	*Cheilanthes*	*Cheilanthes pyramidalis*	JN647886
Ferns	Pteridaceae	*Cheilanthes*	*Cheilanthes pyramidalis*	JN647887
Ferns	Pteridaceae	*Cheilanthes*	*Cheilanthes pyramidalis*	JN647888
Ferns	Pteridaceae	*Cheilanthes*	*Cheilanthes pyramidalis*	JN647889
Ferns	Pteridaceae	*Cheilanthes*	*Cheilanthes pyramidalis*	JN647890
Ferns	Pteridaceae	*Cheilanthes*	*Cheilanthes pyramidalis*	JN647891
Ferns	Woodsiaceae	*Deparia*	*Deparia petersenii*	JN673925
Ferns	Woodsiaceae	*Deparia*	*Deparia petersenii*	AB575589
Ferns	Woodsiaceae	*Deparia*	*Deparia lancea*	JN673910
Ferns	Woodsiaceae	*Deparia*	*Deparia lancea*	JN673911
Ferns	Woodsiaceae	*Deparia*	*Deparia lancea*	JN673912
Ferns	Woodsiaceae	*Deparia*	*Deparia lancea*	JN673913
Ferns	Woodsiaceae	*Deparia*	*Deparia lancea*	JN673914
Ferns	Woodsiaceae	*Deparia*	*Deparia lancea*	JN673915
Ferns	Woodsiaceae	*Deparia*	*Deparia lancea*	JN673916
Ferns	Woodsiaceae	*Deparia*	*Deparia lancea*	JN673917
Ferns	Woodsiaceae	*Deparia*	*Deparia lancea*	JN673918
Ferns	Woodsiaceae	*Deparia*	*Deparia lancea*	JN673919
Ferns	Woodsiaceae	*Deparia*	*Deparia lancea*	AB575585
Ferns	Woodsiaceae	*Deparia*	*Deparia longipes*	JN673920
Ferns	Woodsiaceae	*Deparia*	*Deparia longipes*	JN673921
Ferns	Woodsiaceae	*Deparia*	*Deparia longipes*	JN673922
Ferns	Woodsiaceae	*Deparia*	*Deparia longipes*	JN673923
Ferns	Woodsiaceae	*Deparia*	*Deparia subfluvialis*	JN673926
Ferns	Woodsiaceae	*Deparia*	*Deparia subfluvialis*	JN673927
Ferns	Woodsiaceae	*Deparia*	*Deparia subfluvialis*	JN673928
Gymnosperms	Cupressaceae	*Cupressus*	*Cupressus sempervirens*	FR832556
Gymnosperms	Cupressaceae	*Cupressus*	*Cupressus sempervirens*	FR832557
Gymnosperms	Cupressaceae	*Cupressus*	*Cupressus sempervirens*	FR832558
Gymnosperms	Cupressaceae	*Cupressus*	*Cupressus sempervirens*	FN689387
Gymnosperms	Cupressaceae	*Juniperus*	*Juniperus virginiana*	EU750617
Gymnosperms	Cupressaceae	*Juniperus*	*Juniperus virginiana*	EU750618
Gymnosperms	Cupressaceae	*Juniperus*	*Juniperus virginiana*	EU750619
Gymnosperms	Cupressaceae	*Juniperus*	*Juniperus virginiana*	EU750620
Gymnosperms	Cupressaceae	*Juniperus*	*Juniperus communis*	FR832568
Gymnosperms	Cupressaceae	*Juniperus*	*Juniperus communis*	FR865108
Gymnosperms	Cupressaceae	*Juniperus*	*Juniperus communis*	EU750613
Gymnosperms	Cupressaceae	*Juniperus*	*Juniperus communis*	EU750614
Gymnosperms	Cupressaceae	*Juniperus*	*Juniperus communis*	EU750615
Gymnosperms	Cupressaceae	*Juniperus*	*Juniperus communis*	EU750616
Gymnosperms	Cupressaceae	*Juniperus*	*Juniperus phoenicea*	FR832560
Gymnosperms	Cupressaceae	*Juniperus*	*Juniperus phoenicea*	FR832561
Gymnosperms	Cupressaceae	*Juniperus*	*Juniperus phoenicea*	FR832562
Gymnosperms	Cupressaceae	*Juniperus*	*Juniperus phoenicea*	FN689390
Gymnosperms	Cupressaceae	*Cupressus*	*Cupressus funebris*	JN044326
Gymnosperms	Cupressaceae	*Cupressus*	*Cupressus funebris*	JN044327
Gymnosperms	Cupressaceae	*Cupressus*	*Cupressus funebris*	JN044328
Gymnosperms	Cupressaceae	*Cupressus*	*Cupressus funebris*	JN044329

Gymnosperms	Cupressaceae	*Cupressus*	*Cupressus funebris*	JN044330
Gymnosperms	Cupressaceae	*Cupressus*	*Cupressus duclouxiana*	JN044318
Gymnosperms	Cupressaceae	*Cupressus*	*Cupressus duclouxiana*	JN044319
Gymnosperms	Cupressaceae	*Cupressus*	*Cupressus duclouxiana*	JN044320
Gymnosperms	Cupressaceae	*Cupressus*	*Cupressus duclouxiana*	JN044321
Gymnosperms	Cupressaceae	*Cupressus*	*Cupressus duclouxiana*	JN044322
Gymnosperms	Cupressaceae	*Cupressus*	*Cupressus duclouxiana*	JN044323
Gymnosperms	Cupressaceae	*Juniperus*	*Juniperus przewalskii*	JN045060
Gymnosperms	Cupressaceae	*Juniperus*	*Juniperus przewalskii*	JN045061
Gymnosperms	Cupressaceae	*Juniperus*	*Juniperus przewalskii*	JN045062
Gymnosperms	Cupressaceae	*Juniperus*	*Juniperus przewalskii*	JN045063
Gymnosperms	Cupressaceae	*Juniperus*	*Juniperus przewalskii*	JN045064
Gymnosperms	Cupressaceae	*Juniperus*	*Juniperus przewalskii*	HQ832759
Gymnosperms	Cupressaceae	*Juniperus*	*Juniperus tibetica*	JN045072
Gymnosperms	Cupressaceae	*Juniperus*	*Juniperus tibetica*	JN045073
Gymnosperms	Cupressaceae	*Juniperus*	*Juniperus tibetica*	JN045074
Gymnosperms	Cupressaceae	*Juniperus*	*Juniperus tibetica*	JN045075
Gymnosperms	Cupressaceae	*Juniperus*	*Juniperus tibetica*	JN045076
Gymnosperms	Cupressaceae	*Juniperus*	*Juniperus tibetica*	JN045077
Gymnosperms	Cupressaceae	*Juniperus*	*Juniperus tibetica*	HQ832751
Gymnosperms	Cupressaceae	*Juniperus*	*Juniperus tibetica*	HQ832752
Gymnosperms	Cupressaceae	*Juniperus*	*Juniperus tibetica*	HQ832753
Gymnosperms	Cupressaceae	*Juniperus*	*Juniperus tibetica*	HQ832754
Gymnosperms	Cupressaceae	*Cupressus*	*Cupressus austrotibetica*	JN044324
Gymnosperms	Cupressaceae	*Cupressus*	*Cupressus austrotibetica*	JN044325
Gymnosperms	Cupressaceae	*Cupressus*	*Cupressus gigantea*	JN044316
Gymnosperms	Cupressaceae	*Cupressus*	*Cupressus gigantea*	JN044317
Gymnosperms	Cupressaceae	*Juniperus*	*Juniperus squamata*	JN045069
Gymnosperms	Cupressaceae	*Juniperus*	*Juniperus squamata*	JN045070
Gymnosperms	Cupressaceae	*Juniperus*	*Juniperus squamata*	JN045071
Gymnosperms	Cupressaceae	*Juniperus*	*Juniperus squamata*	HQ832760
Gymnosperms	Cupressaceae	*Juniperus*	*Juniperus pingii*	JN045055
Gymnosperms	Cupressaceae	*Juniperus*	*Juniperus pingii*	JN045056
Gymnosperms	Cupressaceae	*Juniperus*	*Juniperus pingii*	JN045057
Gymnosperms	Cupressaceae	*Juniperus*	*Juniperus pingii*	JN045058
Gymnosperms	Cupressaceae	*Juniperus*	*Juniperus pingii*	JN045059
Gymnosperms	Cupressaceae	*Juniperus*	*Juniperus saltuaria*	JN045065
Gymnosperms	Cupressaceae	*Juniperus*	*Juniperus saltuaria*	JN045066
Gymnosperms	Cupressaceae	*Juniperus*	*Juniperus saltuaria*	HQ832755
Gymnosperms	Cupressaceae	*Juniperus*	*Juniperus saltuaria*	HQ832756
Gymnosperms	Cupressaceae	*Juniperus*	*Juniperus microsperma*	JN045052
Gymnosperms	Cupressaceae	*Juniperus*	*Juniperus microsperma*	JN045053
Gymnosperms	Cupressaceae	*Juniperus*	*Juniperus microsperma*	JN045054
Gymnosperms	Cupressaceae	*Juniperus*	*Juniperus microsperma*	HQ832761
Gymnosperms	Cupressaceae	*Juniperus*	*Juniperus convallium*	JN045047
Gymnosperms	Cupressaceae	*Juniperus*	*Juniperus convallium*	JN045048
Gymnosperms	Cupressaceae	*Juniperus*	*Juniperus convallium*	JN045049
Gymnosperms	Cupressaceae	*Juniperus*	*Juniperus convallium*	JN045050
Gymnosperms	Cupressaceae	*Juniperus*	*Juniperus convallium*	JN045051
Gymnosperms	Cupressaceae	*Juniperus*	*Juniperus convallium*	HQ832757
Gymnosperms	Cupressaceae	*Juniperus*	*Juniperus convallium*	HQ832758
Gymnosperms	Pinaceae	*Picea*	*Picea abies*	FR832550
Gymnosperms	Pinaceae	*Picea*	*Picea abies*	FR832551

Gymnosperms	Pinaceae	*Picea*	*Picea abies*	FR832552
Gymnosperms	Pinaceae	*Picea*	*Picea abies*	FJ493294
Gymnosperms	Pinaceae	*Picea*	*Picea abies*	HQ114857
Gymnosperms	Pinaceae	*Picea*	*Picea abies*	HQ114858
Gymnosperms	Pinaceae	*Picea*	*Picea glauca*	EU750621
Gymnosperms	Pinaceae	*Picea*	*Picea glauca*	EU750622
Gymnosperms	Pinaceae	*Picea*	*Picea glauca*	EU750623
Gymnosperms	Pinaceae	*Picea*	*Picea glauca*	EU750624
Gymnosperms	Pinaceae	*Picea*	*Picea glauca*	HQ114832
Gymnosperms	Pinaceae	*Picea*	*Picea pungens*	HQ114866
Gymnosperms	Pinaceae	*Picea*	*Picea pungens*	HQ114867
Gymnosperms	Pinaceae	*Picea*	*Picea mariana*	EU750625
Gymnosperms	Pinaceae	*Picea*	*Picea mariana*	EU750626
Gymnosperms	Pinaceae	*Picea*	*Picea mariana*	HQ114835
Gymnosperms	Pinaceae	*Pinus*	*Pinus pinea*	FR832538
Gymnosperms	Pinaceae	*Pinus*	*Pinus pinea*	FR832539
Gymnosperms	Pinaceae	*Pinus*	*Pinus pinea*	FR832540
Gymnosperms	Pinaceae	*Pinus*	*Pinus pinea*	FN689389
Gymnosperms	Pinaceae	*Pinus*	*Pinus strobus*	GQ248367
Gymnosperms	Pinaceae	*Pinus*	*Pinus strobus*	GQ435358
Gymnosperms	Pinaceae	*Pinus*	*Pinus strobus*	EF590725
Gymnosperms	Pinaceae	*Pinus*	*Pinus strobus*	EU750629
Gymnosperms	Pinaceae	*Pinus*	*Pinus strobus*	EU750630
Gymnosperms	Pinaceae	*Pinus*	*Pinus strobus*	EU750631
Gymnosperms	Pinaceae	*Pinus*	*Pinus sylvestris*	FR832541
Gymnosperms	Pinaceae	*Pinus*	*Pinus sylvestris*	FR832542
Gymnosperms	Pinaceae	*Pinus*	*Pinus sylvestris*	FR832543
Gymnosperms	Pinaceae	*Pinus*	*Pinus sylvestris*	FJ493296
Gymnosperms	Pinaceae	*Pinus*	*Pinus sylvestris*	EU750632
Gymnosperms	Pinaceae	*Pinus*	*Pinus sylvestris*	EU750633
Gymnosperms	Pinaceae	*Pinus*	*Pinus sylvestris*	EU750634
Gymnosperms	Pinaceae	*Pinus*	*Pinus thunbergii*	GQ463529
Gymnosperms	Pinaceae	*Pinus*	*Pinus thunbergii*	GQ463530
Gymnosperms	Pinaceae	*Pinus*	*Pinus thunbergii*	JN046375
Gymnosperms	Pinaceae	*Pinus*	*Pinus thunbergii*	JN046376
Gymnosperms	Pinaceae	*Pinus*	*Pinus thunbergii*	JN046377
Gymnosperms	Pinaceae	*Pinus*	*Pinus thunbergii*	JN046378
Gymnosperms	Pinaceae	*Pinus*	*Pinus thunbergii*	JN046379
Gymnosperms	Pinaceae	*Pinus*	*Pinus thunbergii*	HQ114893
Gymnosperms	Pinaceae	*Pinus*	*Pinus thunbergii*	HQ849863
Gymnosperms	Pinaceae	*Pinus*	*Pinus banksiana*	EU750627
Gymnosperms	Pinaceae	*Pinus*	*Pinus banksiana*	EU750628
Gymnosperms	Taxaceae	*Amentotaxus*	*Amentotaxus argotaenia*	EF660691
Gymnosperms	Taxaceae	*Amentotaxus*	*Amentotaxus argotaenia*	JN043910
Gymnosperms	Taxaceae	*Amentotaxus*	*Amentotaxus argotaenia*	JN043911
Gymnosperms	Taxaceae	*Amentotaxus*	*Amentotaxus argotaenia*	JN043912
Gymnosperms	Taxaceae	*Amentotaxus*	*Amentotaxus argotaenia*	JN043913
Gymnosperms	Taxaceae	*Amentotaxus*	*Amentotaxus argotaenia*	JN043914
Gymnosperms	Taxaceae	*Taxus*	*Taxus baccata*	EF017303
Gymnosperms	Taxaceae	*Taxus*	*Taxus baccata*	GQ248397
Gymnosperms	Taxaceae	*Taxus*	*Taxus baccata*	FR832569
Gymnosperms	Taxaceae	*Taxus*	*Taxus baccata*	FR832570
Gymnosperms	Taxaceae	*Taxus*	*Taxus baccata*	FR832571

Gymnosperms	Taxaceae	*Taxus*	*Taxus baccata*	FJ493316
Gymnosperms	Taxaceae	*Taxus*	*Taxus baccata*	EF590746
Gymnosperms	Taxaceae	*Taxus*	*Taxus baccata*	HM591111
Gymnosperms	Taxaceae	*Taxus*	*Taxus baccata*	HM591112
Gymnosperms	Taxaceae	*Taxus*	*Taxus baccata*	HM591113
Gymnosperms	Taxaceae	*Taxus*	*Taxus baccata*	HM591114
Gymnosperms	Taxaceae	*Taxus*	*Taxus baccata*	HM591115
Gymnosperms	Pinaceae	*Pinus*	*Pinus mugo*	FR832533
Gymnosperms	Pinaceae	*Pinus*	*Pinus mugo*	FR832534
Gymnosperms	Pinaceae	*Pinus*	*Pinus mugo*	FR832535
Gymnosperms	Ephedraceae	*Ephedra*	*Ephedra sinica*	GQ435178
Gymnosperms	Ephedraceae	*Ephedra*	*Ephedra sinica*	GQ463516
Gymnosperms	Ephedraceae	*Ephedra*	*Ephedra sinica*	AY849347
Gymnosperms	Ephedraceae	*Ephedra*	*Ephedra sinica*	AY849349
Gymnosperms	Araucariaceae	*Araucaria*	*Araucaria araucana*	AM922000
Gymnosperms	Araucariaceae	*Araucaria*	*Araucaria araucana*	AM922001
Gymnosperms	Pinaceae	*Abies*	*Abies alba*	FR832520
Gymnosperms	Pinaceae	*Abies*	*Abies alba*	FR832521
Gymnosperms	Pinaceae	*Abies*	*Abies alba*	FR832522
Gymnosperms	Pinaceae	*Abies*	*Abies alba*	FJ493291
Gymnosperms	Taxaceae	*Amentotaxus*	*Amentotaxus formosana*	GQ463512
Gymnosperms	Taxaceae	*Amentotaxus*	*Amentotaxus formosana*	EF660670
Gymnosperms	Taxaceae	*Amentotaxus*	*Amentotaxus formosana*	JN043915
Gymnosperms	Taxaceae	*Amentotaxus*	*Amentotaxus formosana*	JN043916
Gymnosperms	Taxaceae	*Amentotaxus*	*Amentotaxus formosana*	JN043917
Gymnosperms	Araucariaceae	*Araucaria*	*Araucaria cunninghamii*	GQ463518
Gymnosperms	Araucariaceae	*Araucaria*	*Araucaria cunninghamii*	AM922006
Gymnosperms	Araucariaceae	*Araucaria*	*Araucaria hunsteinii*	FJ173528
Gymnosperms	Araucariaceae	*Araucaria*	*Araucaria hunsteinii*	AM922007
Gymnosperms	Pinaceae	*Pinus*	*Pinus cembra*	FR832523
Gymnosperms	Pinaceae	*Pinus*	*Pinus cembra*	FR832524
Gymnosperms	Pinaceae	*Pinus*	*Pinus nigra*	EU531715
Gymnosperms	Pinaceae	*Pinus*	*Pinus nigra*	FJ493295
Gymnosperms	Pinaceae	*Pinus*	*Pinus nigra*	FN689385
Gymnosperms	Podocarpaceae	*Podocarpus*	*Podocarpus macrophyllus*	GQ463514
Gymnosperms	Podocarpaceae	*Podocarpus*	*Podocarpus macrophyllus*	EF660690
Gymnosperms	Araucariaceae	*Araucaria*	*Araucaria bernieri*	FJ173518
Gymnosperms	Araucariaceae	*Araucaria*	*Araucaria bernieri*	FJ173519
Gymnosperms	Araucariaceae	*Araucaria*	*Araucaria bernieri*	AM922002
Gymnosperms	Araucariaceae	*Araucaria*	*Araucaria biramulata*	FJ173520
Gymnosperms	Araucariaceae	*Araucaria*	*Araucaria biramulata*	FJ173521
Gymnosperms	Araucariaceae	*Araucaria*	*Araucaria biramulata*	FJ173522
Gymnosperms	Araucariaceae	*Araucaria*	*Araucaria biramulata*	AM922004
Gymnosperms	Araucariaceae	*Araucaria*	*Araucaria columnaris*	FJ173523
Gymnosperms	Araucariaceae	*Araucaria*	*Araucaria columnaris*	FJ173524
Gymnosperms	Araucariaceae	*Araucaria*	*Araucaria columnaris*	AM922005
Gymnosperms	Araucariaceae	*Araucaria*	*Araucaria humboldtensis*	FJ173526
Gymnosperms	Araucariaceae	*Araucaria*	*Araucaria humboldtensis*	FJ173527
Gymnosperms	Araucariaceae	*Araucaria*	*Araucaria laubenfelsii*	FJ173529
Gymnosperms	Araucariaceae	*Araucaria*	*Araucaria laubenfelsii*	FJ173530
Gymnosperms	Araucariaceae	*Araucaria*	*Araucaria luxurians*	FJ173531
Gymnosperms	Araucariaceae	*Araucaria*	*Araucaria luxurians*	FJ173532
Gymnosperms	Araucariaceae	*Araucaria*	*Araucaria luxurians*	FJ173533

Gymnosperms	Araucariaceae	*Araucaria*	*Araucaria montana*	FJ173534
Gymnosperms	Araucariaceae	*Araucaria*	*Araucaria montana*	FJ173535
Gymnosperms	Araucariaceae	*Araucaria*	*Araucaria montana*	FJ173536
Gymnosperms	Araucariaceae	*Araucaria*	*Araucaria montana*	FJ173537
Gymnosperms	Araucariaceae	*Araucaria*	*Araucaria montana*	AM922008
Gymnosperms	Araucariaceae	*Araucaria*	*Araucaria muelleri*	FJ173538
Gymnosperms	Araucariaceae	*Araucaria*	*Araucaria muelleri*	AM922009
Gymnosperms	Araucariaceae	*Araucaria*	*Araucaria muelleri*	AM922010
Gymnosperms	Araucariaceae	*Araucaria*	*Araucaria nemorosa*	FJ173539
Gymnosperms	Araucariaceae	*Araucaria*	*Araucaria nemorosa*	FJ173540
Gymnosperms	Araucariaceae	*Araucaria*	*Araucaria nemorosa*	AM922011
Gymnosperms	Araucariaceae	*Araucaria*	*Araucaria rulei*	FJ173541
Gymnosperms	Araucariaceae	*Araucaria*	*Araucaria rulei*	FJ173542
Gymnosperms	Araucariaceae	*Araucaria*	*Araucaria rulei*	AM922012
Gymnosperms	Araucariaceae	*Araucaria*	*Araucaria rulei*	AM922013
Gymnosperms	Araucariaceae	*Araucaria*	*Araucaria schmidii*	FJ173543
Gymnosperms	Araucariaceae	*Araucaria*	*Araucaria schmidii*	FJ173544
Gymnosperms	Araucariaceae	*Araucaria*	*Araucaria schmidii*	FJ173545
Gymnosperms	Araucariaceae	*Araucaria*	*Araucaria scopulorum*	FJ173546
Gymnosperms	Araucariaceae	*Araucaria*	*Araucaria scopulorum*	FJ173547
Gymnosperms	Araucariaceae	*Araucaria*	*Araucaria scopulorum*	FJ173548
Gymnosperms	Araucariaceae	*Araucaria*	*Araucaria subulata*	FJ173549
Gymnosperms	Araucariaceae	*Araucaria*	*Araucaria subulata*	AM922014
Gymnosperms	Pinaceae	*Picea*	*Picea jezoensis*	JN046260
Gymnosperms	Pinaceae	*Picea*	*Picea jezoensis*	JN046261
Gymnosperms	Pinaceae	*Picea*	*Picea jezoensis*	JN046262
Gymnosperms	Pinaceae	*Picea*	*Picea jezoensis*	HQ114854
Gymnosperms	Pinaceae	*Picea*	*Picea jezoensis*	HQ114855
Gymnosperms	Pinaceae	*Picea*	*Picea jezoensis*	HQ114868
Gymnosperms	Pinaceae	*Pinus*	*Pinus bungeana*	GQ435356
Gymnosperms	Pinaceae	*Pinus*	*Pinus bungeana*	GQ435357
Gymnosperms	Pinaceae	*Pinus*	*Pinus halepensis*	EU531714
Gymnosperms	Pinaceae	*Pinus*	*Pinus halepensis*	FR832528
Gymnosperms	Pinaceae	*Pinus*	*Pinus halepensis*	FR832529
Gymnosperms	Pinaceae	*Pinus*	*Pinus halepensis*	FR832530
Gymnosperms	Pinaceae	*Pinus*	*Pinus halepensis*	FR832531
Gymnosperms	Pinaceae	*Pinus*	*Pinus halepensis*	FN689388
Gymnosperms	Pinaceae	*Pinus*	*Pinus parviflora*	GQ248366
Gymnosperms	Pinaceae	*Pinus*	*Pinus parviflora*	EF590724
Gymnosperms	Pinaceae	*Pinus*	*Pinus pinaster*	FR832525
Gymnosperms	Pinaceae	*Pinus*	*Pinus pinaster*	FR832526
Gymnosperms	Pinaceae	*Pinus*	*Pinus pinaster*	FR832527
Gymnosperms	Pinaceae	*Pinus*	*Pinus densiflora*	JN046325
Gymnosperms	Pinaceae	*Pinus*	*Pinus densiflora*	JN046326
Gymnosperms	Pinaceae	*Pinus*	*Pinus densiflora*	JN046327
Gymnosperms	Pinaceae	*Pinus*	*Pinus densiflora*	JN046328
Gymnosperms	Pinaceae	*Pinus*	*Pinus densiflora*	JN046329
Gymnosperms	Pinaceae	*Pinus*	*Pinus densiflora*	HQ849861
Gymnosperms	Taxaceae	*Taxus*	*Taxus x media*	DQ888580
Gymnosperms	Taxaceae	*Taxus*	*Taxus x media*	EF660698
Gymnosperms	Taxaceae	*Taxus*	*Taxus canadensis*	EF017304
Gymnosperms	Taxaceae	*Taxus*	*Taxus canadensis*	GQ274315
Gymnosperms	Taxaceae	*Taxus*	*Taxus canadensis*	GQ435017

Gymnosperms	Pinaceae	*Pinus*	*Pinus massoniana*	HQ427086
Gymnosperms	Pinaceae	*Pinus*	*Pinus massoniana*	JN046355
Gymnosperms	Pinaceae	*Pinus*	*Pinus massoniana*	JN046356
Gymnosperms	Pinaceae	*Pinus*	*Pinus massoniana*	JN046357
Gymnosperms	Pinaceae	*Pinus*	*Pinus massoniana*	JN046358
Gymnosperms	Pinaceae	*Pinus*	*Pinus massoniana*	JN046359
Gymnosperms	Pinaceae	*Pinus*	*Pinus massoniana*	HQ849864
Gymnosperms	Pinaceae	*Pinus*	*Pinus tabuliformis*	JN046360
Gymnosperms	Pinaceae	*Pinus*	*Pinus tabuliformis*	JN046361
Gymnosperms	Pinaceae	*Pinus*	*Pinus tabuliformis*	JN046362
Gymnosperms	Pinaceae	*Pinus*	*Pinus tabuliformis*	JN046363
Gymnosperms	Pinaceae	*Pinus*	*Pinus tabuliformis*	JN046364
Gymnosperms	Pinaceae	*Pinus*	*Pinus yunnanensis*	JN046380
Gymnosperms	Pinaceae	*Pinus*	*Pinus yunnanensis*	JN046381
Gymnosperms	Pinaceae	*Pinus*	*Pinus yunnanensis*	JN046382
Gymnosperms	Pinaceae	*Pinus*	*Pinus yunnanensis*	JN046383
Gymnosperms	Pinaceae	*Pinus*	*Pinus yunnanensis*	JN046384
Gymnosperms	Pinaceae	*Pinus*	*Pinus yunnanensis*	HQ849865
Gymnosperms	Pinaceae	*Pinus*	*Pinus armandii*	JN046330
Gymnosperms	Pinaceae	*Pinus*	*Pinus armandii*	JN046331
Gymnosperms	Pinaceae	*Pinus*	*Pinus armandii*	JN046332
Gymnosperms	Pinaceae	*Pinus*	*Pinus armandii*	JN046333
Gymnosperms	Pinaceae	*Pinus*	*Pinus armandii*	JN046334
Gymnosperms	Pinaceae	*Pinus*	*Pinus armandii*	HQ849869
Gymnosperms	Taxaceae	*Amentotaxus*	*Amentotaxus yunnanensis*	GQ435020
Gymnosperms	Taxaceae	*Amentotaxus*	*Amentotaxus yunnanensis*	EF660681
Gymnosperms	Taxaceae	*Amentotaxus*	*Amentotaxus yunnanensis*	JN043922
Gymnosperms	Taxaceae	*Amentotaxus*	*Amentotaxus yunnanensis*	JN043923
Gymnosperms	Pinaceae	*Picea*	*Picea morrisonicola*	HQ114829
Gymnosperms	Pinaceae	*Picea*	*Picea morrisonicola*	HQ114882
Gymnosperms	Pinaceae	*Abies*	*Abies fabri*	JN043665
Gymnosperms	Pinaceae	*Abies*	*Abies fabri*	JN043666
Gymnosperms	Pinaceae	*Abies*	*Abies fabri*	JN043667
Gymnosperms	Pinaceae	*Abies*	*Abies fabri*	JN043668
Gymnosperms	Pinaceae	*Abies*	*Abies fabri*	JN043669
Gymnosperms	Pinaceae	*Abies*	*Abies fabri*	HQ833528
Gymnosperms	Pinaceae	*Abies*	*Abies fabri*	HQ833529
Gymnosperms	Pinaceae	*Abies*	*Abies fabri*	HQ833530
Gymnosperms	Pinaceae	*Abies*	*Abies fabri*	HQ833531
Gymnosperms	Pinaceae	*Abies*	*Abies fargesii*	JN043670
Gymnosperms	Pinaceae	*Abies*	*Abies fargesii*	JN043671
Gymnosperms	Pinaceae	*Abies*	*Abies fargesii*	JN043672
Gymnosperms	Pinaceae	*Abies*	*Abies fargesii*	JN043673
Gymnosperms	Pinaceae	*Abies*	*Abies fargesii*	JN043674
Gymnosperms	Pinaceae	*Abies*	*Abies fargesii*	HQ833540
Gymnosperms	Pinaceae	*Abies*	*Abies fargesii*	HQ833541
Gymnosperms	Pinaceae	*Abies*	*Abies fargesii*	HQ833542
Gymnosperms	Pinaceae	*Abies*	*Abies fargesii*	HQ833543
Gymnosperms	Pinaceae	*Abies*	*Abies holophylla*	JN043688
Gymnosperms	Pinaceae	*Abies*	*Abies holophylla*	JN043689
Gymnosperms	Pinaceae	*Abies*	*Abies holophylla*	JN043690
Gymnosperms	Pinaceae	*Abies*	*Abies holophylla*	JN043691
Gymnosperms	Pinaceae	*Abies*	*Abies holophylla*	JN043692

Gymnosperms	Pinaceae	*Abies*	*Abies holophylla*	HQ833549
Gymnosperms	Pinaceae	*Abies*	*Abies nephrolepis*	JN043693
Gymnosperms	Pinaceae	*Abies*	*Abies nephrolepis*	JN043694
Gymnosperms	Pinaceae	*Abies*	*Abies nephrolepis*	JN043695
Gymnosperms	Pinaceae	*Abies*	*Abies nephrolepis*	JN043696
Gymnosperms	Pinaceae	*Abies*	*Abies nephrolepis*	JN043697
Gymnosperms	Pinaceae	*Abies*	*Abies nephrolepis*	HQ833547
Gymnosperms	Pinaceae	*Abies*	*Abies nephrolepis*	HQ833548
Gymnosperms	Taxaceae	*Taxus*	*Taxus cuspidata*	DQ888579
Gymnosperms	Taxaceae	*Taxus*	*Taxus cuspidata*	GQ435016
Gymnosperms	Taxaceae	*Taxus*	*Taxus cuspidata*	EF660682
Gymnosperms	Taxaceae	*Taxus*	*Taxus cuspidata*	HM591103
Gymnosperms	Taxaceae	*Taxus*	*Taxus cuspidata*	HM591104
Gymnosperms	Taxaceae	*Taxus*	*Taxus cuspidata*	HM591105
Gymnosperms	Pinaceae	*Picea*	*Picea breweriana*	HQ114874
Gymnosperms	Pinaceae	*Picea*	*Picea breweriana*	HQ114879
Gymnosperms	Podocarpaceae	*Podocarpus*	*Podocarpus matudae*	HM036081
Gymnosperms	Podocarpaceae	*Podocarpus*	*Podocarpus matudae*	HM036082
Gymnosperms	Podocarpaceae	*Podocarpus*	*Podocarpus matudae*	HM036083
Gymnosperms	Podocarpaceae	*Podocarpus*	*Podocarpus matudae*	HM036084
Gymnosperms	Podocarpaceae	*Podocarpus*	*Podocarpus matudae*	HM036085
Gymnosperms	Podocarpaceae	*Podocarpus*	*Podocarpus matudae*	HM036086
Gymnosperms	Podocarpaceae	*Podocarpus*	*Podocarpus matudae*	HM036087
Gymnosperms	Podocarpaceae	*Podocarpus*	*Podocarpus matudae*	HM036088
Gymnosperms	Podocarpaceae	*Podocarpus*	*Podocarpus matudae*	HM036089
Gymnosperms	Podocarpaceae	*Podocarpus*	*Podocarpus matudae*	HM036090
Gymnosperms	Podocarpaceae	*Podocarpus*	*Podocarpus matudae*	HM036091
Gymnosperms	Pinaceae	*Picea*	*Picea smithiana*	JN046305
Gymnosperms	Pinaceae	*Picea*	*Picea smithiana*	JN046306
Gymnosperms	Pinaceae	*Picea*	*Picea smithiana*	JN046307
Gymnosperms	Pinaceae	*Picea*	*Picea smithiana*	JN046308
Gymnosperms	Pinaceae	*Picea*	*Picea smithiana*	HQ114859
Gymnosperms	Pinaceae	*Picea*	*Picea smithiana*	HQ114891
Gymnosperms	Pinaceae	*Abies*	*Abies beshanzuensis*	JN043643
Gymnosperms	Pinaceae	*Abies*	*Abies beshanzuensis*	JN043644
Gymnosperms	Pinaceae	*Abies*	*Abies beshanzuensis*	HQ833553
Gymnosperms	Taxaceae	*Taxus*	*Taxus globosa*	GQ463511
Gymnosperms	Taxaceae	*Taxus*	*Taxus globosa*	EF660673
Gymnosperms	Pinaceae	*Picea*	*Picea wilsonii*	GQ463535
Gymnosperms	Pinaceae	*Picea*	*Picea wilsonii*	JN046311
Gymnosperms	Pinaceae	*Picea*	*Picea wilsonii*	JN046312
Gymnosperms	Pinaceae	*Picea*	*Picea wilsonii*	JN046313
Gymnosperms	Pinaceae	*Picea*	*Picea wilsonii*	JN046314
Gymnosperms	Pinaceae	*Picea*	*Picea wilsonii*	HQ114830
Gymnosperms	Pinaceae	*Picea*	*Picea wilsonii*	HQ114871
Gymnosperms	Pinaceae	*Picea*	*Picea asperata*	JN046239
Gymnosperms	Pinaceae	*Picea*	*Picea asperata*	JN046240
Gymnosperms	Pinaceae	*Picea*	*Picea asperata*	JN046241
Gymnosperms	Pinaceae	*Picea*	*Picea asperata*	JN046242
Gymnosperms	Pinaceae	*Picea*	*Picea asperata*	JN046243
Gymnosperms	Pinaceae	*Picea*	*Picea asperata*	HQ114844
Gymnosperms	Pinaceae	*Picea*	*Picea asperata*	HQ114845
Gymnosperms	Pinaceae	*Picea*	*Picea asperata*	HQ114865

Gymnosperms	Pinaceae	*Picea*	*Picea schrenkiana*	JN046300
Gymnosperms	Pinaceae	*Picea*	*Picea schrenkiana*	JN046301
Gymnosperms	Pinaceae	*Picea*	*Picea schrenkiana*	JN046302
Gymnosperms	Pinaceae	*Picea*	*Picea schrenkiana*	JN046303
Gymnosperms	Pinaceae	*Picea*	*Picea schrenkiana*	JN046304
Gymnosperms	Pinaceae	*Picea*	*Picea schrenkiana*	HQ114838
Gymnosperms	Pinaceae	*Picea*	*Picea schrenkiana*	HQ114839
Gymnosperms	Pinaceae	*Picea*	*Picea spinulosa*	JN046309
Gymnosperms	Pinaceae	*Picea*	*Picea spinulosa*	JN046310
Gymnosperms	Pinaceae	*Picea*	*Picea spinulosa*	HQ114878
Gymnosperms	Pinaceae	*Picea*	*Picea spinulosa*	HQ114892
Gymnosperms	Ephedraceae	*Ephedra*	*Ephedra monosperma*	JN044472
Gymnosperms	Ephedraceae	*Ephedra*	*Ephedra monosperma*	JN044473
Gymnosperms	Ephedraceae	*Ephedra*	*Ephedra frustillata*	GQ248296
Gymnosperms	Ephedraceae	*Ephedra*	*Ephedra frustillata*	AY849355
Gymnosperms	Ephedraceae	*Ephedra*	*Ephedra intermedia*	JN044470
Gymnosperms	Ephedraceae	*Ephedra*	*Ephedra intermedia*	JN044471
Gymnosperms	Ephedraceae	*Ephedra*	*Ephedra equisetina*	GQ435179
Gymnosperms	Ephedraceae	*Ephedra*	*Ephedra equisetina*	GQ435180
Gymnosperms	Ephedraceae	*Ephedra*	*Ephedra equisetina*	AY849352
Gymnosperms	Pinaceae	*Pinus*	*Pinus densata*	JN046335
Gymnosperms	Pinaceae	*Pinus*	*Pinus densata*	JN046336
Gymnosperms	Pinaceae	*Pinus*	*Pinus densata*	JN046337
Gymnosperms	Pinaceae	*Pinus*	*Pinus densata*	JN046338
Gymnosperms	Pinaceae	*Pinus*	*Pinus densata*	JN046339
Gymnosperms	Pinaceae	*Pinus*	*Pinus densata*	JN046340
Gymnosperms	Pinaceae	*Pinus*	*Pinus densata*	JN046341
Gymnosperms	Pinaceae	*Pinus*	*Pinus densata*	JN046342
Gymnosperms	Pinaceae	*Pinus*	*Pinus densata*	JN046343
Gymnosperms	Pinaceae	*Pinus*	*Pinus densata*	JN046344
Gymnosperms	Pinaceae	*Pinus*	*Pinus taiwanensis*	JN046365
Gymnosperms	Pinaceae	*Pinus*	*Pinus taiwanensis*	JN046366
Gymnosperms	Pinaceae	*Pinus*	*Pinus taiwanensis*	JN046367
Gymnosperms	Pinaceae	*Pinus*	*Pinus taiwanensis*	JN046368
Gymnosperms	Pinaceae	*Pinus*	*Pinus taiwanensis*	JN046369
Gymnosperms	Ephedraceae	*Ephedra*	*Ephedra regeliana*	JN044474
Gymnosperms	Ephedraceae	*Ephedra*	*Ephedra regeliana*	JN044475
Gymnosperms	Pinaceae	*Picea*	*Picea likiangensis*	HQ114836
Gymnosperms	Pinaceae	*Picea*	*Picea likiangensis*	HQ114837
Gymnosperms	Pinaceae	*Picea*	*Picea likiangensis*	HQ114846
Gymnosperms	Pinaceae	*Picea*	*Picea likiangensis*	HQ114849
Gymnosperms	Pinaceae	*Picea*	*Picea likiangensis*	HQ114850
Gymnosperms	Pinaceae	*Picea*	*Picea likiangensis*	HQ114888
Gymnosperms	Pinaceae	*Picea*	*Picea koraiensis*	JN046263
Gymnosperms	Pinaceae	*Picea*	*Picea koraiensis*	JN046264
Gymnosperms	Pinaceae	*Picea*	*Picea koraiensis*	JN046265
Gymnosperms	Pinaceae	*Picea*	*Picea koraiensis*	JN046266
Gymnosperms	Pinaceae	*Picea*	*Picea koraiensis*	JN046267
Gymnosperms	Pinaceae	*Picea*	*Picea koraiensis*	HQ114843
Gymnosperms	Pinaceae	*Picea*	*Picea meyeri*	GQ463527
Gymnosperms	Pinaceae	*Picea*	*Picea meyeri*	GQ463528
Gymnosperms	Pinaceae	*Picea*	*Picea meyeri*	GQ865744
Gymnosperms	Pinaceae	*Picea*	*Picea meyeri*	JN046282

Gymnosperms	Pinaceae	*Picea*	*Picea meyeri*	JN046283
Gymnosperms	Pinaceae	*Picea*	*Picea meyeri*	JN046284
Gymnosperms	Pinaceae	*Picea*	*Picea meyeri*	JN046285
Gymnosperms	Pinaceae	*Picea*	*Picea meyeri*	JN046286
Gymnosperms	Pinaceae	*Picea*	*Picea meyeri*	HQ114861
Gymnosperms	Pinaceae	*Picea*	*Picea meyeri*	HQ114884
Gymnosperms	Pinaceae	*Picea*	*Picea meyeri*	HQ114885
Gymnosperms	Pinaceae	*Picea*	*Picea meyeri*	HQ114886
Gymnosperms	Pinaceae	*Picea*	*Picea meyeri*	HQ114887
Gymnosperms	Pinaceae	*Picea*	*Picea meyeri*	HQ114889
Gymnosperms	Pinaceae	*Picea*	*Picea brachytyla*	JN046244
Gymnosperms	Pinaceae	*Picea*	*Picea brachytyla*	JN046245
Gymnosperms	Pinaceae	*Picea*	*Picea brachytyla*	JN046246
Gymnosperms	Pinaceae	*Picea*	*Picea brachytyla*	JN046247
Gymnosperms	Pinaceae	*Picea*	*Picea brachytyla*	HQ114847
Gymnosperms	Pinaceae	*Picea*	*Picea brachytyla*	HQ114856
Gymnosperms	Pinaceae	*Picea*	*Picea purpurea*	JN046295
Gymnosperms	Pinaceae	*Picea*	*Picea purpurea*	JN046296
Gymnosperms	Pinaceae	*Picea*	*Picea purpurea*	JN046297
Gymnosperms	Pinaceae	*Picea*	*Picea purpurea*	JN046298
Gymnosperms	Pinaceae	*Picea*	*Picea purpurea*	JN046299
Gymnosperms	Pinaceae	*Picea*	*Picea purpurea*	HQ114851
Gymnosperms	Pinaceae	*Picea*	*Picea purpurea*	HQ114852
Gymnosperms	Pinaceae	*Picea*	*Picea purpurea*	HQ114853
Gymnosperms	Pinaceae	*Picea*	*Picea crassifolia*	JN046253
Gymnosperms	Pinaceae	*Picea*	*Picea crassifolia*	JN046254
Gymnosperms	Pinaceae	*Picea*	*Picea crassifolia*	JN046255
Gymnosperms	Pinaceae	*Picea*	*Picea crassifolia*	JN046256
Gymnosperms	Pinaceae	*Picea*	*Picea crassifolia*	JN046257
Gymnosperms	Pinaceae	*Picea*	*Picea crassifolia*	HQ114890
Gymnosperms	Pinaceae	*Picea*	*Picea obovata*	JN046290
Gymnosperms	Pinaceae	*Picea*	*Picea obovata*	JN046291
Gymnosperms	Pinaceae	*Picea*	*Picea obovata*	JN046292
Gymnosperms	Pinaceae	*Picea*	*Picea obovata*	JN046293
Gymnosperms	Pinaceae	*Picea*	*Picea obovata*	JN046294
Gymnosperms	Pinaceae	*Picea*	*Picea obovata*	HQ114840
Gymnosperms	Pinaceae	*Picea*	*Picea obovata*	HQ114841
Gymnosperms	Pinaceae	*Picea*	*Picea obovata*	HQ114842
Gymnosperms	Pinaceae	*Picea*	*Picea obovata*	HQ114883
Gymnosperms	Pinaceae	*Abies*	*Abies spectabilis*	JN043703
Gymnosperms	Pinaceae	*Abies*	*Abies spectabilis*	JN043704
Gymnosperms	Pinaceae	*Abies*	*Abies spectabilis*	JN043705
Gymnosperms	Pinaceae	*Abies*	*Abies spectabilis*	JN043706
Gymnosperms	Pinaceae	*Abies*	*Abies spectabilis*	JN043707
Gymnosperms	Pinaceae	*Abies*	*Abies spectabilis*	HQ833523
Gymnosperms	Pinaceae	*Abies*	*Abies spectabilis*	HQ833524
Gymnosperms	Pinaceae	*Picea*	*Picea neoveitchii*	JN046287
Gymnosperms	Pinaceae	*Picea*	*Picea neoveitchii*	JN046288
Gymnosperms	Pinaceae	*Picea*	*Picea neoveitchii*	JN046289
Gymnosperms	Pinaceae	*Picea*	*Picea neoveitchii*	HQ114870
Gymnosperms	Pinaceae	*Picea*	*Picea farreri*	JN046258
Gymnosperms	Pinaceae	*Picea*	*Picea farreri*	JN046259
Gymnosperms	Pinaceae	*Picea*	*Picea farreri*	HQ114831

Gymnosperms	Pinaceae	*Picea*	*Picea farreri*	HQ114872
Gymnosperms	Pinaceae	*Picea*	*Picea farreri*	HQ114873
Gymnosperms	Pinaceae	*Picea*	*Picea farreri*	HQ114880
Gymnosperms	Pinaceae	*Abies*	*Abies chensiensis*	JN043645
Gymnosperms	Pinaceae	*Abies*	*Abies chensiensis*	JN043646
Gymnosperms	Pinaceae	*Abies*	*Abies chensiensis*	JN043647
Gymnosperms	Pinaceae	*Abies*	*Abies chensiensis*	JN043648
Gymnosperms	Pinaceae	*Abies*	*Abies chensiensis*	JN043649
Gymnosperms	Pinaceae	*Abies*	*Abies chensiensis*	HQ833544
Gymnosperms	Pinaceae	*Abies*	*Abies chensiensis*	HQ833545
Gymnosperms	Pinaceae	*Abies*	*Abies chensiensis*	HQ833546
Gymnosperms	Pinaceae	*Abies*	*Abies delavayi*	JN043650
Gymnosperms	Pinaceae	*Abies*	*Abies delavayi*	JN043651
Gymnosperms	Pinaceae	*Abies*	*Abies delavayi*	JN043652
Gymnosperms	Pinaceae	*Abies*	*Abies delavayi*	JN043653
Gymnosperms	Pinaceae	*Abies*	*Abies delavayi*	JN043654
Gymnosperms	Pinaceae	*Abies*	*Abies delavayi*	HQ833557
Gymnosperms	Pinaceae	*Abies*	*Abies delavayi*	HQ833558
Gymnosperms	Pinaceae	*Abies*	*Abies densa*	JN043655
Gymnosperms	Pinaceae	*Abies*	*Abies densa*	JN043656
Gymnosperms	Pinaceae	*Abies*	*Abies densa*	JN043657
Gymnosperms	Pinaceae	*Abies*	*Abies densa*	JN043658
Gymnosperms	Pinaceae	*Abies*	*Abies densa*	JN043659
Gymnosperms	Pinaceae	*Abies*	*Abies densa*	HQ833555
Gymnosperms	Pinaceae	*Abies*	*Abies densa*	HQ833556
Gymnosperms	Pinaceae	*Abies*	*Abies forrestii*	JN043678
Gymnosperms	Pinaceae	*Abies*	*Abies forrestii*	JN043679
Gymnosperms	Pinaceae	*Abies*	*Abies forrestii*	JN043680
Gymnosperms	Pinaceae	*Abies*	*Abies forrestii*	JN043681
Gymnosperms	Pinaceae	*Abies*	*Abies forrestii*	JN043682
Gymnosperms	Pinaceae	*Abies*	*Abies forrestii*	HQ833532
Gymnosperms	Pinaceae	*Abies*	*Abies forrestii*	HQ833533
Gymnosperms	Pinaceae	*Abies*	*Abies forrestii*	HQ833534
Gymnosperms	Pinaceae	*Abies*	*Abies forrestii*	HQ833535
Gymnosperms	Pinaceae	*Abies*	*Abies ziyuanensis*	JN043627
Gymnosperms	Pinaceae	*Abies*	*Abies ziyuanensis*	JN043628
Gymnosperms	Pinaceae	*Abies*	*Abies ziyuanensis*	JN043629
Gymnosperms	Pinaceae	*Abies*	*Abies ziyuanensis*	JN043630
Gymnosperms	Pinaceae	*Abies*	*Abies ziyuanensis*	JN043631
Gymnosperms	Pinaceae	*Abies*	*Abies ziyuanensis*	HQ833551
Gymnosperms	Pinaceae	*Abies*	*Abies ziyuanensis*	HQ833552
Gymnosperms	Taxaceae	*Taxus*	*Taxus fuana*	GQ435018
Gymnosperms	Taxaceae	*Taxus*	*Taxus fuana*	EF660685
Gymnosperms	Taxaceae	*Taxus*	*Taxus fuana*	HM591106
Gymnosperms	Taxaceae	*Taxus*	*Taxus fuana*	HM591107
Gymnosperms	Taxaceae	*Taxus*	*Taxus fuana*	HM591108
Gymnosperms	Taxaceae	*Taxus*	*Taxus fuana*	HM591109
Gymnosperms	Taxaceae	*Taxus*	*Taxus fuana*	HM591110
Gymnosperms	Taxaceae	*Taxus*	*Taxus sumatrana*	GQ463509
Gymnosperms	Taxaceae	*Taxus*	*Taxus sumatrana*	EF660672
Gymnosperms	Taxaceae	*Taxus*	*Taxus sumatrana*	HM591098
Gymnosperms	Taxaceae	*Taxus*	*Taxus sumatrana*	HM591099
Gymnosperms	Taxaceae	*Taxus*	*Taxus sumatrana*	HM591100

Gymnosperms	Taxaceae	*Taxus*	*Taxus sumatrana*	HM591101
Gymnosperms	Taxaceae	*Taxus*	*Taxus sumatrana*	HM591102
Gymnosperms	Pinaceae	*Abies*	*Abies fanjingshanensis*	JN043640
Gymnosperms	Pinaceae	*Abies*	*Abies fanjingshanensis*	JN043641
Gymnosperms	Pinaceae	*Abies*	*Abies fanjingshanensis*	JN043642
Gymnosperms	Pinaceae	*Abies*	*Abies fanjingshanensis*	HQ833550
Gymnosperms	Pinaceae	*Abies*	*Abies yuanbaoshanensis*	JN043632
Gymnosperms	Pinaceae	*Abies*	*Abies yuanbaoshanensis*	JN043633
Gymnosperms	Pinaceae	*Abies*	*Abies yuanbaoshanensis*	JN043634
Gymnosperms	Pinaceae	*Abies*	*Abies yuanbaoshanensis*	HQ833554
Gymnosperms	Pinaceae	*Pinus*	*Pinus latteri*	JN046350
Gymnosperms	Pinaceae	*Pinus*	*Pinus latteri*	JN046351
Gymnosperms	Pinaceae	*Pinus*	*Pinus latteri*	JN046352
Gymnosperms	Pinaceae	*Pinus*	*Pinus latteri*	JN046353
Gymnosperms	Pinaceae	*Pinus*	*Pinus latteri*	JN046354
Gymnosperms	Pinaceae	*Pinus*	*Pinus latteri*	HQ849866
Gymnosperms	Pinaceae	*Pinus*	*Pinus latteri*	HQ849867
Gymnosperms	Pinaceae	*Pinus*	*Pinus latteri*	HQ849868
Gymnosperms	Pinaceae	*Abies*	*Abies ernestii*	JN043660
Gymnosperms	Pinaceae	*Abies*	*Abies ernestii*	JN043661
Gymnosperms	Pinaceae	*Abies*	*Abies ernestii*	JN043662
Gymnosperms	Pinaceae	*Abies*	*Abies ernestii*	JN043663
Gymnosperms	Pinaceae	*Abies*	*Abies ernestii*	JN043664
Gymnosperms	Pinaceae	*Abies*	*Abies ferreana*	JN043675
Gymnosperms	Pinaceae	*Abies*	*Abies ferreana*	JN043676
Gymnosperms	Pinaceae	*Abies*	*Abies ferreana*	JN043677
Gymnosperms	Pinaceae	*Abies*	*Abies ferreana*	HQ833559
Gymnosperms	Pinaceae	*Abies*	*Abies georgei*	JN043683
Gymnosperms	Pinaceae	*Abies*	*Abies georgei*	JN043684
Gymnosperms	Pinaceae	*Abies*	*Abies georgei*	JN043685
Gymnosperms	Pinaceae	*Abies*	*Abies georgei*	JN043686
Gymnosperms	Pinaceae	*Abies*	*Abies georgei*	JN043687
Gymnosperms	Pinaceae	*Abies*	*Abies georgei*	HQ833521
Gymnosperms	Pinaceae	*Abies*	*Abies georgei*	HQ833522
Gymnosperms	Pinaceae	*Abies*	*Abies recurvata*	JN043698
Gymnosperms	Pinaceae	*Abies*	*Abies recurvata*	JN043699
Gymnosperms	Pinaceae	*Abies*	*Abies recurvata*	JN043700
Gymnosperms	Pinaceae	*Abies*	*Abies recurvata*	JN043701
Gymnosperms	Pinaceae	*Abies*	*Abies recurvata*	JN043702
Gymnosperms	Pinaceae	*Abies*	*Abies recurvata*	HQ833516
Gymnosperms	Pinaceae	*Abies*	*Abies recurvata*	HQ833517
Gymnosperms	Pinaceae	*Abies*	*Abies squamata*	JN043708
Gymnosperms	Pinaceae	*Abies*	*Abies squamata*	JN043709
Gymnosperms	Pinaceae	*Abies*	*Abies squamata*	JN043710
Gymnosperms	Pinaceae	*Abies*	*Abies squamata*	JN043711
Gymnosperms	Pinaceae	*Abies*	*Abies squamata*	JN043712
Gymnosperms	Pinaceae	*Abies*	*Abies squamata*	HQ833518
Gymnosperms	Pinaceae	*Abies*	*Abies squamata*	HQ833519
Gymnosperms	Pinaceae	*Abies*	*Abies squamata*	HQ833520
Gymnosperms	Taxaceae	*Amentotaxus*	*Amentotaxus poilanei*	JN043918
Gymnosperms	Taxaceae	*Amentotaxus*	*Amentotaxus poilanei*	JN043919
Gymnosperms	Taxaceae	*Amentotaxus*	*Amentotaxus poilanei*	JN043920
Gymnosperms	Taxaceae	*Amentotaxus*	*Amentotaxus poilanei*	JN043921

Gymnosperms	Pinaceae	*Abies*	*Abies sp. LiujqAB21*	HQ833525
Gymnosperms	Pinaceae	*Abies*	*Abies sp. LiujqAB21*	HQ833526
Gymnosperms	Pinaceae	*Abies*	*Abies sp. LiujqAB21*	HQ833527
Gymnosperms	Pinaceae	*Abies*	*Abies nukiangensis*	JN043635
Gymnosperms	Pinaceae	*Abies*	*Abies nukiangensis*	JN043636
Gymnosperms	Pinaceae	*Abies*	*Abies nukiangensis*	JN043637
Gymnosperms	Pinaceae	*Abies*	*Abies nukiangensis*	JN043638
Gymnosperms	Pinaceae	*Abies*	*Abies nukiangensis*	JN043639
Gymnosperms	Pinaceae	*Abies*	*Abies nukiangensis*	HQ833536
Gymnosperms	Pinaceae	*Abies*	*Abies nukiangensis*	HQ833537
Gymnosperms	Pinaceae	*Abies*	*Abies nukiangensis*	HQ833538
Gymnosperms	Pinaceae	*Abies*	*Abies nukiangensis*	HQ833539
Gymnosperms	Cephalotaxaceae	*Cephalotaxus*	*Cephalotaxus wilsoniana*	GQ435300
Gymnosperms	Cephalotaxaceae	*Cephalotaxus*	*Cephalotaxus wilsoniana*	EF660674
Gymnosperms	Cephalotaxaceae	*Cephalotaxus*	*Cephalotaxus wilsoniana*	GQ865742
Gymnosperms	Taxaceae	*Torreya*	*Torreya nucifera*	GQ463520
Gymnosperms	Taxaceae	*Torreya*	*Torreya nucifera*	EF660697
Gymnosperms	Cephalotaxaceae	*Cephalotaxus*	*Cephalotaxus harringtonia*	GQ463523
Gymnosperms	Cephalotaxaceae	*Cephalotaxus*	*Cephalotaxus harringtonia*	EF660677
Gymnosperms	Cephalotaxaceae	*Cephalotaxus*	*Cephalotaxus harringtonia*	EF660689
Gymnosperms	Cephalotaxaceae	*Cephalotaxus*	*Cephalotaxus harringtonia*	GQ865743
Gymnosperms	Taxaceae	*Torreya*	*Torreya grandis*	GQ435473
Gymnosperms	Taxaceae	*Torreya*	*Torreya grandis*	GQ463537
Gymnosperms	Taxaceae	*Torreya*	*Torreya grandis*	EF660692
Gymnosperms	Cephalotaxaceae	*Cephalotaxus*	*Cephalotaxus fortunei*	EF660695
Gymnosperms	Cephalotaxaceae	*Cephalotaxus*	*Cephalotaxus fortunei*	JN044237
Gymnosperms	Cephalotaxaceae	*Cephalotaxus*	*Cephalotaxus fortunei*	JN044238
Gymnosperms	Cephalotaxaceae	*Cephalotaxus*	*Cephalotaxus fortunei*	JN044239
Gymnosperms	Cephalotaxaceae	*Cephalotaxus*	*Cephalotaxus fortunei*	JN044240
Gymnosperms	Cephalotaxaceae	*Cephalotaxus*	*Cephalotaxus fortunei*	JN044241
Gymnosperms	Cephalotaxaceae	*Cephalotaxus*	*Cephalotaxus fortunei*	JN044242
Gymnosperms	Cephalotaxaceae	*Cephalotaxus*	*Cephalotaxus fortunei*	JN044243
Gymnosperms	Taxaceae	*Torreya*	*Torreya californica*	GQ435299
Gymnosperms	Taxaceae	*Torreya*	*Torreya californica*	EF660699
Gymnosperms	Cephalotaxaceae	*Cephalotaxus*	*Cephalotaxus mannii*	GQ463521
Gymnosperms	Cephalotaxaceae	*Cephalotaxus*	*Cephalotaxus mannii*	EF660675
Gymnosperms	Cephalotaxaceae	*Cephalotaxus*	*Cephalotaxus mannii*	JN044249
Gymnosperms	Cephalotaxaceae	*Cephalotaxus*	*Cephalotaxus mannii*	JN044250
Gymnosperms	Cephalotaxaceae	*Cephalotaxus*	*Cephalotaxus mannii*	JN044251
Gymnosperms	Cephalotaxaceae	*Cephalotaxus*	*Cephalotaxus mannii*	JN044252
Gymnosperms	Cephalotaxaceae	*Cephalotaxus*	*Cephalotaxus mannii*	JN044253
Gymnosperms	Cephalotaxaceae	*Cephalotaxus*	*Cephalotaxus mannii*	JN044254
Gymnosperms	Cephalotaxaceae	*Cephalotaxus*	*Cephalotaxus mannii*	JN044255
Gymnosperms	Cephalotaxaceae	*Cephalotaxus*	*Cephalotaxus mannii*	JN044256
Gymnosperms	Cephalotaxaceae	*Cephalotaxus*	*Cephalotaxus mannii*	JN044257
Gymnosperms	Cephalotaxaceae	*Cephalotaxus*	*Cephalotaxus sinensis*	EF660687
Gymnosperms	Cephalotaxaceae	*Cephalotaxus*	*Cephalotaxus sinensis*	JN044260
Gymnosperms	Cephalotaxaceae	*Cephalotaxus*	*Cephalotaxus sinensis*	JN044261
Gymnosperms	Cephalotaxaceae	*Cephalotaxus*	*Cephalotaxus sinensis*	JN044262
Gymnosperms	Cephalotaxaceae	*Cephalotaxus*	*Cephalotaxus sinensis*	JN044263
Gymnosperms	Cephalotaxaceae	*Cephalotaxus*	*Cephalotaxus sinensis*	JN044264
Gymnosperms	Cephalotaxaceae	*Cephalotaxus*	*Cephalotaxus sinensis*	JN044265
Gymnosperms	Cephalotaxaceae	*Cephalotaxus*	*Cephalotaxus sinensis*	JN044266

Gymnosperms	Cephalotaxaceae	*Cephalotaxus*	*Cephalotaxus sinensis*	JN044267
Gymnosperms	Cephalotaxaceae	*Cephalotaxus*	*Cephalotaxus sinensis*	JN044268
Gymnosperms	Cephalotaxaceae	*Cephalotaxus*	*Cephalotaxus sinensis*	JN044269
Gymnosperms	Cephalotaxaceae	*Cephalotaxus*	*Cephalotaxus sinensis*	JN044270
Gymnosperms	Cephalotaxaceae	*Cephalotaxus*	*Cephalotaxus sinensis*	JN044271
Gymnosperms	Taxaceae	*Torreya*	*Torreya fargesii*	EF660694
Gymnosperms	Taxaceae	*Torreya*	*Torreya fargesii*	JN047455
Gymnosperms	Taxaceae	*Torreya*	*Torreya fargesii*	JN047456
Gymnosperms	Cephalotaxaceae	*Cephalotaxus*	*Cephalotaxus oliveri*	EF660701
Gymnosperms	Cephalotaxaceae	*Cephalotaxus*	*Cephalotaxus oliveri*	JN044258
Gymnosperms	Cephalotaxaceae	*Cephalotaxus*	*Cephalotaxus oliveri*	JN044259
Gymnosperms	Cephalotaxaceae	*Cephalotaxus*	*Cephalotaxus hainanensis*	GQ435297
Gymnosperms	Cephalotaxaceae	*Cephalotaxus*	*Cephalotaxus hainanensis*	GQ435298
Gymnosperms	Cephalotaxaceae	*Cephalotaxus*	*Cephalotaxus hainanensis*	EF660688
Gymnosperms	Cephalotaxaceae	*Cephalotaxus*	*Cephalotaxus hainanensis*	GQ865736
Gymnosperms	Cephalotaxaceae	*Cephalotaxus*	*Cephalotaxus hainanensis*	GQ865737
Gymnosperms	Cephalotaxaceae	*Cephalotaxus*	*Cephalotaxus hainanensis*	GQ865738
Gymnosperms	Cephalotaxaceae	*Cephalotaxus*	*Cephalotaxus hainanensis*	GQ865739
Gymnosperms	Cephalotaxaceae	*Cephalotaxus*	*Cephalotaxus hainanensis*	GQ865740
Gymnosperms	Cephalotaxaceae	*Cephalotaxus*	*Cephalotaxus hainanensis*	GQ865741
Gymnosperms	Cephalotaxaceae	*Cephalotaxus*	*Cephalotaxus drupacea*	GQ435302
Gymnosperms	Cephalotaxaceae	*Cephalotaxus*	*Cephalotaxus drupacea*	EF660684
Gymnosperms	Cephalotaxaceae	*Cephalotaxus*	*Cephalotaxus griffithii*	GQ463522
Gymnosperms	Cephalotaxaceae	*Cephalotaxus*	*Cephalotaxus griffithii*	EF660669
Gymnosperms	Cephalotaxaceae	*Cephalotaxus*	*Cephalotaxus lanceolata*	GQ463510
Gymnosperms	Cephalotaxaceae	*Cephalotaxus*	*Cephalotaxus lanceolata*	EF660676
Gymnosperms	Cephalotaxaceae	*Cephalotaxus*	*Cephalotaxus lanceolata*	JN044247
Gymnosperms	Cephalotaxaceae	*Cephalotaxus*	*Cephalotaxus lanceolata*	JN044248
Monocotyledons	Alismataceae	*Echinodorus*	*Echinodorus cordifolius*	HM367302
Monocotyledons	Alismataceae	*Echinodorus*	*Echinodorus cordifolius*	HM367303
Monocotyledons	Alismataceae	*Echinodorus*	*Echinodorus cordifolius*	HM367311
Monocotyledons	Alismataceae	*Echinodorus*	*Echinodorus cordifolius*	HM367315
Monocotyledons	Alismataceae	*Echinodorus*	*Echinodorus cordifolius*	HM367317
Monocotyledons	Alismataceae	*Echinodorus*	*Echinodorus cordifolius*	HM367318
Monocotyledons	Colchicaceae	*Colchicum*	*Colchicum speciosum*	JF934161
Monocotyledons	Colchicaceae	*Colchicum*	*Colchicum speciosum*	JF934162
Monocotyledons	Colchicaceae	*Colchicum*	*Colchicum speciosum*	JF934163
Monocotyledons	Juncaceae	*Juncus*	*Juncus effusus*	HQ596739
Monocotyledons	Juncaceae	*Juncus*	*Juncus effusus*	GQ434955
Monocotyledons	Juncaceae	*Juncus*	*Juncus effusus*	GQ434956
Monocotyledons	Alismataceae	*Alisma*	*Alisma plantago-aquatica*	GQ435456
Monocotyledons	Alismataceae	*Alisma*	*Alisma plantago-aquatica*	JN043778
Monocotyledons	Alismataceae	*Alisma*	*Alisma plantago-aquatica*	JN043779
Monocotyledons	Alismataceae	*Alisma*	*Alisma plantago-aquatica*	JN043780
Monocotyledons	Alismataceae	*Alisma*	*Alisma plantago-aquatica*	JN043781
Monocotyledons	Alismataceae	*Alisma*	*Alisma plantago-aquatica*	JN043782
Monocotyledons	Alismataceae	*Alisma*	*Alisma plantago-aquatica*	JN043783
Monocotyledons	Alismataceae	*Alisma*	*Alisma plantago-aquatica*	JN043784
Monocotyledons	Alismataceae	*Alisma*	*Alisma plantago-aquatica*	JN043785
Monocotyledons	Alismataceae	*Alisma*	*Alisma plantago-aquatica*	JN043786
Monocotyledons	Potamogetonaceae	*Potamogeton*	*Potamogeton amplifolius*	DQ786563
Monocotyledons	Potamogetonaceae	*Potamogeton*	*Potamogeton amplifolius*	DQ786564
Monocotyledons	Potamogetonaceae	*Potamogeton*	*Potamogeton richardsonii*	DQ786549

Monocotyledons	Potamogetonaceae	*Potamogeton*	*Potamogeton richardsonii*	DQ786550
Monocotyledons	Potamogetonaceae	*Potamogeton*	*Potamogeton richardsonii*	DQ786551
Monocotyledons	Arecaceae	*Sabal*	*Sabal minor*	GQ248388
Monocotyledons	Arecaceae	*Sabal*	*Sabal minor*	EF688519
Monocotyledons	Asparagaceae	*Polygonatum*	*Polygonatum humile*	JN046415
Monocotyledons	Asparagaceae	*Polygonatum*	*Polygonatum humile*	JN046416
Monocotyledons	Asparagaceae	*Polygonatum*	*Polygonatum humile*	JN046417
Monocotyledons	Asparagaceae	*Polygonatum*	*Polygonatum humile*	JN046418
Monocotyledons	Asparagaceae	*Maianthemum*	*Maianthemum racemosum*	HQ596763
Monocotyledons	Asparagaceae	*Maianthemum*	*Maianthemum racemosum*	EU850237
Monocotyledons	Asparagaceae	*Maianthemum*	*Maianthemum racemosum*	EU850238
Monocotyledons	Asparagaceae	*Maianthemum*	*Maianthemum racemosum*	JN045467
Monocotyledons	Asparagaceae	*Maianthemum*	*Maianthemum racemosum*	JN045468
Monocotyledons	Colchicaceae	*Colchicum*	*Colchicum autumnale*	FR865109
Monocotyledons	Colchicaceae	*Colchicum*	*Colchicum autumnale*	FN675817
Monocotyledons	Colchicaceae	*Colchicum*	*Colchicum autumnale*	JF934068
Monocotyledons	Colchicaceae	*Colchicum*	*Colchicum autumnale*	JF934069
Monocotyledons	Colchicaceae	*Colchicum*	*Colchicum autumnale*	JF934070
Monocotyledons	Bromeliaceae	*Aechmea*	*Aechmea allenii*	EF110654
Monocotyledons	Bromeliaceae	*Aechmea*	*Aechmea allenii*	HQ913722
Monocotyledons	Poaceae	*Elymus*	*Elymus repens*	FJ395519
Monocotyledons	Poaceae	*Elymus*	*Elymus repens*	HQ596680
Monocotyledons	Poaceae	*Elymus*	*Elymus repens*	EU531722
Monocotyledons	Poaceae	*Stipa*	*Stipa parviflora*	EU204777
Monocotyledons	Poaceae	*Stipa*	*Stipa parviflora*	EU204778
Monocotyledons	Poaceae	*Elymus*	*Elymus sibiricus*	HQ221837
Monocotyledons	Poaceae	*Elymus*	*Elymus sibiricus*	HQ221842
Monocotyledons	Alismataceae	*Echinodorus*	*Echinodorus grandiflorus*	HM367294
Monocotyledons	Alismataceae	*Echinodorus*	*Echinodorus grandiflorus*	HM367297
Monocotyledons	Alismataceae	*Echinodorus*	*Echinodorus grandiflorus*	HM367309
Monocotyledons	Cymodoceaceae	*Halodule*	*Halodule pinifolia*	AB571183
Monocotyledons	Cymodoceaceae	*Halodule*	*Halodule pinifolia*	AB571184
Monocotyledons	Cymodoceaceae	*Halodule*	*Halodule pinifolia*	AB571185
Monocotyledons	Cymodoceaceae	*Halodule*	*Halodule pinifolia*	AB571186
Monocotyledons	Cymodoceaceae	*Halodule*	*Halodule pinifolia*	AB571187
Monocotyledons	Cymodoceaceae	*Halodule*	*Halodule pinifolia*	AB571188
Monocotyledons	Cymodoceaceae	*Halodule*	*Halodule pinifolia*	AB571189
Monocotyledons	Cymodoceaceae	*Halodule*	*Halodule pinifolia*	AB571190
Monocotyledons	Hydrocharitaceae	*Najas*	*Najas marina*	HQ687180
Monocotyledons	Hydrocharitaceae	*Najas*	*Najas marina*	HQ687181
Monocotyledons	Hydrocharitaceae	*Najas*	*Najas marina*	HQ687182
Monocotyledons	Hydrocharitaceae	*Najas*	*Najas marina*	HQ687183
Monocotyledons	Potamogetonaceae	*Potamogeton*	*Potamogeton crispus*	DQ786527
Monocotyledons	Potamogetonaceae	*Potamogeton*	*Potamogeton crispus*	DQ786528
Monocotyledons	Potamogetonaceae	*Potamogeton*	*Potamogeton gramineus*	DQ786534
Monocotyledons	Potamogetonaceae	*Potamogeton*	*Potamogeton gramineus*	EF174574
Monocotyledons	Potamogetonaceae	*Potamogeton*	*Potamogeton gramineus*	FN668445
Monocotyledons	Poaceae	*Zizania*	*Zizania latifolia*	GU177392
Monocotyledons	Poaceae	*Zizania*	*Zizania latifolia*	GU177393
Monocotyledons	Hydrocharitaceae	*Najas*	*Najas minor*	HQ687184
Monocotyledons	Hydrocharitaceae	*Najas*	*Najas minor*	HQ687185
Monocotyledons	Hydrocharitaceae	*Najas*	*Najas minor*	HQ687186
Monocotyledons	Hydrocharitaceae	*Najas*	*Najas minor*	HQ687187

Monocotyledons	Poaceae	*Agrostis*	*Agrostis stolonifera*	HQ596586
Monocotyledons	Poaceae	*Agrostis*	*Agrostis stolonifera*	HQ596587
Monocotyledons	Cyperaceae	*Carex*	*Carex laxiflora*	HQ596627
Monocotyledons	Cyperaceae	*Carex*	*Carex laxiflora*	HQ596628
Monocotyledons	Poaceae	*Zizania*	*Zizania aquatica*	GU177368
Monocotyledons	Poaceae	*Zizania*	*Zizania aquatica*	GU177376
Monocotyledons	Poaceae	*Zizania*	*Zizania aquatica*	GU177377
Monocotyledons	Poaceae	*Zizania*	*Zizania aquatica*	GU177378
Monocotyledons	Poaceae	*Zizania*	*Zizania aquatica*	GU177379
Monocotyledons	Poaceae	*Zizania*	*Zizania aquatica*	GU177380
Monocotyledons	Poaceae	*Zizania*	*Zizania aquatica*	GU177381
Monocotyledons	Poaceae	*Zizania*	*Zizania aquatica*	GU177382
Monocotyledons	Poaceae	*Zizania*	*Zizania aquatica*	GU177386
Monocotyledons	Poaceae	*Zizania*	*Zizania aquatica*	GU177387
Monocotyledons	Poaceae	*Zizania*	*Zizania aquatica*	GU177388
Monocotyledons	Poaceae	*Zizania*	*Zizania aquatica*	GU177390
Monocotyledons	Orchidaceae	*Cymbidium*	*Cymbidium ensifolium*	FJ527765
Monocotyledons	Orchidaceae	*Cymbidium*	*Cymbidium ensifolium*	FJ527766
Monocotyledons	Asparagaceae	*Polygonatum*	*Polygonatum odoratum*	GQ434924
Monocotyledons	Asparagaceae	*Polygonatum*	*Polygonatum odoratum*	GQ434925
Monocotyledons	Potamogetonaceae	*Potamogeton*	*Potamogeton nodosus*	DQ786541
Monocotyledons	Potamogetonaceae	*Potamogeton*	*Potamogeton nodosus*	DQ786565
Monocotyledons	Asparagaceae	*Polygonatum*	*Polygonatum cirrhifolium*	EU850212
Monocotyledons	Asparagaceae	*Polygonatum*	*Polygonatum cirrhifolium*	JN046400
Monocotyledons	Asparagaceae	*Polygonatum*	*Polygonatum cirrhifolium*	JN046401
Monocotyledons	Asparagaceae	*Polygonatum*	*Polygonatum cirrhifolium*	JN046402
Monocotyledons	Asparagaceae	*Polygonatum*	*Polygonatum cirrhifolium*	JN046403
Monocotyledons	Asparagaceae	*Polygonatum*	*Polygonatum cirrhifolium*	JN046404
Monocotyledons	Asparagaceae	*Polygonatum*	*Polygonatum cirrhifolium*	JN046405
Monocotyledons	Asparagaceae	*Polygonatum*	*Polygonatum cirrhifolium*	JN046406
Monocotyledons	Asparagaceae	*Polygonatum*	*Polygonatum cirrhifolium*	JN046407
Monocotyledons	Asparagaceae	*Polygonatum*	*Polygonatum cirrhifolium*	JN046408
Monocotyledons	Asparagaceae	*Polygonatum*	*Polygonatum cirrhifolium*	JN046409
Monocotyledons	Asparagaceae	*Polygonatum*	*Polygonatum cirrhifolium*	JN046410
Monocotyledons	Asparagaceae	*Polygonatum*	*Polygonatum cirrhifolium*	JN046411
Monocotyledons	Asparagaceae	*Polygonatum*	*Polygonatum cirrhifolium*	JN046412
Monocotyledons	Asparagaceae	*Maianthemum*	*Maianthemum japonicum*	GQ434900
Monocotyledons	Asparagaceae	*Maianthemum*	*Maianthemum japonicum*	EU850231
Monocotyledons	Asparagaceae	*Maianthemum*	*Maianthemum japonicum*	EU850232
Monocotyledons	Asparagaceae	*Maianthemum*	*Maianthemum japonicum*	EU850240
Monocotyledons	Asparagaceae	*Maianthemum*	*Maianthemum japonicum*	JN045454
Monocotyledons	Asparagaceae	*Maianthemum*	*Maianthemum japonicum*	JN045455
Monocotyledons	Asparagaceae	*Maianthemum*	*Maianthemum japonicum*	JN045456
Monocotyledons	Asparagaceae	*Maianthemum*	*Maianthemum japonicum*	JN045457
Monocotyledons	Asparagaceae	*Maianthemum*	*Maianthemum japonicum*	JN045458
Monocotyledons	Asparagaceae	*Maianthemum*	*Maianthemum japonicum*	JN045459
Monocotyledons	Poaceae	*Zizania*	*Zizania palustris*	GU177369
Monocotyledons	Poaceae	*Zizania*	*Zizania palustris*	GU177370
Monocotyledons	Poaceae	*Zizania*	*Zizania palustris*	GU177371
Monocotyledons	Poaceae	*Zizania*	*Zizania palustris*	GU177372
Monocotyledons	Poaceae	*Zizania*	*Zizania palustris*	GU177373
Monocotyledons	Poaceae	*Zizania*	*Zizania palustris*	GU177374
Monocotyledons	Poaceae	*Zizania*	*Zizania palustris*	GU177375

Monocotyledons	Poaceae	*Zizania*	*Zizania palustris*	GU177383
Monocotyledons	Poaceae	*Zizania*	*Zizania palustris*	GU177384
Monocotyledons	Poaceae	*Zizania*	*Zizania palustris*	GU177385
Monocotyledons	Poaceae	*Zizania*	*Zizania palustris*	GU177389
Monocotyledons	Bromeliaceae	*Aechmea*	*Aechmea distichantha*	JN204613
Monocotyledons	Bromeliaceae	*Aechmea*	*Aechmea distichantha*	JN204614
Monocotyledons	Zingiberaceae	*Gagnepainia*	*Gagnepainia godefroyi*	GQ386049
Monocotyledons	Zingiberaceae	*Gagnepainia*	*Gagnepainia godefroyi*	GQ386050
Monocotyledons	Arecaceae	*Arenga*	*Arenga hookeriana*	JF345017
Monocotyledons	Arecaceae	*Arenga*	*Arenga hookeriana*	JF345025
Monocotyledons	Arecaceae	*Arenga*	*Arenga hookeriana*	JF345026
Monocotyledons	Arecaceae	*Arenga*	*Arenga hookeriana*	JF345027
Monocotyledons	Arecaceae	*Arenga*	*Arenga hookeriana*	JF345028
Monocotyledons	Arecaceae	*Wallichia*	*Wallichia disticha*	JF345073
Monocotyledons	Arecaceae	*Wallichia*	*Wallichia disticha*	JF345074
Monocotyledons	Orchidaceae	*Cischweinfia*	*Cischweinfia dasyandra*	FJ564133
Monocotyledons	Orchidaceae	*Cischweinfia*	*Cischweinfia dasyandra*	FJ564646
Monocotyledons	Orchidaceae	*Cuitlauzina*	*Cuitlauzina pendula*	FJ564076
Monocotyledons	Orchidaceae	*Cuitlauzina*	*Cuitlauzina pendula*	FJ564664
Monocotyledons	Orchidaceae	*Fernandezia*	*Fernandezia ionanthera*	FJ564101
Monocotyledons	Orchidaceae	*Fernandezia*	*Fernandezia ionanthera*	FJ564527
Monocotyledons	Orchidaceae	*Lockhartia*	*Lockhartia oerstedii*	EU213746
Monocotyledons	Orchidaceae	*Lockhartia*	*Lockhartia oerstedii*	FJ563978
Monocotyledons	Arecaceae	*Arenga*	*Arenga caudata*	JF345007
Monocotyledons	Arecaceae	*Arenga*	*Arenga caudata*	JF345008
Monocotyledons	Arecaceae	*Arenga*	*Arenga caudata*	JF345009
Monocotyledons	Arecaceae	*Arenga*	*Arenga caudata*	JF345010
Monocotyledons	Arecaceae	*Arenga*	*Arenga caudata*	JF345011
Monocotyledons	Arecaceae	*Arenga*	*Arenga caudata*	JF345012
Monocotyledons	Arecaceae	*Arenga*	*Arenga caudata*	JF345013
Monocotyledons	Arecaceae	*Arenga*	*Arenga caudata*	JF345014
Monocotyledons	Arecaceae	*Arenga*	*Arenga caudata*	JF345015
Monocotyledons	Arecaceae	*Arenga*	*Arenga caudata*	JF345016
Monocotyledons	Arecaceae	*Arenga*	*Arenga undulatifolia*	JF345039
Monocotyledons	Arecaceae	*Arenga*	*Arenga undulatifolia*	JF345040
Monocotyledons	Orchidaceae	*Prosthechea*	*Prosthechea cochleata*	EU213757
Monocotyledons	Orchidaceae	*Prosthechea*	*Prosthechea cochleata*	EU213758
Monocotyledons	Arecaceae	*Hyphaene*	*Hyphaene coriacea*	EU213775
Monocotyledons	Arecaceae	*Hyphaene*	*Hyphaene coriacea*	EU213776
Monocotyledons	Arecaceae	*Hyphaene*	*Hyphaene coriacea*	EU213777
Monocotyledons	Arecaceae	*Hyphaene*	*Hyphaene coriacea*	EU213778
Monocotyledons	Orchidaceae	*Rhynchostele*	*Rhynchostele beloglossa*	EU213707
Monocotyledons	Orchidaceae	*Rhynchostele*	*Rhynchostele beloglossa*	FJ564093
Monocotyledons	Orchidaceae	*Rhynchostele*	*Rhynchostele beloglossa*	FJ564134
Monocotyledons	Orchidaceae	*Comparettia*	*Comparettia macroplectron*	FJ564052
Monocotyledons	Orchidaceae	*Comparettia*	*Comparettia macroplectron*	FJ564656
Monocotyledons	Orchidaceae	*Cyrtochilum*	*Cyrtochilum cimiciferum*	FJ563997
Monocotyledons	Orchidaceae	*Cyrtochilum*	*Cyrtochilum cimiciferum*	FJ564239
Monocotyledons	Orchidaceae	*Cyrtochilum*	*Cyrtochilum cimiciferum*	FJ564518
Monocotyledons	Orchidaceae	*Cyrtochilum*	*Cyrtochilum edwardii*	FJ564091
Monocotyledons	Orchidaceae	*Cyrtochilum*	*Cyrtochilum edwardii*	FJ564669
Monocotyledons	Orchidaceae	*Erycina*	*Erycina crista-galli*	EU213751
Monocotyledons	Orchidaceae	*Erycina*	*Erycina crista-galli*	FJ564144

Monocotyledons	Orchidaceae	*Erycina*	*Erycina echinata*	FJ564063
Monocotyledons	Orchidaceae	*Erycina*	*Erycina echinata*	FJ564434
Monocotyledons	Orchidaceae	*Erycina*	*Erycina hyalinobulbon*	FJ564026
Monocotyledons	Orchidaceae	*Erycina*	*Erycina hyalinobulbon*	FJ564435
Monocotyledons	Orchidaceae	*Erycina*	*Erycina pumilio*	EU213737
Monocotyledons	Orchidaceae	*Erycina*	*Erycina pumilio*	EU213738
Monocotyledons	Orchidaceae	*Erycina*	*Erycina pumilio*	FJ564149
Monocotyledons	Orchidaceae	*Erycina*	*Erycina pumilio*	FJ564556
Monocotyledons	Orchidaceae	*Erycina*	*Erycina pusilla*	FJ564021
Monocotyledons	Orchidaceae	*Erycina*	*Erycina pusilla*	FJ564542
Monocotyledons	Orchidaceae	*Ionopsis*	*Ionopsis minutiflora*	FJ564136
Monocotyledons	Orchidaceae	*Ionopsis*	*Ionopsis minutiflora*	FJ564462
Monocotyledons	Orchidaceae	*Ionopsis*	*Ionopsis minutiflora*	FJ564565
Monocotyledons	Orchidaceae	*Ionopsis*	*Ionopsis satyrioides*	FJ564135
Monocotyledons	Orchidaceae	*Ionopsis*	*Ionopsis satyrioides*	FJ564266
Monocotyledons	Orchidaceae	*Ionopsis*	*Ionopsis utricularioides*	FJ564022
Monocotyledons	Orchidaceae	*Ionopsis*	*Ionopsis utricularioides*	FJ564559
Monocotyledons	Orchidaceae	*Gomesa*	*Gomesa flexuosa*	FJ564049
Monocotyledons	Orchidaceae	*Gomesa*	*Gomesa flexuosa*	FJ564546
Monocotyledons	Orchidaceae	*Gomesa*	*Gomesa flexuosa*	FJ564662
Monocotyledons	Orchidaceae	*Rhynchostele*	*Rhynchostele bictoniensis*	GQ248385
Monocotyledons	Orchidaceae	*Rhynchostele*	*Rhynchostele bictoniensis*	EU213764
Monocotyledons	Orchidaceae	*Rhynchostele*	*Rhynchostele bictoniensis*	FJ564004
Monocotyledons	Orchidaceae	*Tolumnia*	*Tolumnia calochila*	FJ564087
Monocotyledons	Orchidaceae	*Tolumnia*	*Tolumnia calochila*	FJ564232
Monocotyledons	Orchidaceae	*Trichopilia*	*Trichopilia subulata*	FJ564073
Monocotyledons	Orchidaceae	*Trichopilia*	*Trichopilia subulata*	FJ564550
Monocotyledons	Orchidaceae	*Cyrtochilum*	*Cyrtochilum angustatum*	FJ564068
Monocotyledons	Orchidaceae	*Cyrtochilum*	*Cyrtochilum angustatum*	FJ564260
Monocotyledons	Orchidaceae	*Cyrtochilum*	*Cyrtochilum pardinum*	FJ564092
Monocotyledons	Orchidaceae	*Cyrtochilum*	*Cyrtochilum pardinum*	FJ564170
Monocotyledons	Orchidaceae	*Cyrtochilum*	*Cyrtochilum ramosissimum*	FJ564056
Monocotyledons	Orchidaceae	*Cyrtochilum*	*Cyrtochilum ramosissimum*	FJ564483
Monocotyledons	Orchidaceae	*Cyrtochilum*	*Cyrtochilum tricostatum*	FJ564086
Monocotyledons	Orchidaceae	*Cyrtochilum*	*Cyrtochilum tricostatum*	FJ564102
Monocotyledons	Orchidaceae	*Cyrtochilum*	*Cyrtochilum tricostatum*	FJ564151
Monocotyledons	Orchidaceae	*Cyrtochilum*	*Cyrtochilum tricostatum*	FJ564243
Monocotyledons	Orchidaceae	*Cyrtochilum*	*Cyrtochilum tricostatum*	FJ564247
Monocotyledons	Orchidaceae	*Cyrtochilum*	*Cyrtochilum tricostatum*	FJ564486
Monocotyledons	Orchidaceae	*Miltoniopsis*	*Miltoniopsis vexillaria*	FJ564060
Monocotyledons	Orchidaceae	*Miltoniopsis*	*Miltoniopsis vexillaria*	FJ564341
Monocotyledons	Araceae	*Wolffia*	*Wolffia australiana*	GU454554
Monocotyledons	Araceae	*Wolffia*	*Wolffia australiana*	GU454555
Monocotyledons	Araceae	*Wolffia*	*Wolffia brasiliensis*	GU454557
Monocotyledons	Araceae	*Wolffia*	*Wolffia brasiliensis*	GU454558
Monocotyledons	Araceae	*Wolffia*	*Wolffia brasiliensis*	GU454559
Monocotyledons	Araceae	*Wolffia*	*Wolffia columbiana*	GU454560
Monocotyledons	Araceae	*Wolffia*	*Wolffia columbiana*	GU454561
Monocotyledons	Araceae	*Wolffia*	*Wolffia columbiana*	GU454562
Monocotyledons	Araceae	*Wolffia*	*Wolffia columbiana*	GU454563
Monocotyledons	Araceae	*Wolffia*	*Wolffia columbiana*	GU454564
Monocotyledons	Araceae	*Wolffia*	*Wolffia columbiana*	HQ596901
Monocotyledons	Araceae	*Wolffia*	*Wolffia globosa*	GU454567

Monocotyledons	Araceae	*Wolffia*	*Wolffia globosa*	GU454568
Monocotyledons	Araceae	*Wolffia*	*Wolffia globosa*	GU454569
Monocotyledons	Araceae	*Wolffia*	*Wolffia globosa*	GU454570
Monocotyledons	Araceae	*Wolffia*	*Wolffia globosa*	GU454571
Monocotyledons	Araceae	*Wolffia*	*Wolffia globosa*	GU454572
Monocotyledons	Araceae	*Wolffia*	*Wolffia globosa*	GU454573
Monocotyledons	Araceae	*Wolffiella*	*Wolffiella gladiata*	GU454531
Monocotyledons	Araceae	*Wolffiella*	*Wolffiella gladiata*	GU454532
Monocotyledons	Araceae	*Wolffiella*	*Wolffiella gladiata*	GU454533
Monocotyledons	Araceae	*Wolffiella*	*Wolffiella gladiata*	GU454534
Monocotyledons	Araceae	*Wolffiella*	*Wolffiella gladiata*	GU454535
Monocotyledons	Araceae	*Wolffiella*	*Wolffiella lingulata*	GU454537
Monocotyledons	Araceae	*Wolffiella*	*Wolffiella lingulata*	GU454538
Monocotyledons	Araceae	*Wolffiella*	*Wolffiella lingulata*	GU454539
Monocotyledons	Araceae	*Wolffiella*	*Wolffiella lingulata*	GU454540
Monocotyledons	Araceae	*Wolffiella*	*Wolffiella lingulata*	GU454541
Monocotyledons	Araceae	*Wolffiella*	*Wolffiella neotropica*	GU454542
Monocotyledons	Araceae	*Wolffiella*	*Wolffiella neotropica*	GU454543
Monocotyledons	Araceae	*Wolffiella*	*Wolffiella neotropica*	GU454544
Monocotyledons	Araceae	*Wolffiella*	*Wolffiella oblonga*	GU454545
Monocotyledons	Araceae	*Wolffiella*	*Wolffiella oblonga*	GU454546
Monocotyledons	Araceae	*Wolffiella*	*Wolffiella oblonga*	GU454547
Monocotyledons	Araceae	*Wolffiella*	*Wolffiella oblonga*	GU454548
Monocotyledons	Araceae	*Wolffiella*	*Wolffiella oblonga*	GU454549
Monocotyledons	Araceae	*Wolffiella*	*Wolffiella rotunda*	GU454550
Monocotyledons	Araceae	*Wolffiella*	*Wolffiella rotunda*	GU454551
Monocotyledons	Poaceae	*Paspalum*	*Paspalum quadrifarium*	AY941123
Monocotyledons	Poaceae	*Paspalum*	*Paspalum quadrifarium*	AY941127
Monocotyledons	Orchidaceae	*Caucaea*	*Caucaea cucullata*	FJ564196
Monocotyledons	Orchidaceae	*Caucaea*	*Caucaea cucullata*	FJ564530
Monocotyledons	Orchidaceae	*Caucaea*	*Caucaea phalaenopsis*	FJ564006
Monocotyledons	Orchidaceae	*Caucaea*	*Caucaea phalaenopsis*	FJ564529
Monocotyledons	Orchidaceae	*Caucaea*	*Caucaea radiata*	FJ564281
Monocotyledons	Orchidaceae	*Caucaea*	*Caucaea radiata*	FJ564582
Monocotyledons	Orchidaceae	*Cyrtochilum*	*Cyrtochilum meirax*	FJ564444
Monocotyledons	Orchidaceae	*Cyrtochilum*	*Cyrtochilum meirax*	FJ564608
Monocotyledons	Orchidaceae	*Miltoniopsis*	*Miltoniopsis bismarkii*	FJ564469
Monocotyledons	Orchidaceae	*Miltoniopsis*	*Miltoniopsis bismarkii*	FJ564619
Monocotyledons	Potamogetonaceae	*Potamogeton*	*Potamogeton lucens*	DQ786538
Monocotyledons	Potamogetonaceae	*Potamogeton*	*Potamogeton lucens*	EF174573
Monocotyledons	Potamogetonaceae	*Potamogeton*	*Potamogeton natans*	DQ786540
Monocotyledons	Potamogetonaceae	*Potamogeton*	*Potamogeton natans*	HQ596798
Monocotyledons	Asparagaceae	*Maianthemum*	*Maianthemum bifolium*	EU850234
Monocotyledons	Asparagaceae	*Maianthemum*	*Maianthemum bifolium*	EU850235
Monocotyledons	Asparagaceae	*Maianthemum*	*Maianthemum bifolium*	JN045441
Monocotyledons	Asparagaceae	*Maianthemum*	*Maianthemum bifolium*	JN045442
Monocotyledons	Asparagaceae	*Maianthemum*	*Maianthemum bifolium*	JN045443
Monocotyledons	Zingiberaceae	*Gagnepainia*	*Gagnepainia thoreliana*	GQ386051
Monocotyledons	Zingiberaceae	*Gagnepainia*	*Gagnepainia thoreliana*	GQ386052
Monocotyledons	Zingiberaceae	*Gagnepainia*	*Gagnepainia thoreliana*	GQ386053
Monocotyledons	Zingiberaceae	*Gagnepainia*	*Gagnepainia thoreliana*	GQ386054
Monocotyledons	Alismataceae	*Echinodorus*	*Echinodorus uruguayensis*	HM367293
Monocotyledons	Alismataceae	*Echinodorus*	*Echinodorus uruguayensis*	HM367295

Monocotyledons	Alismataceae	*Echinodorus*	*Echinodorus uruguayensis*	HM367316
Monocotyledons	Potamogetonaceae	*Potamogeton*	*Potamogeton diversifolius*	DQ786529
Monocotyledons	Potamogetonaceae	*Potamogeton*	*Potamogeton diversifolius*	DQ786530
Monocotyledons	Colchicaceae	*Colchicum*	*Colchicum lusitanum*	DQ088320
Monocotyledons	Colchicaceae	*Colchicum*	*Colchicum lusitanum*	JF934123
Monocotyledons	Colchicaceae	*Colchicum*	*Colchicum lusitanum*	JF934124
Monocotyledons	Colchicaceae	*Colchicum*	*Colchicum montanum*	DQ088324
Monocotyledons	Colchicaceae	*Colchicum*	*Colchicum montanum*	JF934134
Monocotyledons	Colchicaceae	*Colchicum*	*Colchicum doerfleri*	JF934095
Monocotyledons	Colchicaceae	*Colchicum*	*Colchicum doerfleri*	JF934110
Monocotyledons	Colchicaceae	*Colchicum*	*Colchicum luteum*	EU237059
Monocotyledons	Colchicaceae	*Colchicum*	*Colchicum luteum*	JF934125
Monocotyledons	Colchicaceae	*Colchicum*	*Colchicum macrophyllum*	JF934127
Monocotyledons	Colchicaceae	*Colchicum*	*Colchicum macrophyllum*	JF934128
Monocotyledons	Poaceae	*Dendrocalamus*	*Dendrocalamus strictus*	GU391004
Monocotyledons	Poaceae	*Dendrocalamus*	*Dendrocalamus strictus*	GU063120
Monocotyledons	Potamogetonaceae	*Potamogeton*	*Potamogeton maackianus*	JN046453
Monocotyledons	Potamogetonaceae	*Potamogeton*	*Potamogeton maackianus*	JN046454
Monocotyledons	Potamogetonaceae	*Potamogeton*	*Potamogeton maackianus*	JN046455
Monocotyledons	Potamogetonaceae	*Potamogeton*	*Potamogeton maackianus*	JN046456
Monocotyledons	Potamogetonaceae	*Potamogeton*	*Potamogeton maackianus*	JN046457
Monocotyledons	Orchidaceae	*Prosthechea*	*Prosthechea fragrans*	EU213759
Monocotyledons	Orchidaceae	*Prosthechea*	*Prosthechea fragrans*	EU213760
Monocotyledons	Orchidaceae	*Cyrtochilum*	*Cyrtochilum serratum*	FJ564014
Monocotyledons	Orchidaceae	*Cyrtochilum*	*Cyrtochilum serratum*	FJ564123
Monocotyledons	Asparagaceae	*Polygonatum*	*Polygonatum acuminatifolium*	JN046398
Monocotyledons	Asparagaceae	*Polygonatum*	*Polygonatum acuminatifolium*	JN046399
Monocotyledons	Asparagaceae	*Polygonatum*	*Polygonatum sibiricum*	GQ434879
Monocotyledons	Asparagaceae	*Polygonatum*	*Polygonatum sibiricum*	GQ434880
Monocotyledons	Asparagaceae	*Polygonatum*	*Polygonatum sibiricum*	EU850210
Monocotyledons	Asparagaceae	*Polygonatum*	*Polygonatum verticillatum*	JN046419
Monocotyledons	Asparagaceae	*Polygonatum*	*Polygonatum verticillatum*	JN046420
Monocotyledons	Asparagaceae	*Polygonatum*	*Polygonatum verticillatum*	JN046421
Monocotyledons	Asparagaceae	*Polygonatum*	*Polygonatum verticillatum*	JN046422
Monocotyledons	Asparagaceae	*Maianthemum*	*Maianthemum atropurpureum*	EU850218
Monocotyledons	Asparagaceae	*Maianthemum*	*Maianthemum atropurpureum*	EU850219
Monocotyledons	Asparagaceae	*Maianthemum*	*Maianthemum atropurpureum*	EU850223
Monocotyledons	Asparagaceae	*Maianthemum*	*Maianthemum atropurpureum*	JN045433
Monocotyledons	Asparagaceae	*Maianthemum*	*Maianthemum atropurpureum*	JN045434
Monocotyledons	Asparagaceae	*Maianthemum*	*Maianthemum atropurpureum*	JN045435
Monocotyledons	Asparagaceae	*Maianthemum*	*Maianthemum atropurpureum*	JN045436
Monocotyledons	Asparagaceae	*Maianthemum*	*Maianthemum atropurpureum*	JN045437
Monocotyledons	Asparagaceae	*Maianthemum*	*Maianthemum atropurpureum*	JN045438
Monocotyledons	Asparagaceae	*Maianthemum*	*Maianthemum atropurpureum*	JN045439
Monocotyledons	Asparagaceae	*Maianthemum*	*Maianthemum atropurpureum*	JN045440
Monocotyledons	Asparagaceae	*Maianthemum*	*Maianthemum paniculatum*	EU850242
Monocotyledons	Asparagaceae	*Maianthemum*	*Maianthemum paniculatum*	EU850243
Monocotyledons	Asparagaceae	*Maianthemum*	*Maianthemum purpureum*	EU850221
Monocotyledons	Asparagaceae	*Maianthemum*	*Maianthemum purpureum*	JN045460
Monocotyledons	Asparagaceae	*Maianthemum*	*Maianthemum purpureum*	JN045461
Monocotyledons	Asparagaceae	*Maianthemum*	*Maianthemum purpureum*	JN045462
Monocotyledons	Asparagaceae	*Maianthemum*	*Maianthemum purpureum*	JN045463
Monocotyledons	Asparagaceae	*Maianthemum*	*Maianthemum purpureum*	JN045464

Monocotyledons	Asparagaceae	*Maianthemum*	*Maianthemum purpureum*	JN045465
Monocotyledons	Asparagaceae	*Maianthemum*	*Maianthemum purpureum*	JN045466
Monocotyledons	Asparagaceae	*Maianthemum*	*Maianthemum tatsienense*	EU850228
Monocotyledons	Asparagaceae	*Maianthemum*	*Maianthemum tatsienense*	JN045469
Monocotyledons	Asparagaceae	*Maianthemum*	*Maianthemum tatsienense*	JN045470
Monocotyledons	Asparagaceae	*Maianthemum*	*Maianthemum tatsienense*	JN045471
Monocotyledons	Asparagaceae	*Maianthemum*	*Maianthemum tatsienense*	JN045472
Monocotyledons	Asparagaceae	*Maianthemum*	*Maianthemum tatsienense*	JN045473
Monocotyledons	Asparagaceae	*Maianthemum*	*Maianthemum tatsienense*	JN045474
Monocotyledons	Asparagaceae	*Maianthemum*	*Maianthemum tubiferum*	EU850225
Monocotyledons	Asparagaceae	*Maianthemum*	*Maianthemum tubiferum*	JN045475
Monocotyledons	Colchicaceae	*Colchicum*	*Colchicum filifolium*	DQ088323
Monocotyledons	Colchicaceae	*Colchicum*	*Colchicum filifolium*	JF934102
Monocotyledons	Bromeliaceae	*Aechmea*	*Aechmea nudicaulis*	JN204618
Monocotyledons	Bromeliaceae	*Aechmea*	*Aechmea nudicaulis*	JN204619
Monocotyledons	Bromeliaceae	*Tillandsia*	*Tillandsia tenuifolia*	JN204667
Monocotyledons	Bromeliaceae	*Tillandsia*	*Tillandsia tenuifolia*	JN204668
Monocotyledons	Juncaceae	*Juncus*	*Juncus dudleyi*	HQ596737
Monocotyledons	Juncaceae	*Juncus*	*Juncus dudleyi*	HQ596738
Monocotyledons	Poaceae	*Elymus*	*Elymus brevipes*	HQ221851
Monocotyledons	Poaceae	*Elymus*	*Elymus brevipes*	HQ652802
Monocotyledons	Cyperaceae	*Carex*	*Carex hitchcockiana*	FJ597291
Monocotyledons	Cyperaceae	*Carex*	*Carex hitchcockiana*	FJ597292
Monocotyledons	Cyperaceae	*Carex*	*Carex hitchcockiana*	FJ597293
Monocotyledons	Cyperaceae	*Carex*	*Carex hitchcockiana*	FJ597294
Monocotyledons	Cyperaceae	*Carex*	*Carex oligocarpa*	FJ597295
Monocotyledons	Cyperaceae	*Carex*	*Carex oligocarpa*	FJ597296
Monocotyledons	Cyperaceae	*Carex*	*Carex oligocarpa*	DQ006177
Monocotyledons	Poaceae	*Paspalum*	*Paspalum dasypleurum*	AY769128
Monocotyledons	Poaceae	*Paspalum*	*Paspalum dasypleurum*	DQ104301
Monocotyledons	Poaceae	*Paspalum*	*Paspalum dilatatum*	AY769130
Monocotyledons	Poaceae	*Paspalum*	*Paspalum dilatatum*	DQ104295
Monocotyledons	Poaceae	*Paspalum*	*Paspalum dilatatum*	DQ104296
Monocotyledons	Poaceae	*Paspalum*	*Paspalum dilatatum*	DQ104297
Monocotyledons	Poaceae	*Paspalum*	*Paspalum dilatatum*	DQ104298
Monocotyledons	Poaceae	*Paspalum*	*Paspalum dilatatum*	DQ104299
Monocotyledons	Poaceae	*Paspalum*	*Paspalum pauciciliatum*	AY769144
Monocotyledons	Poaceae	*Paspalum*	*Paspalum pauciciliatum*	DQ104302
Monocotyledons	Poaceae	*Paspalum*	*Paspalum urvillei*	AY769148
Monocotyledons	Poaceae	*Paspalum*	*Paspalum urvillei*	DQ104300
Monocotyledons	Cymodoceaceae	*Halodule*	*Halodule uninervis*	AB571191
Monocotyledons	Cymodoceaceae	*Halodule*	*Halodule uninervis*	AB571192
Monocotyledons	Cymodoceaceae	*Halodule*	*Halodule uninervis*	AB571193
Monocotyledons	Cymodoceaceae	*Halodule*	*Halodule uninervis*	AB571194
Monocotyledons	Cymodoceaceae	*Halodule*	*Halodule uninervis*	AB571195
Monocotyledons	Bromeliaceae	*Aechmea*	*Aechmea racinae*	EF110659
Monocotyledons	Bromeliaceae	*Aechmea*	*Aechmea racinae*	HQ913655
Monocotyledons	Cyperaceae	*Carex*	*Carex amphibola*	FJ597264
Monocotyledons	Cyperaceae	*Carex*	*Carex amphibola*	FJ597265
Monocotyledons	Cyperaceae	*Carex*	*Carex amphibola*	FJ597266
Monocotyledons	Cyperaceae	*Carex*	*Carex amphibola*	DQ006175
Monocotyledons	Poaceae	*Calamagrostis*	*Calamagrostis muiriana*	DQ113917
Monocotyledons	Poaceae	*Calamagrostis*	*Calamagrostis muiriana*	DQ113918

Monocotyledons	Poaceae	*Calamagrostis*	*Calamagrostis muiriana*	DQ113919
Monocotyledons	Poaceae	*Calamagrostis*	*Calamagrostis muiriana*	DQ113920
Monocotyledons	Poaceae	*Calamagrostis*	*Calamagrostis muiriana*	DQ113921
Monocotyledons	Poaceae	*Calamagrostis*	*Calamagrostis breweri*	DQ113922
Monocotyledons	Poaceae	*Calamagrostis*	*Calamagrostis breweri*	DQ113923
Monocotyledons	Poaceae	*Calamagrostis*	*Calamagrostis breweri*	DQ113924
Monocotyledons	Poaceae	*Calamagrostis*	*Calamagrostis breweri*	DQ113925
Monocotyledons	Poaceae	*Agrostis*	*Agrostis gigantea*	FJ395454
Monocotyledons	Poaceae	*Agrostis*	*Agrostis gigantea*	HQ596584
Monocotyledons	Poaceae	*Agrostis*	*Agrostis gigantea*	HQ596585
Monocotyledons	Orchidaceae	*Fernandezia*	*Fernandezia tica*	FJ564340
Monocotyledons	Orchidaceae	*Fernandezia*	*Fernandezia tica*	FJ564442
Monocotyledons	Alismataceae	*Alisma*	*Alisma gramineum*	JN043776
Monocotyledons	Alismataceae	*Alisma*	*Alisma gramineum*	JN043777
Monocotyledons	Hydrocharitaceae	*Najas*	*Najas gracillima*	HQ687168
Monocotyledons	Hydrocharitaceae	*Najas*	*Najas gracillima*	HQ687169
Monocotyledons	Hydrocharitaceae	*Najas*	*Najas gracillima*	HQ687170
Monocotyledons	Hydrocharitaceae	*Najas*	*Najas gracillima*	HQ687171
Monocotyledons	Hydrocharitaceae	*Najas*	*Najas gracillima*	HQ687172
Monocotyledons	Hydrocharitaceae	*Najas*	*Najas gracillima*	HQ687173
Monocotyledons	Potamogetonaceae	*Potamogeton*	*Potamogeton illinoensis*	DQ786536
Monocotyledons	Potamogetonaceae	*Potamogeton*	*Potamogeton illinoensis*	DQ786537
Monocotyledons	Poaceae	*Stipa*	*Stipa papposa*	EU489274
Monocotyledons	Poaceae	*Stipa*	*Stipa papposa*	EU204706
Monocotyledons	Poaceae	*Stipa*	*Stipa papposa*	EU204707
Monocotyledons	Poaceae	*Stipa*	*Stipa papposa*	EU204708
Monocotyledons	Poaceae	*Stipa*	*Stipa papposa*	EU204709
Monocotyledons	Poaceae	*Stipa*	*Stipa papposa*	EU204710
Monocotyledons	Poaceae	*Elymus*	*Elymus nutans*	HQ221835
Monocotyledons	Poaceae	*Elymus*	*Elymus nutans*	HQ221845
Monocotyledons	Poaceae	*Elymus*	*Elymus nutans*	HQ221847
Monocotyledons	Asparagaceae	*Maianthemum*	*Maianthemum canadense*	HQ596762
Monocotyledons	Asparagaceae	*Maianthemum*	*Maianthemum canadense*	EU850236
Monocotyledons	Orchidaceae	*Cuitlauzina*	*Cuitlauzina candida*	FJ564070
Monocotyledons	Orchidaceae	*Cuitlauzina*	*Cuitlauzina candida*	FJ564670
Monocotyledons	Orchidaceae	*Comparettia*	*Comparettia bennettii*	FJ564115
Monocotyledons	Orchidaceae	*Comparettia*	*Comparettia bennettii*	FJ564365
Monocotyledons	Orchidaceae	*Tsiorchis*	*Tsiorchis kimballiana*	HQ404447
Monocotyledons	Orchidaceae	*Tsiorchis*	*Tsiorchis kimballiana*	HQ404448
Monocotyledons	Orchidaceae	*Tsiorchis*	*Tsiorchis kimballiana*	HQ404449
Monocotyledons	Orchidaceae	*Tsiorchis*	*Tsiorchis kimballiana*	HQ404450
Monocotyledons	Orchidaceae	*Tsiorchis*	*Tsiorchis kimballiana*	HQ404451
Monocotyledons	Orchidaceae	*Tsiorchis*	*Tsiorchis kimballiana*	HQ404452
Monocotyledons	Alismataceae	*Echinodorus*	*Echinodorus floribundus*	HM367287
Monocotyledons	Alismataceae	*Echinodorus*	*Echinodorus floribundus*	HM367288
Monocotyledons	Alismataceae	*Echinodorus*	*Echinodorus floribundus*	HM367305
Monocotyledons	Alismataceae	*Echinodorus*	*Echinodorus grisebachii*	HM367271
Monocotyledons	Alismataceae	*Echinodorus*	*Echinodorus grisebachii*	HM367272
Monocotyledons	Alismataceae	*Echinodorus*	*Echinodorus grisebachii*	HM367273
Monocotyledons	Alismataceae	*Echinodorus*	*Echinodorus grisebachii*	HM367275
Monocotyledons	Alismataceae	*Echinodorus*	*Echinodorus grisebachii*	HM367276
Monocotyledons	Alismataceae	*Echinodorus*	*Echinodorus grisebachii*	HM367277
Monocotyledons	Alismataceae	*Echinodorus*	*Echinodorus grisebachii*	HM367278

Monocotyledons	Alismataceae	*Echinodorus*	*Echinodorus grisebachii*	HM367279
Monocotyledons	Alismataceae	*Echinodorus*	*Echinodorus grisebachii*	HM367280
Monocotyledons	Alismataceae	*Echinodorus*	*Echinodorus grisebachii*	HM367281
Monocotyledons	Alismataceae	*Echinodorus*	*Echinodorus longiscapus*	HM367289
Monocotyledons	Alismataceae	*Echinodorus*	*Echinodorus longiscapus*	HM367292
Monocotyledons	Alismataceae	*Echinodorus*	*Echinodorus paniculatus*	HM367285
Monocotyledons	Alismataceae	*Echinodorus*	*Echinodorus paniculatus*	HM367306
Monocotyledons	Bromeliaceae	*Aechmea*	*Aechmea lingulata*	EF110667
Monocotyledons	Bromeliaceae	*Aechmea*	*Aechmea lingulata*	JN204616
Monocotyledons	Bromeliaceae	*Aechmea*	*Aechmea lingulata*	JN204617
Monocotyledons	Bromeliaceae	*Aechmea*	*Aechmea lingulata*	HQ913652
Monocotyledons	Poaceae	*Calamagrostis*	*Calamagrostis erectifolia*	GQ248257
Monocotyledons	Poaceae	*Calamagrostis*	*Calamagrostis erectifolia*	EF590676
Monocotyledons	Arecaceae	*Sabal*	*Sabal etonia*	GQ248387
Monocotyledons	Arecaceae	*Sabal*	*Sabal etonia*	EF688515
Monocotyledons	Hydrocharitaceae	*Najas*	*Najas graminea*	HQ687174
Monocotyledons	Hydrocharitaceae	*Najas*	*Najas graminea*	HQ687175
Monocotyledons	Hydrocharitaceae	*Najas*	*Najas graminea*	HQ687176
Monocotyledons	Hydrocharitaceae	*Najas*	*Najas graminea*	HQ687177
Monocotyledons	Hydrocharitaceae	*Najas*	*Najas graminea*	HQ687178
Monocotyledons	Hydrocharitaceae	*Najas*	*Najas graminea*	HQ687179
Monocotyledons	Orchidaceae	*Paraholcoglossum*	*Paraholcoglossum subulifolium*	HQ404477
Monocotyledons	Orchidaceae	*Paraholcoglossum*	*Paraholcoglossum subulifolium*	HQ404478
Monocotyledons	Orchidaceae	*Paraholcoglossum*	*Paraholcoglossum subulifolium*	HQ404479
Monocotyledons	Orchidaceae	*Rhynchostele*	*Rhynchostele cordata*	GQ248386
Monocotyledons	Orchidaceae	*Rhynchostele*	*Rhynchostele cordata*	FJ564158
Monocotyledons	Orchidaceae	*Rhynchostele*	*Rhynchostele cordata*	FJ564666
Monocotyledons	Arecaceae	*Hyphaene*	*Hyphaene petersiana*	EU213779
Monocotyledons	Arecaceae	*Hyphaene*	*Hyphaene petersiana*	EU213780
Monocotyledons	Colchicaceae	*Colchicum*	*Colchicum kesselringii*	EU237058
Monocotyledons	Colchicaceae	*Colchicum*	*Colchicum kesselringii*	JF934114
Monocotyledons	Colchicaceae	*Colchicum*	*Colchicum robustum*	EU237060
Monocotyledons	Colchicaceae	*Colchicum*	*Colchicum robustum*	JF934152
Monocotyledons	Colchicaceae	*Colchicum*	*Colchicum robustum*	JF934153
Monocotyledons	Colchicaceae	*Colchicum*	*Colchicum robustum*	JF934154
Monocotyledons	Orchidaceae	*Prosthechea*	*Prosthechea radiata*	EU213761
Monocotyledons	Orchidaceae	*Prosthechea*	*Prosthechea radiata*	EU213762
Monocotyledons	Orchidaceae	*Prosthechea*	*Prosthechea radiata*	EU213763
Monocotyledons	Orchidaceae	*Trichopilia*	*Trichopilia tortilis*	EU213769
Monocotyledons	Orchidaceae	*Trichopilia*	*Trichopilia tortilis*	EU213770
Monocotyledons	Orchidaceae	*Trichopilia*	*Trichopilia tortilis*	EU213771
Monocotyledons	Orchidaceae	*Trichopilia*	*Trichopilia turialbae*	EU213772
Monocotyledons	Orchidaceae	*Trichopilia*	*Trichopilia turialbae*	EU213773
Monocotyledons	Orchidaceae	*Trichopilia*	*Trichopilia turialbae*	EU213774
Monocotyledons	Orchidaceae	*Trichopilia*	*Trichopilia turialbae*	FJ564132
Monocotyledons	Orchidaceae	*Trichopilia*	*Trichopilia turialbae*	FJ564632
Monocotyledons	Poaceae	*Gigantochloa*	*Gigantochloa scortechinii*	GU063129
Monocotyledons	Poaceae	*Gigantochloa*	*Gigantochloa scortechinii*	HQ697908
Monocotyledons	Poaceae	*Gigantochloa*	*Gigantochloa scortechinii*	HQ697909
Monocotyledons	Poaceae	*Gigantochloa*	*Gigantochloa scortechinii*	HQ697910
Monocotyledons	Poaceae	*Gigantochloa*	*Gigantochloa scortechinii*	HQ697911
Monocotyledons	Poaceae	*Gigantochloa*	*Gigantochloa scortechinii*	HQ697912
Monocotyledons	Orchidaceae	*Paraholcoglossum*	*Paraholcoglossum amesianum*	HQ404436

Monocotyledons	Orchidaceae	*Paraholcoglossum*	*Paraholcoglossum amesianum*	HQ404437
Monocotyledons	Orchidaceae	*Paraholcoglossum*	*Paraholcoglossum amesianum*	HQ404438
Monocotyledons	Orchidaceae	*Paraholcoglossum*	*Paraholcoglossum amesianum*	HQ404439
Monocotyledons	Orchidaceae	*Paraholcoglossum*	*Paraholcoglossum amesianum*	HQ404440
Monocotyledons	Orchidaceae	*Paraholcoglossum*	*Paraholcoglossum amesianum*	HQ404441
Monocotyledons	Orchidaceae	*Tsiorchis*	*Tsiorchis wangii*	HQ404480
Monocotyledons	Orchidaceae	*Tsiorchis*	*Tsiorchis wangii*	HQ404481
Monocotyledons	Orchidaceae	*Tsiorchis*	*Tsiorchis wangii*	HQ404482
Monocotyledons	Asparagaceae	*Maianthemum*	*Maianthemum dahuricum*	EU850233
Monocotyledons	Asparagaceae	*Maianthemum*	*Maianthemum dahuricum*	JN045444
Monocotyledons	Asparagaceae	*Maianthemum*	*Maianthemum gigas*	EU850241
Monocotyledons	Asparagaceae	*Maianthemum*	*Maianthemum gigas*	JN045445
Monocotyledons	Asparagaceae	*Maianthemum*	*Maianthemum gongshanense*	EU850214
Monocotyledons	Asparagaceae	*Maianthemum*	*Maianthemum gongshanense*	JN045446
Monocotyledons	Asparagaceae	*Maianthemum*	*Maianthemum henryi*	EU850213
Monocotyledons	Asparagaceae	*Maianthemum*	*Maianthemum henryi*	EU850217
Monocotyledons	Asparagaceae	*Maianthemum*	*Maianthemum henryi*	EU850224
Monocotyledons	Asparagaceae	*Maianthemum*	*Maianthemum henryi*	JN045447
Monocotyledons	Asparagaceae	*Maianthemum*	*Maianthemum henryi*	JN045448
Monocotyledons	Asparagaceae	*Maianthemum*	*Maianthemum henryi*	JN045449
Monocotyledons	Asparagaceae	*Maianthemum*	*Maianthemum henryi*	JN045450
Monocotyledons	Asparagaceae	*Maianthemum*	*Maianthemum henryi*	JN045451
Monocotyledons	Asparagaceae	*Maianthemum*	*Maianthemum henryi*	JN045452
Monocotyledons	Asparagaceae	*Maianthemum*	*Maianthemum henryi*	JN045453
Monocotyledons	Poaceae	*Dendrocalamus*	*Dendrocalamus pendulus*	HQ697902
Monocotyledons	Poaceae	*Dendrocalamus*	*Dendrocalamus pendulus*	HQ697903
Monocotyledons	Poaceae	*Dendrocalamus*	*Dendrocalamus pendulus*	HQ697904
Monocotyledons	Poaceae	*Gigantochloa*	*Gigantochloa balui*	GU391008
Monocotyledons	Poaceae	*Gigantochloa*	*Gigantochloa balui*	GU063125
Monocotyledons	Orchidaceae	*Gomesa*	*Gomesa colorata*	FJ564107
Monocotyledons	Orchidaceae	*Gomesa*	*Gomesa colorata*	EU935667
Monocotyledons	Orchidaceae	*Gomesa*	*Gomesa cornigera*	FJ564387
Monocotyledons	Orchidaceae	*Gomesa*	*Gomesa cornigera*	EU935680
Monocotyledons	Orchidaceae	*Gomesa*	*Gomesa echinata*	FJ564383
Monocotyledons	Orchidaceae	*Gomesa*	*Gomesa echinata*	EU935677
Monocotyledons	Orchidaceae	*Gomesa*	*Gomesa kautskyi*	FJ564400
Monocotyledons	Orchidaceae	*Gomesa*	*Gomesa kautskyi*	EU935686
Monocotyledons	Orchidaceae	*Gomesa*	*Gomesa lietzei*	FJ564638
Monocotyledons	Orchidaceae	*Gomesa*	*Gomesa lietzei*	EU935689
Monocotyledons	Orchidaceae	*Gomesa*	*Gomesa pubes*	FJ563995
Monocotyledons	Orchidaceae	*Gomesa*	*Gomesa pubes*	EU935681
Monocotyledons	Orchidaceae	*Tolumnia*	*Tolumnia pulchella*	FJ564265
Monocotyledons	Orchidaceae	*Tolumnia*	*Tolumnia pulchella*	EU935692
Monocotyledons	Orchidaceae	*Gomesa*	*Gomesa sarcodes*	FJ564399
Monocotyledons	Orchidaceae	*Gomesa*	*Gomesa sarcodes*	EU935682
Monocotyledons	Orchidaceae	*Gomesa*	*Gomesa silvana*	FJ564672
Monocotyledons	Orchidaceae	*Gomesa*	*Gomesa silvana*	EU935676
Monocotyledons	Orchidaceae	*Gomesa*	*Gomesa truncata*	FJ564384
Monocotyledons	Orchidaceae	*Gomesa*	*Gomesa truncata*	EU935691
Monocotyledons	Orchidaceae	*Gomesa*	*Gomesa venusta*	FJ564310
Monocotyledons	Orchidaceae	*Gomesa*	*Gomesa venusta*	EU935668
Monocotyledons	Orchidaceae	*Gomesa*	*Gomesa widgrenii*	FJ564671
Monocotyledons	Orchidaceae	*Gomesa*	*Gomesa widgrenii*	EU935679

Monocotyledons	Orchidaceae	*Gomesa*	*Gomesa barbata*	FJ564410
Monocotyledons	Orchidaceae	*Gomesa*	*Gomesa barbata*	EU935662
Monocotyledons	Orchidaceae	*Gomesa*	*Gomesa concolor*	FJ564261
Monocotyledons	Orchidaceae	*Gomesa*	*Gomesa concolor*	FJ564663
Monocotyledons	Orchidaceae	*Gomesa*	*Gomesa concolor*	EU935664
Monocotyledons	Orchidaceae	*Gomesa*	*Gomesa hydrophila*	FJ564396
Monocotyledons	Orchidaceae	*Gomesa*	*Gomesa hydrophila*	EU935665
Monocotyledons	Orchidaceae	*Erycina*	*Erycina glossomystax*	FJ564592
Monocotyledons	Orchidaceae	*Erycina*	*Erycina glossomystax*	FJ564593
Monocotyledons	Orchidaceae	*Erycina*	*Erycina glossomystax*	FJ564595
Monocotyledons	Orchidaceae	*Lockhartia*	*Lockhartia bennettii*	FJ563975
Monocotyledons	Orchidaceae	*Lockhartia*	*Lockhartia bennettii*	FJ564528
Monocotyledons	Orchidaceae	*Odontoglossum*	*Odontoglossum sanguineum*	FJ564028
Monocotyledons	Orchidaceae	*Odontoglossum*	*Odontoglossum sanguineum*	FJ564501
Monocotyledons	Orchidaceae	*Ornithocephalus*	*Ornithocephalus bicornis*	FJ564567
Monocotyledons	Orchidaceae	*Ornithocephalus*	*Ornithocephalus bicornis*	FJ564647
Monocotyledons	Orchidaceae	*Ornithocephalus*	*Ornithocephalus suarezii*	FJ564563
Monocotyledons	Orchidaceae	*Ornithocephalus*	*Ornithocephalus suarezii*	FJ564566
Monocotyledons	Orchidaceae	*Trichocentrum*	*Trichocentrum lindenii*	FJ564304
Monocotyledons	Orchidaceae	*Trichocentrum*	*Trichocentrum lindenii*	FJ564545
Monocotyledons	Orchidaceae	*Trichocentrum*	*Trichocentrum stipitatum*	FJ564126
Monocotyledons	Orchidaceae	*Trichocentrum*	*Trichocentrum stipitatum*	FJ564659
Monocotyledons	Orchidaceae	*Gomesa*	*Gomesa warmingii*	FJ564089
Monocotyledons	Orchidaceae	*Gomesa*	*Gomesa warmingii*	FJ564391
Monocotyledons	Orchidaceae	*Gomesa*	*Gomesa warmingii*	FJ564409
Monocotyledons	Orchidaceae	*Cischweinfia*	*Cischweinfia popowiana*	FJ564312
Monocotyledons	Orchidaceae	*Cischweinfia*	*Cischweinfia popowiana*	FJ564679
Monocotyledons	Orchidaceae	*Cischweinfia*	*Cischweinfia pusilla*	FJ564122
Monocotyledons	Orchidaceae	*Cischweinfia*	*Cischweinfia pusilla*	FJ564651
Monocotyledons	Orchidaceae	*Cuitlauzina*	*Cuitlauzina pulchella*	FJ564003
Monocotyledons	Orchidaceae	*Cuitlauzina*	*Cuitlauzina pulchella*	FJ564174
Monocotyledons	Orchidaceae	*Cyrtochilum*	*Cyrtochilum hoeijeri*	FJ564313
Monocotyledons	Orchidaceae	*Cyrtochilum*	*Cyrtochilum hoeijeri*	FJ564443
Monocotyledons	Orchidaceae	*Cyrtochilum*	*Cyrtochilum trifurcatum*	FJ564110
Monocotyledons	Orchidaceae	*Cyrtochilum*	*Cyrtochilum trifurcatum*	FJ564504
Monocotyledons	Orchidaceae	*Ornithocephalus*	*Ornithocephalus dalstroemii*	FJ563990
Monocotyledons	Orchidaceae	*Ornithocephalus*	*Ornithocephalus dalstroemii*	FJ564601
Monocotyledons	Orchidaceae	*Ornithocephalus*	*Ornithocephalus dalstroemii*	FJ564613
Monocotyledons	Orchidaceae	*Tolumnia*	*Tolumnia guianensis*	FJ564218
Monocotyledons	Orchidaceae	*Tolumnia*	*Tolumnia guianensis*	FJ564233
Monocotyledons	Orchidaceae	*Tolumnia*	*Tolumnia prionochila*	FJ564219
Monocotyledons	Orchidaceae	*Tolumnia*	*Tolumnia prionochila*	FJ564252
Monocotyledons	Orchidaceae	*Comparettia*	*Comparettia corydaloides*	FJ564280
Monocotyledons	Orchidaceae	*Comparettia*	*Comparettia corydaloides*	FJ564360
Monocotyledons	Orchidaceae	*Comparettia*	*Comparettia tungurahuae*	FJ564523
Monocotyledons	Orchidaceae	*Comparettia*	*Comparettia tungurahuae*	FJ564611
Monocotyledons	Orchidaceae	*Gomesa*	*Gomesa forbesii*	FJ564408
Monocotyledons	Orchidaceae	*Gomesa*	*Gomesa forbesii*	FJ564681
Monocotyledons	Orchidaceae	*Gomesa*	*Gomesa gardneri*	FJ564684
Monocotyledons	Orchidaceae	*Gomesa*	*Gomesa gardneri*	EU935666
Monocotyledons	Orchidaceae	*Gomesa*	*Gomesa longipes*	FJ564358
Monocotyledons	Orchidaceae	*Gomesa*	*Gomesa longipes*	FJ564394
Monocotyledons	Orchidaceae	*Gomesa*	*Gomesa varicosa*	FJ564411

Monocotyledons	Orchidaceae	*Gomesa*	*Gomesa varicosa*	FJ564682
Monocotyledons	Orchidaceae	*Trichocentrum*	*Trichocentrum cosymbephorum*	FJ564301
Monocotyledons	Orchidaceae	*Trichocentrum*	*Trichocentrum cosymbephorum*	FJ564473
Monocotyledons	Bromeliaceae	*Tillandsia*	*Tillandsia bulbosa*	JN204663
Monocotyledons	Bromeliaceae	*Tillandsia*	*Tillandsia bulbosa*	JN204664
Monocotyledons	Orchidaceae	*Cymbidium*	*Cymbidium haematodes*	HM008996
Monocotyledons	Orchidaceae	*Cymbidium*	*Cymbidium haematodes*	JN412741
Monocotyledons	Cyperaceae	*Carex*	*Carex acidicola*	FJ597262
Monocotyledons	Cyperaceae	*Carex*	*Carex acidicola*	FJ597263
Monocotyledons	Cyperaceae	*Carex*	*Carex acidicola*	FJ597311
Monocotyledons	Cyperaceae	*Carex*	*Carex bulbostylis*	FJ597268
Monocotyledons	Cyperaceae	*Carex*	*Carex bulbostylis*	FJ597269
Monocotyledons	Cyperaceae	*Carex*	*Carex calcifugens*	FJ597270
Monocotyledons	Cyperaceae	*Carex*	*Carex calcifugens*	FJ597271
Monocotyledons	Cyperaceae	*Carex*	*Carex conoidea*	FJ597272
Monocotyledons	Cyperaceae	*Carex*	*Carex conoidea*	FJ597273
Monocotyledons	Cyperaceae	*Carex*	*Carex corrugata*	FJ597274
Monocotyledons	Cyperaceae	*Carex*	*Carex corrugata*	FJ597275
Monocotyledons	Cyperaceae	*Carex*	*Carex corrugata*	FJ597276
Monocotyledons	Cyperaceae	*Carex*	*Carex corrugata*	FJ597277
Monocotyledons	Cyperaceae	*Carex*	*Carex flaccosperma*	FJ597278
Monocotyledons	Cyperaceae	*Carex*	*Carex flaccosperma*	FJ597279
Monocotyledons	Cyperaceae	*Carex*	*Carex flaccosperma*	FJ597280
Monocotyledons	Cyperaceae	*Carex*	*Carex glaucodea*	FJ597281
Monocotyledons	Cyperaceae	*Carex*	*Carex glaucodea*	FJ597282
Monocotyledons	Cyperaceae	*Carex*	*Carex glaucodea*	FJ597283
Monocotyledons	Cyperaceae	*Carex*	*Carex glaucodea*	FJ597284
Monocotyledons	Cyperaceae	*Carex*	*Carex glaucodea*	FJ597285
Monocotyledons	Cyperaceae	*Carex*	*Carex godfreyi*	FJ597286
Monocotyledons	Cyperaceae	*Carex*	*Carex godfreyi*	FJ597287
Monocotyledons	Cyperaceae	*Carex*	*Carex grisea*	FJ597288
Monocotyledons	Cyperaceae	*Carex*	*Carex grisea*	FJ597289
Monocotyledons	Cyperaceae	*Carex*	*Carex grisea*	FJ597290
Monocotyledons	Cyperaceae	*Carex*	*Carex ouachitana*	FJ597297
Monocotyledons	Cyperaceae	*Carex*	*Carex ouachitana*	FJ597298
Monocotyledons	Cyperaceae	*Carex*	*Carex ouachitana*	FJ597299
Monocotyledons	Cyperaceae	*Carex*	*Carex paeninsulae*	FJ597300
Monocotyledons	Cyperaceae	*Carex*	*Carex paeninsulae*	FJ597301
Monocotyledons	Cyperaceae	*Carex*	*Carex paeninsulae*	FJ597302
Monocotyledons	Cyperaceae	*Carex*	*Carex pigra*	FJ597303
Monocotyledons	Cyperaceae	*Carex*	*Carex pigra*	FJ597304
Monocotyledons	Cyperaceae	*Carex*	*Carex planispicata*	FJ597305
Monocotyledons	Cyperaceae	*Carex*	*Carex planispicata*	FJ597306
Monocotyledons	Cyperaceae	*Carex*	*Carex planispicata*	FJ597307
Monocotyledons	Cyperaceae	*Carex*	*Carex thornei*	FJ597308
Monocotyledons	Cyperaceae	*Carex*	*Carex thornei*	FJ597309
Monocotyledons	Cyperaceae	*Carex*	*Carex thornei*	FJ597310
Monocotyledons	Bromeliaceae	*Billbergia*	*Billbergia euphemiae*	JN204630
Monocotyledons	Bromeliaceae	*Billbergia*	*Billbergia euphemiae*	JN204631
Monocotyledons	Bromeliaceae	*Billbergia*	*Billbergia euphemiae*	JN204632
Monocotyledons	Bromeliaceae	*Aechmea*	*Aechmea blumenavii*	JN204599
Monocotyledons	Bromeliaceae	*Aechmea*	*Aechmea blumenavii*	JN204600
Monocotyledons	Bromeliaceae	*Aechmea*	*Aechmea bromeliifolia*	JN204601

Monocotyledons	Bromeliaceae	*Aechmea*	*Aechmea bromeliifolia*	JN204602
Monocotyledons	Bromeliaceae	*Aechmea*	*Aechmea bromeliifolia*	JN204603
Monocotyledons	Bromeliaceae	*Aechmea*	*Aechmea bromeliifolia*	HQ913649
Monocotyledons	Bromeliaceae	*Aechmea*	*Aechmea coelestis*	JN204608
Monocotyledons	Bromeliaceae	*Aechmea*	*Aechmea coelestis*	JN204609
Monocotyledons	Bromeliaceae	*Aechmea*	*Aechmea ramosa*	JN204623
Monocotyledons	Bromeliaceae	*Aechmea*	*Aechmea ramosa*	JN204624
Monocotyledons	Bromeliaceae	*Aechmea*	*Aechmea recurvata*	JN204625
Monocotyledons	Bromeliaceae	*Aechmea*	*Aechmea recurvata*	JN204626
Monocotyledons	Bromeliaceae	*Aechmea*	*Aechmea recurvata*	JN204627
Monocotyledons	Arecaceae	*Arenga*	*Arenga engleri*	JF345020
Monocotyledons	Arecaceae	*Arenga*	*Arenga engleri*	JF345021
Monocotyledons	Arecaceae	*Arenga*	*Arenga engleri*	JF345022
Monocotyledons	Arecaceae	*Arenga*	*Arenga engleri*	JF345023
Monocotyledons	Orchidaceae	*Odontoglossum*	*Odontoglossum hybrid cultivar*	GU136252
Monocotyledons	Orchidaceae	*Odontoglossum*	*Odontoglossum hybrid cultivar*	GU136260
Monocotyledons	Arecaceae	*Arenga*	*Arenga westerhoutii*	JF345041
Monocotyledons	Arecaceae	*Arenga*	*Arenga westerhoutii*	JF345042
Monocotyledons	Alismataceae	*Echinodorus*	*Echinodorus hybrid cultivar*	HM367313
Monocotyledons	Alismataceae	*Echinodorus*	*Echinodorus hybrid cultivar*	HM367319
Monocotyledons	Alismataceae	*Echinodorus*	*Echinodorus hybrid cultivar*	HM367320
Monocotyledons	Alismataceae	*Echinodorus*	*Echinodorus hybrid cultivar*	HM367321
Monocotyledons	Alismataceae	*Echinodorus*	*Echinodorus hybrid cultivar*	HM367322
Monocotyledons	Alismataceae	*Echinodorus*	*Echinodorus hybrid cultivar*	HM367326
Monocotyledons	Alismataceae	*Echinodorus*	*Echinodorus hybrid cultivar*	HM367331
Monocotyledons	Alismataceae	*Echinodorus*	*Echinodorus hybrid cultivar*	HM367333
Monocotyledons	Alismataceae	*Echinodorus*	*Echinodorus hybrid cultivar*	HM367338
Monocotyledons	Alismataceae	*Echinodorus*	*Echinodorus hybrid cultivar*	HM367339
Monocotyledons	Asparagaceae	*Polygonatum*	*Polygonatum griffithii*	JN046413
Monocotyledons	Asparagaceae	*Polygonatum*	*Polygonatum griffithii*	JN046414
Monocotyledons	Asparagaceae	*Tupistra*	*Tupistra grandistigma*	JN047462
Monocotyledons	Asparagaceae	*Tupistra*	*Tupistra grandistigma*	JN047463
Monocotyledons	Asparagaceae	*Tupistra*	*Tupistra grandistigma*	JN047464
Monocotyledons	Asparagaceae	*Tupistra*	*Tupistra grandistigma*	JN047465
Monocotyledons	Asparagaceae	*Tupistra*	*Tupistra grandistigma*	JN047466
Monocotyledons	Asparagaceae	*Tupistra*	*Tupistra longispica*	JN047467
Monocotyledons	Asparagaceae	*Tupistra*	*Tupistra longispica*	JN047468
Monocotyledons	Asparagaceae	*Tupistra*	*Tupistra longispica*	JN047469
Monocotyledons	Asparagaceae	*Tupistra*	*Tupistra longispica*	JN047470
Monocotyledons	Asparagaceae	*Tupistra*	*Tupistra longispica*	JN047471
Monocotyledons	Asparagaceae	*Tupistra*	*Tupistra pingbianensis*	JN047472
Monocotyledons	Asparagaceae	*Tupistra*	*Tupistra pingbianensis*	JN047473
Monocotyledons	Asparagaceae	*Tupistra*	*Tupistra pingbianensis*	JN047474
Monocotyledons	Asparagaceae	*Tupistra*	*Tupistra pingbianensis*	JN047475
Monocotyledons	Hydrocharitaceae	*Najas*	*Najas oguraensis*	HQ687188
Monocotyledons	Hydrocharitaceae	*Najas*	*Najas oguraensis*	HQ687189
Monocotyledons	Hydrocharitaceae	*Najas*	*Najas oguraensis*	HQ687190
Monocotyledons	Hydrocharitaceae	*Najas*	*Najas chinensis*	HQ687191
Monocotyledons	Hydrocharitaceae	*Najas*	*Najas chinensis*	HQ687192
Monocotyledons	Hydrocharitaceae	*Najas*	*Najas chinensis*	HQ687193
Monocotyledons	Arecaceae	*Arenga*	*Arenga hastata*	JF345005
Monocotyledons	Arecaceae	*Arenga*	*Arenga hastata*	JF345019
Monocotyledons	Arecaceae	*Arenga*	*Arenga hastata*	JF345024

Monocotyledons	Arecaceae	*Arenga*	*Arenga obtusifolia*	JF345032
Monocotyledons	Arecaceae	*Arenga*	*Arenga obtusifolia*	JF345033
Monocotyledons	Arecaceae	*Arenga*	*Arenga ryukyuensis*	JF345036
Monocotyledons	Arecaceae	*Arenga*	*Arenga ryukyuensis*	JF345037
Monocotyledons	Arecaceae	*Wallichia*	*Wallichia gracilis*	JF345072
Monocotyledons	Arecaceae	*Wallichia*	*Wallichia gracilis*	JF345075
Monocotyledons	Arecaceae	*Wallichia*	*Wallichia gracilis*	JF345076
Monocotyledons	Bromeliaceae	*Aechmea*	*Aechmea bicolor*	JN204597
Monocotyledons	Bromeliaceae	*Aechmea*	*Aechmea bicolor*	JN204598
Monocotyledons	Bromeliaceae	*Aechmea*	*Aechmea caudata*	JN204605
Monocotyledons	Bromeliaceae	*Aechmea*	*Aechmea caudata*	JN204606
Monocotyledons	Bromeliaceae	*Aechmea*	*Aechmea caudata*	JN204607
Monocotyledons	Bromeliaceae	*Aechmea*	*Aechmea curranii*	JN204610
Monocotyledons	Bromeliaceae	*Aechmea*	*Aechmea curranii*	JN204611
Monocotyledons	Bromeliaceae	*Aechmea*	*Aechmea curranii*	JN204612
Monocotyledons	Bromeliaceae	*Aechmea*	*Aechmea phanerophlebia*	JN204620
Monocotyledons	Bromeliaceae	*Aechmea*	*Aechmea phanerophlebia*	JN204621
Monocotyledons	Bromeliaceae	*Aechmea*	*Aechmea phanerophlebia*	JN204622
Monocotyledons	Bromeliaceae	*Billbergia*	*Billbergia distachya*	JN204628
Monocotyledons	Bromeliaceae	*Billbergia*	*Billbergia distachya*	JN204629
Monocotyledons	Bromeliaceae	*Billbergia*	*Billbergia chlorostica*	JN204633
Monocotyledons	Bromeliaceae	*Billbergia*	*Billbergia chlorostica*	JN204634
Monocotyledons	Bromeliaceae	*Billbergia*	*Billbergia chlorostica*	JN204635
Monocotyledons	Bromeliaceae	*Tillandsia*	*Tillandsia polystachia*	JN204665
Monocotyledons	Bromeliaceae	*Tillandsia*	*Tillandsia polystachia*	JN204666
Monocotyledons	Colchicaceae	*Colchicum*	*Colchicum arenarium*	JF934062
Monocotyledons	Colchicaceae	*Colchicum*	*Colchicum arenarium*	JF934063
Monocotyledons	Colchicaceae	*Colchicum*	*Colchicum atticum*	JF934066
Monocotyledons	Colchicaceae	*Colchicum*	*Colchicum atticum*	JF934067
Monocotyledons	Colchicaceae	*Colchicum*	*Colchicum bivonae*	JF934073
Monocotyledons	Colchicaceae	*Colchicum*	*Colchicum bivonae*	JF934074
Monocotyledons	Colchicaceae	*Colchicum*	*Colchicum boissieri*	JF934075
Monocotyledons	Colchicaceae	*Colchicum*	*Colchicum boissieri*	JF934076
Monocotyledons	Colchicaceae	*Colchicum*	*Colchicum freynii*	JF934103
Monocotyledons	Colchicaceae	*Colchicum*	*Colchicum freynii*	JF934104
Monocotyledons	Colchicaceae	*Colchicum*	*Colchicum ritchii*	JF934150
Monocotyledons	Colchicaceae	*Colchicum*	*Colchicum ritchii*	JF934151
Monocotyledons	Colchicaceae	*Colchicum*	*Colchicum schimperi*	JF934156
Monocotyledons	Colchicaceae	*Colchicum*	*Colchicum schimperi*	JF934157
Monocotyledons	Colchicaceae	*Colchicum*	*Colchicum trigynum*	JF934170
Monocotyledons	Colchicaceae	*Colchicum*	*Colchicum trigynum*	JF934171
Monocotyledons	Colchicaceae	*Colchicum*	*Colchicum triphyllum*	JF934172
Monocotyledons	Colchicaceae	*Colchicum*	*Colchicum triphyllum*	JF934173
Monocotyledons	Colchicaceae	*Colchicum*	*Colchicum triphyllum*	JF934174
Monocotyledons	Zosteraceae	*Zostera*	*Zostera marina*	DQ786516
Monocotyledons	Zosteraceae	*Zostera*	*Zostera marina*	JN225326
Monocotyledons	Zosteraceae	*Zostera*	*Zostera marina*	JN225327
Monocotyledons	Zosteraceae	*Zostera*	*Zostera marina*	JN225328
Monocotyledons	Iridaceae	*Iris*	*Iris germanica*	JN044979
Monocotyledons	Iridaceae	*Iris*	*Iris germanica*	JN044980
Monocotyledons	Iridaceae	*Iris*	*Iris chrysographes*	JN044960
Monocotyledons	Iridaceae	*Iris*	*Iris chrysographes*	JN044961
Monocotyledons	Iridaceae	*Iris*	*Iris chrysographes*	JN044962

Monocotyledons	Iridaceae	*Iris*	*Iris ensata*	JN044975
Monocotyledons	Iridaceae	*Iris*	*Iris ensata*	JN044976
Monocotyledons	Zosteraceae	*Zostera*	*Zostera noltei*	JN225329
Monocotyledons	Zosteraceae	*Zostera*	*Zostera noltei*	JN225330
Monocotyledons	Iridaceae	*Iris*	*Iris domestica*	GQ434907
Monocotyledons	Iridaceae	*Iris*	*Iris domestica*	GQ434908
Monocotyledons	Iridaceae	*Iris*	*Iris pseudacorus*	JN045000
Monocotyledons	Iridaceae	*Iris*	*Iris pseudacorus*	JN045001
Monocotyledons	Iridaceae	*Iris*	*Iris pseudacorus*	JN045002
Monocotyledons	Iridaceae	*Iris*	*Iris tectorum*	GQ435429
Monocotyledons	Iridaceae	*Iris*	*Iris tectorum*	GQ435430
Monocotyledons	Iridaceae	*Iris*	*Iris tectorum*	JN045015
Monocotyledons	Iridaceae	*Iris*	*Iris tectorum*	JN045016
Monocotyledons	Iridaceae	*Iris*	*Iris tectorum*	JN045017
Monocotyledons	Iridaceae	*Iris*	*Iris tectorum*	JN045018
Monocotyledons	Iridaceae	*Iris*	*Iris tectorum*	JN045019
Monocotyledons	Iridaceae	*Iris*	*Iris tectorum*	JN045020
Monocotyledons	Iridaceae	*Iris*	*Iris forrestii*	JN044977
Monocotyledons	Iridaceae	*Iris*	*Iris forrestii*	JN044978
Monocotyledons	Iridaceae	*Iris*	*Iris dichotoma*	JN044973
Monocotyledons	Iridaceae	*Iris*	*Iris dichotoma*	JN044974
Monocotyledons	Zosteraceae	*Zostera*	*Zostera muelleri*	GU906227
Monocotyledons	Zosteraceae	*Zostera*	*Zostera muelleri*	GU906228
Monocotyledons	Iridaceae	*Iris*	*Iris humilis*	FM253735
Monocotyledons	Iridaceae	*Iris*	*Iris humilis*	FM253736
Monocotyledons	Iridaceae	*Iris*	*Iris humilis*	FM253737
Monocotyledons	Iridaceae	*Iris*	*Iris humilis*	FM253738
Monocotyledons	Iridaceae	*Iris*	*Iris humilis*	FM253739
Monocotyledons	Iridaceae	*Iris*	*Iris humilis*	FM253740
Monocotyledons	Iridaceae	*Iris*	*Iris humilis*	FM253741
Monocotyledons	Iridaceae	*Iris*	*Iris humilis*	FM253742
Monocotyledons	Iridaceae	*Iris*	*Iris humilis*	FM253743
Monocotyledons	Iridaceae	*Iris*	*Iris humilis*	FM253744
Monocotyledons	Iridaceae	*Iris*	*Iris humilis*	FM253745
Monocotyledons	Iridaceae	*Iris*	*Iris humilis*	FM253746
Monocotyledons	Iridaceae	*Iris*	*Iris humilis*	FM253747
Monocotyledons	Iridaceae	*Iris*	*Iris lactea*	JN044989
Monocotyledons	Iridaceae	*Iris*	*Iris lactea*	JN044990
Monocotyledons	Iridaceae	*Iris*	*Iris lactea*	JN044991
Monocotyledons	Iridaceae	*Iris*	*Iris lactea*	JN044992
Monocotyledons	Iridaceae	*Iris*	*Iris loczyi*	JN044994
Monocotyledons	Iridaceae	*Iris*	*Iris loczyi*	JN044995
Monocotyledons	Iridaceae	*Iris*	*Iris loczyi*	JN044996
Monocotyledons	Iridaceae	*Iris*	*Iris ruthenica*	JN045005
Monocotyledons	Iridaceae	*Iris*	*Iris ruthenica*	JN045006
Monocotyledons	Iridaceae	*Iris*	*Iris ruthenica*	JN045007
Monocotyledons	Iridaceae	*Iris*	*Iris ruthenica*	JN045008
Monocotyledons	Iridaceae	*Iris*	*Iris ruthenica*	JN045009
Monocotyledons	Iridaceae	*Iris*	*Iris sanguinea*	JN045010
Monocotyledons	Iridaceae	*Iris*	*Iris sanguinea*	JN045011
Monocotyledons	Iridaceae	*Iris*	*Iris sanguinea*	JN045012
Monocotyledons	Iridaceae	*Iris*	*Iris tenuifolia*	JN045021
Monocotyledons	Iridaceae	*Iris*	*Iris tenuifolia*	JN045022

Monocotyledons	Iridaceae	*Iris*	*Iris tigridia*	JN045023
Monocotyledons	Iridaceae	*Iris*	*Iris tigridia*	JN045024
Monocotyledons	Iridaceae	*Iris*	*Iris uniflora*	JN045025
Monocotyledons	Iridaceae	*Iris*	*Iris uniflora*	JN045026
Monocotyledons	Iridaceae	*Iris*	*Iris ventricosa*	JN045028
Monocotyledons	Iridaceae	*Iris*	*Iris ventricosa*	JN045029
Monocotyledons	Zingiberaceae	*Amomum*	*Amomum glabrum*	JN043930
Monocotyledons	Zingiberaceae	*Amomum*	*Amomum glabrum*	JN043931
Monocotyledons	Zingiberaceae	*Amomum*	*Amomum longipetiolatum*	JN043938
Monocotyledons	Zingiberaceae	*Amomum*	*Amomum longipetiolatum*	JN043939
Monocotyledons	Zingiberaceae	*Amomum*	*Amomum villosum*	GQ118656
Monocotyledons	Zingiberaceae	*Amomum*	*Amomum villosum*	GU180423
Monocotyledons	Iridaceae	*Iris*	*Iris delavayi*	JN044971
Monocotyledons	Iridaceae	*Iris*	*Iris delavayi*	JN044972
Monocotyledons	Iridaceae	*Iris*	*Iris japonica*	JN044983
Monocotyledons	Iridaceae	*Iris*	*Iris japonica*	JN044984
Monocotyledons	Iridaceae	*Iris*	*Iris japonica*	JN044985
Monocotyledons	Iridaceae	*Iris*	*Iris japonica*	JN044986
Monocotyledons	Iridaceae	*Iris*	*Iris japonica*	JN044987
Monocotyledons	Iridaceae	*Iris*	*Iris japonica*	JN044988
Monocotyledons	Iridaceae	*Iris*	*Iris missouriensis*	EF434718
Monocotyledons	Iridaceae	*Iris*	*Iris missouriensis*	EF434719
Monocotyledons	Iridaceae	*Iris*	*Iris missouriensis*	EF434720
Monocotyledons	Iridaceae	*Iris*	*Iris missouriensis*	EF434721
Monocotyledons	Iridaceae	*Iris*	*Iris missouriensis*	EF434723
Monocotyledons	Iridaceae	*Iris*	*Iris missouriensis*	EF434724
Monocotyledons	Xanthorrhoeaceae	*Haworthia*	*Haworthia aristata*	HQ646850
Monocotyledons	Xanthorrhoeaceae	*Haworthia*	*Haworthia aristata*	HQ646851
Monocotyledons	Xanthorrhoeaceae	*Haworthia*	*Haworthia attenuata*	HQ646880
Monocotyledons	Xanthorrhoeaceae	*Haworthia*	*Haworthia attenuata*	HQ646881
Monocotyledons	Xanthorrhoeaceae	*Haworthia*	*Haworthia cymbiformis*	HQ646865
Monocotyledons	Xanthorrhoeaceae	*Haworthia*	*Haworthia cymbiformis*	HQ646866
Monocotyledons	Xanthorrhoeaceae	*Haworthia*	*Haworthia cooperi*	HQ646854
Monocotyledons	Xanthorrhoeaceae	*Haworthia*	*Haworthia cooperi*	HQ646855
Monocotyledons	Xanthorrhoeaceae	*Haworthia*	*Haworthia cooperi*	HQ646856
Monocotyledons	Xanthorrhoeaceae	*Haworthia*	*Haworthia cooperi*	HQ646857
Monocotyledons	Xanthorrhoeaceae	*Haworthia*	*Haworthia cooperi*	HQ646858
Monocotyledons	Xanthorrhoeaceae	*Haworthia*	*Haworthia cooperi*	HQ646859
Monocotyledons	Xanthorrhoeaceae	*Haworthia*	*Haworthia cooperi*	HQ646860
Monocotyledons	Xanthorrhoeaceae	*Haworthia*	*Haworthia cooperi*	HQ646861
Monocotyledons	Xanthorrhoeaceae	*Haworthia*	*Haworthia cooperi*	HQ646862
Monocotyledons	Xanthorrhoeaceae	*Haworthia*	*Haworthia cooperi*	HQ646863
Monocotyledons	Xanthorrhoeaceae	*Haworthia*	*Haworthia cooperi*	HQ646864
Monocotyledons	Xanthorrhoeaceae	*Haworthia*	*Haworthia glauca*	HQ646884
Monocotyledons	Xanthorrhoeaceae	*Haworthia*	*Haworthia glauca*	HQ646885
Monocotyledons	Xanthorrhoeaceae	*Haworthia*	*Haworthia glauca*	HQ646886
Monocotyledons	Zingiberaceae	*Amomum*	*Amomum compactum*	GQ118660
Monocotyledons	Zingiberaceae	*Amomum*	*Amomum compactum*	JN043924
Monocotyledons	Zingiberaceae	*Amomum*	*Amomum compactum*	JN043925
Monocotyledons	Zingiberaceae	*Amomum*	*Amomum compactum*	JN043926
Monocotyledons	Zingiberaceae	*Amomum*	*Amomum koenigii*	JN043932
Monocotyledons	Zingiberaceae	*Amomum*	*Amomum koenigii*	JN043933
Monocotyledons	Zingiberaceae	*Amomum*	*Amomum koenigii*	JN043934

Monocotyledons	Zingiberaceae	*Amomum*	*Amomum koenigii*	JN043935
Monocotyledons	Zingiberaceae	*Amomum*	*Amomum maximum*	JN043940
Monocotyledons	Zingiberaceae	*Amomum*	*Amomum maximum*	JN043941
Monocotyledons	Zingiberaceae	*Amomum*	*Amomum maximum*	JN043942
Monocotyledons	Zingiberaceae	*Amomum*	*Amomum menglaense*	JN043943
Monocotyledons	Zingiberaceae	*Amomum*	*Amomum menglaense*	JN043944
Monocotyledons	Zingiberaceae	*Amomum*	*Amomum menglaense*	JN043945
Monocotyledons	Zingiberaceae	*Amomum*	*Amomum menglaense*	JN043946
Monocotyledons	Zingiberaceae	*Amomum*	*Amomum purpureorubrum*	JN043959
Monocotyledons	Zingiberaceae	*Amomum*	*Amomum purpureorubrum*	JN043960
Monocotyledons	Zingiberaceae	*Amomum*	*Amomum quadratolaminare*	JN043961
Monocotyledons	Zingiberaceae	*Amomum*	*Amomum quadratolaminare*	JN043962
Monocotyledons	Zingiberaceae	*Amomum*	*Amomum quadratolaminare*	JN043963
Monocotyledons	Zingiberaceae	*Amomum*	*Amomum quadratolaminare*	JN043964
Monocotyledons	Zingiberaceae	*Amomum*	*Amomum quadratolaminare*	JN043965
Monocotyledons	Zingiberaceae	*Amomum*	*Amomum sericeum*	JN043972
Monocotyledons	Zingiberaceae	*Amomum*	*Amomum sericeum*	JN043973
Monocotyledons	Zingiberaceae	*Amomum*	*Amomum subcapitatum*	JN043974
Monocotyledons	Zingiberaceae	*Amomum*	*Amomum subcapitatum*	JN043975
Monocotyledons	Orchidaceae	*Rhynchostylis*	*Rhynchostylis retusa*	GQ251332
Monocotyledons	Orchidaceae	*Rhynchostylis*	*Rhynchostylis retusa*	GQ251333
Monocotyledons	Orchidaceae	*Rhynchostylis*	*Rhynchostylis retusa*	GQ251334
Monocotyledons	Orchidaceae	*Rhynchostylis*	*Rhynchostylis gigantea*	GQ251323
Monocotyledons	Orchidaceae	*Rhynchostylis*	*Rhynchostylis gigantea*	GQ251324
Monocotyledons	Orchidaceae	*Rhynchostylis*	*Rhynchostylis gigantea*	GQ251325
Monocotyledons	Orchidaceae	*Rhynchostylis*	*Rhynchostylis gigantea*	GQ251326
Monocotyledons	Orchidaceae	*Rhynchostylis*	*Rhynchostylis gigantea*	GQ251327
Monocotyledons	Orchidaceae	*Rhynchostylis*	*Rhynchostylis gigantea*	GQ251328
Monocotyledons	Orchidaceae	*Rhynchostylis*	*Rhynchostylis gigantea*	GQ251329
Monocotyledons	Orchidaceae	*Rhynchostylis*	*Rhynchostylis gigantea*	GQ251330
Monocotyledons	Orchidaceae	*Rhynchostylis*	*Rhynchostylis gigantea*	GQ251331
Monocotyledons	Iridaceae	*Iris*	*Iris wattii*	JN045030
Monocotyledons	Iridaceae	*Iris*	*Iris wattii*	JN045031
Monocotyledons	Iridaceae	*Iris*	*Iris collettii*	JN044963
Monocotyledons	Iridaceae	*Iris*	*Iris collettii*	JN044964
Monocotyledons	Iridaceae	*Iris*	*Iris collettii*	JN044965
Monocotyledons	Zingiberaceae	*Amomum*	*Amomum petaloideum*	JN043955
Monocotyledons	Zingiberaceae	*Amomum*	*Amomum petaloideum*	JN043956
Monocotyledons	Zingiberaceae	*Amomum*	*Amomum petaloideum*	JN043957
Monocotyledons	Zingiberaceae	*Amomum*	*Amomum petaloideum*	JN043958
Monocotyledons	Iridaceae	*Iris*	*Iris mandshurica*	FM253719
Monocotyledons	Iridaceae	*Iris*	*Iris mandshurica*	FM253720
Monocotyledons	Iridaceae	*Iris*	*Iris mandshurica*	FM253721
Monocotyledons	Iridaceae	*Iris*	*Iris mandshurica*	FM253722
Monocotyledons	Iridaceae	*Iris*	*Iris mandshurica*	FM253723
Monocotyledons	Iridaceae	*Iris*	*Iris mandshurica*	FM253724
Monocotyledons	Iridaceae	*Iris*	*Iris mandshurica*	FM253725
Monocotyledons	Iridaceae	*Iris*	*Iris mandshurica*	FM253726
Monocotyledons	Iridaceae	*Iris*	*Iris mandshurica*	FM253727
Monocotyledons	Iridaceae	*Iris*	*Iris mandshurica*	FM253728
Monocotyledons	Iridaceae	*Iris*	*Iris mandshurica*	FM253729
Monocotyledons	Iridaceae	*Iris*	*Iris mandshurica*	FM253730
Monocotyledons	Iridaceae	*Iris*	*Iris mandshurica*	FM253731

Monocotyledons	Iridaceae	*Iris*	*Iris mandshurica*	FM253732
Monocotyledons	Iridaceae	*Iris*	*Iris mandshurica*	FM253733
Monocotyledons	Iridaceae	*Iris*	*Iris mandshurica*	FM253734
Monocotyledons	Orchidaceae	*Aulosepalum*	*Aulosepalum pyramidale*	AM884884
Monocotyledons	Orchidaceae	*Aulosepalum*	*Aulosepalum pyramidale*	AM884885
Monocotyledons	Orchidaceae	*Aulosepalum*	*Aulosepalum ramentaceum*	AM884882
Monocotyledons	Orchidaceae	*Aulosepalum*	*Aulosepalum ramentaceum*	AM884883
Monocotyledons	Orchidaceae	*Aulosepalum*	*Aulosepalum oestlundii*	AM884886
Monocotyledons	Orchidaceae	*Aulosepalum*	*Aulosepalum oestlundii*	AM884887
Monocotyledons	Orchidaceae	*Aulosepalum*	*Aulosepalum hemichreum*	AM884889
Monocotyledons	Orchidaceae	*Aulosepalum*	*Aulosepalum hemichreum*	AM884890
Monocotyledons	Orchidaceae	*Holcoglossum*	*Holcoglossum flavescens*	HQ404442
Monocotyledons	Orchidaceae	*Holcoglossum*	*Holcoglossum flavescens*	HQ404443
Monocotyledons	Orchidaceae	*Holcoglossum*	*Holcoglossum flavescens*	HQ404444
Monocotyledons	Orchidaceae	*Holcoglossum*	*Holcoglossum flavescens*	HQ404445
Monocotyledons	Orchidaceae	*Holcoglossum*	*Holcoglossum flavescens*	HQ404446
Monocotyledons	Orchidaceae	*Holcoglossum*	*Holcoglossum lingulatum*	HQ404453
Monocotyledons	Orchidaceae	*Holcoglossum*	*Holcoglossum lingulatum*	HQ404454
Monocotyledons	Orchidaceae	*Holcoglossum*	*Holcoglossum lingulatum*	HQ404455
Monocotyledons	Orchidaceae	*Holcoglossum*	*Holcoglossum nujiangense*	HQ404456
Monocotyledons	Orchidaceae	*Holcoglossum*	*Holcoglossum nujiangense*	HQ404457
Monocotyledons	Orchidaceae	*Holcoglossum*	*Holcoglossum nujiangense*	HQ404458
Monocotyledons	Orchidaceae	*Holcoglossum*	*Holcoglossum nujiangense*	HQ404459
Monocotyledons	Orchidaceae	*Holcoglossum*	*Holcoglossum nujiangense*	HQ404460
Monocotyledons	Orchidaceae	*Holcoglossum*	*Holcoglossum rupestre*	HQ404467
Monocotyledons	Orchidaceae	*Holcoglossum*	*Holcoglossum rupestre*	HQ404468
Monocotyledons	Orchidaceae	*Holcoglossum*	*Holcoglossum rupestre*	HQ404469
Monocotyledons	Orchidaceae	*Holcoglossum*	*Holcoglossum rupestre*	HQ404470
Monocotyledons	Orchidaceae	*Holcoglossum*	*Holcoglossum rupestre*	HQ404471
Monocotyledons	Orchidaceae	*Holcoglossum*	*Holcoglossum sinicum*	HQ404472
Monocotyledons	Orchidaceae	*Holcoglossum*	*Holcoglossum sinicum*	HQ404473
Monocotyledons	Orchidaceae	*Holcoglossum*	*Holcoglossum sinicum*	HQ404474
Monocotyledons	Orchidaceae	*Holcoglossum*	*Holcoglossum sinicum*	HQ404475
Monocotyledons	Orchidaceae	*Holcoglossum*	*Holcoglossum sinicum*	HQ404476
Monocotyledons	Orchidaceae	*Holcoglossum*	*Holcoglossum weixiense*	HQ404483
Monocotyledons	Orchidaceae	*Holcoglossum*	*Holcoglossum weixiense*	HQ404484
Monocotyledons	Orchidaceae	*Holcoglossum*	*Holcoglossum weixiense*	HQ404485
Monocotyledons	Orchidaceae	*Holcoglossum*	*Holcoglossum weixiense*	HQ404486
Monocotyledons	Orchidaceae	*Holcoglossum*	*Holcoglossum weixiense*	HQ404487
Monocotyledons	Iridaceae	*Iris*	*Iris vorobievii*	FM253702
Monocotyledons	Iridaceae	*Iris*	*Iris vorobievii*	FM253703
Monocotyledons	Iridaceae	*Iris*	*Iris vorobievii*	FM253704
Monocotyledons	Iridaceae	*Iris*	*Iris vorobievii*	FM253705
Monocotyledons	Iridaceae	*Iris*	*Iris vorobievii*	FM253706
Monocotyledons	Iridaceae	*Iris*	*Iris vorobievii*	FM253707
Monocotyledons	Iridaceae	*Iris*	*Iris vorobievii*	FM253708
Monocotyledons	Iridaceae	*Iris*	*Iris vorobievii*	FM253709
Monocotyledons	Iridaceae	*Iris*	*Iris vorobievii*	FM253710
Monocotyledons	Iridaceae	*Iris*	*Iris vorobievii*	FM253711
Monocotyledons	Iridaceae	*Iris*	*Iris vorobievii*	FM253712
Monocotyledons	Iridaceae	*Iris*	*Iris vorobievii*	FM253713
Monocotyledons	Iridaceae	*Iris*	*Iris vorobievii*	FM253714
Monocotyledons	Iridaceae	*Iris*	*Iris vorobievii*	FM253715

Monocotyledons	Iridaceae	*Iris*	*Iris vorobievii*	FM253716
Monocotyledons	Iridaceae	*Iris*	*Iris vorobievii*	FM253717
Monocotyledons	Iridaceae	*Iris*	*Iris vorobievii*	FM253718
Monocotyledons	Eriocaulaceae	*Paepalanthus*	*Paepalanthus lamarckii*	EU924377
Monocotyledons	Eriocaulaceae	*Paepalanthus*	*Paepalanthus lamarckii*	HQ843070
Monocotyledons	Iridaceae	*Iris*	*Iris bulleyana*	JN044956
Monocotyledons	Iridaceae	*Iris*	*Iris bulleyana*	JN044957
Monocotyledons	Iridaceae	*Iris*	*Iris bulleyana*	JN044958
Monocotyledons	Iridaceae	*Iris*	*Iris decora*	JN044969
Monocotyledons	Iridaceae	*Iris*	*Iris decora*	JN044970
Monocotyledons	Zingiberaceae	*Amomum*	*Amomum longiligulare*	GQ118658
Monocotyledons	Zingiberaceae	*Amomum*	*Amomum longiligulare*	GQ464983
Monocotyledons	Zingiberaceae	*Amomum*	*Amomum longiligulare*	GU180431
Monocotyledons	Zingiberaceae	*Amomum*	*Amomum krervanh*	GQ118657
Monocotyledons	Zingiberaceae	*Amomum*	*Amomum krervanh*	JN043936
Monocotyledons	Zingiberaceae	*Amomum*	*Amomum krervanh*	JN043937
Monocotyledons	Orchidaceae	*Rhynchostylis*	*Rhynchostylis coelestis*	GQ251335
Monocotyledons	Orchidaceae	*Rhynchostylis*	*Rhynchostylis coelestis*	GQ251336
Monocotyledons	Orchidaceae	*Rhynchostylis*	*Rhynchostylis coelestis*	GQ251337
Monocotyledons	Iridaceae	*Iris*	*Iris rossii*	JN045003
Monocotyledons	Iridaceae	*Iris*	*Iris rossii*	JN045004
Monocotyledons	Eriocaulaceae	*Paepalanthus*	*Paepalanthus sp. MJGA-2011*	HQ843079
Monocotyledons	Eriocaulaceae	*Paepalanthus*	*Paepalanthus sp. MJGA-2011*	HQ843080
Monocotyledons	Eriocaulaceae	*Paepalanthus*	*Paepalanthus sp. MJGA-2011*	HQ843081
Monocotyledons	Eriocaulaceae	*Paepalanthus*	*Paepalanthus sp. MJGA-2011*	HQ843082
Monocotyledons	Orchidaceae	*Holcoglossum*	*Holcoglossum quasipinifolium*	HQ404462
Monocotyledons	Orchidaceae	*Holcoglossum*	*Holcoglossum quasipinifolium*	HQ404463
Monocotyledons	Orchidaceae	*Holcoglossum*	*Holcoglossum quasipinifolium*	HQ404464
Monocotyledons	Orchidaceae	*Holcoglossum*	*Holcoglossum quasipinifolium*	HQ404465
Monocotyledons	Orchidaceae	*Holcoglossum*	*Holcoglossum quasipinifolium*	HQ404466
Monocotyledons	Iridaceae	*Iris*	*Iris anguifuga*	JN044954
Monocotyledons	Iridaceae	*Iris*	*Iris anguifuga*	JN044955
Monocotyledons	Iridaceae	*Iris*	*Iris confusa*	JN044966
Monocotyledons	Iridaceae	*Iris*	*Iris confusa*	JN044967
Monocotyledons	Iridaceae	*Iris*	*Iris confusa*	JN044968
Monocotyledons	Iridaceae	*Iris*	*Iris goniocarpa*	JN044981
Monocotyledons	Iridaceae	*Iris*	*Iris goniocarpa*	JN044982
Monocotyledons	Iridaceae	*Iris*	*Iris speculatrix*	JN045013
Monocotyledons	Iridaceae	*Iris*	*Iris speculatrix*	JN045014
Monocotyledons	Zingiberaceae	*Amomum*	*Amomum dealbatum*	JN043927
Monocotyledons	Zingiberaceae	*Amomum*	*Amomum dealbatum*	JN043928
Monocotyledons	Zingiberaceae	*Amomum*	*Amomum dealbatum*	JN043929
Monocotyledons	Zingiberaceae	*Amomum*	*Amomum microcarpum*	JN043947
Monocotyledons	Zingiberaceae	*Amomum*	*Amomum microcarpum*	JN043948
Monocotyledons	Zingiberaceae	*Amomum*	*Amomum muricarpum*	JN043949
Monocotyledons	Zingiberaceae	*Amomum*	*Amomum muricarpum*	JN043950
Monocotyledons	Zingiberaceae	*Amomum*	*Amomum muricarpum*	JN043951
Monocotyledons	Zingiberaceae	*Amomum*	*Amomum neoaurantiacum*	JN043952
Monocotyledons	Zingiberaceae	*Amomum*	*Amomum neoaurantiacum*	JN043953
Monocotyledons	Zingiberaceae	*Amomum*	*Amomum neoaurantiacum*	JN043954
Monocotyledons	Zingiberaceae	*Amomum*	*Amomum repoeense*	JN043966
Monocotyledons	Zingiberaceae	*Amomum*	*Amomum repoeense*	JN043967
Monocotyledons	Zingiberaceae	*Amomum*	*Amomum repoeense*	JN043968

Monocotyledons	Zingiberaceae	*Amomum*	*Amomum repoeense*	JN043969
Monocotyledons	Zingiberaceae	*Amomum*	*Amomum verrucosum*	JN043976
Monocotyledons	Zingiberaceae	*Amomum*	*Amomum verrucosum*	JN043977
Monocotyledons	Zingiberaceae	*Amomum*	*Amomum verrucosum*	JN043978
Monocotyledons	Zingiberaceae	*Amomum*	*Amomum scarlatinum*	JN043970
Monocotyledons	Zingiberaceae	*Amomum*	*Amomum scarlatinum*	JN043971
Monocotyledons	Xanthorrhoeaceae	*Haworthia*	*Haworthia mucronata*	HQ646870
Monocotyledons	Xanthorrhoeaceae	*Haworthia*	*Haworthia mucronata*	HQ646871
Monocotyledons	Xanthorrhoeaceae	*Haworthia*	*Haworthia pulchella*	HQ646874
Monocotyledons	Xanthorrhoeaceae	*Haworthia*	*Haworthia pulchella*	HQ646875
Monocotyledons	Xanthorrhoeaceae	*Haworthia*	*Haworthia rossouwii*	HQ646877
Monocotyledons	Xanthorrhoeaceae	*Haworthia*	*Haworthia rossouwii*	HQ646878
Monocotyledons	Xanthorrhoeaceae	*Haworthia*	*Haworthia venosa*	HQ646890
Monocotyledons	Xanthorrhoeaceae	*Haworthia*	*Haworthia venosa*	HQ646891
Monocotyledons	Xanthorrhoeaceae	*Haworthia*	*Haworthia venosa*	HQ646892
Monocotyledons	Araceae	*Alocasia*	*Alocasia macrorrhizos*	JN406926
Monocotyledons	Araceae	*Alocasia*	*Alocasia macrorrhizos*	JN406927
Monocotyledons	Araceae	*Alocasia*	*Alocasia macrorrhizos*	JN406928
Monocotyledons	Araceae	*Alocasia*	*Alocasia macrorrhizos*	JN406929
Monocotyledons	Araceae	*Alocasia*	*Alocasia macrorrhizos*	JF828142
Monocotyledons	Araceae	*Colocasia*	*Colocasia esculenta*	GU135448
Monocotyledons	Araceae	*Colocasia*	*Colocasia esculenta*	JF828139
Monocotyledons	Acoraceae	*Acorus*	*Acorus calamus*	GQ248235
Monocotyledons	Acoraceae	*Acorus*	*Acorus calamus*	GQ435387
Monocotyledons	Acoraceae	*Acorus*	*Acorus calamus*	FJ874970
Monocotyledons	Acoraceae	*Acorus*	*Acorus calamus*	FJ874971
Monocotyledons	Acoraceae	*Acorus*	*Acorus calamus*	FJ874972
Monocotyledons	Acoraceae	*Acorus*	*Acorus calamus*	FJ874973
Monocotyledons	Acoraceae	*Acorus*	*Acorus calamus*	FJ874974
Monocotyledons	Acoraceae	*Acorus*	*Acorus calamus*	FJ874975
Monocotyledons	Acoraceae	*Acorus*	*Acorus calamus*	FJ874976
Monocotyledons	Acoraceae	*Acorus*	*Acorus calamus*	FJ874977
Monocotyledons	Acoraceae	*Acorus*	*Acorus calamus*	DQ008894
Monocotyledons	Acoraceae	*Acorus*	*Acorus calamus*	DQ008899
Monocotyledons	Acoraceae	*Acorus*	*Acorus calamus*	EU814673
Monocotyledons	Acoraceae	*Acorus*	*Acorus calamus*	EU814674
Monocotyledons	Acoraceae	*Acorus*	*Acorus calamus*	EU814675
Monocotyledons	Acoraceae	*Acorus*	*Acorus calamus*	EU814676
Monocotyledons	Acoraceae	*Acorus*	*Acorus calamus*	JF708224
Monocotyledons	Araceae	*Lemna*	*Lemna gibba*	GU454504
Monocotyledons	Araceae	*Lemna*	*Lemna gibba*	GU454505
Monocotyledons	Araceae	*Lemna*	*Lemna gibba*	GU454506
Monocotyledons	Araceae	*Lemna*	*Lemna gibba*	GU454507
Monocotyledons	Araceae	*Lemna*	*Lemna gibba*	GU454508
Monocotyledons	Araceae	*Lemna*	*Lemna gibba*	GU454509
Monocotyledons	Araceae	*Lemna*	*Lemna minor*	GU454511
Monocotyledons	Araceae	*Lemna*	*Lemna minor*	GU454512
Monocotyledons	Araceae	*Lemna*	*Lemna minor*	GU454513
Monocotyledons	Araceae	*Lemna*	*Lemna minor*	GU454514
Monocotyledons	Araceae	*Lemna*	*Lemna minor*	GU454515
Monocotyledons	Araceae	*Lemna*	*Lemna minor*	GU454516
Monocotyledons	Araceae	*Lemna*	*Lemna minor*	FJ395556
Monocotyledons	Araceae	*Lemna*	*Lemna minor*	GQ434996

Monocotyledons	Poaceae	*Oryza*	*Oryza rufipogon*	GU575244
Monocotyledons	Poaceae	*Oryza*	*Oryza rufipogon*	GU575248
Monocotyledons	Poaceae	*Oryza*	*Oryza rufipogon*	GU575259
Monocotyledons	Poaceae	*Oryza*	*Oryza rufipogon*	GU575260
Monocotyledons	Poaceae	*Oryza*	*Oryza sativa*	GU575253
Monocotyledons	Poaceae	*Oryza*	*Oryza sativa*	GU575271
Monocotyledons	Poaceae	*Oryza*	*Oryza sativa*	GU575284
Monocotyledons	Poaceae	*Oryza*	*Oryza sativa*	GQ435008
Monocotyledons	Poaceae	*Panicum*	*Panicum miliaceum*	HQ596781
Monocotyledons	Poaceae	*Panicum*	*Panicum miliaceum*	FR667854
Monocotyledons	Poaceae	*Poa*	*Poa pratensis*	FJ395514
Monocotyledons	Poaceae	*Poa*	*Poa pratensis*	HQ596794
Monocotyledons	Poaceae	*Poa*	*Poa pratensis*	FJ766223
Monocotyledons	Poaceae	*Bambusa*	*Bambusa multiplex*	GU390999
Monocotyledons	Poaceae	*Bambusa*	*Bambusa multiplex*	GU063085
Monocotyledons	Poaceae	*Thinopyrum*	*Thinopyrum bessarabicum*	HQ221789
Monocotyledons	Poaceae	*Thinopyrum*	*Thinopyrum bessarabicum*	HQ221813
Monocotyledons	Poaceae	*Thinopyrum*	*Thinopyrum bessarabicum*	HQ652840
Monocotyledons	Poaceae	*Pseudoroegneria*	*Pseudoroegneria spicata*	HQ221770
Monocotyledons	Poaceae	*Pseudoroegneria*	*Pseudoroegneria spicata*	HQ652795
Monocotyledons	Araceae	*Spirodela*	*Spirodela polyrhiza*	GU454487
Monocotyledons	Araceae	*Spirodela*	*Spirodela polyrhiza*	GU454488
Monocotyledons	Araceae	*Spirodela*	*Spirodela polyrhiza*	GU454489
Monocotyledons	Araceae	*Spirodela*	*Spirodela polyrhiza*	GU454490
Monocotyledons	Araceae	*Spirodela*	*Spirodela polyrhiza*	GU454491
Monocotyledons	Araceae	*Spirodela*	*Spirodela polyrhiza*	GU454492
Monocotyledons	Araceae	*Spirodela*	*Spirodela polyrhiza*	GU454493
Monocotyledons	Poaceae	*Cenchrus*	*Cenchrus ciliaris*	HQ876941
Monocotyledons	Poaceae	*Cenchrus*	*Cenchrus ciliaris*	HQ876942
Monocotyledons	Poaceae	*Glyceria*	*Glyceria borealis*	DQ665463
Monocotyledons	Poaceae	*Glyceria*	*Glyceria borealis*	DQ665464
Monocotyledons	Poaceae	*Glyceria*	*Glyceria borealis*	DQ665465
Monocotyledons	Poaceae	*Glyceria*	*Glyceria borealis*	DQ665466
Monocotyledons	Poaceae	*Glyceria*	*Glyceria borealis*	DQ665467
Monocotyledons	Poaceae	*Glyceria*	*Glyceria borealis*	DQ665468
Monocotyledons	Poaceae	*Glyceria*	*Glyceria borealis*	DQ665469
Monocotyledons	Poaceae	*Glyceria*	*Glyceria borealis*	DQ665470
Monocotyledons	Poaceae	*Glyceria*	*Glyceria striata*	DQ665535
Monocotyledons	Poaceae	*Glyceria*	*Glyceria striata*	DQ665536
Monocotyledons	Poaceae	*Glyceria*	*Glyceria striata*	DQ665537
Monocotyledons	Poaceae	*Glyceria*	*Glyceria striata*	DQ665538
Monocotyledons	Poaceae	*Glyceria*	*Glyceria striata*	DQ665539
Monocotyledons	Poaceae	*Glyceria*	*Glyceria striata*	DQ665540
Monocotyledons	Poaceae	*Glyceria*	*Glyceria striata*	DQ665541
Monocotyledons	Poaceae	*Glyceria*	*Glyceria striata*	DQ665542
Monocotyledons	Poaceae	*Glyceria*	*Glyceria striata*	DQ665543
Monocotyledons	Poaceae	*Glyceria*	*Glyceria striata*	DQ665544
Monocotyledons	Poaceae	*Glyceria*	*Glyceria striata*	DQ665545
Monocotyledons	Poaceae	*Glyceria*	*Glyceria striata*	DQ665546
Monocotyledons	Poaceae	*Glyceria*	*Glyceria striata*	HQ596721
Monocotyledons	Poaceae	*Glyceria*	*Glyceria striata*	HQ596722
Monocotyledons	Poaceae	*Panicum*	*Panicum virgatum*	JF901575
Monocotyledons	Poaceae	*Panicum*	*Panicum virgatum*	JF901576

Monocotyledons	Dioscoreaceae	*Tacca*	*Tacca plantaginea*	JN850592
Monocotyledons	Dioscoreaceae	*Tacca*	*Tacca plantaginea*	JN047352
Monocotyledons	Dioscoreaceae	*Tacca*	*Tacca plantaginea*	JN047353
Monocotyledons	Dioscoreaceae	*Tacca*	*Tacca plantaginea*	JN047354
Monocotyledons	Dioscoreaceae	*Tacca*	*Tacca plantaginea*	JN047355
Monocotyledons	Dioscoreaceae	*Tacca*	*Tacca plantaginea*	JN047356
Monocotyledons	Dioscoreaceae	*Tacca*	*Tacca plantaginea*	JN047357
Monocotyledons	Dioscoreaceae	*Tacca*	*Tacca plantaginea*	JN047358
Monocotyledons	Poaceae	*Eragrostis*	*Eragrostis cilianensis*	HQ876961
Monocotyledons	Poaceae	*Eragrostis*	*Eragrostis cilianensis*	HQ876962
Monocotyledons	Poaceae	*Eragrostis*	*Eragrostis cilianensis*	HQ876963
Monocotyledons	Poaceae	*Eragrostis*	*Eragrostis cilianensis*	HQ876964
Monocotyledons	Araceae	*Spirodela*	*Spirodela intermedia*	GU454484
Monocotyledons	Araceae	*Spirodela*	*Spirodela intermedia*	GU454485
Monocotyledons	Araceae	*Spirodela*	*Spirodela intermedia*	GU454486
Monocotyledons	Poaceae	*Glyceria*	*Glyceria declinata*	DQ665475
Monocotyledons	Poaceae	*Glyceria*	*Glyceria declinata*	DQ665476
Monocotyledons	Poaceae	*Glyceria*	*Glyceria declinata*	DQ665477
Monocotyledons	Poaceae	*Glyceria*	*Glyceria declinata*	DQ665478
Monocotyledons	Poaceae	*Glyceria*	*Glyceria declinata*	DQ665479
Monocotyledons	Poaceae	*Glyceria*	*Glyceria declinata*	DQ665480
Monocotyledons	Poaceae	*Glyceria*	*Glyceria declinata*	DQ665481
Monocotyledons	Poaceae	*Glyceria*	*Glyceria declinata*	DQ665482
Monocotyledons	Poaceae	*Glyceria*	*Glyceria declinata*	DQ665483
Monocotyledons	Poaceae	*Glyceria*	*Glyceria declinata*	DQ665484
Monocotyledons	Orchidaceae	*Cypripedium*	*Cypripedium calceolus*	GQ248281
Monocotyledons	Orchidaceae	*Cypripedium*	*Cypripedium calceolus*	JF796982
Monocotyledons	Orchidaceae	*Cypripedium*	*Cypripedium flavum*	JQ004986
Monocotyledons	Orchidaceae	*Cypripedium*	*Cypripedium flavum*	JF796948
Monocotyledons	Acoraceae	*Acorus*	*Acorus gramineus*	GQ248236
Monocotyledons	Acoraceae	*Acorus*	*Acorus gramineus*	FJ874978
Monocotyledons	Acoraceae	*Acorus*	*Acorus gramineus*	FJ874979
Monocotyledons	Acoraceae	*Acorus*	*Acorus gramineus*	FJ874980
Monocotyledons	Acoraceae	*Acorus*	*Acorus gramineus*	FJ874981
Monocotyledons	Acoraceae	*Acorus*	*Acorus gramineus*	FJ874982
Monocotyledons	Acoraceae	*Acorus*	*Acorus gramineus*	DQ008892
Monocotyledons	Acoraceae	*Acorus*	*Acorus gramineus*	DQ008895
Monocotyledons	Acoraceae	*Acorus*	*Acorus gramineus*	DQ008898
Monocotyledons	Acoraceae	*Acorus*	*Acorus gramineus*	EU814677
Monocotyledons	Acoraceae	*Acorus*	*Acorus gramineus*	EU814679
Monocotyledons	Poaceae	*Bambusa*	*Bambusa vulgaris*	FJ644251
Monocotyledons	Poaceae	*Bambusa*	*Bambusa vulgaris*	GU063097
Monocotyledons	Poaceae	*Bambusa*	*Bambusa vulgaris*	EF589631
Monocotyledons	Poaceae	*Jarava*	*Jarava ichu*	EU489267
Monocotyledons	Poaceae	*Jarava*	*Jarava ichu*	EU489268
Monocotyledons	Dioscoreaceae	*Tacca*	*Tacca leontopetaloides*	JN850588
Monocotyledons	Dioscoreaceae	*Tacca*	*Tacca leontopetaloides*	JN047344
Monocotyledons	Dioscoreaceae	*Tacca*	*Tacca leontopetaloides*	JN047345
Monocotyledons	Dioscoreaceae	*Tacca*	*Tacca leontopetaloides*	JN047346
Monocotyledons	Dioscoreaceae	*Tacca*	*Tacca leontopetaloides*	JN047347
Monocotyledons	Dioscoreaceae	*Tacca*	*Tacca leontopetaloides*	JN047348
Monocotyledons	Dioscoreaceae	*Tacca*	*Tacca leontopetaloides*	JN047349
Monocotyledons	Dioscoreaceae	*Tacca*	*Tacca leontopetaloides*	JN047350

Monocotyledons	Dioscoreaceae	*Tacca*	*Tacca leontopetaloides*	JN047351
Monocotyledons	Poaceae	*Glyceria*	*Glyceria grandis*	DQ665507
Monocotyledons	Poaceae	*Glyceria*	*Glyceria grandis*	DQ665508
Monocotyledons	Poaceae	*Glyceria*	*Glyceria grandis*	DQ665509
Monocotyledons	Poaceae	*Glyceria*	*Glyceria grandis*	HQ596720
Monocotyledons	Dioscoreaceae	*Tacca*	*Tacca chantieri*	JN850585
Monocotyledons	Dioscoreaceae	*Tacca*	*Tacca chantieri*	EF590744
Monocotyledons	Dioscoreaceae	*Tacca*	*Tacca chantieri*	JN047324
Monocotyledons	Dioscoreaceae	*Tacca*	*Tacca chantieri*	JN047325
Monocotyledons	Dioscoreaceae	*Tacca*	*Tacca chantieri*	JN047326
Monocotyledons	Dioscoreaceae	*Tacca*	*Tacca chantieri*	JN047327
Monocotyledons	Dioscoreaceae	*Tacca*	*Tacca chantieri*	JN047328
Monocotyledons	Poaceae	*Thinopyrum*	*Thinopyrum intermedium*	HQ221771
Monocotyledons	Poaceae	*Thinopyrum*	*Thinopyrum intermedium*	HQ221772
Monocotyledons	Poaceae	*Thinopyrum*	*Thinopyrum intermedium*	HQ221773
Monocotyledons	Poaceae	*Thinopyrum*	*Thinopyrum intermedium*	HQ221774
Monocotyledons	Poaceae	*Thinopyrum*	*Thinopyrum intermedium*	HQ221775
Monocotyledons	Poaceae	*Thinopyrum*	*Thinopyrum intermedium*	HQ221776
Monocotyledons	Poaceae	*Thinopyrum*	*Thinopyrum intermedium*	HQ221777
Monocotyledons	Poaceae	*Thinopyrum*	*Thinopyrum intermedium*	HQ221778
Monocotyledons	Poaceae	*Thinopyrum*	*Thinopyrum intermedium*	HQ221779
Monocotyledons	Poaceae	*Thinopyrum*	*Thinopyrum intermedium*	HQ221780
Monocotyledons	Poaceae	*Thinopyrum*	*Thinopyrum intermedium*	HQ221781
Monocotyledons	Poaceae	*Thinopyrum*	*Thinopyrum intermedium*	HQ221782
Monocotyledons	Poaceae	*Thinopyrum*	*Thinopyrum intermedium*	HQ221783
Monocotyledons	Poaceae	*Thinopyrum*	*Thinopyrum intermedium*	HQ221784
Monocotyledons	Poaceae	*Thinopyrum*	*Thinopyrum intermedium*	HQ221785
Monocotyledons	Poaceae	*Thinopyrum*	*Thinopyrum intermedium*	HQ221786
Monocotyledons	Poaceae	*Thinopyrum*	*Thinopyrum intermedium*	HQ221787
Monocotyledons	Poaceae	*Thinopyrum*	*Thinopyrum intermedium*	HQ221788
Monocotyledons	Poaceae	*Thinopyrum*	*Thinopyrum intermedium*	HQ221790
Monocotyledons	Poaceae	*Thinopyrum*	*Thinopyrum intermedium*	HQ221792
Monocotyledons	Poaceae	*Thinopyrum*	*Thinopyrum intermedium*	HQ221793
Monocotyledons	Poaceae	*Thinopyrum*	*Thinopyrum intermedium*	HQ221794
Monocotyledons	Poaceae	*Thinopyrum*	*Thinopyrum intermedium*	HQ221795
Monocotyledons	Poaceae	*Thinopyrum*	*Thinopyrum intermedium*	HQ221796
Monocotyledons	Poaceae	*Thinopyrum*	*Thinopyrum intermedium*	HQ221797
Monocotyledons	Poaceae	*Thinopyrum*	*Thinopyrum intermedium*	HQ221798
Monocotyledons	Poaceae	*Thinopyrum*	*Thinopyrum intermedium*	HQ221799
Monocotyledons	Poaceae	*Thinopyrum*	*Thinopyrum intermedium*	HQ221800
Monocotyledons	Poaceae	*Thinopyrum*	*Thinopyrum intermedium*	HQ221801
Monocotyledons	Poaceae	*Thinopyrum*	*Thinopyrum intermedium*	HQ221802
Monocotyledons	Poaceae	*Thinopyrum*	*Thinopyrum intermedium*	HQ221803
Monocotyledons	Poaceae	*Thinopyrum*	*Thinopyrum intermedium*	HQ221804
Monocotyledons	Poaceae	*Thinopyrum*	*Thinopyrum intermedium*	HQ221805
Monocotyledons	Poaceae	*Thinopyrum*	*Thinopyrum intermedium*	HQ221806
Monocotyledons	Poaceae	*Poa*	*Poa annua*	HQ596792
Monocotyledons	Poaceae	*Poa*	*Poa annua*	HQ596793
Monocotyledons	Poaceae	*Poa*	*Poa annua*	FJ493300
Monocotyledons	Poaceae	*Poa*	*Poa annua*	EU750479
Monocotyledons	Poaceae	*Poa*	*Poa annua*	EU750480
Monocotyledons	Orchidaceae	*Dendrobium*	*Dendrobium nobile*	FJ216469
Monocotyledons	Orchidaceae	*Dendrobium*	*Dendrobium nobile*	FJ216482

Monocotyledons	Orchidaceae	*Dendrobium*	*Dendrobium nobile*	GQ248287
Monocotyledons	Orchidaceae	*Dendrobium*	*Dendrobium nobile*	EU672797
Monocotyledons	Orchidaceae	*Dendrobium*	*Dendrobium nobile*	EF590687
Monocotyledons	Orchidaceae	*Dendrobium*	*Dendrobium nobile*	EU887941
Monocotyledons	Orchidaceae	*Dendrobium*	*Dendrobium nobile*	EU887942
Monocotyledons	Orchidaceae	*Dendrobium*	*Dendrobium fimbriatum*	FJ216468
Monocotyledons	Orchidaceae	*Dendrobium*	*Dendrobium fimbriatum*	EU672798
Monocotyledons	Orchidaceae	*Dendrobium*	*Dendrobium loddigesii*	EU881986
Monocotyledons	Orchidaceae	*Dendrobium*	*Dendrobium loddigesii*	EU887940
Monocotyledons	Orchidaceae	*Dendrobium*	*Dendrobium thyrsiflorum*	EU672799
Monocotyledons	Orchidaceae	*Dendrobium*	*Dendrobium thyrsiflorum*	EU887929
Monocotyledons	Poaceae	*Nassella*	*Nassella tenuis*	EU204723
Monocotyledons	Poaceae	*Nassella*	*Nassella tenuis*	EU204724
Monocotyledons	Orchidaceae	*Cattleya*	*Cattleya aclandiae*	GQ248259
Monocotyledons	Orchidaceae	*Cattleya*	*Cattleya aclandiae*	EU140023
Monocotyledons	Orchidaceae	*Cattleya*	*Cattleya labiata*	EU140040
Monocotyledons	Orchidaceae	*Cattleya*	*Cattleya labiata*	EU140041
Monocotyledons	Acoraceae	*Acorus*	*Acorus tatarinowii*	GQ435343
Monocotyledons	Acoraceae	*Acorus*	*Acorus tatarinowii*	GQ435344
Monocotyledons	Acoraceae	*Acorus*	*Acorus tatarinowii*	GQ435345
Monocotyledons	Acoraceae	*Acorus*	*Acorus tatarinowii*	FJ874983
Monocotyledons	Acoraceae	*Acorus*	*Acorus tatarinowii*	FJ874984
Monocotyledons	Acoraceae	*Acorus*	*Acorus tatarinowii*	FJ874985
Monocotyledons	Acoraceae	*Acorus*	*Acorus tatarinowii*	DQ008890
Monocotyledons	Acoraceae	*Acorus*	*Acorus tatarinowii*	DQ008891
Monocotyledons	Acoraceae	*Acorus*	*Acorus tatarinowii*	DQ008897
Monocotyledons	Acoraceae	*Acorus*	*Acorus tatarinowii*	EU814678
Monocotyledons	Poaceae	*Pseudoroegneria*	*Pseudoroegneria strigosa*	HQ221768
Monocotyledons	Poaceae	*Pseudoroegneria*	*Pseudoroegneria strigosa*	HQ652797
Monocotyledons	Orchidaceae	*Oncidium*	*Oncidium hybrid cultivar*	GU136268
Monocotyledons	Orchidaceae	*Oncidium*	*Oncidium hybrid cultivar*	GU136276
Monocotyledons	Orchidaceae	*Oncidium*	*Oncidium hybrid cultivar*	GU136284
Monocotyledons	Orchidaceae	*Oncidium*	*Oncidium hybrid cultivar*	GU175343
Monocotyledons	Orchidaceae	*Oncidium*	*Oncidium hybrid cultivar*	GU175351
Monocotyledons	Orchidaceae	*Cattleya*	*Cattleya bicolor*	EU140026
Monocotyledons	Orchidaceae	*Cattleya*	*Cattleya bicolor*	EU140027
Monocotyledons	Orchidaceae	*Cattleya*	*Cattleya nobilior*	GQ248260
Monocotyledons	Orchidaceae	*Cattleya*	*Cattleya nobilior*	EU140047
Monocotyledons	Orchidaceae	*Encyclia*	*Encyclia cordigera*	EU213732
Monocotyledons	Orchidaceae	*Encyclia*	*Encyclia cordigera*	EU213733
Monocotyledons	Orchidaceae	*Encyclia*	*Encyclia cordigera*	EU213734
Monocotyledons	Orchidaceae	*Encyclia*	*Encyclia cordigera*	EU213735
Monocotyledons	Orchidaceae	*Dendrobium*	*Dendrobium devonianum*	FJ216478
Monocotyledons	Orchidaceae	*Dendrobium*	*Dendrobium devonianum*	GQ162802
Monocotyledons	Orchidaceae	*Dendrobium*	*Dendrobium gratiosissimum*	FJ216472
Monocotyledons	Orchidaceae	*Dendrobium*	*Dendrobium gratiosissimum*	GQ153536
Monocotyledons	Orchidaceae	*Dendrobium*	*Dendrobium moniliforme*	GQ162803
Monocotyledons	Orchidaceae	*Dendrobium*	*Dendrobium moniliforme*	EU672796
Monocotyledons	Orchidaceae	*Dendrobium*	*Dendrobium moniliforme*	EU887938
Monocotyledons	Orchidaceae	*Dendrobium*	*Dendrobium officinale*	FJ216479
Monocotyledons	Orchidaceae	*Dendrobium*	*Dendrobium officinale*	GQ153537
Monocotyledons	Orchidaceae	*Phalaenopsis*	*Phalaenopsis amabilis*	FJ460367
Monocotyledons	Orchidaceae	*Phalaenopsis*	*Phalaenopsis amabilis*	FJ460368

Monocotyledons	Orchidaceae	*Phalaenopsis*	*Phalaenopsis amabilis*	FJ460369
Monocotyledons	Orchidaceae	*Phalaenopsis*	*Phalaenopsis amabilis*	FJ460370
Monocotyledons	Orchidaceae	*Phalaenopsis*	*Phalaenopsis amabilis*	FJ460373
Monocotyledons	Orchidaceae	*Phalaenopsis*	*Phalaenopsis amabilis*	FJ460374
Monocotyledons	Orchidaceae	*Phalaenopsis*	*Phalaenopsis amabilis*	FJ460388
Monocotyledons	Orchidaceae	*Phalaenopsis*	*Phalaenopsis amabilis*	FJ460389
Monocotyledons	Orchidaceae	*Phalaenopsis*	*Phalaenopsis amabilis*	FJ460390
Monocotyledons	Orchidaceae	*Phalaenopsis*	*Phalaenopsis amabilis*	FJ460394
Monocotyledons	Orchidaceae	*Phalaenopsis*	*Phalaenopsis amabilis*	FJ460397
Monocotyledons	Orchidaceae	*Phalaenopsis*	*Phalaenopsis amabilis*	FJ460399
Monocotyledons	Orchidaceae	*Phalaenopsis*	*Phalaenopsis amabilis*	FJ460400
Monocotyledons	Orchidaceae	*Phalaenopsis*	*Phalaenopsis amabilis*	FJ460404
Monocotyledons	Poaceae	*Setaria*	*Setaria verticillata*	FJ766230
Monocotyledons	Poaceae	*Setaria*	*Setaria verticillata*	HQ876991
Monocotyledons	Orchidaceae	*Cypripedium*	*Cypripedium parviflorum*	HQ596661
Monocotyledons	Orchidaceae	*Cypripedium*	*Cypripedium parviflorum*	JF796968
Monocotyledons	Orchidaceae	*Dendrobium*	*Dendrobium crepidatum*	FJ216488
Monocotyledons	Orchidaceae	*Dendrobium*	*Dendrobium crepidatum*	GQ162800
Monocotyledons	Orchidaceae	*Dendrobium*	*Dendrobium crepidatum*	EU887935
Monocotyledons	Orchidaceae	*Dendrobium*	*Dendrobium aphyllum*	FJ216486
Monocotyledons	Orchidaceae	*Dendrobium*	*Dendrobium aphyllum*	EU887939
Monocotyledons	Orchidaceae	*Dendrobium*	*Dendrobium chrysanthum*	GQ162805
Monocotyledons	Orchidaceae	*Dendrobium*	*Dendrobium chrysanthum*	EU887934
Monocotyledons	Orchidaceae	*Oncidium*	*Oncidium harryanum*	FJ564027
Monocotyledons	Orchidaceae	*Oncidium*	*Oncidium harryanum*	FJ564165
Monocotyledons	Orchidaceae	*Oncidium*	*Oncidium cheirophorum*	FJ564148
Monocotyledons	Orchidaceae	*Oncidium*	*Oncidium cheirophorum*	FJ564308
Monocotyledons	Orchidaceae	*Oncidium*	*Oncidium ghiesbreghtianum*	FJ564050
Monocotyledons	Orchidaceae	*Oncidium*	*Oncidium ghiesbreghtianum*	FJ564152
Monocotyledons	Orchidaceae	*Oncidium*	*Oncidium ghiesbreghtianum*	FJ564657
Monocotyledons	Orchidaceae	*Oncidium*	*Oncidium reichenheimii*	FJ564013
Monocotyledons	Orchidaceae	*Oncidium*	*Oncidium reichenheimii*	FJ564175
Monocotyledons	Orchidaceae	*Oncidium*	*Oncidium leucochilum*	FJ564012
Monocotyledons	Orchidaceae	*Oncidium*	*Oncidium leucochilum*	FJ564635
Monocotyledons	Orchidaceae	*Oncidium*	*Oncidium poikilostalix*	FJ564029
Monocotyledons	Orchidaceae	*Oncidium*	*Oncidium poikilostalix*	FJ564660
Monocotyledons	Poaceae	*Cenchrus*	*Cenchrus purpureus*	GU135321
Monocotyledons	Poaceae	*Cenchrus*	*Cenchrus purpureus*	GU135404
Monocotyledons	Orchidaceae	*Oncidium*	*Oncidium vulcanicum*	FJ564153
Monocotyledons	Orchidaceae	*Oncidium*	*Oncidium vulcanicum*	FJ564248
Monocotyledons	Orchidaceae	*Oncidium*	*Oncidium aureum*	FJ564096
Monocotyledons	Orchidaceae	*Oncidium*	*Oncidium aureum*	FJ564562
Monocotyledons	Orchidaceae	*Oncidium*	*Oncidium armatum*	FJ564169
Monocotyledons	Orchidaceae	*Oncidium*	*Oncidium armatum*	FJ564193
Monocotyledons	Orchidaceae	*Oncidium*	*Oncidium aspidorhinum*	FJ564203
Monocotyledons	Orchidaceae	*Oncidium*	*Oncidium aspidorhinum*	FJ564256
Monocotyledons	Orchidaceae	*Oncidium*	*Oncidium cirrhosum*	FJ564032
Monocotyledons	Orchidaceae	*Oncidium*	*Oncidium cirrhosum*	FJ564180
Monocotyledons	Orchidaceae	*Oncidium*	*Oncidium cirrhosum*	FJ564569
Monocotyledons	Orchidaceae	*Oncidium*	*Oncidium alexandrae*	FJ564166
Monocotyledons	Orchidaceae	*Oncidium*	*Oncidium alexandrae*	FJ564188
Monocotyledons	Orchidaceae	*Oncidium*	*Oncidium lehmannii*	FJ564484
Monocotyledons	Orchidaceae	*Oncidium*	*Oncidium lehmannii*	FJ564541

Monocotyledons	Orchidaceae	*Oncidium*	*Oncidium epidendroides*	FJ564173
Monocotyledons	Orchidaceae	*Oncidium*	*Oncidium epidendroides*	FJ564201
Monocotyledons	Orchidaceae	*Oncidium*	*Oncidium epidendroides*	FJ564241
Monocotyledons	Orchidaceae	*Oncidium*	*Oncidium epidendroides*	FJ564488
Monocotyledons	Orchidaceae	*Oncidium*	*Oncidium hallii*	FJ563973
Monocotyledons	Orchidaceae	*Oncidium*	*Oncidium hallii*	FJ564159
Monocotyledons	Orchidaceae	*Oncidium*	*Oncidium luteopurpureum*	FJ564071
Monocotyledons	Orchidaceae	*Oncidium*	*Oncidium luteopurpureum*	FJ564540
Monocotyledons	Orchidaceae	*Oncidium*	*Oncidium nevadense*	FJ564090
Monocotyledons	Orchidaceae	*Oncidium*	*Oncidium nevadense*	FJ564150
Monocotyledons	Orchidaceae	*Oncidium*	*Oncidium portmannii*	FJ564164
Monocotyledons	Orchidaceae	*Oncidium*	*Oncidium portmannii*	FJ564172
Monocotyledons	Orchidaceae	*Oncidium*	*Oncidium portmannii*	FJ564490
Monocotyledons	Orchidaceae	*Oncidium*	*Oncidium tenuoides*	FJ564192
Monocotyledons	Orchidaceae	*Oncidium*	*Oncidium tenuoides*	FJ564531
Monocotyledons	Orchidaceae	*Oncidium*	*Oncidium baueri*	FJ564224
Monocotyledons	Orchidaceae	*Oncidium*	*Oncidium baueri*	FJ564534
Monocotyledons	Orchidaceae	*Oncidium*	*Oncidium chrysomorphum*	FJ564189
Monocotyledons	Orchidaceae	*Oncidium*	*Oncidium chrysomorphum*	FJ564513
Monocotyledons	Orchidaceae	*Oncidium*	*Oncidium endocharis*	FJ564088
Monocotyledons	Orchidaceae	*Oncidium*	*Oncidium endocharis*	FJ564369
Monocotyledons	Orchidaceae	*Oncidium*	*Oncidium fuscatum*	FJ564038
Monocotyledons	Orchidaceae	*Oncidium*	*Oncidium fuscatum*	FJ564227
Monocotyledons	Orchidaceae	*Oncidium*	*Oncidium heteranthum*	FJ564537
Monocotyledons	Orchidaceae	*Oncidium*	*Oncidium heteranthum*	FJ564618
Monocotyledons	Orchidaceae	*Oncidium*	*Oncidium incurvum*	FJ564437
Monocotyledons	Orchidaceae	*Oncidium*	*Oncidium incurvum*	FJ564630
Monocotyledons	Orchidaceae	*Oncidium*	*Oncidium isthmi*	FJ564221
Monocotyledons	Orchidaceae	*Oncidium*	*Oncidium isthmi*	FJ564543
Monocotyledons	Orchidaceae	*Oncidium*	*Oncidium retusum*	FJ564005
Monocotyledons	Orchidaceae	*Oncidium*	*Oncidium retusum*	FJ564215
Monocotyledons	Orchidaceae	*Oncidium*	*Oncidium retusum*	FJ564578
Monocotyledons	Orchidaceae	*Oncidium*	*Oncidium retusum*	FJ564580
Monocotyledons	Orchidaceae	*Oncidium*	*Oncidium gramineum*	FJ564238
Monocotyledons	Orchidaceae	*Oncidium*	*Oncidium gramineum*	FJ564500
Monocotyledons	Orchidaceae	*Oncidium*	*Oncidium gramineum*	FJ564617
Monocotyledons	Orchidaceae	*Oncidium*	*Oncidium tigroides*	FJ564548
Monocotyledons	Orchidaceae	*Oncidium*	*Oncidium tigroides*	FJ564570
Monocotyledons	Araceae	*Lemna*	*Lemna aequinoctialis*	GU454500
Monocotyledons	Araceae	*Lemna*	*Lemna aequinoctialis*	GU454501
Monocotyledons	Araceae	*Lemna*	*Lemna aequinoctialis*	GU454502
Monocotyledons	Araceae	*Lemna*	*Lemna minuta*	GU454517
Monocotyledons	Araceae	*Lemna*	*Lemna minuta*	GU454518
Monocotyledons	Araceae	*Lemna*	*Lemna minuta*	GU454519
Monocotyledons	Araceae	*Lemna*	*Lemna minuta*	GU454527
Monocotyledons	Araceae	*Lemna*	*Lemna trisulca*	GU454521
Monocotyledons	Araceae	*Lemna*	*Lemna trisulca*	GU454522
Monocotyledons	Araceae	*Lemna*	*Lemna trisulca*	GU454523
Monocotyledons	Araceae	*Lemna*	*Lemna turionifera*	GU454524
Monocotyledons	Araceae	*Lemna*	*Lemna turionifera*	GU454525
Monocotyledons	Araceae	*Lemna*	*Lemna valdiviana*	GU454526
Monocotyledons	Araceae	*Lemna*	*Lemna valdiviana*	GU454528
Monocotyledons	Araceae	*Lemna*	*Lemna valdiviana*	GU454529

Monocotyledons	Orchidaceae	*Dendrobium*	*Dendrobium chrysotoxum*	EU672792
Monocotyledons	Orchidaceae	*Dendrobium*	*Dendrobium chrysotoxum*	EU887923
Monocotyledons	Orchidaceae	*Dendrobium*	*Dendrobium chrysotoxum*	EU887924
Monocotyledons	Orchidaceae	*Dendrobium*	*Dendrobium chrysotoxum*	EU887925
Monocotyledons	Orchidaceae	*Dendrobium*	*Dendrobium densiflorum*	FJ216480
Monocotyledons	Orchidaceae	*Dendrobium*	*Dendrobium densiflorum*	FJ216487
Monocotyledons	Orchidaceae	*Dendrobium*	*Dendrobium lindleyi*	GQ248286
Monocotyledons	Orchidaceae	*Dendrobium*	*Dendrobium lindleyi*	EF590688
Monocotyledons	Orchidaceae	*Dendrobium*	*Dendrobium williamsonii*	EU672795
Monocotyledons	Orchidaceae	*Dendrobium*	*Dendrobium williamsonii*	EU887927
Monocotyledons	Poaceae	*Eragrostis*	*Eragrostis tenuifolia*	GQ248300
Monocotyledons	Poaceae	*Eragrostis*	*Eragrostis tenuifolia*	HQ876971
Monocotyledons	Poaceae	*Eragrostis*	*Eragrostis tenuifolia*	HQ876972
Monocotyledons	Dioscoreaceae	*Tacca*	*Tacca integrifolia*	JN850586
Monocotyledons	Dioscoreaceae	*Tacca*	*Tacca integrifolia*	JN850587
Monocotyledons	Dioscoreaceae	*Tacca*	*Tacca integrifolia*	EF590745
Monocotyledons	Dioscoreaceae	*Tacca*	*Tacca integrifolia*	JN047329
Monocotyledons	Dioscoreaceae	*Tacca*	*Tacca integrifolia*	JN047330
Monocotyledons	Dioscoreaceae	*Tacca*	*Tacca integrifolia*	JN047331
Monocotyledons	Dioscoreaceae	*Tacca*	*Tacca integrifolia*	JN047332
Monocotyledons	Dioscoreaceae	*Tacca*	*Tacca integrifolia*	JN047333
Monocotyledons	Dioscoreaceae	*Tacca*	*Tacca integrifolia*	JN047334
Monocotyledons	Dioscoreaceae	*Tacca*	*Tacca integrifolia*	JN047335
Monocotyledons	Dioscoreaceae	*Tacca*	*Tacca integrifolia*	JN047336
Monocotyledons	Dioscoreaceae	*Tacca*	*Tacca integrifolia*	JN047337
Monocotyledons	Dioscoreaceae	*Tacca*	*Tacca integrifolia*	JN047338
Monocotyledons	Dioscoreaceae	*Tacca*	*Tacca integrifolia*	JN047339
Monocotyledons	Dioscoreaceae	*Tacca*	*Tacca integrifolia*	JN047340
Monocotyledons	Dioscoreaceae	*Tacca*	*Tacca integrifolia*	JN047341
Monocotyledons	Dioscoreaceae	*Tacca*	*Tacca integrifolia*	JN047342
Monocotyledons	Dioscoreaceae	*Tacca*	*Tacca integrifolia*	JN047343
Monocotyledons	Orchidaceae	*Dendrobium*	*Dendrobium primulinum*	GQ153535
Monocotyledons	Orchidaceae	*Dendrobium*	*Dendrobium primulinum*	EU887936
Monocotyledons	Orchidaceae	*Dendrobium*	*Dendrobium primulinum*	EU887937
Monocotyledons	Orchidaceae	*Dendrobium*	*Dendrobium wardianum*	GQ162801
Monocotyledons	Orchidaceae	*Dendrobium*	*Dendrobium wardianum*	EU887930
Monocotyledons	Orchidaceae	*Dendrobium*	*Dendrobium wardianum*	EU887931
Monocotyledons	Araceae	*Alocasia*	*Alocasia odora*	DQ786512
Monocotyledons	Araceae	*Alocasia*	*Alocasia odora*	JF828144
Monocotyledons	Orchidaceae	*Dendrobium*	*Dendrobium hancockii*	FJ216481
Monocotyledons	Orchidaceae	*Dendrobium*	*Dendrobium hancockii*	EU672800
Monocotyledons	Poaceae	*Nassella*	*Nassella trichotoma*	EU489305
Monocotyledons	Poaceae	*Nassella*	*Nassella trichotoma*	EU204726
Monocotyledons	Poaceae	*Nassella*	*Nassella trichotoma*	EU204727
Monocotyledons	Poaceae	*Bambusa*	*Bambusa bambos*	GU390993
Monocotyledons	Poaceae	*Bambusa*	*Bambusa bambos*	GU063075
Monocotyledons	Poaceae	*Eragrostis*	*Eragrostis heteromera*	HQ876966
Monocotyledons	Poaceae	*Eragrostis*	*Eragrostis heteromera*	HQ876967
Monocotyledons	Orchidaceae	*Phalaenopsis*	*Phalaenopsis aphrodite*	FJ460375
Monocotyledons	Orchidaceae	*Phalaenopsis*	*Phalaenopsis aphrodite*	FJ460376
Monocotyledons	Orchidaceae	*Phalaenopsis*	*Phalaenopsis aphrodite*	FJ460377
Monocotyledons	Orchidaceae	*Phalaenopsis*	*Phalaenopsis aphrodite*	FJ460378
Monocotyledons	Orchidaceae	*Phalaenopsis*	*Phalaenopsis aphrodite*	FJ460379

Monocotyledons	Orchidaceae	*Phalaenopsis*	*Phalaenopsis aphrodite*	FJ460384
Monocotyledons	Orchidaceae	*Phalaenopsis*	*Phalaenopsis aphrodite*	FJ460401
Monocotyledons	Orchidaceae	*Phalaenopsis*	*Phalaenopsis aphrodite*	FJ460402
Monocotyledons	Orchidaceae	*Phalaenopsis*	*Phalaenopsis aphrodite*	FJ460403
Monocotyledons	Orchidaceae	*Phalaenopsis*	*Phalaenopsis sanderiana*	FJ460366
Monocotyledons	Orchidaceae	*Phalaenopsis*	*Phalaenopsis sanderiana*	FJ460380
Monocotyledons	Orchidaceae	*Phalaenopsis*	*Phalaenopsis sanderiana*	FJ460381
Monocotyledons	Orchidaceae	*Bulbophyllum*	*Bulbophyllum nutans*	EF200433
Monocotyledons	Orchidaceae	*Bulbophyllum*	*Bulbophyllum nutans*	EF200434
Monocotyledons	Orchidaceae	*Bulbophyllum*	*Bulbophyllum nutans*	EF200435
Monocotyledons	Orchidaceae	*Bulbophyllum*	*Bulbophyllum nutans*	EF200436
Monocotyledons	Poaceae	*Urochloa*	*Urochloa dictyoneura*	HQ876937
Monocotyledons	Poaceae	*Urochloa*	*Urochloa dictyoneura*	HQ876938
Monocotyledons	Poaceae	*Glyceria*	*Glyceria fluitans*	DQ665497
Monocotyledons	Poaceae	*Glyceria*	*Glyceria fluitans*	DQ665498
Monocotyledons	Poaceae	*Glyceria*	*Glyceria fluitans*	DQ665499
Monocotyledons	Poaceae	*Glyceria*	*Glyceria fluitans*	DQ665500
Monocotyledons	Poaceae	*Glyceria*	*Glyceria fluitans*	DQ665501
Monocotyledons	Poaceae	*Glyceria*	*Glyceria fluitans*	DQ665502
Monocotyledons	Poaceae	*Glyceria*	*Glyceria fluitans*	DQ665503
Monocotyledons	Poaceae	*Glyceria*	*Glyceria fluitans*	DQ665504
Monocotyledons	Poaceae	*Glyceria*	*Glyceria fluitans*	DQ665505
Monocotyledons	Poaceae	*Glyceria*	*Glyceria fluitans*	DQ665506
Monocotyledons	Acoraceae	*Acorus*	*Acorus americanus*	FJ874967
Monocotyledons	Acoraceae	*Acorus*	*Acorus americanus*	FJ874968
Monocotyledons	Acoraceae	*Acorus*	*Acorus americanus*	FJ874969
Monocotyledons	Acoraceae	*Acorus*	*Acorus americanus*	DQ008896
Monocotyledons	Acoraceae	*Acorus*	*Acorus americanus*	EU814671
Monocotyledons	Acoraceae	*Acorus*	*Acorus americanus*	EU814672
Monocotyledons	Poaceae	*Poa*	*Poa compressa*	EU750481
Monocotyledons	Poaceae	*Poa*	*Poa compressa*	EU750482
Monocotyledons	Poaceae	*Poa*	*Poa compressa*	EU750483
Monocotyledons	Poaceae	*Setaria*	*Setaria pumila*	HQ596843
Monocotyledons	Poaceae	*Setaria*	*Setaria pumila*	HQ876989
Monocotyledons	Poaceae	*Bambusa*	*Bambusa tuldoides*	GU391001
Monocotyledons	Poaceae	*Bambusa*	*Bambusa tuldoides*	GU063083
Monocotyledons	Poaceae	*Bambusa*	*Bambusa sinospinosa*	GU391000
Monocotyledons	Poaceae	*Bambusa*	*Bambusa sinospinosa*	GU063077
Monocotyledons	Poaceae	*Bambusa*	*Bambusa blumeana*	GU390994
Monocotyledons	Poaceae	*Bambusa*	*Bambusa blumeana*	GU063076
Monocotyledons	Poaceae	*Glyceria*	*Glyceria canadensis*	DQ665471
Monocotyledons	Poaceae	*Glyceria*	*Glyceria canadensis*	DQ665472
Monocotyledons	Poaceae	*Glyceria*	*Glyceria canadensis*	DQ665473
Monocotyledons	Poaceae	*Glyceria*	*Glyceria canadensis*	DQ665474
Monocotyledons	Poaceae	*Glyceria*	*Glyceria elata*	DQ665485
Monocotyledons	Poaceae	*Glyceria*	*Glyceria elata*	DQ665486
Monocotyledons	Poaceae	*Glyceria*	*Glyceria elata*	DQ665487
Monocotyledons	Poaceae	*Glyceria*	*Glyceria elata*	DQ665488
Monocotyledons	Poaceae	*Glyceria*	*Glyceria elata*	DQ665489
Monocotyledons	Poaceae	*Glyceria*	*Glyceria elata*	DQ665490
Monocotyledons	Poaceae	*Glyceria*	*Glyceria elata*	DQ665491
Monocotyledons	Poaceae	*Glyceria*	*Glyceria elata*	DQ665492
Monocotyledons	Poaceae	*Glyceria*	*Glyceria elata*	DQ665493

Monocotyledons	Poaceae	*Glyceria*	*Glyceria elata*	DQ665494
Monocotyledons	Poaceae	*Glyceria*	*Glyceria elata*	DQ665495
Monocotyledons	Poaceae	*Glyceria*	*Glyceria elata*	DQ665496
Monocotyledons	Poaceae	*Glyceria*	*Glyceria leptostachya*	DQ665510
Monocotyledons	Poaceae	*Glyceria*	*Glyceria leptostachya*	DQ665511
Monocotyledons	Poaceae	*Glyceria*	*Glyceria leptostachya*	DQ665512
Monocotyledons	Poaceae	*Glyceria*	*Glyceria leptostachya*	DQ665513
Monocotyledons	Poaceae	*Glyceria*	*Glyceria leptostachya*	DQ665514
Monocotyledons	Poaceae	*Glyceria*	*Glyceria maxima*	DQ665515
Monocotyledons	Poaceae	*Glyceria*	*Glyceria maxima*	DQ665516
Monocotyledons	Poaceae	*Glyceria*	*Glyceria notata*	DQ665518
Monocotyledons	Poaceae	*Glyceria*	*Glyceria notata*	DQ665519
Monocotyledons	Poaceae	*Glyceria*	*Glyceria occidentalis*	DQ665520
Monocotyledons	Poaceae	*Glyceria*	*Glyceria occidentalis*	DQ665521
Monocotyledons	Poaceae	*Glyceria*	*Glyceria occidentalis*	DQ665522
Monocotyledons	Poaceae	*Glyceria*	*Glyceria occidentalis*	DQ665523
Monocotyledons	Poaceae	*Glyceria*	*Glyceria occidentalis*	DQ665524
Monocotyledons	Poaceae	*Glyceria*	*Glyceria occidentalis*	DQ665525
Monocotyledons	Poaceae	*Glyceria*	*Glyceria occidentalis*	DQ665526
Monocotyledons	Poaceae	*Glyceria*	*Glyceria occidentalis*	DQ665527
Monocotyledons	Poaceae	*Glyceria*	*Glyceria occidentalis*	DQ665528
Monocotyledons	Poaceae	*Glyceria*	*Glyceria occidentalis*	DQ665529
Monocotyledons	Poaceae	*Glyceria*	*Glyceria septentrionalis*	DQ665530
Monocotyledons	Poaceae	*Glyceria*	*Glyceria septentrionalis*	DQ665531
Monocotyledons	Poaceae	*Glyceria*	*Glyceria septentrionalis*	DQ665532
Monocotyledons	Poaceae	*Glyceria*	*Glyceria septentrionalis*	DQ665533
Monocotyledons	Poaceae	*Glyceria*	*Glyceria septentrionalis*	DQ665534
Monocotyledons	Poaceae	*Jarava*	*Jarava leptostachya*	EU489270
Monocotyledons	Poaceae	*Jarava*	*Jarava leptostachya*	EU489271
Monocotyledons	Poaceae	*Nassella*	*Nassella cordobensis*	EU204713
Monocotyledons	Poaceae	*Nassella*	*Nassella cordobensis*	EU204714
Monocotyledons	Poaceae	*Nassella*	*Nassella hyalina*	EU204715
Monocotyledons	Poaceae	*Nassella*	*Nassella hyalina*	EU204716
Monocotyledons	Poaceae	*Nassella*	*Nassella hyalina*	EU204717
Monocotyledons	Poaceae	*Nassella*	*Nassella neesiana*	EU489297
Monocotyledons	Poaceae	*Nassella*	*Nassella neesiana*	EU204719
Monocotyledons	Poaceae	*Nassella*	*Nassella neesiana*	EU204720
Monocotyledons	Orchidaceae	*Bulbophyllum*	*Bulbophyllum longiflorum*	EF200405
Monocotyledons	Orchidaceae	*Bulbophyllum*	*Bulbophyllum longiflorum*	EF200406
Monocotyledons	Poaceae	*Eragrostis*	*Eragrostis pectinacea*	HQ596686
Monocotyledons	Poaceae	*Eragrostis*	*Eragrostis pectinacea*	HQ596687
Monocotyledons	Poaceae	*Eragrostis*	*Eragrostis pectinacea*	GQ248299
Monocotyledons	Poaceae	*Eragrostis*	*Eragrostis pectinacea*	EF590696
Monocotyledons	Orchidaceae	*Bulbophyllum*	*Bulbophyllum analamazoatrae*	EF200408
Monocotyledons	Orchidaceae	*Bulbophyllum*	*Bulbophyllum analamazoatrae*	EF200409
Monocotyledons	Orchidaceae	*Bulbophyllum*	*Bulbophyllum elliottii*	EF200388
Monocotyledons	Orchidaceae	*Bulbophyllum*	*Bulbophyllum elliottii*	EF200389
Monocotyledons	Orchidaceae	*Bulbophyllum*	*Bulbophyllum elliottii*	EF200390
Monocotyledons	Orchidaceae	*Bulbophyllum*	*Bulbophyllum elliottii*	EF200391
Monocotyledons	Orchidaceae	*Bulbophyllum*	*Bulbophyllum francoisii*	EF200411
Monocotyledons	Orchidaceae	*Bulbophyllum*	*Bulbophyllum francoisii*	EF200417
Monocotyledons	Orchidaceae	*Bulbophyllum*	*Bulbophyllum henrici*	EF200480
Monocotyledons	Orchidaceae	*Bulbophyllum*	*Bulbophyllum henrici*	EF200491

Monocotyledons	Orchidaceae	*Bulbophyllum*	*Bulbophyllum longivaginans*	EF200463
Monocotyledons	Orchidaceae	*Bulbophyllum*	*Bulbophyllum longivaginans*	EF200464
Monocotyledons	Orchidaceae	*Bulbophyllum*	*Bulbophyllum matitanense*	EF200393
Monocotyledons	Orchidaceae	*Bulbophyllum*	*Bulbophyllum matitanense*	EF200396
Monocotyledons	Orchidaceae	*Bulbophyllum*	*Bulbophyllum oxycalyx*	EF200412
Monocotyledons	Orchidaceae	*Bulbophyllum*	*Bulbophyllum oxycalyx*	EF200416
Monocotyledons	Orchidaceae	*Bulbophyllum*	*Bulbophyllum pachypus*	EF200466
Monocotyledons	Orchidaceae	*Bulbophyllum*	*Bulbophyllum pachypus*	EF200471
Monocotyledons	Orchidaceae	*Bulbophyllum*	*Bulbophyllum rauhii*	EF200413
Monocotyledons	Orchidaceae	*Bulbophyllum*	*Bulbophyllum rauhii*	EF200414
Monocotyledons	Orchidaceae	*Bulbophyllum*	*Bulbophyllum falcatum*	GQ339616
Monocotyledons	Orchidaceae	*Bulbophyllum*	*Bulbophyllum falcatum*	JF693811
Monocotyledons	Orchidaceae	*Cypripedium*	*Cypripedium debile*	JF796983
Monocotyledons	Orchidaceae	*Cypripedium*	*Cypripedium debile*	JF796986
Monocotyledons	Orchidaceae	*Cypripedium*	*Cypripedium debile*	JF796987
Monocotyledons	Orchidaceae	*Cypripedium*	*Cypripedium henryi*	JQ080173
Monocotyledons	Orchidaceae	*Cypripedium*	*Cypripedium henryi*	JF796956
Monocotyledons	Poaceae	*Jarava*	*Jarava eriostachya*	EU204702
Monocotyledons	Poaceae	*Jarava*	*Jarava eriostachya*	EU204703
Monocotyledons	Poaceae	*Jarava*	*Jarava eriostachya*	EU204704
Monocotyledons	Poaceae	*Jarava*	*Jarava eriostachya*	EU204705
Monocotyledons	Poaceae	*Jarava*	*Jarava humilis*	EU489263
Monocotyledons	Poaceae	*Jarava*	*Jarava humilis*	EU489264
Monocotyledons	Poaceae	*Jarava*	*Jarava humilis*	EU489265
Monocotyledons	Poaceae	*Jarava*	*Jarava humilis*	EU204732
Monocotyledons	Poaceae	*Jarava*	*Jarava humilis*	EU204733
Monocotyledons	Orchidaceae	*Encyclia*	*Encyclia alata*	EU213730
Monocotyledons	Orchidaceae	*Encyclia*	*Encyclia alata*	EU213731
Monocotyledons	Orchidaceae	*Oncidium*	*Oncidium obryzatoides*	EU213752
Monocotyledons	Orchidaceae	*Oncidium*	*Oncidium obryzatoides*	FJ564374
Monocotyledons	Orchidaceae	*Oncidium*	*Oncidium storkii*	EU213753
Monocotyledons	Orchidaceae	*Oncidium*	*Oncidium storkii*	FJ564375
Monocotyledons	Poaceae	*Jarava*	*Jarava frigida*	EU489260
Monocotyledons	Poaceae	*Jarava*	*Jarava frigida*	EU489261
Monocotyledons	Orchidaceae	*Leochilus*	*Leochilus inconspicuus*	FJ564430
Monocotyledons	Orchidaceae	*Leochilus*	*Leochilus inconspicuus*	FJ564440
Monocotyledons	Orchidaceae	*Leochilus*	*Leochilus leiboldi*	FJ564139
Monocotyledons	Orchidaceae	*Leochilus*	*Leochilus leiboldi*	FJ564428
Monocotyledons	Orchidaceae	*Oncidium*	*Oncidium echinops*	FJ564327
Monocotyledons	Orchidaceae	*Oncidium*	*Oncidium echinops*	FJ564505
Monocotyledons	Orchidaceae	*Oncidium*	*Oncidium echinops*	FJ564594
Monocotyledons	Orchidaceae	*Oncidium*	*Oncidium peruvianoides*	FJ564458
Monocotyledons	Orchidaceae	*Oncidium*	*Oncidium peruvianoides*	FJ564512
Monocotyledons	Poaceae	*Tripogon*	*Tripogon wightii*	FJ610317
Monocotyledons	Poaceae	*Tripogon*	*Tripogon wightii*	FJ610318
Monocotyledons	Poaceae	*Tripogon*	*Tripogon wightii*	FJ610319
Monocotyledons	Poaceae	*Tripogon*	*Tripogon wightii*	FJ610320
Monocotyledons	Poaceae	*Tripogon*	*Tripogon wightii*	FJ610321
Monocotyledons	Orchidaceae	*Oncidium*	*Oncidium heterodactylum*	FJ564516
Monocotyledons	Orchidaceae	*Oncidium*	*Oncidium heterodactylum*	FJ564536
Monocotyledons	Orchidaceae	*Oncidium*	*Oncidium heterodactylum*	FJ564538
Monocotyledons	Orchidaceae	*Oncidium*	*Oncidium iricolor*	FJ564371
Monocotyledons	Orchidaceae	*Oncidium*	*Oncidium iricolor*	FJ564628

Monocotyledons	Orchidaceae	*Oncidium*	*Oncidium boothianum*	FJ564535
Monocotyledons	Orchidaceae	*Oncidium*	*Oncidium boothianum*	FJ564581
Monocotyledons	Orchidaceae	*Oncidium*	*Oncidium boothianum*	FJ564588
Monocotyledons	Orchidaceae	*Oncidium*	*Oncidium boothianum*	FJ564589
Monocotyledons	Orchidaceae	*Oncidium*	*Oncidium schmidtianum*	FJ564514
Monocotyledons	Orchidaceae	*Oncidium*	*Oncidium schmidtianum*	FJ564576
Monocotyledons	Orchidaceae	*Oncidium*	*Oncidium schroederianum*	FJ564198
Monocotyledons	Orchidaceae	*Oncidium*	*Oncidium schroederianum*	FJ564429
Monocotyledons	Orchidaceae	*Cypripedium*	*Cypripedium micranthum*	GQ248282
Monocotyledons	Orchidaceae	*Cypripedium*	*Cypripedium micranthum*	JF796964
Monocotyledons	Poaceae	*Tripogon*	*Tripogon copei*	FJ707304
Monocotyledons	Poaceae	*Tripogon*	*Tripogon copei*	FJ707305
Monocotyledons	Poaceae	*Tripogon*	*Tripogon copei*	FJ707306
Monocotyledons	Poaceae	*Tripogon*	*Tripogon copei*	FJ707307
Monocotyledons	Poaceae	*Tripogon*	*Tripogon copei*	FJ707308
Monocotyledons	Poaceae	*Bambusa*	*Bambusa distegia*	GU390995
Monocotyledons	Poaceae	*Bambusa*	*Bambusa distegia*	GU063081
Monocotyledons	Orchidaceae	*Bulbophyllum*	*Bulbophyllum exaltatum*	GQ339640
Monocotyledons	Orchidaceae	*Bulbophyllum*	*Bulbophyllum exaltatum*	GQ339641
Monocotyledons	Poaceae	*Cenchrus*	*Cenchrus stramineus*	HQ876987
Monocotyledons	Poaceae	*Cenchrus*	*Cenchrus stramineus*	HQ876988
Monocotyledons	Poaceae	*Sporobolus*	*Sporobolus ioclados*	HQ876994
Monocotyledons	Poaceae	*Sporobolus*	*Sporobolus ioclados*	HQ876995
Monocotyledons	Poaceae	*Sporobolus*	*Sporobolus rangei*	HQ877000
Monocotyledons	Poaceae	*Sporobolus*	*Sporobolus rangei*	HQ877001
Monocotyledons	Poaceae	*Urochloa*	*Urochloa brachyura*	HQ877006
Monocotyledons	Poaceae	*Urochloa*	*Urochloa brachyura*	HQ877007
Monocotyledons	Poaceae	*Urochloa*	*Urochloa brachyura*	HQ877008
Monocotyledons	Araceae	*Colocasia*	*Colocasia lihengiae*	JF828133
Monocotyledons	Araceae	*Colocasia*	*Colocasia lihengiae*	JF828137
Monocotyledons	Dioscoreaceae	*Tacca*	*Tacca ampliplacenta*	JN850584
Monocotyledons	Dioscoreaceae	*Tacca*	*Tacca ampliplacenta*	JN047314
Monocotyledons	Dioscoreaceae	*Tacca*	*Tacca ampliplacenta*	JN047315
Monocotyledons	Dioscoreaceae	*Tacca*	*Tacca ampliplacenta*	JN047316
Monocotyledons	Dioscoreaceae	*Tacca*	*Tacca ampliplacenta*	JN047317
Monocotyledons	Dioscoreaceae	*Tacca*	*Tacca ampliplacenta*	JN047318
Monocotyledons	Dioscoreaceae	*Tacca*	*Tacca ampliplacenta*	JN047319
Monocotyledons	Dioscoreaceae	*Tacca*	*Tacca ampliplacenta*	JN047320
Monocotyledons	Dioscoreaceae	*Tacca*	*Tacca ampliplacenta*	JN047321
Monocotyledons	Dioscoreaceae	*Tacca*	*Tacca ampliplacenta*	JN047322
Monocotyledons	Dioscoreaceae	*Tacca*	*Tacca ampliplacenta*	JN047323
Monocotyledons	Dioscoreaceae	*Tacca*	*Tacca subflabellata*	JN850593
Monocotyledons	Dioscoreaceae	*Tacca*	*Tacca subflabellata*	JN047359
Monocotyledons	Dioscoreaceae	*Tacca*	*Tacca subflabellata*	JN047360
Monocotyledons	Dioscoreaceae	*Tacca*	*Tacca subflabellata*	JN047361
Monocotyledons	Dioscoreaceae	*Tacca*	*Tacca subflabellata*	JN047362
Monocotyledons	Poaceae	*Festuca*	*Festuca arundinacea*	HQ596699
Monocotyledons	Poaceae	*Festuca*	*Festuca arundinacea*	HQ596753
Monocotyledons	Musaceae	*Musa*	*Musa acuminata*	FJ871854
Monocotyledons	Musaceae	*Musa*	*Musa acuminata*	FJ871855
Monocotyledons	Musaceae	*Musa*	*Musa acuminata*	FJ871856
Monocotyledons	Musaceae	*Musa*	*Musa acuminata*	FJ871857
Monocotyledons	Musaceae	*Musa*	*Musa acuminata*	FJ871858

Monocotyledons	Musaceae	*Musa*	*Musa acuminata*	FJ871859
Monocotyledons	Musaceae	*Musa*	*Musa acuminata*	FJ871860
Monocotyledons	Musaceae	*Musa*	*Musa acuminata*	FJ871861
Monocotyledons	Musaceae	*Musa*	*Musa acuminata*	FJ871862
Monocotyledons	Musaceae	*Musa*	*Musa acuminata*	FJ871863
Monocotyledons	Musaceae	*Musa*	*Musa acuminata*	FJ871864
Monocotyledons	Musaceae	*Musa*	*Musa acuminata*	FJ871865
Monocotyledons	Musaceae	*Musa*	*Musa acuminata*	FJ871866
Monocotyledons	Musaceae	*Musa*	*Musa acuminata*	FJ871867
Monocotyledons	Musaceae	*Musa*	*Musa acuminata*	FJ871868
Monocotyledons	Musaceae	*Musa*	*Musa acuminata*	FJ871869
Monocotyledons	Musaceae	*Musa*	*Musa acuminata*	FJ871915
Monocotyledons	Musaceae	*Musa*	*Musa acuminata*	FJ871916
Monocotyledons	Dioscoreaceae	*Dioscorea*	*Dioscorea japonica*	JQ260355
Monocotyledons	Dioscoreaceae	*Dioscorea*	*Dioscorea japonica*	JQ260356
Monocotyledons	Dioscoreaceae	*Dioscorea*	*Dioscorea japonica*	JQ260357
Monocotyledons	Dioscoreaceae	*Dioscorea*	*Dioscorea japonica*	JQ260358
Monocotyledons	Dioscoreaceae	*Dioscorea*	*Dioscorea japonica*	JQ260359
Monocotyledons	Dioscoreaceae	*Dioscorea*	*Dioscorea japonica*	GQ166695
Monocotyledons	Amaryllidaceae	*Allium*	*Allium tuberosum*	GQ434884
Monocotyledons	Amaryllidaceae	*Allium*	*Allium tuberosum*	GQ434885
Monocotyledons	Amaryllidaceae	*Allium*	*Allium tuberosum*	GQ434886
Monocotyledons	Amaryllidaceae	*Allium*	*Allium tuberosum*	GQ434887
Monocotyledons	Amaryllidaceae	*Allium*	*Allium tuberosum*	GQ434888
Monocotyledons	Asparagaceae	*Asparagus*	*Asparagus officinalis*	HM990123
Monocotyledons	Asparagaceae	*Asparagus*	*Asparagus officinalis*	HM990126
Monocotyledons	Asparagaceae	*Asparagus*	*Asparagus officinalis*	HM990127
Monocotyledons	Asparagaceae	*Asparagus*	*Asparagus officinalis*	HM990129
Monocotyledons	Asparagaceae	*Asparagus*	*Asparagus officinalis*	HM990131
Monocotyledons	Asparagaceae	*Asparagus*	*Asparagus officinalis*	HM990132
Monocotyledons	Asparagaceae	*Asparagus*	*Asparagus officinalis*	HM990133
Monocotyledons	Asparagaceae	*Asparagus*	*Asparagus officinalis*	HM990134
Monocotyledons	Asparagaceae	*Asparagus*	*Asparagus officinalis*	HM990135
Monocotyledons	Asparagaceae	*Asparagus*	*Asparagus officinalis*	HM990136
Monocotyledons	Asparagaceae	*Asparagus*	*Asparagus officinalis*	HM990137
Monocotyledons	Asparagaceae	*Asparagus*	*Asparagus officinalis*	HM990139
Monocotyledons	Asparagaceae	*Asparagus*	*Asparagus officinalis*	HM990142
Monocotyledons	Asparagaceae	*Asparagus*	*Asparagus officinalis*	HM990143
Monocotyledons	Asparagaceae	*Asparagus*	*Asparagus officinalis*	HM990144
Monocotyledons	Asparagaceae	*Asparagus*	*Asparagus officinalis*	HM990146
Monocotyledons	Asparagaceae	*Asparagus*	*Asparagus officinalis*	HM990147
Monocotyledons	Pandanaceae	*Pandanus*	*Pandanus tectorius*	JN407018
Monocotyledons	Pandanaceae	*Pandanus*	*Pandanus tectorius*	JN407019
Monocotyledons	Pandanaceae	*Pandanus*	*Pandanus tectorius*	JN407020
Monocotyledons	Typhaceae	*Typha*	*Typha latifolia*	EU750605
Monocotyledons	Typhaceae	*Typha*	*Typha latifolia*	EU750606
Monocotyledons	Typhaceae	*Typha*	*Typha latifolia*	HQ913730
Monocotyledons	Commelinaceae	*Commelina*	*Commelina benghalensis*	JN044308
Monocotyledons	Commelinaceae	*Commelina*	*Commelina benghalensis*	JN044309
Monocotyledons	Commelinaceae	*Commelina*	*Commelina benghalensis*	JN044310
Monocotyledons	Dioscoreaceae	*Dioscorea*	*Dioscorea bulbifera*	JQ260317
Monocotyledons	Dioscoreaceae	*Dioscorea*	*Dioscorea bulbifera*	JQ260318
Monocotyledons	Dioscoreaceae	*Dioscorea*	*Dioscorea bulbifera*	JQ260319

Monocotyledons	Dioscoreaceae	*Dioscorea*	*Dioscorea bulbifera*	JQ260320
Monocotyledons	Dioscoreaceae	*Dioscorea*	*Dioscorea bulbifera*	JQ260321
Monocotyledons	Dioscoreaceae	*Dioscorea*	*Dioscorea bulbifera*	JQ260322
Monocotyledons	Dioscoreaceae	*Dioscorea*	*Dioscorea bulbifera*	JQ260323
Monocotyledons	Dioscoreaceae	*Dioscorea*	*Dioscorea bulbifera*	JQ260324
Monocotyledons	Dioscoreaceae	*Dioscorea*	*Dioscorea bulbifera*	JQ260325
Monocotyledons	Dioscoreaceae	*Dioscorea*	*Dioscorea bulbifera*	JQ260326
Monocotyledons	Dioscoreaceae	*Dioscorea*	*Dioscorea bulbifera*	JQ260327
Monocotyledons	Dioscoreaceae	*Dioscorea*	*Dioscorea bulbifera*	JQ260328
Monocotyledons	Dioscoreaceae	*Dioscorea*	*Dioscorea bulbifera*	JQ260329
Monocotyledons	Dioscoreaceae	*Dioscorea*	*Dioscorea bulbifera*	GQ248290
Monocotyledons	Dioscoreaceae	*Dioscorea*	*Dioscorea bulbifera*	GU135334
Monocotyledons	Bromeliaceae	*Vriesea*	*Vriesea malzinei*	FM958006
Monocotyledons	Bromeliaceae	*Vriesea*	*Vriesea malzinei*	HQ913733
Monocotyledons	Smilacaceae	*Smilax*	*Smilax china*	GU372808
Monocotyledons	Smilacaceae	*Smilax*	*Smilax china*	GU372814
Monocotyledons	Smilacaceae	*Smilax*	*Smilax china*	GU372817
Monocotyledons	Melanthiaceae	*Veratrum*	*Veratrum album*	JF807737
Monocotyledons	Melanthiaceae	*Veratrum*	*Veratrum album*	JF807738
Monocotyledons	Melanthiaceae	*Veratrum*	*Veratrum album*	JF807739
Monocotyledons	Poaceae	*Kengyilia*	*Kengyilia alatavica*	JN045083
Monocotyledons	Poaceae	*Kengyilia*	*Kengyilia alatavica*	JN045084
Monocotyledons	Poaceae	*Kengyilia*	*Kengyilia alatavica*	HQ652842
Monocotyledons	Melanthiaceae	*Trillium*	*Trillium erectum*	DQ404230
Monocotyledons	Melanthiaceae	*Trillium*	*Trillium erectum*	HQ596871
Monocotyledons	Poaceae	*Festuca*	*Festuca rubra*	DQ369760
Monocotyledons	Poaceae	*Festuca*	*Festuca rubra*	DQ369762
Monocotyledons	Poaceae	*Festuca*	*Festuca rubra*	FJ395494
Monocotyledons	Poaceae	*Piptatherum*	*Piptatherum miliaceum*	EU204759
Monocotyledons	Poaceae	*Piptatherum*	*Piptatherum miliaceum*	EU204760
Monocotyledons	Poaceae	*Piptatherum*	*Piptatherum miliaceum*	EU204761
Monocotyledons	Musaceae	*Musa*	*Musa schizocarpa*	FJ871921
Monocotyledons	Musaceae	*Musa*	*Musa schizocarpa*	FJ871922
Monocotyledons	Musaceae	*Musa*	*Musa balbisiana*	FJ871870
Monocotyledons	Musaceae	*Musa*	*Musa balbisiana*	FJ871871
Monocotyledons	Musaceae	*Musa*	*Musa balbisiana*	FJ871872
Monocotyledons	Musaceae	*Musa*	*Musa balbisiana*	FJ871873
Monocotyledons	Musaceae	*Musa*	*Musa balbisiana*	FJ871874
Monocotyledons	Musaceae	*Musa*	*Musa balbisiana*	FJ871875
Monocotyledons	Musaceae	*Musa*	*Musa balbisiana*	FJ871876
Monocotyledons	Melanthiaceae	*Trillium*	*Trillium grandiflorum*	DQ404229
Monocotyledons	Melanthiaceae	*Trillium*	*Trillium grandiflorum*	HQ596872
Monocotyledons	Dioscoreaceae	*Dioscorea*	*Dioscorea alata*	JQ260391
Monocotyledons	Dioscoreaceae	*Dioscorea*	*Dioscorea alata*	JQ260392
Monocotyledons	Dioscoreaceae	*Dioscorea*	*Dioscorea alata*	JQ260393
Monocotyledons	Dioscoreaceae	*Dioscorea*	*Dioscorea alata*	JQ260394
Monocotyledons	Dioscoreaceae	*Dioscorea*	*Dioscorea alata*	JQ260395
Monocotyledons	Dioscoreaceae	*Dioscorea*	*Dioscorea alata*	GU135445
Monocotyledons	Dioscoreaceae	*Dioscorea*	*Dioscorea polystachya*	JQ260344
Monocotyledons	Dioscoreaceae	*Dioscorea*	*Dioscorea polystachya*	JQ260345
Monocotyledons	Dioscoreaceae	*Dioscorea*	*Dioscorea polystachya*	JQ260346
Monocotyledons	Dioscoreaceae	*Dioscorea*	*Dioscorea polystachya*	JQ260347
Monocotyledons	Dioscoreaceae	*Dioscorea*	*Dioscorea polystachya*	JQ260348

Monocotyledons	Dioscoreaceae	*Dioscorea*	*Dioscorea polystachya*	JQ260349
Monocotyledons	Dioscoreaceae	*Dioscorea*	*Dioscorea polystachya*	JQ260350
Monocotyledons	Dioscoreaceae	*Dioscorea*	*Dioscorea polystachya*	JQ260351
Monocotyledons	Dioscoreaceae	*Dioscorea*	*Dioscorea polystachya*	JQ260352
Monocotyledons	Dioscoreaceae	*Dioscorea*	*Dioscorea polystachya*	JQ260353
Monocotyledons	Dioscoreaceae	*Dioscorea*	*Dioscorea polystachya*	JQ260354
Monocotyledons	Dioscoreaceae	*Dioscorea*	*Dioscorea communis*	FJ395544
Monocotyledons	Dioscoreaceae	*Dioscorea*	*Dioscorea communis*	FR865112
Monocotyledons	Typhaceae	*Typha*	*Typha angustifolia*	GQ435010
Monocotyledons	Typhaceae	*Typha*	*Typha angustifolia*	EU750603
Monocotyledons	Typhaceae	*Typha*	*Typha angustifolia*	EU750604
Monocotyledons	Liliaceae	*Lloydia*	*Lloydia serotina*	AM238530
Monocotyledons	Liliaceae	*Lloydia*	*Lloydia serotina*	AM409337
Monocotyledons	Liliaceae	*Lloydia*	*Lloydia serotina*	EU939288
Monocotyledons	Dioscoreaceae	*Dioscorea*	*Dioscorea tokoro*	JQ260262
Monocotyledons	Dioscoreaceae	*Dioscorea*	*Dioscorea tokoro*	JQ260263
Monocotyledons	Dioscoreaceae	*Dioscorea*	*Dioscorea tokoro*	JQ260264
Monocotyledons	Dioscoreaceae	*Dioscorea*	*Dioscorea tokoro*	JQ260265
Monocotyledons	Dioscoreaceae	*Dioscorea*	*Dioscorea gracillima*	JQ260281
Monocotyledons	Dioscoreaceae	*Dioscorea*	*Dioscorea gracillima*	JQ260282
Monocotyledons	Dioscoreaceae	*Dioscorea*	*Dioscorea gracillima*	JQ260283
Monocotyledons	Dioscoreaceae	*Dioscorea*	*Dioscorea gracillima*	JQ260284
Monocotyledons	Dioscoreaceae	*Dioscorea*	*Dioscorea gracillima*	JQ260285
Monocotyledons	Dioscoreaceae	*Dioscorea*	*Dioscorea gracillima*	JQ260286
Monocotyledons	Dioscoreaceae	*Dioscorea*	*Dioscorea gracillima*	JQ260287
Monocotyledons	Dioscoreaceae	*Dioscorea*	*Dioscorea gracillima*	JQ260288
Monocotyledons	Dioscoreaceae	*Dioscorea*	*Dioscorea gracillima*	JQ260289
Monocotyledons	Dioscoreaceae	*Dioscorea*	*Dioscorea gracillima*	JQ260290
Monocotyledons	Dioscoreaceae	*Dioscorea*	*Dioscorea nipponica*	JQ260253
Monocotyledons	Dioscoreaceae	*Dioscorea*	*Dioscorea nipponica*	JQ260254
Monocotyledons	Dioscoreaceae	*Dioscorea*	*Dioscorea nipponica*	JQ260255
Monocotyledons	Dioscoreaceae	*Dioscorea*	*Dioscorea nipponica*	JQ260256
Monocotyledons	Dioscoreaceae	*Dioscorea*	*Dioscorea nipponica*	GQ435354
Monocotyledons	Dioscoreaceae	*Dioscorea*	*Dioscorea nipponica*	DQ098159
Monocotyledons	Dioscoreaceae	*Dioscorea*	*Dioscorea nipponica*	DQ124706
Monocotyledons	Dioscoreaceae	*Dioscorea*	*Dioscorea septemloba*	JQ260301
Monocotyledons	Dioscoreaceae	*Dioscorea*	*Dioscorea septemloba*	JQ260302
Monocotyledons	Melanthiaceae	*Veratrum*	*Veratrum viride*	JF807784
Monocotyledons	Melanthiaceae	*Veratrum*	*Veratrum viride*	JF807785
Monocotyledons	Cyperaceae	*Cyperus*	*Cyperus involucratus*	GU135417
Monocotyledons	Cyperaceae	*Cyperus*	*Cyperus involucratus*	GU135444
Monocotyledons	Liliaceae	*Lilium*	*Lilium pumilum*	HM053713
Monocotyledons	Liliaceae	*Lilium*	*Lilium pumilum*	HM053714
Monocotyledons	Liliaceae	*Lilium*	*Lilium pumilum*	HM053715
Monocotyledons	Liliaceae	*Lilium*	*Lilium pumilum*	HM053716
Monocotyledons	Liliaceae	*Lilium*	*Lilium pumilum*	HM053717
Monocotyledons	Liliaceae	*Lilium*	*Lilium pumilum*	HM053718
Monocotyledons	Liliaceae	*Lilium*	*Lilium pumilum*	HM053719
Monocotyledons	Liliaceae	*Lilium*	*Lilium pumilum*	HM053720
Monocotyledons	Liliaceae	*Lilium*	*Lilium pumilum*	HM053721
Monocotyledons	Liliaceae	*Lilium*	*Lilium pumilum*	HM053722
Monocotyledons	Liliaceae	*Lilium*	*Lilium pumilum*	GQ434916
Monocotyledons	Melanthiaceae	*Trillium*	*Trillium ovatum*	DQ404228

Monocotyledons	Melanthiaceae	*Trillium*	*Trillium ovatum*	AY727187
Monocotyledons	Melanthiaceae	*Paris*	*Paris fargesii*	DQ404251
Monocotyledons	Melanthiaceae	*Paris*	*Paris fargesii*	JN045717
Monocotyledons	Melanthiaceae	*Paris*	*Paris fargesii*	JN045718
Monocotyledons	Melanthiaceae	*Paris*	*Paris fargesii*	JN045719
Monocotyledons	Melanthiaceae	*Paris*	*Paris fargesii*	JN045720
Monocotyledons	Melanthiaceae	*Paris*	*Paris fargesii*	JN045721
Monocotyledons	Melanthiaceae	*Paris*	*Paris fargesii*	JN045722
Monocotyledons	Melanthiaceae	*Paris*	*Paris thibetica*	DQ404250
Monocotyledons	Melanthiaceae	*Paris*	*Paris thibetica*	JN045763
Monocotyledons	Melanthiaceae	*Paris*	*Paris thibetica*	JN045764
Monocotyledons	Melanthiaceae	*Paris*	*Paris thibetica*	JN045765
Monocotyledons	Melanthiaceae	*Paris*	*Paris thibetica*	JN045766
Monocotyledons	Melanthiaceae	*Paris*	*Paris thibetica*	JN045767
Monocotyledons	Melanthiaceae	*Paris*	*Paris thibetica*	JN045768
Monocotyledons	Melanthiaceae	*Paris*	*Paris thibetica*	JN045769
Monocotyledons	Melanthiaceae	*Paris*	*Paris thibetica*	JN045770
Monocotyledons	Melanthiaceae	*Paris*	*Paris thibetica*	JN045771
Monocotyledons	Melanthiaceae	*Paris*	*Paris incompleta*	DQ404237
Monocotyledons	Melanthiaceae	*Paris*	*Paris incompleta*	JN045723
Monocotyledons	Melanthiaceae	*Paris*	*Paris incompleta*	JN045724
Monocotyledons	Melanthiaceae	*Paris*	*Paris incompleta*	JN045725
Monocotyledons	Liliaceae	*Lilium*	*Lilium philadelphicum*	DQ122704
Monocotyledons	Liliaceae	*Lilium*	*Lilium philadelphicum*	DQ122705
Monocotyledons	Liliaceae	*Lilium*	*Lilium philadelphicum*	DQ122706
Monocotyledons	Liliaceae	*Lilium*	*Lilium philadelphicum*	DQ122707
Monocotyledons	Liliaceae	*Lilium*	*Lilium philadelphicum*	DQ122708
Monocotyledons	Liliaceae	*Lilium*	*Lilium philadelphicum*	DQ122709
Monocotyledons	Liliaceae	*Lilium*	*Lilium philadelphicum*	DQ122710
Monocotyledons	Liliaceae	*Lilium*	*Lilium philadelphicum*	DQ122711
Monocotyledons	Liliaceae	*Lilium*	*Lilium philadelphicum*	DQ122712
Monocotyledons	Liliaceae	*Lilium*	*Lilium philadelphicum*	DQ122713
Monocotyledons	Liliaceae	*Lilium*	*Lilium philadelphicum*	DQ122714
Monocotyledons	Liliaceae	*Lilium*	*Lilium philadelphicum*	DQ122715
Monocotyledons	Liliaceae	*Lilium*	*Lilium philadelphicum*	DQ122716
Monocotyledons	Liliaceae	*Lilium*	*Lilium philadelphicum*	DQ122717
Monocotyledons	Liliaceae	*Lilium*	*Lilium philadelphicum*	DQ122718
Monocotyledons	Liliaceae	*Lilium*	*Lilium philadelphicum*	DQ122719
Monocotyledons	Liliaceae	*Lilium*	*Lilium philadelphicum*	DQ122720
Monocotyledons	Amaryllidaceae	*Allium*	*Allium macrostemon*	GQ434917
Monocotyledons	Amaryllidaceae	*Allium*	*Allium macrostemon*	GQ434918
Monocotyledons	Amaryllidaceae	*Allium*	*Allium macrostemon*	GQ434919
Monocotyledons	Poaceae	*Festuca*	*Festuca idahoensis*	DQ369764
Monocotyledons	Poaceae	*Festuca*	*Festuca idahoensis*	DQ369766
Monocotyledons	Poaceae	*Festuca*	*Festuca idahoensis*	DQ369770
Monocotyledons	Poaceae	*Festuca*	*Festuca idahoensis*	DQ369774
Monocotyledons	Poaceae	*Festuca*	*Festuca idahoensis*	DQ369778
Monocotyledons	Poaceae	*Festuca*	*Festuca valesiaca*	DQ369758
Monocotyledons	Poaceae	*Festuca*	*Festuca valesiaca*	DQ369776
Monocotyledons	Asparagaceae	*Asparagus*	*Asparagus cochinchinensis*	GQ434909
Monocotyledons	Asparagaceae	*Asparagus*	*Asparagus cochinchinensis*	GQ434910
Monocotyledons	Asparagaceae	*Asparagus*	*Asparagus cochinchinensis*	GQ434911
Monocotyledons	Poaceae	*Kengyilia*	*Kengyilia rigidula*	JN045115

Monocotyledons	Poaceae	*Kengyilia*	*Kengyilia rigidula*	JN045116
Monocotyledons	Poaceae	*Kengyilia*	*Kengyilia rigidula*	JN045117
Monocotyledons	Poaceae	*Kengyilia*	*Kengyilia rigidula*	HQ221838
Monocotyledons	Poaceae	*Kengyilia*	*Kengyilia rigidula*	HQ221841
Monocotyledons	Poaceae	*Kengyilia*	*Kengyilia rigidula*	HQ221848
Monocotyledons	Poaceae	*Kengyilia*	*Kengyilia rigidula*	HQ221849
Monocotyledons	Poaceae	*Kengyilia*	*Kengyilia rigidula*	HQ221850
Monocotyledons	Poaceae	*Kengyilia*	*Kengyilia rigidula*	HQ652818
Monocotyledons	Poaceae	*Kengyilia*	*Kengyilia rigidula*	HQ652819
Monocotyledons	Poaceae	*Kengyilia*	*Kengyilia rigidula*	HQ652826
Monocotyledons	Poaceae	*Kengyilia*	*Kengyilia rigidula*	HQ652827
Monocotyledons	Poaceae	*Kengyilia*	*Kengyilia rigidula*	HQ652830
Monocotyledons	Poaceae	*Kengyilia*	*Kengyilia rigidula*	HQ652831
Monocotyledons	Poaceae	*Kengyilia*	*Kengyilia rigidula*	HQ652833
Monocotyledons	Poaceae	*Kengyilia*	*Kengyilia grandiglumis*	JN045092
Monocotyledons	Poaceae	*Kengyilia*	*Kengyilia grandiglumis*	JN045093
Monocotyledons	Poaceae	*Kengyilia*	*Kengyilia grandiglumis*	JN045094
Monocotyledons	Poaceae	*Kengyilia*	*Kengyilia grandiglumis*	HQ652811
Monocotyledons	Poaceae	*Kengyilia*	*Kengyilia grandiglumis*	HQ652829
Monocotyledons	Poaceae	*Kengyilia*	*Kengyilia grandiglumis*	HQ652851
Monocotyledons	Poaceae	*Kengyilia*	*Kengyilia kokonorica*	JN045098
Monocotyledons	Poaceae	*Kengyilia*	*Kengyilia kokonorica*	JN045099
Monocotyledons	Poaceae	*Kengyilia*	*Kengyilia kokonorica*	JN045100
Monocotyledons	Poaceae	*Kengyilia*	*Kengyilia kokonorica*	JN045101
Monocotyledons	Poaceae	*Kengyilia*	*Kengyilia kokonorica*	HQ652832
Monocotyledons	Poaceae	*Kengyilia*	*Kengyilia kokonorica*	HQ652834
Monocotyledons	Poaceae	*Kengyilia*	*Kengyilia kokonorica*	HQ652857
Monocotyledons	Poaceae	*Kengyilia*	*Kengyilia kokonorica*	HQ652858
Monocotyledons	Poaceae	*Kengyilia*	*Kengyilia laxiflora*	JN045102
Monocotyledons	Poaceae	*Kengyilia*	*Kengyilia laxiflora*	JN045103
Monocotyledons	Poaceae	*Kengyilia*	*Kengyilia laxiflora*	JN045104
Monocotyledons	Poaceae	*Kengyilia*	*Kengyilia laxiflora*	HQ652835
Monocotyledons	Poaceae	*Kengyilia*	*Kengyilia laxiflora*	HQ652836
Monocotyledons	Poaceae	*Kengyilia*	*Kengyilia laxiflora*	HQ652859
Monocotyledons	Poaceae	*Kengyilia*	*Kengyilia melanthera*	JN045108
Monocotyledons	Poaceae	*Kengyilia*	*Kengyilia melanthera*	JN045109
Monocotyledons	Poaceae	*Kengyilia*	*Kengyilia melanthera*	JN045110
Monocotyledons	Poaceae	*Kengyilia*	*Kengyilia melanthera*	HQ221839
Monocotyledons	Poaceae	*Kengyilia*	*Kengyilia melanthera*	HQ221840
Monocotyledons	Poaceae	*Kengyilia*	*Kengyilia melanthera*	HQ221843
Monocotyledons	Poaceae	*Kengyilia*	*Kengyilia melanthera*	HQ221844
Monocotyledons	Poaceae	*Kengyilia*	*Kengyilia melanthera*	HQ221846
Monocotyledons	Poaceae	*Kengyilia*	*Kengyilia melanthera*	HQ652801
Monocotyledons	Poaceae	*Kengyilia*	*Kengyilia melanthera*	HQ652810
Monocotyledons	Poaceae	*Kengyilia*	*Kengyilia melanthera*	HQ652864
Monocotyledons	Poaceae	*Kengyilia*	*Kengyilia melanthera*	HQ652865
Monocotyledons	Poaceae	*Kengyilia*	*Kengyilia melanthera*	HQ652866
Monocotyledons	Poaceae	*Kengyilia*	*Kengyilia mutica*	JN045113
Monocotyledons	Poaceae	*Kengyilia*	*Kengyilia mutica*	JN045114
Monocotyledons	Poaceae	*Kengyilia*	*Kengyilia mutica*	HQ652823
Monocotyledons	Poaceae	*Kengyilia*	*Kengyilia mutica*	HQ652825
Monocotyledons	Poaceae	*Kengyilia*	*Kengyilia mutica*	HQ652867
Monocotyledons	Poaceae	*Kengyilia*	*Kengyilia thoroldiana*	JN045121

Monocotyledons	Poaceae	*Kengyilia*	*Kengyilia thoroldiana*	JN045122
Monocotyledons	Poaceae	*Kengyilia*	*Kengyilia thoroldiana*	JN045123
Monocotyledons	Poaceae	*Kengyilia*	*Kengyilia thoroldiana*	HQ652814
Monocotyledons	Poaceae	*Kengyilia*	*Kengyilia thoroldiana*	HQ652815
Monocotyledons	Poaceae	*Kengyilia*	*Kengyilia thoroldiana*	HQ652816
Monocotyledons	Poaceae	*Kengyilia*	*Kengyilia thoroldiana*	HQ652824
Monocotyledons	Poaceae	*Kengyilia*	*Kengyilia batalinii*	JN045085
Monocotyledons	Poaceae	*Kengyilia*	*Kengyilia batalinii*	JN045086
Monocotyledons	Poaceae	*Kengyilia*	*Kengyilia batalinii*	JN045087
Monocotyledons	Poaceae	*Kengyilia*	*Kengyilia batalinii*	HQ652843
Monocotyledons	Poaceae	*Kengyilia*	*Kengyilia batalinii*	HQ652844
Monocotyledons	Poaceae	*Kengyilia*	*Kengyilia batalinii*	HQ652845
Monocotyledons	Poaceae	*Kengyilia*	*Kengyilia batalinii*	HQ652846
Monocotyledons	Musaceae	*Musa*	*Musa basjoo*	FJ871913
Monocotyledons	Musaceae	*Musa*	*Musa basjoo*	FJ871914
Monocotyledons	Musaceae	*Musa*	*Musa basjoo*	JN045497
Monocotyledons	Musaceae	*Musa*	*Musa basjoo*	JN045498
Monocotyledons	Musaceae	*Musa*	*Musa basjoo*	JN045499
Monocotyledons	Amaryllidaceae	*Allium*	*Allium przewalskianum*	GU121988
Monocotyledons	Amaryllidaceae	*Allium*	*Allium przewalskianum*	GU121989
Monocotyledons	Amaryllidaceae	*Allium*	*Allium przewalskianum*	GU121990
Monocotyledons	Amaryllidaceae	*Allium*	*Allium przewalskianum*	GU121991
Monocotyledons	Dioscoreaceae	*Dioscorea*	*Dioscorea decipiens*	JQ260396
Monocotyledons	Dioscoreaceae	*Dioscorea*	*Dioscorea decipiens*	JQ260397
Monocotyledons	Dioscoreaceae	*Dioscorea*	*Dioscorea decipiens*	JQ260398
Monocotyledons	Dioscoreaceae	*Dioscorea*	*Dioscorea decipiens*	JQ260399
Monocotyledons	Dioscoreaceae	*Dioscorea*	*Dioscorea glabra*	JQ260372
Monocotyledons	Dioscoreaceae	*Dioscorea*	*Dioscorea glabra*	JQ260373
Monocotyledons	Dioscoreaceae	*Dioscorea*	*Dioscorea glabra*	JQ260374
Monocotyledons	Dioscoreaceae	*Dioscorea*	*Dioscorea hispida*	JQ260341
Monocotyledons	Dioscoreaceae	*Dioscorea*	*Dioscorea hispida*	JQ260342
Monocotyledons	Dioscoreaceae	*Dioscorea*	*Dioscorea pentaphylla*	JQ260336
Monocotyledons	Dioscoreaceae	*Dioscorea*	*Dioscorea pentaphylla*	JQ260337
Monocotyledons	Dioscoreaceae	*Dioscorea*	*Dioscorea pentaphylla*	JQ260338
Monocotyledons	Dioscoreaceae	*Dioscorea*	*Dioscorea pentaphylla*	JQ260339
Monocotyledons	Musaceae	*Musa*	*Musa beccarii*	FJ871905
Monocotyledons	Musaceae	*Musa*	*Musa beccarii*	FJ871906
Monocotyledons	Liliaceae	*Erythronium*	*Erythronium elegans*	EU311877
Monocotyledons	Liliaceae	*Erythronium*	*Erythronium elegans*	EU311878
Monocotyledons	Liliaceae	*Erythronium*	*Erythronium elegans*	EU311879
Monocotyledons	Liliaceae	*Erythronium*	*Erythronium elegans*	EU311880
Monocotyledons	Liliaceae	*Erythronium*	*Erythronium elegans*	EU311881
Monocotyledons	Liliaceae	*Erythronium*	*Erythronium elegans*	EU311882
Monocotyledons	Liliaceae	*Erythronium*	*Erythronium elegans*	EU311883
Monocotyledons	Liliaceae	*Erythronium*	*Erythronium elegans*	EU311884
Monocotyledons	Liliaceae	*Erythronium*	*Erythronium elegans*	EU311885
Monocotyledons	Liliaceae	*Erythronium*	*Erythronium elegans*	EU311886
Monocotyledons	Liliaceae	*Erythronium*	*Erythronium elegans*	EU311887
Monocotyledons	Liliaceae	*Erythronium*	*Erythronium elegans*	EU311888
Monocotyledons	Liliaceae	*Erythronium*	*Erythronium elegans*	EU311889
Monocotyledons	Liliaceae	*Erythronium*	*Erythronium elegans*	EU311890
Monocotyledons	Liliaceae	*Erythronium*	*Erythronium elegans*	EU311891
Monocotyledons	Liliaceae	*Erythronium*	*Erythronium elegans*	EU311892

Monocotyledons	Liliaceae	*Erythronium*	*Erythronium klamathense*	EU311852
Monocotyledons	Liliaceae	*Erythronium*	*Erythronium klamathense*	EU311853
Monocotyledons	Liliaceae	*Erythronium*	*Erythronium montanum*	EU311854
Monocotyledons	Liliaceae	*Erythronium*	*Erythronium montanum*	EU311855
Monocotyledons	Liliaceae	*Erythronium*	*Erythronium montanum*	EU311856
Monocotyledons	Liliaceae	*Erythronium*	*Erythronium montanum*	EU311857
Monocotyledons	Liliaceae	*Erythronium*	*Erythronium montanum*	EU311858
Monocotyledons	Liliaceae	*Erythronium*	*Erythronium montanum*	EU311859
Monocotyledons	Liliaceae	*Erythronium*	*Erythronium montanum*	EU311860
Monocotyledons	Liliaceae	*Erythronium*	*Erythronium montanum*	EU311861
Monocotyledons	Liliaceae	*Erythronium*	*Erythronium oregonum*	EU311862
Monocotyledons	Liliaceae	*Erythronium*	*Erythronium oregonum*	EU311863
Monocotyledons	Liliaceae	*Erythronium*	*Erythronium oregonum*	EU311864
Monocotyledons	Liliaceae	*Erythronium*	*Erythronium oregonum*	EU311865
Monocotyledons	Liliaceae	*Erythronium*	*Erythronium oregonum*	EU311866
Monocotyledons	Liliaceae	*Erythronium*	*Erythronium oregonum*	EU311867
Monocotyledons	Liliaceae	*Erythronium*	*Erythronium quinaultense*	EU311893
Monocotyledons	Liliaceae	*Erythronium*	*Erythronium quinaultense*	EU311894
Monocotyledons	Liliaceae	*Erythronium*	*Erythronium quinaultense*	EU311895
Monocotyledons	Liliaceae	*Erythronium*	*Erythronium quinaultense*	EU311896
Monocotyledons	Liliaceae	*Erythronium*	*Erythronium quinaultense*	EU311897
Monocotyledons	Liliaceae	*Erythronium*	*Erythronium quinaultense*	EU311898
Monocotyledons	Liliaceae	*Erythronium*	*Erythronium quinaultense*	EU311899
Monocotyledons	Liliaceae	*Erythronium*	*Erythronium revolutum*	EU311868
Monocotyledons	Liliaceae	*Erythronium*	*Erythronium revolutum*	EU311869
Monocotyledons	Liliaceae	*Erythronium*	*Erythronium revolutum*	EU311870
Monocotyledons	Liliaceae	*Erythronium*	*Erythronium revolutum*	EU311871
Monocotyledons	Liliaceae	*Erythronium*	*Erythronium revolutum*	EU311872
Monocotyledons	Liliaceae	*Erythronium*	*Erythronium revolutum*	EU311873
Monocotyledons	Liliaceae	*Erythronium*	*Erythronium revolutum*	EU311874
Monocotyledons	Liliaceae	*Erythronium*	*Erythronium revolutum*	EU311875
Monocotyledons	Liliaceae	*Erythronium*	*Erythronium revolutum*	EU311876
Monocotyledons	Melanthiaceae	*Veratrum*	*Veratrum nigrum*	GQ434892
Monocotyledons	Melanthiaceae	*Veratrum*	*Veratrum nigrum*	FN675816
Monocotyledons	Colchicaceae	*Androcymbium*	*Androcymbium austrocapense*	DQ088279
Monocotyledons	Colchicaceae	*Androcymbium*	*Androcymbium austrocapense*	DQ088280
Monocotyledons	Melanthiaceae	*Paris*	*Paris cronquistii*	JN045701
Monocotyledons	Melanthiaceae	*Paris*	*Paris cronquistii*	JN045702
Monocotyledons	Melanthiaceae	*Paris*	*Paris cronquistii*	JN045703
Monocotyledons	Melanthiaceae	*Paris*	*Paris daliensis*	DQ404260
Monocotyledons	Melanthiaceae	*Paris*	*Paris daliensis*	JN045704
Monocotyledons	Melanthiaceae	*Paris*	*Paris daliensis*	JN045705
Monocotyledons	Melanthiaceae	*Paris*	*Paris luquanensis*	GU178892
Monocotyledons	Melanthiaceae	*Paris*	*Paris luquanensis*	JN045726
Monocotyledons	Melanthiaceae	*Paris*	*Paris luquanensis*	JN045727
Monocotyledons	Melanthiaceae	*Paris*	*Paris luquanensis*	JN045728
Monocotyledons	Melanthiaceae	*Paris*	*Paris marmorata*	DQ404256
Monocotyledons	Melanthiaceae	*Paris*	*Paris marmorata*	GU178890
Monocotyledons	Melanthiaceae	*Paris*	*Paris marmorata*	JN045735
Monocotyledons	Melanthiaceae	*Paris*	*Paris marmorata*	JN045736
Monocotyledons	Melanthiaceae	*Paris*	*Paris marmorata*	JN045737
Monocotyledons	Melanthiaceae	*Paris*	*Paris marmorata*	JN045738
Monocotyledons	Melanthiaceae	*Paris*	*Paris marmorata*	JN045739

Monocotyledons	Araceae	*Arisaema*	*Arisaema flavum*	JN043994
Monocotyledons	Araceae	*Arisaema*	*Arisaema flavum*	JN043995
Monocotyledons	Araceae	*Arisaema*	*Arisaema heterophyllum*	JN043996
Monocotyledons	Araceae	*Arisaema*	*Arisaema heterophyllum*	JN043997
Monocotyledons	Araceae	*Arisaema*	*Arisaema heterophyllum*	JN043998
Monocotyledons	Araceae	*Arisaema*	*Arisaema rhizomatum*	JN043999
Monocotyledons	Araceae	*Arisaema*	*Arisaema rhizomatum*	JN044000
Monocotyledons	Musaceae	*Musa*	*Musa velutina*	FJ871917
Monocotyledons	Musaceae	*Musa*	*Musa velutina*	FJ871918
Monocotyledons	Musaceae	*Musa*	*Musa textilis*	FJ871896
Monocotyledons	Musaceae	*Musa*	*Musa textilis*	FJ871897
Monocotyledons	Musaceae	*Musa*	*Musa textilis*	FJ871898
Monocotyledons	Araceae	*Arisaema*	*Arisaema erubescens*	JN043992
Monocotyledons	Araceae	*Arisaema*	*Arisaema erubescens*	JN043993
Monocotyledons	Melanthiaceae	*Paris*	*Paris quadrifolia*	DQ404238
Monocotyledons	Melanthiaceae	*Paris*	*Paris quadrifolia*	JN045757
Monocotyledons	Melanthiaceae	*Paris*	*Paris quadrifolia*	JN045758
Monocotyledons	Melanthiaceae	*Paris*	*Paris quadrifolia*	JN045759
Monocotyledons	Asparagaceae	*Asparagus*	*Asparagus densiflorus*	HM990125
Monocotyledons	Asparagaceae	*Asparagus*	*Asparagus densiflorus*	HM990128
Monocotyledons	Asparagaceae	*Asparagus*	*Asparagus densiflorus*	HM990130
Monocotyledons	Asparagaceae	*Asparagus*	*Asparagus densiflorus*	HM990141
Monocotyledons	Colchicaceae	*Androcymbium*	*Androcymbium gramineum*	DQ088292
Monocotyledons	Colchicaceae	*Androcymbium*	*Androcymbium gramineum*	DQ088293
Monocotyledons	Colchicaceae	*Androcymbium*	*Androcymbium wyssianum*	DQ088316
Monocotyledons	Colchicaceae	*Androcymbium*	*Androcymbium wyssianum*	DQ088317
Monocotyledons	Colchicaceae	*Androcymbium*	*Androcymbium huntleyi*	DQ088297
Monocotyledons	Colchicaceae	*Androcymbium*	*Androcymbium huntleyi*	DQ088298
Monocotyledons	Colchicaceae	*Androcymbium*	*Androcymbium irroratum*	DQ088299
Monocotyledons	Colchicaceae	*Androcymbium*	*Androcymbium irroratum*	DQ088300
Monocotyledons	Colchicaceae	*Androcymbium*	*Androcymbium irroratum*	DQ088301
Monocotyledons	Colchicaceae	*Androcymbium*	*Androcymbium irroratum*	DQ088302
Monocotyledons	Colchicaceae	*Androcymbium*	*Androcymbium irroratum*	DQ088303
Monocotyledons	Colchicaceae	*Androcymbium*	*Androcymbium poeltianum*	DQ088308
Monocotyledons	Colchicaceae	*Androcymbium*	*Androcymbium poeltianum*	DQ088309
Monocotyledons	Bromeliaceae	*Vriesea*	*Vriesea carinata*	JN204671
Monocotyledons	Bromeliaceae	*Vriesea*	*Vriesea carinata*	JN204672
Monocotyledons	Dioscoreaceae	*Dioscorea*	*Dioscorea cirrhosa*	JQ260362
Monocotyledons	Dioscoreaceae	*Dioscorea*	*Dioscorea cirrhosa*	JQ260363
Monocotyledons	Dioscoreaceae	*Dioscorea*	*Dioscorea cirrhosa*	JQ260364
Monocotyledons	Dioscoreaceae	*Dioscorea*	*Dioscorea cirrhosa*	JQ260365
Monocotyledons	Dioscoreaceae	*Dioscorea*	*Dioscorea cirrhosa*	JQ260366
Monocotyledons	Dioscoreaceae	*Dioscorea*	*Dioscorea cirrhosa*	JQ260367
Monocotyledons	Dioscoreaceae	*Dioscorea*	*Dioscorea cirrhosa*	JQ260368
Monocotyledons	Dioscoreaceae	*Dioscorea*	*Dioscorea cirrhosa*	JQ260369
Monocotyledons	Dioscoreaceae	*Dioscorea*	*Dioscorea cirrhosa*	JQ260370
Monocotyledons	Dioscoreaceae	*Dioscorea*	*Dioscorea zingiberensis*	JQ260266
Monocotyledons	Dioscoreaceae	*Dioscorea*	*Dioscorea zingiberensis*	JQ260267
Monocotyledons	Dioscoreaceae	*Dioscorea*	*Dioscorea zingiberensis*	JQ260268
Monocotyledons	Dioscoreaceae	*Dioscorea*	*Dioscorea zingiberensis*	JQ260269
Monocotyledons	Dioscoreaceae	*Dioscorea*	*Dioscorea zingiberensis*	JQ260270
Monocotyledons	Dioscoreaceae	*Dioscorea*	*Dioscorea zingiberensis*	JQ260271
Monocotyledons	Dioscoreaceae	*Dioscorea*	*Dioscorea zingiberensis*	JQ260272

Monocotyledons	Dioscoreaceae	*Dioscorea*	*Dioscorea zingiberensis*	JQ260273
Monocotyledons	Dioscoreaceae	*Dioscorea*	*Dioscorea zingiberensis*	JQ260274
Monocotyledons	Dioscoreaceae	*Dioscorea*	*Dioscorea zingiberensis*	JQ260275
Monocotyledons	Dioscoreaceae	*Dioscorea*	*Dioscorea zingiberensis*	DQ131104
Monocotyledons	Dioscoreaceae	*Dioscorea*	*Dioscorea zingiberensis*	DQ131105
Monocotyledons	Dioscoreaceae	*Dioscorea*	*Dioscorea zingiberensis*	DQ191746
Monocotyledons	Dioscoreaceae	*Dioscorea*	*Dioscorea zingiberensis*	DQ191747
Monocotyledons	Dioscoreaceae	*Dioscorea*	*Dioscorea zingiberensis*	DQ191748
Monocotyledons	Dioscoreaceae	*Dioscorea*	*Dioscorea zingiberensis*	DQ191749
Monocotyledons	Bromeliaceae	*Nidularium*	*Nidularium procerum*	JN204655
Monocotyledons	Bromeliaceae	*Nidularium*	*Nidularium procerum*	JN204656
Monocotyledons	Dioscoreaceae	*Dioscorea*	*Dioscorea panthaica*	JQ260278
Monocotyledons	Dioscoreaceae	*Dioscorea*	*Dioscorea panthaica*	JQ260279
Monocotyledons	Dioscoreaceae	*Dioscorea*	*Dioscorea panthaica*	DQ098160
Monocotyledons	Dioscoreaceae	*Dioscorea*	*Dioscorea panthaica*	DQ124704
Monocotyledons	Dioscoreaceae	*Dioscorea*	*Dioscorea panthaica*	DQ124705
Monocotyledons	Dioscoreaceae	*Dioscorea*	*Dioscorea collettii*	JQ260291
Monocotyledons	Dioscoreaceae	*Dioscorea*	*Dioscorea collettii*	JQ260292
Monocotyledons	Dioscoreaceae	*Dioscorea*	*Dioscorea collettii*	JQ260293
Monocotyledons	Dioscoreaceae	*Dioscorea*	*Dioscorea collettii*	JQ260294
Monocotyledons	Poaceae	*Festuca*	*Festuca roemeri*	DQ369768
Monocotyledons	Poaceae	*Festuca*	*Festuca roemeri*	DQ369772
Monocotyledons	Poaceae	*Festuca*	*Festuca roemeri*	DQ369780
Monocotyledons	Poaceae	*Festuca*	*Festuca roemeri*	DQ369782
Monocotyledons	Poaceae	*Festuca*	*Festuca roemeri*	DQ369784
Monocotyledons	Poaceae	*Festuca*	*Festuca roemeri*	DQ369786
Monocotyledons	Poaceae	*Festuca*	*Festuca roemeri*	DQ369788
Monocotyledons	Poaceae	*Festuca*	*Festuca roemeri*	DQ369790
Monocotyledons	Melanthiaceae	*Paris*	*Paris delavayi*	GU178891
Monocotyledons	Melanthiaceae	*Paris*	*Paris delavayi*	JN045706
Monocotyledons	Melanthiaceae	*Paris*	*Paris delavayi*	JN045707
Monocotyledons	Melanthiaceae	*Paris*	*Paris delavayi*	JN045708
Monocotyledons	Melanthiaceae	*Paris*	*Paris delavayi*	JN045709
Monocotyledons	Melanthiaceae	*Paris*	*Paris delavayi*	JN045710
Monocotyledons	Melanthiaceae	*Paris*	*Paris delavayi*	JN045711
Monocotyledons	Melanthiaceae	*Paris*	*Paris dulongensis*	DQ404241
Monocotyledons	Melanthiaceae	*Paris*	*Paris dulongensis*	JN045712
Monocotyledons	Melanthiaceae	*Paris*	*Paris dulongensis*	JN045713
Monocotyledons	Melanthiaceae	*Paris*	*Paris dunniana*	DQ404259
Monocotyledons	Melanthiaceae	*Paris*	*Paris dunniana*	JN045714
Monocotyledons	Melanthiaceae	*Paris*	*Paris dunniana*	JN045715
Monocotyledons	Melanthiaceae	*Paris*	*Paris dunniana*	JN045716
Monocotyledons	Melanthiaceae	*Paris*	*Paris mairei*	DQ404247
Monocotyledons	Melanthiaceae	*Paris*	*Paris mairei*	JN045729
Monocotyledons	Melanthiaceae	*Paris*	*Paris mairei*	JN045730
Monocotyledons	Melanthiaceae	*Paris*	*Paris mairei*	JN045731
Monocotyledons	Melanthiaceae	*Paris*	*Paris mairei*	JN045732
Monocotyledons	Melanthiaceae	*Paris*	*Paris mairei*	JN045733
Monocotyledons	Melanthiaceae	*Paris*	*Paris mairei*	JN045734
Monocotyledons	Melanthiaceae	*Paris*	*Paris rugosa*	DQ404245
Monocotyledons	Melanthiaceae	*Paris*	*Paris rugosa*	JN045760
Monocotyledons	Melanthiaceae	*Paris*	*Paris rugosa*	JN045761
Monocotyledons	Melanthiaceae	*Paris*	*Paris rugosa*	JN045762

Monocotyledons	Melanthiaceae	*Paris*	*Paris vaniotii*	DQ404243
Monocotyledons	Melanthiaceae	*Paris*	*Paris vaniotii*	JN045772
Monocotyledons	Melanthiaceae	*Paris*	*Paris vaniotii*	JN045773
Monocotyledons	Melanthiaceae	*Paris*	*Paris vaniotii*	JN045774
Monocotyledons	Melanthiaceae	*Paris*	*Paris vietnamensis*	DQ404246
Monocotyledons	Melanthiaceae	*Paris*	*Paris vietnamensis*	GU178893
Monocotyledons	Melanthiaceae	*Paris*	*Paris vietnamensis*	JN045775
Monocotyledons	Melanthiaceae	*Paris*	*Paris vietnamensis*	JN045776
Monocotyledons	Melanthiaceae	*Paris*	*Paris vietnamensis*	JN045777
Monocotyledons	Melanthiaceae	*Paris*	*Paris vietnamensis*	JN045778
Monocotyledons	Melanthiaceae	*Paris*	*Paris vietnamensis*	JN045779
Monocotyledons	Melanthiaceae	*Paris*	*Paris vietnamensis*	JN045780
Monocotyledons	Dioscoreaceae	*Dioscorea*	*Dioscorea persimilis*	JQ260379
Monocotyledons	Dioscoreaceae	*Dioscorea*	*Dioscorea persimilis*	JQ260380
Monocotyledons	Dioscoreaceae	*Dioscorea*	*Dioscorea persimilis*	JQ260381
Monocotyledons	Dioscoreaceae	*Dioscorea*	*Dioscorea persimilis*	JQ260382
Monocotyledons	Dioscoreaceae	*Dioscorea*	*Dioscorea persimilis*	JQ260383
Monocotyledons	Dioscoreaceae	*Dioscorea*	*Dioscorea futschauensis*	JQ260299
Monocotyledons	Dioscoreaceae	*Dioscorea*	*Dioscorea futschauensis*	JQ260300
Monocotyledons	Dioscoreaceae	*Dioscorea*	*Dioscorea exalata*	JQ260385
Monocotyledons	Dioscoreaceae	*Dioscorea*	*Dioscorea exalata*	JQ260386
Monocotyledons	Dioscoreaceae	*Dioscorea*	*Dioscorea exalata*	JQ260387
Monocotyledons	Dioscoreaceae	*Dioscorea*	*Dioscorea exalata*	JQ260388
Monocotyledons	Dioscoreaceae	*Dioscorea*	*Dioscorea exalata*	JQ260389
Monocotyledons	Dioscoreaceae	*Dioscorea*	*Dioscorea exalata*	JQ260390
Monocotyledons	Dioscoreaceae	*Dioscorea*	*Dioscorea kamoonensis*	JQ260330
Monocotyledons	Dioscoreaceae	*Dioscorea*	*Dioscorea kamoonensis*	JQ260331
Monocotyledons	Dioscoreaceae	*Dioscorea*	*Dioscorea kamoonensis*	JQ260332
Monocotyledons	Melanthiaceae	*Veratrum*	*Veratrum oxysepalum*	JF807759
Monocotyledons	Melanthiaceae	*Veratrum*	*Veratrum oxysepalum*	JF807760
Monocotyledons	Melanthiaceae	*Veratrum*	*Veratrum oxysepalum*	JF807761
Monocotyledons	Melanthiaceae	*Veratrum*	*Veratrum oxysepalum*	JF807762
Monocotyledons	Melanthiaceae	*Veratrum*	*Veratrum oxysepalum*	JF807763
Monocotyledons	Melanthiaceae	*Veratrum*	*Veratrum oxysepalum*	JF807764
Monocotyledons	Melanthiaceae	*Veratrum*	*Veratrum oxysepalum*	JF807765
Monocotyledons	Melanthiaceae	*Veratrum*	*Veratrum oxysepalum*	JF807766
Monocotyledons	Melanthiaceae	*Veratrum*	*Veratrum oxysepalum*	JF807767
Monocotyledons	Melanthiaceae	*Veratrum*	*Veratrum oxysepalum*	JF807768
Monocotyledons	Melanthiaceae	*Veratrum*	*Veratrum oxysepalum*	JF807769
Monocotyledons	Melanthiaceae	*Veratrum*	*Veratrum oxysepalum*	JF807770
Monocotyledons	Melanthiaceae	*Veratrum*	*Veratrum oxysepalum*	JF807771
Monocotyledons	Dioscoreaceae	*Dioscorea*	*Dioscorea fordii*	JQ260375
Monocotyledons	Dioscoreaceae	*Dioscorea*	*Dioscorea fordii*	JQ260376
Monocotyledons	Dioscoreaceae	*Dioscorea*	*Dioscorea fordii*	JQ260377
Monocotyledons	Dioscoreaceae	*Dioscorea*	*Dioscorea fordii*	JQ260378
Monocotyledons	Poaceae	*Kengyilia*	*Kengyilia gobicola*	JN045090
Monocotyledons	Poaceae	*Kengyilia*	*Kengyilia gobicola*	JN045091
Monocotyledons	Poaceae	*Kengyilia*	*Kengyilia gobicola*	HQ652849
Monocotyledons	Poaceae	*Kengyilia*	*Kengyilia gobicola*	HQ652850
Monocotyledons	Poaceae	*Kengyilia*	*Kengyilia hirsuta*	JN045095
Monocotyledons	Poaceae	*Kengyilia*	*Kengyilia hirsuta*	JN045096
Monocotyledons	Poaceae	*Kengyilia*	*Kengyilia hirsuta*	JN045097
Monocotyledons	Poaceae	*Kengyilia*	*Kengyilia hirsuta*	HQ652852

Monocotyledons	Poaceae	*Kengyilia*	*Kengyilia hirsuta*	HQ652853
Monocotyledons	Poaceae	*Kengyilia*	*Kengyilia hirsuta*	HQ652854
Monocotyledons	Poaceae	*Kengyilia*	*Kengyilia hirsuta*	HQ652855
Monocotyledons	Poaceae	*Kengyilia*	*Kengyilia longiglumis*	JN045105
Monocotyledons	Poaceae	*Kengyilia*	*Kengyilia longiglumis*	JN045106
Monocotyledons	Poaceae	*Kengyilia*	*Kengyilia longiglumis*	JN045107
Monocotyledons	Poaceae	*Kengyilia*	*Kengyilia longiglumis*	HQ652791
Monocotyledons	Poaceae	*Kengyilia*	*Kengyilia longiglumis*	HQ652860
Monocotyledons	Poaceae	*Kengyilia*	*Kengyilia longiglumis*	HQ652861
Monocotyledons	Poaceae	*Kengyilia*	*Kengyilia longiglumis*	HQ652862
Monocotyledons	Poaceae	*Kengyilia*	*Kengyilia longiglumis*	HQ652863
Monocotyledons	Poaceae	*Kengyilia*	*Kengyilia stenachyra*	JN045118
Monocotyledons	Poaceae	*Kengyilia*	*Kengyilia stenachyra*	JN045119
Monocotyledons	Poaceae	*Kengyilia*	*Kengyilia stenachyra*	JN045120
Monocotyledons	Poaceae	*Kengyilia*	*Kengyilia stenachyra*	HQ652812
Monocotyledons	Poaceae	*Kengyilia*	*Kengyilia stenachyra*	HQ652820
Monocotyledons	Poaceae	*Kengyilia*	*Kengyilia stenachyra*	HQ652828
Monocotyledons	Poaceae	*Kengyilia*	*Kengyilia tahelacana*	HQ652813
Monocotyledons	Poaceae	*Kengyilia*	*Kengyilia tahelacana*	HQ652822
Monocotyledons	Liliaceae	*Lloydia*	*Lloydia yunnanensis*	AM493964
Monocotyledons	Liliaceae	*Lloydia*	*Lloydia yunnanensis*	EU939287
Monocotyledons	Commelinaceae	*Commelina*	*Commelina imberbis*	GQ248271
Monocotyledons	Commelinaceae	*Commelina*	*Commelina imberbis*	EF590681
Monocotyledons	Heliconiaceae	*Heliconia*	*Heliconia bihai*	GQ248311
Monocotyledons	Heliconiaceae	*Heliconia*	*Heliconia bihai*	EF590700
Monocotyledons	Heliconiaceae	*Heliconia*	*Heliconia caribaea*	GQ248312
Monocotyledons	Heliconiaceae	*Heliconia*	*Heliconia caribaea*	EF590701
Monocotyledons	Dioscoreaceae	*Dioscorea*	*Dioscorea nitens*	JQ260314
Monocotyledons	Dioscoreaceae	*Dioscorea*	*Dioscorea nitens*	JQ260315
Monocotyledons	Dioscoreaceae	*Dioscorea*	*Dioscorea nitens*	JQ260316
Monocotyledons	Dioscoreaceae	*Dioscorea*	*Dioscorea subcalva*	JQ260309
Monocotyledons	Dioscoreaceae	*Dioscorea*	*Dioscorea subcalva*	JQ260310
Monocotyledons	Dioscoreaceae	*Dioscorea*	*Dioscorea subcalva*	JQ260311
Monocotyledons	Dioscoreaceae	*Dioscorea*	*Dioscorea subcalva*	JQ260312
Monocotyledons	Melanthiaceae	*Veratrum*	*Veratrum dolichopetalum*	JF807743
Monocotyledons	Melanthiaceae	*Veratrum*	*Veratrum dolichopetalum*	JF807744
Monocotyledons	Melanthiaceae	*Veratrum*	*Veratrum dolichopetalum*	JF807745
Monocotyledons	Melanthiaceae	*Veratrum*	*Veratrum dolichopetalum*	JF807746
Monocotyledons	Melanthiaceae	*Veratrum*	*Veratrum dolichopetalum*	JF807747
Monocotyledons	Melanthiaceae	*Veratrum*	*Veratrum dolichopetalum*	JF807748
Monocotyledons	Melanthiaceae	*Veratrum*	*Veratrum dolichopetalum*	JF807749
Monocotyledons	Melanthiaceae	*Veratrum*	*Veratrum dolichopetalum*	JF807750
Monocotyledons	Melanthiaceae	*Veratrum*	*Veratrum dolichopetalum*	JF807751
Monocotyledons	Melanthiaceae	*Veratrum*	*Veratrum dolichopetalum*	JF807752
Monocotyledons	Melanthiaceae	*Veratrum*	*Veratrum dolichopetalum*	JF807753
Monocotyledons	Melanthiaceae	*Veratrum*	*Veratrum dolichopetalum*	JF807754
Monocotyledons	Melanthiaceae	*Veratrum*	*Veratrum dolichopetalum*	JF807755
Monocotyledons	Melanthiaceae	*Veratrum*	*Veratrum dolichopetalum*	JF807756
Monocotyledons	Melanthiaceae	*Veratrum*	*Veratrum dolichopetalum*	JF807757
Monocotyledons	Melanthiaceae	*Veratrum*	*Veratrum dolichopetalum*	JF807758
Monocotyledons	Poaceae	*Amelichloa*	*Amelichloa brachychaeta*	EU204669
Monocotyledons	Poaceae	*Amelichloa*	*Amelichloa brachychaeta*	EU204670
Monocotyledons	Poaceae	*Amelichloa*	*Amelichloa brachychaeta*	EU204671

Monocotyledons	Poaceae	*Amelichloa*	*Amelichloa brachychaeta*	EU204672
Monocotyledons	Poaceae	*Amelichloa*	*Amelichloa brachychaeta*	EU204673
Monocotyledons	Poaceae	*Amelichloa*	*Amelichloa brachychaeta*	EU204674
Monocotyledons	Poaceae	*Amelichloa*	*Amelichloa caudata*	EU489241
Monocotyledons	Poaceae	*Amelichloa*	*Amelichloa caudata*	EU204675
Monocotyledons	Poaceae	*Amelichloa*	*Amelichloa caudata*	EU204676
Monocotyledons	Poaceae	*Amelichloa*	*Amelichloa caudata*	EU204677
Monocotyledons	Poaceae	*Amelichloa*	*Amelichloa caudata*	EU204678
Monocotyledons	Poaceae	*Amelichloa*	*Amelichloa caudata*	EU204679
Monocotyledons	Poaceae	*Amelichloa*	*Amelichloa clandestina*	EU489242
Monocotyledons	Poaceae	*Amelichloa*	*Amelichloa clandestina*	EU204680
Monocotyledons	Poaceae	*Amelichloa*	*Amelichloa clandestina*	EU204681
Monocotyledons	Poaceae	*Amelichloa*	*Amelichloa clandestina*	EU204682
Monocotyledons	Poaceae	*Amelichloa*	*Amelichloa clandestina*	EU204683
Monocotyledons	Poaceae	*Piptatherum*	*Piptatherum holciforme*	EU204755
Monocotyledons	Poaceae	*Piptatherum*	*Piptatherum holciforme*	EU204756
Monocotyledons	Musaceae	*Musa*	*Musa campestris*	FJ871907
Monocotyledons	Musaceae	*Musa*	*Musa campestris*	FJ871908
Monocotyledons	Musaceae	*Musa*	*Musa coccinea*	FJ871911
Monocotyledons	Musaceae	*Musa*	*Musa coccinea*	FJ871912
Monocotyledons	Musaceae	*Musa*	*Musa itinerans*	FJ871877
Monocotyledons	Musaceae	*Musa*	*Musa itinerans*	FJ871878
Monocotyledons	Musaceae	*Musa*	*Musa itinerans*	FJ871879
Monocotyledons	Musaceae	*Musa*	*Musa itinerans*	FJ871880
Monocotyledons	Musaceae	*Musa*	*Musa itinerans*	FJ871881
Monocotyledons	Musaceae	*Musa*	*Musa itinerans*	FJ871882
Monocotyledons	Musaceae	*Musa*	*Musa itinerans*	FJ871883
Monocotyledons	Musaceae	*Musa*	*Musa itinerans*	FJ871884
Monocotyledons	Musaceae	*Musa*	*Musa lutea*	FJ871909
Monocotyledons	Musaceae	*Musa*	*Musa lutea*	FJ871910
Monocotyledons	Musaceae	*Musa*	*Musa maclayi*	FJ871899
Monocotyledons	Musaceae	*Musa*	*Musa maclayi*	FJ871900
Monocotyledons	Musaceae	*Musa*	*Musa maclayi*	FJ871901
Monocotyledons	Musaceae	*Musa*	*Musa nagensium*	FJ871919
Monocotyledons	Musaceae	*Musa*	*Musa nagensium*	FJ871920
Monocotyledons	Musaceae	*Musa*	*Musa rubinea*	FJ871889
Monocotyledons	Musaceae	*Musa*	*Musa rubinea*	FJ871890
Monocotyledons	Musaceae	*Musa*	*Musa rubinea*	FJ871891
Monocotyledons	Musaceae	*Musa*	*Musa rubinea*	FJ871892
Monocotyledons	Musaceae	*Musa*	*Musa yunnanensis*	FJ871885
Monocotyledons	Musaceae	*Musa*	*Musa yunnanensis*	FJ871886
Monocotyledons	Musaceae	*Musa*	*Musa yunnanensis*	FJ871887
Monocotyledons	Musaceae	*Musa*	*Musa yunnanensis*	FJ871888
Monocotyledons	Orchidaceae	*Grandiphyllum*	*Grandiphyllum auriculum*	FJ564447
Monocotyledons	Orchidaceae	*Grandiphyllum*	*Grandiphyllum auriculum*	FJ564676
Monocotyledons	Orchidaceae	*Grandiphyllum*	*Grandiphyllum hians*	FJ564025
Monocotyledons	Orchidaceae	*Grandiphyllum*	*Grandiphyllum hians*	FJ564235
Monocotyledons	Musaceae	*Musa*	*Musa paracoccinea*	FJ871902
Monocotyledons	Musaceae	*Musa*	*Musa paracoccinea*	FJ871903
Monocotyledons	Musaceae	*Musa*	*Musa paracoccinea*	FJ871904
Monocotyledons	Musaceae	*Musa*	*Musa viridis*	FJ871893
Monocotyledons	Musaceae	*Musa*	*Musa viridis*	FJ871894
Monocotyledons	Musaceae	*Musa*	*Musa viridis*	FJ871895

Monocotyledons	Asparagaceae	*Asparagus*	*Asparagus aethiopicus*	GU135328
Monocotyledons	Asparagaceae	*Asparagus*	*Asparagus aethiopicus*	GU135434
Monocotyledons	Asparagaceae	*Asparagus*	*Asparagus aethiopicus*	HM990140
Monocotyledons	Cyperaceae	*Cyperus*	*Cyperus prolifer*	GU135397
Monocotyledons	Cyperaceae	*Cyperus*	*Cyperus prolifer*	HQ705804
Monocotyledons	Smilacaceae	*Smilax*	*Smilax glabra*	GU372812
Monocotyledons	Smilacaceae	*Smilax*	*Smilax glabra*	GU372815
Monocotyledons	Smilacaceae	*Smilax*	*Smilax glabra*	GU372820
Monocotyledons	Smilacaceae	*Smilax*	*Smilax glabra*	JN047204
Monocotyledons	Smilacaceae	*Smilax*	*Smilax glabra*	JN047205
Monocotyledons	Asparagaceae	*Asparagus*	*Asparagus trichophyllus*	GQ434913
Monocotyledons	Asparagaceae	*Asparagus*	*Asparagus trichophyllus*	GQ434914
Monocotyledons	Dioscoreaceae	*Dioscorea*	*Dioscorea menglaensis*	JQ260334
Monocotyledons	Dioscoreaceae	*Dioscorea*	*Dioscorea menglaensis*	JQ260335
Monocotyledons	Arecaceae	*Caryota*	*Caryota maxima*	HQ415573
Monocotyledons	Arecaceae	*Caryota*	*Caryota maxima*	JF345044
Monocotyledons	Arecaceae	*Caryota*	*Caryota maxima*	JF345045
Monocotyledons	Arecaceae	*Caryota*	*Caryota maxima*	JF345050
Monocotyledons	Arecaceae	*Caryota*	*Caryota maxima*	JF345051
Monocotyledons	Arecaceae	*Caryota*	*Caryota maxima*	JF345052
Monocotyledons	Arecaceae	*Caryota*	*Caryota maxima*	JF345053
Monocotyledons	Arecaceae	*Caryota*	*Caryota maxima*	JF345065
Monocotyledons	Poaceae	*Kengyilia*	*Kengyilia geminata*	JN045088
Monocotyledons	Poaceae	*Kengyilia*	*Kengyilia geminata*	JN045089
Monocotyledons	Poaceae	*Kengyilia*	*Kengyilia geminata*	HQ652847
Monocotyledons	Poaceae	*Kengyilia*	*Kengyilia geminata*	HQ652848
Monocotyledons	Smilacaceae	*Smilax*	*Smilax lanceifolia*	JN047206
Monocotyledons	Smilacaceae	*Smilax*	*Smilax lanceifolia*	JN047207
Monocotyledons	Commelinaceae	*Commelina*	*Commelina paludosa*	JN044311
Monocotyledons	Commelinaceae	*Commelina*	*Commelina paludosa*	JN044312
Monocotyledons	Melanthiaceae	*Paris*	*Paris caobangensis*	JN045699
Monocotyledons	Melanthiaceae	*Paris*	*Paris caobangensis*	JN045700
Monocotyledons	Melanthiaceae	*Veratrum*	*Veratrum patulum*	JF807772
Monocotyledons	Melanthiaceae	*Veratrum*	*Veratrum patulum*	JF807773
Monocotyledons	Melanthiaceae	*Veratrum*	*Veratrum patulum*	JF807774
Monocotyledons	Melanthiaceae	*Veratrum*	*Veratrum patulum*	JF807775
Monocotyledons	Melanthiaceae	*Veratrum*	*Veratrum patulum*	JF807776
Monocotyledons	Melanthiaceae	*Veratrum*	*Veratrum patulum*	JF807777
Monocotyledons	Melanthiaceae	*Veratrum*	*Veratrum patulum*	JF807778
Monocotyledons	Melanthiaceae	*Veratrum*	*Veratrum patulum*	JF807779
Monocotyledons	Melanthiaceae	*Veratrum*	*Veratrum patulum*	JF807780
Monocotyledons	Arecaceae	*Caryota*	*Caryota obtusa*	JF345046
Monocotyledons	Arecaceae	*Caryota*	*Caryota obtusa*	JF345047
Monocotyledons	Arecaceae	*Caryota*	*Caryota obtusa*	JF345061
Monocotyledons	Arecaceae	*Caryota*	*Caryota obtusa*	JF345062
Monocotyledons	Arecaceae	*Caryota*	*Caryota obtusa*	JF345063
Monocotyledons	Arecaceae	*Caryota*	*Caryota obtusa*	JF345064
Monocotyledons	Arecaceae	*Caryota*	*Caryota kiriwongensis*	JF345048
Monocotyledons	Arecaceae	*Caryota*	*Caryota kiriwongensis*	JF345049
Monocotyledons	Arecaceae	*Caryota*	*Caryota monostachya*	JF345056
Monocotyledons	Arecaceae	*Caryota*	*Caryota monostachya*	JF345057
Monocotyledons	Arecaceae	*Caryota*	*Caryota monostachya*	JF345058
Monocotyledons	Arecaceae	*Caryota*	*Caryota no*	JF345059

Monocotyledons	Arecaceae	*Caryota*	*Caryota no*	JF345060
Monocotyledons	Cyperaceae	*Cyperus*	*Cyperus buchholzii*	HQ705798
Monocotyledons	Cyperaceae	*Cyperus*	*Cyperus buchholzii*	HQ705799
Monocotyledons	Cyperaceae	*Cyperus*	*Cyperus debilissimus*	HQ705806
Monocotyledons	Cyperaceae	*Cyperus*	*Cyperus debilissimus*	HQ705807
Monocotyledons	Cyperaceae	*Cyperus*	*Cyperus debilissimus*	HQ705808
Monocotyledons	Cyperaceae	*Cyperus*	*Cyperus friburgensis*	HQ705791
Monocotyledons	Cyperaceae	*Cyperus*	*Cyperus friburgensis*	HQ705792
Monocotyledons	Cyperaceae	*Cyperus*	*Cyperus waterloti*	HQ705824
Monocotyledons	Cyperaceae	*Cyperus*	*Cyperus waterloti*	HQ705825
Monocotyledons	Pandanaceae	*Pandanus*	*Pandanus austrosinensis*	JN407016
Monocotyledons	Pandanaceae	*Pandanus*	*Pandanus austrosinensis*	JN407017
Monocotyledons	Bromeliaceae	*Hohenbergia*	*Hohenbergia ramageana*	JN204639
Monocotyledons	Bromeliaceae	*Hohenbergia*	*Hohenbergia ramageana*	JN204640
Monocotyledons	Bromeliaceae	*Hohenbergia*	*Hohenbergia ridleyi*	JN204641
Monocotyledons	Bromeliaceae	*Hohenbergia*	*Hohenbergia ridleyi*	JN204642
Monocotyledons	Bromeliaceae	*Nidularium*	*Nidularium altimontanum*	JN204646
Monocotyledons	Bromeliaceae	*Nidularium*	*Nidularium altimontanum*	JN204647
Monocotyledons	Bromeliaceae	*Nidularium*	*Nidularium angustibracteatum*	JN204648
Monocotyledons	Bromeliaceae	*Nidularium*	*Nidularium angustibracteatum*	JN204649
Monocotyledons	Bromeliaceae	*Nidularium*	*Nidularium innocentii*	JN204650
Monocotyledons	Bromeliaceae	*Nidularium*	*Nidularium innocentii*	JN204651
Monocotyledons	Bromeliaceae	*Nidularium*	*Nidularium innocentii*	JN204652
Monocotyledons	Bromeliaceae	*Nidularium*	*Nidularium krisgreeniae*	JN204653
Monocotyledons	Bromeliaceae	*Nidularium*	*Nidularium krisgreeniae*	JN204654
Monocotyledons	Bromeliaceae	*Pitcairnia*	*Pitcairnia encholirioides*	JN204657
Monocotyledons	Bromeliaceae	*Pitcairnia*	*Pitcairnia encholirioides*	JN204658
Monocotyledons	Bromeliaceae	*Pitcairnia*	*Pitcairnia flammea*	JN204659
Monocotyledons	Bromeliaceae	*Pitcairnia*	*Pitcairnia flammea*	JN204660
Monocotyledons	Bromeliaceae	*Vriesea*	*Vriesea cacuminis*	JN204669
Monocotyledons	Bromeliaceae	*Vriesea*	*Vriesea cacuminis*	JN204670
Monocotyledons	Bromeliaceae	*Vriesea*	*Vriesea erythrodactylon*	JN204675
Monocotyledons	Bromeliaceae	*Vriesea*	*Vriesea erythrodactylon*	JN204676
Monocotyledons	Bromeliaceae	*Vriesea*	*Vriesea erythrodactylon*	JN204677
Monocotyledons	Bromeliaceae	*Vriesea*	*Vriesea friburgensis*	JN204678
Monocotyledons	Bromeliaceae	*Vriesea*	*Vriesea friburgensis*	JN204679
Monocotyledons	Bromeliaceae	*Vriesea*	*Vriesea friburgensis*	JN204680
Monocotyledons	Bromeliaceae	*Vriesea*	*Vriesea friburgensis*	JN204681
Monocotyledons	Bromeliaceae	*Vriesea*	*Vriesea heterostachys*	JN204683
Monocotyledons	Bromeliaceae	*Vriesea*	*Vriesea heterostachys*	JN204684
Monocotyledons	Bromeliaceae	*Vriesea*	*Vriesea incurvata*	JN204685
Monocotyledons	Bromeliaceae	*Vriesea*	*Vriesea incurvata*	JN204686
Monocotyledons	Bromeliaceae	*Vriesea*	*Vriesea longicaulis*	JN204687
Monocotyledons	Bromeliaceae	*Vriesea*	*Vriesea longicaulis*	JN204688
Monocotyledons	Bromeliaceae	*Vriesea*	*Vriesea platynema*	JN204673
Monocotyledons	Bromeliaceae	*Vriesea*	*Vriesea platynema*	JN204689
Monocotyledons	Bromeliaceae	*Vriesea*	*Vriesea platynema*	JN204690
Monocotyledons	Bromeliaceae	*Vriesea*	*Vriesea procera*	JN204691
Monocotyledons	Bromeliaceae	*Vriesea*	*Vriesea procera*	JN204692
Monocotyledons	Bromeliaceae	*Vriesea*	*Vriesea procera*	JN204693
Monocotyledons	Hypoxidaceae	*Pauridia*	*Pauridia longituba*	FM206348
Monocotyledons	Hypoxidaceae	*Pauridia*	*Pauridia longituba*	FM206349
Monocotyledons	Hypoxidaceae	*Pauridia*	*Pauridia longituba*	FM206350

Monocotyledons	Hypoxidaceae	*Pauridia*	*Pauridia longituba*	FM206351
Monocotyledons	Hypoxidaceae	*Pauridia*	*Pauridia longituba*	FM206352
Monocotyledons	Hypoxidaceae	*Pauridia*	*Pauridia longituba*	FM206353
Monocotyledons	Hypoxidaceae	*Pauridia*	*Pauridia longituba*	FM206354
Monocotyledons	Hypoxidaceae	*Pauridia*	*Pauridia longituba*	FM206355
Monocotyledons	Hypoxidaceae	*Pauridia*	*Pauridia longituba*	FM206356
Monocotyledons	Hypoxidaceae	*Pauridia*	*Pauridia longituba*	FM206357
Monocotyledons	Hypoxidaceae	*Pauridia*	*Pauridia longituba*	FM206358
Monocotyledons	Hypoxidaceae	*Pauridia*	*Pauridia longituba*	FM206359
Monocotyledons	Hypoxidaceae	*Pauridia*	*Pauridia longituba*	FM206360
Monocotyledons	Hypoxidaceae	*Pauridia*	*Pauridia longituba*	FM206361
Monocotyledons	Hypoxidaceae	*Pauridia*	*Pauridia longituba*	FM206362
Monocotyledons	Hypoxidaceae	*Pauridia*	*Pauridia longituba*	FM206363
Monocotyledons	Hypoxidaceae	*Pauridia*	*Pauridia longituba*	FM206364
Monocotyledons	Hypoxidaceae	*Pauridia*	*Pauridia longituba*	FM206365
Monocotyledons	Hypoxidaceae	*Pauridia*	*Pauridia longituba*	FM206366
Monocotyledons	Hypoxidaceae	*Pauridia*	*Pauridia longituba*	FM206367
Monocotyledons	Hypoxidaceae	*Pauridia*	*Pauridia longituba*	FM206368
Monocotyledons	Hypoxidaceae	*Pauridia*	*Pauridia longituba*	FM206369
Monocotyledons	Hypoxidaceae	*Pauridia*	*Pauridia longituba*	FM206370
Monocotyledons	Hypoxidaceae	*Pauridia*	*Pauridia longituba*	FM206371
Monocotyledons	Hypoxidaceae	*Pauridia*	*Pauridia longituba*	FM206372
Monocotyledons	Hypoxidaceae	*Pauridia*	*Pauridia longituba*	FM206373
Monocotyledons	Hypoxidaceae	*Pauridia*	*Pauridia longituba*	FM206374
Monocotyledons	Hypoxidaceae	*Pauridia*	*Pauridia longituba*	FM206375
Monocotyledons	Hypoxidaceae	*Pauridia*	*Pauridia longituba*	FM206376
Monocotyledons	Hypoxidaceae	*Pauridia*	*Pauridia longituba*	FM206377
Monocotyledons	Hypoxidaceae	*Pauridia*	*Pauridia longituba*	FM206378
Monocotyledons	Hypoxidaceae	*Pauridia*	*Pauridia longituba*	FM206379
Monocotyledons	Hypoxidaceae	*Pauridia*	*Pauridia longituba*	FM206380
Monocotyledons	Hyacinthaceae	*Hyacinthoides*	*Hyacinthoides aristidis*	FJ423302
Monocotyledons	Hyacinthaceae	*Hyacinthoides*	*Hyacinthoides aristidis*	FJ423303
Monocotyledons	Hyacinthaceae	*Hyacinthoides*	*Hyacinthoides hispanica*	FJ423321
Monocotyledons	Hyacinthaceae	*Hyacinthoides*	*Hyacinthoides hispanica*	FJ423322
Monocotyledons	Hyacinthaceae	*Hyacinthoides*	*Hyacinthoides hispanica*	FJ423323
Monocotyledons	Hyacinthaceae	*Hyacinthoides*	*Hyacinthoides hispanica*	FJ423324
Monocotyledons	Hyacinthaceae	*Hyacinthoides*	*Hyacinthoides hispanica*	FJ423325
Monocotyledons	Hyacinthaceae	*Hyacinthoides*	*Hyacinthoides hispanica*	FJ423326
Monocotyledons	Hyacinthaceae	*Hyacinthoides*	*Hyacinthoides hispanica*	FJ423327
Monocotyledons	Hyacinthaceae	*Hyacinthoides*	*Hyacinthoides hispanica*	FJ423328
Monocotyledons	Hyacinthaceae	*Hyacinthoides*	*Hyacinthoides hispanica*	FJ423329
Monocotyledons	Hyacinthaceae	*Hyacinthoides*	*Hyacinthoides hispanica*	FJ423330
Monocotyledons	Hyacinthaceae	*Hyacinthoides*	*Hyacinthoides hispanica*	FJ423331
Monocotyledons	Hyacinthaceae	*Hyacinthoides*	*Hyacinthoides hispanica*	FJ423332
Monocotyledons	Hyacinthaceae	*Hyacinthoides*	*Hyacinthoides hispanica*	FJ423333
Monocotyledons	Hyacinthaceae	*Hyacinthoides*	*Hyacinthoides hispanica*	FJ423334
Monocotyledons	Hyacinthaceae	*Hyacinthoides*	*Hyacinthoides hispanica*	FJ423335
Monocotyledons	Hyacinthaceae	*Hyacinthoides*	*Hyacinthoides hispanica*	FJ423336
Monocotyledons	Hyacinthaceae	*Hyacinthoides*	*Hyacinthoides hispanica*	FJ423337
Monocotyledons	Hyacinthaceae	*Hyacinthoides*	*Hyacinthoides hispanica*	FJ423338
Monocotyledons	Hyacinthaceae	*Hyacinthoides*	*Hyacinthoides hispanica*	FJ423340
Monocotyledons	Hyacinthaceae	*Hyacinthoides*	*Hyacinthoides hispanica*	FJ423341
Monocotyledons	Hyacinthaceae	*Hyacinthoides*	*Hyacinthoides hispanica*	FJ423348

Monocotyledons	Hyacinthaceae	*Hyacinthoides*	*Hyacinthoides hispanica*	FJ423349
Monocotyledons	Hyacinthaceae	*Hyacinthoides*	*Hyacinthoides hispanica*	FJ423350
Monocotyledons	Hyacinthaceae	*Hyacinthoides*	*Hyacinthoides hispanica*	FJ423351
Monocotyledons	Hyacinthaceae	*Hyacinthoides*	*Hyacinthoides hispanica*	FJ423352
Monocotyledons	Hyacinthaceae	*Hyacinthoides*	*Hyacinthoides italica*	FJ423289
Monocotyledons	Hyacinthaceae	*Hyacinthoides*	*Hyacinthoides italica*	FJ423290
Monocotyledons	Hyacinthaceae	*Hyacinthoides*	*Hyacinthoides italica*	FJ423291
Monocotyledons	Hyacinthaceae	*Hyacinthoides*	*Hyacinthoides italica*	FJ423292
Monocotyledons	Hyacinthaceae	*Hyacinthoides*	*Hyacinthoides italica*	FJ423293
Monocotyledons	Hyacinthaceae	*Hyacinthoides*	*Hyacinthoides lingulata*	FJ423294
Monocotyledons	Hyacinthaceae	*Hyacinthoides*	*Hyacinthoides lingulata*	FJ423295
Monocotyledons	Hyacinthaceae	*Hyacinthoides*	*Hyacinthoides lingulata*	FJ423296
Monocotyledons	Hyacinthaceae	*Hyacinthoides*	*Hyacinthoides lingulata*	FJ423297
Monocotyledons	Hyacinthaceae	*Hyacinthoides*	*Hyacinthoides lingulata*	FJ423298
Monocotyledons	Hyacinthaceae	*Hyacinthoides*	*Hyacinthoides non-scripta*	FJ423316
Monocotyledons	Hyacinthaceae	*Hyacinthoides*	*Hyacinthoides non-scripta*	FJ423317
Monocotyledons	Hyacinthaceae	*Hyacinthoides*	*Hyacinthoides non-scripta*	FJ423318
Monocotyledons	Hyacinthaceae	*Hyacinthoides*	*Hyacinthoides non-scripta*	FJ423319
Monocotyledons	Hyacinthaceae	*Hyacinthoides*	*Hyacinthoides non-scripta*	FJ423320
Monocotyledons	Hyacinthaceae	*Hyacinthoides*	*Hyacinthoides reverchonii*	FJ423304
Monocotyledons	Hyacinthaceae	*Hyacinthoides*	*Hyacinthoides reverchonii*	FJ423305
Monocotyledons	Hyacinthaceae	*Hyacinthoides*	*Hyacinthoides reverchonii*	FJ423306
Monocotyledons	Hyacinthaceae	*Hyacinthoides*	*Hyacinthoides reverchonii*	FJ423307
Monocotyledons	Hyacinthaceae	*Hyacinthoides*	*Hyacinthoides reverchonii*	FJ423308
Monocotyledons	Liliaceae	*Gagea*	*Gagea lutea*	AM409354
Monocotyledons	Liliaceae	*Gagea*	*Gagea lutea*	EU939250
Monocotyledons	Zingiberaceae	*Alpinia*	*Alpinia galanga*	GQ435039
Monocotyledons	Zingiberaceae	*Alpinia*	*Alpinia galanga*	GQ435040
Monocotyledons	Zingiberaceae	*Alpinia*	*Alpinia galanga*	GQ435041
Monocotyledons	Zingiberaceae	*Alpinia*	*Alpinia galanga*	EU552522
Monocotyledons	Zingiberaceae	*Alpinia*	*Alpinia galanga*	EU552528
Monocotyledons	Zingiberaceae	*Alpinia*	*Alpinia galanga*	GU180424
Monocotyledons	Zingiberaceae	*Alpinia*	*Alpinia galanga*	JN043838
Monocotyledons	Zingiberaceae	*Alpinia*	*Alpinia galanga*	JN043839
Monocotyledons	Zingiberaceae	*Alpinia*	*Alpinia galanga*	JN043840
Monocotyledons	Zingiberaceae	*Alpinia*	*Alpinia galanga*	JN043841
Monocotyledons	Zingiberaceae	*Alpinia*	*Alpinia galanga*	JN043842
Monocotyledons	Zingiberaceae	*Alpinia*	*Alpinia galanga*	JN043843
Monocotyledons	Zingiberaceae	*Alpinia*	*Alpinia zerumbet*	GQ435051
Monocotyledons	Zingiberaceae	*Alpinia*	*Alpinia zerumbet*	GU180443
Monocotyledons	Zingiberaceae	*Alpinia*	*Alpinia zerumbet*	GU180444
Monocotyledons	Zingiberaceae	*Alpinia*	*Alpinia zerumbet*	GU180445
Monocotyledons	Zingiberaceae	*Alpinia*	*Alpinia zerumbet*	JN043874
Monocotyledons	Zingiberaceae	*Alpinia*	*Alpinia zerumbet*	JN043875
Monocotyledons	Zingiberaceae	*Alpinia*	*Alpinia zerumbet*	JN043876
Monocotyledons	Zingiberaceae	*Alpinia*	*Alpinia zerumbet*	JN043877
Monocotyledons	Zingiberaceae	*Alpinia*	*Alpinia zerumbet*	JN043878
Monocotyledons	Zingiberaceae	*Boesenbergia*	*Boesenbergia plicata*	DQ408316
Monocotyledons	Zingiberaceae	*Boesenbergia*	*Boesenbergia plicata*	DQ408317
Monocotyledons	Zingiberaceae	*Boesenbergia*	*Boesenbergia rotunda*	DQ408325
Monocotyledons	Zingiberaceae	*Boesenbergia*	*Boesenbergia rotunda*	DQ408326
Monocotyledons	Zingiberaceae	*Boesenbergia*	*Boesenbergia rotunda*	DQ408327
Monocotyledons	Zingiberaceae	*Kaempferia*	*Kaempferia elegans*	GQ386005

Monocotyledons	Zingiberaceae	*Kaempferia*	*Kaempferia elegans*	GQ386006
Monocotyledons	Zingiberaceae	*Kaempferia*	*Kaempferia elegans*	GQ386007
Monocotyledons	Zingiberaceae	*Kaempferia*	*Kaempferia elegans*	GQ386008
Monocotyledons	Zingiberaceae	*Kaempferia*	*Kaempferia elegans*	GQ386009
Monocotyledons	Zingiberaceae	*Kaempferia*	*Kaempferia elegans*	GQ386010
Monocotyledons	Zingiberaceae	*Kaempferia*	*Kaempferia elegans*	GQ386011
Monocotyledons	Zingiberaceae	*Kaempferia*	*Kaempferia elegans*	GU180452
Monocotyledons	Zingiberaceae	*Kaempferia*	*Kaempferia elegans*	GU180453
Monocotyledons	Zingiberaceae	*Kaempferia*	*Kaempferia galanga*	GQ385977
Monocotyledons	Zingiberaceae	*Kaempferia*	*Kaempferia galanga*	GQ385979
Monocotyledons	Zingiberaceae	*Kaempferia*	*Kaempferia galanga*	GQ385980
Monocotyledons	Zingiberaceae	*Kaempferia*	*Kaempferia galanga*	GQ435048
Monocotyledons	Zingiberaceae	*Kaempferia*	*Kaempferia galanga*	GQ435049
Monocotyledons	Zingiberaceae	*Kaempferia*	*Kaempferia galanga*	EU552523
Monocotyledons	Zingiberaceae	*Kaempferia*	*Kaempferia galanga*	GU180437
Monocotyledons	Zingiberaceae	*Kaempferia*	*Kaempferia galanga*	GU180438
Monocotyledons	Zingiberaceae	*Kaempferia*	*Kaempferia galanga*	GU180439
Monocotyledons	Zingiberaceae	*Kaempferia*	*Kaempferia parviflora*	DQ408334
Monocotyledons	Zingiberaceae	*Kaempferia*	*Kaempferia parviflora*	GQ386012
Monocotyledons	Zingiberaceae	*Kaempferia*	*Kaempferia parviflora*	GQ386013
Monocotyledons	Zingiberaceae	*Kaempferia*	*Kaempferia parviflora*	GQ386014
Monocotyledons	Zingiberaceae	*Kaempferia*	*Kaempferia parviflora*	GQ386015
Monocotyledons	Zingiberaceae	*Kaempferia*	*Kaempferia roscoeana*	GQ386029
Monocotyledons	Zingiberaceae	*Kaempferia*	*Kaempferia roscoeana*	GQ386030
Monocotyledons	Zingiberaceae	*Kaempferia*	*Kaempferia rotunda*	GQ386031
Monocotyledons	Zingiberaceae	*Kaempferia*	*Kaempferia rotunda*	GQ386032
Monocotyledons	Zingiberaceae	*Kaempferia*	*Kaempferia rotunda*	GQ386033
Monocotyledons	Zingiberaceae	*Kaempferia*	*Kaempferia rotunda*	GQ386034
Monocotyledons	Zingiberaceae	*Kaempferia*	*Kaempferia rotunda*	GQ386035
Monocotyledons	Zingiberaceae	*Kaempferia*	*Kaempferia rotunda*	GQ386036
Monocotyledons	Zingiberaceae	*Kaempferia*	*Kaempferia rotunda*	GQ386037
Monocotyledons	Zingiberaceae	*Kaempferia*	*Kaempferia rotunda*	GU180430
Monocotyledons	Zingiberaceae	*Alpinia*	*Alpinia blepharocalyx*	JN043817
Monocotyledons	Zingiberaceae	*Alpinia*	*Alpinia blepharocalyx*	JN043818
Monocotyledons	Zingiberaceae	*Alpinia*	*Alpinia blepharocalyx*	JN043819
Monocotyledons	Zingiberaceae	*Alpinia*	*Alpinia conchigera*	JN043826
Monocotyledons	Zingiberaceae	*Alpinia*	*Alpinia conchigera*	JN043827
Monocotyledons	Zingiberaceae	*Alpinia*	*Alpinia conchigera*	JN043828
Monocotyledons	Zingiberaceae	*Alpinia*	*Alpinia intermedia*	JN043850
Monocotyledons	Zingiberaceae	*Alpinia*	*Alpinia intermedia*	JN043851
Monocotyledons	Zingiberaceae	*Alpinia*	*Alpinia intermedia*	JN043852
Monocotyledons	Zingiberaceae	*Alpinia*	*Alpinia intermedia*	JN043853
Monocotyledons	Zingiberaceae	*Alpinia*	*Alpinia nigra*	JN043858
Monocotyledons	Zingiberaceae	*Alpinia*	*Alpinia nigra*	JN043859
Monocotyledons	Zingiberaceae	*Alpinia*	*Alpinia polyantha*	GU180425
Monocotyledons	Zingiberaceae	*Alpinia*	*Alpinia polyantha*	JN043871
Monocotyledons	Zingiberaceae	*Alpinia*	*Alpinia polyantha*	JN043872
Monocotyledons	Zingiberaceae	*Alpinia*	*Alpinia polyantha*	JN043873
Monocotyledons	Amaryllidaceae	*Lycoris*	*Lycoris squamigera*	GQ923941
Monocotyledons	Amaryllidaceae	*Lycoris*	*Lycoris squamigera*	HM748829
Monocotyledons	Zingiberaceae	*Alpinia*	*Alpinia aquatica*	JN043815
Monocotyledons	Zingiberaceae	*Alpinia*	*Alpinia aquatica*	JN043816
Monocotyledons	Zingiberaceae	*Alpinia*	*Alpinia formosana*	JN043833

Monocotyledons	Zingiberaceae	*Alpinia*	*Alpinia formosana*	JN043834
Monocotyledons	Zingiberaceae	*Alpinia*	*Alpinia guinanensis*	JN043846
Monocotyledons	Zingiberaceae	*Alpinia*	*Alpinia guinanensis*	JN043847
Monocotyledons	Zingiberaceae	*Alpinia*	*Alpinia hainanensis*	GU180421
Monocotyledons	Zingiberaceae	*Alpinia*	*Alpinia hainanensis*	JN043848
Monocotyledons	Zingiberaceae	*Alpinia*	*Alpinia hainanensis*	JN043849
Monocotyledons	Zingiberaceae	*Alpinia*	*Alpinia oxyphylla*	EU552526
Monocotyledons	Zingiberaceae	*Alpinia*	*Alpinia oxyphylla*	GU180446
Monocotyledons	Zingiberaceae	*Alpinia*	*Alpinia oxyphylla*	GU180447
Monocotyledons	Zingiberaceae	*Alpinia*	*Alpinia oxyphylla*	JN043863
Monocotyledons	Zingiberaceae	*Alpinia*	*Alpinia oxyphylla*	JN043864
Monocotyledons	Zingiberaceae	*Alpinia*	*Alpinia oxyphylla*	JN043865
Monocotyledons	Zingiberaceae	*Alpinia*	*Alpinia oxyphylla*	JN043866
Monocotyledons	Zingiberaceae	*Curcuma*	*Curcuma aromatica*	GQ435050
Monocotyledons	Zingiberaceae	*Curcuma*	*Curcuma aromatica*	EU552527
Monocotyledons	Zingiberaceae	*Curcuma*	*Curcuma aromatica*	GU180440
Monocotyledons	Zingiberaceae	*Curcuma*	*Curcuma aromatica*	GU180441
Monocotyledons	Zingiberaceae	*Curcuma*	*Curcuma aromatica*	GU180442
Monocotyledons	Zingiberaceae	*Curcuma*	*Curcuma aromatica*	GU180448
Monocotyledons	Zingiberaceae	*Curcuma*	*Curcuma aromatica*	GU180449
Monocotyledons	Zingiberaceae	*Curcuma*	*Curcuma aromatica*	GU180450
Monocotyledons	Zingiberaceae	*Curcuma*	*Curcuma aromatica*	GU180451
Monocotyledons	Zingiberaceae	*Curcuma*	*Curcuma kwangsiensis*	GQ435042
Monocotyledons	Zingiberaceae	*Curcuma*	*Curcuma kwangsiensis*	GQ435043
Monocotyledons	Zingiberaceae	*Curcuma*	*Curcuma kwangsiensis*	EU552520
Monocotyledons	Zingiberaceae	*Curcuma*	*Curcuma kwangsiensis*	GU180429
Monocotyledons	Zingiberaceae	*Curcuma*	*Curcuma kwangsiensis*	JF730251
Monocotyledons	Zingiberaceae	*Curcuma*	*Curcuma longa*	FJ687416
Monocotyledons	Zingiberaceae	*Curcuma*	*Curcuma longa*	GU180434
Monocotyledons	Zingiberaceae	*Curcuma*	*Curcuma longa*	GU180435
Monocotyledons	Zingiberaceae	*Curcuma*	*Curcuma longa*	GU180436
Monocotyledons	Zingiberaceae	*Curcuma*	*Curcuma longa*	JF730221
Monocotyledons	Zingiberaceae	*Curcuma*	*Curcuma longa*	JF730222
Monocotyledons	Zingiberaceae	*Curcuma*	*Curcuma longa*	JF730223
Monocotyledons	Zingiberaceae	*Curcuma*	*Curcuma longa*	JF730224
Monocotyledons	Zingiberaceae	*Curcuma*	*Curcuma longa*	JF730225
Monocotyledons	Zingiberaceae	*Curcuma*	*Curcuma longa*	JF730226
Monocotyledons	Zingiberaceae	*Curcuma*	*Curcuma longa*	JF730227
Monocotyledons	Zingiberaceae	*Curcuma*	*Curcuma longa*	JF730228
Monocotyledons	Zingiberaceae	*Curcuma*	*Curcuma longa*	JF730229
Monocotyledons	Zingiberaceae	*Curcuma*	*Curcuma longa*	JF730230
Monocotyledons	Zingiberaceae	*Curcuma*	*Curcuma longa*	JF730231
Monocotyledons	Zingiberaceae	*Curcuma*	*Curcuma longa*	JF730232
Monocotyledons	Zingiberaceae	*Curcuma*	*Curcuma longa*	JF730233
Monocotyledons	Zingiberaceae	*Curcuma*	*Curcuma longa*	JF730234
Monocotyledons	Zingiberaceae	*Curcuma*	*Curcuma longa*	JF730235
Monocotyledons	Zingiberaceae	*Curcuma*	*Curcuma longa*	JF730236
Monocotyledons	Zingiberaceae	*Curcuma*	*Curcuma longa*	JF730237
Monocotyledons	Zingiberaceae	*Curcuma*	*Curcuma longa*	JF730238
Monocotyledons	Zingiberaceae	*Curcuma*	*Curcuma longa*	JF730239
Monocotyledons	Zingiberaceae	*Curcuma*	*Curcuma longa*	JF730253
Monocotyledons	Zingiberaceae	*Curcuma*	*Curcuma longa*	JF730254
Monocotyledons	Zingiberaceae	*Curcuma*	*Curcuma longa*	JF730255

Monocotyledons	Zingiberaceae	*Curcuma*	*Curcuma phaeocaulis*	EU552524
Monocotyledons	Zingiberaceae	*Curcuma*	*Curcuma phaeocaulis*	JF730247
Monocotyledons	Zingiberaceae	*Curcuma*	*Curcuma phaeocaulis*	JF730248
Monocotyledons	Zingiberaceae	*Curcuma*	*Curcuma phaeocaulis*	JF730249
Monocotyledons	Zingiberaceae	*Curcuma*	*Curcuma phaeocaulis*	JF730256
Monocotyledons	Zingiberaceae	*Curcuma*	*Curcuma phaeocaulis*	JF730257
Monocotyledons	Zingiberaceae	*Curcuma*	*Curcuma sichuanensis*	JF730240
Monocotyledons	Zingiberaceae	*Curcuma*	*Curcuma sichuanensis*	JF730241
Monocotyledons	Zingiberaceae	*Curcuma*	*Curcuma sichuanensis*	JF730242
Monocotyledons	Zingiberaceae	*Curcuma*	*Curcuma sichuanensis*	JF730243
Monocotyledons	Zingiberaceae	*Curcuma*	*Curcuma sichuanensis*	JF730244
Monocotyledons	Zingiberaceae	*Curcuma*	*Curcuma sichuanensis*	JF730245
Monocotyledons	Zingiberaceae	*Curcuma*	*Curcuma sichuanensis*	JF730246
Monocotyledons	Zingiberaceae	*Curcuma*	*Curcuma wenyujin*	EU552525
Monocotyledons	Zingiberaceae	*Curcuma*	*Curcuma wenyujin*	JF730252
Monocotyledons	Zingiberaceae	*Curcuma*	*Curcuma zedoaria*	FJ687417
Monocotyledons	Zingiberaceae	*Curcuma*	*Curcuma zedoaria*	GU180426
Monocotyledons	Amaryllidaceae	*Lycoris*	*Lycoris aurea*	GQ923938
Monocotyledons	Amaryllidaceae	*Lycoris*	*Lycoris aurea*	HM748822
Monocotyledons	Stemonaceae	*Stemona*	*Stemona tuberosa*	GQ434873
Monocotyledons	Stemonaceae	*Stemona*	*Stemona tuberosa*	GQ434874
Monocotyledons	Stemonaceae	*Stemona*	*Stemona tuberosa*	AB373199
Monocotyledons	Liliaceae	*Gagea*	*Gagea bohemica*	AM932484
Monocotyledons	Liliaceae	*Gagea*	*Gagea bohemica*	AM265595
Monocotyledons	Liliaceae	*Gagea*	*Gagea bohemica*	AM282987
Monocotyledons	Liliaceae	*Gagea*	*Gagea villosa*	AM282991
Monocotyledons	Liliaceae	*Gagea*	*Gagea villosa*	AM282992
Monocotyledons	Liliaceae	*Gagea*	*Gagea villosa*	EU939276
Monocotyledons	Liliaceae	*Gagea*	*Gagea villosa*	EU939277
Monocotyledons	Liliaceae	*Gagea*	*Gagea villosa*	EU939279
Monocotyledons	Liliaceae	*Gagea*	*Gagea villosa*	FN868213
Monocotyledons	Liliaceae	*Gagea*	*Gagea villosa*	FN868214
Monocotyledons	Liliaceae	*Gagea*	*Gagea villosa*	FN868215
Monocotyledons	Liliaceae	*Gagea*	*Gagea villosa*	FN868216
Monocotyledons	Liliaceae	*Gagea*	*Gagea villosa*	FN868217
Monocotyledons	Liliaceae	*Gagea*	*Gagea villosa*	FN868218
Monocotyledons	Liliaceae	*Gagea*	*Gagea villosa*	FN868219
Monocotyledons	Liliaceae	*Gagea*	*Gagea villosa*	FN868220
Monocotyledons	Liliaceae	*Gagea*	*Gagea minima*	AM238519
Monocotyledons	Liliaceae	*Gagea*	*Gagea minima*	AM238524
Monocotyledons	Liliaceae	*Gagea*	*Gagea minima*	AM282996
Monocotyledons	Zingiberaceae	*Alpinia*	*Alpinia calcarata*	JN043820
Monocotyledons	Zingiberaceae	*Alpinia*	*Alpinia calcarata*	JN043821
Monocotyledons	Zingiberaceae	*Alpinia*	*Alpinia calcarata*	JN043822
Monocotyledons	Zingiberaceae	*Alpinia*	*Alpinia calcarata*	JN043823
Monocotyledons	Zingiberaceae	*Alpinia*	*Alpinia carolinensis*	JN043824
Monocotyledons	Zingiberaceae	*Alpinia*	*Alpinia carolinensis*	JN043825
Monocotyledons	Zingiberaceae	*Alpinia*	*Alpinia elegans*	JN043829
Monocotyledons	Zingiberaceae	*Alpinia*	*Alpinia elegans*	JN043830
Monocotyledons	Zingiberaceae	*Alpinia*	*Alpinia foxworthyi*	JN043835
Monocotyledons	Zingiberaceae	*Alpinia*	*Alpinia foxworthyi*	JN043836
Monocotyledons	Zingiberaceae	*Alpinia*	*Alpinia foxworthyi*	JN043837
Monocotyledons	Zingiberaceae	*Alpinia*	*Alpinia officinarum*	EU552529

Monocotyledons	Zingiberaceae	*Alpinia*	*Alpinia officinarum*	GU180427
Monocotyledons	Zingiberaceae	*Alpinia*	*Alpinia officinarum*	GU180428
Monocotyledons	Zingiberaceae	*Kaempferia*	*Kaempferia pulchra*	GQ386025
Monocotyledons	Zingiberaceae	*Kaempferia*	*Kaempferia pulchra*	GQ386026
Monocotyledons	Zingiberaceae	*Kaempferia*	*Kaempferia pulchra*	GQ386027
Monocotyledons	Zingiberaceae	*Kaempferia*	*Kaempferia pulchra*	GQ386028
Monocotyledons	Amaryllidaceae	*Lycoris*	*Lycoris albiflora*	GQ923943
Monocotyledons	Amaryllidaceae	*Lycoris*	*Lycoris albiflora*	HM748820
Monocotyledons	Zingiberaceae	*Alpinia*	*Alpinia jianganfeng*	JN043854
Monocotyledons	Zingiberaceae	*Alpinia*	*Alpinia jianganfeng*	JN043855
Monocotyledons	Amaryllidaceae	*Lycoris*	*Lycoris radiata*	GQ923942
Monocotyledons	Amaryllidaceae	*Lycoris*	*Lycoris radiata*	HM748828
Monocotyledons	Zingiberaceae	*Kaempferia*	*Kaempferia angustifolia*	GQ386041
Monocotyledons	Zingiberaceae	*Kaempferia*	*Kaempferia angustifolia*	GQ386042
Monocotyledons	Zingiberaceae	*Boesenbergia*	*Boesenbergia longiflora*	DQ408311
Monocotyledons	Zingiberaceae	*Boesenbergia*	*Boesenbergia longiflora*	DQ408312
Monocotyledons	Amaryllidaceae	*Lycoris*	*Lycoris chinensis*	GQ923949
Monocotyledons	Amaryllidaceae	*Lycoris*	*Lycoris chinensis*	HM748827
Monocotyledons	Amaryllidaceae	*Lycoris*	*Lycoris longituba*	GQ923944
Monocotyledons	Amaryllidaceae	*Lycoris*	*Lycoris longituba*	HM748817
Monocotyledons	Liliaceae	*Gagea*	*Gagea graeca*	AM779097
Monocotyledons	Liliaceae	*Gagea*	*Gagea graeca*	AM939647
Monocotyledons	Liliaceae	*Gagea*	*Gagea graeca*	AM939648
Monocotyledons	Liliaceae	*Gagea*	*Gagea graeca*	EU939285
Monocotyledons	Zingiberaceae	*Alpinia*	*Alpinia kwangsiensis*	GU180422
Monocotyledons	Zingiberaceae	*Alpinia*	*Alpinia kwangsiensis*	JN043856
Monocotyledons	Zingiberaceae	*Alpinia*	*Alpinia kwangsiensis*	JN043857
Monocotyledons	Amaryllidaceae	*Lycoris*	*Lycoris anhuiensis*	GQ923948
Monocotyledons	Amaryllidaceae	*Lycoris*	*Lycoris anhuiensis*	HM748823
Monocotyledons	Amaryllidaceae	*Lycoris*	*Lycoris sprengeri*	GQ923945
Monocotyledons	Amaryllidaceae	*Lycoris*	*Lycoris sprengeri*	HM748815
Monocotyledons	Amaryllidaceae	*Lycoris*	*Lycoris rosea*	GQ923947
Monocotyledons	Amaryllidaceae	*Lycoris*	*Lycoris rosea*	HM748825
Monocotyledons	Amaryllidaceae	*Lycoris*	*Lycoris haywardii*	GQ923950
Monocotyledons	Amaryllidaceae	*Lycoris*	*Lycoris haywardii*	HM748816
Monocotyledons	Amaryllidaceae	*Lycoris*	*Lycoris incarnata*	GQ923939
Monocotyledons	Amaryllidaceae	*Lycoris*	*Lycoris incarnata*	HM748830
Monocotyledons	Amaryllidaceae	*Lycoris*	*Lycoris straminea*	GQ923946
Monocotyledons	Amaryllidaceae	*Lycoris*	*Lycoris straminea*	HM748826
Monocotyledons	Liliaceae	*Gagea*	*Gagea liotardii*	AM238521
Monocotyledons	Liliaceae	*Gagea*	*Gagea liotardii*	AM238522
Monocotyledons	Liliaceae	*Gagea*	*Gagea liotardii*	AM238531
Monocotyledons	Liliaceae	*Gagea*	*Gagea confusa*	EU939238
Monocotyledons	Liliaceae	*Gagea*	*Gagea confusa*	EU939239
Monocotyledons	Liliaceae	*Gagea*	*Gagea reticulata*	AM238528
Monocotyledons	Liliaceae	*Gagea*	*Gagea reticulata*	AM287270
Monocotyledons	Liliaceae	*Gagea*	*Gagea reticulata*	EU939254
Monocotyledons	Liliaceae	*Gagea*	*Gagea reticulata*	EU939256
Monocotyledons	Liliaceae	*Gagea*	*Gagea reticulata*	EU939257
Monocotyledons	Liliaceae	*Gagea*	*Gagea reticulata*	EU939258
Monocotyledons	Liliaceae	*Gagea*	*Gagea reticulata*	EU939259
Monocotyledons	Liliaceae	*Gagea*	*Gagea reticulata*	EU939260
Monocotyledons	Liliaceae	*Gagea*	*Gagea afghanica*	EU939221

Monocotyledons	Liliaceae	*Gagea*	*Gagea afghanica*	EU939222
Monocotyledons	Liliaceae	*Gagea*	*Gagea afghanica*	EU939223
Monocotyledons	Liliaceae	*Gagea*	*Gagea bulbifera*	FR690829
Monocotyledons	Liliaceae	*Gagea*	*Gagea bulbifera*	FR690830
Monocotyledons	Liliaceae	*Gagea*	*Gagea bulbifera*	EU939226
Monocotyledons	Liliaceae	*Gagea*	*Gagea bulbifera*	EU939227
Monocotyledons	Liliaceae	*Gagea*	*Gagea foliosa*	AM265596
Monocotyledons	Liliaceae	*Gagea*	*Gagea foliosa*	AM282993
Monocotyledons	Liliaceae	*Gagea*	*Gagea dschungarica*	EU939240
Monocotyledons	Liliaceae	*Gagea*	*Gagea dschungarica*	EU939241
Monocotyledons	Liliaceae	*Gagea*	*Gagea chomutovae*	EU939234
Monocotyledons	Liliaceae	*Gagea*	*Gagea chomutovae*	EU939235
Monocotyledons	Zingiberaceae	*Boesenbergia*	*Boesenbergia curtisii*	DQ408309
Monocotyledons	Zingiberaceae	*Boesenbergia*	*Boesenbergia curtisii*	DQ408310
Monocotyledons	Zingiberaceae	*Boesenbergia*	*Boesenbergia longipes*	DQ408313
Monocotyledons	Zingiberaceae	*Boesenbergia*	*Boesenbergia longipes*	DQ408314
Monocotyledons	Zingiberaceae	*Kaempferia*	*Kaempferia candida*	GQ386003
Monocotyledons	Zingiberaceae	*Kaempferia*	*Kaempferia candida*	GQ386004
Monocotyledons	Zingiberaceae	*Kaempferia*	*Kaempferia marginata*	GQ385976
Monocotyledons	Zingiberaceae	*Kaempferia*	*Kaempferia marginata*	GQ385981
Monocotyledons	Zingiberaceae	*Kaempferia*	*Kaempferia marginata*	GQ385982
Monocotyledons	Zingiberaceae	*Kaempferia*	*Kaempferia marginata*	GQ385983
Monocotyledons	Zingiberaceae	*Kaempferia*	*Kaempferia marginata*	GQ385984
Monocotyledons	Liliaceae	*Gagea*	*Gagea gageoides*	EU939246
Monocotyledons	Liliaceae	*Gagea*	*Gagea gageoides*	EU939247
Monocotyledons	Liliaceae	*Gagea*	*Gagea alexeenkoana*	EU939224
Monocotyledons	Liliaceae	*Gagea*	*Gagea alexeenkoana*	EU939225
Monocotyledons	Zingiberaceae	*Alpinia*	*Alpinia oblongifolia*	JN043860
Monocotyledons	Zingiberaceae	*Alpinia*	*Alpinia oblongifolia*	JN043861
Monocotyledons	Zingiberaceae	*Alpinia*	*Alpinia oblongifolia*	JN043862
Monocotyledons	Liliaceae	*Gagea*	*Gagea granulosa*	FR690847
Monocotyledons	Liliaceae	*Gagea*	*Gagea granulosa*	FR690848
Monocotyledons	Liliaceae	*Gagea*	*Gagea granulosa*	AM238517
Monocotyledons	Liliaceae	*Gagea*	*Gagea granulosa*	AM238518
Monocotyledons	Liliaceae	*Gagea*	*Gagea ova*	FR690838
Monocotyledons	Liliaceae	*Gagea*	*Gagea ova*	FR690839
Monocotyledons	Liliaceae	*Gagea*	*Gagea ova*	AM238526
Monocotyledons	Liliaceae	*Gagea*	*Gagea ova*	AM265588
Monocotyledons	Liliaceae	*Gagea*	*Gagea vegeta*	AM238520
Monocotyledons	Liliaceae	*Gagea*	*Gagea vegeta*	EU939274
Monocotyledons	Liliaceae	*Gagea*	*Gagea vegeta*	EU939275
Monocotyledons	Zingiberaceae	*Curcuma*	*Curcuma cochinchinensis*	GQ248280
Monocotyledons	Zingiberaceae	*Curcuma*	*Curcuma cochinchinensis*	EF590684
Monocotyledons	Liliaceae	*Gagea*	*Gagea tenera*	AM238527
Monocotyledons	Liliaceae	*Gagea*	*Gagea tenera*	EU939272
Monocotyledons	Liliaceae	*Gagea*	*Gagea tenera*	EU939273
Monocotyledons	Liliaceae	*Gagea*	*Gagea glacialis*	AM265586
Monocotyledons	Liliaceae	*Gagea*	*Gagea glacialis*	FN868203
Monocotyledons	Liliaceae	*Gagea*	*Gagea glacialis*	FN868204
Monocotyledons	Liliaceae	*Gagea*	*Gagea glacialis*	FN868205
Monocotyledons	Liliaceae	*Gagea*	*Gagea glacialis*	FN868206
Monocotyledons	Liliaceae	*Gagea*	*Gagea glacialis*	FN868207
Monocotyledons	Liliaceae	*Gagea*	*Gagea glacialis*	FN868208

Monocotyledons	Liliaceae	*Gagea*	*Gagea glacialis*	FN868209
Monocotyledons	Liliaceae	*Gagea*	*Gagea glacialis*	FN868210
Monocotyledons	Liliaceae	*Gagea*	*Gagea granatellii*	AM265592
Monocotyledons	Liliaceae	*Gagea*	*Gagea granatellii*	AM282988
Monocotyledons	Liliaceae	*Gagea*	*Gagea granatellii*	AM282989
Monocotyledons	Liliaceae	*Gagea*	*Gagea granatellii*	AM282990
Monocotyledons	Liliaceae	*Gagea*	*Gagea stipitata*	AM265594
Monocotyledons	Liliaceae	*Gagea*	*Gagea stipitata*	EU939268
Monocotyledons	Liliaceae	*Gagea*	*Gagea stipitata*	EU939269
Monocotyledons	Liliaceae	*Gagea*	*Gagea stipitata*	EU939270
Monocotyledons	Liliaceae	*Gagea*	*Gagea stipitata*	EU939271
Monocotyledons	Liliaceae	*Gagea*	*Gagea sicula*	AM409350
Monocotyledons	Liliaceae	*Gagea*	*Gagea sicula*	AM409351
Monocotyledons	Liliaceae	*Gagea*	*Gagea fragifera*	AM282994
Monocotyledons	Liliaceae	*Gagea*	*Gagea fragifera*	AM282995
Monocotyledons	Liliaceae	*Gagea*	*Gagea fragifera*	EU939243
Monocotyledons	Liliaceae	*Gagea*	*Gagea fragifera*	EU939244
Monocotyledons	Liliaceae	*Gagea*	*Gagea fragifera*	EU939245
Monocotyledons	Liliaceae	*Gagea*	*Gagea fragifera*	FN868197
Monocotyledons	Liliaceae	*Gagea*	*Gagea fragifera*	FN868198
Monocotyledons	Liliaceae	*Gagea*	*Gagea fragifera*	FN868199
Monocotyledons	Liliaceae	*Gagea*	*Gagea fragifera*	FN868200
Monocotyledons	Liliaceae	*Gagea*	*Gagea fragifera*	FN868201
Monocotyledons	Liliaceae	*Gagea*	*Gagea fragifera*	FN868202
Monocotyledons	Liliaceae	*Gagea*	*Gagea polidorii*	AM282999
Monocotyledons	Liliaceae	*Gagea*	*Gagea polidorii*	FN868211
Monocotyledons	Liliaceae	*Gagea*	*Gagea polidorii*	FN868212
Monocotyledons	Poaceae	*Pappostipa*	*Pappostipa vaginata*	EU489283
Monocotyledons	Poaceae	*Pappostipa*	*Pappostipa vaginata*	EU489284
Monocotyledons	Poaceae	*Pappostipa*	*Pappostipa vaginata*	EU204749
Monocotyledons	Poaceae	*Pappostipa*	*Pappostipa vaginata*	EU204750
Monocotyledons	Liliaceae	*Gagea*	*Gagea soleirolii*	AM409352
Monocotyledons	Liliaceae	*Gagea*	*Gagea soleirolii*	EU939297
Monocotyledons	Zingiberaceae	*Curcuma*	*Curcuma attenuata*	GQ248279
Monocotyledons	Zingiberaceae	*Curcuma*	*Curcuma attenuata*	EF590683
Monocotyledons	Poaceae	*Pappostipa*	*Pappostipa chrysophylla*	EU489257
Monocotyledons	Poaceae	*Pappostipa*	*Pappostipa chrysophylla*	EU489258
Monocotyledons	Poaceae	*Pappostipa*	*Pappostipa chrysophylla*	EU489259
Monocotyledons	Poaceae	*Pappostipa*	*Pappostipa chrysophylla*	EU204729
Monocotyledons	Poaceae	*Pappostipa*	*Pappostipa chrysophylla*	EU204730
Monocotyledons	Poaceae	*Pappostipa*	*Pappostipa speciosa*	EU489279
Monocotyledons	Poaceae	*Pappostipa*	*Pappostipa speciosa*	EU489281
Monocotyledons	Poaceae	*Pappostipa*	*Pappostipa speciosa*	EU489282
Monocotyledons	Poaceae	*Pappostipa*	*Pappostipa speciosa*	EU204736
Monocotyledons	Poaceae	*Pappostipa*	*Pappostipa speciosa*	EU204737
Monocotyledons	Poaceae	*Pappostipa*	*Pappostipa speciosa*	EU204738
Monocotyledons	Poaceae	*Pappostipa*	*Pappostipa speciosa*	EU204739
Monocotyledons	Poaceae	*Pappostipa*	*Pappostipa speciosa*	EU204740
Monocotyledons	Poaceae	*Pappostipa*	*Pappostipa speciosa*	EU204741
Monocotyledons	Poaceae	*Pappostipa*	*Pappostipa speciosa*	EU204742
Monocotyledons	Poaceae	*Pappostipa*	*Pappostipa speciosa*	EU204744
Monocotyledons	Poaceae	*Pappostipa*	*Pappostipa speciosa*	EU204745
Monocotyledons	Poaceae	*Pappostipa*	*Pappostipa major*	EU204743

Monocotyledons	Poaceae	*Pappostipa*	*Pappostipa major*	EU204746
Monocotyledons	Stemonaceae	*Stemona*	*Stemona collinsae*	AB373194
Monocotyledons	Stemonaceae	*Stemona*	*Stemona collinsae*	AB373195
Monocotyledons	Hypoxidaceae	*Pauridia*	*Pauridia minuta*	FM201362
Monocotyledons	Hypoxidaceae	*Pauridia*	*Pauridia minuta*	FM201363
Monocotyledons	Hypoxidaceae	*Pauridia*	*Pauridia minuta*	FM201364
Monocotyledons	Hypoxidaceae	*Pauridia*	*Pauridia minuta*	FM201365
Monocotyledons	Hypoxidaceae	*Pauridia*	*Pauridia minuta*	FM201366
Monocotyledons	Hypoxidaceae	*Pauridia*	*Pauridia minuta*	FM201367
Monocotyledons	Hypoxidaceae	*Pauridia*	*Pauridia minuta*	FM201368
Monocotyledons	Hypoxidaceae	*Pauridia*	*Pauridia minuta*	FM201369
Monocotyledons	Hypoxidaceae	*Pauridia*	*Pauridia minuta*	FM201370
Monocotyledons	Hypoxidaceae	*Pauridia*	*Pauridia minuta*	FM201371
Monocotyledons	Hypoxidaceae	*Pauridia*	*Pauridia minuta*	FM201372
Monocotyledons	Hypoxidaceae	*Pauridia*	*Pauridia minuta*	FM201373
Monocotyledons	Hypoxidaceae	*Pauridia*	*Pauridia minuta*	FM201374
Monocotyledons	Hypoxidaceae	*Pauridia*	*Pauridia minuta*	FM201375
Monocotyledons	Hypoxidaceae	*Pauridia*	*Pauridia minuta*	FM201376
Monocotyledons	Hypoxidaceae	*Pauridia*	*Pauridia minuta*	FM201377
Monocotyledons	Hypoxidaceae	*Pauridia*	*Pauridia minuta*	FM201378
Monocotyledons	Hypoxidaceae	*Pauridia*	*Pauridia minuta*	FM201379
Monocotyledons	Hypoxidaceae	*Pauridia*	*Pauridia minuta*	FM201380
Monocotyledons	Hypoxidaceae	*Pauridia*	*Pauridia minuta*	FM201381
Monocotyledons	Hypoxidaceae	*Pauridia*	*Pauridia minuta*	FM201382
Monocotyledons	Hypoxidaceae	*Pauridia*	*Pauridia minuta*	FM201383
Monocotyledons	Hypoxidaceae	*Pauridia*	*Pauridia minuta*	FM201384
Monocotyledons	Hypoxidaceae	*Pauridia*	*Pauridia minuta*	FM201385
Monocotyledons	Hypoxidaceae	*Pauridia*	*Pauridia minuta*	FM201386
Monocotyledons	Hypoxidaceae	*Pauridia*	*Pauridia minuta*	FM201387
Monocotyledons	Hypoxidaceae	*Pauridia*	*Pauridia minuta*	FM201388
Monocotyledons	Hypoxidaceae	*Pauridia*	*Pauridia minuta*	FM201389
Monocotyledons	Hypoxidaceae	*Pauridia*	*Pauridia minuta*	FM201390
Monocotyledons	Hypoxidaceae	*Pauridia*	*Pauridia minuta*	FM201391
Monocotyledons	Hypoxidaceae	*Pauridia*	*Pauridia minuta*	FM201392
Monocotyledons	Hypoxidaceae	*Pauridia*	*Pauridia minuta*	FM201393
Monocotyledons	Hypoxidaceae	*Pauridia*	*Pauridia minuta*	FM201394
Monocotyledons	Hypoxidaceae	*Pauridia*	*Pauridia minuta*	FM201395
Monocotyledons	Hypoxidaceae	*Pauridia*	*Pauridia minuta*	FM201396
Monocotyledons	Hypoxidaceae	*Pauridia*	*Pauridia minuta*	FM201397
Monocotyledons	Hypoxidaceae	*Pauridia*	*Pauridia minuta*	FM201398
Monocotyledons	Hypoxidaceae	*Pauridia*	*Pauridia minuta*	FM201399
Monocotyledons	Hypoxidaceae	*Pauridia*	*Pauridia minuta*	FM201400
Monocotyledons	Hypoxidaceae	*Pauridia*	*Pauridia minuta*	FM201401
Monocotyledons	Hypoxidaceae	*Pauridia*	*Pauridia minuta*	FM201402
Monocotyledons	Hypoxidaceae	*Pauridia*	*Pauridia minuta*	FM201403
Monocotyledons	Hypoxidaceae	*Pauridia*	*Pauridia minuta*	FM201404
Monocotyledons	Hypoxidaceae	*Pauridia*	*Pauridia minuta*	FM201405
Monocotyledons	Hypoxidaceae	*Pauridia*	*Pauridia minuta*	FM201406
Monocotyledons	Hypoxidaceae	*Pauridia*	*Pauridia minuta*	FM201407
Monocotyledons	Hypoxidaceae	*Pauridia*	*Pauridia minuta*	FM201408
Monocotyledons	Hypoxidaceae	*Pauridia*	*Pauridia minuta*	FM201409
Monocotyledons	Hypoxidaceae	*Pauridia*	*Pauridia minuta*	FM201410
Monocotyledons	Hypoxidaceae	*Pauridia*	*Pauridia minuta*	FM201411

Monocotyledons	Hypoxidaceae	*Pauridia*	*Pauridia minuta*	FM201412
Monocotyledons	Hypoxidaceae	*Pauridia*	*Pauridia minuta*	FM201413
Monocotyledons	Hypoxidaceae	*Pauridia*	*Pauridia minuta*	FM201414
Monocotyledons	Hypoxidaceae	*Pauridia*	*Pauridia minuta*	FM201415
Monocotyledons	Hypoxidaceae	*Pauridia*	*Pauridia minuta*	FM201416
Monocotyledons	Hypoxidaceae	*Pauridia*	*Pauridia minuta*	FM201417
Monocotyledons	Hypoxidaceae	*Pauridia*	*Pauridia minuta*	FM201418
Monocotyledons	Hypoxidaceae	*Pauridia*	*Pauridia minuta*	FM201419
Monocotyledons	Hypoxidaceae	*Pauridia*	*Pauridia minuta*	FM201420
Monocotyledons	Hypoxidaceae	*Pauridia*	*Pauridia minuta*	FM201421
Monocotyledons	Hypoxidaceae	*Pauridia*	*Pauridia minuta*	FM201422
Monocotyledons	Hypoxidaceae	*Pauridia*	*Pauridia minuta*	FM201423
Monocotyledons	Hypoxidaceae	*Pauridia*	*Pauridia minuta*	FM201424
Monocotyledons	Hypoxidaceae	*Pauridia*	*Pauridia minuta*	FM201425
Monocotyledons	Hypoxidaceae	*Pauridia*	*Pauridia minuta*	FM201426
Monocotyledons	Hypoxidaceae	*Pauridia*	*Pauridia minuta*	FM201427
Monocotyledons	Hypoxidaceae	*Pauridia*	*Pauridia minuta*	FM201428
Monocotyledons	Hypoxidaceae	*Pauridia*	*Pauridia minuta*	FM201429
Monocotyledons	Hypoxidaceae	*Pauridia*	*Pauridia minuta*	FM201430
Monocotyledons	Hypoxidaceae	*Pauridia*	*Pauridia minuta*	FM201431
Monocotyledons	Hypoxidaceae	*Pauridia*	*Pauridia minuta*	FM201432
Monocotyledons	Hypoxidaceae	*Pauridia*	*Pauridia minuta*	FM201433
Monocotyledons	Hypoxidaceae	*Pauridia*	*Pauridia minuta*	FM201434
Monocotyledons	Hypoxidaceae	*Pauridia*	*Pauridia minuta*	FM201435
Monocotyledons	Hypoxidaceae	*Pauridia*	*Pauridia minuta*	FM201436
Monocotyledons	Hypoxidaceae	*Pauridia*	*Pauridia minuta*	FM201437
Monocotyledons	Hypoxidaceae	*Pauridia*	*Pauridia minuta*	FM201438
Monocotyledons	Hypoxidaceae	*Pauridia*	*Pauridia minuta*	FM201439
Monocotyledons	Hypoxidaceae	*Pauridia*	*Pauridia minuta*	FM201440
Monocotyledons	Hypoxidaceae	*Pauridia*	*Pauridia minuta*	FM201441
Monocotyledons	Hypoxidaceae	*Pauridia*	*Pauridia minuta*	FM201442
Monocotyledons	Hypoxidaceae	*Pauridia*	*Pauridia minuta*	FM201443
Monocotyledons	Hypoxidaceae	*Pauridia*	*Pauridia minuta*	FM201444
Monocotyledons	Hypoxidaceae	*Pauridia*	*Pauridia minuta*	FM201445
Monocotyledons	Hypoxidaceae	*Pauridia*	*Pauridia minuta*	FM201446
Monocotyledons	Hypoxidaceae	*Pauridia*	*Pauridia minuta*	FM201447
Monocotyledons	Hypoxidaceae	*Pauridia*	*Pauridia minuta*	FM201448
Monocotyledons	Hypoxidaceae	*Pauridia*	*Pauridia minuta*	FM201449
Monocotyledons	Hypoxidaceae	*Pauridia*	*Pauridia minuta*	FM201450
Monocotyledons	Hypoxidaceae	*Pauridia*	*Pauridia minuta*	FM201451
Monocotyledons	Liliaceae	*Gagea*	*Gagea caroli-kochii*	EU939228
Monocotyledons	Liliaceae	*Gagea*	*Gagea caroli-kochii*	EU939229
Monocotyledons	Liliaceae	*Gagea*	*Gagea chlorantha*	EU939230
Monocotyledons	Liliaceae	*Gagea*	*Gagea chlorantha*	EU939231
Monocotyledons	Liliaceae	*Gagea*	*Gagea chlorantha*	EU939232
Monocotyledons	Liliaceae	*Gagea*	*Gagea chlorantha*	EU939233
Monocotyledons	Liliaceae	*Gagea*	*Gagea commutata*	EU939237
Monocotyledons	Liliaceae	*Gagea*	*Gagea commutata*	EU939296
Monocotyledons	Liliaceae	*Gagea*	*Gagea iranica*	EU939248
Monocotyledons	Liliaceae	*Gagea*	*Gagea iranica*	EU939249
Monocotyledons	Liliaceae	*Gagea*	*Gagea setifolia*	EU939253
Monocotyledons	Liliaceae	*Gagea*	*Gagea setifolia*	EU939261
Monocotyledons	Liliaceae	*Gagea*	*Gagea setifolia*	EU939262

Monocotyledons	Liliaceae	*Gagea*	*Gagea setifolia*	EU939263
Monocotyledons	Liliaceae	*Gagea*	*Gagea setifolia*	EU939264
Monocotyledons	Liliaceae	*Gagea*	*Gagea setifolia*	EU939265
Monocotyledons	Liliaceae	*Gagea*	*Gagea setifolia*	EU939266
Monocotyledons	Liliaceae	*Gagea*	*Gagea setifolia*	EU939267
Monocotyledons	Liliaceae	*Gagea*	*Gagea uliginosa*	EU939281
Monocotyledons	Liliaceae	*Gagea*	*Gagea uliginosa*	EU939282
Monocotyledons	Liliaceae	*Gagea*	*Gagea uliginosa*	EU939283
Monocotyledons	Liliaceae	*Gagea*	*Gagea lojaconoi*	AM282997
Monocotyledons	Liliaceae	*Gagea*	*Gagea lojaconoi*	AM282998
Monocotyledons	Hyacinthaceae	*Hyacinthoides*	*Hyacinthoides ciliolata*	FJ423299
Monocotyledons	Hyacinthaceae	*Hyacinthoides*	*Hyacinthoides ciliolata*	FJ423300
Monocotyledons	Hyacinthaceae	*Hyacinthoides*	*Hyacinthoides ciliolata*	FJ423301
Monocotyledons	Hyacinthaceae	*Hyacinthoides*	*Hyacinthoides flahaultiana*	FJ423313
Monocotyledons	Hyacinthaceae	*Hyacinthoides*	*Hyacinthoides flahaultiana*	FJ423314
Monocotyledons	Hyacinthaceae	*Hyacinthoides*	*Hyacinthoides flahaultiana*	FJ423315
Monocotyledons	Hyacinthaceae	*Hyacinthoides*	*Hyacinthoides mauritanica*	FJ423309
Monocotyledons	Hyacinthaceae	*Hyacinthoides*	*Hyacinthoides mauritanica*	FJ423310
Monocotyledons	Hyacinthaceae	*Hyacinthoides*	*Hyacinthoides mauritanica*	FJ423311
Monocotyledons	Hyacinthaceae	*Hyacinthoides*	*Hyacinthoides mauritanica*	FJ423312
Monocotyledons	Hyacinthaceae	*Hyacinthoides*	*Hyacinthoides paivae*	FJ423346
Monocotyledons	Hyacinthaceae	*Hyacinthoides*	*Hyacinthoides paivae*	FJ423347
Monocotyledons	Liliaceae	*Gagea*	*Gagea brentae*	FN868195
Monocotyledons	Liliaceae	*Gagea*	*Gagea brentae*	FN868196
Monocotyledons	Zingiberaceae	*Kaempferia*	*Kaempferia sp. JT-2010a*	GQ385994
Monocotyledons	Zingiberaceae	*Kaempferia*	*Kaempferia sp. JT-2010a*	GQ385995
Monocotyledons	Zingiberaceae	*Kaempferia*	*Kaempferia sp. JT-2010b*	GQ385998
Monocotyledons	Zingiberaceae	*Kaempferia*	*Kaempferia sp. JT-2010b*	GQ385999
Monocotyledons	Zingiberaceae	*Kaempferia*	*Kaempferia fallax*	GQ386018
Monocotyledons	Zingiberaceae	*Kaempferia*	*Kaempferia fallax*	GQ386019
Monocotyledons	Zingiberaceae	*Kaempferia*	*Kaempferia fallax*	GQ386020
Monocotyledons	Zingiberaceae	*Kaempferia*	*Kaempferia fallax*	GQ386021
Monocotyledons	Zingiberaceae	*Kaempferia*	*Kaempferia filifolia*	GQ386022
Monocotyledons	Zingiberaceae	*Kaempferia*	*Kaempferia filifolia*	GQ386023
Monocotyledons	Zingiberaceae	*Kaempferia*	*Kaempferia filifolia*	GQ386024
Monocotyledons	Zingiberaceae	*Kaempferia*	*Kaempferia laotica*	GQ385985
Monocotyledons	Zingiberaceae	*Kaempferia*	*Kaempferia laotica*	GQ385987
Monocotyledons	Zingiberaceae	*Kaempferia*	*Kaempferia larsenii*	GQ385989
Monocotyledons	Zingiberaceae	*Kaempferia*	*Kaempferia larsenii*	GQ385990
Monocotyledons	Zingiberaceae	*Kaempferia*	*Kaempferia larsenii*	GQ385991
Monocotyledons	Zingiberaceae	*Kaempferia*	*Kaempferia larsenii*	GQ385992
Monocotyledons	Zingiberaceae	*Kaempferia*	*Kaempferia larsenii*	GQ385993
Monocotyledons	Zingiberaceae	*Kaempferia*	*Kaempferia sp. JT-2010c*	GQ386043
Monocotyledons	Zingiberaceae	*Kaempferia*	*Kaempferia sp. JT-2010c*	GQ386044
Monocotyledons	Zingiberaceae	*Kaempferia*	*Kaempferia sp. JT-2010c*	GQ386045
Monocotyledons	Zingiberaceae	*Kaempferia*	*Kaempferia sp. JT-2010c*	GQ386046
Monocotyledons	Zingiberaceae	*Kaempferia*	*Kaempferia sp. JT-2010d*	GQ385996
Monocotyledons	Zingiberaceae	*Kaempferia*	*Kaempferia sp. JT-2010d*	GQ385997
Monocotyledons	Zingiberaceae	*Kaempferia*	*Kaempferia siamensis*	GQ386039
Monocotyledons	Zingiberaceae	*Kaempferia*	*Kaempferia siamensis*	GQ386040
Monocotyledons	Zingiberaceae	*Kaempferia*	*Kaempferia sp. nov. 1 JT-2010*	GQ386001
Monocotyledons	Zingiberaceae	*Kaempferia*	*Kaempferia sp. nov. 1 JT-2010*	GQ386002
Monocotyledons	Liliaceae	*Gagea*	*Gagea davlianidzeae*	FR690831

Monocotyledons	Liliaceae	*Gagea*	*Gagea davlianidzeae*	FR690832
Monocotyledons	Liliaceae	*Gagea*	*Gagea davlianidzeae*	FR690833
Monocotyledons	Liliaceae	*Gagea*	*Gagea davlianidzeae*	FR690834
Monocotyledons	Liliaceae	*Gagea*	*Gagea davlianidzeae*	FR690835
Monocotyledons	Liliaceae	*Gagea*	*Gagea davlianidzeae*	FR690836
Monocotyledons	Liliaceae	*Gagea*	*Gagea davlianidzeae*	FR690837
Monocotyledons	Liliaceae	*Gagea*	*Gagea rufidula*	FR690840
Monocotyledons	Liliaceae	*Gagea*	*Gagea rufidula*	FR690841
Monocotyledons	Liliaceae	*Gagea*	*Gagea rufidula*	FR690842
Monocotyledons	Liliaceae	*Gagea*	*Gagea rufidula*	FR690843
Monocotyledons	Liliaceae	*Gagea*	*Gagea nigra*	FR691010
Monocotyledons	Liliaceae	*Gagea*	*Gagea nigra*	FR691011
Monocotyledons	Liliaceae	*Gagea*	*Gagea nigra*	FR691012
Monocotyledons	Liliaceae	*Gagea*	*Gagea nigra*	FR691013
Monocotyledons	Liliaceae	*Gagea*	*Gagea nigra*	FR691014
Monocotyledons	Liliaceae	*Gagea*	*Gagea nigra*	FR691015
Monocotyledons	Liliaceae	*Gagea*	*Gagea nigra*	FR691016
Monocotyledons	Liliaceae	*Gagea*	*Gagea nigra*	FR691017
Monocotyledons	Liliaceae	*Gagea*	*Gagea nigra*	FR691018
Monocotyledons	Liliaceae	*Gagea*	*Gagea nigra*	FR691019
Monocotyledons	Liliaceae	*Gagea*	*Gagea nigra*	FR691020
Monocotyledons	Liliaceae	*Gagea*	*Gagea nigra*	FR691021
Monocotyledons	Liliaceae	*Gagea*	*Gagea nigra*	FR691022
Monocotyledons	Liliaceae	*Gagea*	*Gagea nigra*	FR691023
Monocotyledons	Liliaceae	*Gagea*	*Gagea nigra*	FR691024
Monocotyledons	Liliaceae	*Gagea*	*Gagea xiphoidea*	FR691044
Monocotyledons	Liliaceae	*Gagea*	*Gagea xiphoidea*	FR691045
Monocotyledons	Liliaceae	*Gagea*	*Gagea angelae*	FR691028
Monocotyledons	Liliaceae	*Gagea*	*Gagea angelae*	FR691029
Monocotyledons	Liliaceae	*Gagea*	*Gagea angelae*	FR691030
Monocotyledons	Liliaceae	*Gagea*	*Gagea angelae*	FR691031
Monocotyledons	Liliaceae	*Gagea*	*Gagea angelae*	FR691032
Monocotyledons	Liliaceae	*Gagea*	*Gagea angelae*	FR691033
Monocotyledons	Liliaceae	*Gagea*	*Gagea angelae*	FR691034
Monocotyledons	Liliaceae	*Gagea*	*Gagea angelae*	FR691035
Monocotyledons	Liliaceae	*Gagea*	*Gagea angelae*	FR691036
Monocotyledons	Liliaceae	*Gagea*	*Gagea angelae*	FR691037
Monocotyledons	Liliaceae	*Gagea*	*Gagea huochengensis*	FR691038
Monocotyledons	Liliaceae	*Gagea*	*Gagea huochengensis*	FR691039
Monocotyledons	Liliaceae	*Gagea*	*Gagea huochengensis*	FR691040
Monocotyledons	Liliaceae	*Gagea*	*Gagea huochengensis*	FR691041
Monocotyledons	Liliaceae	*Gagea*	*Gagea huochengensis*	FR691042
Monocotyledons	Liliaceae	*Gagea*	*Gagea huochengensis*	FR691043
Monocotyledons	Liliaceae	*Gagea*	*Gagea jensii*	FR691025
Monocotyledons	Liliaceae	*Gagea*	*Gagea jensii*	FR691026
Monocotyledons	Liliaceae	*Gagea*	*Gagea jensii*	FR691027
Monocotyledons	Poaceae	*Digitaria*	*Digitaria macroblephara*	HQ876950
Monocotyledons	Poaceae	*Digitaria*	*Digitaria macroblephara*	HQ876951
Monocotyledons	Poaceae	*Digitaria*	*Digitaria macroblephara*	HQ876952
Monocotyledons	Poaceae	*Digitaria*	*Digitaria abyssinica*	HQ876953
Monocotyledons	Poaceae	*Digitaria*	*Digitaria abyssinica*	HQ876954
Monocotyledons	Poaceae	*Digitaria*	*Digitaria ternata*	HQ876955
Monocotyledons	Poaceae	*Digitaria*	*Digitaria ternata*	HQ876956

Monocotyledons	Zingiberaceae	*Alpinia*	*Alpinia emaculata*	JN043831
Monocotyledons	Zingiberaceae	*Alpinia*	*Alpinia emaculata*	JN043832
Monocotyledons	Zingiberaceae	*Alpinia*	*Alpinia platychilus*	JN043867
Monocotyledons	Zingiberaceae	*Alpinia*	*Alpinia platychilus*	JN043868
Monocotyledons	Zingiberaceae	*Alpinia*	*Alpinia platychilus*	JN043869
Monocotyledons	Zingiberaceae	*Alpinia*	*Alpinia platychilus*	JN043870
Monocotyledons	Zingiberaceae	*Alpinia*	*Alpinia graminifolia*	JN043844
Monocotyledons	Zingiberaceae	*Alpinia*	*Alpinia graminifolia*	JN043845
Mosses	Polytrichaceae	*Polytrichum*	*Polytrichum commune*	EU750671
Mosses	Polytrichaceae	*Polytrichum*	*Polytrichum commune*	EU750672
Mosses	Polytrichaceae	*Polytrichum*	*Polytrichum commune*	EF590730
Mosses	Dicranaceae	*Dicranum*	*Dicranum scoparium*	GQ428018
Mosses	Dicranaceae	*Dicranum*	*Dicranum scoparium*	GQ428016
Mosses	Dicranaceae	*Dicranum*	*Dicranum scoparium*	GQ428012
Mosses	Dicranaceae	*Dicranum*	*Dicranum scoparium*	GQ428010
Mosses	Dicranaceae	*Dicranum*	*Dicranum scoparium*	GQ428008
Mosses	Dicranaceae	*Dicranum*	*Dicranum scoparium*	GQ428006
Mosses	Dicranaceae	*Dicranum*	*Dicranum scoparium*	GQ428004
Mosses	Dicranaceae	*Dicranum*	*Dicranum scoparium*	GQ428002
Mosses	Dicranaceae	*Dicranum*	*Dicranum scoparium*	GQ428000
Mosses	Dicranaceae	*Dicranum*	*Dicranum scoparium*	GQ427998
Mosses	Dicranaceae	*Dicranum*	*Dicranum scoparium*	GQ427996
Mosses	Dicranaceae	*Dicranum*	*Dicranum scoparium*	GQ427994
Mosses	Dicranaceae	*Dicranum*	*Dicranum scoparium*	GQ427992
Mosses	Dicranaceae	*Dicranum*	*Dicranum scoparium*	GQ428017
Mosses	Dicranaceae	*Dicranum*	*Dicranum scoparium*	GQ428015
Mosses	Dicranaceae	*Dicranum*	*Dicranum scoparium*	GQ428013
Mosses	Dicranaceae	*Dicranum*	*Dicranum scoparium*	GQ428011
Mosses	Dicranaceae	*Dicranum*	*Dicranum scoparium*	GQ428009
Mosses	Dicranaceae	*Dicranum*	*Dicranum scoparium*	GQ428007
Mosses	Dicranaceae	*Dicranum*	*Dicranum scoparium*	GQ428005
Mosses	Dicranaceae	*Dicranum*	*Dicranum scoparium*	GQ428003
Mosses	Dicranaceae	*Dicranum*	*Dicranum scoparium*	GQ428001
Mosses	Dicranaceae	*Dicranum*	*Dicranum scoparium*	GQ427999
Mosses	Dicranaceae	*Dicranum*	*Dicranum scoparium*	GQ427997
Mosses	Dicranaceae	*Dicranum*	*Dicranum scoparium*	GQ427995
Mosses	Dicranaceae	*Dicranum*	*Dicranum scoparium*	GQ427993
Mosses	Dicranaceae	*Dicranum*	*Dicranum scoparium*	GQ427991
Mosses	Dicranaceae	*Dicranum*	*Dicranum scoparium*	GQ248289
Mosses	Dicranaceae	*Dicranum*	*Dicranum scoparium*	GU068473
Mosses	Dicranaceae	*Dicranum*	*Dicranum scoparium*	GU068471
Mosses	Dicranaceae	*Dicranum*	*Dicranum scoparium*	GU068469
Mosses	Dicranaceae	*Dicranum*	*Dicranum scoparium*	GU068467
Mosses	Dicranaceae	*Dicranum*	*Dicranum scoparium*	GU068465
Mosses	Dicranaceae	*Dicranum*	*Dicranum scoparium*	GU068463
Mosses	Dicranaceae	*Dicranum*	*Dicranum scoparium*	GU068461
Mosses	Dicranaceae	*Dicranum*	*Dicranum scoparium*	GU068459
Mosses	Dicranaceae	*Dicranum*	*Dicranum scoparium*	GU068457
Mosses	Dicranaceae	*Dicranum*	*Dicranum scoparium*	GU068455
Mosses	Dicranaceae	*Dicranum*	*Dicranum scoparium*	GU068453
Mosses	Dicranaceae	*Dicranum*	*Dicranum scoparium*	GU068451
Mosses	Dicranaceae	*Dicranum*	*Dicranum scoparium*	GU068449
Mosses	Dicranaceae	*Dicranum*	*Dicranum scoparium*	GU068474

Mosses	Dicranaceae	*Dicranum*	*Dicranum scoparium*	GU068472
Mosses	Dicranaceae	*Dicranum*	*Dicranum scoparium*	GU068470
Mosses	Dicranaceae	*Dicranum*	*Dicranum scoparium*	GU068468
Mosses	Dicranaceae	*Dicranum*	*Dicranum scoparium*	GU068466
Mosses	Dicranaceae	*Dicranum*	*Dicranum scoparium*	GU068464
Mosses	Dicranaceae	*Dicranum*	*Dicranum scoparium*	GU068462
Mosses	Dicranaceae	*Dicranum*	*Dicranum scoparium*	GU068460
Mosses	Dicranaceae	*Dicranum*	*Dicranum scoparium*	GU068458
Mosses	Dicranaceae	*Dicranum*	*Dicranum scoparium*	GU068456
Mosses	Dicranaceae	*Dicranum*	*Dicranum scoparium*	GU068454
Mosses	Dicranaceae	*Dicranum*	*Dicranum scoparium*	GU068452
Mosses	Dicranaceae	*Dicranum*	*Dicranum scoparium*	GU068450
Mosses	Dicranaceae	*Dicranum*	*Dicranum scoparium*	GU068448
Mosses	Dicranaceae	*Dicranum*	*Dicranum scoparium*	EF590690
Mosses	Orthotrichaceae	*Orthotrichum*	*Orthotrichum stramineum*	EU163553
Mosses	Orthotrichaceae	*Orthotrichum*	*Orthotrichum stramineum*	FJ036891
Mosses	Orthotrichaceae	*Orthotrichum*	*Orthotrichum stramineum*	FJ036889
Mosses	Orthotrichaceae	*Orthotrichum*	*Orthotrichum stramineum*	FJ036890
Mosses	Brachytheciaceae	*Brachythecium*	*Brachythecium rutabulum*	GQ428022
Mosses	Brachytheciaceae	*Brachythecium*	*Brachythecium rutabulum*	FJ572511
Mosses	Brachytheciaceae	*Brachythecium*	*Brachythecium rutabulum*	FJ572512
Mosses	Hypnaceae	*Hypnum*	*Hypnum cupressiforme*	GQ849569
Mosses	Hypnaceae	*Hypnum*	*Hypnum cupressiforme*	GQ849570
Mosses	Hypnaceae	*Hypnum*	*Hypnum cupressiforme*	GQ849568
Mosses	Orthotrichaceae	*Orthotrichum*	*Orthotrichum anomalum*	EU163551
Mosses	Orthotrichaceae	*Orthotrichum*	*Orthotrichum anomalum*	GQ370333
Mosses	Orthotrichaceae	*Orthotrichum*	*Orthotrichum lyellii*	AY312911
Mosses	Orthotrichaceae	*Orthotrichum*	*Orthotrichum lyellii*	FJ036875
Mosses	Orthotrichaceae	*Orthotrichum*	*Orthotrichum lyellii*	FJ036873
Mosses	Orthotrichaceae	*Orthotrichum*	*Orthotrichum lyellii*	FJ036874
Mosses	Orthotrichaceae	*Orthotrichum*	*Orthotrichum lyellii*	FJ036872
Mosses	Orthotrichaceae	*Nyholmiella*	*Nyholmiella obtusifolia*	GQ370351
Mosses	Orthotrichaceae	*Nyholmiella*	*Nyholmiella obtusifolia*	GQ370349
Mosses	Orthotrichaceae	*Nyholmiella*	*Nyholmiella obtusifolia*	GQ370350
Mosses	Orthotrichaceae	*Nyholmiella*	*Nyholmiella obtusifolia*	GQ370348
Mosses	Grimmiaceae	*Schistidium*	*Schistidium apocarpum*	GU808911
Mosses	Grimmiaceae	*Schistidium*	*Schistidium apocarpum*	GU808918
Mosses	Grimmiaceae	*Schistidium*	*Schistidium apocarpum*	GU808912
Mosses	Grimmiaceae	*Schistidium*	*Schistidium apocarpum*	EU163518
Mosses	Grimmiaceae	*Schistidium*	*Schistidium apocarpum*	GQ428033
Mosses	Mniaceae	*Plagiomnium*	*Plagiomnium cuspidatum*	FJ572549
Mosses	Mniaceae	*Plagiomnium*	*Plagiomnium cuspidatum*	FJ572547
Mosses	Mniaceae	*Plagiomnium*	*Plagiomnium cuspidatum*	FJ572548
Mosses	Mniaceae	*Plagiomnium*	*Plagiomnium cuspidatum*	EU750667
Mosses	Mniaceae	*Plagiomnium*	*Plagiomnium cuspidatum*	EU750668
Mosses	Orthotrichaceae	*Orthotrichum*	*Orthotrichum speciosum*	FJ036865
Mosses	Orthotrichaceae	*Orthotrichum*	*Orthotrichum speciosum*	FJ036863
Mosses	Orthotrichaceae	*Orthotrichum*	*Orthotrichum speciosum*	FJ036866
Mosses	Orthotrichaceae	*Orthotrichum*	*Orthotrichum speciosum*	FJ036864
Mosses	Brachytheciaceae	*Brachythecium*	*Brachythecium salebrosum*	FJ572513
Mosses	Brachytheciaceae	*Brachythecium*	*Brachythecium salebrosum*	FJ572514
Mosses	Brachytheciaceae	*Brachythecium*	*Brachythecium salebrosum*	AY312896
Mosses	Brachytheciaceae	*Brachythecium*	*Brachythecium salebrosum*	EU750661

Mosses	Brachytheciaceae	*Brachythecium*	*Brachythecium salebrosum*	EU750660
Mosses	Brachytheciaceae	*Brachythecium*	*Brachythecium oxycladon*	EU750657
Mosses	Brachytheciaceae	*Brachythecium*	*Brachythecium oxycladon*	EU750656
Mosses	Brachytheciaceae	*Brachythecium*	*Brachythecium plumosum*	FJ572509
Mosses	Brachytheciaceae	*Brachythecium*	*Brachythecium plumosum*	FJ572510
Mosses	Ptychomniaceae	*Ptychomnion*	*Ptychomnion aciculare*	EU125860
Mosses	Ptychomniaceae	*Ptychomnion*	*Ptychomnion aciculare*	EU125859
Mosses	Mniaceae	*Plagiomnium*	*Plagiomnium undulatum*	EU163539
Mosses	Mniaceae	*Plagiomnium*	*Plagiomnium undulatum*	GQ428032
Mosses	Dicranaceae	*Dicranum*	*Dicranum polysetum*	EU163523
Mosses	Dicranaceae	*Dicranum*	*Dicranum polysetum*	EU750665
Mosses	Dicranaceae	*Dicranum*	*Dicranum polysetum*	EU750666
Mosses	Sphagnaceae	*Sphagnum*	*Sphagnum quinquefarium*	AY309617
Mosses	Sphagnaceae	*Sphagnum*	*Sphagnum quinquefarium*	GQ428034
Mosses	Polytrichaceae	*Polytrichum*	*Polytrichum juniperinum*	GQ248374
Mosses	Polytrichaceae	*Polytrichum*	*Polytrichum juniperinum*	FJ572555
Mosses	Polytrichaceae	*Polytrichum*	*Polytrichum juniperinum*	FJ572554
Mosses	Polytrichaceae	*Polytrichum*	*Polytrichum juniperinum*	EU750675
Mosses	Polytrichaceae	*Polytrichum*	*Polytrichum juniperinum*	EU750673
Mosses	Polytrichaceae	*Polytrichum*	*Polytrichum juniperinum*	EU750674
Mosses	Polytrichaceae	*Polytrichum*	*Polytrichum juniperinum*	EF590731
Mosses	Grimmiaceae	*Grimmia*	*Grimmia laevigata*	GU808929
Mosses	Grimmiaceae	*Grimmia*	*Grimmia laevigata*	GU808942
Mosses	Grimmiaceae	*Codriophorus*	*Codriophorus fascicularis*	GU808889
Mosses	Grimmiaceae	*Codriophorus*	*Codriophorus fascicularis*	GU808887
Mosses	Grimmiaceae	*Codriophorus*	*Codriophorus fascicularis*	GU808890
Mosses	Grimmiaceae	*Codriophorus*	*Codriophorus fascicularis*	GU808888
Mosses	Grimmiaceae	*Codriophorus*	*Codriophorus fascicularis*	FJ572563
Mosses	Grimmiaceae	*Codriophorus*	*Codriophorus fascicularis*	FJ572562
Mosses	Sphagnaceae	*Sphagnum*	*Sphagnum subsecundum*	FJ572571
Mosses	Sphagnaceae	*Sphagnum*	*Sphagnum subsecundum*	FJ572572
Mosses	Ptychomniaceae	*Ptychomnion*	*Ptychomnion cygnisetum*	EU125862
Mosses	Ptychomniaceae	*Ptychomnion*	*Ptychomnion cygnisetum*	EU125863
Mosses	Ptychomniaceae	*Ptychomnion*	*Ptychomnion cygnisetum*	EU125861
Mosses	Grimmiaceae	*Grimmia*	*Grimmia pilifera*	GU808937
Mosses	Grimmiaceae	*Grimmia*	*Grimmia pilifera*	GU808935
Mosses	Grimmiaceae	*Grimmia*	*Grimmia pilifera*	GU808938
Mosses	Grimmiaceae	*Grimmia*	*Grimmia pilifera*	GU808936
Mosses	Grimmiaceae	*Grimmia*	*Grimmia pilifera*	GU808934
Mosses	Grimmiaceae	*Niphotrichum*	*Niphotrichum japonicum*	GU808901
Mosses	Grimmiaceae	*Niphotrichum*	*Niphotrichum japonicum*	GU808899
Mosses	Grimmiaceae	*Niphotrichum*	*Niphotrichum japonicum*	GU808897
Mosses	Grimmiaceae	*Niphotrichum*	*Niphotrichum japonicum*	GU808902
Mosses	Grimmiaceae	*Niphotrichum*	*Niphotrichum japonicum*	GU808900
Mosses	Grimmiaceae	*Niphotrichum*	*Niphotrichum japonicum*	GU808898
Mosses	Grimmiaceae	*Schistidium*	*Schistidium strictum*	GU808917
Mosses	Grimmiaceae	*Schistidium*	*Schistidium strictum*	GU808915
Mosses	Grimmiaceae	*Schistidium*	*Schistidium strictum*	GU808916
Mosses	Grimmiaceae	*Schistidium*	*Schistidium strictum*	GU808914
Mosses	Orthotrichaceae	*Orthotrichum*	*Orthotrichum affine*	FJ036879
Mosses	Orthotrichaceae	*Orthotrichum*	*Orthotrichum affine*	FJ036877
Mosses	Orthotrichaceae	*Orthotrichum*	*Orthotrichum affine*	FJ036878
Mosses	Orthotrichaceae	*Orthotrichum*	*Orthotrichum affine*	FJ036876

Mosses	Grimmiaceae	*Grimmia*	*Grimmia longirostris*	GU808933
Mosses	Grimmiaceae	*Grimmia*	*Grimmia longirostris*	GU808931
Mosses	Grimmiaceae	*Grimmia*	*Grimmia longirostris*	GU808932
Mosses	Grimmiaceae	*Grimmia*	*Grimmia longirostris*	GU808928
Mosses	Grimmiaceae	*Grimmia*	*Grimmia montana*	GU808943
Mosses	Grimmiaceae	*Grimmia*	*Grimmia montana*	EF429434
Mosses	Grimmiaceae	*Grimmia*	*Grimmia montana*	EF429432
Mosses	Grimmiaceae	*Grimmia*	*Grimmia montana*	EF429430
Mosses	Grimmiaceae	*Grimmia*	*Grimmia montana*	EF429428
Mosses	Grimmiaceae	*Grimmia*	*Grimmia montana*	EF429426
Mosses	Grimmiaceae	*Grimmia*	*Grimmia montana*	EF429424
Mosses	Grimmiaceae	*Grimmia*	*Grimmia montana*	EF429422
Mosses	Grimmiaceae	*Grimmia*	*Grimmia montana*	EF429420
Mosses	Grimmiaceae	*Grimmia*	*Grimmia montana*	EF429418
Mosses	Grimmiaceae	*Grimmia*	*Grimmia montana*	EF429416
Mosses	Grimmiaceae	*Grimmia*	*Grimmia montana*	EF429414
Mosses	Grimmiaceae	*Grimmia*	*Grimmia montana*	EF429412
Mosses	Grimmiaceae	*Grimmia*	*Grimmia montana*	EF429410
Mosses	Grimmiaceae	*Grimmia*	*Grimmia montana*	EF429408
Mosses	Grimmiaceae	*Grimmia*	*Grimmia montana*	EF429406
Mosses	Grimmiaceae	*Grimmia*	*Grimmia montana*	EF429404
Mosses	Grimmiaceae	*Grimmia*	*Grimmia montana*	EF429400
Mosses	Grimmiaceae	*Grimmia*	*Grimmia montana*	EF429398
Mosses	Grimmiaceae	*Grimmia*	*Grimmia montana*	EF429396
Mosses	Grimmiaceae	*Grimmia*	*Grimmia montana*	EF429394
Mosses	Grimmiaceae	*Grimmia*	*Grimmia montana*	EF429392
Mosses	Grimmiaceae	*Grimmia*	*Grimmia montana*	EF429390
Mosses	Grimmiaceae	*Grimmia*	*Grimmia montana*	EF429388
Mosses	Grimmiaceae	*Grimmia*	*Grimmia montana*	EF429386
Mosses	Grimmiaceae	*Grimmia*	*Grimmia montana*	EF429384
Mosses	Grimmiaceae	*Grimmia*	*Grimmia montana*	EF429433
Mosses	Grimmiaceae	*Grimmia*	*Grimmia montana*	EF429431
Mosses	Grimmiaceae	*Grimmia*	*Grimmia montana*	EF429429
Mosses	Grimmiaceae	*Grimmia*	*Grimmia montana*	EF429427
Mosses	Grimmiaceae	*Grimmia*	*Grimmia montana*	EF429425
Mosses	Grimmiaceae	*Grimmia*	*Grimmia montana*	EF429423
Mosses	Grimmiaceae	*Grimmia*	*Grimmia montana*	EF429421
Mosses	Grimmiaceae	*Grimmia*	*Grimmia montana*	EF429419
Mosses	Grimmiaceae	*Grimmia*	*Grimmia montana*	EF429417
Mosses	Grimmiaceae	*Grimmia*	*Grimmia montana*	EF429415
Mosses	Grimmiaceae	*Grimmia*	*Grimmia montana*	EF429413
Mosses	Grimmiaceae	*Grimmia*	*Grimmia montana*	EF429411
Mosses	Grimmiaceae	*Grimmia*	*Grimmia montana*	EF429409
Mosses	Grimmiaceae	*Grimmia*	*Grimmia montana*	EF429407
Mosses	Grimmiaceae	*Grimmia*	*Grimmia montana*	EF429405
Mosses	Grimmiaceae	*Grimmia*	*Grimmia montana*	EF429403
Mosses	Grimmiaceae	*Grimmia*	*Grimmia montana*	EF429401
Mosses	Grimmiaceae	*Grimmia*	*Grimmia montana*	EF429399
Mosses	Grimmiaceae	*Grimmia*	*Grimmia montana*	EF429397
Mosses	Grimmiaceae	*Grimmia*	*Grimmia montana*	EF429395
Mosses	Grimmiaceae	*Grimmia*	*Grimmia montana*	EF429393
Mosses	Grimmiaceae	*Grimmia*	*Grimmia montana*	EF429391
Mosses	Grimmiaceae	*Grimmia*	*Grimmia montana*	EF429389

Mosses	Grimmiaceae	*Grimmia*	*Grimmia montana*	EF429387
Mosses	Grimmiaceae	*Grimmia*	*Grimmia montana*	EF429385
Mosses	Grimmiaceae	*Grimmia*	*Grimmia montana*	EF429383
Mosses	Grimmiaceae	*Grimmia*	*Grimmia montana*	EF429381
Mosses	Grimmiaceae	*Grimmia*	*Grimmia ovalis*	GU808927
Mosses	Grimmiaceae	*Grimmia*	*Grimmia ovalis*	GU808925
Mosses	Grimmiaceae	*Grimmia*	*Grimmia ovalis*	GU808926
Mosses	Grimmiaceae	*Grimmia*	*Grimmia ovalis*	GU808924
Mosses	Grimmiaceae	*Grimmia*	*Grimmia unicolor*	GU808946
Mosses	Grimmiaceae	*Grimmia*	*Grimmia unicolor*	GU808944
Mosses	Ptychomniaceae	*Ptychomnion*	*Ptychomnion densifolium*	EU125864
Mosses	Ptychomniaceae	*Ptychomnion*	*Ptychomnion densifolium*	EU125865
Mosses	Ptychomniaceae	*Ptychomnion*	*Ptychomnion densifolium*	EU125855
Mosses	Ptychomniaceae	*Ptychomnion*	*Ptychomnion subaciculare*	EU125858
Mosses	Ptychomniaceae	*Ptychomnion*	*Ptychomnion subaciculare*	EU125857
Mosses	Grimmiaceae	*Schistidium*	*Schistidium trichodon*	HM989829
Mosses	Grimmiaceae	*Schistidium*	*Schistidium trichodon*	HM989827
Mosses	Orthotrichaceae	*Orthotrichum*	*Orthotrichum sordidum*	FJ036871
Mosses	Orthotrichaceae	*Orthotrichum*	*Orthotrichum sordidum*	FJ036870
Mosses	Mniaceae	*Plagiomnium*	*Plagiomnium drummondii*	EU750669
Mosses	Mniaceae	*Plagiomnium*	*Plagiomnium drummondii*	EU750670
Mosses	Dicranaceae	*Dicranum*	*Dicranum flagellare*	GQ248288
Mosses	Dicranaceae	*Dicranum*	*Dicranum flagellare*	EU750663
Mosses	Dicranaceae	*Dicranum*	*Dicranum flagellare*	EU750664
Mosses	Dicranaceae	*Dicranum*	*Dicranum flagellare*	EU750662
Mosses	Dicranaceae	*Dicranum*	*Dicranum flagellare*	EF590689
Mosses	Hypnaceae	*Hypnum*	*Hypnum jutlandicum*	GQ849575
Mosses	Hypnaceae	*Hypnum*	*Hypnum jutlandicum*	GQ849574
Mosses	Orthotrichaceae	*Orthotrichum*	*Orthotrichum fastigiatum*	FJ036883
Mosses	Orthotrichaceae	*Orthotrichum*	*Orthotrichum fastigiatum*	FJ036881
Mosses	Orthotrichaceae	*Orthotrichum*	*Orthotrichum fastigiatum*	FJ036884
Mosses	Orthotrichaceae	*Orthotrichum*	*Orthotrichum fastigiatum*	FJ036882
Mosses	Orthotrichaceae	*Orthotrichum*	*Orthotrichum fastigiatum*	FJ036880
Mosses	Orthotrichaceae	*Nyholmiella*	*Nyholmiella gymnostoma*	GQ370355
Mosses	Orthotrichaceae	*Nyholmiella*	*Nyholmiella gymnostoma*	GQ370353
Mosses	Orthotrichaceae	*Nyholmiella*	*Nyholmiella gymnostoma*	GQ370354
Mosses	Orthotrichaceae	*Nyholmiella*	*Nyholmiella gymnostoma*	GQ370352
Mosses	Orthotrichaceae	*Orthotrichum*	*Orthotrichum rupestre*	GQ428030
Mosses	Orthotrichaceae	*Orthotrichum*	*Orthotrichum rupestre*	GQ370346
Mosses	Orthotrichaceae	*Orthotrichum*	*Orthotrichum striatum*	FJ036887
Mosses	Orthotrichaceae	*Orthotrichum*	*Orthotrichum striatum*	FJ036885
Mosses	Orthotrichaceae	*Orthotrichum*	*Orthotrichum striatum*	FJ036886
Mosses	Hypnaceae	*Hypnum*	*Hypnum andoi*	EU163585
Mosses	Hypnaceae	*Hypnum*	*Hypnum andoi*	GQ849567
Mosses	Orthotrichaceae	*Orthotrichum*	*Orthotrichum pylaisii*	FJ036869
Mosses	Orthotrichaceae	*Orthotrichum*	*Orthotrichum pylaisii*	FJ036868
Mosses	Polytrichaceae	*Polytrichum*	*Polytrichum sphaerothecium*	FJ572557
Mosses	Polytrichaceae	*Polytrichum*	*Polytrichum sphaerothecium*	FJ572556
Mosses	Grimmiaceae	*Niphotrichum*	*Niphotrichum ericoides*	GU808907
Mosses	Grimmiaceae	*Niphotrichum*	*Niphotrichum ericoides*	GU808905
Mosses	Grimmiaceae	*Niphotrichum*	*Niphotrichum ericoides*	GU808906
Mosses	Grimmiaceae	*Niphotrichum*	*Niphotrichum ericoides*	FJ572565
Mosses	Grimmiaceae	*Niphotrichum*	*Niphotrichum ericoides*	FJ572566

Mosses	Grimmiaceae	*Niphotrichum*	*Niphotrichum ericoides*	FJ572564
Mosses	Grimmiaceae	*Grimmia*	*Grimmia obtusifolia*	GU808941
Mosses	Grimmiaceae	*Grimmia*	*Grimmia obtusifolia*	GU808939
Mosses	Grimmiaceae	*Grimmia*	*Grimmia obtusifolia*	GU808940
Mosses	Grimmiaceae	*Codriophorus*	*Codriophorus anomodontoides*	GU808895
Mosses	Grimmiaceae	*Codriophorus*	*Codriophorus anomodontoides*	GU808893
Mosses	Grimmiaceae	*Codriophorus*	*Codriophorus anomodontoides*	GU808891
Mosses	Grimmiaceae	*Codriophorus*	*Codriophorus anomodontoides*	GU808896
Mosses	Grimmiaceae	*Codriophorus*	*Codriophorus anomodontoides*	GU808894
Mosses	Grimmiaceae	*Codriophorus*	*Codriophorus anomodontoides*	GU808892
Mosses	Grimmiaceae	*Niphotrichum*	*Niphotrichum barbuloides*	GU808903
Mosses	Grimmiaceae	*Niphotrichum*	*Niphotrichum barbuloides*	GU808904
Mosses	Grimmiaceae	*Bucklandiella*	*Bucklandiella himalayana*	GU808883
Mosses	Grimmiaceae	*Bucklandiella*	*Bucklandiella himalayana*	GU808881
Mosses	Grimmiaceae	*Bucklandiella*	*Bucklandiella himalayana*	GU808882
Mosses	Grimmiaceae	*Bucklandiella*	*Bucklandiella subsecunda*	GU808885
Mosses	Grimmiaceae	*Bucklandiella*	*Bucklandiella subsecunda*	GU808886
Mosses	Grimmiaceae	*Bucklandiella*	*Bucklandiella subsecunda*	GU808884
Mosses	Hypnaceae	*Hypnum*	*Hypnum cupressiforme var. lacunosum*	GQ849571
Mosses	Hypnaceae	*Hypnum*	*Hypnum cupressiforme var. lacunosum*	GQ849572
