# Supplementary material for: Utility of the trnH–psbA Intergenic Spacer Region and Its Combinations as Plant DNA Barcodes: A Meta-Analysis
Source: PLoS One. 2012 Nov 14;7(11):e48833. doi: 10.1371/journal.pone.0048833 (PMC3498263; doi:10.1371/journal.pone.0048833)
Supplement: Table S4 — List of trnH – psbA , ITS2, rbcL , and matK samples with the same voucher numbers. The names of the corresponding group, family, genus, and species, the voucher number, as well as the GenBank accession number for each sample are shown. (PDF) [file pone.0048833.s004.pdf]

Table S4. List of *trnH–psbA* , ITS2, *rbcL* , and *matK* samples with the same voucher numbers. The names of the corresponding group, family, genus, and species, the voucher number, as well as the GenBank accession number for each sample are shown.

					GenBank accesstion no.			
**Taxonomy**	**Family**	**Genus**	**Species**	**Voucher no.**	**ITS2**	***trnH-***	***rbcL***	***matK***
Eudicotyledons	Zygophyllaceae	*Zygophyllum*	*Zygophyllum rosowii*	H62	JF979032	JN047542	JF944811	JF956823
Eudicotyledons	Zygophyllaceae	*Zygophyllum*	*Zygophyllum rosowii*	D1507	JF979033	JN047543	JF944812	JF956824
Eudicotyledons	Zygophyllaceae	*Zygophyllum*	*Zygophyllum obliquum*	H68	JF979030	JN047538	JF944807	JF956821
Eudicotyledons	Zygophyllaceae	*Zygophyllum*	*Zygophyllum obliquum*	H67	JF979031	JN047539	JF944808	JF956822
Eudicotyledons	Zygophyllaceae	*Zygophyllum*	*Zygophyllum macropodum*	D1553	JF979028	JN047536	JF944805	JF956819
Eudicotyledons	Zygophyllaceae	*Zygophyllum*	*Zygophyllum macropodum*	D1508	JF979029	JN047537	JF944806	JF956820
Eudicotyledons	Zygophyllaceae	*Zygophyllum*	*Zygophyllum fabago*	H74	JF979025	JN047533	JF944801	JF956815
Eudicotyledons	Zygophyllaceae	*Zygophyllum*	*Zygophyllum fabago*	D485	JF979027	JN047534	JF944803	JF956817
Eudicotyledons	Zygophyllaceae	*Zygophyllum*	*Zygophyllum brachypterum*	H65	JF979024	JN047532	JF944800	JF956814
Eudicotyledons	Zygophyllaceae	*Zygophyllum*	*Zygophyllum brachypterum*	H66	JF979023	JN047531	JF944799	JF956813
Eudicotyledons	Adoxaceae	*Viburnum*	*Viburnum utile*	Egolf_2336-E	AY265156	AY62742	HQ59177	HQ59163
Eudicotyledons	Adoxaceae	*Viburnum*	*Viburnum utile*	Z348	JF979017	JN047526	JF944793	JF956807
Eudicotyledons	Adoxaceae	*Viburnum*	*Viburnum utile*	Z347	JF979018	JN047527	JF944794	JF956808
Eudicotyledons	Adoxaceae	*Viburnum*	*Viburnum foetidum*	Z333	JF979008	JN047509	JF944776	JF956790
Eudicotyledons	Adoxaceae	*Viburnum*	*Viburnum foetidum*	Z330	JF979009	JN047512	JF944779	JF956793
Eudicotyledons	Adoxaceae	*Viburnum*	*Viburnum foetidum*	C.-H._Lin_563	HQ591963	HQ592075	HQ59172	HQ59158
Eudicotyledons	Adoxaceae	*Viburnum*	*Viburnum foetidum*	D1233	JF979010	JN047513	JF944780	JF956794
Eudicotyledons	Adoxaceae	*Viburnum*	*Viburnum erosum*	Z328	JF979006	JN047503	JF944770	JF956784
Eudicotyledons	Adoxaceae	*Viburnum*	*Viburnum erosum*	Z326	JF979007	JN047505	JF944772	JF956786
Eudicotyledons	Adoxaceae	*Viburnum*	*Viburnum dilatatum*	Z323	JF979003	JN047497	JF944764	JF956778
Eudicotyledons	Adoxaceae	*Viburnum*	*Viburnum dilatatum*	Z322	JF979004	JN047498	JF944765	JF956779
Eudicotyledons	Adoxaceae	*Viburnum*	*Viburnum dilatatum*	Z319	JF979005	JN047501	JF944768	JF956782
Eudicotyledons	Adoxaceae	*Viburnum*	*Viburnum cylindricum*	Z318	JF978999	JN047490	JF944757	JF956772
Eudicotyledons	Adoxaceae	*Viburnum*	*Viburnum cylindricum*	Z314	JF979002	JN047494	JF944761	JF956776
Eudicotyledons	Adoxaceae	*Viburnum*	*Viburnum cylindricum*	Z316	JF979001	JN047492	JF944759	JF956774
Eudicotyledons	Adoxaceae	*Viburnum*	*Viburnum cylindricum*	Z317	JF979000	JN047491	JF944758	JF956773
Eudicotyledons	Adoxaceae	*Viburnum*	*Viburnum betulifolium*	D940	JF978997	JN047487	JF944754	JF956769
Eudicotyledons	Adoxaceae	*Viburnum*	*Viburnum betulifolium*	D1266	JF978998	JN047489	JF944756	JF956771
Eudicotyledons	Adoxaceae	*Viburnum*	*Viburnum betulifolium*	Z312	JF978996	JN047485	JF944752	JF956767
Eudicotyledons	Adoxaceae	*Viburnum*	*Viburnum betulifolium*	Z313	JF978995	JN047484	JF944751	JF956766
Eudicotyledons	Adoxaceae	*Viburnum*	*Viburnum awabuki*	Liu_141	HQ591951	HQ592060	HQ59170	HQ59156
Eudicotyledons	Adoxaceae	*Viburnum*	*Viburnum awabuki*	Z345	JF979014	JN047523	JF944790	JF956804
Eudicotyledons	Adoxaceae	*Viburnum*	*Viburnum awabuki*	Z344	JF979015	JN047524	JF944791	JF956805
Eudicotyledons	Adoxaceae	*Viburnum*	*Viburnum awabuki*	Z343	JF979016	JN047525	JF944792	JF956806
Eudicotyledons	Cucurbitaceae	*Thladiantha*	*Thladiantha villosula*	LiHT10179	JF978974	JN047449	JF944711	JF956741
Eudicotyledons	Cucurbitaceae	*Thladiantha*	*Thladiantha villosula*	LiHT10173	JF978975	JN047450	JF944712	JF956742

Eudicotyledons	Cucurbitaceae	*Thladiantha*	*Thladiantha villosula*	LiHT214	JF978970	JN047445	JF944707	JF956737
Eudicotyledons	Cucurbitaceae	*Thladiantha*	*Thladiantha villosula*	LiHT203	JF978971	JN047446	JF944708	JF956738
Eudicotyledons	Cucurbitaceae	*Thladiantha*	*Thladiantha villosula*	LiHT10180	JF978973	JN047448	JF944710	JF956740
Eudicotyledons	Cucurbitaceae	*Thladiantha*	*Thladiantha villosula*	LiHT10181	JF978972	JN047447	JF944709	JF956739
Eudicotyledons	Cucurbitaceae	*Thladiantha*	*Thladiantha oliveri*	LiHT151	JF978966	JN047441	JF944703	JF956733
Eudicotyledons	Cucurbitaceae	*Thladiantha*	*Thladiantha oliveri*	LiHT174	JF978965	JN047440	JF944702	JF956732
Eudicotyledons	Cucurbitaceae	*Thladiantha*	*Thladiantha oliveri*	LiHT14	JF978967	JN047442	JF944704	JF956734
Eudicotyledons	Cucurbitaceae	*Thladiantha*	*Thladiantha oliveri*	LiHT080200	JF978969	JN047444	JF944706	JF956736
Eudicotyledons	Cucurbitaceae	*Thladiantha*	*Thladiantha oliveri*	LiHT225	JF978964	JN047439	JF944701	JF956731
Eudicotyledons	Cucurbitaceae	*Thladiantha*	*Thladiantha oliveri*	LiHT1031	JF978968	JN047443	JF944705	JF956735
Eudicotyledons	Cucurbitaceae	*Thladiantha*	*Thladiantha nudiflora*	LiHT1036	JF978958	JN047433	JF944695	JF956725
Eudicotyledons	Cucurbitaceae	*Thladiantha*	*Thladiantha nudiflora*	LiHT1013	JF978962	JN047437	JF944699	JF956729
Eudicotyledons	Cucurbitaceae	*Thladiantha*	*Thladiantha nudiflora*	LiHT1062	JF978955	JN047430	JF944692	JF956722
Eudicotyledons	Cucurbitaceae	*Thladiantha*	*Thladiantha nudiflora*	LiHT1047	JF978956	JN047431	JF944693	JF956723
Eudicotyledons	Cucurbitaceae	*Thladiantha*	*Thladiantha nudiflora*	LiHT080199	JF978963	JN047438	JF944700	JF956730
Eudicotyledons	Cucurbitaceae	*Thladiantha*	*Thladiantha nudiflora*	LiHT1020	JF978961	JN047436	JF944698	JF956728
Eudicotyledons	Cucurbitaceae	*Thladiantha*	*Thladiantha nudiflora*	LiHT1021	JF978960	JN047435	JF944697	JF956727
Eudicotyledons	Cucurbitaceae	*Thladiantha*	*Thladiantha nudiflora*	LiHT1024	JF978959	JN047434	JF944696	JF956726
Eudicotyledons	Cucurbitaceae	*Thladiantha*	*Thladiantha nudiflora*	LiHT1037	JF978957	JN047432	JF944694	JF956724
Eudicotyledons	Cucurbitaceae	*Thladiantha*	*Thladiantha nudiflora*	LiHT297	JF978954	JN047429	JF944691	JF956721
Eudicotyledons	Cucurbitaceae	*Thladiantha*	*Thladiantha montana*	LiHT10182	JF978952	JN047427	JF944689	JF956719
Eudicotyledons	Cucurbitaceae	*Thladiantha*	*Thladiantha montana*	LiHT10183	JF978951	JN047426	JF944688	JF956718
Eudicotyledons	Cucurbitaceae	*Thladiantha*	*Thladiantha montana*	LiHT10186	JF978950	JN047425	JF944687	JF956717
Eudicotyledons	Cucurbitaceae	*Thladiantha*	*Thladiantha montana*	LiHT10187	JF978949	JN047424	JF944686	JF956716
Eudicotyledons	Cucurbitaceae	*Thladiantha*	*Thladiantha montana*	LiHT092404	JF978953	JN047428	JF944690	JF956720
Eudicotyledons	Cucurbitaceae	*Thladiantha*	*Thladiantha maculata*	LiHT307	JF978945	JN047420	JF944682	JF956712
Eudicotyledons	Cucurbitaceae	*Thladiantha*	*Thladiantha maculata*	LiHT314	JF978944	JN047419	JF944681	JF956711
Eudicotyledons	Cucurbitaceae	*Thladiantha*	*Thladiantha maculata*	LiHT315	JF978943	JN047418	JF944680	JF956710
Eudicotyledons	Cucurbitaceae	*Thladiantha*	*Thladiantha maculata*	LiHT298	JF978948	JN047423	JF944685	JF956715
Eudicotyledons	Cucurbitaceae	*Thladiantha*	*Thladiantha maculata*	LiHT305	JF978947	JN047422	JF944684	JF956714
Eudicotyledons	Cucurbitaceae	*Thladiantha*	*Thladiantha maculata*	LiHT306	JF978946	JN047421	JF944683	JF956713
Eudicotyledons	Cucurbitaceae	*Thladiantha*	*Thladiantha longisepala*	LiHT218	JF978941	JN047416	JF944678	JF956708
Eudicotyledons	Cucurbitaceae	*Thladiantha*	*Thladiantha longisepala*	LiHT1038	JF978942	JN047417	JF944679	JF956709
Eudicotyledons	Cucurbitaceae	*Thladiantha*	*Thladiantha longisepala*	LiHT224	JF978940	JN047415	JF944677	JF956707
Eudicotyledons	Cucurbitaceae	*Thladiantha*	*Thladiantha lijiangensis*	LiHT10178	JF978938	JN047413	JF944675	JF956705
Eudicotyledons	Cucurbitaceae	*Thladiantha*	*Thladiantha lijiangensis*	LiHT10176	JF978939	JN047414	JF944676	JF956706
Eudicotyledons	Cucurbitaceae	*Thladiantha*	*Thladiantha lijiangensis*	LiHT10174	JF978937	JN047412	JF944674	JF956704
Eudicotyledons	Cucurbitaceae	*Thladiantha*	*Thladiantha lijiangensis*	LiHT212	JF978933	JN047408	JF944670	JF956700
Eudicotyledons	Cucurbitaceae	*Thladiantha*	*Thladiantha lijiangensis*	LiHT209	JF978934	JN047409	JF944671	JF956701
Eudicotyledons	Cucurbitaceae	*Thladiantha*	*Thladiantha lijiangensis*	LiHT194	JF978935	JN047410	JF944672	JF956702

Eudicotyledons	Cucurbitaceae	*Thladiantha*	*Thladiantha lijiangensis*	LiHT10175	JF978936	JN047411	JF944673	JF956703
Eudicotyledons	Cucurbitaceae	*Thladiantha*	*Thladiantha hookeri*	LiHT10145	JF978931	JN047406	JF944668	JF956698
Eudicotyledons	Cucurbitaceae	*Thladiantha*	*Thladiantha hookeri*	LiHT10148	JF978930	JN047405	JF944667	JF956697
Eudicotyledons	Cucurbitaceae	*Thladiantha*	*Thladiantha hookeri*	LiHT10192	JF978929	JN047404	JF944666	JF956696
Eudicotyledons	Cucurbitaceae	*Thladiantha*	*Thladiantha hookeri*	LiHT092568	JF978932	JN047407	JF944669	JF956699
Eudicotyledons	Cucurbitaceae	*Thladiantha*	*Thladiantha hookeri*	LiHT10194	JF978928	JN047403	JF944665	JF956695
Eudicotyledons	Cucurbitaceae	*Thladiantha*	*Thladiantha henryi*	LiHT1053-3	JF978925	JN047400	JF944662	JF956692
Eudicotyledons	Cucurbitaceae	*Thladiantha*	*Thladiantha henryi*	LiHT1053-2	JF978926	JN047401	JF944663	JF956693
Eudicotyledons	Cucurbitaceae	*Thladiantha*	*Thladiantha henryi*	LiHT1053-1	JF978927	JN047402	JF944664	JF956694
Eudicotyledons	Cucurbitaceae	*Thladiantha*	*Thladiantha grandisepala*	LiHT043	JF978923	JN047398	JF944660	JF956690
Eudicotyledons	Cucurbitaceae	*Thladiantha*	*Thladiantha grandisepala*	LiHT10142-3	JF978920	JN047395	JF944657	JF956687
Eudicotyledons	Cucurbitaceae	*Thladiantha*	*Thladiantha grandisepala*	LiHT10142-2	JF978921	JN047396	JF944658	JF956688
Eudicotyledons	Cucurbitaceae	*Thladiantha*	*Thladiantha grandisepala*	LiHT10142-1	JF978922	JN047397	JF944659	JF956689
Eudicotyledons	Cucurbitaceae	*Thladiantha*	*Thladiantha grandisepala*	LiHT10147	JF978919	JN047394	JF944656	JF956686
Eudicotyledons	Cucurbitaceae	*Thladiantha*	*Thladiantha grandisepala*	LiHT10160	JF978918	JN047393	JF944655	JF956685
Eudicotyledons	Cucurbitaceae	*Thladiantha*	*Thladiantha grandisepala*	LiHT10163	JF978917	JN047392	JF944654	JF956684
Eudicotyledons	Cucurbitaceae	*Thladiantha*	*Thladiantha grandisepala*	LiHT03	JF978924	JN047399	JF944661	JF956691
Eudicotyledons	Cucurbitaceae	*Thladiantha*	*Thladiantha dentata*	LiHT10195	JF978913	JN047388	JF944650	JF956680
Eudicotyledons	Cucurbitaceae	*Thladiantha*	*Thladiantha dentata*	LiHT10191	JF978915	JN047390	JF944652	JF956682
Eudicotyledons	Cucurbitaceae	*Thladiantha*	*Thladiantha dentata*	LiHT10193	JF978914	JN047389	JF944651	JF956681
Eudicotyledons	Cucurbitaceae	*Thladiantha*	*Thladiantha dentata*	LiHT10188	JF978916	JN047391	JF944653	JF956683
Eudicotyledons	Cucurbitaceae	*Thladiantha*	*Thladiantha davidii*	LiHT1019	JF978912	JN047387	JF944649	JF956679
Eudicotyledons	Cucurbitaceae	*Thladiantha*	*Thladiantha davidii*	LiHT1022	JF978911	JN047386	JF944648	JF956678
Eudicotyledons	Cucurbitaceae	*Thladiantha*	*Thladiantha davidii*	LiHT1023	JF978910	JN047385	JF944647	JF956677
Eudicotyledons	Cucurbitaceae	*Thladiantha*	*Thladiantha davidii*	LiHT1026	JF978909	JN047384	JF944646	JF956676
Eudicotyledons	Cucurbitaceae	*Thladiantha*	*Thladiantha cordifolia*	LiHT10137	JF978903	JN047378	JF944640	JF956670
Eudicotyledons	Cucurbitaceae	*Thladiantha*	*Thladiantha cordifolia*	LiHT081041	JF978908	JN047383	JF944645	JF956675
Eudicotyledons	Cucurbitaceae	*Thladiantha*	*Thladiantha cordifolia*	LiHT1010	JF978907	JN047382	JF944644	JF956674
Eudicotyledons	Cucurbitaceae	*Thladiantha*	*Thladiantha cordifolia*	LiHT1016	JF978902	JN047377	JF944639	JF956669
Eudicotyledons	Cucurbitaceae	*Thladiantha*	*Thladiantha cordifolia*	LiHT1056	JF978901	JN047376	JF944638	JF956668
Eudicotyledons	Cucurbitaceae	*Thladiantha*	*Thladiantha cordifolia*	LiHT1087	JF978899	JN047374	JF944636	JF956666
Eudicotyledons	Cucurbitaceae	*Thladiantha*	*Thladiantha cordifolia*	LiHT1083	JF978900	JN047375	JF944637	JF956667
Eudicotyledons	Cucurbitaceae	*Thladiantha*	*Thladiantha cordifolia*	LiHT1095	JF978897	JN047372	JF944634	JF956664
Eudicotyledons	Cucurbitaceae	*Thladiantha*	*Thladiantha cordifolia*	LiHT1094	JF978898	JN047373	JF944635	JF956665
Eudicotyledons	Cucurbitaceae	*Thladiantha*	*Thladiantha cordifolia*	LiHT10119	JF978905	JN047380	JF944642	JF956672
Eudicotyledons	Cucurbitaceae	*Thladiantha*	*Thladiantha cordifolia*	LiHT10104	JF978906	JN047381	JF944643	JF956673
Eudicotyledons	Cucurbitaceae	*Thladiantha*	*Thladiantha cordifolia*	LiHT10125	JF978904	JN047379	JF944641	JF956671
Eudicotyledons	Cucurbitaceae	*Thladiantha*	*Thladiantha capitata*	LiHT1050	JF978896	JN047371	JF944633	JF956663
Eudicotyledons	Cucurbitaceae	*Thladiantha*	*Thladiantha capitata*	LiHT204	JF978892	JN047367	JF944629	JF956659
Eudicotyledons	Cucurbitaceae	*Thladiantha*	*Thladiantha capitata*	LiHT1051-3	JF978893	JN047368	JF944630	JF956660

Eudicotyledons	Cucurbitaceae	*Thladiantha*	*Thladiantha capitata*	LiHT1051-1	JF978895	JN047370	JF944632	JF956662
Eudicotyledons	Cucurbitaceae	*Thladiantha*	*Thladiantha capitata*	LiHT1051-2	JF978894	JN047369	JF944631	JF956661
Eudicotyledons	Asteraceae	*Syncalathium*	*Syncalathium kawaguchii*	BouffordDE-40822	JF978845	JN047311	JF944566	JF956598
Eudicotyledons	Asteraceae	*Syncalathium*	*Syncalathium kawaguchii*	ZhangJW-093	JF978843	JN047309	JF944564	JF956596
Eudicotyledons	Asteraceae	*Syncalathium*	*Syncalathium kawaguchii*	NieZL-903	JF978844	JN047310	JF944565	JF956597
Eudicotyledons	Asteraceae	*Syncalathium*	*Syncalathium disciforme*	ZhangJW-1010	JF978840	JN047306	JF944558	JF956590
Eudicotyledons	Asteraceae	*Syncalathium*	*Syncalathium disciforme*	BouffordDE-39737	JF978842	JN047308	JF944560	JF956592
Eudicotyledons	Asteraceae	*Syncalathium*	*Syncalathium disciforme*	BouffordDE-40137	JF978841	JN047307	JF944559	JF956591
Eudicotyledons	Asteraceae	*Syncalathium*	*Syncalathium chrysocephalum*	BouffordDE-41025	JF978838	JN047304	JF944555	JF956587
Eudicotyledons	Asteraceae	*Syncalathium*	*Syncalathium chrysocephalum*	BouffordDE-41418	JF978837	JN047303	JF944554	JF956586
Eudicotyledons	Amaranthaceae	*Suaeda*	*Suaeda glauca*	Z460	JF978817	JN047267	JF944515	JF956553
Eudicotyledons	Amaranthaceae	*Suaeda*	*Suaeda glauca*	Z459	JF978818	JN047268	JF944516	JF956554
Eudicotyledons	Amaranthaceae	*Suaeda*	*Suaeda glauca*	Z461	JF978816	JN047266	JF944514	JF956552
Eudicotyledons	Amaranthaceae	*Suaeda*	*Suaeda corniculata*	D1321	JF978815	JN047265	JF944513	JF956551
Eudicotyledons	Amaranthaceae	*Suaeda*	*Suaeda corniculata*	D1338	JF978814	JN047264	JF944512	JF956550
Eudicotyledons	Stachyuraceae	*Stachyurus*	*Stachyurus himalaicus*	H14	JF978810	JN047255	JF944504	JF956541
Eudicotyledons	Stachyuraceae	*Stachyurus*	*Stachyurus himalaicus*	H12	JF978811	JN047257	JF944506	JF956543
Eudicotyledons	Stachyuraceae	*Stachyurus*	*Stachyurus chinensis*	C650	JF978809	JN047254	JF944503	JF956540
Eudicotyledons	Stachyuraceae	*Stachyurus*	*Stachyurus chinensis*	H8	JF978807	JN047251	JF944500	JF956537
Eudicotyledons	Stachyuraceae	*Stachyurus*	*Stachyurus chinensis*	H7	JF978808	JN047252	JF944501	JF956538
Eudicotyledons	Asteraceae	*Soroseris*	*Soroseris hirsuta*	BouffordDE-41585	JF978806	JN047250	JF944491	JF956530
Eudicotyledons	Asteraceae	*Soroseris*	*Soroseris hirsuta*	SCSB-W1036	JF978805	JN047249	JF944490	JF956529
Eudicotyledons	Asteraceae	*Soroseris*	*Soroseris glomerata*	ZhangJW-033	JF978802	JN047247	JF944484	JF956523
Eudicotyledons	Asteraceae	*Soroseris*	*Soroseris glomerata*	SunH-ZX0904	JF978804	JN047248	JF944486	JF956525
Eudicotyledons	Asteraceae	*Soroseris*	*Soroseris erysimoides*	ZhangJW-1009	JF978799	JN047243	JF944477	JF956515
Eudicotyledons	Asteraceae	*Soroseris*	*Soroseris erysimoides*	BouffordDE-41027	JF978800	JN047244	JF944478	JF956516
Eudicotyledons	Solanaceae	*Solanum*	*Solanum spirale*	H40	JF978798	JN047229	JF944463	JF956501
Eudicotyledons	Solanaceae	*Solanum*	*Solanum spirale*	H41	JF978797	JN047228	JF944462	JF956500
Eudicotyledons	Solanaceae	*Solanum*	*Solanum nigrum*	H37	JF978794	JN047222	JF944456	JF956494
Eudicotyledons	Solanaceae	*Solanum*	*Solanum nigrum*	H35	JF978795	JN047224	JF944458	JF956496
Eudicotyledons	Solanaceae	*Solanum*	*Solanum nigrum*	H38	JF978793	JN047221	JF944455	JF956493
Eudicotyledons	Solanaceae	*Solanum*	*Solanum nigrum*	H39	JF978792	JN047220	JF944454	JF956492
Eudicotyledons	Solanaceae	*Solanum*	*Solanum aculeatissimum*	H47	JF978787	JN047208	JF944441	JF956479
Eudicotyledons	Solanaceae	*Solanum*	*Solanum aculeatissimum*	H46	JF978788	JN047209	JF944442	JF956480
Eudicotyledons	Solanaceae	*Solanum*	*Solanum aculeatissimum*	H49	JF978790	JN047211	JF944444	JF956482
Eudicotyledons	Solanaceae	*Solanum*	*Solanum aculeatissimum*	H48	JF978791	JN047212	JF944445	JF956483
Eudicotyledons	Solanaceae	*Solanum*	*Solanum aculeatissimum*	H50	JF978789	JN047210	JF944443	JF956481
Eudicotyledons	Asteraceae	*Sinosenecio*	*Sinosenecio yilingii*	Yangqe0128	JF978636	JN047201	JF944297	JF956330

Eudicotyledons	Asteraceae	*Sinosenecio*	*Sinosenecio yilingii*	Yangqe0126	JF978638	JN047203	JF944299	JF956332
Eudicotyledons	Asteraceae	*Sinosenecio*	*Sinosenecio yilingii*	Yangqe0127	JF978637	JN047202	JF944298	JF956331
Eudicotyledons	Asteraceae	*Sinosenecio*	*Sinosenecio villifer*	Yangqe0137	JF978635	JN047200	JF944296	JF956329
Eudicotyledons	Asteraceae	*Sinosenecio*	*Sinosenecio villifer*	Yangqe0138	JF978634	JN047199	JF944295	JF956328
Eudicotyledons	Asteraceae	*Sinosenecio*	*Sinosenecio sungpanensis*	Yangqe0076	JF978632	JN047196	JF944292	JF956325
Eudicotyledons	Asteraceae	*Sinosenecio*	*Sinosenecio sungpanensis*	Yangqe0075	JF978633	JN047197	JF944293	JF956326
Eudicotyledons	Asteraceae	*Sinosenecio*	*Sinosenecio subcoriaceus*	Yangqe0112	JF978631	JN047193	JF944289	JF956321
Eudicotyledons	Asteraceae	*Sinosenecio*	*Sinosenecio subcoriaceus*	Yangqe0115	JF978628	JN047191	JF944286	JF956318
Eudicotyledons	Asteraceae	*Sinosenecio*	*Sinosenecio subcoriaceus*	Yangqe0114	JF978629	JN047192	JF944287	JF956319
Eudicotyledons	Asteraceae	*Sinosenecio*	*Sinosenecio subcoriaceus*	Yangqe0116	JF978627	JN047190	JF944285	JF956317
Eudicotyledons	Asteraceae	*Sinosenecio*	*Sinosenecio sp. DZL-2011*	Yangqe0152	JF978569	JN047126	JF944218	JF956252
Eudicotyledons	Asteraceae	*Sinosenecio*	*Sinosenecio sp. DZL-2011*	Yangqe0151	JF978570	JN047127	JF944219	JF956253
Eudicotyledons	Asteraceae	*Sinosenecio*	*Sinosenecio sp. DZL-2011*	Yangqe0150	JF978571	JN047128	JF944220	JF956254
Eudicotyledons	Asteraceae	*Sinosenecio*	*Sinosenecio sichuanicus*	Yangqe0157	JF978625	JN047188	JF944283	JF956315
Eudicotyledons	Asteraceae	*Sinosenecio*	*Sinosenecio sichuanicus*	Yangqe0156	JF978626	JN047189	JF944284	JF956316
Eudicotyledons	Asteraceae	*Sinosenecio*	*Sinosenecio septilobus*	Yangqe0070	JF978623	JN047186	JF944281	JF956313
Eudicotyledons	Asteraceae	*Sinosenecio*	*Sinosenecio septilobus*	Yangqe0071	JF978622	JN047185	JF944280	JF956312
Eudicotyledons	Asteraceae	*Sinosenecio*	*Sinosenecio septilobus*	Yangqe0069	JF978624	JN047187	JF944282	JF956314
Eudicotyledons	Asteraceae	*Sinosenecio*	*Sinosenecio palmatisectus*	Yangqe0087	JF978619	JN047180	JF944277	JF956309
Eudicotyledons	Asteraceae	*Sinosenecio*	*Sinosenecio palmatisectus*	Yangqe0086	JF978620	JN047181	JF944278	JF956310
Eudicotyledons	Asteraceae	*Sinosenecio*	*Sinosenecio oldhamianus*	Yangqe0083	JF978616	JN047177	JF944274	JF956306
Eudicotyledons	Asteraceae	*Sinosenecio*	*Sinosenecio oldhamianus*	Yangqe0082	JF978617	JN047178	JF944275	JF956307
Eudicotyledons	Asteraceae	*Sinosenecio*	*Sinosenecio oldhamianus*	Yangqe0081	JF978618	JN047179	JF944276	JF956308
Eudicotyledons	Asteraceae	*Sinosenecio*	*Sinosenecio ligularioides*	Yangqe0124	JF978614	JN047175	JF944272	JF956304
Eudicotyledons	Asteraceae	*Sinosenecio*	*Sinosenecio ligularioides*	Yangqe0125	JF978613	JN047174	JF944271	JF956303
Eudicotyledons	Asteraceae	*Sinosenecio*	*Sinosenecio ligularioides*	Yangqe0123	JF978615	JN047176	JF944273	JF956305
Eudicotyledons	Asteraceae	*Sinosenecio*	*Sinosenecio latouchei*	Yangqe0080	JF978609	JN047170	JF944267	JF956299
Eudicotyledons	Asteraceae	*Sinosenecio*	*Sinosenecio latouchei*	Yangqe0077	JF978612	JN047173	JF944270	JF956302
Eudicotyledons	Asteraceae	*Sinosenecio*	*Sinosenecio latouchei*	Yangqe0078	JF978611	JN047172	JF944269	JF956301
Eudicotyledons	Asteraceae	*Sinosenecio*	*Sinosenecio latouchei*	Yangqe0079	JF978610	JN047171	JF944268	JF956300
Eudicotyledons	Asteraceae	*Sinosenecio*	*Sinosenecio koreanus*	Yangqe0153	JF978608	JN047169	JF944266	JF956298
Eudicotyledons	Asteraceae	*Sinosenecio*	*Sinosenecio koreanus*	Yangqe0155	JF978607	JN047167	JF944265	JF956296
Eudicotyledons	Asteraceae	*Sinosenecio*	*Sinosenecio jiuhuashanicus*	Yangqe0074	JF978604	JN047164	JF944262	JF956293
Eudicotyledons	Asteraceae	*Sinosenecio*	*Sinosenecio jiuhuashanicus*	Yangqe0072	JF978606	JN047166	JF944264	JF956295
Eudicotyledons	Asteraceae	*Sinosenecio*	*Sinosenecio jiuhuashanicus*	Yangqe0073	JF978605	JN047165	JF944263	JF956294
Eudicotyledons	Asteraceae	*Sinosenecio*	*Sinosenecio homogyniphyllus*	Yangqe0132	JF978601	JN047161	JF944259	JF956290
Eudicotyledons	Asteraceae	*Sinosenecio*	*Sinosenecio homogyniphyllus*	Yangqe0130	JF978602	JN047162	JF944260	JF956291

Eudicotyledons	Asteraceae	*Sinosenecio*	*Sinosenecio homogyniphyllus*	Yangqe0129	JF978603	JN047163	JF944261	JF956292
Eudicotyledons	Asteraceae	*Sinosenecio*	*Sinosenecio homogyniphyllus*	Yangqe0136	JF978600	JN047159	JF944256	JF956287
Eudicotyledons	Asteraceae	*Sinosenecio*	*Sinosenecio guangxiensis*	Yangqe0102	JF978599	JN047158	JF944255	JF956286
Eudicotyledons	Asteraceae	*Sinosenecio*	*Sinosenecio guangxiensis*	Yangqe0104	JF978597	JN047156	JF944253	JF956285
Eudicotyledons	Asteraceae	*Sinosenecio*	*Sinosenecio globiger*	Yangqe0096	JF978592	JN047151	JF944247	JF956279
Eudicotyledons	Asteraceae	*Sinosenecio*	*Sinosenecio globiger*	Yangqe0098	JF978591	JN047150	JF944245	JF956277
Eudicotyledons	Asteraceae	*Sinosenecio*	*Sinosenecio globiger*	Yangqe0094	JF978594	JN047152	JF944249	JF956281
Eudicotyledons	Asteraceae	*Sinosenecio*	*Sinosenecio globiger*	Yangqe0100	JF978590	JN047149	JF944243	JF956276
Eudicotyledons	Asteraceae	*Sinosenecio*	*Sinosenecio euosmus*	Yangqe0146	JF978587	JN047145	JF944239	JF956272
Eudicotyledons	Asteraceae	*Sinosenecio*	*Sinosenecio euosmus*	Yangqe0147	JF978586	JN047144	JF944238	JF956271
Eudicotyledons	Asteraceae	*Sinosenecio*	*Sinosenecio euosmus*	Yangqe0145	JF978588	JN047146	JF944240	JF956273
Eudicotyledons	Asteraceae	*Sinosenecio*	*Sinosenecio euosmus*	Yangqe0143	JF978589	JN047148	JF944242	JF956275
Eudicotyledons	Asteraceae	*Sinosenecio*	*Sinosenecio dryas*	Yangqe0120	JF978584	JN047142	JF944234	JF956268
Eudicotyledons	Asteraceae	*Sinosenecio*	*Sinosenecio dryas*	Yangqe0121	JF978583	JN047141	JF944233	JF956267
Eudicotyledons	Asteraceae	*Sinosenecio*	*Sinosenecio dryas*	Yangqe0122	JF978582	JN047140	JF944232	JF956266
Eudicotyledons	Asteraceae	*Sinosenecio*	*Sinosenecio denticulatus*	Yangqe0090	JF978580	JN047138	JF944230	JF956264
Eudicotyledons	Asteraceae	*Sinosenecio*	*Sinosenecio denticulatus*	Yangqe0092	JF978578	JN047136	JF944228	JF956262
Eudicotyledons	Asteraceae	*Sinosenecio*	*Sinosenecio denticulatus*	Yangqe0089	JF978581	JN047139	JF944231	JF956265
Eudicotyledons	Asteraceae	*Sinosenecio*	*Sinosenecio denticulatus*	Yangqe0091	JF978579	JN047137	JF944229	JF956263
Eudicotyledons	Asteraceae	*Sinosenecio*	*Sinosenecio*	Yangqe0109	JF978577	JN047135	JF944227	JF956261
Eudicotyledons	Asteraceae	*Sinosenecio*	*Sinosenecio*	Yangqe0111	JF978575	JN047133	JF944225	JF956259
Eudicotyledons	Asteraceae	*Sinosenecio*	*Sinosenecio*	Yangqe0110	JF978576	JN047134	JF944226	JF956260
Eudicotyledons	Caryophyllacea	*Silene*	*Silene firma*	Z388	JF978561	JN047120	JF944209	JF956243
Eudicotyledons	Caryophyllacea	*Silene*	*Silene firma*	Z390	JF978559	JN047118	JF944207	JF956241
Eudicotyledons	Caryophyllacea	*Silene*	*Silene firma*	Z389	JF978560	JN047119	JF944208	JF956242
Eudicotyledons	Caryophyllacea	*Silene*	*Silene baccifera*	Z383	JF978558	JN047117	JF944206	JF956240
Eudicotyledons	Caryophyllacea	*Silene*	*Silene baccifera*	Z385	JF978556	JN047115	JF944204	JF956238
Eudicotyledons	Caryophyllacea	*Silene*	*Silene baccifera*	Z384	JF978557	JN047116	JF944205	JF956239
Eudicotyledons	Caryophyllacea	*Silene*	*Silene baccifera*	Z387	JF978554	JN047113	JF944202	JF956236
Eudicotyledons	Caryophyllacea	*Silene*	*Silene baccifera*	Z386	JF978555	JN047114	JF944203	JF956237
Eudicotyledons	Caryophyllacea	*Silene*	*Silene aprica*	Z379	JF978545	JN047104	JF944193	JF956227
Eudicotyledons	Caryophyllacea	*Silene*	*Silene aprica*	A526	JF978547	JN047106	JF944195	JF956229
Eudicotyledons	Caryophyllacea	*Silene*	*Silene aprica*	A520	JF978552	JN047111	JF944200	JF956234
Eudicotyledons	Caryophyllacea	*Silene*	*Silene aprica*	A521	JF978551	JN047110	JF944199	JF956233
Eudicotyledons	Caryophyllacea	*Silene*	*Silene aprica*	A525	JF978548	JN047107	JF944196	JF956230
Eudicotyledons	Caryophyllacea	*Silene*	*Silene aprica*	Z380	JF978544	JN047103	JF944192	JF956226
Eudicotyledons	Caryophyllacea	*Silene*	*Silene aprica*	A519	JF978553	JN047112	JF944201	JF956235
Eudicotyledons	Caryophyllacea	*Silene*	*Silene aprica*	A524	JF978549	JN047108	JF944197	JF956231

Eudicotyledons	Caryophyllacea	*Silene*	*Silene aprica*	A527	JF978546	JN047105	JF944194	JF956228
Eudicotyledons	Caryophyllacea	*Silene*	*Silene aprica*	A522	JF978550	JN047109	JF944198	JF956232
Eudicotyledons	Caryophyllacea	*Silene*	*Silene aprica*	Z382	JF978543	JN047101	JF944190	JF956224
Eudicotyledons	Adoxaceae	*Sambucus*	*Sambucus chinensis*	Z300	JF978514	JN047071	JF944177	JF956203
Eudicotyledons	Adoxaceae	*Sambucus*	*Sambucus chinensis*	Z301	JF978513	JN047070	JF944176	JF956202
Eudicotyledons	Adoxaceae	*Sambucus*	*Sambucus chinensis*	Z304	JF978522	JN047079	JF944185	JF956211
Eudicotyledons	Adoxaceae	*Sambucus*	*Sambucus chinensis*	Z306	JF978520	JN047077	JF944183	JF956209
Eudicotyledons	Adoxaceae	*Sambucus*	*Sambucus chinensis*	Z302	JF978512	JN047069	JF944175	JF956201
Eudicotyledons	Adoxaceae	*Sambucus*	*Sambucus chinensis*	Z305	JF978521	JN047078	JF944184	JF956210
Eudicotyledons	Adoxaceae	*Sambucus*	*Sambucus chinensis*	Z297	JF978517	JN047074	JF944180	JF956206
Eudicotyledons	Adoxaceae	*Sambucus*	*Sambucus chinensis*	Z296	JF978518	JN047075	JF944181	JF956207
Eudicotyledons	Adoxaceae	*Sambucus*	*Sambucus chinensis*	Z295	JF978519	JN047076	JF944182	JF956208
Eudicotyledons	Adoxaceae	*Sambucus*	*Sambucus chinensis*	Z299	JF978515	JN047072	JF944178	JF956204
Eudicotyledons	Adoxaceae	*Sambucus*	*Sambucus chinensis*	Z298	JF978516	JN047073	JF944179	JF956205
Eudicotyledons	Adoxaceae	*Sambucus*	*Sambucus adnata*	A511	JF978509	JN047067	JF944172	JF956198
Eudicotyledons	Adoxaceae	*Sambucus*	*Sambucus adnata*	A510	JF978510	JN047068	JF944173	JF956199
Eudicotyledons	Adoxaceae	*Sambucus*	*Sambucus adnata*	Z292	JF978502	JN047060	JF944164	JF956191
Eudicotyledons	Adoxaceae	*Sambucus*	*Sambucus adnata*	D1178	JF978504	JN047062	JF944166	JF956193
Eudicotyledons	Adoxaceae	*Sambucus*	*Sambucus adnata*	A512	JF978508	JN047066	JF944171	JF956197
Eudicotyledons	Adoxaceae	*Sambucus*	*Sambucus adnata*	A514	JF978506	JN047064	JF944169	JF956195
Eudicotyledons	Adoxaceae	*Sambucus*	*Sambucus adnata*	A516	JF978505	JN047063	JF944168	JF956194
Eudicotyledons	Adoxaceae	*Sambucus*	*Sambucus adnata*	A513	JF978507	JN047065	JF944170	JF956196
Eudicotyledons	Adoxaceae	*Sambucus*	*Sambucus adnata*	Z293	JF978501	JN047059	JF944163	JF956190
Eudicotyledons	Adoxaceae	*Sambucus*	*Sambucus adnata*	Z294	JF978500	JN047058	JF944162	JF956189
Eudicotyledons	Adoxaceae	*Sambucus*	*Sambucus adnata*	D955	JF978503	JN047061	JF944165	JF956192
Eudicotyledons	Grossulariaceae	*Ribes*	*Ribes himalense*	D190	JF978475	JN047044	JF944129	JF956167
Eudicotyledons	Grossulariaceae	*Ribes*	*Ribes himalense*	D957	JF978473	JN047042	JF944127	JF956166
Eudicotyledons	Grossulariaceae	*Ribes*	*Ribes alpestre*	A492	JF978468	JN047034	JF944119	JF956158
Eudicotyledons	Grossulariaceae	*Ribes*	*Ribes alpestre*	A487	JF978472	JN047039	JF944124	JF956163
Eudicotyledons	Grossulariaceae	*Ribes*	*Ribes alpestre*	A489	JF978470	JN047037	JF944122	JF956161
Eudicotyledons	Grossulariaceae	*Ribes*	*Ribes alpestre*	A488	JF978471	JN047038	JF944123	JF956162
Eudicotyledons	Grossulariaceae	*Ribes*	*Ribes alpestre*	A490	JF978469	JN047036	JF944121	JF956160
Eudicotyledons	Ericaceae	*Rhododendron*	*Rhododendron yunnanense*	B259	JF978464	JN047029	JF944114	JF956154
Eudicotyledons	Ericaceae	*Rhododendron*	*Rhododendron yunnanense*	B697	JF978462	JN047027	JF944112	JF956153
Eudicotyledons	Ericaceae	*Rhododendron*	*Rhododendron yunnanense*	B699	JF978461	JN047026	JF944111	JF956152
Eudicotyledons	Ericaceae	*Rhododendron*	*Rhododendron yunnanense*	B700	JF978460	JN047025	JF944110	JF956151
Eudicotyledons	Ericaceae	*Rhododendron*	*Rhododendron xanthostephanum*	B693	JF978456	JN047021	JF944106	JF956148
Eudicotyledons	Ericaceae	*Rhododendron*	*Rhododendron xanthostephanum*	B692	JF978457	JN047022	JF944107	JF956149

Eudicotyledons	Ericaceae	*Rhododendron*	*Rhododendron xanthostephanum*	B691	JF978458	JN047023	JF944108	JF956150
Eudicotyledons	Ericaceae	*Rhododendron*	*Rhododendron wardii*	B684	JF978453	JN047018	JF944103	JF956146
Eudicotyledons	Ericaceae	*Rhododendron*	*Rhododendron wardii*	B687	JF978452	JN047016	JF944101	JF956145
Eudicotyledons	Ericaceae	*Rhododendron*	*Rhododendron wardii*	B683	JF978454	JN047019	JF944104	JF956147
Eudicotyledons	Ericaceae	*Rhododendron*	*Rhododendron wallichii*	B682	JF978450	JN047014	JF944099	JF956143
Eudicotyledons	Ericaceae	*Rhododendron*	*Rhododendron wallichii*	B223	JF978451	JN047015	JF944100	JF956144
Eudicotyledons	Ericaceae	*Rhododendron*	*Rhododendron virgatum*	B679	JF978449	JN047013	JF944098	JF956142
Eudicotyledons	Ericaceae	*Rhododendron*	*Rhododendron virgatum*	B680	JF978448	JN047012	JF944097	JF956141
Eudicotyledons	Ericaceae	*Rhododendron*	*Rhododendron vernicosum*	B677	JF978447	JN047011	JF944096	JF956140
Eudicotyledons	Ericaceae	*Rhododendron*	*Rhododendron vernicosum*	B678	JF978446	JN047010	JF944095	JF956139
Eudicotyledons	Ericaceae	*Rhododendron*	*Rhododendron*	B673	JF978445	JN047009	JF944094	JF956138
Eudicotyledons	Ericaceae	*Rhododendron*	*Rhododendron*	B674	JF978444	JN047008	JF944093	JF956137
Eudicotyledons	Ericaceae	*Rhododendron*	*Rhododendron*	B670	JF978442	JN047006	JF944091	JF956135
Eudicotyledons	Ericaceae	*Rhododendron*	*Rhododendron*	B671	JF978441	JN047005	JF944090	JF956134
Eudicotyledons	Ericaceae	*Rhododendron*	*Rhododendron*	B558	JF978443	JN047007	JF944092	JF956136
Eudicotyledons	Ericaceae	*Rhododendron*	*Rhododendron triflorum*	B666	JF978439	JN047003	JF944088	JF956133
Eudicotyledons	Ericaceae	*Rhododendron*	*Rhododendron triflorum*	B667	JF978438	JN047002	JF944087	JF956132
Eudicotyledons	Ericaceae	*Rhododendron*	*Rhododendron trichostomum*	B664	JF978435	JN046999	JF944084	JF956129
Eudicotyledons	Ericaceae	*Rhododendron*	*Rhododendron trichostomum*	B662	JF978437	JN047001	JF944086	JF956131
Eudicotyledons	Ericaceae	*Rhododendron*	*Rhododendron trichostomum*	B663	JF978436	JN047000	JF944085	JF956130
Eudicotyledons	Ericaceae	*Rhododendron*	*Rhododendron trichocladum*	B638	JF978434	JN046998	JF944083	JF956128
Eudicotyledons	Ericaceae	*Rhododendron*	*Rhododendron trichocladum*	B659	JF978433	JN046997	JF944082	JF956127
Eudicotyledons	Ericaceae	*Rhododendron*	*Rhododendron trichocladum*	B660	JF978432	JN046996	JF944081	JF956126
Eudicotyledons	Ericaceae	*Rhododendron*	*Rhododendron traillianum*	B658	JF978427	JN046991	JF944076	JF956121
Eudicotyledons	Ericaceae	*Rhododendron*	*Rhododendron traillianum*	B655	JF978428	JN046992	JF944077	JF956122
Eudicotyledons	Ericaceae	*Rhododendron*	*Rhododendron traillianum*	B654	JF978429	JN046993	JF944078	JF956123
Eudicotyledons	Ericaceae	*Rhododendron*	*Rhododendron telmateium*	B650	JF978422	JN046986	JF944071	JF956117
Eudicotyledons	Ericaceae	*Rhododendron*	*Rhododendron telmateium*	B210	JF978424	JN046988	JF944073	JF956118
Eudicotyledons	Ericaceae	*Rhododendron*	*Rhododendron stewartianum*	B636	JF978415	JN046979	JF944063	JF956110
Eudicotyledons	Ericaceae	*Rhododendron*	*Rhododendron stewartianum*	B632	JF978416	JN046980	JF944064	JF956111

Eudicotyledons	Ericaceae	*Rhododendron*	*Rhododendron stewartianum*	B272	JF978418	JN046982	JF944066	JF956113
Eudicotyledons	Ericaceae	*Rhododendron*	*Rhododendron stewartianum*	B637	JF978414	JN046978	JF944062	JF956109
Eudicotyledons	Ericaceae	*Rhododendron*	*Rhododendron stewartianum*	B262	JF978419	JN046983	JF944067	JF956114
Eudicotyledons	Ericaceae	*Rhododendron*	*Rhododendron stewartianum*	B275	JF978417	JN046981	JF944065	JF956112
Eudicotyledons	Ericaceae	*Rhododendron*	*Rhododendron*	B627	JF978413	JN046975	JF944059	JF956108
Eudicotyledons	Ericaceae	*Rhododendron*	*Rhododendron*	B628	JF978412	JN046974	JF944058	JF956107
Eudicotyledons	Ericaceae	*Rhododendron*	*Rhododendron sphaeroblastum*	B626	JF978409	JN046971	JF944055	JF956104
Eudicotyledons	Ericaceae	*Rhododendron*	*Rhododendron sphaeroblastum*	B303	JF978410	JN046972	JF944056	JF956105
Eudicotyledons	Ericaceae	*Rhododendron*	*Rhododendron sinonuttallii*	B622	JF978407	JN046969	JF944053	JF956102
Eudicotyledons	Ericaceae	*Rhododendron*	*Rhododendron sinonuttallii*	B620	JF978408	JN046970	JF944054	JF956103
Eudicotyledons	Ericaceae	*Rhododendron*	*Rhododendron sinogrande*	B617	JF978404	JN046966	JF944050	JF956099
Eudicotyledons	Ericaceae	*Rhododendron*	*Rhododendron sinogrande*	B616	JF978405	JN046967	JF944051	JF956100
Eudicotyledons	Ericaceae	*Rhododendron*	*Rhododendron sinogrande*	B245	JF978406	JN046968	JF944052	JF956101
Eudicotyledons	Ericaceae	*Rhododendron*	*Rhododendron sinogrande*	B619	JF978402	JN046964	JF944048	JF956098
Eudicotyledons	Ericaceae	*Rhododendron*	*Rhododendron selense*	B379	JF978395	JN046955	JF944039	JF956091
Eudicotyledons	Ericaceae	*Rhododendron*	*Rhododendron selense*	B263	JF978397	JN046957	JF944041	JF956093
Eudicotyledons	Ericaceae	*Rhododendron*	*Rhododendron selense*	B274	JF978396	JN046956	JF944040	JF956092
Eudicotyledons	Ericaceae	*Rhododendron*	*Rhododendron*	B572	JF978381	JN046940	JF944024	JF956077
Eudicotyledons	Ericaceae	*Rhododendron*	*Rhododendron*	B571	JF978382	JN046941	JF944025	JF956078
Eudicotyledons	Ericaceae	*Rhododendron*	*Rhododendron*	B590	JF978378	JN046937	JF944021	JF956074
Eudicotyledons	Ericaceae	*Rhododendron*	*Rhododendron*	B569	JF978383	JN046942	JF944026	JF956079
Eudicotyledons	Ericaceae	*Rhododendron*	*Rhododendron*	B589	JF978379	JN046938	JF944022	JF956075
Eudicotyledons	Ericaceae	*Rhododendron*	*Rhododendron*	B242	JF978384	JN046943	JF944027	JF956080
Eudicotyledons	Ericaceae	*Rhododendron*	*Rhododendron*	B588	JF978380	JN046939	JF944023	JF956076
Eudicotyledons	Ericaceae	*Rhododendron*	*Rhododendron racemosum*	B565	JF978371	JN046932	JF944014	JF956067
Eudicotyledons	Ericaceae	*Rhododendron*	*Rhododendron racemosum*	B564	JF978372	JN046933	JF944015	JF956068
Eudicotyledons	Ericaceae	*Rhododendron*	*Rhododendron racemosum*	B563	JF978373	JN046934	JF944016	JF956069
Eudicotyledons	Ericaceae	*Rhododendron*	*Rhododendron primuliflorum*	B665	JF978367	JN046929	JF944010	JF956063
Eudicotyledons	Ericaceae	*Rhododendron*	*Rhododendron primuliflorum*	B203	JF978369	JN046931	JF944012	JF956065
Eudicotyledons	Ericaceae	*Rhododendron*	*Rhododendron primuliflorum*	B209	JF978368	JN046930	JF944011	JF956064
Eudicotyledons	Ericaceae	*Rhododendron*	*Rhododendron pendulum*	B219	JF978359	JN046921	JF944002	JF956055

Eudicotyledons	Ericaceae	*Rhododendron*	*Rhododendron pendulum*	B540	JF978358	JN046920	JF944001	JF956054
Eudicotyledons	Ericaceae	*Rhododendron*	*Rhododendron*	B536	JF978357	JN046919	JF944000	JF956053
Eudicotyledons	Ericaceae	*Rhododendron*	*Rhododendron*	B538	JF978355	JN046917	JF943998	JF956051
Eudicotyledons	Ericaceae	*Rhododendron*	*Rhododendron*	B537	JF978356	JN046918	JF943999	JF956052
Eudicotyledons	Ericaceae	*Rhododendron*	*Rhododendron oreotrephes*	B257	JF978352	JN046914	JF943995	JF956050
Eudicotyledons	Ericaceae	*Rhododendron*	*Rhododendron oreotrephes*	B531	JF978348	JN046909	JF943990	JF956046
Eudicotyledons	Ericaceae	*Rhododendron*	*Rhododendron oreotrephes*	B527	JF978350	JN046911	JF943992	JF956048
Eudicotyledons	Ericaceae	*Rhododendron*	*Rhododendron oreotrephes*	B529	JF978349	JN046910	JF943991	JF956047
Eudicotyledons	Ericaceae	*Rhododendron*	*Rhododendron oreotrephes*	B644	JF978347	JN046908	JF943989	JF956045
Eudicotyledons	Ericaceae	*Rhododendron*	*Rhododendron oreotrephes*	B526	JF978351	JN046912	JF943993	JF956049
Eudicotyledons	Ericaceae	*Rhododendron*	*Rhododendron nyingchiense*	B524	JF978345	JN046906	JF943987	JF956043
Eudicotyledons	Ericaceae	*Rhododendron*	*Rhododendron nyingchiense*	B523	JF978346	JN046907	JF943988	JF956044
Eudicotyledons	Ericaceae	*Rhododendron*	*Rhododendron nivale*	B647	JF978337	JN046898	JF943979	JF956035
Eudicotyledons	Ericaceae	*Rhododendron*	*Rhododendron nivale*	B247	JF978342	JN046903	JF943984	JF956040
Eudicotyledons	Ericaceae	*Rhododendron*	*Rhododendron nivale*	B207	JF978344	JN046905	JF943986	JF956042
Eudicotyledons	Ericaceae	*Rhododendron*	*Rhododendron nivale*	B208	JF978343	JN046904	JF943985	JF956041
Eudicotyledons	Ericaceae	*Rhododendron*	*Rhododendron nivale*	B532	JF978338	JN046899	JF943980	JF956036
Eudicotyledons	Ericaceae	*Rhododendron*	*Rhododendron nivale*	B522	JF978339	JN046900	JF943981	JF956037
Eudicotyledons	Ericaceae	*Rhododendron*	*Rhododendron nivale*	B648	JF978336	JN046897	JF943978	JF956034
Eudicotyledons	Ericaceae	*Rhododendron*	*Rhododendron nivale*	B518	JF978341	JN046902	JF943983	JF956039
Eudicotyledons	Ericaceae	*Rhododendron*	*Rhododendron nivale*	B520	JF978340	JN046901	JF943982	JF956038
Eudicotyledons	Ericaceae	*Rhododendron*	*Rhododendron monanthum*	B284	JF978333	JN046889	JF943970	JF956030
Eudicotyledons	Ericaceae	*Rhododendron*	*Rhododendron monanthum*	B508	JF978331	JN046887	JF943968	JF956028
Eudicotyledons	Ericaceae	*Rhododendron*	*Rhododendron monanthum*	B507	JF978332	JN046888	JF943969	JF956029
Eudicotyledons	Ericaceae	*Rhododendron*	*Rhododendron*	B505	JF978327	JN046883	JF943964	JF956024
Eudicotyledons	Ericaceae	*Rhododendron*	*Rhododendron*	B503	JF978328	JN046884	JF943965	JF956025
Eudicotyledons	Ericaceae	*Rhododendron*	*Rhododendron*	B502	JF978329	JN046885	JF943966	JF956026
Eudicotyledons	Ericaceae	*Rhododendron*	*Rhododendron*	B501	JF978330	JN046886	JF943967	JF956027
Eudicotyledons	Ericaceae	*Rhododendron*	*Rhododendron mariesii*	B499	JF978322	JN046878	JF943960	JF956019
Eudicotyledons	Ericaceae	*Rhododendron*	*Rhododendron mariesii*	B498	JF978323	JN046879	JF943961	JF956020
Eudicotyledons	Ericaceae	*Rhododendron*	*Rhododendron maddenii*	B497	JF978319	JN046875	JF943957	JF956017
Eudicotyledons	Ericaceae	*Rhododendron*	*Rhododendron maddenii*	B221	JF978321	JN046877	JF943959	JF956018
Eudicotyledons	Ericaceae	*Rhododendron*	*Rhododendron mackenzianum*	B495	JF978317	JN046873	JF943956	JF956016
Eudicotyledons	Ericaceae	*Rhododendron*	*Rhododendron mackenzianum*	B496	JF978316	JN046872	JF943955	JF956015
Eudicotyledons	Ericaceae	*Rhododendron*	*Rhododendron lukiangense*	B244	JF978315	JN046871	JF943954	JF956014
Eudicotyledons	Ericaceae	*Rhododendron*	*Rhododendron lukiangense*	B492	JF978314	JN046870	JF943953	JF956013

Eudicotyledons	Ericaceae	*Rhododendron*	*Rhododendron leptothrium*	B484	JF978312	JN046868	JF943951	JF956012
Eudicotyledons	Ericaceae	*Rhododendron*	*Rhododendron leptothrium*	B487	JF978311	JN046867	JF943950	JF956011
Eudicotyledons	Ericaceae	*Rhododendron*	*Rhododendron lepidotum*	B482	JF978304	JN046860	JF943943	JF956006
Eudicotyledons	Ericaceae	*Rhododendron*	*Rhododendron lepidotum*	B611	JF978299	JN046855	JF943938	JF956001
Eudicotyledons	Ericaceae	*Rhododendron*	*Rhododendron lepidotum*	B483	JF978303	JN046859	JF943942	JF956005
Eudicotyledons	Ericaceae	*Rhododendron*	*Rhododendron lepidotum*	B227	JF978306	JN046862	JF943945	JF956008
Eudicotyledons	Ericaceae	*Rhododendron*	*Rhododendron lepidotum*	B236	JF978305	JN046861	JF943944	JF956007
Eudicotyledons	Ericaceae	*Rhododendron*	*Rhododendron lepidotum*	B609	JF978300	JN046856	JF943939	JF956002
Eudicotyledons	Ericaceae	*Rhododendron*	*Rhododendron lepidotum*	B215	JF978307	JN046863	JF943946	JF956009
Eudicotyledons	Ericaceae	*Rhododendron*	*Rhododendron lepidotum*	B214	JF978308	JN046864	JF943947	JF956010
Eudicotyledons	Ericaceae	*Rhododendron*	*Rhododendron lepidotum*	B556	JF978302	JN046858	JF943941	JF956004
Eudicotyledons	Ericaceae	*Rhododendron*	*Rhododendron lepidotum*	B607	JF978301	JN046857	JF943940	JF956003
Eudicotyledons	Ericaceae	*Rhododendron*	*Rhododendron lacteum*	B477	JF978298	JN046854	JF943937	JF956000
Eudicotyledons	Ericaceae	*Rhododendron*	*Rhododendron lacteum*	B478	JF978297	JN046853	JF943936	JF955999
Eudicotyledons	Ericaceae	*Rhododendron*	*Rhododendron lacteum*	B479	JF978296	JN046852	JF943935	JF955998
Eudicotyledons	Ericaceae	*Rhododendron*	*Rhododendron keleticum*	B255	JF978295	JN046851	JF943934	JF955997
Eudicotyledons	Ericaceae	*Rhododendron*	*Rhododendron keleticum*	B473	JF978294	JN046850	JF943933	JF955996
Eudicotyledons	Ericaceae	*Rhododendron*	*Rhododendron irroratum*	B468	JF978292	JN046848	JF943931	JF955994
Eudicotyledons	Ericaceae	*Rhododendron*	*Rhododendron irroratum*	B470	JF978290	JN046846	JF943929	JF955993
Eudicotyledons	Ericaceae	*Rhododendron*	*Rhododendron irroratum*	B465	JF978293	JN046849	JF943932	JF955995
Eudicotyledons	Ericaceae	*Rhododendron*	*Rhododendron impeditum*	B463	JF978287	JN046843	JF943926	JF955990
Eudicotyledons	Ericaceae	*Rhododendron*	*Rhododendron impeditum*	B199	JF978289	JN046845	JF943928	JF955992
Eudicotyledons	Ericaceae	*Rhododendron*	*Rhododendron impeditum*	B294	JF978288	JN046844	JF943927	JF955991
Eudicotyledons	Ericaceae	*Rhododendron*	*Rhododendron hippophaeoides*	B240	JF978286	JN046841	JF943925	JF955989
Eudicotyledons	Ericaceae	*Rhododendron*	*Rhododendron hippophaeoides*	B461	JF978285	JN046840	JF943924	JF955988
Eudicotyledons	Ericaceae	*Rhododendron*	*Rhododendron heliolepis*	B286	JF978284	JN046839	JF943923	JF955987
Eudicotyledons	Ericaceae	*Rhododendron*	*Rhododendron heliolepis*	B460	JF978281	JN046836	JF943920	JF955985
Eudicotyledons	Ericaceae	*Rhododendron*	*Rhododendron heliolepis*	B304	JF978283	JN046838	JF943922	JF955986
Eudicotyledons	Ericaceae	*Rhododendron*	*Rhododendron haematodes*	B458	JF978275	JN046830	JF943914	JF955979
Eudicotyledons	Ericaceae	*Rhododendron*	*Rhododendron haematodes*	B457	JF978276	JN046831	JF943915	JF955980
Eudicotyledons	Ericaceae	*Rhododendron*	*Rhododendron haematodes*	B456	JF978277	JN046832	JF943916	JF955981
Eudicotyledons	Ericaceae	*Rhododendron*	*Rhododendron haematodes*	B450	JF978280	JN046835	JF943919	JF955984
Eudicotyledons	Ericaceae	*Rhododendron*	*Rhododendron haematodes*	B453	JF978278	JN046833	JF943917	JF955982
Eudicotyledons	Ericaceae	*Rhododendron*	*Rhododendron haematodes*	B452	JF978279	JN046834	JF943918	JF955983
Eudicotyledons	Ericaceae	*Rhododendron*	*Rhododendron glischrum*	B446	JF978274	JN046829	JF943913	JF955978
Eudicotyledons	Ericaceae	*Rhododendron*	*Rhododendron glischrum*	B448	JF978273	JN046828	JF943912	JF955977
Eudicotyledons	Ericaceae	*Rhododendron*	*Rhododendron genestierianum*	B444	JF978272	JN046827	JF943911	JF955976

Eudicotyledons	Ericaceae	*Rhododendron*	*Rhododendron genestierianum*	B445	JF978271	JN046826	JF943910	JF955975
Eudicotyledons	Ericaceae	*Rhododendron*	*Rhododendron fulvum*	B439	JF978268	JN046823	JF943907	JF955974
Eudicotyledons	Ericaceae	*Rhododendron*	*Rhododendron fulvum*	B440	JF978267	JN046822	JF943906	JF955973
Eudicotyledons	Ericaceae	*Rhododendron*	*Rhododendron fragariiflorum*	B560	JF978265	JN046820	JF943904	JF955971
Eudicotyledons	Ericaceae	*Rhododendron*	*Rhododendron fragariiflorum*	B437	JF978266	JN046821	JF943905	JF955972
Eudicotyledons	Ericaceae	*Rhododendron*	*Rhododendron forrestii*	B435	JF978264	JN046819	JF943903	JF955970
Eudicotyledons	Ericaceae	*Rhododendron*	*Rhododendron forrestii*	B436	JF978263	JN046818	JF943902	JF955969
Eudicotyledons	Ericaceae	*Rhododendron*	*Rhododendron floccigerum*	B434	JF978259	JN046814	JF943898	JF955965
Eudicotyledons	Ericaceae	*Rhododendron*	*Rhododendron floccigerum*	B418	JF978262	JN046817	JF943901	JF955968
Eudicotyledons	Ericaceae	*Rhododendron*	*Rhododendron floccigerum*	B431	JF978260	JN046815	JF943899	JF955966
Eudicotyledons	Ericaceae	*Rhododendron*	*Rhododendron floccigerum*	B430	JF978261	JN046816	JF943900	JF955967
Eudicotyledons	Ericaceae	*Rhododendron*	*Rhododendron fastigiatum*	B300	JF978256	JN046811	JF943895	JF955962
Eudicotyledons	Ericaceae	*Rhododendron*	*Rhododendron fastigiatum*	B425	JF978254	JN046809	JF943893	JF955960
Eudicotyledons	Ericaceae	*Rhododendron*	*Rhododendron fastigiatum*	B424	JF978255	JN046810	JF943894	JF955961
Eudicotyledons	Ericaceae	*Rhododendron*	*Rhododendron fastigiatum*	B426	JF978253	JN046808	JF943892	JF955959
Eudicotyledons	Ericaceae	*Rhododendron*	*Rhododendron emarginatum*	B415	JF978248	JN046803	JF943887	JF955955
Eudicotyledons	Ericaceae	*Rhododendron*	*Rhododendron emarginatum*	B414	JF978249	JN046804	JF943888	JF955956
Eudicotyledons	Ericaceae	*Rhododendron*	*Rhododendron edgeworthii*	B406	JF978246	JN046801	JF943885	JF955953
Eudicotyledons	Ericaceae	*Rhododendron*	*Rhododendron edgeworthii*	B408	JF978245	JN046800	JF943884	JF955952
Eudicotyledons	Ericaceae	*Rhododendron*	*Rhododendron edgeworthii*	B410	JF978243	JN046798	JF943882	JF955950
Eudicotyledons	Ericaceae	*Rhododendron*	*Rhododendron edgeworthii*	B405	JF978247	JN046802	JF943886	JF955954
Eudicotyledons	Ericaceae	*Rhododendron*	*Rhododendron edgeworthii*	B409	JF978244	JN046799	JF943883	JF955951
Eudicotyledons	Ericaceae	*Rhododendron*	*Rhododendron delavayi*	B402	JF978238	JN046793	JF943877	JF955945
Eudicotyledons	Ericaceae	*Rhododendron*	*Rhododendron delavayi*	B400	JF978240	JN046795	JF943879	JF955947
Eudicotyledons	Ericaceae	*Rhododendron*	*Rhododendron delavayi*	B401	JF978239	JN046794	JF943878	JF955946
Eudicotyledons	Ericaceae	*Rhododendron*	*Rhododendron delavayi*	B398	JF978242	JN046797	JF943881	JF955949
Eudicotyledons	Ericaceae	*Rhododendron*	*Rhododendron delavayi*	B399	JF978241	JN046796	JF943880	JF955948
Eudicotyledons	Ericaceae	*Rhododendron*	*Rhododendron decorum*	B385	JF978235	JN046790	JF943874	JF955942
Eudicotyledons	Ericaceae	*Rhododendron*	*Rhododendron decorum*	B390	JF978233	JN046788	JF943872	JF955940
Eudicotyledons	Ericaceae	*Rhododendron*	*Rhododendron decorum*	B393	JF978232	JN046786	JF943870	JF955938
Eudicotyledons	Ericaceae	*Rhododendron*	*Rhododendron decorum*	B383	JF978237	JN046792	JF943876	JF955944
Eudicotyledons	Ericaceae	*Rhododendron*	*Rhododendron decorum*	B384	JF978236	JN046791	JF943875	JF955943
Eudicotyledons	Ericaceae	*Rhododendron*	*Rhododendron decorum*	B389	JF978234	JN046789	JF943873	JF955941
Eudicotyledons	Ericaceae	*Rhododendron*	*Rhododendron complexum*	B423	JF978230	JN046784	JF943868	JF955936
Eudicotyledons	Ericaceae	*Rhododendron*	*Rhododendron complexum*	B213	JF978231	JN046785	JF943869	JF955937

Eudicotyledons	Ericaceae	*Rhododendron*	*Rhododendron cephalanthum*	B368	JF978222	JN046776	JF943861	JF955929
Eudicotyledons	Ericaceae	*Rhododendron*	*Rhododendron cephalanthum*	B366	JF978223	JN046777	JF943862	JF955930
Eudicotyledons	Ericaceae	*Rhododendron*	*Rhododendron campylocarpum*	B360	JF978218	JN046770	JF943855	JF955924
Eudicotyledons	Ericaceae	*Rhododendron*	*Rhododendron campylocarpum*	B353	JF978221	JN046774	JF943859	JF955927
Eudicotyledons	Ericaceae	*Rhododendron*	*Rhododendron campylocarpum*	B354	JF978220	JN046773	JF943858	JF955926
Eudicotyledons	Ericaceae	*Rhododendron*	*Rhododendron campylocarpum*	B355	JF978219	JN046772	JF943857	JF955925
Eudicotyledons	Ericaceae	*Rhododendron*	*Rhododendron bureavii*	B297	JF978215	JN046765	JF943851	JF955920
Eudicotyledons	Ericaceae	*Rhododendron*	*Rhododendron bureavii*	B306	JF978214	JN046764	JF943850	JF955919
Eudicotyledons	Ericaceae	*Rhododendron*	*Rhododendron beesianum*	B335	JF978212	JN046761	JF943848	JF955917
Eudicotyledons	Ericaceae	*Rhododendron*	*Rhododendron beesianum*	B336	JF978211	JN046760	JF943847	JF955916
Eudicotyledons	Ericaceae	*Rhododendron*	*Rhododendron beesianum*	B337	JF978210	JN046759	JF943846	JF955915
Eudicotyledons	Ericaceae	*Rhododendron*	*Rhododendron beesianum*	B338	JF978209	JN046758	JF943845	JF955914
Eudicotyledons	Ericaceae	*Rhododendron*	*Rhododendron beesianum*	B334	JF978213	JN046762	JF943849	JF955918
Eudicotyledons	Ericaceae	*Rhododendron*	*Rhododendron bainbridgeanum*	B331	JF978208	JN046757	JF943844	JF955913
Eudicotyledons	Ericaceae	*Rhododendron*	*Rhododendron bainbridgeanum*	B332	JF978207	JN046756	JF943843	JF955912
Eudicotyledons	Ericaceae	*Rhododendron*	*Rhododendron arizelum*	B330	JF978203	JN046752	JF943839	JF955908
Eudicotyledons	Ericaceae	*Rhododendron*	*Rhododendron arizelum*	B328	JF978205	JN046754	JF943841	JF955910
Eudicotyledons	Ericaceae	*Rhododendron*	*Rhododendron arizelum*	B266	JF978206	JN046755	JF943842	JF955911
Eudicotyledons	Ericaceae	*Rhododendron*	*Rhododendron arizelum*	B329	JF978204	JN046753	JF943840	JF955909
Eudicotyledons	Ericaceae	*Rhododendron*	*Rhododendron arboreum*	B396	JF978200	JN046749	JF943836	JF955905
Eudicotyledons	Ericaceae	*Rhododendron*	*Rhododendron arboreum*	B395	JF978201	JN046750	JF943837	JF955906
Eudicotyledons	Ericaceae	*Rhododendron*	*Rhododendron arboreum*	B397	JF978199	JN046748	JF943835	JF955904
Eudicotyledons	Ericaceae	*Rhododendron*	*Rhododendron arboreum*	B394	JF978202	JN046751	JF943838	JF955907
Eudicotyledons	Ericaceae	*Rhododendron*	*Rhododendron araiophyllum*	B326	JF978197	JN046746	JF943833	JF955902
Eudicotyledons	Ericaceae	*Rhododendron*	*Rhododendron araiophyllum*	B325	JF978198	JN046747	JF943834	JF955903
Eudicotyledons	Ericaceae	*Rhododendron*	*Rhododendron aperantum*	B324	JF978194	JN046743	JF943830	JF955899
Eudicotyledons	Ericaceae	*Rhododendron*	*Rhododendron aperantum*	B323	JF978195	JN046744	JF943831	JF955900
Eudicotyledons	Ericaceae	*Rhododendron*	*Rhododendron aperantum*	B322	JF978196	JN046745	JF943832	JF955901
Eudicotyledons	Ericaceae	*Rhododendron*	*Rhododendron anthosphaerum*	B258	JF978193	JN046742	JF943829	JF955898

Eudicotyledons	Ericaceae	*Rhododendron*	*Rhododendron anthosphaerum*	B318	JF978192	JN046741	JF943828	JF955897
Eudicotyledons	Ericaceae	*Rhododendron*	*Rhododendron anthosphaerum*	B321	JF978190	JN046739	JF943826	JF955895
Eudicotyledons	Ericaceae	*Rhododendron*	*Rhododendron anthosphaerum*	B319	JF978191	JN046740	JF943827	JF955896
Eudicotyledons	Ericaceae	*Rhododendron*	*Rhododendron agastum*	B317	JF978185	JN046735	JF943822	JF955892
Eudicotyledons	Ericaceae	*Rhododendron*	*Rhododendron agastum*	B315	JF978186	JN046736	JF943823	JF955893
Eudicotyledons	Ericaceae	*Rhododendron*	*Rhododendron*	B310	JF978182	JN046732	JF943819	JF955891
Eudicotyledons	Ericaceae	*Rhododendron*	*Rhododendron*	B311	JF978181	JN046731	JF943818	JF955890
Eudicotyledons	Crassulaceae	*Rhodiola*	*Rhodiola kirilowii*	D473	JF978178	JN046726	JF943813	JF955887
Eudicotyledons	Crassulaceae	*Rhodiola*	*Rhodiola kirilowii*	D1668	JF978179	JN046727	JF943814	JF955888
Eudicotyledons	Crassulaceae	*Rhodiola*	*Rhodiola fastigiata*	D1630	JF978176	JN046724	JF943811	JF955885
Eudicotyledons	Crassulaceae	*Rhodiola*	*Rhodiola fastigiata*	D097	JF978177	JN046725	JF943812	JF955886
Eudicotyledons	Crassulaceae	*Rhodiola*	*Rhodiola bupleuroides*	D1654	JF978172	JN046719	JF943806	JF955883
Eudicotyledons	Crassulaceae	*Rhodiola*	*Rhodiola bupleuroides*	D1251	JF978173	JN046720	JF943807	JF955884
Eudicotyledons	Polygonaceae	*Rheum*	*Rheum tanguticum*	PS2904MT07	GQ434863	GQ43548	GQ43677	GQ43430
Eudicotyledons	Polygonaceae	*Rheum*	*Rheum tanguticum*	PS2904MT09	GQ434864	GQ43548	GQ43677	GQ43430
Eudicotyledons	Polygonaceae	*Rheum*	*Rheum tanguticum*	PS2904MT03	GQ434862	GQ43548	GQ43676	GQ43430
Eudicotyledons	Polygonaceae	*Rheum*	*Rheum officinale*	PS2902MT09	GQ434859	GQ43547	GQ43676	GQ43429
Eudicotyledons	Polygonaceae	*Rheum*	*Rheum officinale*	PS2902MT08	GQ434858	GQ43547	GQ43676	GQ43429
Eudicotyledons	Polygonaceae	*Rheum*	*Rheum officinale*	PS2902MT03	GQ434857	GQ43547	GQ43676	GQ43429
Eudicotyledons	Brassicaceae	*Pugionium*	*Pugionium dolabratum*	Liujq0042	JF978167	JN046694	JF943782	JF955858
Eudicotyledons	Brassicaceae	*Pugionium*	*Pugionium dolabratum*	Liujq0033	JF978169	JN046696	JF943784	JF955860
Eudicotyledons	Brassicaceae	*Pugionium*	*Pugionium dolabratum*	Liujq0034-1	JF978168	JN046695	JF943783	JF955859
Eudicotyledons	Brassicaceae	*Pugionium*	*Pugionium dolabratum*	Liujq0022	JF978170	JN046697	JF943785	JF955861
Eudicotyledons	Brassicaceae	*Pugionium*	*Pugionium dolabratum*	Liujq0004	JF978171	JN046698	JF943786	JF955862
Eudicotyledons	Brassicaceae	*Pugionium*	*Pugionium cornutum*	Liujq0018-1	JF978166	JN046693	JF943781	JF955857
Eudicotyledons	Brassicaceae	*Pugionium*	*Pugionium cornutum*	Liujq0061	JF978164	JN046691	JF943779	JF955855
Eudicotyledons	Brassicaceae	*Pugionium*	*Pugionium cornutum*	Liujq0066	JF978162	JN046689	JF943777	JF955853
Eudicotyledons	Brassicaceae	*Pugionium*	*Pugionium cornutum*	Liujq0065	JF978163	JN046690	JF943778	JF955854
Eudicotyledons	Brassicaceae	*Pugionium*	*Pugionium cornutum*	Liujq0050	JF978165	JN046692	JF943780	JF955856
Eudicotyledons	Orobanchaceae	*Pterygiella*	*Pterygiella suffruticosa*	DongLN-LC2	JF978161	JN046688	JF943776	JF955852
Eudicotyledons	Orobanchaceae	*Pterygiella*	*Pterygiella suffruticosa*	DongLN-ML10	JF978160	JN046687	JF943775	JF955851
Eudicotyledons	Orobanchaceae	*Pterygiella*	*Pterygiella suffruticosa*	DongLN-SP1B	JF978158	JN046685	JF943773	JF955849
Eudicotyledons	Orobanchaceae	*Pterygiella*	*Pterygiella suffruticosa*	DongLN-SP35	JF978157	JN046684	JF943772	JF955848
Eudicotyledons	Orobanchaceae	*Pterygiella*	*Pterygiella suffruticosa*	DongLN-ML11	JF978159	JN046686	JF943774	JF955850
Eudicotyledons	Orobanchaceae	*Pterygiella*	*Pterygiella nigrescens*	DongLN-NS1	JF978156	JN046683	JF943771	JF955847
Eudicotyledons	Orobanchaceae	*Pterygiella*	*Pterygiella nigrescens*	DongLN-NS15	JF978155	JN046682	JF943770	JF955846
Eudicotyledons	Orobanchaceae	*Pterygiella*	*Pterygiella nigrescens*	DongLN-NS9	JF978154	JN046681	JF943769	JF955845

Eudicotyledons	Orobanchaceae	*Pterygiella*	*Pterygiella duclouxii*	DongLN-MZ8	JF978151	JN046678	JF943766	JF955842
Eudicotyledons	Orobanchaceae	*Pterygiella*	*Pterygiella duclouxii*	DongLN-SP1A	JF978150	JN046677	JF943765	JF955841
Eudicotyledons	Orobanchaceae	*Pterygiella*	*Pterygiella duclouxii*	DongLN-XC12	JF978149	JN046676	JF943764	JF955840
Eudicotyledons	Orobanchaceae	*Pterygiella*	*Pterygiella duclouxii*	DongLN-DL11	JF978153	JN046680	JF943768	JF955844
Eudicotyledons	Orobanchaceae	*Pterygiella*	*Pterygiella duclouxii*	DongLN-ML6A	JF978152	JN046679	JF943767	JF955843
Eudicotyledons	Orobanchaceae	*Pterygiella*	*Pterygiella duclouxii*	DongLN-XS5	JF978148	JN046675	JF943763	JF955839
Eudicotyledons	Orobanchaceae	*Pterygiella*	*Pterygiella cylindrica*	DongLN-ML1	JF978147	JN046674	JF943762	JF955838
Eudicotyledons	Orobanchaceae	*Pterygiella*	*Pterygiella cylindrica*	DongLN-ML6B	JF978145	JN046672	JF943760	JF955836
Eudicotyledons	Orobanchaceae	*Pterygiella*	*Pterygiella cylindrica*	DongLN-ML13	JF978146	JN046673	JF943761	JF955837
Eudicotyledons	Rosaceae	*Prunus*	*Prunus persica*	ZhouSL-f-pendula-Henan-032	JF978124	JN046657	JF943751	JF955825
Eudicotyledons	Rosaceae	*Prunus*	*Prunus persica*	ZhouSL-Henan-046	JF978122	JN046655	JF943749	JF955823
Eudicotyledons	Rosaceae	*Prunus*	*Prunus persica*	ZhouSL-Henan-045	JF978123	JN046656	JF943750	JF955824
Eudicotyledons	Rosaceae	*Prunus*	*Prunus persica*	ZhouSL-f-duplex-Henan-029	JF978125	JN046658	JF943752	JF955826
Eudicotyledons	Rosaceae	*Prunus*	*Prunus persica*	PS1117MT01	JF421469	GQ43526	GQ43659	GQ43420
Eudicotyledons	Rosaceae	*Prunus*	*Prunus mume*	ZhouSL-136	JF978118	JN046650	JF943748	JF955822
Eudicotyledons	Rosaceae	*Prunus*	*Prunus mume*	ZhouSL-Hubei-127	JF978116	JN046648	JF943746	JF955820
Eudicotyledons	Rosaceae	*Prunus*	*Prunus mume*	ZhouSL-Hubei-126	JF978117	JN046649	JF943747	JF955821
Eudicotyledons	Rosaceae	*Prunus*	*Prunus mira*	ZhouSL-Henan-028	JF978114	JN046646	JF943745	JF955818
Eudicotyledons	Rosaceae	*Prunus*	*Prunus mira*	ZhouSL-Sichuan-093	JF978112	JN046644	JF943744	JF955817
Eudicotyledons	Rosaceae	*Prunus*	*Prunus kansuensis*	ZhouSL-Henan-024	JF978098	JN046631	JF943738	JF955809
Eudicotyledons	Rosaceae	*Prunus*	*Prunus kansuensis*	ZhouSL-Henan-026	JF978097	JN046630	JF943737	JF955808
Eudicotyledons	Rosaceae	*Prunus*	*Prunus ferganensis*	ZhouSL-Henan-020	JF978089	JN046623	JF943733	JF955802
Eudicotyledons	Rosaceae	*Prunus*	*Prunus ferganensis*	ZhouSL-Henan-019	JF978090	JN046624	JF943734	JF955803
Eudicotyledons	Rosaceae	*Prunus*	*Prunus armeniaca*	ZhouSL-Liaojing-123	JF978106	JN046637	JF943741	JF955812
Eudicotyledons	Rosaceae	*Prunus*	*Prunus armeniaca*	ZhouSL-Liaojing-125	JF978104	JN046635	JF943739	JF955810
Eudicotyledons	Rosaceae	*Prunus*	*Prunus armeniaca*	ZhouSL-Liaojing-124	JF978105	JN046636	JF943740	JF955811
Eudicotyledons	Primulaceae	*Primula*	*Primula yunnanensis*	GXJ098	JF978069	JN046601	JF943728	JF955793
Eudicotyledons	Primulaceae	*Primula*	*Primula yunnanensis*	GXJ099	JF978068	JN046600	JF943727	JF955792
Eudicotyledons	Primulaceae	*Primula*	*Primula wangii*	GXJ271	JF978066	JN046598	JF943725	JF955790
Eudicotyledons	Primulaceae	*Primula*	*Primula wangii*	GXJ270	JF978067	JN046599	JF943726	JF955791
Eudicotyledons	Primulaceae	*Primula*	*Primula tardiflora*	GXJ222	JF978064	JN046594	JF943721	JF955786
Eudicotyledons	Primulaceae	*Primula*	*Primula tardiflora*	GXJ204	JF978065	JN046595	JF943722	JF955787
Eudicotyledons	Primulaceae	*Primula*	*Primula szechuanica*	GXJ181	JF978063	JN046593	JF943720	JF955785
Eudicotyledons	Primulaceae	*Primula*	*Primula szechuanica*	GXJ182	JF978062	JN046592	JF943719	JF955784
Eudicotyledons	Primulaceae	*Primula*	*Primula spicata*	GXJ219	JF978060	JN046590	JF943717	JF955782
Eudicotyledons	Primulaceae	*Primula*	*Primula spicata*	GXJ188	JF978061	JN046591	JF943718	JF955783
Eudicotyledons	Primulaceae	*Primula*	*Primula sonchifolia*	GXJ102	JF978059	JN046589	JF943716	JF955781
Eudicotyledons	Primulaceae	*Primula*	*Primula sonchifolia*	GXJ103	JF978058	JN046588	JF943715	JF955780
Eudicotyledons	Primulaceae	*Primula*	*Primula sonchifolia*	GXJ104	JF978057	JN046587	JF943714	JF955779
Eudicotyledons	Primulaceae	*Primula*	*Primula sonchifolia*	GXJ105	JF978056	JN046586	JF943713	JF955778

Eudicotyledons	Primulaceae	*Primula*	*Primula sonchifolia*	GXJ106	JF978055	JN046585	JF943712	JF955777
Eudicotyledons	Primulaceae	*Primula*	*Primula sinensis*	GXJ225	JF978052	JN046582	JF943709	JF955774
Eudicotyledons	Primulaceae	*Primula*	*Primula sinensis*	GXJ224	JF978053	JN046583	JF943710	JF955775
Eudicotyledons	Primulaceae	*Primula*	*Primula sinensis*	GXJ207	JF978054	JN046584	JF943711	JF955776
Eudicotyledons	Primulaceae	*Primula*	*Primula sikkimensis*	GXJ147	JF978051	JN046581	JF943708	JF955773
Eudicotyledons	Primulaceae	*Primula*	*Primula sikkimensis*	GXJ151	JF978047	JN046577	JF943704	JF955769
Eudicotyledons	Primulaceae	*Primula*	*Primula sikkimensis*	GXJ150	JF978048	JN046578	JF943705	JF955770
Eudicotyledons	Primulaceae	*Primula*	*Primula sikkimensis*	GXJ149	JF978049	JN046579	JF943706	JF955771
Eudicotyledons	Primulaceae	*Primula*	*Primula sikkimensis*	GXJ148	JF978050	JN046580	JF943707	JF955772
Eudicotyledons	Primulaceae	*Primula*	*Primula septemloba*	GXJ129	JF978046	JN046576	JF943703	JF955768
Eudicotyledons	Primulaceae	*Primula*	*Primula septemloba*	GXJ130	JF978045	JN046575	JF943702	JF955767
Eudicotyledons	Primulaceae	*Primula*	*Primula rugosa*	GXJ265	JF978044	JN046574	JF943701	JF955764
Eudicotyledons	Primulaceae	*Primula*	*Primula rugosa*	GXJ267	JF978042	JN046572	JF943699	JF955762
Eudicotyledons	Primulaceae	*Primula*	*Primula rugosa*	GXJ266	JF978043	JN046573	JF943700	JF955763
Eudicotyledons	Primulaceae	*Primula*	*Primula pycnoloba*	GXJ239	JF978039	JN046569	JF943696	JF955759
Eudicotyledons	Primulaceae	*Primula*	*Primula pycnoloba*	GXJ237	JF978041	JN046571	JF943698	JF955761
Eudicotyledons	Primulaceae	*Primula*	*Primula pycnoloba*	GXJ238	JF978040	JN046570	JF943697	JF955760
Eudicotyledons	Primulaceae	*Primula*	*Primula pulchella*	GXJ117	JF978034	JN046564	JF943691	JF955754
Eudicotyledons	Primulaceae	*Primula*	*Primula pulchella*	GXJ114	JF978037	JN046567	JF943694	JF955757
Eudicotyledons	Primulaceae	*Primula*	*Primula pulchella*	GXJ116	JF978035	JN046565	JF943692	JF955755
Eudicotyledons	Primulaceae	*Primula*	*Primula pulchella*	GXJ115	JF978036	JN046566	JF943693	JF955756
Eudicotyledons	Primulaceae	*Primula*	*Primula pulchella*	GXJ113	JF978038	JN046568	JF943695	JF955758
Eudicotyledons	Primulaceae	*Primula*	*Primula prattii*	GXJ136-2	JF978032	JN046562	JF943689	JF955752
Eudicotyledons	Primulaceae	*Primula*	*Primula prattii*	GXJ136-1	JF978033	JN046563	JF943690	JF955753
Eudicotyledons	Primulaceae	*Primula*	*Primula polyneura*	GXJ274	JF978029	JN046559	JF943686	JF955749
Eudicotyledons	Primulaceae	*Primula*	*Primula polyneura*	GXJ165	JF978030	JN046560	JF943687	JF955750
Eudicotyledons	Primulaceae	*Primula*	*Primula polyneura*	GXJ164	JF978031	JN046561	JF943688	JF955751
Eudicotyledons	Primulaceae	*Primula*	*Primula partschiana*	GXJ208	JF978028	JN046558	JF943685	JF955748
Eudicotyledons	Primulaceae	*Primula*	*Primula partschiana*	GXJ209	JF978027	JN046557	JF943684	JF955747
Eudicotyledons	Primulaceae	*Primula*	*Primula ovalifolia*	GXJ202	JF978023	JN046553	JF943680	JF955743
Eudicotyledons	Primulaceae	*Primula*	*Primula ovalifolia*	GXJ199	JF978026	JN046556	JF943683	JF955746
Eudicotyledons	Primulaceae	*Primula*	*Primula ovalifolia*	GXJ203	JF978022	JN046552	JF943679	JF955742
Eudicotyledons	Primulaceae	*Primula*	*Primula ovalifolia*	GXJ201	JF978024	JN046554	JF943681	JF955744
Eudicotyledons	Primulaceae	*Primula*	*Primula ovalifolia*	GXJ200	JF978025	JN046555	JF943682	JF955745
Eudicotyledons	Primulaceae	*Primula*	*Primula oreodoxa*	GXJ223	JF978020	JN046550	JF943677	JF955740
Eudicotyledons	Primulaceae	*Primula*	*Primula oreodoxa*	GXJ206	JF978021	JN046551	JF943678	JF955741
Eudicotyledons	Primulaceae	*Primula*	*Primula obconica*	GXJ110	JF978018	JN046548	JF943675	JF955738
Eudicotyledons	Primulaceae	*Primula*	*Primula obconica*	GXJ109	JF978019	JN046549	JF943676	JF955739
Eudicotyledons	Primulaceae	*Primula*	*Primula munroi*	GXJ146	JF978013	JN046543	JF943670	JF955733
Eudicotyledons	Primulaceae	*Primula*	*Primula munroi*	GXJ142	JF978016	JN046546	JF943673	JF955736

Eudicotyledons	Primulaceae	*Primula*	*Primula munroi*	GXJ143	JF978015	JN046545	JF943672	JF955735
Eudicotyledons	Primulaceae	*Primula*	*Primula munroi*	GXJ144	JF978014	JN046544	JF943671	JF955734
Eudicotyledons	Primulaceae	*Primula*	*Primula moupinensis*	GXJ263	JF978008	JN046538	JF943665	JF955728
Eudicotyledons	Primulaceae	*Primula*	*Primula moupinensis*	GXJ262	JF978009	JN046539	JF943666	JF955729
Eudicotyledons	Primulaceae	*Primula*	*Primula moupinensis*	GXJ261	JF978010	JN046540	JF943667	JF955730
Eudicotyledons	Primulaceae	*Primula*	*Primula moupinensis*	GXJ260	JF978011	JN046541	JF943668	JF955731
Eudicotyledons	Primulaceae	*Primula*	*Primula moupinensis*	GXJ259	JF978012	JN046542	JF943669	JF955732
Eudicotyledons	Primulaceae	*Primula*	*Primula membranifolia*	GXJ141-1	JF978007	JN046537	JF943664	JF955727
Eudicotyledons	Primulaceae	*Primula*	*Primula membranifolia*	GXJ141-2	JF978006	JN046536	JF943663	JF955726
Eudicotyledons	Primulaceae	*Primula*	*Primula melanops*	GXJ118-2	JF978004	JN046534	JF943661	JF955724
Eudicotyledons	Primulaceae	*Primula*	*Primula melanops*	GXJ118-1	JF978005	JN046535	JF943662	JF955725
Eudicotyledons	Primulaceae	*Primula*	*Primula malvacea*	GXJ221	JF978000	JN046530	JF943657	JF955720
Eudicotyledons	Primulaceae	*Primula*	*Primula malvacea*	GXJ197	JF978002	JN046532	JF943659	JF955722
Eudicotyledons	Primulaceae	*Primula*	*Primula malvacea*	GXJ196	JF978003	JN046533	JF943660	JF955723
Eudicotyledons	Primulaceae	*Primula*	*Primula malvacea*	GXJ198	JF978001	JN046531	JF943658	JF955721
Eudicotyledons	Primulaceae	*Primula*	*Primula kialensis*	GXJ258	JF977997	JN046528	JF943654	JF955717
Eudicotyledons	Primulaceae	*Primula*	*Primula kialensis*	GXJ257	JF977998	JN046529	JF943655	JF955718
Eudicotyledons	Primulaceae	*Primula*	*Primula heucherifolia*	GXJ205-2	JF977995	JN046526	JF943652	JF955715
Eudicotyledons	Primulaceae	*Primula*	*Primula heucherifolia*	GXJ205-1	JF977996	JN046527	JF943653	JF955716
Eudicotyledons	Primulaceae	*Primula*	*Primula gemmifera*	GXJ252	JF977994	JN046525	JF943651	JF955714
Eudicotyledons	Primulaceae	*Primula*	*Primula gemmifera*	GXJ254	JF977993	JN046523	JF943649	JF955712
Eudicotyledons	Primulaceae	*Primula*	*Primula gemmifera*	GXJ255	JF977992	JN046522	JF943648	JF955711
Eudicotyledons	Primulaceae	*Primula*	*Primula fasciculata*	GXJ250	JF977990	JN046520	JF943646	JF955709
Eudicotyledons	Primulaceae	*Primula*	*Primula fasciculata*	GXJ251	JF977989	JN046519	JF943645	JF955708
Eudicotyledons	Primulaceae	*Primula*	*Primula fasciculata*	GXJ249	JF977991	JN046521	JF943647	JF955710
Eudicotyledons	Primulaceae	*Primula*	*Primula faberi*	GXJ275	JF977987	JN046517	JF943643	JF955706
Eudicotyledons	Primulaceae	*Primula*	*Primula faberi*	GXJ100	JF977988	JN046518	JF943644	JF955707
Eudicotyledons	Primulaceae	*Primula*	*Primula epilosa*	GXJ248	JF977984	JN046514	JF943640	JF955703
Eudicotyledons	Primulaceae	*Primula*	*Primula epilosa*	GXJ247	JF977985	JN046515	JF943641	JF955704
Eudicotyledons	Primulaceae	*Primula*	*Primula epilosa*	GXJ246	JF977986	JN046516	JF943642	JF955705
Eudicotyledons	Primulaceae	*Primula*	*Primula diantha*	GXJ183	JF977983	JN046511	JF943637	JF955700
Eudicotyledons	Primulaceae	*Primula*	*Primula diantha*	GXJ185	JF977982	JN046510	JF943636	JF955699
Eudicotyledons	Primulaceae	*Primula*	*Primula diantha*	GXJ278	JF977981	JN046509	JF943635	JF955698
Eudicotyledons	Primulaceae	*Primula*	*Primula diantha*	GXJ279	JF977980	JN046508	JF943634	JF955697
Eudicotyledons	Primulaceae	*Primula*	*Primula denticulata*	GXJ189	JF977979	JN046507	JF943633	JF955696
Eudicotyledons	Primulaceae	*Primula*	*Primula denticulata*	GXJ193	JF977976	JN046504	JF943630	JF955693
Eudicotyledons	Primulaceae	*Primula*	*Primula denticulata*	GXJ192	JF977977	JN046505	JF943631	JF955694
Eudicotyledons	Primulaceae	*Primula*	*Primula denticulata*	GXJ190	JF977978	JN046506	JF943632	JF955695
Eudicotyledons	Primulaceae	*Primula*	*Primula deflexa*	GXJ140	JF977972	JN046500	JF943626	JF955689
Eudicotyledons	Primulaceae	*Primula*	*Primula deflexa*	GXJ137	JF977975	JN046503	JF943629	JF955692

Eudicotyledons	Primulaceae	*Primula*	*Primula deflexa*	GXJ138	JF977974	JN046502	JF943628	JF955691
Eudicotyledons	Primulaceae	*Primula*	*Primula deflexa*	GXJ139	JF977973	JN046501	JF943627	JF955690
Eudicotyledons	Primulaceae	*Primula*	*Primula chionantha*	GXJ162	JF977968	JN046496	JF943622	JF955685
Eudicotyledons	Primulaceae	*Primula*	*Primula chionantha*	GXJ160	JF977970	JN046498	JF943624	JF955687
Eudicotyledons	Primulaceae	*Primula*	*Primula chionantha*	GXJ161	JF977969	JN046497	JF943623	JF955686
Eudicotyledons	Primulaceae	*Primula*	*Primula chionantha*	GXJ163	JF977967	JN046495	JF943621	JF955684
Eudicotyledons	Primulaceae	*Primula*	*Primula chapaensis*	GXJ211	JF977965	JN046493	JF943619	JF955682
Eudicotyledons	Primulaceae	*Primula*	*Primula chapaensis*	GXJ210	JF977966	JN046494	JF943620	JF955683
Eudicotyledons	Primulaceae	*Primula*	*Primula calderiana*	GXJ244	JF977960	JN046488	JF943614	JF955677
Eudicotyledons	Primulaceae	*Primula*	*Primula calderiana*	GXJ281	JF977958	JN046486	JF943612	JF955675
Eudicotyledons	Primulaceae	*Primula*	*Primula calderiana*	GXJ245	JF977959	JN046487	JF943613	JF955676
Eudicotyledons	Primulaceae	*Primula*	*Primula calderiana*	GXJ243	JF977961	JN046489	JF943615	JF955678
Eudicotyledons	Primulaceae	*Primula*	*Primula boreiocalliantha*	GXJ179	JF977957	JN046485	JF943611	JF955674
Eudicotyledons	Primulaceae	*Primula*	*Primula boreiocalliantha*	GXJ217	JF977956	JN046484	JF943610	JF955673
Eudicotyledons	Primulaceae	*Primula*	*Primula blinii*	GXJ135	JF977952	JN046480	JF943606	JF955669
Eudicotyledons	Primulaceae	*Primula*	*Primula blinii*	GXJ131	JF977955	JN046483	JF943609	JF955672
Eudicotyledons	Primulaceae	*Primula*	*Primula blinii*	GXJ133	JF977953	JN046481	JF943607	JF955670
Eudicotyledons	Primulaceae	*Primula*	*Primula blinii*	GXJ132	JF977954	JN046482	JF943608	JF955671
Eudicotyledons	Primulaceae	*Primula*	*Primula blattariformis*	GXJ220	JF977949	JN046477	JF943603	JF955666
Eudicotyledons	Primulaceae	*Primula*	*Primula blattariformis*	GXJ195	JF977950	JN046478	JF943604	JF955667
Eudicotyledons	Primulaceae	*Primula*	*Primula blattariformis*	GXJ194	JF977951	JN046479	JF943605	JF955668
Eudicotyledons	Primulaceae	*Primula*	*Primula bellidifolia*	GXJ241	JF977947	JN046475	JF943601	JF955664
Eudicotyledons	Primulaceae	*Primula*	*Primula bellidifolia*	GXJ242	JF977946	JN046474	JF943600	JF955663
Eudicotyledons	Primulaceae	*Primula*	*Primula bellidifolia*	GXJ240	JF977948	JN046476	JF943602	JF955665
Eudicotyledons	Primulaceae	*Primula*	*Primula bella*	GXJ096	JF977944	JN046472	JF943598	JF955661
Eudicotyledons	Primulaceae	*Primula*	*Primula bella*	GXJ097	JF977943	JN046471	JF943597	JF955660
Eudicotyledons	Primulaceae	*Primula*	*Primula bella*	GXJ095	JF977945	JN046473	JF943599	JF955662
Eudicotyledons	Primulaceae	*Primula*	*Primula aromatica*	GXJ272	JF977942	JN046470	JF943596	JF955659
Eudicotyledons	Primulaceae	*Primula*	*Primula aromatica*	GXJ273	JF977941	JN046469	JF943595	JF955658
Eudicotyledons	Primulaceae	*Primula*	*Primula amethystina*	GXJ121	JF977940	JN046468	JF943594	JF955657
Eudicotyledons	Primulaceae	*Primula*	*Primula amethystina*	GXJ120	JF977938	JN046466	JF943592	JF955655
Eudicotyledons	Primulaceae	*Primula*	*Primula amethystina*	GXJ119	JF977939	JN046467	JF943593	JF955656
Eudicotyledons	Primulaceae	*Primula*	*Primula alpicola*	GXJ232	JF977937	JN046465	JF943591	JF955654
Eudicotyledons	Primulaceae	*Primula*	*Primula alpicola*	GXJ233	JF977936	JN046464	JF943590	JF955653
Eudicotyledons	Primulaceae	*Primula*	*Primula alpicola*	GXJ236	JF977933	JN046461	JF943587	JF955650
Eudicotyledons	Primulaceae	*Primula*	*Primula alpicola*	GXJ234	JF977935	JN046463	JF943589	JF955652
Eudicotyledons	Primulaceae	*Primula*	*Primula alpicola*	GXJ235	JF977934	JN046462	JF943588	JF955651
Eudicotyledons	Primulaceae	*Primula*	*Primula agleniana*	GXJ229	JF977932	JN046460	JF943586	JF955649
Eudicotyledons	Primulaceae	*Primula*	*Primula agleniana*	GXJ230	JF977931	JN046459	JF943585	JF955648
Eudicotyledons	Primulaceae	*Primula*	*Primula agleniana*	GXJ231	JF977930	JN046458	JF943584	JF955647

Eudicotyledons	Apiaceae	*Pleurospermu*	*Pleurospermum uralense*	NASLQX033	JF977837	JN046395	JF943471	JF955563
Eudicotyledons	Apiaceae	*Pleurospermu*	*Pleurospermum uralense*	NASLQX032	JF977838	JN046396	JF943472	JF955564
Eudicotyledons	Apiaceae	*Pleurospermu*	*Pleurospermum uralense*	NASLQX031	JF977839	JN046397	JF943473	JF955565
Eudicotyledons	Apiaceae	*Pleurospermu*	*Pleurospermum uralense*	NASLQX037	JF977836	JN046394	JF943470	JF955562
Eudicotyledons	Apiaceae	*Pleurospermu*	*Pleurospermum uralense*	NASLQX039	JF977835	JN046393	JF943469	JF955561
Eudicotyledons	Apiaceae	*Pleurospermu*	*Pleurospermum giraldii*	NASLQX051	JF977834	JN046392	JF943468	JF955560
Eudicotyledons	Apiaceae	*Pleurospermu*	*Pleurospermum giraldii*	NASLQX053	JF977832	JN046390	JF943466	JF955558
Eudicotyledons	Apiaceae	*Pleurospermu*	*Pleurospermum giraldii*	NASLQX052	JF977833	JN046391	JF943467	JF955559
Eudicotyledons	Apiaceae	*Pleurospermu*	*Pleurospermum giraldii*	NASLQX054	JF977831	JN046389	JF943465	JF955557
Eudicotyledons	Apiaceae	*Pleurospermu*	*Pleurospermum cristatum*	NASLQX050	JF977827	JN046386	JF943461	JF955556
Eudicotyledons	Apiaceae	*Pleurospermu*	*Pleurospermum cristatum*	NASLQX059	JF977826	JN046385	JF943460	JF955555
Eudicotyledons	Apiaceae	*Peucedanum*	*Peucedanum wawrae*	NASLQX011	JF977825	JN046238	JF943307	JF955414
Eudicotyledons	Apiaceae	*Peucedanum*	*Peucedanum wawrae*	NASLQX013	JF977823	JN046236	JF943305	JF955412
Eudicotyledons	Apiaceae	*Peucedanum*	*Peucedanum wawrae*	NASLQX012	JF977824	JN046237	JF943306	JF955413
Eudicotyledons	Apiaceae	*Peucedanum*	*Peucedanum medicum*	NASLQX010	JF977813	JN046227	JF943295	JF955402
Eudicotyledons	Apiaceae	*Peucedanum*	*Peucedanum medicum*	NASLQX061	JF977812	JN046226	JF943294	JF955401
Eudicotyledons	Apiaceae	*Peucedanum*	*Peucedanum medicum*	NASLQX062	JF977811	JN046225	JF943293	JF955400
Eudicotyledons	Apiaceae	*Peucedanum*	*Peucedanum medicum*	NASLQX003	JF977815	JN046228	JF943297	JF955404
Eudicotyledons	Apiaceae	*Peucedanum*	*Peucedanum ledebourielloides*	NASLQX049	JF977808	JN046222	JF943290	JF955397
Eudicotyledons	Apiaceae	*Peucedanum*	*Peucedanum ledebourielloides*	NASLQX015	JF977809	JN046223	JF943291	JF955398
Eudicotyledons	Apiaceae	*Peucedanum*	*Peucedanum ledebourielloides*	NASLQX014	JF977810	JN046224	JF943292	JF955399
Eudicotyledons	Apiaceae	*Peucedanum*	*Peucedanum japonicum*	NASLQX006	JF977806	JN046220	JF943288	JF955395
Eudicotyledons	Apiaceae	*Peucedanum*	*Peucedanum japonicum*	NASLQX007	JF977805	JN046219	JF943287	JF955394
Eudicotyledons	Apiaceae	*Peucedanum*	*Peucedanum japonicum*	NASLQX005	JF977807	JN046221	JF943289	JF955396
Eudicotyledons	Apiaceae	*Peucedanum*	*Peucedanum caespitosum*	NASLQX065	JF977802	JN046216	JF943284	JF955391
Eudicotyledons	Apiaceae	*Peucedanum*	*Peucedanum caespitosum*	NASLQX035	JF977803	JN046217	JF943285	JF955392
Eudicotyledons	Apiaceae	*Peucedanum*	*Peucedanum caespitosum*	NASLQX034	JF977804	JN046218	JF943286	JF955393
Eudicotyledons	Apiaceae	*Peucedanum*	*Peucedanum ampliatum*	NASLQX020	JF977801	JN046215	JF943283	JF955390
Eudicotyledons	Apiaceae	*Peucedanum*	*Peucedanum ampliatum*	NASLQX021	JF977800	JN046214	JF943282	JF955389
Eudicotyledons	Apiaceae	*Peucedanum*	*Peucedanum ampliatum*	NASLQX022	JF977799	JN046213	JF943281	JF955388
Eudicotyledons	Orobanchaceae	*Pedicularis*	*Pedicularis vialii*	P121-2-HW-LIDZ1275	JF977782	JN046195	JF943265	JF955371
Eudicotyledons	Orobanchaceae	*Pedicularis*	*Pedicularis vialii*	P121-1-HW-HW10191	JF977783	JN046196	JF943266	JF955372
Eudicotyledons	Orobanchaceae	*Pedicularis*	*Pedicularis variegata*	P120-1-HW-07ML101B	JF977781	JN046194	JF943264	JF955370
Eudicotyledons	Orobanchaceae	*Pedicularis*	*Pedicularis variegata*	P120-2-HW-07ML101A	JF977780	JN046193	JF943263	JF955369
Eudicotyledons	Orobanchaceae	*Pedicularis*	*Pedicularis urceolata*	P119-3-HW-HW10294	JF977776	JN046189	JF943259	JF955365
Eudicotyledons	Orobanchaceae	*Pedicularis*	*Pedicularis urceolata*	P119-HW-LIDZ1211	JF977778	JN046191	JF943261	JF955367
Eudicotyledons	Orobanchaceae	*Pedicularis*	*Pedicularis urceolata*	P119-5-HW-LIDZ1115	JF977779	JN046192	JF943262	JF955368

Eudicotyledons	Orobanchaceae	*Pedicularis*	*Pedicularis urceolata*	P119-2-HW-HW10276	JF977777	JN046190	JF943260	JF955366
Eudicotyledons	Orobanchaceae	*Pedicularis*	*Pedicularis urceolata*	P119-4-HW-HW10315	JF977775	JN046188	JF943258	JF955364
Eudicotyledons	Orobanchaceae	*Pedicularis*	*Pedicularis umbelliformis*	P178-1-HW-HW10134	JF977774	JN046187	JF943257	JF955363
Eudicotyledons	Orobanchaceae	*Pedicularis*	*Pedicularis umbelliformis*	P178-2-HW-HW10176	JF977773	JN046186	JF943256	JF955362
Eudicotyledons	Orobanchaceae	*Pedicularis*	*Pedicularis tsekouensis*	P187-1-HW-HW10167A	JF977772	JN046185	JF943255	JF955361
Eudicotyledons	Orobanchaceae	*Pedicularis*	*Pedicularis tsekouensis*	P187-3-HW-HW10167C	JF977770	JN046183	JF943253	JF955359
Eudicotyledons	Orobanchaceae	*Pedicularis*	*Pedicularis tsekouensis*	P187-2-HW-HW10167B	JF977771	JN046184	JF943254	JF955360
Eudicotyledons	Orobanchaceae	*Pedicularis*	*Pedicularis tricolor*	P118-3-HW-LIDZ1227	JF977768	JN046181	JF943251	JF955357
Eudicotyledons	Orobanchaceae	*Pedicularis*	*Pedicularis tricolor*	P118-HW-YWB069	JF977767	JN046180	JF943250	JF955356
Eudicotyledons	Orobanchaceae	*Pedicularis*	*Pedicularis tricolor*	P118-2-HW-HW10149	JF977769	JN046182	JF943252	JF955358
Eudicotyledons	Orobanchaceae	*Pedicularis*	*Pedicularis trichoglossa*	P116-2-HW-HW10256	JF977766	JN046179	JF943249	JF955355
Eudicotyledons	Orobanchaceae	*Pedicularis*	*Pedicularis trichoglossa*	P116-HW-081468	JF977765	JN046178	JF943248	JF955354
Eudicotyledons	Orobanchaceae	*Pedicularis*	*Pedicularis tongolensis*	P115-4-HW-HW10229	JF977763	JN046176	JF943246	JF955352
Eudicotyledons	Orobanchaceae	*Pedicularis*	*Pedicularis tongolensis*	P115-5-HW-HW10308	JF977762	JN046175	JF943245	JF955351
Eudicotyledons	Orobanchaceae	*Pedicularis*	*Pedicularis tongolensis*	P115-2-HW-HW10139	JF977764	JN046177	JF943247	JF955353
Eudicotyledons	Orobanchaceae	*Pedicularis*	*Pedicularis tongolensis*	P115-HW-LIDZ1251	JF977760	JN046173	JF943243	JF955349
Eudicotyledons	Orobanchaceae	*Pedicularis*	*Pedicularis tongolensis*	P115-6-HW-LWL096	JF977761	JN046174	JF943244	JF955350
Eudicotyledons	Orobanchaceae	*Pedicularis*	*Pedicularis tibetica*	P114-HW-LIDZ1085	JF977756	JN046169	JF943239	JF955345
Eudicotyledons	Orobanchaceae	*Pedicularis*	*Pedicularis tibetica*	P114-4-HW-LIDZ1155	JF977757	JN046170	JF943240	JF955346
Eudicotyledons	Orobanchaceae	*Pedicularis*	*Pedicularis tibetica*	P114-3-HW-HW10333	JF977758	JN046171	JF943241	JF955347
Eudicotyledons	Orobanchaceae	*Pedicularis*	*Pedicularis tibetica*	P114-2-HW-HW10309	JF977759	JN046172	JF943242	JF955348
Eudicotyledons	Orobanchaceae	*Pedicularis*	*Pedicularis thamnophila*	P113-4-HW-LIDZ1019	JF977752	JN046165	JF943235	JF955341
Eudicotyledons	Orobanchaceae	*Pedicularis*	*Pedicularis thamnophila*	P113-2-HW-LIDZ1014	JF977754	JN046167	JF943237	JF955343
Eudicotyledons	Orobanchaceae	*Pedicularis*	*Pedicularis thamnophila*	P113-3-HW-LIDZ1016	JF977753	JN046166	JF943236	JF955342
Eudicotyledons	Orobanchaceae	*Pedicularis*	*Pedicularis thamnophila*	P113-1-HW-LIDZ0990	JF977755	JN046168	JF943238	JF955344
Eudicotyledons	Orobanchaceae	*Pedicularis*	*Pedicularis thamnophila*	P113-5-HW-LIDZ1002	JF977751	JN046164	JF943234	JF955340
Eudicotyledons	Orobanchaceae	*Pedicularis*	*Pedicularis tenuisecta*	P112-2-HW-2004204	JF977750	JN046163	JF943233	JF955339
Eudicotyledons	Orobanchaceae	*Pedicularis*	*Pedicularis tenuisecta*	P112-3-HW-HW10160	JF977749	JN046162	JF943232	JF955338
Eudicotyledons	Orobanchaceae	*Pedicularis*	*Pedicularis tenuisecta*	P112-5-HW-LIDZ0995	JF977748	JN046161	JF943231	JF955337
Eudicotyledons	Orobanchaceae	*Pedicularis*	*Pedicularis tachanensis*	P110-4-HW-LH8484	JF977744	JN046158	JF943227	JF955334
Eudicotyledons	Orobanchaceae	*Pedicularis*	*Pedicularis tachanensis*	P110-5-HW-LIDZ1062	JF977743	JN046157	JF943226	JF955333
Eudicotyledons	Orobanchaceae	*Pedicularis*	*Pedicularis tachanensis*	P110-2-HW-HW10355	JF977746	JN046160	JF943229	JF955336
Eudicotyledons	Orobanchaceae	*Pedicularis*	*Pedicularis tachanensis*	P110-3-HW-HW10370	JF977745	JN046159	JF943228	JF955335
Eudicotyledons	Orobanchaceae	*Pedicularis*	*Pedicularis superba*	P109-4-HW-LIDZ1266	JF977740	JN046154	JF943223	JF955330
Eudicotyledons	Orobanchaceae	*Pedicularis*	*Pedicularis superba*	P109-3-HW-LIDZ1074	JF977741	JN046155	JF943224	JF955331
Eudicotyledons	Orobanchaceae	*Pedicularis*	*Pedicularis superba*	P109-5-HW-LIDZ1578	JF977739	JN046153	JF943222	JF955329
Eudicotyledons	Orobanchaceae	*Pedicularis*	*Pedicularis superba*	P109-1-HW-LIDZ1007	JF977742	JN046156	JF943225	JF955332
Eudicotyledons	Orobanchaceae	*Pedicularis*	*Pedicularis sorbifolia*	P100-2-HW-LIDZ1176C	JF977732	JN046146	JF943215	JF955322
Eudicotyledons	Orobanchaceae	*Pedicularis*	*Pedicularis sorbifolia*	P100-3-HW-LIDZ1176A	JF977731	JN046145	JF943214	JF955321
Eudicotyledons	Orobanchaceae	*Pedicularis*	*Pedicularis sorbifolia*	P100-1-HW-LIDZ1176B	JF977733	JN046147	JF943216	JF955323

Eudicotyledons	Orobanchaceae	*Pedicularis*	*Pedicularis sikangensis*	P161-3-HW-HW10332	JF977720	JN046134	JF943203	JF955311
Eudicotyledons	Orobanchaceae	*Pedicularis*	*Pedicularis sikangensis*	P161-2-HW-HW10318	JF977721	JN046135	JF943204	JF955312
Eudicotyledons	Orobanchaceae	*Pedicularis*	*Pedicularis sikangensis*	P161-1-HW-HW10262	JF977722	JN046136	JF943205	JF955313
Eudicotyledons	Orobanchaceae	*Pedicularis*	*Pedicularis sigmoidea*	P96-2-HW-HW10015	JF977719	JN046133	JF943202	JF955310
Eudicotyledons	Orobanchaceae	*Pedicularis*	*Pedicularis sigmoidea*	P96-HW-LIDZ1584	JF977718	JN046132	JF943201	JF955309
Eudicotyledons	Orobanchaceae	*Pedicularis*	*Pedicularis salviiflora*	P94-4-HW-HW10057	JF977715	JN046129	JF943198	JF955306
Eudicotyledons	Orobanchaceae	*Pedicularis*	*Pedicularis salviiflora*	P94-2-HW-YWB269	JF977717	JN046131	JF943200	JF955308
Eudicotyledons	Orobanchaceae	*Pedicularis*	*Pedicularis salviiflora*	P94-3-HW-HW10033	JF977716	JN046130	JF943199	JF955307
Eudicotyledons	Orobanchaceae	*Pedicularis*	*Pedicularis rupicola*	P93-5-HW-081277	JF977711	JN046125	JF943194	JF955302
Eudicotyledons	Orobanchaceae	*Pedicularis*	*Pedicularis rupicola*	P93-HW-08916	JF977708	JN046122	JF943191	JF955299
Eudicotyledons	Orobanchaceae	*Pedicularis*	*Pedicularis rupicola*	P93-3-HW-LIDZ1219	JF977713	JN046127	JF943196	JF955304
Eudicotyledons	Orobanchaceae	*Pedicularis*	*Pedicularis rupicola*	P93-7-HW-GLM102879	JF977709	JN046123	JF943192	JF955300
Eudicotyledons	Orobanchaceae	*Pedicularis*	*Pedicularis rupicola*	P93-4-HW-08907	JF977712	JN046126	JF943195	JF955303
Eudicotyledons	Orobanchaceae	*Pedicularis*	*Pedicularis rupicola*	P93-6-HW-HW10128	JF977710	JN046124	JF943193	JF955301
Eudicotyledons	Orobanchaceae	*Pedicularis*	*Pedicularis rupicola*	P93-2-HW-LIDZ1236	JF977714	JN046128	JF943197	JF955305
Eudicotyledons	Orobanchaceae	*Pedicularis*	*Pedicularis roylei*	P92-2-HW-HW10266	JF977707	JN046121	JF943190	JF955298
Eudicotyledons	Orobanchaceae	*Pedicularis*	*Pedicularis roylei*	P92-3-HW-HW10288	JF977706	JN046120	JF943189	JF955297
Eudicotyledons	Orobanchaceae	*Pedicularis*	*Pedicularis rizhaoensis*	P91-1-HW-LIDZ1254B	JF977705	JN046119	JF943188	JF955296
Eudicotyledons	Orobanchaceae	*Pedicularis*	*Pedicularis rizhaoensis*	P91-2-HW-LIDZ1254A	JF977704	JN046118	JF943187	JF955295
Eudicotyledons	Orobanchaceae	*Pedicularis*	*Pedicularis rigida*	P90-3-HW-LIDZ0950	JF977701	JN046115	JF943184	JF955292
Eudicotyledons	Orobanchaceae	*Pedicularis*	*Pedicularis rigida*	P90-4-HW-HW10040	JF977700	JN046114	JF943183	JF955291
Eudicotyledons	Orobanchaceae	*Pedicularis*	*Pedicularis rigida*	P90-2-HW-LIDZ0947	JF977702	JN046116	JF943185	JF955293
Eudicotyledons	Orobanchaceae	*Pedicularis*	*Pedicularis rigida*	P90-1-HW-05123	JF977703	JN046117	JF943186	JF955294
Eudicotyledons	Orobanchaceae	*Pedicularis*	*Pedicularis rhynchodonta*	P179-2-HW-HW10283	JF977698	JN046112	JF943181	JF955289
Eudicotyledons	Orobanchaceae	*Pedicularis*	*Pedicularis rhynchodonta*	P179-1-HW-HW10261	JF977699	JN046113	JF943182	JF955290
Eudicotyledons	Orobanchaceae	*Pedicularis*	*Pedicularis rhynchodonta*	P179-5-HW-HW10272	JF977695	JN046109	JF943178	JF955286
Eudicotyledons	Orobanchaceae	*Pedicularis*	*Pedicularis rhynchodonta*	P179-4-HW-HW10321	JF977696	JN046110	JF943179	JF955287
Eudicotyledons	Orobanchaceae	*Pedicularis*	*Pedicularis rhynchodonta*	P179-3-HW-HW10296	JF977697	JN046111	JF943180	JF955288
Eudicotyledons	Orobanchaceae	*Pedicularis*	*Pedicularis rhodotricha*	P80-2-HW-08846	JF977694	JN046108	JF943177	JF955285
Eudicotyledons	Orobanchaceae	*Pedicularis*	*Pedicularis rhodotricha*	P80-5-HW-LIDZ1238	JF977691	JN046105	JF943174	JF955282
Eudicotyledons	Orobanchaceae	*Pedicularis*	*Pedicularis rhodotricha*	P80-HW-LIDZ1117	JF977690	JN046104	JF943173	JF955281
Eudicotyledons	Orobanchaceae	*Pedicularis*	*Pedicularis rhodotricha*	P80-4-HW-LIDZ1135	JF977692	JN046106	JF943175	JF955283
Eudicotyledons	Orobanchaceae	*Pedicularis*	*Pedicularis rhodotricha*	P80-3-HW-03052	JF977693	JN046107	JF943176	JF955284
Eudicotyledons	Orobanchaceae	*Pedicularis*	*Pedicularis rhinanthoides*	P79-1-HW-2004249	JF977689	JN046103	JF943172	JF955280
Eudicotyledons	Orobanchaceae	*Pedicularis*	*Pedicularis rhinanthoides*	P79-2-HW-08915	JF977688	JN046102	JF943171	JF955279
Eudicotyledons	Orobanchaceae	*Pedicularis*	*Pedicularis rhinanthoides*	P79-3-HW-08938	JF977687	JN046101	JF943170	JF955278
Eudicotyledons	Orobanchaceae	*Pedicularis*	*Pedicularis rhinanthoides*	P79-5-HW-LIDZ1158	JF977685	JN046099	JF943168	JF955276
Eudicotyledons	Orobanchaceae	*Pedicularis*	*Pedicularis rhinanthoides*	P79-4-HW-LIDZ1215	JF977686	JN046100	JF943169	JF955277
Eudicotyledons	Orobanchaceae	*Pedicularis*	*Pedicularis rex*	P78-5-HW-LIDZ1193	JF977680	JN046094	JF943163	JF955271
Eudicotyledons	Orobanchaceae	*Pedicularis*	*Pedicularis rex*	P78-1-HW-LIDZ0962	JF977684	JN046098	JF943167	JF955275

Eudicotyledons	Orobanchaceae	*Pedicularis*	*Pedicularis rex*	P78-3-HW-LIDZ0983	JF977682	JN046096	JF943165	JF955273
Eudicotyledons	Orobanchaceae	*Pedicularis*	*Pedicularis rex*	P78-4-HW-LIDZ1011	JF977681	JN046095	JF943164	JF955272
Eudicotyledons	Orobanchaceae	*Pedicularis*	*Pedicularis rex*	P78-2-HW-LIDZ0979	JF977683	JN046097	JF943166	JF955274
Eudicotyledons	Orobanchaceae	*Pedicularis*	*Pedicularis resupinata*	P77-2-HW-HJ019	JF977679	JN046093	JF943162	JF955270
Eudicotyledons	Orobanchaceae	*Pedicularis*	*Pedicularis resupinata*	P77-HW-LIDZ544	JF977677	JN046091	JF943160	JF955268
Eudicotyledons	Orobanchaceae	*Pedicularis*	*Pedicularis resupinata*	P77-4-HW-LIDZ519	JF977678	JN046092	JF943161	JF955269
Eudicotyledons	Orobanchaceae	*Pedicularis*	*Pedicularis pseudoversicolor*	P183-2-HW-HW10124	JF977675	JN046089	JF943158	JF955266
Eudicotyledons	Orobanchaceae	*Pedicularis*	*Pedicularis pseudoversicolor*	P183-1-HW-HW10099	JF977676	JN046090	JF943159	JF955267
Eudicotyledons	Orobanchaceae	*Pedicularis*	*Pedicularis pseudoversicolor*	P183-3-HW-HW10165	JF977674	JN046088	JF943157	JF955265
Eudicotyledons	Orobanchaceae	*Pedicularis*	*Pedicularis pseudomelampyriflora*	P74-4-HW-LIDZ0993	JF977671	JN046085	JF943154	JF955262
Eudicotyledons	Orobanchaceae	*Pedicularis*	*Pedicularis pseudomelampyriflora*	P74-5-HW-LIDZ1000	JF977670	JN046084	JF943153	JF955261
Eudicotyledons	Orobanchaceae	*Pedicularis*	*Pedicularis pseudomelampyriflora*	P74-HW-08873	JF977669	JN046083	JF943152	JF955260
Eudicotyledons	Orobanchaceae	*Pedicularis*	*Pedicularis pseudomelampyriflora*	P74-3-HW-HW10213	JF977672	JN046086	JF943155	JF955263
Eudicotyledons	Orobanchaceae	*Pedicularis*	*Pedicularis pseudomelampyriflora*	P74-2-HW-LIDZ1292	JF977673	JN046087	JF943156	JF955264
Eudicotyledons	Orobanchaceae	*Pedicularis*	*Pedicularis przewalskii*	P73-3-HW-HW10265	JF977667	JN046081	JF943150	JF955258
Eudicotyledons	Orobanchaceae	*Pedicularis*	*Pedicularis przewalskii*	P73-2-HW-HW10198	JF977668	JN046082	JF943151	JF955259
Eudicotyledons	Orobanchaceae	*Pedicularis*	*Pedicularis przewalskii*	P73-HW-LIDZ1235	JF977666	JN046080	JF943149	JF955257
Eudicotyledons	Orobanchaceae	*Pedicularis*	*Pedicularis oxycarpa*	P71-3-HW-Yang002	JF977663	JN046077	JF943146	JF955254
Eudicotyledons	Orobanchaceae	*Pedicularis*	*Pedicularis oxycarpa*	P71-2-HW-LIDZ1038	JF977664	JN046078	JF943147	JF955255
Eudicotyledons	Orobanchaceae	*Pedicularis*	*Pedicularis oxycarpa*	P71-1-HW-LIDZ1299	JF977665	JN046079	JF943148	JF955256
Eudicotyledons	Orobanchaceae	*Pedicularis*	*Pedicularis oxycarpa*	P71-6-HW-HW10372	JF977660	JN046074	JF943143	JF955252
Eudicotyledons	Orobanchaceae	*Pedicularis*	*Pedicularis oxycarpa*	P71-4-HW-LIDZ1536	JF977662	JN046076	JF943145	JF955253
Eudicotyledons	Orobanchaceae	*Pedicularis*	*Pedicularis orthocoryne*	P130-2-HW-GLM102994	JF977659	JN046073	JF943142	JF955251
Eudicotyledons	Orobanchaceae	*Pedicularis*	*Pedicularis orthocoryne*	P130-HW-YWB506	JF977658	JN046072	JF943141	JF955250
Eudicotyledons	Orobanchaceae	*Pedicularis*	*Pedicularis oederi*	P69-3-HW-GLM103003	JF977656	JN046070	JF943139	JF955248
Eudicotyledons	Orobanchaceae	*Pedicularis*	*Pedicularis oederi*	P69-HW-03053	JF977654	JN046068	JF943137	JF955246
Eudicotyledons	Orobanchaceae	*Pedicularis*	*Pedicularis oederi*	P69-2-HW-GLM102876	JF977657	JN046071	JF943140	JF955249
Eudicotyledons	Orobanchaceae	*Pedicularis*	*Pedicularis oederi*	P69-4-HW-GLM103015	JF977655	JN046069	JF943138	JF955247
Eudicotyledons	Orobanchaceae	*Pedicularis*	*Pedicularis nigra*	P68-2-HW-XCL159	JF977653	JN046067	JF943136	JF955245
Eudicotyledons	Orobanchaceae	*Pedicularis*	*Pedicularis nigra*	P68-HW-LIDZ1331	JF977652	JN046066	JF943135	JF955244
Eudicotyledons	Orobanchaceae	*Pedicularis*	*Pedicularis mussotii*	P65-3-HW-HW10310	JF977650	JN046064	JF943133	JF955242
Eudicotyledons	Orobanchaceae	*Pedicularis*	*Pedicularis mussotii*	P65-HW-LIDZ1088	JF977647	JN046061	JF943130	JF955239

Eudicotyledons	Orobanchaceae	*Pedicularis*	*Pedicularis mussotii*	P65-4-HW-HW10328	JF977649	JN046063	JF943132	JF955241
Eudicotyledons	Orobanchaceae	*Pedicularis*	*Pedicularis mussotii*	P65-5-HW-LIDZ1134	JF977648	JN046062	JF943131	JF955240
Eudicotyledons	Orobanchaceae	*Pedicularis*	*Pedicularis mussotii*	P65-2-HW-HW10295	JF977651	JN046065	JF943134	JF955243
Eudicotyledons	Orobanchaceae	*Pedicularis*	*Pedicularis megalantha*	P61-2-HW-081122	JF977646	JN046060	JF943129	JF955238
Eudicotyledons	Orobanchaceae	*Pedicularis*	*Pedicularis megalantha*	P61-HW-081191	JF977645	JN046059	JF943128	JF955237
Eudicotyledons	Orobanchaceae	*Pedicularis*	*Pedicularis maxonii*	P60-2-HW-HP9542	JF977643	JN046057	JF943126	JF955235
Eudicotyledons	Orobanchaceae	*Pedicularis*	*Pedicularis maxonii*	P60-1-HW-LIDZ1579	JF977644	JN046058	JF943127	JF955236
Eudicotyledons	Orobanchaceae	*Pedicularis*	*Pedicularis macrosiphon*	P165-2-HW-GLM092443	JF977642	JN046056	JF943125	JF955234
Eudicotyledons	Orobanchaceae	*Pedicularis*	*Pedicularis macrosiphon*	P165-HW-GLM07486	JF977640	JN046054	JF943123	JF955232
Eudicotyledons	Orobanchaceae	*Pedicularis*	*Pedicularis macrosiphon*	P165-3-HW-GLM102963	JF977641	JN046055	JF943124	JF955233
Eudicotyledons	Orobanchaceae	*Pedicularis*	*Pedicularis macrorhyncha*	P185-2-HW-HW10125B	JF977638	JN046052	JF943121	JF955230
Eudicotyledons	Orobanchaceae	*Pedicularis*	*Pedicularis macrorhyncha*	P185-1-HW-HW10125A	JF977639	JN046053	JF943122	JF955231
Eudicotyledons	Orobanchaceae	*Pedicularis*	*Pedicularis lyrata*	P59-2-HW-LIDZ1109	JF977636	JN046050	JF943119	JF955228
Eudicotyledons	Orobanchaceae	*Pedicularis*	*Pedicularis lyrata*	P59-1-HW-LIDZ1093	JF977637	JN046051	JF943120	JF955229
Eudicotyledons	Orobanchaceae	*Pedicularis*	*Pedicularis lyrata*	P59-5-HW-LIDZ1200	JF977633	JN046047	JF943116	JF955225
Eudicotyledons	Orobanchaceae	*Pedicularis*	*Pedicularis lyrata*	P59-3-HW-LIDZ1175	JF977635	JN046049	JF943118	JF955227
Eudicotyledons	Orobanchaceae	*Pedicularis*	*Pedicularis lyrata*	P59-4-HW-LIDZ1083	JF977634	JN046048	JF943117	JF955226
Eudicotyledons	Orobanchaceae	*Pedicularis*	*Pedicularis lyrata*	P59-7-HW-LIDZ1206	JF977631	JN046045	JF943114	JF955223
Eudicotyledons	Orobanchaceae	*Pedicularis*	*Pedicularis lyrata*	P59-6-HW-08914	JF977632	JN046046	JF943115	JF955224
Eudicotyledons	Orobanchaceae	*Pedicularis*	*Pedicularis lutescens*	P58-6-HW-HW10329	JF977626	JN046040	JF943109	JF955218
Eudicotyledons	Orobanchaceae	*Pedicularis*	*Pedicularis lutescens*	P58-3-HW-LIDZ1009	JF977628	JN046042	JF943111	JF955220
Eudicotyledons	Orobanchaceae	*Pedicularis*	*Pedicularis lutescens*	P58-5-HW-HW10203	JF977627	JN046041	JF943110	JF955219
Eudicotyledons	Orobanchaceae	*Pedicularis*	*Pedicularis lutescens*	P58-1-HW-LIDZ1267	JF977630	JN046044	JF943113	JF955222
Eudicotyledons	Orobanchaceae	*Pedicularis*	*Pedicularis lutescens*	P58-2-HW-YWB005	JF977629	JN046043	JF943112	JF955221
Eudicotyledons	Orobanchaceae	*Pedicularis*	*Pedicularis longipes*	P57-2-HW-HW10305	JF977625	JN046039	JF943108	JF955217
Eudicotyledons	Orobanchaceae	*Pedicularis*	*Pedicularis longipes*	P57-HW-LIDZ1205	JF977624	JN046038	JF943107	JF955216
Eudicotyledons	Orobanchaceae	*Pedicularis*	*Pedicularis longicaulis*	P55-HW-LIDZ1303	JF977615	JN046029	JF943098	JF955207
Eudicotyledons	Orobanchaceae	*Pedicularis*	*Pedicularis longicaulis*	P55-2-HW-LIDZ0986	JF977618	JN046032	JF943101	JF955210
Eudicotyledons	Orobanchaceae	*Pedicularis*	*Pedicularis longicaulis*	P55-4-HW-YWB270	JF977616	JN046030	JF943099	JF955208
Eudicotyledons	Orobanchaceae	*Pedicularis*	*Pedicularis longicaulis*	P55-3-HW-LIDZ1333	JF977617	JN046031	JF943100	JF955209
Eudicotyledons	Orobanchaceae	*Pedicularis*	*Pedicularis lecomtei*	P52-HW-LIDZ1257A	JF977612	JN046028	JF943095	JF955204
Eudicotyledons	Orobanchaceae	*Pedicularis*	*Pedicularis lecomtei*	P52-1-HW-LIDZ1257B	JF977611	JN046027	JF943094	JF955203
Eudicotyledons	Orobanchaceae	*Pedicularis*	*Pedicularis lecomtei*	P52-2-HW-HW10197	JF977610	JN046026	JF943093	JF955202
Eudicotyledons	Orobanchaceae	*Pedicularis*	*Pedicularis latituba*	P51-3-HW-HW10268	JF977608	JN046024	JF943091	JF955200
Eudicotyledons	Orobanchaceae	*Pedicularis*	*Pedicularis latituba*	P51-HW-LIDZ1208	JF977607	JN046023	JF943090	JF955199
Eudicotyledons	Orobanchaceae	*Pedicularis*	*Pedicularis latituba*	P51-2-HW-HW10252	JF977609	JN046025	JF943092	JF955201
Eudicotyledons	Orobanchaceae	*Pedicularis*	*Pedicularis lachnoglossa*	P50-HW-LIDZ1094	JF977602	JN046018	JF943085	JF955194
Eudicotyledons	Orobanchaceae	*Pedicularis*	*Pedicularis lachnoglossa*	P50-2-HW-HW10051	JF977606	JN046022	JF943089	JF955198
Eudicotyledons	Orobanchaceae	*Pedicularis*	*Pedicularis lachnoglossa*	P50-4-HW-HW10206	JF977604	JN046020	JF943087	JF955196
Eudicotyledons	Orobanchaceae	*Pedicularis*	*Pedicularis lachnoglossa*	P50-5-HW-HW10228	JF977603	JN046019	JF943086	JF955195

Eudicotyledons	Orobanchaceae	*Pedicularis*	*Pedicularis lachnoglossa*	P50-3-HW-HW10140	JF977605	JN046021	JF943088	JF955197
Eudicotyledons	Orobanchaceae	*Pedicularis*	*Pedicularis labordei*	P49-1-HW-LIDZ1302	JF977601	JN046017	JF943084	JF955193
Eudicotyledons	Orobanchaceae	*Pedicularis*	*Pedicularis labordei*	P49-5-HW-LIDZ1335	JF977597	JN046013	JF943080	JF955189
Eudicotyledons	Orobanchaceae	*Pedicularis*	*Pedicularis labordei*	P49-3-HW-05121	JF977599	JN046015	JF943082	JF955191
Eudicotyledons	Orobanchaceae	*Pedicularis*	*Pedicularis labordei*	P49-4-HW-Yang003	JF977598	JN046014	JF943081	JF955190
Eudicotyledons	Orobanchaceae	*Pedicularis*	*Pedicularis labordei*	P49-2-HW-05120	JF977600	JN046016	JF943083	JF955192
Eudicotyledons	Orobanchaceae	*Pedicularis*	*Pedicularis kansuensis*	P47-5-HW-08880	JF977593	JN046009	JF943076	JF955186
Eudicotyledons	Orobanchaceae	*Pedicularis*	*Pedicularis kansuensis*	P47-3-HW-LIDZ1090	JF977595	JN046011	JF943078	JF955188
Eudicotyledons	Orobanchaceae	*Pedicularis*	*Pedicularis kansuensis*	P47-4-HW-LIDZ1207	JF977594	JN046010	JF943077	JF955187
Eudicotyledons	Orobanchaceae	*Pedicularis*	*Pedicularis integrifolia*	P45-2-HW-HW10151	JF977591	JN046007	JF943074	JF955184
Eudicotyledons	Orobanchaceae	*Pedicularis*	*Pedicularis integrifolia*	P45-4-HW-HW10230	JF977589	JN046005	JF943072	JF955182
Eudicotyledons	Orobanchaceae	*Pedicularis*	*Pedicularis integrifolia*	P45-3-HW-LIDZ1159	JF977590	JN046006	JF943073	JF955183
Eudicotyledons	Orobanchaceae	*Pedicularis*	*Pedicularis integrifolia*	P45-HW-08920	JF977588	JN046004	JF943071	JF955181
Eudicotyledons	Orobanchaceae	*Pedicularis*	*Pedicularis ingens*	P173-3-HW-HW10271	JF977586	JN046002	JF943069	JF955179
Eudicotyledons	Orobanchaceae	*Pedicularis*	*Pedicularis ingens*	P173-2-HW-HW10264	JF977587	JN046003	JF943070	JF955180
Eudicotyledons	Orobanchaceae	*Pedicularis*	*Pedicularis gyrorhyncha*	P43-3-HW-HW10161	JF977584	JN046000	JF943067	JF955177
Eudicotyledons	Orobanchaceae	*Pedicularis*	*Pedicularis gyrorhyncha*	P43-4-HW-HW10212	JF977583	JN045999	JF943066	JF955176
Eudicotyledons	Orobanchaceae	*Pedicularis*	*Pedicularis gyrorhyncha*	P43-1-HW-LIDZ1248	JF977585	JN046001	JF943068	JF955178
Eudicotyledons	Orobanchaceae	*Pedicularis*	*Pedicularis gruina*	P41-3-HW-LIDZ1058	JF977580	JN045996	JF943063	JF955173
Eudicotyledons	Orobanchaceae	*Pedicularis*	*Pedicularis gruina*	P41-6-HW-HW10358	JF977577	JN045993	JF943060	JF955170
Eudicotyledons	Orobanchaceae	*Pedicularis*	*Pedicularis gruina*	P41-1-HW-08720	JF977582	JN045998	JF943065	JF955175
Eudicotyledons	Orobanchaceae	*Pedicularis*	*Pedicularis gruina*	P41-2-HW-LIDZ1598	JF977581	JN045997	JF943064	JF955174
Eudicotyledons	Orobanchaceae	*Pedicularis*	*Pedicularis gruina*	P41-5-HW-LIDZ1054	JF977578	JN045994	JF943061	JF955171
Eudicotyledons	Orobanchaceae	*Pedicularis*	*Pedicularis gruina*	P41-4-HW-J199	JF977579	JN045995	JF943062	JF955172
Eudicotyledons	Orobanchaceae	*Pedicularis*	*Pedicularis gracilis*	P39-3-HW-08973	JF977573	JN045989	JF943056	JF955166
Eudicotyledons	Orobanchaceae	*Pedicularis*	*Pedicularis gracilis*	P39-5-HW-LIDZ1304	JF977572	JN045988	JF943055	JF955165
Eudicotyledons	Orobanchaceae	*Pedicularis*	*Pedicularis gracilis*	P39-2-HW-081128	JF977574	JN045990	JF943057	JF955167
Eudicotyledons	Orobanchaceae	*Pedicularis*	*Pedicularis gracilis*	P39-1-HW-J171	JF977575	JN045991	JF943058	JF955168
Eudicotyledons	Orobanchaceae	*Pedicularis*	*Pedicularis glabrescens*	P38-HW-LIDZ1272	JF977568	JN045984	JF943051	JF955161
Eudicotyledons	Orobanchaceae	*Pedicularis*	*Pedicularis glabrescens*	P38-5-HW-HW10098	JF977569	JN045985	JF943052	JF955162
Eudicotyledons	Orobanchaceae	*Pedicularis*	*Pedicularis glabrescens*	P38-2-HW-HW10211	JF977571	JN045987	JF943054	JF955164
Eudicotyledons	Orobanchaceae	*Pedicularis*	*Pedicularis glabrescens*	P38-4-HW-HW10162	JF977570	JN045986	JF943053	JF955163
Eudicotyledons	Orobanchaceae	*Pedicularis*	*Pedicularis floribunda*	P37-2-HW-LIDZ1187	JF977567	JN045983	JF943050	JF955160
Eudicotyledons	Orobanchaceae	*Pedicularis*	*Pedicularis floribunda*	P37-HW-LIDZ1190	JF977566	JN045982	JF943049	JF955159
Eudicotyledons	Orobanchaceae	*Pedicularis*	*Pedicularis fengii*	P158-2-HW-Yu606	JF977564	JN045980	JF943047	JF955157
Eudicotyledons	Orobanchaceae	*Pedicularis*	*Pedicularis fengii*	P158-1-HW-HW10153	JF977565	JN045981	JF943048	JF955158
Eudicotyledons	Orobanchaceae	*Pedicularis*	*Pedicularis fengii*	P158-3-HW-HW10369	JF977563	JN045979	JF943046	JF955156
Eudicotyledons	Orobanchaceae	*Pedicularis*	*Pedicularis elwesii*	P33-2-HW-LIDZ1259	JF977561	JN045977	JF943044	JF955154
Eudicotyledons	Orobanchaceae	*Pedicularis*	*Pedicularis elwesii*	P33-3-HW-HW10204	JF977560	JN045976	JF943043	JF955153
Eudicotyledons	Orobanchaceae	*Pedicularis*	*Pedicularis elwesii*	P33-4-HW-081419	JF977559	JN045975	JF943042	JF955152

Eudicotyledons	Orobanchaceae	*Pedicularis*	*Pedicularis elwesii*	P33-1-HW-LL07019	JF977562	JN045978	JF943045	JF955155
Eudicotyledons	Orobanchaceae	*Pedicularis*	*Pedicularis dunniana*	P31-HW-LIDZ1585	JF977556	JN045972	JF943039	JF955149
Eudicotyledons	Orobanchaceae	*Pedicularis*	*Pedicularis dunniana*	P31-3-HW-HW10341	JF977557	JN045973	JF943040	JF955150
Eudicotyledons	Orobanchaceae	*Pedicularis*	*Pedicularis dunniana*	P31-2-HW-HW10138	JF977558	JN045974	JF943041	JF955151
Eudicotyledons	Orobanchaceae	*Pedicularis*	*Pedicularis dolichocymba*	P30-HW-08827	JF977554	JN045970	JF943037	JF955147
Eudicotyledons	Orobanchaceae	*Pedicularis*	*Pedicularis dolichocymba*	P30-2-HW-HW10129	JF977555	JN045971	JF943038	JF955148
Eudicotyledons	Orobanchaceae	*Pedicularis*	*Pedicularis dissectifolia*	P106-2-HW-HW10102	JF977553	JN045969	JF943036	JF955146
Eudicotyledons	Orobanchaceae	*Pedicularis*	*Pedicularis dissectifolia*	P106-HW-HP9544	JF977552	JN045968	JF943035	JF955145
Eudicotyledons	Orobanchaceae	*Pedicularis*	*Pedicularis dichotoma*	P28-3-HW-LIDZ1125	JF977550	JN045966	JF943033	JF955143
Eudicotyledons	Orobanchaceae	*Pedicularis*	*Pedicularis dichotoma*	P28-HW-08811	JF977548	JN045964	JF943031	JF955141
Eudicotyledons	Orobanchaceae	*Pedicularis*	*Pedicularis dichotoma*	P28-4-HW-LIDZ1278	JF977549	JN045965	JF943032	JF955142
Eudicotyledons	Orobanchaceae	*Pedicularis*	*Pedicularis dichotoma*	P28-2-HW-081584	JF977551	JN045967	JF943034	JF955144
Eudicotyledons	Orobanchaceae	*Pedicularis*	*Pedicularis densispica*	P27-1-HW-08722	JF977547	JN045963	JF943030	JF955140
Eudicotyledons	Orobanchaceae	*Pedicularis*	*Pedicularis densispica*	P27-4-HW-03062	JF977545	JN045961	JF943028	JF955138
Eudicotyledons	Orobanchaceae	*Pedicularis*	*Pedicularis densispica*	P27-3-HW-03061	JF977546	JN045962	JF943029	JF955139
Eudicotyledons	Orobanchaceae	*Pedicularis*	*Pedicularis densispica*	P27-7-HW-HW10210	JF977543	JN045959	JF943026	JF955136
Eudicotyledons	Orobanchaceae	*Pedicularis*	*Pedicularis densispica*	P27-6-HW-HW10231	JF977544	JN045960	JF943027	JF955137
Eudicotyledons	Orobanchaceae	*Pedicularis*	*Pedicularis deltoidea*	P26-2-HW-HW10007	JF977542	JN045958	JF943025	JF955135
Eudicotyledons	Orobanchaceae	*Pedicularis*	*Pedicularis deltoidea*	P26-HW-LIDZ1309	JF977541	JN045957	JF943024	JF955134
Eudicotyledons	Orobanchaceae	*Pedicularis*	*Pedicularis decorissima*	P25-2-HW-LIDZ1162	JF977540	JN045956	JF943023	JF955133
Eudicotyledons	Orobanchaceae	*Pedicularis*	*Pedicularis decorissima*	P25-HW-YWB203	JF977539	JN045955	JF943022	JF955132
Eudicotyledons	Orobanchaceae	*Pedicularis*	*Pedicularis decora*	P32-HW-LIDZ1171A	JF977537	JN045953	JF943020	JF955130
Eudicotyledons	Orobanchaceae	*Pedicularis*	*Pedicularis decora*	P32-1-HW-HW10242	JF977538	JN045954	JF943021	JF955131
Eudicotyledons	Orobanchaceae	*Pedicularis*	*Pedicularis debilis*	P180-1-HW-HW10174	JF977536	JN045952	JF943019	JF955129
Eudicotyledons	Orobanchaceae	*Pedicularis*	*Pedicularis debilis*	P180-2-HW-HW10106	JF977535	JN045951	JF943018	JF955128
Eudicotyledons	Orobanchaceae	*Pedicularis*	*Pedicularis debilis*	P180-3-HW-HW10131	JF977534	JN045950	JF943017	JF955127
Eudicotyledons	Orobanchaceae	*Pedicularis*	*Pedicularis davidii*	P23-3-HW-YWB206	JF977532	JN045948	JF943015	JF955125
Eudicotyledons	Orobanchaceae	*Pedicularis*	*Pedicularis davidii*	P23-2-HW-LIDZ1059	JF977533	JN045949	JF943016	JF955126
Eudicotyledons	Orobanchaceae	*Pedicularis*	*Pedicularis davidii*	P23-HW-J238	JF977531	JN045947	JF943014	JF955124
Eudicotyledons	Orobanchaceae	*Pedicularis*	*Pedicularis cymbalaria*	P22-3-HW-LIDZ1209	JF977528	JN045944	JF943011	JF955121
Eudicotyledons	Orobanchaceae	*Pedicularis*	*Pedicularis cymbalaria*	P22-2-HW-HP9804	JF977529	JN045945	JF943012	JF955122
Eudicotyledons	Orobanchaceae	*Pedicularis*	*Pedicularis cymbalaria*	P22-1-HW-HP9536	JF977530	JN045946	JF943013	JF955123
Eudicotyledons	Orobanchaceae	*Pedicularis*	*Pedicularis cymbalaria*	P22-5-HW-HW10116	JF977527	JN045943	JF943010	JF955120
Eudicotyledons	Orobanchaceae	*Pedicularis*	*Pedicularis cyathophylloides*	P20-1-HW-YWB132	JF977526	JN045942	JF943009	JF955119
Eudicotyledons	Orobanchaceae	*Pedicularis*	*Pedicularis cyathophylloides*	P20-4-HW-36271	JF977524	JN045940	JF943007	JF955117
Eudicotyledons	Orobanchaceae	*Pedicularis*	*Pedicularis cyathophylloides*	P20-6-HW-41954	JF977522	JN045938	JF943005	JF955115

Eudicotyledons	Orobanchaceae	*Pedicularis*	*Pedicularis cyathophylloides*	P20-2-HW-LIDZ1194	JF977525	JN045941	JF943008	JF955118
Eudicotyledons	Orobanchaceae	*Pedicularis*	*Pedicularis cyathophylloides*	P20-5-HW-41478	JF977523	JN045939	JF943006	JF955116
Eudicotyledons	Orobanchaceae	*Pedicularis*	*Pedicularis cyathophylla*	P19-6-HW-HW10215	JF977517	JN045933	JF943000	JF955110
Eudicotyledons	Orobanchaceae	*Pedicularis*	*Pedicularis cyathophylla*	P19-1-HW-LIDZ1180	JF977521	JN045937	JF943004	JF955114
Eudicotyledons	Orobanchaceae	*Pedicularis*	*Pedicularis cyathophylla*	P19-5-HW-LIDZ1268	JF977518	JN045934	JF943001	JF955111
Eudicotyledons	Orobanchaceae	*Pedicularis*	*Pedicularis cyathophylla*	P19-3-HW-LIDZ1198	JF977520	JN045936	JF943003	JF955113
Eudicotyledons	Orobanchaceae	*Pedicularis*	*Pedicularis cyathophylla*	P19-4-HW-LIDZ1204	JF977519	JN045935	JF943002	JF955112
Eudicotyledons	Orobanchaceae	*Pedicularis*	*Pedicularis crenata*	P16-4-HW-LIDZ1026	JF977513	JN045929	JF942996	JF955106
Eudicotyledons	Orobanchaceae	*Pedicularis*	*Pedicularis crenata*	P16-2-HW-LIDZ1020	JF977515	JN045931	JF942998	JF955108
Eudicotyledons	Orobanchaceae	*Pedicularis*	*Pedicularis crenata*	P16-3-HW-HW10182	JF977514	JN045930	JF942997	JF955107
Eudicotyledons	Orobanchaceae	*Pedicularis*	*Pedicularis crenata*	P16-1-HW-LIDZ1244	JF977516	JN045932	JF942999	JF955109
Eudicotyledons	Orobanchaceae	*Pedicularis*	*Pedicularis cranolopha*	P15-2-HW-LIDZ1221	JF977511	JN045927	JF942994	JF955104
Eudicotyledons	Orobanchaceae	*Pedicularis*	*Pedicularis cranolopha*	P15-3-HW-LIDZ1232	JF977510	JN045926	JF942993	JF955103
Eudicotyledons	Orobanchaceae	*Pedicularis*	*Pedicularis cranolopha*	P15-5-HW-LIDZ1197	JF977508	JN045924	JF942991	JF955101
Eudicotyledons	Orobanchaceae	*Pedicularis*	*Pedicularis cranolopha*	P15-7-HW-HW10300	JF977506	JN045922	JF942989	JF955099
Eudicotyledons	Orobanchaceae	*Pedicularis*	*Pedicularis cranolopha*	P15-4-HW-LIDZ1082	JF977509	JN045925	JF942992	JF955102
Eudicotyledons	Orobanchaceae	*Pedicularis*	*Pedicularis cranolopha*	P15-6-HW-HW10227	JF977507	JN045923	JF942990	JF955100
Eudicotyledons	Orobanchaceae	*Pedicularis*	*Pedicularis cranolopha*	P15-1-HW-LIDZ1220	JF977512	JN045928	JF942995	JF955105
Eudicotyledons	Orobanchaceae	*Pedicularis*	*Pedicularis confertiflora*	P14-2-HW-LIDZ1524	JF977504	JN045920	JF942987	JF955097
Eudicotyledons	Orobanchaceae	*Pedicularis*	*Pedicularis confertiflora*	P14-5-HW-08845	JF977501	JN045917	JF942984	JF955094
Eudicotyledons	Orobanchaceae	*Pedicularis*	*Pedicularis confertiflora*	P14-1-HW-LIDZ1310	JF977505	JN045921	JF942988	JF955098
Eudicotyledons	Orobanchaceae	*Pedicularis*	*Pedicularis confertiflora*	P14-4-HW-03056	JF977502	JN045918	JF942985	JF955095
Eudicotyledons	Orobanchaceae	*Pedicularis*	*Pedicularis confertiflora*	P14-3-HW-LIDZ1528	JF977503	JN045919	JF942986	JF955096
Eudicotyledons	Orobanchaceae	*Pedicularis*	*Pedicularis comptoniifolia*	P13-2-HW-HW10180	JF977500	JN045916	JF942983	JF955093
Eudicotyledons	Orobanchaceae	*Pedicularis*	*Pedicularis comptoniifolia*	P13-4-HW-LIDZ0996	JF977499	JN045915	JF942982	JF955092
Eudicotyledons	Orobanchaceae	*Pedicularis*	*Pedicularis clarkei*	P12-2-HW-081390B	JF977496	JN045913	JF942979	JF955090
Eudicotyledons	Orobanchaceae	*Pedicularis*	*Pedicularis clarkei*	P12-1-HW-081390A	JF977497	JN045914	JF942980	JF955091
Eudicotyledons	Orobanchaceae	*Pedicularis*	*Pedicularis cinerascens*	P184-1-HW-HW10311A	JF977495	JN045912	JF942978	JF955089
Eudicotyledons	Orobanchaceae	*Pedicularis*	*Pedicularis cinerascens*	P184-2-HW-HW10311B	JF977494	JN045911	JF942977	JF955088
Eudicotyledons	Orobanchaceae	*Pedicularis*	*Pedicularis cephalantha*	P10-3-HW-LIDZ1036	JF977491	JN045909	JF942974	JF955085
Eudicotyledons	Orobanchaceae	*Pedicularis*	*Pedicularis cephalantha*	P10-7-HW-HW10093	JF977487	JN045905	JF942970	JF955081
Eudicotyledons	Orobanchaceae	*Pedicularis*	*Pedicularis cephalantha*	P10-6-HW-HW10013	JF977488	JN045906	JF942971	JF955082
Eudicotyledons	Orobanchaceae	*Pedicularis*	*Pedicularis cephalantha*	P10-2-HW-LIDZ1013	JF977492	JN045910	JF942975	JF955086
Eudicotyledons	Orobanchaceae	*Pedicularis*	*Pedicularis cephalantha*	P10-5-HW-YWB500	JF977489	JN045907	JF942972	JF955083
Eudicotyledons	Orobanchaceae	*Pedicularis*	*Pedicularis cephalantha*	P10-4-HW-J208	JF977490	JN045908	JF942973	JF955084
Eudicotyledons	Orobanchaceae	*Pedicularis*	*Pedicularis brevilabris*	P9-HW-LIDZ1172	JF977483	JN045901	JF942966	JF955077
Eudicotyledons	Orobanchaceae	*Pedicularis*	*Pedicularis brevilabris*	P9-4-HW-HW10325	JF977484	JN045902	JF942967	JF955078
Eudicotyledons	Orobanchaceae	*Pedicularis*	*Pedicularis brevilabris*	P9-2-HW-HW10250	JF977486	JN045904	JF942969	JF955080

Eudicotyledons	Orobanchaceae	*Pedicularis*	*Pedicularis brevilabris*	P9-3-HW-HW10304	JF977485	JN045903	JF942968	JF955079
Eudicotyledons	Orobanchaceae	*Pedicularis*	*Pedicularis batangensis*	P8-3-HW-LIDZ1095	JF977480	JN045898	JF942963	JF955074
Eudicotyledons	Orobanchaceae	*Pedicularis*	*Pedicularis batangensis*	P8-1-HW-LIDZ1067	JF977482	JN045900	JF942965	JF955076
Eudicotyledons	Orobanchaceae	*Pedicularis*	*Pedicularis batangensis*	P8-4-HW-LIDZ1188	JF977479	JN045897	JF942962	JF955073
Eudicotyledons	Orobanchaceae	*Pedicularis*	*Pedicularis batangensis*	P8-2-HW-LIDZ1124	JF977481	JN045899	JF942964	JF955075
Eudicotyledons	Orobanchaceae	*Pedicularis*	*Pedicularis axillaris*	P7-2-HW-HW10005	JF977477	JN045895	JF942960	JF955071
Eudicotyledons	Orobanchaceae	*Pedicularis*	*Pedicularis axillaris*	P7-4-HW-HW10207	JF977475	JN045893	JF942958	JF955069
Eudicotyledons	Orobanchaceae	*Pedicularis*	*Pedicularis axillaris*	P7-1-HW-LIDZ1290	JF977478	JN045896	JF942961	JF955072
Eudicotyledons	Orobanchaceae	*Pedicularis*	*Pedicularis axillaris*	P7-3-HW-HW10097	JF977476	JN045894	JF942959	JF955070
Eudicotyledons	Orobanchaceae	*Pedicularis*	*Pedicularis anas*	P4-3-HW-HW10255	JF977472	JN045890	JF942955	JF955066
Eudicotyledons	Orobanchaceae	*Pedicularis*	*Pedicularis anas*	P4-2-HW-LIDZ1214	JF977473	JN045891	JF942956	JF955067
Eudicotyledons	Orobanchaceae	*Pedicularis*	*Pedicularis anas*	P4-4-HW-HW10274	JF977471	JN045889	JF942954	JF955065
Eudicotyledons	Orobanchaceae	*Pedicularis*	*Pedicularis anas*	P4-1-HW-LIDZ1136	JF977474	JN045892	JF942957	JF955068
Eudicotyledons	Orobanchaceae	*Pedicularis*	*Pedicularis anas*	P4-5-HW-HW10293	JF977470	JN045888	JF942953	JF955064
Eudicotyledons	Orobanchaceae	*Pedicularis*	*Pedicularis amplituba*	P66-2-HW-LIDZ1519B	JF977468	JN045886	JF942951	JF955062
Eudicotyledons	Orobanchaceae	*Pedicularis*	*Pedicularis amplituba*	P66-1-HW-LIDZ1519A	JF977469	JN045887	JF942952	JF955063
Eudicotyledons	Orobanchaceae	*Pedicularis*	*Pedicularis alopecuros*	P3-5-HW-LIDZ1334	JF977463	JN045881	JF942946	JF955057
Eudicotyledons	Orobanchaceae	*Pedicularis*	*Pedicularis alopecuros*	P3-6-HW-HP9687	JF977462	JN045880	JF942945	JF955056
Eudicotyledons	Orobanchaceae	*Pedicularis*	*Pedicularis alopecuros*	P3-2-HW-LIDZ1055	JF977466	JN045884	JF942949	JF955060
Eudicotyledons	Orobanchaceae	*Pedicularis*	*Pedicularis alopecuros*	P3-1-HW-LIDZ0994	JF977467	JN045885	JF942950	JF955061
Eudicotyledons	Orobanchaceae	*Pedicularis*	*Pedicularis alopecuros*	P3-3-HW-Yang004	JF977465	JN045883	JF942948	JF955059
Eudicotyledons	Orobanchaceae	*Pedicularis*	*Pedicularis alopecuros*	P3-4-HW-LIDZ1502	JF977464	JN045882	JF942947	JF955058
Eudicotyledons	Orobanchaceae	*Pedicularis*	*Pedicularis alaschanica*	P1-2-HW-08913	JF977460	JN045878	JF942943	JF955054
Eudicotyledons	Orobanchaceae	*Pedicularis*	*Pedicularis alaschanica*	P1-1-HW-YWB201	JF977461	JN045879	JF942944	JF955055
Eudicotyledons	Orobanchaceae	*Pedicularis*	*Pedicularis alaschanica*	P1-3-HW-08929	JF977459	JN045877	JF942942	JF955053
Eudicotyledons	Celastraceae	*Parnassia*	*Parnassia yunnanensis*	wud05053	JF977455	JN045872	JF942936	JF955048
Eudicotyledons	Celastraceae	*Parnassia*	*Parnassia yunnanensis*	wud032-1	JF977457	JN045875	JF942939	JF955050
Eudicotyledons	Celastraceae	*Parnassia*	*Parnassia yunnanensis*	wud032-2	JF977456	JN045874	JF942938	JF955049
Eudicotyledons	Celastraceae	*Parnassia*	*Parnassia yunnanensis*	Wud03066	JF811127	JF802397	JF802302	JF802492
Eudicotyledons	Celastraceae	*Parnassia*	*Parnassia yunnanensis*	wud03066	JF811127	JF802397	JF802302	JF802492
Eudicotyledons	Celastraceae	*Parnassia*	*Parnassia yunnanensis*	Wud032-1	JF977457	JN045875	JF942939	JF955050
Eudicotyledons	Celastraceae	*Parnassia*	*Parnassia yunnanensis*	Wud032-2	JF977456	JN045874	JF942938	JF955049
Eudicotyledons	Celastraceae	*Parnassia*	*Parnassia yunnanensis*	Wud04209	JF811130	JF802400	JF802305	JF802495
Eudicotyledons	Celastraceae	*Parnassia*	*Parnassia wightiana*	wud019	JF977453	JN045870	JF942934	JF955046
Eudicotyledons	Celastraceae	*Parnassia*	*Parnassia wightiana*	Wud124	JF811123	JF802393	JF802298	JF802488
Eudicotyledons	Celastraceae	*Parnassia*	*Parnassia wightiana*	Wud1388	JF811125	JF802395	JF802300	JF802490
Eudicotyledons	Celastraceae	*Parnassia*	*Parnassia wightiana*	Wud019	JF977453	JN045870	JF942934	JF955046
Eudicotyledons	Celastraceae	*Parnassia*	*Parnassia wightiana*	wud1388	JF811125	JF802395	JF802300	JF802490
Eudicotyledons	Celastraceae	*Parnassia*	*Parnassia wightiana*	Wud090571	JF977421	JN045836	JF942892	JF955009
Eudicotyledons	Celastraceae	*Parnassia*	*Parnassia wightiana*	Wud246	JF811126	JF802396	JF802301	JF802491

Eudicotyledons	Celastraceae	*Parnassia*	*Parnassia wightiana*	Wud024	JF977452	JN045869	JF942933	JF955045
Eudicotyledons	Celastraceae	*Parnassia*	*Parnassia wightiana*	wud09483	JF811122	JF802392	JF802297	JF802487
Eudicotyledons	Celastraceae	*Parnassia*	*Parnassia wightiana*	wud007	JF977454	JN045871	JF942935	JF955047
Eudicotyledons	Celastraceae	*Parnassia*	*Parnassia wightiana*	Wud09483	JF811122	JF802392	JF802297	JF802487
Eudicotyledons	Celastraceae	*Parnassia*	*Parnassia wightiana*	Wud0904	JF811120	JF802390	JF802295	JF802485
Eudicotyledons	Celastraceae	*Parnassia*	*Parnassia wightiana*	wud024	JF977452	JN045869	JF942933	JF955045
Eudicotyledons	Celastraceae	*Parnassia*	*Parnassia wightiana*	Wud128	JF811124	JF802394	JF802299	JF802489
Eudicotyledons	Celastraceae	*Parnassia*	*Parnassia wightiana*	wud0904	JF811120	JF802390	JF802295	JF802485
Eudicotyledons	Celastraceae	*Parnassia*	*Parnassia wightiana*	Wud02012	JF811118	JF802388	JF802293	JF802483
Eudicotyledons	Celastraceae	*Parnassia*	*Parnassia wightiana*	Wud007	JF977454	JN045871	JF942935	JF955047
Eudicotyledons	Celastraceae	*Parnassia*	*Parnassia viridiflora*	wud090664	JF977446	JN045864	JF942927	JF955040
Eudicotyledons	Celastraceae	*Parnassia*	*Parnassia viridiflora*	Wud090664	JF977446	JN045864	JF942927	JF955040
Eudicotyledons	Celastraceae	*Parnassia*	*Parnassia viridiflora*	Wud03065	JF811114	JF802384	JF802289	JF802479
Eudicotyledons	Celastraceae	*Parnassia*	*Parnassia venusta*	wud129	JF977445	JN045863	JF942926	JF955039
Eudicotyledons	Celastraceae	*Parnassia*	*Parnassia venusta*	Wud2322	JF811113	JF802383	JF802288	JF802478
Eudicotyledons	Celastraceae	*Parnassia*	*Parnassia venusta*	wud2322	JF811113	JF802383	JF802288	JF802478
Eudicotyledons	Celastraceae	*Parnassia*	*Parnassia venusta*	Wud129_3	JF811112	JF802382	JF802287	JF802477
Eudicotyledons	Celastraceae	*Parnassia*	*Parnassia venusta*	Wud129	JF977445	JN045863	JF942926	JF955039
Eudicotyledons	Celastraceae	*Parnassia*	*Parnassia trinervis*	wud091569	JF977440	JN045858	JF942921	JF955034
Eudicotyledons	Celastraceae	*Parnassia*	*Parnassia trinervis*	Wud03124	JF811108	JF802378	JF802283	JF802473
Eudicotyledons	Celastraceae	*Parnassia*	*Parnassia trinervis*	Wud05130	JF811109	JF802379	JF802284	JF802474
Eudicotyledons	Celastraceae	*Parnassia*	*Parnassia trinervis*	wud03124	JF811108	JF802378	JF802283	JF802473
Eudicotyledons	Celastraceae	*Parnassia*	*Parnassia trinervis*	Wud03081	JF811107	JF802377	JF802282	JF802472
Eudicotyledons	Celastraceae	*Parnassia*	*Parnassia trinervis*	Wud2100	JF811110	JF802380	JF802285	JF802475
Eudicotyledons	Celastraceae	*Parnassia*	*Parnassia trinervis*	wud0193	JF977443	JN045861	JF942924	JF955037
Eudicotyledons	Celastraceae	*Parnassia*	*Parnassia trinervis*	WudQ0193	JF811106	JF802376	JF802281	JF802471
Eudicotyledons	Celastraceae	*Parnassia*	*Parnassia trinervis*	wud03081	JF811107	JF802377	JF802282	JF802472
Eudicotyledons	Celastraceae	*Parnassia*	*Parnassia tenella*	wud120	JF977437	JN045853	JF942915	JF955029
Eudicotyledons	Celastraceae	*Parnassia*	*Parnassia tenella*	wud125-1	JF977436	JN045852	JF942914	JF955028
Eudicotyledons	Celastraceae	*Parnassia*	*Parnassia tenella*	Wud120	JF977437	JN045853	JF942915	JF955029
Eudicotyledons	Celastraceae	*Parnassia*	*Parnassia tenella*	Wud103062	JF811102	JF802372	JF802277	JF802467
Eudicotyledons	Celastraceae	*Parnassia*	*Parnassia tenella*	Wud02002	JF811101	JF802371	JF802276	JF802466
Eudicotyledons	Celastraceae	*Parnassia*	*Parnassia tenella*	Wud125-11	JF811105	JF802375	JF802280	JF802470
Eudicotyledons	Celastraceae	*Parnassia*	*Parnassia tenella*	Wud125-1	JF977436	JN045852	JF942914	JF955028
Eudicotyledons	Celastraceae	*Parnassia*	*Parnassia tenella*	wud103062	JF811102	JF802372	JF802277	JF802467
Eudicotyledons	Celastraceae	*Parnassia*	*Parnassia submysorensis*	Wud03002	JF811099	JF802369	JF802274	JF802464
Eudicotyledons	Celastraceae	*Parnassia*	*Parnassia submysorensis*	Wud05198	JF811100	JF802370	JF802275	JF802465
Eudicotyledons	Celastraceae	*Parnassia*	*Parnassia perciliata*	Wud026-10	JF811097	JF802367	JF802272	JF802462
Eudicotyledons	Celastraceae	*Parnassia*	*Parnassia perciliata*	Wud026-1	JF977431	JN045851	JF942908	JF955023
Eudicotyledons	Celastraceae	*Parnassia*	*Parnassia perciliata*	Wud281	JF977429	JN045847	JF942904	JF955019

Eudicotyledons	Celastraceae	*Parnassia*	*Parnassia perciliata*	wud026-1	JF977431	JN045851	JF942908	JF955023
Eudicotyledons	Celastraceae	*Parnassia*	*Parnassia perciliata*	wud281	JF977429	JN045847	JF942904	JF955019
Eudicotyledons	Celastraceae	*Parnassia*	*Parnassia perciliata*	wud029-1	JF977430	JN045849	JF942906	JF955021
Eudicotyledons	Celastraceae	*Parnassia*	*Parnassia palustris*	Duncan_&_Tait_138	JF811094	JF802364	JF802270	JF802459
Eudicotyledons	Celastraceae	*Parnassia*	*Parnassia palustris*	wud020672	JF977426	JN045842	JF942900	JF955014
Eudicotyledons	Celastraceae	*Parnassia*	*Parnassia palustris*	Wud1001_3	JF811093	JF802363	JF802269	JF802458
Eudicotyledons	Celastraceae	*Parnassia*	*Parnassia palustris*	wud249	JF977427	JN045844	JF942901	JF955016
Eudicotyledons	Celastraceae	*Parnassia*	*Parnassia palustris*	N.A._Brjummitt_&_Bridger_M.A._249	JF811095	JF802365	JF795347	JF802460
Eudicotyledons	Celastraceae	*Parnassia*	*Parnassia palustris*	Wud06050	JF811092	JF802362	JF802268	JF802457
Eudicotyledons	Celastraceae	*Parnassia*	*Parnassia palustris*	wud138	JF977428	JN045845	JF942902	JF955017
Eudicotyledons	Celastraceae	*Parnassia*	*Parnassia nubicola*	Wud1496	JF811089	JF802359	JF795345	JF802454
Eudicotyledons	Celastraceae	*Parnassia*	*Parnassia nubicola*	M.F._Watson_28	JF811090	JF802360	JF795344	JF802455
Eudicotyledons	Celastraceae	*Parnassia*	*Parnassia noemiae*	Peter_Warten_020672	JF811091	JF802361	JF802267	JF802456
Eudicotyledons	Celastraceae	*Parnassia*	*Parnassia noemiae*	Wud083-2	JF811088	JF802358	JF802266	JF802453
Eudicotyledons	Celastraceae	*Parnassia*	*Parnassia noemiae*	wud02015	JF977425	JN045839	JF942897	JF955013
Eudicotyledons	Celastraceae	*Parnassia*	*Parnassia noemiae*	Wud083_1	JN188291	JN188290	JN188289	JN188288
Eudicotyledons	Celastraceae	*Parnassia*	*Parnassia noemiae*	Wud02015	JF977425	JN045839	JF942897	JF955013
Eudicotyledons	Celastraceae	*Parnassia*	*Parnassia mysorensis*	wud090571	JF977421	JN045836	JF942892	JF955009
Eudicotyledons	Celastraceae	*Parnassia*	*Parnassia mysorensis*	wud02010	JF977422	JN045837	JF942893	JF955010
Eudicotyledons	Celastraceae	*Parnassia*	*Parnassia mysorensis*	Wud02010	JF977422	JN045837	JF942893	JF955010
Eudicotyledons	Celastraceae	*Parnassia*	*Parnassia mysorensis*	Wud090727	JF811083	JF802353	JF802261	JF802448
Eudicotyledons	Celastraceae	*Parnassia*	*Parnassia mysorensis*	wud123	JF977420	JN045835	JF942891	JF955008
Eudicotyledons	Celastraceae	*Parnassia*	*Parnassia mysorensis*	Wud131	JF811086	JF802356	JF802264	JF802451
Eudicotyledons	Celastraceae	*Parnassia*	*Parnassia mysorensis*	Wud123	JF977420	JN045835	JF942891	JF955008
Eudicotyledons	Celastraceae	*Parnassia*	*Parnassia mysorensis*	Wud090835	JF811084	JF802354	JF802262	JF802449
Eudicotyledons	Celastraceae	*Parnassia*	*Parnassia mysorensis*	wud131	JF811086	JF802356	JF802264	JF802451
Eudicotyledons	Celastraceae	*Parnassia*	*Parnassia lutea*	Wud0339	JF811080	JF802350	JF802258	JF802445
Eudicotyledons	Celastraceae	*Parnassia*	*Parnassia lutea*	Guobz9460	JF811081	JF802351	JF802259	JF802446
Eudicotyledons	Celastraceae	*Parnassia*	*Parnassia longipetala*	Wud08029	JF811078	JF802348	JF802256	JF802443
Eudicotyledons	Celastraceae	*Parnassia*	*Parnassia longipetala*	wud09945	JF977415	JN045832	JF942886	JF955002
Eudicotyledons	Celastraceae	*Parnassia*	*Parnassia longipetala*	Wud09945	JF977415	JN045832	JF942886	JF955002
Eudicotyledons	Celastraceae	*Parnassia*	*Parnassia longipetala*	wud08029	JF811078	JF802348	JF802256	JF802443
Eudicotyledons	Celastraceae	*Parnassia*	*Parnassia leptophylla*	wud0902-4	JF977414	JN045831	JF942885	JF955001
Eudicotyledons	Celastraceae	*Parnassia*	*Parnassia leptophylla*	wud0902-8	JF977413	JN045829	JF942884	JF955000
Eudicotyledons	Celastraceae	*Parnassia*	*Parnassia leptophylla*	Wud0902-4	JF977414	JN045831	JF942885	JF955001
Eudicotyledons	Celastraceae	*Parnassia*	*Parnassia leptophylla*	Wud0902-8	JF977413	JN045829	JF942884	JF955000
Eudicotyledons	Celastraceae	*Parnassia*	*Parnassia laxmannii*	wud1696	JF977410	JN045826	JF942881	JF954997
Eudicotyledons	Celastraceae	*Parnassia*	*Parnassia laxmannii*	wud091562	JF977411	JN045827	JF942882	JF954998
Eudicotyledons	Celastraceae	*Parnassia*	*Parnassia kotzebuei*	Halliday_G.A203	JF811074	JF802344	JF802252	JF802439

Eudicotyledons	Celastraceae	*Parnassia*	*Parnassia kotzebuei*	Vornova_T5634	JF811075	JF802345	JF802253	JF802440
Eudicotyledons	Celastraceae	*Parnassia*	*Parnassia kangdingensis*	Wud108-11	JF811072	JF802342	JF802250	JF802437
Eudicotyledons	Celastraceae	*Parnassia*	*Parnassia kangdingensis*	Wud108-2	JF811073	JF802343	JF802251	JF802438
Eudicotyledons	Celastraceae	*Parnassia*	*Parnassia guilinensis*	wud012-1	JF977405	JN045819	JF942873	JF954993
Eudicotyledons	Celastraceae	*Parnassia*	*Parnassia guilinensis*	Wud04042	JF811071	JF802341	JF802249	JF802436
Eudicotyledons	Celastraceae	*Parnassia*	*Parnassia guilinensis*	wud04042	JF811071	JF802341	JF802249	JF802436
Eudicotyledons	Celastraceae	*Parnassia*	*Parnassia guilinensis*	Wud012-2	JF811070	JF802340	JF802248	JF802435
Eudicotyledons	Celastraceae	*Parnassia*	*Parnassia guilinensis*	Wud012-1	JF977405	JN045819	JF942873	JF954993
Eudicotyledons	Celastraceae	*Parnassia*	*Parnassia grandifolia*	John_Taylor23596	JF811068	JF802338	JF802246	JF802433
Eudicotyledons	Celastraceae	*Parnassia*	*Parnassia grandifolia*	Mcpougall_WB1512	JF811067	JF802337	JF802245	JF802432
Eudicotyledons	Celastraceae	*Parnassia*	*Parnassia foliosa*	Wud0905-2	JF811064	JF802334	JF802242	JF802429
Eudicotyledons	Celastraceae	*Parnassia*	*Parnassia foliosa*	Wud0905-3	JF811065	JF802335	JF802243	JF802430
Eudicotyledons	Celastraceae	*Parnassia*	*Parnassia foliosa*	Wud0905-1	JF811063	JF802333	JF802241	JF802428
Eudicotyledons	Celastraceae	*Parnassia*	*Parnassia foliosa*	Wud09130	JF811066	JF802336	JF802244	JF802431
Eudicotyledons	Celastraceae	*Parnassia*	*Parnassia foliosa*	wud0905-2	JF811064	JF802334	JF802242	JF802429
Eudicotyledons	Celastraceae	*Parnassia*	*Parnassia foliosa*	wud0905-1	JF811063	JF802333	JF802241	JF802428
Eudicotyledons	Celastraceae	*Parnassia*	*Parnassia foliosa*	wud09130	JF811066	JF802336	JF802244	JF802431
Eudicotyledons	Celastraceae	*Parnassia*	*Parnassia farreri*	wud126	JF977395	JN045811	JF942862	JF954985
Eudicotyledons	Celastraceae	*Parnassia*	*Parnassia farreri*	wud03051	JF977397	JN045812	JF942864	JF954987
Eudicotyledons	Celastraceae	*Parnassia*	*Parnassia farreri*	Wud2319	JF811062	JF802332	JF802240	JF802427
Eudicotyledons	Celastraceae	*Parnassia*	*Parnassia farreri*	Wud126	JF977395	JN045811	JF942862	JF954985
Eudicotyledons	Celastraceae	*Parnassia*	*Parnassia farreri*	Wud03051	JF977397	JN045812	JF942864	JF954987
Eudicotyledons	Celastraceae	*Parnassia*	*Parnassia farreri*	wud2319	JF811062	JF802332	JF802240	JF802427
Eudicotyledons	Celastraceae	*Parnassia*	*Parnassia faberi*	wud02017	JF977393	JN045809	JF942859	JF954982
Eudicotyledons	Celastraceae	*Parnassia*	*Parnassia faberi*	Wud0901-2	JF811058	JF802328	JF802236	JF802423
Eudicotyledons	Celastraceae	*Parnassia*	*Parnassia faberi*	Wud0901-1	JF811057	JF802327	JF802235	JF802422
Eudicotyledons	Celastraceae	*Parnassia*	*Parnassia faberi*	Wud0907	JF811059	JF802329	JF802237	JF802424
Eudicotyledons	Celastraceae	*Parnassia*	*Parnassia faberi*	wud03013	JF977392	JN045808	JF942858	JF954981
Eudicotyledons	Celastraceae	*Parnassia*	*Parnassia faberi*	Wud02017	JF977393	JN045809	JF942859	JF954982
Eudicotyledons	Celastraceae	*Parnassia*	*Parnassia faberi*	Wud02013	JF811054	JF802324	JF802233	JF802419
Eudicotyledons	Celastraceae	*Parnassia*	*Parnassia faberi*	wud0901-2	JF811058	JF802328	JF802236	JF802423
Eudicotyledons	Celastraceae	*Parnassia*	*Parnassia faberi*	wud0901-1	JF811057	JF802327	JF802235	JF802422
Eudicotyledons	Celastraceae	*Parnassia*	*Parnassia faberi*	Wud03013	JF977392	JN045808	JF942858	JF954981
Eudicotyledons	Celastraceae	*Parnassia*	*Parnassia esquirolii*	Wud900_1	JF811052	JF802322	JF802231	JF802417
Eudicotyledons	Celastraceae	*Parnassia*	*Parnassia esquirolii*	Wud900_3	JF811053	JF802323	JF802232	JF802418
Eudicotyledons	Celastraceae	*Parnassia*	*Parnassia epunctulata*	wud1526	JF977388	JN045804	JF942853	JF954976
Eudicotyledons	Celastraceae	*Parnassia*	*Parnassia epunctulata*	Wud03890	JF811050	JF802320	JF802229	JF802415
Eudicotyledons	Celastraceae	*Parnassia*	*Parnassia epunctulata*	Wud1526	JF977388	JN045804	JF942853	JF954976
Eudicotyledons	Celastraceae	*Parnassia*	*Parnassia epunctulata*	wud03890	JF811050	JF802320	JF802229	JF802415
Eudicotyledons	Celastraceae	*Parnassia*	*Parnassia dilatata*	Wud020-10	JF811047	JF802317	JF802226	JF802412

Eudicotyledons	Celastraceae	*Parnassia*	*Parnassia dilatata*	Wud020-5	JF811049	JF802319	JF802228	JF802414
Eudicotyledons	Celastraceae	*Parnassia*	*Parnassia dilatata*	wud020-20	JF977386	JN045802	JF942851	JF954974
Eudicotyledons	Celastraceae	*Parnassia*	*Parnassia dilatata*	Wud020-20	JF977386	JN045802	JF942851	JF954974
Eudicotyledons	Celastraceae	*Parnassia*	*Parnassia dilatata*	wud020-5	JF811049	JF802319	JF802228	JF802414
Eudicotyledons	Celastraceae	*Parnassia*	*Parnassia dilatata*	wud020-10	JF811047	JF802317	JF802226	JF802412
Eudicotyledons	Celastraceae	*Parnassia*	*Parnassia deqenensis*	Wud05178	JF811046	JF802316	JF802225	JF802411
Eudicotyledons	Celastraceae	*Parnassia*	*Parnassia deqenensis*	Wud03064	JF811045	JF802315	JF802224	JF802410
Eudicotyledons	Celastraceae	*Parnassia*	*Parnassia delavayi*	Wud091522	JF811041	JF802311	JF802220	JF802406
Eudicotyledons	Celastraceae	*Parnassia*	*Parnassia delavayi*	wud127	JF977382	JN045798	JF942845	JF954968
Eudicotyledons	Celastraceae	*Parnassia*	*Parnassia delavayi*	Wud1553	JF811044	JF802314	JF802223	JF802409
Eudicotyledons	Celastraceae	*Parnassia*	*Parnassia delavayi*	wud1413	JF977381	JN045797	JF942844	JF954967
Eudicotyledons	Celastraceae	*Parnassia*	*Parnassia delavayi*	Wud127	JF977382	JN045798	JF942845	JF954968
Eudicotyledons	Celastraceae	*Parnassia*	*Parnassia delavayi*	wud1553	JF811044	JF802314	JF802223	JF802409
Eudicotyledons	Celastraceae	*Parnassia*	*Parnassia delavayi*	Wud02014	JF811040	JF802310	JF802219	JF802405
Eudicotyledons	Celastraceae	*Parnassia*	*Parnassia delavayi*	Wud1413	JF977381	JN045797	JF942844	JF954967
Eudicotyledons	Celastraceae	*Parnassia*	*Parnassia brevistyla*	wud150	JF977371	JN045786	JF942833	JF954958
Eudicotyledons	Celastraceae	*Parnassia*	*Parnassia brevistyla*	wud05051	JF977374	JN045789	JF942836	JF954961
Eudicotyledons	Celastraceae	*Parnassia*	*Parnassia brevistyla*	wud1177	JF977373	JN045788	JF942835	JF954960
Eudicotyledons	Celastraceae	*Parnassia*	*Parnassia brevistyla*	wud0091628	JF977375	JN045790	JF942837	JF954962
Eudicotyledons	Celastraceae	*Parnassia*	*Parnassia brevistyla*	wud1288	JF977372	JN045787	JF942834	JF954959
Eudicotyledons	Celastraceae	*Parnassia*	*Parnassia asarifolia*	wud1027	JF977369	JN045782	JF942831	JF954956
Eudicotyledons	Celastraceae	*Parnassia*	*Parnassia asarifolia*	PROP0816	JF811037	JF802307	JF802206	JF802402
Eudicotyledons	Celastraceae	*Parnassia*	*Parnassia asarifolia*	Mcneilus_VE1027	JF811038	JF802308	JF802207	JF802403
Eudicotyledons	Celastraceae	*Parnassia*	*Parnassia asarifolia*	wud7103	JF977368	JN045781	JF942830	JF954955
Eudicotyledons	Celastraceae	*Parnassia*	*Parnassia asarifolia*	Lena_Arrtz_&_Marlin_PK_71	JF811039	JF802309	JF802208	JF802404
Eudicotyledons	Celastraceae	*Parnassia*	*Parnassia asarifolia*	wud0816	JF977370	JN045783	JF942832	JF954957
Eudicotyledons	Araliaceae	*Panax*	*Panax japonicus*	J1(PE)	HQ112425	HQ112873	HQ11260	HQ11306
Eudicotyledons	Araliaceae	*Panax*	*Panax japonicus*	PS1477MT04	GQ434783	GQ43540	GQ43671	GQ43427
Eudicotyledons	Araliaceae	*Panax*	*Panax ginseng*	PS1467MT01	JF421520	GQ43539	GQ43670	GQ43426
Eudicotyledons	Araliaceae	*Panax*	*Panax ginseng*	Zhou002(PE)	HQ112416	HQ112864	HQ11259	HQ11305
Eudicotyledons	Polygonaceae	*Oxyria*	*Oxyria sinensis*	W26	JF977267	JN045697	JF942758	JF954854
Eudicotyledons	Polygonaceae	*Oxyria*	*Oxyria sinensis*	D189	JF977268	JN045698	JF942759	JF954855
Eudicotyledons	Polygonaceae	*Oxyria*	*Oxyria digyna*	D1229	JF977266	JN045695	JF942756	JF954852
Eudicotyledons	Polygonaceae	*Oxyria*	*Oxyria digyna*	D1354	JF977265	JN045694	JF942755	JF954851
Eudicotyledons	Polygonaceae	*Oxyria*	*Oxyria digyna*	W25	JF977262	JN045690	JF942751	JF954847
Eudicotyledons	Polygonaceae	*Oxyria*	*Oxyria digyna*	D2061	JF977263	JN045692	JF942753	JF954849
Eudicotyledons	Polygonaceae	*Oxyria*	*Oxyria digyna*	D1755	JF977264	JN045693	JF942754	JF954850
Eudicotyledons	Betulaceae	*Ostryopsis*	*Ostryopsis nobilis*	Ostryopsis07	JF977258	JN045686	JF942747	JF954843
Eudicotyledons	Betulaceae	*Ostryopsis*	*Ostryopsis nobilis*	Ostryopsis02	JF977261	JN045689	JF942750	JF954846
Eudicotyledons	Betulaceae	*Ostryopsis*	*Ostryopsis nobilis*	Ostryopsis03	JF977260	JN045688	JF942749	JF954845

Eudicotyledons	Betulaceae	*Ostryopsis*	*Ostryopsis nobilis*	Ostryopsis10	JF977257	JN045685	JF942746	JF954842
Eudicotyledons	Betulaceae	*Ostryopsis*	*Ostryopsis nobilis*	Ostryopsis05	JF977259	JN045687	JF942748	JF954844
Eudicotyledons	Betulaceae	*Ostryopsis*	*Ostryopsis intermedia*	Ostryopsis01	JF977256	JN045684	JF942745	JF954841
Eudicotyledons	Betulaceae	*Ostryopsis*	*Ostryopsis intermedia*	Ostryopsis06	JF977254	JN045682	JF942743	JF954839
Eudicotyledons	Betulaceae	*Ostryopsis*	*Ostryopsis intermedia*	Ostryopsis09	JF977252	JN045680	JF942741	JF954837
Eudicotyledons	Betulaceae	*Ostryopsis*	*Ostryopsis intermedia*	Ostryopsis08	JF977253	JN045681	JF942742	JF954838
Eudicotyledons	Betulaceae	*Ostryopsis*	*Ostryopsis intermedia*	Ostryopsis04	JF977255	JN045683	JF942744	JF954840
Eudicotyledons	Betulaceae	*Ostryopsis*	*Ostryopsis davidiana*	Ostryopsis12	JF977250	JN045678	JF942739	JF954835
Eudicotyledons	Betulaceae	*Ostryopsis*	*Ostryopsis davidiana*	Ostryopsis13	JF977249	JN045677	JF942738	JF954834
Eudicotyledons	Betulaceae	*Ostryopsis*	*Ostryopsis davidiana*	Ostryopsis16	JF977246	JN045674	JF942735	JF954831
Eudicotyledons	Betulaceae	*Ostryopsis*	*Ostryopsis davidiana*	Ostryopsis18	JF977244	JN045672	JF942733	JF954829
Eudicotyledons	Betulaceae	*Ostryopsis*	*Ostryopsis davidiana*	Ostryopsis19	JF977243	JN045671	JF942732	JF954828
Eudicotyledons	Betulaceae	*Ostryopsis*	*Ostryopsis davidiana*	Ostryopsis14	JF977248	JN045676	JF942737	JF954833
Eudicotyledons	Betulaceae	*Ostryopsis*	*Ostryopsis davidiana*	Ostryopsis15	JF977247	JN045675	JF942736	JF954832
Eudicotyledons	Betulaceae	*Ostryopsis*	*Ostryopsis davidiana*	Ostryopsis17	JF977245	JN045673	JF942734	JF954830
Eudicotyledons	Betulaceae	*Ostryopsis*	*Ostryopsis davidiana*	Ostryopsis11	JF977251	JN045679	JF942740	JF954836
Eudicotyledons	Betulaceae	*Ostryopsis*	*Ostryopsis davidiana*	Ostryopsis20	JF977242	JN045670	JF942731	JF954827
Eudicotyledons	Apiaceae	*Osmorhiza*	*Osmorhiza occidentalis*	PL4633	JF977240	JN045668	JF942729	JF954823
Eudicotyledons	Apiaceae	*Osmorhiza*	*Osmorhiza occidentalis*	W7116-10	JF977239	JN045667	JF942728	JF954822
Eudicotyledons	Apiaceae	*Osmorhiza*	*Osmorhiza occidentalis*	W7120-11	JF977238	JN045666	JF942727	JF954821
Eudicotyledons	Apiaceae	*Osmorhiza*	*Osmorhiza occidentalis*	H5560	JF977241	JN045669	JF942730	JF954824
Eudicotyledons	Apiaceae	*Osmorhiza*	*Osmorhiza longistylis*	W7151-1	JF977233	JN045661	JF942722	JF954816
Eudicotyledons	Apiaceae	*Osmorhiza*	*Osmorhiza longistylis*	WH564	JF977232	JN045660	JF942721	JF954815
Eudicotyledons	Apiaceae	*Osmorhiza*	*Osmorhiza longistylis*	W5094-2	JF977234	JN045662	JF942723	JF954817
Eudicotyledons	Apiaceae	*Osmorhiza*	*Osmorhiza glabrata*	T2419	JF977231	JN045659	JF942720	JF954814
Eudicotyledons	Apiaceae	*Osmorhiza*	*Osmorhiza glabrata*	W7464-16	JF977229	JN045657	JF942718	JF954812
Eudicotyledons	Apiaceae	*Osmorhiza*	*Osmorhiza glabrata*	Wen7456	JF977228	JN045656	JF942717	JF954811
Eudicotyledons	Apiaceae	*Osmorhiza*	*Osmorhiza glabrata*	W7315	JF977230	JN045658	JF942719	JF954813
Eudicotyledons	Apiaceae	*Osmorhiza*	*Osmorhiza depauperata*	W10456-1	JF977227	JN045655	JF942716	JF954810
Eudicotyledons	Apiaceae	*Osmorhiza*	*Osmorhiza depauperata*	W4742	JF977226	JN045654	JF942715	JF954809
Eudicotyledons	Apiaceae	*Osmorhiza*	*Osmorhiza depauperata*	W7387	JF977225	JN045653	JF942714	JF954808
Eudicotyledons	Apiaceae	*Osmorhiza*	*Osmorhiza depauperata*	W7415	JF977224	JN045652	JF942713	JF954807
Eudicotyledons	Apiaceae	*Osmorhiza*	*Osmorhiza claytonii*	W7139-2	JF977223	JN045651	JF942712	JF954806
Eudicotyledons	Apiaceae	*Osmorhiza*	*Osmorhiza claytonii*	Wen7300	JF977222	JN045650	JF942711	JF954805
Eudicotyledons	Apiaceae	*Osmorhiza*	*Osmorhiza brachypoda*	W7114	JF977221	JN045649	JF942710	JF954804
Eudicotyledons	Apiaceae	*Osmorhiza*	*Osmorhiza brachypoda*	W7122	JF977220	JN045648	JF942709	JF954803
Eudicotyledons	Apiaceae	*Osmorhiza*	*Osmorhiza berteroi*	W7374	JF977217	JN045643	JF942703	JF954797
Eudicotyledons	Apiaceae	*Osmorhiza*	*Osmorhiza berteroi*	Wen4732	JF977216	JN045642	JF942702	JF954796
Eudicotyledons	Apiaceae	*Osmorhiza*	*Osmorhiza berteroi*	Wen7397	JF977215	JN045641	JF942701	JF954795
Eudicotyledons	Apiaceae	*Osmorhiza*	*Osmorhiza berteroi*	W10465	JF977218	JN045646	JF942706	JF954800

Eudicotyledons	Apiaceae	*Osmorhiza*	*Osmorhiza aristata*	Lee-Han	JF977214	JN045640	JF942700	JF954794
Eudicotyledons	Apiaceae	*Osmorhiza*	*Osmorhiza aristata*	W5722-2	JF977213	JN045639	JF942699	JF954793
Eudicotyledons	Apiaceae	*Osmorhiza*	*Osmorhiza aristata*	W8500	JF977212	JN045638	JF942698	JF954792
Eudicotyledons	Rubiaceae	*Oldenlandia*	*Oldenlandia tenuipes*	WRJ1234-1	JF976508	JN044788	JF941806	JF953919
Eudicotyledons	Rubiaceae	*Oldenlandia*	*Oldenlandia tenuipes*	WRJ1234-2	JF976507	JN044787	JF941805	JF953918
Eudicotyledons	Rubiaceae	*Oldenlandia*	*Oldenlandia tenelliflora*	WRJ0992	JF976505	JN044785	JF941803	JF953916
Eudicotyledons	Rubiaceae	*Oldenlandia*	*Oldenlandia tenelliflora*	WRJ0012	JF976506	JN044786	JF941804	JF953917
Eudicotyledons	Rubiaceae	*Oldenlandia*	*Oldenlandia ovatifolia*	WRJ0020-2	JF976497	JN044777	JF941795	JF953908
Eudicotyledons	Rubiaceae	*Oldenlandia*	*Oldenlandia ovatifolia*	WRJ0020-1	JF976498	JN044778	JF941796	JF953909
Eudicotyledons	Rubiaceae	*Oldenlandia*	*Oldenlandia corymbosa*	WRJ0050	JF976491	JN044771	JF941789	JF953902
Eudicotyledons	Rubiaceae	*Oldenlandia*	*Oldenlandia corymbosa*	WRJ0016	JF976492	JN044772	JF941790	JF953903
Eudicotyledons	Rubiaceae	*Oldenlandia*	*Oldenlandia chrysotricha*	WRJ1230	JF976488	JN044768	JF941786	JF953901
Eudicotyledons	Rubiaceae	*Oldenlandia*	*Oldenlandia chrysotricha*	WRJCXX001	JF976487	JN044767	JF941785	JF953900
Eudicotyledons	Rubiaceae	*Oldenlandia*	*Oldenlandia bodinieri*	WRJ1247	JF976482	JN044762	JF941780	JF953895
Eudicotyledons	Rubiaceae	*Oldenlandia*	*Oldenlandia bodinieri*	WRJ1253-1	JF976481	JN044761	JF941779	JF953894
Eudicotyledons	Rubiaceae	*Oldenlandia*	*Oldenlandia bodinieri*	WRJ1253-2	JF976480	JN044760	JF941778	JF953893
Eudicotyledons	Cornaceae	*Nyssa*	*Nyssa sylvatica*	zhangcq0087	JF977172	JN045595	JF942654	JF954748
Eudicotyledons	Cornaceae	*Nyssa*	*Nyssa sylvatica*	zhangcq0088	JF977171	JN045594	JF942653	JF954747
Eudicotyledons	Cornaceae	*Nyssa*	*Nyssa sinensis*	zhangcq0089	JF977170	JN045593	JF942652	JF954746
Eudicotyledons	Cornaceae	*Nyssa*	*Nyssa sinensis*	zhangcq0090	JF977169	JN045592	JF942651	JF954745
Eudicotyledons	Cornaceae	*Nyssa*	*Nyssa shangszeensis*	zhangcq0096	JF977168	JN045588	JF942646	JF954740
Eudicotyledons	Cornaceae	*Nyssa*	*Nyssa shangszeensis*	zhangcq0098	JF977167	JN045587	JF942645	JF954739
Eudicotyledons	Nitrariaceae	*Nitraria*	*Nitraria sibirica*	D455	JF977164	JN045581	JF942637	JF954731
Eudicotyledons	Nitrariaceae	*Nitraria*	*Nitraria sibirica*	D454	JF977165	JN045582	JF942638	JF954732
Eudicotyledons	Nitrariaceae	*Nitraria*	*Nitraria sibirica*	D1164	JF977166	JN045583	JF942639	JF954733
Eudicotyledons	Nitrariaceae	*Nitraria*	*Nitraria roborowskii*	D1575	JF977163	JN045580	JF942636	JF954730
Eudicotyledons	Nitrariaceae	*Nitraria*	*Nitraria roborowskii*	D453	JF977162	JN045579	JF942635	JF954729
Eudicotyledons	Rubiaceae	*Mussaenda*	*Mussaenda treutleri*	ZDX044	JF977129	JN045534	JF942590	JF954682
Eudicotyledons	Rubiaceae	*Mussaenda*	*Mussaenda treutleri*	ZDX045	JF977128	JN045533	JF942589	JF954681
Eudicotyledons	Rubiaceae	*Mussaenda*	*Mussaenda pubescens*	ZDX006	JF977118	JN045522	JF942578	JF954670
Eudicotyledons	Rubiaceae	*Mussaenda*	*Mussaenda pubescens*	ZDX004	JF977120	JN045524	JF942580	JF954672
Eudicotyledons	Rubiaceae	*Mussaenda*	*Mussaenda pubescens*	ZDX005	JF977119	JN045523	JF942579	JF954671
Eudicotyledons	Rubiaceae	*Mussaenda*	*Mussaenda pubescens*	ZDX007	JF977117	JN045521	JF942577	JF954669
Eudicotyledons	Rubiaceae	*Mussaenda*	*Mussaenda pubescens*	ZDX001	JF977123	JN045527	JF942583	JF954675
Eudicotyledons	Rubiaceae	*Mussaenda*	*Mussaenda pubescens*	ZDX002	JF977122	JN045526	JF942582	JF954674
Eudicotyledons	Rubiaceae	*Mussaenda*	*Mussaenda pubescens*	ZDX003	JF977121	JN045525	JF942581	JF954673
Eudicotyledons	Rubiaceae	*Mussaenda*	*Mussaenda pubescens*	ZDX008	JF977116	JN045520	JF942576	JF954668
Eudicotyledons	Rubiaceae	*Mussaenda*	*Mussaenda macrophylla*	ZDX027	JF977113	JN045517	JF942573	JF954664
Eudicotyledons	Rubiaceae	*Mussaenda*	*Mussaenda macrophylla*	ZDX028	JF977112	JN045516	JF942572	JF954663
Eudicotyledons	Rubiaceae	*Mussaenda*	*Mussaenda laxiflora*	ZDX024	JF977111	JN045514	JF942570	JF954661

Eudicotyledons	Rubiaceae	*Mussaenda*	*Mussaenda laxiflora*	ZDX025	JF977110	JN045513	JF942569	JF954660
Eudicotyledons	Rubiaceae	*Mussaenda*	*Mussaenda kwangtungensis*	ZDX021	JF977108	JN045510	JF942566	JF954657
Eudicotyledons	Rubiaceae	*Mussaenda*	*Mussaenda kwangtungensis*	ZDX020	JF977109	JN045511	JF942567	JF954658
Eudicotyledons	Rubiaceae	*Mussaenda*	*Mussaenda hossei*	ZDX017	JF977106	JN045508	JF942565	JF954656
Eudicotyledons	Rubiaceae	*Mussaenda*	*Mussaenda hossei*	ZDX018	JF977105	JN045507	JF942564	JF954655
Eudicotyledons	Rubiaceae	*Mussaenda*	*Mussaenda divaricata*	ZDX015	JF977103	JN045501	JF942558	JF954649
Eudicotyledons	Rubiaceae	*Mussaenda*	*Mussaenda divaricata*	ZDX016	JF977102	JN045500	JF942557	JF954648
Eudicotyledons	Rubiaceae	*Mussaenda*	*Mussaenda divaricata*	ZDX014	JF977104	JN045502	JF942559	JF954650
Eudicotyledons	Rubiaceae	*Morinda*	*Morinda umbellata*	ZDX050	JF977056	JN045495	JF942552	JF954643
Eudicotyledons	Rubiaceae	*Morinda*	*Morinda umbellata*	ZDX049	JF977057	JN045496	JF942553	JF954644
Eudicotyledons	Rubiaceae	*Morinda*	*Morinda umbellata*	ZDX051	JF977055	JN045494	JF942551	JF954642
Eudicotyledons	Rubiaceae	*Morinda*	*Morinda officinalis*	ZDX056	JF977047	JN045486	JF942543	JF954634
Eudicotyledons	Rubiaceae	*Morinda*	*Morinda officinalis*	ZDX055	JF977048	JN045487	JF942544	JF954635
Eudicotyledons	Rubiaceae	*Morinda*	*Morinda officinalis*	ZDX054	JF977049	JN045488	JF942545	JF954636
Eudicotyledons	Rubiaceae	*Morinda*	*Morinda hainanensis*	ZDX062	JF977044	JN045483	JF942540	JF954631
Eudicotyledons	Rubiaceae	*Morinda*	*Morinda hainanensis*	ZDX061	JF977045	JN045484	JF942541	JF954632
Eudicotyledons	Rubiaceae	*Morinda*	*Morinda hainanensis*	ZDX060	JF977046	JN045485	JF942542	JF954633
Eudicotyledons	Rubiaceae	*Morinda*	*Morinda callicarpifolia*	ZDX068	JF977043	JN045482	JF942539	JF954630
Eudicotyledons	Rubiaceae	*Morinda*	*Morinda callicarpifolia*	ZDX069	JF977042	JN045481	JF942538	JF954629
Eudicotyledons	Rubiaceae	*Morinda*	*Morinda badia*	ZDX067	JF977040	JN045476	JF942533	JF954624
Eudicotyledons	Rubiaceae	*Morinda*	*Morinda badia*	ZDX066	JF977041	JN045477	JF942534	JF954625
Eudicotyledons	Rubiaceae	*Luculia*	*Luculia yunnanensis*	Zhouw0001	JF976883	JN045281	JF942333	JF954419
Eudicotyledons	Rubiaceae	*Luculia*	*Luculia yunnanensis*	Zhouw0002	JF976882	JN045280	JF942332	JF954418
Eudicotyledons	Rubiaceae	*Luculia*	*Luculia yunnanensis*	Zhouw0004	JF976880	JN045278	JF942330	JF954416
Eudicotyledons	Rubiaceae	*Luculia*	*Luculia yunnanensis*	Zhouw0005	JF976879	JN045277	JF942329	JF954415
Eudicotyledons	Rubiaceae	*Luculia*	*Luculia yunnanensis*	Zhouw0003	JF976881	JN045279	JF942331	JF954417
Eudicotyledons	Rubiaceae	*Luculia*	*Luculia pinceana*	Zhouw0009	JF976871	JN045269	JF942321	JF954407
Eudicotyledons	Rubiaceae	*Luculia*	*Luculia pinceana*	Zhouw0010	JF976870	JN045268	JF942320	JF954406
Eudicotyledons	Rubiaceae	*Luculia*	*Luculia pinceana*	Zhouw0008	JF976872	JN045270	JF942322	JF954408
Eudicotyledons	Rubiaceae	*Luculia*	*Luculia pinceana*	Zhouw0007	JF976873	JN045271	JF942323	JF954409
Eudicotyledons	Rubiaceae	*Luculia*	*Luculia pinceana*	Zhouw0011	JF976869	JN045267	JF942319	JF954405
Eudicotyledons	Caprifoliaceae	*Lonicera*	*Lonicera rupicola*	Z288	JF976868	JN045258	JF942310	JF954401
Eudicotyledons	Caprifoliaceae	*Lonicera*	*Lonicera rupicola*	Z291	JF976866	JN045255	JF942307	JF954398
Eudicotyledons	Caprifoliaceae	*Lonicera*	*Lonicera rupicola*	Z290	JF976867	JN045256	JF942308	JF954399
Eudicotyledons	Caprifoliaceae	*Lonicera*	*Lonicera hispida*	D1947	JF976864	JN045249	JF942301	JF954393
Eudicotyledons	Caprifoliaceae	*Lonicera*	*Lonicera hispida*	Z281	JF976863	JN045248	JF942300	JF954392
Eudicotyledons	Caprifoliaceae	*Lonicera*	*Lonicera hispida*	Z282	JF976862	JN045247	JF942299	JF954391
Eudicotyledons	Caprifoliaceae	*Lonicera*	*Lonicera hispida*	Z283	JF976861	JN045246	JF942298	JF954390
Eudicotyledons	Oleaceae	*Ligustrum*	*Ligustrum xingrenense*	OLgz_031	JF830335	JF830292	JF830443	JF830520
Eudicotyledons	Oleaceae	*Ligustrum*	*Ligustrum xingrenense*	OLgz_025	JF830337	JF830291	JF830442	JF830519

Eudicotyledons	Oleaceae	*Ligustrum*	*Ligustrum xingrenense*	OLgz_021	JF830336	JF830293	JF830444	JF830521
Eudicotyledons	Oleaceae	*Ligustrum*	*Ligustrum*	OL_0803	JF830400	JF830269	JF830420	JF830497
Eudicotyledons	Oleaceae	*Ligustrum*	*Ligustrum*	QYLIST_01	JF830401	JF830267	JF830418	JF830495
Eudicotyledons	Oleaceae	*Ligustrum*	*Ligustrum*	QYLIST_02	JF830402	JF830268	JF830419	JF830496
Eudicotyledons	Oleaceae	*Ligustrum*	*Ligustrum sempervirens*	Ye_&_Liu_455-1	JF830381	JF830259	JF830410	JF830488
Eudicotyledons	Oleaceae	*Ligustrum*	*Ligustrum sempervirens*	Ye_&_Liu_455-2	JF830383	JF830258	JF830409	JF830487
Eudicotyledons	Oleaceae	*Ligustrum*	*Ligustrum sempervirens*	Ye_&_Liu_455-3	JF830382	JF830260	JF830411	JF830489
Eudicotyledons	Oleaceae	*Ligustrum*	*Ligustrum robustum*	IMDXPG1602	JF976854	JN045239	JF942293	JF954385
Eudicotyledons	Oleaceae	*Ligustrum*	*Ligustrum robustum*	IMDXPG1603	JF976853	JN045238	JF942292	JF954384
Eudicotyledons	Oleaceae	*Ligustrum*	*Ligustrum robustum*	IMDXPG1601	JF976855	JN045240	JF942294	JF954386
Eudicotyledons	Oleaceae	*Ligustrum*	*Ligustrum quihoui*	Dong_&_Ren_0139	JF830357	JF830275	JF830426	JF830503
Eudicotyledons	Oleaceae	*Ligustrum*	*Ligustrum quihoui*	Chen_20100001	JF830358	JF830270	JF830421	JF830498
Eudicotyledons	Oleaceae	*Ligustrum*	*Ligustrum quihoui*	QYLIQU_01	JF830355	JF830271	JF830422	JF830499
Eudicotyledons	Oleaceae	*Ligustrum*	*Ligustrum quihoui*	QYLIQU_02	JF830354	JF830272	JF830423	JF830500
Eudicotyledons	Oleaceae	*Ligustrum*	*Ligustrum quihoui*	PS0977MT01	GQ434621	GQ43522	GQ43654	GQ43417
Eudicotyledons	Oleaceae	*Ligustrum*	*Ligustrum ovalifolium*	OLlgs_003	JF830390	JF830305	JF830456	JF830533
Eudicotyledons	Oleaceae	*Ligustrum*	*Ligustrum ovalifolium*	OLOV_12	JF830394	JF830312	JF830463	JF830540
Eudicotyledons	Oleaceae	*Ligustrum*	*Ligustrum ovalifolium*	OLOV_09	JF830393	JF830309	JF830460	JF830537
Eudicotyledons	Oleaceae	*Ligustrum*	*Ligustrum ovalifolium*	Chen_2010036	JF830396	JF830307	JF830458	JF830535
Eudicotyledons	Oleaceae	*Ligustrum*	*Ligustrum ovalifolium*	QYLIOV_04	JF830392	JF830304	JF830455	JF830532
Eudicotyledons	Oleaceae	*Ligustrum*	*Ligustrum ovalifolium*	QYLIOV_02	JF830391	JF830306	JF830457	JF830534
Eudicotyledons	Oleaceae	*Ligustrum*	*Ligustrum ovalifolium*	QYLIOV_03	JF830388	JF830310	JF830461	JF830538
Eudicotyledons	Oleaceae	*Ligustrum*	*Ligustrum lucidum*	IMDXPG1501	JF976848	JN045229	JF942289	JF954378
Eudicotyledons	Oleaceae	*Ligustrum*	*Ligustrum lucidum*	IMDXPG1503	JF976846	JN045227	JF942287	JF954376
Eudicotyledons	Oleaceae	*Ligustrum*	*Ligustrum lucidum*	IMDXPG1502	JF976847	JN045228	JF942288	JF954377
Eudicotyledons	Oleaceae	*Ligustrum*	*Ligustrum lucidum*	OLLU_012	JF830345	JF830331	JF830482	JF830558
Eudicotyledons	Oleaceae	*Ligustrum*	*Ligustrum lucidum*	OLLU_011	JF830344	JF830327	JF830478	JF830554
Eudicotyledons	Oleaceae	*Ligustrum*	*Ligustrum lucidum*	OLLU_005	JF830343	JF830332	JF830483	JF830559
Eudicotyledons	Oleaceae	*Ligustrum*	*Ligustrum lucidum*	OLLU_004	JF830347	JF830329	JF830480	JF830556
Eudicotyledons	Oleaceae	*Ligustrum*	*Ligustrum lucidum*	OLLU_001	JF830341	JF830333	JF830484	JF830560
Eudicotyledons	Oleaceae	*Ligustrum*	*Ligustrum lucidum*	OLLU_003	JF830346	JF830328	JF830479	JF830555
Eudicotyledons	Oleaceae	*Ligustrum*	*Ligustrum lucidum*	OLLU_009	JF830342	JF830330	JF830481	JF830557
Eudicotyledons	Oleaceae	*Ligustrum*	*Ligustrum japonicum*	OLJA_010	JF830405	JF830326	JF830477	JF830553
Eudicotyledons	Oleaceae	*Ligustrum*	*Ligustrum japonicum*	OLJA_013	JF830403	JF830322	JF830473	JF830550
Eudicotyledons	Oleaceae	*Ligustrum*	*Ligustrum japonicum*	OLJA_008	JF830406	JF830323	JF830474	JF830551
Eudicotyledons	Oleaceae	*Ligustrum*	*Ligustrum japonicum*	OLJA_005	JF830407	JF830324	JF830475	JF830552
Eudicotyledons	Oleaceae	*Ligustrum*	*Ligustrum henryi*	QYLIHE_007	JF830373	JF830266	JF830417	JF830494
Eudicotyledons	Oleaceae	*Ligustrum*	*Ligustrum henryi*	IMDXPG2203	JF976842	JN045222	JF942284	JF954373
Eudicotyledons	Oleaceae	*Ligustrum*	*Ligustrum henryi*	IMDXPG2202	JF976843	JN045223	JF942285	JF954374
Eudicotyledons	Oleaceae	*Ligustrum*	*Ligustrum henryi*	QYLIHE_001	JF830372	JF830265	JF830416	JF830493

Eudicotyledons	Oleaceae	*Ligustrum*	*Ligustrum henryi*	QYLIHE_003	JF830371	JF830263	JF830414	JF830491
Eudicotyledons	Oleaceae	*Ligustrum*	*Ligustrum henryi*	QYLIHE_005	JF830374	JF830262	JF830413	JF830490
Eudicotyledons	Oleaceae	*Ligustrum*	*Ligustrum henryi*	QYLIHE_004	JF830375	JF830264	JF830415	JF830492
Eudicotyledons	Oleaceae	*Ligustrum*	*Ligustrum gracile*	OL_0301	JF830348	JF830316	JF830467	JF830544
Eudicotyledons	Oleaceae	*Ligustrum*	*Ligustrum gracile*	OL_0304	JF830350	JF830318	JF830469	JF830546
Eudicotyledons	Oleaceae	*Ligustrum*	*Ligustrum gracile*	Ye_&_Liu_509	JF830349	JF830317	JF830468	JF830545
Eudicotyledons	Oleaceae	*Ligustrum*	*Ligustrum expansum*	OLyc_124	JF830359	JF830279	JF830430	JF830507
Eudicotyledons	Oleaceae	*Ligustrum*	*Ligustrum expansum*	OLhn_007	JF830360	JF830280	JF830431	JF830508
Eudicotyledons	Oleaceae	*Ligustrum*	*Ligustrum expansum*	OLhn_001	JF830361	JF830281	JF830432	JF830509
Eudicotyledons	Oleaceae	*Ligustrum*	*Ligustrum delavayanum*	OLlgs_001	JF830384	JF830294	JF830445	JF830522
Eudicotyledons	Oleaceae	*Ligustrum*	*Ligustrum delavayanum*	OLjfs_045	JF830386	JF830295	JF830446	JF830523
Eudicotyledons	Oleaceae	*Ligustrum*	*Ligustrum delavayanum*	OL_2110	JF830385	JF830297	JF830448	JF830525
Eudicotyledons	Oleaceae	*Ligustrum*	*Ligustrum delavayanum*	OL_2108	JF830387	JF830296	JF830447	JF830524
Eudicotyledons	Oleaceae	*Ligustrum*	*Ligustrum confusum*	OLyn_119	JF830353	JF830278	JF830429	JF830506
Eudicotyledons	Oleaceae	*Ligustrum*	*Ligustrum confusum*	OLlj_046	JF830351	JF830276	JF830427	JF830504
Eudicotyledons	Oleaceae	*Ligustrum*	*Ligustrum confusum*	OLyn_108	JF830352	JF830277	JF830428	JF830505
Eudicotyledons	Asteraceae	*Ligularia*	*Ligularia virgaurea*	PG090939	JF976840	JN045219	JF942283	JF954372
Eudicotyledons	Asteraceae	*Ligularia*	*Ligularia virgaurea*	PG090960	JF976838	JN045217	JF942281	JF954370
Eudicotyledons	Asteraceae	*Ligularia*	*Ligularia virgaurea*	PG090954	JF976839	JN045218	JF942282	JF954371
Eudicotyledons	Asteraceae	*Ligularia*	*Ligularia virgaurea*	PG090966	JF976837	JN045216	JF942280	JF954369
Eudicotyledons	Asteraceae	*Ligularia*	*Ligularia vellerea*	PG090914	JF976835	JN045214	JF942278	JF954367
Eudicotyledons	Asteraceae	*Ligularia*	*Ligularia vellerea*	PG090911	JF976836	JN045215	JF942279	JF954368
Eudicotyledons	Asteraceae	*Ligularia*	*Ligularia vellerea*	PG090932	JF976834	JN045213	JF942277	JF954366
Eudicotyledons	Asteraceae	*Ligularia*	*Ligularia tongolensis*	PG090949	JF976831	JN045210	JF942274	JF954363
Eudicotyledons	Asteraceae	*Ligularia*	*Ligularia tongolensis*	PG090965	JF976830	JN045209	JF942273	JF954362
Eudicotyledons	Asteraceae	*Ligularia*	*Ligularia tongolensis*	PG090906	JF976833	JN045212	JF942276	JF954365
Eudicotyledons	Asteraceae	*Ligularia*	*Ligularia subspicata*	PG090981	JF976824	JN045203	JF942267	JF954356
Eudicotyledons	Asteraceae	*Ligularia*	*Ligularia subspicata*	PG090959	JF976826	JN045205	JF942269	JF954358
Eudicotyledons	Asteraceae	*Ligularia*	*Ligularia subspicata*	PG090972	JF976825	JN045204	JF942268	JF954357
Eudicotyledons	Asteraceae	*Ligularia*	*Ligularia subspicata*	PG090912	JF976829	JN045208	JF942272	JF954361
Eudicotyledons	Asteraceae	*Ligularia*	*Ligularia subspicata*	PG090938	JF976827	JN045206	JF942270	JF954359
Eudicotyledons	Asteraceae	*Ligularia*	*Ligularia subspicata*	PG090925	JF976828	JN045207	JF942271	JF954360
Eudicotyledons	Asteraceae	*Ligularia*	*Ligularia pleurocaulis*	PG090958	JF976822	JN045201	JF942265	JF954354
Eudicotyledons	Asteraceae	*Ligularia*	*Ligularia pleurocaulis*	PG090940	JF976823	JN045202	JF942266	JF954355
Eudicotyledons	Asteraceae	*Ligularia*	*Ligularia dictyoneura*	PG090968	JF976814	JN045189	JF942253	JF954342
Eudicotyledons	Asteraceae	*Ligularia*	*Ligularia dictyoneura*	PG090922	JF976815	JN045191	JF942255	JF954344
Eudicotyledons	Asteraceae	*Ligularia*	*Ligularia cymbulifera*	PG090937	JF976810	JN045185	JF942249	JF954338
Eudicotyledons	Asteraceae	*Ligularia*	*Ligularia cymbulifera*	PG090975	JF976807	JN045182	JF942246	JF954335
Eudicotyledons	Asteraceae	*Ligularia*	*Ligularia cymbulifera*	PG090915	JF976812	JN045187	JF942251	JF954340
Eudicotyledons	Asteraceae	*Ligularia*	*Ligularia cymbulifera*	PG090910	JF976813	JN045188	JF942252	JF954341

Eudicotyledons	Asteraceae	*Ligularia*	*Ligularia cymbulifera*	PG090936	JF976811	JN045186	JF942250	JF954339
Eudicotyledons	Asteraceae	*Ligularia*	*Ligularia cymbulifera*	PG090962	JF976808	JN045183	JF942247	JF954336
Eudicotyledons	Rubiaceae	*Leptodermis*	*Leptodermis vestita*	WRJ1120C	JF976805	JN045178	JF942242	JF954331
Eudicotyledons	Rubiaceae	*Leptodermis*	*Leptodermis vestita*	WRJ1119D	JF976806	JN045179	JF942243	JF954332
Eudicotyledons	Rubiaceae	*Leptodermis*	*Leptodermis scabrida*	WRJ42	JF976798	JN045171	JF942237	JF954326
Eudicotyledons	Rubiaceae	*Leptodermis*	*Leptodermis scabrida*	WRJ38C	JF976800	JN045173	JF942239	JF954328
Eudicotyledons	Rubiaceae	*Leptodermis*	*Leptodermis scabrida*	WRJ38D	JF976799	JN045172	JF942238	JF954327
Eudicotyledons	Rubiaceae	*Leptodermis*	*Leptodermis pilosa*	WRJ55C	JF976786	JN045157	JF942226	JF954315
Eudicotyledons	Rubiaceae	*Leptodermis*	*Leptodermis pilosa*	WRJ2C	JF976787	JN045158	JF942227	JF954316
Eudicotyledons	Rubiaceae	*Leptodermis*	*Leptodermis ovata*	WRJ1106C	JF976785	JN045156	JF942225	JF954314
Eudicotyledons	Rubiaceae	*Leptodermis*	*Leptodermis ovata*	WRJ1107D	JF976784	JN045155	JF942224	JF954313
Eudicotyledons	Rubiaceae	*Leptodermis*	*Leptodermis ludlowii*	WRJ43D	JF976801	JN045174	JF942240	JF954329
Eudicotyledons	Rubiaceae	*Leptodermis*	*Leptodermis ludlowii*	WRJ43C	JF976802	JN045175	JF942241	JF954330
Eudicotyledons	Rubiaceae	*Leptodermis*	*Leptodermis hirsutiflora*	WRJ44D	JF976782	JN045153	JF942222	JF954311
Eudicotyledons	Rubiaceae	*Leptodermis*	*Leptodermis hirsutiflora*	WRJ44C	JF976783	JN045154	JF942223	JF954312
Eudicotyledons	Rubiaceae	*Leptodermis*	*Leptodermis buxifolia*	WRJ45D	JF976778	JN045149	JF942220	JF954309
Eudicotyledons	Rubiaceae	*Leptodermis*	*Leptodermis buxifolia*	WRJ45C	JF976779	JN045150	JF942221	JF954310
Eudicotyledons	Brassicaceae	*Lepidium*	*Lepidium ruderale*	D1577	JF976777	JN045148	JF942219	JF954308
Eudicotyledons	Brassicaceae	*Lepidium*	*Lepidium ruderale*	Z770	JF976776	JN045147	JF942218	JF954307
Eudicotyledons	Brassicaceae	*Lepidium*	*Lepidium ruderale*	Z772	JF976774	JN045145	JF942216	JF954305
Eudicotyledons	Brassicaceae	*Lepidium*	*Lepidium ruderale*	Z771	JF976775	JN045146	JF942217	JF954306
Eudicotyledons	Brassicaceae	*Lepidium*	*Lepidium perfoliatum*	Z767	JF976773	JN045144	JF942215	JF954304
Eudicotyledons	Brassicaceae	*Lepidium*	*Lepidium perfoliatum*	Z769	JF976771	JN045142	JF942213	JF954302
Eudicotyledons	Brassicaceae	*Lepidium*	*Lepidium perfoliatum*	Z768	JF976772	JN045143	JF942214	JF954303
Eudicotyledons	Brassicaceae	*Lepidium*	*Lepidium apetalum*	D1318	JF976756	JN045128	JF942200	JF954289
Eudicotyledons	Brassicaceae	*Lepidium*	*Lepidium apetalum*	A375	JF976767	JN045138	JF942209	JF954299
Eudicotyledons	Brassicaceae	*Lepidium*	*Lepidium apetalum*	A377	JF976765	JN045136	JF942207	JF954297
Eudicotyledons	Brassicaceae	*Lepidium*	*Lepidium apetalum*	A376	JF976766	JN045137	JF942208	JF954298
Eudicotyledons	Brassicaceae	*Lepidium*	*Lepidium apetalum*	A385	JF976757	JN045129	JF942201	JF954290
Eudicotyledons	Brassicaceae	*Lepidium*	*Lepidium apetalum*	A380	JF976762	JN045134	JF942205	JF954295
Eudicotyledons	Brassicaceae	*Lepidium*	*Lepidium apetalum*	A382	JF976760	JN045132	JF942203	JF954293
Eudicotyledons	Brassicaceae	*Lepidium*	*Lepidium apetalum*	Z766	JF976753	JN045126	JF942197	JF954287
Eudicotyledons	Brassicaceae	*Lepidium*	*Lepidium apetalum*	A374	JF976768	JN045139	JF942210	JF954300
Eudicotyledons	Brassicaceae	*Lepidium*	*Lepidium apetalum*	A378	JF976764	JN045135	JF942206	JF954296
Eudicotyledons	Brassicaceae	*Lepidium*	*Lepidium apetalum*	A381	JF976761	JN045133	JF942204	JF954294
Eudicotyledons	Brassicaceae	*Lepidium*	*Lepidium apetalum*	Z765	JF976754	JN045127	JF942198	JF954288
Eudicotyledons	Brassicaceae	*Lepidium*	*Lepidium apetalum*	A384	JF976758	JN045130	JF942202	JF954291
Eudicotyledons	Brassicaceae	*Lepidium*	*Lepidium apetalum*	Z764	JF976769	JN045140	JF942211	JF954301
Eudicotyledons	Aquifoliaceae	*Ilex*	*Ilex cornuta*	Z148	JF976683	JN044939	JF941994	JF954088
Eudicotyledons	Aquifoliaceae	*Ilex*	*Ilex cornuta*	Z147	JF976684	JN044940	JF941995	JF954089

Eudicotyledons	Aquifoliaceae	*Ilex*	*Ilex cornuta*	Z145	JF976686	JN044942	JF941997	JF954091
Eudicotyledons	Aquifoliaceae	*Ilex*	*Ilex cornuta*	Z143	JF976688	JN044943	JF941999	JF954093
Eudicotyledons	Aquifoliaceae	*Ilex*	*Ilex cornuta*	Z149	JF976682	JN044938	JF941993	JF954087
Eudicotyledons	Aquifoliaceae	*Ilex*	*Ilex cornuta*	Z146	JF976685	JN044941	JF941996	JF954090
Eudicotyledons	Aquifoliaceae	*Ilex*	*Ilex chinensis*	Z142	JF976677	JN044933	JF941987	JF954082
Eudicotyledons	Aquifoliaceae	*Ilex*	*Ilex chinensis*	Z141	JF976678	JN044934	JF941988	JF954083
Eudicotyledons	Aquifoliaceae	*Ilex*	*Ilex chinensis*	Z138	JF976680	JN044936	JF941991	JF954085
Eudicotyledons	Aquifoliaceae	*Ilex*	*Ilex chinensis*	Z139	JF976679	JN044935	JF941990	JF954084
Eudicotyledons	Aquifoliaceae	*Ilex*	*Ilex chinensis*	Z137	JF976681	JN044937	JF941992	JF954086
Eudicotyledons	Hypericaceae	*Hypericum*	*Hypericum przewalskii*	A327	JF976672	JN044932	JF941982	JF954079
Eudicotyledons	Hypericaceae	*Hypericum*	*Hypericum przewalskii*	A328	JF976671	JN044931	JF941981	JF954078
Eudicotyledons	Hypericaceae	*Hypericum*	*Hypericum przewalskii*	A329	JF976670	JN044930	JF941980	JF954077
Eudicotyledons	Hypericaceae	*Hypericum*	*Hypericum monogynum*	A326	JF976668	JN044928	JF941978	JF954075
Eudicotyledons	Hypericaceae	*Hypericum*	*Hypericum monogynum*	A323	JF976669	JN044929	JF941979	JF954076
Eudicotyledons	Hydrangeaceae	*Hydrangea*	*Hydrangea heteromalla*	D1682	JF976655	JN044926	JF941962	JF954071
Eudicotyledons	Hydrangeaceae	*Hydrangea*	*Hydrangea heteromalla*	D1242	JF976656	JN044927	JF941963	JF954072
Eudicotyledons	Hydrangeaceae	*Hydrangea*	*Hydrangea aspera*	H4	JF976654	JN044923	JF941959	JF954068
Eudicotyledons	Hydrangeaceae	*Hydrangea*	*Hydrangea aspera*	H5	JF976653	JN044922	JF941958	JF954067
Eudicotyledons	Hydrangeaceae	*Hydrangea*	*Hydrangea anomala*	D978	JF976652	JN044921	JF941957	JF954066
Eudicotyledons	Hydrangeaceae	*Hydrangea*	*Hydrangea anomala*	H1	JF976651	JN044920	JF941956	JF954065
Eudicotyledons	Hydrangeaceae	*Hydrangea*	*Hydrangea anomala*	H21	JF976650	JN044919	JF941955	JF954064
Eudicotyledons	Elaeagnaceae	*Hippophae*	*Hippophae tibetana*	LiujqH004	JF976646	JN044915	JF941951	JF954060
Eudicotyledons	Elaeagnaceae	*Hippophae*	*Hippophae tibetana*	LiujqH005	JF976645	JN044914	JF941950	JF954059
Eudicotyledons	Elaeagnaceae	*Hippophae*	*Hippophae tibetana*	LiujqH001	JF976649	JN044918	JF941954	JF954063
Eudicotyledons	Elaeagnaceae	*Hippophae*	*Hippophae tibetana*	LiujqH002	JF976648	JN044917	JF941953	JF954062
Eudicotyledons	Elaeagnaceae	*Hippophae*	*Hippophae tibetana*	LiujqH003	JF976647	JN044916	JF941952	JF954061
Eudicotyledons	Elaeagnaceae	*Hippophae*	*Hippophae salicifolia*	LiujqH024	JF976641	JN044910	JF941946	JF954055
Eudicotyledons	Elaeagnaceae	*Hippophae*	*Hippophae salicifolia*	LiujqH022	JF976643	JN044912	JF941948	JF954057
Eudicotyledons	Elaeagnaceae	*Hippophae*	*Hippophae salicifolia*	LiujqH023	JF976642	JN044911	JF941947	JF954056
Eudicotyledons	Elaeagnaceae	*Hippophae*	*Hippophae salicifolia*	LiujqH021	JF976644	JN044913	JF941949	JF954058
Eudicotyledons	Elaeagnaceae	*Hippophae*	*Hippophae salicifolia*	LiujqH025	JF976640	JN044909	JF941945	JF954054
Eudicotyledons	Cucurbitaceae	*Hemsleya*	*Hemsleya zhejiangensis*	LiHT0513	JF976603	JN044883	JF941905	JF954014
Eudicotyledons	Cucurbitaceae	*Hemsleya*	*Hemsleya zhejiangensis*	LiHT0511	JF976605	JN044885	JF941907	JF954016
Eudicotyledons	Cucurbitaceae	*Hemsleya*	*Hemsleya zhejiangensis*	LiHT0512	JF976604	JN044884	JF941906	JF954015
Eudicotyledons	Cucurbitaceae	*Hemsleya*	*Hemsleya turbinata*	LiHT10151	JF976601	JN044881	JF941903	JF954012
Eudicotyledons	Cucurbitaceae	*Hemsleya*	*Hemsleya turbinata*	LiHT10150	JF976602	JN044882	JF941904	JF954013
Eudicotyledons	Cucurbitaceae	*Hemsleya*	*Hemsleya turbinata*	LiHT10153	JF976599	JN044879	JF941901	JF954010
Eudicotyledons	Cucurbitaceae	*Hemsleya*	*Hemsleya turbinata*	LiHT10152	JF976600	JN044880	JF941902	JF954011
Eudicotyledons	Cucurbitaceae	*Hemsleya*	*Hemsleya sp. HTL-2007*	LiHT0543	JF976564	JN044844	JF941866	JF953975
Eudicotyledons	Cucurbitaceae	*Hemsleya*	*Hemsleya sp. HTL-2007*	LiHT0545	JF976562	JN044842	JF941864	JF953973

Eudicotyledons	Cucurbitaceae	*Hemsleya*	*Hemsleya sp. HTL-2007*	LiHT0544	JF976563	JN044843	JF941865	JF953974
Eudicotyledons	Cucurbitaceae	*Hemsleya*	*Hemsleya pengxianensis*	LiHT0533	JF976597	JN044877	JF941899	JF954008
Eudicotyledons	Cucurbitaceae	*Hemsleya*	*Hemsleya pengxianensis*	LiHT1054	JF976596	JN044876	JF941898	JF954007
Eudicotyledons	Cucurbitaceae	*Hemsleya*	*Hemsleya pengxianensis*	LiHT0532	JF976598	JN044878	JF941900	JF954009
Eudicotyledons	Cucurbitaceae	*Hemsleya*	*Hemsleya panlongqi*	LiHT0540	JF976595	JN044875	JF941897	JF954006
Eudicotyledons	Cucurbitaceae	*Hemsleya*	*Hemsleya panlongqi*	LiHT0541	JF976594	JN044874	JF941896	JF954005
Eudicotyledons	Cucurbitaceae	*Hemsleya*	*Hemsleya panlongqi*	LiHT0542	JF976593	JN044873	JF941895	JF954004
Eudicotyledons	Cucurbitaceae	*Hemsleya*	*Hemsleya panacis-*	LiHT10133-1	JF976592	JN044872	JF941894	JF954003
Eudicotyledons	Cucurbitaceae	*Hemsleya*	*Hemsleya panacis-*	LiHT10133-3	JF976590	JN044870	JF941892	JF954001
Eudicotyledons	Cucurbitaceae	*Hemsleya*	*Hemsleya panacis-*	LiHT10133-2	JF976591	JN044871	JF941893	JF954002
Eudicotyledons	Cucurbitaceae	*Hemsleya*	*Hemsleya panacis-*	LiHT10136	JF976589	JN044869	JF941891	JF954000
Eudicotyledons	Cucurbitaceae	*Hemsleya*	*Hemsleya omeiensis*	LiHT0536	JF976587	JN044867	JF941889	JF953998
Eudicotyledons	Cucurbitaceae	*Hemsleya*	*Hemsleya omeiensis*	LiHT0539	JF976586	JN044866	JF941888	JF953997
Eudicotyledons	Cucurbitaceae	*Hemsleya*	*Hemsleya omeiensis*	LiHT0535	JF976588	JN044868	JF941890	JF953999
Eudicotyledons	Cucurbitaceae	*Hemsleya*	*Hemsleya mitrata*	LiHT0553	JF976582	JN044862	JF941884	JF953993
Eudicotyledons	Cucurbitaceae	*Hemsleya*	*Hemsleya mitrata*	LiHT0552	JF976583	JN044863	JF941885	JF953994
Eudicotyledons	Cucurbitaceae	*Hemsleya*	*Hemsleya mitrata*	LiHT0551	JF976584	JN044864	JF941886	JF953995
Eudicotyledons	Cucurbitaceae	*Hemsleya*	*Hemsleya mitrata*	LiHT0550	JF976585	JN044865	JF941887	JF953996
Eudicotyledons	Cucurbitaceae	*Hemsleya*	*Hemsleya macrosperma*	LiHT0557	JF976581	JN044861	JF941883	JF953992
Eudicotyledons	Cucurbitaceae	*Hemsleya*	*Hemsleya macrosperma*	LiHT0558	JF976580	JN044860	JF941882	JF953991
Eudicotyledons	Cucurbitaceae	*Hemsleya*	*Hemsleya macrosperma*	LiHT0571	JF976579	JN044859	JF941881	JF953990
Eudicotyledons	Cucurbitaceae	*Hemsleya*	*Hemsleya macrosperma*	LiHT0572	JF976578	JN044858	JF941880	JF953989
Eudicotyledons	Cucurbitaceae	*Hemsleya*	*Hemsleya macrocarpa*	LiHT10149	JF976574	JN044854	JF941876	JF953985
Eudicotyledons	Cucurbitaceae	*Hemsleya*	*Hemsleya macrocarpa*	LiHT10159	JF976573	JN044853	JF941875	JF953984
Eudicotyledons	Cucurbitaceae	*Hemsleya*	*Hemsleya macrocarpa*	LiHT10162	JF976572	JN044852	JF941874	JF953983
Eudicotyledons	Cucurbitaceae	*Hemsleya*	*Hemsleya macrocarpa*	LiHT0583	JF976577	JN044857	JF941879	JF953988
Eudicotyledons	Cucurbitaceae	*Hemsleya*	*Hemsleya macrocarpa*	LiHT05837	JF976575	JN044855	JF941877	JF953986
Eudicotyledons	Cucurbitaceae	*Hemsleya*	*Hemsleya macrocarpa*	LiHT05836	JF976576	JN044856	JF941878	JF953987
Eudicotyledons	Cucurbitaceae	*Hemsleya*	*Hemsleya longicarpa*	LiHT0534	JF976571	JN044851	JF941873	JF953982
Eudicotyledons	Cucurbitaceae	*Hemsleya*	*Hemsleya longicarpa*	LiHT1055	JF976570	JN044850	JF941872	JF953981
Eudicotyledons	Cucurbitaceae	*Hemsleya*	*Hemsleya lijiangensis*	LiHT10201-1	JF976566	JN044846	JF941868	JF953977
Eudicotyledons	Cucurbitaceae	*Hemsleya*	*Hemsleya lijiangensis*	LiHT0546	JF976569	JN044849	JF941871	JF953980
Eudicotyledons	Cucurbitaceae	*Hemsleya*	*Hemsleya lijiangensis*	LiHT0547	JF976568	JN044848	JF941870	JF953979
Eudicotyledons	Cucurbitaceae	*Hemsleya*	*Hemsleya lijiangensis*	LiHT0548	JF976567	JN044847	JF941869	JF953978
Eudicotyledons	Cucurbitaceae	*Hemsleya*	*Hemsleya lijiangensis*	LiHT10202-2	JF976565	JN044845	JF941867	JF953976
Eudicotyledons	Cucurbitaceae	*Hemsleya*	*Hemsleya graciliflora*	LiHT0568-2	JF976560	JN044840	JF941862	JF953971
Eudicotyledons	Cucurbitaceae	*Hemsleya*	*Hemsleya graciliflora*	LiHT0569A	JF976558	JN044838	JF941860	JF953969
Eudicotyledons	Cucurbitaceae	*Hemsleya*	*Hemsleya graciliflora*	LiHT0568	JF976561	JN044841	JF941863	JF953972
Eudicotyledons	Cucurbitaceae	*Hemsleya*	*Hemsleya graciliflora*	LiHT0569	JF976559	JN044839	JF941861	JF953970
Eudicotyledons	Cucurbitaceae	*Hemsleya*	*Hemsleya gigantha*	LiHT0526	JF976557	JN044837	JF941859	JF953968

Eudicotyledons	Cucurbitaceae	*Hemsleya*	*Hemsleya gigantha*	LiHT0527	JF976556	JN044836	JF941858	JF953967
Eudicotyledons	Cucurbitaceae	*Hemsleya*	*Hemsleya gigantha*	LiHT0528	JF976555	JN044835	JF941857	JF953966
Eudicotyledons	Cucurbitaceae	*Hemsleya*	*Hemsleya gigantha*	LiHT0529	JF976554	JN044834	JF941856	JF953965
Eudicotyledons	Cucurbitaceae	*Hemsleya*	*Hemsleya endecaphylla*	LiHT10184	JF976553	JN044833	JF941855	JF953964
Eudicotyledons	Cucurbitaceae	*Hemsleya*	*Hemsleya endecaphylla*	LiHT10185	JF976552	JN044832	JF941854	JF953963
Eudicotyledons	Cucurbitaceae	*Hemsleya*	*Hemsleya ellipsoidea*	LiHT0517	JF976548	JN044828	JF941850	JF953959
Eudicotyledons	Cucurbitaceae	*Hemsleya*	*Hemsleya ellipsoidea*	LiHT0516	JF976549	JN044829	JF941851	JF953960
Eudicotyledons	Cucurbitaceae	*Hemsleya*	*Hemsleya ellipsoidea*	LiHT0515	JF976550	JN044830	JF941852	JF953961
Eudicotyledons	Cucurbitaceae	*Hemsleya*	*Hemsleya ellipsoidea*	LiHT0514	JF976551	JN044831	JF941853	JF953962
Eudicotyledons	Cucurbitaceae	*Hemsleya*	*Hemsleya ellipsoidea*	LiHT1060	JF976547	JN044827	JF941849	JF953958
Eudicotyledons	Cucurbitaceae	*Hemsleya*	*Hemsleya dipterygia*	LiHT0564	JF976544	JN044824	JF941846	JF953955
Eudicotyledons	Cucurbitaceae	*Hemsleya*	*Hemsleya dipterygia*	LiHT0562	JF976546	JN044826	JF941848	JF953957
Eudicotyledons	Cucurbitaceae	*Hemsleya*	*Hemsleya dipterygia*	LiHT0563	JF976545	JN044825	JF941847	JF953956
Eudicotyledons	Cucurbitaceae	*Hemsleya*	*Hemsleya chinensis*	LiHT0518	JF976543	JN044823	JF941845	JF953954
Eudicotyledons	Cucurbitaceae	*Hemsleya*	*Hemsleya chinensis*	LiHT0522	JF976539	JN044819	JF941841	JF953950
Eudicotyledons	Cucurbitaceae	*Hemsleya*	*Hemsleya chinensis*	LiHT0523	JF976538	JN044818	JF941840	JF953949
Eudicotyledons	Cucurbitaceae	*Hemsleya*	*Hemsleya chinensis*	LiHT0520	JF976541	JN044821	JF941843	JF953952
Eudicotyledons	Cucurbitaceae	*Hemsleya*	*Hemsleya chinensis*	LiHT0521	JF976540	JN044820	JF941842	JF953951
Eudicotyledons	Cucurbitaceae	*Hemsleya*	*Hemsleya chinensis*	LiHT0524	JF976537	JN044817	JF941839	JF953948
Eudicotyledons	Cucurbitaceae	*Hemsleya*	*Hemsleya chinensis*	LiHT0525	JF976536	JN044816	JF941838	JF953947
Eudicotyledons	Cucurbitaceae	*Hemsleya*	*Hemsleya chinensis*	LiHT0519	JF976542	JN044822	JF941844	JF953953
Eudicotyledons	Cucurbitaceae	*Hemsleya*	*Hemsleya chengyihana*	LiHT10144	JF976531	JN044811	JF941833	JF953942
Eudicotyledons	Cucurbitaceae	*Hemsleya*	*Hemsleya chengyihana*	LiHT0555	JF976534	JN044814	JF941836	JF953945
Eudicotyledons	Cucurbitaceae	*Hemsleya*	*Hemsleya chengyihana*	LiHT0554	JF976535	JN044815	JF941837	JF953946
Eudicotyledons	Cucurbitaceae	*Hemsleya*	*Hemsleya chengyihana*	LiHT10140	JF976533	JN044813	JF941835	JF953944
Eudicotyledons	Cucurbitaceae	*Hemsleya*	*Hemsleya chengyihana*	LiHT10141	JF976532	JN044812	JF941834	JF953943
Eudicotyledons	Cucurbitaceae	*Hemsleya*	*Hemsleya carnosiflora*	LiHT0581-1	JF976530	JN044810	JF941832	JF953941
Eudicotyledons	Cucurbitaceae	*Hemsleya*	*Hemsleya carnosiflora*	LiHT0581-2	JF976529	JN044809	JF941831	JF953940
Eudicotyledons	Cucurbitaceae	*Hemsleya*	*Hemsleya carnosiflora*	LiHT0584	JF976528	JN044808	JF941830	JF953939
Eudicotyledons	Cucurbitaceae	*Hemsleya*	*Hemsleya carnosiflora*	LiHT0585	JF976527	JN044807	JF941829	JF953938
Eudicotyledons	Cucurbitaceae	*Hemsleya*	*Hemsleya amabilis*	LiHT10190	JF976521	JN044801	JF941823	JF953932
Eudicotyledons	Cucurbitaceae	*Hemsleya*	*Hemsleya amabilis*	LiHT10197	JF976519	JN044799	JF941821	JF953930
Eudicotyledons	Cucurbitaceae	*Hemsleya*	*Hemsleya amabilis*	LiHT10196	JF976520	JN044800	JF941822	JF953931
Eudicotyledons	Cucurbitaceae	*Hemsleya*	*Hemsleya amabilis*	LiHT10201-2	JF976516	JN044796	JF941818	JF953927
Eudicotyledons	Cucurbitaceae	*Hemsleya*	*Hemsleya amabilis*	LiHT10189	JF976522	JN044802	JF941824	JF953933
Eudicotyledons	Cucurbitaceae	*Hemsleya*	*Hemsleya amabilis*	LiHT0586	JF976523	JN044803	JF941825	JF953934
Eudicotyledons	Cucurbitaceae	*Hemsleya*	*Hemsleya amabilis*	LiHT0559	JF976526	JN044806	JF941828	JF953937
Eudicotyledons	Cucurbitaceae	*Hemsleya*	*Hemsleya amabilis*	LiHT10199-1	JF976518	JN044798	JF941820	JF953929
Eudicotyledons	Cucurbitaceae	*Hemsleya*	*Hemsleya amabilis*	LiHT10199-2	JF976517	JN044797	JF941819	JF953928
Eudicotyledons	Cucurbitaceae	*Hemsleya*	*Hemsleya amabilis*	LiHT0561	JF976524	JN044804	JF941826	JF953935

Eudicotyledons	Cucurbitaceae	*Hemsleya*	*Hemsleya amabilis*	LiHT0560	JF976525	JN044805	JF941827	JF953936
Eudicotyledons	Cucurbitaceae	*Hemsleya*	*Hemsleya amabilis*	LiHT10202-1	JF976515	JN044795	JF941817	JF953926
Eudicotyledons	Rubiaceae	*Hedyotis*	*Hedyotis yangchunensis*	WRJ1270-1	JF976514	JN044792	JF941814	JF953925
Eudicotyledons	Rubiaceae	*Hedyotis*	*Hedyotis yangchunensis*	WRJ1270-2	JF976513	JN044791	JF941813	JF953924
Eudicotyledons	Rubiaceae	*Hedyotis*	*Hedyotis uncinella*	WRJ1217	JF976509	JN044789	JF941807	JF953920
Eudicotyledons	Rubiaceae	*Hedyotis*	*Hedyotis uncinella*	WRJ0015	JF976510	JN044790	JF941808	JF953921
Eudicotyledons	Rubiaceae	*Hedyotis*	*Hedyotis shiuyingiae*	WRJ1255-2	JF976503	JN044783	JF941801	JF953914
Eudicotyledons	Rubiaceae	*Hedyotis*	*Hedyotis shiuyingiae*	WRJ1255-1	JF976504	JN044784	JF941802	JF953915
Eudicotyledons	Rubiaceae	*Hedyotis*	*Hedyotis shenzhenensis*	WRJ1262-1	JF976502	JN044782	JF941800	JF953913
Eudicotyledons	Rubiaceae	*Hedyotis*	*Hedyotis shenzhenensis*	WRJ1262-2	JF976501	JN044781	JF941799	JF953912
Eudicotyledons	Rubiaceae	*Hedyotis*	*Hedyotis pulcherrima*	WRJ1233-2	JF976499	JN044779	JF941797	JF953910
Eudicotyledons	Rubiaceae	*Hedyotis*	*Hedyotis pulcherrima*	WRJ1233-1	JF976500	JN044780	JF941798	JF953911
Eudicotyledons	Rubiaceae	*Hedyotis*	*Hedyotis effusa*	WRJ1268-2	JF976495	JN044775	JF941793	JF953906
Eudicotyledons	Rubiaceae	*Hedyotis*	*Hedyotis effusa*	WRJ1268-1	JF976496	JN044776	JF941794	JF953907
Eudicotyledons	Rubiaceae	*Hedyotis*	*Hedyotis costata*	WRJGBOWS1447	JF976493	JN044773	JF941791	JF953904
Eudicotyledons	Rubiaceae	*Hedyotis*	*Hedyotis costata*	WRJ1149	JF976494	JN044774	JF941792	JF953905
Eudicotyledons	Rubiaceae	*Hedyotis*	*Hedyotis caudatifolia*	WRJ1269	JF976485	JN044765	JF941783	JF953898
Eudicotyledons	Rubiaceae	*Hedyotis*	*Hedyotis caudatifolia*	WRJ1229	JF976486	JN044766	JF941784	JF953899
Eudicotyledons	Rubiaceae	*Hedyotis*	*Hedyotis cantoniensis*	WRJ1250	JF976484	JN044764	JF941782	JF953897
Eudicotyledons	Rubiaceae	*Hedyotis*	*Hedyotis cantoniensis*	WRJ1263	JF976483	JN044763	JF941781	JF953896
Eudicotyledons	Rubiaceae	*Hedyotis*	*Hedyotis auricularia*	WRJ0013	JF976477	JN044759	JF941775	JF953890
Eudicotyledons	Rubiaceae	*Hedyotis*	*Hedyotis auricularia*	WRJ1185	JF976476	JN044758	JF941774	JF953889
Eudicotyledons	Gentianaceae	*Gentiana*	*Gentiana triflora*	2005-2703A	GQ864019	GQ86403	JN162109	JN162095
Eudicotyledons	Gentianaceae	*Gentiana*	*Gentiana triflora*	2005-2703B	GQ864020	GQ86403	JN162110	JN162096
Eudicotyledons	Gentianaceae	*Gentiana*	*Gentiana scabra*	2005-2702A	GQ864015	GQ86402	JN162105	JN162093
Eudicotyledons	Gentianaceae	*Gentiana*	*Gentiana scabra*	2005-2702B	GQ864016	GQ86402	JN162106	JN162094
Eudicotyledons	Gentianaceae	*Gentiana*	*Gentiana rigescens*	2005-2704A	GQ864021	GQ86403	JN162111	JN162099
Eudicotyledons	Gentianaceae	*Gentiana*	*Gentiana rigescens*	2005-2704B	GQ864022	GQ86403	JN162112	JN162100
Eudicotyledons	Gentianaceae	*Gentiana*	*Gentiana manshurica*	2005-2701C	GQ864017	GQ86402	JN162107	JN162097
Eudicotyledons	Gentianaceae	*Gentiana*	*Gentiana manshurica*	2005-2701D	GQ864018	GQ86403	JN162108	JN162098
Eudicotyledons	Ericaceae	*Gaultheria*	*Gaultheria wardii*	LuLu-LL-07ZQ-3	JF976454	JN044726	JF941743	JF953865
Eudicotyledons	Ericaceae	*Gaultheria*	*Gaultheria wardii*	LuLu-LL-07ZQ-2	JF976455	JN044727	JF941744	JF953866
Eudicotyledons	Ericaceae	*Gaultheria*	*Gaultheria wardii*	LuLu-LL-07ZQ-1	JF976456	JN044728	JF941745	JF953867
Eudicotyledons	Ericaceae	*Gaultheria*	*Gaultheria wardii*	LuLu-LL-07ZQ-4	JF976453	JN044725	JF941742	JF953864
Eudicotyledons	Ericaceae	*Gaultheria*	*Gaultheria wardii*	LuLu-06-0067-4	JF976461	JN044733	JF941750	JF953872
Eudicotyledons	Ericaceae	*Gaultheria*	*Gaultheria wardii*	LuLu-06-0067-5	JF976460	JN044732	JF941749	JF953871
Eudicotyledons	Ericaceae	*Gaultheria*	*Gaultheria wardii*	LuLu-06-0067-3	JF976452	JN044724	JF941741	JF953863
Eudicotyledons	Ericaceae	*Gaultheria*	*Gaultheria wardii*	LuLu-LL-07301-3	JF976459	JN044731	JF941748	JF953870
Eudicotyledons	Ericaceae	*Gaultheria*	*Gaultheria wardii*	LuLu-LL-07301-4	JF976458	JN044730	JF941747	JF953869
Eudicotyledons	Ericaceae	*Gaultheria*	*Gaultheria wardii*	LuLu-LL-07301-5	JF976457	JN044729	JF941746	JF953868

Eudicotyledons	Ericaceae	*Gaultheria*	*Gaultheria trigonoclada*	LuLu-LL-07216-4	JF976450	JN044720	JF941737	JF953861
Eudicotyledons	Ericaceae	*Gaultheria*	*Gaultheria trigonoclada*	LuLu-LL-07216-5	JF976449	JN044719	JF941736	JF953860
Eudicotyledons	Ericaceae	*Gaultheria*	*Gaultheria trigonoclada*	LuLu-LL-07216-3	JF976451	JN044721	JF941738	JF953862
Eudicotyledons	Ericaceae	*Gaultheria*	*Gaultheria trichophylla*	LuLu-LL-07155-3	JF976447	JN044716	JF941733	JF953858
Eudicotyledons	Ericaceae	*Gaultheria*	*Gaultheria trichophylla*	LuLu-06-0019-2	JF976448	JN044717	JF941734	JF953859
Eudicotyledons	Ericaceae	*Gaultheria*	*Gaultheria tetramera*	LuLu-Liuj-09490-1	JF976444	JN044707	JF941724	JF953855
Eudicotyledons	Ericaceae	*Gaultheria*	*Gaultheria tetramera*	LuLu-06-0070-2	JF976446	JN044709	JF941726	JF953857
Eudicotyledons	Ericaceae	*Gaultheria*	*Gaultheria tetramera*	LuLu-06-0070-3	JF976445	JN044708	JF941725	JF953856
Eudicotyledons	Ericaceae	*Gaultheria*	*Gaultheria suborbicularis*	LuLu-LL-07307-3	JF976441	JN044703	JF941720	JF953852
Eudicotyledons	Ericaceae	*Gaultheria*	*Gaultheria suborbicularis*	LuLu-LL-07307-2	JF976442	JN044704	JF941721	JF953853
Eudicotyledons	Ericaceae	*Gaultheria*	*Gaultheria suborbicularis*	LuLu-LL-07307-1	JF976443	JN044705	JF941722	JF953854
Eudicotyledons	Ericaceae	*Gaultheria*	*Gaultheria suborbicularis*	LuLu-LL-07307-4	JF976440	JN044702	JF941719	JF953851
Eudicotyledons	Ericaceae	*Gaultheria*	*Gaultheria straminea*	LuLu-LL-07306-4	JF976438	JN044700	JF941717	JF953849
Eudicotyledons	Ericaceae	*Gaultheria*	*Gaultheria straminea*	LuLu-LL-07306-5	JF976437	JN044699	JF941716	JF953848
Eudicotyledons	Ericaceae	*Gaultheria*	*Gaultheria straminea*	LuLu-LL-07306-3	JF976439	JN044701	JF941718	JF953850
Eudicotyledons	Ericaceae	*Gaultheria*	*Gaultheria sinensis*	LuLu-06-0040-2	JF976435	JN044696	JF941713	JF953846
Eudicotyledons	Ericaceae	*Gaultheria*	*Gaultheria sinensis*	LuLu-06-0021-3	JF976436	JN044697	JF941714	JF953847
Eudicotyledons	Ericaceae	*Gaultheria*	*Gaultheria sinensis*	LuLu-LL-07133-3	JF976434	JN044695	JF941712	JF953845
Eudicotyledons	Ericaceae	*Gaultheria*	*Gaultheria sinensis*	LuLu-LL06-15-2	JF976433	JN044694	JF941711	JF953844
Eudicotyledons	Ericaceae	*Gaultheria*	*Gaultheria semi-infera*	LuLu-06-0017-2	JF976432	JN044689	JF941706	JF953843
Eudicotyledons	Ericaceae	*Gaultheria*	*Gaultheria semi-infera*	LuLu-LL-07312-2	JF976428	JN044685	JF941702	JF953839
Eudicotyledons	Ericaceae	*Gaultheria*	*Gaultheria semi-infera*	LuLu-LL-06QQ-2	JF976429	JN044686	JF941703	JF953840
Eudicotyledons	Ericaceae	*Gaultheria*	*Gaultheria semi-infera*	LuLu-LL-06QQ-1	JF976430	JN044687	JF941704	JF953841
Eudicotyledons	Ericaceae	*Gaultheria*	*Gaultheria semi-infera*	LuLu-06-103-2	JF976431	JN044688	JF941705	JF953842
Eudicotyledons	Ericaceae	*Gaultheria*	*Gaultheria pyrolifolia*	LuLu-LL-07117-3	JF976427	JN044681	JF941698	JF953838
Eudicotyledons	Ericaceae	*Gaultheria*	*Gaultheria pyrolifolia*	LuLu-LL-07117-4	JF976426	JN044680	JF941697	JF953837
Eudicotyledons	Ericaceae	*Gaultheria*	*Gaultheria pyrolifolia*	LuLu-LL-07117-5	JF976425	JN044679	JF941696	JF953836
Eudicotyledons	Ericaceae	*Gaultheria*	*Gaultheria pseudonotabilis*	LuLu-06-0045-1	JF976424	JN044677	JF941694	JF953835
Eudicotyledons	Ericaceae	*Gaultheria*	*Gaultheria pseudonotabilis*	LuLu-06-0045-2	JF976423	JN044676	JF941693	JF953834
Eudicotyledons	Ericaceae	*Gaultheria*	*Gaultheria pseudonotabilis*	LuLu-06-0045-4	JF976422	JN044675	JF941692	JF953833
Eudicotyledons	Ericaceae	*Gaultheria*	*Gaultheria pseudonotabilis*	LuLu-06-0045-5	JF976421	JN044674	JF941691	JF953832
Eudicotyledons	Ericaceae	*Gaultheria*	*Gaultheria praticola*	LuLu-LL-07140-3	JF976415	JN044668	JF941685	JF953826
Eudicotyledons	Ericaceae	*Gaultheria*	*Gaultheria praticola*	LuLu-LL-07140-1	JF976416	JN044669	JF941686	JF953827
Eudicotyledons	Ericaceae	*Gaultheria*	*Gaultheria praticola*	LuLu-LL-07140-4	JF976414	JN044667	JF941684	JF953825
Eudicotyledons	Ericaceae	*Gaultheria*	*Gaultheria praticola*	LuLu-06-0056-1	JF976420	JN044673	JF941690	JF953831
Eudicotyledons	Ericaceae	*Gaultheria*	*Gaultheria praticola*	LuLu-06-0056-2	JF976419	JN044672	JF941689	JF953830
Eudicotyledons	Ericaceae	*Gaultheria*	*Gaultheria praticola*	LuLu-06-0056-4	JF976418	JN044671	JF941688	JF953829
Eudicotyledons	Ericaceae	*Gaultheria*	*Gaultheria praticola*	LuLu-06-0056-5	JF976417	JN044670	JF941687	JF953828
Eudicotyledons	Ericaceae	*Gaultheria*	*Gaultheria praticola*	LuLu-LL-07140-5	JF976413	JN044666	JF941683	JF953824
Eudicotyledons	Ericaceae	*Gaultheria*	*Gaultheria nummularioides*	LuLu-LL-07304-3	JF976410	JN044663	JF941680	JF953821

Eudicotyledons	Ericaceae	*Gaultheria*	*Gaultheria nummularioides*	LuLu-07-010-3	JF976412	JN044665	JF941682	JF953823
Eudicotyledons	Ericaceae	*Gaultheria*	*Gaultheria nummularioides*	LuLu-GLGS20182-1	JF976411	JN044664	JF941681	JF953822
Eudicotyledons	Ericaceae	*Gaultheria*	*Gaultheria*	LuLu-LL06-01-2	JF976406	JN044652	JF941669	JF953817
Eudicotyledons	Ericaceae	*Gaultheria*	*Gaultheria*	LuLu-LL06-01-3	JF976405	JN044651	JF941668	JF953816
Eudicotyledons	Ericaceae	*Gaultheria*	*Gaultheria leucocarpa*	LuLu-Liu1001-1	JF976398	JN044643	JF941660	JF953810
Eudicotyledons	Ericaceae	*Gaultheria*	*Gaultheria leucocarpa*	LuLu-LL06-10-2	JF976393	JN044638	JF941655	JF953805
Eudicotyledons	Ericaceae	*Gaultheria*	*Gaultheria leucocarpa*	LuLu-Liu1001-2	JF976397	JN044642	JF941659	JF953809
Eudicotyledons	Ericaceae	*Gaultheria*	*Gaultheria leucocarpa*	LuLu-LL06-09-1	JF976396	JN044641	JF941658	JF953808
Eudicotyledons	Ericaceae	*Gaultheria*	*Gaultheria leucocarpa*	LuLu-LL06-09-2	JF976395	JN044640	JF941657	JF953807
Eudicotyledons	Ericaceae	*Gaultheria*	*Gaultheria leucocarpa*	LuLu-LTX001-2	JF976391	JN044636	JF941653	JF953804
Eudicotyledons	Ericaceae	*Gaultheria*	*Gaultheria leucocarpa*	LuLu-LU2001-2	JF976385	JN044630	JF941647	JF953798
Eudicotyledons	Ericaceae	*Gaultheria*	*Gaultheria leucocarpa*	LuLu-LU002-1	JF976390	JN044635	JF941652	JF953803
Eudicotyledons	Ericaceae	*Gaultheria*	*Gaultheria leucocarpa*	LuLu-LU002-2	JF976389	JN044634	JF941651	JF953802
Eudicotyledons	Ericaceae	*Gaultheria*	*Gaultheria leucocarpa*	LuLu-03-1607-1	JF976404	JN044649	JF941666	JF953815
Eudicotyledons	Ericaceae	*Gaultheria*	*Gaultheria leucocarpa*	LuLu-03-1607-2	JF976403	JN044648	JF941665	JF953814
Eudicotyledons	Ericaceae	*Gaultheria*	*Gaultheria leucocarpa*	LuLu-LU2001-1	JF976386	JN044631	JF941648	JF953799
Eudicotyledons	Ericaceae	*Gaultheria*	*Gaultheria leucocarpa*	LuLu-HE001-2	JF976399	JN044644	JF941661	JF953811
Eudicotyledons	Ericaceae	*Gaultheria*	*Gaultheria leucocarpa*	LuLu-LU1001-1	JF976388	JN044633	JF941650	JF953801
Eudicotyledons	Ericaceae	*Gaultheria*	*Gaultheria leucocarpa*	LuLu-LU1001-2	JF976387	JN044632	JF941649	JF953800
Eudicotyledons	Ericaceae	*Gaultheria*	*Gaultheria leucocarpa*	LuLu-LL06-10-1	JF976394	JN044639	JF941656	JF953806
Eudicotyledons	Ericaceae	*Gaultheria*	*Gaultheria leucocarpa*	LuLu-07-011-1	JF976402	JN044647	JF941664	JF953813
Eudicotyledons	Ericaceae	*Gaultheria*	*Gaultheria leucocarpa*	LuLu-07-011-2	JF976401	JN044646	JF941663	JF953812
Eudicotyledons	Ericaceae	*Gaultheria*	*Gaultheria jingdongensis*	LuLu-06-19A-3	JF976383	JN044628	JF941645	JF953797
Eudicotyledons	Ericaceae	*Gaultheria*	*Gaultheria jingdongensis*	LuLu-06-19A-4	JF976382	JN044627	JF941644	JF953796
Eudicotyledons	Ericaceae	*Gaultheria*	*Gaultheria hypochlora*	LuLu-LL-06-0012-3	JF976381	JN044625	JF941642	JF953795
Eudicotyledons	Ericaceae	*Gaultheria*	*Gaultheria hypochlora*	LuLu-LL-07135-3	JF976380	JN044624	JF941641	JF953794
Eudicotyledons	Ericaceae	*Gaultheria*	*Gaultheria hookeri*	LuLu-03-1500-2	JF976379	JN044619	JF941636	JF953793
Eudicotyledons	Ericaceae	*Gaultheria*	*Gaultheria hookeri*	LuLu-LL-07089-3	JF976376	JN044616	JF941633	JF953790
Eudicotyledons	Ericaceae	*Gaultheria*	*Gaultheria hookeri*	LuLu-LL-06DYK-1	JF976378	JN044618	JF941635	JF953792
Eudicotyledons	Ericaceae	*Gaultheria*	*Gaultheria hookeri*	LuLu-LL-06DYK-2	JF976377	JN044617	JF941634	JF953791
Eudicotyledons	Ericaceae	*Gaultheria*	*Gaultheria heteromera*	LuLu-LL-07316A-3	JF976374	JN044611	JF941628	JF953788
Eudicotyledons	Ericaceae	*Gaultheria*	*Gaultheria heteromera*	LuLu-LL-07316A-2	JF976375	JN044612	JF941629	JF953789
Eudicotyledons	Ericaceae	*Gaultheria*	*Gaultheria heteromera*	LuLu-LL-07316A-4	JF976373	JN044610	JF941627	JF953787
Eudicotyledons	Ericaceae	*Gaultheria*	*Gaultheria griffithiana*	LuLu-06-100-1	JF976369	JN044605	JF941622	JF953783
Eudicotyledons	Ericaceae	*Gaultheria*	*Gaultheria griffithiana*	LuLu-LL-07169-3	JF976364	JN044600	JF941617	JF953778
Eudicotyledons	Ericaceae	*Gaultheria*	*Gaultheria griffithiana*	LuLu-06-0026-2	JF976372	JN044608	JF941625	JF953786
Eudicotyledons	Ericaceae	*Gaultheria*	*Gaultheria griffithiana*	LuLu-06-008-2	JF976370	JN044606	JF941623	JF953784
Eudicotyledons	Ericaceae	*Gaultheria*	*Gaultheria griffithiana*	LuLu-06-008-1	JF976371	JN044607	JF941624	JF953785
Eudicotyledons	Ericaceae	*Gaultheria*	*Gaultheria griffithiana*	LuLu-06-100-2	JF976368	JN044604	JF941621	JF953782
Eudicotyledons	Ericaceae	*Gaultheria*	*Gaultheria griffithiana*	LuLu-LL-06DSF-2	JF976365	JN044601	JF941618	JF953779

Eudicotyledons	Ericaceae	*Gaultheria*	*Gaultheria griffithiana*	LuLu-Liuj-09511-1	JF976367	JN044603	JF941620	JF953781
Eudicotyledons	Ericaceae	*Gaultheria*	*Gaultheria griffithiana*	LuLu-LL-06DSF-1	JF976366	JN044602	JF941619	JF953780
Eudicotyledons	Ericaceae	*Gaultheria*	*Gaultheria fragrantissima*	LuLu-LJJMC-1	JF976357	JN044590	JF941607	JF953771
Eudicotyledons	Ericaceae	*Gaultheria*	*Gaultheria fragrantissima*	LuLu-LJJMC-2	JF976356	JN044589	JF941606	JF953770
Eudicotyledons	Ericaceae	*Gaultheria*	*Gaultheria fragrantissima*	LuLu-LL-07305-3	JF976355	JN044588	JF941605	JF953769
Eudicotyledons	Ericaceae	*Gaultheria*	*Gaultheria fragrantissima*	LuLu-LU001-2	JF976352	JN044585	JF941602	JF953766
Eudicotyledons	Ericaceae	*Gaultheria*	*Gaultheria fragrantissima*	LuLu-LU001-1	JF976353	JN044586	JF941603	JF953767
Eudicotyledons	Ericaceae	*Gaultheria*	*Gaultheria fragrantissima*	LuLu-06-0027-2	JF976361	JN044594	JF941611	JF953775
Eudicotyledons	Ericaceae	*Gaultheria*	*Gaultheria fragrantissima*	LuLu-06-002-1	JF976363	JN044596	JF941613	JF953777
Eudicotyledons	Ericaceae	*Gaultheria*	*Gaultheria fragrantissima*	LuLu-06-002-2	JF976362	JN044595	JF941612	JF953776
Eudicotyledons	Ericaceae	*Gaultheria*	*Gaultheria fragrantissima*	LuLu-LL06-07-2	JF976354	JN044587	JF941604	JF953768
Eudicotyledons	Ericaceae	*Gaultheria*	*Gaultheria fragrantissima*	LuLu-07-008-2	JF976358	JN044591	JF941608	JF953772
Eudicotyledons	Ericaceae	*Gaultheria*	*Gaultheria fragrantissima*	LuLu-07-007-3	JF976360	JN044593	JF941610	JF953774
Eudicotyledons	Ericaceae	*Gaultheria*	*Gaultheria dumicola*	LuLu-07-009-3	JF976349	JN044575	JF941592	JF953763
Eudicotyledons	Ericaceae	*Gaultheria*	*Gaultheria dumicola*	LuLu-06-66-3	JF976350	JN044576	JF941593	JF953764
Eudicotyledons	Ericaceae	*Gaultheria*	*Gaultheria dumicola*	LuLu-06-101-3	JF976351	JN044577	JF941594	JF953765
Eudicotyledons	Ericaceae	*Gaultheria*	*Gaultheria dolichopoda*	LuLu-06-0005-4	JF976346	JN044568	JF941585	JF953760
Eudicotyledons	Ericaceae	*Gaultheria*	*Gaultheria dolichopoda*	LuLu-06-0005-2	JF976348	JN044570	JF941587	JF953762
Eudicotyledons	Ericaceae	*Gaultheria*	*Gaultheria dolichopoda*	LuLu-06-0005-3	JF976347	JN044569	JF941586	JF953761
Eudicotyledons	Ericaceae	*Gaultheria*	*Gaultheria codonantha*	LuLu-LL-07303-5	JF976343	JN044562	JF941579	JF953757
Eudicotyledons	Ericaceae	*Gaultheria*	*Gaultheria codonantha*	LuLu-LL-07303-4	JF976344	JN044563	JF941580	JF953758
Eudicotyledons	Ericaceae	*Gaultheria*	*Gaultheria codonantha*	LuLu-LL-07303-3	JF976345	JN044564	JF941581	JF953759
Eudicotyledons	Ericaceae	*Gaultheria*	*Gaultheria cardiosepala*	LuLu-Zhang001-1	JF976340	JN044558	JF941575	JF953754
Eudicotyledons	Ericaceae	*Gaultheria*	*Gaultheria cardiosepala*	LuLu-05-0016-2	JF976342	JN044560	JF941577	JF953756
Eudicotyledons	Ericaceae	*Gaultheria*	*Gaultheria cardiosepala*	LuLu-06-0022-3	JF976341	JN044559	JF941576	JF953755
Eudicotyledons	Ericaceae	*Gaultheria*	*Gaultheria brevistipes*	LuLu-LL-07300-4	JF976338	JN044554	JF941571	JF953752
Eudicotyledons	Ericaceae	*Gaultheria*	*Gaultheria brevistipes*	LuLu-LL-07300-5	JF976337	JN044553	JF941570	JF953751
Eudicotyledons	Ericaceae	*Gaultheria*	*Gaultheria brevistipes*	LuLu-LL-07300-3	JF976339	JN044555	JF941572	JF953753
Eudicotyledons	Moraceae	*Ficus*	*Ficus variolosa*	LHQ2010055	JF976331	JN044545	JF941561	JF953748
Eudicotyledons	Moraceae	*Ficus*	*Ficus variolosa*	LHQ2009336	JF976333	JN044547	JF941563	JF953749
Eudicotyledons	Moraceae	*Ficus*	*Ficus tinctoria*	LHQ2009260	JF976330	JN044544	JF941560	JF953747
Eudicotyledons	Moraceae	*Ficus*	*Ficus tinctoria*	LHQ2009278	JF976329	JN044543	JF941559	JF953746
Eudicotyledons	Moraceae	*Ficus*	*Ficus tinctoria*	LHQ2009443	JF976328	JN044542	JF941558	JF953745
Eudicotyledons	Moraceae	*Ficus*	*Ficus semicordata*	LHQ2009346	JF976327	JN044539	JF941555	JF953744
Eudicotyledons	Moraceae	*Ficus*	*Ficus semicordata*	LHQ2009454	JF976325	JN044536	JF941552	JF953742
Eudicotyledons	Moraceae	*Ficus*	*Ficus cyrtophylla*	LHQ2009397	JF976318	JN044514	JF941525	JF953729
Eudicotyledons	Moraceae	*Ficus*	*Ficus cyrtophylla*	LHQ2009303	JF976319	JN044515	JF941526	JF953730
Eudicotyledons	Brassicaceae	*Draba*	*Draba nemorosa*	Z762	JF976275	JN044450	JF941445	JF953669
Eudicotyledons	Brassicaceae	*Draba*	*Draba nemorosa*	Z763	JF976274	JN044449	JF941444	JF953668
Eudicotyledons	Brassicaceae	*Draba*	*Draba lanceolata*	A191	JF976272	JN044447	JF941442	JF953666

Eudicotyledons	Brassicaceae	*Draba*	*Draba lanceolata*	A190	JF976273	JN044448	JF941443	JF953667
Eudicotyledons	Brassicaceae	*Draba*	*Draba lanceolata*	A192	JF976271	JN044446	JF941441	JF953665
Eudicotyledons	Brassicaceae	*Draba*	*Draba lanceolata*	A194	JF976269	JN044445	JF941439	JF953663
Eudicotyledons	Solanaceae	*Datura*	*Datura stramonium*	PS1145MT03	JN244320	JN244370	JN244353	JN244334
Eudicotyledons	Solanaceae	*Datura*	*Datura stramonium*	PS1145MT01	JN244318	JN244368	JN244351	JN244349
Eudicotyledons	Solanaceae	*Datura*	*Datura stramonium*	PS1145MT02	JN244319	JN244369	JN244352	JN244350
Eudicotyledons	Solanaceae	*Datura*	*Datura stramonium*	PS1145MT05	JN244322	JN244372	JN244355	JN244336
Eudicotyledons	Solanaceae	*Datura*	*Datura stramonium*	PS1145MT04	JN244321	JN244371	JN244354	JN244335
Eudicotyledons	Solanaceae	*Datura*	*Datura stramonium*	PS1145MT06	JN244323	JN244373	JN244356	JN244337
Eudicotyledons	Solanaceae	*Datura*	*Datura stramonium*	PS1145MT07	JN244324	JN244374	JN244357	JN244338
Eudicotyledons	Solanaceae	*Datura*	*Datura metel*	PS1152MT01	GQ434671	GQ43528	JN244364	GQ43422
Eudicotyledons	Solanaceae	*Datura*	*Datura metel*	PS1152MT03	JN244331	JN244381	JN244365	JN244346
Eudicotyledons	Solanaceae	*Datura*	*Datura metel*	PS1152MT05	JN244333	JN244383	JN244367	JN244348
Eudicotyledons	Solanaceae	*Datura*	*Datura metel*	PS1152MT04	JN244332	JN244382	JN244366	JN244347
Eudicotyledons	Solanaceae	*Datura*	*Datura inoxia*	PS1146MT02	JN244326	JN244376	JN244359	JN244340
Eudicotyledons	Solanaceae	*Datura*	*Datura inoxia*	PS1146MT03	JN244327	JN244377	JN244360	JN244341
Eudicotyledons	Solanaceae	*Datura*	*Datura inoxia*	PS1146MT01	JN244325	JN244375	JN244358	JN244339
Eudicotyledons	Campanulaceae	*Cyananthus*	*Cyananthus macrocalyx*	SunH-07ZX-2426	JF976208	JN044360	JF941348	JF953626
Eudicotyledons	Campanulaceae	*Cyananthus*	*Cyananthus macrocalyx*	SunH-SC0058	JF976207	JN044359	JF941347	JF953625
Eudicotyledons	Campanulaceae	*Cyananthus*	*Cyananthus macrocalyx*	SunH-SC11162	JF976206	JN044358	JF941346	JF953624
Eudicotyledons	Campanulaceae	*Cyananthus*	*Cyananthus macrocalyx*	SunH-YN11237	JF976205	JN044357	JF941345	JF953623
Eudicotyledons	Campanulaceae	*Cyananthus*	*Cyananthus lichiangensis*	SunH-SC11104	JF976200	JN044353	JF941340	JF953618
Eudicotyledons	Campanulaceae	*Cyananthus*	*Cyananthus lichiangensis*	SunH-YN11216	JF976198	JN044351	JF941338	JF953616
Eudicotyledons	Campanulaceae	*Cyananthus*	*Cyananthus lichiangensis*	SunH-SC0068	JF976201	JN044354	JF941341	JF953619
Eudicotyledons	Campanulaceae	*Cyananthus*	*Cyananthus lichiangensis*	SunH-SC11193	JF976199	JN044352	JF941339	JF953617
Eudicotyledons	Campanulaceae	*Cyananthus*	*Cyananthus inflatus*	NieZL-817	JF976197	JN044350	JF941337	JF953615
Eudicotyledons	Campanulaceae	*Cyananthus*	*Cyananthus inflatus*	ZhouZ-001	JF976194	JN044346	JF941333	JF953611
Eudicotyledons	Campanulaceae	*Cyananthus*	*Cyananthus inflatus*	NieZL-955	JF976196	JN044349	JF941336	JF953614
Eudicotyledons	Campanulaceae	*Cyananthus*	*Cyananthus inflatus*	SunH-YN11236	JF976195	JN044347	JF941334	JF953612
Eudicotyledons	Campanulaceae	*Cyananthus*	*Cyananthus incanus*	NieZL-757	JF976192	JN044344	JF941331	JF953609
Eudicotyledons	Campanulaceae	*Cyananthus*	*Cyananthus incanus*	NieZL-858	JF976191	JN044343	JF941330	JF953608
Eudicotyledons	Campanulaceae	*Cyananthus*	*Cyananthus incanus*	NieZL-1045	JF976193	JN044345	JF941332	JF953610
Eudicotyledons	Campanulaceae	*Cyananthus*	*Cyananthus incanus*	NieZL-913	JF976190	JN044342	JF941329	JF953607
Eudicotyledons	Campanulaceae	*Cyananthus*	*Cyananthus incanus*	SunH-SC11225	JF976189	JN044341	JF941328	JF953606
Eudicotyledons	Campanulaceae	*Cyananthus*	*Cyananthus hookeri*	NieZL-1028	JF976188	JN044340	JF941327	JF953605
Eudicotyledons	Campanulaceae	*Cyananthus*	*Cyananthus hookeri*	SunH-07ZX-2383	JF976186	JN044339	JF941325	JF953603
Eudicotyledons	Campanulaceae	*Cyananthus*	*Cyananthus hookeri*	SunH-SC11194	JF976184	JN044337	JF941323	JF953601
Eudicotyledons	Campanulaceae	*Cyananthus*	*Cyananthus hookeri*	SunH-SC11160	JF976185	JN044338	JF941324	JF953602
Eudicotyledons	Campanulaceae	*Cyananthus*	*Cyananthus dolichosceles*	SunH-SC11198	JF976181	JN044332	JF941318	JF953597
Eudicotyledons	Campanulaceae	*Cyananthus*	*Cyananthus dolichosceles*	SunH-SC11215	JF976180	JN044331	JF941317	JF953596

Eudicotyledons	Campanulaceae	*Cyananthus*	*Cyananthus dolichosceles*	SunH-SC11103	JF976183	JN044334	JF941320	JF953598
Eudicotyledons	Meliaceae	*Cipadessa*	*Cipadessa cinerascens*	W18	JF976159	JN044305	JF941283	JF953560
Eudicotyledons	Meliaceae	*Cipadessa*	*Cipadessa cinerascens*	W19	JF976158	JN044304	JF941282	JF953559
Eudicotyledons	Meliaceae	*Cipadessa*	*Cipadessa baccifera*	W63	JF976155	JN044301	JF941279	JF953556
Eudicotyledons	Meliaceae	*Cipadessa*	*Cipadessa baccifera*	C464	JF976157	JN044303	JF941281	JF953558
Eudicotyledons	Meliaceae	*Cipadessa*	*Cipadessa baccifera*	W62	JF976156	JN044302	JF941280	JF953557
Eudicotyledons	Meliaceae	*Cipadessa*	*Cipadessa baccifera*	W64	JF976154	JN044300	JF941278	JF953555
Eudicotyledons	Meliaceae	*Cipadessa*	*Cipadessa baccifera*	W65	JF976153	JN044299	JF941277	JF953554
Eudicotyledons	Amaranthaceae	*Chenopodium*	*Chenopodium glaucum*	Z440	JF976149	JN044295	JF941273	JF953550
Eudicotyledons	Amaranthaceae	*Chenopodium*	*Chenopodium glaucum*	Z439	JF976150	JN044296	JF941274	JF953551
Eudicotyledons	Amaranthaceae	*Chenopodium*	*Chenopodium glaucum*	Z438	JF976151	JN044297	JF941275	JF953552
Eudicotyledons	Amaranthaceae	*Chenopodium*	*Chenopodium glaucum*	D1163	JF976152	JN044298	JF941276	JF953553
Eudicotyledons	Amaranthaceae	*Chenopodium*	*Chenopodium foliosum*	D1587	JF976147	JN044293	JF941271	JF953548
Eudicotyledons	Amaranthaceae	*Chenopodium*	*Chenopodium foliosum*	D1560	JF976148	JN044294	JF941272	JF953549
Eudicotyledons	Amaranthaceae	*Chenopodium*	*Chenopodium album*	A105	JF976141	JN044287	JF941266	JF953542
Eudicotyledons	Amaranthaceae	*Chenopodium*	*Chenopodium album*	A99	JF976136	JN044281	JF941260	JF953536
Eudicotyledons	Amaranthaceae	*Chenopodium*	*Chenopodium album*	A98	JF976137	JN044282	JF941261	JF953537
Eudicotyledons	Amaranthaceae	*Chenopodium*	*Chenopodium album*	A96	JF976138	JN044284	JF941263	JF953539
Eudicotyledons	Amaranthaceae	*Chenopodium*	*Chenopodium album*	Z433	JF976131	JN044276	JF941255	JF953532
Eudicotyledons	Amaranthaceae	*Chenopodium*	*Chenopodium album*	Z432	JF976132	JN044277	JF941256	JF953533
Eudicotyledons	Amaranthaceae	*Chenopodium*	*Chenopodium album*	Z431	JF976133	JN044278	JF941257	JF953534
Eudicotyledons	Amaranthaceae	*Chenopodium*	*Chenopodium album*	Z437	JF976127	JN044272	JF941251	JF953528
Eudicotyledons	Amaranthaceae	*Chenopodium*	*Chenopodium album*	Z436	JF976128	JN044273	JF941252	JF953529
Eudicotyledons	Amaranthaceae	*Chenopodium*	*Chenopodium album*	Z435	JF976129	JN044274	JF941253	JF953530
Eudicotyledons	Amaranthaceae	*Chenopodium*	*Chenopodium album*	Z434	JF976130	JN044275	JF941254	JF953531
Eudicotyledons	Amaranthaceae	*Chenopodium*	*Chenopodium album*	A106	JF976140	JN044286	JF941265	JF953541
Eudicotyledons	Amaranthaceae	*Chenopodium*	*Chenopodium album*	A107	JF976139	JN044285	JF941264	JF953540
Eudicotyledons	Amaranthaceae	*Chenopodium*	*Chenopodium album*	A104	JF976142	JN044288	JF941267	JF953543
Eudicotyledons	Amaranthaceae	*Chenopodium*	*Chenopodium album*	A102	JF976144	JN044290	JF941269	JF953545
Eudicotyledons	Amaranthaceae	*Chenopodium*	*Chenopodium album*	A103	JF976143	JN044289	JF941268	JF953544
Eudicotyledons	Amaranthaceae	*Chenopodium*	*Chenopodium album*	A100	JF976146	JN044292	JF941270	JF953547
Eudicotyledons	Amaranthaceae	*Chenopodium*	*Chenopodium album*	D1162	JF976135	JN044280	JF941259	JF953535
Eudicotyledons	Ericaceae	*Cassiope*	*Cassiope selaginoides*	B015	JF976093	JN044185	JF941144	JF953438
Eudicotyledons	Ericaceae	*Cassiope*	*Cassiope selaginoides*	B012	JF976094	JN044186	JF941145	JF953439
Eudicotyledons	Ericaceae	*Cassiope*	*Cassiope selaginoides*	B025	JF976088	JN044181	JF941140	JF953433
Eudicotyledons	Ericaceae	*Cassiope*	*Cassiope selaginoides*	B021	JF976092	JN044184	JF941143	JF953437
Eudicotyledons	Ericaceae	*Cassiope*	*Cassiope selaginoides*	B022	JF976091	JN044183	JF941142	JF953436
Eudicotyledons	Ericaceae	*Cassiope*	*Cassiope selaginoides*	B023	JF976090	JN044182	JF941141	JF953435
Eudicotyledons	Ericaceae	*Cassiope*	*Cassiope pectinata*	B017	JF976086	JN044179	JF941138	JF953431
Eudicotyledons	Ericaceae	*Cassiope*	*Cassiope pectinata*	B016	JF976087	JN044180	JF941139	JF953432

Eudicotyledons	Ericaceae	*Cassiope*	*Cassiope fastigiata*	B011	JF976084	JN044177	JF941136	JF953430
Eudicotyledons	Ericaceae	*Cassiope*	*Cassiope fastigiata*	B013	JF976083	JN044176	JF941135	JF953429
Eudicotyledons	Ericaceae	*Cassiope*	*Cassiope dendrotricha*	B020	JF976081	JN044173	JF941132	JF953426
Eudicotyledons	Ericaceae	*Cassiope*	*Cassiope dendrotricha*	B019	JF976082	JN044174	JF941133	JF953427
Eudicotyledons	Brassicaceae	*Cardamine*	*Cardamine tangutorum*	A85	JF976079	JN044171	JF941130	JF953425
Eudicotyledons	Brassicaceae	*Cardamine*	*Cardamine tangutorum*	A86	JF976078	JN044170	JF941129	JF953424
Eudicotyledons	Brassicaceae	*Cardamine*	*Cardamine tangutorum*	A87	JF976077	JN044169	JF941128	JF953423
Eudicotyledons	Brassicaceae	*Cardamine*	*Cardamine macrophylla*	D322	JF976075	JN044167	JF941126	JF953421
Eudicotyledons	Brassicaceae	*Cardamine*	*Cardamine macrophylla*	D1706	JF976076	JN044168	JF941127	JF953422
Eudicotyledons	Brassicaceae	*Cardamine*	*Cardamine impatiens*	Z752	JF976071	JN044162	JF941121	JF953416
Eudicotyledons	Brassicaceae	*Cardamine*	*Cardamine impatiens*	Z751	JF976072	JN044163	JF941122	JF953417
Eudicotyledons	Brassicaceae	*Cardamine*	*Cardamine impatiens*	Z749	JF976073	JN044165	JF941124	JF953419
Eudicotyledons	Brassicaceae	*Cardamine*	*Cardamine impatiens*	Z748	JF976074	JN044166	JF941125	JF953420
Eudicotyledons	Polygonaceae	*Bistorta*	*Bistorta vivipara*	D1426	JF977874	JN046450	JF943527	JF955598
Eudicotyledons	Polygonaceae	*Bistorta*	*Bistorta vivipara*	D1548	JF977873	JN046449	JF943526	JF955597
Eudicotyledons	Polygonaceae	*Bistorta*	*Bistorta vivipara*	D1612	JF977872	JN046448	JF943525	JF955596
Eudicotyledons	Polygonaceae	*Bistorta*	*Bistorta macrophylla*	A462	JF977855	JN046429	JF943506	JF955587
Eudicotyledons	Polygonaceae	*Bistorta*	*Bistorta macrophylla*	A455	JF977861	JN046434	JF943511	JF955593
Eudicotyledons	Polygonaceae	*Bistorta*	*Bistorta macrophylla*	A461	JF977856	JN046430	JF943507	JF955588
Eudicotyledons	Polygonaceae	*Bistorta*	*Bistorta macrophylla*	A463	JF977854	JN046428	JF943505	JF955586
Eudicotyledons	Polygonaceae	*Bistorta*	*Bistorta macrophylla*	A458	JF977858	JN046432	JF943509	JF955590
Eudicotyledons	Polygonaceae	*Bistorta*	*Bistorta macrophylla*	A457	JF977859	JN046433	JF943510	JF955591
Eudicotyledons	Polygonaceae	*Bistorta*	*Bistorta macrophylla*	A459	JF977857	JN046431	JF943508	JF955589
Eudicotyledons	Begoniaceae	*Begonia*	*Begonia villifolia*	SYM-CKF-275	JF976059	JN044143	JF941106	JF953395
Eudicotyledons	Begoniaceae	*Begonia*	*Begonia villifolia*	SYM-GBOWS0813-sample3	JF976055	JN044139	JF941104	JF953394
Eudicotyledons	Begoniaceae	*Begonia*	*Begonia platycarpa*	SYM-B2005-016-sample1	JF976038	JN044119	JF941088	JF953378
Eudicotyledons	Begoniaceae	*Begonia*	*Begonia platycarpa*	SYM-B2005-016-sample3	JF976036	JN044117	JF941086	JF953376
Eudicotyledons	Begoniaceae	*Begonia*	*Begonia platycarpa*	SYM-B2005-016-sample2	JF976037	JN044118	JF941087	JF953377
Eudicotyledons	Begoniaceae	*Begonia*	*Begonia platycarpa*	SYM-B2005-029-sample3	JF976033	JN044115	JF941083	JF953373
Eudicotyledons	Begoniaceae	*Begonia*	*Begonia platycarpa*	SYM-B2005-029-sample1	JF976035	JN044116	JF941085	JF953375
Eudicotyledons	Begoniaceae	*Begonia*	*Begonia megalophyllaria*	SYM-D-33-sample4	JF976023	JN044100	JF941052	JF953355
Eudicotyledons	Begoniaceae	*Begonia*	*Begonia megalophyllaria*	SYM-82887	JF976027	JN044104	JF941056	JF953359
Eudicotyledons	Begoniaceae	*Begonia*	*Begonia megalophyllaria*	SYM-GBOWS341	JF976022	JN044099	JF941051	JF953354
Eudicotyledons	Begoniaceae	*Begonia*	*Begonia megalophyllaria*	SYM-D-33-sample1	JF976026	JN044103	JF941055	JF953358
Eudicotyledons	Begoniaceae	*Begonia*	*Begonia megalophyllaria*	SYM-D-33-sample2	JF976025	JN044102	JF941054	JF953357
Eudicotyledons	Begoniaceae	*Begonia*	*Begonia megalophyllaria*	SYM-D-33-sample3	JF976024	JN044101	JF941053	JF953356
Eudicotyledons	Begoniaceae	*Begonia*	*Begonia masoniana*	SYM-B2005-12-sample2	JF976020	JN044097	JF941048	JF953351
Eudicotyledons	Begoniaceae	*Begonia*	*Begonia masoniana*	SYM-B2005-53-sample1	JF976019	JN044096	JF941047	JF953350
Eudicotyledons	Begoniaceae	*Begonia*	*Begonia masoniana*	SYM-B2005-53-sample2	JF976021	JN044098	JF941050	JF953353
Eudicotyledons	Begoniaceae	*Begonia*	*Begonia longistyla*	SYM-40778-sample5	JF976016	JN044088	JF941032	JF953342

Eudicotyledons	Begoniaceae	*Begonia*	*Begonia longistyla*	SYM-40778-sample6	JF976015	JN044087	JF941031	JF953341
Eudicotyledons	Begoniaceae	*Begonia*	*Begonia longistyla*	SYM-40778-sample1	JF976018	JN044092	JF941036	JF953346
Eudicotyledons	Begoniaceae	*Begonia*	*Begonia longistyla*	SYM-40778-sample3	JF976017	JN044090	JF941034	JF953344
Eudicotyledons	Begoniaceae	*Begonia*	*Begonia longicarpa*	SYM-GBOWS1279-sample3	JF976011	JN044076	JF941022	JF953331
Eudicotyledons	Begoniaceae	*Begonia*	*Begonia longicarpa*	SYM-GBOWS1279-sample2	JF976012	JN044077	JF941023	JF953332
Eudicotyledons	Begoniaceae	*Begonia*	*Begonia longicarpa*	SYM-GBOWS1279-sample1	JF976013	JN044078	JF941024	JF953333
Eudicotyledons	Begoniaceae	*Begonia*	*Begonia hekouensis*	SYM-GBOWS1236-sample8	JF975997	JN044063	JF941009	JF953323
Eudicotyledons	Begoniaceae	*Begonia*	*Begonia hekouensis*	SYM-GBOWS1236-sample9	JF975996	JN044062	JF941008	JF953322
Eudicotyledons	Begoniaceae	*Begonia*	*Begonia hekouensis*	SYM-GBOWS1236-sample1	JF976000	JN044067	JF941013	JF953326
Eudicotyledons	Begoniaceae	*Begonia*	*Begonia hekouensis*	SYM-GBOWS1236-sample20	JF975998	JN044065	JF941011	JF953325
Eudicotyledons	Begoniaceae	*Begonia*	*Begonia daweishanensis*	SYM-D-34-sample3	JF975977	JN044036	JF940981	JF953302
Eudicotyledons	Begoniaceae	*Begonia*	*Begonia daweishanensis*	SYM-D-34-sample2	JF975978	JN044037	JF940982	JF953303
Eudicotyledons	Begoniaceae	*Begonia*	*Begonia daweishanensis*	SYM-D-34-sample1	JF975979	JN044038	JF940983	JF953304
Eudicotyledons	Begoniaceae	*Begonia*	*Begonia daweishanensis*	SYM-D-34-sample6	JF975974	JN044033	JF940979	JF953299
Eudicotyledons	Begoniaceae	*Begonia*	*Begonia daweishanensis*	SYM-D-34-sample4	JF975976	JN044035	JF940980	JF953301
Eudicotyledons	Begoniaceae	*Begonia*	*Begonia crystallina*	SYM-D-012-sample2	JF975966	JN044023	JF940966	JF953296
Eudicotyledons	Begoniaceae	*Begonia*	*Begonia crystallina*	SYM-D-012-sample1	JF975967	JN044024	JF940967	JF953297
Eudicotyledons	Begoniaceae	*Begonia*	*Begonia biflora*	SYM-32236-sample2	JF975963	JN044014	JF940954	JF953286
Eudicotyledons	Begoniaceae	*Begonia*	*Begonia biflora*	SYM-32236-sample3	JF975962	JN044013	JF940953	JF953285
Eudicotyledons	Begoniaceae	*Begonia*	*Begonia biflora*	SYM-32236-sample6	JF975960	JN044010	JF940951	JF953283
Eudicotyledons	Begoniaceae	*Begonia*	*Begonia biflora*	SYM-32236-sample4	JF975961	JN044012	JF940952	JF953284
Eudicotyledons	Begoniaceae	*Begonia*	*Begonia biflora*	SYM-20484	JF975965	JN044016	JF940956	JF953287
Eudicotyledons	Begoniaceae	*Begonia*	*Begonia biflora*	SYM-44123	JF975959	JN044009	JF940950	JF953282
Eudicotyledons	Begoniaceae	*Begonia*	*Begonia baviensis*	SYM-D-57-sample2	JF975957	JN044007	JF940947	JF953279
Eudicotyledons	Begoniaceae	*Begonia*	*Begonia baviensis*	SYM-D-57-sample1	JF975958	JN044008	JF940948	JF953280
Eudicotyledons	Begoniaceae	*Begonia*	*Begonia baviensis*	SYM-GBOWS1324-sample1	JF975956	JN044006	JF940946	JF953278
Eudicotyledons	Begoniaceae	*Begonia*	*Begonia baviensis*	SYM-GBOWS1324-sample2	JF975954	JN044004	JF940944	JF953276
Eudicotyledons	Begoniaceae	*Begonia*	*Begonia baviensis*	SYM-GBOWS1324-sample3	JF975953	JN044003	JF940943	JF953275
Eudicotyledons	Begoniaceae	*Begonia*	*Begonia baviensis*	SYM-GBOWS1324-sample13	JF975955	JN044005	JF940945	JF953277
Eudicotyledons	Amaranthaceae	*Amaranthus*	*Amaranthus tricolor*	Z30	JF975876	JN043909	JF940812	JF953165
Eudicotyledons	Amaranthaceae	*Amaranthus*	*Amaranthus tricolor*	Z31	JF975875	JN043908	JF940811	JF953164
Eudicotyledons	Amaranthaceae	*Amaranthus*	*Amaranthus tricolor*	Z34	JF975874	JN043905	JF940808	JF953161
Eudicotyledons	Amaranthaceae	*Amaranthus*	*Amaranthus tricolor*	Z35	JF975873	JN043904	JF940807	JF953160
Eudicotyledons	Amaranthaceae	*Amaranthus*	*Amaranthus tricolor*	Z36	JF975872	JN043903	JF940806	JF953159
Eudicotyledons	Amaranthaceae	*Amaranthus*	*Amaranthus tricolor*	Z37	JF975871	JN043902	JF940805	JF953158
Eudicotyledons	Amaranthaceae	*Amaranthus*	*Amaranthus spinosus*	Z27	JF975865	JN043896	JF940799	JF953152
Eudicotyledons	Amaranthaceae	*Amaranthus*	*Amaranthus spinosus*	Z26	JF975866	JN043897	JF940800	JF953153
Eudicotyledons	Amaranthaceae	*Amaranthus*	*Amaranthus spinosus*	Z25	JF975867	JN043898	JF940801	JF953154
Eudicotyledons	Amaranthaceae	*Amaranthus*	*Amaranthus spinosus*	Z24	JF975868	JN043899	JF940802	JF953155
Eudicotyledons	Amaranthaceae	*Amaranthus*	*Amaranthus spinosus*	Z23	JF975869	JN043900	JF940803	JF953156

Eudicotyledons	Amaranthaceae	*Amaranthus*	*Amaranthus spinosus*	Z22	JF975870	JN043901	JF940804	JF953157
Eudicotyledons	Amaranthaceae	*Amaranthus*	*Amaranthus spinosus*	Z29	JF975863	JN043894	JF940797	JF953150
Eudicotyledons	Amaranthaceae	*Amaranthus*	*Amaranthus spinosus*	Z28	JF975864	JN043895	JF940798	JF953151
Eudicotyledons	Amaranthaceae	*Amaranthus*	*Amaranthus retroflexus*	Z50	JF975859	JN043886	JF940789	JF953142
Eudicotyledons	Amaranthaceae	*Amaranthus*	*Amaranthus retroflexus*	Z47	JF975860	JN043889	JF940792	JF953145
Eudicotyledons	Amaranthaceae	*Amaranthus*	*Amaranthus retroflexus*	Z46	JF975861	JN043890	JF940793	JF953146
Eudicotyledons	Amaranthaceae	*Amaranthus*	*Amaranthus retroflexus*	Z44	JF975862	JN043892	JF940795	JF953148
Eudicotyledons	Amaranthaceae	*Amaranthus*	*Amaranthus hybridus*	Z21	JF975857	JN043883	JF940786	JF953140
Eudicotyledons	Amaranthaceae	*Amaranthus*	*Amaranthus hybridus*	Z20	JF975858	JN043884	JF940787	JF953141
Eudicotyledons	Amaranthaceae	*Amaranthus*	*Amaranthus albus*	Z4	JF975853	JN043879	JF940782	JF953136
Eudicotyledons	Amaranthaceae	*Amaranthus*	*Amaranthus albus*	Z2	JF975855	JN043881	JF940784	JF953138
Eudicotyledons	Amaranthaceae	*Amaranthus*	*Amaranthus albus*	Z3	JF975854	JN043880	JF940783	JF953137
Eudicotyledons	Amaranthaceae	*Amaranthus*	*Amaranthus albus*	Z1	JF975856	JN043882	JF940785	JF953139
Eudicotyledons	Ranunculaceae	*Aconitum*	*Aconitum sinomontanum*	Yangqe0166	JF975824	JN043775	JF940678	JF953043
Eudicotyledons	Ranunculaceae	*Aconitum*	*Aconitum sinomontanum*	Yangqe0167	JF975823	JN043774	JF940677	JF953042
Eudicotyledons	Ranunculaceae	*Aconitum*	*Aconitum sinomontanum*	Yangqe0195	JF975821	JN043772	JF940675	JF953040
Eudicotyledons	Ranunculaceae	*Aconitum*	*Aconitum sinomontanum*	Yangqe0194	JF975822	JN043773	JF940676	JF953041
Eudicotyledons	Ranunculaceae	*Aconitum*	*Aconitum scaposum*	Yangqe0168	JF975818	JN043769	JF940674	JF953039
Eudicotyledons	Ranunculaceae	*Aconitum*	*Aconitum scaposum*	Yangqe0201	JF975815	JN043766	JF940670	JF953037
Eudicotyledons	Ranunculaceae	*Aconitum*	*Aconitum scaposum*	Yangqe0200	JF975816	JN043767	JF940671	JF953038
Eudicotyledons	Ranunculaceae	*Aconitum*	*Aconitum monticola*	Yangqe0174	JF975814	JN043765	JF940669	JF953036
Eudicotyledons	Ranunculaceae	*Aconitum*	*Aconitum monticola*	Yangqe0191	JF975813	JN043764	JF940668	JF953035
Eudicotyledons	Ranunculaceae	*Aconitum*	*Aconitum longecassidatum*	Yangqe0228	JF975808	JN043758	JF940662	JF953030
Eudicotyledons	Ranunculaceae	*Aconitum*	*Aconitum longecassidatum*	Yangqe0165	JF975811	JN043761	JF940665	JF953032
Eudicotyledons	Ranunculaceae	*Aconitum*	*Aconitum longecassidatum*	Yangqe0198	JF975809	JN043759	JF940663	JF953031
Eudicotyledons	Ranunculaceae	*Aconitum*	*Aconitum longecassidatum*	Yangqe0229	JF975807	JN043757	JF940661	JF953029
Eudicotyledons	Ranunculaceae	*Aconitum*	*Aconitum longecassidatum*	Yangqe0164	JF975812	JN043762	JF940666	JF953033
Eudicotyledons	Ranunculaceae	*Aconitum*	*Aconitum barbatum*	Yangqe0209	JF975799	JN043748	JF940653	JF953020
Eudicotyledons	Ranunculaceae	*Aconitum*	*Aconitum barbatum*	Yangqe0208	JF975800	JN043749	JF940654	JF953021
Eudicotyledons	Ranunculaceae	*Aconitum*	*Aconitum barbatum*	Yangqe0177	JF975802	JN043751	JF940655	JF953023
Eudicotyledons	Ranunculaceae	*Aconitum*	*Aconitum barbatum*	Yangqe0176	JF975803	JN043752	JF940656	JF953024
Eudicotyledons	Ranunculaceae	*Aconitum*	*Aconitum barbatum*	Yangqe0210	JF975798	JN043747	JF940652	JF953019
Eudicotyledons	Ranunculaceae	*Aconitum*	*Aconitum angustius*	Yangqe0223	JF975790	JN043739	JF940644	JF953011
Eudicotyledons	Ranunculaceae	*Aconitum*	*Aconitum angustius*	Yangqe0212	JF975794	JN043743	JF940648	JF953015
Eudicotyledons	Ranunculaceae	*Aconitum*	*Aconitum angustius*	Yangqe0213	JF975793	JN043742	JF940647	JF953014
Eudicotyledons	Ranunculaceae	*Aconitum*	*Aconitum angustius*	Yangqe0211	JF975795	JN043744	JF940649	JF953016
Eudicotyledons	Ranunculaceae	*Aconitum*	*Aconitum angustius*	Yangqe0214	JF975792	JN043741	JF940646	JF953013
Eudicotyledons	Ranunculaceae	*Aconitum*	*Aconitum angustius*	Yangqe0222	JF975791	JN043740	JF940645	JF953012
Eudicotyledons	Ranunculaceae	*Aconitum*	*Aconitum angustius*	Yangqe0227	JF975787	JN043736	JF940641	JF953008
Eudicotyledons	Ranunculaceae	*Aconitum*	*Aconitum angustius*	Yangqe0226	JF975788	JN043737	JF940642	JF953009

Eudicotyledons	Ranunculaceae	*Aconitum*	*Aconitum angustius*	Yangqe0225	JF975789	JN043738	JF940643	JF953010
Eudicotyledons	Ranunculaceae	*Aconitum*	*Aconitum angustius*	Yangqe0185	JF975796	JN043745	JF940650	JF953017
Eudicotyledons	Ranunculaceae	*Aconitum*	*Aconitum alboviolaceum*	Yangqe0172	JF975785	JN043734	JF940639	JF953006
Eudicotyledons	Ranunculaceae	*Aconitum*	*Aconitum alboviolaceum*	Yangqe0202	JF975784	JN043733	JF940638	JF953005
Eudicotyledons	Ranunculaceae	*Aconitum*	*Aconitum alboviolaceum*	Yangqe0171	JF975786	JN043735	JF940640	JF953007
Eudicotyledons	Ranunculaceae	*Aconitum*	*Aconitum alboviolaceum*	Yangqe0203	JF975783	JN043732	JF940637	JF953004
Eudicotyledons	Ranunculaceae	*Aconitum*	*Aconitum alboviolaceum*	Yangqe0204	JF975782	JN043731	JF940636	JF953003
Eudicotyledons	Aceraceae	*Acer*	*Acer tataricum*	Z86	JF975780	JN043729	JF940633	JF953000
Eudicotyledons	Aceraceae	*Acer*	*Acer tataricum*	Z85	JF975781	JN043730	JF940634	JF953001
Eudicotyledons	Aceraceae	*Acer*	*Acer pectinatum*	Z52	JF975779	JN043727	JF940631	JF952997
Eudicotyledons	Aceraceae	*Acer*	*Acer pectinatum*	Z53	JF975778	JN043726	JF940630	JF952996
Eudicotyledons	Aceraceae	*Acer*	*Acer fabri*	Z89	JF975776	JN043724	JF940626	JF952992
Eudicotyledons	Aceraceae	*Acer*	*Acer fabri*	Z88	JF975777	JN043725	JF940627	JF952993
Eudicotyledons	Aceraceae	*Acer*	*Acer fabri*	Z92	JF975774	JN043722	JF940624	JF952990
Eudicotyledons	Aceraceae	*Acer*	*Acer fabri*	Z91	JF975775	JN043723	JF940625	JF952991
Eudicotyledons	Aceraceae	*Acer*	*Acer davidii*	Z79	JF975770	JN043713	JF940614	JF952981
Eudicotyledons	Aceraceae	*Acer*	*Acer davidii*	Z72	JF975773	JN043719	JF940621	JF952987
Eudicotyledons	Aceraceae	*Acer*	*Acer davidii*	Z73	JF975772	JN043718	JF940620	JF952986
Eudicotyledons	Aceraceae	*Acer*	*Acer davidii*	Z75	JF975771	JN043716	JF940618	JF952984
Gymnosperms	Cephalotaxacea	*Cephalotaxus*	*Cephalotaxus sinensis*	C123	JF976116	JN044261	JF941240	JF953517
Gymnosperms	Cephalotaxacea	*Cephalotaxus*	*Cephalotaxus sinensis*	C125	JF976115	JN044260	JF941239	JF953516
Gymnosperms	Cephalotaxacea	*Cephalotaxus*	*Cephalotaxus sinensis*	C035	JF976125	JN044270	JF941249	JF953526
Gymnosperms	Cephalotaxacea	*Cephalotaxus*	*Cephalotaxus sinensis*	C037	JF976124	JN044269	JF941248	JF953525
Gymnosperms	Cephalotaxacea	*Cephalotaxus*	*Cephalotaxus sinensis*	C032	JF976126	JN044271	JF941250	JF953527
Gymnosperms	Cephalotaxacea	*Cephalotaxus*	*Cephalotaxus sinensis*	C057	JF976122	JN044267	JF941246	JF953523
Gymnosperms	Cephalotaxacea	*Cephalotaxus*	*Cephalotaxus sinensis*	C052	JF976123	JN044268	JF941247	JF953524
Gymnosperms	Cephalotaxacea	*Cephalotaxus*	*Cephalotaxus sinensis*	C113	JF976117	JN044262	JF941241	JF953518
Gymnosperms	Cephalotaxacea	*Cephalotaxus*	*Cephalotaxus sinensis*	C083	JF976121	JN044266	JF941245	JF953522
Gymnosperms	Cephalotaxacea	*Cephalotaxus*	*Cephalotaxus sinensis*	C107	JF976119	JN044264	JF941243	JF953520
Gymnosperms	Cephalotaxacea	*Cephalotaxus*	*Cephalotaxus sinensis*	C096	JF976120	JN044265	JF941244	JF953521
Gymnosperms	Cephalotaxacea	*Cephalotaxus*	*Cephalotaxus sinensis*	C109	JF976118	JN044263	JF941242	JF953519
Gymnosperms	Cephalotaxacea	*Cephalotaxus*	*Cephalotaxus oliveri*	C041	JF976114	JN044259	JF941238	JF953515
Gymnosperms	Cephalotaxacea	*Cephalotaxus*	*Cephalotaxus oliveri*	C044	JF976113	JN044258	JF941237	JF953514
Gymnosperms	Cephalotaxacea	*Cephalotaxus*	*Cephalotaxus mannii*	C124	JF976107	JN044249	JF941228	JF953505
Gymnosperms	Cephalotaxacea	*Cephalotaxus*	*Cephalotaxus mannii*	C021	JF976110	JN044255	JF941234	JF953511
Gymnosperms	Cephalotaxacea	*Cephalotaxus*	*Cephalotaxus mannii*	C019	JF976111	JN044256	JF941235	JF953512
Gymnosperms	Cephalotaxacea	*Cephalotaxus*	*Cephalotaxus mannii*	C111	JF976108	JN044250	JF941229	JF953506
Gymnosperms	Cephalotaxacea	*Cephalotaxus*	*Cephalotaxus mannii*	5456	#N/A	#N/A	#N/A	#N/A
Gymnosperms	Cephalotaxacea	*Cephalotaxus*	*Cephalotaxus mannii*	KIB-C039	JF976109	JN044251	JF941230	JF953507
Gymnosperms	Cephalotaxacea	*Cephalotaxus*	*Cephalotaxus lanceolata*	C116	JF976105	JN044247	JF941226	JF953503

Gymnosperms	Cephalotaxacea	*Cephalotaxus*	*Cephalotaxus lanceolata*	C031	JF976106	JN044248	JF941227	JF953504
Gymnosperms	Cephalotaxacea	*Cephalotaxus*	*Cephalotaxus fortunei*	C002	JF976101	JN044243	JF941222	JF953499
Gymnosperms	Cephalotaxacea	*Cephalotaxus*	*Cephalotaxus fortunei*	KIB-C017	JF976097	JN044239	JF941218	JF953495
Gymnosperms	Cephalotaxacea	*Cephalotaxus*	*Cephalotaxus fortunei*	KIB-C008	JF976100	JN044242	JF941221	JF953498
Gymnosperms	Cephalotaxacea	*Cephalotaxus*	*Cephalotaxus fortunei*	C018	JF976096	JN044238	JF941217	JF953494
Gymnosperms	Cephalotaxacea	*Cephalotaxus*	*Cephalotaxus fortunei*	C011	JF976099	JN044241	JF941220	JF953497
Gymnosperms	Cephalotaxacea	*Cephalotaxus*	*Cephalotaxus fortunei*	C014	JF976098	JN044240	JF941219	JF953496
Gymnosperms	Cephalotaxacea	*Cephalotaxus*	*Cephalotaxus fortunei*	C087	JF976095	JN044237	JF941216	JF953493
Gymnosperms	Taxaceae	*Amentotaxus*	*Amentotaxus yunnanensis*	Am21	JF975889	JN043923	JF940826	JF953179
Gymnosperms	Taxaceae	*Amentotaxus*	*Amentotaxus yunnanensis*	Am22	JF975888	JN043922	JF940825	JF953178
Gymnosperms	Taxaceae	*Amentotaxus*	*Amentotaxus poilanei*	Am12	JF975887	JN043921	JF940824	JF953177
Gymnosperms	Taxaceae	*Amentotaxus*	*Amentotaxus poilanei*	Am19	JF975886	JN043920	JF940823	JF953176
Gymnosperms	Taxaceae	*Amentotaxus*	*Amentotaxus poilanei*	Am20	JF975885	JN043919	JF940822	JF953175
Gymnosperms	Taxaceae	*Amentotaxus*	*Amentotaxus formosana*	Am09	JF975884	JN043917	JF940820	JF953173
Gymnosperms	Taxaceae	*Amentotaxus*	*Amentotaxus formosana*	Am11	JF975882	JN043915	JF940818	JF953171
Gymnosperms	Taxaceae	*Amentotaxus*	*Amentotaxus formosana*	Am10	JF975883	JN043916	JF940819	JF953172
Gymnosperms	Taxaceae	*Amentotaxus*	*Amentotaxus argotaenia*	Am03	JF975880	JN043913	JF940816	JF953169
Gymnosperms	Taxaceae	*Amentotaxus*	*Amentotaxus argotaenia*	Am01	JF975881	JN043914	JF940817	JF953170
Gymnosperms	Taxaceae	*Amentotaxus*	*Amentotaxus argotaenia*	Am06	JF975877	JN043910	JF940813	JF953166
Gymnosperms	Taxaceae	*Amentotaxus*	*Amentotaxus argotaenia*	Am04	JF975879	JN043912	JF940815	JF953168
Gymnosperms	Taxaceae	*Amentotaxus*	*Amentotaxus argotaenia*	Am05	JF975878	JN043911	JF940814	JF953167
Monocotyledons	Amaryllidaceae	*Allium*	*Allium cyaneum*	Li_Q-Q_092201	HQ690268	HQ690365	HQ69049	HQ69031
Monocotyledons	Amaryllidaceae	*Allium*	*Allium cyaneum*	Li_Q-Q_09080806	HQ690563	HQ690626	HQ69044	HQ69071
Monocotyledons	Amaryllidaceae	*Allium*	*Allium cyaneum*	Ma_X-G_09080403	HQ690267	HQ690364	HQ69048	HQ69031
Monocotyledons	Amaryllidaceae	*Allium*	*Allium victorialis*	Li_Q-Q_MH09072524	HQ690550	HQ690620	HQ69043	HQ69070
Monocotyledons	Amaryllidaceae	*Allium*	*Allium victorialis*	Zhao_L-H_LQQ10081501	HQ690559	HQ690390	HQ69043	HQ69070
Monocotyledons	Amaryllidaceae	*Allium*	*Allium victorialis*	Zhao_L-H_10071904	HQ690558	HQ690389	HQ69042	HQ69069
Monocotyledons	Amaryllidaceae	*Allium*	*Allium mairei*	Peng_L_LQQ2010082801	HQ690289	HQ690394	HQ69051	HQ69034
Monocotyledons	Amaryllidaceae	*Allium*	*Allium mairei*	Wang_Z-X_2010091008	HQ690574	HQ690659	HQ69046	HQ69074
Monocotyledons	Amaryllidaceae	*Allium*	*Allium mairei*	Wang_Z-X_WZX2010091102	HQ690290	HQ690395	HQ69051	HQ69034
Monocotyledons	Amaryllidaceae	*Allium*	*Allium ramosum*	Wang_C-B_09068	HQ690285	HQ690385	HQ69051	HQ69033
Monocotyledons	Amaryllidaceae	*Allium*	*Allium ramosum*	Wang_C-B_09056	HQ690284	HQ690384	HQ69050	HQ69033
Monocotyledons	Amaryllidaceae	*Allium*	*Allium ramosum*	Li_Q-Q_&_Wei_X-Q_2009060502	HQ690565	HQ690628	HQ690447	HQ690716
Monocotyledons	Amaryllidaceae	*Allium*	*Allium wallichii*	Li_Q-Q_BT09072818	HQ690250	HQ690345	HQ69046	HQ69029
Monocotyledons	Amaryllidaceae	*Allium*	*Allium wallichii*	Li_Q-Q_YC09072907	HQ690251	HQ690346	HQ69047	HQ69029
Monocotyledons	Amaryllidaceae	*Allium*	*Allium wallichii*	Yu_Y_yy10080905	HQ690252	HQ690347	HQ69047	HQ69029
Monocotyledons	Amaryllidaceae	*Allium*	*Allium wallichii*	Li_Q-Q_MH09072515	HQ690249	HQ690344	HQ69046	HQ69029
Monocotyledons	Amaryllidaceae	*Allium*	*Allium wallichii*	Li_Q-Q_YL09072630	HQ690566	HQ690629	HQ69044	HQ69071
Monocotyledons	Amaryllidaceae	*Allium*	*Allium wallichii*	Liu_S_&_Gao_P_20100903-	HQ690253	HQ690348	HQ69047	HQ69029

Monocotyledons	Amaryllidaceae	*Allium*	*Allium macranthum*	Li_Q-Q_092206	HQ690255	HQ690630	HQ69047	HQ69029
Monocotyledons	Amaryllidaceae	*Allium*	*Allium macranthum*	Li_Q-Q_092303	HQ690254	HQ690350	HQ69047	HQ69029
Monocotyledons	Amaryllidaceae	*Allium*	*Allium macranthum*	Li_Q-Q_09080901	HQ690562	HQ690349	HQ69044	HQ69071
Monocotyledons	Amaryllidaceae	*Allium*	*Allium macranthum*	Gao_Y-D_G2010081702	HQ690256	HQ690351	HQ69047	HQ69029
Monocotyledons	Amaryllidaceae	*Allium*	*Allium sikkimense*	Gao_Y-D_G2010090201	HQ690264	HQ690360	HQ69048	HQ69030
Monocotyledons	Amaryllidaceae	*Allium*	*Allium sikkimense*	Li_Q-Q_BW09080105	HQ690572	HQ690355	HQ69048	HQ69030
Monocotyledons	Amaryllidaceae	*Allium*	*Allium sikkimense*	Li_Q-Q_&_Wei_X-Q_092207	HQ690259	HQ690354	HQ69047	HQ69030
Monocotyledons	Amaryllidaceae	*Allium*	*Allium sikkimense*	Li_Q-Q_09080804	HQ690263	HQ690359	HQ69048	HQ69030
Monocotyledons	Amaryllidaceae	*Allium*	*Allium sikkimense*	Peng_L_PL2010083003	HQ690261	HQ690357	HQ69048	HQ69030
Monocotyledons	Amaryllidaceae	*Allium*	*Allium sikkimense*	Huang_D-Q_2010091201	HQ690260	HQ690356	HQ69048	HQ69030
Monocotyledons	Amaryllidaceae	*Allium*	*Allium sikkimense*	Li_Q-Q_2008082201	HQ690262	HQ690358	HQ69048	HQ69030
Monocotyledons	Amaryllidaceae	*Allium*	*Allium sikkimense*	Li_Q-Q_2008081602	HQ690257	HQ690352	HQ69047	HQ69030
Monocotyledons	Amaryllidaceae	*Allium*	*Allium condensatum*	Wang_C-B_0922	HQ690573	HQ690658	HQ69046	HQ69074
Monocotyledons	Amaryllidaceae	*Allium*	*Allium condensatum*	Zhao_L-H_LQQ100718	HQ690291	HQ690397	HQ69051	HQ69034
Monocotyledons	Amaryllidaceae	*Allium*	*Allium forrestii*	Gao_Y-D_G2010090202	HQ690265	HQ690362	HQ69048	HQ69031
Monocotyledons	Amaryllidaceae	*Allium*	*Allium forrestii*	Ma_X-G_m10083103	HQ690266	HQ690363	HQ69048	HQ69031
Monocotyledons	Amaryllidaceae	*Allium*	*Allium maowenense*	Li_Q-Q_2008082104	HQ690278	HQ690377	HQ69050	HQ69032
Monocotyledons	Amaryllidaceae	*Allium*	*Allium maowenense*	Huang_D-Q_2010091401	HQ690280	HQ690379	HQ69050	HQ69032
Monocotyledons	Amaryllidaceae	*Allium*	*Allium maowenense*	Ma_X-G_mxg09101301	HQ690279	HQ690378	HQ69050	HQ69032
Monocotyledons	Amaryllidaceae	*Allium*	*Allium maowenense*	Li_Q-Q_2008081904	HQ690277	HQ690376	HQ69050	HQ69032
Monocotyledons	Amaryllidaceae	*Allium*	*Allium prattii*	Gao_Y-D_20100718	HQ690552	HQ690622	HQ69043	HQ69070
Monocotyledons	Amaryllidaceae	*Allium*	*Allium prattii*	Xu_Y_P090809	HQ690540	HQ690591	HQ69041	HQ69067
Monocotyledons	Amaryllidaceae	*Allium*	*Allium prattii*	Li_Q-Q_&_Wei_X-Q_2009060903	HQ690531	HQ690584	HQ690407	HQ690669
Monocotyledons	Amaryllidaceae	*Allium*	*Allium prattii*	Liu_S_&_Gao_P_10903	HQ690551	HQ690621	HQ69043	HQ69070
Monocotyledons	Amaryllidaceae	*Allium*	*Allium prattii*	Li_Q-Q_&_Wei_X-Q_2009062101	HQ690532	HQ690586	HQ690409	HQ690671
Monocotyledons	Amaryllidaceae	*Allium*	*Allium prattii*	Li_Q-Q_BT09072812	HQ690541	HQ690596	HQ69041	HQ69068
Monocotyledons	Amaryllidaceae	*Allium*	*Allium prattii*	Li_Q-Q_BT09072810	HQ690534	HQ690588	HQ69041	HQ69067
Monocotyledons	Amaryllidaceae	*Allium*	*Allium prattii*	Li_Q-Q_BW09080104	HQ690538	HQ690594	HQ69041	HQ69067
Monocotyledons	Amaryllidaceae	*Allium*	*Allium prattii*	Li_Q-Q_YC09072921	HQ690535	HQ690589	HQ69041	HQ69067
Monocotyledons	Amaryllidaceae	*Allium*	*Allium prattii*	Li_Q-Q_BW09080102	HQ690537	HQ690590	HQ69041	HQ69067
Monocotyledons	Amaryllidaceae	*Allium*	*Allium rude*	Li_Q-Q_2008081902	HQ690269	HQ690366	HQ69049	HQ69031
Monocotyledons	Amaryllidaceae	*Allium*	*Allium rude*	Huang_D-Q_2010091402	HQ690271	HQ690369	HQ69049	HQ69031
Monocotyledons	Amaryllidaceae	*Allium*	*Allium rude*	Li_Q-Q_2008082103	HQ690270	HQ690367	HQ69049	HQ69031
Monocotyledons	Amaryllidaceae	*Allium*	*Allium rude*	Li_Q-Q_092202	HQ690561	HQ690623	HQ69044	HQ69070
Monocotyledons	Amaryllidaceae	*Allium*	*Allium xichuanense*	Li_Q-Q_2008081901	HQ690564	HQ690627	HQ69044	HQ69071
Monocotyledons	Amaryllidaceae	*Allium*	*Allium xichuanense*	Li_Q-Q_092203	HQ690273	HQ690371	HQ69049	HQ69032
Monocotyledons	Amaryllidaceae	*Allium*	*Allium xichuanense*	Li_Q-Q_092103	HQ690272	HQ690370	HQ69049	HQ69031
Monocotyledons	Asparagaceae	*Tupistra*	*Tupistra pingbianensis*	zhangcq0012	JF978993	JN047474	JF944736	JF956764

Monocotyledons	Asparagaceae	*Tupistra*	*Tupistra pingbianensis*	zhangcq0013	JF978992	JN047473	JF944735	JF956763
Monocotyledons	Asparagaceae	*Tupistra*	*Tupistra pingbianensis*	zhangcq0011	JF978994	JN047475	JF944737	JF956765
Monocotyledons	Asparagaceae	*Tupistra*	*Tupistra pingbianensis*	zhangcq0014	JF978991	JN047472	JF944734	JF956762
Monocotyledons	Asparagaceae	*Tupistra*	*Tupistra longispica*	zhangcq0017	JF978989	JN047470	JF944731	JF956760
Monocotyledons	Asparagaceae	*Tupistra*	*Tupistra longispica*	zhangcq0020	JF978986	JN047467	JF944728	JF956757
Monocotyledons	Asparagaceae	*Tupistra*	*Tupistra longispica*	zhangcq0018	JF978988	JN047469	JF944730	JF956759
Monocotyledons	Asparagaceae	*Tupistra*	*Tupistra longispica*	zhangcq0019	JF978987	JN047468	JF944729	JF956758
Monocotyledons	Asparagaceae	*Tupistra*	*Tupistra longispica*	zhangcq0016	JF978990	JN047471	JF944732	JF956761
Monocotyledons	Dioscoreaceae	*Tacca*	*Tacca subflabellata*	ZL-tacca-012	JF978886	JN047361	JF944623	JF956653
Monocotyledons	Dioscoreaceae	*Tacca*	*Tacca subflabellata*	ZL014	JF978884	JN047359	JF944621	JF956652
Monocotyledons	Dioscoreaceae	*Tacca*	*Tacca subflabellata*	ZL-tacca-011	JF978887	JN047362	JF944624	JF956654
Monocotyledons	Dioscoreaceae	*Tacca*	*Tacca plantaginea*	ZL002	JF978882	JN047357	JF944619	JF956650
Monocotyledons	Dioscoreaceae	*Tacca*	*Tacca plantaginea*	ZL001	JF978883	JN047358	JF944620	JF956651
Monocotyledons	Dioscoreaceae	*Tacca*	*Tacca plantaginea*	ZL047	JF978877	JN047352	JF944614	JF956645
Monocotyledons	Dioscoreaceae	*Tacca*	*Tacca plantaginea*	ZL006	JF978880	JN047355	JF944617	JF956648
Monocotyledons	Dioscoreaceae	*Tacca*	*Tacca plantaginea*	ZL005	JF978881	JN047356	JF944618	JF956649
Monocotyledons	Dioscoreaceae	*Tacca*	*Tacca plantaginea*	ZL-tacca-009	JF978879	JN047354	JF944616	JF956647
Monocotyledons	Dioscoreaceae	*Tacca*	*Tacca plantaginea*	ZL-tacca-010	JF978878	JN047353	JF944615	JF956646
Monocotyledons	Dioscoreaceae	*Tacca*	*Tacca integrifolia*	ZL036	JF978862	JN047329	JF944593	JF956624
Monocotyledons	Dioscoreaceae	*Tacca*	*Tacca integrifolia*	ZL034	JF978864	JN047331	JF944595	JF956626
Monocotyledons	Dioscoreaceae	*Tacca*	*Tacca integrifolia*	ZL035	JF978863	JN047330	JF944594	JF956625
Monocotyledons	Dioscoreaceae	*Tacca*	*Tacca integrifolia*	ZL033	JF978865	JN047332	JF944596	JF956627
Monocotyledons	Dioscoreaceae	*Tacca*	*Tacca integrifolia*	ZL031	JF978867	JN047334	JF944598	JF956629
Monocotyledons	Dioscoreaceae	*Tacca*	*Tacca integrifolia*	ZL008	JF978875	JN047342	JF944606	JF956636
Monocotyledons	Dioscoreaceae	*Tacca*	*Tacca integrifolia*	ZL030	JF978868	JN047335	JF944599	JF956630
Monocotyledons	Dioscoreaceae	*Tacca*	*Tacca integrifolia*	ZL007	JF978876	JN047343	JF944607	JF956637
Monocotyledons	Dioscoreaceae	*Tacca*	*Tacca integrifolia*	ZL023	JF978873	JN047340	JF944604	JF956634
Monocotyledons	Dioscoreaceae	*Tacca*	*Tacca integrifolia*	ZL032	JF978866	JN047333	JF944597	JF956628
Monocotyledons	Dioscoreaceae	*Tacca*	*Tacca integrifolia*	ZL025	JF978871	JN047338	JF944602	JF956632
Monocotyledons	Dioscoreaceae	*Tacca*	*Tacca integrifolia*	ZL024	JF978872	JN047339	JF944603	JF956633
Monocotyledons	Dioscoreaceae	*Tacca*	*Tacca integrifolia*	ZL022	JF978874	JN047341	JF944605	JF956635
Monocotyledons	Dioscoreaceae	*Tacca*	*Tacca integrifolia*	ZL029	JF978869	JN047336	JF944600	JF956631
Monocotyledons	Dioscoreaceae	*Tacca*	*Tacca chantieri*	ZL050	JF978860	JN047327	JF944591	JF956622
Monocotyledons	Dioscoreaceae	*Tacca*	*Tacca chantieri*	ZL051	JF978859	JN047326	JF944590	JF956621
Monocotyledons	Dioscoreaceae	*Tacca*	*Tacca chantieri*	ZL-tacca-052	JF978858	JN047325	JF944589	JF956620
Monocotyledons	Dioscoreaceae	*Tacca*	*Tacca chantieri*	ZL053	JF978857	JN047324	JF944588	JF956619
Monocotyledons	Dioscoreaceae	*Tacca*	*Tacca chantieri*	ZL-tacca-049	JF978861	JN047328	JF944592	JF956623
Monocotyledons	Dioscoreaceae	*Tacca*	*Tacca ampliplacenta*	ZL038	JF978855	JN047322	JF944586	JF956617
Monocotyledons	Dioscoreaceae	*Tacca*	*Tacca ampliplacenta*	ZL037	JF978856	JN047323	JF944587	JF956618
Monocotyledons	Dioscoreaceae	*Tacca*	*Tacca ampliplacenta*	ZL044	JF978849	JN047316	JF944580	JF956611

Monocotyledons	Dioscoreaceae	*Tacca*	*Tacca ampliplacenta*	ZL043	JF978850	JN047317	JF944581	JF956612
Monocotyledons	Dioscoreaceae	*Tacca*	*Tacca ampliplacenta*	ZL045	JF978848	JN047315	JF944579	JF956610
Monocotyledons	Dioscoreaceae	*Tacca*	*Tacca ampliplacenta*	ZL042	JF978851	JN047318	JF944582	JF956613
Monocotyledons	Dioscoreaceae	*Tacca*	*Tacca ampliplacenta*	ZL046	JF978847	JN047314	JF944578	JF956609
Monocotyledons	Dioscoreaceae	*Tacca*	*Tacca ampliplacenta*	ZL-tacca-041	JF978852	JN047319	JF944583	JF956614
Monocotyledons	Dioscoreaceae	*Tacca*	*Tacca ampliplacenta*	ZL039	JF978854	JN047321	JF944585	JF956616
Monocotyledons	Dioscoreaceae	*Tacca*	*Tacca ampliplacenta*	ZL040	JF978853	JN047320	JF944584	JF956615
Monocotyledons	Smilacaceae	*Smilax*	*Smilax lanceifolia*	FCX-Li39926	JF978724	JN047206	JF944382	JF956420
Monocotyledons	Smilacaceae	*Smilax*	*Smilax lanceifolia*	FCX-Fu08TS068	JF978726	JN047207	JF944384	JF956422
Monocotyledons	Smilacaceae	*Smilax*	*Smilax glabra*	FCX-Fu810307	JF978700	JN047204	JF944361	JF956400
Monocotyledons	Smilacaceae	*Smilax*	*Smilax glabra*	FCX-BQ0905194	JF978702	JN047205	JF944363	JF956402
Monocotyledons	Melanthiaceae	*Paris*	*Paris vietnamensis*	JYH77A	JF977364	JN045777	JF942827	JF954951
Monocotyledons	Melanthiaceae	*Paris*	*Paris vietnamensis*	JYH75A	JF977367	JN045780	JF942829	JF954954
Monocotyledons	Melanthiaceae	*Paris*	*Paris vietnamensis*	JYH75C	JF977365	JN045778	JF942828	JF954952
Monocotyledons	Melanthiaceae	*Paris*	*Paris vaniotii*	JYH85C	JF977359	JN045772	JF942825	JF954946
Monocotyledons	Melanthiaceae	*Paris*	*Paris vaniotii*	JYH85A	JF977361	JN045774	JF942826	JF954948
Monocotyledons	Melanthiaceae	*Paris*	*Paris thibetica*	JYH60D	JF977356	JN045769	JF942823	JF954943
Monocotyledons	Melanthiaceae	*Paris*	*Paris thibetica*	JYH60B	JF977357	JN045770	JF942824	JF954944
Monocotyledons	Melanthiaceae	*Paris*	*Paris thibetica*	JYH67F	JF977353	JN045766	JF942820	JF954940
Monocotyledons	Melanthiaceae	*Paris*	*Paris thibetica*	JYH67D	JF977355	JN045768	JF942822	JF954942
Monocotyledons	Melanthiaceae	*Paris*	*Paris thibetica*	JYH67E	JF977354	JN045767	JF942821	JF954941
Monocotyledons	Melanthiaceae	*Paris*	*Paris thibetica*	JYH69B	JF977351	JN045764	JF942819	JF954938
Monocotyledons	Melanthiaceae	*Paris*	*Paris thibetica*	JYH69C	JF977350	JN045763	JF942818	JF954937
Monocotyledons	Melanthiaceae	*Paris*	*Paris rugosa*	JYH80C	JF977347	JN045760	JF942815	JF954934
Monocotyledons	Melanthiaceae	*Paris*	*Paris rugosa*	JYH80A	JF977349	JN045762	JF942817	JF954936
Monocotyledons	Melanthiaceae	*Paris*	*Paris rugosa*	JYH80B	JF977348	JN045761	JF942816	JF954935
Monocotyledons	Melanthiaceae	*Paris*	*Paris quadrifolia*	JYH79C	JF977344	JN045757	JF942812	JF954931
Monocotyledons	Melanthiaceae	*Paris*	*Paris quadrifolia*	JYH79A	JF977346	JN045759	JF942814	JF954933
Monocotyledons	Melanthiaceae	*Paris*	*Paris quadrifolia*	JYH79B	JF977345	JN045758	JF942813	JF954932
Monocotyledons	Melanthiaceae	*Paris*	*Paris marmorata*	JYH50A	JF977314	JN045737	JF942785	JF954901
Monocotyledons	Melanthiaceae	*Paris*	*Paris marmorata*	JYH50C	JF977312	JN045735	JF942784	JF954899
Monocotyledons	Melanthiaceae	*Paris*	*Paris marmorata*	JYH49A	JF977316	JN045739	JF942788	JF954904
Monocotyledons	Melanthiaceae	*Paris*	*Paris mairei*	JYH53B	JF977307	JN045733	JF942781	JF954894
Monocotyledons	Melanthiaceae	*Paris*	*Paris mairei*	JYH63C	JF977305	JN045731	JF942780	JF954892
Monocotyledons	Melanthiaceae	*Paris*	*Paris luquanensis*	JYH82B	JF977298	JN045727	JF942777	JF954885
Monocotyledons	Melanthiaceae	*Paris*	*Paris luquanensis*	JYH82A	JF977299	JN045728	JF942778	JF954886
Monocotyledons	Melanthiaceae	*Paris*	*Paris luquanensis*	JYH82C	JF977297	JN045726	JF942776	JF954884
Monocotyledons	Melanthiaceae	*Paris*	*Paris incompleta*	JYH81B	JF977295	JN045724	JF942774	JF954882
Monocotyledons	Melanthiaceae	*Paris*	*Paris incompleta*	JYH81A	JF977296	JN045725	JF942775	JF954883
Monocotyledons	Melanthiaceae	*Paris*	*Paris fargesii*	JYH87A	JF977293	JN045722	JF942773	JF954880

Monocotyledons	Melanthiaceae	*Paris*	*Paris fargesii*	JYH87B	JF977292	JN045721	JF942772	JF954879
Monocotyledons	Melanthiaceae	*Paris*	*Paris fargesii*	JYH87C	JF977291	JN045720	JF942771	JF954878
Monocotyledons	Melanthiaceae	*Paris*	*Paris fargesii*	JYH90A	JF977290	JN045719	JF942770	JF954877
Monocotyledons	Melanthiaceae	*Paris*	*Paris fargesii*	JYH90B	JF977289	JN045718	JF942769	JF954876
Monocotyledons	Melanthiaceae	*Paris*	*Paris fargesii*	JYH90C	JF977288	JN045717	JF942768	JF954875
Monocotyledons	Melanthiaceae	*Paris*	*Paris dunniana*	JYH76B	JF977286	JN045715	JF942766	JF954873
Monocotyledons	Melanthiaceae	*Paris*	*Paris dunniana*	JYH76A	JF977287	JN045716	JF942767	JF954874
Monocotyledons	Melanthiaceae	*Paris*	*Paris delavayi*	JYH89B	JF977277	JN045707	JF942762	JF954864
Monocotyledons	Melanthiaceae	*Paris*	*Paris delavayi*	JYH89A	JF977278	JN045708	JF942763	JF954865
Monocotyledons	Melanthiaceae	*Paris*	*Paris caobangensis*	JYH78B	JF977269	JN045699	JF942760	JF954856
Monocotyledons	Melanthiaceae	*Paris*	*Paris caobangensis*	JYH78A	JF977270	JN045700	JF942761	JF954857
Monocotyledons	Asparagaceae	*Maianthemum*	*Maianthemum racemosum*	Nie_&_Meng_524	EU850031	EU850238	EU85009	EU85026
Monocotyledons	Asparagaceae	*Maianthemum*	*Maianthemum racemosum*	MengY-n369	JF977038	JN045467	JF942524	JF954615
Monocotyledons	Asparagaceae	*Maianthemum*	*Maianthemum racemosum*	MengY-n311	JF977039	JN045468	JF942525	JF954616
Monocotyledons	Asparagaceae	*Maianthemum*	*Maianthemum racemosum*	Wen_8562	EU850030	EU850237	EU85009	EU85026
Monocotyledons	Asparagaceae	*Maianthemum*	*Maianthemum purpureum*	MengY-z6	JF977032	JN045460	JF942517	JF954609
Monocotyledons	Asparagaceae	*Maianthemum*	*Maianthemum purpureum*	MengY-z5	JF977033	JN045461	JF942518	JF954610
Monocotyledons	Asparagaceae	*Maianthemum*	*Maianthemum purpureum*	MengY-n268	JF977034	JN045463	JF942520	JF954612
Monocotyledons	Asparagaceae	*Maianthemum*	*Maianthemum purpureum*	MengY-n265	JF977036	JN045465	JF942522	JF954614
Monocotyledons	Asparagaceae	*Maianthemum*	*Maianthemum purpureum*	Meng_05_1	EU850014	EU850221	EU85008	EU85025
Monocotyledons	Asparagaceae	*Maianthemum*	*Maianthemum purpureum*	MengY-n267	JF977035	JN045464	JF942521	JF954613
Monocotyledons	Asparagaceae	*Maianthemum*	*Maianthemum japonicum*	Zhangsr0258	JF977028	JN045456	JF942513	JF954606
Monocotyledons	Asparagaceae	*Maianthemum*	*Maianthemum japonicum*	Zhangsr0261	JF977027	JN045454	JF942511	JF954604
Monocotyledons	Asparagaceae	*Maianthemum*	*Maianthemum japonicum*	Meng_223	EU850024	EU850231	EU85009	EU85026
Monocotyledons	Asparagaceae	*Maianthemum*	*Maianthemum japonicum*	MengY-n207	JF977031	JN045459	JF942516	JF954608
Monocotyledons	Asparagaceae	*Maianthemum*	*Maianthemum henryi*	Wen_9017	EU850010	EU850217	EU85007	EU85024
Monocotyledons	Asparagaceae	*Maianthemum*	*Maianthemum henryi*	MengY-n398	JF977025	JN045452	JF942509	JF954602
Monocotyledons	Asparagaceae	*Maianthemum*	*Maianthemum henryi*	MengY-z2	JF977023	JN045450	JF942506	JF954599
Monocotyledons	Asparagaceae	*Maianthemum*	*Maianthemum henryi*	Nie_&_Meng_311	EU850017	EU850224	EU85008	EU85025
Monocotyledons	Asparagaceae	*Maianthemum*	*Maianthemum gongshanense*	Nie_&_Meng_301	EU850007	EU850214	EU85007<br>6	EU85024<br>6
Monocotyledons	Asparagaceae	*Maianthemum*	*Maianthemum gongshanense*	MengY-n192	JF977022	JN045446	JF942502	JF954595
Monocotyledons	Asparagaceae	*Maianthemum*	*Maianthemum gigas*	Martinez_39002	EU850034	EU850241	EU85009	EU85027
Monocotyledons	Asparagaceae	*Maianthemum*	*Maianthemum gigas*	MengY-n394	JF977021	JN045445	JF942501	JF954594
Monocotyledons	Asparagaceae	*Maianthemum*	*Maianthemum bifolium*	Meng_211	EU850028	EU850235	EU85009	EU85026
Monocotyledons	Asparagaceae	*Maianthemum*	*Maianthemum bifolium*	Wen_8530	EU850027	EU850234	EU85009	EU85026
Monocotyledons	Asparagaceae	*Maianthemum*	*Maianthemum bifolium*	MengY-n280	JF977019	JN045443	JF942499	JF954592
Monocotyledons	Asparagaceae	*Maianthemum*	*Maianthemum atropurpureum*	MengY-z11	JF977013	JN045435	JF942494	JF954586

Monocotyledons	Asparagaceae	*Maianthemum*	*Maianthemum atropurpureum*	MengY-n208	JF977017	JN045439	JF942495	JF954589
Monocotyledons	Asparagaceae	*Maianthemum*	*Maianthemum atropurpureum*	MengY-z13	JF977012	JN045434	JF942493	JF954585
Monocotyledons	Asparagaceae	*Maianthemum*	*Maianthemum atropurpureum*	Nie_&_Meng_309	EU850016	EU850223	EU85008 2	EU85025 5
Monocotyledons	Poaceae	*Kengyilia*	*Kengyilia thoroldiana*	ZYH-2884	JF976751	JN045122	JF942193	JF954283
Monocotyledons	Poaceae	*Kengyilia*	*Kengyilia thoroldiana*	ZYH-2888	JF976750	JN045121	JF942192	JF954282
Monocotyledons	Poaceae	*Kengyilia*	*Kengyilia thoroldiana*	PI531686	EF011703	HQ652814	HQ65272	HQ65264
Monocotyledons	Poaceae	*Kengyilia*	*Kengyilia thoroldiana*	ZYH-2878	JF976752	JN045123	JF942194	JF954284
Monocotyledons	Poaceae	*Kengyilia*	*Kengyilia stenachyra*	ZYH-2128	JF976749	JN045120	JF942191	JF954281
Monocotyledons	Poaceae	*Kengyilia*	*Kengyilia stenachyra*	W622128	EF011702	HQ652820	HQ65273	HQ65265
Monocotyledons	Poaceae	*Kengyilia*	*Kengyilia stenachyra*	ZYH-2305	JF976748	JN045119	JF942190	JF954280
Monocotyledons	Poaceae	*Kengyilia*	*Kengyilia stenachyra*	ZYH-3122	JF976747	JN045118	JF942189	JF954279
Monocotyledons	Poaceae	*Kengyilia*	*Kengyilia rigidula*	ZYH-3111	JF976746	JN045117	JF942188	JF954278
Monocotyledons	Poaceae	*Kengyilia*	*Kengyilia rigidula*	W622130	EF011701	HQ652830	HQ65273	HQ65265
Monocotyledons	Poaceae	*Kengyilia*	*Kengyilia rigidula*	ZYH-3127	JF976744	JN045115	JF942186	JF954276
Monocotyledons	Poaceae	*Kengyilia*	*Kengyilia rigidula*	ZYH-3113	JF976745	JN045116	JF942187	JF954277
Monocotyledons	Poaceae	*Kengyilia*	*Kengyilia rigidula*	ZY3113	AY740887	HQ221849	HQ65275	HQ65267
Monocotyledons	Poaceae	*Kengyilia*	*Kengyilia mutica*	ZYH-2875	JF976743	JN045114	JF942185	JF954275
Monocotyledons	Poaceae	*Kengyilia*	*Kengyilia mutica*	ZYH-2889	JF976742	JN045113	JF942184	JF954274
Monocotyledons	Poaceae	*Kengyilia*	*Kengyilia mutica*	Y2873	EF011707	HQ652867	HQ65273	HQ65265
Monocotyledons	Poaceae	*Kengyilia*	*Kengyilia melanthera*	ZYH-9509	JF976737	JN045108	JF942179	JF954269
Monocotyledons	Poaceae	*Kengyilia*	*Kengyilia melanthera*	ZYH-2708	JF976739	JN045110	JF942181	JF954271
Monocotyledons	Poaceae	*Kengyilia*	*Kengyilia melanthera*	ZYH-3044	JF976738	JN045109	JF942180	JF954270
Monocotyledons	Poaceae	*Kengyilia*	*Kengyilia longiglumis*	ZY3119	EF011697	HQ652863	HQ65275	HQ65267
Monocotyledons	Poaceae	*Kengyilia*	*Kengyilia longiglumis*	ZYH-3104	JF976736	JN045107	JF942178	JF954268
Monocotyledons	Poaceae	*Kengyilia*	*Kengyilia longiglumis*	ZYH-3119	JF976735	JN045106	JF942177	JF954267
Monocotyledons	Poaceae	*Kengyilia*	*Kengyilia longiglumis*	ZYH-3121	JF976734	JN045105	JF942176	JF954266
Monocotyledons	Poaceae	*Kengyilia*	*Kengyilia laxiflora*	ZYH-8242	JF976733	JN045104	JF942175	JF954265
Monocotyledons	Poaceae	*Kengyilia*	*Kengyilia laxiflora*	ZYH-8243	JF976732	JN045103	JF942174	JF954264
Monocotyledons	Poaceae	*Kengyilia*	*Kengyilia kokonorica*	Y2880	EF011706	HQ652857	HQ65274	HQ65266
Monocotyledons	Poaceae	*Kengyilia*	*Kengyilia kokonorica*	ZYH-2880	JF976729	JN045099	JF942171	JF954260
Monocotyledons	Poaceae	*Kengyilia*	*Kengyilia kokonorica*	ZYH-2980	JF976728	JN045098	JF942170	JF954259
Monocotyledons	Poaceae	*Kengyilia*	*Kengyilia kokonorica*	ZYH-2870	JF976731	JN045101	JF942173	JF954262
Monocotyledons	Poaceae	*Kengyilia*	*Kengyilia kokonorica*	ZYH-2872	JF976730	JN045100	JF942172	JF954261
Monocotyledons	Poaceae	*Kengyilia*	*Kengyilia hirsuta*	ZYH-2368	JF976726	JN045096	JF942168	JF954257
Monocotyledons	Poaceae	*Kengyilia*	*Kengyilia hirsuta*	ZYH-1618	JF976727	JN045097	JF942169	JF954258
Monocotyledons	Poaceae	*Kengyilia*	*Kengyilia hirsuta*	ZYH-3068	JF976725	JN045095	JF942167	JF954256
Monocotyledons	Poaceae	*Kengyilia*	*Kengyilia grandiglumis*	ZYH-2853	JF976724	JN045094	JF942166	JF954255

Monocotyledons	Poaceae	*Kengyilia*	*Kengyilia grandiglumis*	ZYH-2857	JF976722	JN045092	JF942164	JF954253
Monocotyledons	Poaceae	*Kengyilia*	*Kengyilia grandiglumis*	ZYH-2856	JF976723	JN045093	JF942165	JF954254
Monocotyledons	Poaceae	*Kengyilia*	*Kengyilia gobicola*	Y9503	EF015600	HQ652850	HQ65276	HQ65268
Monocotyledons	Poaceae	*Kengyilia*	*Kengyilia gobicola*	ZYH-9502	JF976721	JN045091	JF942163	JF954252
Monocotyledons	Poaceae	*Kengyilia*	*Kengyilia gobicola*	ZYH-9503	JF976720	JN045090	JF942162	JF954251
Monocotyledons	Poaceae	*Kengyilia*	*Kengyilia geminata*	ZYH-3096	JF976719	JN045089	JF942161	JF954250
Monocotyledons	Poaceae	*Kengyilia*	*Kengyilia geminata*	ZYH-3097	JF976718	JN045088	JF942160	JF954249
Monocotyledons	Poaceae	*Kengyilia*	*Kengyilia batalinii*	PI565002	EF011693	HQ652846	HQ65275	HQ65268
Monocotyledons	Poaceae	*Kengyilia*	*Kengyilia batalinii*	ZYH-7361	JF976715	JN045085	JF942157	JF954246
Monocotyledons	Poaceae	*Kengyilia*	*Kengyilia batalinii*	ZYH-5002	JF976716	JN045086	JF942158	JF954247
Monocotyledons	Poaceae	*Kengyilia*	*Kengyilia batalinii*	ZYH-1563	JF976717	JN045087	JF942159	JF954248
Monocotyledons	Poaceae	*Kengyilia*	*Kengyilia alatavica*	ZYH-1562	JF976714	JN045084	JF942156	JF954245
Monocotyledons	Poaceae	*Kengyilia*	*Kengyilia alatavica*	PI565001	EF011694	HQ652842	HQ65275	HQ65267
Monocotyledons	Poaceae	*Kengyilia*	*Kengyilia alatavica*	ZYH-5001	JF976713	JN045083	JF942155	JF954244
Monocotyledons	Araceae	*Arisaema*	*Arisaema flavum*	D1472	JF975899	JN043994	JF940894	JF953250
Monocotyledons	Araceae	*Arisaema*	*Arisaema flavum*	D1012	JF975900	JN043995	JF940896	JF953251
Monocotyledons	Araceae	*Arisaema*	*Arisaema erubescens*	D300	JF975892	JN043992	JF940887	JF953245
Monocotyledons	Araceae	*Arisaema*	*Arisaema erubescens*	A65	JF975897	JN043993	JF940892	JF953249
Monocotyledons	Zingiberaceae	*Alpinia*	*Alpinia zerumbet*	PS0532MT04	GU180368	GU180445	GU18054	GU18041
Monocotyledons	Zingiberaceae	*Alpinia*	*Alpinia zerumbet*	PS0532MT03	GU180367	GU180444	GU18053	GU18041
Monocotyledons	Zingiberaceae	*Alpinia*	*Alpinia officinarum*	PS0519MT01	GQ434443	GU180427	GU18051	GU18039
Monocotyledons	Zingiberaceae	*Alpinia*	*Alpinia officinarum*	PS0519MT04	GU180360	GU180428	GU18051	GU18039
Monocotyledons	Zingiberaceae	*Alpinia*	*Alpinia galanga*	PS0515MT05	GQ434441	GQ43504	GQ43640	GU18038
Monocotyledons	Zingiberaceae	*Alpinia*	*Alpinia galanga*	PS0515MT04	GU180358	GU180424	GU18051	GU18038
Monocotyledons	Zingiberaceae	*Alpinia*	*Alpinia galanga*	PS0515MT03	GU180357	GQ43504	GU18051	GU18038
Monocotyledons	Zingiberaceae	*Alpinia*	*Alpinia galanga*	PS0515MT02	GU180356	GQ43503	GU18051	GU18038
Monocotyledons	Amaryllidaceae	*Allium*	*Allium przewalskianum*	Wei_X-Q_WXQ2009072702	HQ690282	HQ690381	HQ69050	HQ69033
Monocotyledons	Amaryllidaceae	*Allium*	*Allium przewalskianum*	Wei_X-Q_2009072802	HQ690281	HQ690380	HQ69050	HQ69032
Monocotyledons	Amaryllidaceae	*Allium*	*Allium przewalskianum*	Wang_C-B_09098	HQ690568	HQ690633	HQ69045	HQ69071
Monocotyledons	Amaryllidaceae	*Allium*	*Allium przewalskianum*	Wei_X-Q_WXQ2009072505	HQ690283	HQ690382	HQ69050	HQ69033
Monocotyledons	Alismataceae	*Alisma*	*Alisma plantago-aquatica*	WQF2083	JF975838	JN043783	JF940699	JF953060
Monocotyledons	Alismataceae	*Alisma*	*Alisma plantago-aquatica*	WQF2086	JF975837	JN043782	JF940698	JF953059
Monocotyledons	Alismataceae	*Alisma*	*Alisma plantago-aquatica*	WQF2147	JF975833	JN043778	JF940694	JF953058
Monocotyledons	Alismataceae	*Alisma*	*Alisma gramineum*	WQF2160	JF975831	JN043776	JF940692	JF953056
Monocotyledons	Alismataceae	*Alisma*	*Alisma gramineum*	WQF2053	JF975832	JN043777	JF940693	JF953057
