# Supplementary material for: Utility of the trnH–psbA Intergenic Spacer Region and Its Combinations as Plant DNA Barcodes: A Meta-Analysis
Source: PLoS One. 2012 Nov 14;7(11):e48833. doi: 10.1371/journal.pone.0048833 (PMC3498263; doi:10.1371/journal.pone.0048833)
Supplement: Table S5 — Percentages of species with inversions in different families. (PDF) [file pone.0048833.s005.pdf]

Table S5. Percentages of species with inversions in different families.

Family	No. of species	No. of sequences	No. of species having inversions in their sequences	Percentage of species having inversions (%)
Heliconiaceae	2	4	2	100.0
Melianthaceae	3	10	2	66.7
Podocarpaceae	2	13	1	50.0
Papaveraceae	2	5	1	50.0
Loranthaceae	10	37	5	50.0
Hypoxidaceae	2	123	1	50.0
Stemonaceae	2	5	1	50.0
Arecaceae	19	66	8	42.1
Saxifragaceae	10	102	4	40.0
Polytrichaceae	3	12	1	33.3
Paeoniaceae	6	36	2	33.3
Gesneriaceae	18	45	6	33.3
Lamiaceae	100	429	30	30.0
Hyacinthaceae	10	59	3	30.0
Oleaceae	54	308	15	27.8
Gentianaceae	19	56	5	26.3
Onagraceae	4	11	1	25.0
Melanthiaceae	25	132	6	24.0
Ranunculaceae	65	363	13	20.0
Brassicaceae	20	84	4	20.0
Melastomataceae	5	15	1	20.0
Araceae	26	97	5	19.2
Rutaceae	11	47	2	18.2
Bromeliaceae	39	94	7	17.9
Hydrocharitaceae	6	26	1	16.7
Caryophyllaceae	19	247	3	15.8
Meliaceae	7	32	1	14.3
Polygonaceae	29	148	4	13.8
Apiaceae	29	103	4	13.8
Crassulaceae	9	32	1	11.1
Moraceae	30	502	3	10.0
Potamogetonaceae	10	25	1	10.0
Cephalotaxaceae	10	59	1	10.0
Tamaricaceae	11	56	1	9.1
Fagaceae	23	173	2	8.7
Malvaceae	59	232	5	8.5
Loasaceae	24	88	2	8.3
Asparagaceae	28	142	2	7.1
Phyllanthaceae	14	53	1	7.1
Plantaginaceae	43	720	3	7.0
Rosaceae	130	580	9	6.9
Caprifoliaceae	15	73	1	6.7
Iridaceae	31	128	2	6.5
Fabaceae	130	515	8	6.2
Solanaceae	82	746	5	6.1
Orobanchaceae	121	508	7	5.8
Pinaceae	62	359	3	4.8
Ericaceae	145	785	7	4.8
Primulaceae	102	361	4	3.9
Begoniaceae	29	144	1	3.4
Rubiaceae	59	199	2	3.4
Vitaceae	92	423	3	3.3
Orchidaceae	188	488	6	3.2

Celastraceae	35	232	1	2.9
Poaceae	110	494	3	2.7
Asteraceae	282	1654	4	1.4
Cucurbitaceae	84	475	1	1.2
Dicranaceae	3	64	0	0.0
Mniaceae	3	9	0	0.0
Polypodiaceae	3	9	0	0.0
Cupressaceae	15	73	0	0.0
Ephedraceae	6	15	0	0.0
Betulaceae	47	205	0	0.0
Amaranthaceae	12	77	0	0.0
Cactaceae	30	117	0	0.0
Rhamnaceae	2	7	0	0.0
Passifloraceae	8	41	0	0.0
Salicaceae	25	59	0	0.0
Sapotaceae	17	45	0	0.0
Myrtaceae	13	34	0	0.0
Combretaceae	6	16	0	0.0
Santalaceae	2	8	0	0.0
Euphorbiaceae	5	13	0	0.0
Linaceae	3	15	0	0.0
Anacardiaceae	3	9	0	0.0
Burseraceae	23	375	0	0.0
Geraniaceae	4	10	0	0.0
Oxalidaceae	5	31	0	0.0
Araliaceae	34	124	0	0.0
Apocynaceae	19	51	0	0.0
Convolvulaceae	8	69	0	0.0
Acanthaceae	7	24	0	0.0
Adoxaceae	22	99	0	0.0
Aquifoliaceae	13	44	0	0.0
Proteaceae	2	6	0	0.0
Campanulaceae	12	40	0	0.0
Platanaceae	3	16	0	0.0
Plumbaginaceae	3	57	0	0.0
Alismataceae	10	51	0	0.0
Cyperaceae	24	66	0	0.0
Musaceae	17	72	0	0.0
Zingiberaceae	79	297	0	0.0
Amaryllidaceae	15	36	0	0.0
Dioscoreaceae	31	197	0	0.0
Liliaceae	51	258	0	0.0
Smilacaceae	3	10	0	0.0
Pandanaceae	2	5	0	0.0
Typhaceae	2	6	0	0.0
Commelinaceae	3	7	0	0.0
Sphagnaceae	2	4	0	0.0
Pteridaceae	21	70	0	0.0
Juncaceae	2	5	0	0.0
Juglandaceae	5	11	0	0.0
Ebenaceae	3	7	0	0.0
Styracaceae	2	5	0	0.0
Symplocaceae	5	204	0	0.0
Boraginaceae	2	48	0	0.0
Verbenaceae	2	4	0	0.0
Chrysobalanaceae	6	14	0	0.0

Grossulariaceae	10	32	0	0.0
Hydrangeaceae	13	36	0	0.0
Violaceae	7	14	0	0.0
Taxaceae	15	68	0	0.0
Araucariaceae	16	46	0	0.0
Balsaminaceae	2	4	0	0.0
Cymodoceaceae	2	13	0	0.0
Elaeagnaceae	2	10	0	0.0
Elaeocarpaceae	2	4	0	0.0
Eriocaulaceae	2	6	0	0.0
Loganiaceae	4	13	0	0.0
Nothofagaceae	8	47	0	0.0
Theaceae	19	65	0	0.0
Xanthorrhoeaceae	9	29	0	0.0
Zosteraceae	3	8	0	0.0
Dryopteridaceae	26	64	0	0.0
Hymenophyllaceae	9	34	0	0.0
Lindsaeaceae	24	56	0	0.0
Lomariopsidaceae	2	4	0	0.0
Brachytheciaceae	4	12	0	0.0
Thymelaeaceae	5	56	0	0.0
Colchicaceae	25	61	0	0.0
Berberidaceae	14	83	0	0.0
Aspleniaceae	6	18	0	0.0
Hamamelidaceae	9	61	0	0.0
Cornaceae	13	46	0	0.0
Acoraceae	4	44	0	0.0
Zygophyllaceae	8	122	0	0.0
Orthotrichaceae	12	41	0	0.0
Hypnaceae	4	9	0	0.0
Grimmiaceae	17	116	0	0.0
Caryocaraceae	4	104	0	0.0
Stachyuraceae	3	10	0	0.0
Altingiaceae	4	33	0	0.0
Nitrariaceae	6	23	0	0.0
Ptychomniaceae	4	10	0	0.0
Woodsiaceae	4	20	0	0.0
Hypericaceae	2	5	0	0.0
Lygodiaceae	3	17	0	0.0
Aceraceae	19	445	0	0.0
