# Supplementary material for: Utility of the trnH–psbA Intergenic Spacer Region and Its Combinations as Plant DNA Barcodes: A Meta-Analysis
Source: PLoS One. 2012 Nov 14;7(11):e48833. doi: 10.1371/journal.pone.0048833 (PMC3498263; doi:10.1371/journal.pone.0048833)
Supplement: Table S6 — Percentages of species with inversions in different genera. (PDF) [file pone.0048833.s006.pdf]

Table S6. Percentages of species with inversions in different genera.

Genus	No. of species	No. of sequences	No. of species having inversions in their sequences	Percentage of species having inversions (%)
Heliconia	2	4	2	100.0
Gentianopsis	3	9	3	100.0
Helixanthera	2	7	2	100.0
Wallichia	2	5	2	100.0
Cristaria	2	4	2	100.0
Mentha	9	85	7	77.8
Chaenomeles	3	10	2	66.7
Melianthus	3	10	2	66.7
Conradina	6	20	4	66.7
Origanum	5	22	3	60.0
Thymus	5	25	3	60.0
Podocarpus	2	13	1	50.0
Papaver	2	5	1	50.0
Capsella	4	13	2	50.0
Euonymus	2	29	1	50.0
Thinopyrum	2	37	1	50.0
Cymbidium	2	4	1	50.0
Eranthis	4	41	2	50.0
Schizolobium	2	12	1	50.0
Pitcairnia	2	4	1	50.0
Cardamine	4	15	2	50.0
Pauridia	2	123	1	50.0
Stemona	2	5	1	50.0
Arenga	8	30	4	50.0
Bistorta	2	12	1	50.0
Oxyria	2	56	1	50.0
Wolffia	4	18	2	50.0
Brosimum	2	212	1	50.0
Macrosolen	2	6	1	50.0
Scurrula	2	5	1	50.0
Chamerion	2	7	1	50.0
Phlomis	2	27	1	50.0
Caesalpinia	7	75	3	42.9
Festuca	5	20	2	40.0
Caryota	5	21	2	40.0
Mitella	10	102	4	40.0
Clinopodium	5	13	2	40.0
Fraxinus	37	213	14	37.8
Silene	8	197	3	37.5
Cyrtandra	16	41	6	37.5
Aechmea	14	36	5	35.7
Polytrichum	3	12	1	33.3
Coptis	6	39	2	33.3
Ranunculus	6	15	2	33.3
Paeonia	6	36	2	33.3
Billbergia	3	8	1	33.3
Valeriana	3	13	1	33.3
Schotia	6	49	2	33.3
Salvia	18	49	6	33.3
Aeonium	3	6	1	33.3
Angelica	3	9	1	33.3
Hypochaeris	3	88	1	33.3
Melastoma	3	10	1	33.3
Carapa	3	18	1	33.3
Isodon	12	55	4	33.3
Aconitum	22	170	7	31.8
Paris	16	81	5	31.3
Hyacinthoides	10	59	3	30.0
Citrus	7	36	2	28.6

Lemna	7	29	2	28.6
Rubus	7	39	2	28.6
Canavalia	4	15	1	25.0
Pyracantha	4	23	1	25.0
Cayratia	4	15	1	25.0
Taxillus	4	19	1	25.0
Agalinis	27	90	6	22.2
Veronica	10	70	2	20.0
Asparagus	5	29	1	20.0
Polygonum	5	19	1	20.0
Ampelopsis	10	36	2	20.0
Wolffiella	5	20	1	20.0
Petunia	12	507	2	16.7
Najas	6	26	1	16.7
Gentiana	6	14	1	16.7
Heracleum	6	17	1	16.7
Peucedanum	6	19	1	16.7
Veratrum	6	45	1	16.7
Althaea	6	23	1	16.7
Cliffortia	13	26	2	15.4
Polygonatum	7	31	1	14.3
Malva	14	72	2	14.3
Encelia	7	20	1	14.3
Potentilla	15	89	2	13.3
Castanopsis	15	54	2	13.3
Oncidium	39	93	5	12.8
Osmorhiza	8	31	1	12.5
Oldenlandia	9	39	1	11.1
Pinus	20	102	2	10.0
Potamogeton	10	25	1	10.0
Lycium	10	32	1	10.0
Swertia	10	33	1	10.0
Cephalotaxus	10	59	1	10.0
Blumea	10	22	1	10.0
Synthyris	10	549	1	10.0
Myricaria	11	56	1	9.1
Solanum	23	85	2	8.7
Mentzelia	24	88	2	8.3
Persicaria	12	37	1	8.3
Hedyotis	13	54	1	7.7
Primula	54	205	4	7.4
Ficus	28	290	2	7.1
Phyllanthus	14	53	1	7.1
Rhododendron	101	351	7	6.9
Ligustrum	15	86	1	6.7
Iris	31	128	2	6.5
Picea	21	123	1	4.8
Sinosenecio	24	76	1	4.2
Inga	27	84	1	3.7
Begonia	29	144	1	3.4
Momordica	30	94	1	3.3
Pedicularis	83	301	1	1.2
Dicranum	3	64	0	0.0
Plagiomnium	3	9	0	0.0
Dryopteris	26	64	0	0.0
Abies	21	134	0	0.0
Ephedra	6	15	0	0.0
Betula	4	13	0	0.0
Quercus	4	102	0	0.0
Alnus	23	110	0	0.0
Chenopodium	4	31	0	0.0
Amaranthus	5	37	0	0.0

Dianthus	9	46	0	0.0
Cerastium	2	4	0	0.0
Vitis	31	243	0	0.0
Parthenocissus	5	15	0	0.0
Rhamnus	2	7	0	0.0
Rumex	5	14	0	0.0
Rheum	3	10	0	0.0
Gossypium	4	37	0	0.0
Bryonia	10	145	0	0.0
Trichosanthes	9	38	0	0.0
Passiflora	8	41	0	0.0
Populus	5	13	0	0.0
Brassica	2	5	0	0.0
Raphanus	2	9	0	0.0
Manilkara	3	8	0	0.0
Fragaria	4	10	0	0.0
Malus	3	7	0	0.0
Prunus	19	89	0	0.0
Rosa	17	41	0	0.0
Parnassia	33	203	0	0.0
Ribes	10	32	0	0.0
Acacia	17	67	0	0.0
Albizia	3	8	0	0.0
Leucaena	5	19	0	0.0
Trifolium	2	8	0	0.0
Wisteria	5	16	0	0.0
Eucalyptus	3	7	0	0.0
Linum	3	15	0	0.0
Bursera	15	354	0	0.0
Acer	19	445	0	0.0
Geranium	4	10	0	0.0
Oxalis	5	31	0	0.0
Hydrocotyle	4	29	0	0.0
Hedera	6	15	0	0.0
Panax	7	33	0	0.0
Capsicum	12	42	0	0.0
Datura	3	20	0	0.0
Hyoscyamus	2	4	0	0.0
Convolvulus	5	61	0	0.0
Leonurus	2	6	0	0.0
Scutellaria	5	28	0	0.0
Antirrhinum	19	65	0	0.0
Sambucus	5	29	0	0.0
Viburnum	17	70	0	0.0
Ambrosia	2	8	0	0.0
Arctium	2	6	0	0.0
Artemisia	3	8	0	0.0
Helianthus	2	5	0	0.0
Lactuca	3	9	0	0.0
Ligularia	13	41	0	0.0
Arnica	9	18	0	0.0
Cornus	6	16	0	0.0
Nyssa	7	30	0	0.0
Ilex	13	44	0	0.0
Lobelia	4	10	0	0.0
Hamamelis	4	15	0	0.0
Liquidambar	4	33	0	0.0
Platanus	3	16	0	0.0
Alocasia	2	7	0	0.0
Colocasia	2	4	0	0.0
Acorus	4	44	0	0.0
Spirodela	2	10	0	0.0

Oryza	2	8	0	0.0
Panicum	2	4	0	0.0
Poa	3	11	0	0.0
Setaria	2	4	0	0.0
Bambusa	7	15	0	0.0
Cenchrus	3	6	0	0.0
Cyperus	6	13	0	0.0
Musa	17	72	0	0.0
Dioscorea	25	139	0	0.0
Allium	3	12	0	0.0
Lilium	2	28	0	0.0
Pandanus	2	5	0	0.0
Typha	2	6	0	0.0
Commelina	3	7	0	0.0
Carpinus	8	31	0	0.0
Echinodorus	8	39	0	0.0
Epilobium	2	4	0	0.0
Halodule	2	13	0	0.0
Juniperus	10	54	0	0.0
Sabal	2	4	0	0.0
Achillea	10	62	0	0.0
Ardisia	2	5	0	0.0
Carex	18	53	0	0.0
Cassiope	4	14	0	0.0
Chrysanthemum	7	46	0	0.0
Chrysophyllum	3	7	0	0.0
Colchicum	19	46	0	0.0
Corylus	4	9	0	0.0
Cupressus	5	19	0	0.0
Diospyros	3	7	0	0.0
Enkianthus	2	8	0	0.0
Erica	3	213	0	0.0
Eupatorium	3	9	0	0.0
Gaultheria	29	184	0	0.0
Gazania	10	20	0	0.0
Juncus	2	5	0	0.0
Ostrya	5	14	0	0.0
Ruellia	7	24	0	0.0
Styrax	2	5	0	0.0
Tragopogon	8	22	0	0.0
Viola	7	14	0	0.0
Sphagnum	2	4	0	0.0
Adiantum	3	6	0	0.0
Pteris	3	9	0	0.0
Lygodium	3	17	0	0.0
Alisma	2	12	0	0.0
Tillandsia	3	6	0	0.0
Agrostis	2	5	0	0.0
Calamagrostis	3	11	0	0.0
Dendrocalamus	2	5	0	0.0
Elymus	4	10	0	0.0
Stipa	2	8	0	0.0
Zizania	3	25	0	0.0
Maianthemum	13	68	0	0.0
Cyananthus	8	30	0	0.0
Juglans	5	11	0	0.0
Senecio	6	18	0	0.0
Lepidium	4	26	0	0.0
Astragalus	4	19	0	0.0
Fagus	4	17	0	0.0
Asclepias	2	6	0	0.0
Glechoma	2	6	0	0.0

Teucrium	4	8	0	0.0
Verbena	2	4	0	0.0
Berberis	14	83	0	0.0
Anemone	7	40	0	0.0
Licania	4	10	0	0.0
Hydrangea	11	32	0	0.0
Amelanchier	4	8	0	0.0
Crataegus	26	192	0	0.0
Photinia	5	11	0	0.0
Sorbaria	3	9	0	0.0
Mandragora	2	7	0	0.0
Galium	2	5	0	0.0
Psychotria	7	18	0	0.0
Amentotaxus	4	19	0	0.0
Taxus	7	39	0	0.0
Haworthia	9	29	0	0.0
Araucaria	16	46	0	0.0
Elaeocarpus	2	4	0	0.0
Strychnos	4	13	0	0.0
Nolana	15	38	0	0.0
Nothofagus	8	47	0	0.0
Plantago	4	36	0	0.0
Zostera	3	8	0	0.0
Crepidomanes	4	12	0	0.0
Asplenium	6	18	0	0.0
Lindsaea	22	52	0	0.0
Odontosoria	2	4	0	0.0
Nephrolepis	2	4	0	0.0
Impatiens	2	4	0	0.0
Santalum	2	8	0	0.0
Phalaenopsis	3	26	0	0.0
Cheilanthes	15	55	0	0.0
Urochloa	2	5	0	0.0
Dendrobium	18	47	0	0.0
Glyceria	12	86	0	0.0
Nassella	5	13	0	0.0
Pseudoroegneria	2	4	0	0.0
Cypripedium	6	13	0	0.0
Bulbophyllum	13	30	0	0.0
Encyclia	2	6	0	0.0
Cattleya	4	8	0	0.0
Hymenophyllum	3	15	0	0.0
Eragrostis	4	13	0	0.0
Ajuga	2	6	0	0.0
Sporobolus	2	4	0	0.0
Ocimum	2	17	0	0.0
Rosmarinus	2	8	0	0.0
Leea	2	5	0	0.0
Salix	18	42	0	0.0
Arctotis	18	38	0	0.0
Carlina	4	8	0	0.0
Centaurea	8	65	0	0.0
Cirsium	4	11	0	0.0
Doniophyton	2	4	0	0.0
Erigeron	3	15	0	0.0
Haplocarpha	4	8	0	0.0
Inula	4	11	0	0.0
Lychnophora	3	214	0	0.0
Saussurea	2	4	0	0.0
Santolina	10	39	0	0.0
Eryngium	2	13	0	0.0
Luculia	2	10	0	0.0

Morinda	6	18	0	0.0
Mussaenda	10	30	0	0.0
Canarium	3	6	0	0.0
Nitraria	3	8	0	0.0
Peganum	3	15	0	0.0
Trichilia	2	7	0	0.0
Cipadessa	2	7	0	0.0
Tacca	6	58	0	0.0
Castilleja	3	47	0	0.0
Limonium	3	57	0	0.0
Suaeda	3	9	0	0.0
Delphinium	18	46	0	0.0
Glycyrrhiza	2	7	0	0.0
Dendropanax	14	36	0	0.0
Schefflera	3	11	0	0.0
Pulsatilla	2	12	0	0.0
Andira	2	6	0	0.0
Hippophae	2	10	0	0.0
Hohenbergia	2	4	0	0.0
Nidularium	5	11	0	0.0
Vriesea	10	25	0	0.0
Pleurospermum	4	14	0	0.0
Lonicera	12	60	0	0.0
Erythronium	6	48	0	0.0
Smilax	3	10	0	0.0
Trillium	3	6	0	0.0
Taraxacum	2	7	0	0.0
Hypnum	4	9	0	0.0
Torreya	4	10	0	0.0
Sonchus	3	15	0	0.0
Arisaema	4	9	0	0.0
Kengyilia	15	101	0	0.0
Corylopsis	3	10	0	0.0
Pieris	6	15	0	0.0
Piptatherum	2	5	0	0.0
Alstonia	2	6	0	0.0
Orthotrichum	10	33	0	0.0
Brachythecium	4	12	0	0.0
Lamium	16	46	0	0.0
Chamaecrista	2	4	0	0.0
Dalbergia	8	31	0	0.0
Lespedeza	4	8	0	0.0
Senna	11	29	0	0.0
Vitex	3	8	0	0.0
Symplocos	5	204	0	0.0
Hypericum	2	5	0	0.0
Sanguisorba	2	6	0	0.0
Androcymbium	6	15	0	0.0
Lloydia	2	5	0	0.0
Solidago	11	314	0	0.0
Craspedia	2	4	0	0.0
Leucogenes	3	15	0	0.0
Raoulia	2	6	0	0.0
Schima	3	9	0	0.0
Stewartia	16	56	0	0.0
Lysimachia	39	112	0	0.0
Schizophragma	2	4	0	0.0
Leptodermis	8	19	0	0.0
Lavatera	7	19	0	0.0
Fockea	3	9	0	0.0
Vincetoxicum	3	6	0	0.0
Loropetalum	2	36	0	0.0

Hemsleya	21	112	0	0.0
Schistidium	3	11	0	0.0
Alcea	9	18	0	0.0
Deparia	4	20	0	0.0
Digitaria	3	7	0	0.0
Larrea	2	109	0	0.0
Zygophyllum	6	13	0	0.0
Daphne	5	56	0	0.0
Spathelia	4	11	0	0.0
Aspalathus	2	4	0	0.0
Caryocar	4	104	0	0.0
Symphyotrichum	9	24	0	0.0
Ostryopsis	3	28	0	0.0
Cordia	2	48	0	0.0
Couepia	2	4	0	0.0
Gagea	41	177	0	0.0
Lycoris	12	24	0	0.0
Grewia	3	8	0	0.0
Grabowskia	3	11	0	0.0
Aspidosperma	2	4	0	0.0
Pouteria	6	16	0	0.0
Stachyurus	3	10	0	0.0
Draba	2	6	0	0.0
Cistanche	4	23	0	0.0
Malope	2	6	0	0.0
Alpinia	23	83	0	0.0
Ptychomnion	4	10	0	0.0
Boesenbergia	5	11	0	0.0
Kaempferia	19	74	0	0.0
Anthemis	2	4	0	0.0
Tanacetum	8	16	0	0.0
Combretum	6	16	0	0.0
Curcuma	9	61	0	0.0
Croton	5	13	0	0.0
Tupistra	3	14	0	0.0
Mikania	2	10	0	0.0
Gigantochloa	2	8	0	0.0
Opuntia	4	17	0	0.0
Gagnepainia	2	6	0	0.0
Omphalogramma	7	39	0	0.0
Jacquemontia	3	8	0	0.0
Casearia	2	4	0	0.0
Hyphaene	2	6	0	0.0
Grimmia	7	73	0	0.0
Anthyllis	9	21	0	0.0
Psidium	2	4	0	0.0
Caralluma	4	8	0	0.0
Tolpis	5	10	0	0.0
Prosthechea	3	7	0	0.0
Ampelocissus	2	4	0	0.0
Cischweinfia	3	6	0	0.0
Cuitlauzina	3	6	0	0.0
Cyrtochilum	10	25	0	0.0
Fernandezia	2	4	0	0.0
Lockhartia	2	4	0	0.0
Ornithocephalus	3	7	0	0.0
Trichocentrum	3	6	0	0.0
Trichopilia	3	10	0	0.0
Picconia	2	9	0	0.0
Mammillaria	2	4	0	0.0
Paspalum	5	14	0	0.0
Calliandra	2	4	0	0.0

Cissus	8	19	0	0.0
Tetrastigma	30	86	0	0.0
Pfeiffera	6	13	0	0.0
Rhipsalis	11	27	0	0.0
Lepismium	3	8	0	0.0
Comparettia	4	8	0	0.0
Erycina	6	15	0	0.0
Gomesa	20	43	0	0.0
Ionopsis	3	7	0	0.0
Miltoniopsis	2	4	0	0.0
Odontoglossum	2	4	0	0.0
Rhynchostele	3	9	0	0.0
Tolumnia	4	8	0	0.0
Pyrrosia	3	9	0	0.0
Eperua	2	8	0	0.0
Protium	5	15	0	0.0
Caucaea	3	6	0	0.0
Galinsoga	2	5	0	0.0
Melampodium	14	234	0	0.0
Syzygium	3	9	0	0.0
Paepalanthus	2	6	0	0.0
Amomum	21	62	0	0.0
Enceliopsis	2	4	0	0.0
Aronia	3	6	0	0.0
Hoodia	3	12	0	0.0
Rhodiola	6	26	0	0.0
Faurea	2	6	0	0.0
Thladiantha	14	86	0	0.0
Tarasa	6	13	0	0.0
Aulosepalum	4	8	0	0.0
Micropholis	5	14	0	0.0
Rhynchostylis	3	15	0	0.0
Miconia	2	5	0	0.0
Cota	2	6	0	0.0
Palaua	3	7	0	0.0
Searsia	3	9	0	0.0
Elephantopus	2	9	0	0.0
Holcoglossum	7	33	0	0.0
Glossoloma	2	4	0	0.0
Myrcia	5	14	0	0.0
Jarava	5	15	0	0.0
Jacobaea	2	4	0	0.0
Leochilus	2	4	0	0.0
Tripogon	2	10	0	0.0
Polyphlebium	2	7	0	0.0
Mariosousa	2	4	0	0.0
Vachellia	2	17	0	0.0
Pugionium	2	10	0	0.0
Amelichloa	3	17	0	0.0
Soroseris	4	16	0	0.0
Grandiphyllum	2	4	0	0.0
Dasiphora	2	14	0	0.0
Niphotrichum	3	14	0	0.0
Bucklandiella	2	6	0	0.0
Codriophorus	2	12	0	0.0
Pityopsis	5	12	0	0.0
Chaetoseris	2	4	0	0.0
Razafimandimbisonia	2	6	0	0.0
Nyholmiella	2	8	0	0.0
Pterygiella	4	47	0	0.0
Pappostipa	4	23	0	0.0
Pilosocereus	4	48	0	0.0

Nabalus	7	16	0	0.0
Talipariti	3	25	0	0.0
Syncalathium	7	24	0	0.0
Paraholcoglossum	2	9	0	0.0
Tsiorchis	2	9	0	0.0
