# Supplementary material for: Utility of the trnH–psbA Intergenic Spacer Region and Its Combinations as Plant DNA Barcodes: A Meta-Analysis
Source: PLoS One. 2012 Nov 14;7(11):e48833. doi: 10.1371/journal.pone.0048833 (PMC3498263; doi:10.1371/journal.pone.0048833)
Supplement: Table S7 — Percentages of sequences with rps19 insertions in different families. (PDF) [file pone.0048833.s007.pdf]

Table S7. Percentages of sequences with *rps19* insertions in different families.

**Family**	**No. of sequences**	**No. of sequences with *rps19***	**Percentage of sequences with *rps19* insertions (%)**
Poaceae	494	494	100.0
Orchidaceae	488	488	100.0
Asparagaceae	142	142	100.0
Hypoxidaceae	123	123	100.0
Bromeliaceae	94	94	100.0
Arecaceae	66	66	100.0
Hyacinthaceae	59	59	100.0
Amaryllidaceae	36	36	100.0
Xanthorrhoeaceae	29	29	100.0
Commelinaceae	7	7	100.0
Typhaceae	6	6	100.0
Eriocaulaceae	6	6	100.0
Juncaceae	5	5	100.0
Stemonaceae	5	5	100.0
Zingiberaceae	297	294	99.0
Iridaceae	128	120	93.8
Zygophyllaceae	122	109	89.3
Cyperaceae	66	53	80.3
Musaceae	72	54	75.0
Acoraceae	44	33	75.0
Smilacaceae	10	7	70.0
Melanthiaceae	132	87	65.9
Pandanaceae	5	3	60.0
Fagaceae	173	90	52.0
Liliaceae	258	76	29.5
Dioscoreaceae	197	56	28.4
Aquifoliaceae	44	10	22.7
Colchicaceae	61	12	19.7
Melastomataceae	15	1	6.7
Polygonaceae	148	7	4.7
Acanthaceae	24	1	4.2
Apiaceae	103	4	3.9
Grossulariaceae	32	1	3.1
Amaranthaceae	77	1	1.3
Araceae	97	1	1.0
Rosaceae	580	5	0.9
Rubiaceae	199	1	0.5
Lamiaceae	429	2	0.5
Asteraceae	1654	7	0.4
Fabaceae	515	1	0.2
Solanaceae	746	1	0.1
Polytrichaceae	12	0	0.0
Dicranaceae	64	0	0.0
Mniaceae	9	0	0.0
Polypodiaceae	9	0	0.0
Pinaceae	359	0	0.0
Podocarpaceae	13	0	0.0
Cupressaceae	73	0	0.0
Ephedraceae	15	0	0.0
Ranunculaceae	363	0	0.0
Papaveraceae	5	0	0.0
Moraceae	502	0	0.0

Betulaceae	205	0	0.0
Caryophyllaceae	247	0	0.0
Cactaceae	117	0	0.0
Vitaceae	423	0	0.0
Rhamnaceae	7	0	0.0
Malvaceae	232	0	0.0
Cucurbitaceae	475	0	0.0
Begoniaceae	144	0	0.0
Passifloraceae	41	0	0.0
Salicaceae	59	0	0.0
Brassicaceae	84	0	0.0
Sapotaceae	45	0	0.0
Crassulaceae	32	0	0.0
Saxifragaceae	102	0	0.0
Myrtaceae	34	0	0.0
Onagraceae	11	0	0.0
Combretaceae	16	0	0.0
Santalaceae	8	0	0.0
Loranthaceae	37	0	0.0
Euphorbiaceae	13	0	0.0
Linaceae	15	0	0.0
Anacardiaceae	9	0	0.0
Burseraceae	375	0	0.0
Geraniaceae	10	0	0.0
Oxalidaceae	31	0	0.0
Araliaceae	124	0	0.0
Apocynaceae	51	0	0.0
Convolvulaceae	69	0	0.0
Oleaceae	308	0	0.0
Caprifoliaceae	73	0	0.0
Adoxaceae	99	0	0.0
Celastraceae	232	0	0.0
Proteaceae	6	0	0.0
Primulaceae	361	0	0.0
Ericaceae	785	0	0.0
Campanulaceae	40	0	0.0
Platanaceae	16	0	0.0
Plumbaginaceae	57	0	0.0
Alismataceae	51	0	0.0
Heliconiaceae	4	0	0.0
Sphagnaceae	4	0	0.0
Pteridaceae	70	0	0.0
Potamogetonaceae	25	0	0.0
Juglandaceae	11	0	0.0
Ebenaceae	7	0	0.0
Styracaceae	5	0	0.0
Symplocaceae	204	0	0.0
Gentianaceae	56	0	0.0
Boraginaceae	48	0	0.0
Verbenaceae	4	0	0.0
Chrysobalanaceae	14	0	0.0
Hydrangeaceae	36	0	0.0
Rutaceae	47	0	0.0
Violaceae	14	0	0.0
Paeoniaceae	36	0	0.0
Taxaceae	68	0	0.0

Araucariaceae	46	0	0.0
Balsaminaceae	4	0	0.0
Cymodoceaceae	13	0	0.0
Elaeagnaceae	10	0	0.0
Elaeocarpaceae	4	0	0.0
Gesneriaceae	45	0	0.0
Hydrocharitaceae	26	0	0.0
Loganiaceae	13	0	0.0
Nothofagaceae	47	0	0.0
Theaceae	65	0	0.0
Zosteraceae	8	0	0.0
Dryopteridaceae	64	0	0.0
Hymenophyllaceae	34	0	0.0
Lindsaeaceae	56	0	0.0
Lomariopsidaceae	4	0	0.0
Brachytheciaceae	12	0	0.0
Loasaceae	88	0	0.0
Thymelaeaceae	56	0	0.0
Berberidaceae	83	0	0.0
Aspleniaceae	18	0	0.0
Hamamelidaceae	61	0	0.0
Cornaceae	46	0	0.0
Meliaceae	32	0	0.0
Cephalotaxaceae	59	0	0.0
Orthotrichaceae	41	0	0.0
Hypnaceae	9	0	0.0
Tamaricaceae	56	0	0.0
Grimmiaceae	116	0	0.0
Caryocaraceae	104	0	0.0
Melianthaceae	10	0	0.0
Stachyuraceae	10	0	0.0
Altingiaceae	33	0	0.0
Nitrariaceae	23	0	0.0
Orobanchaceae	508	0	0.0
Ptychomniaceae	10	0	0.0
Plantaginaceae	720	0	0.0
Phyllanthaceae	53	0	0.0
Woodsiaceae	20	0	0.0
Hypericaceae	5	0	0.0
Lygodiaceae	17	0	0.0
Aceraceae	445	0	0.0
