# Supplementary material for: Utility of the trnH–psbA Intergenic Spacer Region and Its Combinations as Plant DNA Barcodes: A Meta-Analysis
Source: PLoS One. 2012 Nov 14;7(11):e48833. doi: 10.1371/journal.pone.0048833 (PMC3498263; doi:10.1371/journal.pone.0048833)
Supplement: Table S8 — Percentages of sequences with rps19 insertions in different genera. (PDF) [file pone.0048833.s008.pdf]

Table S8. Percentages of sequences with *rps19* insertions in different genera.

Genus	No. of sequences	No. of sequences with *rps19* insertion	Percentage of sequences with *rps19* insertions (%)
Oryza	8	8	100.0
Panicum	4	4	100.0
Poa	11	11	100.0
Setaria	4	4	100.0
Bambusa	15	15	100.0
Cenchrus	6	6	100.0
Thinopyrum	37	37	100.0
Festuca	20	20	100.0
Allium	12	12	100.0
Asparagus	29	29	100.0
Lilium	28	28	100.0
Caryota	21	21	100.0
Typha	6	6	100.0
Commelina	7	7	100.0
Sabal	4	4	100.0
Carex	53	53	100.0
Juncus	5	5	100.0
Cymbidium	4	4	100.0
Aechmea	36	36	100.0
Billbergia	8	8	100.0
Tillandsia	6	6	100.0
Agrostis	5	5	100.0
Calamagrostis	11	11	100.0
Dendrocalamus	5	5	100.0
Elymus	10	10	100.0
Stipa	8	8	100.0
Zizania	25	25	100.0
Maianthemum	68	68	100.0
Polygonatum	31	31	100.0
Haworthia	29	29	100.0
Phalaenopsis	26	26	100.0
Urochloa	5	5	100.0
Dendrobium	47	47	100.0
Glyceria	86	86	100.0
Nassella	13	13	100.0
Pseudoroegneria	4	4	100.0
Cypripedium	13	13	100.0
Bulbophyllum	30	30	100.0
Encyclia	6	6	100.0
Cattleya	8	8	100.0
Eragrostis	13	13	100.0
Sporobolus	4	4	100.0
Oncidium	93	93	100.0
Hohenbergia	4	4	100.0
Nidularium	11	11	100.0
Pitcairnia	4	4	100.0
Vriesea	25	25	100.0
Erythronium	48	48	100.0
Paris	81	81	100.0
Trillium	6	6	100.0
Kengyilia	101	101	100.0
Piptatherum	5	5	100.0

Digitaria	7	7	100.0
Larrea	109	109	100.0
Pauridia	123	123	100.0
Hyacinthoides	59	59	100.0
Lycoris	24	24	100.0
Stemona	5	5	100.0
Boesenbergia	11	11	100.0
Tupistra	14	14	100.0
Gigantochloa	8	8	100.0
Gagnepainia	6	6	100.0
Arenga	30	30	100.0
Hyphaene	6	6	100.0
Wallichia	5	5	100.0
Prosthechea	7	7	100.0
Cischweinfia	6	6	100.0
Cuitlauzina	6	6	100.0
Cyrtochilum	25	25	100.0
Fernandezia	4	4	100.0
Lockhartia	4	4	100.0
Ornithocephalus	7	7	100.0
Trichocentrum	6	6	100.0
Trichopilia	10	10	100.0
Paspalum	14	14	100.0
Comparettia	8	8	100.0
Erycina	15	15	100.0
Gomesa	43	43	100.0
Ionopsis	7	7	100.0
Miltoniopsis	4	4	100.0
Odontoglossum	4	4	100.0
Rhynchostele	9	9	100.0
Tolumnia	8	8	100.0
Caucaea	6	6	100.0
Paepalanthus	6	6	100.0
Amomum	62	62	100.0
Aulosepalum	8	8	100.0
Rhynchostylis	15	15	100.0
Holcoglossum	33	33	100.0
Jarava	15	15	100.0
Leochilus	4	4	100.0
Tripogon	10	10	100.0
Amelichloa	17	17	100.0
Grandiphyllum	4	4	100.0
Pappostipa	23	23	100.0
Paraholcoglossum	9	9	100.0
Tsiorchis	9	9	100.0
Alpinia	83	82	98.8
Kaempferia	74	73	98.6
Curcuma	61	60	98.4
Iris	128	120	93.8
Quercus	102	90	88.2
Tacca	58	49	84.5
Acorus	44	33	75.0
Musa	72	54	75.0
Smilax	10	7	70.0
Pandanus	5	3	60.0
Androcymbium	15	8	53.3

Rumex	14	6	42.9
Pleurospermum	14	4	28.6
Jacobaea	4	1	25.0
Ilex	44	10	22.7
Galium	5	1	20.0
Arctium	6	1	16.7
Glechoma	6	1	16.7
Trifolium	8	1	12.5
Eupatorium	9	1	11.1
Sorbaria	9	1	11.1
Melastoma	10	1	10.0
Cirsium	11	1	9.1
Colchicum	46	4	8.7
Sonchus	15	1	6.7
Senecio	18	1	5.6
Polygonum	19	1	5.3
Dioscorea	139	7	5.0
Ruellia	24	1	4.2
Lemna	29	1	3.4
Chenopodium	31	1	3.2
Ribes	32	1	3.1
Rosa	41	1	2.4
Prunus	89	2	2.2
Achillea	62	1	1.6
Solanum	85	1	1.2
Mentha	85	1	1.2
Potentilla	89	1	1.1
Citrus	36	0	0.0
Polytrichum	12	0	0.0
Dicranum	64	0	0.0
Plagiomnium	9	0	0.0
Dryopteris	64	0	0.0
Abies	134	0	0.0
Picea	123	0	0.0
Pinus	102	0	0.0
Podocarpus	13	0	0.0
Ephedra	15	0	0.0
Coptis	39	0	0.0
Ranunculus	15	0	0.0
Papaver	5	0	0.0
Ficus	290	0	0.0
Betula	13	0	0.0
Alnus	110	0	0.0
Amaranthus	37	0	0.0
Dianthus	46	0	0.0
Silene	197	0	0.0
Cerastium	4	0	0.0
Vitis	243	0	0.0
Parthenocissus	15	0	0.0
Rhamnus	7	0	0.0
Rheum	10	0	0.0
Gossypium	37	0	0.0
Bryonia	145	0	0.0
Momordica	94	0	0.0
Trichosanthes	38	0	0.0
Begonia	144	0	0.0

Passiflora	41	0	0.0
Populus	13	0	0.0
Brassica	5	0	0.0
Capsella	13	0	0.0
Raphanus	9	0	0.0
Manilkara	8	0	0.0
Fragaria	10	0	0.0
Malus	7	0	0.0
Parnassia	203	0	0.0
Acacia	67	0	0.0
Albizia	8	0	0.0
Canavalia	15	0	0.0
Leucaena	19	0	0.0
Wisteria	16	0	0.0
Eucalyptus	7	0	0.0
Linum	15	0	0.0
Bursera	354	0	0.0
Acer	445	0	0.0
Geranium	10	0	0.0
Oxalis	31	0	0.0
Hydrocotyle	29	0	0.0
Hedera	15	0	0.0
Panax	33	0	0.0
Capsicum	42	0	0.0
Datura	20	0	0.0
Hyoscyamus	4	0	0.0
Petunia	507	0	0.0
Convolvulus	61	0	0.0
Leonurus	6	0	0.0
Scutellaria	28	0	0.0
Antirrhinum	65	0	0.0
Veronica	70	0	0.0
Sambucus	29	0	0.0
Viburnum	70	0	0.0
Ambrosia	8	0	0.0
Artemisia	8	0	0.0
Helianthus	5	0	0.0
Lactuca	9	0	0.0
Ligularia	41	0	0.0
Arnica	18	0	0.0
Cornus	16	0	0.0
Nyssa	30	0	0.0
Euonymus	29	0	0.0
Rhododendron	351	0	0.0
Lobelia	10	0	0.0
Hamamelis	15	0	0.0
Liquidambar	33	0	0.0
Platanus	16	0	0.0
Alocasia	7	0	0.0
Colocasia	4	0	0.0
Spirodela	10	0	0.0
Cyperus	13	0	0.0
Heliconia	4	0	0.0
Carpinus	31	0	0.0
Echinodorus	39	0	0.0
Epilobium	4	0	0.0

Halodule	13	0	0.0
Juniperus	54	0	0.0
Najas	26	0	0.0
Potamogeton	25	0	0.0
Ardisia	5	0	0.0
Cassiope	14	0	0.0
Chrysanthemum	46	0	0.0
Chrysophyllum	7	0	0.0
Corylus	9	0	0.0
Cupressus	19	0	0.0
Diospyros	7	0	0.0
Enkianthus	8	0	0.0
Erica	213	0	0.0
Gaultheria	184	0	0.0
Gazania	20	0	0.0
Ligustrum	86	0	0.0
Ostrya	14	0	0.0
Paeonia	36	0	0.0
Styrax	5	0	0.0
Tragopogon	22	0	0.0
Viola	14	0	0.0
Sphagnum	4	0	0.0
Adiantum	6	0	0.0
Pteris	9	0	0.0
Lygodium	17	0	0.0
Alisma	12	0	0.0
Cyananthus	30	0	0.0
Juglans	11	0	0.0
Lepidium	26	0	0.0
Valeriana	13	0	0.0
Schotia	49	0	0.0
Astragalus	19	0	0.0
Fagus	17	0	0.0
Asclepias	6	0	0.0
Gentiana	14	0	0.0
Salvia	49	0	0.0
Teucrium	8	0	0.0
Verbena	4	0	0.0
Berberis	83	0	0.0
Anemone	40	0	0.0
Licania	10	0	0.0
Aeonium	6	0	0.0
Hydrangea	32	0	0.0
Amelanchier	8	0	0.0
Crataegus	192	0	0.0
Photinia	11	0	0.0
Rubus	39	0	0.0
Lycium	32	0	0.0
Mandragora	7	0	0.0
Psychotria	18	0	0.0
Amentotaxus	19	0	0.0
Taxus	39	0	0.0
Araucaria	46	0	0.0
Elaeocarpus	4	0	0.0
Strychnos	13	0	0.0
Nolana	38	0	0.0

Nothofagus	47	0	0.0
Plantago	36	0	0.0
Zostera	8	0	0.0
Crepidomanes	12	0	0.0
Asplenium	18	0	0.0
Lindsaea	52	0	0.0
Odontosoria	4	0	0.0
Nephrolepis	4	0	0.0
Impatiens	4	0	0.0
Santalum	8	0	0.0
Chaenomeles	10	0	0.0
Cheilanthes	55	0	0.0
Eranthis	41	0	0.0
Mentzelia	88	0	0.0
Hymenophyllum	15	0	0.0
Ajuga	6	0	0.0
Fraxinus	213	0	0.0
Ocimum	17	0	0.0
Origanum	22	0	0.0
Rosmarinus	8	0	0.0
Leea	5	0	0.0
Swertia	33	0	0.0
Salix	42	0	0.0
Mitella	102	0	0.0
Heracleum	17	0	0.0
Angelica	9	0	0.0
Arctotis	38	0	0.0
Carlina	8	0	0.0
Centaurea	65	0	0.0
Doniophyton	4	0	0.0
Erigeron	15	0	0.0
Haplocarpha	8	0	0.0
Inula	11	0	0.0
Lychnophora	214	0	0.0
Saussurea	4	0	0.0
Santolina	39	0	0.0
Eryngium	13	0	0.0
Pedicularis	301	0	0.0
Luculia	10	0	0.0
Morinda	18	0	0.0
Mussaenda	30	0	0.0
Oldenlandia	39	0	0.0
Canarium	6	0	0.0
Nitraria	8	0	0.0
Peganum	15	0	0.0
Trichilia	7	0	0.0
Cipadessa	7	0	0.0
Agalinis	90	0	0.0
Castilleja	47	0	0.0
Limonium	57	0	0.0
Suaeda	9	0	0.0
Delphinium	46	0	0.0
Glycyrrhiza	7	0	0.0
Dendropanax	36	0	0.0
Schefflera	11	0	0.0
Schizolobium	12	0	0.0

Pulsatilla	12	0	0.0
Andira	6	0	0.0
Hippophae	10	0	0.0
Cyrtandra	41	0	0.0
Aconitum	170	0	0.0
Osmorhiza	31	0	0.0
Peucedanum	19	0	0.0
Lonicera	60	0	0.0
Primula	205	0	0.0
Taraxacum	7	0	0.0
Hypnum	9	0	0.0
Thymus	25	0	0.0
Cephalotaxus	59	0	0.0
Torreya	10	0	0.0
Arisaema	9	0	0.0
Veratrum	45	0	0.0
Cardamine	15	0	0.0
Gentianopsis	9	0	0.0
Corylopsis	10	0	0.0
Pieris	15	0	0.0
Alstonia	6	0	0.0
Orthotrichum	33	0	0.0
Brachythecium	12	0	0.0
Lamium	46	0	0.0
Caesalpinia	75	0	0.0
Chamaecrista	4	0	0.0
Dalbergia	31	0	0.0
Lespedeza	8	0	0.0
Senna	29	0	0.0
Vitex	8	0	0.0
Symplocos	204	0	0.0
Hypericum	5	0	0.0
Sanguisorba	6	0	0.0
Hedyotis	54	0	0.0
Hypochaeris	88	0	0.0
Phyllanthus	53	0	0.0
Lloydia	5	0	0.0
Solidago	314	0	0.0
Craspedia	4	0	0.0
Leucogenes	15	0	0.0
Raoulia	6	0	0.0
Schima	9	0	0.0
Stewartia	56	0	0.0
Lysimachia	112	0	0.0
Schizophragma	4	0	0.0
Leptodermis	19	0	0.0
Persicaria	37	0	0.0
Lavatera	19	0	0.0
Fockea	9	0	0.0
Vincetoxicum	6	0	0.0
Loropetalum	36	0	0.0
Hemsleya	112	0	0.0
Schistidium	11	0	0.0
Alcea	18	0	0.0
Deparia	20	0	0.0
Zygophyllum	13	0	0.0

Daphne	56	0	0.0
Spathelia	11	0	0.0
Aspalathus	4	0	0.0
Helixanthera	7	0	0.0
Caryocar	104	0	0.0
Blumea	22	0	0.0
Symphyotrichum	24	0	0.0
Ostryopsis	28	0	0.0
Cordia	48	0	0.0
Couepia	4	0	0.0
Gagea	177	0	0.0
Grewia	8	0	0.0
Grabowskia	11	0	0.0
Aspidosperma	4	0	0.0
Melianthus	10	0	0.0
Pouteria	16	0	0.0
Stachyurus	10	0	0.0
Draba	6	0	0.0
Cistanche	23	0	0.0
Malope	6	0	0.0
Ptychomnion	10	0	0.0
Malva	72	0	0.0
Sinosenecio	76	0	0.0
Anthemis	4	0	0.0
Tanacetum	16	0	0.0
Combretum	16	0	0.0
Croton	13	0	0.0
Mikania	10	0	0.0
Opuntia	17	0	0.0
Omphalogramma	39	0	0.0
Jacquemontia	8	0	0.0
Casearia	4	0	0.0
Castanopsis	54	0	0.0
Grimmia	73	0	0.0
Anthyllis	21	0	0.0
Psidium	4	0	0.0
Caralluma	8	0	0.0
Tolpis	10	0	0.0
Ampelopsis	36	0	0.0
Ampelocissus	4	0	0.0
Picconia	9	0	0.0
Mammillaria	4	0	0.0
Cliffortia	26	0	0.0
Bistorta	12	0	0.0
Oxyria	56	0	0.0
Pyracantha	23	0	0.0
Althaea	23	0	0.0
Calliandra	4	0	0.0
Cayratia	15	0	0.0
Cissus	19	0	0.0
Tetrastigma	86	0	0.0
Pfeiffera	13	0	0.0
Rhipsalis	27	0	0.0
Lepismium	8	0	0.0
Pyrrosia	9	0	0.0
Wolffia	18	0	0.0

Wolffiella	20	0	0.0
Eperua	8	0	0.0
Inga	84	0	0.0
Synthyris	549	0	0.0
Protium	15	0	0.0
Galinsoga	5	0	0.0
Melampodium	234	0	0.0
Syzygium	9	0	0.0
Clinopodium	13	0	0.0
Conradina	20	0	0.0
Encelia	20	0	0.0
Myricaria	56	0	0.0
Enceliopsis	4	0	0.0
Aronia	6	0	0.0
Brosimum	212	0	0.0
Hoodia	12	0	0.0
Carapa	18	0	0.0
Rhodiola	26	0	0.0
Isodon	55	0	0.0
Faurea	6	0	0.0
Thladiantha	86	0	0.0
Tarasa	13	0	0.0
Macrosolen	6	0	0.0
Scurrula	5	0	0.0
Taxillus	19	0	0.0
Micropholis	14	0	0.0
Chamerion	7	0	0.0
Cristaria	4	0	0.0
Miconia	5	0	0.0
Cota	6	0	0.0
Palaua	7	0	0.0
Searsia	9	0	0.0
Phlomis	27	0	0.0
Elephantopus	9	0	0.0
Glossoloma	4	0	0.0
Myrcia	14	0	0.0
Polyphlebium	7	0	0.0
Mariosousa	4	0	0.0
Vachellia	17	0	0.0
Pugionium	10	0	0.0
Soroseris	16	0	0.0
Dasiphora	14	0	0.0
Niphotrichum	14	0	0.0
Bucklandiella	6	0	0.0
Codriophorus	12	0	0.0
Pityopsis	12	0	0.0
Chaetoseris	4	0	0.0
Razafimandimbiso	6	0	0.0
Nyholmiella	8	0	0.0
Pterygiella	47	0	0.0
Pilosocereus	48	0	0.0
Nabalus	16	0	0.0
Talipariti	25	0	0.0
Syncalathium	24	0	0.0
