# Supplementary material for: Utility of the trnH–psbA Intergenic Spacer Region and Its Combinations as Plant DNA Barcodes: A Meta-Analysis
Source: PLoS One. 2012 Nov 14;7(11):e48833. doi: 10.1371/journal.pone.0048833 (PMC3498263; doi:10.1371/journal.pone.0048833)
Supplement: Table S9 — Intra- and interspecific distances of congeneric species in the five major plant taxonomic groups. (PDF) [file pone.0048833.s009.pdf]

Table S9. Intra- and interspecific distances of congeneric species in the five major plant taxonomic groups.

**Taxa**	**Eudicotyledons**	**Monocotyledons**	**Gymnosperms**	**Ferns**	**Mosses**
Theta	0.0145±0.0590	0.0080±0.0216	0.0027±0.0073	0.0045±0.0145	0.0113±0.0245
Coalescent depth	0.0238±0.0871	0.0135±0.0418	0.0070±0.0360	0.0071±0.0267	0.0174±0.0352
All interspecific distance	0.0447±0.1021	0.0283±0.0407	0.0086±0.0171	0.0302±0.0430	0.0206±0.0197
Theta prime	0.0474±0.0736	0.0246±0.0306	0.0151±0.0147	0.0790±0.0959	0.0297±0.0261
Minimum interspecific distance	0.0104±0.0280	0.0068±0.0183	0.0028±0.0080	0.0269±0.0572	0.0108±0.0147
