# Supplementary material for: Utility of the trnH–psbA Intergenic Spacer Region and Its Combinations as Plant DNA Barcodes: A Meta-Analysis
Source: PLoS One. 2012 Nov 14;7(11):e48833. doi: 10.1371/journal.pone.0048833 (PMC3498263; doi:10.1371/journal.pone.0048833)
Supplement: Table S10 — Identification success rates of trnH – psbA using BLAST and BLAST+P distance in selected families with fewer than 20 species. (PDF) [file pone.0048833.s010.pdf]

Table S10. Identification success rates of *trnH–psbA* using BLAST and BLAST+P distance in selected families with fewer than 20 species.

Family	No. of genera	No. of species	No. of samples	BLAST Success (%)	BLAST+ P distance Success (%)
Caryophyllaceae	3	19	247	52.6	69.2
Apocynaceae	7	19	51	49.0	58.8
Arecaceae	5	19	66	51.5	60.6
Gentianaceae	3	19	56	71.4	91.1
Theaceae	2	19	65	43.1	47.7
Aceraceae	1	19	445	51.7	54.2
Gesneriaceae	2	18	45	48.9	57.8
Sapotaceae	4	17	45	48.9	68.9
Musaceae	1	17	72	23.6	51.4
Grimmiaceae	5	17	116	68.1	76.7
Araucariaceae	1	16	46	28.3	34.8
Cupressaceae	2	15	73	67.1	67.1
Caprifoliaceae	2	15	73	76.7	80.8
Amaryllidaceae	2	15	36	38.9	58.3
Taxaceae	3	15	68	50.0	58.8
Berberidaceae	1	14	83	10.8	12.0
Phyllanthaceae	1	14	53	96.2	100.0
Myrtaceae	4	13	34	82.4	88.2
Aquifoliaceae	1	13	44	43.2	70.5
Hydrangeaceae	2	13	36	77.8	83.3
Cornaceae	2	13	46	39.1	43.5
Amaranthaceae	3	12	77	61.0	61.0
Campanulaceae	2	12	40	82.5	85.0
Orthotrichaceae	2	12	41	41.5	51.2
Rutaceae	2	11	47	57.4	76.6
Tamaricaceae	1	11	56	58.9	67.9
Saxifragaceae	1	10	102	61.8	92.2
Loranthaceae	4	10	37	100.0	100.0
Alismataceae	2	10	51	25.5	25.5
Potamogetonaceae	1	10	25	68.0	68.0
Grossulariaceae	1	10	32	65.6	65.6
Hyacinthaceae	1	10	59	81.4	86.4
Cephalotaxaceae	1	10	59	18.6	18.6
Crassulaceae	2	9	32	93.8	96.9
Xanthorrhoeaceae	1	9	29	48.3	48.3
Hymenophyllaceae	3	9	34	100.0	100.0
Hamamelidaceae	3	9	61	63.9	68.9
Passifloraceae	1	8	41	100.0	100.0
Convolvulaceae	2	8	69	27.5	31.9
Nothofagaceae	1	8	47	21.3	21.3
Zygophyllaceae	2	8	122	96.7	96.7
Acanthaceae	1	7	24	33.3	50.0
Violaceae	1	7	14	57.1	100.0
Meliaceae	3	7	32	78.1	90.6
Ephedraceae	1	6	15	40.0	40.0
Combretaceae	1	6	16	93.8	93.8
Chrysobalanaceae	2	6	14	100.0	100.0
Paeoniaceae	1	6	36	55.6	100.0
Hydrocharitaceae	1	6	26	84.6	84.6

Aspleniaceae	1	6	18	100.0	100.0
Nitrariaceae	2	6	23	26.1	82.6
Euphorbiaceae	1	5	13	100.0	100.0
Oxalidaceae	1	5	31	96.8	100.0
Juglandaceae	1	5	11	54.5	54.5
Symplocaceae	1	5	204	84.8	100.0
Thymelaeaceae	1	5	56	91.1	100.0
Melastomataceae	2	5	15	86.7	100.0
Onagraceae	2	4	11	100.0	100.0
Geraniaceae	1	4	10	70.0	100.0
Loganiaceae	1	4	13	84.6	100.0
Brachytheciaceae	1	4	12	83.3	100.0
Acoraceae	1	4	44	75.0	75.0
Hypnaceae	1	4	9	22.2	22.2
Caryocaraceae	1	4	104	39.4	47.1
Altingiaceae	1	4	33	87.9	87.9
Ptychomniaceae	1	4	10	40.0	40.0
Woodsiaceae	1	4	20	100.0	100.0
Polytrichaceae	1	3	12	91.7	100.0
Dicranaceae	1	3	64	100.0	100.0
Mniaceae	1	3	9	100.0	100.0
Polypodiaceae	1	3	9	66.7	66.7
Linaceae	1	3	15	13.3	13.3
Anacardiaceae	1	3	9	0.0	100.0
Platanaceae	1	3	16	0.0	0.0
Plumbaginaceae	1	3	57	12.3	87.7
Smilacaceae	1	3	10	100.0	100.0
Commelinaceae	1	3	7	100.0	100.0
Ebenaceae	1	3	7	28.6	28.6
Zosteraceae	1	3	8	100.0	100.0
Melianthaceae	1	3	10	30.0	100.0
Stachyuraceae	1	3	10	30.0	50.0
Lygodiaceae	1	3	17	100.0	100.0
Podocarpaceae	1	2	13	100.0	100.0
Papaveraceae	1	2	5	100.0	100.0
Rhamnaceae	1	2	7	100.0	100.0
Santalaceae	1	2	8	100.0	100.0
Proteaceae	1	2	6	0.0	0.0
Heliconiaceae	1	2	4	100.0	100.0
Hypoxidaceae	1	2	123	0.0	46.3
Pandanaceae	1	2	5	100.0	100.0
Typhaceae	1	2	6	50.0	50.0
Sphagnaceae	1	2	4	100.0	100.0
Juncaceae	1	2	5	100.0	100.0
Styracaceae	1	2	5	100.0	100.0
Boraginaceae	1	2	48	100.0	100.0
Verbenaceae	1	2	4	100.0	100.0
Balsaminaceae	1	2	4	100.0	100.0
Cymodoceaceae	1	2	13	100.0	100.0
Elaeagnaceae	1	2	10	100.0	100.0
Elaeocarpaceae	1	2	4	100.0	100.0
Eriocaulaceae	1	2	6	100.0	100.0
Lomariopsidaceae	1	2	4	50.0	50.0

Stemonaceae	1	2	5	100.0	100.0
Hypericaceae	1	2	5	100.0	100.0
