# Supplementary material for: Utility of the trnH–psbA Intergenic Spacer Region and Its Combinations as Plant DNA Barcodes: A Meta-Analysis
Source: PLoS One. 2012 Nov 14;7(11):e48833. doi: 10.1371/journal.pone.0048833 (PMC3498263; doi:10.1371/journal.pone.0048833)
Supplement: Table S11 — Identification success rates of trnH – psbA using BLAST and BLAST+P distance in genera with fewer than 20 species. (PDF) [file pone.0048833.s011.pdf]

Table S11. Identification success rates of *trnH–psbA* using BLAST and BLAST+P distance in genera with fewer than 20 species.

Genus	No. of species	No. of samples	BLAST Success (%)	BLAST+ P distance Success (%)
Prunus	19	89	33.7	41.6
Acer	19	445	51.7	54.2
Antirrhinum	19	65	24.6	24.6
Colchicum	19	46	58.7	60.9
Kaempferia	19	74	24.3	51.4
Carex	18	53	3.8	52.8
Salvia	18	49	75.5	81.6
Dendrobium	18	47	95.7	95.7
Salix	18	42	61.9	69.0
Arctotis	18	38	28.9	31.6
Delphinium	18	46	19.6	28.3
Rosa	17	41	22.0	56.1
Acacia	17	67	95.5	100.0
Viburnum	17	70	18.6	54.3
Musa	17	72	23.6	51.4
Araucaria	16	46	28.3	34.8
Cyrtandra	16	41	43.9	53.7
Paris	16	81	22.2	49.4
Lamium	16	46	37.0	60.9
Stewartia	16	56	41.1	44.6
Bursera	15	354	85.6	88.7
Ligustrum	15	86	52.3	54.7
Potentilla	15	89	36.0	49.4
Nolana	15	38	36.8	52.6
Cheilanthes	15	55	43.6	45.5
Kengyilia	15	101	21.8	28.7
Castanopsis	15	54	7.4	40.7
Aechmea	14	36	22.2	33.3
Berberis	14	83	10.8	12.0
Dendropanax	14	36	41.7	41.7
Phyllanthus	14	53	96.2	100.0
Malva	14	72	18.1	44.4
Melampodium	14	234	95.7	95.7
Thladiantha	14	86	58.1	96.5
Ligularia	13	41	22.0	26.8
Ilex	13	44	43.2	70.5
Maianthemum	13	68	32.4	45.6
Bulbophyllum	13	30	90.0	90.0
Hedyotis	13	54	50.0	88.9
Cliffortia	13	26	76.9	88.5
Capsicum	12	42	71.4	71.4
Petunia	12	507	9.9	25.2
Glyceria	12	86	9.3	9.3
Lonicera	12	60	71.7	76.7
Persicaria	12	37	48.6	70.3
Lycoris	12	24	16.7	45.8
Isodon	12	55	49.1	58.2
Hydrangea	11	32	75.0	81.3
Senna	11	29	75.9	100.0

Solidago	11	314	2.2	5.1
Rhipsalis	11	27	55.6	74.1
Myricaria	11	56	58.9	67.9
Bryonia	10	145	64.1	64.1
Ribes	10	32	65.6	65.6
Veronica	10	70	28.6	42.9
Juniperus	10	54	55.6	55.6
Potamogeton	10	25	68.0	68.0
Achillea	10	62	8.1	22.6
Gazania	10	20	25.0	55.0
Lycium	10	32	78.1	84.4
Swertia	10	33	72.7	97.0
Mitella	10	102	61.8	92.2
Santolina	10	39	10.3	10.3
Mussaenda	10	30	56.7	73.3
Vriesea	10	25	20.0	20.0
Cephalotaxus	10	59	18.6	18.6
Orthotrichum	10	33	45.5	57.6
Blumea	10	22	100.0	100.0
Hyacinthoides	10	59	81.4	86.4
Ampelopsis	10	36	30.6	50.0
Cyrtochilum	10	25	76.0	76.0
Synthyris	10	549	19.5	23.7
Dianthus	9	46	13.0	69.6
Trichosanthes	9	38	81.6	81.6
Arnica	9	18	38.9	50.0
Mentha	9	85	49.4	61.2
Haworthia	9	29	48.3	48.3
Oldenlandia	9	39	64.1	92.3
Alcea	9	18	5.6	5.6
Symphyotrichum	9	24	8.3	33.3
Curcuma	9	61	19.7	42.6
Anthyllis	9	21	90.5	100.0
Silene	8	197	60.9	68.5
Passiflora	8	41	100.0	100.0
Carpinus	8	31	45.2	74.2
Echinodorus	8	39	30.8	30.8
Tragopogon	8	22	90.9	95.5
Cyananthus	8	30	76.7	80.0
Nothofagus	8	47	21.3	21.3
Centaurea	8	65	33.8	38.5
Osmorhiza	8	31	35.5	58.1
Dalbergia	8	31	93.5	93.5
Leptodermis	8	19	89.5	100.0
Tanacetum	8	16	12.5	50.0
Arenga	8	30	20.0	26.7
Cissus	8	19	100.0	100.0
Citrus	7	36	52.8	75.0
Panax	7	33	66.7	66.7
Nyssa	7	30	6.7	13.3
Lemna	7	29	79.3	79.3
Bambusa	7	15	26.7	26.7
Chrysanthemum	7	46	21.7	32.6

Ruellia	7	24	33.3	50.0
Viola	7	14	57.1	100.0
Polygonatum	7	31	29.0	29.0
Anemone	7	40	62.5	85.0
Rubus	7	39	94.9	94.9
Psychotria	7	18	88.9	100.0
Taxus	7	39	43.6	59.0
Caesalpinia	7	75	85.3	100.0
Lavatera	7	19	31.6	73.7
Omphalogramma	7	39	82.1	100.0
Grimmia	7	73	80.8	80.8
Encelia	7	20	25.0	45.0
Holcoglossum	7	33	42.4	42.4
Nabalus	7	16	31.3	31.3
Syncalathium	7	24	75.0	87.5
Ephedra	6	15	40.0	40.0
Coptis	6	39	66.7	69.2
Ranunculus	6	15	46.7	60.0
Hedera	6	15	26.7	26.7
Cornus	6	16	100.0	100.0
Cyperus	6	13	100.0	100.0
Najas	6	26	84.6	84.6
Paeonia	6	36	55.6	100.0
Senecio	6	18	61.1	72.2
Schotia	6	49	18.4	49.0
Gentiana	6	14	71.4	71.4
Asplenium	6	18	100.0	100.0
Cypripedium	6	13	100.0	100.0
Heracleum	6	17	23.5	29.4
Morinda	6	18	0.0	5.6
Tacca	6	58	60.3	79.3
Peucedanum	6	19	100.0	100.0
Erythronium	6	48	29.2	31.3
Veratrum	6	45	31.1	31.1
Pieris	6	15	80.0	86.7
Androcymbium	6	15	100.0	100.0
Zygophyllum	6	13	69.2	69.2
Pouteria	6	16	50.0	56.3
Combretum	6	16	93.8	93.8
Althaea	6	23	21.7	52.2
Pfeiffera	6	13	7.7	69.2
Erycina	6	15	100.0	100.0
Conradina	6	20	50.0	65.0
Rhodiola	6	26	92.3	96.2
Tarasa	6	13	69.2	100.0
Amaranthus	5	37	18.9	18.9
Parthenocissus	5	15	46.7	46.7
Rumex	5	14	64.3	64.3
Populus	5	13	84.6	100.0
Leucaena	5	19	10.5	10.5
Wisteria	5	16	68.8	75.0
Oxalis	5	31	96.8	100.0
Convolvulus	5	61	26.2	26.2

Scutellaria	5	28	92.9	100.0
Sambucus	5	29	62.1	96.6
Festuca	5	20	25.0	25.0
Asparagus	5	29	82.8	89.7
Caryota	5	21	81.0	85.7
Cupressus	5	19	100.0	100.0
Ostrya	5	14	42.9	100.0
Juglans	5	11	54.5	54.5
Photinia	5	11	54.5	63.6
Nassella	5	13	23.1	23.1
Origanum	5	22	63.6	68.2
Polygonum	5	19	100.0	100.0
Nidularium	5	11	0.0	0.0
Thymus	5	25	44.0	80.0
Symplocos	5	204	84.8	100.0
Daphne	5	56	91.1	100.0
Boesenbergia	5	11	54.5	100.0
Croton	5	13	100.0	100.0
Tolpis	5	10	60.0	80.0
Paspalum	5	14	42.9	42.9
Wolffiella	5	20	60.0	70.0
Protium	5	15	60.0	100.0
Clinopodium	5	13	100.0	100.0
Micropholis	5	14	71.4	71.4
Myrcia	5	14	85.7	85.7
Jarava	5	15	60.0	60.0
Pityopsis	5	12	0.0	16.7
Betula	4	13	30.8	30.8
Quercus	4	102	93.1	93.1
Chenopodium	4	31	100.0	100.0
Gossypium	4	37	2.7	32.4
Capsella	4	13	53.8	76.9
Fragaria	4	10	10.0	80.0
Canavalia	4	15	6.7	33.3
Geranium	4	10	70.0	100.0
Hydrocotyle	4	29	72.4	100.0
Lobelia	4	10	100.0	100.0
Hamamelis	4	15	0.0	20.0
Liquidambar	4	33	87.9	87.9
Acorus	4	44	75.0	75.0
Cassiope	4	14	35.7	35.7
Corylus	4	9	44.4	55.6
Elymus	4	10	70.0	80.0
Lepidium	4	26	50.0	50.0
Astragalus	4	19	100.0	100.0
Fagus	4	17	23.5	29.4
Teucrium	4	8	75.0	100.0
Licania	4	10	100.0	100.0
Amelanchier	4	8	25.0	50.0
Amentotaxus	4	19	63.2	63.2
Strychnos	4	13	84.6	100.0
Plantago	4	36	63.9	66.7
Crepidomanes	4	12	100.0	100.0

Eranthis	4	41	100.0	100.0
Cattleya	4	8	75.0	100.0
Eragrostis	4	13	100.0	100.0
Carlina	4	8	100.0	100.0
Cirsium	4	11	90.9	90.9
Haplocarpha	4	8	100.0	100.0
Inula	4	11	100.0	100.0
Pleurospermum	4	14	100.0	100.0
Hypnum	4	9	22.2	22.2
Torreya	4	10	50.0	50.0
Arisaema	4	9	33.3	100.0
Cardamine	4	15	80.0	100.0
Brachythecium	4	12	83.3	100.0
Lespedeza	4	8	50.0	62.5
Deparia	4	20	100.0	100.0
Spathelia	4	11	72.7	81.8
Caryocar	4	104	39.4	47.1
Cistanche	4	23	100.0	100.0
Ptychomnion	4	10	40.0	40.0
Opuntia	4	17	0.0	17.6
Caralluma	4	8	50.0	75.0
Pyracantha	4	23	4.3	21.7
Cayratia	4	15	100.0	100.0
Comparettia	4	8	100.0	100.0
Tolumnia	4	8	100.0	100.0
Wolffia	4	18	100.0	100.0
Aulosepalum	4	8	87.5	87.5
Taxillus	4	19	100.0	100.0
Soroseris	4	16	31.3	31.3
Pterygiella	4	47	19.1	19.1
Pappostipa	4	23	52.2	52.2
Pilosocereus	4	48	52.1	52.1
Polytrichum	3	12	100.0	100.0
Dicranum	3	64	100.0	100.0
Plagiomnium	3	9	100.0	100.0
Rheum	3	10	20.0	100.0
Manilkara	3	8	37.5	62.5
Malus	3	7	42.9	100.0
Albizia	3	8	50.0	100.0
Eucalyptus	3	7	71.4	71.4
Linum	3	15	13.3	13.3
Datura	3	20	75.0	75.0
Artemisia	3	8	100.0	100.0
Lactuca	3	9	100.0	100.0
Platanus	3	16	0.0	0.0
Poa	3	11	54.5	54.5
Cenchrus	3	6	50.0	50.0
Allium	3	12	83.3	83.3
Commelina	3	7	100.0	100.0
Chrysophyllum	3	7	14.3	100.0
Diospyros	3	7	28.6	28.6
Erica	3	213	88.3	98.6
Eupatorium	3	9	100.0	100.0

Adiantum	3	6	100.0	100.0
Pteris	3	9	100.0	100.0
Lygodium	3	17	100.0	100.0
Billbergia	3	8	12.5	62.5
Tillandsia	3	6	100.0	100.0
Calamagrostis	3	11	27.3	27.3
Zizania	3	25	8.0	48.0
Valeriana	3	13	100.0	100.0
Aeonium	3	6	100.0	100.0
Sorbaria	3	9	100.0	100.0
Zostera	3	8	100.0	100.0
Phalaenopsis	3	26	46.2	69.2
Chaenomeles	3	10	100.0	100.0
Hymenophyllum	3	15	100.0	100.0
Angelica	3	9	88.9	100.0
Erigeron	3	15	13.3	13.3
Lychnophora	3	214	23.8	100.0
Canarium	3	6	50.0	50.0
Nitraria	3	8	12.5	50.0
Peganum	3	15	33.3	100.0
Castilleja	3	47	10.6	10.6
Limonium	3	57	12.3	87.7
Suaeda	3	9	100.0	100.0
Schefflera	3	11	100.0	100.0
Smilax	3	10	100.0	100.0
Trillium	3	6	100.0	100.0
Sonchus	3	15	33.3	33.3
Gentianopsis	3	9	66.7	100.0
Corylopsis	3	10	30.0	30.0
Vitex	3	8	37.5	75.0
Hypochaeris	3	88	63.6	67.0
Leucogenes	3	15	13.3	20.0
Schima	3	9	55.6	66.7
Fockea	3	9	100.0	100.0
Vincetoxicum	3	6	50.0	50.0
Schistidium	3	11	36.4	36.4
Digitaria	3	7	100.0	100.0
Ostryopsis	3	28	21.4	71.4
Grewia	3	8	37.5	100.0
Grabowskia	3	11	18.2	18.2
Melianthus	3	10	30.0	100.0
Stachyurus	3	10	30.0	50.0
Tupistra	3	14	28.6	42.9
Jacquemontia	3	8	37.5	75.0
Melastoma	3	10	80.0	100.0
Prosthechea	3	7	100.0	100.0
Cischweinfia	3	6	33.3	33.3
Cuitlauzina	3	6	100.0	100.0
Ornithocephalus	3	7	100.0	100.0
Trichocentrum	3	6	33.3	33.3
Trichopilia	3	10	100.0	100.0
Lepismium	3	8	100.0	100.0
Ionopsis	3	7	100.0	100.0

Rhynchostele	3	9	100.0	100.0
Pyrrosia	3	9	66.7	66.7
Caucaea	3	6	33.3	50.0
Syzygium	3	9	77.8	100.0
Aronia	3	6	0.0	16.7
Hoodia	3	12	0.0	0.0
Carapa	3	18	100.0	100.0
Rhynchostylis	3	15	100.0	100.0
Palaua	3	7	14.3	100.0
Searsia	3	9	0.0	100.0
Amelichloa	3	17	0.0	0.0
Niphotrichum	3	14	85.7	85.7
Talipariti	3	25	8.0	8.0
Podocarpus	2	13	100.0	100.0
Papaver	2	5	100.0	100.0
Cerastium	2	4	100.0	100.0
Rhamnus	2	7	100.0	100.0
Brassica	2	5	100.0	100.0
Raphanus	2	9	44.4	44.4
Trifolium	2	8	100.0	100.0
Hyoscyamus	2	4	25.0	25.0
Leonurus	2	6	100.0	100.0
Ambrosia	2	8	12.5	12.5
Arctium	2	6	33.3	33.3
Helianthus	2	5	100.0	100.0
Euonymus	2	29	100.0	100.0
Alocasia	2	7	71.4	85.7
Colocasia	2	4	100.0	100.0
Spirodela	2	10	100.0	100.0
Oryza	2	8	62.5	62.5
Panicum	2	4	75.0	75.0
Setaria	2	4	100.0	100.0
Thinopyrum	2	37	8.1	100.0
Heliconia	2	4	100.0	100.0
Lilium	2	28	100.0	100.0
Pandanus	2	5	100.0	100.0
Typha	2	6	50.0	50.0
Epilobium	2	4	100.0	100.0
Halodule	2	13	100.0	100.0
Sabal	2	4	75.0	75.0
Ardisia	2	5	100.0	100.0
Enkianthus	2	8	100.0	100.0
Juncus	2	5	100.0	100.0
Styrax	2	5	100.0	100.0
Sphagnum	2	4	100.0	100.0
Cymbidium	2	4	100.0	100.0
Alisma	2	12	8.3	8.3
Agrostis	2	5	20.0	20.0
Dendrocalamus	2	5	100.0	100.0
Stipa	2	8	100.0	100.0
Asclepias	2	6	33.3	33.3
Glechoma	2	6	100.0	100.0
Verbena	2	4	100.0	100.0

Mandragora	2	7	100.0	100.0
Galium	2	5	40.0	60.0
Elaeocarpus	2	4	100.0	100.0
Odontosoria	2	4	50.0	50.0
Nephrolepis	2	4	50.0	50.0
Impatiens	2	4	100.0	100.0
Santalum	2	8	100.0	100.0
Urochloa	2	5	100.0	100.0
Pseudoroegneria	2	4	0.0	25.0
Encyclia	2	6	100.0	100.0
Ajuga	2	6	100.0	100.0
Sporobolus	2	4	100.0	100.0
Ocimum	2	17	35.3	52.9
Rosmarinus	2	8	25.0	25.0
Leea	2	5	100.0	100.0
Doniophyton	2	4	100.0	100.0
Saussurea	2	4	100.0	100.0
Eryngium	2	13	100.0	100.0
Luculia	2	10	0.0	0.0
Trichilia	2	7	100.0	100.0
Cipadessa	2	7	0.0	57.1
Glycyrrhiza	2	7	85.7	85.7
Schizolobium	2	12	66.7	100.0
Pulsatilla	2	12	8.3	58.3
Andira	2	6	100.0	100.0
Hippophae	2	10	100.0	100.0
Hohenbergia	2	4	25.0	100.0
Pitcairnia	2	4	50.0	100.0
Taraxacum	2	7	100.0	100.0
Piptatherum	2	5	60.0	100.0
Alstonia	2	6	50.0	100.0
Chamaecrista	2	4	25.0	100.0
Hypericum	2	5	100.0	100.0
Sanguisorba	2	6	100.0	100.0
Lloydia	2	5	100.0	100.0
Craspedia	2	4	0.0	0.0
Raoulia	2	6	0.0	66.7
Schizophragma	2	4	100.0	100.0
Loropetalum	2	36	100.0	100.0
Larrea	2	109	100.0	100.0
Pauridia	2	123	0.0	46.3
Aspalathus	2	4	100.0	100.0
Helixanthera	2	7	100.0	100.0
Cordia	2	48	100.0	100.0
Couepia	2	4	100.0	100.0
Aspidosperma	2	4	100.0	100.0
Stemona	2	5	100.0	100.0
Draba	2	6	100.0	100.0
Malope	2	6	100.0	100.0
Anthemis	2	4	100.0	100.0
Mikania	2	10	0.0	0.0
Gigantochloa	2	8	37.5	37.5
Gagnepainia	2	6	100.0	100.0

Casearia	2	4	100.0	100.0
Hyphaene	2	6	100.0	100.0
Wallichia	2	5	40.0	100.0
Psidium	2	4	100.0	100.0
Ampelocissus	2	4	100.0	100.0
Fernandezia	2	4	100.0	100.0
Lockhartia	2	4	100.0	100.0
Picconia	2	9	100.0	100.0
Mammillaria	2	4	100.0	100.0
Bistorta	2	12	16.7	41.7
Oxyria	2	56	96.4	100.0
Calliandra	2	4	100.0	100.0
Miltoniopsis	2	4	0.0	0.0
Odontoglossum	2	4	100.0	100.0
Eperua	2	8	100.0	100.0
Galinsoga	2	5	0.0	20.0
Paepalanthus	2	6	100.0	100.0
Enceliopsis	2	4	100.0	100.0
Brosimum	2	212	43.4	100.0
Faurea	2	6	0.0	0.0
Macrosolen	2	6	100.0	100.0
Scurrula	2	5	100.0	100.0
Chamerion	2	7	100.0	100.0
Cristaria	2	4	100.0	100.0
Miconia	2	5	100.0	100.0
Cota	2	6	100.0	100.0
Phlomis	2	27	100.0	100.0
Elephantopus	2	9	100.0	100.0
Glossoloma	2	4	100.0	100.0
Jacobaea	2	4	100.0	100.0
Leochilus	2	4	100.0	100.0
Tripogon	2	10	0.0	0.0
Polyphlebium	2	7	100.0	100.0
Mariosousa	2	4	0.0	100.0
Vachellia	2	17	88.2	100.0
Pugionium	2	10	0.0	0.0
Grandiphyllum	2	4	100.0	100.0
Dasiphora	2	14	64.3	78.6
Bucklandiella	2	6	100.0	100.0
Codriophorus	2	12	100.0	100.0
Chaetoseris	2	4	100.0	100.0
Razafimandimbisonia	2	6	66.7	66.7
Nyholmiella	2	8	25.0	25.0
Paraholcoglossum	2	9	100.0	100.0
Tsiorchis	2	9	100.0	100.0
