# Supplementary material for: Utility of the trnH–psbA Intergenic Spacer Region and Its Combinations as Plant DNA Barcodes: A Meta-Analysis
Source: PLoS One. 2012 Nov 14;7(11):e48833. doi: 10.1371/journal.pone.0048833 (PMC3498263; doi:10.1371/journal.pone.0048833)
Supplement: Table S12 — Detailed information on the identification success rate of single markers at the family level and the corresponding statistical test results. (PDF) [file pone.0048833.s012.pdf]

Table S12. Detailed information on the identification success rate of single markers at the family level and the corresponding statistical test results.

Family	N	ITS2 and *trnH-psbA*				*matK* and *trnH-psbA*				*rbcL* and *trnH-psbA*			
		ITS2 success (%)	*trnH-psbA* success (%)	2-Tail	Result	*matK* success (%)	*trnH-psbA* success (%)	2-Tail	Result	*rbcL* success (%)	*trnH-psbA* success (%)	2-Tail	Result
Ranunculaceae	34	44.1	47.1	1	N/S	23.5	47.1	0.0746	N/S	11.8	47.1	0.0029	*trnH-psbA* > *rbcL*
Moraceae	9	100.0	100.0	N/A	N/A	100.0	100.0	N/A	N/A	22.2	100.0	0.0023	*trnH-psbA* > *rbcL*
Betulaceae	20	50.0	65.0	0.5231	N/S	0.0	65.0	<.0001	*trnH-psbA* > *matK*	10.0	65.0	0.0008	*trnH-psbA* > *rbcL*
Amaranthaceae	53	77.4	64.2	0.1998	N/S	75.5	64.2	0.2901	N/S	52.8	64.2	0.3244	N/S
Caryophyllaceae	19	89.5	100.0	0.4865	N/S	89.5	100.0	0.4865	N/S	52.6	100.0	0.0011	*trnH-psbA* > *rbcL*
Polygonaceae	23	95.7	73.9	0.0959	N/S	69.6	73.9	1	N/S	73.9	73.9	1	N/S
Cucurbitaceae	175	33.7	84.0	<.0001	*trnH-psbA* > ITS2	62.9	84.0	<.0001	*trnH-psbA* > *matK*	14.3	84.0	<.0001	*trnH-psbA* > *rbcL*
Begoniaceae	46	100.0	23.9	<.0001	*trnH-psbA* < ITS2	21.7	23.9	1	N/S	30.4	23.9	0.6398	N/S
Brassicaceae	46	43.5	52.2	0.5315	N/S	39.1	52.2	0.2953	N/S	34.8	52.2	0.1406	N/S
Rosaceae	17	100.0	64.7	0.0184	*trnH-psbA* < ITS2	100.0	64.7	0.0184	*trnH-psbA* < *matK*	41.2	64.7	0.3028	N/S
Crassulaceae	6	100.0	100.0	N/A	N/A	100.0	100.0	N/A	N/A	66.7	100.0	0.4545	N/S
Apiaceae	56	92.9	67.9	0.0015	*trnH-psbA* < ITS2	76.8	67.9	0.3985	N/S	58.9	67.9	0.4329	N/S
Araliaceae	4	100.0	25.0	0.1429	N/S	25.0	25.0	1	N/S	100.0	25.0	0.1429	N/S
Solanaceae	25	100.0	100.0	N/A	N/A	100.0	100.0	N/A	N/A	72.0	100.0	0.0096	*trnH-psbA* > *rbcL*
Oleaceae	58	82.8	81.0	1	N/S	53.4	81.0	0.0028	*trnH-psbA* > *matK*	46.6	81.0	0.0002	*trnH-psbA* > *rbcL*
Caprifoliaceae	7	100.0	100.0	N/A	N/A	100.0	100.0	N/A	N/A	100.0	100.0	N/A	N/A
Adoxaceae	46	82.6	80.4	1	N/S	45.7	80.4	0.001	*trnH-psbA* > *matK*	6.5	80.4	<.0001	*trnH-psbA* > *rbcL*
Asteraceae	100	92.0	53.0	<.0001	*trnH-psbA* < ITS2	48.0	53.0	0.5717	N/S	18.0	53.0	<.0001	*trnH-psbA* > *rbcL*
Aquifoliaceae	11	27.3	100.0	0.001	*trnH-psbA* > ITS2	100.0	100.0	N/A	N/A	100.0	100.0	N/A	N/A
Celastraceae	160	88.1	87.5	1	N/S	58.8	87.5	<.0001	*trnH-psbA* > *matK*	42.5	87.5	<.0001	*trnH-psbA* > *rbcL*
Primulaceae	134	97.0	88.8	0.0153	*trnH-psbA* < ITS2	83.6	88.8	0.288	N/S	67.9	88.8	<.0001	*trnH-psbA* > *rbcL*
Ericaceae	341	29.6	41.9	0.001	*trnH-psbA* > ITS2	43.1	41.9	0.8163	N/S	20.8	41.9	<.0001	*trnH-psbA* > *rbcL*
Campanulaceae	24	91.7	75.0	0.2448	N/S	58.3	75.0	0.3587	N/S	54.2	75.0	0.227	N/S

Alismataceae	5	100.0	0.0	0.0079	*trnH-psbA* < ITS2	0.0	0.0	N/A	N/A	0.0	0.0	N/A	N/A
Araceae	4	100.0	100.0	N/A	N/A	100.0	100.0	N/A	N/A	100.0	100.0	N/A	N/A
Poaceae	48	62.5	39.6	0.0406	*trnH-psbA* < ITS2	25.0	39.6	0.1899	N/S	31.3	39.6	0.5224	N/S
Zingiberaceae	8	100.0	100.0	N/A	N/A	100.0	100.0	N/A	N/A	100.0	100.0	N/A	N/A
Amaryllidaceae	59	79.7	64.4	0.1	N/S	55.9	64.4	0.4521	N/S	64.4	64.4	1	N/S
Dioscoreaceae	39	87.2	82.1	0.7549	N/S	56.4	82.1	0.0262	*trnH-psbA* > *matK*	28.2	82.1	<.0001	*trnH-psbA* > *rbcL*
Smilacaceae	4	100.0	100.0	N/A	N/A	100.0	100.0	N/A	N/A	100.0	100.0	N/A	N/A
Gentianaceae	8	37.5	50.0	1	N/S	100.0	50.0	0.0769	N/S	25.0	50.0	0.6084	N/S
Grossulariaceae	7	100.0	100.0	N/A	N/A	100.0	100.0	N/A	N/A	100.0	100.0	N/A	N/A
Hydrangeaceae	7	42.9	57.1	1	N/S	85.7	57.1	0.5594	N/S	42.9	57.1	1	N/S
Rubiaceae	92	69.6	68.5	1	N/S	59.8	68.5	0.2819	N/S	33.7	68.5	<.0001	*trnH-psbA* > *rbcL*
Taxaceae	13	100.0	46.2	0.0052	*trnH-psbA* < ITS2	38.5	46.2	1	N/S	23.1	46.2	0.411	N/S
Elaeagnaceae	10	100.0	100.0	N/A	N/A	100.0	100.0	N/A	N/A	100.0	100.0	N/A	N/A
Asparagaceae	38	89.5	60.5	0.0071	*trnH-psbA* < ITS2	86.8	60.5	0.0178	*trnH-psbA* < *matK*	55.3	60.5	0.8165	N/S
Cornaceae	6	100.0	16.7	0.0152	*trnH-psbA* < ITS2	33.3	16.7	1	N/S	33.3	16.7	1	N/S
Meliaceae	7	71.4	57.1	1	N/S	42.9	57.1	1	N/S	14.3	57.1	0.2657	N/S
Zygophyllaceae	10	50.0	60.0	1	N/S	30.0	60.0	0.3698	N/S	20.0	60.0	0.1698	N/S
Cephalotaxaceae	29	34.5	13.8	0.1233	N/S	100.0	13.8	<.0001	*trnH-psbA* < *matK*	6.9	13.8	0.6701	N/S
Melanthiaceae	40	82.5	50.0	0.0041	*trnH-psbA* < ITS2	30.0	50.0	0.1095	N/S	37.5	50.0	0.3675	N/S
Stachyuraceae	5	100.0	60.0	0.4444	N/S	0.0	60.0	0.1667	N/S	40.0	60.0	1	N/S
Nitrariaceae	5	0.0	100.0	0.0079	*trnH-psbA* > ITS2	20.0	100.0	0.0476	*trnH-psbA* > *matK*	0.0	100.0	0.0079	*trnH-psbA* > *rbcL*
Orobanchaceae	315	86.7	85.7	0.8175	N/S	75.2	85.7	0.0012	*trnH-psbA* > *matK*	50.5	85.7	<.0001	*trnH-psbA* > *rbcL*
Hypericaceae	5	100.0	100.0	N/A	N/A	100.0	100.0	N/A	N/A	100.0	100.0	N/A	N/A
Aceraceae	12	100.0	100.0	N/A	N/A	100.0	100.0	N/A	N/A	75.0	100.0	0.2174	N/S

N/A: Not Applicable, N/S: Not Significant.
