# Supplementary material for: Utility of the trnH–psbA Intergenic Spacer Region and Its Combinations as Plant DNA Barcodes: A Meta-Analysis
Source: PLoS One. 2012 Nov 14;7(11):e48833. doi: 10.1371/journal.pone.0048833 (PMC3498263; doi:10.1371/journal.pone.0048833)
Supplement: Table S13 — Detailed information on the identification success rate of single markers at the genus level and the corresponding statistical test results. (PDF) [file pone.0048833.s013.pdf]

Table S13. Detailed information on the identification success rate of single markers at the genus level and the corresponding statistical test results.

Genus	N	ITS2 and *trnH-psbA*				*matK* and *trnH-psbA*				*rbcL* and *trnH-psbA*			
		ITS2 success (%)	*trnH-psbA* success (%)	2-Tail	Result	*matK* success (%)	*trnH-psbA* success (%)	2-Tail	Result	*rbcL* success (%)	*trnH-psbA* success (%)	2-Tail	Result
Ficus	9	100.0	100.0	N/A	N/A	100.0	100.0	N/A	N/A	22.2	100.0	0.0023	*trnH-psbA* > *rbcL*
Chenopodium	24	100.0	100.0	N/A	N/A	100.0	100.0	N/A	N/A	100.0	100.0	N/A	N/A
Amaranthus	24	50.0	20.8	0.0687	N/S	45.8	20.8	0.1246	N/S	0.0	20.8	0.0496	*trnH-psbA* > *rbcL*
Silene	19	89.5	100.0	0.4865	N/S	89.5	100.0	0.4865	N/S	52.6	100.0	0.0011	*trnH-psbA* > *rbcL*
Rheum	6	100.0	100.0	N/A	N/A	100.0	100.0	N/A	N/A	100.0	100.0	N/A	N/A
Begonia	46	100.0	23.9	<.0001	*trnH-psbA* < ITS2	21.7	23.9	1	N/S	30.4	23.9	0.6398	N/S
Prunus	17	100.0	64.7	0.0184	*trnH-psbA* < ITS2	100.0	64.7	0.0184	*trnH-psbA* < *matK*	41.2	64.7	0.3028	N/S
Parnassia	160	88.1	87.5	1	N/S	58.8	87.5	<.0001	*trnH-psbA* > *matK*	42.5	87.5	<.0001	*trnH-psbA* > *rbcL*
Ribes	7	100.0	100.0	N/A	N/A	100.0	100.0	N/A	N/A	100.0	100.0	N/A	N/A
Acer	12	100.0	100.0	N/A	N/A	100.0	100.0	N/A	N/A	75.0	100.0	0.2174	N/S
Panax	4	100.0	25.0	0.1429	N/S	25.0	25.0	1	N/S	100.0	25.0	0.1429	N/S
Datura	14	100.0	100.0	N/A	N/A	100.0	100.0	N/A	N/A	50.0	100.0	0.0058	*trnH-psbA* > *rbcL*
Solanum	11	100.0	100.0	N/A	N/A	100.0	100.0	N/A	N/A	100.0	100.0	N/A	N/A
Sambucus	22	81.8	95.5	0.3449	N/S	50.0	95.5	0.0015	*trnH-psbA* > *matK*	0.0	95.5	<.0001	*trnH-psbA* > *rbcL*
Viburnum	24	83.3	66.7	0.3177	N/S	41.7	66.7	0.1468	N/S	12.5	66.7	0.0003	*trnH-psbA* > *rbcL*
Ligularia	26	100.0	30.8	<.0001	*trnH-psbA* < ITS2	42.3	30.8	0.5653	N/S	19.2	30.8	0.523	N/S
Nyssa	6	100.0	16.7	0.0152	*trnH-psbA* < ITS2	33.3	16.7	1	N/S	33.3	16.7	1	N/S
Ilex	11	27.3	100.0	0.001	*trnH-psbA* > ITS2	100.0	100.0	N/A	N/A	100.0	100.0	N/A	N/A
Rhododendron	211	24.2	47.4	<.0001	*trnH-psbA* > ITS2	36.0	47.4	0.023	*trnH-psbA* > *matK*	20.4	47.4	<.0001	*trnH-psbA* > *rbcL*
Allium	59	79.7	64.4	0.1	N/S	55.9	64.4	0.4521	N/S	64.4	64.4	1	N/S
Cassiope	12	33.3	41.7	1	N/S	50.0	41.7	1	N/S	50.0	41.7	1	N/S
Gaultheria	118	39.0	32.2	0.3413	N/S	55.1	32.2	0.0006	*trnH-psbA* < *matK*	18.6	32.2	0.0245	*trnH-psbA* > *rbcL*
Ligustrum	58	82.8	81.0	1	N/S	53.4	81.0	0.0028	*trnH-psbA* > *matK*	46.6	81.0	0.0002	*trnH-psbA* > *rbcL*

Alisma	5	100.0	0.0	0.0079	*trnH-psbA* < ITS2	0.0	0.0	N/A	N/A	0.0	0.0	N/A	N/A
Maianthemum	29	86.2	48.3	0.0045	*trnH-psbA* < ITS2	82.8	48.3	0.0119	*trnH-psbA* < *matK*	41.4	48.3	0.7921	N/S
Cyananthus	24	91.7	75.0	0.2448	N/S	58.3	75.0	0.3587	N/S	54.2	75.0	0.227	N/S
Lepidium	21	33.3	42.9	0.7513	N/S	23.8	42.9	0.3264	N/S	23.8	42.9	0.3264	N/S
Gentiana	8	37.5	50.0	1	N/S	100.0	50.0	0.0769	N/S	25.0	50.0	0.6084	N/S
Hydrangea	7	42.9	57.1	1	N/S	85.7	57.1	0.5594	N/S	42.9	57.1	1	N/S
Amentotaxus	13	100.0	46.2	0.0052	*trnH-psbA* < ITS2	38.5	46.2	1	N/S	23.1	46.2	0.411	N/S
Pedicularis	298	87.9	89.6	0.6042	N/S	77.2	89.6	<.0001	*trnH-psbA* > *matK*	53.4	89.6	<.0001	*trnH-psbA* > *rbcL*
Luculia	10	20.0	0.0	0.4737	N/S	0.0	0.0	N/A	N/A	0.0	0.0	N/A	N/A
Morinda	13	61.5	15.4	0.0414	*trnH-psbA* < ITS2	46.2	15.4	0.2016	N/S	7.7	15.4	1	N/S
Mussaenda	21	28.6	81.0	0.0016	*trnH-psbA* > ITS2	57.1	81.0	0.1809	N/S	14.3	81.0	<.0001	*trnH-psbA* > *rbcL*
Oldenlandia	13	100.0	76.9	0.22	N/S	100.0	76.9	0.22	N/S	69.2	76.9	1	N/S
Nitraria	5	0.0	100.0	0.0079	*trnH-psbA* > ITS2	20.0	100.0	0.0476	*trnH-psbA* > *matK*	0.0	100.0	0.0079	*trnH-psbA* > *rbcL*
Cipadessa	7	71.4	57.1	1	N/S	42.9	57.1	1	N/S	14.3	57.1	0.2657	N/S
Tacca	39	87.2	82.1	0.7549	N/S	56.4	82.1	0.0262	*trnH-psbA* > *matK*	28.2	82.1	<.0001	*trnH-psbA* > *rbcL*
Suaeda	5	100.0	100.0	N/A	N/A	100.0	100.0	N/A	N/A	80.0	100.0	1	N/S
Hippophae	10	100.0	100.0	N/A	N/A	100.0	100.0	N/A	N/A	100.0	100.0	N/A	N/A
Aconitum	34	44.1	47.1	1	N/S	23.5	47.1	0.0746	N/S	11.8	47.1	0.0029	*trnH-psbA* > *rbcL*
Osmorhiza	26	100.0	46.2	<.0001	*trnH-psbA* < ITS2	65.4	46.2	0.264	N/S	42.3	46.2	1	N/S
Peucedanum	19	78.9	78.9	1	N/S	78.9	78.9	1	N/S	57.9	78.9	0.2953	N/S
Pleurospermum	11	100.0	100.0	N/A	N/A	100.0	100.0	N/A	N/A	100.0	100.0	N/A	N/A
Lonicera	7	100.0	100.0	N/A	N/A	100.0	100.0	N/A	N/A	100.0	100.0	N/A	N/A
Primula	134	97.0	88.8	0.0153	*trnH-psbA* < ITS2	83.6	88.8	0.288	N/S	67.9	88.8	<.0001	*trnH-psbA* > *rbcL*
Smilax	4	100.0	100.0	N/A	N/A	100.0	100.0	N/A	N/A	100.0	100.0	N/A	N/A
Paris	40	82.5	50.0	0.0041	*trnH-psbA* < ITS2	30.0	50.0	0.1095	N/S	37.5	50.0	0.3675	N/S
Cephalotaxus	29	34.5	13.8	0.1233	N/S	100.0	13.8	<.0001	*trnH-psbA* < *matK*	6.9	13.8	0.6701	N/S
Arisaema	4	100.0	100.0	N/A	N/A	100.0	100.0	N/A	N/A	100.0	100.0	N/A	N/A

Kengyilia	48	62.5	39.6	0.0406	*trnH-psbA* < ITS2	25.0	39.6	0.1899	N/S	31.3	39.6	0.5224	N/S
Cardamine	9	77.8	100.0	0.4706	N/S	77.8	100.0	0.4706	N/S	55.6	100.0	0.0824	N/S
Hypericum	5	100.0	100.0	N/A	N/A	100.0	100.0	N/A	N/A	100.0	100.0	N/A	N/A
Hedyotis	20	100.0	95.0	1	N/S	80.0	95.0	0.3416	N/S	85.0	95.0	0.605	N/S
Leptodermis	15	100.0	100.0	N/A	N/A	53.3	100.0	0.0063	*trnH-psbA* > *matK*	6.7	100.0	<.0001	*trnH-psbA* > *rbcL*
Hemsleya	91	12.1	72.5	<.0001	*trnH-psbA* > ITS2	48.4	72.5	0.0014	*trnH-psbA* > *matK*	5.5	72.5	<.0001	*trnH-psbA* > *rbcL*
Zygophyllum	10	50.0	60.0	1	N/S	30.0	60.0	0.3698	N/S	20.0	60.0	0.1698	N/S
Ostryopsis	20	50.0	65.0	0.5231	N/S	0.0	65.0	<.0001	*trnH-psbA* > *matK*	10.0	65.0	0.0008	*trnH-psbA* > *rbcL*
Stachyurus	5	100.0	60.0	0.4444	N/S	0.0	60.0	0.1667	N/S	40.0	60.0	1	N/S
Draba	6	100.0	100.0	N/A	N/A	100.0	100.0	N/A	N/A	100.0	100.0	N/A	N/A
Alpinia	8	100.0	100.0	N/A	N/A	100.0	100.0	N/A	N/A	100.0	100.0	N/A	N/A
Sinosenecio	60	90.0	58.3	0.0001	*trnH-psbA* < ITS2	46.7	58.3	0.2727	N/S	6.7	58.3	<.0001	*trnH-psbA* > *rbcL*
Tupistra	9	100.0	100.0	N/A	N/A	100.0	100.0	N/A	N/A	100.0	100.0	N/A	N/A
Bistorta	10	90.0	40.0	0.0573	N/S	30.0	40.0	1	N/S	40.0	40.0	1	N/S
Oxyria	7	100.0	100.0	N/A	N/A	100.0	100.0	N/A	N/A	100.0	100.0	N/A	N/A
Rhodiola	6	100.0	100.0	N/A	N/A	100.0	100.0	N/A	N/A	66.7	100.0	0.4545	N/S
Thladiantha	84	57.1	96.4	<.0001	*trnH-psbA* > ITS2	78.6	96.4	0.0007	*trnH-psbA* > *matK*	23.8	96.4	<.0001	*trnH-psbA* > *rbcL*
Pugionium	10	0.0	0.0	N/A	N/A	0.0	0.0	N/A	N/A	0.0	0.0	N/A	N/A
Soroseris	6	66.7	33.3	0.5671	N/S	16.7	33.3	1	N/S	16.7	33.3	1	N/S
Pterygiella	17	64.7	17.6	0.0134	*trnH-psbA* < ITS2	41.2	17.6	0.2587	N/S	0.0	17.6	0.2273	N/S
Syncalathium	8	100.0	100.0	N/A	N/A	100.0	100.0	N/A	N/A	100.0	100.0	N/A	N/A

N/A: Not Applicable, N/S: Not Significant.
