# Supplementary material for: Utility of the trnH–psbA Intergenic Spacer Region and Its Combinations as Plant DNA Barcodes: A Meta-Analysis
Source: PLoS One. 2012 Nov 14;7(11):e48833. doi: 10.1371/journal.pone.0048833 (PMC3498263; doi:10.1371/journal.pone.0048833)
Supplement: Table S14 — Detailed information on the identification success rate of two-locus combinations at the family level and the corresponding statistical test results. (PDF) [file pone.0048833.s014.pdf]

Table S14. Detailed information on the identification success rate of two-locus combinations at the family level and the corresponding statistical test results.

Family	N	***trnH-psbA*** **+ITS2 and** ***trnH-psbA*** **+*matK***				***trnH-psbA*** **+ITS2 and** ***trnH-psbA*** **+*rbcL***				***matK*** **+*rbcL* and** ***trnH-psbA+ITS2***			
		***trnH-psbA*+ ITS2 success (%)**	***trnH-psbA*+ *matK* success (%)**	**2-Tail**	**Result**	***trnH-psbA*+ ITS2 success (%)**	***trnH-psbA*+ *rbcL* success (%)**	**2-Tail**	**Result**	***matK*+ *rbcL* success (%)**	***trnH-psbA*+ ITS2 success (%)**	**2-Tail**	**Result**
Ranunculaceae	34	58.8	52.9	0.8073	N/S	58.8	47.1	0.4664	N/S	23.5	58.8	0.006	*trnH-psbA* +ITS2>*matK* +*rbcL*
Moraceae	9	100.0	100.0	N/A	N/A	100.0	100.0	N/A	N/A	100.0	100.0	N/A	N/A
Betulaceae	20	65.0	65.0	1	N/S	65.0	70.0	1	N/S	10.0	65.0	8E-04	*trnH-psbA* +ITS2>*matK* +*rbcL*
Amaranthaceae	53	79.2	75.5	0.8169	N/S	79.2	64.2	0.1305	N/S	75.5	79.2	0.817	N/S
Caryophyllaceae	19	100.0	100.0	N/A	N/A	100.0	100.0	N/A	N/A	89.5	100.0	0.487	N/S
Polygonaceae	23	95.7	78.3	0.1868	N/S	95.7	78.3	0.1868	N/S	78.3	95.7	0.187	N/S
Cucurbitaceae	175	84.6	84.6	1	N/S	84.6	84.0	1	N/S	64.0	84.6	<.0001	*trnH-psbA* +ITS2>*matK* +*rbcL*
Begoniaceae	46	100.0	45.7	<.0001	*trnH-psbA* +ITS2>*trnH-psbA* +matK	100.0	54.3	<.0001	*trnH-psbA* +ITS2>*trnH-psbA* +*rbcL*	30.4	100.0	<.0001	*trnH-psbA* +ITS2>*matK* +*rbcL*
Brassicaceae	46	54.3	52.2	1	N/S	54.3	52.2	1	N/S	39.1	54.3	0.21	N/S
Rosaceae	17	100.0	100.0	N/A	N/A	100.0	64.7	0.0184	*trnH-psbA* +ITS2>*trnH-psbA* +*rbcL*	100.0	100.0	N/A	N/A
Crassulaceae	6	100.0	100.0	N/A	N/A	100.0	100.0	N/A	N/A	100.0	100.0	N/A	N/A
Apiaceae	56	98.2	82.1	0.0082	*trnH-psbA* +ITS2>*trnH-psbA* +matK	98.2	73.2	0.0002	*trnH-psbA* +ITS2>*trnH-psbA* +*rbcL*	85.7	98.2	0.032	*trnH-psbA* +ITS2>*matK* +*rbcL*
Araliaceae	4	100.0	50.0	0.4286	N/S	100.0	100.0	N/A	N/A	100.0	100.0	N/A	N/A
Solanaceae	25	100.0	100.0	N/A	N/A	100.0	100.0	N/A	N/A	100.0	100.0	N/A	N/A
Oleaceae	58	91.4	86.2	0.5577	N/S	91.4	82.8	0.2681	N/S	79.3	91.4	0.113	N/S
Caprifoliaceae	7	100.0	100.0	N/A	N/A	100.0	100.0	N/A	N/A	100.0	100.0	N/A	N/A
Adoxaceae	46	93.5	80.4	0.119	N/S	93.5	80.4	0.119	N/S	45.7	93.5	<.0001	*trnH-psbA* +ITS2>*matK* +*rbcL*
Asteraceae	100	96.0	70.0	<.0001	*trnH-psbA* +ITS2>*trnH-psbA* +matK	96.0	53.0	<.0001	*trnH-psbA* +ITS2>*trnH-psbA* +*rbcL*	48.0	96.0	<.0001	*trnH-psbA* +ITS2>*matK* +*rbcL*
Aquifoliaceae	11	100.0	100.0	N/A	N/A	100.0	100.0	N/A	N/A	100.0	100.0	N/A	N/A
Celastraceae	160	98.1	90.6	0.006	*trnH-psbA* +ITS2>*trnH-psbA* +matK	98.1	88.8	0.001	*trnH-psbA* +ITS2>*trnH-psbA* +*rbcL*	71.9	98.1	<.0001	*trnH-psbA* +ITS2>*matK* +*rbcL*
Primulaceae	134	100.0	89.6	<.0001	*trnH-psbA* +ITS2>*trnH-psbA* +matK	100.0	91.8	0.0008	*trnH-psbA* +ITS2>*trnH-psbA* +*rbcL*	87.3	100.0	<.0001	*trnH-psbA* +ITS2>*matK* +*rbcL*
Ericaceae	341	52.2	57.2	0.2184	N/S	52.2	46.6	0.168	N/S	44.6	52.2	0.055	N/S

Campanulaceae	24	100.0	79.2	0.0496	*trnH-psbA*+ITS2>*trnH-psbA*+matK	100.0	75.0	0.0219	*trnH-psbA*+ITS2>*trnH-psbA*+*rbcL*	62.5	100.0	0.002	*trnH-psbA*+ITS2>*matK*+*rbcL*
Alismataceae	5	100.0	0.0	0.0079	*trnH-psbA*+ITS2>*trnH-psbA*+matK	100.0	0.0	0.0079	*trnH-psbA*+ITS2>*trnH-psbA*+*rbcL*	0.0	100.0	0.008	*trnH-psbA*+ITS2>*matK*+*rbcL*
Araceae	4	100.0	100.0	N/A	N/A	100.0	100.0	N/A	N/A	100.0	100.0	N/A	N/A
Poaceae	48	75.0	47.9	0.0114	*trnH-psbA*+ITS2>*trnH-psbA*+matK	75.0	56.3	0.0849	N/S	45.8	75.0	0.006	*trnH-psbA*+ITS2>*matK*+*rbcL*
Zingiberaceae	8	100.0	100.0	N/A	N/A	100.0	100.0	N/A	N/A	100.0	100.0	N/A	N/A
Amaryllidaceae	59	86.4	67.8	0.0273	*trnH-psbA*+ITS2>*trnH-psbA*+matK	86.4	78.0	0.3359	N/S	72.9	86.4	0.108	N/S
Dioscoreaceae	39	89.7	92.3	1	N/S	89.7	82.1	0.517	N/S	56.4	89.7	0.002	*trnH-psbA*+ITS2>*matK*+*rbcL*
Smilacaceae	4	100.0	100.0	N/A	N/A	100.0	100.0	N/A	N/A	100.0	100.0	N/A	N/A
Gentianaceae	8	50.0	100.0	0.0769	N/S	50.0	50.0	1	N/S	100.0	50.0	0.077	N/S
Grossulariaceae	7	100.0	100.0	N/A	N/A	100.0	100.0	N/A	N/A	100.0	100.0	N/A	N/A
Hydrangeaceae	7	57.1	85.7	0.5594	N/S	57.1	57.1	1	N/S	85.7	57.1	0.559	N/S
Rubiaceae	92	82.6	79.3	0.7076	N/S	82.6	72.8	0.1559	N/S	68.5	82.6	0.039	*trnH-psbA*+ITS2>*matK*+*rbcL*
Taxaceae	13	100.0	46.2	0.0052	*trnH-psbA*+ITS2>*trnH-psbA*+matK	100.0	46.2	0.0052	*trnH-psbA*+ITS2>*trnH-psbA*+*rbcL*	38.5	100.0	0.002	*trnH-psbA*+ITS2>*matK*+*rbcL*
Elaeagnaceae	10	100.0	100.0	N/A	N/A	100.0	100.0	N/A	N/A	100.0	100.0	N/A	N/A
Asparagaceae	38	92.1	86.8	0.711	N/S	92.1	68.4	0.019	*trnH-psbA*+ITS2>*trnH-psbA*+*rbcL*	86.8	92.1	0.711	N/S
Cornaceae	6	100.0	33.3	0.0606	N/S	100.0	33.3	0.0606	N/S	33.3	100.0	0.061	N/S
Meliaceae	7	85.7	57.1	0.5594	N/S	85.7	57.1	0.5594	N/S	57.1	85.7	0.559	N/S
Zygophyllaceae	10	70.0	60.0	1	N/S	70.0	60.0	1	N/S	30.0	70.0	0.179	N/S
Cephalotaxaceae	29	41.4	100.0	<.0001	*trnH-psbA*+ITS2<*trnH-psbA*+matK	41.4	13.8	0.0379	*trnH-psbA*+ITS2>*trnH-psbA*+*rbcL*	100.0	41.4	<.0001	*trnH-psbA*+ITS2<*matK*+*rbcL*
Melanthiaceae	40	90.0	50.0	0.0002	*trnH-psbA*+ITS2>*trnH-psbA*+matK	90.0	52.5	0.0004	*trnH-psbA*+ITS2>*trnH-psbA*+*rbcL*	45.0	90.0	<.0001	*trnH-psbA*+ITS2>*matK*+*rbcL*
Stachyuraceae	5	100.0	60.0	0.4444	N/S	100.0	80.0	1	N/S	40.0	100.0	0.167	N/S
Nitrariaceae	5	100.0	100.0	N/A	N/A	100.0	100.0	N/A	N/A	20.0	100.0	0.048	*trnH-psbA*+ITS2>*matK*+*rbcL*
Orobanchaceae	315	95.2	91.1	0.0569	N/S	95.2	86.3	0.0002	*trnH-psbA*+ITS2>*trnH-psbA*+*rbcL*	75.9	95.2	<.0001	*trnH-psbA*+ITS2>*matK*+*rbcL*
Hypericaceae	5	100.0	100.0	N/A	N/A	100.0	100.0	N/A	N/A	100.0	100.0	N/A	N/A
Aceraceae	12	100.0	100.0	N/A	N/A	100.0	100.0	N/A	N/A	100.0	100.0	N/A	N/A

N/A: Not Applicable, N/S: Not Significant.
