# Supplementary material for: Utility of the trnH–psbA Intergenic Spacer Region and Its Combinations as Plant DNA Barcodes: A Meta-Analysis
Source: PLoS One. 2012 Nov 14;7(11):e48833. doi: 10.1371/journal.pone.0048833 (PMC3498263; doi:10.1371/journal.pone.0048833)
Supplement: Table S15 — Detailed information on the identification success rate of two-locus combinations at the genus level and the corresponding statistical test results. (PDF) [file pone.0048833.s015.pdf]

Table S15. Detailed information on the identification success rate of two-locus combinations at the genus level and the corresponding statistical test results.

Genus	N	***trnH-psbA*+ITS2 and *trnH-psbA*+*matK***				***trnH-psbA*+ITS2 and *trnH-psbA*+*rbcL***				***matK*+*rbcL* and *trnH-psbA*+*ITS2***			
		***trnH-psbA*+ ITS2 success (%)**	***trnH-psbA*+ *matK* success (%)**	**2-Tail**	**Result**	***trnH-psbA*+ ITS2 success (%)**	***trnH-psbA*+ *rbcL* success (%)**	**2-Tail**	**Result**	***matK*+ *rbcL* success (%)**	***trnH-psbA*+ ITS2 success (%)**	**2-Tail**	**Result**
Ficus	9	100.0	100.0	N/A	N/A	100.0	100.0	N/A	N/A	100.0	100.0	N/A	N/A
Chenopodium	24	100.0	100.0	N/A	N/A	100.0	100.0	N/A	N/A	100.0	100.0	N/A	N/A
Amaranthus	24	54.2	45.8	0.7732	N/S	54.2	20.8	0.0355	*trnH-psbA*+ITS2>*trnH-psbA*+*rbcL*	45.8	54.2	0.7732	N/S
Silene	19	100.0	100.0	N/A	N/A	100.0	100.0	N/A	N/A	89.5	100.0	0.4865	N/S
Rheum	6	100.0	100.0	N/A	N/A	100.0	100.0	N/A	N/A	100.0	100.0	N/A	N/A
Begonia	46	100.0	45.7	<.0001	*trnH-psbA*+ITS2>*trnH-psbA*+*matK*	100.0	54.3	<.0001	*trnH-psbA*+ITS2>*trnH-psbA*+*rbcL*	30.4	100.0	<.0001	*trnH-psbA*+ITS2>*matK*+*rbcL*
Prunus	17	100.0	100.0	N/A	N/A	100.0	64.7	0.0184	*trnH-psbA*+ITS2>*trnH-psbA*+*rbcL*	100.0	100.0	N/A	N/A
Parnassia	160	98.1	90.6	0.006	*trnH-psbA*+ITS2>*trnH-psbA*+*matK*	98.1	88.8	0.001	*trnH-psbA*+ITS2>*trnH-psbA*+*rbcL*	71.9	98.1	<.0001	*trnH-psbA*+ITS2>*matK*+*rbcL*
Ribes	7	100.0	100.0	N/A	N/A	100.0	100.0	N/A	N/A	100.0	100.0	N/A	N/A
Acer	12	100.0	100.0	N/A	N/A	100.0	100.0	N/A	N/A	100.0	100.0	N/A	N/A
Panax	4	100.0	50.0	0.4286	N/S	100.0	100.0	N/A	N/A	100.0	100.0	N/A	N/A
Datura	14	100.0	100.0	N/A	N/A	100.0	100.0	N/A	N/A	100.0	100.0	N/A	N/A
Solanum	11	100.0	100.0	N/A	N/A	100.0	100.0	N/A	N/A	100.0	100.0	N/A	N/A
Sambucus	22	95.5	95.5	1	N/S	95.5	95.5	1	N/S	50.0	95.5	0.0015	*trnH-psbA*+ITS2>*matK*+*rbcL*
Viburnum	24	91.7	66.7	0.0723	N/S	91.7	66.7	0.0723	N/S	41.7	91.7	0.0005	*trnH-psbA*+ITS2>*matK*+*rbcL*
Ligularia	26	100.0	53.8	<.0001	*trnH-psbA*+ITS2>*trnH-psbA*+*matK*	100.0	30.8	<.0001	*trnH-psbA*+ITS2>*trnH-psbA*+*rbcL*	42.3	100.0	<.0001	*trnH-psbA*+ITS2>*matK*+*rbcL*
Nyssa	6	100.0	33.3	0.0606	N/S	100.0	33.3	0.0606	N/S	33.3	100.0	0.0606	N/S
Ilex	11	100.0	100.0	N/A	N/A	100.0	100.0	N/A	N/A	100.0	100.0	N/A	N/A
Rhododendron	211	52.6	57.3	0.3786	N/S	52.6	50.7	0.7701	N/S	36.5	52.6	0.0012	*trnH-psbA*+ITS2>*matK*+*rbcL*
Allium	59	86.4	67.8	0.0273	*trnH-psbA*+ITS2>*trnH-psbA*+*matK*	86.4	78.0	0.3359	N/S	72.9	86.4	0.1082	N/S
Cassiope	12	75.0	58.3	0.6668	N/S	75.0	58.3	0.6668	N/S	66.7	75.0	1	N/S
Gaultheria	118	49.2	56.8	0.2968	N/S	49.2	38.1	0.115	N/S	56.8	49.2	0.2968	N/S

Ligustrum	58	91.4	86.2	0.5577	N/S	91.4	82.8	0.2681	N/S	79.3	91.4	0.1133	N/S
Alisma	5	100.0	0.0	0.0079	*trnH-psbA*+ITS2>*trnH-psbA*+*matK*	100.0	0.0	0.0079	*trnH-psbA*+ITS2>*trnH-psbA*+*rbcL*	0.0	100.0	0.0079	*trnH-psbA*+ITS2>*matK*+*rbcL*
Maianthemum	29	89.7	82.8	0.7057	N/S	89.7	58.6	0.0148	*trnH-psbA*+ITS2>*trnH-psbA*+*rbcL*	82.8	89.7	0.7057	N/S
Cyananthus	24	100.0	79.2	0.0496	*trnH-psbA*+ITS2>*trnH-psbA*+*matK*	100.0	75.0	0.0219	*trnH-psbA*+ITS2>*trnH-psbA*+*rbcL*	62.5	100.0	0.0016	*trnH-psbA*+ITS2>*matK*+*rbcL*
Lepidium	21	47.6	42.9	1	N/S	47.6	42.9	1	N/S	23.8	47.6	0.1971	N/S
Gentiana	8	50.0	100.0	0.0769	N/S	50.0	50.0	1	N/S	100.0	50.0	0.0769	N/S
Hydrangea	7	57.1	85.7	0.5594	N/S	57.1	57.1	1	N/S	85.7	57.1	0.5594	N/S
Amentotaxus	13	100.0	46.2	0.0052	*trnH-psbA*+ITS2>*trnH-psbA*+*matK*	100.0	46.2	0.0052	*trnH-psbA*+ITS2>*trnH-psbA*+*rbcL*	38.5	100.0	0.0016	*trnH-psbA*+ITS2>*matK*+*rbcL*
Pedicularis	298	97.0	94.0	0.1136	N/S	97.0	90.3	0.0012	*trnH-psbA*+ITS2>*trnH-psbA*+*rbcL*	77.9	97.0	<.0001	*trnH-psbA*+ITS2>*matK*+*rbcL*
Luculia	10	20.0	0.0	0.4737	N/S	20.0	0.0	0.4737	N/S	0.0	20.0	0.4737	N/S
Morinda	13	69.2	53.8	0.6882	N/S	69.2	15.4	0.0154	*trnH-psbA*+ITS2>*trnH-psbA*+*rbcL*	46.2	69.2	0.4283	N/S
Mussaenda	21	81.0	85.7	1	N/S	81.0	90.5	0.6628	N/S	71.4	81.0	0.7186	N/S
Oldenlandia	13	100.0	100.0	N/A	N/A	100.0	84.6	0.48	N/S	100.0	100.0	N/A	N/A
Nitraria	5	100.0	100.0	N/A	N/A	100.0	100.0	N/A	N/A	20.0	100.0	0.0476	*trnH-psbA*+ITS2>*matK*+*rbcL*
Cipadessa	7	85.7	57.1	0.5594	N/S	85.7	57.1	0.5594	N/S	57.1	85.7	0.5594	N/S
Tacca	39	89.7	92.3	1	N/S	89.7	82.1	0.517	N/S	56.4	89.7	0.0018	*trnH-psbA*+ITS2>*matK*+*rbcL*
Suaeda	5	100.0	100.0	N/A	N/A	100.0	100.0	N/A	N/A	100.0	100.0	N/A	N/A
Hippophae	10	100.0	100.0	N/A	N/A	100.0	100.0	N/A	N/A	100.0	100.0	N/A	N/A
Aconitum	34	58.8	52.9	0.8073	N/S	58.8	47.1	0.4664	N/S	23.5	58.8	0.0062	*trnH-psbA*+ITS2>*matK*+*rbcL*
Osmorhiza	26	100.0	76.9	0.0226	*trnH-psbA*+ITS2>*trnH-psbA*+*matK*	100.0	57.7	0.0003	*trnH-psbA*+ITS2>*trnH-psbA*+*rbcL*	84.6	100.0	0.1104	N/S
Peucedanum	19	94.7	78.9	0.3398	N/S	94.7	78.9	0.3398	N/S	78.9	94.7	0.3398	N/S
Pleurospermum	11	100.0	100.0	N/A	N/A	100.0	100.0	N/A	N/A	100.0	100.0	N/A	N/A
Lonicera	7	100.0	100.0	N/A	N/A	100.0	100.0	N/A	N/A	100.0	100.0	N/A	N/A
Primula	134	100.0	89.6	<.0001	*trnH-psbA*+ITS2>*trnH-psbA*+*matK*	100.0	91.8	0.0008	*trnH-psbA*+ITS2>*trnH-psbA*+*rbcL*	87.3	100.0	<.0001	*trnH-psbA*+ITS2>*matK*+*rbcL*
Smilax	4	100.0	100.0	N/A	N/A	100.0	100.0	N/A	N/A	100.0	100.0	N/A	N/A
Paris	40	90.0	50.0	0.0002	*trnH-psbA*+ITS2>*trnH-psbA*+*matK*	90.0	52.5	0.0004	*trnH-psbA*+ITS2>*trnH-psbA*+*rbcL*	45.0	90.0	<.0001	*trnH-psbA*+ITS2>*matK*+*rbcL*
Cephalotaxus	29	41.4	100.0	<.0001	*trnH-psbA*+ITS2<*trnH-psbA*+*matK*	41.4	13.8	0.0379	*trnH-psbA*+ITS2>*trnH-psbA*+*rbcL*	100.0	41.4	<.0001	*trnH-psbA*+ITS2<*matK*+*rbcL*

Arisaema	4	100.0	100.0	N/A	N/A	100.0	100.0	N/A	N/A	100.0	100.0	N/A	N/A
Kengyilia	48	75.0	47.9	0.0114	*trnH-psbA* +ITS2>*trnH-psbA* +*matK*	75.0	56.3	0.0849	N/S	45.8	75.0	0.0063	*trnH-psbA* +ITS2>*matK* +*rbcL*
Cardamine	9	100.0	100.0	N/A	N/A	100.0	100.0	N/A	N/A	77.8	100.0	0.4706	N/S
Hypericum	5	100.0	100.0	N/A	N/A	100.0	100.0	N/A	N/A	100.0	100.0	N/A	N/A
Hedyotis	20	100.0	100.0	N/A	N/A	100.0	100.0	N/A	N/A	100.0	100.0	N/A	N/A
Leptodermis	15	100.0	100.0	N/A	N/A	100.0	100.0	N/A	N/A	60.0	100.0	0.0169	*trnH-psbA* +ITS2>*matK* +*rbcL*
Hemsleya	91	72.5	73.6	1	N/S	72.5	72.5	1	N/S	50.5	72.5	0.0036	*trnH-psbA* +ITS2>*matK* +*rbcL*
Zygophyllum	10	70.0	60.0	1	N/S	70.0	60.0	1	N/S	30.0	70.0	0.1789	N/S
Ostryopsis	20	65.0	65.0	1	N/S	65.0	70.0	1	N/S	10.0	65.0	0.0008	*trnH-psbA* +ITS2>*matK* +*rbcL*
Stachyurus	5	100.0	60.0	0.4444	N/S	100.0	80.0	1	N/S	40.0	100.0	0.1667	N/S
Draba	6	100.0	100.0	N/A	N/A	100.0	100.0	N/A	N/A	100.0	100.0	N/A	N/A
Alpinia	8	100.0	100.0	N/A	N/A	100.0	100.0	N/A	N/A	100.0	100.0	N/A	N/A
Sinosenecio	60	96.7	76.7	0.0022	*trnH-psbA* +ITS2>*trnH-psbA* +*matK*	96.7	58.3	<.0001	*trnH-psbA* +ITS2>*trnH-psbA* +*rbcL*	46.7	96.7	<.0001	*trnH-psbA* +ITS2>*matK* +*rbcL*
Tupistra	9	100.0	100.0	N/A	Na	100.0	100.0	N/A	N/A	100.0	100.0	N/A	N/A
Bistorta	10	90.0	50.0	0.1409	N/S	90.0	50.0	0.1409	N/S	50.0	90.0	0.1409	N/S
Oxyria	7	100.0	100.0	N/A	N/A	100.0	100.0	N/A	N/A	100.0	100.0	N/A	N/A
Rhodiola	6	100.0	100.0	N/A	N/A	100.0	100.0	N/A	N/A	100.0	100.0	N/A	N/A
Thladiantha	84	97.6	96.4	1	N/S	97.6	96.4	1	N/S	78.6	97.6	0.0002	*trnH-psbA* +ITS2>*matK* +*rbcL*
Pugionium	10	0.0	0.0	N/A	N/A	0.0	0.0	N/A	N/A	0.0	0.0	N/A	N/A
Soroseris	6	66.7	33.3	0.5671	N/S	66.7	33.3	0.5671	N/S	16.7	66.7	0.2424	N/S
Pterygiella	17	64.7	41.2	0.3028	N/S	64.7	17.6	0.0134	*trnH-psbA* +ITS2>*trnH-psbA* +*rbcL*	41.2	64.7	0.3028	N/S
Syncalathium	8	100.0	100.0	N/A	N/A	100.0	100.0	N/A	N/A	100.0	100.0	N/A	N/A

N/A: Not Applicable, N/S: Not Significant.
