# Supplementary figures and images for: Utility of the trnH–psbA Intergenic Spacer Region and Its Combinations as Plant DNA Barcodes: A Meta-Analysis
Source: PLoS One. 2012 Nov 14;7(11):e48833. doi: 10.1371/journal.pone.0048833 (PMC3498263; doi:10.1371/journal.pone.0048833)

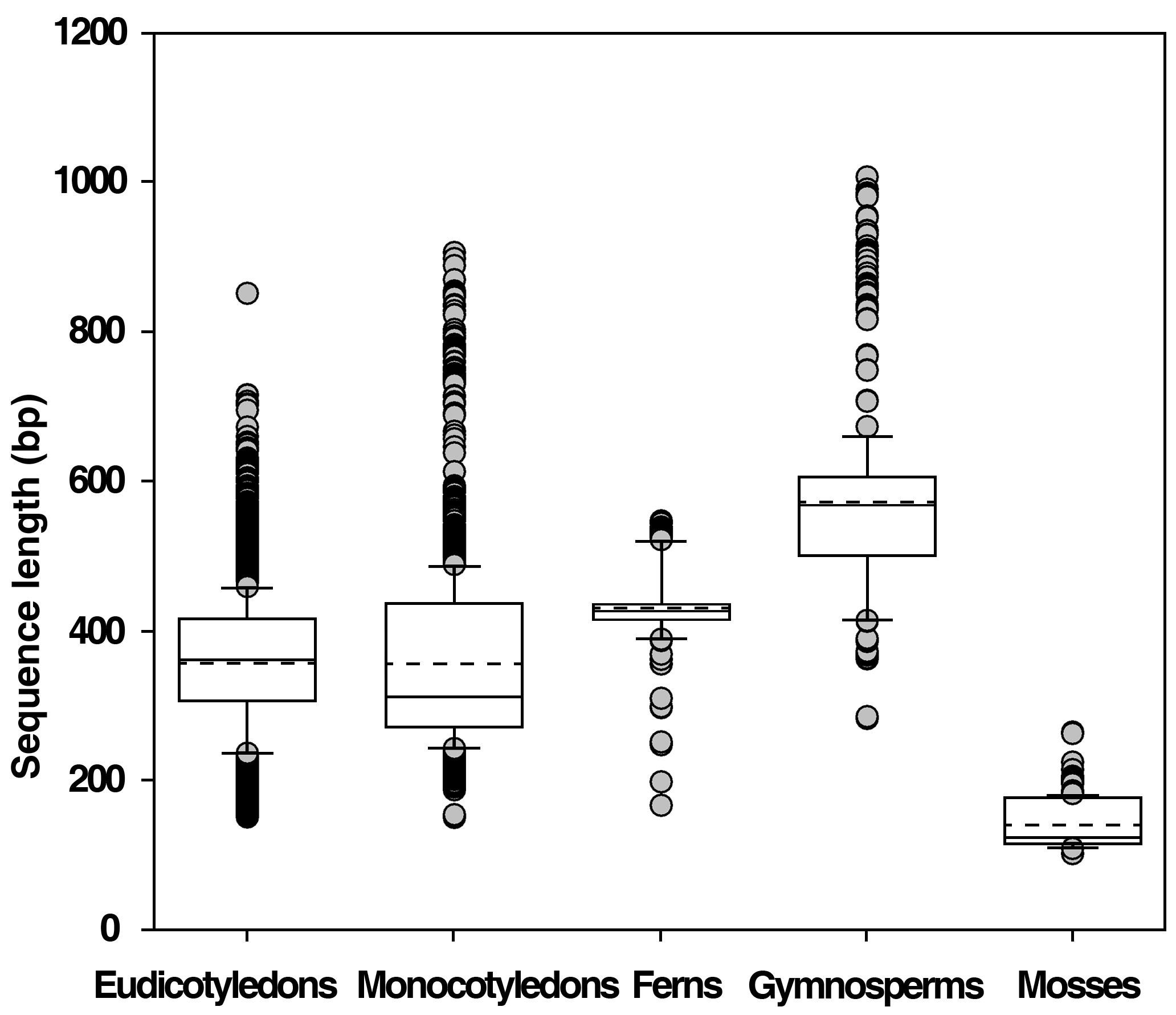

Supplement: Figure S1 — Box plots of the lengths of trnH – psbA sequences in the five major plant taxonomic groups. In each box plot, the box shows the interquartile range of the data, which is defined as the difference between the 75th and 25th percentiles. The continuous and dotted lines across the box represent the median and average values, respectively. (TIF) [file pone.0048833.s016.tif]

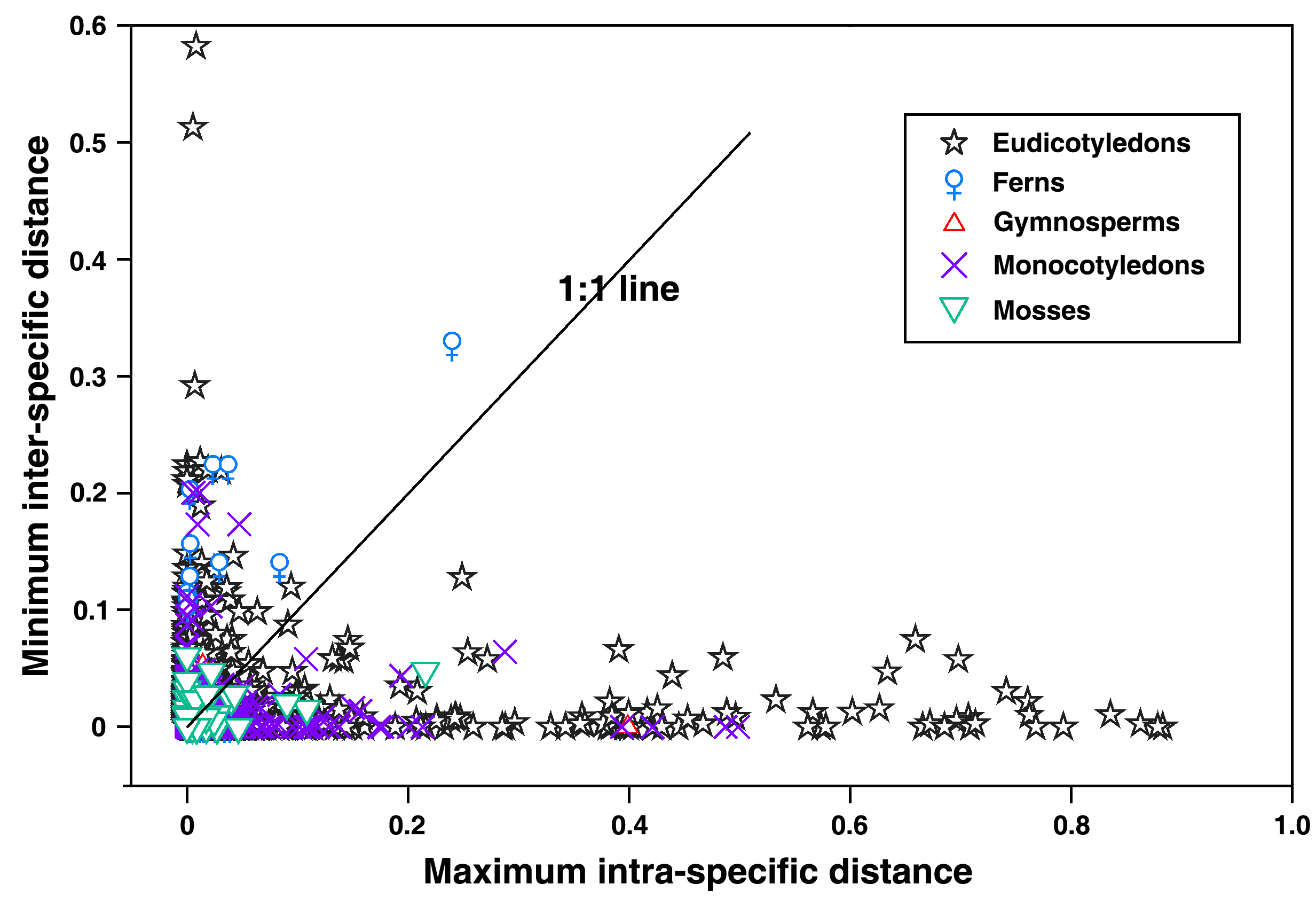

Supplement: Figure S2 — Inter- and intraspecific divergences of trnH – psbA sequences in the five major plant taxonomic groups. Sequence divergence across all species for which sequences of multiple individuals are presented is illustrated. Divergence is shown as a scatter plot between the maximal intraspecific and minimal interspecific distances. A black line is drawn where the two distances are equal. (TIF) [file pone.0048833.s017.tif]
