# Supplementary material for: Utility of the trnH–psbA Intergenic Spacer Region and Its Combinations as Plant DNA Barcodes: A Meta-Analysis
Source: PLoS One. 2012 Nov 14;7(11):e48833. doi: 10.1371/journal.pone.0048833 (PMC3498263; doi:10.1371/journal.pone.0048833)
Supplement: PRISMA Flow Diagram S1 — (DOC) [file pone.0048833.s019.doc]

Identification

Screening

Eligibility

Included

# of Recodes identified through database searching (n=36833)

# of additional records

Identified through other sources (n=0)

Records after duplicates

removed (n=36833)

Records after flanking sequences removed (n=36833)

Records after *rps19* insertions (n=36833)

Records after intraspecific inversions (n=36833)

Records after low-quality and short-sequence removed (n=34673)

Records for sequences from any genus that have more than one sequences, and from any species that have more than one sequences (n=20120)

# of records excludes (n=16713)

Full-text articles assessed for

eligibility (n=242)

# of full-text

articles

excluded,

with reasons (n=0)

Records included in *trnH-psbA* single marker study (meta-analysis) (n=17983)

Records included in single marker and marker combination comparison study (meta-analysis) (n=2190)
